# Genome and Transcriptome Analysis of the Basidiomycetous Yeast *Pseudozyma antarctica* Producing Extracellular Glycolipids, Mannosylerythritol Lipids

**DOI:** 10.1371/journal.pone.0086490

**Published:** 2014-02-24

**Authors:** Tomotake Morita, Hideaki Koike, Hiroko Hagiwara, Emi Ito, Masayuki Machida, Shun Sato, Hiroshi Habe, Dai Kitamoto

**Affiliations:** 1 Research Institute for Innovation in Sustainable Chemistry, National Institute of Advanced Industrial Science and Technology (AIST), Tsukuba Central 5-2, 1-1-1 Higashi, Tsukuba, Ibaraki, Japan; 2 Bioproduction Research Institute, National Institute of Advanced Industrial Science and Technology (AIST), Tsukuba Central 6-9, 1-1-1 Higashi, Tsukuba, Ibaraki, Japan; Yonsei University, Republic of Korea

## Abstract

*Pseudozyma antarctica* is a non-pathogenic phyllosphere yeast known as an excellent producer of mannosylerythritol lipids (MELs), multi-functional extracellular glycolipids, from vegetable oils. To clarify the genetic characteristics of *P. antarctica*, we analyzed the 18 Mb genome of *P. antarctica* T-34. On the basis of KOG analysis, the number of genes (219 genes) categorized into lipid transport and metabolism classification in *P. antarctica* was one and a half times larger than that of yeast *Saccharomyces cerevisiae* (140 genes). The gene encoding an ATP/citrate lyase (ACL) related to acetyl-CoA synthesis conserved in oleaginous strains was found in *P. antarctica* genome: the single ACL gene possesses the four domains identical to that of the human gene, whereas the other oleaginous ascomycetous species have the two genes covering the four domains. *P. antarctica* genome exhibited a remarkable degree of synteny to *U. maydis* genome, however, the comparison of the gene expression profiles under the culture on the two carbon sources, glucose and soybean oil, by the DNA microarray method revealed that transcriptomes between the two species were significantly different. In *P. antarctica*, expression of the gene sets relating fatty acid metabolism were markedly up-regulated under the oily conditions compared with glucose. Additionally, MEL biosynthesis cluster of *P. antarctica* was highly expressed regardless of the carbon source as compared to *U. maydis*. These results strongly indicate that *P. antarctica* has an oleaginous nature which is relevant to its non-pathogenic and MEL-overproducing characteristics. The analysis and dataset contribute to stimulate the development of improved strains with customized properties for high yield production of functional bio-based materials.

## Introduction

*Pseudozyma antarctica* is an anamorphic basidiomycetous yeast belonging to the Ustilagomycetes, a group that includes the smut fungus *Ustilago maydis* [1], [2]. To date, more than 15 species of the genus *Pseudozyma* have been reported and the number is still expanding [3], [4], [5], [6]. Of the genus *Pseudozyma*, *P. antarctica* was initially isolated on the bottom of a lake in Antarctica as *Candida antarctica*, and found to produce industry-relevant extracellular lipases at high levels. Their modification was thoroughly investigated to enhance the efficiency of the reaction and expand their industrial application [7], [8], [9]. Very recently, the extracellular production of an excellent biodegradable plastic–degrading enzyme, which degrades poly-butylene succinate and poly-butylene succinate-co-adipate, was found in the strains of *P. antarctica* isolated from plant surfaces [10].

**Figure 1:**
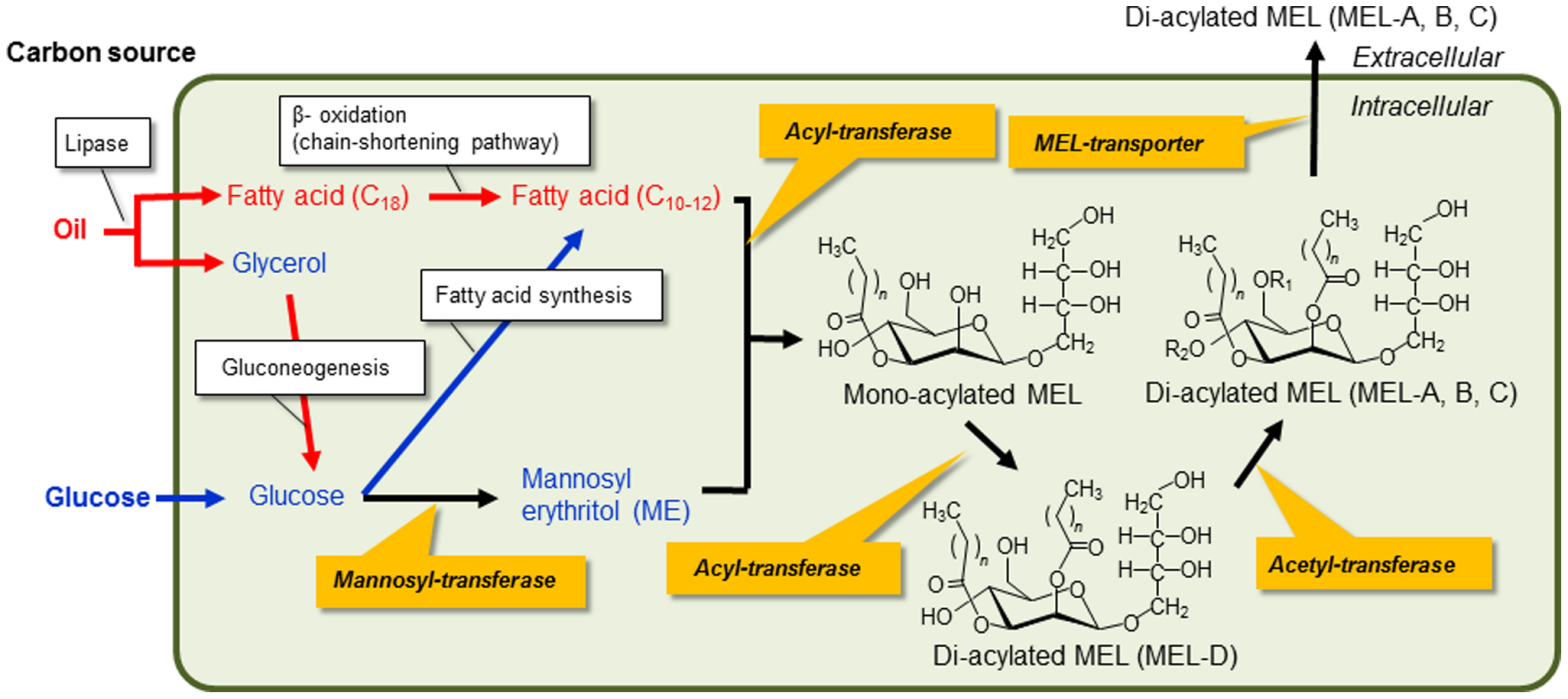
The biosynthesis and chemical structure of mannosylerythritol lipids. MEL-A: R_1_ = Ac, R_2_ = Ac; MEL-B: R_1_ = Ac, R_2_ = H; MEL-C: R_1_ = H, R_2_ = Ac; *n* = 4–12; *m* = 6–16.

Two decades ago, *P. antarctica* T-34 was isolated as a producer of the extracellular glycolipids, mannosylerythritol lipids (MELs, Fig. 1), which consist of 4-*O*-*β*-D-mannopyranosyl-*meso*-erythritol as the hydrophilic moiety and fatty acids as the hydrophobic moiety, at Tsukuba, Japan [11]. MELs have gained high expectations not only as eco-friendly biosurfactants, due to their excellent surface activity, but have also attracted considerable interest in recent years due to their unique properties, including self-assembling, antitumor and cell-differentiation induction activities, as well as moisturizing and hair repairing properties [12], [13], [14]. MELs were recently commercially utilized as a cosmetic ingredient, SurfMellow®, by the Japanese company Toyobo Co., Ltd. (http://www.toyobo-global.com/seihin/cosme/surfmellow.htm). Further improvements for the commercial production of MELs, and their applications to life-science, nanotechnology and environmental technology, are currently in progress [15], [16], [17].

Previously, a gene cluster for MEL biosynthesis expressing under conditions of nitrogen starvation was identified by a genetic study in *U. maydis* [18], [19]. It has been reported that *U. maydis* simultaneously produces a mixture of extracellular glycolipids, MELs and cellobiose lipids, at a yield of 30 g/L in a culture containing vegetable oils as carbon source [20], [21], [22]. In contrast, *P. antarctica* T-34 only produces MELs in large amounts when the cells are grown in a culture containing vegetable oil as carbon source and sodium nitrate as nitrogen source, and the production yield reaches 140 g/L using *n*-alkanes as carbon source [23]. The other species of the genus *Pseudozyma* are also capable of producing large amounts of MELs from vegetable oil as the sole carbon source [24], [25], [26]. Therefore the *Pseudozyma* species have great potential for the industrial large scale production of MELs using massive amounts of vegetable oil.

The fatty acid parts of the MELs are formed via chain shorting pathways like beta-oxidation, and their composition depends on the oil supplied as carbon source [27]. The MELs produced from vegetable oil containing mainly C_18_ fatty acids by *P. antarctica* mainly contain C_10_ and C_12_ fatty acids as the hydrophobic part [28], while the MELs produced from vegetable oil by *P. siamensis*, *P. shanxiensis*, and *U. maydis* are mainly C_16_ and C_4_ fatty acids [20]. Furthermore, the mono-acetylated type of MELs, MEL-B and MEL-C, which are deacetylated MEL-A, are the main product of the species of the genus *Pseudozyma* such as *P. tsukubaensis*, *P. hubeiensis*, and *P. graminicola* [17]. Recently, we also identified the mono- and tri-acylated types of MELs [29], [30]. The physicochemical properties of each MEL derivative change drastically depending on slight differences in the chemical structure. The development of the genetically modified strains of the genus *Pseudozyma* will thus probably generate novel industrial strains which are able to produce more useful bio-based materials, including glycolipids, from oil-biomass. However, there is little information on the genetic characteristics of these MEL producers. We thus have sequenced the genome of *P. antarctica* T-34 and reported the gene cluster for MEL biosynthesis [31].

Here we have demonstrated detailed analysis of *P. antarctica* T-34 genome, and revealed oleaginous nature of the yeast on the basis of the sequence analysis of the encoding genes. In addition, a comparative genomic and transcriptomic studies between *P. antarctica* and *U. maydis* have revealed that *P. antarctica* closely related to *U. maydis* at the genome level, while the gene expression pattern is extremely different under the oily conditions. Besides the importance of understanding gene expression for MEL biosynthesis in *P. antarctica* to further improve genetic engineering and its commercial use, insight into the fatty acid metabolism will inspire more effective strategies to use feedstocks for production of functional bio-based materials.

## Materials and Methods

### Microorganisms

*P. antarctica* (formerly *Candida antarctica*) T-34 used in this study was isolated as a MEL producer using soybean oil as the sole carbon source [11]. *U. maydis* DSM14603 ( = UM521) was obtained from the Deutsche Sammlung von Mikroorganismen und Zellkulturen GmbH, Germany. Stock cultures were cultivated for 3 days at 25°C on a YM plate medium containing 1% glucose, 0.5% peptone, 0.3% yeast extract, 0.3% malt extract and 3.0% agar. They were stored at 4°C and renewed every 2 weeks.

### Culture Conditions

Seed cultures were prepared by inoculating cells grown on slants into test tubes containing YM medium (1% glucose, 0.5% peptone, 0.3% yeast extract, and 0.3% malt extract) at 25°C on a rotary shaker at 300 rpm for 2 days. Seed cultures (1 ml) were transferred to 200 ml Erlenmeyer flask containing 20 ml of an experimental medium (0.03% MgSO_4_, 0.03% KH_2_PO_4_, 0.1% yeast extract, pH 6.0, containing different carbon), and then incubated as above for 7 days.

### Isolation and Detection of Glycolipids

After cultivation, the culture broth including glycolipids was extracted with an equal volume of ethyl acetate. The ethyl acetate extracts were analyzed by thin-layer chromatography (TLC). TLC was performed by chloroform–methanol–NH_4_OH (65∶15∶2, by vol.) as the solvent system. Visualization was performed by spraying 0.3% of the anthrone–sulfate reagents on the TLC plate and heating it at 90°C for 5 min [11]. Purified MEL-A, MEL-B and MEL-C, which were prepared from soybean oil by *P. antarctica* T-34, were used in the following experiments as a standard.

### High-performance Liquid Chromatography (HPLC)

The quantification of di-acylated MEL was carried out by HPLC on a silica gel column (Inertsil SIL 100A 5 µm, 4.6×250 mm; GL science Inc., Japan) with a low temperature-evaporative light scattering detector (Model 300S ELSD; SofTA Corporation, Thornton, CO) using a gradient solvent program consisting of various proportions of chloroform and methanol (from 100∶0 to 0∶100, v/v) at a flow rate of 1 ml/min [25]. The quantification of di-acylated MEL was based on the standard curve using the pure MEL fraction, which was prepared by *P. antarctica* T-34, as described previously [24]. All the measurements reported here are calculated values from at least three independent experiments.

### Purification of MELs

The ethyl acetate fraction was separated and evaporated. The concentrated glycolipids were dissolved in chloroform and then purified by silica gel (Wako-gel C-200; Wako, Osaka, Japan) column chromatography using a gradient elution of chloroform/acetone (10∶0 to 0∶10, v/v) mixtures as solvent systems [28].

### Genome Annotation and Comparative Genomic Analysis

The genome was annotated using the homology with the genes of known function in public database. All predicted proteins were functionally annotated using KEGG [32] for metabolic pathways, and KOG [33] for eukaryotic clusters of orthologs as was described previously [31]. GO term(s) were assigned to each gene if aligned pair-wise amino acid sequences with significant homologies (E-value <1e-10) were identified by BLAST+ version 2.2.27 [34] against gene(s) in the newest (as of Jan 2013) GO database [35].

The relation of the scaffold regions was analyzed with the alignments of nucleotide sequences derived by MUMmer 3.0 using NUCmer program with default setting [36]. The dot plots were generated by mummerplot program using an option for the layout of a multiplot to arrange orders and orientations of scaffolds (-l option).

For the annotation of the genes related to metabolism of lipid and carbohydrate (Table S2) the most reference genes for reactions were derived from SGD database (http://www.yeastgenome.org/). Some reference genes known to be lacking from *S. cerevisiae* were derived from *Aspergillus oryzae* and other fungi [37], [38]. Homologous proteins in *P. antarctica* were detected by Blastp with the cutoff of E-value 1e-10 when the aligned region cover more than 80% of the reference gene. The annotation of the yeast CDSs for metabolism was derived from the SGD database. The KOG information of *S. cerevisiae* genes were derived from JGI MycoCosm [39].

### Phylogenetic Analysis of ACL Gene

ATP/citrate lyase genes originated from various fungi were obtained from a web-based fungal resource MycoCosm [39]. The ACL gene from human was obtained from GenBank. The amino acid sequences were aligned using mafft [40] with linsi option. The domain structure of the ACL gene was identified for *P. antarctica* gene (PANT_7c00266) using the pfam server at the Sanger institute searched against the Pfam 27.0 database [41]. The aligned amino acid sequences for N-terminus domain (ATP-grasp domain) and C-terminus domain (CoA binding domain, CoA-ligase, and citrate synthetase) were used for the phylogenetic analysis.

The genealogy of ACL on the bases of the N-domain and C-domain was inferred by maximum-likelihood (ML) analysis using RAxML software package version7.2.8 [42]. The maximum likelihood analysis was performed on the alignments and the best tree inferred from 100 replicates is taken to represent the evolutionary history of the taxa analyzed, in which the initial trees were obtained automatically by feeding a seed for randomization. A more robust approach was used to infer phylogenetic relationship among the amino acid sequences studied by performing a rapid bootstrap ML analysis setting the bootstrap analysis to 100 runs. The PROTGAMMALG model was used for the analysis of the alignment. The trees are drawn to scale, with branch lengths calculated using the default method using FigTree. For both domains, the amino acid sequences of the human ACL were used as an outgroup.

### mRNA Preparation and DNA Microarray Experiments

Total RNA was isolated from cells using ISOGEN (Wako) according to the manufacturer’s instructions. Total RNA was isolated from cells as follows. Cells were ground in liquid nitrogen to a fine powder, and approximately 3 g was mixed with 15 ml ISOGEN solution, followed by the addition of 3 ml chloroform. The mixture was left at room temperature for 3 min and centrifuged at 2,300 g for 20 min. The supernatant was transferred to a fresh tube and mixed with 7.5 ml isopropanol. The mixture was left at room temperature for 10 min and centrifuged at 2,300 g for 20 min. The pellet was dried and dissolved in 300 µl of RNase-free water. mRNA was purified from approximately 200 µg total RNA using an OligotexTM-dT30<Super> mRNA Purification kit (Takara, Shiga, Japan) according to the manufacturer’s instructions.

DNA microarray experiments were performed as described previously [43]. Two cDNA samples labeled with Cy5 and Cy3 for each were mixed and applied onto Nimblegen Gene Expression Arrays (Roche Diagnostics, Tokyo) for hybridization. The slide was scanned to measure the fluorescence intensity of the two fluorophores using a GenePix 4200A scanner (Axon Instruments at Molecular Devices, Sunnyvale, CA).

### Image Quantification and Data Analysis

The acquired microarray images were analyzed with GenePix Pro 6.0 (Axon Instruments at Molecular Devices). Duplicated measurements were performed for each sample using the dye-swap method for determining the average. It should be noted that gene expression was compared based on two disparate culture conditions and the average fluorescence intensities for the two dyes were adjusted to be nearly identical, therefore that the total amount of mRNA per cell was different. After automatic segmentation of the spots, subtracting the local background, and calculating the ratio between the two fluorescence intensities, the reliability for each gene expression was evaluated using two DNA microarray slides from the dye-swap experiment according to the software’s instructions. The resulting data file was analyzed using the R package [44] and Bioconductor [45]. Data were normalized by setting the baseline measurement per spot and per segment of the chip via intensity dependent (LOESS) normalization using the marray module. Duplicated data files generated by the dye-swap experiment for an individual test were merged using the limma module.

### Statistical Analysis of DNA Microarray Experiments

Two values concerning expression level and induction for each gene were obtained for each experiment using a two-color platform. The logarithmic induction ratio (M-value) is defined as log2(Cy5) − log2(Cy3) and the average of the logarithmic signal intensities (A-value) is defined as 0.5[log2(Cy5)+log2(Cy3)], where Cy5 and Cy3 are normalized intensities for each transcript under the respective conditions of using soybean oil and glucose as the sole carbon source [46]. The genes expressed under oily conditions in *P. antarctica* were selected as those showing up-regulation in transcription expression when the vegetable oil conditions were compared to the glucose conditions. Statistical values for differently expression between the 2 conditions were obtained using the limma module of R and Bioconductor.

### Gene Set Enrichment Analysis

Gene set enrichment analysis was performed using the GSEA software v2.0.13 [47]. The Gene Sets were made from the GO terms assigned for each gene. The t-values derived from the limma module were used for the evaluation of the significance of differently expression of each gene and used for running the software as pre-ranked mode. The enriched gene sets were selected if the FDR q-value was better than 0.05.

### Microarray Data Accession Number

The data discussed in this publication have been deposited in NCBI’s Gene Expression Omnibus and are accessible through GEO Series accession number GSE47171 (http://www.ncbi.nlm.nih.gov/geo/query/acc.cgi?acc_GSE47171).

## Results

### Analysis of *Pseudozyma antarctica* Genome

**Figure 2:**
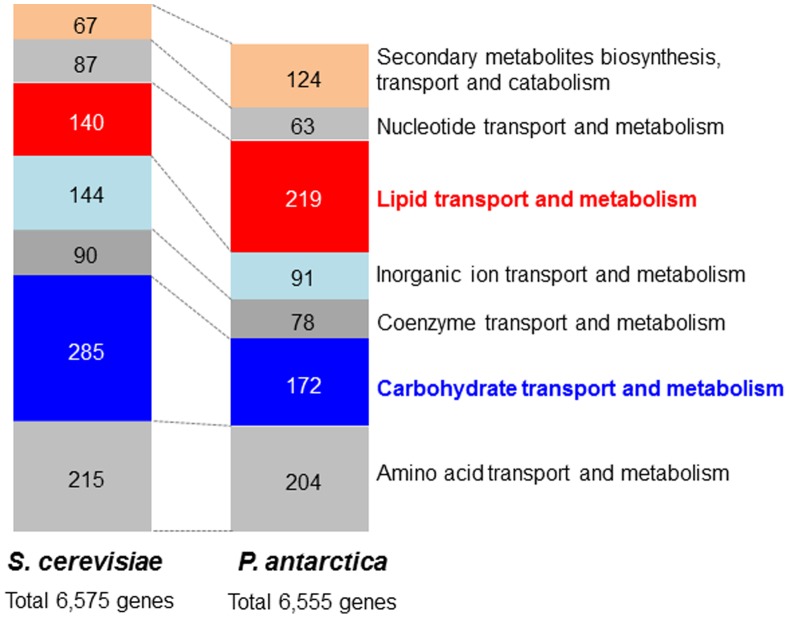
Comparison of the number of genes categorized on the basis of KOG classification between *P. antarctica* and *S. cerevisiae*. The number of genes categorized in KOG relating metabolism were shown in bar chart. The categories shown are as follows; Q, secondary metabolisoms, transport and catabolism; F, nucleotide transport and metabolism; I, lipid transport and metabolism; P, inorganic ion transport and metabolism; H, coenzyme transport and metabolism; G, carbohydrate transport and metabolism; E, amino acid transport and metabolism.

**Figure 3:**
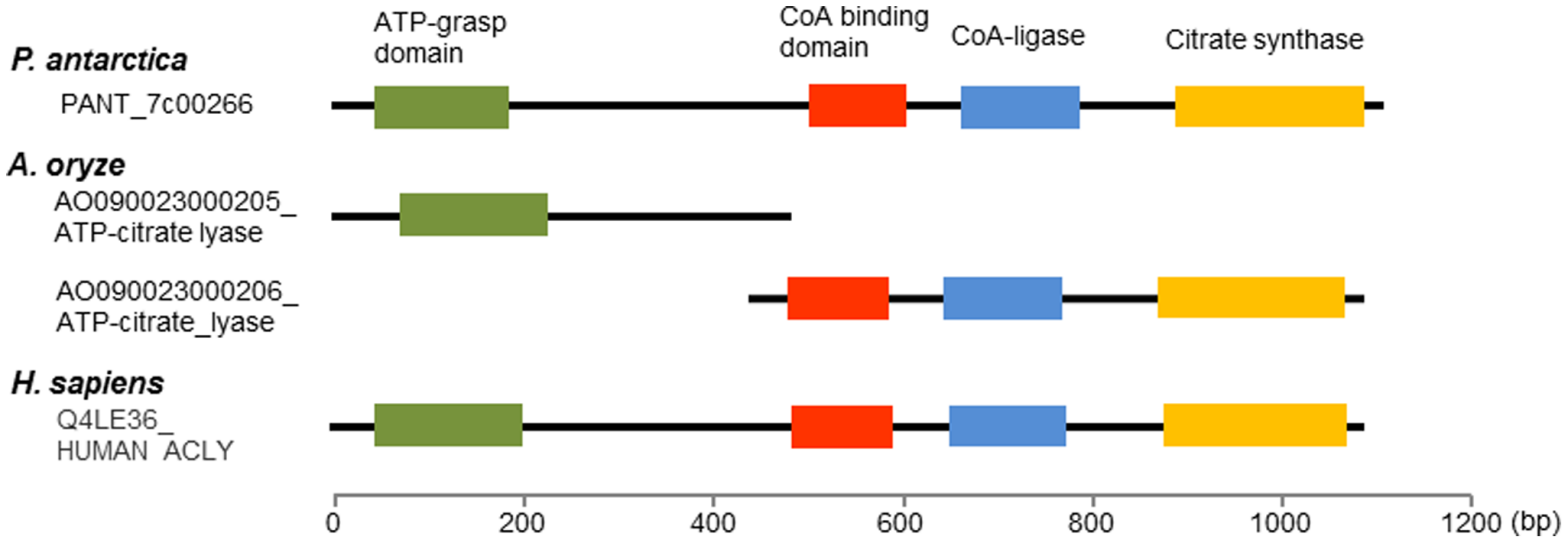
The domain structures of gene(s) encoding ATP/citrate lyase in *P. antarctica*, *A. oryze*, and *H. sapiens*. Single genes possessing the four domains, ATP-grasp domain (green), CoA binding domain (red), CoA-ligase (blue) and Citrate synthase (yellow), are found in *P. antarctica* (PANT_7c00266) and *Homo sapiens* (Q4LE36_HUMAN ACLY). *Aspergillus oryze* contains two unlinked ACL genes (AO090023000205_ATP-citrate_lyase and AO090023000206_ATP-citrate_lyase).

**Figure 4:**
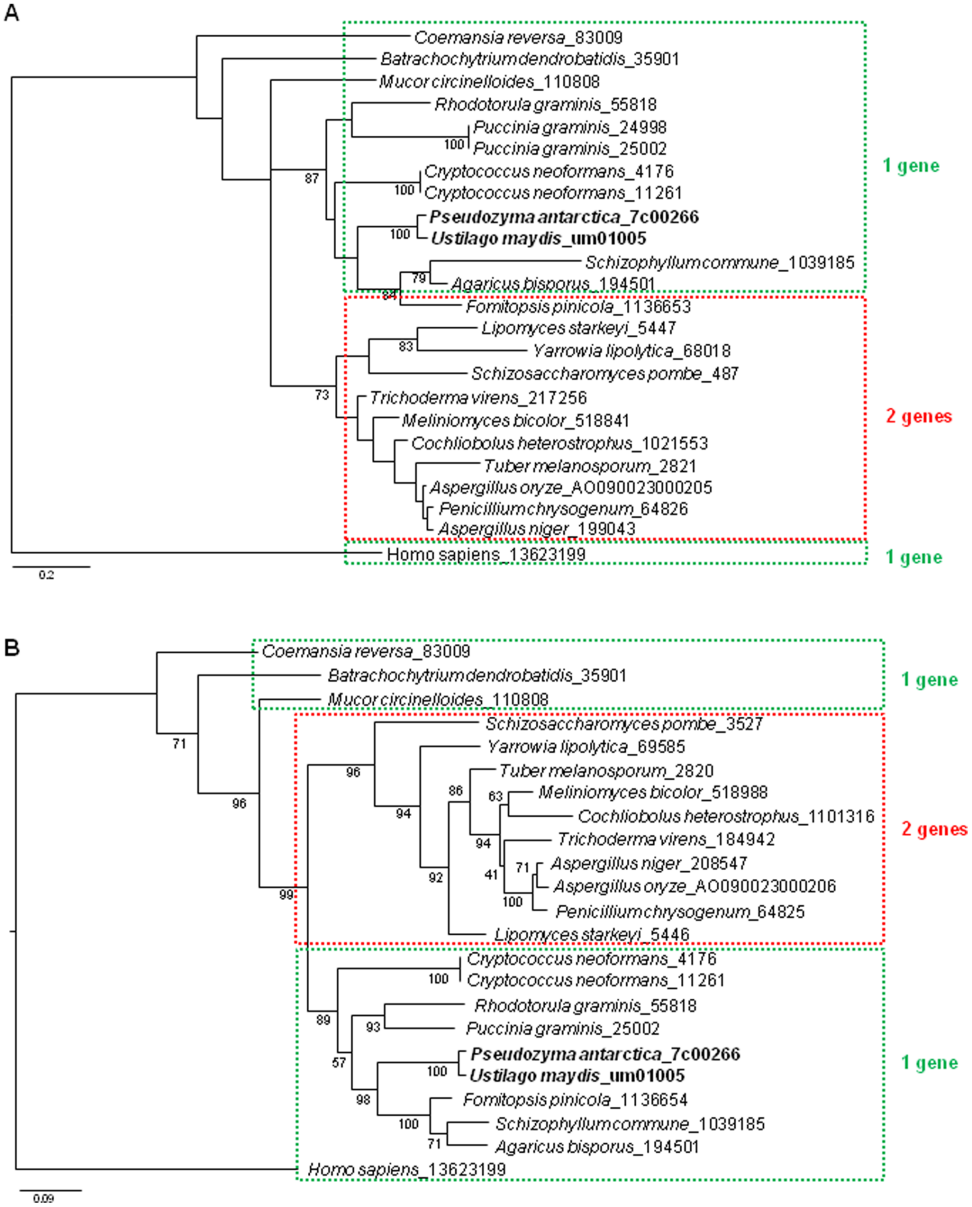
Phylogenetic analysis of the ATP/citrate lyase. The phylogenetic tree was constructed for the N-terminus using amino acid sequences of ATP-grasp domain (A) and the C-terminus using amino acid sequences of the three domains, CoA binding, CoA-ligase and Citrate synthase, (B) of ACL genes. The regions of the domains were determined on the basis of Pfam database.

The 18 Mb genome sequence including 6,543 genes of *P. antarctica* T-34 was reported previously [31]. Of the total 4201 genes listed on the basis of the KOG classification (Table S1), oleaginous character of the yeast was found by comparing the encoded genes with those of a model yeast *Saccharomyces cerevisiae*, which has the similar number of the genes. The 219 genes were categorized into the lipid transport and metabolism classification, while 140 genes were in the same category in *S. cerevisiae*, indicating *P. antarctica* potentially possesses the expanded lipid metabolism pathway (Fig. 2, Table S1). Among the genes listed in Table S2, there are many for peroxisomal and mitochondrial beta-oxidation in *P. antarctica* (42 genes) compared with *S. cerevisiae* (9 genes). Surprisingly, a gene encoding ATP/citrate liase (ACL), which has been known that oleaginous species could synthesize acetyl-CoA via ACL for fatty acid synthesis, was found in *P. antarctica* genome, besides the genes is not conserved in non-oleaginous species such as *S. cerevisiae* [37], [48], [49]. Additionally, the single gene of *P. antarctica* (PANT_7c00266) conserved the four domains, *i.e*., ATP-grasp domain, CoA binding domain, CoA-ligase, and citrate synthetase, as the same as human gene, whereas ascomycetous oleaginous microbes such as *Aspergillus oryzae* conserve the two genes encoding the ACL subunits (Fig. 3). There is no ACL homologous in *Saccharomycotina*, except oleaginous species of *Yarrowia lipolytica* and *Lipomyces starkeyi*. Further phylogenic tree based on the amino acid sequences of oleaginous species and human displayed that the ACL genes of *P. antarctica* (PANT_7c00266) located in a clade of the Basidiomycota (Fig. 4), possessing single genes corresponding to human. The genes encoding the subunits of ACL in the Ascomycota, e.g., *Aspergillus oryzae*, *Yarrowia lipolytica* and *Lipomyces starkeyi,* are localized in the same clade on the phylogenic trees created with both N-terminal region (ATP-grasp domain) and C-terminal region (CoA binding domain, CoA-ligase, and citrate synthetase). This result suggests the molecular phylogenic trees of ACL are well identical to the result of taxonomical studies reported previously.

### Comparison with *Ustilago maydis* Genome

**Figure 5:**
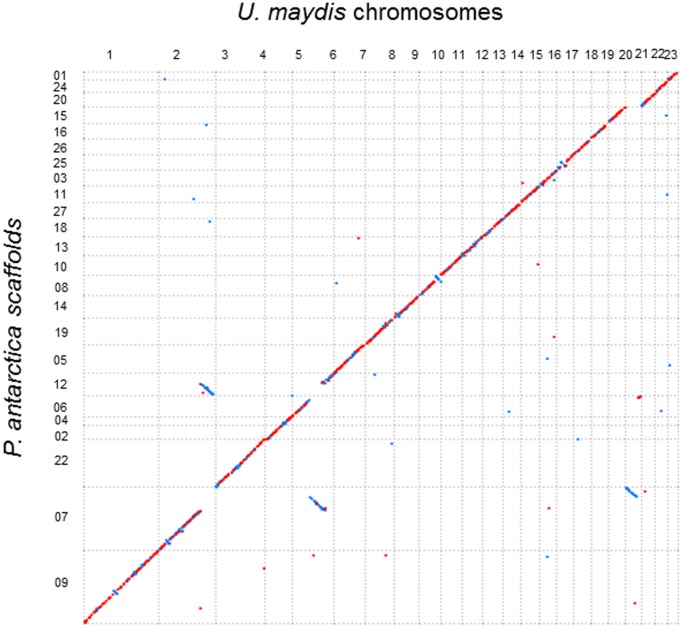
Comparison of *P. antarctica* and *U. maydis* genomes. Synteny (diagonal lines) of predicted protein-encoding genes on 27 scaffolds of *P. antarctica* compared to those on 23 chromosomes of *U. maydis*. The forward matches are displayed in red, while the reverse matches are displayed in cyan.

*P. antarctica* is taxonomically related to *U. maydis*, which is a pathogenic basidiomycetous fungus that infects maize [1], [2]. The genome size of *U. maydis* (20.5 Mb) [50] is larger than that of *P. antarctica* (18.0 Mb, predicted from the size of total length of scaffolds). *U. maydis* possesses 23 chromosomes, while the genome sequence of *P. antarctica* was assembled into 27 scaffolds [31]. Chromosomal segments that retain orthologous genes can be assigned between *P. antarctica* and *U. maydis*. The genomes of *P. antarctica* and *U. maydis* exhibit a remarkable degree of synteny (Fig. 5) despite an average amino acid identity of predicted proteins of only 72.0%.

The orthologous genes (e-value ≤1e-5) between *P. antarctica* and *U. maydis* were analyzed by bidirectional best hit analysis of amino acid sequences. The 5,987 of the *P. antarctica* genes showed significant homology to gene(s) of *U. maydis*, and 5,707 of the *U. maydis* genes showed significant homology to gene(s) of *P. antarctica*. Accordingly, 5,482 genes were estimated as the orthologous genes between *P. antarctica* and *U. maydis* (Table S3).

Genes encoding polysaccharide hydrolases, polysaccharide lyases and pectin esterases are considered to be signatures of necrotrophic fungi that use such enzymes to degrade living and dead plant tissue. *U. maydis* contains only 33 such hydrolytic enzymes, in contrast with 138 and 103 for *M. grisea* [51] and *F. graminearum* respectively. Similarly *P. antarctica* conserved only 41 of the genes encoding, polysaccharide hydrolases, polysaccharide lyases, and pectin esterases (Table S4). The minimal set of hydrolytic enzymes found in *P. antarctica* and *U. maydis* seems perfectly in line with its biotrophic lifestyle, in which damage to the host should be minimized and the release of cell wall fragments, which often trigger plant defense responses, has to be avoided [52].

*U. maydis* contains 13 of the gene clusters that appear to share characteristics with bacterial pathogenicity islands, in that they both express secreted gene products involved in pathogenicity [53], and express the gene products involved in pathogenicity [50]. Cluster 5B containing 5 genes, whose deletion shows non-pathogenic phenotype in *U. maydis*, is conserved at a high genetic similarity in non-pathogenic *P. antarctica* (Table S5). On the other hand only 14 genes of cluster 19A containing 28 genes, whose deletion markedly reduces the virulence in *U. maydis*, are conserved in *P. antarctica*. The genes on the other clusters are also conserved in *P. antarctica*, although some genes are lacking. Consequently, the *P. antarctica* genome partially conserves orthologous genes corresponding to gene clusters related to the plant invasion in *U. maydis*. These genes in the clusters conserved in *P. antarctica* are likely to play a role in living on the leaf surface in the non-pathogenic species, *P. antarctica*.

These results suggest that the genome organization and gene sets of *P. antarctica* are highly identical to *U. maydis*, except for the critical genes for plant pathogenicity of *U. maydis*.

### Transcriptome Analysis of *P. antarctica*

The production efficiency for MEL from vegetable oils by *P. antarctica* has been shown to be higher than that by *U. maydis*, though they share significant segments of chromosomes. Therefore, we assumed that the gene expression profiles between the fungal species should be different under the oily conditions. To reveal the transcriptomic profiles of *P. antarctica* under the conditions of high MEL production, we designed a DNA microarray of both the species, *P. antarctica* T-34 (the accession no. DF196767 to DF196793) and *U. maydis* UM521 (MIPS *Ustilago maydis* DataBase, http://mips.helmholtz-muenchen.de/genre/proj/ustilago), and analyzed the transcriptomes. These species were cultured in a medium containing 5% soybean oil or 10% glucose as sole carbon source, and 0.5% sodium nitrate as the main nitrogen source for 7 days, the glycolipids were extracted with an equal amount of ethyl acetate, and then MEL production was confirmed by TLC analysis using the anthrone staining method. *P. antarctica* T-34 produced large amounts of MELs with soybean oil after 7 days. During this cultivation, almost all of the soybean oil had been consumed by *P. antarctica* T-34 and the cell growth had reached a stationary phase at 3 days cultivation. *P. antarctica* T-34 is also able to produce MELs from glucose (Fig. S1). In contrast, *U. maydis* UM521 produced low amounts of MELs, and almost all of the soybean oil remained in the culture.

**Figure 6:**
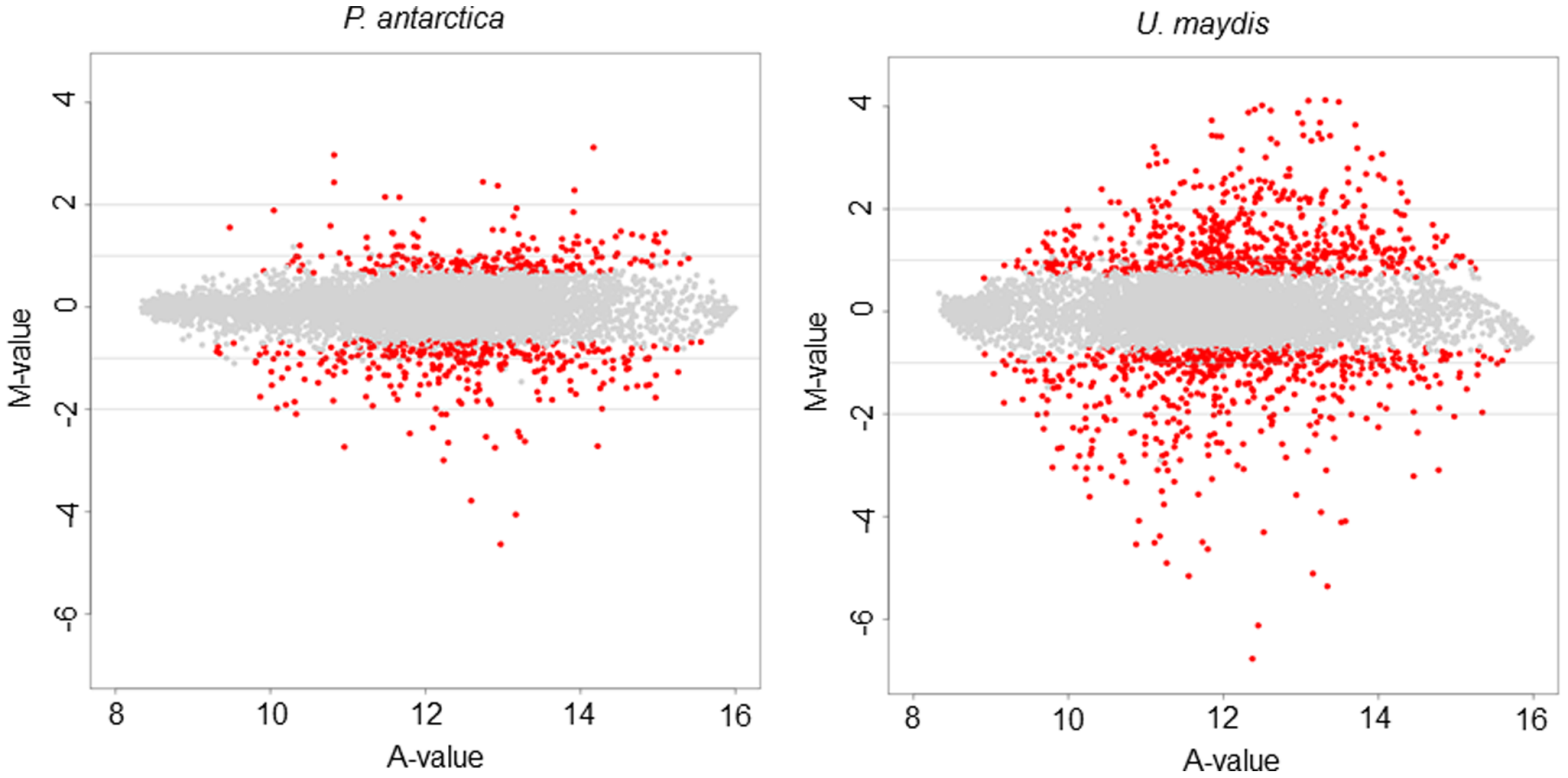
Distribution of the gene expression of *P. antarctica* and *U. maydis*. Scatter plots showing the distribution of the average of logarithmic signal intensities (A-value, ordinate) and logarithmic induction ratio (M-value, abscissa) of gene transcription compared under the conditions of vegetable oil and glucose in *P. antarctica* and *U. maydis*. Each gene is represented as a dot. The red dots show the statistically significant up- and down-regulated genes (p-val <0.005).

Transcriptional expression analysis was performed on a two-color microarray platform using the dye swap method. The relative abundances of each transcript were compared between cells grown in the presence of soybean oil and of glucose as sole carbon source. The logarithmic induction ratio (M-value), the average of the logarithmic signal intensities (A-value), and statistics for differently expression were obtained from the DNA microarray experiments after processing the raw data (Table S6). As shown in Figure 6, the distribution of the logarithmic induction ratio of *P. antarctica* under the oily conditions was less than that of *U. maudis*. Therefore the gene expression of *U. maydis* would be regulated more depending on the carbon source in comparison to *P. antarctica*.

### Identification of Differentially Expressed Genes

The significance of the different expression was determined by comparing the relative transcript abundance of each gene between the condition with soybean oil and glucose (Table S7). In *P. antarctica*, 755 genes are expressed at significantly different levels (p-val <0.005), and of the genes, 92 genes (12.2%) do not have significant homology with known sequences in public databases. On the other hand, in *U. maydis*, 1449 genes are expressed at significantly different levels, and of the genes, 588 genes (40.6%) do not have significant homology to the same databases. Additionally, of the differentially expressed genes, 230 genes in *P. antarctica* (30.5%) and 424 genes in *U. maydis* (29.3%) are assigned to the referential canonical pathway in Kyoto Encyclopedia of Genes and Genomes (KEGG) database. Consequently, in *U. maydis*, the gene expression of unknown function would be more up-regulated with vegetable oil.

To further characterize the gene sets enriched in the conditions, the transcriptome was analyzed by the gene set enrichment analysis. The top 50 enriched gene sets are presented in Table S8. In *P. antarctica*, the 17 gene sets showing highly different expression (FDR q-value <0.01) under oily conditions, 14 sets are responsible for fatty acid metabolism, while the gene set enriched under the glucose conditions are mainly related to carbohydrate metabolism. On the other hand, in *U. maydis*, the gene sets picked as enriched are less than *P. antarctica*. These results suggest that the unknown genes responsible for plant pathogenicity may be induced in *U. maydis* under the oily conditions, while the gene sets for fatty acid metabolism are predominantly expressed in *P. antarctica*, allowing to adapt to the oily conditions.

### The Gene Cluster for Biosynthesis of Mannosylerythritol Lipids

**Figure 7:**
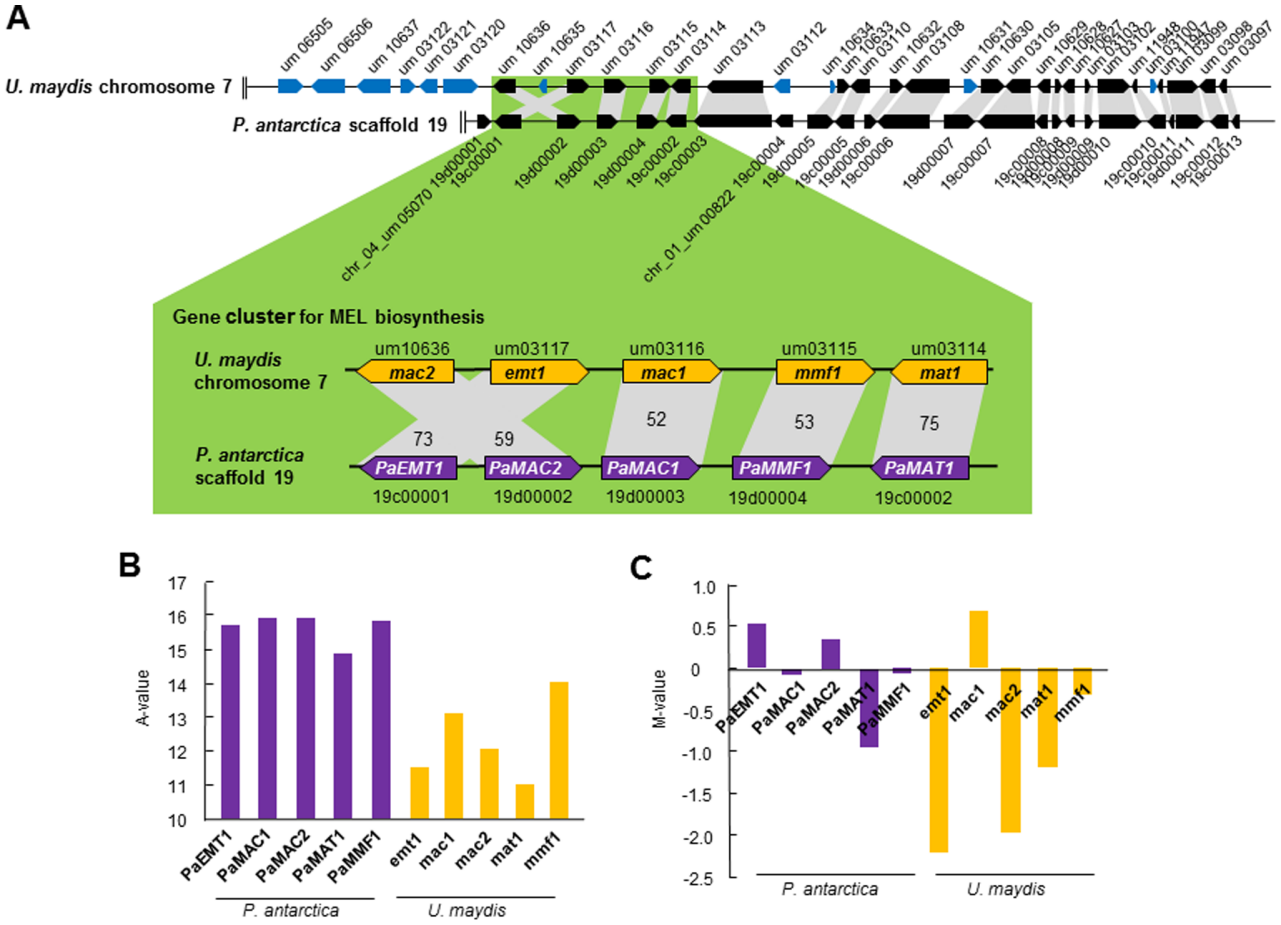
MEL biosynthesis cluster of *P. antarctica*. (A) The gene cluster for MEL biosynthesis in *P. antarctica* is located on scaffold 19: *PaEMT1* (PANT_19c00001), *PaMAC1* (PANT_19d00003), *PaMAC2* (PANT_19d00002), *PaMMF1* (PANT_19d00004), and *PaMAT1* (PANT_19c00002). The gene cluster for MEL biosynthesis in *U. maydis* are *emt1* (um03117), *mac1* (um03116), *mac2* (10636), *mmf1* (um03115), and *mat1* (um03114) on chromosome 7. *Blue* represents *U. maydis*-specific genes. *Black* indicates orthologous genes. 19d00001 and 19c00004 are orthologous genes to um05070 (chromosome 4 of *U. maydis*) and um00822 (chromosome 1 of *U. maydis*), respectively. The gene expression (A-value) (B) and induction ratio (M-value) (C) of gene transcription compared under the conditions of vegetable oil and glucose in *P. antarctica* and *U. maydis*.

The gene cluster responsible for MEL biosynthesis in *P. antarctica* was found in the vicinity of the terminal of scaffold 19, corresponding to chromosome 7 of *U. maydis* (Fig. 7A) [31]. The predicted amino acid sequences encoded by the five genes consisting the cluster, *PaEMT1* (erythritol/mannose transferase), *PaMAC1*, *PaMAC2* (mannose/acyl transferase), *PaMMF1* (mannosylerythritol lipids transporter), and *PaMAT1* (mannose/acetyl transferase) of *P. antarctica* share a high identity of 73, 59, 52, 75, and 53% with those of *U. maydis* respectively. Of the genes on the cluster, *PaEMT1* and *PaMAC1* of *P. antarctica* are rearranged compared with *U. maydis*. Scaffold 19 of *P. antarctica* lacked 6 of the unknown genes near the MEL biosynthesis genes on the terminal at chromosome 7 of *U. maydis*: the conserved hypothetical *Ustilago*-specific protein (um06505), the conserved hypothetical protein (um06506, um10637, um03120), and the binding protein related to beta-1,3-glucan (um03122, um03121). In contrast, the reverse side of genes toward the center of the chromosome from the MEL biosynthesis cluster highly conserved the genes orthologous between *P. antarctica* and *U. maydis*.

Particularly, the average of the logarithmic signal intensities of *PaEMT1*, *PaMAC1*, *PaMAC2*, *PaMMF1*, and *PaMAT1* were 15.7, 15.9, 15.9, 15.9, and 14.9 respectively, corresponding to an average of 15.7±0.43 indicating very high expression among genes (see Fig. 6 for the distribution). Hence the relative expression intensity of the genes on MEL biosynthesis cluster in *P. antarctica* was high by DNA microarray (Fig. 7B). The induction ratio described as binary logarithm of *PaEMT1*, *PaMAC1*, *PaMAC2*, *PaMMF1*, and *PaMAT1* were 0.54, −0.05, 0.34, −0.003, and −0.95 respectively, corresponding to an average of −0.02±0.57. In contrast, the induction ratio described as binary logarithm of *emt1* (um03117), *mac1* (um03116), *mac2* (10636), *mmf1* (um03115), and *mat1* (um03114) in *U. maydis* were −2.23, 0.68, −1.97, −0.33, and −1.19 respectively (Fig. 7C), corresponding to an average of −1.00±1.20. Therefore, the gene cluster in *P. antarctica* is highly expressed regardless of whether the carbon source is soybean oil or glucose, while the expression of the gene cluster in *U. maydis* was suppressed by vegetable oil.

### Update of EST Annotation for the Frequently Expressed Genes during MEL Production

Our previous EST analysis intended to obtain the genes for MEL biosynthesis revealed that there are many unknown genes with high expression under MEL production conditions [54]. The information of *P. antarctica* genome annotation presented here allow us to update the previous EST data (Table S9), demonstrating that the EST contigs of 14 (information storage and processing), 25 (cellular processes), 36 (metabolism), and 53 (poorly characterized) were frequently expressed when the species was grown in a medium containing soybean oil as its sole carbon source. Also, EST contigs contained 4 genes of MEL biosynthesis cluster as frequently expressing genes: PA_004, PA_028, PA_141, and PA_085 identified as *PaEMT1* (PANT_19c00001), *PaMAC1* (PANT_19d00003), *PaMAC2* (PANT_19d00002), and *PaMMF1* (PANT_19d00004) respectively. On the basis of the present transcriptomic analysis, the average expression level of all of the EST contigs were 13.5±1.69, indicating these contigs contain genes which frequently express. It is interesting the average of the induction ratio of the EST contigs 0.15±0.51, indicating constant high expression regardless of whether the carbon source is soybean oil or glucose. These results strongly support that the transcriptional profile presented here is well corresponding to the previous EST results.

## Discussion

*P. antarctica* is known to be taxonomically related to *U. maydis*, which is a smut pathogen and forms extracellular glycolipids [20], [55]. The genome information of *U. maydis* has been described in detail, and genetic tools for its molecular biological study as a model microorganism of plant pathogens have also been developed [50]. In contrast, *P. antarctica* is potentially a model microorganism of a non-pathogenic phyllosphere yeast [10] besides its role as a promising MEL producer in industry [17], although genetic information of *P. antarctica* has been limited. The *P. antarctica* genomic analysis presented here provides not only the genomic properties of *P. antarctica* but also allows us to demonstration of the comparative transcriptomic analysis between *P. antarctica* and *U. maydis*. We found that the phyllosphere basidiomycetous yeast has a high potential to produce MELs as a novel oleaginous yeast which might be relevant to its life style.

The comparison of the encoded genes of a Basidomycota yeast *P. antarctica* with those of an Ascomycota yeast *S. cerevisiae* which possesses similar number of genes has revealed interesting characteristics. The Basidomycota yeasts possess a greater number of genes relating to lipid metabolism and secondary metabolism while the Ascomycota yeast possesses more of genes related to carbohydrate metabolism (Fig. 2). We then focused our attention on the gene encoding ACL because this enzyme has been known to be conserved among oleaginous species and involved in effective supply of acetyl-CoA for fatty acid biosynthesis [37]. Interestingly, the single gene of *P. antarctica* contains all of the four domains important for the enzymatic reaction the same as the human gene. On the other hand, ascomycetes fungi possess the 2 genes encoding the subunits of the enzyme. The non-oleaginous yeast *S. cerevisiae* do not have the gene encoding the enzyme. Therefore, the genomic characteristics indicate the oleaginous nature of the fungus which might be shared among fungi and animal species except for the most Ascomycota fungi.

The *P. antarctica* genome has highly conserved synteny compared to *U. maydis* (Fig. 5), and 83.6% of its genes are orthologous to those of *U. maydis* (Table S3). There are fewer sets of gene families encoding plant cell wall degrading enzymes in *P. antarctica* similar to *U. maydis* than in the other necrotrophic fungi, *e.g.*, *M. grisea* and *F. graminearum* (Table S4). The gene clusters responsible for plant invasion of *U. maydis* were also poorly conserved in the genome of non-pathogenic *P. antarctica* (Table S5). The gene clusters have recently been also found in *Sporisorium reilianum*, which is a maize pathogenic fungus related to *U. maydis*, by comparative genomics [56]. In comparison to these plant pathogenic fungi, non-pathogenic *P. antarctica* particularly conserved fewer genes corresponding to cluster 19A, which markedly decreased the virulence of *U. maydis* by disruption of these genes: *P. antarctica* conserved only 14 of the genes, including 6 of the genes orthologous to *U. maydis*, while *U. maydis* and *S. reilianum* possess 28 and 33 of the genes in the cluster respectively. On the other hand, genes in clusters 1A, 2B, 3A, 5B, 9A, and 10A of *U. maydis* are likely to be highly conserved in *P. antarctica*. The deficiency in these genes of *P. antarctica* seems to allow them to localize the surface of leaves without virulence by the same mechanism as *U. maydis*. Further comparative genomics of these closely related species may enable us to identify the new virulence genes [56]. Accordingly, the results of the genome sequence comparison display the difference between the plant pathogen and the non-pathogenic yeast, but nevertheless the genome sequence of the non-pathogenic *P. antarctica* is very similar to that of the plant-pathogenic *U. maydis*.

Various MEL producers were isolated from the surfaces of leaves, and concentrated at the genus *Pseudozyma*, which formed various types of MELs including di-acetylated MEL (MEL-A), mono-acetylated MEL (MEL-B or MEL-C) [57]. All of these *Pseudozyma* strains have no plant pathogenesis. These facts suggest that *Pseudozyma* strains possibly produce MELs to stay on the surface of leaves without virulence. Previously, analysis of the gene disrupted mutants lacking MEL formation indicated that MELs would not relate to plant pathogenesis of *U. maydis* [18], [19]. Further genome sequencing and comparison of these MEL producers may provide insight into the genes related to plant virulence or infection, and help us to resolve in detail the questions of why these phyllosphere strains produce extracellular glycolipids.

In *P. antarctica*, fatty acids derived from vegetable oil by enzymatic degradation are processed via chain shortening pathways like beta-oxidation, and then the intermediates are directly transferred into mannosylerythritol, forming MELs [27]. Hence *P. antarctica* efficiently provides fatty acids into the pathway for MEL biosynthesis from supplied vegetable oil. From the present results of transcriptome, the gene sets responsible for fatty acid metabolism in *P. antarctica* on the basis of gene set enrichment analysis showed higher expression compared with *U. maydis*. These results correspond to the fact that *P. antarctica* possibly has an advantageous for adaptation to the oily conditions based on the present result that *P. antarctica* have a lot of genes categorized in fatty acid transport and metabolism classification compared with *S. cerevisiae*. On the contrary, *P. antarctica* have few genes categorized in carbohydrate transport and metabolism classification compared with *S. cerevisiae*. Taken together, these findings may show the difference in each habitat in nature: *P. antarctica* is a phyllosphere yeast locating on the surface of leaves in which cuticle layer of phylloplane consists of polyester of fatty acids [58], *U. maydis* invades as a plant pathogenic fungi forming a corn smut containing glucose, fructose and sucrose as the main soluble sugars.

The gene cluster for MEL biosynthesis of *P. antarctica* was found on the basis of the amino acid identity (Fig. 7) [31]. In the MEL biosynthetic pathway, the hydrophobic part, mannosylerythritol, is initially formed by the reaction of an erythritol/mannose transferase (*PaEMT1*), and then MELs produced via the reactions of acyl transferases (*PaMAC1* and *PaMAC2*) and an acetyl transferase (*PaMAT1*) [19]. MELs are possibly secreted by the putative transporter (*PaMMF1*). Of the 5 genes, *PaEMT1* of *P. antarctica* has been characterized by developing the gene deletion mutant, lacking the MEL production property [59]. This result supports the conclusion that the gene cluster of *P. antarctica* works in the same way as that of *U. maydis*. The 5 genes of the cluster are highly expressed in *P. antarctica* regardless of the carbon source, vegetable oil or glucose, whereas these genes in *U. maydis* were suppressed by oil. Therefore *P. antarctica* may be able to produce MELs under oily conditions due to the high expression of the gene cluster for MEL biosynthesis, while the gene cluster expression of *U. maydis* is sensitive to various nutrients, including carbon and nitrogen sources [18], [19], [20]. It is important to decrease production costs by using raw materials to expand industrial application, and these characteristics of *P. antarctica* show it has the advantage for mass production of MELs of being able to use a variety of raw materials such as crude vegetable oils.

The basidiomycetous yeast species of the genus *Pseudozyma* are remarkable industrial microorganisms for extracellular production of various fatty acids, however, genetic studies, including gene manipulation, have been limited compared with ascomycetous species, *e.g.*, *Saccharomyces cerevisiae*, *Schizosaccharomyces pombe*, and *Aspergillus oryzae*. The present genetic study of *P. antarctica*, a highly oil-assuming and glycolipid-producing yeast, should provide novel aspects for not only development of useful industrial strains by genetic modification, but also understanding of phytopathological mechanisms, including the molecular mechanism of invasion into plant cells and molecular evolution of these basidiomycetous genera.

## Conclusions

The genomic and transcriptomic analysis here hints at the great potential of *P. antarctica* as an industrial microorganism for producing functional bio-based materials. The oleaginous nature of *P. antarctica* was explored for the first time on the basis of genomic analysis. By differentially expressed genes and gene set enrichment analysis, the genes related to fatty acid metabolism was significantly up-regulated under the conditions with vegetable oil. The gene cluster for MEL biosynthesis was highly expressed regardless of whether the carbon source is glucose or soybean oil in *P. antarctica*. These practical characteristics allow the yeast to produce large amounts of extracellular glycolipids, MELs, and are useful in developing novel bio-based materials like functional lipids. Furthermore, these results that the gene expression profiles are different between *P. antarctica* and *U. maydis*, while on the contrary the genome organization and gene contents are nearly the same between the 2 species, would be relevant to its oleaginous character. The availability of the *P. antarctica* genome sequence will accelerate efforts to advance studies and technologies for biotechnology.

## Supporting Information

Figure S1 **Production of MELs, oil-assimilation, and cell growth of** ***P. antarctica*** **and** ***U. maydis*** **with the presence of glucose or soybean oil as carbon source.** (A) *P. antarctica* T-34 and *U. maydis* UM521 were cultivated in 20 ml of the medium containing 5% soybean oil or 10% glucose as sole carbon source at 25°C. MELs were extracted from the cultured medium using an equal amount of ethyl acetate, and the organic solvent fraction (12 µl) was spotted on a TLC plate. The spots were visualized with the anthrone reagent. The purified MELs, *i.e.*, MEL-A, MEL-B, and MEL-C, were used as the standard. (B) To display clearly the production of MELs by *U. maydis*, the fractions (12 ml) from the culture grown with glucose for 3 days were concentrated by evaporation, dissolved in 2 ml of ethyl acetate, and spotted on TLC plate (6 µl). (C) The amounts of di-acylated MELs were quantified by HPLC (blue column). The purified MELs, *i.e.*, MEL-A, MEL-B and MEL-C, were used as the standard. Dry cell weight is represented by a hollow column.
(PDF)

Tables S1 **Comparison of the number of genes between** ***P. antarctica*** **and** ***S. cerevisiae*** **on the basis of KOG classification.**
(PDF)

Tables S2 ***P. antaractica*** **genes related to predicted lipogenesis pathway, homologous to** ***S. cerevisiae*** **genes.**
(PDF)

Tables S3 **Analysis of the orthologous genes between** ***P. antarctica*** **and** ***U. maydis*** **.**
(PDF)

Table S4 **Putative plant cell wall-degrading enzymes in** ***P. antarctica*** **and other fungi.**
(PDF)

Table S5 **Gene clusters for secreted proteins in** ***P. antarctica*** **.**
(PDF)

Table S6 **Transcriptome analysis of** ***P. antarctica*** **and** ***U. maydis*** **under the oily conditions.**
(PDF)

Table S7 **Differentially expressed genes analysis.**
(PDF)

Table S8 **The top 50 most enriched gene sets.**
(XLSX)

Table S9 **Re-annotation of the previous EST clones using the results of the present genome annotation.**
(XLS)
