# Supplementary figures and images for: Genome and Transcriptome Analysis of the Basidiomycetous Yeast Pseudozyma antarctica Producing Extracellular Glycolipids, Mannosylerythritol Lipids
Source: PLoS One. 2014 Feb 24;9(2):e86490. doi: 10.1371/journal.pone.0086490 (PMC3933340; doi:10.1371/journal.pone.0086490)

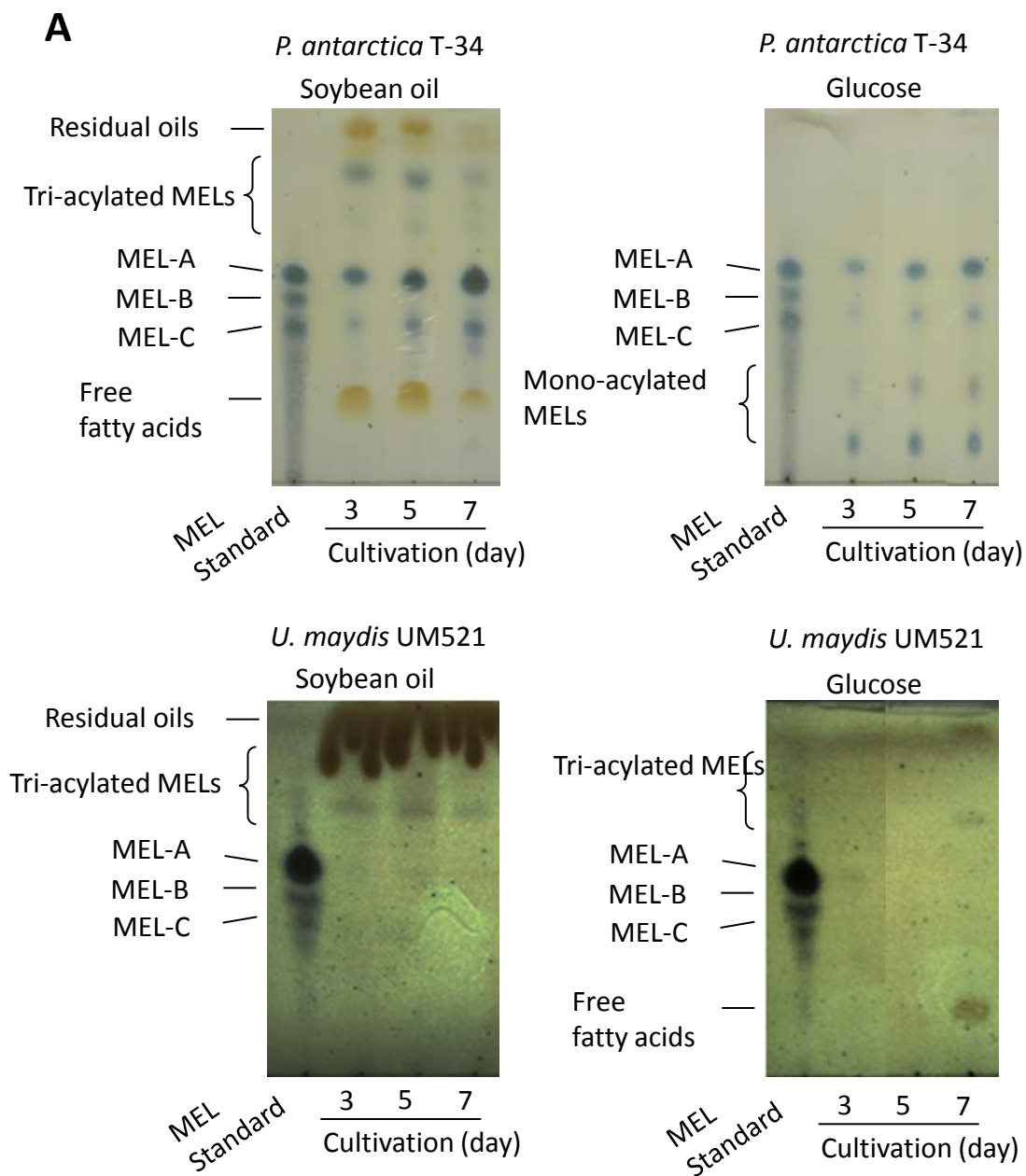

**B**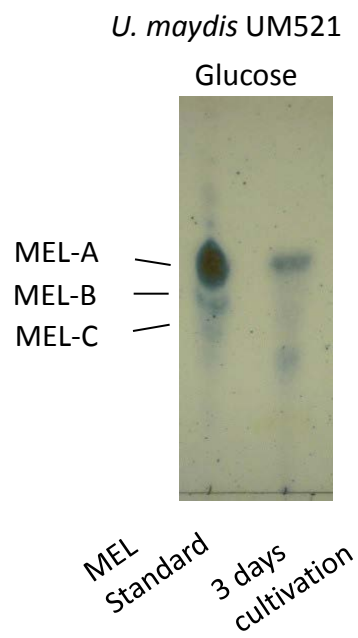**C**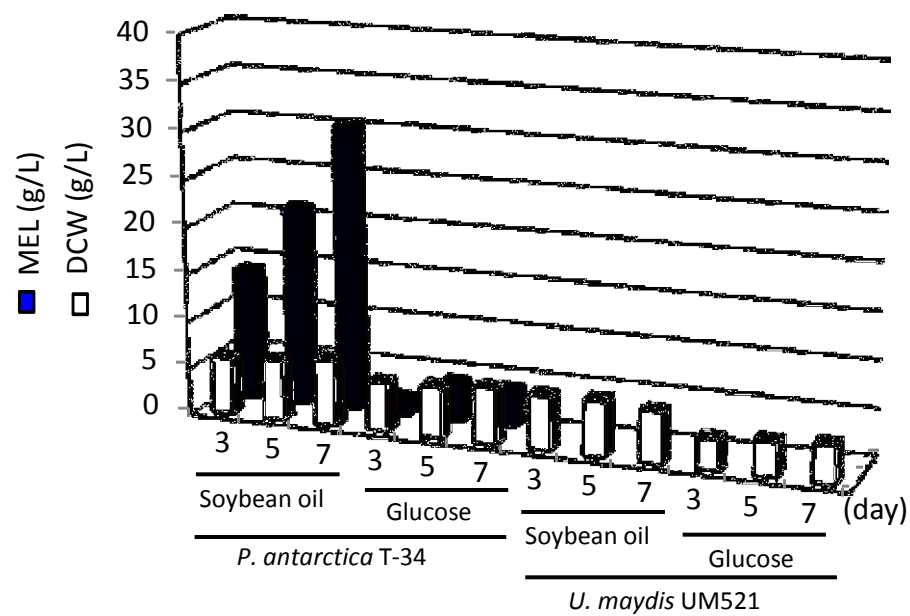

Supplement: Figure S1 — Production of MELs, oil-assimilation, and cell growth of P. antarctica and U. maydis with the presence of glucose or soybean oil as carbon source. (A) P. antarctica T-34 and U. maydis UM521 were cultivated in 20 ml of the medium containing 5% soybean oil or 10% glucose as sole carbon source at 25°C. MELs were extracted from the cultured medium using an equal amount of ethyl acetate, and the organic solvent fraction (12 µl) was spotted on a TLC plate. The spots were visualized with the anthrone reagent. The purified MELs, i.e., MEL-A, MEL-B, and MEL-C, were used as the standard. (B) To display clearly the production of MELs by U. maydis, the fractions (12 ml) from the culture grown with glucose for 3 days were concentrated by evaporation, dissolved in 2 ml of ethyl acetate, and spotted on TLC plate (6 µl). (C) The amounts of di-acylated MELs were quantified by HPLC (blue column). The purified MELs, i.e., MEL-A, MEL-B and MEL-C, were used as the standard. Dry cell weight is represented by a hollow column. (PDF) [file pone.0086490.s001.pdf]
