# Supplementary material for: Genome and Transcriptome Analysis of the Basidiomycetous Yeast Pseudozyma antarctica Producing Extracellular Glycolipids, Mannosylerythritol Lipids
Source: PLoS One. 2014 Feb 24;9(2):e86490. doi: 10.1371/journal.pone.0086490 (PMC3933340; doi:10.1371/journal.pone.0086490)
Supplement: Tables S1 — Comparison of the number of genes between P. antarctica and S. cerevisiae on the basis of KOG classification. (PDF) [file pone.0086490.s002.pdf]

Supplementary Table S1

Comparison of the number of genes between *P. antarctica* and *S. cerevisiae* on the basis of KOG classification

#	Description	*S. cerevisiae* The number of genes	*P. antarctica* The number of genes
	Metabolism		
E	Amino acid transport and metabolism	215	204
G	Carbohydrate transport and metabolism	285	172
H	Coenzyme transport and metabolism	90	78
P	Inorganic ion transport and metabolism	144	91
I	Lipid transport and metabolism	140	219
F	Nucleotide transport and metabolism	87	63
Q	Secondary metabolites biosynthesis, transport and catabolisı	67	124
C	Energy production and conversion	200	206
	Cellular processes and signaling		
D	Cell cycle control, cell division, chromosome partitioning	199	139
N	Cell motility	1	3
M	Cell wall/membrane/envelope biogenesis	69	48
Z	Cytoskeleton	104	95
V	Defense mechanisms	45	31
W	Extracellular structures	18	4
U	Intracellular trafficking, secretion, and vesicular transport	351	256
Y	Nuclear structure	90	24
O	Posttranslational modification, protein turnover, chaperones	471	373
T	Signal transduction mechanisms	342	291
	Information strage nad processing		
B	Chromatin structure and dynamics	106	78
A	RNA processing and modification	219	203
L	Replication, recombination and repair	174	164
K	Transcription	312	216
J	Translation, ribosomal structure and biogenesis	381	287
	Poorly characterized		
S	Function unknown	309	244
R	General function prediction only	605	588
