# Supplementary material for: Genome and Transcriptome Analysis of the Basidiomycetous Yeast Pseudozyma antarctica Producing Extracellular Glycolipids, Mannosylerythritol Lipids
Source: PLoS One. 2014 Feb 24;9(2):e86490. doi: 10.1371/journal.pone.0086490 (PMC3933340; doi:10.1371/journal.pone.0086490)
Supplement: Tables S2 — P. antaractica genes related to predicted lipogenesis pathway, homologous to S. cerevisiae genes. (PDF) [file pone.0086490.s003.pdf]

Supplementary Table S2

P. antaractica genes related to predicted lipogenesis pathway, homologous to *S. cerevisiae, A. oryze* and *R. oryzae* genes

Predicted lipogenesis pathway	EC_name	*S. cerevisiae* genes	*A. oryze* genes *R. oryzae* gene	*P. antarctica* genes; Cover_ratio>0.8	Ref
**pentose phosphate pathway**					
glucose-6-phosphate 1-dehydrogenase	EC1.1.1.49	YNL241C		3d00023	YC*
6-phosphogluconolactonase	EC3.1.1.31	YHR163W		10d00035	YC*
6-phosphogluconolactonase	EC3.1.1.31	YGR248W		10d00035	YC*
phosphogluconate dehydro-genase (major)	EC1.1.1.44	YHR183W		5c00123	YC*
phosphogluconate dehydro-genase (minor)	EC1.1.1.44	YGR256W		5c00123	YC*
ribose 5-phosphate isomerase	EC5.3.1.6	YOR095C		18c00079	YC*
ribulose-5-phosphate-3-epimerase	EC5.1.3.1	YJL121C		26c00050	YC*
transketolase (major)	EC2.2.1.1	YPR074C		1c00048	YC*
transketolase (minor)	EC2.2.1.1	YBR117C		1c00048	YC*
transaldolase	EC2.2.1.2	YLR354C		18c00087	YC*
**glycolysis**					
Hexokinase	EC2.7.1.1	YFR053C		19d00014, 6d00069	YC*
Hexokinase	EC2.7.1.1	YGL253W		19d00014, 6d00069	YC*
glucokinase	EC2.7.1.2	YCL040W		6d00069, 19d00014	YC*
glucose-6-phosphate isomerase	EC5.3.1.9	YBR196C		13c00058	YC*
phosphofructokinase (alpha)	EC2.7.1.11	YGR240C			YC*
phosphofructokinase (beta)	EC2.7.1.11	YMR205C			YC*
fructose-bisphosphatase	EC3.1.3.11	YLR377C		5d00053	YC*
aldolase A fructose-bisphosphate	EC4.1.2.13	YKL060C		9d00334	YC*
glyceraldehyde 3-phosphate dehydrogenase (stati	EC1.2.1.12	YJL052W		12c00136	YC*
glyceraldehyde 3-phosphate dehydrogenase	EC1.2.1.12	YJR009C		12c00136	YC*
glyceraldehyde 3-phosphate dehydrogenase	EC1.2.1.12	YGR192C		12c00136	YC*
phosphoglycerate kinase	EC2.7.2.3	YCR012W		26c00011	YC*
triose-phosphate isomerase	EC5.3.1.1	YDR050C		14d00042	YC*
phosphoglycerate mutase	EC5.4.2.1	YKL152C		15d00053	YC*
enolase	EC4.2.1.11	YGR254W		14d00015	YC*
enolase	EC4.2.1.11	YHR174W		14d00015	YC*
pyruvate kinase	EC2.7.1.40	YOR347C		9d00083	YC*
pyruvate kinase	EC2.7.1.40	YAL038W		9d00083	YC*
lactate dehydrogenase	EC1.1.1.27	YDL174C		7d00256	YC*
lactate dehydrogenase	EC1.1.1.27	YDL178W		12d00055, 7d00256	YC*
phosphoenolpyruvate carboxykinase	EC4.1.1.32	YKR097W		2d00021	YC*
**fatty acid synthesis**					
acetyl-CoA carboxylase	EC6.4.1.2	YNR016C		27c00072	YC*
acetyl-CoA carboxylase (mito)	EC6.4.1.2	YMR207C		27c00072	YC*
fatty acid synthase (beta subunit)	EC2.3.1.86	YKL182W		19c00074	YC*
fatty acid synthase (alpha subunit)	EC2.3.1.86	YPL231W		15d00092, 19c00074	YC*
putative fatty acid sythetase (mito)	EC2.3.1.86	YOR221C			YC*
putative fatty acid sythetase (mito)	EC2.3.1.86	YER061C		15d00093, 19c00074, 15d00089, 1c00034	YC*
putative fatty acid sythetase (mito)	EC2.3.1.86	YKL055C		8d00015	YC*
fatty acid elongase	EC2.3.1.-	YJL196C		13c00047	YC*
acyl-CoA thioesterase	EC3.1.2.27		AO090012000721	25c00010	Tamano [38]
**malate/pyruvate cycle**					
malic enzyme	EC1.1.1.40	YKL029C		7c00096	YC*
pyruvate carboxylase	EC6.4.1.1	YGL062W		7d00254	YC*
pyruvate carboxylase	EC6.4.1.1	YBR218C		7d00254	YC*
ATP-citrate lyase (subunit 1)	EC2.3.3.8		AO090023000205	7c00266	Tamano [38]
ATP-citrate lyase (subunit 2)	EC2.3.3.8		AO090023000206	7c00266	Tamano [38]
Acetyl-CoA synthetase	EC6.2.1.1	YLR153C		19d00026, 9c00217, 2d00022, 9d00141, 7c00326, 7d00009, 2d00039	YC*
Acetyl-CoA synthetase	EC6.2.1.1	YAL054C		19d00026, 9c00217, 2d00022, 7c00326, 2d00039	YC*
**tricarboxylic acid cycle**					
pyruvate dehydrogenase E1 (subB)	EC1.2.4.1	YBR221C		20d00018, 5d00012	YC*
pyruvate dehydrogenase (E1 alpha)	EC1.2.4.1	YER178W		10d00008, 25c00043	YC*
dihydrolipoamideacetyltransferase (E2)	EC2.3.1.12	YNL071W		9c00324, 14c00100	YC*
Dihydrolipoamide dehydrogenase (sub E3)	EC1.2.4.1	YFL018C		12c00126, 7c00197	YC*
citrate synthase	EC2.3.3.1	YNR001C		22c00098	YC*
citrate synthase (iso)	EC2.3.3.1	YPR001W		22c00098	YC*
aconitase	EC4.2.1.3	YLR304C		19d00127, 5c00060	YC*
aconitase	EC4.2.1.3	YJL200C		5c00060, 19d00127	YC*
isocitrate dehydrogenase (sub)	EC1.1.1.41	YNL037C		12c00053, 12d00048, 26d00008, 9c00226, 16c00084, 7d00148	YC*

isocitrate dehydrogenase (sub)	EC1.1.1.41	YOR136W		12d00048, 12c00053, 26d00008, 9c00226, 16c00084	YC*
2-oxoglutarate dehydrogenase	EC1.2.4.2	YIL125W		11c00079	YC*
dihydrolipoamidesuccinyltransferase	EC2.3.1.61	YDR148C		22d00047	YC*
succinyl-CoA ligase (subA)	EC6.2.1.4, EC6.2.1.5	YOR142W		22d00119, 22c00256	YC*
succinyl-CoA ligase (subB)	EC6.2.1.4, EC6.2.1.5	YGR244C		9d00182	YC*
succinate dehydrogenase (sub)	EC1.3.5.1	YKL148C		7d00183	YC*
succinate dehydrogenase (minor)	EC1.3.5.1	YJL045W		7d00183	YC*
succinate dehydrogenase (sub)	EC1.3.5.1	YLL041C		7c00348	YC*
succinate dehydrogenase (sub)	EC1.3.5.1	YKL141W		13c00067	YC*
succinate dehydrogenase (sub)	EC1.3.5.1	YDR178W			YC*
fumarase	EC4.2.1.2	YPL262W		9d00400	YC*
malate dehydrogenase	EC1.1.1.37	YKL085W		9c00225	YC*
malate dehydrogenase (cytosolic)	EC1.1.1.37	YOL126C		9c00225	YC*
malate dehydrogenase (peroxisome)	EC1.1.1.37	YDL078C		9c00225	YC*
**peroxisomal beta-oxidation**					
fatty acyl-coenzyme A synthetase (long chain)	EC6.2.1.3	YMR246W		10c00058	YC*
fatty acyl-coenzyme A synthetase (long chain)	EC6.2.1.3	YOR317W		10c00058, 16c00012	YC*
fatty acyl-coenzyme A synthetase (long chain)	EC6.2.1.3	YBR041W		5d00115, 22c00012, 7c00326, 6c00032	YC*
fatty acyl-coenzyme A synthetase	EC6.2.1.3	YER015W		16c00012, 10c00058	YC*
fatty acyl-coenzyme A synthetase	EC6.2.1.3	YIL009W		10c00058, 16c00012	YC*
Fatty acyl-coenzyme A oxidase	EC1.3.3.6	YGL205W		3d00016	YC*
Peroxisomal multifunctional b-oxidation protein	EC4.2.1.74	YKR009C		9d00080	YC*
3-Ketoacyl thiolase	EC2.3.1.16	YIL160C		5c00047, 7d00227, 22d00199, 14c00041	YC*
acyl-CoA thioesterase	EC3.1.2.2	YJR019C		9c00187	YC*
**mitochondrial beta-oxidation**					
Acyl-CoA dehydrogenase	EC1.3.99.-		AO090005000482	9c00077	Microbiology [37]
Acyl-CoA dehydrogenase	EC1.3.99.-		AO090023000711	22c00013, 10c00046, 26d00031	Microbiology [37]
3-Hydroxyacyl-CoA dehydrogenase (LCHAD)	EC1.1.1.35		AO090206000053	6c00105, 7c00216, 15c00001	Microbiology [37]
Enoyl-CoA dehydratase	EC4.2.1.17		AO090011000537	3d00051, 22d00252, 22d00236, 24d00001, 6c00098, 14d00106, 1c00016	Microbiology [37]
Acetyl-CoA acetyltransferase	EC2.3.1.9		AO090103000406	14c00041, 22d00199, 7d00227, 5c00047, 8d00008	Microbiology [37]
3-Hydroxyacyl-CoA dehydrogenase (zHAD)	EC1.1.1.35		RO3G_10340	18c00036, 27c00018, 12c00049, 20c00015, 22d00143, 9d00328, 7d00314, 9c00142, 2c00059, 24c00050	Microbiology [37]

*SGD database (http://www.yeastgenome.org/)
