# Supplementary material for: Genome and Transcriptome Analysis of the Basidiomycetous Yeast Pseudozyma antarctica Producing Extracellular Glycolipids, Mannosylerythritol Lipids
Source: PLoS One. 2014 Feb 24;9(2):e86490. doi: 10.1371/journal.pone.0086490 (PMC3933340; doi:10.1371/journal.pone.0086490)
Supplement: Tables S3 — Analysis of the orthologous genes between P. antarctica and U. maydis . (PDF) [file pone.0086490.s004.pdf]

Supplementary Table S3

Analysis of the orthologous genes between *P. antarctica* and *U. maydis*

	Pa to Um			Um to Pa		
	P. antarctica gene	*U. maydis* gene	e-value	*P. antarctica* gene	*U. maydis* gene	e-value
NNN[1]	1d00043	um01656	2.00E-44	um01656	22c00121	0
NNN	1c00005	um06368	8.00E-30	um06368	1c00004	1.00E-135
BBH[2]	1c00018	um06383	0	um06383	1c00018	0
BBH	1c00013	um10993	3.00E-44	um10993	1c00013	3.00E-36
BBH	1d00007	um06359	8.00E-63	um06359	1d00007	1.00E-68
BBH	1d00039	um06450	1.00E-179	um06450	1d00039	1.00E-179
BBH	1d00020	um06404	1.00E-125	um06404	1d00020	1.00E-125
BBH	1d00016	um06397	1.00E-33	um06397	1d00016	6.00E-28
NNN	1c00033	um06418	1.00E-101	um06418	1c00034	0
BBH	1c00025	um06398	7.00E-97	um06398	1c00025	1.00E-101
BBH	1d00029	um06421	0	um06421	1d00029	0
BBH	1d00006	um12336.2	0	um12336.2	1d00006	0
BBH	1c00010	um06363	0	um06363	1c00010	0
BBH	1c00016	um11001	1.00E-94	um11001	1c00016	7.00E-99
BBH	1d00036	um12046	7.00E-19	um12046	1d00036	1.00E-19
BBH	1c00019	um06385	1.00E-104	um06385	1c00019	1.00E-105
NNN	1d00031	um04032	7.00E-25	um04032	13c00090	0
BBH	1d00017	um06400	0	um06400	1d00017	0
BBH	1d00048	um06454	1.00E-122	um06454	1d00048	1.00E-111
BBH	1d00050	um06456	0	um06456	1d00050	0
BBH	1d00021	um06406	4.00E-79	um06406	1d00021	5.00E-81
BBH	1c00007	um06371	0	um06371	1c00007	0
BBH	1d00013	um06391	0	um06391	1d00013	0
BBH	1c00041	um06440	1.00E-136	um06440	1c00041	1.00E-166
BBH	1d00027	um06417	1.00E-136	um06417	1d00027	1.00E-136
NNN	1c00011	um06360	1.00E-62			
BBH	1d00037	um06445	1.00E-102	um06445	1d00037	1.00E-102
BBH	1c00017	um06381	5.00E-75	um06381	1c00017	2.00E-68
BBH	1d00041	um15103	3.00E-32	um15103	1d00041	3.00E-62
BBH	1d00005	um06362	0	um06362	1d00005	0
BBH	1c00031	um06412	0	um06412	1c00031	0
BBH	1c00047	um12050	7.00E-58	um12050	1c00047	8.00E-63
BBH	1c00038	um06427	1.00E-148	um06427	1c00038	1.00E-139
BBH	1c00044	um12047	4.00E-35	um12047	1c00044	2.00E-41
NNN	1c00001	um01656	7.00E-43	um01656	22c00121	0
NNN	1d00051	um02896	3.00E-30	um02896	19d00125	0
BBH	1c00045	um12048	1.00E-155	um12048	1c00045	1.00E-148
NNN	1d00004	um12337	0			
BBH	1d00026	um06414	0	um06414	1d00026	0
BBH	1c00014	um06377	1.00E-123	um06377	1c00014	1.00E-118
BBH	1d00049	um06455	6.00E-98	um06455	1d00049	2.00E-97
BBH	1c00023	um06394	1.00E-86	um06394	1c00023	2.00E-85
BBH	1d00024	um06411	0	um06411	1d00024	0
BBH	1c00040	um06434	0	um06434	1c00040	0
BBH	1c00046	um06448	3.00E-30	um06448	1c00046	1.00E-30
BBH	1d00014	um06395	1.00E-107	um06395	1d00014	1.00E-111
BBH	1c00039	um06430	0	um06430	1c00039	0
BBH	1c00004	um06368	1.00E-136	um06368	1c00004	1.00E-135
BBH	1c00032	um11239	0	um11239	1c00032	0
NNN	1d00034	um05038	0	um05038	3c00070	0
BBH	1c00050	um06453	1.00E-123	um06453	1c00050	1.00E-123
BBH	1c00022	um11005	1.00E-146	um11005	1c00022	1.00E-157
BBH	1d00035	um06443	1.00E-119	um06443	1d00035	1.00E-119
BBH	1c00042	um12044	2.00E-20	um12044	1c00042	1.00E-20
BBH	1c00029	um06407	0	um06407	1c00029	0

BBH	1d00025	um06413	5.00E-76	um06413	1d00025	2.00E-70
BBH	1c00021	um06388	2.00E-44	um06388	1c00021	2.00E-44
BBH	1c00052	um04091	0	um04091	1c00052	0
BBH	1d00002	um10996	1.00E-180	um10996	1d00002	0
BBH	1c00020	um06387	1.00E-56	um06387	1c00020	2.00E-57
BBH	1d00011	um06386	1.00E-143	um06386	1d00011	1.00E-146
NNN	1c00049	um05861	1.00E-62	um05861	7d00035	0
BBH	1c00008	um06372	0	um06372	1c00008	0
BBH	1c00006	um06370	1.00E-106	um06370	1c00006	1.00E-103
NNN	1d00032	um06428	0			
BBH	1c00030	um06410	0	um06410	1c00030	0
BBH	1c00043	um12045	0	um12045	1c00043	0
BBH	1d00012	um06389	1.00E-80	um06389	1d00012	1.00E-100
NNN	1c00048	um04967	1.00E-152	um04967	3d00047	1.00E-177
BBH	1c00028	um06405	8.00E-75	um06405	1c00028	6.00E-81
BBH	1c00037	um06426	1.00E-147	um06426	1c00037	1.00E-147
BBH	1c00015	um06378	3.00E-48	um06378	1c00015	8.00E-62
BBH	1c00009	um06365.2	1.00E-139	um06365.2	1c00009	1.00E-125
BBH	1c00027	um11007	1.00E-135	um11007	1c00027	1.00E-133
NNN	1d00044	um04806	1.00E-20	um04806	26c00044	0
BBH	1d00033	um06429.2	1.00E-137	um06429.2	1d00033	1.00E-137
BBH	1d00018	um06401	0	um06401	1d00018	0
BBH	1d00022	um06408	1.00E-136	um06408	1d00022	1.00E-136
BBH	1d00009	um11000	3.00E-52	um11000	1d00009	1.00E-52
BBH	1d00038	um12049	9.00E-26	um12049	1d00038	2.00E-26
BBH	1c00003	um06374	1.00E-169	um06374	1c00003	1.00E-174
BBH	1c00034	um06418	0	um06418	1c00034	0
NNN	1d00042	um01656	7.00E-44	um01656	22c00121	0
NNN	1c00036	um04046	1.00E-31	um04046	13c00097	0
NNN	1d00008	um05580	2.00E-24	um05580	9d00320	3.00E-37
BBH	1d00019	um06402	0	um06402	1d00019	0
BBH	1c00026	um06399	1.00E-111	um06399	1c00026	1.00E-111
BBH	1d00030	um06425	1.00E-170	um06425	1d00030	1.00E-138
BBH	1d00028	um06420	0	um06420	1d00028	0
BBH	1c00012	um06358	1.00E-174	um06358	1c00012	0
BBH	1c00035	um06422	1.00E-159	um06422	1c00035	1.00E-158
NNN	1d00045	um11256	1.00E-35			
BBH	1d00023	um11238	1.00E-53	um11238	1d00023	1.00E-53
BBH	1d00003	um06367	1.00E-102	um06367	1d00003	0
BBH	2d00054	um05199	0	um05199	2d00054	0
BBH	2c00084	um05239	1.00E-177	um05239	2c00084	1.00E-177
BBH	2d00035	um05156	0	um05156	2d00035	0
BBH	2d00011	um05108	0	um05108	2d00011	0
BBH	2c00049	um05179	0	um05179	2c00049	0
BBH	2c00079	um05233	0	um05233	2c00079	0
BBH	2c00030	um05143	1.00E-163	um05143	2c00030	1.00E-150
BBH	2c00044	um05170	0	um05170	2c00044	0
BBH	2d00073	um11787	1.00E-130	um11787	2d00073	1.00E-130
BBH	2d00009	um05105	1.00E-156	um05105	2d00009	1.00E-156
BBH	2d00047	um05186	0	um05186	2d00047	0
BBH	2c00072	um05222	2.00E-81	um05222	2c00072	3.00E-83
BBH	2c00052	um05185	1.00E-174	um05185	2c00052	1.00E-172
BBH	2d00051	um05191.2	1.00E-112	um05191.2	2d00051	1.00E-124
BBH	2c00018	um05117	0	um05117	2c00018	0
NNN	2d00004	um05088	1.00E-53			
BBH	2c00028	um05137	1.00E-117	um05137	2c00028	1.00E-120
BBH	2c00064	um05209	0	um05209	2c00064	0
BBH	2d00060	um05208	0	um05208	2d00060	0
NNN	2c00011	um05104	2.00E-16			
BBH	2c00073	um05223	0	um05223	2c00073	0
BBH	2c00083	um05237	6.00E-53	um05237	2c00083	1.00E-47
BBH	2d00048	um10195	0	um10195	2d00048	0

BBH	2d00053	um05197	1.00E-139	um05197	2d00053	1.00E-139
BBH	2d00074	um05238	4.00E-63	um05238	2d00074	1.00E-76
BBH	2c00038	um05160	0	um05160	2c00038	0
BBH	2c00053	um10194	7.00E-33	um10194	2c00053	1.00E-32
BBH	2c00010	um05101	0	um05101	2c00010	0
BBH	2c00058	um05198	0	um05198	2c00058	0
BBH	2c00023	um05126	0	um05126	2c00023	0
BBH	2d00024	um05136	0	um05136	2d00024	0
NNN	2c00017	um05115	0			
BBH	2d00068	um05225	8.00E-25	um05225	2d00068	2.00E-29
BBH	2d00034	um05153	0	um05153	2d00034	0
BBH	2c00004	um05097	1.00E-127	um05097	2c00004	1.00E-131
BBH	2d00075	um05240	0	um05240	2d00075	0
BBH	2d00020	um05127	1.00E-167	um05127	2d00020	1.00E-163
BBH	2d00002	um11779	0	um11779	2d00002	0
BBH	2c00057	um11567.2	2.00E-42	um11567.2	2c00057	2.00E-42
BBH	2c00027	um10179	5.00E-53	um10179	2c00027	4.00E-53
BBH	2c00045	um05171	0	um05171	2c00045	0
BBH	2d00045	um05182	2.00E-90	um05182	2d00045	2.00E-93
BBH	2c00020	um05122	0	um05122	2c00020	0
BBH	2c00054	um10196	2.00E-54	um10196	2c00054	3.00E-55
BBH	2d00016	um10174	0	um10174	2d00016	0
BBH	2c00024	um05128	2.00E-40	um05128	2c00024	4.00E-41
BBH	2c00037	um05158	0	um05158	2c00037	0
BBH	2d00019	um05125	0	um05125	2d00019	0
BBH	2c00026	um05132	0	um05132	2c00026	1.00E-168
BBH	2d00033	um05151	1.00E-113	um05151	2d00033	1.00E-142
BBH	2c00056	um05194	0	um05194	2c00056	0
BBH	2c00016	um05114	0	um05114	2c00016	0
BBH	2c00003	um10170	0	um10170	2c00003	0
BBH	2d00061	um05210	3.00E-95	um05210	2d00061	1.00E-94
BBH	2c00031	um10183	1.00E-141	um10183	2c00031	1.00E-148
BBH	2d00046	um05184	1.00E-102	um05184	2d00046	1.00E-105
BBH	2c00046	um05173	0	um05173	2c00046	0
BBH	2d00010	um10173	5.00E-67	um10173	2d00010	3.00E-68
BBH	2c00043	um05166	0	um05166	2c00043	0
BBH	2c00055	um10198	0	um10198	2c00055	0
BBH	2d00008	um05103	0	um05103	2d00008	0
BBH	2c00060	um05202	1.00E-114	um05202	2c00060	1.00E-114
BBH	2c00075	um05226	6.00E-46	um05226	2c00075	7.00E-55
BBH	2c00002	um05090	1.00E-116	um05090	2c00002	1.00E-118
BBH	2d00069	um05227	1.00E-91	um05227	2d00069	1.00E-91
BBH	2c00025	um10178	4.00E-19	um10178	2c00025	4.00E-24
BBH	2c00067	um05213	0	um05213	2c00067	0
BBH	2c00074	um15076	3.00E-15	um15076	2c00074	1.00E-18
BBH	2d00007	um05102	5.00E-27	um05102	2d00007	5.00E-27
BBH	2d00056	um05201	1.00E-146	um05201	2d00056	1.00E-147
BBH	2c00032	um05148	0	um05148	2c00032	0
NNN	2c00015	um05112	0			
BBH	2d00058	um11568	0	um11568	2d00058	0
BBH	2d00065	um05219	0	um05219	2d00065	0
BBH	2d00044	um05178	1.00E-67	um05178	2d00044	9.00E-54
BBH	2c00008	um10172	0	um10172	2c00008	0
BBH	2d00039	um10189	0	um10189	2d00039	0
BBH	2d00018	um10175	0	um10175	2d00018	0
BBH	2d00027	um10180	1.00E-44	um10180	2d00027	3.00E-41
BBH	2d00064	um05215	2.00E-71	um05215	2d00064	4.00E-74
BBH	2c00061	um11569	1.00E-167	um11569	2c00061	1.00E-170
BBH	2d00014	um05116.2	1.00E-121	um05116.2	2d00014	1.00E-124
BBH	2c00085	um05241	0	um05241	2c00085	0
BBH	2d00063	um05214	0	um05214	2d00063	0
BBH	2d00038	um05163	1.00E-86	um05163	2d00038	1.00E-85

BBH	2c00001	um05085	1.00E-105	um05085	2c00001	1.00E-99
NNN	2d00049	um10197	1.00E-71			
BBH	2c00039	um05162	0	um05162	2c00039	0
BBH	2c00014	um05111	1.00E-109	um05111	2c00014	1.00E-114
BBH	2c00034	um05152	0	um05152	2c00034	0
BBH	2d00021	um05130	0	um05130	2d00021	0
BBH	2c00081	um11785	0	um11785	2c00081	0
BBH	2d00043	um10191	1.00E-147	um10191	2d00043	1.00E-152
BBH	2c00047	um05174	1.00E-158	um05174	2c00047	1.00E-173
BBH	2d00032	um05149	1.00E-116	um05149	2d00032	1.00E-122
BBH	2d00028	um05142	0	um05142	2d00028	0
BBH	2c00068	um05216	1.00E-161	um05216	2c00068	1.00E-175
NNN	2c00007	um01656	3.00E-29	um01656	22c00121	0
BBH	2c00048	um05177	0	um05177	2c00048	0
BBH	2d00037	um05161	1.00E-78	um05161	2d00037	2.00E-89
BBH	2d00062	um11570	1.00E-99	um11570	2d00062	2.00E-97
BBH	2c00076	um05229	0	um05229	2c00076	0
BBH	2d00015	um05118	1.00E-154	um05118	2d00015	0
BBH	2d00005	um05091.2	1.00E-105	um05091.2	2d00005	1.00E-115
BBH	2c00080	um11783	3.00E-59	um11783	2c00080	2.00E-63
BBH	2c00033	um10184	0	um10184	2c00033	0
BBH	2d00070	um05228	2.00E-34	um05228	2d00070	9.00E-31
NNN	2d00040	um05167	5.00E-37			
NNN	2c00006	um01656	6.00E-53	um01656	22c00121	0
BBH	2d00025	um05138	1.00E-74	um05138	2d00025	2.00E-73
BBH	2d00031	um05145	1.00E-107	um05145	2d00031	1.00E-115
BBH	2c00013	um05109	1.00E-127	um05109	2c00013	1.00E-127
BBH	2c00040	um10188	0	um10188	2c00040	0
BBH	2c00077	um05231	3.00E-79	um05231	2c00077	5.00E-78
BBH	2d00071	um05230	0	um05230	2d00071	0
BBH	2c00036	um10187	3.00E-46	um10187	2c00036	3.00E-35
BBH	2d00023	um05134	0	um05134	2d00023	0
BBH	2c00069	um05217	1.00E-108	um05217	2c00069	1.00E-117
BBH	2d00041	um12158	1.00E-28	um12158	2d00041	1.00E-26
BBH	2c00051	um05183	5.00E-68	um05183	2c00051	1.00E-66
BBH	2c00041	um05169	0	um05169	2c00041	0
BBH	2d00012	um05110	0	um05110	2d00012	0
BBH	2c00070	um05218	0	um05218	2c00070	0
BBH	2d00026	um05139	1.00E-123	um05139	2d00026	1.00E-123
BBH	2d00067	um05224	0	um05224	2d00067	0
BBH	2d00066	um11572	1.00E-77	um11572	2d00066	7.00E-93
BBH	2c00029	um05141	0	um05141	2c00029	0
BBH	2c00005	um12157	3.00E-48	um12157	2c00005	4.00E-55
BBH	2c00009	um05100	2.00E-53	um05100	2c00009	8.00E-52
NNN	2d00052	um15018	1.00E-57			
BBH	2c00063	um05207	3.00E-39	um05207	2c00063	8.00E-37
NNN	2c00019	um10174	6.00E-22	um10174	2d00016	0
BBH	2c00022	um10177	0	um10177	2c00022	0
BBH	2d00042	um12159	0	um12159	2d00042	0
BBH	2d00059	um05206	0	um05206	2d00059	0
BBH	2c00050	um05180	1.00E-139	um05180	2c00050	1.00E-137
BBH	2c00042	um10190.2	0	um10190.2	2c00042	0
BBH	2c00059	um05200	0	um05200	2c00059	0
BBH	2d00022	um05131	0	um05131	2d00022	0
BBH	2c00035	um10186	9.00E-57	um10186	2c00035	4.00E-59
BBH	2c00071	um11571	9.00E-69	um11571	2c00071	9.00E-68
BBH	2d00030	um10181	0	um10181	2d00030	0
BBH	2d00036	um05159	3.00E-58	um05159	2d00036	3.00E-56
BBH	2c00012	um05107	0	um05107	2c00012	0
BBH	2c00082	um11786	9.00E-97	um11786	2c00082	3.00E-91
BBH	2c00078	um05232	0	um05232	2c00078	0
BBH	2d00003	um05087	2.00E-88	um05087	2d00003	2.00E-94

BBH	2d00013	um05113.2	0	um05113.2	2d00013	0
BBH	3d00089	um10715	1.00E-145	um10715	3d00089	1.00E-149
BBH	3c00041	um11558	1.00E-113	um11558	3c00041	1.00E-140
BBH	3c00065	um12295	2.00E-75	um12295	3c00065	4.00E-71
BBH	3c00076	um05054	1.00E-120	um05054	3c00076	1.00E-117
BBH	3d00041	um11553	0	um11553	3d00041	0
BBH	3d00024	um11163.2	0	um11163.2	3d00024	0
BBH	3c00021	um12291	2.00E-87	um12291	3c00021	2.00E-76
BBH	3c00049	um04994	0	um04994	3c00049	0
BBH	3d00046	um04966	0	um04966	3d00046	0
BBH	3d00019	um04923	0	um04923	3d00019	0
BBH	3c00003	um04914	1.00E-157	um04914	3c00003	1.00E-163
BBH	3d00015	um04898	1.00E-158	um04898	3d00015	1.00E-167
BBH	3c00062	um05034	0	um05034	3c00062	0
BBH	3d00009	um11157	1.00E-90	um11157	3d00009	3.00E-91
BBH	3d00034	um04943	1.00E-47	um04943	3d00034	2.00E-41
BBH	3c00001	um04916	1.00E-142	um04916	3c00001	1.00E-137
BBH	3d00078	um05031	1.00E-105	um05031	3d00078	1.00E-105
BBH	3d00095	um05059	0	um05059	3d00095	0
BBH	3d00002	um04922	1.00E-119	um04922	3d00002	1.00E-119
BBH	3d00051	um11556	1.00E-137	um11556	3d00051	1.00E-146
BBH	3c00059	um05014	0	um05014	3c00059	0
BBH	3c00071	um10711	1.00E-120	um10711	3c00071	1.00E-120
BBH	3d00064	um12294	1.00E-101	um12294	3d00064	5.00E-97
BBH	3c00048	um04992.2	1.00E-50	um04992.2	3c00048	4.00E-47
BBH	3d00070	um05017.2	1.00E-171	um05017.2	3d00070	1.00E-166
BBH	3d00088	um10714	3.00E-55	um10714	3d00088	5.00E-62
BBH	3c00004	um04912	5.00E-48	um04912	3c00004	6.00E-44
BBH	3c00040	um04977	1.00E-170	um04977	3c00040	1.00E-160
BBH	3d00014	um04899	0	um04899	3d00014	0
BBH	3c00066	um10709	0	um10709	3c00066	0
BBH	3c00022	um04939	1.00E-177	um04939	3c00022	1.00E-177
BBH	3c00033	um04962	1.00E-100	um04962	3c00033	1.00E-101
BBH	3c00014	um04928	0	um04928	3c00014	0
BBH	3d00003	um11161	1.00E-117	um11161	3d00003	1.00E-117
BBH	3d00094	um05058	3.00E-61	um05058	3d00094	3.00E-61
BBH	3c00070	um05038	0	um05038	3c00070	0
BBH	3d00050	um04971	0	um04971	3d00050	0
BBH	3d00007	um04909	1.00E-153	um04909	3d00007	1.00E-164
BBH	3c00058	um05013	0	um05013	3c00058	0
BBH	3d00045	um04965	9.00E-42	um04965	3d00045	8.00E-36
BBH	3d00057	um12013	0	um12013	3d00057	0
BBH	3c00034	um04964	0	um04964	3c00034	0
BBH	3d00079	um05028	0	um05028	3d00079	0
BBH	3c00051	um04999	1.00E-159	um04999	3c00051	1.00E-147
BBH	3c00038	um04972	1.00E-119	um04972	3c00038	1.00E-119
BBH	3d00036	um04947	0	um04947	3d00036	0
BBH	3c00009	um04902	1.00E-153	um04902	3c00009	1.00E-152
BBH	3d00063	um04998	1.00E-170	um04998	3d00063	1.00E-117
BBH	3d00077	um05025	0	um05025	3d00077	0
BBH	3d00043	um04960	8.00E-66	um04960	3d00043	1.00E-63
BBH	3c00043	um12012	3.00E-72	um12012	3c00043	1.00E-73
NNN	3c00010	um04902	1.00E-34	um04902	3c00009	1.00E-152
BBH	3c00023	um04942	1.00E-103	um04942	3c00023	1.00E-117
BBH	3c00050	um11559	0	um11559	3c00050	0
BBH	3c00042	um04979	0	um04979	3c00042	0
BBH	3d00097	um05062	3.00E-70	um05062	3d00097	1.00E-70
BBH	3d00042	um04956	0	um04956	3d00042	0
BBH	3c00067	um05026	8.00E-19	um05026	3c00067	1.00E-19
BBH	3d00056	um04985	4.00E-95	um04985	3d00056	3.00E-99
BBH	3c00053	um12293	1.00E-73	um12293	3c00053	3.00E-73
BBH	3d00029	um04934	1.00E-172	um04934	3d00029	1.00E-168

BBH	3c00029	um04954.2	5.00E-13	um04954.2	3c00029	5.00E-11
BBH	3d00031	um11769	1.00E-124	um11769	3d00031	1.00E-123
BBH	3d00096	um05060	5.00E-35	um05060	3d00096	5.00E-34
BBH	3c00024	um04944	0	um04944	3c00024	0
BBH	3d00013	um04900	0	um04900	3d00013	0
BBH	3d00062	um04997	0	um04997	3d00062	0
BBH	3d00047	um04967	1.00E-177	um04967	3d00047	1.00E-177
BBH	3d00055	um04980	6.00E-60	um04980	3d00055	2.00E-96
BBH	3d00025	um04931	0	um04931	3d00025	0
BBH	3d00028	um12011	1.00E-21	um12011	3d00028	4.00E-25
BBH	3c00012	um04897	1.00E-179	um04897	3c00012	1.00E-179
BBH	3c00037	um04970	0	um04970	3c00037	0
BBH	3d00008	um04906	0	um04906	3d00008	0
BBH	3c00002	um04915	1.00E-132	um04915	3c00002	1.00E-132
BBH	3d00083	um05039	0	um05039	3d00083	0
NNN	3d00023	um04930	0			
BBH	3c00031	um04959	0	um04959	3c00031	0
BBH	3c00075	um05052	0	um05052	3c00075	0
BBH	3d00030	um11768	1.00E-19	um11768	3d00030	3.00E-26
BBH	3d00091	um05055	6.00E-39	um05055	3d00091	2.00E-36
BBH	3d00061	um04995	5.00E-44	um04995	3d00061	1.00E-45
NNN	3d00049	um01996	8.00E-78			
NNN	3c00080	um02742	1.00E-101	um02742	5c00029	0
BBH	3c00068	um05030	1.00E-159	um05030	3c00068	1.00E-159
BBH	3c00026	um04950	0	um04950	3c00026	0
BBH	3d00073	um05033	0	um05033	3d00073	0
BBH	3c00074	um05050	0	um05050	3c00074	0
BBH	3c00011	um04901	0	um04901	3c00011	0
BBH	3d00022	um04927	2.00E-57	um04927	3d00022	9.00E-55
BBH	3c00055	um05005	2.00E-45	um05005	3c00055	2.00E-45
BBH	3c00061	um05018	1.00E-159	um05018	3c00061	1.00E-153
BBH	3d00085	um05045	0	um05045	3d00085	0
BBH	3c00039	um04975	0	um04975	3c00039	0
BBH	3d00076	um05023	0	um05023	3d00076	0
BBH	3d00087	um10713.2	2.00E-90	um10713.2	3d00087	1.00E-89
BBH	3c00045	um04983	0	um04983	3c00045	0
BBH	3c00018	um15100	1.00E-74	um15100	3c00018	9.00E-64
BBH	3d00037	um04948	2.00E-43	um04948	3d00037	2.00E-43
BBH	3d00033	um11770	4.00E-95	um11770	3d00033	4.00E-95
BBH	3c00079	um12068	2.00E-98	um12068	3c00079	8.00E-99
BBH	3d00090	um05053	2.00E-81	um05053	3d00090	2.00E-81
BBH	3d00054	um11557	1.00E-85	um11557	3d00054	2.00E-84
BBH	3d00075	um05022	0	um05022	3d00075	0
BBH	3c00030	um11554	0	um11554	3c00030	0
BBH	3d00068	um11563	2.00E-75	um11563	3d00068	2.00E-80
NNN	3d00102	um02062	2.00E-40	um02062	6c00122	0
BBH	3d00060	um04993	1.00E-77	um04993	3d00060	6.00E-87
BBH	3d00048	um04969	1.00E-111	um04969	3d00048	1.00E-120
BBH	3d00067	um11562	5.00E-22	um11562	3d00067	3.00E-21
BBH	3c00073	um12297	3.00E-70	um12297	3c00073	1.00E-65
BBH	3c00007	um04905	0	um04905	3c00007	0
BBH	3d00010	um12289	1.00E-114	um12289	3d00010	1.00E-114
NNN	3d00059	um04991	0			
BBH	3c00044	um04982	1.00E-174	um04982	3c00044	0
BBH	3c00069	um05036	1.00E-121	um05036	3c00069	1.00E-126
BBH	3d00084	um10712	1.00E-117	um10712	3d00084	1.00E-114
BBH	3c00017	um04932	8.00E-78	um04932	3c00017	3.00E-80
BBH	3c00032	um04961	0	um04961	3c00032	0
BBH	3d00004	um04913	1.00E-118	um04913	3d00004	1.00E-120
BBH	3d00039	um04951	0	um04951	3d00039	0
NNN	3c00028	um11552	3.00E-87			
BBH	3c00054	um05004	1.00E-134	um05004	3c00054	1.00E-107

BBH	3c00057	um11564	6.00E-39	um11564	3c00057	8.00E-21
BBH	3d00044	um04963	3.00E-73	um04963	3d00044	5.00E-73
BBH	3d00093	um05057	0	um05057	3d00093	0
BBH	3d00071	um05019	0	um05019	3d00071	0
BBH	3d00053	um04976	0	um04976	3d00053	0
NNN	3d00081	um05040	7.00E-37	um05040	3d00082	1.00E-57
BBH	3d00021	um04926	0	um04926	3d00021	0
BBH	3c00078	um05061	0	um05061	3c00078	0
BBH	3d00069	um05015	3.00E-28	um05015	3d00069	6.00E-20
BBH	3c00047	um04989	2.00E-89	um04989	3c00047	1.00E-100
NNN	3d00032	um11769	1.00E-100	um11769	3d00031	1.00E-123
BBH	3c00005	um04908	2.00E-56	um04908	3c00005	4.00E-65
NNN	3c00008	um02490	1.00E-112	um02490	12c00135	0
BBH	3d00082	um05040	9.00E-58	um05040	3d00082	1.00E-57
NNN	3c00060	um10707	2.00E-18			
BBH	3d00099	um05064	0	um05064	3d00099	0
BBH	3d00066	um11561	1.00E-51	um11561	3d00066	9.00E-52
BBH	3d00017	um04896	0	um04896	3d00017	0
BBH	3d00026	um12009	2.00E-49	um12009	3d00026	8.00E-47
BBH	3d00100	um05065	0	um05065	3d00100	0
BBH	3d00005	um04910	0	um04910	3d00005	0
BBH	3d00038	um12292	2.00E-36	um12292	3d00038	2.00E-41
BBH	3c00064	um05035	0	um05035	3c00064	0
BBH	3d00001	um04920	0	um04920	3d00001	0
BBH	3c00056	um05007	0	um05007	3c00056	0
BBH	3d00092	um12298	0	um12298	3d00092	0
BBH	3c00072	um05044	1.00E-89	um05044	3c00072	8.00E-81
BBH	3d00052	um04974	8.00E-27	um04974	3d00052	4.00E-27
BBH	3d00020	um04925	1.00E-138	um04925	3d00020	1.00E-136
BBH	3c00016	um12008	0	um12008	3c00016	0
BBH	3c00063	um05037	1.00E-141	um05037	3c00063	1.00E-141
BBH	3d00080	um05042.2	1.00E-114	um05042.2	3d00080	1.00E-130
BBH	3c00006	um04907	0	um04907	3c00006	0
BBH	3d00074	um10708	1.00E-127	um10708	3d00074	1.00E-122
BBH	3c00025	um11771	3.00E-67	um11771	3c00025	2.00E-66
BBH	3c00046	um04988	1.00E-146	um04988	3c00046	1.00E-146
BBH	3c00020	um04936	1.00E-101	um04936	3c00020	2.00E-99
BBH	3d00016	um04324	0	um04324	3d00016	0
BBH	3d00072	um05032	0	um05032	3d00072	0
BBH	3c00019	um11767	1.00E-145	um11767	3c00019	1.00E-152
BBH	3d00098	um05063	0	um05063	3d00098	0
BBH	3d00065	um11560	0	um11560	3d00065	0
NNN	3d00086	um05046	6.00E-37			
BBH	4d00042	um06344	0	um06344	4d00042	0
BBH	4d00005	um06295	0	um06295	4d00005	0
BBH	4d00027	um06317	0	um06317	4d00027	0
BBH	4c00009	um10763	1.00E-158	um10763	4c00009	1.00E-162
BBH	4c00008	um10765	1.00E-59	um10765	4c00008	4.00E-61
NNN	4c00006	um06292	4.00E-48	um06292	4c00007	8.00E-91
BBH	4d00035	um10785	1.00E-120	um10785	4d00035	1.00E-126
BBH	4c00041	um06346	8.00E-60	um06346	4c00041	9.00E-64
BBH	4d00015	um12332	1.00E-43	um12332	4d00015	7.00E-44
BBH	4c00024	um12334	1.00E-22	um12334	4c00024	3.00E-18
BBH	4d00032	um06326	1.00E-111	um06326	4d00032	1.00E-124
BBH	4d00006	um06294	0	um06294	4d00006	0
BBH	4d00024	um10773	3.00E-81	um10773	4d00024	5.00E-92
BBH	4d00045	um05614	3.00E-78	um05614	4d00045	3.00E-78
BBH	4c00025	um10776	1.00E-172	um10776	4c00025	1.00E-175
BBH	4d00014	um06281	0	um06281	4d00014	0
BBH	4c00019	um10769	0	um10769	4c00019	0
BBH	4c00040	um15092	1.00E-167	um15092	4c00040	1.00E-168
BBH	4d00033	um10782	0	um10782	4d00033	0

BBH	4d00012	um15029	1.00E-66	um15029	4d00012	3.00E-69
BBH	4c00031	um12335	8.00E-79	um12335	4c00031	1.00E-86
BBH	4c00022	um10775	0	um10775	4c00022	0
BBH	4c00038	um06338	0	um06338	4c00038	0
BBH	4c00030	um10781	1.00E-120	um10781	4c00030	1.00E-128
BBH	4d00019	um10761	0	um10761	4d00019	0
BBH	4d00010	um06273	0	um06273	4d00010	0
BBH	4c00028	um10780	5.00E-63	um10780	4c00028	5.00E-61
BBH	4d00025	um06312	8.00E-50	um06312	4d00025	3.00E-46
NNN	4d00003	um10772	3.00E-23			
BBH	4d00044	um06347	0	um06347	4d00044	0
BBH	4d00030	um10779	0	um10779	4d00030	0
BBH	4d00011	um06274	0	um06274	4d00011	0
BBH	4c00032	um06329	1.00E-118	um06329	4c00032	1.00E-114
BBH	4d00013	um06280	0	um06280	4d00013	0
BBH	4d00022	um06305	2.00E-70	um06305	4d00022	3.00E-84
BBH	4c00039	um12042	0	um12042	4c00039	0
BBH	4c00023	um06316	0	um06316	4c00023	0
BBH	4c00010	um06288	0	um06288	4c00010	0
BBH	4d00004	um10770	1.00E-139	um10770	4d00004	1.00E-147
BBH	4c00029	um06323	2.00E-50	um06323	4c00029	3.00E-50
BBH	4c00012	um15067	8.00E-29	um15067	4c00012	1.00E-25
BBH	4d00038	um06333	0	um06333	4d00038	0
BBH	4c00037	um06337	0	um06337	4c00037	0
BBH	4d00031	um06324	2.00E-90	um06324	4d00031	2.00E-90
BBH	4c00033	um10784	1.00E-172	um10784	4c00033	1.00E-172
BBH	4d00020	um06268	0	um06268	4d00020	0
BBH	4c00016	um06270	1.00E-177	um06270	4c00016	1.00E-161
BBH	4c00020	um06304.2	1.00E-144	um06304.2	4c00020	1.00E-147
BBH	4d00017	um06269	0	um06269	4d00017	0
BBH	4c00002	um06296	0	um06296	4c00002	0
BBH	4d00023	um06306	0	um06306	4d00023	0
BBH	4c00011	um06276	2.00E-36	um06276	4c00011	3.00E-35
NNN	4c00043	um12236	2.00E-13	um12236	8d00015	1.00E-138
BBH	4d00039	um06335	0	um06335	4d00039	0
BBH	4d00021	um10768	0	um10768	4d00021	1.00E-178
BBH	4c00015	um10760	9.00E-92	um10760	4c00015	8.00E-92
BBH	4d00018	um06271	1.00E-73	um06271	4d00018	2.00E-64
NNN	4d00036	um03801	9.00E-44	um03801	27c00064	2.00E-54
BBH	4d00002	um06299	0	um06299	4d00002	0
BBH	4d00041	um06341	0	um06341	4d00041	0
BBH	4d00029	um10777	0	um10777	4d00029	0
BBH	4c00035	um06334	0	um06334	4c00035	0
BBH	4c00042	um06348	0	um06348	4c00042	0
BBH	4c00014	um06284	0	um06284	4c00014	0
BBH	4c00018	um12039	3.00E-77	um12039	4c00018	2.00E-80
BBH	4c00003	um06298	1.00E-124	um06298	4c00003	1.00E-117
BBH	4d00007	um06290	1.00E-61	um06290	4d00007	2.00E-62
BBH	4d00008	um10764	4.00E-33	um10764	4d00008	6.00E-39
BBH	4c00026	um10778	0	um10778	4c00026	0
BBH	4d00001	um10767	0	um10767	4d00001	0
BBH	4d00026	um06314	0	um06314	4d00026	0
BBH	4c00036	um06336	1.00E-152	um06336	4c00036	1.00E-154
BBH	4c00013	um06278	3.00E-70	um06278	4c00013	1.00E-136
BBH	4c00004	um10771	4.00E-71	um10771	4c00004	4.00E-74
BBH	4d00037	um06332	4.00E-96	um06332	4d00037	1.00E-102
BBH	4d00016	um06285	0	um06285	4d00016	0
BBH	4c00007	um06292	3.00E-90	um06292	4c00007	8.00E-91
BBH	4d00009	um06287	0	um06287	4d00009	0
BBH	4d00043	um06345	0	um06345	4d00043	0
BBH	4d00034	um10783	4.00E-79	um10783	4d00034	2.00E-80
BBH	4d00040	um12041	0	um12041	4d00040	0

BBH	5d00070	um02667	0	um02667	5d00070	0
BBH	5d00084	um02641	1.00E-129	um02641	5d00084	1.00E-150
BBH	5d00067	um10308	0	um10308	5d00067	0
BBH	5c00025	um02755	0	um02755	5c00025	0
BBH	5c00164	um02508	0	um02508	5c00164	0
BBH	5c00027	um15091	6.00E-94	um15091	5c00027	1.00E-110
BBH	5d00039	um11720	0	um11720	5d00039	0
BBH	5d00061	um10315	2.00E-18	um10315	5d00061	1.00E-19
BBH	5c00062	um11922	0	um11922	5c00062	0
BBH	5d00092	um02628	0	um02628	5d00092	0
BBH	5c00097	um10294	0	um10294	5c00097	0
BBH	5d00141	um11413	1.00E-18	um11413	5d00141	2.00E-19
BBH	5c00003	um02810	0	um02810	5c00003	0
BBH	5c00008	um02796	0	um02796	5c00008	0
BBH	5c00125	um02574	1.00E-180	um02574	5c00125	1.00E-180
BBH	5d00052	um11927	4.00E-80	um11927	5d00052	2.00E-80
BBH	5c00101	um10285	0	um10285	5c00101	0
BBH	5c00083	um02651	1.00E-170	um02651	5c00083	1.00E-174
BBH	5d00130	um02552	0	um02552	5d00130	0
BBH	5c00138	um02546	1.00E-109	um02546	5c00138	1.00E-121
BBH	5d00051	um11925	2.00E-51	um11925	5d00051	3.00E-51
BBH	5c00076	um02665	8.00E-41	um02665	5c00076	6.00E-40
BBH	5c00071	um10307	1.00E-150	um10307	5c00071	1.00E-148
BBH	5c00052	um11926	0	um11926	5c00052	0
BBH	5c00118	um02584	4.00E-62	um02584	5c00118	4.00E-62
BBH	5d00108	um02596	5.00E-50	um02596	5d00108	5.00E-51
BBH	5d00112	um10279	0	um10279	5d00112	0
BBH	5d00003	um02799	0	um02799	5d00003	0
BBH	5d00096	um10293.2	0	um10293.2	5d00096	0
BBH	5c00150	um02527	0	um02527	5c00150	0
BBH	5c00111	um02599	1.00E-179	um02599	5c00111	0
BBH	5d00019	um02760	0	um02760	5d00019	0
BBH	5c00044	um02717	2.00E-85	um02717	5c00044	1.00E-101
BBH	5d00006	um10604	0	um10604	5d00006	0
BBH	5d00118	um02575	1.00E-161	um02575	5d00118	1.00E-161
BBH	5d00042	um02712	3.00E-27	um02712	5d00042	3.00E-30
BBH	5c00090	um10295	0	um10295	5c00090	0
BBH	5c00059	um11928	1.00E-144	um11928	5c00059	1.00E-145
BBH	5d00077	um02653	0	um02653	5d00077	0
NNN	5c00144	um02535	1.00E-14	um02535	5c00145	1.00E-14
BBH	5c00032	um11727.2	1.00E-138	um11727.2	5c00032	1.00E-129
BBH	5d00109	um02594.2	1.00E-164	um02594.2	5d00109	1.00E-163
NNN	5d00069	um02668	0			
BBH	5d00105	um02607	0	um02607	5d00105	0
BBH	5c00120	um02581	0	um02581	5c00120	0
BBH	5d00091	um02626	0	um02626	5d00091	0
BBH	5d00126	um02561	0	um02561	5d00126	0
BBH	5c00163	um11406	0	um11406	5c00163	0
NNN	5c00096	um02632	5.00E-69			
BBH	5d00064	um10311	7.00E-31	um10311	5d00064	4.00E-32
BBH	5c00162	um02510	7.00E-97	um02510	5c00162	1.00E-122
BBH	5c00132	um11715	0	um11715	5c00132	0
BBH	5c00016	um02774	1.00E-162	um02774	5c00016	1.00E-161
NNN	5d00029	um02736	1.00E-36	um02736	5c00034	0
BBH	5c00034	um02736	0	um02736	5c00034	0
NNN	5c00102	um02615	1.00E-165			
BBH	5c00112	um02597	0	um02597	5c00112	0
NNN	5c00022	um04481	2.00E-49	um04481	11d00086	0
NNN	5d00071	um02662	1.00E-86	um02662	5d00072	0
BBH	5c00077	um02664	0	um02664	5c00077	0
BBH	5d00078	um02652	2.00E-16	um02652	5d00078	2.00E-26
BBH	5d00041	um02711	0	um02711	5d00041	0

BBH	5c00148	um02530	3.00E-91	um02530	5c00148	8.00E-82
BBH	5d00099	um10287	0	um10287	5d00099	0
BBH	5d00100	um10286	3.00E-56	um10286	5d00100	1.00E-56
BBH	5c00058	um02701.2	1.00E-114	um02701.2	5c00058	1.00E-115
BBH	5d00136	um02531	1.00E-152	um02531	5d00136	1.00E-152
BBH	5d00004	um02792	0	um02792	5d00004	0
NNN	5c00143	um02535	6.00E-16	um02535	5c00145	1.00E-14
BBH	5d00115	um02583	0	um02583	5d00115	0
BBH	5c00115	um02591	0	um02591	5c00115	0
BBH	5c00082	um02654	1.00E-142	um02654	5c00082	1.00E-140
BBH	5d00110	um02592	1.00E-174	um02592	5d00110	1.00E-177
NNN	5d00059	um10313	5.00E-38	um10313	16c00076	2.00E-51
BBH	5c00098	um02619	0	um02619	5c00098	0
NNN	5c00157	um11410	0			
BBH	5c00073	um02683	0	um02683	5c00073	0
BBH	5c00005	um02802	0	um02802	5c00005	0
BBH	5d00079	um02649	0	um02649	5d00079	0
BBH	5c00029	um02742	0	um02742	5c00029	0
BBH	5c00091	um02620	1.00E-95	um02620	5c00091	5.00E-92
BBH	5c00153	um02521	1.00E-177	um02521	5c00153	1.00E-167
BBH	5c00064	um15061.2	0	um15061.2	5c00064	0
BBH	5c00011	um02784	1.00E-165	um02784	5c00011	0
NNN	5c00043	um02719	1.00E-165			
BBH	5d00085	um02639	0	um02639	5d00085	0
BBH	5d00037	um02720.2	1.00E-165	um02720.2	5d00037	1.00E-166
BBH	5d00090	um02624	1.00E-178	um02624	5d00090	1.00E-167
NNN	5c00114	um10280	6.00E-31			
BBH	5c00092	um02622	1.00E-101	um02622	5c00092	9.00E-89
BBH	5c00042	um02725	0	um02725	5c00042	0
NNN	5c00057	um02700	7.00E-73			
BBH	5c00113	um02595	0	um02595	5c00113	0
BBH	5d00001	um11961	3.00E-21	um11961	5d00001	3.00E-21
BBH	5d00125	um02564	3.00E-69	um02564	5d00125	3.00E-67
BBH	5d00047	um02707	3.00E-57	um02707	5d00047	2.00E-56
BBH	5d00114	um02586	0	um02586	5d00114	0
BBH	5c00149	um02529	0	um02529	5c00149	0
BBH	5d00089	um02623	0	um02623	5d00089	0
BBH	5d00106	um02600	0	um02600	5d00106	0
BBH	5d00098	um02618.2	1.00E-165	um02618.2	5d00098	1.00E-174
BBH	5d00143	um11412	1.00E-34	um11412	5d00143	1.00E-34
BBH	5c00123	um02577	0	um02577	5c00123	0
BBH	5c00145	um02535	1.00E-14	um02535	5c00145	1.00E-14
BBH	5d00058	um02678	1.00E-104	um02678	5d00058	1.00E-110
NNN	5d00122	um02568	0			
BBH	5d00072	um02662	0	um02662	5d00072	0
BBH	5c00079	um10305	0	um10305	5c00079	0
BBH	5c00131	um02556	2.00E-76	um02556	5c00131	3.00E-43
BBH	5d00020	um02754	2.00E-94	um02754	5d00020	2.00E-94
BBH	5d00137	um02528	0	um02528	5d00137	0
BBH	5c00050	um11716	3.00E-40	um11716	5c00050	3.00E-40
BBH	5d00012	um02778	0	um02778	5d00012	0
BBH	5c00010	um10605	1.00E-167	um10605	5c00010	1.00E-155
NNN	5c00037	um02732	7.00E-24			
BBH	5d00103	um10284	0	um10284	5d00103	0
BBH	5c00105	um02610	1.00E-137	um02610	5c00105	1.00E-129
BBH	5c00154	um02519	0	um02519	5c00154	0
NNN	5d00011	um01868	1.00E-49	um01868	22d00215	0
BBH	5c00019	um02768	4.00E-34	um02768	5c00019	4.00E-34
BBH	5c00147	um11414	1.00E-105	um11414	5c00147	1.00E-114
BBH	5d00057	um02676	0	um02676	5d00057	0
NNN	5d00068	um02685	0			
BBH	5d00017	um02765	0	um02765	5d00017	0

BBH	5d00120	um02571	0	um02571	5d00120	0
BBH	5c00085	um10302.2	2.00E-33	um10302.2	5c00085	9.00E-51
BBH	5d00031	um02733.2	0	um02733.2	5d00031	0
BBH	5d00086	um02638	0	um02638	5d00086	0
BBH	5c00065	um15096	1.00E-137	um15096	5c00065	1.00E-135
BBH	5c00026	um02750	1.00E-133	um02750	5c00026	1.00E-142
BBH	5c00024	um02757	1.00E-165	um02757	5c00024	1.00E-175
BBH	5d00062	um02686.2	0	um02686.2	5d00062	0
BBH	5c00126	um02572	1.00E-176	um02572	5c00126	1.00E-180
NNN	5c00146	um02535	9.00E-13	um02535	5c00145	1.00E-14
BBH	5d00138	um02525.2	0	um02525.2	5d00138	0
BBH	5d00097	um02635	3.00E-85	um02635	5d00097	5.00E-86
BBH	5d00142	um02520	0	um02520	5d00142	0
BBH	5c00056	um02694	1.00E-63	um02694	5c00056	4.00E-67
BBH	5c00117	um02585	0	um02585	5c00117	0
BBH	5d00002	um02801	0	um02801	5d00002	0
BBH	5c00084	um12194	1.00E-163	um12194	5c00084	1.00E-159
BBH	5d00050	um02697	0	um02697	5d00050	0
BBH	5d00128	um02557	0	um02557	5d00128	0
BBH	5d00018	um02763	0	um02763	5d00018	0
BBH	5c00036	um11723	0	um11723	5c00036	0
BBH	5c00134	um02553	1.00E-133	um02553	5c00134	1.00E-118
BBH	5c00070	um02672	8.00E-56	um02672	5c00070	4.00E-51
NNN	5c00100	um15047	0			
BBH	5c00109	um02603	0	um02603	5c00109	0
BBH	5c00018	um02771	8.00E-89	um02771	5c00018	9.00E-91
BBH	5d00104	um10283	0	um10283	5d00104	0
BBH	5c00133	um02555	1.00E-106	um02555	5c00133	1.00E-113
BBH	5d00022	um02752	0	um02752	5d00022	0
BBH	5d00055	um11924	0	um11924	5d00055	0
BBH	5c00038	um02730	0	um02730	5c00038	0
BBH	5c00041	um02721	1.00E-117	um02721	5c00041	1.00E-119
BBH	5d00014	um02772	1.00E-96	um02772	5d00014	9.00E-99
BBH	5d00113	um02587	0	um02587	5d00113	0
BBH	5c00055	um02696	0	um02696	5c00055	0
BBH	5c00119	um02582	0	um02582	5c00119	0
BBH	5c00087	um10300	0	um10300	5c00087	0
BBH	5d00046	um02704	0	um02704	5d00046	0
BBH	5d00073	um10306	1.00E-125	um10306	5d00073	1.00E-127
BBH	5c00106	um02606	1.00E-145	um02606	5c00106	1.00E-120
BBH	5c00021	um11931	1.00E-83	um11931	5c00021	7.00E-87
BBH	5d00124	um10276	0	um10276	5d00124	0
NNN	5c00080	um10304	1.00E-170			
BBH	5d00036	um02724	0	um02724	5d00036	0
BBH	5d00074	um02659.2	1.00E-128	um02659.2	5d00074	1.00E-137
BBH	5d00131	um02549	1.00E-61	um02549	5d00131	2.00E-87
BBH	5d00030	um11724	1.00E-119	um11724	5d00030	1.00E-128
BBH	5d00080	um10299	2.00E-95	um10299	5d00080	1.00E-91
BBH	5d00087	um02637	1.00E-166	um02637	5d00087	1.00E-174
BBH	5d00127	um02559	2.00E-61	um02559	5d00127	3.00E-61
BBH	5c00151	um02526	2.00E-55	um02526	5c00151	3.00E-47
BBH	5c00155	um02517	0	um02517	5c00155	0
BBH	5c00028	um11929	0	um11929	5c00028	0
BBH	5d00101	um12193.2	0	um12193.2	5d00101	0
BBH	5c00031	um02739	0	um02739	5c00031	0
BBH	5d00040	um02710	4.00E-34	um02710	5d00040	4.00E-34
NNN	5c00004	um01711	3.00E-65	um01711	22d00133	0
BBH	5d00129	um02554	0	um02554	5d00129	0
BBH	5d00056	um02690	1.00E-165	um02690	5d00056	1.00E-152
BBH	5d00021	um02753	5.00E-99	um02753	5d00021	1.00E-96
NNN	5c00152	um02524	0			
NNN	5c00012	um10602	0			

BBH	5d00081	um02645	1.00E-77	um02645	5d00081	1.00E-78
BBH	5d00013	um10598	1.00E-80	um10598	5d00013	1.00E-80
BBH	5c00015	um02775	2.00E-75	um02775	5c00015	2.00E-76
BBH	5d00026	um02740	0	um02740	5d00026	0
BBH	5d00066	um10309	0	um10309	5d00066	0
NNN	5c00086	um10301	7.00E-85			
BBH	5d00132	um02548	0	um02548	5d00132	0
BBH	5c00103	um02613	0	um02613	5c00103	0
BBH	5d00045	um11717	0	um11717	5d00045	0
BBH	5c00136	um02550	2.00E-38	um02550	5c00136	4.00E-36
BBH	5c00049	um11718	0	um11718	5c00049	0
BBH	5c00066	um02688	1.00E-137	um02688	5c00066	1.00E-149
BBH	5d00007	um02783	0	um02783	5d00007	0
BBH	5d00016	um10597	2.00E-92	um10597	5d00016	1.00E-103
BBH	5c00129	um02560	4.00E-98	um02560	5c00129	4.00E-98
BBH	5d00024	um02751	8.00E-73	um02751	5d00024	9.00E-76
BBH	5d00038	um02718	0	um02718	5d00038	0
BBH	5c00156	um11411	3.00E-50	um11411	5c00156	2.00E-51
BBH	5d00035	um02723.2	0	um02723.2	5d00035	0
BBH	5d00095	um02631	0	um02631	5d00095	0
BBH	5d00048	um02708	5.00E-17	um02708	5d00048	3.00E-31
BBH	5c00140	um02543	0	um02543	5c00140	0
BBH	5c00072	um02684	1.00E-163	um02684	5c00072	1.00E-164
BBH	5d00121	um10277.2	0	um10277.2	5d00121	0
BBH	5c00078	um02663	1.00E-122	um02663	5c00078	1.00E-118
BBH	5c00039	um02729	1.00E-145	um02729	5c00039	1.00E-147
BBH	5c00124	um02576	0	um02576	5c00124	0
BBH	5d00065	um10310	1.00E-157	um10310	5d00065	1.00E-159
BBH	5c00007	um02797	6.00E-37	um02797	5c00007	1.00E-34
BBH	5d00102	um02614	0	um02614	5d00102	0
BBH	5d00088	um10290	0	um10290	5d00088	0
BBH	5c00093	um02625	0	um02625	5c00093	0
BBH	5d00008	um02782	0	um02782	5d00008	0
BBH	5c00128	um02562	0	um02562	5c00128	0
NNN	5d00116	um02579	2.00E-90			
NNN	5c00046	um02716	2.00E-40			
BBH	5d00009	um02779	0	um02779	5d00009	0
BBH	5d00133	um11713	0	um11713	5d00133	0
BBH	5c00074	um02682	0	um02682	5c00074	0
BBH	5c00089	um02640	1.00E-179	um02640	5c00089	0
BBH	5d00075	um02657	0	um02657	5d00075	0
BBH	5d00147	um11409	0	um11409	5d00147	0
BBH	5d00044	um11719	1.00E-137	um11719	5d00044	1.00E-137
BBH	5c00107	um02605.2	0	um02605.2	5c00107	0
BBH	5d00140	um02523	1.00E-102	um02523	5d00140	1.00E-103
BBH	5c00069	um02674	7.00E-34	um02674	5c00069	4.00E-30
BBH	5c00014	um02777	1.00E-125	um02777	5c00014	1.00E-125
BBH	5c00023	um02758	1.00E-175	um02758	5c00023	1.00E-175
BBH	5c00130	um02558	1.00E-140	um02558	5c00130	1.00E-151
BBH	5c00033	um11725	1.00E-178	um11725	5c00033	0
BBH	5c00135	um02551	4.00E-35	um02551	5c00135	2.00E-40
BBH	5c00139	um15046	1.00E-173	um15046	5c00139	1.00E-173
BBH	5d00053	um02703	0	um02703	5d00053	0
BBH	5c00095	um02630	0	um02630	5c00095	0
BBH	5d00063	um02687	0	um02687	5d00063	0
BBH	5c00141	um02542	1.00E-103	um02542	5c00141	1.00E-100
BBH	5c00104	um02611	6.00E-83	um02611	5c00104	8.00E-97
BBH	5c00006	um10607	3.00E-37	um10607	5c00006	1.00E-42
BBH	5d00025	um02743	1.00E-142	um02743	5d00025	1.00E-144
BBH	5d00049	um02693	0	um02693	5d00049	0
BBH	5d00034	um11722	1.00E-174	um11722	5d00034	1.00E-175
NNN	5c00035	um02736	3.00E-15	um02736	5c00034	0

BBH	5c00088	um10296	2.00E-37	um10296	5c00088	9.00E-38
BBH	5d00093	um02629	1.00E-139	um02629	5d00093	1.00E-139
BBH	5c00122	um02578	0	um02578	5c00122	0
BBH	5d00146	um02514.2	1.00E-63	um02514.2	5d00146	1.00E-63
BBH	5c00068	um10312	8.00E-42	um10312	5c00068	8.00E-42
BBH	5d00043	um02713	1.00E-111	um02713	5d00043	1.00E-106
BBH	5d00005	um02791	0	um02791	5d00005	0
BBH	5d00134	um11418	2.00E-22	um11418	5d00134	4.00E-22
BBH	5c00127	um12192	5.00E-72	um12192	5c00127	3.00E-66
BBH	5c00047	um02715	0	um02715	5c00047	0
BBH	5c00081	um02656	0	um02656	5c00081	0
BBH	5d00028	um11728	3.00E-46	um11728	5d00028	5.00E-46
NNN	5c00160	um11407	0			
BBH	5c00040	um02727	5.00E-82	um02727	5c00040	2.00E-83
BBH	5d00083	um10297	1.00E-121	um10297	5d00083	1.00E-130
BBH	5d00076	um02655	5.00E-55	um02655	5d00076	2.00E-51
BBH	5c00159	um11408.2	1.00E-165	um11408.2	5c00159	1.00E-165
BBH	5c00017	um02773	4.00E-45	um02773	5c00017	4.00E-45
BBH	5d00123	um02567	1.00E-168	um02567	5d00123	1.00E-163
NNN	5d00023	um05151	1.00E-29	um05151	2d00033	1.00E-142
BBH	5c00075	um02669.2	2.00E-90	um02669.2	5c00075	2.00E-90
BBH	5c00067	um02677	0	um02677	5c00067	0
BBH	5c00009	um10606	0	um10606	5c00009	0
BBH	5c00094	um02627	7.00E-44	um02627	5c00094	1.00E-45
BBH	5d00107	um02598	0	um02598	5d00107	0
BBH	5c00030	um02741	0	um02741	5c00030	0
BBH	5d00145	um02516	2.00E-75	um02516	5d00145	1.00E-77
BBH	5c00060	um11923	0	um11923	5c00060	0
BBH	5c00121	um02580.2	1.00E-131	um02580.2	5c00121	1.00E-125
BBH	5c00142	um02540	1.00E-20	um02540	5c00142	1.00E-20
BBH	5d00033	um02731	0	um02731	5d00033	1.00E-175
BBH	5c00110	um02602	0	um02602	5c00110	0
BBH	5c00020	um10596	0	um10596	5c00020	0
BBH	5d00082	um02642	1.00E-76	um02642	5d00082	2.00E-82
NNN	6c00077	um10063	0			
BBH	6c00044	um02206	1.00E-147	um02206	6c00044	1.00E-146
BBH	6d00095	um02118	1.00E-165	um02118	6d00095	1.00E-174
BBH	6c00022	um15025	7.00E-72	um15025	6c00022	1.00E-66
BBH	6c00124	um11461	1.00E-174	um11461	6c00124	1.00E-175
BBH	6c00008	um05967	0	um05967	6c00008	0
BBH	6c00121	um02063	0	um02063	6c00121	0
BBH	6c00130	um11908	0	um11908	6c00130	0
BBH	6c00115	um02075	0	um02075	6c00115	0
BBH	6d00076	um02163	1.00E-127	um02163	6d00076	1.00E-121
BBH	6d00042	um02220	0	um02220	6d00042	0
BBH	6c00026	um12026	1.00E-133	um12026	6c00026	1.00E-141
BBH	6d00082	um02150	0	um02150	6d00082	0
BBH	6c00094	um11465	3.00E-43	um11465	6c00094	2.00E-42
BBH	6c00064	um02172	1.00E-171	um02172	6c00064	1.00E-168
BBH	6c00083	um02125	0	um02125	6c00083	0
BBH	6d00025	um02238	3.00E-55	um02238	6d00025	6.00E-55
BBH	6c00007	um05968	0	um05968	6c00007	0
BBH	6d00061	um10067	7.00E-47	um10067	6d00061	1.00E-48
BBH	6c00109	um15090	0	um15090	6c00109	0
BBH	6d00060	um02190.2	1.00E-41	um02190.2	6d00060	5.00E-33
BBH	6d00124	um02055	0	um02055	6d00124	0
NNN	6c00015	um11635	4.00E-22	um11635	13c00107	8.00E-87
BBH	6d00101	um02101	2.00E-72	um02101	6d00101	4.00E-72
BBH	6d00094	um10055	0	um10055	6d00094	0
BBH	6d00119	um02066	1.00E-101	um02066	6d00119	1.00E-129
BBH	6d00038	um02226	1.00E-129	um02226	6d00038	1.00E-139
BBH	6d00114	um11911	1.00E-148	um11911	6d00114	1.00E-148

BBH	6d00008	um05964	0	um05964	6d00008	0
BBH	6c00060	um02178	1.00E-144	um02178	6c00060	1.00E-143
NNN	6d00015	um05944	1.00E-11			
BBH	6c00129	um02045	4.00E-43	um02045	6c00129	8.00E-43
BBH	6d00054	um02793	0	um02793	6d00054	0
BBH	6c00123	um11462	1.00E-141	um11462	6c00123	1.00E-151
BBH	6c00078	um02134	1.00E-138	um02134	6c00078	1.00E-129
BBH	6d00135	um15031	0	um15031	6d00135	0
BBH	6d00053	um02203	0	um02203	6d00053	0
NNN	6d00022	um05928	2.00E-17	um05928	6d00023	5.00E-26
BBH	6c00137	um06493	0	um06493	6c00137	0
BBH	6c00021	um12066	2.00E-96	um12066	6c00021	7.00E-89
BBH	6c00042	um02208	0	um02208	6c00042	0
BBH	6c00025	um05937	0	um05937	6c00025	0
BBH	6c00055	um02185	8.00E-83	um02185	6c00055	5.00E-83
BBH	6c00099	um12182	2.00E-74	um12182	6c00099	6.00E-73
NNN	6c00103	um02103	3.00E-69			
BBH	6c00002	um05974	1.00E-118	um05974	6c00002	1.00E-110
BBH	6c00116	um11912	1.00E-156	um11912	6c00116	1.00E-172
BBH	6d00129	um02046	1.00E-134	um02046	6d00129	1.00E-137
BBH	6d00075	um02164	0	um02164	6d00075	0
BBH	6c00043	um02207	0	um02207	6c00043	0
BBH	6c00058	um10066	2.00E-58	um10066	6c00058	2.00E-58
BBH	6d00083	um02156.2	6.00E-85	um02156.2	6d00083	8.00E-88
BBH	6c00086	um10056	0	um10056	6c00086	0
BBH	6d00016	um05942	1.00E-138	um05942	6d00016	1.00E-168
BBH	6c00063	um02175	0	um02175	6c00063	0
BBH	6c00110	um15077	0	um15077	6c00110	0
BBH	6d00069	um10064	0	um10064	6d00069	0
BBH	6d00068	um02179	6.00E-95	um02179	6d00068	1.00E-86
BBH	6d00032	um02229	7.00E-26	um02229	6d00032	7.00E-26
BBH	6c00036	um02221	0	um02221	6c00036	0
BBH	6c00117	um15045	0	um15045	6c00117	0
BBH	6d00104	um02113	7.00E-43	um02113	6d00104	1.00E-50
BBH	6d00113	um11913	0	um11913	6d00113	0
BBH	6d00012	um05951	0	um05951	6d00012	0
BBH	6d00126	um02052	0	um02052	6d00126	0
BBH	6c00101	um02102	0	um02102	6c00101	0
BBH	6d00074	um02165	2.00E-95	um02165	6d00074	2.00E-95
BBH	6c00114	um02077	1.00E-105	um02077	6c00114	1.00E-107
BBH	6c00006	um05971	0	um05971	6c00006	0
BBH	6c00039	um02216	1.00E-114	um02216	6c00039	1.00E-119
BBH	6c00108	um02088	1.00E-147	um02088	6c00108	1.00E-145
BBH	6c00091	um02116	0	um02116	6c00091	0
BBH	6d00080	um02147	0	um02147	6d00080	0
BBH	6d00120	um02064	4.00E-53	um02064	6d00120	8.00E-54
BBH	6c00080	um10060.2	6.00E-99	um10060.2	6c00080	1.00E-112
BBH	6d00045	um02215	0	um02215	6d00045	0
BBH	6c00071	um02158	0	um02158	6c00071	0
BBH	6c00136	um06491	0	um06491	6c00136	0
BBH	6c00041	um10073	0	um10073	6c00041	0
BBH	6d00108	um12181	0	um12181	6d00108	0
BBH	6c00050	um02198	4.00E-73	um02198	6c00050	5.00E-73
BBH	6d00089	um02133	1.00E-124	um02133	6d00089	1.00E-112
BBH	6c00127	um11458	0	um11458	6c00127	0
BBH	6c00017	um05952	0	um05952	6c00017	0
BBH	6d00117	um02070	1.00E-110	um02070	6d00117	1.00E-113
BBH	6d00092	um02127	1.00E-75	um02127	6d00092	2.00E-76
BBH	6c00052	um02191	1.00E-145	um02191	6c00052	1.00E-151
NNN	6c00062	um10065	4.00E-70			
BBH	6d00056	um02197	1.00E-162	um02197	6d00056	1.00E-156
BBH	6d00044	um02217	0	um02217	6d00044	0

BBH	6c00072	um02144	0	um02144	6c00072	0
BBH	6d00050	um10072	0	um10072	6d00050	0
BBH	6c00066	um02168.2	0	um02168.2	6c00066	0
NNN	6c00107	um11470	9.00E-44			
BBH	6c00088	um02202	4.00E-63	um02202	6c00088	4.00E-71
BBH	6c00085	um02123	0	um02123	6c00085	0
BBH	6d00067	um02180	0	um02180	6d00067	0
BBH	6c00100	um12183	0	um12183	6c00100	0
BBH	6c00027	um12025	1.00E-121	um12025	6c00027	1.00E-133
BBH	6c00122	um02062	0	um02062	6c00122	0
BBH	6c00092	um02115	0	um02115	6c00092	0
BBH	6c00038	um02218	0	um02218	6c00038	0
BBH	6d00073	um02166	0	um02166	6d00073	0
NNN	6c00102	um02103	1.00E-108			
BBH	6d00107	um02089	0	um02089	6d00107	1.00E-171
BBH	6d00102	um02108	0	um02108	6d00102	0
BBH	6c00135	um12074	0	um12074	6c00135	0
BBH	6c00005	um05972	0	um05972	6c00005	0
BBH	6c00061	um02177.2	0	um02177.2	6c00061	0
BBH	6d00007	um05966	0	um05966	6d00007	0
BBH	6d00081	um02148	0	um02148	6d00081	0
BBH	6c00082	um02126	1.00E-156	um02126	6c00082	1.00E-170
BBH	6d00047	um02211	0	um02211	6d00047	1.00E-180
BBH	6d00055	um02199	1.00E-119	um02199	6d00055	1.00E-123
BBH	6d00123	um11460	0	um11460	6d00123	0
NNN	6d00091	um10059	7.00E-33			
BBH	6d00014	um05947.2	2.00E-69	um05947.2	6d00014	7.00E-80
BBH	6d00093	um10057	1.00E-65	um10057	6d00093	2.00E-65
BBH	6c00065	um02169	0	um02169	6c00065	0
NNN	6d00072	um02167	1.00E-152			
BBH	6d00019	um05936	1.00E-135	um05936	6d00019	1.00E-135
BBH	6c00032	um02235	0	um02235	6c00032	0
BBH	6d00021	um05933	0	um05933	6d00021	0
NNN	6d00087	um02135	2.00E-29	um02135	6d00088	1.00E-30
BBH	6c00112	um11915	3.00E-34	um11915	6c00112	1.00E-33
BBH	6d00066	um02181	0	um02181	6d00066	0
BBH	6d00026	um02151	1.00E-136	um02151	6d00026	1.00E-136
BBH	6d00058	um02196	4.00E-27	um02196	6d00058	3.00E-29
BBH	6d00037	um02227	0	um02227	6d00037	0
BBH	6d00128	um11910	1.00E-173	um11910	6d00128	1.00E-177
BBH	6d00115	um02072	1.00E-140	um02072	6d00115	1.00E-140
BBH	6c00106	um02104.2	0	um02104.2	6c00106	0
BBH	6d00110	um02081	0	um02081	6d00110	0
BBH	6c00011	um05961	1.00E-178	um05961	6c00011	0
BBH	6c00079	um02132	1.00E-122	um02132	6c00079	1.00E-129
BBH	6c00070	um02159	0	um02159	6c00070	0
BBH	6d00105	um02111	1.00E-142	um02111	6d00105	1.00E-142
BBH	6d00100	um11468	0	um11468	6d00100	0
BBH	6c00059	um02182	1.00E-154	um02182	6c00059	1.00E-154
BBH	6c00120	um02065	0	um02065	6c00120	0
BBH	6d00010	um10863	1.00E-172	um10863	6d00010	1.00E-169
NNN	6c00048	um10071	6.00E-37			
BBH	6c00045	um02201.2	1.00E-139	um02201.2	6c00045	1.00E-135
BBH	6c00054	um10069	1.00E-157	um10069	6c00054	1.00E-155
BBH	6c00119	um02068	8.00E-24	um02068	6c00119	9.00E-26
BBH	6d00046	um02212	0	um02212	6d00046	0
BBH	6c00074	um02149	2.00E-71	um02149	6c00074	1.00E-72
BBH	6c00134	um02035	1.00E-162	um02035	6c00134	1.00E-152
BBH	6d00071	um02170	1.00E-159	um02170	6d00071	1.00E-159
BBH	6c00097	um02095	1.00E-147	um02095	6c00097	1.00E-151
BBH	6c00068	um02162	4.00E-70	um02162	6c00068	4.00E-70
BBH	6d00131	um02039	1.00E-137	um02039	6d00131	1.00E-137

BBH	6c00081	um02128	1.00E-101	um02128	6c00081	7.00E-89
BBH	6d00079	um12094	9.00E-41	um12094	6d00079	9.00E-41
BBH	6d00065	um12097	4.00E-33	um12097	6d00065	4.00E-33
BBH	6c00098	um02097	1.00E-164	um02097	6c00098	1.00E-143
BBH	6c00031	um05923	1.00E-128	um05923	6c00031	1.00E-127
BBH	6c00133	um11907.2	0	um11907.2	6c00133	1.00E-180
BBH	6c00012	um05960	0	um05960	6c00012	0
BBH	6d00088	um02135	3.00E-22	um02135	6d00088	1.00E-30
BBH	6c00019	um05949	0	um05949	6c00019	0
BBH	6d00090	um02131	1.00E-112	um02131	6d00090	1.00E-105
BBH	6d00070	um12096	1.00E-119	um12096	6d00070	1.00E-122
BBH	6d00031	um02232	7.00E-49	um02232	6d00031	3.00E-47
BBH	6d00009	um10864	7.00E-80	um10864	6d00009	7.00E-80
BBH	6d00011	um05954	1.00E-156	um05954	6d00011	1.00E-163
BBH	6c00113	um02079	1.00E-45	um02079	6c00113	2.00E-47
BBH	6d00027	um12316	0	um12316	6d00027	0
BBH	6d00036	um10075	1.00E-42	um10075	6d00036	1.00E-42
BBH	6d00118	um02067	1.00E-123	um02067	6d00118	1.00E-132
BBH	6d00109	um02083	0	um02083	6d00109	0
NNN	6d00132	um02037	0			
BBH	6c00047	um02794	2.00E-65	um02794	6c00047	2.00E-64
BBH	6c00128	um02049	0	um02049	6c00128	0
BBH	6d00098	um02093	1.00E-114	um02093	6d00098	1.00E-101
BBH	6d00130	um02044	0	um02044	6d00130	0
NNN	6d00002	um03408	4.00E-47	um03408	8c00128	0
BBH	6d00041	um02223	1.00E-174	um02223	6d00041	1.00E-174
BBH	6c00067	um12095	4.00E-43	um12095	6c00067	6.00E-47
BBH	6c00090	um02117	1.00E-115	um02117	6c00090	1.00E-123
BBH	6c00051	um10070	0	um10070	6c00051	0
NNN	6d00125	um02053	0			
BBH	6c00056	um11306	1.00E-37	um11306	6c00056	1.00E-47
BBH	6d00103	um11469	3.00E-88	um11469	6d00103	8.00E-87
BBH	6d00040	um02224	0	um02224	6d00040	0
BBH	6d00030	um02236	6.00E-88	um02236	6d00030	2.00E-93
BBH	6c00013	um05959	0	um05959	6c00013	0
BBH	6d00028	um10077	0	um10077	6d00028	0
NNN	6d00003	um04886	2.00E-15	um04886	26d00001	0
NNN	6d00005	um04046	4.00E-49	um04046	13c00097	0
BBH	6d00039	um02225	9.00E-98	um02225	6d00039	2.00E-91
BBH	6d00017	um05940	5.00E-66	um05940	6d00017	1.00E-60
NNN	6c00030	um05924	4.00E-25	um05924	6c00108	1.00E-36
BBH	6d00097	um02092	9.00E-46	um02092	6d00097	1.00E-47
BBH	6d00063	um10068	0	um10068	6d00063	0
BBH	6c00095	um02091	1.00E-148	um02091	6c00095	1.00E-149
BBH	6c00035	um10074	1.00E-44	um10074	6c00035	9.00E-46
BBH	6c00132	um02040	1.00E-166	um02040	6c00132	1.00E-166
BBH	6c00024	um05938	1.00E-135	um05938	6c00024	1.00E-139
BBH	6c00104	um02107	5.00E-74	um02107	6c00104	1.00E-77
BBH	6c00118	um02069	9.00E-59	um02069	6c00118	6.00E-59
BBH	6c00126	um11459.2	1.00E-104	um11459.2	6c00126	1.00E-112
BBH	6c00020	um10861	0	um10861	6c00020	1.00E-177
BBH	6d00084	um02157	0	um02157	6d00084	0
BBH	6c00075	um10062	0	um10062	6c00075	0
BBH	6d00023	um05925	0	um05925	6d00023	0
BBH	6d00133	um02034	1.00E-42	um02034	6d00133	1.00E-42
BBH	6d00049	um02209	0	um02209	6d00049	0
BBH	6c00009	um05965	4.00E-61	um05965	6c00009	2.00E-61
BBH	6c00014	um05958	0	um05958	6c00014	0
BBH	6d00096	um11464	3.00E-59	um11464	6d00096	4.00E-59
BBH	6d00029	um02234	3.00E-62	um02234	6d00029	3.00E-62
BBH	6d00004	um15026	0	um15026	6d00004	0
BBH	6d00006	um10898	1.00E-144	um10898	6d00006	1.00E-144

BBH	6c00105	um02105	1.00E-137	um02105	6c00105	1.00E-144
NNN	6c00053	um02189	1.00E-166			
BBH	6c00010	um10865	1.00E-150	um10865	6c00010	1.00E-150
BBH	6d00121	um04109	3.00E-56	um04109	6d00121	9.00E-59
BBH	6d00116	um02071	6.00E-35	um02071	6d00116	5.00E-30
BBH	6d00127	um02050	0	um02050	6d00127	0
BBH	6d00077	um02160	0	um02160	6d00077	0
BBH	6c00033	um02237	0	um02237	6c00033	0
BBH	6d00062	um02186	1.00E-59	um02186	6d00062	8.00E-66
BBH	6d00020	um05934	1.00E-106	um05934	6d00020	1.00E-97
BBH	6d00052	um02204	0	um02204	6d00052	0
BBH	6d00018	um05939	1.00E-69	um05939	6d00018	7.00E-67
BBH	6d00043	um12099	1.00E-37	um12099	6d00043	1.00E-45
BBH	6c00069	um02161	0	um02161	6c00069	0
BBH	6c00131	um02041	1.00E-166	um02041	6c00131	1.00E-153
BBH	6c00023	um12027	1.00E-54	um12027	6c00023	1.00E-54
BBH	6c00093	um02114	1.00E-132	um02114	6c00093	1.00E-120
BBH	6d00078	um10061	4.00E-66	um10061	6d00078	2.00E-62
BBH	6c00073	um02146	0	um02146	6c00073	0
BBH	6c00034	um11189	0	um11189	6c00034	0
BBH	6c00096	um02094	9.00E-56	um02094	6c00096	1.00E-55
NNN	6c00049	um02213	0			
BBH	6d00106	um11472	3.00E-98	um11472	6d00106	1.00E-103
BBH	6c00111	um11916	1.00E-42	um11916	6c00111	1.00E-42
BBH	6d00134	um11905	0	um11905	6d00134	0
BBH	6c00076	um02154	0	um02154	6c00076	0
BBH	6c00040	um02214.2	1.00E-177	um02214.2	6c00040	1.00E-173
BBH	6d00085	um02142	0	um02142	6d00085	0
BBH	6c00125	um02057	0	um02057	6c00125	0
BBH	6c00084	um02124	1.00E-100	um02124	6c00084	1.00E-101
BBH	7c00025	um05862	0	um05862	7c00025	0
BBH	7d00066	um02249	0	um02249	7d00066	0
BBH	7d00146	um01250	0	um01250	7d00146	0
BBH	7d00030	um05854	0	um05854	7d00030	0
BBH	7c00053	um05917	0	um05917	7c00053	0
BBH	7c00260	um01012	0	um01012	7c00260	0
BBH	7d00114	um02353	5.00E-84	um02353	7d00114	2.00E-84
BBH	7d00216	um01112	1.00E-133	um01112	7d00216	1.00E-129
BBH	7c00359	um05247	0	um05247	7c00359	0
BBH	7d00138	um01265	8.00E-71	um01265	7d00138	3.00E-86
BBH	7c00269	um00992	0	um00992	7c00269	0
BBH	7c00061	um02242	1.00E-171	um02242	7c00061	1.00E-169
BBH	7c00186	um11652	2.00E-60	um11652	7c00186	3.00E-64
BBH	7c00296	um00896	0	um00896	7c00296	0
BBH	7c00278	um04984	9.00E-88	um04984	7c00278	1.00E-96
BBH	7c00038	um05889	0	um05889	7c00038	0
BBH	7d00213	um01115	0	um01115	7d00213	0
BBH	7d00326	um00886	0	um00886	7d00326	0
BBH	7c00075	um11396	0	um11396	7c00075	0
BBH	7d00009	um11191	0	um11191	7d00009	0
BBH	7d00229	um01087	0	um01087	7d00229	0
BBH	7c00002	um05801	0	um05801	7c00002	0
BBH	7c00167	um01186	0	um01186	7c00167	0
BBH	7c00211	um11283	1.00E-121	um11283	7c00211	1.00E-120
BBH	7d00300	um00947	0	um00947	7d00300	0
BBH	7c00206	um01121	1.00E-138	um01121	7c00206	1.00E-138
BBH	7d00044	um05891	2.00E-69	um05891	7d00044	8.00E-64
BBH	7d00293	um10587	0	um10587	7d00293	0
BBH	7d00080	um02282	1.00E-93	um02282	7d00080	1.00E-93
BBH	7d00305	um10611	2.00E-28	um10611	7d00305	2.00E-28
BBH	7c00110	um02297	7.00E-16	um02297	7c00110	1.00E-15
BBH	7d00241	um11852	0	um11852	7d00241	0

BBH	7d00092	um02304	2.00E-25	um02304	7d00092	8.00E-23
BBH	7c00121	um02357	0	um02357	7c00121	0
BBH	7c00228	um11277	2.00E-93	um11277	7c00228	2.00E-93
BBH	7d00048	um05899	1.00E-107	um05899	7d00048	1.00E-98
BBH	7c00114	um02345	3.00E-91	um02345	7c00114	1.00E-89
BBH	7c00273	um00990	1.00E-121	um00990	7c00273	1.00E-122
BBH	7c00081	um02276	1.00E-86	um02276	7c00081	7.00E-96
BBH	7c00007	um11187	4.00E-85	um11187	7c00007	7.00E-94
BBH	7c00070	um02254	7.00E-53	um02254	7c00070	9.00E-55
NNN	7d00170	um01200	0			
BBH	7d00272	um00988	1.00E-111	um00988	7d00272	1.00E-116
BBH	7d00279	um00982	1.00E-75	um00982	7d00279	7.00E-76
BBH	7d00154	um10593	0	um10593	7d00154	0
NNN	7c00042	um05894	3.00E-50	um05894	7c00041	5.00E-88
BBH	7c00245	um01045	1.00E-148	um01045	7c00245	1.00E-145
BBH	7d00355	um00841	0	um00841	7d00355	0
BBH	7d00002	um11184	0	um11184	7d00002	0
NNN	7d00334	um10570	1.00E-170			
BBH	7d00053	um05908	1.00E-101	um05908	7d00053	3.00E-88
BBH	7c00288	um00948	0	um00948	7c00288	0
BBH	7d00294	um10586	0	um10586	7d00294	0
BBH	7c00035	um05880	1.00E-156	um05880	7c00035	1.00E-157
BBH	7c00316	um00933	1.00E-117	um00933	7c00316	1.00E-131
BBH	7d00103	um11705	4.00E-36	um11705	7d00103	8.00E-41
BBH	7d00045	um05893	1.00E-141	um05893	7d00045	1.00E-141
BBH	7d00174	um01194	0	um01194	7d00174	0
BBH	7c00132	um02401	7.00E-54	um02401	7c00132	5.00E-52
BBH	7c00328	um11842	1.00E-12	um11842	7c00328	2.00E-18
BBH	7c00175	um01177	0	um01177	7c00175	0
BBH	7c00069	um02252	0	um02252	7c00069	1.00E-166
BBH	7c00106	um02331	0	um02331	7c00106	0
BBH	7d00249	um01062	0	um01062	7d00249	0
BBH	7d00317	um00919	1.00E-149	um00919	7d00317	1.00E-149
NNN	7c00297	um00898	0			
BBH	7c00352	um00840	0	um00840	7c00352	0
BBH	7c00257	um01017	0	um01017	7c00257	0
BBH	7d00283	um00975	2.00E-63	um00975	7d00283	5.00E-67
BBH	7d00145	um01251	2.00E-68	um01251	7d00145	5.00E-78
BBH	7c00344	um00851	1.00E-145	um00851	7c00344	1.00E-146
BBH	7c00313	um10620	1.00E-137	um10620	7c00313	1.00E-139
BBH	7d00341	um00866	1.00E-163	um00866	7d00341	1.00E-163
BBH	7c00233	um11273	0	um11273	7c00233	0
BBH	7c00309	um00922	1.00E-162	um00922	7c00309	1.00E-160
BBH	7d00263	um01004	3.00E-32	um01004	7d00263	7.00E-31
BBH	7d00124	um02400	0	um02400	7d00124	0
BBH	7c00199	um01139.2	1.00E-79	um01139.2	7c00199	4.00E-84
BBH	7d00189	um01161	0	um01161	7d00189	0
BBH	7c00300	um00903	0	um00903	7c00300	0
BBH	7d00320	um00928	3.00E-62	um00928	7d00320	1.00E-50
BBH	7c00153	um01216	0	um01216	7c00153	0
BBH	7d00109	um02342	1.00E-124	um02342	7d00109	1.00E-113
BBH	7d00091	um02305	7.00E-48	um02305	7d00091	1.00E-58
BBH	7d00228	um01088	0	um01088	7d00228	0
BBH	7d00196	um11649	1.00E-151	um11649	7d00196	1.00E-126
BBH	7d00008	um05811	0	um05811	7d00008	0
BBH	7c00326	um03228	0	um03228	7c00326	0
BBH	7d00278	um10789	5.00E-20	um10789	7d00278	9.00E-23
BBH	7c00076	um02266	0	um02266	7c00076	0
BBH	7d00310	um10614	9.00E-98	um10614	7d00310	1.00E-99
BBH	7d00067	um02253	0	um02253	7d00067	0
BBH	7d00137	um15037	1.00E-154	um15037	7d00137	1.00E-148
BBH	7c00261	um01010	3.00E-75	um01010	7c00261	3.00E-72

BBH	7c00010	um11192	4.00E-54	um11192	7c00010	2.00E-60
BBH	7c00123	um02360	6.00E-32	um02360	7c00123	2.00E-26
BBH	7d00269	um00998	6.00E-39	um00998	7d00269	4.00E-29
BBH	7d00243	um01033.2	0	um01033.2	7d00243	0
BBH	7c00028	um11023	7.00E-57	um11023	7c00028	4.00E-60
BBH	7d00113	um02350	0	um02350	7d00113	0
BBH	7d00059	um05916	0	um05916	7d00059	0
BBH	7d00001	um05437	0	um05437	7d00001	0
BBH	7c00232	um01064	1.00E-49	um01064	7c00232	3.00E-44
BBH	7c00120	um02356	0	um02356	7c00120	0
BBH	7c00270	um10790	0	um10790	7c00270	0
BBH	7c00082	um11401	0	um11401	7c00082	0
BBH	7d00099	um02321	0	um02321	7d00099	0
BBH	7d00242	um01030	2.00E-56	um01030	7d00242	1.00E-54
BBH	7d00049	um05901	6.00E-99	um05901	7d00049	1.00E-104
BBH	7c00111	um11709	2.00E-34	um11709	7c00111	3.00E-44
BBH	7c00168	um01184	1.00E-60	um01184	7c00168	6.00E-65
BBH	7d00252	um01055	0	um01055	7d00252	0
BBH	7c00115	um02347	0	um02347	7c00115	0
BBH	7c00015	um05827	0	um05827	7c00015	0
BBH	7c00207	um01116	0	um01116	7c00207	0
NNN	7c00229	um01076	1.00E-131			
BBH	7c00054	um05919	1.00E-177	um05919	7c00054	1.00E-178
BBH	7c00037	um11036	0	um11036	7c00037	0
BBH	7c00185	um01171	0	um01171	7c00185	0
BBH	7d00183	um01172	0	um01172	7d00183	0
BBH	7c00001	um05800	0	um05800	7c00001	0
BBH	7d00295	um00955	0	um00955	7d00295	0
BBH	7d00282	um11849	3.00E-39	um11849	7d00282	4.00E-39
BBH	7c00133	um10144	1.00E-118	um10144	7c00133	1.00E-109
BBH	7c00055	um11802	7.00E-41	um11802	7c00055	6.00E-40
BBH	7c00062	um02244	0	um02244	7c00062	0
BBH	7d00204	um01133	0	um01133	7d00204	0
BBH	7d00052	um05907	1.00E-160	um05907	7d00052	1.00E-161
BBH	7c00016	um05828	0	um05828	7c00016	0
BBH	7c00345	um11253	1.00E-125	um11253	7c00345	1.00E-147
BBH	7d00173	um01195	0	um01195	7d00173	0
BBH	7c00256	um01018	1.00E-67	um01018	7c00256	2.00E-67
BBH	7d00017	um05830	1.00E-35	um05830	7d00017	2.00E-38
BBH	7c00041	um05894	1.00E-88	um05894	7c00041	5.00E-88
BBH	7c00312	um00924	1.00E-164	um00924	7c00312	1.00E-165
BBH	7c00283	um10590.2	1.00E-110	um10590.2	7c00283	1.00E-104
BBH	7d00236	um01070	0	um01070	7d00236	0
BBH	7d00271	um00989	0	um00989	7d00271	0
BBH	7c00137	um10142	1.00E-111	um10142	7c00137	1.00E-111
BBH	7d00094	um02301	1.00E-163	um02301	7d00094	1.00E-164
BBH	7c00099	um11701	1.00E-114	um11701	7c00099	1.00E-101
BBH	7d00331	um00882	1.00E-174	um00882	7d00331	1.00E-175
BBH	7c00301	um00904	0	um00904	7c00301	0
BBH	7c00308	um00913	0	um00913	7c00308	0
BBH	7c00112	um02337	0	um02337	7c00112	0
BBH	7c00279	um11846	0	um11846	7c00279	0
BBH	7d00132	um02413	4.00E-70	um02413	7d00132	1.00E-71
BBH	7d00356	um00836	0	um00836	7d00356	0
BBH	7d00227	um01090	0	um01090	7d00227	0
BBH	7c00148	um01244	0	um01244	7c00148	0
BBH	7c00198	um11643	2.00E-62	um11643	7c00198	6.00E-65
BBH	7d00026	um05848	1.00E-102	um05848	7d00026	1.00E-105
BBH	7d00023	um05840	0	um05840	7d00023	0
BBH	7d00262	um10796	0	um10796	7d00262	0
BBH	7d00063	um12101	2.00E-32	um12101	7d00063	9.00E-33
BBH	7c00329	um11840	1.00E-129	um11840	7c00329	1.00E-124

BBH	7d00039	um11026	0	um11026	7d00039	0
BBH	7c00067	um10082	7.00E-93	um10082	7c00067	2.00E-93
BBH	7d00318	um00930	0	um00930	7d00318	0
BBH	7d00153	um01232	0	um01232	7d00153	0
BBH	7c00353	um11245	1.00E-171	um11245	7c00353	0
NNN	7d00144	um01252	0			
BBH	7c00191	um01150	1.00E-26	um01150	7c00191	1.00E-26
BBH	7c00244	um01042	0	um01042	7c00244	0
BBH	7c00299	um00902	0	um00902	7c00299	0
NNN	7c00098	um12186	0			
BBH	7d00342	um00864	1.00E-143	um00864	7d00342	1.00E-138
BBH	7c00208	um01117	1.00E-102	um01117	7c00208	1.00E-103
BBH	7c00177	um01173	1.00E-133	um01173	7c00177	1.00E-138
BBH	7d00302	um10625	9.00E-50	um10625	7d00302	3.00E-52
BBH	7c00045	um05898.2	1.00E-152	um05898.2	7c00045	1.00E-145
BBH	7c00122	um10129	0	um10129	7c00122	0
BBH	7c00027	um11021	0	um11021	7c00027	0
BBH	7c00339	um11259	2.00E-36	um11259	7c00339	3.00E-38
BBH	7d00209	um01123	0	um01123	7d00209	0
BBH	7d00161	um01215	0	um01215	7d00161	0
BBH	7c00226	um01080	0	um01080	7c00226	0
BBH	7d00231	um01082	4.00E-86	um01082	7d00231	2.00E-79
NNN	7d00148	um01245	6.00E-68	um01245	16c00084	5.00E-72
BBH	7d00046	um05895	1.00E-109	um05895	7d00046	1.00E-119
BBH	7c00022	um05839	0	um05839	7c00022	0
BBH	7c00275	um00984	1.00E-178	um00984	7c00275	1.00E-179
BBH	7c00018	um05831	0	um05831	7c00018	0
BBH	7c00036	um11035	0	um11035	7c00036	0
BBH	7c00184	um01170	0	um01170	7c00184	1.00E-178
BBH	7d00116	um02358	1.00E-139	um02358	7d00116	1.00E-123
BBH	7d00118	um10132	9.00E-22	um10132	7d00118	5.00E-19
BBH	7c00051	um11797	0	um11797	7c00051	0
BBH	7c00248	um11272	3.00E-94	um11272	7c00248	3.00E-98
BBH	7d00264	um10794	0	um10794	7d00264	0
BBH	7c00262	um01009	0	um01009	7c00262	0
NNN	7d00100	um02324	5.00E-72			
BBH	7d00064	um02243	2.00E-76	um02243	7d00064	2.00E-80
BBH	7d00028	um05850	1.00E-176	um05850	7d00028	1.00E-180
BBH	7d00237	um01063	3.00E-97	um01063	7d00237	2.00E-98
BBH	7c00170	um01181	1.00E-179	um01181	7c00170	0
BBH	7d00136	um10225	3.00E-49	um10225	7d00136	6.00E-43
BBH	7c00077	um02268	1.00E-141	um02268	7c00077	1.00E-144
BBH	7d00198	um01149	0	um01149	7d00198	0
BBH	7d00195	um01152	0	um01152	7d00195	0
BBH	7d00210	um01120	0	um01120	7d00210	0
BBH	7c00165	um01191	0	um01191	7c00165	0
BBH	7d00287	um00966	0	um00966	7d00287	0
BBH	7d00324	um00891	1.00E-174	um00891	7d00324	0
BBH	7c00282	um12122	3.00E-42	um12122	7c00282	2.00E-48
BBH	7c00097	um02303	7.00E-79	um02303	7c00097	8.00E-82
BBH	7d00141	um11866	1.00E-173	um11866	7d00141	0
BBH	7d00131	um02371	1.00E-171	um02371	7d00131	1.00E-178
BBH	7d00035	um05861	0	um05861	7d00035	0
BBH	7d00147	um15035	4.00E-96	um15035	7d00147	1.00E-104
BBH	7d00354	um12113	6.00E-42	um12113	7d00354	7.00E-42
NNN	7c00302	um10615	8.00E-87			
BBH	7d00188	um01169	0	um01169	7d00188	0
BBH	7d00125	um02398	0	um02398	7d00125	0
BBH	7c00156	um10594	0	um10594	7c00156	0
BBH	7c00163	um01197	0	um01197	7c00163	0
BBH	7c00231	um01072	1.00E-160	um01072	7c00231	1.00E-154
BBH	7c00212	um11282.2	0	um11282.2	7c00212	0

BBH	7d00265	um10793	1.00E-59	um10793	7d00265	1.00E-59
BBH	7c00149	um01243	7.00E-94	um01243	7c00149	8.00E-94
NNN	7c00291	um00940	7.00E-23			
BBH	7d00018	um05832	6.00E-37	um05832	7d00018	9.00E-40
BBH	7d00038	um11024	3.00E-23	um11024	7d00038	1.00E-23
NNN	7c00176	um01175	0			
BBH	7c00138	um10141	5.00E-85	um10141	7c00138	5.00E-89
BBH	7c00298	um10613	1.00E-163	um10613	7c00298	1.00E-162
BBH	7c00030	um05870	1.00E-139	um05870	7c00030	1.00E-122
BBH	7d00332	um11841	1.00E-110	um11841	7d00332	1.00E-104
BBH	7d00164	um01210	1.00E-77	um01210	7d00164	1.00E-73
BBH	7c00239	um15057	0	um15057	7c00239	0
BBH	7d00004	um05805.2	1.00E-134	um05805.2	7d00004	1.00E-135
BBH	7c00314	um10621	7.00E-57	um10621	7c00314	7.00E-57
BBH	7d00296	um00952	1.00E-109	um00952	7d00296	1.00E-107
BBH	7d00176	um01190	0	um01190	7d00176	0
BBH	7d00156	um01220.2	0	um01220.2	7d00156	0
BBH	7d00207	um01130	1.00E-126	um01130	7d00207	1.00E-125
BBH	7d00074	um11398	0	um11398	7d00074	0
BBH	7c00225	um01081	1.00E-118	um01081	7c00225	1.00E-118
BBH	7c00134	um02397	1.00E-107	um02397	7c00134	1.00E-134
BBH	7c00142	um01264	0	um01264	7c00142	0
BBH	7d00172	um01196	0	um01196	7d00172	0
BBH	7d00177	um11302	4.00E-80	um11302	7d00177	5.00E-76
BBH	7d00162	um01213	6.00E-36	um01213	7d00162	8.00E-39
BBH	7d00169	um01204	2.00E-49	um01204	7d00169	1.00E-50
BBH	7d00230	um01084	0	um01084	7d00230	0
BBH	7d00285	um15034	0	um15034	7d00285	0
BBH	7c00105	um11707	0	um11707	7c00105	0
BBH	7d00254	um01054	0	um01054	7d00254	0
BBH	7d00158	um01224	2.00E-56	um01224	7d00158	5.00E-55
BBH	7d00325	um00890	1.00E-129	um00890	7d00325	1.00E-130
BBH	7d00338	um11263	1.00E-155	um11263	7d00338	1.00E-160
BBH	7c00272	um10792	1.00E-128	um10792	7c00272	1.00E-128
BBH	7d00143	um01257	1.00E-158	um01257	7d00143	1.00E-158
BBH	7c00311	um00931	2.00E-45	um00931	7c00311	6.00E-50
NNN	7d00047	um05897	0			
NNN	7c00178	um01162	4.00E-31	um01162	7c00179	0
BBH	7d00201	um01141	0	um01141	7d00201	0
BBH	7c00003	um05802	0	um05802	7c00003	0
BBH	7c00268	um01001	0	um01001	7c00268	0
BBH	7c00017	um05829	2.00E-42	um05829	7c00017	9.00E-43
BBH	7d00208	um01129.2	0	um01129.2	7d00208	0
BBH	7c00113	um02341	0	um02341	7c00113	0
BBH	7c00209	um01113	1.00E-118	um01113	7c00209	1.00E-118
BBH	7d00244	um11266	0	um11266	7d00244	0
BBH	7c00048	um05906	0	um05906	7c00048	0
BBH	7d00297	um12121	2.00E-54	um12121	7d00297	9.00E-53
BBH	7c00155	um01221	0	um01221	7c00155	0
BBH	7c00078	um11399	0	um11399	7c00078	0
BBH	7d00065	um02246	1.00E-131	um02246	7d00065	1.00E-129
BBH	7c00317	um00923	6.00E-94	um00923	7c00317	1.00E-123
BBH	7d00093	um11918	4.00E-67	um11918	7d00093	1.00E-121
BBH	7d00027	um05849	0	um05849	7d00027	0
BBH	7c00060	um10080	1.00E-128	um10080	7c00060	1.00E-134
BBH	7c00094	um02311	1.00E-107	um02311	7c00094	2.00E-95
BBH	7d00197	um11648	1.00E-82	um11648	7d00197	9.00E-82
BBH	7c00171	um01180	0	um01180	7c00171	0
BBH	7c00253	um01023	1.00E-147	um01023	7c00253	1.00E-147
BBH	7d00060	um11800	0	um11800	7d00060	0
BBH	7d00057	um11798	1.00E-127	um11798	7d00057	1.00E-122
BBH	7d00212	um11858	1.00E-177	um11858	7d00212	1.00E-177

BBH	7c00285	um00958	0	um00958	7c00285	0
BBH	7c00247	um01051	0	um01051	7c00247	0
BBH	7c00200	um11642	0	um11642	7c00200	0
BBH	7d00117	um02361	3.00E-27	um02361	7d00117	3.00E-27
BBH	7d00115	um10128	9.00E-39	um10128	7d00115	3.00E-38
BBH	7c00021	um11195	0	um11195	7c00021	0
BBH	7d00003	um05803	1.00E-179	um05803	7d00003	0
BBH	7c00230	um01074	1.00E-115	um01074	7c00230	1.00E-142
BBH	7d00061	um11801	9.00E-14	um11801	7d00061	6.00E-14
BBH	7c00093	um02314	4.00E-37	um02314	7c00093	4.00E-33
BBH	7c00246	um11270	0	um11270	7c00246	0
BBH	7c00303	um00906.2	2.00E-45	um00906.2	7c00303	3.00E-45
BBH	7d00126	um02395	0	um02395	7d00126	0
BBH	7d00187	um01167	1.00E-148	um01167	7d00187	1.00E-151
BBH	7c00214	um01103	4.00E-55	um01103	7c00214	1.00E-59
BBH	7c00166	um01189	2.00E-73	um01189	7c00166	4.00E-72
BBH	7c00238	um11851	4.00E-70	um11851	7c00238	2.00E-70
NNN	7c00213	um01105	0			
BBH	7d00211	um15060	7.00E-84	um15060	7d00211	6.00E-90
BBH	7d00316	um12117	1.00E-25	um12117	7d00316	3.00E-21
BBH	7c00254	um01021	6.00E-45	um01021	7c00254	4.00E-46
BBH	7d00340	um00867	3.00E-44	um00867	7d00340	1.00E-44
BBH	7d00142	um01258	0	um01258	7d00142	0
BBH	7c00127	um10134	9.00E-93	um10134	7c00127	4.00E-98
BBH	7d00019	um05834	0	um05834	7d00019	0
BBH	7d00083	um02289	0	um02289	7d00083	0
BBH	7d00034	um05860	1.00E-132	um05860	7d00034	1.00E-132
BBH	7c00192	um11645	3.00E-57	um11645	7c00192	3.00E-57
BBH	7c00320	um00937	8.00E-49	um00937	7c00320	8.00E-44
BBH	7c00183	um01168	0	um01168	7c00183	0
BBH	7d00175	um01192	0	um01192	7d00175	0
NNN	7d00155	um01219	3.00E-33			
BBH	7d00206	um01131	0	um01131	7d00206	0
BBH	7c00338	um11260	0	um11260	7c00338	0
BBH	7c00333	um10565	7.00E-70	um10565	7c00333	2.00E-69
BBH	7d00071	um02263	8.00E-71	um02263	7d00071	1.00E-70
BBH	7c00135	um02396	1.00E-89	um02396	7c00135	4.00E-80
BBH	7d00273	um00986	1.00E-101	um00986	7d00273	1.00E-100
BBH	7d00171	um01198	4.00E-98	um01198	7d00171	3.00E-97
BBH	7c00143	um11867	0	um11867	7c00143	0
BBH	7c00310	um00916	1.00E-139	um00916	7c00310	1.00E-155
BBH	7d00078	um11400	1.00E-165	um11400	7d00078	1.00E-165
NNN	7c00343	um11256	0			
BBH	7c00118	um10127	2.00E-74	um10127	7c00118	3.00E-74
BBH	7d00238	um01067	0	um01067	7d00238	0
BBH	7c00052	um11799	8.00E-98	um11799	7c00052	1.00E-101
BBH	7d00079	um02280	0	um02280	7d00079	0
NNN	7c00047	um11038	1.00E-101			
BBH	7c00125	um02365	1.00E-143	um02365	7c00125	1.00E-161
BBH	7d00040	um11028	1.00E-175	um11028	7d00040	1.00E-177
BBH	7d00284	um12123	1.00E-160	um12123	7d00284	1.00E-146
BBH	7d00058	um05915	1.00E-172	um05915	7d00058	0
BBH	7c00139	um10138	1.00E-144	um10138	7c00139	1.00E-151
BBH	7c00058	um05922	0	um05922	7c00058	0
BBH	7d00200	um01144	1.00E-108	um01144	7d00200	1.00E-111
BBH	7d00275	um00980	2.00E-80	um00980	7d00275	5.00E-82
BBH	7d00007	um05809	0	um05809	7d00007	0
BBH	7c00141	um01266	1.00E-140	um01266	7c00141	1.00E-157
BBH	7d00298	um00950	0	um00950	7d00298	0
BBH	7d00077	um02275	0	um02275	7d00077	0
BBH	7c00341	um11258	2.00E-24	um11258	7c00341	1.00E-25
NNN	7c00332	um00876	0			

BBH	7d00257	um01022	5.00E-65	um01022	7d00257	6.00E-67
BBH	7c00065	um02247	0	um02247	7c00065	0
BBH	7c00124	um10130	0	um10130	7c00124	0
BBH	7d00037	um11022	1.00E-168	um11022	7d00037	1.00E-158
BBH	7c00237	um11853	0	um11853	7c00237	0
NNN	7c00284	um10589	0			
BBH	7c00096	um11919	0	um11919	7c00096	0
BBH	7c00086	um11402	4.00E-91	um11402	7c00086	4.00E-86
NNN	7d00072	um02264	1.00E-14			
BBH	7c00039	um05890	1.00E-88	um05890	7c00039	4.00E-88
BBH	7c00159	um01211	1.00E-135	um01211	7c00159	1.00E-132
BBH	7c00337	um11261	2.00E-71	um11261	7c00337	1.00E-71
BBH	7d00266	um10791	1.00E-154	um10791	7d00266	1.00E-142
BBH	7d00179	um01185	1.00E-35	um01185	7d00179	6.00E-45
BBH	7d00012	um05820	0	um05820	7d00012	0
BBH	7d00289	um10588	0	um10588	7d00289	0
BBH	7c00179	um01162	0	um01162	7c00179	0
BBH	7c00126	um02367	1.00E-171	um02367	7c00126	0
BBH	7c00057	um05921	0	um05921	7c00057	0
BBH	7c00029	um11025	0	um11025	7c00029	0
NNN	7c00201	um01128	1.00E-116			
BBH	7d00345	um00859	5.00E-49	um00859	7d00345	3.00E-59
BBH	7d00166	um01206	0	um01206	7d00166	0
BBH	7d00186	um01165	0	um01165	7d00186	0
BBH	7d00245	um11268	0	um11268	7d00245	0
BBH	7c00161	um01207	1.00E-131	um01207	7c00161	1.00E-116
BBH	7d00157	um01222	0	um01222	7d00157	0
BBH	7c00293	um12118	8.00E-18	um12118	7c00293	2.00E-25
BBH	7c00215	um01102	0	um01102	7c00215	0
BBH	7c00252	um01049	0	um01049	7c00252	0
BBH	7c00241	um11265	0	um11265	7c00241	0
BBH	7d00127	um10143	1.00E-116	um10143	7d00127	1.00E-116
BBH	7d00329	um00884	0	um00884	7d00329	0
BBH	7c00024	um11019	1.00E-103	um11019	7c00024	1.00E-105
BBH	7d00246	um11269	8.00E-94	um11269	7d00246	1.00E-90
BBH	7c00264	um01008	0	um01008	7c00264	0
BBH	7d00135	um01269	0	um01269	7d00135	0
BBH	7d00219	um01107	0	um01107	7d00219	0
BBH	7d00313	um00912	1.00E-136	um00912	7d00313	1.00E-138
BBH	7c00182	um01166	0	um01166	7c00182	0
BBH	7c00274	um00987	0	um00987	7c00274	0
BBH	7d00119	um02366	1.00E-26	um02366	7d00119	1.00E-34
BBH	7d00106	um02338	1.00E-108	um02338	7d00106	1.00E-102
BBH	7d00041	um05876	1.00E-132	um05876	7d00041	1.00E-163
BBH	7c00034	um05879	0	um05879	7c00034	0
BBH	7d00150	um01241	2.00E-20	um01241	7d00150	8.00E-24
BBH	7c00223	um01085	0	um01085	7c00223	0
BBH	7d00239	um01025	1.00E-144	um01025	7d00239	1.00E-147
BBH	7d00084	um02291	0	um02291	7d00084	0
BBH	7c00354	um01439	0	um01439	7c00354	0
BBH	7c00012	um05821	2.00E-92	um05821	7c00012	4.00E-93
BBH	7d00190	um01159	1.00E-122	um01159	7d00190	1.00E-129
BBH	7c00085	um02281	0	um02281	7c00085	0
BBH	7c00009	um05812	0	um05812	7c00009	0
BBH	7c00095	um02310	4.00E-48	um02310	7c00095	3.00E-46
BBH	7c00006	um05807	0	um05807	7c00006	0
BBH	7c00202	um12127	1.00E-45	um12127	7c00202	7.00E-41
BBH	7d00330	um00883	1.00E-152	um00883	7d00330	1.00E-144
BBH	7c00129	um10145	0	um10145	7c00129	1.00E-160
BBH	7d00129	um02390	0	um02390	7d00129	0
BBH	7d00301	um00946	1.00E-141	um00946	7d00301	1.00E-149
BBH	7d00224	um11278	1.00E-104	um11278	7d00224	1.00E-104

BBH	7c00119	um02354	4.00E-58	um02354	7c00119	3.00E-58
BBH	7d00335	um00880	0	um00880	7d00335	0
BBH	7d00089	um12185	0	um12185	7d00089	0
BBH	7c00145	um11865	3.00E-91	um11865	7c00145	3.00E-95
BBH	7d00036	um05863	2.00E-38	um05863	7d00036	2.00E-38
BBH	7c00342	um11257.2	2.00E-68	um11257.2	7c00342	2.00E-68
BBH	7c00331	um00878	6.00E-55	um00878	7c00331	1.00E-50
BBH	7c00087	um02284	0	um02284	7c00087	0
BBH	7c00224	um01083	0	um01083	7c00224	0
BBH	7c00322	um15053	0	um15053	7c00322	0
BBH	7d00256	um11275	0	um11275	7d00256	0
BBH	7d00185	um01164	3.00E-12	um01164	7d00185	4.00E-12
BBH	7c00295	um00894	0	um00894	7c00295	0
BBH	7d00233	um01077.2	1.00E-107	um01077.2	7d00233	1.00E-116
BBH	7d00178	um01187	1.00E-105	um01187	7d00178	1.00E-107
BBH	7c00079	um02273	1.00E-92	um02273	7c00079	1.00E-102
BBH	7c00251	um01050	0	um01050	7c00251	0
BBH	7c00059	um10079	0	um10079	7c00059	0
BBH	7c00336	um00869	1.00E-116	um00869	7c00336	1.00E-115
BBH	7d00068	um02255	0	um02255	7d00068	0
BBH	7c00140	um01267	1.00E-146	um01267	7c00140	1.00E-136
BBH	7d00359	um05248	0	um05248	7d00359	0
BBH	7c00050	um15085	0	um15085	7c00050	0
BBH	7d00042	um05878	1.00E-53	um05878	7d00042	3.00E-46
BBH	7d00006	um05806	0	um05806	7d00006	0
NNN	7d00096	um02318.2	0			
BBH	7d00274	um00985	5.00E-86	um00985	7d00274	6.00E-84
BBH	7c00249	um01059	1.00E-148	um01059	7c00249	1.00E-149
BBH	7d00221	um01100	1.00E-24	um01100	7d00221	1.00E-24
NNN	7c00103	um02327	1.00E-168			
BBH	7c00193	um01146	1.00E-128	um01146	7c00193	1.00E-128
BBH	7d00020	um05835	0	um05835	7d00020	0
BBH	7d00322	um00935	0	um00935	7d00322	0
BBH	7c00162	um11304	1.00E-174	um11304	7c00162	1.00E-164
BBH	7c00064	um02245	1.00E-133	um02245	7c00064	1.00E-130
BBH	7c00216	um01099	1.00E-153	um01099	7c00216	1.00E-154
BBH	7c00240	um01031	4.00E-86	um01031	7c00240	3.00E-91
BBH	7c00071	um02256	0	um02256	7c00071	0
BBH	7d00128	um02392	1.00E-159	um02392	7d00128	1.00E-167
NNN	7d00352	um10068	1.00E-71	um10068	6d00063	0
BBH	7d00288	um00965	0	um00965	7d00288	0
BBH	7d00203	um01134	0	um01134	7d00203	0
BBH	7d00070	um02262	0	um02262	7d00070	0
BBH	7d00323	um00936	0	um00936	7d00323	0
BBH	7c00046	um05900	0	um05900	7c00046	0
BBH	7c00130	um02404	1.00E-134	um02404	7c00130	1.00E-133
BBH	7d00013	um05822	0	um05822	7d00013	0
BBH	7c00092	um02300	0	um02300	7c00092	0
BBH	7d00110	um02343	0	um02343	7d00110	0
BBH	7d00033	um05858	0	um05858	7d00033	0
BBH	7c00158	um01229	0	um01229	7c00158	0
BBH	7d00346	um00857	0	um00857	7d00346	0
BBH	7d00299	um00949	0	um00949	7d00299	0
NNN	7d00102	um11703.2	0			
BBH	7c00033	um11027	1.00E-109	um11027	7c00033	1.00E-109
BBH	7d00347	um00855	0	um00855	7d00347	0
BBH	7c00157	um10595	0	um10595	7c00157	0
BBH	7d00314	um00921	0	um00921	7d00314	0
BBH	7c00265	um01006	4.00E-55	um01006	7c00265	5.00E-62
BBH	7d00220	um01104	1.00E-114	um01104	7d00220	1.00E-114
BBH	7d00163	um01212	0	um01212	7d00163	0
BBH	7c00104	um11706	5.00E-27	um11706	7c00104	1.00E-26

BBH	7d00029	um05853	0	um05853	7d00029	0
BBH	7c00355	um00837	0	um00837	7c00355	0
BBH	7c00072	um02258	0	um02258	7c00072	0
BBH	7c00116	um10126	4.00E-91	um10126	7c00116	3.00E-91
BBH	7d00149	um01242	0	um01242	7d00149	0
BBH	7c00023	um05847	0	um05847	7c00023	0
BBH	7c00172	um01179	8.00E-48	um01179	7c00172	4.00E-63
BBH	7d00105	um02336	3.00E-80	um02336	7d00105	3.00E-89
BBH	7c00362	um10545	1.00E-131	um10545	7c00362	1.00E-132
BBH	7d00353	um11247	2.00E-70	um11247	7d00353	2.00E-65
BBH	7d00134	um02412	3.00E-36	um02412	7d00134	9.00E-35
BBH	7d00005	um05804	0	um05804	7d00005	0
BBH	7c00305	um00909	0	um00909	7c00305	0
BBH	7d00095	um02316	1.00E-155	um02316	7d00095	1.00E-150
BBH	7d00336	um10569	0	um10569	7d00336	0
BBH	7c00128	um10136	0	um10136	7c00128	0
BBH	7d00304	um10623	0	um10623	7d00304	0
BBH	7d00290	um00964	0	um00964	7d00290	0
BBH	7d00307	um00897	0	um00897	7d00307	0
BBH	7c00236	um01027	1.00E-151	um01027	7c00236	1.00E-149
BBH	7d00160	um01228	0	um01228	7d00160	0
BBH	7d00081	um02283	3.00E-66	um02283	7d00081	4.00E-54
BBH	7c00040	um05892	1.00E-146	um05892	7c00040	1.00E-131
BBH	7c00164	um11303	0	um11303	7c00164	0
BBH	7c00315	um00929	1.00E-99	um00929	7c00315	2.00E-99
BBH	7d00232	um01079	0	um01079	7d00232	0
BBH	7c00307	um10617	5.00E-41	um10617	7c00307	1.00E-37
BBH	7d00268	um00997	4.00E-94	um00997	7d00268	4.00E-88
BBH	7d00343	um00862	1.00E-52	um00862	7d00343	2.00E-52
BBH	7c00287	um00957	0	um00957	7c00287	0
BBH	7d00270	um00991	1.00E-152	um00991	7d00270	1.00E-149
BBH	7d00082	um02285	0	um02285	7d00082	0
BBH	7d00021	um05836	1.00E-104	um05836	7d00021	1.00E-104
BBH	7c00319	um00934	0	um00934	7c00319	0
BBH	7c00088	um11403	1.00E-110	um11403	7c00088	1.00E-119
BBH	7c00243	um01040	0	um01040	7c00243	0
BBH	7c00090	um02290	1.00E-44	um02290	7c00090	2.00E-47
BBH	7d00291	um00963	0	um00963	7d00291	0
BBH	7d00321	um10622	1.00E-138	um10622	7d00321	1.00E-125
BBH	7d00261	um01011	1.00E-162	um01011	7d00261	1.00E-167
BBH	7c00217	um11279	2.00E-40	um11279	7c00217	4.00E-35
BBH	7d00226	um01091	0	um01091	7d00226	0
BBH	7c00361	um12300	0	um12300	7c00361	0
BBH	7d00090	um02308	3.00E-32	um02308	7d00090	1.00E-34
BBH	7c00190	um01153	0	um01153	7c00190	0
BBH	7d00075	um02271	1.00E-158	um02271	7d00075	1.00E-171
BBH	7c00218	um01096	7.00E-42	um01096	7c00218	3.00E-43
BBH	7d00024	um05841	0	um05841	7d00024	0
BBH	7d00121	um10135	0	um10135	7d00121	0
NNN	7d00014	um02135	8.00E-25	um02135	6d00088	1.00E-30
BBH	7c00197	um11644	0	um11644	7c00197	0
BBH	7c00169	um01182	1.00E-109	um01182	7c00169	1.00E-112
NNN	7d00202	um01138	0			
BBH	7d00032	um05857	0	um05857	7d00032	0
BBH	7c00327	um00885	1.00E-41	um00885	7c00327	1.00E-55
BBH	7c00235	um01026	1.00E-168	um01026	7c00235	1.00E-159
NNN	7d00277	um00981	2.00E-24	um00981	7d00276	2.00E-86
BBH	7c00044	um05896	6.00E-98	um05896	7c00044	1.00E-118
BBH	7d00055	um05912	1.00E-178	um05912	7d00055	1.00E-179
BBH	7c00131	um02403.2	0	um02403.2	7c00131	0
BBH	7c00117	um02351	1.00E-153	um02351	7c00117	1.00E-146
BBH	7c00357	um00835.2	0	um00835.2	7c00357	0

BBH	7c00348	um00844	1.00E-152	um00844	7c00348	1.00E-151
NNN	7d00087	um02297	4.00E-11	um02297	7c00110	1.00E-15
BBH	7d00281	um15055	0	um15055	7d00281	0
BBH	7c00152	um01230	0	um01230	7c00152	0
BBH	7d00010	um05818	0	um05818	7d00010	0
BBH	7d00152	um01233	0	um01233	7d00152	0
BBH	7d00168	um01205	0	um01205	7d00168	0
BBH	7d00101	um02326	0	um02326	7d00101	0
BBH	7c00146	um01249	1.00E-43	um01249	7c00146	2.00E-41
BBH	7c00346	um00848.2	0	um00848.2	7c00346	0
BBH	7d00333	um11839.2	1.00E-74	um11839.2	7d00333	1.00E-94
BBH	7d00234	um01075	0	um01075	7d00234	0
BBH	7c00091	um02292	0	um02292	7c00091	0
BBH	7c00294	um00893	1.00E-120	um00893	7c00294	1.00E-130
BBH	7c00108	um02333	1.00E-24	um02333	7c00108	4.00E-23
BBH	7c00109	um02298	1.00E-12	um02298	7c00109	1.00E-12
BBH	7c00330	um00879	6.00E-99	um00879	7c00330	3.00E-97
BBH	7d00259	um01016	2.00E-53	um01016	7d00259	7.00E-50
BBH	7c00014	um11293	0	um11293	7c00014	0
BBH	7c00101	um02323	1.00E-104	um02323	7c00101	1.00E-102
BBH	7c00073	um02260	0	um02260	7c00073	0
BBH	7d00223	um11280	0	um11280	7d00223	0
BBH	7d00159	um01227	1.00E-165	um01227	7d00159	1.00E-157
BBH	7c00220	um01092	5.00E-89	um01092	7c00220	4.00E-97
BBH	7d00182	um01174	0	um01174	7d00182	0
BBH	7d00192	um11650	0	um11650	7d00192	0
BBH	7c00049	um05911	0	um05911	7c00049	0
BBH	7c00204	um01126	6.00E-62	um01126	7c00204	6.00E-62
BBH	7d00120	um10133	1.00E-133	um10133	7d00120	1.00E-135
BBH	7c00271	um00996.2	0	um00996.2	7c00271	0
BBH	7d00251	um01058	3.00E-96	um01058	7d00251	3.00E-96
BBH	7d00088	um15079	1.00E-28	um15079	7d00088	4.00E-35
BBH	7d00303	um10624.2	0	um10624.2	7d00303	0
BBH	7c00356	um00833	0	um00833	7c00356	0
BBH	7c00019	um05833	0	um05833	7c00019	1.00E-173
BBH	7c00250	um01060	5.00E-73	um01060	7c00250	3.00E-73
BBH	7d00069	um15078	0	um15078	7d00069	0
BBH	7c00151	um01231	1.00E-114	um01231	7c00151	1.00E-130
BBH	7d00043	um05888	1.00E-109	um05888	7d00043	1.00E-117
BBH	7c00281	um00967	4.00E-20	um00967	7c00281	7.00E-27
BBH	7d00112	um02346	0	um02346	7d00112	0
BBH	7c00276	um00978	1.00E-138	um00978	7c00276	1.00E-133
BBH	7d00327	um10647	1.00E-144	um10647	7d00327	1.00E-140
BBH	7d00199	um15050	0	um15050	7d00199	0
BBH	7c00032	um05873	0	um05873	7c00032	0
BBH	7d00319	um00927	1.00E-104	um00927	7d00319	4.00E-94
BBH	7d00098	um02320	2.00E-28	um02320	7d00098	3.00E-28
BBH	7c00266	um01005	0	um01005	7c00266	0
BBH	7d00357	um00832	1.00E-161	um00832	7d00357	0
BBH	7c00306	um00911	0	um00911	7c00306	0
BBH	7d00051	um11041.2	0	um11041.2	7d00051	0
BBH	7c00189	um01154	0	um01154	7c00189	0
BBH	7c00180	um01163	1.00E-112	um01163	7c00180	1.00E-126
BBH	7d00217	um01110	0	um01110	7d00217	0
BBH	7d00311	um00908	2.00E-41	um00908	7d00311	3.00E-50
BBH	7c00258	um10797	0	um10797	7c00258	0
BBH	7d00260	um01013	2.00E-31	um01013	7d00260	9.00E-29
BBH	7d00022	um11196	0	um11196	7d00022	0
BBH	7c00286	um00956	1.00E-162	um00956	7c00286	1.00E-168
NNN	7d00358	um11243	7.00E-60			
BBH	7c00066	um10081	1.00E-71	um10081	7c00066	5.00E-66
BBH	7d00025	um05846	0	um05846	7d00025	0

BBH	7d00122	um02405	1.00E-127	um02405	7d00122	1.00E-135
BBH	7c00196	um01142	1.00E-102	um01142	7c00196	1.00E-109
BBH	7c00031	um05872	3.00E-55	um05872	7c00031	4.00E-61
BBH	7c00080	um02274	9.00E-69	um02274	7c00080	1.00E-70
BBH	7c00242	um11267	0	um11267	7c00242	0
BBH	7c00219	um01093	0	um01093	7c00219	0
NNN	7c00056	um11802	8.00E-31	um11802	7c00055	6.00E-40
BBH	7d00344	um00861	0	um00861	7d00344	0
BBH	7d00031	um05856.2	4.00E-41	um05856.2	7d00031	7.00E-41
BBH	7c00210	um01111	1.00E-127	um01111	7c00210	1.00E-145
BBH	7d00349	um11254	9.00E-27	um11254	7d00349	3.00E-26
BBH	7c00160	um01208	6.00E-74	um01208	7c00160	3.00E-76
BBH	7c00089	um11405	1.00E-34	um11405	7c00089	2.00E-32
BBH	7d00258	um15056	0	um15056	7d00258	0
BBH	7c00147	um01246	1.00E-132	um01246	7c00147	1.00E-133
BBH	7d00151	um01237	3.00E-24	um01237	7d00151	1.00E-23
BBH	7c00347	um11249	1.00E-152	um11249	7c00347	1.00E-152
BBH	7c00187	um01156	0	um01156	7c00187	0
BBH	7d00339	um11262	1.00E-24	um11262	7d00339	4.00E-25
BBH	7c00324	um00887	0	um00887	7c00324	0
BBH	7d00235	um01073	4.00E-41	um01073	7d00235	6.00E-40
BBH	7c00334	um00872	0	um00872	7c00334	0
BBH	7d00350	um00847	5.00E-91	um00847	7d00350	1.00E-97
BBH	7c00349	um11246	0	um11246	7c00349	0
NNN	7c00043	um05896	2.00E-13	um05896	7c00044	1.00E-118
BBH	7c00195	um01143	1.00E-170	um01143	7c00195	1.00E-172
BBH	7c00234	um11274	3.00E-21	um11274	7c00234	3.00E-21
BBH	7d00276	um00981	1.00E-90	um00981	7d00276	2.00E-86
NNN	7c00358	um10544	9.00E-87	um10544	15d00088	3.00E-93
BBH	7d00054	um05910	1.00E-107	um05910	7d00054	1.00E-108
BBH	7d00280	um00983	0	um00983	7d00280	0
BBH	7c00304	um00907	1.00E-166	um00907	7c00304	1.00E-162
BBH	7c00318	um00915	0	um00915	7c00318	0
NNN	7d00267	um00995	1.00E-172			
BBH	7c00340	um12114	2.00E-45	um12114	7c00340	1.00E-62
BBH	7d00292	um00961	1.00E-147	um00961	7d00292	1.00E-143
BBH	7d00351	um11248	5.00E-74	um11248	7d00351	9.00E-80
BBH	7c00351	um00842	0	um00842	7c00351	0
BBH	7d00309	um12116.2	0	um12116.2	7d00309	0
BBH	7d00306	um00895	0	um00895	7d00306	0
BBH	7d00097	um11700.2	1.00E-151	um11700.2	7d00097	1.00E-151
BBH	7d00165	um01209	0	um01209	7d00165	0
NNN	7d00123	um02402	2.00E-53			
BBH	7d00250	um11271	0	um11271	7d00250	0
BBH	7c00335	um00871	0	um00871	7c00335	1.00E-156
BBH	7d00225	um01094	1.00E-129	um01094	7d00225	1.00E-128
BBH	7c00221	um01089	0	um01089	7c00221	0
BBH	7d00248	um01048	0	um01048	7d00248	0
NNN	7d00085	um02293	2.00E-36	um02293	12c00084	8.00E-53
BBH	7d00181	um01176.2	7.00E-56	um01176.2	7d00181	5.00E-60
BBH	7d00222	um11281	0	um11281	7d00222	0
BBH	7c00280	um10591	6.00E-83	um10591	7c00280	4.00E-83
BBH	7c00013	um05825	1.00E-179	um05825	7c00013	1.00E-170
BBH	7c00174	um01178	0	um01178	7c00174	0
BBH	7c00325	um03226	0	um03226	7c00325	0
BBH	7d00218	um11284	0	um11284	7d00218	0
BBH	7d00312	um10616	8.00E-77	um10616	7d00312	8.00E-77
BBH	7c00289	um00945	0	um00945	7c00289	0
BBH	7c00360	um05249	0	um05249	7c00360	0
BBH	7c00267	um10795	0	um10795	7c00267	0
BBH	7c00102	um02325.2	1.00E-99	um02325.2	7c00102	1.00E-100
BBH	7c00259	um01014	6.00E-53	um01014	7c00259	3.00E-47

BBH	7d00180	um11301	0	um11301	7d00180	0
BBH	7c00100	um11702	1.00E-119	um11702	7c00100	1.00E-118
BBH	7c00188	um12128	0	um12128	7c00188	0
BBH	7c00150	um01234	2.00E-64	um01234	7c00150	6.00E-58
BBH	7c00074	um12184	5.00E-48	um12184	7c00074	5.00E-46
BBH	7d00107	um02340	0	um02340	7d00107	0
BBH	7c00020	um11194	1.00E-179	um11194	7c00020	0
BBH	7d00104	um11708	2.00E-47	um11708	7d00104	1.00E-47
BBH	7d00111	um02344	0	um02344	7d00111	0
BBH	7d00328	um03227	1.00E-107	um03227	7d00328	1.00E-108
BBH	7d00215	um01114	1.00E-101	um01114	7d00215	1.00E-100
BBH	7c00277	um11848	2.00E-72	um11848	7c00277	2.00E-72
BBH	7c00205	um01122	0	um01122	7c00205	0
BBH	8d00053	um03522	0	um03522	8d00053	0
BBH	8d00103	um03418	0	um03418	8d00103	0
BBH	8d00114	um12229	1.00E-160	um12229	8d00114	1.00E-160
BBH	8d00029	um03614	0	um03614	8d00029	0
BBH	8d00063	um11090	1.00E-151	um11090	8d00063	1.00E-161
BBH	8d00093	um03450	1.00E-59	um03450	8d00093	2.00E-57
BBH	8c00079	um03485	4.00E-88	um03485	8c00079	4.00E-88
BBH	8c00020	um03596	1.00E-155	um03596	8c00020	1.00E-154
BBH	8d00047	um03541	2.00E-77	um03541	8d00047	3.00E-79
BBH	8c00106	um03435	0	um03435	8c00106	0
BBH	8c00011	um12234	3.00E-46	um12234	8c00011	4.00E-45
BBH	8c00001	um03558	0	um03558	8c00001	0
BBH	8c00091	um03461	1.00E-35	um03461	8c00091	1.00E-36
BBH	8d00054	um12233	1.00E-123	um12233	8d00054	1.00E-128
BBH	8c00064	um03518	0	um03518	8c00064	0
BBH	8c00028	um03608	2.00E-86	um03608	8c00028	1.00E-101
BBH	8c00047	um03540	3.00E-61	um03540	8c00047	6.00E-61
BBH	8c00038	um03556	8.00E-40	um03556	8c00038	4.00E-36
BBH	8c00021	um03598	1.00E-157	um03598	8c00021	1.00E-144
BBH	8d00077	um03478	1.00E-147	um03478	8d00077	1.00E-147
BBH	8c00055	um03530	1.00E-39	um03530	8c00055	3.00E-35
BBH	8d00007	um03570	1.00E-83	um03570	8d00007	2.00E-76
BBH	8d00106	um03422	1.00E-105	um03422	8d00106	1.00E-111
BBH	8d00021	um03605	0	um03605	8d00021	0
BBH	8c00108	um03431	0	um03431	8c00108	0
BBH	8c00012	um10574	0	um10574	8c00012	0
BBH	8d00104	um03419	1.00E-107	um03419	8d00104	1.00E-108
BBH	8d00109	um11070	1.00E-173	um11070	8d00109	1.00E-172
BBH	8c00105	um10438	0	um10438	8c00105	0
BBH	8d00039	um03632	0	um03632	8d00039	0
BBH	8d00028	um03613	0	um03613	8d00028	0
BBH	8d00064	um03496	1.00E-63	um03496	8d00064	2.00E-52
BBH	8c00078	um03491	5.00E-66	um03491	8c00078	1.00E-54
BBH	8c00118	um03411	1.00E-126	um03411	8c00118	1.00E-130
BBH	8c00052	um11100	1.00E-176	um11100	8c00052	1.00E-171
BBH	8d00051	um03528	0	um03528	8d00051	0
BBH	8c00081	um11084.2	0	um11084.2	8c00081	0
BBH	8d00115	um03407	0	um03407	8d00115	0
BBH	8c00065	um03515	0	um03515	8c00065	0
BBH	8c00090	um03464	1.00E-111	um03464	8c00090	1.00E-114
BBH	8d00020	um03602	1.00E-109	um03602	8d00020	1.00E-111
BBH	8d00055	um03514	0	um03514	8d00055	0
BBH	8c00088	um03467	2.00E-71	um03467	8c00088	3.00E-73
BBH	8d00040	um03557	0	um03557	8d00040	0
BBH	8c00033	um03621	1.00E-122	um03621	8c00033	0
BBH	8c00107	um03433	4.00E-62	um03433	8c00107	2.00E-60
BBH	8c00002	um03560	0	um03560	8c00002	0
BBH	8d00078	um03475	0	um03475	8d00078	0
BBH	8d00001	um03559	0	um03559	8d00001	0

BBH	8c00044	um03544	1.00E-153	um03544	8c00044	1.00E-154
BBH	8d00006	um03568	0	um03568	8d00006	0
BBH	8c00127	um03406	1.00E-42	um03406	8c00127	1.00E-44
BBH	8c00077	um03493	7.00E-89	um03493	8c00077	7.00E-82
BBH	8d00086	um03463	0	um03463	8d00086	0
BBH	8c00100	um03448	0	um03448	8c00100	0
BBH	8d00070	um03486	1.00E-135	um03486	8d00070	1.00E-129
BBH	8c00013	um10577	2.00E-87	um10577	8c00013	6.00E-89
BBH	8d00036	um12237	7.00E-30	um12237	8d00036	7.00E-30
BBH	8d00116	um03409	0	um03409	8d00116	0
BBH	8d00101	um11077	0	um11077	8d00101	0
BBH	8d00068	um11086	0	um11086	8d00068	1.00E-180
BBH	8c00117	um11074	0	um11074	8c00117	0
BBH	8d00035	um03624	0	um03624	8d00035	0
BBH	8c00110	um11076	1.00E-117	um11076	8c00110	1.00E-129
BBH	8c00082	um03480	9.00E-99	um03480	8c00082	3.00E-98
BBH	8c00066	um03513	1.00E-101	um03513	8c00066	1.00E-108
BBH	8c00093	um12231	1.00E-26	um12231	8c00093	1.00E-33
NNN	8c00068	um11782	1.00E-66			
BBH	8c00004	um03564	3.00E-35	um03564	8c00004	1.00E-39
BBH	8c00003	um03563	1.00E-107	um03563	8c00003	1.00E-107
BBH	8c00122	um03397	3.00E-27	um03397	8c00122	3.00E-27
BBH	8d00052	um11097	4.00E-15	um11097	8d00052	1.00E-15
BBH	8c00023	um03601	5.00E-25	um03601	8c00023	2.00E-24
BBH	8d00018	um03593	1.00E-149	um03593	8d00018	1.00E-146
BBH	8c00032	um03620	0	um03620	8c00032	0
NNN	8d00071	um11083	1.00E-102			
BBH	8d00037	um03631	1.00E-59	um03631	8d00037	2.00E-67
BBH	8d00073	um10447	1.00E-27	um10447	8d00073	2.00E-31
BBH	8c00014	um03590	1.00E-164	um03590	8c00014	1.00E-180
BBH	8d00087	um03459	0	um03459	8d00087	0
BBH	8c00025	um11505	1.00E-43	um11505	8c00025	1.00E-43
BBH	8c00128	um03408	1.00E-179	um03408	8c00128	0
BBH	8c00076	um03494	0	um03494	8c00076	0
BBH	8d00094	um03446	0	um03446	8d00094	0
BBH	8d00046	um03542	3.00E-83	um03542	8d00046	1.00E-100
BBH	8d00102	um11075	2.00E-20	um11075	8d00102	5.00E-26
BBH	8c00085	um03474	0	um03474	8c00085	0
BBH	8c00109	um11078	1.00E-101	um11078	8c00109	1.00E-107
BBH	8c00046	um03543	1.00E-103	um03543	8c00046	1.00E-104
BBH	8c00041	um03550	1.00E-168	um03550	8c00041	1.00E-163
BBH	8c00022	um03599	0	um03599	8c00022	0
BBH	8c00102	um03445	8.00E-71	um03445	8c00102	8.00E-71
BBH	8d00066	um11088	0	um11088	8d00066	0
BBH	8c00083	um03477	0	um03477	8c00083	0
BBH	8d00013	um10575	2.00E-89	um10575	8d00013	8.00E-92
BBH	8c00054	um11099	0	um11099	8c00054	0
BBH	8c00005	um03565	0	um03565	8c00005	0
BBH	8c00092	um03460	1.00E-53	um03460	8c00092	2.00E-55
NNN	8c00067	um03511	2.00E-88			
BBH	8c00057	um11098	0	um11098	8c00057	0
BBH	8c00112	um03414	0	um03414	8c00112	0
BBH	8c00059	um03524	0	um03524	8c00059	0
BBH	8c00126	um03404	0	um03404	8c00126	0
BBH	8c00095	um03454	0	um03454	8c00095	0
BBH	8c00097	um10444	7.00E-89	um10444	8c00097	6.00E-89
BBH	8c00016	um03588	0	um03588	8c00016	0
BBH	8c00024	um03603	0	um03603	8c00024	0
BBH	8d00088	um03458	1.00E-53	um03458	8d00088	6.00E-49
BBH	8c00048	um03538	0	um03538	8c00048	0
BBH	8d00089	um03456	2.00E-38	um03456	8d00089	9.00E-43
BBH	8c00043	um03547.2	0	um03547.2	8c00043	0

BBH	8d00085	um03465	0	um03465	8d00085	0
BBH	8d00057	um11093.2	2.00E-40	um11093.2	8d00057	2.00E-35
BBH	8c00120	um05791	1.00E-143	um05791	8c00120	1.00E-152
BBH	8c00086	um03473	0	um03473	8c00086	0
BBH	8d00067	um11087	0	um11087	8d00067	0
BBH	8d00044	um03548	1.00E-163	um03548	8d00044	1.00E-163
BBH	8c00101	um10442	1.00E-113	um10442	8c00101	1.00E-109
NNN	8c00125	um03402	0			
BBH	8d00012	um03580	1.00E-158	um03580	8d00012	1.00E-158
BBH	8d00097	um03442	0	um03442	8d00097	0
BBH	8c00075	um03495	1.00E-111	um03495	8c00075	1.00E-104
BBH	8d00074	um03481	3.00E-63	um03481	8d00074	3.00E-66
BBH	8c00119	um03476	2.00E-79	um03476	8c00119	2.00E-83
BBH	8d00043	um03553	3.00E-54	um03553	8d00043	1.00E-58
BBH	8c00056	um03529	1.00E-143	um03529	8c00056	1.00E-144
BBH	8c00061	um03521	1.00E-25	um03521	8c00061	3.00E-45
BBH	8c00006	um03567	4.00E-92	um03567	8c00006	8.00E-84
BBH	8c00071	um11092	1.00E-139	um11092	8c00071	1.00E-139
BBH	8d00090	um12230.2	3.00E-13	um12230.2	8d00090	7.00E-15
BBH	8c00017	um03585	1.00E-106	um03585	8c00017	1.00E-106
BBH	8c00111	um03412	1.00E-52	um03412	8c00111	8.00E-51
NNN	8d00059	um02704	3.00E-66	um02704	5d00046	0
BBH	8c00034	um03623	0	um03623	8c00034	0
NNN	8c00096	um10444	7.00E-70	um10444	8c00097	6.00E-89
BBH	8c00094	um10445	1.00E-140	um10445	8c00094	1.00E-148
BBH	8d00002	um03561	0	um03561	8d00002	0
BBH	8c00031	um03617	0	um03617	8c00031	0
BBH	8c00037	um03629	0	um03629	8c00037	0
BBH	8d00107	um11073	0	um11073	8d00107	0
NNN	8c00040	um03551	0			
BBH	8c00027	um03607	0	um03607	8c00027	0
BBH	8c00049	um03537	0	um03537	8c00049	1.00E-176
BBH	8c00074	um03498	0	um03498	8c00074	0
BBH	8d00045	um11731	1.00E-20	um11731	8d00045	3.00E-25
BBH	8d00015	um12236	1.00E-136	um12236	8d00015	1.00E-138
NNN	8d00025	um02704	1.00E-65	um02704	5d00046	0
BBH	8c00087	um03468	1.00E-85	um03468	8c00087	5.00E-78
BBH	8d00056	um11095.2	0	um11095.2	8d00056	0
BBH	8d00069	um10448	0	um10448	8d00069	0
BBH	8d00110	um03396	0	um03396	8d00110	0
BBH	8d00019	um03597	0	um03597	8d00019	0
BBH	8d00034	um03622	1.00E-137	um03622	8d00034	1.00E-155
BBH	8c00060	um03523	0	um03523	8c00060	0
BBH	8d00096	um10440.2	0	um10440.2	8d00096	0
BBH	8c00045	um15005	0	um15005	8c00045	0
BBH	8d00010	um03576	0	um03576	8d00010	0
BBH	8d00005	um10571	1.00E-180	um10571	8d00005	0
NNN	8d00041	um03557	2.00E-77	um03557	8d00040	0
BBH	8d00023	um03609	0	um03609	8d00023	0
BBH	8c00114	um03417	0	um03417	8c00114	0
BBH	8c00008	um03572	1.00E-85	um03572	8c00008	1.00E-90
BBH	8c00116	um03424	1.00E-45	um03424	8c00116	1.00E-38
BBH	8c00007	um03569	0	um03569	8c00007	0
BBH	8c00062	um03520	0	um03520	8c00062	0
BBH	8c00072	um11091	0	um11091	8c00072	0
BBH	8c00050	um03536	1.00E-160	um03536	8c00050	1.00E-148
BBH	8c00026	um01301	3.00E-18	um01301	8c00026	4.00E-18
BBH	8c00053	um03533	8.00E-66	um03533	8c00053	8.00E-66
BBH	8c00030	um03615	0	um03615	8c00030	0
BBH	8c00036	um03628	0	um03628	8c00036	0
BBH	8d00014	um11960	1.00E-175	um11960	8d00014	0
BBH	8c00029	um03610	0	um03610	8c00029	0

BBH	8c00123	um11067	0	um11067	8c00123	0
BBH	8d00099	um03436	0	um03436	8d00099	0
BBH	8d00108	um03425	0	um03425	8d00108	0
BBH	8d00030	um03616	0	um03616	8d00030	0
BBH	8d00111	um03398	0	um03398	8d00111	0
BBH	8c00104	um10439	2.00E-60	um10439	8c00104	1.00E-60
BBH	8c00073	um11089	1.00E-148	um11089	8c00073	1.00E-146
BBH	8d00016	um11959	5.00E-38	um11959	8d00016	4.00E-38
BBH	8d00050	um03532	1.00E-145	um03532	8d00050	1.00E-140
BBH	8d00024	um03611	0	um03611	8d00024	0
BBH	8c00070	um03506	1.00E-150	um03506	8c00070	1.00E-131
BBH	8d00009	um03573	1.00E-68	um03573	8d00009	7.00E-56
BBH	8d00060	um03505.2	0	um03505.2	8d00060	0
BBH	8d00011	um10573	1.00E-92	um10573	8d00011	2.00E-96
BBH	8c00089	um03466	1.00E-128	um03466	8c00089	1.00E-137
BBH	8d00082	um03471	0	um03471	8d00082	0
BBH	8d00022	um11506	7.00E-72	um11506	8d00022	7.00E-72
BBH	8d00062	um03501	0	um03501	8d00062	0
BBH	8c00042	um03549	0	um03549	8c00042	0
BBH	8d00004	um11732	1.00E-103	um11732	8d00004	3.00E-99
BBH	8c00063	um11096	8.00E-80	um11096	8c00063	1.00E-80
BBH	8c00010	um03575	1.00E-103	um03575	8c00010	1.00E-102
BBH	8c00058	um03525	0	um03525	8c00058	0
BBH	8c00113	um03416	1.00E-134	um03416	8c00113	1.00E-134
BBH	8c00009	um03574	6.00E-59	um03574	8c00009	2.00E-63
BBH	8d00100	um03434	0	um03434	8d00100	0
BBH	8d00113	um03403.2	0	um03403.2	8d00113	0
BBH	8c00115	um03421	0	um03421	8c00115	0
NNN	8c00098	um10443.2	7.00E-32			
BBH	8c00019	um03595	1.00E-162	um03595	8c00019	1.00E-147
BBH	8d00048	um03539	1.00E-143	um03539	8d00048	1.00E-137
BBH	8c00051	um03534	0	um03534	8c00051	0
BBH	8d00112	um11068	0	um11068	8d00112	0
BBH	8c00018	um03583	1.00E-136	um03583	8c00018	1.00E-130
BBH	8c00039	um03554	1.00E-161	um03554	8c00039	1.00E-172
BBH	8d00017	um03584	0	um03584	8d00017	0
NNN	8c00103	um03443	2.00E-75			
BBH	8d00031	um03618	0	um03618	8d00031	0
BBH	8d00105	um11071	1.00E-133	um11071	8d00105	1.00E-140
BBH	8c00124	um03400	0	um03400	8c00124	0
BBH	8d00076	um10446	0	um10446	8d00076	0
BBH	8d00032	um03619	0	um03619	8d00032	0
BBH	8d00042	um03555	0	um03555	8d00042	0
NNN	8d00091	um03451	1.00E-173			
BBH	8d00083	um03470	0	um03470	8d00083	0
BBH	8d00084	um03469	1.00E-179	um03469	8d00084	0
BBH	8d00098	um03437	1.00E-179	um03437	8d00098	1.00E-172
BBH	8d00008	um03571	1.00E-118	um03571	8d00008	1.00E-119
BBH	8d00058	um03508	1.00E-152	um03508	8d00058	1.00E-147
NNN	8d00061	um03504	1.00E-160			
BBH	9c00012	um00025	0	um00025	9c00012	0
BBH	9d00070	um00130	1.00E-121	um00130	9d00070	1.00E-121
BBH	9c00224	um00397	2.00E-46	um00397	9c00224	2.00E-46
BBH	9d00355	um00708	0	um00708	9d00355	0
NNN	9d00335	um00676	0			
BBH	9c00367	um11829	0	um11829	9c00367	0
BBH	9d00194	um00405	1.00E-178	um00405	9d00194	1.00E-175
BBH	9d00228	um00469	3.00E-42	um00469	9d00228	6.00E-47
BBH	9c00177	um00317	7.00E-79	um00317	9c00177	1.00E-78
BBH	9c00341	um10928	4.00E-77	um10928	9c00341	4.00E-88
BBH	9c00030	um10011	0	um10011	9c00030	0
BBH	9c00403	um11625	2.00E-33	um11625	9c00403	2.00E-32

BBH	9d00347	um00695	0	um00695	9d00347	0
BBH	9c00375	um00685	0	um00685	9c00375	0
BBH	9c00024	um00030	0	um00030	9c00024	0
BBH	9d00207	um12151	0	um12151	9d00207	0
BBH	9d00109	um11421	0	um11421	9d00109	0
BBH	9c00393	um11174	5.00E-57	um11174	9c00393	2.00E-56
BBH	9d00256	um00528	0	um00528	9d00256	0
BBH	9d00293	um00598	1.00E-180	um00598	9d00293	1.00E-180
BBH	9c00094	um00156.2	1.00E-118	um00156.2	9c00094	1.00E-121
NNN	9d00123	um00244	3.00E-96			
NNN	9d00312	um05897	1.00E-107			
BBH	9c00141	um11429	5.00E-41	um11429	9c00141	3.00E-41
BBH	9c00184	um00328	1.00E-128	um00328	9c00184	1.00E-131
BBH	9d00379	um00748	2.00E-41	um00748	9d00379	2.00E-41
BBH	9d00340	um11619	3.00E-55	um11619	9d00340	1.00E-55
BBH	9d00183	um00364.2	0	um00364.2	9d00183	0
BBH	9c00251	um00455	1.00E-138	um00455	9c00251	1.00E-151
BBH	9d00044	um10021	1.00E-109	um10021	9d00044	1.00E-109
BBH	9d00016	um12107	0	um12107	9d00016	0
BBH	9c00226	um00407	0	um00407	9c00226	0
BBH	9d00027	um00048	3.00E-70	um00048	9d00027	2.00E-69
BBH	9d00157	um10099	1.00E-106	um10099	9d00157	1.00E-105
BBH	9d00112	um00223	1.00E-136	um00223	9d00112	1.00E-153
BBH	9d00046	um00096	1.00E-108	um00096	9d00046	1.00E-108
BBH	9c00110	um00171	2.00E-30	um00171	9c00110	1.00E-29
BBH	9d00411	um11629	4.00E-68	um11629	9d00411	1.00E-68
BBH	9d00213	um00442	1.00E-19	um00442	9d00213	6.00E-14
BBH	9c00290	um00527	1.00E-41	um00527	9c00290	1.00E-52
BBH	9c00258	um10118	2.00E-76	um10118	9c00258	7.00E-77
BBH	9c00294	um11440.2	1.00E-157	um11440.2	9c00294	1.00E-149
BBH	9c00322	um00592	0	um00592	9c00322	0
BBH	9c00338	um10926	0	um10926	9c00338	0
BBH	9c00261	um00475	0	um00475	9c00261	0
BBH	9c00415	um00767	2.00E-55	um00767	9c00415	1.00E-106
BBH	9c00163	um00290	0	um00290	9c00163	0
BBH	9c00084	um00133	1.00E-159	um00133	9c00084	1.00E-159
NNN	9d00172	um00387	6.00E-56	um00387	16c00076	9.00E-78
BBH	9d00407	um00801	0	um00801	9d00407	0
BBH	9c00361	um00656	0	um00656	9c00361	0
BBH	9c00380	um00696	0	um00696	9c00380	0
BBH	9c00301	um00543	0	um00543	9c00301	0
BBH	9d00358	um00714	0	um00714	9d00358	0
BBH	9c00432	um11630	5.00E-57	um11630	9c00432	6.00E-55
BBH	9c00314	um00575	0	um00575	9c00314	0
BBH	9d00307	um00625	0	um00625	9d00307	0
BBH	9d00233	um00480	1.00E-118	um00480	9d00233	1.00E-123
BBH	9c00218	um00361	0	um00361	9c00218	0
BBH	9c00153	um00266	2.00E-57	um00266	9c00153	8.00E-57
BBH	9d00384	um00760	0	um00760	9d00384	0
BBH	9c00389	um00716.2	8.00E-69	um00716.2	9c00389	7.00E-66
BBH	9c00305	um00555	0	um00555	9c00305	0
NNN	9c00280	um12105	2.00E-38	um12105	9c00281	2.00E-51
BBH	9c00135	um11424	1.00E-163	um11424	9c00135	1.00E-152
BBH	9c00362	um00655	0	um00655	9c00362	0
BBH	9c00103	um00180	1.00E-107	um00180	9c00103	8.00E-93
BBH	9d00166	um11344	1.00E-144	um11344	9d00166	1.00E-146
BBH	9d00263	um00540	1.00E-180	um00540	9d00263	0
BBH	9c00099	um00166	1.00E-115	um00166	9c00099	1.00E-110
BBH	9d00378	um11624.2	1.00E-19	um11624.2	9d00378	8.00E-29
BBH	9c00283	um00505	0	um00505	9c00283	0
BBH	9c00410	um00758	0	um00758	9c00410	0
BBH	9d00257	um00532	5.00E-83	um00532	9d00257	2.00E-96

BBH	9c00104	um00179	8.00E-78	um00179	9c00104	8.00E-81
BBH	9c00392	um00723	1.00E-102	um00723	9c00392	6.00E-97
BBH	9d00003	um00020	1.00E-106	um00020	9d00003	1.00E-105
BBH	9d00277	um00583	0	um00583	9d00277	0
BBH	9d00221	um10116	0	um10116	9d00221	0
BBH	9d00270	um00551	0	um00551	9d00270	0
BBH	9c00119	um00199	1.00E-170	um00199	9c00119	1.00E-157
BBH	9d00179	um00375	0	um00375	9d00179	0
BBH	9d00063	um00115	0	um00115	9d00063	0
BBH	9c00426	um00796	1.00E-109	um00796	9c00426	1.00E-108
BBH	9c00343	um00628	3.00E-20	um00628	9c00343	8.00E-24
BBH	9d00125	um00249	0	um00249	9d00125	0
BBH	9d00068	um10029	0	um10029	9d00068	0
BBH	9c00270	um00496	3.00E-17	um00496	9c00270	3.00E-21
BBH	9d00085	um00160	1.00E-135	um00160	9d00085	1.00E-135
NNN	9c00274	um00501	1.00E-66	um00501	9c00275	5.00E-95
BBH	9d00243	um11431	1.00E-27	um11431	9d00243	9.00E-33
BBH	9d00079	um00149	0	um00149	9d00079	0
BBH	9c00373	um11616	1.00E-34	um11616	9c00373	9.00E-33
BBH	9c00352	um00644.2	0	um00644.2	9c00352	0
BBH	9c00127	um00213	0	um00213	9c00127	0
BBH	9d00113	um00224	3.00E-70	um00224	9d00113	3.00E-70
BBH	9d00108	um00211	8.00E-46	um00211	9d00108	2.00E-43
BBH	9d00008	um00026	1.00E-109	um00026	9d00008	1.00E-110
BBH	9d00206	um00432	0	um00432	9d00206	1.00E-174
BBH	9d00160	um10101	8.00E-52	um10101	9d00160	9.00E-44
BBH	9d00326	um00663	0	um00663	9d00326	0
BBH	9c00142	um00242	0	um00242	9c00142	0
BBH	9c00159	um10086	0	um10086	9c00159	0
BBH	9d00239	um00492	1.00E-153	um00492	9d00239	1.00E-145
NNN	9c00093	um00154	0			
BBH	9d00118	um11427	2.00E-43	um11427	9d00118	2.00E-41
NNN	9c00096	um00161	2.00E-57			
BBH	9c00339	um00623	1.00E-101	um00623	9c00339	9.00E-99
BBH	9c00281	um12105	5.00E-52	um12105	9c00281	2.00E-51
BBH	9d00380	um00749	0	um00749	9d00380	0
BBH	9d00078	um10037	4.00E-20	um10037	9d00078	2.00E-21
BBH	9d00311	um00634	1.00E-117	um00634	9d00311	1.00E-120
BBH	9c00268	um00493	1.00E-107	um00493	9c00268	1.00E-114
BBH	9c00345	um11448	1.00E-115	um11448	9c00345	1.00E-119
BBH	9d00244	um11435	2.00E-75	um11435	9d00244	2.00E-66
BBH	9d00099	um00191	8.00E-48	um00191	9d00099	2.00E-47
BBH	9c00330	um00606	1.00E-115	um00606	9c00330	1.00E-118
BBH	9c00185	um00330	1.00E-134	um00330	9c00185	1.00E-142
BBH	9d00120	um00238	7.00E-88	um00238	9d00120	4.00E-80
BBH	9d00193	um11821	1.00E-105	um11821	9d00193	1.00E-107
BBH	9d00017	um00011	0	um00011	9d00017	0
BBH	9c00040	um00058	1.00E-135	um00058	9c00040	1.00E-141
BBH	9c00253	um00459	0	um00459	9c00253	0
NNN	9d00184	um00362	1.00E-172			
BBH	9c00376	um00688	1.00E-169	um00688	9c00376	1.00E-167
NNN	9c00223	um00395	1.00E-177			
BBH	9c00164	um00292	0	um00292	9c00164	0
BBH	9c00233	um00419	0	um00419	9c00233	0
BBH	9d00271	um10461	0	um10461	9d00271	0
BBH	9d00143	um00291	1.00E-112	um00291	9d00143	1.00E-115
BBH	9d00286	um10467	0	um10467	9d00286	0
BBH	9c00354	um00647	1.00E-165	um00647	9c00354	1.00E-170
BBH	9d00049	um00098	0	um00098	9d00049	0
BBH	9c00295	um00535	0	um00535	9c00295	0
BBH	9d00001	um06489	1.00E-112	um06489	9d00001	1.00E-116
BBH	9c00321	um10469	3.00E-95	um10469	9c00321	3.00E-95

BBH	9d00212	um00439	4.00E-24	um00439	9d00212	2.00E-24
BBH	9c00128	um00215	0	um00215	9c00128	0
BBH	9d00051	um00099	1.00E-175	um00099	9d00051	1.00E-180
BBH	9d00356	um11167	9.00E-72	um11167	9d00356	4.00E-69
NNN	9c00439	um03416	5.00E-30	um03416	8c00113	1.00E-134
BBH	9c00325	um00595	0	um00595	9c00325	0
BBH	9d00167	um00347	0	um00347	9d00167	0
BBH	9d00310	um00632	2.00E-80	um00632	9d00310	2.00E-80
BBH	9c00011	um00013	1.00E-90	um00013	9c00011	3.00E-88
NNN	9d00082	um10040.2	1.00E-101			
BBH	9c00420	um00784.2	3.00E-75	um00784.2	9c00420	4.00E-73
BBH	9d00290	um00588	0	um00588	9d00290	0
BBH	9d00374	um00739	0	um00739	9d00374	0
BBH	9d00383	um00757	0	um00757	9d00383	0
BBH	9c00243	um00438	6.00E-35	um00438	9c00243	7.00E-35
BBH	9d00002	um10000	0	um10000	9d00002	0
BBH	9c00134	um00227	0	um00227	9c00134	0
BBH	9d00285	um00572	1.00E-147	um00572	9d00285	1.00E-167
BBH	9d00397	um00782	7.00E-46	um00782	9d00397	8.00E-61
BBH	9c00070	um10024	8.00E-52	um10024	9c00070	4.00E-39
BBH	9c00306	um10463	0	um10463	9c00306	0
BBH	9c00329	um00604	2.00E-47	um00604	9c00329	2.00E-47
BBH	9d00227	um00465	1.00E-159	um00465	9d00227	1.00E-148
BBH	9d00035	um10018	1.00E-150	um10018	9d00035	1.00E-153
BBH	9c00054	um00078	0	um00078	9c00054	0
BBH	9c00001	um06490	0	um06490	9c00001	0
BBH	9d00137	um00277	1.00E-136	um00277	9d00137	1.00E-134
BBH	9c00360	um00657	0	um00657	9c00360	0
BBH	9c00313	um00578	5.00E-29	um00578	9c00313	3.00E-26
BBH	9d00144	um00293	1.00E-173	um00293	9d00144	1.00E-162
BBH	9c00302	um15006	6.00E-26	um15006	9c00302	3.00E-25
BBH	9c00318	um00564	1.00E-100	um00564	9c00318	1.00E-107
BBH	9c00219	um00360	0	um00360	9c00219	0
BBH	9d00073	um00137	1.00E-168	um00137	9d00073	1.00E-165
BBH	9c00174	um10094	0	um10094	9c00174	0
NNN	9d00130	um00262	1.00E-167			
BBH	9d00232	um00478	5.00E-86	um00478	9d00232	5.00E-94
BBH	9d00185	um11349	1.00E-75	um11349	9d00185	2.00E-93
BBH	9d00342	um11620	6.00E-96	um11620	9d00342	1.00E-96
BBH	9c00118	um10051	3.00E-49	um10051	9c00118	2.00E-49
BBH	9d00093	um10047	0	um10047	9d00093	0
NNN	9c00344	um00630	0			
BBH	9c00154	um10965.2	1.00E-114	um10965.2	9c00154	1.00E-111
BBH	9c00038	um00054	1.00E-102	um00054	9c00038	4.00E-96
BBH	9c00353	um00645	0	um00645	9c00353	0
BBH	9d00060	um10027	0	um10027	9d00060	0
BBH	9d00338	um11617	1.00E-130	um11617	9d00338	1.00E-129
BBH	9c00019	um00006	0	um00006	9c00019	0
BBH	9c00147	um00254	2.00E-51	um00254	9c00147	9.00E-47
BBH	9c00271	um12104	0	um12104	9c00271	0
BBH	9c00275	um00501	7.00E-95	um00501	9c00275	5.00E-95
BBH	9d00349	um00699	3.00E-95	um00699	9d00349	1.00E-109
BBH	9d00278	um00584	0	um00584	9d00278	0
BBH	9c00102	um00182	0	um00182	9c00102	0
NNN	9c00382	um00700	1.00E-139			
BBH	9d00294	um00599	1.00E-175	um00599	9d00294	1.00E-173
BBH	9c00368	um00660.2	0	um00660.2	9c00368	0
BBH	9c00288	um11437	9.00E-89	um11437	9c00288	7.00E-89
BBH	9c00381	um00698.2	4.00E-80	um00698.2	9c00381	2.00E-76
BBH	9d00364	um00722.2	7.00E-82	um00722.2	9d00364	1.00E-105
BBH	9c00064	um00097	1.00E-178	um00097	9c00064	0
BBH	9d00069	um12082	1.00E-119	um12082	9d00069	1.00E-118

BBH	9d00156	um00326	1.00E-68	um00326	9d00156	2.00E-68
BBH	9d00220	um00453	0	um00453	9d00220	0
BBH	9d00377	um11623	1.00E-140	um11623	9d00377	1.00E-142
BBH	9d00262	um00538	1.00E-67	um00538	9d00262	2.00E-64
BBH	9d00404	um15051	0	um15051	9d00404	0
BBH	9d00173	um00386	0	um00386	9d00173	0
NNN	9c00195	um11346	0			
BBH	9d00042	um10020	1.00E-165	um10020	9d00042	6.00E-94
BBH	9c00263	um10121	0	um10121	9c00263	0
BBH	9c00201	um00388	9.00E-94	um00388	9c00201	3.00E-98
BBH	9d00110	um11422	0	um11422	9d00110	0
BBH	9d00121	um00240	1.00E-159	um00240	9d00121	1.00E-159
BBH	9c00048	um00068	0	um00068	9c00048	0
BBH	9c00252	um12154	0	um12154	9c00252	0
BBH	9d00211	um12152	0	um12152	9d00211	0
BBH	9c00232	um10106	0	um10106	9c00232	0
BBH	9d00301	um10922	0	um10922	9d00301	0
BBH	9c00188	um00336	1.00E-30	um00336	9c00188	2.00E-31
BBH	9c00292	um00529	0	um00529	9c00292	0
BBH	9d00281	um12052	3.00E-90	um12052	9d00281	1.00E-72
BBH	9c00400	um00740	0	um00740	9c00400	0
BBH	9c00165	um10091	0	um10091	9c00165	0
BBH	9d00152	um00315	0	um00315	9d00152	0
BBH	9c00086	um00136.2	0	um00136.2	9c00086	1.00E-180
BBH	9d00319	um00658	1.00E-73	um00658	9d00319	1.00E-73
BBH	9c00179	um10097	1.00E-97	um10097	9c00179	7.00E-96
BBH	9c00324	um00594	0	um00594	9c00324	0
BBH	9d00357	um00711	1.00E-101	um00711	9d00357	1.00E-101
BBH	9d00181	um11354	5.00E-68	um11354	9d00181	4.00E-66
BBH	9c00014	um10944	1.00E-121	um10944	9c00014	1.00E-120
BBH	9c00198	um00393	0	um00393	9c00198	0
BBH	9c00090	um12089	1.00E-161	um12089	9c00090	1.00E-167
BBH	9c00095	um00159	1.00E-154	um00159	9c00095	1.00E-140
BBH	9d00034	um00072	1.00E-171	um00072	9d00034	1.00E-180
BBH	9c00222	um11348.2	0	um11348.2	9c00222	0
BBH	9d00180	um00372	0	um00372	9d00180	0
BBH	9d00119	um11428	0	um11428	9d00119	0
BBH	9d00101	um10052	0	um10052	9d00101	0
BBH	9c00346	um00635	0	um00635	9c00346	0
BBH	9c00143	um00245.2	1.00E-146	um00245.2	9c00143	1.00E-139
NNN	9d00209	um10114	6.00E-30	um10114	9d00210	7.00E-31
NNN	9d00142	um00286	4.00E-59			
BBH	9d00223	um00460	1.00E-136	um00460	9d00223	1.00E-140
NNN	9c00391	um03006	5.00E-18	um03006	19c00058	1.00E-139
BBH	9c00262	um00477	0	um00477	9c00262	0
BBH	9c00120	um00202	0	um00202	9c00120	0
BBH	9d00376	um00742	1.00E-113	um00742	9d00376	1.00E-122
BBH	9d00083	um00157	0	um00157	9d00083	0
BBH	9d00135	um00271	0	um00271	9d00135	0
BBH	9c00156	um00273	4.00E-32	um00273	9c00156	3.00E-30
BBH	9c00088	um00140	1.00E-133	um00140	9c00088	1.00E-133
BBH	9d00075	um00141	0	um00141	9d00075	0
BBH	9c00129	um00216	1.00E-165	um00216	9c00129	1.00E-168
BBH	9c00272	um11430	0	um11430	9c00272	0
BBH	9d00396	um00781	1.00E-22	um00781	9d00396	8.00E-25
BBH	9d00272	um10462	1.00E-133	um10462	9d00272	1.00E-139
BBH	9c00317	um00567	1.00E-55	um00567	9c00317	8.00E-56
BBH	9c00106	um00176	0	um00176	9c00106	0
BBH	9d00314	um00639	0	um00639	9d00314	0
BBH	9d00405	um00798	0	um00798	9d00405	0
BBH	9d00265	um00545	6.00E-85	um00545	9d00265	2.00E-78
NNN	9d00415	um01130	6.00E-25	um01130	7d00207	1.00E-125

BBH	9c00063	um00095	1.00E-132	um00095	9c00063	1.00E-132
BBH	9d00005	um12080	0	um12080	9d00005	0
BBH	9d00251	um12106	0	um12106	9d00251	0
BBH	9d00279	um00581	0	um00581	9d00279	0
BBH	9d00247	um11434	1.00E-156	um11434	9d00247	1.00E-153
BBH	9d00240	um15088	0	um15088	9d00240	0
NNN	9d00104	um00205	7.00E-14			
BBH	9d00170	um00392	7.00E-51	um00392	9d00170	7.00E-51
BBH	9d00065	um00120	1.00E-140	um00120	9d00065	1.00E-148
BBH	9d00100	um00195	0	um00195	9d00100	0
BBH	9c00350	um11450	5.00E-82	um11450	9c00350	2.00E-80
BBH	9c00181	um12150	7.00E-87	um12150	9c00181	2.00E-82
BBH	9c00250	um00451	3.00E-97	um00451	9c00250	3.00E-97
BBH	9d00231	um00476	1.00E-141	um00476	9d00231	1.00E-144
BBH	9d00098	um00189	0	um00189	9d00098	0
BBH	9c00037	um00053	1.00E-61	um00053	9c00037	2.00E-52
BBH	9c00216	um00365	1.00E-44	um00365	9c00216	3.00E-48
BBH	9c00377	um00689	0	um00689	9c00377	0
BBH	9c00383	um00701.2	4.00E-15	um00701.2	9c00383	2.00E-20
NNN	9c00051	um10019	3.00E-93	um10019	9c00052	1.00E-148
BBH	9d00305	um10925	7.00E-76	um10925	9d00305	2.00E-85
BBH	9d00238	um00491	0	um00491	9d00238	0
NNN	9d00072	um10032	2.00E-79			
NNN	9d00363	um00721	0			
BBH	9d00248	um00508	4.00E-61	um00508	9d00248	3.00E-61
BBH	9c00303	um00548	0	um00548	9c00303	0
BBH	9c00434	um00812	0	um00812	9c00434	0
BBH	9d00288	um10465.2	0	um10465.2	9d00288	0
BBH	9c00133	um00225	6.00E-65	um00225	9c00133	4.00E-62
BBH	9c00071	um00108	1.00E-166	um00108	9c00071	1.00E-167
BBH	9c00009	um00017	1.00E-150	um00017	9c00009	1.00E-150
BBH	9c00176	um00316	0	um00316	9c00176	0
BBH	9c00171	um00304	0	um00304	9c00171	0
BBH	9c00059	um00087	1.00E-148	um00087	9c00059	1.00E-142
BBH	9c00029	um00043	0	um00043	9c00029	0
BBH	9c00022	um00012	1.00E-43	um00012	9c00022	2.00E-55
BBH	9d00054	um00102	1.00E-130	um00102	9d00054	1.00E-127
NNN	9d00348	um11833	0			
BBH	9c00194	um11345	1.00E-100	um11345	9c00194	1.00E-100
BBH	9c00244	um00440	1.00E-135	um00440	9c00244	1.00E-138
BBH	9c00398	um00734	4.00E-66	um00734	9c00398	5.00E-63
BBH	9d00408	um15030	5.00E-65	um15030	9d00408	4.00E-59
BBH	9c00062	um00094	1.00E-111	um00094	9c00062	1.00E-111
BBH	9c00390	um11171	0	um11171	9c00390	0
BBH	9c00166	um00295	1.00E-109	um00295	9c00166	1.00E-111
BBH	9c00293	um00533	0	um00533	9c00293	0
BBH	9d00151	um00311	0	um00311	9d00151	0
BBH	9c00149	um00259	0	um00259	9c00149	0
BBH	9d00015	um00007	1.00E-123	um00007	9d00015	1.00E-129
BBH	9d00141	um10088	0	um10088	9d00141	0
BBH	9d00182	um11353	0	um11353	9d00182	0
BBH	9c00260	um10120	0	um10120	9c00260	0
BBH	9c00399	um15019	0	um15019	9c00399	0
NNN	9d00336	um00679	0			
BBH	9d00165	um00342	1.00E-139	um00342	9d00165	1.00E-125
BBH	9d00026	um00045	0	um00045	9d00026	0
BBH	9d00088	um00167	0	um00167	9d00088	0
BBH	9c00192	um00343	0	um00343	9c00192	0
BBH	9d00092	um00181	0	um00181	9d00092	0
BBH	9c00310	um00585	0	um00585	9c00310	0
BBH	9c00189	um00338	0	um00338	9c00189	0
BBH	9c00404	um15024	0	um15024	9c00404	0

BBH	9d00410	um00808	1.00E-159	um00808	9d00410	1.00E-166
NNN	9d00045	um02900	1.00E-114	um02900	19d00128	0
BBH	9d00302	um10923.2	0	um10923.2	9d00302	0
BBH	9c00098	um00164.2	1.00E-162	um00164.2	9c00098	1.00E-170
BBH	9c00144	um00247	0	um00247	9c00144	0
BBH	9c00032	um00047	9.00E-71	um00047	9c00032	8.00E-71
BBH	9d00192	um15058	0	um15058	9d00192	0
BBH	9c00273	um00499	1.00E-146	um00499	9c00273	1.00E-140
BBH	9c00238	um00429	0	um00429	9c00238	0
BBH	9d00313	um00638	1.00E-134	um00638	9d00313	1.00E-140
BBH	9c00347	um00636	9.00E-66	um00636	9c00347	2.00E-68
BBH	9c00187	um00334	0	um00334	9c00187	1.00E-158
BBH	9c00282	um00507	6.00E-80	um00507	9c00282	7.00E-83
BBH	9d00222	um00458	1.00E-151	um00458	9d00222	1.00E-152
BBH	9c00337	um00620	6.00E-55	um00620	9c00337	6.00E-55
BBH	9c00323	um00593	2.00E-27	um00593	9c00323	2.00E-27
BBH	9c00023	um00028	4.00E-99	um00028	9c00023	4.00E-99
BBH	9c00117	um00197	0	um00197	9c00117	0
BBH	9d00333	um00673	1.00E-97	um00673	9d00333	1.00E-97
BBH	9d00158	um00329	0	um00329	9d00158	0
NNN	9d00229	um10119	0			
BBH	9d00106	um00209.2	2.00E-20	um00209.2	9d00106	1.00E-20
BBH	9d00037	um00076	0	um00076	9d00037	0
BBH	9d00199	um00417	1.00E-89	um00417	9d00199	2.00E-94
BBH	9c00047	um00067	0	um00067	9c00047	0
BBH	9d00210	um10114	3.00E-27	um10114	9d00210	7.00E-31
BBH	9d00097	um00188	7.00E-73	um00188	9d00097	1.00E-79
BBH	9d00417	um04111	1.00E-117	um04111	9d00417	1.00E-117
BBH	9c00221	um00355	0	um00355	9c00221	0
BBH	9c00083	um00131	1.00E-177	um00131	9c00083	1.00E-177
BBH	9c00351	um11451	1.00E-129	um11451	9c00351	1.00E-134
BBH	9d00091	um00183	1.00E-147	um00183	9d00091	1.00E-152
BBH	9c00186	um00332	0	um00332	9c00186	0
BBH	9c00202	um00385	1.00E-130	um00385	9c00202	1.00E-137
NNN	9d00241	um15088	6.00E-87	um15088	9d00240	0
NNN	9d00062	um00700	8.00E-24			
BBH	9c00246	um00444	1.00E-144	um00444	9c00246	1.00E-170
BBH	9d00150	um00309	1.00E-136	um00309	9d00150	1.00E-137
BBH	9d00409	um00805	2.00E-80	um00805	9d00409	1.00E-87
BBH	9d00334	um00674	0	um00674	9d00334	0
BBH	9d00242	um00495	6.00E-55	um00495	9d00242	5.00E-57
BBH	9c00079	um00124	2.00E-48	um00124	9c00079	2.00E-57
NNN	9d00280	um00579	0			
BBH	9d00368	um00727	0	um00727	9d00368	0
NNN	9d00171	um00390	1.00E-65			
BBH	9d00324	um00666	1.00E-156	um00666	9d00324	1.00E-156
BBH	9d00178	um00376	0	um00376	9d00178	0
BBH	9d00004	um10004	1.00E-139	um10004	9d00004	1.00E-147
BBH	9d00074	um10033	0	um10033	9d00074	0
BBH	9d00136	um00272	1.00E-171	um00272	9d00136	1.00E-162
BBH	9c00105	um00177	0	um00177	9c00105	0
BBH	9c00308	um00561.2	1.00E-125	um00561.2	9c00308	1.00E-123
BBH	9d00250	um00515	0	um00515	9d00250	0
NNN	9d00366	um11779	3.00E-88	um11779	2d00002	0
BBH	9d00264	um00542	0	um00542	9d00264	1.00E-178
BBH	9c00363	um11456	0	um11456	9c00363	1.00E-172
BBH	9d00306	um00622	0	um00622	9d00306	0
BBH	9d00395	um00780	0	um00780	9d00395	0
BBH	9d00402	um10788.2	0	um10788.2	9d00402	0
BBH	9d00394	um00779	1.00E-119	um00779	9d00394	1.00E-119
BBH	9c00116	um00196	6.00E-94	um00196	9c00116	3.00E-95
BBH	9c00433	um00810	1.00E-163	um00810	9c00433	1.00E-171

BBH	9d00287	um00568	1.00E-176	um00568	9d00287	0
BBH	9d00389	um00770	0	um00770	9d00389	0
BBH	9c00072	um00111	1.00E-111	um00111	9c00072	1.00E-116
BBH	9d00057	um10023	0	um10023	9d00057	1.00E-159
BBH	9c00132	um00222.2	1.00E-148	um00222.2	9c00132	1.00E-164
BBH	9c00419	um00778	1.00E-19	um00778	9c00419	1.00E-20
BBH	9d00267	um00547	1.00E-142	um00547	9d00267	1.00E-142
BBH	9c00052	um10019	1.00E-159	um10019	9c00052	1.00E-148
BBH	9c00366	um11830	3.00E-91	um11830	9c00366	2.00E-89
BBH	9d00375	um00741	0	um00741	9d00375	0
BBH	9c00042	um00059	1.00E-152	um00059	9c00042	1.00E-145
BBH	9d00230	um00473	0	um00473	9d00230	0
BBH	9c00121	um00203	1.00E-132	um00203	9c00121	1.00E-145
BBH	9c00304	um00553	0	um00553	9c00304	0
BBH	9d00014	um00003	1.00E-151	um00003	9d00014	1.00E-151
BBH	9d00198	um00412	1.00E-173	um00412	9d00198	1.00E-171
BBH	9c00069	um00105	0	um00105	9c00069	0
BBH	9d00249	um00506	1.00E-101	um00506	9d00249	1.00E-102
NNN	9d00359	um00715	2.00E-98			
NNN	9c00217	um11350.2	0			
BBH	9c00068	um00104	3.00E-54	um00104	9c00068	4.00E-56
BBH	9c00208	um15041	0	um15041	9c00208	0
BBH	9d00218	um00454	0	um00454	9d00218	0
BBH	9c00428	um11837	0	um11837	9c00428	0
BBH	9c00173	um00310	0	um00310	9c00173	0
BBH	9c00031	um00046	0	um00046	9c00031	0
BBH	9c00007	um12081	1.00E-97	um12081	9c00007	3.00E-89
BBH	9c00115	um00193	1.00E-159	um00193	9c00115	1.00E-171
BBH	9d00096	um10044	0	um10044	9d00096	0
BBH	9c00075	um00117	0	um00117	9c00075	0
BBH	9d00036	um00074	8.00E-60	um00074	9d00036	9.00E-60
BBH	9c00057	um00081	1.00E-60	um00081	9c00057	4.00E-61
BBH	9d00237	um00490	0	um00490	9d00237	0
BBH	9c00384	um00702	2.00E-29	um00702	9c00384	2.00E-33
BBH	9d00303	um00614	0	um00614	9d00303	0
BBH	9d00346	um00694.2	0	um00694.2	9d00346	0
BBH	9d00390	um00771	7.00E-75	um00771	9d00390	8.00E-74
BBH	9c00326	um00597	0	um00597	9c00326	0
BBH	9d00103	um00204	0	um00204	9d00103	0
BBH	9d00388	um00768	0	um00768	9d00388	0
BBH	9c00436	um00815	0	um00815	9c00436	0
BBH	9d00134	um00270	1.00E-106	um00270	9d00134	1.00E-113
BBH	9c00073	um00114	1.00E-113	um00114	9c00073	1.00E-110
BBH	9c00027	um00039	1.00E-103	um00039	9c00027	2.00E-92
NNN	9c00414	um11200	1.00E-110			
BBH	9c00438	um11634	0	um11634	9c00438	0
BBH	9d00246	um00510	0	um00510	9d00246	0
BBH	9d00253	um00522	1.00E-36	um00522	9d00253	1.00E-41
BBH	9c00248	um00448	9.00E-29	um00448	9c00248	5.00E-33
BBH	9c00108	um10046	0	um10046	9c00108	0
BBH	9d00089	um10049	0	um10049	9d00089	0
BBH	9c00193	um00344	1.00E-117	um00344	9c00193	1.00E-109
BBH	9d00273	um00557	0	um00557	9d00273	0
BBH	9d00365	um11173	7.00E-48	um11173	9d00365	4.00E-49
BBH	9c00287	um11436	1.00E-123	um11436	9c00287	1.00E-125
BBH	9d00393	um00777	0	um00777	9d00393	0
BBH	9d00283	um00574	0	um00574	9d00283	0
BBH	9d00188	um11608.2	2.00E-74	um11608.2	9d00188	2.00E-74
BBH	9d00128	um00258	1.00E-103	um00258	9d00128	1.00E-111
BBH	9c00130	um00218	0	um00218	9c00130	0
BBH	9d00169	um00394	3.00E-84	um00394	9d00169	6.00E-81
BBH	9c00061	um00092	1.00E-145	um00092	9c00061	1.00E-145

BBH	9d00080	um10038	0	um10038	9d00080	0
BBH	9c00162	um00288	1.00E-170	um00288	9c00162	1.00E-171
BBH	9d00298	um15049	0	um15049	9d00298	0
BBH	9c00050	um12085	4.00E-59	um12085	9c00050	2.00E-56
BBH	9d00176	um00379	0	um00379	9d00176	0
BBH	9d00191	um00400	0	um00400	9d00191	0
BBH	9c00278	um00503	7.00E-31	um00503	9c00278	3.00E-31
BBH	9d00360	um11168	7.00E-40	um11168	9d00360	4.00E-40
BBH	9c00357	um00651	0	um00651	9c00357	0
BBH	9d00067	um00126	0	um00126	9d00067	0
NNN	9c00122	um00206.2	0			
NNN	9d00316	um11452	3.00E-24			
BBH	9c00214	um11352	0	um11352	9c00214	0
BBH	9c00107	um00175	1.00E-148	um00175	9c00107	1.00E-153
BBH	9c00157	um00274	1.00E-147	um00274	9c00157	1.00E-144
NNN	9c00333	um00612	0			
BBH	9c00034	um00050	1.00E-108	um00050	9c00034	1.00E-110
BBH	9c00397	um00738	6.00E-90	um00738	9c00397	9.00E-85
NNN	9d00197	um00410	1.00E-88			
BBH	9d00224	um00461	0	um00461	9d00224	0
NNN	9c00041	um00374	6.00E-60	um00374	9c00209	0
NNN	9d00095	um12091	1.00E-13			
BBH	9c00016	um00002	0	um00002	9c00016	0
BBH	9c00335	um00616	1.00E-84	um00616	9c00335	1.00E-95
BBH	9d00297	um00605	0	um00605	9d00297	0
BBH	9d00190	um00399	1.00E-139	um00399	9d00190	1.00E-134
BBH	9d00028	um00056	0	um00056	9d00028	0
BBH	9d00351	um00703	0	um00703	9d00351	0
BBH	9c00284	um11433	0	um11433	9c00284	0
BBH	9c00199	um00391.2	1.00E-118	um00391.2	9c00199	1.00E-107
BBH	9c00211	um00371	1.00E-116	um00371	9c00211	1.00E-116
BBH	9d00201	um10107	0	um10107	9d00201	0
BBH	9c00247	um02506	6.00E-74	um02506	9c00247	3.00E-75
BBH	9c00237	um00427	0	um00427	9c00237	0
BBH	9c00020	um00008	1.00E-145	um00008	9c00020	1.00E-158
BBH	9c00267	um11825	5.00E-45	um11825	9c00267	4.00E-48
BBH	9d00163	um11340	3.00E-85	um11340	9d00163	1.00E-86
BBH	9c00378	um00691	0	um00691	9c00378	0
BBH	9d00323	um00668	1.00E-179	um00668	9d00323	0
BBH	9c00114	um00192	1.00E-162	um00192	9c00114	1.00E-166
BBH	9d00413	um00813	0	um00813	9d00413	0
BBH	9c00299	um00539	0	um00539	9c00299	0
BBH	9d00090	um00184	1.00E-154	um00184	9d00090	1.00E-150
BBH	9d00040	um00084.2	0	um00084.2	9d00040	0
BBH	9d00021	um10009	0	um10009	9d00021	0
NNN	9c00402	um00745	9.00E-35			
BBH	9d00148	um00305	0	um00305	9d00148	0
BBH	9c00254	um00464	0	um00464	9c00254	0
BBH	9d00153	um00319	1.00E-134	um00319	9d00153	1.00E-155
NNN	9d00086	um00163	1.00E-102			
BBH	9c00146	um00251	1.00E-118	um00251	9c00146	1.00E-112
BBH	9d00399	um00786	1.00E-109	um00786	9d00399	1.00E-107
BBH	9c00167	um00298	0	um00298	9c00167	0
NNN	9c00385	um00702	7.00E-24	um00702	9c00384	2.00E-33
BBH	9c00028	um00040	1.00E-79	um00040	9c00028	1.00E-79
BBH	9d00255	um00526	0	um00526	9d00255	0
BBH	9c00131	um00219	0	um00219	9c00131	0
BBH	9c00386	um11165	0	um11165	9c00386	0
NNN	9d00345	um00693	0			
BBH	9c00435	um11631	1.00E-119	um11631	9c00435	1.00E-137
BBH	9d00140	um00282	0	um00282	9d00140	0
BBH	9d00387	um11201.2	0	um11201.2	9d00387	0

BBH	9c00039	um00055	0	um00055	9c00039	0
BBH	9c00178	um00318	1.00E-146	um00318	9c00178	1.00E-146
BBH	9d00012	um10945	1.00E-159	um10945	9d00012	1.00E-159
BBH	9c00076	um00119	0	um00119	9c00076	0
BBH	9d00217	um00447	1.00E-47	um00447	9d00217	2.00E-41
BBH	9d00266	um10459	0	um10459	9d00266	0
BBH	9c00215	um00366	0	um00366	9c00215	0
BBH	9c00021	um00009	4.00E-95	um00009	9c00021	1.00E-102
BBH	9d00126	um00255	2.00E-86	um00255	9d00126	1.00E-101
BBH	9c00429	um00802	0	um00802	9c00429	0
NNN	9d00289	um00565	0			
BBH	9c00074	um00116	0	um00116	9c00074	0
BBH	9c00058	um00085	0	um00085	9c00058	0
NNN	9c00067	um03407	2.00E-28	um03407	8d00115	0
BBH	9d00261	um11442	0	um11442	9d00261	0
BBH	9d00031	um10015	3.00E-88	um10015	9d00031	1.00E-107
BBH	9d00177	um00378	1.00E-157	um00378	9d00177	1.00E-177
BBH	9c00158	um00275	0	um00275	9c00158	0
BBH	9c00413	um00763	0	um00763	9c00413	0
BBH	9d00299	um00607	2.00E-94	um00607	9d00299	2.00E-91
BBH	9c00279	um00513	0	um00513	9c00279	0
BBH	9c00349	um00637	1.00E-113	um00637	9c00349	1.00E-108
BBH	9c00396	um11172	0	um11172	9c00396	0
BBH	9d00386	um11199	1.00E-124	um11199	9d00386	1.00E-129
BBH	9d00077	um00144	1.00E-139	um00144	9d00077	1.00E-132
BBH	9d00403	um00792	2.00E-24	um00792	9d00403	2.00E-18
BBH	9c00418	um00775	0	um00775	9c00418	0
BBH	9c00370	um00675	0	um00675	9c00370	0
BBH	9d00064	um00118	0	um00118	9d00064	0
NNN	9c00123	um00208	0			
BBH	9d00414	um11632	0	um11632	9d00414	0
BBH	9d00023	um10010	1.00E-62	um10010	9d00023	2.00E-71
BBH	9d00252	um11438	7.00E-60	um11438	9d00252	1.00E-69
BBH	9c00437	um00817	0	um00817	9c00437	0
BBH	9c00249	um00450	0	um00450	9c00249	0
BBH	9d00038	um00077	1.00E-169	um00077	9d00038	1.00E-168
BBH	9c00085	um10031	1.00E-146	um10031	9c00085	1.00E-146
NNN	9d00274	um00560	0			
BBH	9d00392	um00774	1.00E-112	um00774	9d00392	1.00E-112
BBH	9c00312	um10468.2	1.00E-126	um10468.2	9c00312	1.00E-124
BBH	9d00331	um11612	3.00E-66	um11612	9d00331	2.00E-74
BBH	9d00081	um00153	4.00E-42	um00153	9d00081	4.00E-49
BBH	9d00189	um11610	1.00E-143	um11610	9d00189	1.00E-143
BBH	9d00129	um10961	3.00E-80	um10961	9d00129	3.00E-80
BBH	9c00161	um10089.2	1.00E-128	um10089.2	9c00161	1.00E-124
BBH	9c00240	um10113	1.00E-152	um10113	9c00240	1.00E-140
BBH	9d00352	um11164	2.00E-73	um11164	9d00352	3.00E-73
BBH	9c00406	um00750	1.00E-151	um00750	9c00406	1.00E-161
BBH	9d00300	um00608	2.00E-62	um00608	9d00300	2.00E-62
BBH	9c00239	um00430	0	um00430	9c00239	0
BBH	9c00285	um00516	0	um00516	9c00285	0
BBH	9c00170	um00302	1.00E-101	um00302	9c00170	4.00E-98
BBH	9d00339	um00684	1.00E-163	um00684	9d00339	0
NNN	9c00196	um00350	0			
BBH	9c00150	um10963	8.00E-85	um10963	9c00150	1.00E-83
BBH	9d00159	um10100	3.00E-91	um10100	9d00159	3.00E-91
BBH	9c00180	um00322	0	um00322	9c00180	0
BBH	9c00236	um10108	1.00E-112	um10108	9c00236	1.00E-115
BBH	9c00220	um00357	0	um00357	9c00220	0
BBH	9d00055	um00103	0	um00103	9d00055	0
BBH	9c00015	um10946	0	um10946	9c00015	0
BBH	9d00117	um11426	1.00E-165	um11426	9d00117	1.00E-162

BBH	9c00336	um10924	0	um10924	9c00336	0
BBH	9c00296	um00536	1.00E-108	um00536	9c00296	1.00E-104
BBH	9c00209	um00374	0	um00374	9c00209	0
BBH	9c00265	um00483	2.00E-32	um00483	9c00265	2.00E-30
BBH	9c00033	um00049	6.00E-44	um00049	9c00033	5.00E-46
BBH	9c00097	um10041.2	3.00E-85	um10041.2	9c00097	3.00E-70
BBH	9d00094	um00174	0	um00174	9d00094	0
BBH	9d00029	um00057	1.00E-72	um00057	9d00029	3.00E-72
BBH	9c00155	um10966	0	um10966	9c00155	0
BBH	9d00105	um00207.2	1.00E-175	um00207.2	9d00105	0
BBH	9d00200	um00418	0	um00418	9d00200	0
BBH	9c00230	um10104	1.00E-125	um10104	9c00230	1.00E-127
BBH	9c00060	um00089	1.00E-109	um00089	9c00060	1.00E-104
BBH	9d00282	um00576	2.00E-54	um00576	9d00282	1.00E-56
BBH	9d00216	um00446	0	um00446	9d00216	0
BBH	9d00030	um00061	0	um00061	9d00030	0
BBH	9d00332	um00672	1.00E-160	um00672	9d00332	1.00E-171
BBH	9c00365	um00667	2.00E-93	um00667	9c00365	1.00E-93
BBH	9c00049	um10017	1.00E-175	um10017	9c00049	1.00E-175
BBH	9c00423	um00789	0	um00789	9c00423	0
BBH	9c00379	um00692	2.00E-59	um00692	9c00379	3.00E-62
BBH	9c00229	um00411.2	1.00E-143	um00411.2	9c00229	1.00E-137
BBH	9d00164	um11341	1.00E-170	um11341	9d00164	1.00E-168
BBH	9c00430	um11627	1.00E-67	um11627	9c00430	2.00E-67
BBH	9d00147	um12149	3.00E-59	um12149	9d00147	2.00E-50
BBH	9c00200	um00389	0	um00389	9c00200	0
BBH	9c00168	um00299	0	um00299	9c00168	0
BBH	9c00087	um00138	0	um00138	9c00087	0
BBH	9d00043	um00091	1.00E-107	um00091	9d00043	2.00E-95
BBH	9d00304	um00617	1.00E-138	um00617	9d00304	1.00E-139
BBH	9d00013	um00001	1.00E-124	um00001	9d00013	1.00E-124
BBH	9d00245	um00512	6.00E-78	um00512	9d00245	7.00E-78
BBH	9d00087	um10042	0	um10042	9d00087	0
BBH	9d00025	um00042	0	um00042	9d00025	0
BBH	9d00369	um12110	1.00E-114	um12110	9d00369	1.00E-118
BBH	9d00168	um00349.2	2.00E-38	um00349.2	9d00168	9.00E-41
BBH	9c00409	um00756	0	um00756	9c00409	0
BBH	9c00137	um00233	1.00E-45	um00233	9c00137	8.00E-45
BBH	9d00254	um11439	8.00E-59	um11439	9d00254	1.00E-113
BBH	9d00400	um10787	0	um10787	9d00400	0
BBH	9c00101	um10048	6.00E-30	um10048	9c00101	6.00E-30
NNN	9c00124	um00210	3.00E-55			
BBH	9d00275	um00563	3.00E-64	um00563	9d00275	3.00E-69
BBH	9c00421	um00785	0	um00785	9c00421	0
BBH	9d00391	um11202	7.00E-15	um11202	9d00391	3.00E-15
BBH	9c00289	um00525	1.00E-120	um00525	9c00289	1.00E-120
BBH	9d00039	um00082	1.00E-44	um00082	9d00039	1.00E-68
BBH	9d00370	um00737	0	um00737	9d00370	0
NNN	9c00358	um11827	1.00E-106			
BBH	9d00318	um11453	3.00E-66	um11453	9d00318	2.00E-67
BBH	9c00355	um11455	0	um11455	9c00355	0
BBH	9d00059	um10025	0	um10025	9d00059	0
BBH	9c00005	um10002	0	um10002	9c00005	0
BBH	9c00148	um12148	0	um12148	9c00148	0
BBH	9d00362	um00719	2.00E-41	um00719	9d00362	1.00E-45
BBH	9c00417	um00773	1.00E-58	um00773	9c00417	2.00E-58
BBH	9d00385	um11198	0	um11198	9d00385	0
BBH	9c00055	um00079	1.00E-161	um00079	9c00055	1.00E-161
BBH	9c00026	um00037	0	um00037	9c00026	0
NNN	9c00066	um04886	3.00E-21	um04886	26d00001	0
BBH	9d00127	um00256	1.00E-129	um00256	9d00127	1.00E-129
BBH	9c00190	um00340	1.00E-112	um00340	9c00190	1.00E-130

BBH	9c00204	um11604	0	um11604	9c00204	0
BBH	9c00002	um06480	1.00E-27	um06480	9c00002	4.00E-48
BBH	9c00113	um10050	3.00E-33	um10050	9c00113	4.00E-26
BBH	9d00024	um00038	1.00E-142	um00038	9d00024	1.00E-139
BBH	9c00077	um00122	0	um00122	9c00077	0
BBH	9c00311	um00582	0	um00582	9c00311	0
BBH	9d00235	um15059	0	um15059	9d00235	0
BBH	9c00387	um00710	0	um00710	9c00387	0
BBH	9d00131	um00263.2	3.00E-74	um00263.2	9d00131	7.00E-74
BBH	9d00329	um00670	0	um00670	9d00329	0
NNN	9c00316	um00569	0			
BBH	9c00269	um00494	4.00E-79	um00494	9c00269	1.00E-100
BBH	9d00076	um10035	1.00E-23	um10035	9d00076	1.00E-23
BBH	9c00431	um12111	1.00E-105	um12111	9c00431	1.00E-103
BBH	9d00308	um15009	0	um15009	9d00308	0
BBH	9d00371	um00736	0	um00736	9d00371	0
BBH	9d00344	um15016	0	um15016	9d00344	0
BBH	9c00328	um00602	0	um00602	9c00328	0
NNN	9d00146	um00300	0			
BBH	9d00018	um00029	1.00E-174	um00029	9d00018	1.00E-178
BBH	9d00234	um00482	5.00E-16	um00482	9d00234	6.00E-16
BBH	9d00315	um11449	0	um11449	9d00315	0
BBH	9c00231	um10105	0	um10105	9c00231	0
BBH	9d00341	um00687	0	um00687	9d00341	0
BBH	9c00408	um00755	1.00E-120	um00755	9c00408	1.00E-129
BBH	9c00082	um10030	1.00E-108	um10030	9c00082	1.00E-106
BBH	9d00291	um00589	6.00E-93	um00589	9d00291	7.00E-88
BBH	9c00025	um00036	7.00E-67	um00036	9c00025	1.00E-66
BBH	9d00007	um10007	1.00E-130	um10007	9d00007	1.00E-141
BBH	9d00155	um10098	0	um10098	9d00155	0
BBH	9d00161	um00333	0	um00333	9d00161	0
BBH	9c00320	um00590	5.00E-16	um00590	9c00320	4.00E-13
BBH	9c00371	um11613.2	0	um11613.2	9c00371	1.00E-178
BBH	9d00337	um11615.2	2.00E-73	um11615.2	9d00337	1.00E-72
BBH	9c00257	um00468	0	um00468	9c00257	0
BBH	9d00226	um00463	1.00E-157	um00463	9d00226	1.00E-159
BBH	9c00169	um10092.2	0	um10092.2	9c00169	0
BBH	9d00295	um00601	1.00E-135	um00601	9d00295	1.00E-141
BBH	9d00196	um11822	4.00E-66	um11822	9d00196	8.00E-55
BBH	9c00206	um00380	1.00E-170	um00380	9c00206	1.00E-170
BBH	9c00044	um00064	1.00E-149	um00064	9c00044	1.00E-150
BBH	9d00114	um00226	1.00E-122	um00226	9d00114	1.00E-113
BBH	9c00331	um00609	0	um00609	9c00331	0
BBH	9c00175	um10095	0	um10095	9c00175	0
BBH	9d00174	um11606	4.00E-54	um11606	9d00174	2.00E-56
BBH	9c00395	um00728	1.00E-130	um00728	9c00395	1.00E-139
BBH	9c00018	um00005	0	um00005	9c00018	0
BBH	9d00259	um00534	1.00E-167	um00534	9d00259	5.00E-95
BBH	9c00151	um00264	1.00E-124	um00264	9c00151	1.00E-157
BBH	9d00328	um00652	0	um00652	9d00328	0
NNN	9d00353	um00706	1.00E-171			
BBH	9d00186	um00356	0	um00356	9d00186	0
NNN	9c00197	um11347.2	0			
BBH	9c00160	um10087.2	1.00E-121	um10087.2	9c00160	1.00E-114
BBH	9d00205	um10112	9.00E-70	um10112	9d00205	4.00E-71
BBH	9d00033	um00071	0	um00071	9d00033	0
BBH	9c00369	um00671	1.00E-143	um00671	9c00369	1.00E-127
BBH	9c00092	um00152	1.00E-133	um00152	9c00092	1.00E-135
BBH	9c00235	um00424	0	um00424	9c00235	0
BBH	9c00359	um15012	0	um15012	9c00359	0
BBH	9d00276	um12146	1.00E-63	um12146	9d00276	1.00E-64
BBH	9d00154	um00321	0	um00321	9d00154	0

BBH	9d00124	um10956	3.00E-15	um10956	9d00124	4.00E-15
BBH	9d00361	um11170	0	um11170	9d00361	0
BBH	9c00259	um12155.2	0	um12155.2	9c00259	0
BBH	9d00175	um11605	1.00E-128	um11605	9d00175	1.00E-130
BBH	9c00152	um00265	1.00E-88	um00265	9c00152	1.00E-81
BBH	9d00066	um10028	1.00E-37	um10028	9d00066	9.00E-29
BBH	9c00411	um11197	1.00E-177	um11197	9c00411	1.00E-180
BBH	9c00080	um00127	0	um00127	9c00080	0
BBH	9c00046	um10014	3.00E-66	um10014	9c00046	2.00E-67
BBH	9c00401	um00743	0	um00743	9c00401	0
BBH	9c00100	um00168	0	um00168	9c00100	0
BBH	9d00061	um00113	0	um00113	9d00061	0
BBH	9c00286	um00518	0	um00518	9c00286	0
BBH	9c00010	um15048	0	um15048	9c00010	0
NNN	9d00401	um00790	0			
BBH	9c00172	um00308	1.00E-145	um00308	9c00172	1.00E-135
BBH	9c00125	um12147.2	7.00E-85	um12147.2	9c00125	7.00E-85
BBH	9d00020	um00032	2.00E-13	um00032	9d00020	5.00E-14
BBH	9d00354	um11166.2	0	um11166.2	9d00354	0
BBH	9d00053	um00101	0	um00101	9d00053	0
BBH	9c00422	um00788	1.00E-157	um00788	9c00422	1.00E-162
BBH	9d00343	um04309	1.00E-133	um04309	9d00343	1.00E-126
BBH	9c00315	um10466.2	1.00E-139	um10466.2	9c00315	1.00E-139
BBH	9d00372	um00735	1.00E-128	um00735	9d00372	1.00E-142
BBH	9d00102	um10053	5.00E-90	um10053	9d00102	2.00E-80
BBH	9c00210	um11355	2.00E-21	um11355	9c00210	2.00E-21
NNN	9d00132	um10964	1.00E-160			
BBH	9d00309	um11447.2	0	um11447.2	9d00309	0
BBH	9c00327	um12108	1.00E-115	um12108	9c00327	1.00E-113
BBH	9c00213	um00369	9.00E-57	um00369	9c00213	4.00E-61
BBH	9c00388	um00712	0	um00712	9c00388	0
BBH	9c00136	um11425	1.00E-171	um11425	9c00136	0
BBH	9c00264	um00481	5.00E-89	um00481	9c00264	1.00E-81
BBH	9d00382	um00754	2.00E-22	um00754	9d00382	6.00E-20
BBH	9d00317	um00646	1.00E-166	um00646	9d00317	1.00E-149
BBH	9c00427	um00797	1.00E-148	um00797	9c00427	1.00E-160
BBH	9c00300	um11445	1.00E-30	um11445	9c00300	3.00E-31
BBH	9d00019	um00031	1.00E-155	um00031	9d00019	1.00E-156
BBH	9c00241	um00435	1.00E-148	um00435	9c00241	1.00E-147
BBH	9c00191	um11343.2	1.00E-65	um11343.2	9c00191	1.00E-61
NNN	9c00065	um03403.2	2.00E-28	um03403.2	8d00113	0
BBH	9c00309	um00586	3.00E-40	um00586	9c00309	2.00E-34
BBH	9c00078	um00123	0	um00123	9c00078	0
BBH	9c00109	um00172	1.00E-137	um00172	9c00109	1.00E-144
BBH	9c00003	um10001	2.00E-72	um10001	9c00003	1.00E-69
BBH	9d00268	um00549	0	um00549	9d00268	0
BBH	9d00320	um00654	4.00E-94	um00654	9d00320	2.00E-91
BBH	9d00203	um10110	2.00E-37	um10110	9d00203	2.00E-37
BBH	9d00284	um00573	2.00E-38	um00573	9d00284	3.00E-44
BBH	9d00058	um00109	0	um00109	9d00058	0
BBH	9c00056	um00080	0	um00080	9c00056	0
BBH	9d00292	um00596	1.00E-173	um00596	9d00292	1.00E-161
BBH	9c00374	um11618	2.00E-98	um11618	9c00374	6.00E-95
BBH	9c00340	um00624	1.00E-151	um00624	9c00340	1.00E-145
BBH	9c00342	um10929	4.00E-33	um10929	9c00342	2.00E-33
BBH	9c00356	um00650	0	um00650	9c00356	0
BBH	9c00416	um00769	4.00E-79	um00769	9c00416	3.00E-87
BBH	9c00145	um10958	0	um10958	9c00145	0
BBH	9d00202	um00423	2.00E-89	um00423	9d00202	7.00E-98
BBH	9c00205	um00381	0	um00381	9c00205	0
BBH	9d00052	um10022	2.00E-23	um10022	9d00052	1.00E-32
BBH	9c00207	um05082	0	um05082	9c00207	0

BBH	9d00022	um00034	0	um00034	9d00022	0
BBH	9c00372	um00678	0	um00678	9c00372	0
BBH	9c00256	um00467	0	um00467	9c00256	0
BBH	9d00204	um00428	1.00E-157	um00428	9d00204	1.00E-157
BBH	9c00227	um11824	0	um11824	9c00227	0
BBH	9c00126	um00212	1.00E-175	um00212	9c00126	0
BBH	9d00321	um00669	1.00E-139	um00669	9d00321	1.00E-125
BBH	9d00412	um00811	1.00E-118	um00811	9d00412	1.00E-121
BBH	9d00006	um10005	0	um10005	9d00006	0
BBH	9d00084	um12090	1.00E-147	um12090	9d00084	1.00E-150
BBH	9d00406	um00800	0	um00800	9d00406	0
BBH	9d00296	um00603	6.00E-72	um00603	9d00296	1.00E-73
BBH	9d00225	um10117	0	um10117	9d00225	0
BBH	9d00145	um00297	1.00E-158	um00297	9d00145	1.00E-164
BBH	9d00032	um10016	1.00E-168	um10016	9d00032	0
BBH	9d00162	um10102	0	um10102	9d00162	0
BBH	9d00187	um00354	1.00E-153	um00354	9d00187	1.00E-143
BBH	9d00041	um12086	4.00E-93	um12086	9d00041	4.00E-96
BBH	9d00214	um00443	1.00E-102	um00443	9d00214	1.00E-103
BBH	9c00212	um00370	0	um00370	9c00212	0
BBH	9c00407	um00751	0	um00751	9c00407	0
BBH	9c00111	um00169	1.00E-124	um00169	9c00111	1.00E-133
BBH	9c00089	um10034	7.00E-22	um10034	9c00089	6.00E-22
BBH	9d00327	um11828	0	um11828	9d00327	0
NNN	9d00258	um00530	1.00E-111			
BBH	9c00140	um00239	0	um00239	9c00140	0
BBH	9c00139	um00235	0	um00235	9c00139	0
BBH	9c00182	um00325	0	um00325	9c00182	0
BBH	9c00091	um10036	0	um10036	9c00091	0
BBH	9c00234	um00422	1.00E-123	um00422	9c00234	1.00E-119
BBH	9c00332	um00610	2.00E-73	um00610	9c00332	4.00E-72
BBH	9c00036	um10013.2	0	um10013.2	9c00036	0
BBH	9c00225	um00403	1.00E-175	um00403	9c00225	1.00E-176
BBH	9c00266	um00486	0	um00486	9c00266	0
BBH	9d00071	um00132	2.00E-62	um00132	9d00071	5.00E-63
BBH	9d00195	um00406	7.00E-45	um00406	9d00195	3.00E-34
BBH	9c00043	um00063	1.00E-64	um00063	9c00043	2.00E-31
BBH	9c00334	um00615	6.00E-95	um00615	9c00334	6.00E-95
NNN	9d00139	um00280	1.00E-159			
BBH	9d00122	um10955	5.00E-43	um10955	9d00122	5.00E-43
BBH	9c00017	um12014	1.00E-172	um12014	9c00017	1.00E-172
BBH	9c00394	um00729	1.00E-177	um00729	9c00394	1.00E-177
BBH	9d00115	um00229	2.00E-40	um00229	9d00115	4.00E-41
BBH	10d00113	um03656	1.00E-72	um03656	10d00113	2.00E-71
BBH	10c00054	um03743.2	1.00E-171	um03743.2	10c00054	1.00E-166
BBH	10d00018	um03841	0	um03841	10d00018	0
BBH	10d00096	um03692	0	um03692	10d00096	0
BBH	10d00038	um03803	0	um03803	10d00038	0
NNN	10c00077	um10488	7.00E-45			
BBH	10c00026	um03810	1.00E-133	um03810	10c00026	1.00E-133
NNN	10c00025	um03813	0			
BBH	10d00008	um03854	0	um03854	10d00008	0
NNN	10d00058	um03767	3.00E-51			
BBH	10c00062	um03730.2	0	um03730.2	10c00062	0
BBH	10d00075	um10504	1.00E-145	um10504	10d00075	1.00E-149
BBH	10c00042	um03772	0	um03772	10c00042	0
BBH	10c00105	um03640	7.00E-84	um03640	10c00105	3.00E-80
BBH	10d00024	um10511	3.00E-59	um10511	10d00024	7.00E-53
BBH	10d00031	um03821	8.00E-45	um03821	10d00031	5.00E-60
BBH	10d00053	um03776	0	um03776	10d00053	0
BBH	10c00087	um03673	1.00E-128	um03673	10c00087	1.00E-135
BBH	10d00065	um03754	1.00E-100	um03754	10d00065	5.00E-96

BBH	10c00088	um10482	5.00E-53	um10482	10c00088	6.00E-53
BBH	10c00055	um10659	0	um10659	10c00055	0
BBH	10d00010	um03851	0	um03851	10d00010	0
BBH	10d00097	um03691	0	um03691	10d00097	0
BBH	10c00036	um03791	0	um03791	10c00036	0
BBH	10d00114	um03657	7.00E-40	um03657	10d00114	1.00E-37
BBH	10c00076	um03694.2	0	um03694.2	10c00076	0
BBH	10d00098	um05979	0	um05979	10d00098	0
BBH	10d00119	um11963	1.00E-173	um11963	10d00119	0
BBH	10d00110	um03664	1.00E-163	um03664	10d00110	1.00E-174
BBH	10c00084	um03678	0	um03678	10c00084	0
BBH	10d00039	um03802	0	um03802	10d00039	0
BBH	10d00047	um03788	3.00E-34	um03788	10d00047	3.00E-34
BBH	10d00088	um03708.2	1.00E-162	um03708.2	10d00088	1.00E-161
BBH	10d00046	um03789	0	um03789	10d00046	0
BBH	10d00074	um03738	0	um03738	10d00074	0
BBH	10c00063	um03728	0	um03728	10c00063	0
BBH	10c00096	um10474.2	1.00E-134	um10474.2	10c00096	1.00E-136
BBH	10c00047	um03782	1.00E-146	um03782	10c00047	1.00E-156
BBH	10d00036	um03807	3.00E-62	um03807	10d00036	1.00E-69
BBH	10c00032	um11733.2	5.00E-40	um11733.2	10c00032	5.00E-40
BBH	10c00045	um03779	1.00E-108	um03779	10c00045	1.00E-108
BBH	10d00025	um03835	1.00E-21	um03835	10d00025	9.00E-22
NNN	10c00083	um03681.2	0			
BBH	10d00104	um03674	0	um03674	10d00104	0
BBH	10c00027	um03809	0	um03809	10c00027	0
BBH	10d00001	um03863	0	um03863	10d00001	0
BBH	10d00003	um03861	0	um03861	10d00003	0
BBH	10d00064	um03756	0	um03756	10d00064	0
BBH	10d00052	um03775	1.00E-152	um03775	10d00052	1.00E-150
BBH	10c00001	um11107	1.00E-118	um11107	10c00001	1.00E-127
BBH	10d00120	um03643.2	0	um03643.2	10d00120	0
BBH	10d00013	um03847	0	um03847	10d00013	0
BBH	10c00013	um03833	2.00E-66	um03833	10c00013	8.00E-67
BBH	10d00094	um03700	1.00E-146	um03700	10d00094	1.00E-153
BBH	10d00084	um12240	1.00E-166	um12240	10d00084	1.00E-179
NNN	10c00065	um10498	2.00E-77			
BBH	10d00077	um10502	0	um10502	10d00077	0
BBH	10c00079	um03690	0	um03690	10c00079	0
NNN	10c00070	um10492	0			
BBH	10d00021	um03838	1.00E-161	um03838	10d00021	1.00E-161
BBH	10c00102	um03649	6.00E-52	um03649	10c00102	2.00E-45
BBH	10d00068	um03746	1.00E-18	um03746	10d00068	3.00E-19
BBH	10c00052	um03758	0	um03758	10c00052	0
BBH	10d00027	um03829.2	1.00E-140	um03829.2	10d00027	1.00E-144
BBH	10c00081	um03687	6.00E-90	um03687	10c00081	2.00E-86
BBH	10c00030	um03806	3.00E-43	um03806	10c00030	3.00E-39
NNN	10d00067	um03748	7.00E-11	um03748	22c00215	9.00E-16
BBH	10d00051	um10664	0	um10664	10d00051	0
BBH	10d00033	um03815	0	um03815	10d00033	0
BBH	10c00053	um03755	1.00E-101	um03755	10c00053	4.00E-94
BBH	10d00111	um03663	0	um03663	10d00111	0
NNN	10d00078	um03729	0			
BBH	10c00046	um10665	0	um10665	10c00046	0
BBH	10c00080	um03689	1.00E-130	um03689	10c00080	1.00E-128
BBH	10c00050	um03766	1.00E-54	um03766	10c00050	7.00E-95
BBH	10c00044	um03777	0	um03777	10c00044	0
BBH	10c00028	um03808	3.00E-62	um03808	10c00028	5.00E-72
BBH	10d00102	um10484	0	um10484	10d00102	0
BBH	10d00095	um03698	1.00E-108	um03698	10d00095	1.00E-112
BBH	10d00045	um10668	0	um10668	10d00045	0
BBH	10d00056	um10663	1.00E-110	um10663	10d00056	1.00E-106

BBH	10c00064	um10499	1.00E-156	um10499	10c00064	1.00E-156
BBH	10d00085	um03714	0	um03714	10d00085	0
BBH	10c00078	um11588	7.00E-58	um11588	10c00078	1.00E-56
BBH	10d00030	um03822	7.00E-32	um03822	10d00030	8.00E-37
BBH	10c00071	um03702	0	um03702	10c00071	0
NNN	10d00123	um03110	2.00E-42	um03110	19c00005	1.00E-127
BBH	10c00082	um03685	0	um03685	10c00082	0
BBH	10d00086	um10494	0	um10494	10d00086	0
BBH	10c00066	um10497	1.00E-112	um10497	10c00066	1.00E-124
BBH	10c00012	um10509	1.00E-112	um10509	10c00012	1.00E-104
BBH	10c00101	um03652	1.00E-108	um03652	10c00101	1.00E-112
BBH	10d00012	um11104	0	um11104	10d00012	0
BBH	10c00099	um03655	5.00E-85	um03655	10c00099	2.00E-77
BBH	10d00112	um03662	0	um03662	10d00112	0
BBH	10c00095	um03661	1.00E-152	um03661	10c00095	1.00E-157
BBH	10c00086	um10483	1.00E-118	um10483	10c00086	1.00E-125
BBH	10d00009	um03852	0	um03852	10d00009	0
BBH	10c00031	um11734	1.00E-133	um11734	10c00031	1.00E-132
BBH	10d00066	um03753	3.00E-35	um03753	10d00066	8.00E-36
BBH	10c00058	um03737	0	um03737	10c00058	0
BBH	10d00050	um03770	1.00E-124	um03770	10d00050	1.00E-128
BBH	10d00076	um03734	0	um03734	10d00076	0
BBH	10d00059	um03765	0	um03765	10d00059	0
BBH	10c00051	um03761	0	um03761	10c00051	0
BBH	10d00044	um03793	0	um03793	10d00044	0
BBH	10d00101	um03682.2	0	um03682.2	10d00101	0
BBH	10c00094	um10475	1.00E-111	um10475	10c00094	1.00E-115
BBH	10c00049	um10661.2	0	um10661.2	10c00049	0
BBH	10c00020	um03820	0	um03820	10c00020	0
BBH	10c00035	um03792	0	um03792	10c00035	0
NNN	10d00100	um10487	4.00E-15	um10487	7d00279	6.00E-34
BBH	10c00007	um11106	1.00E-21	um11106	10c00007	5.00E-22
NNN	10d00122	um03638	8.00E-44			
BBH	10d00061	um03762	0	um03762	10d00061	0
BBH	10c00004	um03865	1.00E-103	um03865	10c00004	1.00E-103
BBH	10c00067	um10496	1.00E-114	um10496	10c00067	1.00E-159
NNN	10d00048	um10667	6.00E-18	um10667	10d00049	4.00E-52
BBH	10d00007	um12243	0	um12243	10d00007	0
BBH	10d00004	um03860	0	um03860	10d00004	0
BBH	10d00028	um10508	3.00E-80	um10508	10d00028	1.00E-80
BBH	10d00015	um03845	2.00E-54	um03845	10d00015	6.00E-60
BBH	10c00060	um10503	0	um10503	10c00060	0
BBH	10d00081	um03722	0	um03722	10d00081	0
NNN	10c00039	um03784	0			
BBH	10d00060	um03763	1.00E-176	um03763	10d00060	1.00E-180
BBH	10d00117	um15081	4.00E-84	um15081	10d00117	3.00E-80
NNN	10c00097	um03658	1.00E-152			
BBH	10c00093	um03665	0	um03665	10c00093	0
BBH	10c00015	um03830	0	um03830	10c00015	0
BBH	10d00035	um03811	1.00E-144	um03811	10d00035	1.00E-144
BBH	10d00082	um03720	1.00E-179	um03720	10d00082	0
BBH	10d00092	um03701	0	um03701	10d00092	0
BBH	10c00072	um03699	1.00E-162	um03699	10c00072	1.00E-160
BBH	10d00042	um03796	0	um03796	10d00042	0
BBH	10c00021	um03819	0	um03819	10c00021	0
NNN	10d00071	um12241	5.00E-13			
BBH	10c00005	um03867	1.00E-135	um03867	10c00005	1.00E-130
BBH	10c00048	um10662	5.00E-89	um10662	10c00048	3.00E-85
NNN	10c00092	um02185	7.00E-17	um02185	6c00055	5.00E-83
BBH	10d00115	um03654	2.00E-44	um03654	10d00115	5.00E-43
BBH	10c00041	um03771	1.00E-163	um03771	10c00041	1.00E-169
BBH	10d00006	um03857	0	um03857	10d00006	0

BBH	10d00026	um03832	0	um03832	10d00026	0
BBH	10c00068	um10493	1.00E-144	um10493	10c00068	1.00E-142
BBH	10d00020	um03839	0	um03839	10d00020	0
BBH	10c00061	um10500	3.00E-15	um10500	10c00061	3.00E-15
BBH	10c00008	um03853	0	um03853	10c00008	0
BBH	10d00014	um11103	0	um11103	10d00014	0
BBH	10c00104	um03645	0	um03645	10c00104	0
BBH	10d00029	um03824	1.00E-160	um03824	10d00029	1.00E-154
BBH	10d00121	um03641	1.00E-135	um03641	10d00121	1.00E-145
BBH	10d00032	um03818	1.00E-63	um03818	10d00032	4.00E-56
BBH	10d00079	um03726.2	2.00E-76	um03726.2	10d00079	1.00E-71
BBH	10c00073	um10490.2	5.00E-61	um10490.2	10c00073	4.00E-58
BBH	10d00070	um03744	1.00E-26	um03744	10d00070	5.00E-27
NNN	10d00093	um03700	1.00E-86	um03700	10d00094	1.00E-153
BBH	10d00118	um03648	0	um03648	10d00118	0
BBH	10c00014	um03831	1.00E-133	um03831	10c00014	1.00E-137
BBH	10d00083	um03718	1.00E-127	um03718	10d00083	1.00E-132
BBH	10c00018	um10507	1.00E-147	um10507	10c00018	1.00E-147
BBH	10d00063	um03759	1.00E-141	um03759	10d00063	1.00E-137
BBH	10c00011	um03843	1.00E-160	um03843	10c00011	1.00E-165
BBH	10d00002	um03866	0	um03866	10d00002	0
BBH	10c00057	um15082	1.00E-163	um15082	10c00057	1.00E-177
BBH	10d00116	um03653	1.00E-142	um03653	10d00116	1.00E-138
BBH	10d00022	um03837	0	um03837	10d00022	0
BBH	10c00002	um03862	1.00E-166	um03862	10c00002	1.00E-177
BBH	10c00023	um03816	0	um03816	10c00023	0
BBH	10d00055	um10666	0	um10666	10d00055	0
BBH	10d00072	um11964	0	um11964	10d00072	0
BBH	10d00099	um03688	1.00E-169	um03688	10d00099	1.00E-170
NNN	10c00098	um10473	6.00E-87			
BBH	10d00037	um03805	0	um03805	10d00037	0
BBH	10c00040	um03783	0	um03783	10c00040	0
BBH	10d00106	um10479	3.00E-20	um10479	10d00106	1.00E-15
NNN	10c00069	um03707	4.00E-11			
BBH	10c00103	um03647	0	um03647	10c00103	0
NNN	10d00041	um03798	3.00E-24			
BBH	10c00017	um03826	0	um03826	10c00017	0
BBH	10c00089	um10480	7.00E-35	um10480	10c00089	6.00E-42
NNN	10c00074	um03696	0			
BBH	10c00091	um12239	0	um12239	10c00091	0
BBH	10d00090	um03704	2.00E-21	um03704	10d00090	2.00E-21
BBH	10c00003	um03864	0	um03864	10c00003	0
BBH	10c00033	um15083.2	5.00E-60	um15083.2	10c00033	6.00E-69
BBH	10d00103	um03676	0	um03676	10d00103	0
BBH	10d00017	um03842	0	um03842	10d00017	0
BBH	10d00054	um03778	6.00E-55	um03778	10d00054	6.00E-59
BBH	10d00019	um15007	1.00E-175	um15007	10d00019	1.00E-168
BBH	10d00005	um03859	2.00E-79	um03859	10d00005	6.00E-91
BBH	10d00023	um10512	1.00E-112	um10512	10d00023	1.00E-102
BBH	10d00107	um10478	1.00E-101	um10478	10d00107	1.00E-101
BBH	10c00006	um03858	0	um03858	10c00006	0
BBH	10c00010	um11105	0	um11105	10c00010	0
BBH	10d00049	um10667	1.00E-50	um10667	10d00049	4.00E-52
BBH	10d00105	um10481	1.00E-53	um10481	10d00105	1.00E-53
BBH	10c00024	um03814.2	1.00E-162	um03814.2	10c00024	1.00E-165
BBH	10c00043	um03774	0	um03774	10c00043	0
BBH	10c00090	um03669	0	um03669	10c00090	0
BBH	10d00073	um03740	0	um03740	10d00073	0
BBH	10d00062	um03760	0	um03760	10d00062	0
BBH	10d00089	um03706.2	2.00E-42	um03706.2	10d00089	3.00E-49
BBH	10c00075	um03695	0	um03695	10c00075	0
BBH	10d00091	um10491	7.00E-68	um10491	10d00091	3.00E-72

NNN	10d00040	um03798	1.00E-141			
NNN	10c00016	um03828	4.00E-39			
BBH	10d00108	um10477	1.00E-77	um10477	10d00108	1.00E-71
BBH	10d00016	um03844	1.00E-174	um03844	10d00016	0
BBH	10d00011	um03850	0	um03850	10d00011	0
BBH	10c00106	um11962.2	0	um11962.2	10c00106	0
NNN	10c00034	um10669	1.00E-157			
BBH	10d00080	um03724	1.00E-164	um03724	10d00080	1.00E-172
BBH	10c00038	um12242.2	0	um12242.2	10c00038	0
BBH	10d00034	um03812	1.00E-160	um03812	10d00034	1.00E-167
BBH	11c00036	um04378	0	um04378	11c00036	0
BBH	11c00014	um04332	9.00E-93	um04332	11c00014	2.00E-92
BBH	11d00041	um11153	8.00E-31	um11153	11d00041	1.00E-30
BBH	11d00071	um10538	1.00E-167	um10538	11d00071	1.00E-167
BBH	11c00064	um04421	9.00E-38	um04421	11c00064	6.00E-46
BBH	11d00018	um04343.2	4.00E-91	um04343.2	11d00018	8.00E-94
BBH	11c00068	um04428	4.00E-72	um04428	11c00068	9.00E-58
BBH	11d00060	um04441	1.00E-157	um04441	11d00060	1.00E-152
BBH	11c00041	um12062	1.00E-94	um12062	11c00041	1.00E-94
BBH	11c00007	um04320	1.00E-176	um04320	11c00007	0
BBH	11c00086	um04472	7.00E-49	um04472	11c00086	1.00E-47
BBH	11c00020	um04345	1.00E-41	um04345	11c00020	4.00E-42
BBH	11d00077	um04468	0	um04468	11d00077	0
BBH	11d00053	um10522	1.00E-108	um10522	11d00053	2.00E-90
BBH	11d00081	um04473	0	um04473	11d00081	0
BBH	11c00089	um04485	1.00E-145	um04485	11c00089	1.00E-145
BBH	11c00009	um04323	0	um04323	11c00009	0
BBH	11c00037	um04383	1.00E-103	um04383	11c00037	1.00E-103
BBH	11c00056	um04407	0	um04407	11c00056	0
NNN	11c00052	um04402	0			
NNN	11d00009	um11750	0			
BBH	11c00065	um04422	1.00E-136	um04422	11c00065	1.00E-133
BBH	11c00049	um04398	0	um04398	11c00049	0
BBH	11d00038	um11152	1.00E-168	um11152	11d00038	1.00E-174
BBH	11c00013	um04329.2	4.00E-83	um04329.2	11c00013	3.00E-79
BBH	11d00042	um12061	2.00E-22	um12061	11d00042	6.00E-27
BBH	11d00026	um04358	0	um04358	11d00026	0
BBH	11d00047	um04406	1.00E-155	um04406	11d00047	1.00E-156
BBH	11d00049	um10519	1.00E-114	um10519	11d00049	1.00E-118
BBH	11c00032	um04374	0	um04374	11c00032	0
BBH	11c00042	um04389	0	um04389	11c00042	0
BBH	11c00069	um10524	0	um10524	11c00069	0
BBH	11d00006	um04318	0	um04318	11d00006	0
BBH	11d00051	um04417	0	um04417	11d00051	0
BBH	11c00087	um04477	0	um04477	11c00087	0
BBH	11c00088	um04479	0	um04479	11c00088	0
BBH	11d00054	um04423	0	um04423	11d00054	0
BBH	11d00084	um11977	1.00E-109	um11977	11d00084	9.00E-96
BBH	11c00043	um04390	3.00E-58	um04390	11c00043	7.00E-55
BBH	11d00040	um04382	0	um04382	11d00040	0
BBH	11c00053	um12265.2	0	um12265.2	11c00053	0
BBH	11c00001	um02818	1.00E-147	um02818	11c00001	1.00E-130
BBH	11c00021	um04347	0	um04347	11c00021	0
BBH	11c00038	um04384	2.00E-29	um04384	11c00038	5.00E-28
BBH	11d00089	um04484	0	um04484	11d00089	0
BBH	11c00062	um10520	9.00E-79	um10520	11c00062	1.00E-78
BBH	11d00066	um10531	1.00E-48	um10531	11d00066	5.00E-53
BBH	11d00032	um11149	0	um11149	11d00032	0
BBH	11d00030	um11148	1.00E-142	um11148	11d00030	1.00E-141
BBH	11c00030	um05550	0	um05550	11c00030	0
BBH	11d00019	um04344	0	um04344	11d00019	0
BBH	11d00016	um12260	1.00E-74	um12260	11d00016	6.00E-83

BBH	11d00005	um04317	2.00E-65	um04317	11d00005	3.00E-57
BBH	11d00074	um04462	1.00E-131	um04462	11d00074	1.00E-136
BBH	11d00043	um10513	0	um10513	11d00043	0
NNN	11c00071	um10526	0			
BBH	11d00037	um04371	0	um04371	11d00037	0
BBH	11d00025	um04356	0	um04356	11d00025	0
BBH	11c00081	um04456	0	um04456	11c00081	0
BBH	11d00039	um04381	0	um04381	11d00039	0
BBH	11c00048	um04397	0	um04397	11c00048	0
BBH	11c00063	um04419	0	um04419	11c00063	0
BBH	11d00067	um04451	0	um04451	11d00067	0
BBH	11c00054	um04404	1.00E-127	um04404	11c00054	1.00E-122
BBH	11d00017	um11142	0	um11142	11d00017	0
BBH	11c00058	um04411	0	um04411	11c00058	0
BBH	11c00005	um04314	4.00E-57	um04314	11c00005	6.00E-57
BBH	11d00056	um04426	1.00E-147	um04426	11d00056	1.00E-150
BBH	11d00083	um04475	0	um04475	11d00083	0
BBH	11c00022	um04348	0	um04348	11c00022	0
NNN	11c00012	um10843	3.00E-78			
NNN	11d00029	um11148	2.00E-70	um11148	11d00030	1.00E-141
BBH	11d00015	um10847	2.00E-66	um10847	11d00015	2.00E-66
BBH	11d00073	um04461	0	um04461	11d00073	0
BBH	11d00031	um04365	0	um04365	11d00031	0
NNN	11c00028	um12262	1.00E-21	um12262	11c00027	0
BBH	11c00031	um11150	0	um11150	11c00031	0
NNN	11c00019	um04342	1.00E-74			
BBH	11d00044	um04392	1.00E-148	um04392	11d00044	1.00E-139
BBH	11d00003	um04313	6.00E-72	um04313	11d00003	5.00E-72
BBH	11d00068	um10534	0	um10534	11d00068	0
BBH	11c00055	um04405	0	um04405	11c00055	0
BBH	11c00060	um04414	5.00E-85	um04414	11c00060	1.00E-90
BBH	11c00059	um04413	0	um04413	11c00059	0
BBH	11c00044	um04391	0	um04391	11c00044	0
BBH	11c00006	um04319	0	um04319	11c00006	0
BBH	11c00011	um04327	0	um04327	11c00011	0
BBH	11d00028	um04361	1.00E-180	um04361	11d00028	0
BBH	11c00023	um04351	0	um04351	11c00023	0
BBH	11c00004	um12259	0	um12259	11c00004	0
BBH	11d00086	um04481	0	um04481	11d00086	0
BBH	11c00035	um04370	0	um04370	11c00035	0
BBH	11d00061	um04443	0	um04443	11d00061	0
BBH	11c00029	um04362	1.00E-159	um04362	11c00029	1.00E-159
BBH	11c00024	um04352	1.00E-122	um04352	11c00024	1.00E-122
BBH	11d00002	um04310	0	um04310	11d00002	0
BBH	11c00010	um11751.2	0	um11751.2	11c00010	0
BBH	11d00027	um11147	0	um11147	11d00027	0
BBH	11d00058	um10527.2	1.00E-123	um10527.2	11d00058	1.00E-133
BBH	11d00034	um04376	0	um04376	11d00034	0
BBH	11d00064	um04447	2.00E-69	um04447	11d00064	7.00E-73
NNN	11c00072	um10528	0			
BBH	11c00018	um11141	1.00E-101	um11141	11c00018	1.00E-105
BBH	11c00045	um04393	5.00E-89	um04393	11c00045	2.00E-91
BBH	11d00020	um04346	0	um04346	11d00020	0
NNN	11d00022	um04461	5.00E-54	um04461	11d00073	0
BBH	11d00076	um04465	1.00E-129	um04465	11d00076	1.00E-127
BBH	11d00014	um04335	0	um04335	11d00014	0
BBH	11d00046	um04400	0	um04400	11d00046	0
BBH	11c00080	um10535	2.00E-29	um10535	11c00080	8.00E-24
BBH	11d00085	um04478	0	um04478	11d00085	0
BBH	11d00062	um04444	1.00E-146	um04444	11d00062	1.00E-135
BBH	11c00017	um10848	1.00E-168	um10848	11c00017	1.00E-169
BBH	11c00025	um04353	0	um04353	11c00025	0

BBH	11c00070	um04433	0	um04433	11c00070	0
BBH	11d00080	um04471	5.00E-47	um04471	11d00080	6.00E-53
BBH	11c00082	um10539	1.00E-174	um10539	11c00082	1.00E-178
BBH	11c00061	um04416	0	um04416	11c00061	0
BBH	11c00046	um04394	0	um04394	11c00046	0
BBH	11d00021	um04350	0	um04350	11d00021	0
BBH	11d00013	um10845	5.00E-82	um10845	11d00013	2.00E-82
BBH	11d00075	um04464	0	um04464	11d00075	0
BBH	11c00083	um04463	1.00E-158	um04463	11c00083	1.00E-154
BBH	11d00033	um04368	1.00E-122	um04368	11d00033	1.00E-109
BBH	11d00055	um10523	3.00E-90	um10523	11d00055	9.00E-86
BBH	11c00077	um04448	2.00E-69	um04448	11c00077	1.00E-68
BBH	11c00057	um04410	0	um04410	11c00057	0
BBH	11c00050	um04399	1.00E-150	um04399	11c00050	1.00E-150
BBH	11d00090	um11776	1.00E-149	um11776	11d00090	1.00E-152
BBH	11c00016	um10846	1.00E-178	um10846	11c00016	1.00E-178
BBH	11c00033	um11151	1.00E-120	um11151	11c00033	1.00E-116
BBH	11d00063	um12267	0	um12267	11d00063	0
BBH	11c00047	um01038	1.00E-148	um01038	11c00047	1.00E-153
BBH	11d00052	um04420	1.00E-113	um04420	11d00052	1.00E-119
NNN	11c00002	um01877	3.00E-13	um01877	22c00240	0
BBH	11d00088	um11978	1.00E-77	um11978	11d00088	3.00E-68
BBH	11c00074	um04442	0	um04442	11c00074	0
BBH	11d00070	um04457	2.00E-91	um04457	11d00070	2.00E-91
BBH	11c00078	um10532	0	um10532	11c00078	0
BBH	11c00039	um04385	1.00E-108	um04385	11c00039	1.00E-113
BBH	11c00034	um04372	0	um04372	11c00034	0
BBH	11d00069	um10536	1.00E-42	um10536	11d00069	5.00E-38
BBH	11c00084	um04466	1.00E-106	um04466	11c00084	1.00E-106
BBH	11d00012	um04330	7.00E-74	um04330	11d00012	2.00E-83
BBH	11c00051	um04401	0	um04401	11c00051	0
BBH	11c00066	um04424	1.00E-16	um04424	11c00066	2.00E-19
BBH	11d00024	um04355	0	um04355	11d00024	0
BBH	11d00057	um04429	0	um04429	11d00057	0
BBH	11d00082	um04474	0	um04474	11d00082	0
BBH	11c00079	um10533	0	um10533	11c00079	0
BBH	11d00087	um04482	0	um04482	11d00087	0
BBH	11d00072	um04460	1.00E-68	um04460	11d00072	2.00E-71
BBH	11d00036	um12263	2.00E-32	um12263	11d00036	3.00E-34
BBH	11c00027	um12262	0	um12262	11c00027	0
BBH	11c00015	um04333	0	um04333	11c00015	0
NNN	11c00003	um00822	2.00E-14	um00822	19c00004	1.00E-177
BBH	11d00023	um04354	0	um04354	11d00023	0
BBH	11d00045	um10514	2.00E-34	um10514	11d00045	3.00E-31
BBH	11d00011	um11753	1.00E-31	um11753	11d00011	2.00E-26
BBH	11c00075	um04446	2.00E-36	um04446	11c00075	5.00E-34
BBH	11c00085	um04470	0	um04470	11c00085	0
BBH	11c00026	um04357	0	um04357	11c00026	0
BBH	11d00035	um04375	0	um04375	11d00035	0
BBH	11d00050	um04415	1.00E-134	um04415	11d00050	1.00E-134
BBH	11d00078	um10541	0	um10541	11d00078	0
BBH	11c00067	um04427	5.00E-31	um04427	11c00067	4.00E-35
BBH	11d00007	um11749	0	um11749	11d00007	0
BBH	12d00088	um02380	1.00E-47	um02380	12d00088	1.00E-56
BBH	12c00090	um02381	0	um02381	12c00090	0
BBH	12d00045	um01335	0	um01335	12d00045	0
BBH	12d00057	um01310	0	um01310	12d00057	0
BBH	12c00022	um01381	1.00E-63	um01381	12c00022	2.00E-76
BBH	12d00059	um01307	7.00E-98	um01307	12d00059	1.00E-102
BBH	12d00020	um11323	1.00E-152	um11323	12d00020	1.00E-152
BBH	12d00016	um11330	1.00E-108	um11330	12d00016	1.00E-104
BBH	12c00112	um02446	0	um02446	12c00112	0

BBH	12c00077	um01274	0	um01274	12c00077	0
BBH	12c00050	um01334	4.00E-25	um01334	12c00050	2.00E-25
BBH	12c00144	um02809	1.00E-109	um02809	12c00144	1.00E-104
BBH	12d00093	um02433.2	1.00E-161	um02433.2	12d00093	1.00E-169
BBH	12d00137	um02504	0	um02504	12d00137	0
BBH	12c00046	um01341	0	um01341	12c00046	0
BBH	12d00013	um11333	1.00E-166	um11333	12d00013	1.00E-166
NNN	12d00124	um02473	4.00E-12	um02473	12d00091	3.00E-15
BBH	12c00036	um11313	1.00E-164	um11313	12c00036	1.00E-161
BBH	12d00031	um01363	3.00E-94	um01363	12d00031	6.00E-99
BBH	12d00077	um01270	0	um01270	12d00077	0
BBH	12c00029	um01370.2	3.00E-34	um01370.2	12c00029	4.00E-34
BBH	12c00044	um10244	1.00E-90	um10244	12c00044	5.00E-98
BBH	12c00110	um02450	4.00E-60	um02450	12c00110	2.00E-60
BBH	12c00131	um02472	2.00E-87	um02472	12c00131	1.00E-115
BBH	12d00065	um01289	0	um01289	12d00065	0
BBH	12c00017	um11325	1.00E-150	um11325	12c00017	1.00E-139
BBH	12d00070	um10227	2.00E-64	um10227	12d00070	2.00E-64
BBH	12c00016	um11327	0	um11327	12c00016	0
BBH	12d00106	um02415	2.00E-42	um02415	12d00106	6.00E-44
BBH	12d00117	um10165	1.00E-130	um10165	12d00117	1.00E-128
BBH	12c00098	um02439	0	um02439	12c00098	0
BBH	12c00083	um02378	0	um02378	12c00083	0
BBH	12c00122	um02483	1.00E-96	um02483	12c00122	1.00E-135
BBH	12d00087	um02375	0	um02375	12d00087	0
BBH	12c00097	um02436	0	um02436	12c00097	0
BBH	12c00067	um01295	1.00E-79	um01295	12c00067	4.00E-83
BBH	12d00139	um04557	5.00E-22	um04557	12d00139	1.00E-20
BBH	12d00138	um05617	1.00E-125	um05617	12d00138	1.00E-125
NNN	12d00014	um01408	1.00E-179			
BBH	12c00086	um02383	5.00E-58	um02383	12c00086	1.00E-36
BBH	12c00013	um01405	0	um01405	12c00013	0
BBH	12c00111	um10160	0	um10160	12c00111	0
NNN	12d00094	um02293	4.00E-50	um02293	12c00084	8.00E-53
BBH	12d00028	um01374	1.00E-140	um01374	12d00028	1.00E-144
NNN	12d00030	um11315	2.00E-11			
BBH	12d00046	um01332	1.00E-141	um01332	12d00046	1.00E-141
BBH	12d00032	um01359	0	um01359	12d00032	0
BBH	12c00041	um01351	2.00E-30	um01351	12c00041	3.00E-28
BBH	12c00107	um02419	1.00E-105	um02419	12c00107	1.00E-108
BBH	12c00137	um02493	0	um02493	12c00137	0
BBH	12c00132	um10162	7.00E-44	um10162	12c00132	2.00E-35
BBH	12c00079	um12130	1.00E-40	um12130	12c00079	1.00E-40
BBH	12c00027	um01373.2	1.00E-124	um01373.2	12c00027	1.00E-127
NNN	12d00141	um04667	2.00E-41	um04667	27d00089	1.00E-134
BBH	12c00121	um02460	7.00E-93	um02460	12c00121	2.00E-92
NNN	12c00104	um02425	2.00E-53	um02425	12c00103	1.00E-116
BBH	12d00021	um11322	0	um11322	12d00021	0
BBH	12c00130	um02475	1.00E-41	um02475	12c00130	6.00E-38
BBH	12c00128	um10163	8.00E-86	um10163	12c00128	3.00E-86
BBH	12c00055	um01322	0	um01322	12c00055	0
BBH	12d00089	um02388	9.00E-60	um02388	12d00089	2.00E-64
BBH	12c00020	um11319	1.00E-150	um11319	12c00020	1.00E-157
BBH	12d00118	um02487	0	um02487	12d00118	0
BBH	12d00105	um10152	0	um10152	12d00105	0
BBH	12d00135	um02502	0	um02502	12d00135	0
BBH	12c00142	um02499	1.00E-172	um02499	12c00142	1.00E-177
BBH	12d00126	um02469	1.00E-37	um02469	12d00126	3.00E-36
NNN	12c00028	um01372	1.00E-149			
BBH	12d00075	um11855.2	0	um11855.2	12d00075	0
BBH	12d00036	um15038	1.00E-136	um15038	12d00036	1.00E-139
BBH	12d00130	um02464	0	um02464	12d00130	0

BBH	12c00118	um02456	0	um02456	12c00118	0
BBH	12d00114	um02454	1.00E-81	um02454	12d00114	4.00E-83
BBH	12c00048	um10242	6.00E-13	um10242	12c00048	2.00E-16
BBH	12c00019	um01388	1.00E-116	um01388	12c00019	1.00E-112
BBH	12c00085	um10139	1.00E-155	um10139	12c00085	1.00E-148
BBH	12d00123	um12225	6.00E-12	um12225	12d00123	1.00E-11
BBH	12c00056	um10237	0	um10237	12c00056	0
BBH	12c00138	um02495	0	um02495	12c00138	0
BBH	12d00115	um02457	1.00E-140	um02457	12d00115	1.00E-150
BBH	12d00081	um02377	1.00E-180	um02377	12d00081	0
BBH	12d00080	um10146	1.00E-177	um10146	12d00080	1.00E-177
BBH	12c00014	um01403	2.00E-37	um01403	12c00014	2.00E-39
BBH	12c00080	um11864	0	um11864	12c00080	0
BBH	12c00068	um10231	6.00E-95	um10231	12c00068	1.00E-101
BBH	12d00024	um11318	1.00E-82	um11318	12d00024	1.00E-82
BBH	12c00115	um10157	0	um10157	12c00115	0
BBH	12c00125	um02462	0	um02462	12c00125	0
BBH	12d00037	um01350	1.00E-156	um01350	12d00037	0
BBH	12d00056	um01313	3.00E-75	um01313	12d00056	1.00E-78
NNN	12d00063	um01297	2.00E-32			
BBH	12c00004	um01426	6.00E-59	um01426	12c00004	8.00E-59
BBH	12c00039	um11308	0	um11308	12c00039	0
BBH	12d00091	um02473	2.00E-15	um02473	12d00091	3.00E-15
BBH	12c00074	um12131	9.00E-74	um12131	12c00074	8.00E-74
BBH	12c00105	um02422	0	um02422	12c00105	0
BBH	12c00032	um01364	1.00E-100	um01364	12c00032	1.00E-111
BBH	12d00018	um11326	8.00E-92	um11326	12d00018	2.00E-96
BBH	12c00053	um01329	0	um01329	12c00053	0
BBH	12d00004	um01425	0	um01425	12d00004	0
BBH	12c00091	um02385	0	um02385	12c00091	0
BBH	12c00120	um02459	0	um02459	12c00120	0
BBH	12d00066	um10228.2	5.00E-34	um10228.2	12d00066	7.00E-45
BBH	12d00125	um02471	0	um02471	12d00125	0
BBH	12c00103	um02425	1.00E-116	um02425	12c00103	1.00E-116
BBH	12d00015	um01407	0	um01407	12d00015	0
BBH	12c00099	um12190	3.00E-32	um12190	12c00099	1.00E-32
BBH	12c00065	um01296	0	um01296	12c00065	0
BBH	12d00048	um01328	0	um01328	12d00048	0
BBH	12d00022	um01391	1.00E-126	um01391	12d00022	1.00E-126
BBH	12c00117	um12191	1.00E-23	um12191	12c00117	2.00E-23
BBH	12d00078	um02411	0	um02411	12d00078	0
BBH	12d00110	um10159	0	um10159	12d00110	0
BBH	12d00082	um02376	0	um02376	12d00082	0
BBH	12c00033	um01362	1.00E-179	um01362	12c00033	0
BBH	12c00045	um10243	1.00E-122	um10243	12c00045	1.00E-127
BBH	12d00116	um02482	2.00E-96	um02482	12d00116	4.00E-96
BBH	12d00092	um02431	0	um02431	12d00092	0
BBH	12c00135	um02490	0	um02490	12c00135	0
BBH	12d00003	um01429	1.00E-151	um01429	12d00003	1.00E-139
BBH	12c00109	um02414	0	um02414	12c00109	0
BBH	12c00106	um02421	0	um02421	12c00106	0
BBH	12d00023	um01387	2.00E-43	um01387	12d00023	1.00E-43
BBH	12c00031	um01366	1.00E-121	um01366	12c00031	1.00E-121
BBH	12d00055	um01314	0	um01314	12d00055	0
BBH	12d00100	um02440	3.00E-63	um02440	12d00100	3.00E-63
BBH	12d00038	um15080	2.00E-22	um15080	12d00038	1.00E-22
BBH	12c00003	um01427	4.00E-86	um01427	12c00003	2.00E-86
BBH	12c00073	um01284	0	um01284	12c00073	0
BBH	12d00112	um10158	0	um10158	12d00112	0
BBH	12d00073	um12129.2	3.00E-26	um12129.2	12d00073	3.00E-26
BBH	12d00006	um01424	0	um01424	12d00006	0
BBH	12d00025	um01383	0	um01383	12d00025	0

BBH	12c00009	um01414	1.00E-74	um01414	12c00009	8.00E-73
BBH	12d00054	um01316	1.00E-66	um01316	12d00054	2.00E-69
BBH	12d00039	um10246	2.00E-76	um10246	12d00039	1.00E-86
BBH	12c00011	um11331.2	1.00E-31	um11331.2	12c00011	4.00E-33
BBH	12c00054	um01323	2.00E-94	um01323	12c00054	1.00E-80
NNN	12d00062	um10233	7.00E-21			
BBH	12c00119	um02458	1.00E-143	um02458	12c00119	1.00E-147
BBH	12c00123	um02485	4.00E-94	um02485	12c00123	4.00E-94
BBH	12c00136	um10167	1.00E-158	um10167	12c00136	1.00E-163
BBH	12c00076	um10226	5.00E-80	um10226	12c00076	5.00E-80
BBH	12d00102	um10153	1.00E-123	um10153	12d00102	1.00E-129
BBH	12d00119	um02463	1.00E-156	um02463	12d00119	1.00E-148
NNN	12c00066	um01296	1.00E-108	um01296	12c00065	0
BBH	12c00082	um02407	0	um02407	12c00082	0
NNN	12c00002	um04441	4.00E-15	um04441	11d00060	1.00E-152
BBH	12c00059	um01312	0	um01312	12c00059	0
BBH	12d00012	um12136	0	um12136	12d00012	0
NNN	12c00147	um04885	4.00E-13	um04885	26c00001	0
BBH	12c00008	um11334	1.00E-166	um11334	12c00008	1.00E-170
BBH	12c00063	um01304	0	um01304	12c00063	0
BBH	12d00097	um02435	0	um02435	12d00097	0
BBH	12d00041	um12135	1.00E-124	um12135	12d00041	1.00E-119
BBH	12c00025	um01377	1.00E-106	um01377	12c00025	1.00E-105
NNN	12d00108	um02451	3.00E-12			
BBH	12d00120	um10164	4.00E-54	um10164	12d00120	1.00E-54
BBH	12d00133	um02494	1.00E-108	um02494	12d00133	1.00E-112
BBH	12d00019	um15039	2.00E-32	um15039	12d00019	2.00E-64
BBH	12c00094	um02430	4.00E-59	um02430	12c00094	1.00E-73
BBH	12d00074	um01279	0	um01279	12d00074	0
BBH	12c00140	um02498	0	um02498	12c00140	0
BBH	12d00136	um02500	0	um02500	12d00136	0
BBH	12c00129	um02477	3.00E-61	um02477	12c00129	9.00E-63
BBH	12d00035	um11309	4.00E-94	um11309	12d00035	1.00E-100
BBH	12c00075	um01282	0	um01282	12c00075	0
BBH	12c00133	um10161	0	um10161	12c00133	1.00E-143
BBH	12d00017	um01402	0	um01402	12d00017	0
BBH	12d00029	um01371	0	um01371	12d00029	0
BBH	12d00061	um10234.2	0	um10234.2	12d00061	0
BBH	12c00126	um02461	0	um02461	12c00126	0
NNN	12d00005	um06256	4.00E-73	um06256	24c00065	0
BBH	12d00076	um11863	1.00E-141	um11863	12d00076	1.00E-145
NNN	12c00001	um03113	0	um03113	19c00003	0
BBH	12c00023	um01379	9.00E-91	um01379	12c00023	5.00E-94
BBH	12d00085	um10140	4.00E-18	um10140	12d00085	5.00E-18
BBH	12d00053	um10236	3.00E-53	um10236	12d00053	1.00E-53
BBH	12d00010	um01417	1.00E-157	um01417	12d00010	1.00E-170
BBH	12d00101	um02426	4.00E-80	um02426	12d00101	8.00E-78
BBH	12c00081	um10147	1.00E-62	um10147	12c00081	2.00E-62
BBH	12c00012	um01406	1.00E-129	um01406	12c00012	1.00E-142
BBH	12d00084	um02384	4.00E-42	um02384	12d00084	2.00E-41
BBH	12c00051	um01333	1.00E-71	um01333	12c00051	1.00E-71
BBH	12d00042	um01340	2.00E-65	um01340	12d00042	2.00E-65
BBH	12d00064	um01293	0	um01293	12d00064	0
NNN	12c00064	um01301	6.00E-15	um01301	8c00026	4.00E-18
BBH	12c00007	um11335	1.00E-48	um11335	12c00007	2.00E-46
BBH	12d00113	um02452	6.00E-65	um02452	12d00113	2.00E-81
BBH	12d00090	um02429	1.00E-167	um02429	12d00090	1.00E-173
BBH	12c00141	um02501	0	um02501	12c00141	0
BBH	12c00093	um12189	0	um12189	12c00093	0
BBH	12c00092	um02387	0	um02387	12c00092	0
BBH	12d00099	um02438	3.00E-93	um02438	12d00099	1.00E-97
BBH	12d00098	um02437	8.00E-58	um02437	12d00098	2.00E-64

BBH	12c00116	um02453	6.00E-86	um02453	12c00116	1.00E-85
BBH	12c00047	um12134	4.00E-89	um12134	12c00047	1.00E-105
BBH	12c00100	um02442	1.00E-172	um02442	12c00100	1.00E-165
BBH	12d00040	um01344	0	um01344	12d00040	0
BBH	12d00134	um02496	0	um02496	12d00134	0
BBH	12d00104	um02420	1.00E-100	um02420	12d00104	1.00E-105
BBH	12c00070	um10229	1.00E-96	um10229	12c00070	6.00E-96
BBH	12c00084	um02293	3.00E-53	um02293	12c00084	8.00E-53
BBH	12d00127	um02468	1.00E-170	um02468	12d00127	1.00E-176
BBH	12c00102	um02427	0	um02427	12c00102	0
BBH	12d00086	um02374	0	um02374	12d00086	0
BBH	12d00109	um02449	1.00E-50	um02449	12d00109	2.00E-51
BBH	12d00050	um10238	6.00E-93	um10238	12d00050	4.00E-85
BBH	12c00038	um11310	2.00E-99	um11310	12c00038	5.00E-88
BBH	12d00052	um12132	1.00E-55	um12132	12d00052	5.00E-50
BBH	12d00027	um11316	1.00E-170	um11316	12d00027	1.00E-172
BBH	12d00060	um01305	0	um01305	12d00060	0
BBH	12c00024	um01378	0	um01378	12c00024	0
BBH	12d00067	um01285	2.00E-46	um01285	12d00067	1.00E-42
BBH	12c00134	um02465	0	um02465	12c00134	0
BBH	12c00052	um01331.2	0	um01331.2	12c00052	0
BBH	12d00131	um02489	0	um02489	12d00131	0
BBH	12c00096	um10155	1.00E-162	um10155	12c00096	1.00E-170
BBH	12d00122	um02476	1.00E-116	um02476	12d00122	0
BBH	12d00043	um01338	0	um01338	12d00043	0
BBH	12d00072	um01273	0	um01273	12d00072	0
NNN	12c00145	um02809	2.00E-35	um02809	12c00144	1.00E-104
BBH	12d00008	um11336	4.00E-88	um11336	12d00008	8.00E-83
NNN	12c00114	um02444	5.00E-48	um02444	12c00113	1.00E-114
BBH	12c00101	um10156	0	um10156	12c00101	0
BBH	12c00006	um01421	3.00E-72	um01421	12c00006	6.00E-71
BBH	12d00033	um01357	1.00E-39	um01357	12d00033	2.00E-39
BBH	12d00009	um01418	2.00E-91	um01418	12d00009	1.00E-101
BBH	12c00042	um11871	0	um11871	12c00042	0
BBH	12c00108	um02416	1.00E-11	um02416	12c00108	5.00E-18
BBH	12c00061	um01308	7.00E-36	um01308	12c00061	3.00E-31
BBH	12c00139	um02497	8.00E-15	um02497	12c00139	1.00E-19
BBH	12c00037	um11312	1.00E-64	um11312	12c00037	8.00E-65
NNN	12c00078	um01271	1.00E-146			
BBH	12c00113	um02444	1.00E-115	um02444	12c00113	1.00E-114
BBH	12d00026	um11317	1.00E-118	um11317	12d00026	1.00E-116
BBH	12c00030	um01367	0	um01367	12c00030	0
BBH	12c00049	um10241	0	um10241	12c00049	0
BBH	12c00010	um01411.2	1.00E-131	um01411.2	12c00010	1.00E-115
BBH	12d00049	um01327	0	um01327	12d00049	0
BBH	12d00103	um02423	3.00E-84	um02423	12d00103	4.00E-93
BBH	12d00058	um01309	1.00E-154	um01309	12d00058	1.00E-135
BBH	12d00044	um12133	4.00E-23	um12133	12d00044	4.00E-37
BBH	12c00058	um10235	0	um10235	12c00058	0
BBH	12c00127	um02481	0	um02481	12c00127	0
BBH	12d00007	um11337.2	1.00E-148	um11337.2	12d00007	1.00E-152
BBH	12d00111	um02445	0	um02445	12d00111	0
BBH	12d00051	um01321	0	um01321	12d00051	0
BBH	12c00060	um01311	2.00E-63	um01311	12c00060	5.00E-63
NNN	12c00146	um05411	6.00E-80	um05411	15d00014	0
NNN	12c00095	um10154	0			
BBH	12c00062	um01306	0	um01306	12c00062	0
BBH	12c00015	um11328	6.00E-80	um11328	12c00015	7.00E-87
BBH	12c00057	um01318	4.00E-63	um01318	12c00057	8.00E-63
BBH	12c00018	um01397	1.00E-128	um01397	12c00018	1.00E-135
BBH	12d00011	um01415	1.00E-139	um01415	12d00011	1.00E-139
BBH	12d00132	um02492	0	um02492	12d00132	0

BBH	12d00071	um01275	1.00E-108	um01275	12d00071	1.00E-109
BBH	12d00121	um02478	1.00E-170	um02478	12d00121	1.00E-167
BBH	12d00034	um01355	1.00E-119	um01355	12d00034	1.00E-119
BBH	12d00069	um01278	2.00E-89	um01278	12d00069	2.00E-90
BBH	12c00072	um01286	0	um01286	12c00072	0
BBH	12c00124	um10166	0	um10166	12c00124	1.00E-179
NNN	12c00005	um01422	1.00E-116			
BBH	12c00021	um01382	1.00E-175	um01382	12c00021	1.00E-172
BBH	12c00089	um02379	0	um02379	12c00089	0
BBH	13d00090	um04050	0	um04050	13d00090	0
BBH	13d00034	um03938	1.00E-52	um03938	13d00034	6.00E-50
BBH	13c00022	um11289	1.00E-118	um11289	13c00022	1.00E-115
BBH	13d00088	um10578	0	um10578	13d00088	0
BBH	13c00064	um03995	0	um03995	13c00064	0
NNN	13d00108	um10608	2.00E-84	um10608	26d00102	0
BBH	13d00065	um03990	1.00E-143	um03990	13d00065	1.00E-143
BBH	13d00040	um03945	0	um03945	13d00040	0
BBH	13c00037	um03924	1.00E-122	um03924	13c00037	1.00E-122
BBH	13c00029	um03939	2.00E-68	um03939	13c00029	2.00E-69
BBH	13c00108	um04072	6.00E-26	um04072	13c00108	4.00E-31
BBH	13d00059	um03991	1.00E-122	um03991	13d00059	1.00E-148
BBH	13c00011	um03874	0	um03874	13c00011	0
BBH	13d00100	um04066	5.00E-37	um04066	13d00100	2.00E-30
BBH	13c00028	um03941	2.00E-91	um03941	13c00028	2.00E-84
BBH	13c00099	um04049	7.00E-92	um04049	13c00099	1.00E-93
BBH	13d00054	um03975	0	um03975	13d00054	0
BBH	13c00003	um03871	1.00E-145	um03871	13c00003	1.00E-160
BBH	13c00043	um11521	1.00E-145	um11521	13c00043	1.00E-150
BBH	13c00074	um03984	0	um03984	13c00074	0
BBH	13d00017	um03901	0	um03901	13d00017	0
BBH	13c00087	um04015	1.00E-147	um04015	13c00087	1.00E-158
BBH	13d00039	um03925	1.00E-129	um03925	13d00039	1.00E-165
BBH	13c00100	um04051	3.00E-71	um04051	13c00100	2.00E-70
BBH	13d00089	um04047	8.00E-97	um04047	13d00089	1.00E-94
BBH	13c00054	um03974	0	um03974	13c00054	0
BBH	13d00024	um15070	0	um15070	13d00024	0
BBH	13d00060	um11740.2	0	um11740.2	13d00060	0
BBH	13c00097	um04046	0	um04046	13c00097	0
BBH	13d00107	um04076	6.00E-65	um04076	13d00107	7.00E-65
BBH	13c00081	um04022	0	um04022	13c00081	0
BBH	13d00033	um12246	1.00E-112	um12246	13d00033	1.00E-116
BBH	13d00008	um03883	0	um03883	13d00008	0
BBH	13d00070	um03980	0	um03980	13d00070	0
BBH	13d00080	um11057	0	um11057	13d00080	0
BBH	13c00021	um03911	1.00E-142	um03911	13c00021	1.00E-142
BBH	13d00048	um03963	0	um03963	13d00048	0
BBH	13d00001	um11514	0	um11514	13d00001	0
NNN	13d00098	um00372	8.00E-70	um00372	9d00180	0
BBH	13d00018	um11968	1.00E-169	um11968	13d00018	1.00E-178
BBH	13d00079	um11056	1.00E-153	um11056	13d00079	1.00E-153
BBH	13c00065	um03997	0	um03997	13c00065	0
BBH	13d00097	um04062	0	um04062	13d00097	1.00E-160
BBH	13c00107	um11635	2.00E-86	um11635	13c00107	8.00E-87
BBH	13d00011	um11509	1.00E-78	um11509	13d00011	7.00E-83
BBH	13c00004	um03873	1.00E-118	um03873	13c00004	1.00E-118
BBH	13d00016	um11966.2	0	um11966.2	13d00016	0
BBH	13c00012	um03897	2.00E-92	um03897	13c00012	1.00E-92
BBH	13c00041	um03952	1.00E-128	um03952	13c00041	1.00E-132
BBH	13c00027	um03943	1.00E-99	um03943	13c00027	2.00E-99
BBH	13d00051	um11738	0	um11738	13d00051	0
BBH	13d00025	um11288	5.00E-46	um11288	13d00025	9.00E-47
BBH	13d00015	um03898	0	um03898	13d00015	0

BBH	13d00042	um03949	0	um03949	13d00042	0
BBH	13c00008	um11511	2.00E-44	um11511	13c00008	5.00E-40
BBH	13d00101	um04067	1.00E-156	um04067	13d00101	1.00E-160
BBH	13d00003	um03872	3.00E-74	um03872	13d00003	1.00E-77
BBH	13c00053	um03972	1.00E-175	um03972	13c00053	1.00E-167
BBH	13d00036	um03935	1.00E-151	um03935	13d00036	1.00E-147
BBH	13d00077	um11054	1.00E-53	um11054	13d00077	2.00E-54
BBH	13c00094	um04043	1.00E-114	um04043	13c00094	1.00E-121
BBH	13c00066	um11743	1.00E-26	um11743	13c00066	1.00E-26
BBH	13c00020	um03908	0	um03908	13c00020	0
BBH	13d00086	um04042	0	um04042	13d00086	0
BBH	13d00047	um11735	5.00E-95	um11735	13d00047	1.00E-103
BBH	13c00098	um04048	1.00E-52	um04048	13c00098	4.00E-43
BBH	13c00088	um11055	1.00E-115	um11055	13c00088	1.00E-126
BBH	13d00067	um03985	1.00E-136	um03985	13d00067	1.00E-139
BBH	13c00048	um03960	3.00E-28	um03960	13c00048	3.00E-33
BBH	13c00030	um03937	0	um03937	13c00030	0
BBH	13c00035	um03929	1.00E-80	um03929	13c00035	7.00E-90
BBH	13d00026	um03914	1.00E-153	um03914	13d00026	1.00E-157
BBH	13c00106	um04070	4.00E-70	um04070	13c00106	4.00E-70
BBH	13c00013	um11516	5.00E-70	um11516	13c00013	9.00E-64
BBH	13d00102	um15094	0	um15094	13d00102	0
BBH	13c00005	um03890	2.00E-72	um03890	13c00005	4.00E-85
BBH	13c00045	um03956	5.00E-48	um03956	13c00045	2.00E-53
BBH	13d00052	um03970	4.00E-62	um03970	13d00052	5.00E-52
BBH	13d00068	um03983	8.00E-92	um03983	13d00068	4.00E-92
BBH	13d00111	um04080	0	um04080	13d00111	0
BBH	13d00104	um04071	0	um04071	13d00104	0
BBH	13c00079	um11051	1.00E-149	um11051	13c00079	1.00E-148
BBH	13d00041	um03946	1.00E-103	um03946	13d00041	1.00E-107
BBH	13d00087	um11063.2	0	um11063.2	13d00087	0
NNN	13d00109	um04081	2.00E-12	um04081	24d00036	2.00E-14
BBH	13c00067	um11744	6.00E-54	um11744	13c00067	2.00E-46
BBH	13c00052	um03969	0	um03969	13c00052	0
BBH	13d00091	um10579	1.00E-131	um10579	13d00091	1.00E-117
BBH	13c00089	um04026	0	um04026	13c00089	0
BBH	13c00083	um04020	9.00E-75	um04020	13c00083	8.00E-75
NNN	13c00095	um04044	0			
BBH	13c00059	um04005	1.00E-130	um04005	13c00059	1.00E-124
BBH	13d00035	um03936	0	um03936	13d00035	0
BBH	13c00082	um11053	1.00E-35	um11053	13c00082	1.00E-35
BBH	13c00047	um03958	1.00E-130	um03958	13c00047	1.00E-121
BBH	13c00026	um03923	2.00E-63	um03923	13c00026	2.00E-61
BBH	13d00027	um03917	0	um03917	13d00027	0
BBH	13c00038	um12247	3.00E-53	um12247	13c00038	3.00E-53
BBH	13c00039	um03947	0	um03947	13c00039	0
BBH	13d00014	um03876.2	0	um03876.2	13d00014	0
BBH	13c00006	um03888	1.00E-170	um03888	13c00006	1.00E-177
BBH	13c00105	um12251	4.00E-49	um12251	13c00105	1.00E-48
NNN	13c00014	um15099	9.00E-78			
BBH	13d00099	um04064	1.00E-167	um04064	13d00099	1.00E-164
NNN	13d00044	um11525	6.00E-95	um11525	12c00058	1.00E-117
BBH	13d00058	um04006	1.00E-105	um04006	13d00058	1.00E-114
BBH	13d00075	um04021	0	um04021	13d00075	0
NNN	13d00049	um03964	1.00E-105			
BBH	13d00023	um03910	8.00E-51	um03910	13d00023	5.00E-51
BBH	13c00034	um03933	7.00E-19	um03933	13c00034	8.00E-17
BBH	13c00109	um04074	1.00E-143	um04074	13c00109	1.00E-137
BBH	13d00031	um03944	0	um03944	13d00031	0
BBH	13c00093	um11062	1.00E-40	um11062	13c00093	6.00E-41
BBH	13c00078	um11050	1.00E-110	um11050	13c00078	1.00E-111
NNN	13c00010	um05972	1.00E-127	um05972	6c00005	0

BBH	13d00007	um03884	0	um03884	13d00007	0
BBH	13d00028	um03918	1.00E-126	um03918	13d00028	1.00E-143
BBH	13d00073	um11049.2	0	um11049.2	13d00073	0
BBH	13c00051	um03967	0	um03967	13c00051	0
BBH	13c00058	um15008	1.00E-162	um15008	13c00058	1.00E-167
BBH	13d00061	um11741	1.00E-142	um11741	13d00061	1.00E-150
BBH	13c00060	um04004	0	um04004	13c00060	0
BBH	13d00094	um04057	1.00E-168	um04057	13d00094	1.00E-161
BBH	13c00092	um04038	4.00E-70	um04038	13c00092	4.00E-65
BBH	13c00111	um11526	3.00E-78	um11526	13c00111	3.00E-76
BBH	13c00025	um03921	0	um03921	13c00025	0
BBH	13d00074	um04013	2.00E-75	um04013	13d00074	2.00E-80
BBH	13d00020	um03906	1.00E-144	um03906	13d00020	1.00E-159
BBH	13d00093	um04056	0	um04056	13d00093	0
BBH	13d00030	um11291	0	um11291	13d00030	0
BBH	13c00033	um11517	2.00E-41	um11517	13c00033	9.00E-50
BBH	13d00110	um04079	1.00E-124	um04079	13d00110	1.00E-143
BBH	13d00050	um03966	2.00E-19	um03966	13d00050	4.00E-19
BBH	13d00055	um04009	1.00E-124	um04009	13d00055	1.00E-129
BBH	13d00012	um03877	1.00E-150	um03877	13d00012	1.00E-147
BBH	13c00040	um03950	7.00E-63	um03950	13c00040	9.00E-68
BBH	13c00007	um03886	0	um03886	13c00007	0
BBH	13d00076	um04017	3.00E-84	um04017	13d00076	4.00E-90
BBH	13d00029	um11290	0	um11290	13d00029	0
BBH	13d00069	um03982	1.00E-97	um03982	13d00069	1.00E-108
BBH	13d00002	um03892	0	um03892	13d00002	0
BBH	13d00038	um03927	1.00E-139	um03927	13d00038	1.00E-129
NNN	13c00061	um03992	1.00E-101			
BBH	13c00049	um11736	9.00E-75	um11736	13c00049	1.00E-75
BBH	13c00002	um03893	1.00E-114	um03893	13c00002	1.00E-117
BBH	13c00068	um04000	3.00E-42	um04000	13c00068	2.00E-35
BBH	13c00050	um03965.2	1.00E-107	um03965.2	13c00050	1.00E-107
BBH	13c00057	um11046	1.00E-119	um11046	13c00057	1.00E-136
BBH	13d00092	um04055	1.00E-106	um04055	13d00092	0
BBH	13d00005	um12244	3.00E-38	um12244	13d00005	2.00E-40
BBH	13d00103	um04069	0	um04069	13d00103	0
BBH	13c00084	um04019	8.00E-99	um04019	13c00084	1.00E-112
BBH	13c00104	um04059	0	um04059	13c00104	0
BBH	13c00024	um03919	5.00E-62	um03919	13c00024	1.00E-62
BBH	13c00015	um11967	0	um11967	13c00015	0
BBH	13c00077	um03977	3.00E-26	um03977	13c00077	5.00E-26
BBH	13d00043	um03953	2.00E-66	um03953	13d00043	7.00E-60
BBH	13c00070	um11747.2	6.00E-65	um11747.2	13c00070	2.00E-59
BBH	13c00036	um03928	0	um03928	13c00036	0
BBH	13c00023	um03916	3.00E-37	um03916	13c00023	2.00E-39
BBH	13c00017	um03902	0	um03902	13c00017	0
BBH	13c00085	um04016	2.00E-35	um04016	13c00085	1.00E-46
BBH	13c00044	um11522	4.00E-41	um11522	13c00044	3.00E-38
BBH	13d00056	um04007	2.00E-82	um04007	13d00056	1.00E-89
BBH	13d00009	um03881	4.00E-64	um03881	13d00009	7.00E-77
BBH	13c00102	um04054	1.00E-172	um04054	13c00102	1.00E-170
BBH	13d00071	um03978	5.00E-33	um03978	13d00071	5.00E-33
BBH	13d00096	um04060	1.00E-126	um04060	13d00096	1.00E-120
BBH	13c00069	um11745	0	um11745	13c00069	0
BBH	13d00046	um03959	0	um03959	13d00046	0
BBH	13d00082	um04030	3.00E-37	um04030	13d00082	3.00E-26
BBH	13d00081	um04029	1.00E-159	um04029	13d00081	1.00E-159
BBH	13d00037	um03930	1.00E-74	um03930	13d00037	4.00E-75
BBH	13c00056	um11047	0	um11047	13c00056	0
NNN	13d00066	um11739	7.00E-76			
BBH	13c00062	um03994	1.00E-155	um03994	13c00062	1.00E-155
BBH	13d00063	um12250	0	um12250	13d00063	0

BBH	13c00090	um04032	0	um04032	13c00090	0
BBH	13c00071	um03989.2	1.00E-110	um03989.2	13c00071	1.00E-130
BBH	13c00076	um03979	1.00E-168	um03979	13c00076	0
BBH	13c00019	um11970	2.00E-98	um11970	13c00019	1.00E-120
BBH	13d00010	um03880	1.00E-131	um03880	13d00010	1.00E-137
BBH	13c00031	um11518	0	um11518	13c00031	0
BBH	13c00101	um04053	0	um04053	13c00101	0
BBH	13d00032	um03942	0	um03942	13d00032	0
BBH	13d00022	um03909	0	um03909	13d00022	0
BBH	13d00053	um03973	0	um03973	13d00053	0
NNN	13d00072	um11048	0			
BBH	13c00018	um03905	1.00E-171	um03905	13c00018	0
BBH	13d00105	um11636	0	um11636	13d00105	0
BBH	13c00009	um11510	0	um11510	13c00009	0
BBH	13c00091	um04031	0	um04031	13c00091	0
BBH	13c00072	um03987	0	um03987	13c00072	0
BBH	13c00086	um11052	2.00E-30	um11052	13c00086	4.00E-27
BBH	13c00075	um11973	1.00E-140	um11973	13c00075	1.00E-140
BBH	13d00006	um11512	1.00E-121	um11512	13d00006	1.00E-122
BBH	13d00021	um15063	0	um15063	13d00021	0
BBH	13d00095	um04061	1.00E-155	um04061	13d00095	1.00E-155
NNN	13c00063	um03994	1.00E-109	um03994	13c00062	1.00E-155
NNN	13d00064	um11746	1.00E-134			
BBH	13c00055	um03976	1.00E-173	um03976	13c00055	1.00E-176
BBH	14c00123	um03166	2.00E-28	um03166	14c00123	4.00E-27
BBH	14d00074	um10649	1.00E-114	um10649	14d00074	1.00E-104
BBH	14d00021	um03347	0	um03347	14d00021	0
NNN	14c00082	um03232	3.00E-21	um03232	14c00083	2.00E-22
BBH	14c00014	um03361	0	um03361	14c00014	0
BBH	14d00117	um12208	0	um12208	14d00117	0
BBH	14c00032	um03317	1.00E-175	um03317	14c00032	0
BBH	14c00071	um11956	3.00E-84	um11956	14c00071	3.00E-86
BBH	14d00050	um03286	0	um03286	14d00050	0
BBH	14c00053	um03278	0	um03278	14c00053	0
BBH	14c00006	um11016	1.00E-135	um11016	14c00006	1.00E-136
BBH	14d00109	um03171.2	1.00E-159	um03171.2	14d00109	1.00E-160
NNN	14c00061	um03267	0			
BBH	14c00129	um03134	2.00E-52	um03134	14c00129	2.00E-52
BBH	14d00111	um03162	0	um03162	14d00111	0
BBH	14c00090	um10645	1.00E-148	um10645	14c00090	1.00E-132
BBH	14c00119	um03165	1.00E-126	um03165	14c00119	1.00E-110
BBH	14d00036	um10836	0	um10836	14d00036	0
BBH	14d00088	um03204	0	um03204	14d00088	0
BBH	14c00112	um03152	1.00E-129	um03152	14c00112	1.00E-129
BBH	14c00075	um03242	4.00E-30	um03242	14c00075	2.00E-20
BBH	14d00027	um03337	1.00E-105	um03337	14d00027	1.00E-104
BBH	14c00027	um10654	5.00E-14	um10654	14c00027	5.00E-14
BBH	14c00066	um03254	0	um03254	14c00066	0
BBH	14c00105	um03147	0	um03147	14c00105	0
BBH	14c00130	um03132	1.00E-119	um03132	14c00130	1.00E-126
BBH	14d00102	um03192	3.00E-37	um03192	14d00102	1.00E-47
BBH	14d00029	um03331	1.00E-178	um03331	14d00029	0
BBH	14c00017	um03353	1.00E-125	um03353	14c00017	1.00E-130
BBH	14d00022	um03346.2	4.00E-61	um03346.2	14d00022	8.00E-61
BBH	14d00073	um03238	1.00E-154	um03238	14d00073	1.00E-178
BBH	14d00070	um10650	0	um10650	14d00070	0
NNN	14c00100	um10401	0			
BBH	14c00083	um03232	1.00E-22	um03232	14c00083	2.00E-22
BBH	14d00067	um03263	0	um03263	14d00067	0
BBH	14c00062	um10652	1.00E-124	um10652	14c00062	1.00E-116
BBH	14c00065	um03253	0	um03253	14c00065	0
BBH	14d00003	um03383	3.00E-96	um03383	14d00003	1.00E-117

BBH	14d00055	um03276	2.00E-26	um03276	14d00055	3.00E-27
BBH	14d00094	um03148	0	um03148	14d00094	0
BBH	14c00050	um03283	1.00E-47	um03283	14c00050	3.00E-52
BBH	14d00019	um03349	1.00E-124	um03349	14d00019	1.00E-123
BBH	14c00001	um11017	1.00E-143	um11017	14c00001	1.00E-140
BBH	14d00012	um03363	0	um03363	14d00012	0
BBH	14d00089	um03201	4.00E-40	um03201	14d00089	5.00E-40
BBH	14c00135	um05978	1.00E-111	um05978	14c00135	1.00E-111
BBH	14c00121	um03168.2	8.00E-48	um03168.2	14c00121	5.00E-47
BBH	14d00080	um03218	1.00E-127	um03218	14d00080	1.00E-124
BBH	14d00037	um10835	1.00E-51	um10835	14d00037	2.00E-59
BBH	14c00026	um03330	2.00E-81	um03330	14c00026	5.00E-80
BBH	14d00028	um03333	2.00E-80	um03333	14d00028	1.00E-77
BBH	14c00070	um03261	0	um03261	14c00070	0
BBH	14c00023	um12222	1.00E-135	um12222	14c00023	1.00E-137
BBH	14d00106	um03158	1.00E-152	um03158	14d00106	1.00E-152
NNN	14c00084	um10648	0	um10648	14c00045	0
BBH	14c00074	um03244	6.00E-52	um03244	14c00074	8.00E-47
BBH	14d00103	um03194	1.00E-165	um03194	14d00103	1.00E-170
BBH	14d00056	um10824	0	um10824	14d00056	0
BBH	14c00051	um03282	1.00E-149	um03282	14c00051	1.00E-149
BBH	14d00093	um03150	1.00E-122	um03150	14d00093	1.00E-125
BBH	14c00022	um03336	1.00E-153	um03336	14c00022	1.00E-153
BBH	14c00106	um03146	0	um03146	14c00106	0
BBH	14c00068	um11954	0	um11954	14c00068	0
BBH	14c00009	um03373	1.00E-155	um03373	14c00009	1.00E-159
BBH	14c00039	um03304	4.00E-34	um03304	14c00039	4.00E-32
BBH	14d00011	um03365	2.00E-25	um03365	14d00011	7.00E-35
BBH	14c00012	um03366	0	um03366	14c00012	0
BBH	14d00105	um03153	0	um03153	14d00105	0
BBH	14c00126	um12212	5.00E-96	um12212	14c00126	4.00E-92
BBH	14c00024	um10656	0	um10656	14c00024	0
BBH	14c00030	um10842	1.00E-121	um10842	14c00030	1.00E-121
NNN	14d00004	um06453	2.00E-66	um06453	1c00050	1.00E-123
BBH	14c00092	um10643	0	um10643	14c00092	0
BBH	14d00092	um03182	0	um03182	14d00092	0
BBH	14c00059	um03271	1.00E-127	um03271	14c00059	1.00E-109
NNN	14d00122	um10220	4.00E-11	um10220	15c00012	1.00E-174
BBH	14c00025	um03332	2.00E-83	um03332	14c00025	7.00E-86
BBH	14d00025	um03341	0	um03341	14d00025	0
BBH	14c00063	um03250	0	um03250	14c00063	0
BBH	14c00042	um03296	7.00E-86	um03296	14c00042	6.00E-83
BBH	14c00069	um03260	3.00E-23	um03260	14c00069	1.00E-30
BBH	14d00119	um03127	0	um03127	14d00119	0
BBH	14d00081	um10644	1.00E-155	um10644	14d00081	1.00E-161
BBH	14d00113	um03177	9.00E-79	um03177	14d00113	9.00E-79
BBH	14c00056	um03274	1.00E-20	um03274	14c00056	1.00E-20
BBH	14c00124	um03172	1.00E-104	um03172	14c00124	1.00E-95
BBH	14d00079	um03220	1.00E-126	um03220	14d00079	1.00E-133
BBH	14d00041	um03301	0	um03301	14d00041	0
BBH	14d00069	um03266	0	um03266	14d00069	0
BBH	14c00098	um03200	0	um03200	14c00098	1.00E-180
BBH	14d00053	um10827	1.00E-174	um10827	14d00053	0
BBH	14c00031	um10840	0	um10840	14c00031	0
BBH	14c00045	um03293	0	um03293	14c00045	0
BBH	14c00091	um03216	0	um03216	14c00091	0
BBH	14c00088	um03221	1.00E-174	um03221	14c00088	1.00E-179
NNN	14d00001	um00061	4.00E-16	um00061	9d00030	0
BBH	14c00015	um11009	0	um11009	14c00015	0
BBH	14d00104	um10399	1.00E-102	um10399	14d00104	1.00E-106
BBH	14d00020	um03348	1.00E-116	um03348	14d00020	1.00E-111
BBH	14c00008	um03375	1.00E-167	um03375	14c00008	1.00E-161

BBH	14d00026	um12223	6.00E-16	um12223	14d00026	2.00E-18
BBH	14d00108	um03164	0	um03164	14d00108	0
BBH	14c00077	um03239	0	um03239	14c00077	0
BBH	14c00067	um11953	0	um11953	14c00067	0
BBH	14d00042	um03299	1.00E-114	um03299	14d00042	1.00E-115
NNN	14c00003	um03034	1.00E-159	um03034	19c00045	0
BBH	14d00110	um03169	0	um03169	14d00110	0
BBH	14c00086	um03229	0	um03229	14c00086	0
BBH	14c00076	um03240	1.00E-97	um03240	14c00076	6.00E-93
BBH	14c00021	um10657	5.00E-59	um10657	14c00021	2.00E-58
BBH	14d00035	um10837	1.00E-130	um10837	14d00035	1.00E-127
BBH	14c00038	um03305	0	um03305	14c00038	0
BBH	14d00116	um12209	0	um12209	14d00116	0
BBH	14d00058	um10953	7.00E-42	um10953	14d00058	4.00E-35
BBH	14c00111	um12210	1.00E-140	um12210	14c00111	1.00E-155
BBH	14d00082	um10642	1.00E-35	um10642	14d00082	5.00E-36
BBH	14d00091	um10380	0	um10380	14d00091	0
BBH	14c00057	um03273	0	um03273	14c00057	0
BBH	14c00044	um03294	1.00E-14	um03294	14c00044	1.00E-14
BBH	14d00033	um12221	9.00E-77	um12221	14d00033	7.00E-81
BBH	14d00043	um10831	6.00E-93	um10831	14d00043	7.00E-93
BBH	14d00031	um10653	0	um10653	14d00031	0
BBH	14d00083	um10640	1.00E-38	um10640	14d00083	6.00E-39
BBH	14c00113	um10382	1.00E-33	um10382	14c00113	2.00E-39
NNN	14c00115	um10383	1.00E-40	um10383	14c00116	0
BBH	14c00089	um10646	9.00E-31	um10646	14c00089	5.00E-31
BBH	14d00071	um12218	0	um12218	14d00071	0
BBH	14d00115	um03135	1.00E-79	um03135	14d00115	8.00E-78
BBH	14d00009	um03371	3.00E-74	um03371	14d00009	3.00E-71
BBH	14d00099	um03140	0	um03140	14d00099	0
BBH	14c00134	um03126	0	um03126	14c00134	0
BBH	14d00023	um03344	0	um03344	14d00023	0
BBH	14d00090	um03199	0	um03199	14d00090	0
BBH	14d00014	um03358	0	um03358	14d00014	0
BBH	14d00060	um11957	0	um11957	14d00060	0
BBH	14c00103	um10394	1.00E-82	um10394	14c00103	2.00E-90
BBH	14c00128	um10393	2.00E-40	um10393	14c00128	1.00E-49
BBH	14c00020	um03340	3.00E-36	um03340	14c00020	5.00E-36
BBH	14d00077	um10982	1.00E-30	um10982	14d00077	1.00E-28
BBH	14c00013	um11012	3.00E-74	um11012	14c00013	3.00E-70
NNN	14c00018	um12224	1.00E-171			
NNN	14d00002	um05411	3.00E-50	um05411	15d00014	0
BBH	14c00002	um03384	0	um03384	14c00002	0
BBH	14c00054	um10826	3.00E-42	um10826	14c00054	2.00E-35
BBH	14c00079	um03234.2	0	um03234.2	14c00079	0
BBH	14c00037	um03307	4.00E-94	um03307	14c00037	1.00E-100
NNN	14c00087	um03223	5.00E-21	um03223	14d00078	1.00E-29
BBH	14d00064	um12219	9.00E-82	um12219	14d00064	1.00E-88
BBH	14c00116	um10383	0	um10383	14c00116	0
BBH	14c00114	um03156	8.00E-95	um03156	14c00114	1.00E-101
BBH	14c00055	um10825	1.00E-102	um10825	14c00055	1.00E-107
BBH	14c00047	um10830	0	um10830	14c00047	0
BBH	14d00034	um10839	2.00E-61	um10839	14d00034	2.00E-61
BBH	14c00125	um10390	1.00E-81	um10390	14c00125	9.00E-83
BBH	14d00084	um03211	2.00E-97	um03211	14d00084	1.00E-105
BBH	14d00057	um10823	4.00E-14	um10823	14d00057	5.00E-14
BBH	14d00013	um03362	4.00E-69	um03362	14d00013	8.00E-87
BBH	14d00048	um03288	6.00E-31	um03288	14d00048	7.00E-31
BBH	14d00024	um10658	0	um10658	14d00024	1.00E-171
BBH	14c00094	um03210	1.00E-35	um03210	14c00094	9.00E-36
BBH	14c00133	um03128	1.00E-136	um03128	14c00133	1.00E-136
BBH	14c00078	um03237	5.00E-56	um03237	14c00078	5.00E-56

BBH	14c00036	um03309	0	um03309	14c00036	0
BBH	14c00073	um03265	3.00E-63	um03265	14c00073	4.00E-58
BBH	14c00109	um10397	0	um10397	14c00109	0
NNN	14c00120	um03170	0			
BBH	14d00118	um11952	1.00E-163	um11952	14d00118	1.00E-165
BBH	14c00104	um03149	1.00E-164	um03149	14c00104	1.00E-168
BBH	14d00054	um03277	0	um03277	14d00054	0
BBH	14d00063	um03251	1.00E-44	um03251	14d00063	1.00E-33
NNN	14d00044	um03290	2.00E-34	um03290	14d00045	1.00E-145
BBH	14c00049	um03284	2.00E-38	um03284	14c00049	2.00E-38
BBH	14d00112	um10391	1.00E-173	um10391	14d00112	1.00E-178
BBH	14d00098	um03141	0	um03141	14d00098	0
BBH	14c00093	um10641	0	um10641	14c00093	0
BBH	14d00016	um03352	5.00E-61	um03352	14d00016	1.00E-69
BBH	14c00029	um03323	0	um03323	14c00029	0
BBH	14c00046	um03292	0	um03292	14c00046	0
BBH	14d00040	um10833	0	um10833	14d00040	0
BBH	14d00038	um03306	0	um03306	14d00038	0
BBH	14d00045	um03290	1.00E-145	um03290	14d00045	1.00E-145
BBH	14c00041	um03298	0	um03298	14c00041	0
BBH	14c00034	um03312	1.00E-164	um03312	14c00034	1.00E-158
BBH	14c00117	um10384	1.00E-52	um10384	14c00117	2.00E-50
BBH	14d00085	um10639	1.00E-135	um10639	14d00085	1.00E-156
BBH	14c00064	um03252	0	um03252	14c00064	0
BBH	14d00121	um02813	1.00E-166	um02813	14d00121	1.00E-159
BBH	14c00110	um10398	3.00E-79	um10398	14c00110	3.00E-71
BBH	14d00005	um03379	0	um03379	14d00005	0
BBH	14d00052	um03281	0	um03281	14d00052	0
BBH	14d00097	um03143	8.00E-76	um03143	14d00097	6.00E-70
NNN	14c00080	um03232	2.00E-21	um03232	14c00083	2.00E-22
NNN	14d00076	um03233	1.00E-141			
BBH	14c00052	um03280	0	um03280	14c00052	0
BBH	14c00048	um03285	0	um03285	14c00048	0
BBH	14c00035	um03310	1.00E-105	um03310	14c00035	1.00E-111
BBH	14c00005	um03381	1.00E-128	um03381	14c00005	1.00E-124
BBH	14c00101	um10400	1.00E-175	um10400	14c00101	1.00E-175
BBH	14c00016	um03355	6.00E-96	um03355	14c00016	1.00E-89
BBH	14d00107	um10385	0	um10385	14d00107	0
BBH	14d00101	um10396.2	0	um10396.2	14d00101	0
BBH	14c00033	um03315	0	um03315	14c00033	0
BBH	14c00011	um11014	0	um11014	14c00011	0
BBH	14c00122	um03167	1.00E-167	um03167	14c00122	1.00E-166
BBH	14d00018	um03351	0	um03351	14d00018	0
BBH	14d00072	um03241	1.00E-140	um03241	14d00072	1.00E-140
BBH	14d00007	um12228	7.00E-73	um12228	14d00007	7.00E-77
BBH	14c00107	um10381	4.00E-38	um10381	14c00107	6.00E-37
BBH	14d00066	um03262	0	um03262	14d00066	0
BBH	14c00028	um03325	0	um03325	14c00028	0
BBH	14c00007	um03376	0	um03376	14c00007	0
BBH	14c00060	um15098	1.00E-48	um15098	14c00060	3.00E-45
BBH	14d00059	um15004	0	um15004	14d00059	0
BBH	14c00096	um03206	0	um03206	14c00096	0
BBH	14d00046	um03289	1.00E-104	um03289	14d00046	1.00E-79
NNN	14c00137	um02021	9.00E-32	um02021	22c00321	1.00E-137
BBH	14d00015	um03356	0	um03356	14d00015	0
BBH	14d00039	um03303	0	um03303	14d00039	0
BBH	14d00096	um03144	0	um03144	14d00096	0
BBH	14c00131	um10379	0	um10379	14c00131	0
BBH	14d00062	um10651	1.00E-176	um10651	14d00062	0
BBH	14c00118	um10386	5.00E-96	um10386	14c00118	1.00E-92
BBH	14d00087	um15003	0	um15003	14d00087	0
BBH	14d00030	um10655	0	um10655	14d00030	0

BBH	14d00114	um03180	0	um03180	14d00114	0
BBH	14d00086	um03207	4.00E-78	um03207	14d00086	4.00E-78
BBH	14d00120	um03124	1.00E-120	um03124	14d00120	1.00E-122
BBH	14d00075	um03235	0	um03235	14d00075	0
BBH	14c00127	um10392	1.00E-171	um10392	14c00127	1.00E-165
BBH	14c00040	um10832	6.00E-56	um10832	14c00040	6.00E-56
BBH	14d00065	um11955	1.00E-161	um11955	14d00065	1.00E-162
BBH	14c00095	um03209	0	um03209	14c00095	0
BBH	14d00006	um03377	1.00E-87	um03377	14d00006	3.00E-99
BBH	14d00078	um03223	2.00E-31	um03223	14d00078	1.00E-29
BBH	14d00010	um11013	0	um11013	14d00010	0
BBH	14c00108	um03139	4.00E-88	um03139	14c00108	3.00E-90
BBH	14d00008	um12227	6.00E-41	um12227	14d00008	6.00E-41
BBH	14c00102	um03136	9.00E-62	um03136	14c00102	5.00E-60
BBH	14d00068	um03264	0	um03264	14d00068	0
BBH	14d00061	um03246	0	um03246	14d00061	0
BBH	14c00004	um03382	0	um03382	14c00004	0
BBH	14d00095	um03145	2.00E-92	um03145	14d00095	6.00E-90
BBH	14c00010	um03368	9.00E-98	um03368	14c00010	1.00E-110
NNN	15d00091	um10339	1.00E-151	um10339	19c00074	0
BBH	15d00002	um02821	8.00E-27	um02821	15d00002	2.00E-26
BBH	15d00013	um05415	0	um05415	15d00013	0
BBH	15d00063	um05290	1.00E-140	um05290	15d00063	1.00E-138
BBH	15c00043	um05344	0	um05344	15c00043	0
BBH	15c00019	um05403	1.00E-134	um05403	15c00019	1.00E-139
BBH	15d00053	um05339	1.00E-113	um05339	15d00053	1.00E-113
BBH	15d00071	um05276	2.00E-51	um05276	15d00071	2.00E-51
BBH	15d00056	um05332	2.00E-48	um05332	15d00056	3.00E-62
BBH	15d00083	um05254	1.00E-95	um05254	15d00083	5.00E-98
BBH	15c00024	um05387	4.00E-95	um05387	15c00024	1.00E-93
BBH	15c00022	um05396.2	0	um05396.2	15c00022	0
BBH	15d00030	um05378	0	um05378	15d00030	0
BBH	15d00025	um05386	0	um05386	15d00025	0
NNN	15c00094	um03006	5.00E-25	um03006	19c00058	1.00E-139
BBH	15c00065	um05302	2.00E-25	um05302	15c00065	2.00E-25
BBH	15d00041	um05361	0	um05361	15d00041	0
NNN	15d00001	um06459	5.00E-16	um06459	9c00018	2.00E-23
NNN	15c00092	um01996	6.00E-81			
BBH	15d00014	um05411	0	um05411	15d00014	0
NNN	15d00020	um05395	0			
BBH	15d00026	um05385	1.00E-163	um05385	15d00026	1.00E-169
BBH	15c00054	um05328	0	um05328	15c00054	0
BBH	15c00046	um10200	0	um10200	15c00046	0
BBH	15d00054	um12083	1.00E-14	um12083	15d00054	4.00E-14
BBH	15c00008	um05429	1.00E-101	um05429	15c00008	1.00E-122
BBH	15d00033	um05370	3.00E-17	um05370	15d00033	1.00E-20
BBH	15d00064	um10552	0	um10552	15d00064	0
BBH	15d00032	um05373	0	um05373	15d00032	0
BBH	15c00085	um05255	2.00E-40	um05255	15c00085	2.00E-40
BBH	15c00021	um05398.2	0	um05398.2	15c00021	0
BBH	15c00010	um05420	0	um05420	15c00010	0
BBH	15c00064	um05306	5.00E-25	um05306	15c00064	3.00E-21
BBH	15d00008	um05428	1.00E-62	um05428	15d00008	8.00E-64
BBH	15c00084	um10546	1.00E-59	um10546	15c00084	5.00E-53
BBH	15c00070	um10705	1.00E-153	um10705	15c00070	1.00E-153
BBH	15c00059	um10558	0	um10558	15c00059	0
BBH	15c00023	um05392	0	um05392	15c00023	0
BBH	15d00074	um05273	0	um05273	15d00074	0
NNN	15d00086	um10713.2	7.00E-12	um10713.2	3d00087	1.00E-89
NNN	15d00093	um06418	7.00E-96	um06418	1c00034	0
BBH	15c00079	um05261	0	um05261	15c00079	0
BBH	15d00011	um05421	0	um05421	15d00011	0

BBH	15d00058	um05327	2.00E-69	um05327	15d00058	1.00E-78
BBH	15c00053	um15101	4.00E-58	um15101	15c00053	5.00E-58
BBH	15d00073	um05274	0	um05274	15d00073	0
NNN	15d00007	um05431	1.00E-91			
NNN	15c00087	um04806	2.00E-48	um04806	26c00044	0
BBH	15c00006	um05433	0	um05433	15c00006	0
NNN	15c00077	um00372	5.00E-61	um00372	9d00180	0
BBH	15c00003	um05619	1.00E-168	um05619	15c00003	1.00E-157
BBH	15c00033	um05364	1.00E-173	um05364	15c00033	0
BBH	15c00076	um10548	3.00E-97	um10548	15c00076	3.00E-91
BBH	15d00028	um05381	0	um05381	15d00028	0
BBH	15c00063	um05308	2.00E-32	um05308	15c00063	8.00E-30
BBH	15c00078	um05269	0	um05269	15c00078	0
BBH	15c00020	um10215	1.00E-126	um10215	15c00020	1.00E-133
BBH	15c00018	um05405	0	um05405	15c00018	0
BBH	15d00055	um05334	5.00E-92	um05334	15d00055	5.00E-92
BBH	15d00012	um05417	0	um05417	15d00012	0
BBH	15c00062	um05414	0	um05414	15c00062	0
BBH	15d00066	um05286.2	1.00E-139	um05286.2	15d00066	1.00E-139
BBH	15d00022	um05391	1.00E-112	um05391	15d00022	1.00E-105
BBH	15c00005	um05435	1.00E-104	um05435	15c00005	2.00E-91
BBH	15d00038	um05365	0	um05365	15d00038	0
BBH	15d00039	um05363	0	um05363	15d00039	1.00E-169
BBH	15d00029	um10213	0	um10213	15d00029	0
BBH	15d00021	um05393	0	um05393	15d00021	0
BBH	15c00086	um05253	0	um05253	15c00086	0
BBH	15c00034	um05358	3.00E-26	um05358	15c00034	4.00E-21
BBH	15d00076	um05270	1.00E-58	um05270	15d00076	4.00E-60
BBH	15d00040	um05362	0	um05362	15d00040	0
BBH	15c00017	um05406	2.00E-80	um05406	15c00017	2.00E-76
BBH	15c00040	um05347	0	um05347	15c00040	0
BBH	15d00006	um05432.2	0	um05432.2	15d00006	0
BBH	15d00079	um05265	2.00E-29	um05265	15d00079	2.00E-29
BBH	15c00056	um05325	0	um05325	15c00056	0
BBH	15d00088	um10544	1.00E-93	um10544	15d00088	3.00E-93
BBH	15d00004	um06478	0	um06478	15d00004	0
BBH	15c00028	um05377	1.00E-105	um05377	15c00028	1.00E-103
NNN	15c00091	um00919	1.00E-102	um00919	7d00317	1.00E-149
BBH	15c00016	um05407	0	um05407	15c00016	0
NNN	15c00002	um00646	3.00E-48	um00646	9d00317	1.00E-149
BBH	15d00036	um05368	2.00E-39	um05368	15d00036	4.00E-39
BBH	15c00035	um05356	3.00E-49	um05356	15c00035	2.00E-62
BBH	15d00078	um05264	0	um05264	15d00078	1.00E-174
BBH	15d00017	um05404.2	1.00E-146	um05404.2	15d00017	1.00E-107
BBH	15d00005	um05434	1.00E-117	um05434	15d00005	1.00E-121
BBH	15d00052	um05345	1.00E-134	um05345	15d00052	1.00E-130
BBH	15d00067	um12301	4.00E-66	um12301	15d00067	3.00E-69
BBH	15c00055	um05326	0	um05326	15c00055	0
BBH	15d00059	um10559	0	um10559	15d00059	0
BBH	15d00043	um10206	0	um10206	15d00043	0
BBH	15c00074	um10551.2	0	um10551.2	15c00074	0
BBH	15d00075	um05271	1.00E-173	um05271	15d00075	1.00E-172
BBH	15c00048	um05338	1.00E-125	um05338	15c00048	1.00E-132
BBH	15d00080	um05267	1.00E-70	um05267	15d00080	2.00E-70
BBH	15c00009	um05422	0	um05422	15c00009	0
NNN	15d00087	um00005	1.00E-32	um00005	9c00018	0
BBH	15d00003	um03046	1.00E-29	um03046	15d00003	3.00E-29
BBH	15c00015	um05408	0	um05408	15c00015	0
NNN	15c00001	um01099	1.00E-17	um01099	7c00216	1.00E-154
BBH	15c00036	um05355	0	um05355	15c00036	0
BBH	15c00081	um05266	1.00E-153	um05266	15c00081	1.00E-161
BBH	15c00027	um05380	7.00E-68	um05380	15c00027	6.00E-64

BBH	15d00070	um05278	6.00E-64	um05278	15d00070	4.00E-70
BBH	15d00077	um05263	1.00E-113	um05263	15d00077	1.00E-105
BBH	15d00082	um05256	1.00E-158	um05256	15d00082	1.00E-160
BBH	15d00037	um05366	8.00E-60	um05366	15d00037	7.00E-58
BBH	15d00057	um05329	0	um05329	15d00057	0
NNN	15c00088	um01269	8.00E-79	um01269	7d00135	0
BBH	15c00075	um10549	1.00E-116	um10549	15c00075	1.00E-114
NNN	15d00090	um03207	5.00E-30	um03207	14d00086	4.00E-78
BBH	15c00004	um06477	3.00E-48	um06477	15c00004	2.00E-47
BBH	15c00050	um05335	2.00E-64	um05335	15c00050	8.00E-62
BBH	15c00069	um05292	0	um05292	15c00069	0
BBH	15d00068	um05282	3.00E-72	um05282	15d00068	5.00E-74
BBH	15c00068	um05293	0	um05293	15c00068	0
BBH	15c00047	um05340	1.00E-128	um05340	15c00047	1.00E-119
BBH	15c00030	um10211	0	um10211	15c00030	0
BBH	15d00044	um05357	0	um05357	15d00044	0
BBH	15d00018	um05402	1.00E-134	um05402	15d00018	1.00E-140
BBH	15d00060	um10556	2.00E-20	um10556	15d00060	2.00E-20
BBH	15d00042	um10207	0	um10207	15d00042	0
NNN	15c00037	um05353	0			
BBH	15d00023	um05390	1.00E-110	um05390	15d00023	1.00E-110
BBH	15d00034	um10210.2	1.00E-102	um10210.2	15d00034	6.00E-98
BBH	15d00081	um05258	1.00E-134	um05258	15d00081	1.00E-138
BBH	15c00007	um15066	0	um15066	15c00007	0
BBH	15c00083	um05259	1.00E-153	um05259	15c00083	1.00E-156
BBH	15c00012	um10220	1.00E-174	um10220	15c00012	1.00E-174
BBH	15c00026	um05382	0	um05382	15c00026	0
BBH	15c00052	um05331	3.00E-64	um05331	15c00052	3.00E-69
BBH	15c00014	um05412	0	um05412	15c00014	0
BBH	15c00049	um05337	1.00E-146	um05337	15c00049	1.00E-152
BBH	15d00045	um05354.2	3.00E-93	um05354.2	15d00045	9.00E-88
BBH	15c00058	um10560	1.00E-63	um10560	15c00058	9.00E-61
BBH	15c00067	um05295	3.00E-16	um05295	15c00067	4.00E-16
BBH	15d00048	um05348	0	um05348	15d00048	0
BBH	15c00031	um05371	1.00E-128	um05371	15c00031	1.00E-126
BBH	15d00015	um05410	2.00E-79	um05410	15d00015	1.00E-73
BBH	15c00038	um05351	0	um05351	15c00038	1.00E-176
BBH	15c00051	um05333	1.00E-142	um05333	15c00051	1.00E-149
BBH	15d00072	um05275	0	um05275	15d00072	0
BBH	15c00071	um05287	1.00E-178	um05287	15c00071	1.00E-157
BBH	15d00010	um05423	0	um05423	15d00010	0
NNN	15d00092	um10339	0	um10339	19c00074	0
BBH	15d00024	um10214	4.00E-35	um10214	15d00024	2.00E-34
NNN	15d00035	um10209	0			
BBH	15c00041	um05346	0	um05346	15c00041	0
NNN	15d00089	um06418	2.00E-69	um06418	1c00034	0
NNN	15d00069	um05280	8.00E-45			
BBH	15c00013	um05416	0	um05416	15c00013	0
BBH	15c00011	um10221	1.00E-104	um10221	15c00011	1.00E-102
BBH	15c00025	um05383	0	um05383	15c00025	0
BBH	15c00082	um05260	0	um05260	15c00082	0
BBH	15c00032	um10208	1.00E-13	um10208	15c00032	6.00E-12
BBH	15d00084	um05252	1.00E-139	um05252	15d00084	1.00E-139
BBH	15d00046	um05352	1.00E-175	um05352	15d00046	1.00E-171
BBH	15c00057	um10561	0	um10561	15c00057	0
BBH	15c00073	um05283	1.00E-121	um05283	15c00073	1.00E-121
BBH	15d00019	um05401	0	um05401	15d00019	0
BBH	15c00039	um05349	0	um05349	15c00039	0
BBH	15d00062	um05291	1.00E-127	um05291	15d00062	1.00E-110
BBH	15c00080	um05262	0	um05262	15c00080	0
NNN	15c00072	um05285	0			
NNN	16c00002	um11741	4.00E-52	um11741	13d00061	1.00E-150

BBH	16c00039	um05515	3.00E-16	um05515	16c00039	2.00E-17
BBH	16d00050	um05540	0	um05540	16d00050	0
BBH	16c00043	um05522	0	um05522	16c00043	0
BBH	16d00086	um05608	1.00E-42	um05608	16d00086	3.00E-43
BBH	16d00041	um05527	0	um05527	16d00041	0
BBH	16c00070	um05567	2.00E-62	um05567	16c00070	3.00E-63
BBH	16c00068	um10896	0	um10896	16c00068	0
BBH	16c00046	um05526	1.00E-84	um05526	16c00046	2.00E-91
BBH	16d00048	um10355	5.00E-57	um10355	16d00048	2.00E-57
BBH	16d00033	um12305	0	um12305	16d00033	0
BBH	16d00070	um05583	2.00E-31	um05583	16d00070	2.00E-34
BBH	16d00077	um05588	1.00E-166	um05588	16d00077	1.00E-176
BBH	16c00033	um05501	0	um05501	16c00033	0
BBH	16c00089	um05612	1.00E-166	um05612	16c00089	1.00E-165
BBH	16c00062	um05557	0	um05557	16c00062	0
BBH	16c00077	um11178	5.00E-55	um11178	16c00077	4.00E-50
BBH	16d00064	um05570	6.00E-88	um05570	16d00064	2.00E-92
BBH	16d00021	um10751	1.00E-160	um10751	16d00021	1.00E-164
BBH	16c00026	um05490	1.00E-81	um05490	16c00026	6.00E-83
BBH	16c00082	um12308	2.00E-99	um12308	16c00082	1.00E-106
BBH	16c00090	um11182	9.00E-46	um11182	16c00090	1.00E-45
BBH	16d00018	um05472	6.00E-55	um05472	16d00018	6.00E-55
BBH	16d00056	um05552	1.00E-118	um05552	16d00056	1.00E-123
BBH	16d00082	um05605	1.00E-146	um05605	16d00082	1.00E-157
BBH	16d00087	um11513	0	um11513	16d00087	0
BBH	16d00076	um05592	0	um05592	16d00076	0
BBH	16c00059	um11792	1.00E-124	um11792	16c00059	1.00E-109
BBH	16c00078	um11179	3.00E-71	um11179	16c00078	2.00E-67
BBH	16c00001	um04697	0	um04697	16c00001	0
BBH	16c00016	um10747	3.00E-91	um10747	16c00016	4.00E-92
BBH	16c00067	um05563	0	um05563	16c00067	0
NNN	16c00030	um05496	0			
BBH	16d00047	um10357	0	um10357	16d00047	0
BBH	16c00071	um05569	9.00E-49	um05569	16c00071	2.00E-48
BBH	16c00054	um05542	0	um05542	16c00054	0
BBH	16d00042	um05528	1.00E-154	um05528	16d00042	1.00E-155
BBH	16c00034	um05505	1.00E-46	um05505	16c00034	2.00E-45
NNN	16c00088	um05602	1.00E-21	um05602	16d00084	0
BBH	16c00061	um11794	1.00E-36	um11794	16c00061	4.00E-35
BBH	16d00039	um05520	4.00E-61	um05520	16d00039	9.00E-58
BBH	16d00022	um05480	0	um05480	16d00022	0
BBH	16d00027	um10758.2	1.00E-62	um10758.2	16d00027	6.00E-71
BBH	16d00015	um05466	1.00E-117	um05466	16d00015	1.00E-119
BBH	16c00023	um10753	8.00E-44	um10753	16c00023	9.00E-44
BBH	16d00081	um05601	0	um05601	16d00081	0
BBH	16c00091	um05609	0	um05609	16c00091	0
BBH	16d00053	um05545	7.00E-83	um05545	16d00053	1.00E-94
BBH	16d00061	um05564	0	um05564	16d00061	0
BBH	16c00057	um05549	0	um05549	16c00057	0
BBH	16d00062	um10895	2.00E-82	um10895	16d00062	1.00E-141
BBH	16d00009	um05449	0	um05449	16d00009	0
BBH	16c00015	um05465	2.00E-72	um05465	16c00015	3.00E-90
BBH	16c00076	um05581	0	um05581	16c00076	0
BBH	16c00084	um11181	1.00E-173	um11181	16c00084	1.00E-168
BBH	16c00051	um10356	1.00E-140	um10356	16c00051	1.00E-136
BBH	16c00010	um05445	8.00E-73	um05445	16c00010	4.00E-72
BBH	16c00075	um11176	2.00E-29	um11176	16c00075	2.00E-30
BBH	16d00079	um05598	2.00E-77	um05598	16d00079	2.00E-77
NNN	16d00080	um10846	4.00E-65	um10846	11c00016	1.00E-178
NNN	16d00069	um11177	2.00E-60	um11177	16d00068	1.00E-65
BBH	16d00016	um05467	1.00E-169	um05467	16d00016	1.00E-163
BBH	16d00013	um05458	0	um05458	16d00013	0

BBH	16d00067	um05579	0	um05579	16d00067	0
BBH	16c00024	um05486	5.00E-98	um05486	16c00024	1.00E-102
BBH	16c00029	um05495	5.00E-41	um05495	16c00029	1.00E-42
BBH	16d00054	um05547	1.00E-126	um05547	16d00054	1.00E-130
BBH	16c00041	um10350	0	um10350	16c00041	0
BBH	16d00030	um05506	0	um05506	16d00030	0
BBH	16c00031	um10759	1.00E-14	um10759	16c00031	1.00E-15
BBH	16c00052	um05533	1.00E-173	um05533	16c00052	1.00E-171
BBH	16c00058	um11790	0	um11790	16c00058	0
BBH	16c00037	um10348	1.00E-61	um10348	16c00037	2.00E-69
BBH	16d00072	um05596	1.00E-132	um05596	16d00072	1.00E-131
BBH	16c00044	um10351	3.00E-77	um10351	16c00044	2.00E-71
BBH	16d00078	um05586	1.00E-158	um05586	16d00078	1.00E-158
BBH	16c00065	um11795	2.00E-59	um11795	16c00065	9.00E-66
BBH	16d00049	um05538	1.00E-138	um05538	16d00049	1.00E-139
BBH	16d00051	um05541	7.00E-22	um05541	16d00051	2.00E-21
BBH	16d00020	um05475	2.00E-53	um05475	16d00020	1.00E-47
BBH	16c00083	um05597	1.00E-159	um05597	16c00083	1.00E-164
BBH	16d00014	um10746.2	1.00E-115	um10746.2	16d00014	1.00E-115
BBH	16d00068	um11177	5.00E-66	um11177	16d00068	1.00E-65
BBH	16d00071	um05584	0	um05584	16d00071	0
BBH	16d00024	um05485	0	um05485	16d00024	0
NNN	16d00085	um05610	0	um05610	11c00016	1.00E-110
BBH	16c00021	um05481	0	um05481	16c00021	0
BBH	16c00018	um10749	0	um10749	16c00018	0
BBH	16c00032	um05498	0	um05498	16c00032	0
BBH	16d00029	um05507	1.00E-163	um05507	16d00029	1.00E-163
BBH	16c00049	um05531	0	um05531	16c00049	0
BBH	16c00007	um11575	4.00E-93	um11575	16c00007	3.00E-98
BBH	16d00040	um05525	3.00E-80	um05525	16d00040	1.00E-81
BBH	16d00059	um05555	2.00E-47	um05555	16d00059	4.00E-43
BBH	16c00086	um05604	1.00E-53	um05604	16c00086	2.00E-53
BBH	16c00038	um05512	2.00E-82	um05512	16c00038	2.00E-70
BBH	16d00052	um05543	1.00E-168	um05543	16d00052	1.00E-162
BBH	16d00011	um05453	1.00E-89	um05453	16d00011	3.00E-97
BBH	16d00057	um11791	8.00E-81	um11791	16d00057	4.00E-80
BBH	16d00036	um05511	1.00E-109	um05511	16d00036	1.00E-109
BBH	16d00026	um05494	0	um05494	16d00026	0
BBH	16d00065	um05574	1.00E-115	um05574	16d00065	1.00E-110
BBH	16c00017	um05470	0	um05470	16c00017	1.00E-174
BBH	16c00047	um10352	7.00E-50	um10352	16c00047	2.00E-49
BBH	16c00055	um05544	0	um05544	16c00055	0
BBH	16d00004	um11113	5.00E-87	um11113	16d00004	1.00E-100
BBH	16c00012	um05450	0	um05450	16c00012	0
BBH	16d00010	um05452.2	3.00E-93	um05452.2	16d00010	1.00E-102
BBH	16d00008	um05446	1.00E-104	um05446	16d00008	3.00E-97
BBH	16c00050	um10359	7.00E-67	um10359	16c00050	7.00E-67
BBH	16c00006	um11574	7.00E-59	um11574	16c00006	3.00E-59
BBH	16d00035	um05509	6.00E-60	um05509	16d00035	5.00E-56
BBH	16d00074	um05595	4.00E-54	um05595	16d00074	5.00E-55
BBH	16c00042	um05521	0	um05521	16c00042	0
BBH	16d00032	um05502	6.00E-86	um05502	16d00032	3.00E-86
BBH	16d00023	um05482	0	um05482	16d00023	0
BBH	16d00019	um05473	1.00E-160	um05473	16d00019	1.00E-151
BBH	16d00012	um11577	0	um11577	16d00012	0
BBH	16c00022	um12304	4.00E-45	um12304	16c00022	7.00E-44
BBH	16d00058	um05554	1.00E-100	um05554	16d00058	1.00E-118
BBH	16c00027	um10756	1.00E-126	um10756	16c00027	1.00E-126
BBH	16c00035	um05503	0	um05503	16c00035	0
BBH	16c00040	um10349	3.00E-20	um10349	16c00040	2.00E-18
BBH	16c00009	um05443	0	um05443	16c00009	0
BBH	16c00011	um05447	2.00E-99	um05447	16c00011	2.00E-94

BBH	16d00066	um05577	0	um05577	16d00066	0
BBH	16d00038	um05518	1.00E-59	um05518	16d00038	9.00E-63
BBH	16c00085	um05606	4.00E-88	um05606	16c00085	3.00E-88
BBH	16c00056	um05546	1.00E-134	um05546	16c00056	1.00E-135
BBH	16c00005	um05439	2.00E-84	um05439	16c00005	4.00E-85
BBH	16c00074	um05575	1.00E-167	um05575	16c00074	1.00E-174
BBH	16c00066	um05562	1.00E-117	um05562	16c00066	1.00E-108
BBH	16c00079	um05591	1.00E-85	um05591	16c00079	6.00E-82
BBH	16d00017	um10748	0	um10748	16d00017	0
BBH	16d00028	um05499	1.00E-122	um05499	16d00028	1.00E-114
BBH	16d00055	um05548	0	um05548	16d00055	0
BBH	16c00028	um05493	4.00E-70	um05493	16c00028	3.00E-75
BBH	16c00036	um10347	0	um10347	16c00036	0
BBH	16d00075	um05593	2.00E-22	um05593	16d00075	1.00E-15
BBH	16c00081	um05589	3.00E-40	um05589	16c00081	1.00E-40
BBH	16c00069	um12307	0	um12307	16c00069	0
BBH	16d00043	um10353	7.00E-79	um10353	16d00043	1.00E-74
BBH	16c00064	um05560	0	um05560	16c00064	0
NNN	16d00002	um03403.2	3.00E-11	um03403.2	8d00113	0
BBH	16d00006	um05442	1.00E-148	um05442	16d00006	1.00E-139
BBH	16d00046	um05537	0	um05537	16d00046	1.00E-168
BBH	16c00019	um10750	0	um10750	16c00019	0
BBH	16c00072	um05571	1.00E-147	um05571	16c00072	1.00E-147
BBH	16c00004	um04490	7.00E-38	um04490	16c00004	2.00E-37
BBH	16d00031	um05504	0	um05504	16d00031	0
BBH	16d00060	um05559	0	um05559	16d00060	0
BBH	16c00014	um11578	1.00E-117	um11578	16c00014	1.00E-124
BBH	16d00025	um05489	1.00E-161	um05489	16d00025	1.00E-177
BBH	16c00025	um10755	1.00E-39	um10755	16c00025	7.00E-39
BBH	16c00092	um12309	0	um12309	16c00092	0
NNN	16d00084	um01656	3.00E-70	um01656	22c00121	0
BBH	16d00037	um05514	1.00E-175	um05514	16d00037	1.00E-176
NNN	16d00034	um05509	2.00E-45	um05509	16d00035	5.00E-56
BBH	16c00063	um05558	0	um05558	16c00063	0
BBH	16c00087	um05603	1.00E-162	um05603	16c00087	1.00E-162
BBH	16d00005	um05438	1.00E-162	um05438	16d00005	0
BBH	16c00053	um05539	5.00E-96	um05539	16c00053	6.00E-95
BBH	16d00045	um12306	7.00E-97	um12306	16d00045	3.00E-97
BBH	16c00073	um05572	1.00E-159	um05572	16c00073	1.00E-158
BBH	16d00044	um10354	1.00E-130	um10354	16d00044	1.00E-124
NNN	16d00001	um10815	4.00E-99	um10815	22d00064	0
BBH	16d00063	um05568	0	um05568	16d00063	0
BBH	16c00020	um05478	1.00E-101	um05478	16c00020	7.00E-97
BBH	16c00013	um05454	1.00E-116	um05454	16c00013	1.00E-120
BBH	18c00022	um10420	6.00E-39	um10420	18c00022	7.00E-39
NNN	18d00020	um10423	1.00E-74			
BBH	18c00030	um10414	2.00E-69	um10414	18c00030	7.00E-75
BBH	18d00084	um04157	2.00E-97	um04157	18d00084	3.00E-99
NNN	18c00046	um04220	1.00E-39	um04220	18c00047	3.00E-88
NNN	18c00012	um04293	9.00E-21	um04293	18c00006	0
BBH	18c00070	um10893	1.00E-119	um10893	18c00070	1.00E-119
BBH	18c00077	um10889	0	um10889	18c00077	0
BBH	18c00064	um11121	9.00E-25	um11121	18c00064	1.00E-24
BBH	18d00042	um04225.2	0	um04225.2	18d00042	0
BBH	18c00002	um04299	0	um04299	18c00002	0
BBH	18c00027	um04251	1.00E-169	um04251	18c00027	1.00E-165
BBH	18d00057	um11129	2.00E-65	um11129	18d00057	2.00E-56
BBH	18c00038	um04229	2.00E-37	um04229	18c00038	4.00E-37
BBH	18c00085	um04147	0	um04147	18c00085	0
NNN	18d00095	um12254	0			
BBH	18d00109	um04092	2.00E-83	um04092	18d00109	7.00E-83
BBH	18d00060	um04198	1.00E-127	um04198	18d00060	1.00E-122

BBH	18c00090	um15062	2.00E-75	um15062	18c00090	6.00E-72
NNN	18d00113	um11540	9.00E-69	um11540	27d00074	0
BBH	18d00030	um04250	0	um04250	18d00030	0
BBH	18d00036	um10412	0	um10412	18d00036	0
BBH	18d00102	um04126	0	um04126	18d00102	1.00E-165
NNN	18c00062	um04192	0			
BBH	18d00083	um04165	5.00E-38	um04165	18d00083	5.00E-38
BBH	18d00028	um10417	0	um10417	18d00028	0
BBH	18c00043	um10403	7.00E-13	um10403	18c00043	2.00E-12
BBH	18d00079	um04173	0	um04173	18d00079	0
BBH	18d00021	um10421	1.00E-126	um10421	18d00021	1.00E-117
BBH	18d00094	um10886.2	3.00E-91	um10886.2	18d00094	2.00E-92
BBH	18d00008	um04292	3.00E-66	um04292	18d00008	3.00E-66
BBH	18c00019	um04271	2.00E-63	um04271	18c00019	5.00E-63
BBH	18c00055	um04203	5.00E-77	um04203	18c00055	8.00E-81
BBH	18c00086	um04139	0	um04139	18c00086	0
NNN	18d00016	um04277	1.00E-74			
BBH	18c00061	um11125	1.00E-105	um11125	18c00061	1.00E-106
BBH	18c00028	um04249	0	um04249	18c00028	0
BBH	18c00050	um04214	1.00E-129	um04214	18c00050	1.00E-134
BBH	18c00096	um04114	0	um04114	18c00096	0
BBH	18c00037	um10408.2	6.00E-94	um10408.2	18c00037	2.00E-88
BBH	18d00054	um04208	0	um04208	18d00054	0
BBH	18c00003	um04297	1.00E-140	um04297	18c00003	1.00E-132
BBH	18d00031	um04248	2.00E-18	um04248	18d00031	1.00E-14
NNN	18d00101	um02607	1.00E-67	um02607	5d00105	0
BBH	18d00037	um04238	1.00E-148	um04238	18d00037	1.00E-142
BBH	18d00038	um04236	0	um04236	18d00038	0
BBH	18c00079	um04159	1.00E-123	um04159	18c00079	1.00E-123
BBH	18c00069	um04180	7.00E-88	um04180	18c00069	2.00E-93
BBH	18d00061	um04193.2	0	um04193.2	18d00061	0
BBH	18c00081	um04151	0	um04151	18c00081	0
BBH	18d00052	um04211	1.00E-76	um04211	18d00052	2.00E-80
BBH	18c00083	um10888	1.00E-81	um10888	18c00083	3.00E-90
NNN	18c00072	um04171	2.00E-87			
BBH	18d00048	um04217	4.00E-39	um04217	18d00048	3.00E-44
NNN	18d00009	um10433	1.00E-18	um10433	18d00010	1.00E-24
NNN	18d00071	um04181	0			
BBH	18d00040	um04232	0	um04232	18d00040	0
BBH	18c00018	um10426	1.00E-180	um10426	18c00018	1.00E-178
NNN	18c00010	um02490	2.00E-86	um02490	12c00135	0
BBH	18c00040	um04227	1.00E-130	um04227	18c00040	1.00E-134
BBH	18d00104	um04118	1.00E-172	um04118	18d00104	1.00E-163
BBH	18c00057	um04201	0	um04201	18c00057	0
BBH	18c00048	um04219.2	1.00E-164	um04219.2	18c00048	1.00E-156
BBH	18d00086	um04154	1.00E-54	um04154	18d00086	9.00E-55
BBH	18d00001	um10436	1.00E-174	um10436	18d00001	1.00E-174
BBH	18c00009	um04282	0	um04282	18c00009	0
BBH	18d00034	um10413	1.00E-19	um10413	18d00034	7.00E-26
BBH	18c00032	um04239	6.00E-42	um04239	18c00032	3.00E-45
NNN	18d00062	um11271	1.00E-56	um11271	7d00250	0
BBH	18d00017	um04275	0	um04275	18d00017	0
BBH	18d00026	um04253	0	um04253	18d00026	0
BBH	18d00097	um04136	0	um04136	18d00097	0
BBH	18d00055	um11131	1.00E-18	um11131	18d00055	3.00E-16
NNN	18c00095	um04115	0			
BBH	18c00074	um04166	3.00E-64	um04166	18c00074	3.00E-68
BBH	18c00020	um04269	3.00E-56	um04269	18c00020	2.00E-62
NNN	18d00049	um04216	5.00E-48	um04216	18d00050	0
BBH	18d00078	um10892	1.00E-134	um10892	18d00078	1.00E-125
BBH	18c00051	um04212	0	um04212	18c00051	0
BBH	18d00070	um15011.2	0	um15011.2	18d00070	0

BBH	18c00063	um04189	0	um04189	18c00063	0
BBH	18c00017	um10428	1.00E-157	um10428	18c00017	1.00E-158
BBH	18c00071	um04169	0	um04169	18c00071	0
BBH	18c00084	um04150	0	um04150	18c00084	0
BBH	18d00053	um04210	0	um04210	18d00053	0
BBH	18c00045	um04223	1.00E-105	um04223	18c00045	1.00E-116
BBH	18c00058	um11126	0	um11126	18c00058	0
BBH	18d00043	um10407	1.00E-86	um10407	18d00043	5.00E-78
BBH	18c00073	um04172.2	6.00E-83	um04172.2	18c00073	5.00E-88
BBH	18c00094	um04117	1.00E-179	um04117	18c00094	0
BBH	18d00096	um10884	1.00E-143	um10884	18d00096	1.00E-149
BBH	18d00035	um04242	0	um04242	18d00035	0
BBH	18d00063	um04190	1.00E-118	um04190	18d00063	1.00E-115
BBH	18c00001	um04301	0	um04301	18c00001	0
BBH	18c00031	um04241	8.00E-24	um04241	18c00031	4.00E-29
BBH	18c00039	um04228	1.00E-129	um04228	18c00039	1.00E-144
BBH	18d00103	um04125	1.00E-107	um04125	18d00103	1.00E-100
BBH	18c00026	um04254	0	um04254	18c00026	0
BBH	18c00052	um04209	0	um04209	18c00052	0
BBH	18d00033	um04244	2.00E-46	um04244	18d00033	2.00E-46
BBH	18d00068	um04184	1.00E-112	um04184	18d00068	3.00E-75
BBH	18d00012	um10432.2	0	um10432.2	18d00012	0
BBH	18d00019	um04268	1.00E-175	um04268	18d00019	0
NNN	18d00046	um11133	3.00E-39			
NNN	18d00106	um04092	5.00E-82	um04092	18d00109	7.00E-83
BBH	18d00050	um04216	0	um04216	18d00050	0
BBH	18c00060	um04196	1.00E-84	um04196	18c00060	9.00E-84
BBH	18d00007	um04294	1.00E-111	um04294	18d00007	1.00E-99
BBH	18d00091	um04149	1.00E-36	um04149	18d00091	2.00E-41
BBH	18c00034	um12255	0	um12255	18c00034	0
BBH	18d00075	um04177.2	0	um04177.2	18d00075	0
BBH	18c00007	um04290	0	um04290	18c00007	0
BBH	18c00093	um04127	0	um04127	18c00093	0
NNN	18c00042	um04224	0			
BBH	18c00097	um11115	0	um11115	18c00097	0
BBH	18c00076	um04162	0	um04162	18c00076	1.00E-153
BBH	18c00089	um04133	6.00E-86	um04133	18c00089	7.00E-93
BBH	18d00099	um04132	0	um04132	18d00099	0
BBH	18d00004	um10434	0	um10434	18d00004	0
BBH	18d00088	um04161	0	um04161	18d00088	0
NNN	18d00073	um04179	7.00E-33	um04179	18d00072	6.00E-34
BBH	18d00081	um04175	0	um04175	18d00081	0
BBH	18c00015	um10431	1.00E-156	um10431	18c00015	1.00E-150
BBH	18d00015	um04278.2	0	um04278.2	18d00015	0
BBH	18d00051	um04213	0	um04213	18d00051	0
BBH	18c00008	um04288	1.00E-124	um04288	18c00008	1.00E-124
BBH	18d00013	um10429	0	um10429	18d00013	0
NNN	18c00065	um04503	4.00E-39			
NNN	18d00041	um11301	4.00E-59	um11301	7d00180	0
BBH	18d00090	um04152	0	um04152	18d00090	0
BBH	18c00092	um04128	1.00E-177	um04128	18c00092	0
BBH	18c00023	um04262	1.00E-143	um04262	18c00023	1.00E-144
BBH	18d00098	um04134	0	um04134	18d00098	0
BBH	18c00033	um04237	2.00E-48	um04237	18c00033	2.00E-48
BBH	18c00075	um04164	0	um04164	18c00075	0
BBH	18d00005	um04291	0	um04291	18d00005	0
BBH	18c00016	um10430	2.00E-46	um10430	18c00016	5.00E-60
BBH	18c00047	um04220	5.00E-89	um04220	18c00047	3.00E-88
BBH	18d00087	um04158	0	um04158	18d00087	0
BBH	18d00080	um04174	1.00E-109	um04174	18d00080	0
NNN	18c00104	um03416	8.00E-39	um03416	8c00113	1.00E-134
BBH	18d00105	um04116.2	1.00E-116	um04116.2	18d00105	1.00E-123

BBH	18c00066	um11120	4.00E-36	um11120	18c00066	4.00E-36
BBH	18c00059	um04197	0	um04197	18c00059	0
BBH	18c00041	um04226	0	um04226	18c00041	0
BBH	18d00072	um04179	2.00E-34	um04179	18d00072	6.00E-34
NNN	18c00105	um04304	1.00E-124	um04304	24d00061	0
NNN	18c00091	um04130	1.00E-92	um04130	22d00015	1.00E-116
BBH	18d00093	um04146	0	um04146	18d00093	0
BBH	18d00022	um10419	0	um10419	18d00022	0
BBH	18c00088	um10882	3.00E-80	um10882	18c00088	1.00E-80
BBH	18c00056	um04202	0	um04202	18c00056	0
BBH	18d00077	um04168	4.00E-97	um04168	18d00077	1.00E-108
BBH	18c00024	um04260	0	um04260	18c00024	0
BBH	18d00067	um04185	3.00E-37	um04185	18d00067	6.00E-34
BBH	18c00029	um04247	0	um04247	18c00029	0
BBH	18d00010	um10433	2.00E-24	um10433	18d00010	1.00E-24
BBH	18d00056	um04206	0	um04206	18d00056	0
BBH	18d00002	um04300	4.00E-46	um04300	18d00002	3.00E-49
BBH	18d00059	um11127	0	um11127	18d00059	0
BBH	18c00004	um10435	1.00E-169	um10435	18c00004	1.00E-175
BBH	18d00045	um10405.2	1.00E-100	um10405.2	18d00045	1.00E-98
BBH	18c00013	um04283	1.00E-121	um04283	18c00013	1.00E-136
BBH	18c00087	um04138	1.00E-164	um04138	18c00087	1.00E-164
BBH	18c00036	um10410	0	um10410	18c00036	0
BBH	18d00029	um10415	0	um10415	18d00029	0
NNN	18d00047	um04218	0			
BBH	18c00080	um04163	7.00E-18	um04163	18c00080	5.00E-17
BBH	18c00078	um04156	0	um04156	18c00078	0
BBH	18d00039	um04234	1.00E-154	um04234	18d00039	1.00E-151
NNN	18d00076	um04176	0			
BBH	18c00006	um04293	0	um04293	18c00006	0
NNN	18c00011	um06374	3.00E-31	um06374	1c00003	1.00E-174
BBH	18c00053	um11130	3.00E-64	um11130	18c00053	3.00E-64
BBH	18d00023	um04259	1.00E-132	um04259	18d00023	1.00E-136
BBH	18d00032	um12256	1.00E-127	um12256	18d00032	1.00E-128
BBH	18c00021	um10424.2	0	um10424.2	18c00021	0
BBH	18d00064	um04188	0	um04188	18d00064	0
BBH	18d00024	um04258	0	um04258	18d00024	0
BBH	18d00092	um04148	1.00E-114	um04148	18d00092	1.00E-118
BBH	18d00011	um04285	0	um04285	18d00011	0
NNN	18c00102	um04503	4.00E-42			
BBH	18d00044	um10406	0	um10406	18d00044	0
BBH	18c00067	um04182	0	um04182	18c00067	0
BBH	18d00003	um04298	0	um04298	18d00003	0
BBH	18c00049	um11132	1.00E-119	um11132	18c00049	1.00E-130
NNN	18c00044	um10403	3.00E-11	um10403	18c00043	2.00E-12
BBH	18d00018	um04270	0	um04270	18d00018	0
BBH	18c00098	um11114	0	um11114	18c00098	0
BBH	18c00014	um04284	1.00E-123	um04284	18c00014	1.00E-117
BBH	18d00082	um04167	2.00E-96	um04167	18d00082	1.00E-98
BBH	19c00092	um10321	0	um10321	19c00092	0
BBH	19c00124	um02869	0	um02869	19c00124	0
BBH	19c00108	um02902	0	um02902	19c00108	0
BBH	19d00044	um10369	1.00E-139	um10369	19d00044	1.00E-139
BBH	19c00128	um02864	0	um02864	19c00128	0
BBH	19d00096	um10331	3.00E-81	um10331	19d00096	6.00E-82
BBH	19c00043	um10370	1.00E-176	um10370	19c00043	1.00E-177
BBH	19d00108	um02923	1.00E-124	um02923	19d00108	1.00E-124
BBH	19d00028	um03066	3.00E-70	um03066	19d00028	2.00E-86
BBH	19c00141	um11475	1.00E-16	um11475	19c00141	4.00E-16
BBH	19d00114	um02913	1.00E-97	um02913	19d00114	5.00E-98
BBH	19c00038	um03049	0	um03049	19c00038	0
BBH	19c00018	um03085	3.00E-93	um03085	19c00018	2.00E-94

BBH	19c00013	um03097	2.00E-49	um03097	19c00013	1.00E-48
BBH	19c00074	um10339	0	um10339	19c00074	0
BBH	19d00070	um12204	1.00E-113	um12204	19d00070	1.00E-118
BBH	19c00054	um03015	7.00E-77	um03015	19c00054	6.00E-82
BBH	19d00061	um03019.2	2.00E-87	um03019.2	19d00061	1.00E-85
BBH	19c00048	um03007	0	um03007	19c00048	0
BBH	19d00065	um03002	1.00E-118	um03002	19d00065	1.00E-106
BBH	19d00055	um11939	1.00E-51	um11939	19d00055	2.00E-45
BBH	19c00083	um02951	0	um02951	19c00083	0
BBH	19c00023	um12207	4.00E-47	um12207	19c00023	4.00E-49
BBH	19d00097	um02944	0	um02944	19d00097	0
BBH	19d00015	um11944	0	um11944	19d00015	1.00E-174
BBH	19c00042	um03058	0	um03058	19c00042	0
BBH	19d00118	um02905	0	um02905	19d00118	0
BBH	19c00098	um02924	3.00E-71	um02924	19c00098	2.00E-69
BBH	19d00152	um02847	1.00E-175	um02847	19d00152	1.00E-171
BBH	19d00051	um10362	1.00E-51	um10362	19d00051	1.00E-51
BBH	19d00083	um02968	5.00E-51	um02968	19d00083	4.00E-51
BBH	19c00125	um02867	1.00E-58	um02867	19c00125	2.00E-61
BBH	19c00084	um02948	2.00E-50	um02948	19c00084	2.00E-46
BBH	19c00113	um02884	3.00E-73	um02884	19c00113	5.00E-75
BBH	19c00091	um02935.2	3.00E-47	um02935.2	19c00091	3.00E-43
BBH	19c00020	um10375	2.00E-49	um10375	19c00020	1.00E-49
BBH	19c00055	um10361	1.00E-33	um10361	19c00055	3.00E-34
NNN	19c00040	um02791	1.00E-104	um02791	5d00005	0
BBH	19d00020	um03082	0	um03082	19d00020	0
BBH	19d00029	um03065	1.00E-41	um03065	19d00029	2.00E-44
NNN	19c00049	um00210	1.00E-141			
BBH	19d00126	um11497	0	um11497	19d00126	0
NNN	19c00132	um02135	4.00E-29	um02135	6d00088	1.00E-30
BBH	19d00129	um11638	1.00E-91	um11638	19d00129	1.00E-91
NNN	19d00062	um03018	1.00E-106			
BBH	19c00129	um02862	1.00E-180	um02862	19c00129	0
BBH	19d00012	um03096	0	um03096	19d00012	0
BBH	19d00056	um03011	0	um03011	19d00056	0
BBH	19c00115	um02889	1.00E-101	um02889	19c00115	1.00E-97
BBH	19c00010	um11948	4.00E-26	um11948	19c00010	5.00E-27
BBH	19d00109	um02921	1.00E-113	um02921	19d00109	1.00E-114
BBH	19d00066	um03000	1.00E-104	um03000	19d00066	1.00E-97
BBH	19d00106	um10317.2	6.00E-45	um10317.2	19d00106	7.00E-52
BBH	19d00117	um11501	0	um11501	19d00117	0
BBH	19c00064	um02993	0	um02993	19c00064	0
BBH	19d00014	um11945	0	um11945	19d00014	0
BBH	19c00099	um02922	0	um02922	19c00099	0
NNN	19d00153	um02845.2	0			
NNN	19d00052	um03025	6.00E-82	um03025	19d00053	0
BBH	19d00162	um11476	0	um11476	19d00162	0
BBH	19c00077	um12201	1.00E-172	um12201	19c00077	0
BBH	19d00141	um02863	1.00E-112	um02863	19d00141	1.00E-125
BBH	19c00082	um10332	0	um10332	19c00082	0
BBH	19c00112	um11498	1.00E-177	um11498	19c00112	1.00E-177
BBH	19c00032	um03064	1.00E-127	um03064	19c00032	1.00E-137
BBH	19c00085	um10330	0	um10330	19c00085	0
BBH	19c00126	um11486	3.00E-63	um11486	19c00126	4.00E-73
NNN	19d00037	um03049	9.00E-11	um03049	19c00038	0
BBH	19d00067	um02998	0	um02998	19d00067	0
BBH	19d00145	um02855	0	um02855	19d00145	0
BBH	19c00014	um03095	1.00E-102	um03095	19c00014	1.00E-112
BBH	19d00079	um02976.2	0	um02976.2	19d00079	0
BBH	19c00103	um02910	1.00E-117	um02910	19c00103	1.00E-120
BBH	19d00021	um03081	0	um03081	19d00021	1.00E-176
BBH	19d00010	um03102	0	um03102	19d00010	0

BBH	19c00066	um02990	1.00E-131	um02990	19c00066	1.00E-129
BBH	19d00007	um10630	0	um10630	19d00007	0
BBH	19d00042	um10371	1.00E-176	um10371	19d00042	1.00E-173
BBH	19d00049	um03029	1.00E-107	um03029	19d00049	1.00E-108
BBH	19d00136	um02876	4.00E-35	um02876	19d00136	1.00E-32
BBH	19d00082	um10338	1.00E-113	um10338	19d00082	1.00E-120
BBH	19d00018	um03088	2.00E-87	um03088	19d00018	3.00E-89
NNN	19c00135	um11482	5.00E-29	um11482	7d00279	3.00E-19
BBH	19d00098	um10327	4.00E-85	um10327	19d00098	4.00E-90
BBH	19d00011	um03099	2.00E-64	um03099	19d00011	1.00E-47
BBH	19d00101	um02936	0	um02936	19d00101	0
BBH	19c00144	um02827	0	um02827	19c00144	0
BBH	19c00147	um02823	0	um02823	19c00147	0
BBH	19c00123	um11488	2.00E-43	um11488	19c00123	3.00E-43
BBH	19d00001	um05070	1.00E-139	um05070	19d00001	1.00E-139
BBH	19c00006	um03108	0	um03108	19c00006	0
BBH	19c00045	um03034	0	um03034	19c00045	0
BBH	19c00111	um11496	1.00E-93	um11496	19c00111	3.00E-93
BBH	19c00081	um10333	1.00E-70	um10333	19c00081	1.00E-77
BBH	19c00086	um02945	0	um02945	19c00086	0
BBH	19d00004	um03115	0	um03115	19d00004	0
BBH	19d00156	um11481	1.00E-141	um11481	19d00156	1.00E-134
BBH	19d00099	um02938	0	um02938	19d00099	0
BBH	19d00036	um10365	0	um10365	19d00036	0
BBH	19d00121	um02901	1.00E-15	um02901	19d00121	4.00E-19
BBH	19c00025	um10374	1.00E-36	um10374	19c00025	1.00E-36
BBH	19c00053	um11941	0	um11941	19c00053	0
BBH	19d00068	um10344	1.00E-109	um10344	19d00068	1.00E-109
BBH	19c00033	um03063	0	um03063	19c00033	0
NNN	19d00057	um03013	0			
BBH	19d00134	um02878.2	5.00E-78	um02878.2	19d00134	1.00E-74
NNN	19d00154	um02844	5.00E-91			
BBH	19d00043	um03057	1.00E-144	um03057	19d00043	1.00E-137
BBH	19c00030	um03070	0	um03070	19c00030	0
BBH	19c00127	um02865	1.00E-150	um02865	19c00127	1.00E-163
BBH	19d00163	um02830	0	um02830	19d00163	0
BBH	19d00053	um03025	0	um03025	19d00053	0
BBH	19d00022	um03078	0	um03078	19d00022	0
BBH	19c00012	um03098	1.00E-139	um03098	19c00012	1.00E-137
BBH	19c00120	um12197	2.00E-38	um12197	19c00120	3.00E-38
BBH	19c00067	um02989	0	um02989	19c00067	0
BBH	19d00139	um11487	8.00E-84	um11487	19d00139	3.00E-86
BBH	19c00015	um11943.2	5.00E-76	um11943.2	19c00015	1.00E-86
BBH	19d00078	um02979	9.00E-93	um02979	19d00078	5.00E-84
BBH	19c00102	um02912	0	um02912	19c00102	0
BBH	19c00140	um11478	4.00E-71	um11478	19c00140	3.00E-72
BBH	19d00144	um02857	0	um02857	19d00144	0
BBH	19d00090	um10334	1.00E-125	um10334	19d00090	1.00E-125
BBH	19c00094	um10320.2	0	um10320.2	19c00094	0
BBH	19c00008	um10629.2	1.00E-109	um10629.2	19c00008	1.00E-109
NNN	19c00075	um10337	4.00E-93	um10337	7d00279	8.00E-19
BBH	19c00080	um02957	1.00E-122	um02957	19c00080	1.00E-116
BBH	19c00087	um10329	0	um10329	19c00087	0
BBH	19d00084	um02966	7.00E-97	um02966	19d00084	1.00E-103
BBH	19c00009	um10627	1.00E-108	um10627	19c00009	1.00E-113
BBH	19d00039	um10367	0	um10367	19d00039	0
BBH	19d00058	um15002.2	0	um15002.2	19d00058	0
BBH	19d00013	um11946	0	um11946	19d00013	0
BBH	19c00146	um02825	0	um02825	19c00146	0
BBH	19c00022	um03079	0	um03079	19c00022	0
BBH	19d00091	um02954	0	um02954	19d00091	0
BBH	19d00128	um02900	0	um02900	19d00128	0

NNN	19d00138	um01054	5.00E-72	um01054	7d00254	0
BBH	19d00155	um02843	0	um02843	19d00155	0
BBH	19c00035	um03036	1.00E-112	um03036	19c00035	1.00E-124
BBH	19c00110	um11494	9.00E-93	um11494	19c00110	4.00E-96
BBH	19d00054	um03023	1.00E-112	um03023	19d00054	1.00E-109
BBH	19d00157	um02840	1.00E-141	um02840	19d00157	1.00E-143
BBH	19d00005	um10633	1.00E-80	um10633	19d00005	3.00E-90
BBH	19d00069	um10343	0	um10343	19d00069	0
BBH	19d00160	um11477	0	um11477	19d00160	0
BBH	19d00125	um02896	0	um02896	19d00125	0
BBH	19d00059	um03016	0	um03016	19d00059	0
BBH	19d00031	um10364	0	um10364	19d00031	0
BBH	19c00068	um02985	1.00E-146	um02985	19c00068	1.00E-141
BBH	19d00086	um10335	9.00E-41	um10335	19d00086	1.00E-51
BBH	19c00031	um03067	1.00E-99	um03067	19c00031	2.00E-97
BBH	19c00137	um11480	0	um11480	19c00137	0
BBH	19c00026	um03073	2.00E-79	um03073	19c00026	3.00E-74
NNN	19c00150	um03407	3.00E-27	um03407	8d00115	0
BBH	19c00121	um11490	0	um11490	19c00121	0
BBH	19c00073	um02972	1.00E-156	um02972	19c00073	1.00E-134
BBH	19d00159	um02835	0	um02835	19d00159	0
BBH	19d00050	um03028	0	um03028	19d00050	0
BBH	19d00087	um02961	0	um02961	19d00087	0
BBH	19d00123	um11495	7.00E-60	um11495	19d00123	1.00E-63
BBH	19c00116	um15086.2	0	um15086.2	19c00116	0
BBH	19d00040	um10368	0	um10368	19d00040	0
BBH	19d00161	um02833	1.00E-100	um02833	19d00161	6.00E-95
BBH	19d00074	um02984	0	um02984	19d00074	0
NNN	19d00092	um12200	7.00E-18	um12200	19d00093	0
BBH	19c00034	um03040	1.00E-109	um03040	19c00034	1.00E-109
NNN	19d00104	um10319	2.00E-93			
BBH	19c00131	um11485	2.00E-67	um11485	19c00131	4.00E-77
BBH	19c00047	um03027	0	um03027	19c00047	0
BBH	19c00058	um03006	1.00E-143	um03006	19c00058	1.00E-139
BBH	19d00111	um15000.2	0	um15000.2	19d00111	0
NNN	19c00016	um10378	0			
BBH	19d00103	um02933	4.00E-48	um02933	19d00103	3.00E-50
BBH	19d00047	um03032	3.00E-75	um03032	19d00047	1.00E-74
BBH	19c00105	um11500	1.00E-135	um11500	19c00105	0
BBH	19c00004	um00822	1.00E-178	um00822	19c00004	1.00E-177
BBH	19d00024	um03071	0	um03071	19d00024	0
BBH	19c00003	um03113	0	um03113	19c00003	0
BBH	19c00136	um02846.2	0	um02846.2	19c00136	0
BBH	19c00143	um02829	0	um02829	19c00143	0
BBH	19c00079	um02959	5.00E-44	um02959	19c00079	5.00E-44
BBH	19c00051	um03010	1.00E-118	um03010	19c00051	1.00E-124
BBH	19c00118	um02879	1.00E-124	um02879	19c00118	1.00E-146
BBH	19c00060	um03001	1.00E-108	um03001	19c00060	1.00E-107
BBH	19c00070	um02978	1.00E-131	um02978	19c00070	1.00E-133
BBH	19d00019	um10376	1.00E-168	um10376	19d00019	1.00E-170
BBH	19d00006	um10632	6.00E-99	um10632	19d00006	3.00E-94
BBH	19c00027	um10373	1.00E-103	um10373	19c00027	3.00E-92
BBH	19d00030	um03042	4.00E-26	um03042	19d00030	3.00E-29
BBH	19c00134	um11483	5.00E-95	um11483	19c00134	5.00E-95
BBH	19d00132	um02883	8.00E-29	um02883	19d00132	3.00E-29
NNN	19c00072	um10340	1.00E-128			
BBH	19d00085	um10336	0	um10336	19d00085	0
BBH	19d00137	um02874	0	um02874	19d00137	0
NNN	19d00003	um03116	0			
BBH	19d00081	um12202	2.00E-15	um12202	19d00081	2.00E-17
BBH	19c00096	um02929	0	um02929	19c00096	0
BBH	19c00088	um02942	1.00E-140	um02942	19c00088	1.00E-150

BBH	19c00044	um03062	0	um03062	19c00044	0
NNN	19d00135	um02877	0			
BBH	19d00080	um02974	0	um02974	19d00080	0
BBH	19d00041	um03061	1.00E-121	um03061	19d00041	1.00E-126
BBH	19d00105	um10318	2.00E-80	um10318	19d00105	6.00E-86
BBH	19c00078	um02962	0	um02962	19c00078	0
BBH	19c00002	um03114	1.00E-153	um03114	19c00002	1.00E-156
BBH	19c00069	um02983	1.00E-32	um02983	19c00069	2.00E-38
BBH	19d00023	um03074	2.00E-47	um03074	19d00023	2.00E-48
BBH	19d00077	um02980	0	um02980	19d00077	0
BBH	19d00120	um02903	1.00E-112	um02903	19d00120	1.00E-114
BBH	19c00007	um03105	9.00E-22	um03105	19c00007	5.00E-19
BBH	19c00059	um11937	1.00E-76	um11937	19c00059	2.00E-76
BBH	19d00100	um02937	1.00E-133	um02937	19d00100	1.00E-140
BBH	19c00024	um03076	0	um03076	19c00024	0
BBH	19d00048	um12205	0	um12205	19d00048	0
BBH	19c00061	um02999	1.00E-116	um02999	19c00061	1.00E-104
BBH	19d00093	um12200	0	um12200	19d00093	0
BBH	19d00073	um02986	1.00E-92	um02986	19d00073	9.00E-71
BBH	19d00110	um02919	0	um02919	19d00110	0
BBH	19d00113	um02915	0	um02915	19d00113	0
BBH	19c00017	um10377	0	um10377	19c00017	0
BBH	19d00143	um02859	0	um02859	19d00143	0
BBH	19c00148	um11932	0	um11932	19c00148	0
BBH	19c00133	um02850	1.00E-154	um02850	19c00133	1.00E-138
BBH	19c00119	um11491	1.00E-60	um11491	19c00119	9.00E-65
BBH	19d00017	um03089	2.00E-42	um03089	19d00017	6.00E-49
BBH	19c00071	um12203	1.00E-161	um12203	19c00071	1.00E-165
NNN	19d00131	um02888	1.00E-178	um02888	6c00022	4.00E-28
BBH	19d00094	um02950	1.00E-164	um02950	19d00094	1.00E-175
BBH	19c00114	um02886	6.00E-86	um02886	19c00114	1.00E-96
BBH	19d00035	um03045.2	0	um03045.2	19d00035	0
BBH	19d00033	um03037	1.00E-116	um03037	19d00033	1.00E-112
BBH	19d00046	um03033	1.00E-115	um03033	19d00046	1.00E-107
BBH	19c00089	um10326.2	0	um10326.2	19c00089	0
BBH	19c00101	um11934	1.00E-31	um11934	19c00101	8.00E-37
BBH	19c00021	um03080	0	um03080	19c00021	0
BBH	19c00041	um10372	1.00E-144	um10372	19c00041	1.00E-137
BBH	19d00150	um02851	2.00E-19	um02851	19d00150	4.00E-22
BBH	19d00116	um15064	0	um15064	19d00116	0
BBH	19d00089	um02958	0	um02958	19d00089	0
BBH	19c00019	um03083	1.00E-125	um03083	19c00019	1.00E-125
NNN	19d00112	um02917	1.00E-152			
BBH	19d00149	um02852	9.00E-31	um02852	19d00149	1.00E-30
NNN	19d00008	um10628	1.00E-37			
BBH	19d00076	um02981	2.00E-43	um02981	19d00076	2.00E-40
BBH	19c00056	um03020	8.00E-81	um03020	19c00056	2.00E-77
BBH	19c00138	um12196	2.00E-90	um12196	19c00138	1.00E-85
BBH	19c00076	um02967	1.00E-102	um02967	19c00076	1.00E-92
BBH	19d00045	um03035	0	um03035	19d00045	0
BBH	19d00026	um03069	0	um03069	19d00026	0
BBH	19d00063	um03024	0	um03024	19d00063	0
BBH	19c00107	um02904	0	um02904	19c00107	0
BBH	19c00062	um15001	0	um15001	19c00062	0
BBH	19d00072	um10341	0	um10341	19d00072	0
BBH	19d00127	um02899	0	um02899	19d00127	0
BBH	19d00140	um02868	0	um02868	19d00140	0
BBH	19c00001	um03117.2	0	um03117.2	19c00001	0
NNN	19c00036	um03044	0			
BBH	19d00158	um02838.2	2.00E-96	um02838.2	19d00158	2.00E-96
BBH	19d00151	um02849	1.00E-113	um02849	19d00151	1.00E-110
BBH	19d00122	um02891	0	um02891	19d00122	0

BBH	19d00034	um12206	6.00E-38	um12206	19d00034	1.00E-31
BBH	19d00088	um02960	0	um02960	19d00088	0
BBH	19d00142	um02861	2.00E-16	um02861	19d00142	2.00E-17
BBH	19c00005	um03110	1.00E-136	um03110	19c00005	1.00E-127
BBH	19d00016	um03092	1.00E-112	um03092	19d00016	1.00E-109
NNN	19d00102	um10322	0			
BBH	19d00071	um10342	1.00E-93	um10342	19d00071	2.00E-84
BBH	19d00009	um03103	5.00E-73	um03103	19d00009	5.00E-73
BBH	19d00095	um12199	2.00E-58	um12199	19d00095	4.00E-71
BBH	19d00130	um02887	0	um02887	19d00130	0
BBH	19d00164	um02828	0	um02828	19d00164	0
BBH	19d00124	um02895	0	um02895	19d00124	0
BBH	19c00100	um02920	1.00E-123	um02920	19c00100	1.00E-123
BBH	19d00032	um03038	0	um03038	19d00032	0
BBH	19c00109	um02890	1.00E-165	um02890	19c00109	1.00E-165
BBH	19c00039	um10366.2	6.00E-50	um10366.2	19c00039	4.00E-54
BBH	19c00011	um11947	9.00E-29	um11947	19c00011	9.00E-29
NNN	19c00139	um11479	1.00E-151			
BBH	19c00063	um02994	4.00E-91	um02994	19c00063	2.00E-93
BBH	19d00107	um02926	2.00E-54	um02926	19d00107	3.00E-55
BBH	19d00060	um10360	2.00E-39	um10360	19d00060	2.00E-39
BBH	19c00130	um02860	0	um02860	19c00130	0
BBH	19d00027	um03068	1.00E-146	um03068	19d00027	1.00E-147
BBH	19d00064	um03003	0	um03003	19d00064	0
BBH	19d00002	um10636	1.00E-159	um10636	19d00002	1.00E-159
NNN	19c00117	um02881	7.00E-47			
BBH	19d00115	um02911	0	um02911	19d00115	0
BBH	19d00165	um02824	1.00E-131	um02824	19d00165	1.00E-128
BBH	19d00075	um02982	0	um02982	19d00075	0
BBH	19c00057	um03017	1.00E-109	um03017	19c00057	1.00E-124
BBH	19c00142	um11474	1.00E-140	um11474	19c00142	1.00E-150
BBH	19c00090	um10323	4.00E-13	um10323	19c00090	4.00E-16
BBH	19d00133	um02880	2.00E-52	um02880	19d00133	9.00E-71
BBH	19c00046	um10363	2.00E-92	um10363	19c00046	3.00E-87
BBH	19c00050	um03009	1.00E-102	um03009	19c00050	1.00E-109
BBH	20c00048	um06050	0	um06050	20c00048	0
BBH	20c00055	um06040	1.00E-101	um06040	20c00055	1.00E-94
BBH	20c00041	um06066	0	um06066	20c00041	0
BBH	20d00034	um06071	0	um06071	20d00034	0
BBH	20c00016	um06131	1.00E-142	um06131	20c00016	1.00E-137
BBH	20d00048	um06047	1.00E-169	um06047	20d00048	1.00E-164
BBH	20d00019	um11206	1.00E-102	um11206	20d00019	1.00E-100
BBH	20d00076	um05989	0	um05989	20d00076	0
BBH	20c00031	um06085	2.00E-87	um06085	20c00031	1.00E-89
BBH	20d00041	um06059	0	um06059	20d00041	0
BBH	20d00020	um06103	0	um06103	20d00020	0
BBH	20c00056	um06037	7.00E-94	um06037	20c00056	5.00E-88
BBH	20c00043	um06062	0	um06062	20c00043	0
BBH	20d00016	um11208	8.00E-67	um11208	20d00016	1.00E-74
BBH	20d00037	um06065	0	um06065	20d00037	0
BBH	20c00027	um15102	0	um15102	20c00027	0
BBH	20c00075	um05994	0	um05994	20c00075	0
BBH	20d00014	um12322	7.00E-48	um12322	20d00014	2.00E-49
BBH	20d00054	um06032	2.00E-30	um06032	20d00054	2.00E-29
BBH	20c00030	um06087	0	um06087	20c00030	0
BBH	20d00040	um06060	5.00E-87	um06060	20d00040	9.00E-85
BBH	20d00033	um06072	1.00E-110	um06072	20d00033	1.00E-113
BBH	20d00018	um06105	0	um06105	20d00018	0
BBH	20d00077	um05982	1.00E-144	um05982	20d00077	1.00E-133
BBH	20c00040	um06068	1.00E-75	um06068	20c00040	2.00E-79
BBH	20c00026	um06102	1.00E-179	um06102	20c00026	1.00E-176
BBH	20d00065	um05998	4.00E-52	um05998	20d00065	3.00E-53

BBH	20c00065	um06014	1.00E-29	um06014	20c00065	2.00E-22
BBH	20c00015	um06129	0	um06129	20c00015	0
BBH	20d00043	um06056	0	um06056	20d00043	0
NNN	20c00003	um10815	1.00E-84	um10815	22d00064	0
BBH	20d00032	um06075	0	um06075	20d00032	0
BBH	20d00017	um11207	1.00E-126	um11207	20d00017	1.00E-123
BBH	20d00070	um06009	0	um06009	20d00070	0
BBH	20c00072	um06010	0	um06010	20c00072	0
BBH	20d00027	um06088	0	um06088	20d00027	0
BBH	20d00058	um06020	2.00E-78	um06020	20d00058	1.00E-88
BBH	20d00051	um06038	0	um06038	20d00051	0
BBH	20d00052	um06036	1.00E-27	um06036	20d00052	1.00E-25
BBH	20c00028	um06093	1.00E-167	um06093	20c00028	1.00E-160
BBH	20d00006	um11215	0	um11215	20d00006	0
BBH	20d00047	um06049	1.00E-176	um06049	20d00047	1.00E-174
BBH	20c00057	um06035	0	um06035	20c00057	0
BBH	20d00059	um06019	0	um06019	20d00059	0
BBH	20c00062	um12033	1.00E-42	um12033	20c00062	1.00E-54
BBH	20d00010	um06125	1.00E-171	um06125	20d00010	1.00E-170
BBH	20d00039	um06063	0	um06063	20d00039	0
BBH	20c00014	um06128	4.00E-23	um06128	20c00014	1.00E-23
BBH	20d00005	um06143	2.00E-49	um06143	20d00005	2.00E-44
BBH	20c00029	um06090	6.00E-69	um06090	20c00029	1.00E-66
BBH	20d00071	um11591	0	um11591	20d00071	0
BBH	20c00034	um06078	1.00E-174	um06078	20c00034	1.00E-178
BBH	20c00073	um05997	1.00E-163	um05997	20c00073	1.00E-160
BBH	20c00045	um06058	1.00E-147	um06058	20c00045	1.00E-156
BBH	20d00008	um06133	0	um06133	20d00008	0
BBH	20d00066	um06003	5.00E-63	um06003	20d00066	1.00E-62
BBH	20c00009	um11214	0	um11214	20c00009	0
BBH	20d00042	um11600	0	um11600	20d00042	0
BBH	20c00032	um06082	1.00E-138	um06082	20c00032	1.00E-134
BBH	20d00049	um06045	0	um06045	20d00049	0
BBH	20d00026	um06089	9.00E-67	um06089	20d00026	6.00E-82
BBH	20d00031	um06079	0	um06079	20d00031	0
BBH	20d00053	um06034	0	um06034	20d00053	0
BBH	20c00039	um06069	1.00E-178	um06069	20c00039	0
BBH	20c00051	um06046	5.00E-46	um06046	20c00051	1.00E-44
BBH	20d00067	um11593	0	um11593	20d00067	0
BBH	20c00060	um12035	9.00E-63	um12035	20c00060	8.00E-63
BBH	20c00063	um06022	7.00E-86	um06022	20c00063	8.00E-79
BBH	20c00033	um06080	0	um06080	20c00033	0
BBH	20c00070	um06004	4.00E-73	um06004	20c00070	2.00E-73
BBH	20c00044	um06061	0	um06061	20c00044	0
BBH	20c00052	um11597	7.00E-45	um11597	20c00052	5.00E-39
NNN	20c00080	um01758	1.00E-171	um01758	22c00172	0
BBH	20d00045	um11598	1.00E-143	um11598	20d00045	1.00E-143
NNN	20c00061	um12034	1.00E-27			
BBH	20c00068	um06000	0	um06000	20c00068	0
BBH	20d00069	um11595.2	2.00E-39	um11595.2	20d00069	2.00E-39
BBH	20c00023	um06111	1.00E-144	um06111	20c00023	1.00E-152
BBH	20c00006	um06140.2	3.00E-94	um06140.2	20c00006	4.00E-92
BBH	20c00013	um06121	0	um06121	20c00013	0
BBH	20d00082	um05987	0	um05987	20d00082	0
BBH	20d00073	um11590	0	um11590	20d00073	0
BBH	20d00085	um10543	3.00E-87	um10543	20d00085	2.00E-94
NNN	20c00071	um12321	1.00E-117			
NNN	20c00007	um06139	0			
BBH	20d00028	um06086	0	um06086	20d00028	0
BBH	20d00060	um06018.2	1.00E-176	um06018.2	20d00060	0
BBH	20d00012	um11213	0	um11213	20d00012	0
BBH	20d00030	um11601	1.00E-122	um11601	20d00030	1.00E-122

BBH	20d00025	um06092	0	um06092	20d00025	0
BBH	20d00057	um11808	0	um11808	20d00057	0
BBH	20c00069	um06002	1.00E-134	um06002	20c00069	1.00E-139
BBH	20d00007	um06135	5.00E-96	um06135	20d00007	3.00E-86
BBH	20c00035	um06073	1.00E-139	um06073	20c00035	1.00E-131
NNN	20d00061	um11805	1.00E-165			
BBH	20c00053	um06042	0	um06042	20c00053	0
BBH	20c00047	um06053	1.00E-126	um06053	20c00047	1.00E-119
NNN	20c00022	um06111	8.00E-65	um06111	20c00023	1.00E-152
BBH	20d00011	um06130	0	um06130	20d00011	0
BBH	20c00012	um06120	0	um06120	20c00012	0
BBH	20d00050	um06043	1.00E-135	um06043	20d00050	1.00E-134
BBH	20d00036	um06067	0	um06067	20d00036	0
BBH	20d00046	um06051	7.00E-44	um06051	20d00046	4.00E-44
BBH	20d00029	um06083	0	um06083	20d00029	0
BBH	20c00008	um06138	0	um06138	20c00008	0
BBH	20d00079	um05985	6.00E-25	um05985	20d00079	1.00E-30
BBH	20d00083	um05986	1.00E-121	um05986	20d00083	1.00E-121
BBH	20c00011	um06118	0	um06118	20c00011	0
BBH	20d00009	um06119	1.00E-116	um06119	20d00009	1.00E-117
BBH	20c00001	um02811	1.00E-105	um02811	20c00001	1.00E-103
BBH	20d00024	um11602	8.00E-53	um11602	20d00024	2.00E-51
BBH	20c00066	um06012	0	um06012	20c00066	0
BBH	20c00025	um06107	1.00E-102	um06107	20c00025	1.00E-102
BBH	20d00022	um06098	0	um06098	20d00022	0
BBH	20d00055	um06029	0	um06029	20d00055	0
BBH	20d00062	um06013	0	um06013	20d00062	0
BBH	20c00004	um15027	1.00E-138	um15027	20c00004	1.00E-138
BBH	20d00074	um05992	1.00E-103	um05992	20d00074	1.00E-101
BBH	20c00076	um05988	0	um05988	20c00076	0
BBH	20c00078	um05991	0	um05991	20c00078	0
BBH	20c00046	um06055	1.00E-143	um06055	20c00046	1.00E-143
BBH	20d00080	um11589	2.00E-38	um11589	20d00080	2.00E-37
BBH	20d00035	um06070	1.00E-174	um06070	20d00035	1.00E-173
BBH	20c00059	um06031	1.00E-152	um06031	20c00059	1.00E-153
BBH	20d00023	um06097	1.00E-114	um06097	20d00023	1.00E-121
BBH	20c00010	um06134	7.00E-68	um06134	20c00010	9.00E-70
BBH	20d00021	um11204	0	um11204	20d00021	0
BBH	20c00005	um06141	4.00E-72	um06141	20c00005	2.00E-79
BBH	20d00044	um11599	1.00E-129	um11599	20d00044	1.00E-138
NNN	20c00079	um10543	2.00E-56	um10543	20d00085	2.00E-94
BBH	20c00067	um05999	6.00E-39	um05999	20c00067	4.00E-43
BBH	20c00064	um11806.2	0	um11806.2	20c00064	0
BBH	20c00049	um06048	4.00E-99	um06048	20c00049	4.00E-99
BBH	20c00054	um11596	0	um11596	20c00054	0
BBH	20c00037	um06076	0	um06076	20c00037	0
BBH	20c00018	um11211	6.00E-57	um11211	20c00018	7.00E-58
BBH	20c00077	um05984	0	um05984	20c00077	0
BBH	20c00017	um06117	0	um06117	20c00017	0
BBH	20d00068	um11594	7.00E-58	um11594	20d00068	2.00E-56
BBH	20c00058	um06033	6.00E-80	um06033	20c00058	6.00E-74
BBH	20d00056	um06027.2	0	um06027.2	20d00056	0
NNN	20d00081	um11589	4.00E-42	um11589	20d00080	2.00E-37
NNN	21d00001	um04091	2.00E-96	um04091	1c00052	0
NNN	21c00002	um11407	7.00E-63			
BBH	21c00006	um05883	1.00E-66	um05883	21c00006	1.00E-66
BBH	21d00004	um11032	3.00E-27	um11032	21d00004	5.00E-27
BBH	21c00005	um11034	4.00E-74	um11034	21c00005	5.00E-74
BBH	21c00004	um04306	3.00E-83	um04306	21c00004	3.00E-83
NNN	21d00002	um10119	8.00E-16			
BBH	21d00003	um11033	5.00E-68	um11033	21d00003	4.00E-68
BBH	22c00218	um10260	1.00E-117	um10260	22c00218	1.00E-111

BBH	22c00195	um01805	1.00E-120	um01805	22c00195	1.00E-123
BBH	22c00118	um01660	0	um01660	22c00118	1.00E-177
BBH	22d00144	um01733	1.00E-113	um01733	22d00144	1.00E-122
BBH	22d00150	um11368	6.00E-32	um11368	22d00150	6.00E-32
BBH	22d00100	um01628.2	1.00E-62	um01628.2	22d00100	6.00E-43
BBH	22d00172	um01784	5.00E-85	um01784	22d00172	3.00E-85
BBH	22d00222	um01885	1.00E-160	um01885	22d00222	1.00E-170
BBH	22c00288	um01959	0	um01959	22c00288	0
BBH	22c00289	um01961	0	um01961	22c00289	0
BBH	22d00048	um01518	2.00E-88	um01518	22d00048	2.00E-89
NNN	22d00285	um01996	0			
BBH	22d00257	um01942	1.00E-179	um01942	22d00257	1.00E-179
BBH	22d00159	um01753	1.00E-141	um01753	22d00159	1.00E-138
BBH	22d00234	um12174	0	um12174	22d00234	0
BBH	22c00060	um01554	1.00E-63	um01554	22c00060	3.00E-74
BBH	22d00116	um01653	1.00E-167	um01653	22d00116	1.00E-168
BBH	22d00109	um01668	0	um01668	22d00109	0
BBH	22d00059	um01539	4.00E-21	um01539	22d00059	3.00E-20
BBH	22c00041	um10799	5.00E-76	um10799	22c00041	8.00E-71
BBH	22d00197	um01833	0	um01833	22d00197	0
BBH	22c00290	um01962	0	um01962	22c00290	0
BBH	22d00074	um11679	0	um11679	22d00074	0
BBH	22c00176	um01765	1.00E-162	um01765	22c00176	0
BBH	22d00043	um01511	3.00E-54	um01511	22d00043	4.00E-61
BBH	22d00244	um10451	8.00E-25	um10451	22d00244	5.00E-27
BBH	22c00211	um12167	0	um12167	22c00211	0
BBH	22c00052	um01533	7.00E-83	um01533	22c00052	7.00E-83
BBH	22c00227	um01854	0	um01854	22c00227	0
BBH	22c00145	um11362	1.00E-16	um11362	22c00145	9.00E-14
NNN	22d00004	um12138.2	8.00E-24			
BBH	22d00124	um01691	0	um01691	22d00124	0
BBH	22d00168	um01776	1.00E-119	um01776	22d00168	1.00E-112
BBH	22d00279	um01988	0	um01988	22d00279	0
BBH	22d00027	um01482	0	um01482	22d00027	0
BBH	22d00191	um10254	1.00E-173	um10254	22d00191	1.00E-173
BBH	22c00240	um01877	0	um01877	22c00240	0
BBH	22c00278	um01943	0	um01943	22c00278	0
BBH	22c00235	um01867.2	0	um01867.2	22c00235	0
BBH	22d00200	um12169	0	um12169	22d00200	0
BBH	22c00193	um01799	1.00E-152	um01799	22c00193	1.00E-153
BBH	22c00252	um01892	0	um01892	22c00252	0
BBH	22c00308	um11382	1.00E-118	um11382	22c00308	1.00E-122
BBH	22c00069	um01568	2.00E-28	um01568	22c00069	2.00E-27
BBH	22c00103	um11136	1.00E-135	um11136	22c00103	1.00E-134
BBH	22c00026	um11818	1.00E-160	um11818	22c00026	1.00E-159
BBH	22c00185	um01771	7.00E-67	um01771	22c00185	4.00E-67
BBH	22d00134	um01712	1.00E-104	um01712	22d00134	1.00E-104
BBH	22c00003	um01445.2	0	um01445.2	22c00003	0
BBH	22d00250	um01932	0	um01932	22d00250	0
BBH	22d00092	um01613	1.00E-180	um01613	22d00092	1.00E-177
BBH	22c00129	um01687	0	um01687	22c00129	0
BBH	22c00031	um11671.2	5.00E-17	um11671.2	22c00031	1.00E-17
BBH	22c00301	um01985	6.00E-20	um01985	22c00301	7.00E-16
BBH	22c00079	um11692	0	um11692	22c00079	0
BBH	22c00119	um01658	0	um01658	22c00119	0
BBH	22d00080	um01577	2.00E-89	um01577	22d00080	8.00E-97
BBH	22d00295	um02017	1.00E-135	um02017	22d00295	1.00E-139
BBH	22d00110	um01667	2.00E-94	um01667	22d00110	9.00E-85
BBH	22c00297	um11379	0	um11379	22c00297	0
NNN	22d00017	um11660.2	0			
BBH	22c00070	um11681	3.00E-26	um11681	22c00070	6.00E-35
BBH	22d00167	um01777	1.00E-142	um01777	22d00167	1.00E-142

NNN	22c00038	um11881	4.00E-13	um11881	22c00037	0
NNN	22c00168	um01752	1.00E-124			
BBH	22c00263	um01919	2.00E-88	um01919	22c00263	6.00E-87
BBH	22d00068	um10817	2.00E-97	um10817	22d00068	3.00E-97
BBH	22d00089	um01606	0	um01606	22d00089	0
BBH	22d00102	um01632	4.00E-71	um01632	22d00102	6.00E-81
BBH	22d00232	um10270	0	um10270	22d00232	0
BBH	22d00229	um01902.2	0	um01902.2	22d00229	0
BBH	22c00156	um01724	2.00E-81	um01724	22c00156	5.00E-82
BBH	22c00135	um01690	5.00E-26	um01690	22c00135	2.00E-27
NNN	22c00093	um02473	3.00E-13	um02473	12d00091	3.00E-15
NNN	22c00098	um01627	0			
NNN	22c00013	um01466	0	um01466	10c00046	3.00E-74
BBH	22c00259	um01908	2.00E-94	um01908	22c00259	9.00E-97
BBH	22c00204	um01814	1.00E-121	um01814	22c00204	1.00E-150
BBH	22c00313	um02002	0	um02002	22c00313	0
BBH	22d00171	um10876	1.00E-111	um10876	22d00171	1.00E-111
BBH	22d00223	um01886	0	um01886	22d00223	0
BBH	22c00123	um01654	1.00E-122	um01654	22c00123	1.00E-116
BBH	22c00161	um01731	0	um01731	22c00161	0
BBH	22c00087	um01612	0	um01612	22c00087	0
BBH	22d00174	um01788	1.00E-133	um01788	22d00174	1.00E-130
BBH	22c00234	um10263	0	um10263	22c00234	0
BBH	22d00219	um12171	1.00E-144	um12171	22d00219	1.00E-141
BBH	22d00117	um11894	1.00E-146	um11894	22d00117	1.00E-137
BBH	22d00235	um10272	5.00E-32	um10272	22d00235	5.00E-30
BBH	22d00145	um01734	9.00E-54	um01734	22d00145	5.00E-55
BBH	22d00198	um01845	0	um01845	22d00198	0
BBH	22d00184	um01813	0	um01813	22d00184	0
BBH	22d00112	um01664	1.00E-169	um01664	22d00112	1.00E-160
BBH	22c00137	um11358	0	um11358	22c00137	0
BBH	22d00157	um10868	1.00E-159	um10868	22d00157	1.00E-178
BBH	22c00175	um01763	1.00E-154	um01763	22c00175	1.00E-151
BBH	22c00228	um01855	0	um01855	22c00228	0
BBH	22c00217	um01834	1.00E-152	um01834	22c00217	1.00E-161
BBH	22c00084	um01607	9.00E-58	um01607	22c00084	6.00E-62
BBH	22c00261	um01913	1.00E-161	um01913	22c00261	1.00E-158
BBH	22c00099	um11886	2.00E-75	um11886	22c00099	1.00E-76
BBH	22d00091	um11697	0	um11697	22d00091	0
BBH	22d00192	um01824	1.00E-128	um01824	22d00192	1.00E-128
BBH	22d00032	um11674	1.00E-179	um11674	22d00032	0
BBH	22d00211	um01859	0	um01859	22d00211	0
BBH	22c00100	um15073	1.00E-105	um15073	22c00100	1.00E-118
BBH	22c00196	um01804	0	um01804	22c00196	0
BBH	22d00123	um01671	0	um01671	22d00123	0
BBH	22d00169	um01773	5.00E-83	um01773	22d00169	2.00E-83
BBH	22d00021	um11663	1.00E-139	um11663	22d00021	1.00E-139
BBH	22d00019	um11662	1.00E-169	um11662	22d00019	1.00E-169
BBH	22d00254	um01936	0	um01936	22d00254	0
BBH	22d00201	um10258	2.00E-72	um10258	22d00201	7.00E-80
BBH	22c00194	um01800	3.00E-70	um01800	22c00194	3.00E-64
BBH	22c00062	um01556	0	um01556	22c00062	0
BBH	22c00053	um10807	0	um10807	22c00053	0
BBH	22c00298	um01978	2.00E-36	um01978	22c00298	2.00E-36
BBH	22c00180	um10878	8.00E-94	um10878	22c00180	1.00E-92
BBH	22d00042	um01508	1.00E-140	um01508	22d00042	1.00E-138
BBH	22c00061	um10816	3.00E-44	um10816	22c00061	3.00E-54
BBH	22d00075	um11680	1.00E-161	um11680	22d00075	1.00E-168
BBH	22d00060	um10811	2.00E-85	um10811	22d00060	6.00E-84
BBH	22c00077	um01599	1.00E-134	um01599	22c00077	1.00E-121
BBH	22d00010	um01456	2.00E-56	um01456	22d00010	1.00E-54
BBH	22d00056	um01534	2.00E-41	um01534	22d00056	2.00E-42

BBH	22c00110	um01646	0	um01646	22c00110	0
BBH	22c00036	um11880	4.00E-18	um11880	22c00036	4.00E-18
BBH	22d00111	um11890	1.00E-122	um11890	22d00111	1.00E-123
BBH	22c00179	um10879	2.00E-54	um10879	22c00179	2.00E-54
NNN	22d00007	um11654	7.00E-13			
BBH	22c00037	um11881	0	um11881	22c00037	0
BBH	22d00294	um02015	0	um02015	22d00294	0
BBH	22d00202	um01837	0	um01837	22d00202	0
BBH	22c00122	um12161	0	um12161	22c00122	0
BBH	22c00058	um10814	1.00E-133	um10814	22c00058	1.00E-149
NNN	22c00111	um01647	0			
BBH	22c00300	um01984	1.00E-168	um01984	22c00300	1.00E-177
BBH	22c00188	um01786	0	um01786	22c00188	0
BBH	22d00135	um01713	5.00E-33	um01713	22d00135	9.00E-31
BBH	22c00233	um10262	2.00E-38	um10262	22c00233	1.00E-31
BBH	22c00102	um01634	1.00E-53	um01634	22c00102	1.00E-53
BBH	22d00018	um11661.2	0	um11661.2	22d00018	0
BBH	22c00002	um11876	1.00E-106	um11876	22c00002	0
NNN	22c00130	um11357	0			
BBH	22c00262	um01916	2.00E-57	um01916	22c00262	2.00E-44
BBH	22d00233	um10271	2.00E-72	um10271	22d00233	1.00E-65
BBH	22d00251	um01934	1.00E-105	um01934	22d00251	1.00E-105
BBH	22d00090	um01608	1.00E-108	um01608	22d00090	1.00E-101
BBH	22c00014	um01464	0	um01464	22c00014	0
BBH	22c00203	um01812	0	um01812	22c00203	0
BBH	22c00312	um11386	1.00E-109	um11386	22c00312	1.00E-114
BBH	22d00121	um11896	1.00E-138	um11896	22d00121	1.00E-139
BBH	22c00167	um10867	2.00E-86	um10867	22c00167	2.00E-84
BBH	22d00069	um01559	0	um01559	22d00069	0
BBH	22c00258	um12175	0	um12175	22c00258	0
BBH	22c00251	um01891	0	um01891	22c00251	0
BBH	22c00025	um11817	0	um11817	22c00025	0
BBH	22c00153	um11365	0	um11365	22c00153	0
BBH	22c00030	um11668	0	um11668	22c00030	0
BBH	22d00101	um11134	4.00E-54	um11134	22d00101	4.00E-54
BBH	22d00103	um11135	4.00E-39	um11135	22d00103	6.00E-40
BBH	22c00094	um01617	1.00E-134	um01617	22c00094	1.00E-130
BBH	22d00046	um01515	1.00E-161	um01515	22d00046	1.00E-161
BBH	22c00143	um01703	0	um01703	22c00143	0
NNN	22c00220	um10259	2.00E-17			
BBH	22c00142	um01700	0	um01700	22c00142	0
BBH	22c00243	um12172	1.00E-110	um12172	22c00243	1.00E-117
BBH	22d00199	um01843	0	um01843	22d00199	0
BBH	22d00280	um11381	5.00E-71	um11381	22d00280	5.00E-71
BBH	22c00162	um01735	1.00E-133	um01735	22c00162	1.00E-133
BBH	22c00190	um01789	5.00E-40	um01789	22c00190	2.00E-46
BBH	22d00072	um01565	1.00E-124	um01565	22d00072	1.00E-122
BBH	22c00086	um01610	1.00E-147	um01610	22c00086	1.00E-147
BBH	22c00035	um01498	1.00E-149	um01498	22c00035	1.00E-153
BBH	22c00183	um01775	0	um01775	22c00183	0
BBH	22d00025	um11665	0	um11665	22d00025	0
BBH	22c00106	um01639	0	um01639	22c00106	0
BBH	22c00279	um01944	0	um01944	22c00279	0
BBH	22d00210	um01857	1.00E-157	um01857	22d00210	1.00E-162
BBH	22d00218	um01874	0	um01874	22d00218	0
BBH	22c00076	um01597	1.00E-102	um01597	22c00076	1.00E-111
BBH	22d00035	um15072	1.00E-144	um15072	22d00035	1.00E-144
BBH	22d00094	um11699	3.00E-41	um11699	22d00094	2.00E-38
BBH	22c00174	um01761	1.00E-124	um01761	22c00174	1.00E-162
BBH	22d00277	um01983	7.00E-48	um01983	22d00277	4.00E-28
NNN	22d00146	um01736	9.00E-63			
BBH	22c00225	um01851	3.00E-44	um01851	22c00225	2.00E-46

BBH	22d00020	um01470	0	um01470	22d00020	0
BBH	22d00139	um01720	0	um01720	22d00139	0
BBH	22d00162	um12163	9.00E-32	um12163	22d00162	9.00E-32
BBH	22d00252	um01935	1.00E-129	um01935	22d00252	1.00E-122
BBH	22d00236	um10273	1.00E-139	um10273	22d00236	1.00E-139
BBH	22c00126	um11892	1.00E-168	um11892	22c00126	1.00E-177
BBH	22d00287	um11384	8.00E-50	um11384	22d00287	3.00E-56
BBH	22d00293	um02012	0	um02012	22d00293	0
BBH	22d00173	um01787	0	um01787	22d00173	0
BBH	22c00281	um01948	3.00E-37	um01948	22c00281	2.00E-37
BBH	22d00118	um01673	0	um01673	22d00118	0
BBH	22c00048	um01524	4.00E-41	um01524	22c00048	3.00E-48
BBH	22d00057	um01535	5.00E-84	um01535	22d00057	1.00E-86
NNN	22d00005	um10815	3.00E-71	um10815	22d00064	0
BBH	22d00264	um10457	1.00E-55	um10457	22d00264	2.00E-62
BBH	22d00104	um01640	1.00E-150	um01640	22d00104	1.00E-149
BBH	22d00149	um11367	1.00E-145	um11367	22d00149	1.00E-146
NNN	22d00160	um01755	5.00E-99			
BBH	22c00158	um01726	1.00E-100	um01726	22c00158	1.00E-100
BBH	22d00122	um01677	2.00E-44	um01677	22d00122	8.00E-50
BBH	22d00065	um01549	1.00E-134	um01549	22d00065	1.00E-122
BBH	22d00152	um11370.2	1.00E-103	um11370.2	22d00152	1.00E-98
BBH	22c00067	um01564	0	um01564	22c00067	0
BBH	22d00120	um01686.2	0	um01686.2	22d00120	0
BBH	22c00095	um01615	2.00E-94	um01615	22c00095	1.00E-101
BBH	22c00219	um01844	1.00E-146	um01844	22c00219	1.00E-148
BBH	22c00311	um11385	1.00E-128	um11385	22c00311	1.00E-132
BBH	22d00240	um12177	0	um12177	22d00240	0
BBH	22c00315	um02008	0	um02008	22c00315	0
BBH	22c00202	um01811	1.00E-144	um01811	22c00202	1.00E-149
BBH	22c00276	um01939	3.00E-29	um01939	22c00276	4.00E-27
BBH	22d00183	um01808	1.00E-104	um01808	22d00183	1.00E-102
NNN	22c00305	um01990	0	um01990	9d00207	2.00E-19
BBH	22d00136	um01714	0	um01714	22d00136	0
BBH	22c00299	um01981	0	um01981	22c00299	0
BBH	22c00303	um01987	4.00E-38	um01987	22c00303	2.00E-42
NNN	22c00250	um10608	1.00E-133	um10608	26d00102	0
BBH	22d00071	um10822	8.00E-88	um10822	22d00071	1.00E-92
BBH	22c00133	um01680	0	um01680	22c00133	0
BBH	22c00127	um01689	4.00E-41	um01689	22c00127	1.00E-41
BBH	22c00295	um01970	5.00E-49	um01970	22c00295	2.00E-49
BBH	22c00277	um01940	1.00E-49	um01940	22c00277	2.00E-58
BBH	22d00163	um01762	0	um01762	22d00163	0
BBH	22d00190	um10253.2	1.00E-129	um10253.2	22d00190	1.00E-126
BBH	22d00256	um01941	0	um01941	22d00256	0
BBH	22d00049	um01519	0	um01519	22d00049	0
BBH	22d00034	um11677.2	0	um11677.2	22d00034	0
NNN	22c00034	um10207	1.00E-108	um10207	15d00042	0
BBH	22d00266	um01965	0	um01965	22d00266	0
BBH	22c00051	um01531	0	um01531	22c00051	0
BBH	22c00212	um01829	0	um01829	22c00212	0
BBH	22d00292	um02010	0	um02010	22d00292	0
BBH	22c00198	um01802	1.00E-175	um01802	22c00198	1.00E-174
BBH	22c00244	um01882	0	um01882	22c00244	0
BBH	22d00073	um01569	0	um01569	22d00073	0
BBH	22d00176	um01791	0	um01791	22d00176	0
BBH	22c00232	um01863	0	um01863	22c00232	0
BBH	22c00027	um11819	1.00E-142	um11819	22c00027	1.00E-148
BBH	22d00237	um12176	9.00E-57	um12176	22d00237	7.00E-54
BBH	22d00217	um01872	0	um01872	22d00217	0
BBH	22c00282	um01951	1.00E-111	um01951	22c00282	1.00E-111
BBH	22d00119	um01672	1.00E-145	um01672	22d00119	1.00E-136

BBH	22c00049	um01528	0	um01528	22c00049	0
BBH	22c00020	um15040	0	um15040	22c00020	0
BBH	22d00208	um01853	1.00E-134	um01853	22d00208	1.00E-127
BBH	22d00063	um01547	0	um01547	22d00063	0
BBH	22d00147	um01737.2	0	um01737.2	22d00147	0
BBH	22c00125	um11893	2.00E-80	um11893	22c00125	1.00E-83
BBH	22c00117	um15042	1.00E-156	um15042	22c00117	1.00E-158
BBH	22c00226	um01852	0	um01852	22c00226	0
BBH	22d00151	um11369	1.00E-166	um11369	22d00151	1.00E-171
BBH	22c00144	um01705	1.00E-160	um01705	22c00144	1.00E-165
BBH	22c00201	um10248	1.00E-179	um10248	22c00201	1.00E-170
BBH	22c00155	um01723	0	um01723	22c00155	0
BBH	22c00150	um01709	1.00E-52	um01709	22c00150	3.00E-79
NNN	22c00012	um11659	0			
BBH	22c00310	um11383	1.00E-118	um11383	22c00310	1.00E-149
BBH	22c00160	um01730	1.00E-157	um01730	22c00160	1.00E-161
BBH	22c00314	um02005	0	um02005	22c00314	0
BBH	22d00062	um10813	0	um10813	22d00062	0
BBH	22c00068	um01567	1.00E-125	um01567	22c00068	1.00E-125
BBH	22c00096	um11884.2	9.00E-48	um11884.2	22c00096	8.00E-45
BBH	22d00093	um01623	7.00E-59	um01623	22d00093	4.00E-71
BBH	22d00105	um11137.2	1.00E-112	um11137.2	22d00105	1.00E-114
BBH	22d00265	um11374	0	um11374	22d00265	0
BBH	22c00169	um10869.2	1.00E-39	um10869.2	22c00169	9.00E-44
BBH	22c00231	um01862	1.00E-171	um01862	22c00231	1.00E-174
BBH	22d00261	um01950	1.00E-157	um01950	22d00261	1.00E-157
BBH	22d00209	um01856	1.00E-115	um01856	22d00209	1.00E-125
BBH	22c00128	um01688	0	um01688	22c00128	0
BBH	22c00209	um12165	8.00E-96	um12165	22c00209	3.00E-92
BBH	22c00109	um01645	1.00E-112	um01645	22c00109	1.00E-115
BBH	22c00283	um01952	0	um01952	22c00283	0
BBH	22c00134	um01679	0	um01679	22c00134	0
BBH	22d00081	um11685	2.00E-86	um11685	22d00081	3.00E-82
BBH	22c00028	um01479	1.00E-100	um01479	22c00028	5.00E-96
BBH	22c00296	um01974	0	um01974	22c00296	0
BBH	22d00286	um06265	9.00E-22	um06265	22d00286	4.00E-22
BBH	22c00040	um01504	7.00E-34	um01504	22c00040	4.00E-30
BBH	22c00245	um12173	5.00E-17	um12173	22c00245	5.00E-11
BBH	22c00124	um01652	0	um01652	22c00124	0
BBH	22c00260	um01911	1.00E-173	um01911	22c00260	1.00E-173
BBH	22c00304	um01989	1.00E-115	um01989	22c00304	1.00E-120
BBH	22d00186	um01817	9.00E-47	um01817	22d00186	3.00E-54
BBH	22c00302	um01986	0	um01986	22c00302	0
BBH	22c00186	um10875	4.00E-99	um10875	22c00186	1.00E-103
BBH	22c00257	um01905	1.00E-110	um01905	22c00257	1.00E-110
BBH	22d00137	um01716	7.00E-55	um01716	22d00137	3.00E-54
BBH	22d00008	um11656	1.00E-143	um11656	22d00008	1.00E-144
BBH	22c00151	um11364	0	um11364	22c00151	0
BBH	22c00019	um12140	7.00E-30	um12140	22c00019	7.00E-32
BBH	22d00130	um11361.2	0	um11361.2	22d00130	0
BBH	22d00084	um11689	1.00E-143	um11689	22d00084	1.00E-137
BBH	22d00291	um02007	2.00E-88	um02007	22d00291	1.00E-89
NNN	22c00132	um01682	2.00E-82			
BBH	22c00009	um11655	1.00E-116	um11655	22c00009	1.00E-128
NNN	22d00097	um01624	4.00E-37			
BBH	22c00293	um01967	1.00E-106	um01967	22c00293	1.00E-103
BBH	22d00206	um01850	1.00E-82	um01850	22d00206	2.00E-82
BBH	22c00241	um01879	0	um01879	22c00241	0
BBH	22c00010	um11657	0	um11657	22c00010	0
BBH	22c00081	um11695	0	um11695	22c00081	0
NNN	22c00165	um01742	1.00E-129			
NNN	22c00267	um01926	1.00E-130	um01926	22c00268	1.00E-138

BBH	22d00224	um01893	1.00E-135	um01893	22d00224	1.00E-132
BBH	22d00188	um01820	7.00E-54	um01820	22d00188	7.00E-59
NNN	22d00141	um11366	1.00E-129			
BBH	22d00267	um01966	0	um01966	22d00267	0
BBH	22c00104	um01637	1.00E-112	um01637	22c00104	1.00E-112
BBH	22d00216	um01871	0	um01871	22d00216	0
BBH	22c00055	um01540	0	um01540	22c00055	0
BBH	22d00242	um10449.2	2.00E-61	um10449.2	22d00242	1.00E-67
BBH	22c00114	um01670	0	um01670	22c00114	0
BBH	22d00054	um01530	0	um01530	22d00054	0
BBH	22c00239	um10265	3.00E-53	um10265	22c00239	2.00E-50
BBH	22d00014	um01465	1.00E-172	um01465	22d00014	0
BBH	22c00256	um01903	1.00E-143	um01903	22c00256	1.00E-143
BBH	22c00088	um01614	0	um01614	22c00088	0
BBH	22d00275	um01980	0	um01980	22d00275	0
BBH	22c00318	um02016	1.00E-93	um02016	22c00318	1.00E-93
BBH	22d00078	um01574	0	um01574	22d00078	0
BBH	22d00289	um02004	1.00E-102	um02004	22d00289	1.00E-106
BBH	22d00185	um01815	1.00E-120	um01815	22d00185	1.00E-124
BBH	22c00249	um01889	0	um01889	22c00249	0
NNN	22d00129	um11360	0			
BBH	22d00178	um01794	0	um01794	22d00178	0
BBH	22c00273	um01933	2.00E-20	um01933	22c00273	2.00E-20
BBH	22d00175	um01790	0	um01790	22d00175	0
BBH	22d00044	um01513	1.00E-34	um01513	22d00044	2.00E-33
BBH	22c00056	um10810	1.00E-159	um10810	22c00056	1.00E-163
BBH	22c00046	um01521	0	um01521	22c00046	0
BBH	22d00260	um01949	1.00E-85	um01949	22d00260	5.00E-93
BBH	22d00258	um01946	0	um01946	22d00258	0
BBH	22d00050	um01526	1.00E-165	um01526	22d00050	1.00E-168
BBH	22d00001	um06350	2.00E-68	um06350	22d00001	2.00E-68
BBH	22d00024	um01478	1.00E-163	um01478	22d00024	1.00E-177
BBH	22c00173	um01759	1.00E-134	um01759	22c00173	1.00E-101
BBH	22c00149	um11363	1.00E-140	um11363	22c00149	1.00E-138
BBH	22c00075	um01595	2.00E-58	um01595	22c00075	1.00E-63
BBH	22d00164	um10872	1.00E-109	um10872	22d00164	3.00E-99
BBH	22d00155	um01748	1.00E-157	um01748	22d00155	1.00E-168
BBH	22c00320	um02019	1.00E-177	um02019	22c00320	0
BBH	22d00037	um01501	0	um01501	22d00037	0
BBH	22d00193	um01826	0	um01826	22d00193	0
BBH	22c00294	um01968	0	um01968	22c00294	0
BBH	22c00140	um01696	0	um01696	22c00140	0
BBH	22c00215	um03748	3.00E-15	um03748	22c00215	9.00E-16
BBH	22d00271	um01976	4.00E-72	um01976	22d00271	1.00E-72
BBH	22c00230	um01860	4.00E-60	um01860	22c00230	3.00E-60
NNN	22c00107	um01641	8.00E-79			
BBH	22d00138	um01718	1.00E-155	um01718	22d00138	1.00E-168
BBH	22d00290	um02006	1.00E-115	um02006	22d00290	1.00E-106
BBH	22c00285	um01954	1.00E-97	um01954	22c00285	1.00E-100
BBH	22d00083	um11688	4.00E-12	um11688	22d00083	9.00E-12
BBH	22d00288	um01999	0	um01999	22d00288	0
BBH	22d00096	um01616	3.00E-37	um01616	22d00096	5.00E-38
BBH	22c00008	um01450	0	um01450	22c00008	0
BBH	22c00115	um01669	0	um01669	22c00115	0
NNN	22c00307	um11382	2.00E-40	um11382	22c00308	1.00E-122
BBH	22d00131	um01706	2.00E-43	um01706	22d00131	3.00E-38
BBH	22c00184	um01774	0	um01774	22c00184	0
BBH	22c00029	um11666	2.00E-60	um11666	22c00029	2.00E-59
BBH	22d00106	um01643	2.00E-97	um01643	22d00106	3.00E-97
BBH	22c00275	um01937	0	um01937	22c00275	0
BBH	22c00011	um11658	2.00E-45	um11658	22c00011	2.00E-47
BBH	22c00254	um01899	0	um01899	22c00254	0

BBH	22c00080	um11693	1.00E-171	um11693	22c00080	0
BBH	22c00242	um01881	1.00E-157	um01881	22c00242	1.00E-157
BBH	22c00152	um01717	0	um01717	22c00152	0
BBH	22c00223	um01835	0	um01835	22c00223	0
BBH	22d00225	um01894	1.00E-124	um01894	22d00225	1.00E-124
BBH	22d00187	um10251	0	um10251	22d00187	0
NNN	22d00207	um03425	8.00E-14	um03425	8d00108	0
BBH	22d00003	um11875	1.00E-177	um11875	22d00003	1.00E-177
BBH	22c00274	um12342	1.00E-67	um12342	22c00274	1.00E-70
BBH	22d00153	um15043.2	0	um15043.2	22d00153	0
BBH	22d00263	um10456	1.00E-144	um10456	22d00263	1.00E-143
BBH	22c00214	um01830	0	um01830	22c00214	0
BBH	22d00128	um01701	1.00E-134	um01701	22d00128	1.00E-119
BBH	22c00248	um01888	0	um01888	22c00248	0
BBH	22d00238	um10274	2.00E-29	um10274	22d00238	2.00E-29
BBH	22c00207	um01821	0	um01821	22c00207	1.00E-173
BBH	22d00177	um01793	0	um01793	22d00177	0
BBH	22d00272	um01977	1.00E-133	um01977	22d00272	1.00E-134
NNN	22c00090	um01621	0			
BBH	22c00045	um01523	1.00E-49	um01523	22c00045	8.00E-49
BBH	22c00166	um01747	1.00E-156	um01747	22c00166	1.00E-156
BBH	22d00055	um01532	5.00E-72	um01532	22d00055	4.00E-70
BBH	22c00224	um01849	0	um01849	22c00224	0
BBH	22c00238	um01875	0	um01875	22c00238	0
BBH	22c00083	um01605	1.00E-173	um01605	22c00083	1.00E-174
BBH	22c00266	um01922	1.00E-160	um01922	22c00266	1.00E-168
BBH	22d00070	um10821	0	um10821	22d00070	0
NNN	22d00241	um01923	0			
BBH	22d00154	um15074	0	um15074	22d00154	0
BBH	22c00255	um01900	0	um01900	22c00255	0
BBH	22c00057	um01544	0	um01544	22c00057	0
BBH	22d00036	um01499	9.00E-57	um01499	22d00036	2.00E-51
BBH	22d00079	um11684	1.00E-57	um11684	22d00079	7.00E-58
BBH	22c00116	um01665	1.00E-85	um01665	22c00116	5.00E-92
BBH	22d00194	um01827	0	um01827	22d00194	0
BBH	22c00170	um10870	1.00E-138	um10870	22c00170	1.00E-146
BBH	22c00319	um02018	0	um02018	22c00319	0
BBH	22d00095	um01619	0	um01619	22d00095	0
BBH	22d00047	um10803	0	um10803	22d00047	0
BBH	22d00064	um10815	0	um10815	22d00064	0
BBH	22c00066	um10820	6.00E-42	um10820	22c00066	6.00E-42
BBH	22d00114	um01659	5.00E-51	um01659	22d00114	1.00E-65
BBH	22c00197	um01803	0	um01803	22c00197	0
BBH	22d00170	um01772	0	um01772	22d00170	0
BBH	22c00047	um12143	1.00E-76	um12143	22c00047	1.00E-77
BBH	22c00073	um01580	0	um01580	22c00073	0
BBH	22c00172	um01758	0	um01758	22c00172	0
BBH	22d00165	um10874	3.00E-62	um10874	22d00165	1.00E-60
BBH	22c00074	um11687	5.00E-86	um11687	22c00074	1.00E-89
BBH	22d00015	um04130	1.00E-110	um04130	22d00015	1.00E-116
BBH	22c00321	um02021	1.00E-135	um02021	22c00321	1.00E-137
BBH	22d00259	um01947	1.00E-160	um01947	22d00259	1.00E-161
BBH	22d00023	um01475	1.00E-37	um01475	22d00023	5.00E-38
BBH	22d00276	um01982	0	um01982	22d00276	1.00E-163
BBH	22c00163	um01738	1.00E-76	um01738	22c00163	7.00E-77
NNN	22c00138	um11359	0			
BBH	22d00255	um01938	0	um01938	22d00255	0
BBH	22d00066	um01550	1.00E-108	um01550	22d00066	1.00E-113
BBH	22d00142	um01729	0	um01729	22d00142	0
BBH	22d00226	um10268	5.00E-21	um10268	22d00226	7.00E-24
NNN	22c00033	um11675	9.00E-39			
BBH	22c00316	um02011	2.00E-40	um02011	22c00316	7.00E-41

BBH	22d00298	um02023	0	um02023	22d00298	0
BBH	22c00265	um01920	0	um01920	22c00265	0
BBH	22c00015	um01463	1.00E-126	um01463	22c00015	1.00E-126
BBH	22c00023	um11816	0	um11816	22c00023	0
BBH	22d00113	um01662	0	um01662	22d00113	0
BBH	22c00200	um15089	7.00E-93	um15089	22c00200	6.00E-96
BBH	22c00112	um01648.2	1.00E-158	um01648.2	22c00112	1.00E-158
BBH	22d00011	um01458	0	um01458	22d00011	0
BBH	22d00061	um10812	1.00E-134	um10812	22d00061	1.00E-140
BBH	22d00270	um11378	7.00E-64	um11378	22d00270	4.00E-64
BBH	22d00132	um01708	1.00E-179	um01708	22d00132	0
BBH	22c00306	um01992	0	um01992	22c00306	0
BBH	22d00099	um11885	0	um11885	22d00099	0
BBH	22d00087	um11691	0	um11691	22d00087	0
BBH	22c00291	um01963	0	um01963	22c00291	0
NNN	22c00005	um00079	4.00E-98	um00079	9c00055	1.00E-161
BBH	22d00031	um11672	1.00E-133	um11672	22d00031	1.00E-132
BBH	22c00063	um01558	1.00E-120	um01558	22c00063	1.00E-115
BBH	22c00059	um01551	1.00E-137	um01551	22c00059	1.00E-149
BBH	22c00178	um01768	0	um01768	22c00178	0
BBH	22c00072	um01578	0	um01578	22c00072	0
BBH	22d00180	um10880	1.00E-130	um10880	22d00180	1.00E-141
BBH	22c00171	um01756.2	0	um01756.2	22c00171	0
BBH	22d00006	um11653	1.00E-140	um11653	22d00006	1.00E-120
BBH	22d00262	um01953	0	um01953	22d00262	0
BBH	22d00213	um01864	0	um01864	22d00213	0
BBH	22d00029	um11669	8.00E-66	um11669	22d00029	6.00E-62
BBH	22d00203	um10255	1.00E-34	um10255	22d00203	2.00E-33
BBH	22d00195	um12166	4.00E-24	um12166	22d00195	1.00E-23
NNN	22d00022	um12141	1.00E-142			
BBH	22c00054	um10809.2	8.00E-17	um10809.2	22c00054	8.00E-17
BBH	22d00284	um01995	0	um01995	22d00284	0
BBH	22d00230	um01904	1.00E-130	um01904	22d00230	1.00E-130
BBH	22c00139	um01694	0	um01694	22c00139	0
BBH	22d00215	um01868	0	um01868	22d00215	0
BBH	22c00268	um01926	1.00E-137	um01926	22c00268	1.00E-138
BBH	22d00041	um01507	0	um01507	22d00041	0
BBH	22c00191	um01792	1.00E-168	um01792	22c00191	1.00E-172
NNN	22d00098	um01624	0			
NNN	22c00146	um03223	8.00E-15	um03223	14d00078	1.00E-29
BBH	22d00115	um01657	1.00E-160	um01657	22d00115	1.00E-169
BBH	22c00206	um01818	1.00E-140	um01818	22c00206	1.00E-149
BBH	22d00052	um01527	1.00E-148	um01527	22d00052	1.00E-161
NNN	22c00147	um03223	9.00E-18	um03223	14d00078	1.00E-29
BBH	22c00024	um01476	0	um01476	22c00024	0
BBH	22c00237	um01873	0	um01873	22c00237	0
BBH	22c00221	um10257	4.00E-97	um10257	22c00221	2.00E-96
BBH	22c00159	um01728.2	9.00E-86	um01728.2	22c00159	9.00E-88
BBH	22c00121	um01656	0	um01656	22c00121	0
BBH	22c00271	um01928	2.00E-54	um01928	22c00271	4.00E-67
BBH	22d00268	um01969	2.00E-64	um01969	22d00268	1.00E-73
BBH	22d00107	um01649	2.00E-17	um01649	22d00107	3.00E-21
NNN	22c00286	um01957	0			
BBH	22d00012	um01467	0	um01467	22d00012	0
BBH	22d00220	um10266	1.00E-170	um10266	22d00220	1.00E-162
BBH	22c00064	um01560	0	um01560	22c00064	0
BBH	22c00082	um01604	0	um01604	22c00082	0
BBH	22d00127	um01699	0	um01699	22d00127	0
BBH	22d00239	um10275	0	um10275	22d00239	0
BBH	22c00317	um02014	1.00E-116	um02014	22c00317	1.00E-117
BBH	22c00042	um10800	0	um10800	22c00042	0
BBH	22c00032	um11673	1.00E-142	um11673	22c00032	1.00E-153

BBH	22c00022	um11815.2	1.00E-114	um11815.2	22c00022	1.00E-103
BBH	22d00297	um02022	1.00E-174	um02022	22d00297	1.00E-176
BBH	22d00205	um01847	2.00E-92	um01847	22d00205	3.00E-87
BBH	22d00009	um01454	0	um01454	22d00009	0
BBH	22c00164	um01739	1.00E-152	um01739	22c00164	1.00E-164
NNN	22d00189	um10252	1.00E-137			
BBH	22c00253	um01897	0	um01897	22c00253	0
BBH	22c00229	um01858	0	um01858	22c00229	0
NNN	22d00030	um11670	1.00E-18			
BBH	22d00013	um01462	2.00E-68	um01462	22d00013	5.00E-72
BBH	22c00154	um01722	0	um01722	22c00154	0
BBH	22d00125	um01692	1.00E-100	um01692	22d00125	6.00E-98
BBH	22d00269	um01971	2.00E-44	um01971	22d00269	6.00E-44
BBH	22d00274	um01979	3.00E-60	um01979	22d00274	6.00E-59
BBH	22d00067	um01552	0	um01552	22d00067	0
BBH	22c00044	um01512	0	um01512	22c00044	0
BBH	22c00105	um01638	1.00E-120	um01638	22c00105	1.00E-136
BBH	22d00231	um01906	5.00E-50	um01906	22d00231	8.00E-50
BBH	22d00282	um01991	1.00E-174	um01991	22d00282	0
BBH	22d00086	um11690	1.00E-124	um11690	22d00086	1.00E-126
NNN	22d00085	um11689	3.00E-84	um11689	22d00084	1.00E-137
BBH	22d00039	um01505	7.00E-38	um01505	22d00039	1.00E-37
BBH	22d00108	um01651	1.00E-116	um01651	22d00108	1.00E-117
NNN	22c00092	um02473	3.00E-12	um02473	12d00091	3.00E-15
BBH	22c00006	um15093	0	um15093	22c00006	0
BBH	22c00292	um01964	0	um01964	22c00292	0
BBH	22c00018	um01469	0	um01469	22c00018	0
BBH	22c00309	um01997.2	1.00E-16	um01997.2	22c00309	1.00E-14
BBH	22c00177	um10873	1.00E-128	um10873	22c00177	1.00E-129
BBH	22d00140	um01721	0	um01721	22d00140	0
NNN	22c00004	um03403.2	2.00E-12	um03403.2	8d00113	0
BBH	22c00192	um01795	1.00E-152	um01795	22c00192	1.00E-151
BBH	22c00131	um01683.2	1.00E-160	um01683.2	22c00131	1.00E-157
BBH	22d00133	um01711	0	um01711	22d00133	0
BBH	22c00208	um01823	9.00E-77	um01823	22c00208	2.00E-79
BBH	22d00196	um01831	5.00E-97	um01831	22d00196	6.00E-95
BBH	22c00187	um01785	3.00E-90	um01785	22c00187	1.00E-100
BBH	22d00076	um11682	8.00E-78	um11682	22d00076	6.00E-81
BBH	22d00182	um11901	1.00E-177	um11901	22d00182	1.00E-171
BBH	22d00228	um01898	1.00E-150	um01898	22d00228	1.00E-153
BBH	22c00269	um01927	0	um01927	22c00269	0
BBH	22d00038	um11882	7.00E-70	um11882	22d00038	2.00E-67
BBH	22d00045	um01514	0	um01514	22d00045	0
BBH	22c00097	um01626	1.00E-109	um01626	22c00097	1.00E-104
NNN	22d00214	um01866	5.00E-66			
NNN	22d00212	um01861	1.00E-172	um01861	19d00131	1.00E-57
BBH	22d00077	um11683	0	um11683	22d00077	0
BBH	22d00053	um01529	1.00E-124	um01529	22d00053	1.00E-127
BBH	22c00199	um11899	0	um11899	22c00199	0
BBH	22d00249	um10452	0	um10452	22d00249	0
NNN	22c00148	um03223	4.00E-18	um03223	14d00078	1.00E-29
BBH	22c00071	um01576	1.00E-115	um01576	22c00071	1.00E-126
BBH	22d00028	um11667	0	um11667	22d00028	0
BBH	22d00156	um01750	1.00E-163	um01750	22d00156	1.00E-158
BBH	22c00280	um01945	0	um01945	22c00280	0
BBH	22d00126	um01697	0	um01697	22d00126	0
BBH	22c00113	um01650	1.00E-123	um01650	22c00113	1.00E-125
BBH	22c00039	um01503	0	um01503	22c00039	0
BBH	22d00166	um01783	0	um01783	22d00166	0
BBH	22d00221	um01883	1.00E-122	um01883	22d00221	1.00E-122
BBH	22c00085	um11696	3.00E-70	um11696	22c00085	7.00E-70
BBH	22c00065	um01561	0	um01561	22c00065	0

BBH	22c00272	um01931	0	um01931	22c00272	0
BBH	22c00205	um01816	1.00E-141	um01816	22c00205	1.00E-136
BBH	22d00179	um01797	0	um01797	22d00179	0
BBH	22c00043	um01510	0	um01510	22c00043	0
BBH	22c00216	um01832	1.00E-167	um01832	22c00216	1.00E-173
BBH	22c00236	um01869	0	um01869	22c00236	0
BBH	22c00222	um10256	1.00E-174	um10256	22c00222	1.00E-168
BBH	22d00243	um10450	0	um10450	22d00243	0
BBH	22d00143	um01732	0	um01732	22d00143	0
BBH	22d00283	um01994	0	um01994	22d00283	0
BBH	22d00296	um02020	7.00E-68	um02020	22d00296	4.00E-63
BBH	24c00064	um06253	0	um06253	24c00064	0
BBH	24d00045	um06234	1.00E-112	um06234	24d00045	1.00E-114
BBH	24c00026	um06189	0	um06189	24c00026	0
BBH	24d00026	um11222	0	um11222	24d00026	0
BBH	24d00004	um11338	0	um11338	24d00004	0
BBH	24d00014	um06169	4.00E-64	um06169	24d00014	1.00E-67
NNN	24d00039	um11233	1.00E-22	um11233	24d00040	1.00E-101
BBH	24d00055	um06257	0	um06257	24d00055	0
BBH	24c00055	um06237	0	um06237	24c00055	0
BBH	24d00044	um06229	0	um06229	24d00044	0
BBH	24c00043	um06219	1.00E-104	um06219	24c00043	1.00E-100
BBH	24c00071	um06261.2	0	um06261.2	24c00071	0
BBH	24c00065	um06256	0	um06256	24c00065	0
BBH	24d00030	um06200	1.00E-177	um06200	24d00030	0
BBH	24d00022	um06190	1.00E-140	um06190	24d00022	1.00E-129
BBH	24c00033	um06201	0	um06201	24c00033	0
BBH	24d00006	um06156	0	um06156	24d00006	0
BBH	24d00003	um11339	0	um11339	24d00003	0
BBH	24d00015	um11218	3.00E-36	um11218	24d00015	2.00E-30
BBH	24c00003	um06155	0	um06155	24c00003	0
BBH	24d00054	um06255	1.00E-25	um06255	24d00054	7.00E-31
BBH	24d00041	um11234.2	3.00E-22	um11234.2	24d00041	2.00E-14
BBH	24c00053	um06235	0	um06235	24c00053	0
NNN	24d00009	um06148	1.00E-129			
BBH	24c00016	um06167	1.00E-165	um06167	24c00016	1.00E-172
BBH	24c00042	um06215	0	um06215	24c00042	0
NNN	24c00032	um06201	7.00E-21	um06201	24c00033	0
BBH	24d00047	um10971	3.00E-50	um10971	24d00047	1.00E-49
BBH	24d00028	um06197	3.00E-19	um06197	24d00028	5.00E-24
BBH	24c00009	um06162	1.00E-143	um06162	24c00009	1.00E-138
BBH	24c00011	um11300	6.00E-77	um11300	24c00011	2.00E-76
BBH	24d00012	um06153	0	um06153	24d00012	0
BBH	24d00019	um06182	6.00E-85	um06182	24d00019	1.00E-89
BBH	24d00002	um01431	0	um01431	24d00002	0
BBH	24c00006	um11296.2	0	um11296.2	24c00006	0
BBH	24d00010	um11298	6.00E-61	um11298	24d00010	8.00E-69
BBH	24d00031	um06210	0	um06210	24d00031	0
BBH	24d00020	um06183	1.00E-126	um06183	24d00020	1.00E-128
BBH	24d00011	um06151	9.00E-75	um06151	24d00011	7.00E-74
BBH	24c00054	um06236	1.00E-119	um06236	24c00054	1.00E-119
BBH	24c00027	um11221	0	um11221	24c00027	0
BBH	24d00046	um10970	1.00E-132	um10970	24d00046	1.00E-136
BBH	24d00027	um11223	3.00E-54	um11223	24d00027	3.00E-54
BBH	24c00001	um01434	0	um01434	24c00001	0
BBH	24d00056	um06258	0	um06258	24d00056	0
BBH	24d00032	um11227	2.00E-43	um11227	24d00032	1.00E-43
BBH	24c00031	um11226	1.00E-155	um11226	24c00031	1.00E-157
NNN	24c00041	um06215	7.00E-57	um06215	24c00042	0
BBH	24d00021	um04833	2.00E-58	um04833	24d00021	7.00E-55
BBH	24c00017	um06168	1.00E-168	um06168	24c00017	1.00E-159
BBH	24d00001	um01433	1.00E-111	um01433	24d00001	1.00E-107

BBH	24d00013	um06164	0	um06164	24d00013	0
BBH	24c00073	um05648	1.00E-149	um05648	24c00073	1.00E-118
BBH	24c00004	um06158	0	um06158	24c00004	0
BBH	24d00017	um06174	0	um06174	24d00017	0
BBH	24c00040	um06213	4.00E-76	um06213	24c00040	1.00E-75
BBH	24d00033	um11228	6.00E-70	um11228	24d00033	3.00E-65
BBH	24d00025	um06194	0	um06194	24d00025	0
BBH	24d00049	um10972	2.00E-46	um10972	24d00049	1.00E-42
NNN	24d00061	um01944	0	um01944	22c00279	0
NNN	24d00008	um12096	1.00E-19	um12096	6d00070	1.00E-122
BBH	24d00058	um06259	1.00E-118	um06259	24d00058	1.00E-123
NNN	24c00058	um03263	5.00E-51	um03263	14d00067	0
BBH	24c00012	um06152	1.00E-179	um06152	24c00012	0
NNN	24c00059	um12330	1.00E-101			
BBH	24c00030	um11224	2.00E-61	um11224	24c00030	3.00E-59
BBH	24c00039	um06212	1.00E-103	um06212	24c00039	1.00E-111
BBH	24c00069	um04305	0	um04305	24c00069	0
BBH	24d00042	um06226	1.00E-154	um06226	24d00042	1.00E-152
BBH	24c00023	um06186	9.00E-87	um06186	24c00023	8.00E-79
BBH	24c00025	um06188	0	um06188	24c00025	0
BBH	24c00049	um15028	0	um15028	24c00049	0
BBH	24c00018	um11217	8.00E-33	um11217	24c00018	8.00E-33
BBH	24d00036	um11231	0	um11231	24d00036	0
NNN	24d00007	um06326	9.00E-48	um06326	4d00032	1.00E-124
BBH	24c00052	um06231	1.00E-161	um06231	24c00052	1.00E-156
BBH	24c00013	um06154	7.00E-49	um06154	24c00013	1.00E-52
BBH	24c00070	um10979	0	um10979	24c00070	0
BBH	24c00008	um11299	1.00E-110	um11299	24c00008	1.00E-115
BBH	24c00038	um06211	0	um06211	24c00038	0
BBH	24d00048	um12329	0	um12329	24d00048	0
BBH	24c00048	um11235	5.00E-69	um11235	24c00048	2.00E-62
BBH	24d00018	um12326	1.00E-96	um12326	24d00018	2.00E-99
NNN	24c00047	um06224	0			
BBH	24c00024	um06187	0	um06187	24c00024	0
BBH	24d00029	um11225	0	um11225	24d00029	0
BBH	24c00022	um06184	0	um06184	24c00022	0
BBH	24c00007	um11297	0	um11297	24c00007	0
BBH	24d00053	um06251	0	um06251	24d00053	0
BBH	24c00056	um06239	0	um06239	24c00056	0
BBH	24d00063	um05647	0	um05647	24d00063	0
BBH	24c00014	um06163	5.00E-54	um06163	24c00014	1.00E-42
BBH	24c00072	um05646	0	um05646	24c00072	0
BBH	24d00035	um11230	0	um11230	24d00035	0
BBH	24c00028	um06195	1.00E-131	um06195	24c00028	1.00E-137
BBH	24c00029	um12327	0	um12327	24c00029	0
BBH	24c00050	um06228	0	um06228	24c00050	0
BBH	24d00051	um06249	0	um06249	24d00051	0
BBH	24d00023	um06191	3.00E-43	um06191	24d00023	2.00E-35
NNN	24c00066	um06260	0			
NNN	24c00060	um10974.2	0			
BBH	24c00021	um06175	0	um06175	24c00021	0
BBH	24d00062	um05645	9.00E-50	um05645	24d00062	5.00E-50
BBH	24d00037	um11232	0	um11232	24d00037	0
BBH	24d00040	um11233	1.00E-101	um11233	24d00040	1.00E-101
BBH	24c00037	um15068	2.00E-87	um15068	24c00037	3.00E-86
BBH	24c00015	um12325	2.00E-22	um12325	24c00015	2.00E-23
BBH	24d00052	um10976	0	um10976	24d00052	0
BBH	24d00059	um03627	1.00E-133	um03627	24d00059	1.00E-133
BBH	24c00051	um10968	0	um10968	24c00051	0
BBH	24c00035	um06205	0	um06205	24c00035	0
BBH	24d00050	um10973	0	um10973	24d00050	0
BBH	24c00002	um01432	0	um01432	24c00002	0

BBH	24c00005	um06159	0	um06159	24c00005	0
BBH	24c00020	um11220	0	um11220	24c00020	0
BBH	24c00061	um10975.2	1.00E-167	um10975.2	24c00061	1.00E-167
BBH	24c00068	um02028	0	um02028	24c00068	0
BBH	24c00067	um03626	8.00E-77	um03626	24c00067	8.00E-78
BBH	24d00024	um06193	2.00E-74	um06193	24d00024	6.00E-77
BBH	24d00038	um06218	1.00E-96	um06218	24d00038	1.00E-98
BBH	24c00036	um06206	6.00E-88	um06206	24c00036	1.00E-100
BBH	24d00016	um11219	1.00E-128	um11219	24d00016	1.00E-118
BBH	25d00005	um05629	1.00E-159	um05629	25d00005	1.00E-177
BBH	25c00056	um05729	3.00E-87	um05729	25c00056	8.00E-80
BBH	25d00085	um05776	0	um05776	25d00085	0
BBH	25d00011	um05625	1.00E-46	um05625	25d00011	2.00E-46
NNN	25c00082	um05785	1.00E-175			
BBH	25c00067	um05753	0	um05753	25c00067	0
BBH	25d00018	um05654	2.00E-83	um05654	25d00018	4.00E-95
BBH	25c00043	um10735	0	um10735	25c00043	0
NNN	25c00003	um00113	1.00E-161	um00113	9d00061	0
BBH	25d00032	um10742	7.00E-33	um10742	25d00032	6.00E-33
BBH	25c00040	um05677	1.00E-127	um05677	25c00040	1.00E-134
BBH	25c00079	um05789	1.00E-67	um05789	25c00079	3.00E-62
BBH	25d00064	um05737	1.00E-136	um05737	25d00064	1.00E-138
BBH	25d00054	um05720	1.00E-141	um05720	25d00054	1.00E-140
BBH	25c00024	um10938	1.00E-112	um10938	25c00024	1.00E-117
BBH	25d00053	um05656	0	um05656	25d00053	0
NNN	25d00006	um12019	0			
BBH	25d00012	um05640	1.00E-100	um05640	25d00012	3.00E-96
BBH	25c00042	um05674	1.00E-133	um05674	25c00042	1.00E-134
BBH	25c00012	um10718.2	0	um10718.2	25c00012	0
BBH	25c00076	um05769	1.00E-160	um05769	25c00076	1.00E-161
BBH	25d00031	um15084	3.00E-69	um15084	25d00031	3.00E-70
BBH	25d00043	um05676	0	um05676	25d00043	1.00E-179
NNN	25c00002	um01881	2.00E-77	um01881	22c00242	1.00E-157
BBH	25c00083	um11584	1.00E-103	um11584	25c00083	1.00E-107
BBH	25d00065	um05739	0	um05739	25d00065	0
BBH	25d00075	um05761	2.00E-61	um05761	25d00075	1.00E-59
BBH	25d00015	um05632	1.00E-92	um05632	25d00015	2.00E-89
BBH	25c00039	um05678	1.00E-90	um05678	25c00039	1.00E-90
BBH	25d00029	um05719.2	1.00E-100	um05719.2	25d00029	1.00E-121
NNN	25d00022	um05709	1.00E-161			
BBH	25c00051	um05659	0	um05659	25c00051	0
BBH	25c00045	um10729	2.00E-82	um10729	25c00045	5.00E-82
BBH	25d00076	um05766	1.00E-147	um05766	25d00076	1.00E-147
BBH	25d00003	um05626	1.00E-74	um05626	25d00003	1.00E-74
BBH	25c00005	um12018	7.00E-27	um12018	25c00005	3.00E-22
BBH	25c00010	um05641	0	um05641	25c00010	1.00E-174
BBH	25c00064	um05745	0	um05745	25c00064	0
BBH	25d00046	um10731	2.00E-62	um10731	25d00046	1.00E-68
NNN	25c00053	um05724	4.00E-11			
BBH	25c00023	um05714	0	um05714	25c00023	0
BBH	25c00021	um15022.2	6.00E-68	um15022.2	25c00021	1.00E-67
BBH	25d00037	um05690	0	um05690	25d00037	0
BBH	25d00061	um05728	2.00E-65	um05728	25d00061	4.00E-74
BBH	25c00073	um12024	9.00E-25	um12024	25c00073	8.00E-25
BBH	25c00038	um05680	0	um05680	25c00038	0
BBH	25d00060	um05726	0	um05726	25d00060	0
BBH	25c00028	um05700	1.00E-158	um05700	25c00028	1.00E-160
BBH	25d00059	um15023	0	um15023	25d00059	0
BBH	25d00058	um05725	1.00E-104	um05725	25d00058	1.00E-104
BBH	25c00058	um05734	1.00E-110	um05734	25c00058	1.00E-110
BBH	25c00031	um05693	2.00E-86	um05693	25c00031	1.00E-101
BBH	25c00037	um12312	1.00E-124	um12312	25c00037	1.00E-126

BBH	25c00044	um10732	1.00E-149	um10732	25c00044	1.00E-149
BBH	25c00011	um10719	1.00E-132	um10719	25c00011	1.00E-135
BBH	25c00004	um05628.2	8.00E-57	um05628.2	25c00004	3.00E-57
BBH	25c00018	um05653.2	4.00E-96	um05653.2	25c00018	4.00E-79
NNN	25d00004	um05627	1.00E-137			
NNN	25d00017	um05652	1.00E-136			
BBH	25c00022	um10935	0	um10935	25c00022	0
BBH	25c00020	um05704	1.00E-127	um05704	25c00020	1.00E-134
BBH	25d00077	um05767	0	um05767	25d00077	0
BBH	25c00065	um10918	4.00E-22	um10918	25c00065	4.00E-22
BBH	25c00052	um05721	0	um05721	25c00052	1.00E-119
BBH	25c00050	um05660	0	um05660	25c00050	0
BBH	25d00086	um05777	0	um05777	25d00086	0
BBH	25d00070	um05747	0	um05747	25d00070	0
BBH	25d00051	um05658	1.00E-110	um05658	25d00051	1.00E-124
BBH	25d00027	um10939	2.00E-79	um10939	25d00027	3.00E-76
BBH	25d00057	um10941	1.00E-143	um10941	25d00057	1.00E-146
BBH	25d00020	um05706	0	um05706	25d00020	0
BBH	25d00067	um05742	4.00E-77	um05742	25d00067	3.00E-77
BBH	25c00030	um05695	0	um05695	25c00030	0
BBH	25d00045	um05671	0	um05671	25d00045	0
BBH	25c00078	um05790	0	um05790	25c00078	0
BBH	25d00069	um05746	1.00E-158	um05746	25d00069	1.00E-163
BBH	25c00061	um05740	0	um05740	25c00061	0
BBH	25d00035	um10740	3.00E-44	um10740	25d00035	2.00E-44
BBH	25d00030	um05702	1.00E-141	um05702	25d00030	1.00E-149
BBH	25d00050	um10726	1.00E-55	um10726	25d00050	3.00E-56
BBH	25c00036	um05683	7.00E-92	um05683	25c00036	8.00E-92
BBH	25d00078	um05770	1.00E-179	um05770	25d00078	0
BBH	25d00040	um10738	1.00E-165	um10738	25d00040	1.00E-178
BBH	25d00009	um10721	1.00E-180	um10721	25d00009	1.00E-170
BBH	25d00025	um10937	0	um10937	25d00025	1.00E-176
BBH	25d00019	um05705	2.00E-51	um05705	25d00019	5.00E-47
NNN	25c00015	um02704	2.00E-44	um02704	5d00046	0
BBH	25d00036	um05692	0	um05692	25d00036	0
NNN	25d00028	um05718	0			
BBH	25c00026	um10940	1.00E-86	um10940	25c00026	7.00E-84
BBH	25c00047	um05665	1.00E-162	um05665	25c00047	1.00E-162
NNN	25c00087	um03407	2.00E-30	um03407	8d00115	0
BBH	25d00047	um10730	0	um10730	25d00047	0
BBH	25d00068	um10917	7.00E-21	um10917	25d00068	2.00E-21
BBH	25c00062	um10915	0	um10915	25c00062	0
BBH	25c00007	um05649	0	um05649	25c00007	0
BBH	25d00038	um05689	1.00E-146	um05689	25d00038	1.00E-153
NNN	25d00088	um02062	2.00E-64	um02062	6c00122	0
NNN	25c00060	um05644	0	um05644	15d00078	3.00E-22
BBH	25d00081	um11586	6.00E-24	um11586	25d00081	2.00E-30
BBH	25d00056	um05722	0	um05722	25d00056	0
BBH	25c00035	um05684	2.00E-59	um05684	25c00035	1.00E-72
BBH	25d00071	um05748	5.00E-84	um05748	25d00071	5.00E-89
NNN	25d00095	um01943	4.00E-18	um01943	22c00278	0
BBH	25d00013	um10720	0	um10720	25d00013	0
BBH	25c00084	um11583	2.00E-59	um11583	25c00084	3.00E-52
BBH	25c00063	um10916	1.00E-164	um10916	25c00063	1.00E-173
BBH	25c00055	um10914	1.00E-106	um10914	25c00055	1.00E-107
BBH	25d00062	um05731	2.00E-52	um05731	25d00062	4.00E-51
BBH	25c00029	um05694	8.00E-15	um05694	25c00029	8.00E-15
BBH	25d00048	um10727	0	um10727	25d00048	0
BBH	25c00034	um05686	4.00E-36	um05686	25c00034	9.00E-37
NNN	25d00072	um05755.2	0			
NNN	25d00080	um10070	5.00E-37	um10070	6c00051	0
BBH	25c00006	um05631.2	0	um05631.2	25c00006	0

NNN	25d00091	um06076	7.00E-28	um06076	20c00037	0
BBH	25c00017	um10724	1.00E-57	um10724	25c00017	3.00E-58
BBH	25c00072	um05760	4.00E-57	um05760	25c00072	1.00E-58
BBH	25d00063	um05736	0	um05736	25d00063	0
BBH	25d00016	um05651	0	um05651	25d00016	0
BBH	25d00042	um05679	1.00E-108	um05679	25d00042	2.00E-98
BBH	25d00083	um05781.2	4.00E-35	um05781.2	25d00083	2.00E-35
BBH	25d00039	um05687	1.00E-123	um05687	25d00039	1.00E-137
BBH	25d00026	um05715	0	um05715	25d00026	0
NNN	25c00013	um05636	1.00E-105			
BBH	25c00074	um05764	1.00E-104	um05764	25c00074	1.00E-103
BBH	25d00034	um10741	3.00E-53	um10741	25d00034	6.00E-59
BBH	25c00054	um10913	0	um10913	25c00054	0
BBH	25c00033	um05688	1.00E-100	um05688	25c00033	1.00E-111
BBH	25d00073	um05758	0	um05758	25d00073	0
BBH	25c00085	um05774	3.00E-95	um05774	25c00085	5.00E-85
BBH	25c00049	um05663	1.00E-58	um05663	25c00049	1.00E-58
BBH	25c00009	um12017	1.00E-131	um12017	25c00009	1.00E-128
BBH	25c00080	um05787	1.00E-133	um05787	25c00080	1.00E-138
BBH	25c00077	um05771	0	um05771	25c00077	0
NNN	25c00068	um05754	1.00E-149			
BBH	25d00007	um10723	4.00E-58	um10723	25d00007	2.00E-64
BBH	25c00070	um12021	7.00E-99	um12021	25c00070	8.00E-99
BBH	25d00023	um10934	0	um10934	25d00023	0
BBH	25c00066	um05749	0	um05749	25c00066	0
BBH	25d00049	um05662	1.00E-157	um05662	25d00049	1.00E-157
BBH	25c00075	um11581	5.00E-48	um11581	25c00075	1.00E-47
BBH	25d00041	um10737	0	um10737	25d00041	0
BBH	25c00057	um05732	0	um05732	25c00057	0
NNN	25d00093	um05972	1.00E-137	um05972	6c00005	0
BBH	25c00008	um05624	1.00E-129	um05624	25c00008	1.00E-128
BBH	25c00071	um12023	1.00E-138	um12023	25c00071	1.00E-141
BBH	25d00044	um10733	1.00E-72	um10733	25d00044	2.00E-74
BBH	25c00081	um05786.2	1.00E-114	um05786.2	25c00081	1.00E-113
NNN	25d00089	um04886	5.00E-22	um04886	26d00001	0
BBH	25d00024	um10936	2.00E-13	um10936	25d00024	1.00E-15
NNN	25c00032	um05691	9.00E-13			
BBH	25c00041	um05675	1.00E-111	um05675	25c00041	1.00E-110
BBH	25d00033	um05698	0	um05698	25d00033	0
BBH	25c00069	um05756.2	0	um05756.2	25c00069	0
NNN	25c00048	um01980	7.00E-36	um01980	22d00275	0
BBH	25c00014	um05635	4.00E-91	um05635	25c00014	5.00E-90
BBH	25d00008	um10722	0	um10722	25d00008	0
BBH	25d00074	um12022	1.00E-23	um12022	25d00074	4.00E-29
BBH	25c00027	um05703	1.00E-150	um05703	25c00027	1.00E-155
NNN	25d00001	um02021	7.00E-65	um02021	22c00321	1.00E-137
BBH	25d00082	um11585	0	um11585	25d00082	0
BBH	25d00010	um05642	0	um05642	25d00010	0
NNN	26d00046	um04802	5.00E-68			
BBH	26d00102	um10608	0	um10608	26d00102	0
BBH	26c00026	um10703	0	um10703	26c00026	0
BBH	26d00079	um04724	0	um04724	26d00079	0
BBH	26d00021	um04850	1.00E-132	um04850	26d00021	1.00E-134
BBH	26d00078	um04726	1.00E-149	um04726	26d00078	1.00E-157
BBH	26d00097	um10582.2	1.00E-157	um10582.2	26d00097	1.00E-158
NNN	26d00028	um12282	1.00E-29	um12282	26d00029	2.00E-89
BBH	26c00056	um10688	0	um10688	26c00056	0
BBH	26c00044	um04806	0	um04806	26c00044	0
BBH	26d00016	um04855	4.00E-34	um04855	26d00016	1.00E-34
BBH	26c00070	um04753	0	um04753	26c00070	0
BBH	26d00087	um04711	0	um04711	26d00087	0
BBH	26c00015	um12285	4.00E-98	um12285	26c00015	1.00E-105

BBH	26d00086	um04713	1.00E-138	um04713	26d00086	1.00E-138
BBH	26d00058	um04773	0	um04773	26d00058	0
BBH	26d00066	um04758	0	um04758	26d00066	0
BBH	26c00067	um11998	0	um11998	26c00067	0
BBH	26c00001	um04885	0	um04885	26c00001	0
NNN	26c00049	um04800	1.00E-86			
BBH	26d00045	um04807	1.00E-97	um04807	26d00045	1.00E-109
BBH	26d00003	um12006	1.00E-108	um12006	26d00003	1.00E-108
BBH	26d00022	um04848	0	um04848	26d00022	0
BBH	26d00077	um04733	2.00E-72	um04733	26d00077	5.00E-80
BBH	26d00051	um04789	1.00E-129	um04789	26d00051	1.00E-127
BBH	26c00079	um04734	0	um04734	26c00079	0
BBH	26d00020	um04851	7.00E-36	um04851	26d00020	7.00E-31
BBH	26d00001	um04886	0	um04886	26d00001	0
BBH	26c00043	um04808	0	um04808	26c00043	0
BBH	26c00025	um04844	1.00E-88	um04844	26c00025	2.00E-91
BBH	26c00024	um04847	2.00E-50	um04847	26c00024	7.00E-57
BBH	26d00015	um04856	1.00E-106	um04856	26d00015	1.00E-104
BBH	26c00016	um04860	1.00E-146	um04860	26c00016	1.00E-146
BBH	26d00080	um04722	2.00E-66	um04722	26d00080	2.00E-66
BBH	26c00089	um04712	0	um04712	26c00089	0
BBH	26c00055	um04791	0	um04791	26c00055	0
BBH	26d00060	um04778	2.00E-94	um04778	26d00060	7.00E-96
BBH	26d00095	um04702	8.00E-88	um04702	26d00095	9.00E-88
BBH	26c00068	um11997	0	um11997	26c00068	0
BBH	26d00065	um11999	0	um11999	26d00065	0
BBH	26d00044	um04809	1.00E-101	um04809	26d00044	1.00E-104
BBH	26d00096	um04701	1.00E-119	um04701	26d00096	1.00E-116
BBH	26d00038	um10698	6.00E-15	um10698	26d00038	3.00E-16
BBH	26d00004	um04881	0	um04881	26d00004	0
BBH	26d00094	um04703	1.00E-132	um04703	26d00094	1.00E-132
BBH	26d00032	um04830	1.00E-117	um04830	26d00032	1.00E-123
BBH	26c00072	um04749	0	um04749	26c00072	0
BBH	26c00023	um04849	7.00E-21	um04849	26c00023	3.00E-22
BBH	26c00017	um04859	0	um04859	26c00017	0
BBH	26c00058	um10687	1.00E-104	um10687	26c00058	1.00E-104
BBH	26d00069	um10683	0	um10683	26d00069	0
BBH	26c00050	um04797	1.00E-154	um04797	26c00050	1.00E-159
BBH	26d00072	um10682	0	um10682	26d00072	0
BBH	26c00078	um04732	0	um04732	26c00078	0
BBH	26d00048	um04798	9.00E-62	um04798	26d00048	1.00E-63
BBH	26c00094	um12015	8.00E-21	um12015	26c00094	8.00E-21
BBH	26c00008	um10858	8.00E-66	um10858	26c00008	8.00E-66
BBH	26c00069	um04755	0	um04755	26c00069	0
BBH	26c00096	um02808	0	um02808	26c00096	0
BBH	26d00088	um10673	1.00E-145	um10673	26d00088	1.00E-145
BBH	26d00006	um04878	0	um04878	26d00006	0
BBH	26d00084	um04716	0	um04716	26d00084	1.00E-168
BBH	26d00017	um10852	0	um10852	26d00017	0
BBH	26c00075	um04740	1.00E-108	um04740	26c00075	1.00E-114
BBH	26d00068	um10684	1.00E-128	um10684	26d00068	1.00E-131
BBH	26c00018	um10854	0	um10854	26c00018	0
BBH	26c00010	um04872	0	um04872	26c00010	0
BBH	26d00067	um15017	0	um15017	26d00067	0
BBH	26d00047	um04799	8.00E-11	um04799	26d00047	2.00E-15
NNN	26d00031	um04833	0	um04833	24d00021	7.00E-55
BBH	26d00101	um02807	0	um02807	26d00101	1.00E-176
NNN	26d00071	um06336	2.00E-42	um06336	4c00036	1.00E-154
BBH	26c00031	um10701	2.00E-24	um10701	26c00031	4.00E-25
BBH	26c00077	um10680	0	um10680	26c00077	0
NNN	26c00046	um10691.2	0	um10691.2	7c00326	4.00E-20
BBH	26d00027	um10702	8.00E-97	um10702	26d00027	8.00E-97

BBH	26c00039	um04813.2	0	um04813.2	26c00039	0
BBH	26d00029	um12282	7.00E-96	um12282	26d00029	2.00E-89
BBH	26d00085	um04714	3.00E-65	um04714	26d00085	1.00E-66
BBH	26c00091	um04707	1.00E-139	um04707	26c00091	1.00E-144
BBH	26c00061	um04775	1.00E-66	um04775	26c00061	4.00E-69
NNN	26c00040	um04812	0			
BBH	26c00057	um04786	0	um04786	26c00057	0
BBH	26c00082	um10678	0	um10678	26c00082	0
BBH	26d00089	um10672.2	3.00E-31	um10672.2	26d00089	3.00E-44
BBH	26d00054	um10686	1.00E-123	um10686	26d00054	1.00E-135
BBH	26c00063	um04770	2.00E-15	um04770	26c00063	8.00E-18
BBH	26d00008	um04873	0	um04873	26d00008	0
BBH	26d00092	um04705	0	um04705	26d00092	0
BBH	26d00062	um12002	1.00E-126	um12002	26d00062	1.00E-126
BBH	26c00098	um02762	1.00E-110	um02762	26c00098	1.00E-115
BBH	26d00055	um04781	2.00E-44	um04781	26d00055	2.00E-44
BBH	26c00045	um04805	5.00E-72	um04805	26c00045	2.00E-65
BBH	26c00022	um10849	5.00E-79	um10849	26c00022	8.00E-82
BBH	26c00052	um04795	0	um04795	26c00052	0
BBH	26d00025	um10704	1.00E-110	um10704	26d00025	1.00E-109
BBH	26c00036	um04819	0	um04819	26c00036	0
BBH	26d00074	um04739	1.00E-114	um04739	26d00074	1.00E-111
BBH	26c00048	um04801.2	1.00E-104	um04801.2	26c00048	1.00E-104
BBH	26d00019	um04852	9.00E-93	um04852	26d00019	1.00E-173
BBH	26d00093	um04704	0	um04704	26d00093	0
BBH	26d00041	um04818	1.00E-135	um04818	26d00041	1.00E-144
BBH	26d00075	um04737	1.00E-88	um04737	26d00075	4.00E-78
BBH	26c00074	um04744	1.00E-118	um04744	26c00074	1.00E-126
BBH	26c00097	um10609	1.00E-100	um10609	26c00097	3.00E-99
BBH	26d00070	um04752	0	um04752	26d00070	0
BBH	26c00081	um04727	0	um04727	26c00081	0
BBH	26d00010	um12286	0	um12286	26d00010	0
BBH	26c00011	um04871	0	um04871	26c00011	0
BBH	26d00012	um04863	3.00E-77	um04863	26d00012	2.00E-71
BBH	26d00061	um04771	0	um04771	26d00061	0
BBH	26c00012	um04869	0	um04869	26c00012	0
BBH	26c00086	um10675	3.00E-81	um10675	26c00086	3.00E-79
BBH	26c00071	um04751	1.00E-104	um04751	26c00071	1.00E-111
BBH	26c00006	um12007	0	um12007	26c00006	0
BBH	26c00064	um04768	0	um04768	26c00064	0
BBH	26c00093	um04700	6.00E-92	um04700	26c00093	4.00E-86
BBH	26c00059	um04782	0	um04782	26c00059	0
NNN	26c00021	um10496	1.00E-45	um10496	10c00067	1.00E-159
BBH	26c00095	um04696	5.00E-95	um04696	26c00095	2.00E-99
BBH	26d00073	um04743	0	um04743	26d00073	0
BBH	26d00026	um01136	0	um01136	26d00026	0
BBH	26c00051	um04796	0	um04796	26c00051	0
BBH	26c00076	um04738	0	um04738	26c00076	0
BBH	26c00033	um04826	0	um04826	26c00033	0
BBH	26c00084	um04721	5.00E-95	um04721	26c00084	1.00E-114
BBH	26c00047	um10690	4.00E-18	um10690	26c00047	1.00E-22
BBH	26d00005	um04880	0	um04880	26d00005	0
BBH	26c00007	um12005	1.00E-147	um12005	26c00007	1.00E-147
BBH	26d00011	um10855	0	um10855	26d00011	0
BBH	26d00033	um10700	6.00E-38	um10700	26d00033	6.00E-38
BBH	26c00020	um10851	2.00E-88	um10851	26c00020	2.00E-81
BBH	26c00060	um04779	0	um04779	26c00060	0
BBH	26d00081	um04720	3.00E-33	um04720	26d00081	7.00E-37
BBH	26c00090	um12280	6.00E-50	um12280	26c00090	6.00E-50
NNN	26d00090	um10671	1.00E-148			
BBH	26c00065	um04764	0	um04764	26c00065	0
BBH	26d00009	um04870	1.00E-90	um04870	26d00009	6.00E-98

BBH	26d00059	um12004	1.00E-132	um12004	26d00059	1.00E-121
BBH	26c00037	um04817	0	um04817	26c00037	0
BBH	26d00064	um04765	1.00E-146	um04765	26d00064	1.00E-144
BBH	26c00013	um10856	0	um10856	26c00013	0
BBH	26c00029	um04832	1.00E-96	um04832	26c00029	1.00E-114
BBH	26c00088	um04715	3.00E-85	um04715	26c00088	3.00E-87
BBH	26d00014	um12283	1.00E-33	um12283	26d00014	9.00E-23
BBH	26d00037	um10699	0	um10699	26d00037	0
BBH	26c00083	um04723	1.00E-101	um04723	26c00083	1.00E-104
BBH	26d00039	um10695	0	um10695	26d00039	0
BBH	26d00052	um04787	0	um04787	26d00052	0
BBH	26c00028	um04838	1.00E-126	um04838	26c00028	1.00E-125
BBH	26d00030	um04835	0	um04835	26d00030	0
BBH	26c00054	um10689	0	um10689	26c00054	0
BBH	26d00040	um10694	1.00E-167	um10694	26d00040	0
BBH	26d00023	um04846	0	um04846	26d00023	0
BBH	26d00099	um04695	0	um04695	26d00099	0
BBH	26d00043	um04811	0	um04811	26d00043	0
NNN	26c00004	um11514	6.00E-64	um11514	13d00001	0
BBH	26d00082	um10676	9.00E-91	um10676	26d00082	2.00E-95
BBH	26c00066	um04761	1.00E-102	um04761	26c00066	1.00E-102
BBH	26d00053	um04785	1.00E-142	um04785	26d00053	1.00E-147
BBH	26d00050	um04793	0	um04793	26d00050	0
BBH	26d00063	um04767	2.00E-43	um04767	26d00063	2.00E-43
BBH	26d00049	um04794	0	um04794	26d00049	0
BBH	26c00038	um04816	1.00E-101	um04816	26c00038	1.00E-101
BBH	26c00030	um04831	2.00E-67	um04831	26c00030	4.00E-74
BBH	26d00007	um04875	1.00E-129	um04875	26d00007	1.00E-129
BBH	26d00013	um12284	9.00E-61	um12284	26d00013	2.00E-59
BBH	26d00036	um04827	0	um04827	26d00036	0
BBH	26c00087	um10674	4.00E-52	um10674	26c00087	4.00E-53
BBH	26d00098	um10581	1.00E-162	um10581	26d00098	1.00E-157
BBH	26d00042	um10693	2.00E-35	um10693	26d00042	1.00E-30
BBH	26c00073	um04748	3.00E-41	um04748	26c00073	1.00E-38
BBH	26d00024	um04845	0	um04845	26d00024	0
BBH	26c00035	um04820	1.00E-176	um04820	26c00035	1.00E-178
BBH	26c00014	um04865	2.00E-62	um04865	26c00014	3.00E-63
BBH	26d00076	um04736	4.00E-53	um04736	26d00076	8.00E-53
BBH	26c00027	um04840	0	um04840	26c00027	0
BBH	27d00080	um11545	2.00E-28	um11545	27d00080	8.00E-47
BBH	27d00095	um04683	0	um04683	27d00095	0
BBH	27c00098	um04672	1.00E-112	um04672	27c00098	1.00E-104
BBH	27c00084	um04654	1.00E-66	um04654	27c00084	1.00E-91
BBH	27d00077	um04641	0	um04641	27d00077	0
BBH	27d00016	um11754	3.00E-28	um11754	27d00016	5.00E-17
NNN	27c00053	um15015	0			
BBH	27d00032	um15014	0	um15014	27d00032	0
NNN	27c00028	um02562	6.00E-77	um02562	5c00128	0
BBH	27d00073	um11539	1.00E-118	um11539	27d00073	1.00E-109
BBH	27c00088	um12279	8.00E-45	um12279	27c00088	3.00E-43
BBH	27d00089	um04667	1.00E-132	um04667	27d00089	1.00E-134
BBH	27d00039	um10903	1.00E-174	um10903	27d00039	1.00E-174
BBH	27c00093	um04682	0	um04682	27c00093	0
NNN	27d00023	um04535	1.00E-100			
BBH	27d00040	um04566	1.00E-18	um04566	27d00040	1.00E-19
BBH	27d00031	um04551	1.00E-147	um04551	27d00031	1.00E-160
BBH	27c00087	um11551	1.00E-61	um11551	27c00087	5.00E-63
BBH	27d00062	um04610	1.00E-169	um04610	27d00062	1.00E-170
BBH	27c00052	um04592	1.00E-141	um04592	27c00052	1.00E-135
BBH	27d00017	um04524	1.00E-109	um04524	27d00017	1.00E-108
BBH	27c00029	um15013.2	0	um15013.2	27c00029	1.00E-179
BBH	27c00060	um10990	0	um10990	27c00060	0

BBH	27d00055	um04594	0	um04594	27d00055	0
BBH	27d00065	um04622	0	um04622	27d00065	0
BBH	27d00047	um12272	0	um12272	27d00047	0
BBH	27c00041	um04573	3.00E-46	um04573	27c00041	3.00E-46
BBH	27c00078	um12276	2.00E-41	um12276	27c00078	1.00E-53
BBH	27c00013	um04511	1.00E-168	um04511	27c00013	1.00E-169
BBH	27c00074	um11536	1.00E-146	um11536	27c00074	1.00E-146
BBH	27d00074	um11540	0	um11540	27d00074	0
BBH	27d00101	um11765	0	um11765	27d00101	0
BBH	27d00084	um11548	3.00E-68	um11548	27d00084	5.00E-75
BBH	27c00095	um04678	2.00E-46	um04678	27c00095	5.00E-48
BBH	27c00089	um04666	7.00E-66	um04666	27c00089	7.00E-66
BBH	27d00009	um11986	0	um11986	27d00009	0
BBH	27c00032	um04558	1.00E-104	um04558	27c00032	1.00E-106
BBH	27c00033	um04561	1.00E-134	um04561	27c00033	1.00E-134
BBH	27d00061	um04607	0	um04607	27d00061	0
BBH	27c00014	um04514	2.00E-26	um04514	27c00014	2.00E-20
BBH	27c00021	um04531	1.00E-159	um04531	27c00021	1.00E-172
BBH	27d00013	um11990	2.00E-69	um11990	27d00013	4.00E-75
BBH	27d00026	um04541	7.00E-68	um04541	27d00026	2.00E-68
BBH	27c00094	um04680	0	um04680	27c00094	0
BBH	27c00003	um04496	0	um04496	27c00003	0
BBH	27c00079	um04644	1.00E-143	um04644	27c00079	1.00E-149
BBH	27d00048	um04584	0	um04584	27d00048	0
BBH	27d00066	um11533	2.00E-49	um11533	27d00066	9.00E-50
BBH	27d00082	um04649	0	um04649	27d00082	0
BBH	27c00075	um04637	5.00E-79	um04637	27c00075	2.00E-83
NNN	27d00018	um04525	6.00E-36			
BBH	27d00091	um04671	0	um04671	27d00091	0
BBH	27d00008	um04506	1.00E-126	um04506	27d00008	1.00E-135
BBH	27d00052	um04591	0	um04591	27d00052	0
BBH	27c00072	um04629	0	um04629	27c00072	0
BBH	27c00096	um04677	1.00E-139	um04677	27c00096	1.00E-139
BBH	27d00075	um11541	5.00E-84	um11541	27d00075	6.00E-67
NNN	27d00029	um04546	2.00E-92			
BBH	27c00048	um10911	0	um10911	27c00048	0
NNN	27c00015	um04516.2	0			
BBH	27d00097	um04681	1.00E-130	um04681	27d00097	1.00E-147
BBH	27d00071	um11535.2	5.00E-65	um11535.2	27d00071	3.00E-58
BBH	27c00051	um04590	1.00E-146	um04590	27c00051	1.00E-141
NNN	27c00065	um04619	0			
BBH	27c00001	um04499	1.00E-124	um04499	27c00001	1.00E-124
BBH	27d00014	um04522	0	um04522	27d00014	0
BBH	27c00067	um04623	1.00E-176	um04623	27c00067	1.00E-171
BBH	27c00030	um04550	0	um04550	27c00030	0
BBH	27d00067	um04628	2.00E-76	um04628	27d00067	7.00E-70
BBH	27d00049	um10910	1.00E-100	um10910	27d00049	1.00E-104
BBH	27d00087	um04661	6.00E-77	um04661	27d00087	4.00E-77
BBH	27c00076	um15065	0	um15065	27c00076	0
BBH	27c00044	um04578	0	um04578	27c00044	0
BBH	27d00044	um04572	3.00E-85	um04572	27d00044	7.00E-91
BBH	27d00042	um04576	7.00E-61	um04576	27d00042	3.00E-81
BBH	27c00062	um04611	1.00E-77	um04611	27c00062	2.00E-78
BBH	27c00020	um04526.2	2.00E-92	um04526.2	27c00020	3.00E-96
BBH	27d00021	um04532	0	um04532	27d00021	0
BBH	27c00097	um04674	0	um04674	27c00097	0
BBH	27c00085	um04657	0	um04657	27c00085	0
NNN	27d00006	um04503	0			
BBH	27c00049	um04588	9.00E-76	um04588	27c00049	1.00E-75
BBH	27d00007	um12269.2	0	um12269.2	27d00007	0
NNN	27c00050	um10912	1.00E-68			
BBH	27c00023	um11757.2	1.00E-45	um11757.2	27c00023	2.00E-53

NNN	27d00072	um11537	6.00E-83			
BBH	27d00024	um04539	0	um04539	27d00024	0
BBH	27d00037	um04562	0	um04562	27d00037	0
BBH	27c00016	um04517.2	0	um04517.2	27c00016	0
BBH	27d00051	um12273.2	1.00E-28	um12273.2	27d00051	4.00E-20
BBH	27c00045	um04580	1.00E-57	um04580	27c00045	5.00E-58
NNN	27d00068	um04630	4.00E-39	um04630	27d00069	1.00E-176
BBH	27c00077	um04639	0	um04639	27c00077	0
BBH	27d00098	um04679	1.00E-110	um04679	27d00098	1.00E-111
BBH	27d00056	um10983.2	1.00E-163	um10983.2	27d00056	1.00E-156
BBH	27c00063	um04612	0	um04612	27c00063	0
BBH	27d00022	um04533	1.00E-165	um04533	27d00022	1.00E-164
BBH	27c00070	um04627	4.00E-66	um04627	27c00070	8.00E-71
BBH	27c00031	um04553	1.00E-130	um04553	27c00031	1.00E-134
BBH	27c00019	um11991	0	um11991	27c00019	0
BBH	27d00030	um04549	9.00E-86	um04549	27d00030	3.00E-80
BBH	27d00054	um04593	1.00E-52	um04593	27d00054	1.00E-54
BBH	27d00019	um04529	0	um04529	27d00019	0
BBH	27c00017	um04518	0	um04518	27c00017	0
BBH	27d00038	um04560	5.00E-55	um04560	27d00038	1.00E-64
BBH	27d00050	um04587	0	um04587	27d00050	0
BBH	27c00080	um04646	3.00E-71	um04646	27c00080	1.00E-77
BBH	27c00042	um12271	0	um12271	27c00042	0
BBH	27c00068	um04624	0	um04624	27c00068	0
NNN	27d00046	um04581	6.00E-78			
BBH	27c00018	um11989	0	um11989	27c00018	0
BBH	27c00006	um04494	0	um04494	27c00006	0
BBH	27c00057	um04602	0	um04602	27c00057	0
BBH	27c00022	um11756	6.00E-28	um11756	27c00022	3.00E-26
BBH	27d00002	um04487.2	0	um04487.2	27d00002	0
BBH	27d00005	um04501	1.00E-124	um04501	27d00005	1.00E-124
BBH	27d00028	um04544	1.00E-27	um04544	27d00028	3.00E-13
BBH	27c00036	um10904	1.00E-106	um10904	27c00036	1.00E-101
NNN	27c00046	um10909.2	5.00E-28	um10909.2	27c00047	0
BBH	27c00037	um10905	1.00E-124	um10905	27c00037	1.00E-120
BBH	27c00027	um11759	0	um11759	27c00027	0
NNN	27c00025	um10815	7.00E-24	um10815	22d00064	0
BBH	27c00090	um04668	1.00E-101	um04668	27c00090	1.00E-108
BBH	27c00043	um04577	0	um04577	27c00043	0
BBH	27d00043	um10908	0	um10908	27d00043	0
BBH	27c00064	um03801	2.00E-54	um03801	27c00064	2.00E-54
BBH	27d00041	um10906	5.00E-63	um10906	27d00041	3.00E-68
BBH	27c00069	um04625	2.00E-52	um04625	27c00069	2.00E-52
BBH	27d00059	um04603	0	um04603	27d00059	0
BBH	27d00069	um04630	1.00E-160	um04630	27d00069	1.00E-176
NNN	27d00020	um04530	1.00E-157	um04530	11c00050	2.00E-13
BBH	27d00100	um11761	1.00E-165	um11761	27d00100	1.00E-163
BBH	27d00079	um11544	7.00E-76	um11544	27d00079	3.00E-74
BBH	27d00070	um11534	1.00E-146	um11534	27d00070	1.00E-146
BBH	27c00066	um04621	1.00E-131	um04621	27c00066	1.00E-121
BBH	27d00001	um04497	0	um04497	27d00001	0
BBH	27c00009	um11984	5.00E-86	um11984	27c00009	6.00E-99
BBH	27d00088	um04665	0	um04665	27d00088	0
BBH	27c00047	um10909.2	0	um10909.2	27c00047	0
BBH	27d00004	um11983	2.00E-29	um11983	27d00004	5.00E-29
NNN	27d00035	um05602	1.00E-59	um05602	16d00084	0
BBH	27d00027	um04543	0	um04543	27d00027	0
BBH	27c00005	um11982	0	um11982	27c00005	0
BBH	27c00040	um04575	5.00E-24	um04575	27c00040	2.00E-18
BBH	27c00010	um04505	1.00E-157	um04505	27c00010	1.00E-157
BBH	27c00082	um12278	1.00E-45	um12278	27c00082	3.00E-42
BBH	27d00092	um11763	4.00E-93	um11763	27d00092	3.00E-94

BBH	27d00063	um04613	0	um04613	27d00063	0
BBH	27d00058	um04599	6.00E-92	um04599	27d00058	1.00E-107
BBH	27d00083	um04650	1.00E-149	um04650	27d00083	1.00E-148
BBH	27d00011	um11987	2.00E-85	um11987	27d00011	6.00E-97
BBH	27d00010	um04509	8.00E-63	um04509	27d00010	9.00E-61
NNN	27c00026	um01208	8.00E-25	um01208	7c00160	3.00E-76
BBH	27d00034	um10901	1.00E-113	um10901	27d00034	1.00E-113
BBH	27c00024	um04537	8.00E-32	um04537	27c00024	5.00E-28
BBH	27d00060	um04605	7.00E-84	um04605	27d00060	4.00E-82
BBH	27d00003	um04489	3.00E-81	um04489	27d00003	6.00E-75
BBH	27d00033	um04555	0	um04555	27d00033	0
BBH	27c00039	um10907	8.00E-60	um10907	27c00039	3.00E-59
BBH	27d00025	um11758	7.00E-47	um11758	27d00025	1.00E-57
BBH	27c00055	um10986	1.00E-17	um10986	27c00055	4.00E-22
BBH	27c00002	um04498	1.00E-148	um04498	27c00002	1.00E-140
BBH	27c00083	um11549	0	um11549	27c00083	0
BBH	27c00061	um04609	0	um04609	27c00061	0
BBH	27c00059	um10989	1.00E-69	um10989	27c00059	4.00E-78
BBH	27d00086	um04656.2	0	um04656.2	27d00086	0
BBH	27d00081	um11546	4.00E-19	um11546	27d00081	1.00E-24
BBH	27d00064	um11532	1.00E-61	um11532	27d00064	3.00E-67
NNN	27c00073	um04632	6.00E-28			
BBH	27d00057	um10985	1.00E-135	um10985	27d00057	1.00E-136
BBH	27d00045	um04579	0	um04579	27d00045	0
BBH	27c00004	um04488	3.00E-66	um04488	27c00004	7.00E-68
BBH	27c00011	um04508	1.00E-128	um04508	27c00011	1.00E-119
BBH	27d00015	um04523	0	um04523	27d00015	0
NNN	27c00035	um04563	0			
BBH	27c00007	um04495	2.00E-86	um04495	27c00007	6.00E-93
BBH	27c00081	um12277	2.00E-29	um12277	27c00081	1.00E-30
BBH	27d00099	um04676	1.00E-71	um04676	27d00099	1.00E-105
BBH	27c00086	um04659	0	um04659	27c00086	0
BBH	27c00012	um04510	3.00E-37	um04510	27c00012	5.00E-35
BBH	27d00102	um04673	0	um04673	27d00102	0
NNN	27d00085	um04655	1.00E-162			
BBH	27c00058	um10988	4.00E-71	um10988	27c00058	4.00E-71
BBH	27c00092	um04686	0	um04686	27c00092	0
BBH	27c00054	um04598	0	um04598	27c00054	0
BBH	27d00090	um04669	0	um04669	27d00090	0
BBH	48c00001	um00250	3.00E-65	um00250	48c00001	2.00E-53
NNN	89d00001	um02101	1.00E-49	um02101	6d00101	4.00E-72
NNN	156c00001	um05574	1.00E-62	um05574	16d00065	1.00E-110

[1]NNN, not bi-directional best hit

[2]BBH, bi-directional best hit
