# Supplementary material for: Genome and Transcriptome Analysis of the Basidiomycetous Yeast Pseudozyma antarctica Producing Extracellular Glycolipids, Mannosylerythritol Lipids
Source: PLoS One. 2014 Feb 24;9(2):e86490. doi: 10.1371/journal.pone.0086490 (PMC3933340; doi:10.1371/journal.pone.0086490)
Supplement: Table S4 — Putative plant cell wall-degrading enzymes in P. antarctica and other fungi. (PDF) [file pone.0086490.s005.pdf]

Supplementary Table S4

Putative plant cell wall-degrading enzymes in *P. antarctica* and other fungi

**Detailed comparison between *P. antarctica* and *U. maydis***					**Total number of enzymes in various fungi [50]**										
**Family**	**Glycoside hydrolases**	***P. antarctica* genes**	**Pa**	**Um**	**Pa**	**Um**	**An**	**Mg**	**Fg**	**Nc**	**Sc**	**Sp**	**Pc**	**Cc**	**Cn**
1	β−glucosidase	8c00129, 13d00013			0	0	3	3	3	1	0	0	2	2	0
2	β−galactosidase				1	1	9	6	5	5	0	0	0	0	0
	β−mannosidase	2c00076	1	1											
	β−glucuronidase														
3	β−glucosidase	13c00090, 9d00216, 20d00032, 1d00031	4	3	4	3	20	19	18	9	0	1	7	6	3
	xylan 1,4-β-xylosidase														
	alphα-L-arabinofuranosidase														
	β-N-acetylhexosaminidase														
5	endomannanase	22c00082	1	1	11	10	15	13	2	7	6	3	4	1	12
	β−1,4-endoglucanase														
	β−1,3-exoglucanase	9c00139, 7c00332, 7d00186, 11c00030, 20c00026, 9d00068, 15c00030, 12d00021, 20c00034. 20d00008	10	9											
	β−1,6-glucanase 1														
	endoglycoceramidase 1														
6	cellobiohydrolase type II				0	0	2	3	1	3	0	0	1	4	0
7	cellobiohydrolase type I				0	0	3	6	2	5	0	0	5	3	0
8	β−1,4-endoglucanase	7d00341	1	1	1	1	0	0	0	0	0	0	0	0	0
10	endoxylanase	8c00118, 11c00065	2	2	2	2	3	5	5	4	0	0	6	3	0
11	endoxylanase	22d00001	1	1	1	1	2	5	2	2	0	0	1	6	0
12	xyloglucan-endoglucanase				0	0	1	3	3	1	0	0	2	0	0
26	endomannanase				0	0	3	0	0	1	0	0	0	0	0
27	α−galactosidase	27d00006, 18c00065	2	1	1	2	3	4	2	0	5	1	3	0	0
28	endopolygalacturonase				1	1	9	3	6	2	1	0	1	1	1
	exopolygalacturonase	5c00162	1	1											

	xylogalacturonan hydrolase rhamnogalacturonan														
35	β–galactosidase	7c00120	1	1	1	1	4	0	2	2	0	0	1	0	0
36	α–galactosidase				0	0	4	2	2	1	0	0	0	0	1
42	β–galactosidase	6d00052	1	1	1	1	0	0	0	0	0	0	0	0	0
43	endoarabinanase<br>α–L-arabinofuranosidase<br>β–xylosidase 8	12c00003, 8c00113, 18c00104, 9c00439	4	2	2	4	15	18	1	6	0	0	0	1	0
45	β–1,4-endoglucanase	5d00140, 26c00038, 4d00037	3	3	3	3	1	1	0	1	0	0	0	0	0
51	α–L-arabinofuranosidase	7c00355, 22c00212, 26d00002	3	2	3	2	2	3	2	1	0	0	1	0	1
53	endogalactanase				0	0	1	1	1	1	0	0	1	1	0
54	α–L-arabinofuranosidase	22c00021	1		1	0	1	1	1	1	0	0	0	0	0
61	β–1,4-endoglucanase				0	0	9	17	12	14	0	0	12	12	1
62	arabinoxylan arabinofuranohydrolase	9d00343	1	1	1	1	2	3	1	0	0	0	0	1	0
63	α-1,3-glucosidase														
67	α–glucuronidase	5c00036	1		1	0	1	1	1	1	0	0	0	0	0
74	xyloglucan-endoglucanase				0	0	2	1	1	1	0	0	2	1	0
78	α–rhamnosidase				0	0	8	1	3	0	0	0	0	0	2
93	exoarabinanase				0	0	2	1	2	2	0	0	0	0	0
95	α–L-fucosidase				0	0	3	1	2	0	0	0	1	0	0

**Family**	**Polysaccharide lyases**	***P. antarctica* genes**	**Pa**	**Um**	**Pa**	**Um**	**An**	**Mg**	**Fg**	**Nc**	**Sc**	**Sp**	**Pc**	**Cc**	**Cn**
1	pectin lyase<br>pectate lyase	26d00090	1	1	1	1	8	2	6	1	0	0	0	0	0
3	pectate lyase				0	0	5	1	7	1	0	0	0	1	0
4	rhamnogalacturonan lyase				0	0	4	1	1	1	0	0	0	2	1
9	pectate lyase				0	0	1	0	1	0	0	0	0	0	0
11	rhamnogalacturonan lyase				0	0	1	0	1	0	0	0	0	0	0

**Family**	**Carbohydrate esterases**	***P. antarctica* genes**	**Pa**	**Um**	**Pa**	**Um**	**An**	**Mg**	**Fg**	**Nc**	**Sc**	**Sp**	**Pc**	**Cc**	**Cn**

1	acetyl xylan esterase	27d00092	1	1	2	1	3	10	3	7	1	0	0	3	2
	feruloyl esterase	9c00307	1												
	unknown														
8	pectin methyl esterase			1	1	0	3	1	3	1	0	0	1	0	0
12	rhamnogalacturonan acetylesterase unknown				0	0	2	2	1	1	0	0	0	1	0

Abbbreviations: Pa (*Pseudozyma antarctica*), Um (*Ustilago maydis*), An (*Aspergillus nidulans*), Mg (*Magnaporthe grisea*), Fg (*Fusarium graminearum*), Nc (*Neurospora crassa*), Sc (*Saccharomyces cerevisiae*), Sp (*Schizosaccharomyces pombe*), Pc (*Phanerochaete chrysosporium*), Cc (*Coprinus cinereus*), Cn (*Cryptococcus neoformans*).
