# Supplementary material for: Genome and Transcriptome Analysis of the Basidiomycetous Yeast Pseudozyma antarctica Producing Extracellular Glycolipids, Mannosylerythritol Lipids
Source: PLoS One. 2014 Feb 24;9(2):e86490. doi: 10.1371/journal.pone.0086490 (PMC3933340; doi:10.1371/journal.pone.0086490)
Supplement: Table S5 — Gene clusters for secreted proteins in P. antarctica . (PDF) [file pone.0086490.s006.pdf]

Supplementary Table S5

Gene clusters for secreted proteins in *P. antarctica*

**Cluster**	**Virulence of deletion mutants of *U. maydis***	***U. maydis***		***P. antarctica***		**e-value**		**Description**
		**Genes**	**Protein length (aa)**	**Identical genes**	**Protein length (aa)**	**Um to Pa**	**Pa to Um**	
1A	unaffected	um00444	537	9c00246	523	1.00E-144	1.00E-170	hypothetical protein
		um10115	83	9d00215			0.011	
		um00445	224	9c00247			5.00E-81	
		um00446	819	9d00216	819	0	0	beta-glucosidase
		um00447	603	9d00217	417	1.00E-47	2.00E-41	hypothetical protein
2A	increased	um01233	492	7d00152	490	0	0	D-3-phosphoglycerate dehydrogenase
		um01234	441	7c00150	421	2.00E-64	6.00E-58	hypothetical protein
		um01235	574	7d00151			6.00E-21	
		um01236	641	7d00151			3.00E-14	
		um01237	633	7d00151	528	3.00E-24	1.00E-23	hypothetical protein
		**um01238**	306					
		**um01239**	283					
		**um01240**	279					
		um01241	177	7d00150	212	2.00E-20	8.00E-24	hypothetical protein
		um01242	705	7d00149		0	0	Signal recognition particle receptor, alpha subunit
2B	unaffected	um01296	878	12c00065	559	0	0	Nuclear AAA ATPase (VCP subfamily)
		um01297	177	12d00063	703	0	0	
		um01298	266	12d00063			2.00E-13	
		um01299	162	12d00062			0.084	
		um01300	136	12c00064			5.00E-11	
		um01301	142	12c00064	136	6.00E-15	4.00E-18	hypothetical protein
		um01302	149	12c00064			0.032	
		um10233	175	12d00062			3.00E-22	
		um10234	604	12d00061	603	0	0	poly(A)+ mRNA-nucleus export-related protein
3A	unaffected	um01885	354	22d00222	354	1.00E-160	1.00E-170	alcohol dehydrogenase (predicted)
		um01886	589	22d00223	589	0	0	carboxypeptidase C
		um01887	135	22c00245			2.00E-13	
		um01888	583	22d00248	184	0	0	hypothetical protein
		um01889	1006	22d00249	601	0	0	hypothetical protein
5A	unaffected	um02191	758	6c00052	773	1.00E-145	1.00E-151	hypothetical protein
		**um02192**	210					
		**um02193**	113					
		**um02194**	109					
		um10070	558	6c00051	560	0	0	Cytochrome P450 CYP3/CYP5/CYP6/CYP9 subfamilies
		um02196	678	6d00058	469	4.00E-27	3.00E-29	hypothetical protein
		um02197	430	6d00056	438	1.00E-162	1.00E-156	hypothetical protein
5B	non-pathogenic	um02472	638	12c00131	636	2.00E-87	1.00E-115	hypothetical protein

		um02473	135	12d00091	131	4.00E-12	3.00E-15	
		um02474	230	12d00091			1.00E-07	
		um02475	515	12c00130	141	1.00E-41	6.00E-38	
		um02476	554	12d00122	551	0	0	Coatomer subunit delta, putative
6A	reduced	um11414	280	5c00147	279	1.00E-105	1.00E-114	cytoplasm protein
		**um02533**	429					
		um11415	166	5c00143-6			7.00E-12	
		um02535	223	5c00145	155	1.00E-14	1.00E-14	hypothetical protein
		um11416	197	5c00143-6				
		um02537	166	5c00143-6				
		um02538	160	5c00143-6				
		**um11417**	179					
		um02540	208	5c00142	187	1.00E-20	1.00E-20	hypothetical protein
		um02541	152	5d00135			3.00E-26	
		um02542	502	5c00141	487	1.00E-103	1.00E-100	hypothetical protein
8A	unaffected	um03200	1075	14c00098	917	0	1.00E-180	DNA recombinational repair protein BRCA2
		um03201	235	14d00089	196	4.00E-40	5.00E-40	
		**um03202**	126					
		**um03203**	129					
		um03204	2005	14d00088	1981	0	0	chitin synthase-related
9A	unaffected	um03614	489	8d00029	472	0	0	hypothetical protein
		um03615	693	8c00030	710	0	0	hypothetical protein
		um03616	703	8d00030	702	0	0	hypothetical protein
		um03617	2358	8c00031	2332	0	0	nucleolus protein
10A	reduced	um03743	724	10c00054	619	1.00E-171	1.00E-166	Actin-like protein 13E
		um03744	680	10d00070	529	1.00E-26	5.00E-27	hypothetical protein
		um03745	263	10d00069			2.00E-06	
		um03746	304	10d00068	571	1.00E-18	3.00E-19	hypothetical protein
		um03747	223	22c00215	263	7.00E-11	9.00E-16	hypothetical protein
		um03748	238	10d00067			8.00E-12	
		um03749	267	10d00066			9.00E-28	
		um03750	240	10d00069			2.00E-08	
		um03751	282	10d00066			4.00E-24	
		um03752	398	10d00066			7.00E-28	
		um03753	363	10d00066	970	3.00E-35	8.00E-36	hypothetical protein
		um03754	378	10d00065	374	1.00E-100	5.00E-96	protein N-terminal asparagine amidohydrolase
		um10660	309	10c00053			e-102	
19A	Markedly reduced	um05293	799	15c00068	808	0	0	Oligosaccharyltransferase, STT3 subunit
		um05294	204	15c00067			0.057	
		um05295	209	15c00067	198	3.00E-16	4.00E-16	hypothetical protein
		um05296	248	15c00067			9.00E-14	
		**um10553**	214					
		**um10554**	193					
		um05299	234	15c00067			0.12	

		um05300	218					
		um05301	251	15c00063			1.5	
		um05302	207	15c00065	248	2.00E-25	2.00E-25	hypothetical protein
		**um05303**	193					
		um10555	200	15c00064			5.00E-06	
		um05305	227	15c00064			0.023	
		um05306	287	15c00064			6.00E-22	
		um10556	264	15d00060	224	2.00E-20	2.00E-20	hypothetical protein
		um05308	242	15c00063	305	2.00E-32	8.00E-30	hypothetical protein
		um05309	195	15c00064			6.00E-22	
		**um05310**	195					
		**um05311**	220					
		**um05312**	182					
		**um05313**	494					
		**um05314**	198					
		**um10557**	180					
		**um05316**	246					
		**um05317**	178					
		**um05318**	183					
		**um05319**	185					
		um10558	471	15c00059	447	0	0	Tubulin beta chain
22A	unaffected	um11233	254	24d00040	245	1.00E-22	1.00E-101	60s ribosomal protein L2/L8
		um06221	200	24c00044			1.00E-10	
		**um06222**	163					
		**um06223**	216					
		um06224	1225	24c00047			0	
