# Supplementary material for: Genome and Transcriptome Analysis of the Basidiomycetous Yeast Pseudozyma antarctica Producing Extracellular Glycolipids, Mannosylerythritol Lipids
Source: PLoS One. 2014 Feb 24;9(2):e86490. doi: 10.1371/journal.pone.0086490 (PMC3933340; doi:10.1371/journal.pone.0086490)
Supplement: Table S6 — Transcriptome analysis of P. antarctica and U. maydis under the oily conditions. (PDF) [file pone.0086490.s007.pdf]

Supplementary Table S6

Transcriptome analysis of *P. antarctica* and *U. maydis* under the oily conditions

P. antarctica

Gene	M-value	A-value
10c0000	0.00	11.63
10c0000	0.14	12.87
10c0000	-0.32	12.52
10c0000	-0.61	15.04
10c0000	-0.47	11.54
10c0000	0.44	12.28
10c0000	0.14	10.30
10c0000	0.52	12.37
10c0000	0.17	9.69
10c0001	0.41	12.08
10c0001	-0.02	13.52
10c0001	-0.45	11.66
10c0001	-0.28	12.73
10c0001	-0.26	12.53
10c0001	0.43	11.50
10c0001	0.38	10.50
10c0001	-0.31	12.93
10c0001	0.32	10.92
10c0001	-0.06	12.45
10c0002	-0.07	13.01
10c0002	-0.23	12.28
10c0002	-0.18	9.56
10c0002	0.21	13.39
10c0002	-0.25	12.96
10c0002	-0.04	12.03
10c0002	-0.06	12.24
10c0002	-0.28	13.32
10c0002	-0.12	10.78
10c0002	-0.01	9.29
10c0003	-0.21	12.02
10c0003	0.13	14.24
10c0003	-0.21	9.70
10c0003	0.66	14.29
10c0003	-0.37	10.45
10c0003	0.30	11.41
10c0003	-0.40	15.21
10c0003	1.18	11.86
10c0003	0.43	12.92
10c0003	-0.08	13.30
10c0004	0.20	12.41
10c0004	-0.32	12.86
10c0004	-0.13	12.99
10c0004	0.00	13.62
10c0004	0.78	12.01
10c0004	-0.92	11.29
10c0004	0.69	13.82

U. maydis

Gene	M-value	A-value
um00001	-0.26	12.08
um00002	0.03	11.81
um00003	0.42	11.84
um00005	2.22	12.76
um00006	0.00	11.67
um00007	-0.22	12.76
um00008	0.08	11.50
um00009	-0.53	13.18
um00011	-0.97	11.80
um00012	0.30	11.25
um00013	-0.47	12.23
um00017	-0.02	11.80
um00020	-1.32	12.47
um00025	-3.16	10.56
um00026	-0.70	11.97
um00027	-3.94	13.26
um00028	-0.88	13.96
um00029	0.04	9.50
um00030	0.34	10.66
um00031	0.28	12.33
um00032	-0.12	9.03
um00034	1.65	11.45
um00036	-0.19	14.66
um00037	2.55	11.45
um00038	1.22	10.41
um00039	0.48	11.33
um00040	-0.06	11.67
um00041	0.31	12.32
um00042	0.05	10.70
um00043	-0.62	12.00
um00045	1.16	12.91
um00046	-0.63	11.26
um00047	0.57	11.34
um00048	-0.20	12.11
um00049	0.40	11.77
um00050	0.99	14.66
um00052	0.34	12.03
um00053	0.54	10.97
um00054	0.00	9.24
um00055	0.08	10.93
um00056	0.32	9.14
um00057	-0.18	11.97
um00058	0.18	9.57
um00059	-0.28	12.30
um00060	0.51	9.25
um00061	0.11	11.34

10c0004	-0.03	12.10	um00062	0.11	9.04
10c0004	-0.19	11.53	um00063	0.88	12.08
10c0004	0.34	11.61	um00064	-0.27	9.20
10c0005	-0.26	11.59	um00067	0.17	12.75
10c0005	-0.38	10.99	um00068	0.33	10.75
10c0005	0.00	12.00	um00071	-0.06	11.74
10c0005	-0.08	9.69	um00072	-0.63	13.20
10c0005	0.33	11.06	um00074	0.29	11.36
10c0005	0.30	15.08	um00076	-0.05	11.28
10c0005	0.09	11.54	um00077	0.58	11.61
10c0005	0.29	13.04	um00078	1.06	10.67
10c0005	0.37	14.66	um00079	1.73	10.82
10c0005	-0.13	8.55	um00080	-0.32	11.64
10c0006	0.10	10.34	um00081	-0.54	11.27
10c0006	-0.01	8.56	um00082	-1.50	9.85
10c0006	0.43	11.04	um00083	-0.85	9.68
10c0006	-0.37	13.00	um00084	0.05	10.67
10c0006	-0.19	12.38	um00085	0.53	10.79
10c0006	0.05	11.52	um00087	-0.84	13.03
10c0006	-0.20	10.64	um00089	0.11	11.86
10c0006	-0.50	12.75	um00090	0.46	9.94
10c0006	-0.12	12.80	um00091	0.16	11.19
10c0006	0.13	11.25	um00092	0.50	13.07
10c0007	-0.94	12.29	um00094	-0.23	14.49
10c0007	0.14	12.05	um00095	-0.19	12.84
10c0007	-0.16	12.29	um00096	-2.27	14.00
10c0007	0.24	11.49	um00097	0.21	12.35
10c0007	0.06	12.69	um00098	0.54	11.68
10c0007	0.01	11.85	um00099	-0.03	12.51
10c0007	0.74	10.38	um00101	0.02	10.62
10c0007	0.12	10.69	um00102	0.38	9.16
10c0007	-0.41	15.47	um00103	1.02	11.81
10c0007	-0.02	9.65	um00104	-0.17	8.75
10c0008	-0.02	8.72	um00105	-0.18	11.90
10c0008	0.19	11.87	um00108	-1.50	12.77
10c0008	-0.09	11.94	um00109	-0.37	14.14
10c0008	0.40	11.44	um00111	1.25	13.98
10c0008	0.49	15.48	um00113	0.42	11.82
10c0008	-0.10	8.83	um00114	0.30	11.64
10c0008	0.31	9.71	um00115	-2.38	11.82
10c0008	0.09	9.61	um00116	1.21	10.19
10c0008	0.10	11.50	um00117	1.09	11.44
10c0008	-0.01	12.65	um00118	-1.40	9.56
10c0009	0.47	14.38	um00119	0.31	12.34
10c0009	0.09	13.12	um00120	0.15	11.17
10c0009	0.06	11.37	um00122	1.19	11.93
10c0009	0.09	10.51	um00123	0.56	10.89
10c0009	-0.01	13.32	um00124	0.63	12.34
10c0009	-1.05	12.72	um00125	0.61	12.29
10c0009	-0.78	14.03	um00126	-0.20	11.89
10c0009	-0.61	13.97	um00127	0.48	11.52
10c0009	-0.12	13.16	um00130	-0.30	13.53
10c0009	-0.84	13.43	um00131	0.30	11.83
10c0010	-0.01	12.00	um00132	-0.63	11.12

10c0010	-0.10	11.92	um00133	0.60	9.94
10c0010	0.08	9.26	um00136	-0.01	9.94
10c0010	-0.59	14.41	um00137	0.28	11.12
10c0010	0.02	8.81	um00138	-0.83	11.74
10c0010	-0.42	10.90	um00140	0.32	10.77
10c0010	0.17	11.62	um00141	-0.59	9.92
10d0000	0.27	13.65	um00144	0.46	10.57
10d0000	0.03	11.90	um00145	-0.20	11.95
10d0000	-0.45	13.40	um00146	0.50	12.69
10d0000	-0.65	14.30	um00148	0.49	9.55
10d0000	-0.46	11.96	um00149	0.03	11.88
10d0000	0.13	11.55	um00152	0.42	12.10
10d0000	0.48	12.68	um00153	-0.06	13.59
10d0000	-0.98	14.85	um00154	0.19	9.11
10d0000	0.18	11.37	um00156	-0.08	10.07
10d0001	0.07	15.17	um00157	-1.85	14.02
10d0001	0.46	13.03	um00159	-0.33	12.71
10d0001	0.67	12.51	um00160	0.36	14.25
10d0001	0.72	13.33	um00161	0.25	11.26
10d0001	0.08	15.01	um00163	-0.32	11.73
10d0001	0.18	9.36	um00164	1.43	11.88
10d0001	-0.12	12.06	um00166	0.69	10.20
10d0001	0.48	12.61	um00167	-0.10	10.35
10d0001	0.55	12.58	um00168	-0.33	11.56
10d0001	0.14	11.22	um00169	0.07	12.75
10d0002	0.28	13.68	um00171	0.12	11.34
10d0002	-0.31	14.01	um00172	-0.21	9.60
10d0002	0.10	12.84	um00174	-0.39	12.43
10d0002	-0.39	13.39	um00175	0.26	13.10
10d0002	-0.18	9.97	um00176	0.22	13.34
10d0002	-0.86	12.81	um00177	-0.92	12.05
10d0002	0.38	13.10	um00179	0.87	11.41
10d0002	0.50	11.50	um00180	-0.03	10.49
10d0002	-0.01	11.10	um00181	0.14	10.81
10d0002	0.44	14.09	um00182	2.23	10.65
10d0003	0.10	13.97	um00183	0.42	10.85
10d0003	0.26	12.88	um00184	0.09	10.58
10d0003	0.12	10.92	um00187	-0.16	8.63
10d0003	0.02	12.46	um00188	1.15	9.97
10d0003	-0.08	12.50	um00189	0.15	10.29
10d0003	-0.03	14.05	um00191	0.54	10.55
10d0003	-1.17	14.42	um00192	0.25	12.74
10d0003	0.20	12.22	um00193	0.10	12.71
10d0003	-0.23	11.89	um00194	-0.11	8.91
10d0003	0.22	12.41	um00195	0.12	11.90
10d0004	-0.41	12.79	um00196	1.83	13.01
10d0004	-0.05	12.92	um00197	1.33	12.76
10d0004	1.19	13.26	um00199	-0.06	12.28
10d0004	0.28	9.97	um00202	0.47	9.46
10d0004	0.54	12.90	um00203	0.47	10.64
10d0004	-0.14	12.93	um00204	-0.32	13.33
10d0004	0.27	12.84	um00205	2.97	13.91
10d0004	-0.18	12.44	um00206	0.65	11.45
10d0004	0.19	9.94	um00207	0.27	11.86

10d0004	0.20	10.72	um00208	-0.54	10.24
10d0005	-0.08	12.21	um00209	2.34	13.31
10d0005	-0.85	11.18	um00210	-0.40	13.77
10d0005	0.23	14.06	um00211	-0.73	10.57
10d0005	-0.74	13.12	um00212	0.03	11.50
10d0005	0.36	11.19	um00213	0.32	11.84
10d0005	0.27	11.76	um00215	0.30	11.38
10d0005	-0.54	12.27	um00216	-0.26	11.62
10d0005	-0.12	9.94	um00218	0.31	11.09
10d0005	-0.12	13.00	um00219	-0.60	11.68
10d0005	-0.30	13.38	um00221	0.27	12.51
10d0006	0.10	14.27	um00222	0.97	9.86
10d0006	0.25	12.66	um00223	0.45	9.31
10d0006	-0.09	12.18	um00224	0.01	10.69
10d0006	-0.29	12.04	um00225	0.36	11.38
10d0006	0.02	11.36	um00226	0.46	11.65
10d0006	-0.33	11.43	um00227	-0.33	11.35
10d0006	0.01	8.55	um00229	1.59	11.73
10d0006	-0.06	9.21	um00230	1.73	10.13
10d0006	0.11	8.64	um00233	0.13	11.38
10d0006	0.19	10.40	um00235	-0.20	11.24
10d0007	0.35	11.49	um00236	1.27	9.72
10d0007	0.01	11.71	um00238	-0.41	12.75
10d0007	0.06	11.06	um00239	-0.20	11.72
10d0007	0.82	12.42	um00240	0.00	10.76
10d0007	0.52	13.17	um00241	1.84	13.80
10d0007	-0.12	11.99	um00242	0.14	10.55
10d0007	0.16	13.90	um00244	0.04	12.37
10d0007	0.05	13.87	um00245	-0.95	11.55
10d0007	0.26	10.19	um00247	0.66	12.03
10d0007	-0.97	14.86	um00249	0.31	11.89
10d0008	-0.14	13.01	um00250	-0.01	14.72
10d0008	-0.37	12.50	um00251	0.25	10.83
10d0008	-0.23	12.02	um00254	-0.33	9.18
10d0008	0.46	10.06	um00255	0.93	11.72
10d0008	0.16	14.32	um00256	-0.05	13.18
10d0008	0.53	14.76	um00258	-0.11	11.71
10d0008	0.15	13.20	um00259	0.13	10.68
10d0008	0.06	10.50	um00262	-0.22	15.23
10d0008	-0.10	11.95	um00263	0.43	11.97
10d0008	-0.10	11.29	um00264	-0.94	10.74
10d0009	0.21	11.61	um00265	-1.07	12.49
10d0009	-0.09	8.74	um00266	-0.18	11.09
10d0009	0.35	12.77	um00270	0.21	12.41
10d0009	-0.17	12.35	um00271	-1.06	13.56
10d0009	0.22	12.62	um00272	0.46	12.33
10d0009	0.05	13.09	um00273	0.26	14.41
10d0009	-0.63	10.36	um00274	0.10	13.65
10d0009	-0.77	13.56	um00275	-0.22	11.26
10d0009	-0.05	15.89	um00276	0.01	8.42
10d0009	0.56	10.38	um00277	-0.22	10.63
10d0010	-0.11	11.82	um00278	-0.19	9.24
10d0010	0.59	13.88	um00280	-0.20	11.91
10d0010	-0.60	11.86	um00282	0.15	11.56

10d0010	0.04	11.37	um00283	-1.14	11.83
10d0010	0.59	12.61	um00284	0.05	12.17
10d0010	0.21	12.19	um00286	0.84	11.92
10d0010	0.20	10.45	um00288	0.79	11.02
10d0010	-0.25	12.07	um00289	0.07	10.76
10d0010	-0.07	11.23	um00290	-0.30	10.39
10d0010	0.00	8.76	um00291	-0.38	12.18
10d0011	0.11	13.35	um00292	0.40	11.21
10d0011	0.72	13.38	um00293	0.19	10.65
10d0011	-0.08	12.56	um00295	0.66	14.34
10d0011	-0.08	12.68	um00296	-0.69	11.83
10d0011	-0.07	12.04	um00297	0.67	12.10
10d0011	0.21	9.02	um00298	0.33	11.61
10d0011	0.11	11.24	um00299	0.04	11.33
10d0011	-0.08	10.49	um00300	-0.10	10.80
10d0011	-0.02	10.19	um00302	-0.35	10.89
10d0011	0.03	11.23	um00304	-0.48	12.13
10d0012	0.08	8.73	um00305	0.19	10.97
10d0012	0.10	11.47	um00306	-0.07	8.64
10d0012	-0.52	10.35	um00308	0.87	10.48
10d0012	0.24	10.45	um00309	-5.39	13.34
11c0000	-0.16	13.32	um00310	-0.10	10.44
11c0000	-0.20	8.92	um00311	-1.12	12.59
11c0000	-0.05	8.84	um00315	-1.08	13.41
11c0000	0.34	12.20	um00316	-1.60	11.23
11c0000	-0.30	12.16	um00317	0.45	11.18
11c0000	-0.42	12.51	um00318	-0.16	11.47
11c0000	0.18	14.42	um00319	-0.43	11.07
11c0000	-0.43	9.98	um00321	0.15	11.25
11c0000	-0.22	12.07	um00322	0.41	10.33
11c0001	-0.42	13.53	um00325	0.45	11.24
11c0001	-0.09	13.21	um00326	-0.29	14.73
11c0001	-0.53	13.15	um00328	-0.30	10.25
11c0001	0.03	11.48	um00329	0.06	12.28
11c0001	-0.79	13.45	um00330	-1.27	11.77
11c0001	-0.20	13.59	um00332	0.53	10.74
11c0001	0.35	10.50	um00333	0.19	11.67
11c0001	-0.01	9.59	um00334	-0.34	12.66
11c0001	-0.07	12.82	um00336	2.11	13.60
11c0001	-0.16	9.52	um00337	-0.20	9.17
11c0002	0.02	10.74	um00338	0.05	11.77
11c0002	0.29	14.60	um00340	0.53	11.70
11c0002	-0.36	10.91	um00342	-0.08	10.66
11c0002	0.32	13.53	um00343	1.23	14.74
11c0002	-1.24	12.18	um00344	0.05	11.13
11c0002	0.07	9.01	um00347	-0.23	10.73
11c0002	0.50	9.85	um00349	0.39	11.75
11c0002	1.29	14.28	um00350	0.45	13.44
11c0002	1.38	11.25	um00353	0.38	11.98
11c0002	-1.05	12.49	um00354	0.60	10.77
11c0003	-1.53	11.55	um00355	0.41	10.44
11c0003	0.25	10.23	um00356	-0.33	11.47
11c0003	-0.10	13.54	um00357	0.31	13.49
11c0003	-0.38	12.29	um00358	-0.08	8.81

11c0003	-0.25	12.74	um0036	0.25	12.13
11c0003	0.65	10.11	um00361	-0.19	11.69
11c0003	0.09	12.94	um0036	-0.16	12.59
11c0003	-0.54	12.49	um0036	0.00	10.58
11c0003	-0.29	11.60	um0036	-0.15	10.35
11c0003	-0.13	11.05	um0036	-0.47	11.73
11c0004	0.01	8.98	um0036	0.40	10.91
11c0004	-0.31	11.71	um0037	0.35	11.95
11c0004	-0.53	13.55	um00371	2.30	14.30
11c0004	-0.25	12.48	um0037	-0.46	12.29
11c0004	0.22	12.96	um0037	-2.85	12.81
11c0004	0.17	11.13	um0037	0.27	9.73
11c0004	0.19	12.88	um0037	0.37	11.87
11c0004	0.10	13.74	um0037	-0.48	9.71
11c0004	0.17	12.91	um0037	0.23	13.17
11c0004	0.23	13.63	um0038	0.54	11.26
11c0005	0.80	10.85	um00381	0.69	14.03
11c0005	-0.12	13.28	um0038	-0.37	9.10
11c0005	0.13	9.59	um0038	-0.73	11.75
11c0005	0.34	10.88	um0038	-0.79	12.86
11c0005	-0.02	11.18	um0038	-0.25	15.47
11c0005	-0.23	13.24	um0038	-0.14	8.65
11c0005	-0.21	11.63	um0038	-0.12	11.18
11c0005	0.15	15.78	um0039	0.23	10.60
11c0005	-0.14	13.17	um00391	-0.18	12.25
11c0005	0.47	12.15	um0039	-0.73	12.29
11c0006	0.22	10.57	um0039	0.38	10.60
11c0006	0.60	13.72	um0039	0.09	12.04
11c0006	-0.59	12.82	um0039	-0.21	8.66
11c0006	-0.49	12.65	um0039	1.36	11.71
11c0006	0.03	11.38	um0039	0.14	12.11
11c0006	-0.04	9.14	um0040	0.62	11.41
11c0006	-0.18	11.72	um0040	-0.17	14.90
11c0006	-0.25	12.13	um0040	0.15	11.58
11c0006	-0.03	10.28	um0040	1.52	13.62
11c0006	0.81	13.87	um0040	0.61	11.25
11c0007	-0.03	8.89	um0041	0.02	11.54
11c0007	0.61	14.70	um00411	0.95	9.77
11c0007	-0.14	13.44	um0041	0.97	11.32
11c0007	0.03	10.61	um0041	-0.05	10.96
11c0007	-0.28	14.12	um0041	-0.23	12.19
11c0007	0.16	9.13	um0041	-0.38	12.34
11c0007	-0.03	9.19	um0041	-0.39	11.69
11c0007	-0.73	13.65	um0041	0.20	10.19
11c0007	0.02	12.07	um0042	0.23	8.98
11c0007	0.56	14.27	um0042	0.21	12.85
11c0008	-0.10	11.01	um0042	-0.05	13.69
11c0008	0.32	12.57	um0042	-0.23	12.46
11c0008	-0.04	12.45	um0042	-0.19	10.77
11c0008	-0.84	12.85	um0042	2.02	11.13
11c0008	0.10	11.46	um0042	-0.25	12.27
11c0008	-0.10	13.32	um0043	-1.04	14.45
11c0008	-0.40	11.53	um0043	-0.10	13.88
11c0008	-0.12	13.29	um0043	0.34	11.76

11c0008	-0.73	11.13
11c0008	0.12	11.15
11c0009	-0.21	9.96
11d0000	-0.06	8.88
11d0000	0.35	10.66
11d0000	-0.38	13.55
11d0000	0.06	9.21
11d0000	-0.10	9.14
11d0000	-0.26	13.77
11d0000	-0.11	13.73
11d0000	-0.08	8.78
11d0000	0.50	11.39
11d0001	0.03	8.54
11d0001	0.19	11.82
11d0001	-0.02	9.32
11d0001	-0.14	12.90
11d0001	-0.13	13.81
11d0001	0.20	9.14
11d0001	0.13	10.91
11d0001	0.38	12.88
11d0001	0.04	8.65
11d0001	-0.08	11.79
11d0002	0.26	12.28
11d0002	-0.05	12.20
11d0002	0.50	12.86
11d0002	0.31	9.53
11d0002	0.22	12.53
11d0002	0.35	11.68
11d0002	0.44	9.26
11d0002	-0.01	9.63
11d0002	0.20	12.02
11d0002	0.24	11.06
11d0003	0.14	11.80
11d0003	0.40	11.47
11d0003	-0.03	11.52
11d0003	-1.61	13.88
11d0003	-0.34	12.78
11d0003	0.37	12.62
11d0003	-0.02	10.64
11d0003	0.12	12.58
11d0003	0.13	13.70
11d0003	0.66	12.72
11d0004	-0.09	11.81
11d0004	-0.11	12.36
11d0004	-0.01	9.35
11d0004	-0.15	12.72
11d0004	-0.32	12.63
11d0004	-0.41	11.21
11d0004	0.06	15.52
11d0004	0.02	11.61
11d0004	-0.03	8.34
11d0004	-0.03	15.60
11d0005	-0.07	11.40
11d0005	0.09	13.44

um00438	0.06	11.70
um00439	0.16	11.16
um00440	0.14	12.12
um00441	-1.52	9.81
um00442	-0.79	10.10
um00443	0.22	10.25
um00444	0.49	11.11
um00445	-0.20	8.71
um00446	0.09	11.39
um00447	0.41	10.82
um00448	0.84	10.76
um00449	-0.16	8.69
um00450	-0.15	11.82
um00451	0.53	12.55
um00453	0.23	11.29
um00454	-0.65	11.01
um00455	1.33	12.29
um00458	0.22	12.14
um00459	-0.64	10.51
um00460	0.31	11.82
um00461	-0.41	12.99
um00463	0.97	11.64
um00464	-0.37	11.88
um00465	-2.71	10.99
um00466	1.09	10.27
um00467	0.11	10.24
um00468	-0.68	11.69
um00469	0.12	12.76
um00473	0.44	12.35
um00475	0.45	12.99
um00476	-0.05	11.85
um00477	-0.01	9.10
um00478	-0.75	10.91
um00480	-0.33	12.38
um00481	0.58	12.99
um00482	0.43	11.21
um00483	-0.07	11.13
um00486	-1.15	14.47
um00490	0.72	11.85
um00491	0.20	11.62
um00492	-1.39	12.29
um00493	-0.05	11.32
um00494	-0.20	12.14
um00495	-0.77	12.81
um00496	3.67	13.02
um00499	0.64	11.86
um00501	0.15	11.62
um00503	0.36	14.03
um00505	-0.15	12.01
um00506	-0.47	12.02
um00507	0.43	10.93
um00508	-0.23	15.40
um00510	-0.63	11.69
um00512	0.73	13.38

11d0005	0.29	10.77	um00513	0.14	10.92
11d0005	-0.39	11.60	um00515	0.70	12.07
11d0005	0.20	13.25	um00516	0.09	10.70
11d0005	-0.10	11.13	um00518	-0.01	11.80
11d0005	0.65	12.34	um00522	-0.23	8.83
11d0005	-0.15	12.01	um00523	0.12	11.80
11d0005	0.11	12.56	um00525	-0.02	12.04
11d0005	-0.05	9.03	um00526	0.57	11.92
11d0006	-0.12	12.67	um00527	0.43	11.63
11d0006	0.82	13.22	um00528	0.28	10.96
11d0006	0.49	11.87	um00529	4.04	12.50
11d0006	0.60	12.03	um00530	0.92	10.23
11d0006	-0.26	11.18	um00532	-0.20	12.46
11d0006	-0.40	9.44	um00533	-0.35	11.68
11d0006	-0.02	12.06	um00534	-0.22	12.62
11d0006	0.00	12.54	um00535	0.69	11.36
11d0006	0.43	13.66	um00536	0.18	11.29
11d0006	-0.09	10.12	um00538	0.32	9.78
11d0007	0.13	13.43	um00539	0.15	11.56
11d0007	0.22	12.28	um00540	0.38	11.39
11d0007	-0.07	9.73	um00542	-0.22	11.48
11d0007	-0.17	11.93	um00543	0.31	12.69
11d0007	0.10	12.13	um00545	1.00	12.58
11d0007	0.82	12.77	um00547	-0.94	11.42
11d0007	-0.04	12.39	um00548	-0.69	11.98
11d0007	0.30	13.08	um00549	-0.48	11.14
11d0007	0.82	11.85	um00550	-0.44	9.41
11d0007	-0.02	8.78	um00551	-0.48	12.11
11d0008	-0.33	13.08	um00553	-0.14	11.64
11d0008	0.27	12.70	um00555	-0.90	12.64
11d0008	-0.01	13.12	um00557	-0.21	13.56
11d0008	-0.17	13.16	um00558	0.60	9.31
11d0008	0.24	10.84	um00559	0.00	8.69
11d0008	0.09	10.83	um00560	0.48	13.42
11d0008	-0.09	8.94	um00561	0.18	11.89
11d0008	-0.38	12.11	um00562	0.75	12.09
11d0008	-0.15	10.20	um00563	0.25	12.70
11d0008	0.24	12.05	um00564	-0.15	11.52
11d0009	-0.23	12.31	um00565	-0.53	12.56
12c0000	-0.86	9.31	um00567	-1.46	11.90
12c0000	-0.65	11.34	um00568	0.51	12.69
12c0000	-0.22	10.36	um00569	0.33	11.01
12c0000	-0.33	8.95	um00572	0.60	11.30
12c0000	1.70	11.97	um00573	1.87	14.29
12c0000	-0.13	12.82	um00574	0.01	10.97
12c0000	-0.06	11.94	um00575	0.28	11.30
12c0000	0.35	10.66	um00576	0.65	11.46
12c0000	-0.03	12.97	um00578	-0.33	8.91
12c0001	0.16	10.57	um00579	-0.26	12.26
12c0001	0.15	9.95	um00581	-0.35	11.48
12c0001	1.18	11.25	um00582	-0.24	12.60
12c0001	0.43	11.99	um00583	-0.93	13.57
12c0001	-0.84	12.26	um00584	-0.51	13.30
12c0001	-0.55	12.40	um00585	-0.30	12.72

12c0001	0.15	14.17
12c0001	-0.22	13.27
12c0001	-0.17	10.71
12c0001	0.59	14.37
12c0002	-0.31	13.12
12c0002	-0.59	13.48
12c0002	-0.24	12.58
12c0002	-0.34	11.85
12c0002	0.49	13.32
12c0002	-0.07	10.10
12c0002	0.23	10.12
12c0002	-0.40	12.46
12c0002	-0.02	12.69
12c0002	-0.15	11.36
12c0003	-0.12	9.45
12c0003	0.00	9.50
12c0003	-0.04	14.35
12c0003	-0.30	11.37
12c0003	-0.26	9.73
12c0003	0.04	9.16
12c0003	-0.67	13.34
12c0003	-0.46	10.35
12c0003	-0.44	11.63
12c0003	0.48	12.65
12c0004	-0.24	9.66
12c0004	-0.20	10.90
12c0004	0.44	12.39
12c0004	-0.64	12.48
12c0004	0.14	12.80
12c0004	0.44	12.60
12c0004	0.30	13.62
12c0004	-0.55	11.02
12c0004	0.86	11.02
12c0004	-0.08	10.54
12c0005	-0.20	10.08
12c0005	0.04	11.36
12c0005	0.31	11.56
12c0005	0.33	14.99
12c0005	-0.41	12.68
12c0005	-0.08	13.48
12c0005	-0.30	12.57
12c0005	-0.52	15.16
12c0005	-0.42	11.98
12c0005	0.51	12.56
12c0006	0.13	11.75
12c0006	-0.04	12.27
12c0006	0.43	12.60
12c0006	0.04	10.98
12c0006	-0.06	8.75
12c0006	-0.10	11.84
12c0006	-0.10	11.45
12c0006	-0.38	10.53
12c0006	0.20	12.32
12c0006	0.12	8.64

um00586	-0.17	9.89
um00588	0.22	12.41
um00589	0.67	12.69
um00590	-0.15	11.31
um00592	0.24	11.55
um00593	1.53	10.06
um00594	-1.02	13.55
um00595	-1.09	13.54
um00596	-0.04	10.25
um00597	-0.57	11.89
um00598	-0.12	11.82
um00599	0.61	11.45
um00601	0.56	10.82
um00602	-0.15	11.84
um00603	0.16	11.53
um00604	0.41	11.76
um00605	-0.37	11.68
um00606	-1.13	11.70
um00607	-0.17	11.88
um00608	-0.57	12.43
um00609	0.16	11.06
um00610	0.61	12.15
um00612	-0.15	12.27
um00614	0.07	12.31
um00615	0.15	11.64
um00616	-0.17	12.65
um00617	-0.24	11.99
um00620	-0.43	11.72
um00622	-0.46	13.15
um00623	1.11	11.28
um00624	0.19	12.13
um00625	0.21	11.53
um00627	0.01	8.63
um00628	-0.25	8.68
um00629	-0.17	8.70
um00630	-0.45	13.96
um00632	-0.06	11.07
um00634	0.85	12.92
um00635	-0.45	12.42
um00636	0.97	12.72
um00637	-0.50	13.56
um00638	-0.13	14.14
um00639	-0.30	11.62
um00644	-0.43	11.20
um00645	0.34	11.50
um00646	-0.41	13.68
um00647	-0.45	10.98
um00650	-0.18	10.56
um00651	0.12	11.09
um00652	-0.10	11.44
um00654	-0.09	11.65
um00655	-1.22	11.60
um00656	-0.03	11.57
um00657	0.17	12.10

12c0007	0.08	9.92	um00658	0.14	15.29
12c0007	0.07	8.97	um00660	-0.41	11.84
12c0007	0.29	13.14	um00663	-1.15	13.27
12c0007	-0.32	13.69	um00664	-1.55	11.67
12c0007	-0.06	10.96	um00666	0.25	11.94
12c0007	0.55	12.69	um00667	0.21	11.62
12c0007	-0.70	13.51	um00668	-0.09	11.72
12c0007	0.08	11.83	um00669	0.50	11.27
12c0007	0.51	11.84	um00670	0.45	11.67
12c0007	-0.19	9.61	um00671	-0.13	10.16
12c0008	-0.13	10.96	um00672	-1.05	13.35
12c0008	0.19	10.73	um00673	-0.63	14.14
12c0008	-0.09	13.11	um00674	-0.43	15.48
12c0008	0.36	13.59	um00675	-0.06	10.09
12c0008	0.01	8.84	um00676	-0.55	12.23
12c0008	-0.08	11.49	um00678	-0.20	11.01
12c0008	-0.13	8.59	um00679	-1.05	11.85
12c0008	-0.06	8.64	um00684	0.13	10.83
12c0008	-0.05	8.53	um00685	0.05	12.49
12c0008	0.41	11.72	um00687	0.79	11.96
12c0009	-0.46	13.09	um00688	-0.83	12.85
12c0009	0.04	13.05	um00689	0.51	11.04
12c0009	0.09	10.12	um00691	0.12	12.18
12c0009	-0.03	9.61	um00692	-0.51	14.44
12c0009	-1.25	13.45	um00693	-0.10	11.59
12c0009	-0.03	11.81	um00694	0.01	11.46
12c0009	0.34	9.88	um00695	-0.31	12.63
12c0009	-0.17	12.85	um00696	1.19	10.83
12c0009	0.17	13.22	um00698	-0.38	13.14
12c0009	-0.84	12.36	um00699	0.19	10.83
12c0010	-0.51	14.77	um00700	0.19	11.94
12c0010	-0.18	14.48	um00701	-0.13	8.75
12c0010	0.28	13.09	um00702	0.15	11.30
12c0010	0.14	12.17	um00703	0.16	13.18
12c0010	-0.17	11.46	um00706	-0.61	11.48
12c0010	0.16	12.91	um00708	1.61	11.76
12c0010	0.34	13.20	um00710	-0.55	12.19
12c0010	0.11	9.99	um00711	0.15	12.41
12c0010	1.30	14.10	um00712	0.71	11.29
12c0010	0.07	10.77	um00714	-0.16	10.56
12c0011	0.24	10.43	um00715	-0.14	9.11
12c0011	0.29	13.36	um00716	0.32	10.47
12c0011	0.78	11.70	um00719	1.74	11.11
12c0011	-0.49	11.18	um00721	-0.57	12.32
12c0011	-0.67	11.27	um00722	0.41	10.74
12c0011	0.60	13.79	um00723	2.00	12.98
12c0011	0.26	11.44	um00725	0.16	11.33
12c0011	0.10	10.64	um00727	-1.18	10.01
12c0011	-0.07	12.99	um00728	0.14	10.63
12c0011	0.12	12.65	um00729	-0.48	11.24
12c0012	-0.90	13.13	um00731	-0.34	8.69
12c0012	0.50	12.32	um00734	-0.22	12.25
12c0012	0.04	13.20	um00735	0.87	12.44
12c0012	-0.45	14.50	um00736	-0.86	12.20

12c0012	-0.04	12.30	um00737	-0.92	11.86
12c0012	0.36	12.82	um00738	-2.01	12.65
12c0012	-0.10	14.89	um00739	-0.48	10.70
12c0012	-0.11	14.54	um00740	0.09	10.47
12c0012	0.20	10.36	um00741	0.34	10.96
12c0012	0.06	11.78	um00742	0.32	10.98
12c0013	-0.08	8.71	um00743	0.49	10.79
12c0013	0.15	12.01	um00745	-0.17	11.78
12c0013	0.33	12.88	um00748	1.06	11.97
12c0013	0.51	13.31	um00749	-0.78	10.85
12c0013	-0.31	12.55	um00750	1.00	13.31
12c0013	0.10	8.89	um00751	-0.22	12.27
12c0013	-0.01	15.86	um00752	-0.21	8.50
12c0013	0.56	11.48	um00753	-0.38	15.85
12c0013	-0.07	11.87	um00754	1.02	11.83
12c0013	-0.65	12.22	um00755	0.68	12.62
12c0014	-0.35	12.34	um00756	-0.33	14.47
12c0014	0.40	12.54	um00757	-0.23	13.05
12c0014	-0.21	12.78	um00758	0.15	11.76
12c0014	-0.08	10.18	um00760	-0.46	11.09
12c0014	-0.42	9.11	um00762	-0.19	8.53
12c0014	-0.56	9.32	um00763	0.19	11.43
12c0014	-0.51	9.40	um00766	-0.50	10.89
12c0014	0.49	11.54	um00767	-0.21	11.19
12d0000	-0.93	12.20	um00768	-0.44	12.72
12d0000	0.55	10.56	um00769	0.17	10.84
12d0000	-0.97	14.56	um00770	-0.63	12.43
12d0000	-0.58	11.31	um00771	0.23	10.07
12d0000	-0.66	11.03	um00773	-0.72	11.56
12d0000	-0.60	10.52	um00774	0.06	14.00
12d0000	-0.28	9.35	um00775	-0.21	11.54
12d0000	-0.10	12.81	um00776	0.77	10.55
12d0000	-0.18	12.34	um00777	0.42	10.79
12d0001	-0.38	12.91	um00778	-0.14	14.08
12d0001	1.01	13.07	um00779	0.11	11.65
12d0001	0.80	13.82	um00780	0.61	10.83
12d0001	1.25	13.97	um00781	0.21	9.04
12d0001	0.14	14.13	um00782	-0.94	9.45
12d0001	-0.16	12.59	um00783	-0.95	13.02
12d0001	-1.12	13.02	um00784	-0.13	12.15
12d0001	0.00	13.77	um00785	0.58	10.44
12d0001	0.17	12.49	um00786	0.18	10.34
12d0001	0.09	10.15	um00788	-0.11	10.28
12d0002	-0.36	13.25	um00789	0.70	12.92
12d0002	-0.06	10.47	um00790	-0.68	12.84
12d0002	-0.08	10.47	um00792	-0.06	8.84
12d0002	-0.11	14.05	um00793	0.15	8.85
12d0002	0.16	9.82	um00794	-0.28	8.83
12d0002	0.30	11.86	um00795	-0.54	9.50
12d0002	-0.44	10.53	um00796	-0.55	11.35
12d0002	-0.09	11.61	um00797	-1.20	13.54
12d0002	-0.04	8.68	um00798	0.15	10.54
12d0002	-0.03	12.66	um00800	0.35	9.48
12d0003	-0.50	11.31	um00801	-0.29	12.38

12d0003	0.10	11.25	um00802	0.30	11.15
12d0003	0.26	13.64	um00805	0.54	10.93
12d0003	0.31	11.64	um00806	-0.26	11.19
12d0003	-0.27	14.15	um00808	0.09	10.80
12d0003	-0.94	13.77	um00810	-0.29	9.70
12d0003	0.21	12.59	um00811	0.45	12.17
12d0003	0.12	12.51	um00812	-0.43	12.30
12d0003	0.18	13.31	um00813	-1.60	11.74
12d0003	0.14	11.57	um00815	3.02	12.55
12d0004	0.56	13.12	um00816	-0.60	15.78
12d0004	-0.36	12.85	um00817	0.37	11.24
12d0004	0.00	9.86	um00820	-0.02	8.90
12d0004	0.08	11.34	um00821	-0.29	8.74
12d0004	-0.52	12.15	um00822	-0.10	8.40
12d0004	-0.19	13.37	um00823	0.06	12.71
12d0004	-0.02	10.54	um00824	2.65	12.85
12d0004	-0.14	9.86	um00825	1.58	11.24
12d0004	0.31	14.80	um00827	-0.60	10.28
12d0004	0.17	11.96	um00829	-0.31	15.70
12d0005	0.10	10.71	um00830	0.79	11.83
12d0005	-0.17	13.09	um00831	-0.32	15.76
12d0005	-0.20	10.30	um00832	-0.34	11.76
12d0005	0.13	11.14	um00833	0.53	13.80
12d0005	0.20	11.15	um00835	-0.18	10.78
12d0005	0.01	13.12	um00836	0.07	10.71
12d0005	-0.38	12.41	um00837	-0.09	9.41
12d0005	-0.03	12.19	um00840	0.41	10.47
12d0005	-0.09	12.46	um00841	2.47	11.63
12d0005	0.38	11.32	um00842	3.93	12.61
12d0006	0.02	12.10	um00844	2.17	13.12
12d0006	0.12	11.71	um00847	0.02	11.37
12d0006	0.08	9.31	um00848	0.31	12.49
12d0006	0.08	8.69	um00851	-0.16	13.26
12d0006	0.12	13.12	um00855	-0.31	12.83
12d0006	0.01	11.40	um00857	-0.08	11.78
12d0006	-0.53	11.21	um00858	0.01	8.80
12d0006	-0.35	11.31	um00859	1.02	11.56
12d0006	0.03	9.05	um00861	-0.28	11.84
12d0006	0.39	13.34	um00862	0.08	15.12
12d0007	0.50	10.72	um00864	-0.77	12.34
12d0007	0.01	11.00	um00866	-1.08	12.97
12d0007	-0.31	12.94	um00867	0.31	14.59
12d0007	-0.11	10.81	um00869	0.76	11.95
12d0007	-0.12	13.34	um00871	0.13	11.12
12d0007	0.43	13.75	um00872	-0.34	11.73
12d0007	-0.05	12.25	um00874	0.04	8.85
12d0007	-0.34	14.61	um00875	0.52	9.01
12d0007	0.14	14.74	um00876	-4.60	11.80
12d0007	-0.03	8.70	um00878	0.52	11.49
12d0008	0.10	12.15	um00879	0.10	11.19
12d0008	0.78	13.29	um00880	0.03	12.08
12d0008	0.72	14.55	um00882	-0.04	10.72
12d0008	-0.03	9.15	um00883	0.58	11.34
12d0008	-0.59	12.13	um00884	0.51	12.15

12d0008	0.17	9.82	um00885	-0.28	8.87
12d0008	-0.56	13.25	um00886	-0.62	10.63
12d0008	-0.30	12.50	um00887	-0.20	12.45
12d0008	-0.22	11.29	um00890	-0.25	12.25
12d0008	0.20	10.94	um00891	0.01	8.94
12d0009	0.24	12.26	um00893	0.41	12.27
12d0009	-0.23	9.29	um00894	0.20	10.89
12d0009	-0.54	11.57	um00895	-0.13	10.68
12d0009	-0.15	10.74	um00896	-0.63	9.48
12d0009	-0.20	9.24	um00897	-0.78	11.54
12d0009	-0.01	8.47	um00898	-0.38	12.21
12d0009	0.09	8.60	um00902	1.48	12.03
12d0009	0.57	12.32	um00903	0.36	10.80
12d0009	-0.13	8.64	um00904	-0.37	12.94
12d0009	-0.02	8.98	um00906	0.19	12.78
12d0010	-0.05	11.77	um00907	0.28	12.03
12d0010	0.04	9.82	um00908	-0.25	12.50
12d0010	0.17	9.60	um00909	0.01	11.58
12d0010	0.03	8.86	um00911	-0.24	10.62
12d0010	0.00	12.52	um00912	0.07	11.09
12d0010	-0.73	13.41	um00913	-1.50	13.28
12d0010	-0.24	9.98	um00915	0.05	12.13
12d0010	-0.01	8.91	um00916	0.55	11.49
12d0010	-0.47	10.73	um00919	-0.57	15.98
12d0010	-0.66	12.82	um00920	-0.65	11.03
12d0011	0.32	12.85	um00921	0.12	10.45
12d0011	-0.06	12.35	um00922	0.60	11.25
12d0011	0.31	12.66	um00923	-0.13	13.06
12d0011	-0.01	11.49	um00924	-0.61	15.95
12d0011	0.09	12.19	um00926	0.13	14.93
12d0011	0.43	11.95	um00927	0.10	10.61
12d0011	0.39	11.95	um00928	0.62	11.85
12d0011	0.15	9.03	um00929	0.43	12.36
12d0011	-0.19	10.59	um00930	0.22	11.25
12d0011	-0.03	12.83	um00931	0.16	10.29
12d0012	-0.16	11.42	um00933	-0.51	12.79
12d0012	0.22	12.27	um00934	-1.29	14.52
12d0012	0.07	12.68	um00935	0.80	12.73
12d0012	-0.09	8.99	um00936	0.03	12.85
12d0012	0.03	8.62	um00937	0.21	12.08
12d0012	0.42	11.77	um00940	0.22	14.07
12d0012	-0.13	12.23	um00945	-1.25	13.67
12d0012	0.11	11.61	um00946	1.03	13.82
12d0012	-0.02	8.93	um00947	0.20	12.26
12d0012	-0.04	9.06	um00948	0.31	11.13
12d0013	-0.24	10.40	um00949	0.43	12.69
12d0013	-0.15	14.15	um00950	-0.09	11.84
12d0013	0.01	9.54	um00952	0.18	11.81
12d0013	-0.73	14.04	um00955	-1.91	14.10
12d0013	-0.53	12.85	um00956	0.10	12.00
12d0013	0.59	12.01	um00957	0.11	12.23
12d0013	0.14	11.84	um00958	0.53	11.74
12d0013	-0.10	10.05	um00961	-0.61	15.59
12d0013	-0.45	12.45	um00963	0.55	10.73

12d0013	-0.16	11.40	um00964	0.08	11.17
12d0014	0.06	8.80	um00965	0.62	11.30
12d0014	-0.33	11.09	um00966	0.32	10.93
13c0000	-0.13	8.57	um00967	-0.56	15.80
13c0000	-0.24	12.19	um00968	2.94	11.04
13c0000	0.17	12.97	um00970	1.64	12.05
13c0000	-0.70	12.89	um00971	0.49	11.43
13c0000	0.29	10.34	um00975	-0.28	14.24
13c0000	0.09	9.32	um00978	0.11	12.04
13c0000	0.07	11.09	um00980	-0.29	13.33
13c0000	0.37	10.24	um00981	0.09	11.18
13c0000	-0.04	12.08	um00982	-0.45	15.11
13c0001	-0.10	8.88	um00983	-0.23	11.28
13c0001	0.05	11.85	um00984	-0.71	12.37
13c0001	-0.14	11.97	um00985	-1.00	11.43
13c0001	-0.29	11.67	um00986	0.14	13.69
13c0001	0.48	12.50	um00987	-0.05	12.38
13c0001	0.41	12.90	um00988	0.25	11.75
13c0001	0.05	10.63	um00989	0.50	11.36
13c0001	0.30	12.06	um00990	-0.22	11.44
13c0001	0.52	12.52	um00991	-0.17	12.60
13c0001	0.44	11.05	um00992	1.19	13.58
13c0002	-3.02	12.24	um00995	-0.39	11.15
13c0002	-0.26	11.52	um00996	-0.31	12.07
13c0002	-0.66	13.02	um00997	0.09	12.07
13c0002	0.38	13.00	um00998	0.93	11.19
13c0002	0.11	10.87	um01001	-0.01	10.65
13c0002	0.63	14.34	um01004	-0.53	11.29
13c0002	-0.44	15.67	um01005	-1.34	14.60
13c0002	-0.95	11.72	um01006	-0.26	13.84
13c0002	0.02	11.27	um01008	0.11	10.36
13c0002	-0.15	12.07	um01009	0.16	13.03
13c0003	0.09	11.52	um01010	-0.26	11.75
13c0003	-0.01	13.03	um01011	-0.38	10.87
13c0003	0.12	12.51	um01012	-0.53	11.52
13c0003	-0.13	10.34	um01013	-0.02	12.33
13c0003	0.28	14.38	um01014	-0.81	14.25
13c0003	0.55	14.37	um01016	0.64	10.12
13c0003	0.01	11.84	um01017	-0.19	11.69
13c0003	-1.24	15.26	um01018	0.07	15.04
13c0003	-0.54	10.95	um01021	-0.15	11.57
13c0003	0.32	13.05	um01022	-0.27	13.35
13c0004	0.25	10.64	um01023	-0.39	12.59
13c0004	-0.17	13.43	um01024	-0.26	8.63
13c0004	-0.43	11.32	um01025	-1.32	10.05
13c0004	-0.73	12.42	um01026	0.27	11.83
13c0004	-0.24	11.75	um01027	0.29	11.18
13c0004	-0.20	10.20	um01030	0.46	11.74
13c0004	-0.13	8.50	um01031	-0.15	11.99
13c0004	-1.41	13.53	um01032	-0.10	12.67
13c0004	-0.43	11.64	um01033	-0.44	11.81
13c0004	-0.10	12.39	um01037	0.74	12.40
13c0005	0.01	12.15	um01038	0.53	12.33
13c0005	0.04	13.69	um01040	0.07	13.18

13c0005	0.35	13.37	um01041	0.01	8.72
13c0005	0.17	13.09	um01042	-0.05	11.23
13c0005	0.70	13.06	um01045	-0.15	12.23
13c0005	0.22	15.16	um01047	-0.12	8.78
13c0005	0.45	12.92	um01048	-0.66	12.66
13c0005	0.29	11.43	um01049	-0.02	14.01
13c0005	0.61	14.12	um01050	-0.59	11.32
13c0005	-0.02	12.66	um01051	-0.78	11.56
13c0006	0.36	14.41	um01052	-5.14	13.15
13c0006	0.01	10.88	um01053	-0.15	8.45
13c0006	0.39	13.15	um01054	-0.76	14.16
13c0006	0.12	11.49	um01055	0.08	13.66
13c0006	0.32	10.21	um01058	0.18	11.52
13c0006	0.43	12.07	um01059	-0.18	11.88
13c0006	0.04	11.01	um01060	0.03	15.13
13c0006	0.55	12.06	um01061	-0.03	11.38
13c0006	0.10	14.43	um01062	-0.68	12.82
13c0006	0.00	11.99	um01063	0.24	14.63
13c0007	-0.71	10.97	um01064	-0.06	11.62
13c0007	0.14	12.06	um01065	1.06	10.87
13c0007	-0.26	12.47	um01067	0.09	12.27
13c0007	0.34	9.08	um01070	-1.05	13.57
13c0007	0.27	11.99	um01072	0.19	12.27
13c0007	-0.28	12.70	um01073	-0.45	9.64
13c0007	0.16	10.09	um01074	0.33	11.17
13c0007	-0.13	8.59	um01075	-0.40	11.62
13c0007	-1.05	11.67	um01076	0.25	11.65
13c0007	-0.54	14.15	um01077	0.20	13.94
13c0008	0.28	13.34	um01079	0.05	9.43
13c0008	0.26	12.99	um01080	0.03	11.64
13c0008	0.55	11.07	um01081	0.06	11.64
13c0008	-1.18	12.64	um01082	1.32	13.77
13c0008	-0.68	13.22	um01083	0.20	10.69
13c0008	-0.47	10.19	um01084	0.29	12.45
13c0008	0.12	10.82	um01085	0.83	11.31
13c0008	0.19	10.71	um01087	0.16	12.50
13c0008	0.41	13.27	um01088	-0.71	11.94
13c0008	0.24	11.50	um01089	-0.14	11.26
13c0009	0.14	9.66	um01090	1.52	13.43
13c0009	-0.05	12.65	um01091	0.39	10.86
13c0009	-0.10	8.61	um01092	0.33	10.43
13c0009	-0.33	8.86	um01093	0.32	12.40
13c0009	-0.22	11.97	um01094	-0.12	12.28
13c0009	-1.16	14.62	um01096	-0.01	13.20
13c0009	0.61	12.89	um01099	-0.28	13.73
13c0009	-0.39	12.38	um01100	0.48	10.99
13c0009	-0.29	13.08	um01101	0.37	12.04
13c0009	0.18	11.53	um01102	0.30	10.79
13c0010	-0.32	12.49	um01103	-0.30	14.99
13c0010	-0.28	12.34	um01104	0.11	11.06
13c0010	-0.15	13.70	um01105	-0.30	12.98
13c0010	-0.38	10.11	um01107	0.10	11.54
13c0010	-0.02	12.04	um01110	-0.78	11.83
13c0010	0.05	12.01	um01111	-0.44	10.73

13c0010	-0.13	9.68	um01112	0.80	10.57
13c0010	0.31	10.74	um01113	0.10	10.72
13c0010	0.13	9.73	um01114	0.68	13.26
13c0010	-0.15	12.47	um01115	0.62	11.86
13c0011	0.10	9.80	um01116	-0.40	12.49
13c0011	-0.11	15.27	um01117	0.26	11.79
13d0000	-2.11	12.21	um01118	-0.27	8.40
13d0000	0.14	11.49	um01120	0.16	10.30
13d0000	-0.86	12.12	um01121	0.22	13.01
13d0000	-0.59	12.22	um01122	0.73	11.86
13d0000	0.06	10.36	um01123	-0.15	11.61
13d0000	-0.53	11.23	um01125	-0.10	8.40
13d0000	0.09	13.86	um01126	-0.17	11.56
13d0000	0.09	12.87	um01128	0.53	10.79
13d0000	-0.23	12.45	um01129	-0.19	11.22
13d0001	-0.44	12.08	um01130	-0.83	9.57
13d0001	-0.19	11.95	um01131	0.33	11.74
13d0001	-0.03	8.70	um01132	-0.27	8.64
13d0001	0.01	8.67	um01133	-0.11	11.02
13d0001	-0.84	12.74	um01134	0.96	11.60
13d0001	0.42	12.99	um01135	0.98	10.27
13d0001	0.41	12.26	um01136	-1.00	11.65
13d0001	-0.22	11.94	um01138	-0.44	10.31
13d0001	-0.06	13.37	um01139	0.48	11.27
13d0001	0.06	10.20	um01141	-0.77	11.84
13d0002	-0.54	12.45	um01142	-0.78	12.08
13d0002	0.23	12.67	um01143	-1.01	15.60
13d0002	0.02	12.64	um01144	0.53	11.05
13d0002	0.40	11.26	um01145	-0.15	8.75
13d0002	0.29	12.73	um01146	0.05	12.22
13d0002	0.33	9.90	um01148	0.04	11.68
13d0002	0.38	13.19	um01149	-0.31	13.09
13d0002	0.30	12.42	um01150	-0.13	15.18
13d0002	0.16	10.48	um01152	0.24	10.86
13d0002	-0.22	13.39	um01153	-0.23	10.85
13d0003	-0.28	13.08	um01154	-0.11	13.28
13d0003	0.54	13.45	um01156	0.24	12.19
13d0003	0.64	13.99	um01159	0.22	12.01
13d0003	-0.24	12.10	um01161	0.19	12.67
13d0003	-0.30	11.20	um01162	-0.45	11.74
13d0003	0.53	12.35	um01163	-0.96	11.96
13d0003	-0.59	12.62	um01164	0.92	10.70
13d0003	-0.02	11.70	um01165	-3.03	11.15
13d0003	-0.29	10.39	um01166	-0.24	11.36
13d0003	0.03	11.93	um01167	-0.02	11.43
13d0004	0.51	13.61	um01168	-0.49	12.01
13d0004	0.53	11.76	um01169	-0.25	12.73
13d0004	0.10	12.25	um01170	-0.37	11.08
13d0004	-0.51	11.94	um01171	1.92	13.44
13d0004	0.39	10.67	um01172	0.91	13.15
13d0004	0.10	8.52	um01173	0.49	11.10
13d0004	-0.59	13.66	um01174	0.31	11.70
13d0004	-0.03	11.89	um01175	0.16	11.22
13d0004	0.43	12.45	um01176	-0.03	11.98

13d0004	0.05	12.18	um01177	0.07	11.55
13d0005	0.02	11.03	um01178	-0.07	11.95
13d0005	-0.16	12.19	um01179	0.92	11.71
13d0005	-0.49	11.23	um01180	0.15	12.54
13d0005	-0.20	14.86	um01181	0.32	12.50
13d0005	0.24	13.53	um01182	2.21	12.26
13d0005	0.05	11.33	um01184	0.56	12.70
13d0005	0.24	14.95	um01185	0.36	12.11
13d0005	-0.17	10.54	um01186	-0.50	13.00
13d0005	0.09	11.59	um01187	-4.47	11.73
13d0005	0.41	12.43	um01189	-0.57	15.76
13d0006	0.15	12.39	um01190	0.47	11.07
13d0006	-1.05	14.32	um01191	0.57	11.70
13d0006	0.53	11.99	um01192	1.55	11.93
13d0006	0.55	12.76	um01194	-0.69	12.96
13d0006	-0.31	11.96	um01195	-0.38	12.28
13d0006	0.16	10.95	um01196	0.24	12.02
13d0006	-0.06	10.16	um01197	0.14	11.58
13d0006	0.06	10.84	um01198	-0.41	10.98
13d0006	0.00	12.66	um01200	0.42	10.26
13d0006	0.18	13.45	um01201	0.08	10.46
13d0007	0.18	12.89	um01202	0.02	10.67
13d0007	0.03	8.69	um01204	1.22	9.49
13d0007	-0.98	13.45	um01205	-0.93	14.19
13d0007	0.64	11.50	um01206	-0.36	11.24
13d0007	0.25	11.90	um01207	0.45	12.45
13d0007	0.03	13.15	um01208	0.25	13.19
13d0007	-0.01	11.90	um01209	-0.80	13.25
13d0007	-0.02	11.46	um01210	0.22	11.86
13d0007	0.14	11.95	um01211	-0.42	10.97
13d0007	-0.14	14.37	um01212	1.73	12.69
13d0008	-0.05	8.98	um01213	-1.74	12.32
13d0008	0.76	12.16	um01214	-0.42	9.02
13d0008	-0.01	11.25	um01215	0.15	11.65
13d0008	-0.09	8.63	um01216	0.37	12.10
13d0008	-0.14	8.58	um01217	-0.24	8.53
13d0008	-0.09	8.66	um01219	0.04	10.95
13d0008	-0.40	11.56	um01220	-0.46	13.30
13d0008	-1.34	12.00	um01221	-0.47	15.88
13d0008	0.28	12.18	um01222	0.24	11.47
13d0008	-0.56	11.80	um01224	0.99	12.16
13d0009	0.21	13.96	um01226	-0.16	8.74
13d0009	-0.83	11.86	um01227	0.08	11.41
13d0009	0.53	12.80	um01228	0.53	12.48
13d0009	0.54	15.15	um01229	-0.25	11.83
13d0009	-0.08	14.10	um01230	-0.29	11.39
13d0009	-0.36	10.94	um01231	-0.34	12.11
13d0009	-1.54	11.94	um01232	-1.72	13.28
13d0009	0.54	12.38	um01233	-1.02	13.44
13d0009	0.67	11.34	um01234	-0.19	8.83
13d0009	-2.45	13.20	um01235	-0.26	8.59
13d0010	0.20	11.10	um01236	-0.13	8.70
13d0010	0.17	12.35	um01237	-0.60	9.08
13d0010	-0.49	13.03	um01238	-0.34	8.47

13d0010	0.34	13.60	um01239	-0.09	8.61
13d0010	-0.18	12.60	um01240	-0.25	8.62
13d0010	0.11	10.77	um01241	-0.31	8.57
13d0010	-0.14	9.62	um01242	0.07	11.81
13d0010	-0.01	14.11	um01243	0.61	11.87
13d0010	0.03	9.16	um01244	0.13	11.94
13d0010	0.24	11.07	um01245	-0.31	13.89
13d0011	0.48	13.25	um01246	0.38	12.23
13d0011	-0.32	13.64	um01249	-0.09	12.37
14c0000	-0.80	11.27	um01250	0.83	12.97
14c0000	0.71	10.46	um01251	0.50	12.06
14c0000	-0.42	9.12	um01252	-0.24	11.72
14c0000	-1.10	9.81	um01257	0.54	13.54
14c0000	-1.20	13.02	um01258	-0.84	11.22
14c0000	-0.12	10.88	um01262	-0.16	9.40
14c0000	0.42	11.35	um01264	-0.45	10.72
14c0000	0.07	10.46	um01265	0.68	11.13
14c0000	-0.16	13.47	um01266	0.00	12.13
14c0001	0.24	14.41	um01267	0.30	11.89
14c0001	-0.02	13.43	um01269	0.24	14.27
14c0001	-0.41	11.92	um01270	0.04	11.87
14c0001	-0.35	10.61	um01271	-0.05	10.36
14c0001	0.10	13.34	um01273	0.22	11.11
14c0001	0.52	11.73	um01274	-0.24	11.09
14c0001	0.06	9.65	um01275	-0.16	12.99
14c0001	-0.09	12.73	um01278	0.21	11.69
14c0001	0.31	12.51	um01279	-0.51	12.36
14c0001	0.03	11.48	um01281	-0.33	13.65
14c0002	-0.15	13.57	um01282	-0.62	11.60
14c0002	-0.19	12.32	um01284	0.29	12.53
14c0002	-0.28	12.54	um01285	-0.14	11.74
14c0002	0.08	12.71	um01286	0.05	13.08
14c0002	0.06	11.21	um01287	-0.35	8.67
14c0002	-0.29	11.79	um01289	-0.97	9.16
14c0002	0.04	12.02	um01292	1.06	12.08
14c0002	-0.22	11.00	um01293	0.82	11.83
14c0002	-0.08	10.24	um01295	0.49	11.44
14c0002	-0.24	13.51	um01296	0.23	10.35
14c0003	0.23	12.28	um01297	-0.10	8.61
14c0003	0.42	13.26	um01298	-0.23	8.49
14c0003	-0.58	11.12	um01299	-0.09	8.51
14c0003	0.26	12.94	um01300	-0.28	8.66
14c0003	0.37	13.80	um01301	-0.10	8.53
14c0003	-1.72	11.91	um01302	-0.02	8.64
14c0003	0.09	13.01	um01304	0.38	10.45
14c0003	-0.48	11.38	um01305	0.42	10.31
14c0003	-0.56	13.51	um01306	0.65	11.68
14c0003	-0.08	12.12	um01307	0.06	11.18
14c0004	0.21	9.13	um01308	0.01	10.86
14c0004	0.99	14.21	um01309	0.41	11.42
14c0004	0.26	10.21	um01310	0.26	11.34
14c0004	-0.37	12.45	um01311	0.64	14.21
14c0004	-1.46	10.62	um01312	0.50	11.57
14c0004	0.36	14.48	um01313	-0.07	11.33

14c0004	-0.36	11.91
14c0004	-0.33	12.24
14c0004	0.10	11.92
14c0004	-0.34	13.48
14c0005	-0.01	11.47
14c0005	-1.23	12.97
14c0005	0.01	13.63
14c0005	-0.06	9.65
14c0005	-0.29	11.14
14c0005	0.48	12.62
14c0005	0.06	9.32
14c0005	-0.06	11.70
14c0005	0.04	9.14
14c0005	0.30	10.60
14c0006	-0.19	11.02
14c0006	-0.10	11.01
14c0006	-0.80	11.49
14c0006	-0.12	13.03
14c0006	0.22	12.90
14c0006	0.12	12.98
14c0006	-0.21	14.02
14c0006	0.71	13.32
14c0006	-0.08	11.33
14c0006	-0.68	11.71
14c0007	-0.30	12.81
14c0007	0.14	11.79
14c0007	0.18	9.84
14c0007	-0.63	12.96
14c0007	0.22	11.34
14c0007	0.15	11.06
14c0007	-0.14	12.73
14c0007	0.18	13.67
14c0007	-0.13	10.26
14c0007	-0.01	11.65
14c0008	-0.05	12.08
14c0008	0.07	8.68
14c0008	0.01	8.73
14c0008	-0.07	8.43
14c0008	-0.37	13.98
14c0008	-0.09	10.51
14c0008	-0.05	11.90
14c0008	0.17	8.67
14c0008	0.05	9.54
14c0008	0.05	11.32
14c0009	0.42	11.99
14c0009	0.22	13.19
14c0009	0.22	11.47
14c0009	-0.21	13.39
14c0009	-0.04	9.75
14c0009	0.37	13.39
14c0009	-0.03	12.54
14c0009	-0.51	10.63
14c0009	0.10	12.11
14c0009	-0.15	9.16

um0131	-0.20	11.85
um0131	-0.26	13.22
um0131	-0.22	15.02
um0132	0.24	12.58
um0132	0.05	12.10
um0132	0.32	11.24
um0132	-0.43	11.29
um0132	-0.54	13.51
um0132	-0.51	13.75
um0133	0.61	9.94
um0133	-0.72	11.23
um0133	0.70	11.35
um0133	0.06	11.50
um0133	0.51	10.45
um0133	1.44	12.90
um0133	0.00	11.08
um0134	0.33	12.20
um0134	-0.31	13.50
um0134	0.05	11.73
um0134	0.60	11.29
um0135	-0.04	12.16
um0135	2.57	11.49
um0135	-0.05	13.52
um0135	0.17	12.26
um0135	-0.17	11.94
um0136	-0.22	8.64
um0136	-0.10	9.10
um0136	-2.25	10.06
um0136	0.23	11.05
um0136	-0.58	14.42
um0136	-0.45	15.84
um0136	0.32	12.58
um0137	-0.26	12.53
um0137	0.79	12.43
um0137	-0.08	10.73
um0137	0.05	11.02
um0137	0.35	9.75
um0137	-0.21	8.73
um0137	-0.22	8.66
um0137	-1.15	11.49
um0137	0.89	12.25
um0138	0.32	12.17
um0138	1.02	12.65
um0138	-0.08	11.18
um0138	0.08	8.73
um0138	0.29	12.68
um0138	-0.02	11.64
um0138	0.29	8.88
um0139	0.79	12.64
um0139	0.49	12.04
um0139	-0.05	8.54
um0139	1.66	13.30
um0139	0.92	10.89
um0140	-0.45	12.53

14c0010	-0.86	10.93	um01403	0.65	11.23
14c0010	0.09	10.34	um01405	-0.42	11.64
14c0010	-0.04	11.13	um01406	-2.20	12.62
14c0010	-0.72	12.16	um01407	-0.61	13.26
14c0010	-1.08	10.65	um01408	-0.21	12.76
14c0010	-0.15	11.95	um01411	1.28	12.41
14c0010	0.47	12.59	um01414	0.81	12.99
14c0010	-0.15	8.98	um01415	0.28	11.63
14c0010	-0.57	10.97	um01417	0.04	11.82
14c0010	0.05	15.89	um01418	0.41	11.47
14c0011	0.02	12.12	um01421	0.98	11.81
14c0011	-0.57	11.33	um01422	1.58	14.28
14c0011	-0.57	11.78	um01424	1.64	9.96
14c0011	-0.29	10.54	um01425	1.00	9.72
14c0011	-0.27	11.75	um01426	0.04	9.05
14c0011	0.27	13.56	um01427	-0.13	8.58
14c0011	0.63	12.88	um01428	-0.81	11.56
14c0011	-0.19	13.44	um01429	-0.89	12.26
14c0011	0.41	12.36	um01431	-1.14	14.33
14c0011	0.35	13.13	um01432	-1.43	14.80
14c0012	0.00	12.46	um01433	-0.91	14.84
14c0012	-0.01	11.02	um01434	-1.67	12.62
14c0012	0.20	13.77	um01435	0.40	9.47
14c0012	0.58	10.81	um01436	-0.91	11.13
14c0012	-0.02	8.84	um01437	-0.28	8.57
14c0012	-0.19	11.41	um01438	-0.31	8.55
14c0012	0.23	10.39	um01439	0.34	10.85
14c0012	-0.53	13.30	um01441	0.69	11.83
14c0012	0.16	12.55	um01442	-0.24	15.63
14c0012	0.20	9.46	um01443	-0.31	10.09
14c0013	0.06	11.21	um01445	-0.52	10.88
14c0013	0.53	13.93	um01450	-0.88	11.54
14c0013	0.01	8.83	um01454	-0.49	10.51
14c0013	0.56	10.68	um01455	-0.23	9.03
14c0013	-0.16	11.29	um01456	-0.18	8.98
14c0013	-0.15	12.87	um01458	-0.65	10.90
14c0013	-0.21	12.51	um01462	0.83	11.46
14c0013	-0.11	8.50	um01463	-0.05	13.11
14d0000	0.09	8.72	um01464	-0.04	11.13
14d0000	-0.06	8.84	um01465	0.11	9.20
14d0000	0.05	11.59	um01466	2.57	14.07
14d0000	-0.20	8.98	um01467	0.14	11.22
14d0000	-0.07	9.71	um01469	0.37	11.12
14d0000	-0.20	11.82	um01470	-0.26	10.80
14d0000	0.39	12.25	um01475	0.04	10.32
14d0000	-0.06	8.84	um01476	-1.74	10.41
14d0000	0.31	10.56	um01478	0.52	14.04
14d0001	-0.40	11.99	um01479	0.12	11.93
14d0001	-0.51	12.56	um01481	-0.05	9.39
14d0001	0.32	11.75	um01482	0.52	11.91
14d0001	-0.07	12.30	um01495	-0.13	12.64
14d0001	-0.03	12.77	um01498	-0.53	13.35
14d0001	0.25	15.55	um01499	-0.01	12.05
14d0001	-0.52	12.22	um01501	0.16	11.92

14d0001	-0.05	9.19	um01503	-0.07	11.89
14d0001	0.01	12.36	um01504	-0.07	14.02
14d0001	-0.54	15.29	um01505	-0.24	14.21
14d0002	0.04	14.01	um01507	-0.84	11.73
14d0002	0.24	13.48	um01508	-1.94	12.06
14d0002	0.27	11.55	um01510	-0.41	10.95
14d0002	0.16	12.80	um01511	0.07	10.83
14d0002	0.17	13.55	um01512	0.01	11.52
14d0002	0.29	12.64	um01513	-1.13	14.46
14d0002	-0.05	13.79	um01514	0.05	12.90
14d0002	0.19	11.19	um01515	-0.91	12.26
14d0002	-0.05	11.25	um01518	-0.47	12.88
14d0002	0.25	11.70	um01519	0.05	12.73
14d0003	0.42	12.94	um01521	-0.50	12.52
14d0003	0.15	9.97	um01522	-0.08	8.44
14d0003	0.06	8.94	um01523	-0.42	8.72
14d0003	-0.63	11.65	um01524	-0.21	9.17
14d0003	-0.37	13.29	um01526	0.29	11.43
14d0003	-0.37	11.04	um01527	-0.46	9.40
14d0003	-0.76	12.24	um01528	0.22	9.17
14d0003	0.04	11.60	um01529	0.04	11.86
14d0003	0.05	13.53	um01530	0.03	13.10
14d0003	-0.62	12.23	um01531	-0.12	10.93
14d0004	0.22	12.67	um01532	0.68	11.11
14d0004	-0.11	13.20	um01533	-0.65	11.90
14d0004	0.23	11.77	um01534	0.26	10.81
14d0004	-0.43	11.80	um01535	-0.09	12.35
14d0004	-0.06	8.54	um01539	0.09	11.04
14d0004	-0.72	12.32	um01540	-0.41	11.52
14d0004	-0.37	12.75	um01544	0.03	12.51
14d0004	-0.53	12.69	um01547	-0.63	14.95
14d0004	-0.06	11.41	um01549	0.46	11.55
14d0004	-0.04	8.76	um01550	0.33	12.30
14d0005	-0.44	13.68	um01551	-0.08	11.26
14d0005	0.41	11.45	um01552	-0.31	11.61
14d0005	-1.31	12.69	um01553	-0.16	13.30
14d0005	0.07	11.55	um01554	-0.19	11.51
14d0005	0.03	12.05	um01556	0.63	10.63
14d0005	0.37	12.35	um01558	0.71	11.12
14d0005	0.04	12.45	um01559	0.15	11.52
14d0005	-0.39	11.46	um01560	-0.74	11.73
14d0005	0.17	9.39	um01561	0.63	12.12
14d0005	1.31	15.00	um01564	-0.25	12.25
14d0006	0.93	11.85	um01565	-0.21	11.96
14d0006	-0.84	11.99	um01566	0.08	10.88
14d0006	0.18	11.89	um01567	-1.24	11.79
14d0006	-0.11	11.19	um01568	0.44	12.40
14d0006	-0.40	12.23	um01569	-0.15	10.98
14d0006	0.30	10.96	um01573	0.26	11.23
14d0006	-0.28	12.44	um01574	1.00	12.37
14d0006	0.46	13.61	um01576	0.52	11.34
14d0006	0.19	12.99	um01577	-0.18	12.11
14d0006	0.04	13.63	um01578	0.50	10.82
14d0007	0.22	13.79	um01580	-0.06	11.75

14d0007	-0.05	12.50	um01582	2.32	12.67
14d0007	-0.09	12.54	um01583	1.78	11.99
14d0007	0.08	11.90	um01584	0.94	9.35
14d0007	0.08	11.23	um01585	1.70	12.06
14d0007	-0.48	11.58	um01587	0.24	9.18
14d0007	-0.18	12.85	um01588	2.28	12.76
14d0007	0.05	8.73	um01589	0.50	8.99
14d0007	-0.04	8.89	um01591	1.74	11.93
14d0007	1.09	13.20	um01595	0.01	11.23
14d0008	-0.73	13.29	um01597	0.75	11.25
14d0008	-0.25	14.31	um01599	1.77	11.75
14d0008	-0.80	12.48	um01604	-0.24	10.52
14d0008	-0.08	9.27	um01605	0.59	10.98
14d0008	0.22	12.41	um01606	-0.36	12.31
14d0008	-0.19	12.58	um01607	0.72	13.27
14d0008	-0.63	11.87	um01608	0.06	12.02
14d0008	0.16	13.27	um01610	0.08	13.21
14d0008	0.12	13.90	um01612	0.25	12.09
14d0008	-0.02	8.57	um01613	0.03	11.85
14d0009	-0.06	14.92	um01614	2.55	12.54
14d0009	-0.27	13.12	um01615	0.16	11.56
14d0009	-0.03	13.52	um01616	0.18	11.61
14d0009	-0.35	13.45	um01617	0.17	11.42
14d0009	-1.07	9.81	um01619	-0.03	11.91
14d0009	0.11	10.77	um01621	0.01	12.20
14d0009	-0.35	12.42	um01623	0.70	11.85
14d0009	-0.11	12.39	um01624	-0.18	13.73
14d0009	-0.53	12.29	um01626	-1.02	10.94
14d0009	-0.52	12.48	um01627	-0.88	14.93
14d0010	-0.01	8.40	um01628	0.23	11.54
14d0010	0.00	12.85	um01632	0.56	14.06
14d0010	0.13	14.52	um01634	0.34	14.89
14d0010	-0.40	12.07	um01637	-0.48	13.45
14d0010	-0.41	12.05	um01638	-0.13	10.84
14d0010	0.01	8.83	um01639	-0.47	12.98
14d0010	1.02	14.45	um01640	-1.03	12.28
14d0010	0.25	12.47	um01641	0.05	11.81
14d0010	-0.26	12.89	um01643	0.71	14.06
14d0010	-0.11	10.95	um01645	-0.14	11.73
14d0011	0.65	11.22	um01646	-0.43	10.58
14d0011	0.26	13.24	um01647	-1.31	10.32
14d0011	0.09	11.55	um01648	-0.01	11.03
14d0011	-0.27	10.69	um01649	-0.20	8.69
14d0011	0.34	14.31	um01650	-1.19	10.73
14d0011	-1.91	10.20	um01651	0.26	14.19
14d0011	-0.12	12.63	um01652	-0.13	11.39
14d0011	0.06	11.96	um01653	-0.44	12.20
14d0011	-0.21	15.11	um01654	-0.15	12.79
14d0011	0.84	12.51	um01656	0.84	14.98
14d0012	-0.14	10.65	um01657	-0.04	12.08
14d0012	-0.58	11.71	um01658	0.09	11.44
14d0012	-0.01	11.88	um01659	0.01	10.26
14d0012	-0.19	9.15	um01660	0.33	11.95
156c000	-0.02	13.08	um01661	-0.02	9.14

15c0000	1.56	9.48	um01662	-0.43	11.76
15c0000	1.92	10.05	um01663	-0.60	10.98
15c0000	0.76	11.23	um01664	0.75	12.98
15c0000	-0.13	10.36	um01665	1.13	13.42
15c0000	-0.35	12.13	um01667	0.41	13.13
15c0000	0.31	12.19	um01668	0.35	11.18
15c0000	0.27	11.33	um01669	-0.12	11.84
15c0000	0.22	11.77	um01670	-0.31	12.00
15c0000	-0.43	15.33	um01671	0.71	11.42
15c0001	0.40	11.99	um01672	-0.54	14.56
15c0001	0.03	15.59	um01673	-0.09	11.89
15c0001	0.35	12.59	um01677	-0.41	9.47
15c0001	0.34	11.36	um01679	-0.58	10.90
15c0001	-0.50	9.39	um01680	0.13	11.83
15c0001	0.34	12.65	um01682	0.47	11.08
15c0001	0.36	12.89	um01683	0.12	12.53
15c0001	-0.55	12.34	um01684	0.32	9.29
15c0001	0.05	13.42	um01686	-0.57	12.84
15c0001	-0.66	14.16	um01687	0.08	12.06
15c0002	-0.24	12.92	um01688	0.82	11.16
15c0002	0.06	12.13	um01689	-0.15	8.73
15c0002	-0.12	9.29	um01690	-0.06	9.06
15c0002	0.23	11.82	um01691	0.20	10.10
15c0002	0.17	11.85	um01692	-0.35	12.32
15c0002	-0.07	12.86	um01694	0.23	9.66
15c0002	0.08	13.30	um01695	0.00	8.78
15c0002	-0.24	12.44	um01696	0.73	10.02
15c0002	-0.75	12.58	um01697	-1.01	12.10
15c0002	-0.14	9.99	um01698	-0.07	8.93
15c0003	-0.30	13.53	um01699	-0.75	12.35
15c0003	0.24	12.78	um01700	0.04	12.49
15c0003	0.07	8.90	um01701	-0.16	10.66
15c0003	0.04	11.70	um01702	-0.38	10.47
15c0003	-0.10	9.51	um01703	-0.02	10.91
15c0003	-1.50	12.17	um01705	-0.26	11.46
15c0003	-0.89	14.66	um01706	0.02	10.64
15c0003	0.57	10.90	um01708	-1.18	12.55
15c0003	0.06	13.04	um01709	0.30	10.38
15c0003	-0.32	13.19	um01710	-1.39	12.28
15c0004	0.12	11.62	um01711	-0.81	14.83
15c0004	-0.28	12.51	um01712	-0.07	14.44
15c0004	-0.14	8.71	um01713	1.35	10.92
15c0004	-0.01	12.55	um01714	0.64	14.49
15c0004	-0.06	9.74	um01716	-0.51	8.81
15c0004	-0.50	9.56	um01717	0.17	11.27
15c0004	0.11	12.97	um01718	0.12	9.51
15c0004	-0.32	13.72	um01720	-0.53	11.56
15c0004	0.23	12.48	um01721	-0.58	11.08
15c0004	0.02	13.67	um01722	-1.37	9.81
15c0005	-0.19	11.13	um01723	3.44	13.23
15c0005	0.25	11.20	um01724	1.02	11.86
15c0005	-0.32	11.49	um01725	-0.32	15.43
15c0005	0.61	13.06	um01726	-1.55	12.72
15c0005	0.38	10.58	um01728	0.50	13.23

15c0005	0.27	12.86	um0172	0.01	10.37
15c0005	0.66	13.65	um0173	-0.55	10.55
15c0005	0.17	13.58	um0173	-0.15	8.96
15c0005	-0.11	12.12	um0173	-0.10	11.21
15c0005	0.66	13.19	um0173	-0.09	9.24
15c0006	0.04	8.72	um0173	-0.09	8.58
15c0006	0.00	8.50	um0173	0.35	12.92
15c0006	0.60	14.70	um0173	0.59	11.69
15c0006	0.07	8.65	um0173	0.64	11.72
15c0006	0.12	8.69	um0173	0.69	11.58
15c0006	0.09	8.56	um0173	0.07	12.80
15c0006	-0.29	8.80	um0174	-0.33	11.98
15c0006	0.06	8.81	um0174	-0.15	10.31
15c0006	0.43	11.63	um0174	1.54	12.88
15c0006	0.10	13.15	um0174	-0.32	12.37
15c0007	-0.24	9.73	um0175	1.13	11.67
15c0007	0.31	12.14	um0175	0.27	11.82
15c0007	0.23	13.08	um0175	-0.03	12.33
15c0007	-0.07	13.55	um0175	-1.33	11.11
15c0007	0.06	11.71	um0175	-0.13	14.14
15c0007	-0.30	11.73	um0175	0.12	8.60
15c0007	0.26	11.48	um0175	-0.52	12.08
15c0007	0.13	12.81	um0175	0.15	11.17
15c0007	0.16	12.64	um0176	0.15	10.35
15c0007	0.02	12.47	um0176	0.06	10.65
15c0008	0.50	12.08	um0176	0.11	12.08
15c0008	0.35	12.85	um0176	0.66	11.05
15c0008	-0.04	13.23	um0176	0.06	11.81
15c0008	-0.56	12.43	um0177	-1.03	12.82
15c0008	-0.20	11.24	um0177	-0.04	11.22
15c0008	-0.49	12.02	um0177	-0.35	11.01
15c0008	0.77	11.91	um0177	-0.27	10.09
15c0008	-0.03	11.11	um0177	-0.84	12.44
15c0008	-0.16	10.11	um0177	0.12	10.70
15c0008	-0.10	8.77	um0177	-0.30	12.39
15c0009	0.28	8.56	um0177	0.13	9.64
15c0009	0.02	10.02	um0177	-0.18	8.56
15c0009	-0.09	9.71	um0178	0.77	13.11
15c0009	0.00	8.73	um0178	-1.02	11.60
15c0009	0.15	8.78	um0178	-0.06	14.00
15c0009	-0.02	8.58	um0178	-3.03	10.09
15c0009	-0.08	8.63	um0178	0.17	11.34
15d0000	2.16	11.48	um0178	-1.03	10.73
15d0000	-0.04	15.66	um0178	-0.27	14.67
15d0000	0.00	10.24	um0178	0.07	13.07
15d0000	0.24	12.23	um0179	0.57	10.77
15d0000	0.27	13.43	um0179	-0.61	10.15
15d0000	0.01	10.90	um0179	1.28	13.06
15d0000	-0.59	11.95	um0179	1.59	11.12
15d0000	-0.71	12.89	um0179	-0.09	11.12
15d0000	-0.14	8.79	um0179	-0.13	12.49
15d0001	-0.26	13.06	um0179	0.52	10.63
15d0001	-0.06	13.41	um0179	0.11	11.84
15d0001	0.49	12.89	um0179	0.20	14.60

15d0001	0.12	12.39	um01800	-1.03	11.91
15d0001	-0.58	9.97	um01802	-0.28	12.84
15d0001	0.19	12.51	um01803	0.14	12.42
15d0001	-0.06	12.12	um01804	-2.46	10.31
15d0001	-0.11	10.20	um01805	2.17	12.00
15d0001	0.03	11.46	um01808	-0.05	12.91
15d0001	0.45	13.68	um01811	-0.02	12.92
15d0002	0.25	11.52	um01812	-2.19	13.21
15d0002	0.15	12.42	um01813	-0.90	11.90
15d0002	0.03	15.78	um01814	-0.01	11.57
15d0002	0.32	10.73	um01815	0.80	12.49
15d0002	0.09	14.87	um01816	0.41	11.15
15d0002	-0.53	13.20	um01817	0.05	10.54
15d0002	-1.21	12.13	um01818	-0.51	11.88
15d0002	0.14	8.69	um01820	0.21	9.12
15d0002	-0.21	12.08	um01821	0.57	12.20
15d0002	0.25	15.86	um01823	0.63	9.58
15d0003	0.44	13.27	um01824	0.85	11.88
15d0003	0.27	11.93	um01826	-0.98	10.46
15d0003	0.11	13.27	um01827	1.51	13.06
15d0003	0.04	9.18	um01829	0.15	8.97
15d0003	-0.39	15.42	um01830	0.31	10.68
15d0003	-0.29	13.01	um01831	0.72	10.39
15d0003	-0.18	13.03	um01832	0.48	13.08
15d0003	0.02	15.95	um01833	0.05	11.89
15d0003	-0.07	13.53	um01834	0.24	10.61
15d0003	0.37	11.81	um01835	1.03	12.63
15d0004	0.01	14.24	um01837	-0.19	12.24
15d0004	0.26	10.37	um01841	-0.16	8.60
15d0004	-0.04	12.53	um01842	-0.07	9.39
15d0004	0.04	10.36	um01843	-0.14	11.18
15d0004	0.41	12.30	um01844	-0.17	8.68
15d0004	-0.44	11.75	um01845	0.34	12.13
15d0004	-0.37	12.24	um01847	1.59	10.81
15d0004	-0.14	9.02	um01849	-0.27	11.36
15d0004	0.13	12.19	um01850	1.03	14.29
15d0004	-0.24	9.61	um01851	0.34	14.50
15d0005	-0.36	8.89	um01852	0.37	12.16
15d0005	-0.21	8.97	um01853	-0.24	11.10
15d0005	-0.06	12.74	um01854	1.10	9.80
15d0005	0.16	11.15	um01855	0.18	9.09
15d0005	0.13	11.54	um01856	0.94	10.57
15d0005	-0.36	13.01	um01857	0.24	10.64
15d0005	0.47	10.71	um01858	-1.93	11.01
15d0005	-0.18	12.06	um01859	-1.33	11.56
15d0005	0.03	11.11	um01860	0.55	10.74
15d0005	-0.27	11.91	um01861	1.04	12.75
15d0006	0.06	8.48	um01862	0.24	10.41
15d0006	0.10	8.36	um01863	0.77	9.63
15d0006	0.06	13.33	um01864	0.51	10.58
15d0006	-0.42	11.41	um01866	-0.09	12.41
15d0006	0.24	14.02	um01867	-0.41	10.80
15d0006	0.13	11.37	um01868	1.14	12.30
15d0006	0.14	11.14	um01869	0.03	11.92

15d0006	0.56	12.13	um01870	0.14	13.29
15d0006	0.13	12.36	um01871	1.85	11.75
15d0006	0.07	12.63	um01872	2.66	13.84
15d0007	-0.34	10.02	um01873	-0.16	11.44
15d0007	0.15	9.51	um01874	0.33	10.79
15d0007	0.30	13.14	um01875	0.36	11.51
15d0007	0.28	12.84	um01877	0.56	11.32
15d0007	-0.44	12.15	um01879	0.37	12.41
15d0007	0.13	12.13	um01881	-0.48	13.45
15d0007	-0.78	13.00	um01882	-1.08	14.06
15d0007	0.02	12.04	um01883	-0.27	13.16
15d0007	0.09	9.27	um01885	-0.42	11.57
15d0007	-0.02	9.24	um01886	-0.02	14.32
15d0008	-0.53	12.42	um01888	-0.30	12.56
15d0008	-0.21	12.80	um01889	0.53	12.12
15d0008	-0.26	11.28	um01890	-0.41	8.89
15d0008	0.10	13.25	um01891	0.02	10.61
15d0008	-0.82	10.68	um01892	0.09	12.01
15d0008	0.04	8.69	um01893	0.28	11.88
15d0008	0.10	9.15	um01894	1.03	14.15
15d0008	-0.02	8.91	um01896	0.03	10.82
15d0008	0.07	9.22	um01897	0.17	8.85
15d0008	-0.09	9.17	um01898	-2.48	11.20
15d0009	-0.17	8.76	um01899	1.14	10.09
15d0009	-0.11	8.66	um01900	0.02	13.17
15d0009	-0.08	8.65	um01901	-0.18	10.80
15d0009	0.12	8.55	um01902	-0.05	13.64
15d0009	-0.04	8.48	um01903	-0.49	11.40
16c0000	-0.30	8.89	um01904	-0.69	11.98
16c0000	0.15	8.73	um01905	0.67	12.06
16c0000	0.02	11.59	um01906	0.87	11.87
16c0000	0.00	9.38	um01908	0.34	10.83
16c0000	-0.93	12.62	um01911	1.31	12.81
16c0000	0.59	13.89	um01913	0.16	10.34
16c0000	-0.26	11.88	um01916	0.18	11.26
16c0000	-0.02	8.97	um01917	1.05	11.78
16c0000	0.02	9.58	um01919	0.41	12.34
16c0001	-0.26	13.27	um01920	0.36	11.59
16c0001	0.38	12.33	um01922	1.38	11.12
16c0001	1.04	14.93	um01923	0.14	11.93
16c0001	0.22	12.11	um01926	-0.43	11.84
16c0001	-0.02	12.01	um01927	-0.10	12.46
16c0001	0.05	10.92	um01928	-0.42	12.15
16c0001	0.21	10.68	um01929	0.02	12.57
16c0001	-0.67	12.55	um01931	0.08	9.98
16c0001	0.23	12.46	um01932	-0.20	10.15
16c0001	0.15	12.20	um01933	-0.99	13.74
16c0002	-0.44	13.48	um01934	-0.44	12.72
16c0002	0.19	12.70	um01935	-0.10	13.30
16c0002	-0.06	8.78	um01936	-0.95	14.27
16c0002	0.13	10.32	um01937	0.08	11.53
16c0002	-0.44	12.24	um01938	-0.02	12.62
16c0002	-0.22	12.42	um01939	-0.27	11.48
16c0002	0.38	14.29	um01940	-0.03	11.91

16c0002	-0.21	9.55	um01941	0.57	10.60
16c0002	-0.52	10.36	um01942	-0.20	12.14
16c0002	-1.54	10.02	um01943	0.69	10.69
16c0003	-0.02	13.70	um01944	-0.16	8.81
16c0003	0.06	10.04	um01945	-0.79	11.50
16c0003	-0.60	14.41	um01946	0.26	10.95
16c0003	0.34	13.72	um01947	-3.23	10.23
16c0003	-0.26	11.64	um01948	-0.52	11.55
16c0003	-0.15	13.62	um01949	-3.25	10.75
16c0003	-0.12	12.68	um01950	-0.07	9.11
16c0003	-0.37	11.32	um01951	-1.77	11.35
16c0003	0.08	12.72	um01952	1.59	9.81
16c0003	-0.30	12.46	um01953	0.92	10.80
16c0004	-0.31	10.54	um01954	0.17	10.91
16c0004	-0.77	12.80	um01957	-0.10	11.41
16c0004	-0.09	12.53	um01958	0.35	11.02
16c0004	-0.14	12.33	um01959	0.56	10.91
16c0004	-0.31	12.59	um01961	-0.23	9.18
16c0004	-0.64	12.71	um01962	0.61	12.62
16c0004	-0.32	11.68	um01963	0.34	11.16
16c0004	-0.13	11.31	um01964	0.19	10.72
16c0004	-0.17	10.85	um01965	0.14	11.26
16c0004	-0.14	13.57	um01966	0.29	12.50
16c0005	-0.82	12.34	um01967	-0.59	12.12
16c0005	0.26	10.65	um01968	0.78	10.58
16c0005	0.45	13.99	um01969	0.70	11.48
16c0005	-0.46	11.24	um01970	1.15	13.31
16c0005	-0.24	11.83	um01971	2.76	13.61
16c0005	-0.31	13.02	um01974	0.02	12.06
16c0005	0.09	11.84	um01976	-1.28	11.58
16c0005	0.15	15.13	um01977	-0.81	10.25
16c0005	-0.26	13.47	um01978	-0.13	12.79
16c0005	-0.28	12.82	um01979	0.55	10.45
16c0006	-0.08	10.26	um01980	0.05	11.97
16c0006	0.10	12.75	um01981	0.60	12.00
16c0006	0.77	12.81	um01982	-0.18	10.92
16c0006	0.90	12.67	um01983	0.56	10.89
16c0006	-0.04	12.10	um01984	1.43	14.88
16c0006	-0.40	11.79	um01985	0.30	9.58
16c0006	0.37	9.12	um01986	0.92	14.26
16c0006	-0.03	12.77	um01987	0.18	9.14
16c0006	0.16	13.56	um01988	0.10	10.91
16c0006	0.98	13.67	um01989	-1.20	10.60
16c0007	-0.54	13.46	um01990	0.08	10.73
16c0007	-0.29	9.63	um01991	-0.15	11.14
16c0007	0.64	14.35	um01992	-0.77	11.98
16c0007	-0.94	11.59	um01994	-0.32	13.75
16c0007	-0.11	11.75	um01995	0.34	12.02
16c0007	0.37	12.07	um01996	-0.47	11.81
16c0007	-0.65	13.23	um01997	1.55	13.22
16c0007	0.02	10.46	um01999	0.14	12.20
16c0007	0.15	13.28	um02001	0.94	11.97
16c0007	0.65	11.13	um02002	-0.41	11.44
16c0008	0.67	10.91	um02003	-0.16	9.01

16c0008	0.16	11.54	um02004	-0.18	10.72
16c0008	0.30	10.42	um02005	-0.80	11.20
16c0008	0.20	11.25	um02006	0.97	9.81
16c0008	0.07	9.46	um02007	-0.56	12.50
16c0008	-0.44	10.35	um02008	1.29	12.11
16c0008	0.06	13.89	um02010	-0.44	11.09
16c0008	0.22	12.11	um02011	-0.15	9.69
16c0008	-0.06	8.68	um02012	1.38	11.46
16c0008	-0.13	11.48	um02014	0.10	12.38
16c0009	0.08	9.71	um02015	-0.18	11.87
16c0009	0.10	12.13	um02016	-0.10	11.93
16c0009	-0.32	11.67	um02017	0.08	11.38
16d0000	0.02	10.25	um02018	-0.12	11.63
16d0000	0.60	11.71	um02019	0.21	13.05
16d0000	0.04	12.36	um02020	-0.21	10.75
16d0000	0.09	12.92	um02021	0.17	8.99
16d0000	0.46	12.29	um02022	0.20	9.85
16d0000	-0.26	10.83	um02023	1.64	13.26
16d0000	0.07	8.55	um02024	-0.52	12.54
16d0000	0.18	11.66	um02028	0.56	11.29
16d0000	0.53	13.25	um02031	0.40	10.34
16d0001	0.05	9.80	um02034	0.33	11.80
16d0001	0.16	13.00	um02035	1.10	10.75
16d0001	-0.28	12.45	um02037	0.88	11.18
16d0001	0.24	12.27	um02039	0.29	11.39
16d0001	0.58	10.00	um02040	0.77	10.93
16d0001	0.23	12.43	um02041	0.24	11.47
16d0001	0.08	8.85	um02044	0.21	10.74
16d0001	-0.11	12.23	um02045	-0.05	11.98
16d0001	-0.03	10.30	um02046	-0.59	13.71
16d0001	-0.29	12.95	um02049	0.16	12.01
16d0002	-0.33	13.01	um02050	0.30	11.52
16d0002	0.08	11.92	um02052	1.24	12.32
16d0002	-0.69	11.31	um02053	-0.16	10.47
16d0002	0.00	14.64	um02055	-0.03	11.93
16d0002	0.21	12.74	um02056	0.24	12.23
16d0002	0.56	12.63	um02057	-0.29	12.53
16d0002	0.00	12.80	um02059	0.10	11.05
16d0002	0.07	11.23	um02062	0.04	12.03
16d0002	0.28	13.04	um02063	-0.86	11.20
16d0002	-0.54	11.91	um02064	0.17	14.16
16d0003	0.17	12.54	um02065	-0.09	12.33
16d0003	-0.16	13.06	um02066	-0.06	12.29
16d0003	-0.17	11.89	um02067	-0.14	11.56
16d0003	0.24	13.08	um02068	0.59	11.03
16d0003	0.12	11.46	um02069	0.27	12.35
16d0003	0.32	12.17	um02070	0.15	12.13
16d0003	0.27	10.18	um02071	0.58	12.33
16d0003	-0.29	12.81	um02072	-0.99	11.61
16d0003	0.59	13.37	um02075	0.14	11.69
16d0003	-0.51	10.36	um02077	0.56	10.98
16d0004	-0.11	12.78	um02079	0.10	14.74
16d0004	-0.78	12.42	um02080	2.32	12.01
16d0004	-1.87	12.67	um02081	-0.42	12.43

16d0004	-0.47	10.20	um02083	-0.10	11.61
16d0004	-0.04	11.75	um02088	0.03	13.59
16d0004	-0.21	12.08	um02089	-0.10	11.04
16d0004	-0.39	12.69	um02091	-0.33	11.44
16d0004	-0.38	14.33	um02092	-2.54	11.97
16d0004	0.16	10.50	um02093	-0.12	12.54
16d0004	-0.02	12.37	um02094	0.14	11.75
16d0005	0.74	12.76	um02095	1.44	13.67
16d0005	-1.13	12.58	um02096	-0.21	8.45
16d0005	-0.89	13.71	um02097	1.27	12.49
16d0005	-0.12	12.77	um02101	-0.52	12.69
16d0005	0.35	11.51	um02102	0.11	11.62
16d0005	0.28	11.54	um02103	-0.24	11.41
16d0005	-0.41	12.78	um02104	0.52	11.41
16d0005	-0.32	11.93	um02105	0.69	13.02
16d0005	0.01	12.28	um02107	0.14	12.01
16d0005	-0.06	14.38	um02108	0.28	10.56
16d0006	0.44	14.17	um02111	-0.38	9.20
16d0006	0.59	13.77	um02112	0.37	10.56
16d0006	0.37	13.29	um02113	0.34	11.24
16d0006	0.38	13.19	um02114	-0.61	11.58
16d0006	-0.67	12.36	um02115	0.54	8.91
16d0006	-0.27	12.81	um02116	-0.03	10.96
16d0006	0.39	12.64	um02117	-1.24	10.74
16d0006	-0.05	12.13	um02118	-0.09	11.28
16d0006	-1.07	13.54	um02119	1.09	10.15
16d0006	-0.39	11.38	um02123	0.15	11.46
16d0007	-1.04	13.88	um02124	-0.38	11.54
16d0007	-0.46	14.96	um02125	-0.87	11.42
16d0007	0.46	13.11	um02126	0.39	12.48
16d0007	-0.68	11.02	um02127	0.33	11.35
16d0007	-0.57	11.86	um02128	-0.21	11.35
16d0007	0.09	11.87	um02131	0.93	11.71
16d0007	1.49	12.87	um02132	0.08	12.03
16d0007	-0.03	11.57	um02133	-0.44	11.03
16d0007	-1.24	10.74	um02134	-0.59	11.57
16d0007	0.18	10.45	um02135	0.14	9.96
16d0008	-0.18	10.59	um02136	-0.17	8.84
16d0008	-0.08	11.25	um02137	-0.32	9.14
16d0008	0.20	11.84	um02138	0.01	8.86
16d0008	0.03	9.16	um02139	0.44	11.02
16d0008	0.08	8.40	um02140	0.36	9.42
16d0008	0.21	13.54	um02141	0.00	9.13
16d0008	-0.45	11.78	um02142	0.67	11.06
16d0008	-0.43	10.70	um02144	0.19	9.13
17d0000	0.07	8.73	um02146	-0.21	13.09
18c0000	0.22	10.78	um02147	-0.25	11.23
18c0000	0.00	13.32	um02148	0.55	11.29
18c0000	-0.05	12.28	um02149	0.51	10.50
18c0000	-0.18	10.93	um02150	-0.86	12.73
18c0000	-0.09	8.80	um02151	-0.18	12.99
18c0000	0.86	11.85	um02152	0.76	9.96
18c0000	-0.30	12.73	um02154	-0.61	12.25
18c0000	-0.50	11.35	um02156	0.31	10.19

18c0000	0.26	12.97	um02157	1.29	10.08
18c0001	0.07	8.89	um02158	-0.86	15.05
18c0001	-0.69	10.70	um02159	-0.19	12.18
18c0001	0.25	10.57	um02160	1.12	11.20
18c0001	0.42	11.54	um02161	1.69	12.75
18c0001	-0.14	12.44	um02162	0.44	12.38
18c0001	0.14	12.46	um02163	-0.17	9.94
18c0001	-0.13	13.11	um02164	-0.81	12.19
18c0001	-0.40	10.59	um02165	-0.65	12.46
18c0001	0.70	13.70	um02166	1.60	11.85
18c0001	0.19	10.59	um02167	-0.13	12.11
18c0002	0.03	12.21	um02168	-0.12	12.27
18c0002	-0.11	11.19	um02169	2.52	11.99
18c0002	0.04	9.57	um02170	0.81	10.83
18c0002	0.04	13.29	um02172	1.50	11.41
18c0002	0.32	12.19	um02174	-0.15	8.66
18c0002	-0.17	8.86	um02175	-1.99	11.88
18c0002	-0.49	11.52	um02177	-0.02	10.85
18c0002	0.19	13.33	um02178	-0.12	12.32
18c0002	0.02	12.39	um02179	-0.38	11.99
18c0002	-0.53	11.72	um02180	0.26	10.96
18c0003	-0.10	9.10	um02181	0.28	12.40
18c0003	-1.17	10.84	um02182	1.62	13.79
18c0003	-0.09	12.39	um02185	0.76	11.29
18c0003	0.30	10.48	um02186	-0.08	10.85
18c0003	0.85	13.32	um02189	0.71	12.11
18c0003	0.60	11.20	um02190	-0.21	11.28
18c0003	0.44	12.62	um02191	-1.90	12.82
18c0003	0.05	9.32	um02192	0.13	9.37
18c0003	-0.10	11.41	um02193	-0.15	8.71
18c0003	-0.35	14.77	um02194	-0.25	8.64
18c0004	0.16	14.38	um02196	-0.34	8.58
18c0004	-0.23	12.97	um02197	1.48	11.84
18c0004	-0.50	12.33	um02198	1.46	12.68
18c0004	-0.11	9.13	um02199	0.65	10.65
18c0004	-0.24	8.71	um02201	-1.31	11.11
18c0004	0.04	13.82	um02202	-0.24	11.93
18c0004	-0.47	12.45	um02203	1.42	10.72
18c0004	-0.08	9.78	um02204	0.83	9.42
18c0004	-0.78	12.06	um02206	-0.33	9.28
18c0004	-0.04	10.32	um02207	-1.80	11.89
18c0005	-0.04	10.97	um02208	1.59	13.45
18c0005	0.20	14.06	um02209	-0.39	11.82
18c0005	0.05	11.56	um02211	0.16	12.24
18c0005	-0.45	9.73	um02212	0.30	11.67
18c0005	-0.52	11.92	um02213	0.66	12.34
18c0005	0.14	10.75	um02214	-0.31	11.42
18c0005	0.28	12.52	um02215	-4.84	11.27
18c0005	0.05	11.55	um02216	0.04	11.61
18c0005	0.10	12.28	um02217	-0.22	12.41
18c0005	0.12	11.70	um02218	0.24	11.79
18c0006	-0.58	12.22	um02219	0.60	14.65
18c0006	0.00	11.94	um02220	0.75	11.09
18c0006	-0.45	13.27	um02221	0.24	12.62

18c0006	0.04	11.44	um02222	-0.14	8.83
18c0006	-0.24	13.13	um02223	0.75	10.60
18c0006	0.70	11.76	um02224	0.40	11.23
18c0006	0.00	9.51	um02225	0.37	10.60
18c0006	-0.03	12.27	um02226	-0.14	10.44
18c0006	0.02	8.79	um02227	0.39	10.93
18c0006	-0.24	10.91	um02229	-0.15	9.38
18c0007	0.31	10.37	um02230	0.35	10.63
18c0007	0.62	13.76	um02231	0.21	9.10
18c0007	-0.41	11.78	um02232	0.58	12.30
18c0007	-0.27	11.29	um02234	0.23	11.64
18c0007	-0.47	11.77	um02235	1.41	12.56
18c0007	0.04	12.38	um02236	0.88	10.59
18c0007	0.26	13.11	um02237	-0.32	11.89
18c0007	0.30	10.31	um02238	-0.58	10.25
18c0007	0.13	14.41	um02239	0.29	9.57
18c0007	-0.24	14.19	um02242	0.27	10.56
18c0008	-0.51	11.93	um02243	0.18	11.76
18c0008	-0.22	13.44	um02244	-0.05	12.18
18c0008	-0.19	8.63	um02245	-0.16	13.45
18c0008	-0.43	11.37	um02246	-0.45	11.03
18c0008	0.06	12.86	um02247	0.62	10.95
18c0008	0.24	13.09	um02249	0.27	12.13
18c0008	0.04	13.88	um02251	1.46	10.36
18c0008	0.30	10.10	um02252	-0.27	11.08
18c0008	0.06	10.70	um02253	-0.20	11.78
18c0008	-0.14	10.87	um02254	0.77	11.96
18c0009	-0.09	10.72	um02255	-0.03	12.28
18c0009	0.05	8.79	um02256	-0.96	10.35
18c0009	-0.15	12.30	um02258	-0.58	13.81
18c0009	-0.60	13.68	um02259	0.29	10.97
18c0009	0.24	11.63	um02260	-0.22	12.26
18c0009	0.50	13.78	um02262	-0.07	11.33
18c0009	0.02	9.27	um02263	0.20	12.55
18c0009	0.23	13.37	um02264	0.31	12.21
18c0009	0.69	10.98	um02266	-0.12	12.77
18c0009	0.01	8.99	um02267	-0.53	15.66
18c0010	0.18	9.81	um02268	0.25	11.45
18c0010	0.26	10.99	um02271	0.26	10.84
18c0010	-0.18	9.19	um02272	0.15	13.34
18c0010	-0.19	8.53	um02273	0.94	12.76
18c0010	-0.21	8.96	um02274	0.50	10.45
18c0010	-0.40	11.15	um02275	0.52	10.75
18c0010	0.19	8.83	um02276	0.29	14.62
18d0000	0.31	10.87	um02280	0.44	12.49
18d0000	0.02	9.53	um02281	0.25	11.41
18d0000	0.45	12.64	um02282	0.25	13.01
18d0000	0.25	11.81	um02283	-0.26	11.19
18d0000	0.87	11.61	um02284	-0.36	10.07
18d0000	0.04	8.49	um02285	-0.61	12.15
18d0000	0.24	9.97	um02286	0.49	12.19
18d0000	-0.02	8.61	um02289	-0.22	11.43
18d0000	0.33	9.55	um02290	-0.51	12.92
18d0001	-0.04	10.91	um02291	-0.43	11.29

18d0001	1.40	14.80	um02292	0.40	11.31
18d0001	-0.15	11.78	um02293	-0.11	8.71
18d0001	0.55	13.26	um02294	-0.19	8.56
18d0001	0.08	10.51	um02295	-0.47	8.60
18d0001	-0.35	13.30	um02296	-0.25	9.18
18d0001	-0.21	11.75	um02297	0.34	10.65
18d0001	0.66	12.80	um02298	-0.20	9.49
18d0001	0.40	13.27	um02299	-0.11	8.71
18d0001	-0.12	13.38	um02300	-0.69	13.04
18d0002	-0.53	12.11	um02301	0.10	14.71
18d0002	-1.54	12.68	um02303	0.47	11.81
18d0002	-1.11	14.07	um02304	-0.63	10.20
18d0002	-1.03	12.72	um02305	0.15	12.84
18d0002	-0.02	13.45	um02308	0.19	10.86
18d0002	-0.08	8.96	um02310	0.19	11.94
18d0002	0.49	12.59	um02311	-0.06	10.97
18d0002	0.04	9.04	um02312	0.41	11.56
18d0002	0.32	12.81	um02313	0.06	8.82
18d0002	0.17	12.56	um02314	-0.57	9.42
18d0003	-0.40	13.18	um02316	0.56	11.49
18d0003	-0.34	12.51	um02318	0.62	11.33
18d0003	-0.05	9.25	um02320	-0.19	13.72
18d0003	-0.42	12.22	um02321	-0.21	10.25
18d0003	0.29	9.22	um02323	0.36	12.69
18d0003	0.24	12.64	um02324	0.59	12.33
18d0003	0.03	12.57	um02325	0.63	10.34
18d0003	0.09	12.53	um02326	0.35	12.10
18d0003	0.46	12.51	um02327	-0.13	10.80
18d0003	-0.19	12.91	um02331	-0.50	12.90
18d0004	0.34	12.08	um02332	-0.71	9.31
18d0004	0.81	13.26	um02333	0.50	9.96
18d0004	0.10	12.32	um02336	0.97	11.97
18d0004	-0.53	10.74	um02337	-0.08	11.01
18d0004	-0.55	11.82	um02338	0.32	11.88
18d0004	0.03	11.08	um02340	-0.33	12.74
18d0004	-0.01	9.34	um02341	-0.35	12.07
18d0004	-0.35	13.01	um02342	-0.16	10.72
18d0004	0.04	9.24	um02343	0.07	11.44
18d0004	0.43	11.00	um02344	-0.13	12.01
18d0005	0.04	12.27	um02345	-0.54	10.26
18d0005	0.12	11.77	um02346	0.32	11.03
18d0005	0.12	12.04	um02347	-0.79	14.67
18d0005	-0.10	13.12	um02350	-0.31	13.42
18d0005	0.10	12.69	um02351	0.03	11.71
18d0005	-0.50	13.61	um02353	0.13	15.21
18d0005	0.33	11.39	um02354	0.30	11.50
18d0005	-0.05	9.02	um02356	0.28	11.93
18d0005	-0.10	10.19	um02357	0.04	12.93
18d0005	-0.28	10.77	um02358	0.21	11.63
18d0006	-0.37	11.90	um02360	-0.39	14.43
18d0006	-0.19	11.04	um02361	-0.06	15.32
18d0006	0.12	13.26	um02363	1.02	9.78
18d0006	-0.25	15.50	um02365	-0.16	12.25
18d0006	-0.43	12.82	um02366	0.51	11.61

18d0006	0.06	8.78	um02367	0.08	11.02
18d0006	0.14	8.52	um02370	-0.18	8.63
18d0006	0.01	8.86	um02371	1.72	13.77
18d0006	0.23	12.23	um02374	-0.47	10.50
18d0006	0.13	11.81	um02375	-0.38	12.64
18d0007	0.48	12.47	um02376	-0.16	11.99
18d0007	0.96	13.04	um02377	0.52	14.24
18d0007	-0.08	13.47	um02378	-0.10	12.15
18d0007	-0.03	10.96	um02379	-0.07	8.70
18d0007	0.24	13.74	um02380	0.59	11.22
18d0007	0.04	13.41	um02381	-0.71	13.96
18d0007	0.52	13.53	um02382	-1.00	10.15
18d0007	0.25	12.78	um02383	0.70	10.61
18d0007	-0.38	12.23	um02384	-0.33	13.79
18d0007	0.25	13.39	um02385	-0.27	11.99
18d0008	0.01	12.38	um02386	-0.19	12.55
18d0008	0.52	12.37	um02387	-0.07	9.35
18d0008	-0.69	13.91	um02388	-0.45	13.28
18d0008	-0.52	12.11	um02390	-0.28	11.56
18d0008	-0.08	10.65	um02392	0.40	11.54
18d0008	0.21	10.53	um02395	0.16	13.87
18d0008	-0.35	11.64	um02396	0.68	14.69
18d0008	0.60	12.36	um02397	0.05	12.46
18d0008	0.00	12.16	um02398	-0.90	12.00
18d0008	-0.17	9.12	um02400	0.41	11.93
18d0009	1.06	14.48	um02401	0.62	11.02
18d0009	-0.08	11.12	um02402	0.00	12.59
18d0009	0.45	12.05	um02403	-0.23	11.32
18d0009	0.47	13.20	um02404	-0.80	11.18
18d0009	0.49	13.21	um02405	-0.23	12.87
18d0009	0.29	13.28	um02407	-0.27	12.09
18d0009	0.23	11.81	um02410	0.99	11.30
18d0009	-0.07	13.47	um02411	-0.89	13.91
18d0009	-0.19	10.00	um02412	0.81	14.10
18d0009	-0.03	12.02	um02413	1.27	11.44
18d0010	0.16	12.85	um02414	0.24	11.03
18d0010	0.43	10.87	um02415	-0.76	10.84
18d0010	0.02	11.85	um02416	2.14	11.21
18d0010	-0.04	8.58	um02419	0.18	10.29
18d0010	-0.05	10.71	um02420	0.22	12.31
18d0010	0.09	11.30	um02421	-0.07	11.04
18d0010	-0.31	9.64	um02422	-0.16	12.51
18d0010	0.01	8.71	um02423	-0.21	8.63
18d0010	0.03	8.62	um02425	0.59	12.20
18d0010	0.15	10.03	um02426	-0.07	12.42
18d0011	0.19	9.01	um02427	0.38	11.32
18d0011	0.00	8.44	um02428	0.86	10.78
18d0011	-1.91	11.32	um02429	0.27	11.99
18d0011	0.04	8.64	um02430	1.34	14.83
18d0011	0.02	8.62	um02431	0.39	11.19
18d0011	-0.35	8.99	um02433	0.44	12.07
18d0011	-0.03	8.50	um02435	-0.43	10.36
19c0000	0.54	15.69	um02436	-0.47	11.96
19c0000	-0.95	14.88	um02437	-0.12	14.27

19c0000	0.00	11.32	um02438	-0.24	8.93
19c0000	0.01	8.55	um02439	0.28	11.85
19c0000	-1.92	12.47	um02440	0.07	15.51
19c0000	0.31	12.25	um02442	-0.57	14.73
19c0000	-0.07	8.63	um02444	0.32	11.51
19c0000	-0.13	10.85	um02445	-0.11	11.89
19c0000	-0.85	12.37	um02446	0.11	11.86
19c0001	-0.10	10.07	um02449	0.63	11.17
19c0001	-0.07	10.16	um02450	-0.23	15.54
19c0001	0.07	12.14	um02451	0.04	9.02
19c0001	-0.01	11.87	um02452	0.14	11.40
19c0001	-0.25	10.56	um02453	-0.18	14.36
19c0001	-0.04	8.75	um02454	-0.34	10.96
19c0001	0.09	13.34	um02456	-0.10	12.08
19c0001	0.38	11.56	um02457	-0.48	10.74
19c0001	0.10	11.84	um02458	0.31	10.78
19c0001	-0.07	13.36	um02459	1.76	10.43
19c0002	-0.08	10.30	um02460	0.63	13.29
19c0002	0.07	14.68	um02461	0.08	14.26
19c0002	0.15	12.57	um02462	0.21	11.98
19c0002	-0.21	11.23	um02463	0.49	12.88
19c0002	0.25	9.93	um02464	0.28	10.86
19c0002	0.25	10.88	um02465	-0.70	11.86
19c0002	-0.26	10.13	um02466	-0.43	8.66
19c0002	-0.26	11.97	um02468	0.39	11.25
19c0002	-0.01	8.74	um02469	-0.01	12.67
19c0002	0.61	11.49	um02471	-0.38	10.73
19c0003	-0.04	9.47	um02472	-0.41	10.75
19c0003	-0.07	9.88	um02473	-0.27	8.65
19c0003	0.22	11.15	um02474	-0.18	8.83
19c0003	-0.22	10.19	um02475	-0.15	9.76
19c0003	0.42	11.43	um02476	-0.36	12.37
19c0003	-0.29	12.56	um02477	0.55	14.52
19c0003	-0.34	12.40	um02478	-0.37	10.94
19c0003	0.06	8.64	um02481	0.85	12.23
19c0003	0.27	11.47	um02482	0.37	11.17
19c0003	-0.06	10.76	um02483	0.25	12.64
19c0004	0.11	8.96	um02485	-0.49	13.73
19c0004	-0.15	12.24	um02487	0.09	11.32
19c0004	0.43	11.91	um02489	-0.01	14.22
19c0004	0.12	11.16	um02490	0.36	9.16
19c0004	0.72	11.18	um02491	-0.63	15.84
19c0004	-1.83	12.82	um02492	-0.70	11.72
19c0004	0.04	9.12	um02493	-0.39	10.52
19c0004	-0.09	11.87	um02494	0.19	13.02
19c0004	-0.21	12.50	um02495	-0.49	13.28
19c0004	-0.43	11.98	um02496	-0.44	11.34
19c0005	-0.20	12.33	um02497	-0.62	10.85
19c0005	0.30	13.02	um02498	-0.29	12.28
19c0005	-0.03	8.81	um02499	-0.06	11.80
19c0005	0.08	12.20	um02500	0.20	11.03
19c0005	-0.11	11.72	um02501	-0.42	10.96
19c0005	-0.34	11.28	um02502	-1.08	11.97
19c0005	0.00	11.90	um02504	0.34	10.70

19c0005	0.05	12.90	um02506	0.07	8.59
19c0005	-0.15	14.14	um02508	2.12	14.37
19c0005	0.45	10.10	um02510	-0.09	8.91
19c0006	0.11	11.78	um02514	0.42	12.04
19c0006	0.07	9.99	um02516	0.29	12.21
19c0006	0.49	13.33	um02517	0.93	12.19
19c0006	0.47	10.68	um02519	0.08	11.96
19c0006	-0.28	12.57	um02520	0.09	11.18
19c0006	-0.07	9.33	um02521	1.36	11.13
19c0006	-0.01	12.38	um02523	-0.17	8.96
19c0006	-0.15	12.81	um02524	-0.27	11.02
19c0006	-0.43	10.16	um02525	0.03	11.08
19c0006	-0.32	11.80	um02526	0.18	10.74
19c0007	-0.29	13.94	um02527	-0.22	12.81
19c0007	-0.16	12.00	um02528	0.90	12.47
19c0007	-0.53	13.71	um02529	0.79	10.40
19c0007	0.16	13.45	um02530	-0.34	11.90
19c0007	-0.49	13.83	um02531	0.32	11.55
19c0007	-0.23	11.36	um02533	-0.16	9.35
19c0007	-0.20	13.86	um02535	-0.18	8.49
19c0007	0.06	12.29	um02537	-0.11	8.58
19c0007	-0.34	13.72	um02538	-0.03	8.59
19c0007	0.07	9.05	um02540	-0.16	10.04
19c0008	-0.33	12.82	um02541	-0.19	12.06
19c0008	0.24	11.94	um02542	0.81	11.27
19c0008	0.01	12.62	um02543	0.37	10.69
19c0008	-0.17	12.02	um02546	0.34	12.54
19c0008	-0.27	11.35	um02548	-0.78	10.70
19c0008	-0.18	12.34	um02549	-0.25	8.60
19c0008	0.12	12.93	um02550	0.41	11.34
19c0008	0.31	13.28	um02551	-0.05	10.76
19c0008	-0.56	13.40	um02552	-0.11	10.84
19c0008	0.45	15.04	um02553	0.09	11.40
19c0009	-0.21	11.12	um02554	0.44	12.45
19c0009	0.13	11.49	um02555	0.19	11.36
19c0009	-0.02	12.16	um02556	-0.26	11.91
19c0009	0.00	8.61	um02557	-0.48	10.06
19c0009	-0.10	12.27	um02558	0.30	10.52
19c0009	0.00	9.54	um02559	-0.25	14.45
19c0009	-0.31	13.11	um02560	-0.25	13.30
19c0009	-0.08	8.82	um02561	0.07	11.98
19c0009	-0.56	14.57	um02562	-0.10	13.39
19c0009	0.66	12.64	um02564	-0.08	11.10
19c0010	0.15	11.55	um02565	1.27	11.05
19c0010	0.20	11.36	um02567	0.10	11.62
19c0010	0.30	12.55	um02568	-0.33	11.03
19c0010	-0.10	11.87	um02571	0.21	12.83
19c0010	-0.14	9.63	um02572	-0.13	11.41
19c0010	0.32	13.56	um02573	0.73	10.98
19c0010	0.09	8.73	um02574	0.64	11.36
19c0010	-0.30	12.55	um02575	-0.21	12.49
19c0010	0.19	13.17	um02576	-0.24	11.08
19c0010	-0.32	12.26	um02577	-0.75	15.66
19c0011	0.16	12.42	um02578	-0.49	12.86

19c0011	0.22	11.66	um02579	-1.08	13.04
19c0011	-0.28	14.67	um02580	-0.20	11.28
19c0011	0.19	12.14	um02581	0.53	14.65
19c0011	-0.04	10.06	um02582	-0.39	11.56
19c0011	-0.75	13.10	um02583	-1.33	12.85
19c0011	-0.03	12.34	um02584	-0.66	12.95
19c0011	0.12	12.34	um02585	-2.76	10.68
19c0011	0.08	11.06	um02586	-0.66	13.28
19c0011	0.21	10.35	um02587	-0.03	12.61
19c0012	-0.05	8.92	um02589	0.00	8.91
19c0012	0.06	11.64	um02590	0.93	11.06
19c0012	-0.28	9.80	um02591	0.00	10.94
19c0012	0.13	10.63	um02592	0.34	14.29
19c0012	0.34	11.74	um02594	0.65	11.03
19c0012	0.35	10.69	um02595	0.65	11.62
19c0012	0.22	15.39	um02596	-0.51	12.36
19c0012	-0.63	11.58	um02597	0.28	9.53
19c0012	0.47	12.25	um02598	-0.33	9.08
19c0012	0.21	12.12	um02599	-0.20	8.95
19c0013	0.15	13.76	um02600	-0.07	11.44
19c0013	-0.06	9.55	um02601	-0.27	8.92
19c0013	-0.22	8.88	um02602	0.61	11.37
19c0013	-0.15	11.77	um02603	0.46	11.31
19c0013	-0.31	11.66	um02604	-0.25	8.71
19c0013	0.16	9.75	um02605	0.49	11.40
19c0013	-0.02	12.33	um02606	0.12	11.55
19c0013	-0.22	12.07	um02607	0.28	12.11
19c0013	0.11	13.34	um02609	-0.04	12.81
19c0013	0.47	12.89	um02610	1.09	12.05
19c0014	-0.05	11.65	um02611	-0.66	13.68
19c0014	-0.21	10.73	um02613	-0.33	11.41
19c0014	0.17	12.45	um02614	-0.05	11.75
19c0014	-0.20	11.36	um02615	0.05	9.73
19c0014	0.15	12.17	um02618	0.60	10.99
19c0014	-0.24	10.91	um02619	-0.07	10.94
19c0014	-1.15	12.65	um02620	-0.09	13.23
19c0014	-0.20	11.26	um02622	-0.02	13.73
19c0014	-0.17	10.94	um02623	-0.17	11.66
19c0014	0.00	11.99	um02624	0.60	12.85
19c0015	-0.08	9.71	um02625	-4.48	10.88
19d0000	0.40	13.91	um02626	-4.44	11.11
19d0000	0.34	15.72	um02627	0.17	10.39
19d0000	-0.04	15.92	um02628	-0.40	10.99
19d0000	0.00	15.92	um02629	-0.46	8.73
19d0000	0.38	9.61	um02630	-0.63	12.50
19d0000	-0.86	11.51	um02631	0.02	11.64
19d0000	0.38	10.92	um02632	-0.53	12.18
19d0000	0.07	9.80	um02635	-0.37	11.68
19d0000	-1.48	14.29	um02637	-0.04	12.61
19d0001	-0.66	12.98	um02638	-0.03	11.72
19d0001	0.20	12.82	um02639	-0.46	12.65
19d0001	0.21	12.14	um02640	1.05	12.79
19d0001	-0.38	14.53	um02641	1.16	12.09
19d0001	-0.21	14.24	um02642	-1.22	13.56

19d0001	-0.29	11.25	um02645	0.33	11.59
19d0001	0.01	8.86	um02649	0.35	11.29
19d0001	-0.09	10.52	um02651	-0.06	13.01
19d0001	-0.13	10.97	um02652	1.21	14.15
19d0001	0.48	11.72	um02653	-0.44	12.45
19d0002	0.74	12.89	um02654	0.66	10.77
19d0002	0.26	13.71	um02655	0.68	11.10
19d0002	0.56	12.32	um02656	0.04	11.22
19d0002	0.53	11.44	um02657	-0.07	11.55
19d0002	0.63	13.29	um02659	-0.25	11.05
19d0002	-0.10	8.94	um02662	0.35	11.47
19d0002	-0.28	14.63	um02663	-0.04	12.30
19d0002	-0.25	14.43	um02664	0.02	11.11
19d0002	1.01	10.33	um02665	-0.34	13.98
19d0002	0.11	8.75	um02666	-0.22	13.80
19d0003	-0.27	11.76	um02667	0.05	11.59
19d0003	-0.04	9.17	um02668	-0.64	11.75
19d0003	0.23	11.83	um02669	0.68	12.25
19d0003	0.01	13.39	um02672	-0.18	13.04
19d0003	0.22	9.95	um02674	0.43	10.92
19d0003	-0.05	14.51	um02676	0.43	11.49
19d0003	0.84	13.45	um02677	0.34	11.11
19d0003	0.13	8.59	um02678	-0.59	12.46
19d0003	-0.04	12.40	um02682	0.51	11.03
19d0003	0.18	13.89	um02683	0.24	10.55
19d0004	0.47	13.30	um02684	-0.17	12.59
19d0004	-0.58	13.22	um02685	0.17	10.52
19d0004	-0.21	10.94	um02686	-0.43	11.59
19d0004	-0.09	11.28	um02687	-0.02	12.21
19d0004	0.00	12.22	um02688	-0.08	11.48
19d0004	0.52	12.91	um02690	-0.31	9.76
19d0004	-0.85	12.41	um02693	-0.79	13.18
19d0004	-0.39	14.02	um02694	0.70	11.75
19d0004	0.39	12.28	um02696	0.06	12.26
19d0004	-0.41	12.99	um02697	0.02	11.88
19d0005	-0.19	12.60	um02700	-0.02	11.25
19d0005	0.01	10.10	um02701	3.38	12.62
19d0005	0.20	11.80	um02703	0.71	13.83
19d0005	0.20	12.71	um02704	1.89	11.89
19d0005	0.17	9.58	um02707	2.04	13.82
19d0005	-0.15	9.62	um02708	1.11	14.06
19d0005	-0.46	11.74	um02709	-0.85	13.00
19d0005	0.93	13.18	um02710	-0.80	15.67
19d0005	0.19	13.22	um02711	0.11	11.20
19d0005	-0.93	12.72	um02712	0.72	13.22
19d0006	0.06	10.86	um02713	0.15	10.12
19d0006	-0.29	10.37	um02715	0.24	14.84
19d0006	0.29	11.53	um02716	0.62	11.03
19d0006	0.45	12.19	um02717	-0.14	13.15
19d0006	-0.40	12.53	um02718	-0.24	11.33
19d0006	-0.33	11.44	um02719	0.24	12.46
19d0006	0.07	11.07	um02720	-2.32	12.49
19d0006	0.02	12.40	um02721	-1.27	11.43
19d0006	-0.41	11.51	um02722	-0.09	8.58

19d0006	0.30	13.12	um02723	-0.63	9.40
19d0007	0.43	13.33	um02724	-0.37	11.26
19d0007	0.21	13.32	um02725	0.07	11.03
19d0007	-0.19	12.80	um02727	0.42	11.95
19d0007	-0.70	12.48	um02729	-0.25	11.87
19d0007	0.24	10.09	um02730	-0.35	12.56
19d0007	0.16	12.14	um02731	-0.13	11.75
19d0007	-0.97	11.54	um02732	-0.38	12.33
19d0007	-0.89	9.94	um02733	-0.14	12.37
19d0007	-0.07	11.51	um02736	-0.02	11.77
19d0007	-0.40	13.61	um02739	-0.22	11.80
19d0008	-0.32	11.32	um02740	0.47	9.61
19d0008	-0.48	12.41	um02741	-0.92	11.27
19d0008	-0.12	12.08	um02742	0.42	12.42
19d0008	0.02	10.68	um02743	-0.36	12.55
19d0008	-0.09	11.35	um02744	-0.60	11.83
19d0008	0.08	12.11	um02745	-0.24	8.91
19d0008	0.11	11.15	um02746	1.82	12.33
19d0008	0.53	12.73	um02747	1.92	12.28
19d0008	0.20	13.91	um02750	-0.39	14.21
19d0008	0.54	12.78	um02751	0.02	10.85
19d0009	0.25	10.90	um02752	-0.34	10.04
19d0009	-0.82	13.40	um02753	1.22	9.78
19d0009	0.06	9.79	um02754	-0.35	13.55
19d0009	0.16	12.29	um02755	0.11	11.52
19d0009	-0.61	11.19	um02756	-0.14	8.96
19d0009	0.03	10.83	um02757	0.06	9.00
19d0009	-0.02	9.56	um02758	-0.57	8.89
19d0009	0.64	12.05	um02759	-0.07	8.75
19d0009	-0.73	11.48	um02760	-0.49	10.64
19d0009	-0.26	12.36	um02762	0.23	11.03
19d0010	0.05	12.68	um02763	0.71	14.54
19d0010	0.16	12.14	um02765	0.83	11.32
19d0010	0.57	13.63	um02768	-0.47	11.52
19d0010	0.57	9.15	um02769	0.22	13.48
19d0010	-0.11	12.25	um02770	0.29	9.04
19d0010	-0.28	10.75	um02771	0.59	11.84
19d0010	-0.20	14.17	um02772	0.21	11.86
19d0010	-0.12	13.68	um02773	0.24	15.32
19d0010	-0.14	11.13	um02774	0.21	12.19
19d0010	-0.18	9.14	um02775	-0.26	10.11
19d0011	0.71	11.92	um02777	-0.11	11.99
19d0011	0.36	12.71	um02778	0.67	13.59
19d0011	-0.20	15.82	um02779	-0.32	10.35
19d0011	0.13	13.00	um02782	-0.17	13.86
19d0011	0.05	11.15	um02783	-0.72	13.70
19d0011	0.05	12.65	um02784	0.14	10.93
19d0011	0.47	12.83	um02786	0.40	9.99
19d0011	0.36	13.58	um02787	0.54	9.70
19d0011	0.29	13.24	um02788	-0.20	8.64
19d0011	0.06	8.67	um02789	0.30	9.35
19d0012	-0.18	11.77	um02791	-1.37	12.33
19d0012	-0.11	8.66	um02792	0.29	12.32
19d0012	-0.32	12.36	um02793	0.51	9.69

19d0012	-0.04	10.92
19d0012	0.12	11.76
19d0012	0.45	13.32
19d0012	-1.00	13.60
19d0012	0.87	14.94
19d0012	1.19	11.70
19d0012	-1.04	12.41
19d0013	0.36	12.14
19d0013	0.08	8.92
19d0013	-0.11	10.26
19d0013	-0.41	11.92
19d0013	-0.16	9.53
19d0013	0.12	9.82
19d0013	0.00	11.23
19d0013	-0.36	12.06
19d0013	0.51	10.90
19d0013	-0.27	13.06
19d0014	0.46	12.50
19d0014	0.25	11.19
19d0014	-0.28	11.15
19d0014	0.32	13.38
19d0014	0.34	13.04
19d0014	-0.10	9.24
19d0014	0.02	8.66
19d0014	-0.01	8.46
19d0014	-0.10	8.87
19d0014	0.00	8.40
19d0015	-0.27	8.68
19d0015	-0.15	9.90
19d0015	0.07	9.78
19d0015	0.00	12.58
19d0015	-0.45	13.50
19d0015	-0.19	13.29
19d0015	0.06	8.78
19d0015	0.02	11.80
19d0015	-0.13	11.51
19d0015	-0.56	12.30
19d0016	0.13	12.83
19d0016	0.02	12.26
19d0016	0.12	12.91
19d0016	0.00	12.35
19d0016	-0.06	11.34
19d0016	-0.27	11.90
1c00001	0.17	9.06
1c00002	0.06	13.99
1c00003	0.28	13.10
1c00004	0.48	11.62
1c00005	-0.14	12.79
1c00006	0.14	9.19
1c00007	0.15	10.89
1c00008	0.12	11.78
1c00009	0.35	12.75
1c00010	0.41	12.63
1c00011	-0.42	13.81

um02794	0.00	13.18
um02796	-1.29	13.54
um02797	-0.44	12.84
um02799	0.42	12.86
um02801	-2.08	14.98
um02802	-0.36	11.30
um02803	-0.37	9.35
um02804	-0.19	9.68
um02807	-0.25	8.62
um02808	0.29	10.22
um02809	-1.64	11.95
um02810	-0.78	13.26
um02811	-2.70	12.06
um02812	-0.24	8.58
um02813	1.23	9.94
um02814	0.04	9.13
um02816	0.39	11.34
um02817	0.66	9.52
um02818	0.83	9.82
um02819	0.94	11.01
um02820	-0.96	9.41
um02821	0.49	10.07
um02823	-0.27	10.13
um02824	-0.18	11.27
um02825	-0.52	11.62
um02826	-0.25	8.66
um02827	0.30	12.22
um02828	-0.05	11.27
um02829	-0.02	11.02
um02830	-0.10	11.66
um02833	-0.37	12.79
um02835	-0.98	12.73
um02836	1.36	9.68
um02838	1.01	12.14
um02840	0.09	11.78
um02843	-0.66	12.66
um02844	0.05	12.18
um02845	-0.33	11.18
um02846	-0.37	11.53
um02847	0.28	10.79
um02849	-0.28	12.00
um02850	0.01	10.34
um02851	0.09	9.17
um02852	-0.47	8.70
um02853	-0.26	8.36
um02854	-0.25	8.64
um02855	-0.18	11.62
um02856	-0.21	12.21
um02857	0.11	11.58
um02859	-0.53	12.52
um02860	-0.17	12.77
um02861	-0.09	11.97
um02862	0.51	11.80
um02863	0.48	10.83

1c00012	0.13	11.85	um02864	0.42	10.86
1c00013	-0.27	10.72	um02865	2.29	13.29
1c00014	0.46	12.20	um02867	-0.27	13.08
1c00015	-0.27	12.78	um02868	-0.11	10.82
1c00016	-0.67	11.63	um02869	0.34	12.03
1c00017	-0.91	12.29	um02874	-0.25	10.31
1c00018	-0.23	11.49	um02876	0.74	12.73
1c00019	0.58	13.56	um02877	-0.35	8.85
1c00020	0.02	10.07	um02878	-0.30	11.48
1c00021	0.05	10.54	um02879	0.41	10.59
1c00022	0.56	13.18	um02880	0.97	11.49
1c00023	-0.02	9.81	um02881	0.17	11.32
1c00024	-0.09	11.13	um02883	0.03	13.37
1c00025	0.02	12.37	um02884	0.45	12.52
1c00026	0.43	12.17	um02886	0.36	10.88
1c00027	-0.39	12.41	um02887	0.70	11.59
1c00028	-0.57	12.81	um02888	0.31	10.60
1c00029	0.08	12.74	um02889	0.26	12.30
1c00030	-0.14	11.94	um02890	-0.13	11.28
1c00031	-0.20	12.16	um02891	-0.26	11.85
1c00032	-0.25	12.29	um02895	-0.04	12.20
1c00033	-0.34	9.44	um02896	0.28	11.65
1c00034	-0.18	9.35	um02899	0.68	14.56
1c00035	0.95	12.77	um02900	0.40	12.67
1c00036	0.39	14.60	um02901	0.20	9.18
1c00037	0.07	13.08	um02902	0.15	11.31
1c00038	-0.52	11.89	um02903	0.71	11.72
1c00039	-0.34	12.45	um02904	-0.03	11.40
1c00040	-0.02	13.52	um02905	-0.66	13.26
1c00041	1.37	11.90	um02910	0.25	11.80
1c00042	0.04	9.56	um02911	0.24	12.28
1c00043	-0.37	12.84	um02912	-0.43	12.29
1c00044	-0.12	10.44	um02913	0.01	10.75
1c00045	0.18	12.58	um02915	-1.01	10.66
1c00046	-0.06	11.61	um02917	-1.99	14.46
1c00047	0.70	12.99	um02919	0.05	11.54
1c00048	-0.26	9.56	um02920	0.52	11.56
1c00049	0.04	10.03	um02921	1.12	11.20
1c00050	0.10	10.62	um02922	-0.92	12.09
1c00051	-0.36	13.33	um02923	-2.35	11.88
1c00052	-0.22	11.85	um02924	0.21	14.57
1d00001	0.17	9.11	um02925	-0.07	9.74
1d00002	0.44	11.84	um02926	-0.06	13.88
1d00003	-1.27	11.73	um02927	-0.14	8.86
1d00004	0.24	10.50	um02929	-0.58	12.76
1d00005	0.12	14.15	um02933	0.72	11.20
1d00006	0.15	8.78	um02935	-0.99	10.21
1d00007	0.84	10.41	um02936	-0.11	11.20
1d00008	0.04	10.39	um02937	0.20	11.78
1d00009	-0.21	11.36	um02938	0.29	11.22
1d00010	-0.39	11.55	um02942	-1.01	11.80
1d00011	-0.13	12.16	um02944	0.15	11.53
1d00012	0.20	10.94	um02945	-0.37	11.45
1d00013	0.28	14.22	um02948	0.44	11.51

1d00014	0.33	13.45	um02950	0.92	11.79
1d00015	-0.24	12.65	um02951	0.18	10.65
1d00016	0.46	12.14	um02954	-1.04	10.82
1d00017	1.08	14.21	um02955	0.36	14.44
1d00018	0.20	12.07	um02957	0.49	11.79
1d00019	-0.04	12.21	um02958	0.16	12.07
1d00020	-0.18	11.13	um02959	-0.98	15.45
1d00021	-0.02	10.50	um02960	-0.21	12.95
1d00022	-0.59	11.81	um02961	0.34	11.19
1d00023	0.05	15.02	um02962	-0.33	12.43
1d00024	0.26	11.56	um02966	0.11	10.93
1d00025	-0.11	11.99	um02967	-0.96	14.06
1d00026	0.20	9.77	um02968	-0.04	11.86
1d00027	-0.31	12.18	um02972	-0.49	12.86
1d00028	-0.23	12.76	um02974	1.03	10.75
1d00029	-0.03	13.79	um02976	-0.60	11.90
1d00030	0.04	12.40	um02978	1.14	13.82
1d00031	-0.13	9.79	um02979	0.32	10.79
1d00032	0.25	10.08	um02980	0.93	10.26
1d00033	-0.03	13.30	um02981	-0.41	9.25
1d00034	-0.13	10.11	um02982	-0.11	11.07
1d00035	0.30	12.56	um02983	0.11	10.31
1d00036	-0.13	11.19	um02984	-5.11	11.55
1d00037	-0.21	12.61	um02985	0.22	13.18
1d00038	-0.15	10.37	um02986	1.87	13.02
1d00039	-0.22	14.09	um02989	0.19	12.12
1d00040	-1.75	14.97	um02990	0.34	11.96
1d00041	0.87	13.31	um02993	-0.31	11.20
1d00042	0.07	8.64	um02994	-1.18	11.74
1d00043	0.10	8.72	um02998	-0.43	11.95
1d00044	0.14	11.43	um02999	0.06	13.74
1d00045	0.03	8.66	um03000	0.32	11.98
1d00046	-0.09	10.22	um03001	0.24	11.31
1d00047	-0.16	11.99	um03002	1.18	14.32
1d00048	0.64	10.07	um03003	0.21	13.04
1d00049	0.88	11.84	um03006	-0.69	14.47
1d00050	0.11	9.76	um03007	0.50	12.09
1d00051	0.04	12.65	um03009	-0.11	14.08
20c0000	-0.19	10.50	um03010	-0.22	12.34
20c0000	-0.22	10.91	um03011	-0.30	10.93
20c0000	-0.70	9.53	um03013	0.53	11.58
20c0000	0.03	9.71	um03015	0.99	12.07
20c0000	0.08	11.49	um03016	1.27	11.92
20c0000	0.28	10.52	um03017	-0.29	12.61
20c0000	0.08	13.35	um03018	0.03	11.45
20c0000	-0.31	14.31	um03019	-0.12	11.51
20c0000	-0.02	12.17	um03020	0.47	10.60
20c0001	-0.42	11.59	um03023	0.56	10.24
20c0001	-0.41	14.03	um03024	0.28	9.08
20c0001	-0.18	9.95	um03025	-0.63	11.13
20c0001	-0.44	12.14	um03027	-0.26	10.50
20c0001	-0.01	9.01	um03028	-0.20	11.77
20c0001	-0.56	13.42	um03029	-0.48	12.23
20c0001	0.50	10.75	um03032	1.39	14.39

20c0001	0.32	11.48	um0303	0.36	11.78
20c0001	0.00	9.47	um0303	1.21	14.44
20c0001	-0.01	9.39	um0303	-0.43	10.76
20c0002	0.11	8.68	um0303	-0.13	10.56
20c0002	0.44	11.78	um0303	-0.36	11.85
20c0002	0.19	9.85	um0303	0.09	11.44
20c0002	0.52	13.81	um0303	0.02	9.04
20c0002	0.32	10.43	um0304	2.42	12.60
20c0002	0.17	9.68	um0304	0.21	11.53
20c0002	-0.74	14.02	um0304	-0.19	11.63
20c0002	-0.06	10.56	um0304	-0.15	12.80
20c0002	-0.02	11.36	um0304	-0.19	8.60
20c0002	0.49	12.14	um0304	0.22	10.56
20c0003	-0.34	12.53	um0304	0.42	9.57
20c0003	-0.04	8.65	um0305	0.18	11.65
20c0003	-0.35	13.55	um0305	0.24	11.71
20c0003	-0.08	13.20	um0305	-0.12	11.48
20c0003	-0.63	13.53	um0305	0.26	11.83
20c0003	-0.29	9.35	um0306	0.59	11.84
20c0003	-0.14	9.44	um0306	0.05	11.01
20c0003	-0.15	9.97	um0306	1.26	11.02
20c0003	-0.02	8.75	um0306	-0.12	11.19
20c0003	-0.19	11.91	um0306	-0.50	8.82
20c0004	0.36	12.79	um0306	0.52	9.54
20c0004	0.54	13.43	um0306	-1.48	9.88
20c0004	0.13	9.37	um0306	1.05	13.27
20c0004	0.28	12.34	um0306	1.03	15.08
20c0004	0.19	12.05	um0307	-0.16	11.15
20c0004	-0.35	12.12	um0307	-0.13	13.23
20c0004	0.01	15.74	um0307	-0.41	11.32
20c0004	0.04	12.70	um0307	0.23	14.42
20c0004	-0.23	11.73	um0307	0.52	10.63
20c0004	0.08	11.79	um0307	0.59	12.50
20c0005	-0.25	12.82	um0307	-0.24	11.33
20c0005	-0.27	11.66	um0308	0.12	13.97
20c0005	-0.60	11.62	um0308	-0.55	11.30
20c0005	0.32	10.97	um0308	-0.45	11.85
20c0005	0.74	14.45	um0308	-0.49	14.64
20c0005	0.65	14.30	um0308	-0.53	15.22
20c0005	-0.10	11.70	um0308	-0.73	12.51
20c0005	-0.04	13.44	um0308	0.05	11.35
20c0005	0.16	10.27	um0309	-0.32	8.49
20c0005	-0.08	12.77	um0309	-0.13	8.56
20c0006	-0.62	12.62	um0309	-0.26	9.00
20c0006	0.32	10.36	um0309	-0.67	10.97
20c0006	0.19	8.80	um0309	-0.32	11.63
20c0006	-0.63	12.89	um0309	0.35	11.38
20c0006	0.36	12.60	um0309	0.31	11.01
20c0006	-0.16	10.05	um0309	0.26	11.55
20c0006	0.81	11.90	um0310	-0.30	13.46
20c0006	-0.39	11.58	um0310	-0.21	11.38
20c0006	0.05	12.87	um0310	-0.74	14.65
20c0006	-0.23	10.66	um0310	0.87	10.56
20c0007	-0.07	8.87	um0310	-0.61	12.29

20c0007	-0.12	10.77	um03110	-6.11	12.45
20c0007	0.35	12.65	um03112	-0.24	8.77
20c0007	-0.05	9.44	um03113	-0.53	11.68
20c0007	-0.26	9.73	um03114	-1.19	11.02
20c0007	0.24	11.34	um03115	-0.33	14.07
20c0007	-0.18	10.08	um03116	-1.97	13.18
20c0007	-0.03	14.13	um03117	-2.23	11.53
20c0007	0.28	11.61	um03120	-1.00	9.43
20c0007	-2.00	12.14	um03121	-0.09	9.41
20c0008	-1.06	14.58	um03122	-0.40	9.65
20c0008	-1.63	12.70	um03124	0.63	10.24
20d0000	-0.29	12.20	um03126	0.67	12.58
20d0000	0.10	10.44	um03127	0.33	11.72
20d0000	-0.02	15.93	um03128	0.17	10.54
20d0000	0.17	10.26	um03129	0.85	10.62
20d0000	-0.17	12.70	um03132	-0.23	11.92
20d0000	0.15	11.58	um03134	-0.03	11.53
20d0000	-0.24	12.43	um03135	3.30	11.11
20d0000	-1.20	12.36	um03136	0.35	10.93
20d0000	0.14	15.29	um03138	-0.06	9.80
20d0001	-1.01	10.04	um03139	1.89	11.90
20d0001	0.21	12.59	um03140	0.56	11.71
20d0001	0.56	11.67	um03141	0.36	10.80
20d0001	-0.41	14.98	um03143	0.21	11.89
20d0001	-0.16	8.70	um03144	0.70	12.29
20d0001	0.14	8.80	um03145	-0.12	11.63
20d0001	-0.84	11.72	um03146	-0.09	10.86
20d0001	0.03	11.49	um03147	0.00	11.05
20d0001	-0.06	14.47	um03148	-0.27	8.72
20d0001	0.00	8.58	um03149	-1.19	9.44
20d0002	0.52	12.74	um03150	-0.23	11.96
20d0002	0.25	12.55	um03152	-1.02	12.07
20d0002	0.31	15.12	um03153	-0.29	12.52
20d0002	0.65	11.96	um03154	-0.28	8.92
20d0002	-0.22	9.31	um03156	-0.37	13.33
20d0002	0.46	11.47	um03158	2.36	12.04
20d0002	-0.46	12.00	um03162	-0.26	12.91
20d0002	0.36	10.99	um03164	0.54	11.73
20d0002	-0.03	12.54	um03165	0.64	12.48
20d0002	0.28	10.95	um03166	0.71	12.04
20d0003	0.77	13.54	um03167	0.27	12.51
20d0003	0.69	10.56	um03168	1.07	12.10
20d0003	-0.61	11.71	um03169	3.28	12.69
20d0003	-1.16	12.19	um03170	-0.14	10.93
20d0003	0.38	10.12	um03171	0.02	13.60
20d0003	-0.16	15.32	um03172	-0.27	9.68
20d0003	-0.31	13.91	um03175	-0.10	11.52
20d0003	0.37	12.97	um03177	3.39	13.38
20d0003	-1.06	13.81	um03178	0.11	11.32
20d0003	-0.05	15.15	um03180	0.51	12.75
20d0004	-0.13	10.47	um03182	-0.38	14.16
20d0004	-0.44	14.12	um03184	0.51	12.06
20d0004	0.21	14.08	um03192	-0.04	13.37
20d0004	-0.92	11.50	um03194	-0.22	12.19

20d0004	-0.31	12.50	um03196	0.21	9.57
20d0004	0.04	12.55	um03199	-1.00	14.41
20d0004	0.50	11.99	um03200	-0.52	10.99
20d0004	0.26	11.94	um03201	-0.16	8.71
20d0004	-0.09	13.79	um03202	-0.17	8.50
20d0004	0.33	13.53	um03204	-0.84	12.30
20d0005	-0.16	11.75	um03206	-0.38	11.30
20d0005	-0.83	11.95	um03207	0.12	12.37
20d0005	0.02	10.17	um03209	-0.49	13.38
20d0005	-0.10	14.52	um03210	-0.74	13.86
20d0005	-0.01	12.02	um03211	0.85	11.21
20d0005	0.27	12.18	um03216	-0.14	12.37
20d0005	-0.17	13.49	um03218	-0.01	13.21
20d0005	-0.21	12.86	um03220	0.83	13.53
20d0005	0.16	11.68	um03221	-0.91	12.13
20d0005	-0.73	13.92	um03223	-0.48	8.71
20d0006	-0.08	11.36	um03226	-0.08	10.95
20d0006	0.06	11.74	um03227	-0.07	12.99
20d0006	0.35	12.11	um03228	1.55	11.14
20d0006	0.10	8.66	um03229	1.05	12.96
20d0006	0.03	9.04	um03231	0.01	9.67
20d0006	-0.45	14.52	um03232	0.57	11.23
20d0006	0.02	9.79	um03233	0.18	12.69
20d0006	0.97	11.28	um03234	-0.55	11.08
20d0006	-0.42	11.68	um03235	-0.06	11.54
20d0006	1.08	10.89	um03237	0.06	15.28
20d0007	0.12	12.45	um03238	-0.26	11.00
20d0007	0.15	11.36	um03239	-0.13	12.88
20d0007	-0.05	9.32	um03240	0.16	12.57
20d0007	0.58	14.43	um03241	-0.34	10.80
20d0007	0.29	12.14	um03242	0.31	10.47
20d0007	0.11	8.84	um03244	-0.06	10.05
20d0007	0.63	11.12	um03246	1.06	14.31
20d0007	0.16	10.43	um03249	0.51	10.73
20d0007	-0.02	9.01	um03250	-0.03	12.51
20d0007	-0.95	12.85	um03251	0.24	10.49
20d0008	0.40	10.22	um03252	-0.20	13.17
20d0008	0.14	11.17	um03253	0.61	10.91
20d0008	-0.39	12.48	um03254	0.34	12.12
20d0008	0.28	11.40	um03256	0.69	12.09
20d0008	-1.84	10.81	um03260	0.23	11.88
20d0008	-1.46	13.24	um03261	-0.17	11.11
21c0000	-0.77	12.49	um03262	-0.65	10.27
21c0000	-0.30	9.09	um03263	0.27	11.69
21c0000	-0.17	8.99	um03264	-0.19	11.93
21c0000	-0.45	12.20	um03265	-0.08	14.05
21c0000	-0.63	13.55	um03266	-0.40	11.21
21c0000	-0.30	15.82	um03267	0.19	12.16
21d0000	-0.03	10.08	um03268	0.32	10.62
21d0000	-0.02	8.61	um03271	0.10	10.76
21d0000	-0.74	14.95	um03273	-0.09	11.51
21d0000	-0.17	12.40	um03274	-0.22	8.91
22c0000	0.10	8.49	um03276	0.36	11.33
22c0000	0.04	11.14	um03277	0.09	11.50

22c0000	0.12	11.61	um0327	0.62	12.51
22c0000	-0.09	11.06	um0328	0.08	12.70
22c0000	0.03	11.03	um03281	-1.03	10.83
22c0000	0.54	13.72	um0328	-0.57	11.89
22c0000	0.28	10.70	um0328	-0.40	12.86
22c0000	0.13	11.86	um0328	-0.80	15.29
22c0000	-0.54	11.55	um0328	0.12	11.16
22c0001	0.15	12.67	um0328	-0.56	11.70
22c0001	-0.05	9.19	um0328	-0.25	12.21
22c0001	0.73	11.33	um0328	0.98	12.34
22c0001	0.70	13.11	um0329	-0.88	11.35
22c0001	0.33	12.93	um0329	0.59	11.59
22c0001	-0.68	13.41	um0329	0.23	12.68
22c0001	0.73	11.49	um0329	-0.32	11.28
22c0001	0.02	9.02	um0329	1.04	10.72
22c0001	-0.68	12.50	um0329	0.32	9.06
22c0001	0.02	10.29	um0329	0.48	13.91
22c0002	0.50	13.30	um0329	-0.83	14.61
22c0002	-0.03	9.06	um03301	-0.36	11.82
22c0002	0.09	11.46	um0330	0.38	10.97
22c0002	0.35	11.64	um0330	-0.61	15.02
22c0002	-2.71	10.96	um0330	0.31	12.99
22c0002	-0.09	13.25	um0330	0.05	12.01
22c0002	0.50	13.56	um0330	-0.10	10.95
22c0002	-0.27	13.00	um0330	-0.53	13.25
22c0002	0.19	12.52	um0330	0.24	13.00
22c0002	-0.12	10.84	um0331	2.46	11.85
22c0003	0.28	12.26	um0331	0.12	11.91
22c0003	-0.13	10.02	um0331	0.35	8.94
22c0003	0.10	12.31	um0331	0.03	9.19
22c0003	-0.60	10.72	um0331	-0.69	12.59
22c0003	0.52	10.49	um0331	0.33	9.83
22c0003	-1.01	13.44	um0331	-0.21	12.28
22c0003	0.03	12.15	um0332	-0.24	11.70
22c0003	0.09	13.34	um0332	-1.65	12.39
22c0003	0.30	11.97	um0332	-0.52	9.91
22c0003	0.24	12.23	um0333	0.30	12.60
22c0004	0.03	11.70	um03331	-0.23	11.40
22c0004	0.38	9.86	um0333	0.35	10.73
22c0004	0.16	12.43	um0333	-0.39	11.89
22c0004	-0.48	12.32	um0333	-0.40	12.69
22c0004	0.08	11.83	um0333	-0.15	12.44
22c0004	0.06	8.74	um0334	0.36	12.97
22c0004	-0.07	10.42	um03341	-0.49	11.60
22c0004	-0.01	10.72	um0334	0.55	11.08
22c0004	0.12	10.51	um0334	0.13	12.48
22c0004	-0.15	8.73	um0334	0.13	11.23
22c0005	0.00	8.84	um0334	-0.80	12.67
22c0005	0.22	11.97	um0334	-0.04	12.95
22c0005	0.07	12.52	um0334	-0.65	15.61
22c0005	0.26	13.16	um03351	0.80	12.53
22c0005	-0.33	11.32	um0335	-0.37	12.42
22c0005	0.00	12.51	um0335	0.19	11.04
22c0005	0.17	12.83	um0335	0.58	12.98

22c0005	0.25	13.26	um03355	-0.02	12.58
22c0005	-0.24	12.82	um03356	-1.06	15.53
22c0005	-0.03	10.68	um03358	-0.39	11.58
22c0006	0.04	11.28	um03361	-0.03	12.72
22c0006	-0.36	10.60	um03362	0.50	12.32
22c0006	0.03	10.93	um03363	-0.10	10.00
22c0006	0.34	11.42	um03365	0.48	12.99
22c0006	-0.41	11.37	um03366	0.09	10.64
22c0006	-0.34	12.12	um03368	0.21	11.29
22c0006	0.37	10.44	um03371	0.47	11.39
22c0006	0.54	13.61	um03372	1.77	12.13
22c0006	0.01	12.02	um03373	0.62	12.11
22c0006	-0.48	11.52	um03375	-0.54	10.10
22c0007	-0.14	11.63	um03376	-0.65	10.41
22c0007	0.00	9.99	um03377	-0.49	11.80
22c0007	-0.07	12.76	um03379	0.62	10.31
22c0007	-0.53	12.86	um03381	-4.29	12.52
22c0007	-0.18	11.74	um03382	-0.85	10.03
22c0007	-0.40	11.32	um03383	-0.01	12.56
22c0007	0.51	11.84	um03384	0.99	11.17
22c0007	0.70	11.84	um03386	-0.40	9.66
22c0007	-0.03	9.06	um03387	-0.15	8.53
22c0007	-0.98	12.71	um03388	-0.26	9.41
22c0008	-0.66	13.23	um03389	-0.10	8.75
22c0008	-0.04	9.13	um03392	-0.77	9.30
22c0008	-0.16	10.14	um03393	-0.15	8.52
22c0008	-0.25	11.32	um03394	0.76	11.12
22c0008	0.25	11.61	um03395	0.41	9.53
22c0008	0.42	10.43	um03396	0.75	10.65
22c0008	0.13	10.06	um03397	2.26	11.15
22c0008	0.29	13.40	um03398	2.49	14.28
22c0008	1.51	14.52	um03400	1.72	12.28
22c0008	0.01	9.63	um03402	3.66	13.25
22c0009	-0.29	12.48	um03403	0.36	11.30
22c0009	-0.47	11.87	um03404	0.52	9.03
22c0009	0.00	8.89	um03406	0.23	9.04
22c0009	-0.04	8.82	um03407	0.08	8.87
22c0009	0.08	11.65	um03408	1.10	10.56
22c0009	0.29	12.49	um03409	0.15	8.91
22c0009	-0.26	10.69	um03411	0.08	9.91
22c0009	-0.24	11.93	um03412	-0.65	13.14
22c0009	0.23	15.68	um03413	0.41	11.06
22c0009	-0.12	9.69	um03414	-0.56	9.44
22c0010	-0.10	13.03	um03415	2.01	10.75
22c0010	-0.20	11.81	um03416	0.63	10.95
22c0010	-0.23	15.23	um03417	-1.13	11.28
22c0010	-0.22	11.65	um03418	-0.77	12.96
22c0010	0.09	9.38	um03419	-0.18	11.37
22c0010	-0.29	11.63	um03421	-0.11	11.50
22c0010	0.28	14.29	um03422	0.19	11.85
22c0010	-0.17	9.17	um03424	-0.34	12.70
22c0010	0.06	9.07	um03425	-0.38	12.25
22c0010	0.01	13.37	um03431	0.02	12.59
22c0011	0.17	12.60	um03433	-0.04	12.09

22c0011	-0.66	11.91	um03434	0.13	11.02
22c0011	0.32	11.57	um03435	-0.03	10.98
22c0011	-0.36	10.18	um03436	0.73	13.77
22c0011	0.14	12.35	um03437	0.27	11.54
22c0011	-0.11	13.01	um03439	-0.20	8.74
22c0011	0.07	14.04	um03440	-0.92	9.45
22c0011	0.11	14.08	um03442	0.11	11.74
22c0011	0.35	12.87	um03443	-0.39	13.48
22c0011	-0.14	11.14	um03445	-0.34	15.67
22c0012	0.00	8.72	um03446	-0.46	11.82
22c0012	-1.08	12.72	um03448	0.30	11.79
22c0012	-1.03	12.42	um03449	-0.07	14.66
22c0012	-0.16	9.83	um03450	-0.02	11.96
22c0012	0.05	12.52	um03451	2.21	13.54
22c0012	0.08	10.98	um03454	0.01	11.62
22c0012	-0.34	13.14	um03456	-0.24	13.31
22c0012	-0.04	8.65	um03458	-0.56	11.84
22c0012	0.19	13.14	um03459	-0.17	11.74
22c0012	-0.40	11.74	um03460	0.38	12.57
22c0013	0.32	12.74	um03461	0.33	11.73
22c0013	-0.02	12.12	um03462	-0.41	8.71
22c0013	-0.44	10.96	um03463	0.03	12.11
22c0013	0.02	11.91	um03464	0.25	11.21
22c0013	0.37	12.85	um03465	-0.39	12.03
22c0013	0.00	8.63	um03466	0.46	11.52
22c0013	0.01	8.51	um03467	0.24	12.36
22c0013	0.48	11.39	um03468	-0.90	13.24
22c0013	-0.30	10.53	um03469	-0.33	12.20
22c0013	0.56	11.18	um03470	-1.53	12.39
22c0014	0.19	9.39	um03471	0.08	11.38
22c0014	-0.14	8.95	um03472	-0.34	10.11
22c0014	-0.09	13.08	um03473	-0.47	11.43
22c0014	-0.26	11.48	um03474	1.42	11.46
22c0014	0.07	10.74	um03475	-4.01	10.91
22c0014	-0.10	8.83	um03476	-0.34	11.87
22c0014	0.14	8.57	um03477	-0.19	12.51
22c0014	-0.01	8.63	um03478	0.29	11.98
22c0014	-0.08	8.85	um03480	0.63	12.54
22c0014	0.63	11.99	um03481	0.13	11.29
22c0015	0.04	11.04	um03483	-0.18	8.80
22c0015	-0.40	9.52	um03485	2.82	12.21
22c0015	-0.44	12.79	um03486	0.76	12.13
22c0015	-0.84	14.29	um03487	0.62	11.64
22c0015	-0.18	9.87	um03491	0.07	12.31
22c0015	-0.17	11.18	um03493	0.29	12.00
22c0015	-0.05	9.59	um03494	0.12	13.48
22c0015	-0.27	9.85	um03495	-0.33	11.69
22c0015	-0.17	13.24	um03496	-0.56	10.45
22c0015	-0.30	11.89	um03497	-0.65	10.21
22c0016	0.04	11.05	um03498	-0.24	11.86
22c0016	-0.18	10.70	um03501	-0.65	11.28
22c0016	0.18	9.49	um03504	-0.55	11.80
22c0016	-0.04	11.04	um03505	-0.04	10.64
22c0016	-0.57	13.90	um03506	1.94	12.37

22c0016	-0.20	12.04
22c0016	0.79	12.42
22c0016	-0.36	11.64
22c0016	-0.13	11.35
22c0016	-0.17	11.55
22c0017	-0.35	14.95
22c0017	0.33	14.31
22c0017	0.05	12.80
22c0017	-0.56	12.72
22c0017	0.17	10.64
22c0017	-0.12	12.66
22c0017	0.31	11.81
22c0017	-0.15	11.20
22c0017	-0.25	11.77
22c0017	0.32	9.88
22c0018	-0.07	10.15
22c0018	0.06	9.31
22c0018	-0.06	8.87
22c0018	0.53	12.29
22c0018	-0.33	11.92
22c0018	-0.14	11.37
22c0018	0.38	10.12
22c0018	-0.12	11.16
22c0018	-0.10	12.35
22c0018	0.06	8.88
22c0019	0.09	9.36
22c0019	0.44	12.04
22c0019	-0.60	12.30
22c0019	0.33	13.55
22c0019	-0.49	10.98
22c0019	0.20	10.90
22c0019	0.70	13.31
22c0019	-0.18	12.73
22c0019	0.06	13.20
22c0019	0.21	12.50
22c0020	-0.33	15.72
22c0020	-0.26	11.96
22c0020	0.01	9.93
22c0020	0.57	13.05
22c0020	0.37	11.91
22c0020	-0.10	11.27
22c0020	-0.10	10.50
22c0020	-0.07	10.37
22c0020	-2.64	13.28
22c0020	-0.29	12.21
22c0021	0.05	9.13
22c0021	-0.15	13.48
22c0021	-0.59	12.75
22c0021	-0.25	8.59
22c0021	-0.94	12.33
22c0021	-0.54	10.44
22c0021	0.00	12.22
22c0021	-0.08	11.29
22c0021	0.26	11.81

um03507	1.93	12.22
um03508	1.55	11.60
um03511	0.05	14.60
um03513	-0.67	13.14
um03514	-0.18	10.89
um03515	-0.10	12.29
um03517	-0.06	12.32
um03518	-0.15	12.06
um03520	-0.19	10.40
um03521	0.81	11.02
um03522	-1.47	13.42
um03523	-2.11	11.43
um03524	-0.76	10.38
um03525	0.28	11.45
um03528	-0.06	11.74
um03529	-0.37	11.63
um03530	0.40	12.19
um03532	0.02	11.25
um03533	-0.08	14.68
um03534	0.19	11.39
um03535	0.03	8.87
um03536	0.14	11.57
um03537	-0.41	13.02
um03538	-0.24	12.28
um03539	-0.42	14.47
um03540	0.03	12.00
um03541	0.06	8.90
um03542	-0.13	10.19
um03543	0.71	10.96
um03544	0.09	11.36
um03546	-0.16	8.63
um03547	0.55	10.04
um03548	1.41	12.01
um03549	0.09	11.09
um03550	0.29	11.91
um03551	-0.41	9.87
um03552	-0.46	12.16
um03553	0.19	10.02
um03554	-0.07	10.96
um03555	-0.09	11.04
um03556	2.46	12.25
um03557	0.51	11.15
um03558	-3.70	11.23
um03559	-2.35	9.68
um03560	-3.60	10.28
um03561	-1.81	9.17
um03562	-0.18	8.61
um03563	-2.05	9.60
um03564	-0.86	8.92
um03565	0.36	11.43
um03567	0.15	10.75
um03568	1.75	11.13
um03569	-0.08	9.64
um03570	0.39	11.55

22c0021	0.08	8.71	um03571	-0.38	14.44
22c0022	-0.24	9.93	um03572	0.05	10.07
22c0022	-0.02	11.97	um03573	0.54	10.74
22c0022	0.56	12.63	um03574	-0.35	12.90
22c0022	0.01	13.87	um03575	-0.14	11.29
22c0022	-0.29	9.29	um03576	-1.91	11.54
22c0022	-0.43	9.82	um03580	0.03	11.94
22c0022	0.72	13.05	um03583	0.42	11.70
22c0022	-0.10	9.04	um03584	0.27	11.55
22c0022	-0.41	10.99	um03585	-3.59	12.94
22c0022	-0.15	10.94	um03586	0.26	8.98
22c0023	0.08	9.18	um03588	0.22	12.37
22c0023	-0.41	11.43	um03590	0.36	11.86
22c0023	-0.36	9.65	um03593	0.00	12.94
22c0023	-0.09	11.26	um03595	0.02	12.56
22c0023	-0.47	13.84	um03596	0.30	12.00
22c0023	0.03	11.40	um03597	-0.52	12.79
22c0023	0.10	10.66	um03598	0.17	12.59
22c0023	0.37	13.58	um03599	-0.22	14.75
22c0023	-1.15	12.96	um03601	0.74	12.60
22c0023	-0.14	12.46	um03602	-0.39	12.93
22c0024	-0.21	13.15	um03603	-0.31	10.90
22c0024	-0.20	12.11	um03604	-0.16	8.51
22c0024	-0.50	11.88	um03605	-0.96	9.69
22c0024	-0.72	13.95	um03607	-0.38	11.39
22c0024	0.63	14.13	um03608	0.09	11.53
22c0024	0.47	11.21	um03609	0.11	11.37
22c0024	-0.14	8.63	um03610	-0.50	11.71
22c0024	-0.08	8.74	um03611	-0.50	11.61
22c0024	0.55	12.03	um03612	0.11	8.91
22c0024	0.51	12.51	um03613	-0.41	11.67
22c0025	-0.46	10.28	um03614	-2.59	11.37
22c0025	-0.04	11.36	um03615	-2.05	13.65
22c0025	0.78	13.15	um03616	-0.55	11.77
22c0025	-0.13	10.43	um03617	-0.30	10.97
22c0025	0.48	11.53	um03618	-0.08	11.05
22c0025	0.52	11.96	um03619	-0.52	8.84
22c0025	-0.14	10.89	um03620	1.57	12.31
22c0025	0.28	10.68	um03621	0.01	11.14
22c0025	-0.56	14.35	um03622	-0.67	13.44
22c0025	-0.07	12.72	um03623	0.81	11.75
22c0026	1.50	15.08	um03624	-0.25	11.50
22c0026	0.25	12.73	um03626	0.07	11.01
22c0026	-0.04	11.13	um03627	-0.09	12.16
22c0026	-0.73	12.33	um03628	0.32	12.19
22c0026	-0.05	8.79	um03629	0.05	12.80
22c0026	0.46	12.44	um03630	0.61	10.18
22c0026	-0.24	12.36	um03631	-0.41	11.66
22c0026	-0.10	12.29	um03632	-0.35	12.41
22c0026	-0.11	12.47	um03635	0.02	11.16
22c0026	0.14	12.36	um03636	-0.18	9.57
22c0027	-0.03	8.95	um03638	0.48	11.59
22c0027	0.35	13.11	um03640	0.33	13.14
22c0027	0.44	11.44	um03641	0.12	10.97

22c0027	-0.05	13.77	um03642	-0.51	9.20
22c0027	0.14	9.50	um03643	-0.11	8.80
22c0027	-0.04	11.94	um03644	-0.27	8.47
22c0027	0.14	12.02	um03645	-0.50	8.85
22c0027	-0.19	12.04	um03647	0.08	13.22
22c0027	-0.76	11.15	um03648	-0.36	11.38
22c0027	0.04	8.66	um03649	0.99	11.37
22c0028	-0.42	12.52	um03650	-0.18	8.85
22c0028	-0.35	10.36	um03652	0.01	12.44
22c0028	-0.49	12.05	um03653	0.25	12.05
22c0028	0.10	10.47	um03654	0.18	12.88
22c0028	0.04	8.75	um03655	-0.03	14.37
22c0028	-0.20	12.41	um03656	0.16	11.04
22c0028	-0.64	10.00	um03657	-0.36	11.85
22c0028	0.58	13.83	um03658	-1.18	11.84
22c0028	0.47	13.73	um03659	1.00	13.20
22c0028	0.80	11.63	um03661	0.52	12.04
22c0029	-0.50	12.64	um03662	-0.29	12.87
22c0029	0.23	12.01	um03663	-1.53	12.70
22c0029	-0.59	11.40	um03664	2.55	12.36
22c0029	-0.61	14.14	um03665	3.17	12.24
22c0029	-0.25	12.63	um03669	-0.37	12.37
22c0029	-0.13	12.96	um03673	-0.39	8.98
22c0029	-0.19	12.63	um03674	0.75	12.34
22c0029	0.05	12.51	um03676	-0.37	9.98
22c0029	-0.05	10.30	um03677	-0.42	8.61
22c0029	0.12	13.18	um03678	1.43	13.93
22c0030	-2.37	12.10	um03680	0.51	12.43
22c0030	-0.07	8.97	um03681	0.05	10.98
22c0030	-0.04	14.59	um03682	0.56	12.04
22c0030	-0.18	9.16	um03685	-0.15	12.02
22c0030	-0.62	12.15	um03687	-0.48	12.06
22c0030	0.42	12.15	um03688	-2.28	10.81
22c0030	0.49	11.56	um03689	-1.81	11.13
22c0030	1.03	11.61	um03690	-2.03	11.36
22c0030	1.41	13.20	um03691	-1.74	11.52
22c0030	-1.18	12.96	um03692	-0.44	10.13
22c0031	-0.52	12.13	um03694	-1.69	11.89
22c0031	-0.22	12.31	um03695	0.15	10.86
22c0031	-0.03	10.37	um03696	0.33	11.75
22c0031	-0.73	11.66	um03698	-0.17	11.45
22c0031	0.02	11.94	um03699	-0.02	13.58
22c0031	-0.60	11.03	um03700	0.07	13.73
22c0031	-0.08	8.78	um03701	-0.13	11.99
22c0031	-0.37	12.82	um03702	-0.03	10.79
22c0031	0.06	10.30	um03704	0.40	15.00
22c0031	-0.46	11.76	um03706	2.14	11.09
22c0032	-1.20	13.52	um03707	0.42	12.42
22c0032	-0.17	8.69	um03708	-0.12	11.43
22d0000	-0.04	8.96	um03713	1.33	9.73
22d0000	0.11	9.15	um03714	0.58	13.22
22d0000	0.01	12.21	um03717	-0.45	12.10
22d0000	-0.07	10.91	um03718	0.19	13.45
22d0000	-0.26	10.67	um03720	-0.24	11.84

22d0000	0.52	12.32	um03722	0.12	11.38
22d0000	-0.22	9.99	um03724	-0.12	12.00
22d0000	0.30	11.90	um03726	-0.65	15.78
22d0000	-0.06	10.23	um03728	-0.41	12.59
22d0001	0.04	8.85	um03729	-0.45	10.60
22d0001	0.16	11.99	um03730	0.08	11.02
22d0001	0.35	12.24	um03734	-1.49	14.64
22d0001	0.61	11.19	um03737	0.57	14.57
22d0001	-0.13	9.43	um03738	-0.04	11.85
22d0001	-0.09	8.53	um03740	-0.71	10.54
22d0001	1.00	10.36	um03743	0.30	11.32
22d0001	0.52	13.57	um03744	-0.26	8.83
22d0001	-0.32	12.71	um03745	-0.33	8.51
22d0001	-0.36	14.25	um03746	-0.20	8.60
22d0002	-0.05	12.17	um03747	-0.11	8.57
22d0002	0.13	11.18	um03748	0.01	8.78
22d0002	-0.24	11.16	um03749	1.01	10.10
22d0002	-0.01	10.65	um03750	0.59	9.49
22d0002	0.68	13.76	um03751	-0.33	8.74
22d0002	0.19	10.73	um03752	-0.33	8.56
22d0002	0.12	9.92	um03753	1.16	10.57
22d0002	0.53	13.47	um03754	1.02	10.86
22d0002	0.08	12.42	um03755	0.06	13.77
22d0002	0.02	10.89	um03756	-0.52	10.16
22d0003	-0.05	12.91	um03757	-0.05	8.95
22d0003	-0.12	12.24	um03758	-0.17	11.05
22d0003	0.64	12.46	um03759	0.43	11.31
22d0003	0.88	13.51	um03760	-0.53	12.59
22d0003	0.62	14.11	um03761	-0.87	10.86
22d0003	0.20	13.92	um03762	-0.04	11.42
22d0003	-0.23	14.32	um03763	-0.30	13.42
22d0003	-0.64	11.40	um03764	-0.51	9.38
22d0003	-0.17	10.82	um03765	-0.24	12.40
22d0003	-0.03	15.52	um03766	0.06	10.90
22d0004	0.07	12.80	um03767	0.10	11.63
22d0004	-0.42	14.04	um03770	0.46	12.40
22d0004	-0.29	10.75	um03771	-0.33	12.46
22d0004	-0.58	10.60	um03772	-0.21	12.36
22d0004	-0.44	15.69	um03774	-0.07	11.24
22d0004	-0.09	12.96	um03775	-0.55	13.35
22d0004	-0.26	11.90	um03776	-2.61	10.31
22d0004	0.30	13.84	um03777	-0.18	11.32
22d0004	-0.29	12.29	um03778	-0.05	11.19
22d0004	0.10	14.14	um03779	-0.48	10.92
22d0005	-0.47	12.53	um03782	-0.06	10.98
22d0005	-0.08	8.61	um03783	0.09	11.90
22d0005	0.11	10.84	um03784	-0.10	13.54
22d0005	-0.74	13.09	um03788	0.19	12.21
22d0005	-0.10	12.83	um03789	-0.56	11.73
22d0005	0.21	12.35	um03791	0.07	15.01
22d0005	-0.25	11.80	um03792	-0.65	9.78
22d0005	0.04	12.23	um03793	0.49	11.55
22d0005	-0.18	11.92	um03796	0.35	12.29
22d0005	0.01	11.72	um03797	-0.09	8.73

22d0006	-0.43	12.62	um03798	-0.26	12.23
22d0006	-0.14	12.19	um03801	-0.49	13.44
22d0006	-0.01	12.91	um03802	0.22	10.51
22d0006	-0.65	15.55	um03803	-0.08	11.14
22d0006	-4.08	13.17	um03805	-0.32	11.32
22d0006	-0.60	12.33	um03806	0.86	10.91
22d0006	0.08	12.61	um03807	0.74	13.35
22d0006	0.26	13.11	um03808	-0.46	10.83
22d0006	-0.19	12.51	um03809	-0.17	12.24
22d0006	0.34	12.40	um03810	0.18	11.24
22d0007	-0.77	13.11	um03811	-0.29	14.29
22d0007	0.07	11.41	um03812	-0.23	12.16
22d0007	-0.38	12.63	um03813	-0.22	11.63
22d0007	-0.34	12.45	um03814	-0.17	12.52
22d0007	0.32	11.62	um03815	0.26	11.44
22d0007	0.55	12.35	um03816	0.44	11.69
22d0007	-0.42	11.91	um03817	0.92	11.41
22d0007	0.70	12.54	um03818	0.65	11.56
22d0007	0.00	12.00	um03819	-0.10	11.75
22d0007	-0.19	12.58	um03820	0.27	11.81
22d0008	-0.49	10.82	um03821	-0.24	8.83
22d0008	-0.14	11.02	um03822	0.79	14.38
22d0008	0.09	9.04	um03823	-0.10	8.92
22d0008	-0.19	9.80	um03824	-0.88	13.15
22d0008	0.02	11.47	um03826	-0.15	12.04
22d0008	-0.05	11.67	um03828	-0.16	8.81
22d0008	-0.40	12.85	um03829	0.20	10.16
22d0008	0.24	11.41	um03830	-0.25	11.91
22d0008	-0.76	10.57	um03831	0.33	12.12
22d0008	0.15	13.01	um03832	-0.14	11.42
22d0009	0.19	12.56	um03833	-0.07	14.84
22d0009	-0.59	10.90	um03835	-0.81	11.07
22d0009	0.00	11.71	um03837	-0.28	11.11
22d0009	-0.12	10.67	um03838	-0.95	13.98
22d0009	-2.56	12.78	um03839	-0.01	12.07
22d0009	-0.59	15.32	um03841	-0.23	11.58
22d0009	-0.17	12.68	um03842	-0.63	11.76
22d0009	0.00	8.70	um03843	-0.15	12.05
22d0009	0.34	13.74	um03844	0.34	11.30
22d0009	0.42	12.99	um03845	0.75	14.24
22d0010	-0.34	12.04	um03847	0.09	14.36
22d0010	-0.55	14.08	um03850	0.46	13.78
22d0010	-0.31	15.46	um03851	-0.17	14.62
22d0010	0.35	11.99	um03852	-0.53	10.78
22d0010	-0.44	13.79	um03853	-0.48	11.32
22d0010	0.14	10.93	um03854	-1.02	14.47
22d0010	-0.19	13.77	um03857	-0.02	11.58
22d0010	-0.02	8.54	um03858	-0.45	11.99
22d0010	0.08	10.71	um03859	-0.38	11.81
22d0010	-0.04	13.01	um03860	-1.02	13.92
22d0011	0.26	15.44	um03861	-0.36	11.77
22d0011	-0.34	12.02	um03862	0.08	11.85
22d0011	-0.03	13.93	um03863	-0.27	12.95
22d0011	0.32	13.12	um03864	-0.55	11.94

22d0011	-0.11	9.75	um03865	0.43	13.65
22d0011	0.33	13.51	um03866	0.17	10.94
22d0011	-0.14	9.57	um03867	0.03	11.31
22d0011	-0.04	12.74	um03869	0.60	12.65
22d0011	0.44	11.84	um03871	-0.22	11.20
22d0011	0.55	10.16	um03872	0.39	12.44
22d0012	-0.04	12.98	um03873	0.00	13.14
22d0012	-0.04	10.88	um03874	-0.03	11.00
22d0012	0.56	14.68	um03875	-0.25	12.25
22d0012	-0.32	13.67	um03876	0.13	12.34
22d0012	0.07	11.50	um03877	-0.23	8.99
22d0012	-0.34	11.82	um03880	0.15	12.72
22d0012	0.00	14.16	um03881	1.27	10.72
22d0012	-0.52	13.05	um03882	1.89	10.90
22d0012	0.03	11.03	um03883	-0.05	11.45
22d0012	-0.05	11.31	um03884	-0.61	12.63
22d0013	-0.02	12.50	um03886	-0.12	9.96
22d0013	-0.39	11.88	um03888	1.00	10.84
22d0013	-0.35	12.48	um03890	0.91	11.11
22d0013	-0.81	14.22	um03892	0.36	10.85
22d0013	-0.07	11.88	um03893	0.42	11.59
22d0013	-0.11	9.45	um03897	-0.99	12.67
22d0013	0.33	14.10	um03898	0.06	11.81
22d0013	-0.28	9.10	um03901	-0.21	11.13
22d0013	-1.10	9.53	um03902	-0.62	12.08
22d0013	0.16	12.58	um03905	-0.25	11.30
22d0014	-0.50	12.30	um03906	-0.33	11.00
22d0014	0.13	9.03	um03908	-0.22	9.22
22d0014	-0.21	10.99	um03909	1.91	12.52
22d0014	0.24	12.29	um03910	0.60	14.39
22d0014	-0.31	10.62	um03911	0.61	11.04
22d0014	0.04	9.15	um03914	0.40	13.54
22d0014	-0.64	12.08	um03916	-0.12	15.69
22d0014	-0.30	10.74	um03917	0.53	10.94
22d0014	0.12	10.75	um03918	-0.27	11.38
22d0014	-0.22	12.22	um03919	0.26	13.49
22d0015	0.53	9.18	um03921	-0.83	13.73
22d0015	-0.07	10.41	um03923	0.87	14.94
22d0015	0.21	10.57	um03924	-2.01	15.34
22d0015	0.64	10.95	um03925	0.49	11.84
22d0015	-0.59	12.88	um03927	0.00	10.31
22d0015	-0.46	14.21	um03928	0.12	9.67
22d0015	0.14	9.81	um03929	-0.53	12.50
22d0015	0.29	11.12	um03930	-0.16	11.19
22d0015	-0.24	12.39	um03932	0.39	9.21
22d0015	0.25	12.19	um03933	-0.17	13.69
22d0016	-0.56	13.27	um03935	-1.65	11.46
22d0016	0.12	8.79	um03936	0.17	11.51
22d0016	0.11	9.28	um03937	0.29	12.37
22d0016	-0.53	12.45	um03938	-0.02	14.17
22d0016	-0.24	12.41	um03939	0.08	11.01
22d0016	0.13	9.62	um03941	0.25	10.85
22d0016	0.57	9.26	um03942	-0.90	13.10
22d0016	-0.41	12.64	um03943	-0.04	11.61

22d0016	-0.19	10.94	um03944	-0.33	12.26
22d0016	-0.33	11.21	um03945	-0.59	12.45
22d0017	-0.40	11.63	um03946	0.27	10.99
22d0017	-0.02	10.15	um03947	-0.03	12.51
22d0017	-0.45	11.11	um03948	0.01	10.05
22d0017	-0.34	12.07	um03949	-0.75	11.65
22d0017	-0.01	9.08	um03950	0.01	11.07
22d0017	-0.34	11.41	um03952	0.29	13.05
22d0017	-0.56	12.35	um03953	0.33	11.39
22d0017	-0.17	11.19	um03956	-0.27	13.62
22d0017	-0.97	10.72	um03958	-0.57	13.63
22d0017	-0.12	12.37	um03959	-0.42	13.56
22d0018	-0.56	12.31	um03960	0.49	11.20
22d0018	-0.25	9.41	um03963	-0.42	10.64
22d0018	-0.67	13.11	um03964	0.28	11.31
22d0018	-0.29	12.06	um03965	0.10	12.28
22d0018	-0.74	11.97	um03966	0.22	10.72
22d0018	-0.07	11.50	um03967	-0.21	12.49
22d0018	-0.51	11.27	um03969	0.26	11.83
22d0018	0.06	12.29	um03970	0.00	12.76
22d0018	-0.03	9.00	um03971	0.12	11.41
22d0018	0.51	12.26	um03972	0.18	11.70
22d0019	0.23	10.63	um03973	0.08	12.82
22d0019	-0.45	12.73	um03974	-0.48	12.16
22d0019	-0.81	11.13	um03975	0.06	13.23
22d0019	0.10	13.82	um03976	0.25	12.63
22d0019	0.18	13.43	um03977	-1.41	14.43
22d0019	-0.21	10.11	um03978	2.23	10.55
22d0019	-0.71	11.79	um03979	0.80	11.21
22d0019	0.20	11.94	um03980	-0.02	11.32
22d0019	-0.40	12.07	um03982	0.94	11.53
22d0019	0.12	11.35	um03983	0.53	12.19
22d0020	0.30	13.57	um03984	-0.22	11.33
22d0020	0.02	11.17	um03985	0.24	10.38
22d0020	-0.25	13.29	um03986	-0.21	11.80
22d0020	-1.00	13.04	um03987	-0.23	10.73
22d0020	-0.11	8.91	um03989	0.63	10.98
22d0020	-0.29	11.13	um03990	-1.47	11.52
22d0020	-0.74	11.68	um03991	0.29	11.35
22d0020	0.06	12.03	um03992	-0.32	9.65
22d0020	-0.12	11.11	um03994	0.55	11.81
22d0020	0.36	10.90	um03995	-2.37	11.40
22d0021	-0.40	11.91	um03997	-0.42	11.80
22d0021	0.07	10.78	um04000	1.06	14.31
22d0021	-0.75	12.01	um04004	-0.38	11.23
22d0021	-0.12	12.84	um04005	0.47	12.30
22d0021	-0.41	11.86	um04006	0.17	11.20
22d0021	-0.31	13.80	um04007	0.13	14.46
22d0021	0.23	11.45	um04009	0.17	11.23
22d0021	0.62	13.95	um04013	0.29	11.04
22d0021	-0.17	13.06	um04014	1.66	14.70
22d0021	0.30	10.06	um04015	0.52	11.74
22d0022	-0.35	12.75	um04016	0.05	10.82
22d0022	0.09	9.12	um04017	0.39	11.61

22d0022	0.51	10.78	um04019	-0.56	12.00
22d0022	0.26	15.14	um04020	0.04	12.42
22d0022	-0.42	11.21	um04021	0.23	12.24
22d0022	-0.82	12.65	um04022	0.25	11.44
22d0022	-0.24	13.24	um04026	0.26	10.94
22d0022	0.10	10.08	um04027	0.12	9.44
22d0022	0.10	11.64	um04029	-0.31	12.81
22d0022	0.15	12.08	um04030	0.64	11.20
22d0023	0.12	10.52	um04031	0.20	10.86
22d0023	-0.40	11.26	um04032	0.20	9.27
22d0023	-0.12	15.62	um04033	-0.36	8.62
22d0023	-0.09	10.55	um04034	0.08	9.23
22d0023	0.03	14.33	um04035	0.12	9.83
22d0023	0.23	10.34	um04038	-0.18	8.72
22d0023	1.15	13.15	um04039	-0.05	8.63
22d0023	-0.06	12.16	um04040	0.17	9.69
22d0023	-0.07	9.26	um04042	0.06	11.14
22d0023	0.91	11.93	um04043	-0.42	10.79
22d0024	0.04	11.05	um04044	0.27	13.83
22d0024	0.42	13.12	um04046	0.20	11.91
22d0024	-0.08	10.05	um04047	-0.16	12.06
22d0024	0.07	12.46	um04048	0.44	11.95
22d0024	-0.16	11.52	um04049	0.39	12.03
22d0024	0.08	8.85	um04050	-0.22	12.15
22d0024	0.09	9.52	um04051	0.26	11.58
22d0024	-1.87	12.44	um04053	0.32	11.96
22d0024	0.15	11.29	um04054	0.25	11.39
22d0024	-0.41	15.07	um04055	1.06	11.87
22d0025	0.15	11.81	um04056	1.03	11.19
22d0025	0.13	9.69	um04057	-0.36	13.30
22d0025	-0.12	9.96	um04059	0.45	11.40
22d0025	0.02	11.00	um04060	1.20	14.31
22d0025	-0.08	13.88	um04061	-0.31	11.08
22d0025	0.71	13.59	um04062	0.38	10.78
22d0025	1.06	12.44	um04063	1.22	11.80
22d0025	0.39	13.03	um04064	0.79	11.99
22d0025	-0.20	11.54	um04065	0.01	10.53
22d0025	1.18	13.76	um04066	0.18	11.27
22d0026	-1.27	10.01	um04067	-0.26	11.42
22d0026	-1.12	10.38	um04069	0.23	12.43
22d0026	0.18	11.11	um04070	0.64	12.66
22d0026	-0.27	12.52	um04071	0.09	12.59
22d0026	-0.23	9.98	um04072	0.28	10.89
22d0026	-0.01	12.99	um04074	0.31	11.21
22d0026	-0.33	10.61	um04076	0.61	12.07
22d0026	0.34	12.18	um04079	0.34	12.26
22d0026	-0.03	11.90	um04080	-0.24	12.92
22d0026	0.38	9.06	um04081	-0.23	13.05
22d0027	-0.01	11.48	um04082	-0.24	8.78
22d0027	-1.30	14.97	um04083	-0.01	9.60
22d0027	-0.11	9.38	um04084	-0.12	8.72
22d0027	-0.79	10.60	um04085	-0.13	9.80
22d0027	0.15	9.47	um04086	-0.52	9.11
22d0027	0.01	11.36	um04087	-0.28	9.55

22d0027	-0.46	12.76	um04089	1.24	13.94
22d0027	-0.14	9.79	um04091	-0.65	11.91
22d0027	0.03	10.51	um04092	-0.28	9.98
22d0027	-0.38	12.31	um04093	-0.18	8.47
22d0028	-0.57	11.49	um04094	0.73	12.89
22d0028	-0.10	8.68	um04095	-0.63	9.52
22d0028	-0.82	12.76	um04096	-1.28	9.71
22d0028	0.27	11.93	um04097	-0.48	8.82
22d0028	-0.56	12.55	um04098	-0.15	8.54
22d0028	0.23	14.39	um04100	-0.10	8.62
22d0028	0.86	12.34	um04101	-1.32	9.91
22d0028	0.05	10.48	um04104	-2.30	10.16
22d0028	0.10	13.02	um04105	-1.20	9.52
22d0028	-0.54	11.96	um04106	-2.70	11.26
22d0029	0.39	9.65	um04107	-1.81	10.23
22d0029	0.28	13.47	um04109	-1.19	9.71
22d0029	-0.02	11.47	um04111	-0.06	8.71
22d0029	-0.18	11.79	um04114	-0.97	11.29
22d0029	-0.06	13.17	um04115	-0.45	13.39
22d0029	-0.26	9.62	um04116	-0.31	13.13
22d0029	-0.11	10.15	um04117	0.19	13.33
22d0029	0.10	12.63	um04118	0.12	11.63
22d0029	0.05	11.02	um04119	-0.21	8.57
24c0000	0.20	15.67	um04120	0.09	9.01
24c0000	0.02	15.88	um04123	1.75	12.08
24c0000	0.12	11.02	um04125	0.36	9.21
24c0000	-0.92	11.93	um04126	0.58	11.94
24c0000	-0.43	13.42	um04127	-0.26	12.44
24c0000	0.17	12.61	um04128	-0.37	11.03
24c0000	-0.48	14.36	um04129	0.01	10.27
24c0000	0.02	13.58	um04130	-0.12	9.06
24c0000	-0.79	15.33	um04131	-0.34	10.65
24c0001	0.07	9.41	um04132	0.05	11.15
24c0001	-0.96	13.31	um04133	-0.64	11.91
24c0001	-0.05	14.18	um04134	0.44	12.36
24c0001	0.13	11.35	um04135	0.29	9.87
24c0001	0.15	11.20	um04136	-0.26	13.16
24c0001	-0.07	8.92	um04138	-0.61	15.88
24c0001	0.36	13.08	um04139	0.42	12.34
24c0001	0.14	11.46	um04145	-2.17	12.08
24c0001	-0.02	10.33	um04146	0.17	10.82
24c0001	0.13	12.02	um04147	0.20	11.98
24c0002	0.22	12.07	um04148	0.15	12.12
24c0002	0.10	13.06	um04149	0.60	11.13
24c0002	0.35	12.18	um04150	-0.46	11.05
24c0002	-0.78	11.38	um04151	0.46	12.32
24c0002	0.15	12.22	um04152	-0.27	14.02
24c0002	0.52	14.49	um04154	1.05	11.99
24c0002	0.23	11.94	um04156	-0.02	13.35
24c0002	0.48	11.83	um04157	0.31	10.48
24c0002	-0.09	13.32	um04158	-0.32	10.85
24c0002	0.29	11.86	um04159	0.43	11.82
24c0003	0.23	11.66	um04160	0.29	9.53
24c0003	0.30	11.93	um04161	0.10	10.97

24c0003	0.06	11.26	um04162	-0.48	12.12
24c0003	-0.21	12.84	um04163	0.54	11.02
24c0003	0.09	8.48	um04164	0.25	11.96
24c0003	0.32	10.73	um04165	-1.49	12.37
24c0003	0.10	11.67	um04166	-0.29	11.25
24c0003	0.19	11.00	um04167	0.05	13.82
24c0003	0.20	12.38	um04168	0.22	11.44
24c0003	-0.68	12.43	um04169	-0.50	12.44
24c0004	-0.28	12.03	um04171	0.67	12.30
24c0004	0.33	12.79	um04172	0.00	11.01
24c0004	0.48	12.57	um04173	-0.42	12.45
24c0004	-0.54	12.20	um04174	0.04	10.86
24c0004	-0.12	8.67	um04175	-0.81	13.02
24c0004	-0.08	8.63	um04176	-0.15	12.28
24c0004	-0.04	8.80	um04177	0.34	12.38
24c0004	0.67	13.03	um04179	-0.37	13.64
24c0004	0.28	13.92	um04180	0.21	10.77
24c0004	0.51	13.27	um04181	1.94	14.23
24c0005	0.08	11.33	um04182	-0.30	11.43
24c0005	0.66	13.43	um04183	0.93	11.67
24c0005	-0.46	13.71	um04184	-0.17	11.12
24c0005	-0.53	13.93	um04185	0.15	9.12
24c0005	0.28	13.26	um04186	-0.30	12.31
24c0005	0.51	13.32	um04187	-0.04	11.25
24c0005	-0.31	13.81	um04188	0.04	12.23
24c0005	-0.45	11.80	um04189	-0.52	11.35
24c0005	-0.09	11.58	um04190	-0.73	15.82
24c0005	-0.14	8.68	um04191	0.70	11.14
24c0006	0.07	13.56	um04192	-0.34	12.61
24c0006	-0.52	15.41	um04193	0.74	11.00
24c0006	-0.16	11.68	um04194	0.76	12.11
24c0006	-0.03	9.06	um04196	0.18	13.09
24c0006	0.08	8.82	um04197	-0.20	11.20
24c0006	0.52	10.87	um04198	-0.32	13.18
24c0006	0.16	12.74	um04201	0.27	11.62
24c0006	0.25	12.09	um04202	0.00	11.41
24c0006	1.02	12.08	um04203	0.17	10.87
24c0006	-0.16	9.11	um04205	-0.24	8.56
24c0007	0.42	12.38	um04206	0.39	10.75
24c0007	0.76	13.41	um04208	0.25	13.82
24c0007	-0.19	12.54	um04209	-0.01	11.16
24c0007	0.27	11.36	um04210	-0.40	11.22
24d0000	-0.17	14.17	um04211	0.45	10.95
24d0000	0.32	15.21	um04212	0.58	12.45
24d0000	-0.01	15.96	um04213	0.28	11.85
24d0000	0.01	13.96	um04214	0.27	13.04
24d0000	-0.35	11.34	um04216	0.98	11.45
24d0000	0.47	12.71	um04217	-0.07	11.03
24d0000	-0.37	10.80	um04218	0.04	12.48
24d0000	0.02	8.75	um04219	0.34	11.59
24d0000	-0.22	10.94	um04220	-0.40	12.53
24d0001	-0.24	11.86	um04223	0.09	12.99
24d0001	-0.08	10.17	um04224	-0.80	10.43
24d0001	0.78	12.18	um04225	-0.03	11.57

24d0001	0.71	13.13	um04226	-0.22	12.17
24d0001	-0.31	12.04	um04227	-0.22	12.68
24d0001	0.23	12.36	um04228	-0.15	13.30
24d0001	-0.11	12.25	um04229	0.27	11.45
24d0001	-0.07	12.48	um04230	0.00	11.74
24d0001	0.19	12.51	um04232	0.17	11.30
24d0001	-0.20	14.07	um04234	0.01	9.96
24d0002	0.11	12.17	um04236	0.00	11.70
24d0002	0.88	14.65	um04237	0.29	14.96
24d0002	0.59	10.53	um04238	-0.08	12.04
24d0002	-0.06	9.35	um04239	0.27	13.69
24d0002	-0.02	12.59	um04241	-0.05	10.31
24d0002	0.13	12.46	um04242	-0.13	11.85
24d0002	0.05	12.38	um04244	0.62	12.36
24d0002	0.16	10.32	um04247	1.84	12.18
24d0002	0.16	12.35	um04248	-0.06	8.84
24d0002	0.08	12.95	um04249	0.05	12.09
24d0003	0.26	11.63	um04250	-0.02	11.69
24d0003	0.34	11.75	um04251	0.14	11.39
24d0003	-0.23	11.95	um04253	-0.70	12.18
24d0003	-0.28	11.05	um04254	0.31	11.85
24d0003	0.68	11.13	um04257	-0.19	8.65
24d0003	0.12	12.87	um04258	-0.17	11.65
24d0003	-0.21	12.18	um04259	-0.39	11.97
24d0003	-0.13	15.81	um04260	0.16	10.79
24d0003	-0.45	15.49	um04262	-0.42	12.92
24d0003	-0.16	9.55	um04265	0.40	10.83
24d0004	0.01	10.95	um04268	-0.63	13.05
24d0004	-0.05	9.29	um04269	0.35	11.56
24d0004	0.23	12.99	um04270	-0.72	12.67
24d0004	-0.06	8.66	um04271	-0.21	12.43
24d0004	0.31	11.96	um04275	0.27	11.53
24d0004	-0.01	11.22	um04277	0.59	13.71
24d0004	0.38	13.93	um04278	-0.11	11.61
24d0004	-0.15	12.20	um04282	-0.39	10.93
24d0004	-0.33	12.94	um04283	-0.33	12.09
24d0004	0.13	8.68	um04284	0.64	11.98
24d0005	-0.04	13.07	um04285	0.38	14.71
24d0005	0.76	13.12	um04286	-0.52	9.28
24d0005	1.32	14.96	um04288	-0.77	12.17
24d0005	-0.25	12.91	um04290	0.48	11.33
24d0005	2.46	10.82	um04291	-0.30	10.39
24d0005	0.01	12.05	um04292	-0.35	12.06
24d0005	-0.05	13.12	um04293	-0.04	10.58
24d0005	0.24	12.61	um04294	-0.06	14.94
24d0005	-0.30	12.94	um04297	0.01	12.19
24d0005	0.15	13.23	um04298	0.14	11.97
24d0006	-0.32	11.99	um04299	-0.27	12.27
24d0006	-0.11	8.36	um04300	0.39	11.22
24d0006	-0.32	11.44	um04301	0.12	10.64
24d0006	0.06	12.41	um04304	2.04	9.99
25c0000	-0.05	8.57	um04305	0.51	9.03
25c0000	-0.90	11.90	um04306	1.19	13.99
25c0000	0.56	11.85	um04307	0.64	11.40

25c0000	-0.58	12.33	um04308	0.81	11.62
25c0000	0.71	13.40	um04309	3.90	12.33
25c0000	-0.45	11.71	um04310	0.38	10.85
25c0000	0.15	12.42	um04312	-0.40	10.72
25c0000	-0.05	11.41	um04313	0.06	11.52
25c0000	-0.18	11.67	um04314	-0.36	15.18
25c0001	-0.40	12.66	um04315	-0.08	8.54
25c0001	0.18	12.37	um04316	-0.29	8.83
25c0001	-0.50	12.14	um04317	0.39	12.44
25c0001	-0.42	13.32	um04318	0.25	13.33
25c0001	-0.57	12.12	um04319	0.24	12.69
25c0001	-0.91	9.35	um04320	-0.39	13.20
25c0001	-0.14	9.01	um04322	-1.19	12.94
25c0001	0.04	9.69	um04323	-0.43	12.35
25c0001	0.20	11.47	um04324	-0.07	14.33
25c0001	-0.62	11.99	um04327	0.19	12.93
25c0002	-1.81	13.47	um04329	0.69	11.52
25c0002	0.13	10.35	um04330	0.20	11.03
25c0002	0.14	11.64	um04332	0.15	12.61
25c0002	0.19	12.32	um04333	-0.96	12.66
25c0002	-0.10	15.75	um04335	-2.57	11.80
25c0002	-0.31	10.77	um04342	-0.07	9.15
25c0002	-0.12	9.47	um04343	0.42	9.73
25c0002	0.08	12.79	um04344	-0.31	11.06
25c0002	0.25	13.34	um04345	-0.38	14.23
25c0002	0.02	10.66	um04346	0.05	11.03
25c0003	0.03	13.59	um04347	1.80	11.88
25c0003	-0.20	9.22	um04348	-0.06	11.01
25c0003	-0.14	8.89	um04350	0.32	11.88
25c0003	-0.44	12.54	um04351	0.24	12.47
25c0003	1.00	14.27	um04352	-0.59	12.18
25c0003	0.05	12.06	um04353	-3.04	11.28
25c0003	-0.50	12.01	um04354	-2.47	11.38
25c0003	0.22	12.77	um04355	0.24	12.39
25c0003	-0.10	12.95	um04356	-0.20	10.58
25c0003	-0.04	11.81	um04357	-2.84	11.44
25c0004	-0.18	13.45	um04358	-3.26	11.37
25c0004	-0.47	12.15	um04361	0.21	10.52
25c0004	-0.33	12.75	um04362	0.56	12.58
25c0004	-0.77	11.51	um04364	2.07	10.85
25c0004	-0.20	12.96	um04365	-0.13	10.75
25c0004	0.30	12.79	um04367	-0.12	8.76
25c0004	0.25	11.73	um04368	0.26	10.24
25c0004	0.38	12.64	um04370	-0.39	11.72
25c0004	-0.25	11.57	um04371	-0.17	12.58
25c0004	0.23	14.52	um04372	-0.27	11.86
25c0005	-0.05	12.22	um04374	-0.11	13.52
25c0005	0.39	12.08	um04375	-0.16	11.24
25c0005	0.53	11.50	um04376	-0.61	11.03
25c0005	-0.15	9.11	um04378	-0.53	11.41
25c0005	0.06	11.45	um04379	-2.77	11.81
25c0005	-0.02	11.11	um04380	0.15	12.16
25c0005	-0.03	10.61	um04381	-0.15	11.92
25c0005	0.01	13.13	um04382	0.88	12.65

25c0005	0.20	10.18	um04383	0.10	13.36
25c0005	-0.14	12.27	um04384	0.90	13.51
25c0006	-0.82	11.83	um04385	0.45	11.73
25c0006	0.23	12.14	um04388	-0.23	13.39
25c0006	-0.21	12.52	um04389	-0.38	11.95
25c0006	0.14	12.36	um04390	-0.03	12.44
25c0006	0.33	12.62	um04391	0.10	11.90
25c0006	0.15	9.87	um04392	-0.12	12.52
25c0006	0.42	12.03	um04393	-0.90	10.36
25c0006	0.32	12.15	um04394	-0.55	11.93
25c0006	0.56	9.71	um04397	-0.57	12.01
25c0006	-0.04	11.69	um04398	-0.51	12.56
25c0007	0.01	9.48	um04399	0.39	13.54
25c0007	0.62	13.72	um04400	-0.51	15.44
25c0007	0.19	9.31	um04401	-0.01	12.99
25c0007	-0.09	9.05	um04402	-0.15	11.34
25c0007	-0.28	13.57	um04404	0.59	10.49
25c0007	-0.33	13.55	um04405	-0.34	12.23
25c0007	-0.29	13.14	um04406	-0.55	11.92
25c0007	-0.32	14.00	um04407	0.22	11.63
25c0007	-0.43	13.17	um04410	-1.52	13.67
25c0007	-0.32	10.20	um04411	0.03	11.04
25c0008	-0.89	12.31	um04413	-0.41	11.53
25c0008	-1.29	11.74	um04414	-0.50	10.43
25c0008	-1.08	11.33	um04415	-0.12	10.61
25c0008	0.35	12.55	um04416	-0.31	11.15
25c0008	-0.05	11.34	um04417	-0.43	13.05
25c0008	-0.71	9.44	um04419	0.40	12.37
25c0008	0.08	8.63	um04420	0.85	12.14
25c0008	-0.17	9.01	um04421	0.50	10.60
25c0008	-0.07	8.83	um04422	-0.02	10.07
25c0008	0.00	8.54	um04423	-0.52	11.61
25c0009	0.10	8.69	um04424	-0.08	9.92
25d0000	0.16	8.75	um04426	-0.46	13.57
25d0000	-1.35	11.28	um04427	0.57	11.66
25d0000	-0.23	11.80	um04428	-0.28	11.64
25d0000	-0.07	14.30	um04429	-0.76	12.79
25d0000	0.06	12.92	um04430	0.02	13.23
25d0000	0.72	12.08	um04433	1.45	9.97
25d0000	0.25	10.19	um04441	0.20	13.50
25d0000	0.11	11.69	um04442	-0.47	13.16
25d0000	-0.11	12.30	um04443	-0.82	11.57
25d0001	-0.12	15.85	um04444	1.73	11.20
25d0001	0.23	10.75	um04446	0.15	12.87
25d0001	-0.02	10.29	um04447	0.30	12.11
25d0001	0.31	12.65	um04448	-0.51	12.71
25d0001	0.14	9.27	um04451	-0.12	12.41
25d0001	0.36	15.19	um04456	-0.03	12.50
25d0001	0.18	12.09	um04457	0.01	13.60
25d0001	0.09	12.34	um04460	0.01	11.22
25d0001	-0.31	11.81	um04461	-0.04	12.06
25d0001	-0.24	12.55	um04462	-0.10	11.36
25d0002	0.59	13.29	um04463	0.14	12.73
25d0002	-0.02	8.96	um04464	-0.07	11.70

25d0002	-0.43	12.31	um04465	0.35	10.97
25d0002	0.35	12.67	um04466	-0.41	11.75
25d0002	-0.42	12.91	um04468	0.11	11.50
25d0002	0.21	9.66	um04470	-0.35	13.15
25d0002	0.10	12.60	um04471	-0.38	12.70
25d0002	0.11	12.35	um04472	0.44	10.93
25d0002	-1.39	13.54	um04473	0.09	11.11
25d0002	0.55	12.49	um04474	0.63	12.06
25d0003	-0.46	13.29	um04475	0.38	12.02
25d0003	0.20	14.74	um04477	-0.27	12.05
25d0003	-0.28	11.51	um04478	-0.50	12.35
25d0003	0.21	12.98	um04479	1.44	12.92
25d0003	-0.06	11.09	um04480	1.59	12.06
25d0003	-0.14	10.51	um04481	0.93	10.28
25d0003	0.46	13.15	um04482	-2.10	13.38
25d0003	0.66	12.78	um04484	-1.40	12.19
25d0003	0.14	15.64	um04485	-0.06	11.79
25d0003	-0.29	13.39	um04486	0.29	10.88
25d0004	0.20	13.03	um04487	0.08	11.23
25d0004	0.43	12.35	um04488	0.37	12.09
25d0004	-0.18	12.44	um04489	-0.17	12.35
25d0004	0.03	13.50	um04490	0.12	15.40
25d0004	0.50	14.26	um04491	1.14	10.63
25d0004	-0.22	14.02	um04494	0.05	11.78
25d0004	-0.34	13.45	um04495	-0.20	10.95
25d0004	-1.27	13.41	um04496	-0.86	11.19
25d0004	0.09	13.12	um04497	-0.66	13.09
25d0004	0.59	10.49	um04498	-0.31	12.07
25d0005	-0.24	12.99	um04499	-0.58	12.62
25d0005	0.27	11.63	um04500	0.01	12.19
25d0005	0.37	12.08	um04501	0.17	10.70
25d0005	-0.50	12.60	um04503	-0.01	8.85
25d0005	-0.03	13.10	um04505	-0.03	11.30
25d0005	-0.02	8.85	um04506	1.38	11.62
25d0005	0.18	12.22	um04508	0.55	10.39
25d0005	0.28	11.63	um04509	0.14	11.24
25d0005	-0.57	12.53	um04510	-0.30	11.81
25d0005	0.11	13.43	um04511	0.06	13.05
25d0006	-0.11	12.09	um04512	-0.44	10.29
25d0006	-0.45	13.25	um04514	-0.22	12.67
25d0006	0.64	9.38	um04516	0.11	11.44
25d0006	0.34	13.17	um04517	0.19	11.28
25d0006	-0.20	13.06	um04518	-0.33	12.09
25d0006	-0.10	12.13	um04522	0.31	10.63
25d0006	0.29	10.67	um04523	-0.11	14.37
25d0006	-0.21	11.26	um04524	-0.37	11.27
25d0006	0.19	9.27	um04525	0.44	12.77
25d0006	-0.27	10.73	um04526	-0.25	8.68
25d0007	0.24	14.05	um04528	2.19	12.84
25d0007	0.11	10.64	um04529	-0.49	9.78
25d0007	1.22	10.38	um04530	-1.51	13.53
25d0007	0.32	11.46	um04531	0.32	12.58
25d0007	0.15	11.26	um04532	0.47	12.04
25d0007	-0.63	11.51	um04533	-0.86	13.01

25d0007	-0.17	10.89	um04535	-0.60	11.20
25d0007	-0.40	12.04	um04537	0.20	10.75
25d0007	0.15	10.52	um04538	-1.12	11.65
25d0007	0.63	12.20	um04539	-0.11	11.88
25d0008	-1.38	11.74	um04541	-0.12	12.94
25d0008	0.02	8.70	um04542	-0.62	11.56
25d0008	-0.69	10.24	um04543	0.49	11.37
25d0008	0.12	9.01	um04544	-0.29	12.86
25d0008	-0.44	10.23	um04546	0.25	10.47
25d0008	0.38	14.58	um04549	0.31	11.46
25d0008	0.00	12.78	um04550	-1.03	12.41
25d0008	-0.04	9.68	um04551	-0.04	11.45
25d0008	0.19	9.37	um04553	1.12	13.46
25d0008	-0.08	9.00	um04555	-0.67	12.43
25d0009	-0.22	8.91	um04557	2.97	11.14
25d0009	-0.10	8.54	um04558	0.07	9.98
25d0009	-0.03	8.64	um04559	0.70	9.98
25d0009	-0.25	8.89	um04560	0.46	12.65
25d0009	-0.09	9.05	um04561	0.85	11.39
25d0009	-0.20	8.89	um04562	0.05	13.97
26c0000	-0.68	10.15	um04563	0.33	10.63
26c0000	-0.12	8.76	um04566	-0.35	11.81
26c0000	-0.01	8.41	um04570	-0.11	8.65
26c0000	-0.08	8.60	um04572	0.13	10.52
26c0000	-0.43	9.08	um04573	0.46	12.42
26c0000	-0.14	10.48	um04575	2.58	12.46
26c0000	0.17	9.98	um04576	0.05	12.37
26c0000	0.12	10.34	um04577	-3.23	14.46
26c0000	0.00	8.96	um04578	-1.22	11.86
26c0001	0.75	14.40	um04579	0.22	13.39
26c0001	0.48	14.72	um04580	-0.01	12.52
26c0001	0.20	12.81	um04581	0.22	13.90
26c0001	0.22	11.25	um04584	-0.33	12.43
26c0001	0.19	10.13	um04587	0.35	10.40
26c0001	0.00	12.06	um04588	-0.37	15.63
26c0001	0.04	12.01	um04590	-0.16	12.54
26c0001	0.25	12.02	um04591	0.30	11.12
26c0001	-0.16	12.08	um04592	-0.40	12.32
26c0001	-0.06	12.66	um04593	0.08	11.52
26c0002	-0.05	13.00	um04594	-0.15	11.64
26c0002	-0.86	12.84	um04598	-0.51	12.44
26c0002	-0.01	12.04	um04599	0.41	11.27
26c0002	0.18	12.37	um04601	0.09	8.83
26c0002	-0.18	13.41	um04602	-0.62	11.97
26c0002	-0.44	11.76	um04603	-0.32	11.45
26c0002	0.51	11.91	um04604	-0.08	13.78
26c0002	-0.33	12.24	um04605	-0.13	12.84
26c0002	-0.34	11.17	um04607	-0.18	10.50
26c0002	0.52	12.71	um04609	0.11	12.01
26c0003	0.43	10.49	um04610	-0.04	11.28
26c0003	0.16	11.43	um04611	-0.07	14.71
26c0003	-0.18	10.63	um04612	-0.86	11.96
26c0003	0.62	12.99	um04613	-0.12	10.88
26c0003	0.57	10.17	um04614	-0.98	9.36

26c0003	0.50	11.64
26c0003	-0.52	14.20
26c0003	0.58	12.97
26c0003	-0.09	8.73
26c0003	0.18	11.06
26c0004	0.27	13.26
26c0004	0.38	10.66
26c0004	-0.01	8.91
26c0004	-0.94	13.27
26c0004	0.04	11.68
26c0004	-0.08	9.11
26c0004	-0.16	9.25
26c0004	0.19	9.26
26c0004	-0.09	9.79
26c0004	0.00	14.32
26c0005	-0.24	13.42
26c0005	0.07	13.90
26c0005	0.17	12.02
26c0005	-0.07	9.10
26c0005	0.24	11.47
26c0005	-0.46	11.68
26c0005	0.09	12.09
26c0005	0.51	13.32
26c0005	-0.61	12.44
26c0005	0.29	13.41
26c0006	-0.02	8.55
26c0006	0.16	11.22
26c0006	-0.11	8.74
26c0006	0.14	10.68
26c0006	0.01	13.88
26c0006	0.05	11.27
26c0006	-0.56	13.10
26c0006	0.44	12.21
26c0006	0.22	12.46
26c0006	0.12	12.91
26c0007	0.66	11.69
26c0007	0.34	11.75
26c0007	0.11	13.25
26c0007	0.16	12.73
26c0007	-0.48	11.99
26c0007	-0.45	11.11
26c0007	0.16	13.15
26c0007	0.45	11.40
26c0007	0.21	11.15
26c0007	0.30	13.25
26c0008	-0.95	11.05
26c0008	-0.22	11.52
26c0008	-0.17	11.06
26c0008	-0.51	12.51
26c0008	-0.26	12.72
26c0008	0.14	9.78
26c0008	0.22	13.12
26c0008	-0.39	11.99
26c0008	-0.31	13.14

um04615	1.91	11.69
um04616	1.69	11.97
um04619	-0.11	12.66
um04621	0.00	10.50
um04622	0.52	11.73
um04623	-0.42	10.67
um04624	-0.06	11.81
um04625	0.56	10.78
um04627	-0.12	10.99
um04628	-0.23	10.30
um04629	-2.24	13.12
um04630	0.39	11.71
um04632	-0.17	15.62
um04637	0.14	11.29
um04639	-0.14	12.47
um04641	-0.38	14.47
um04644	-0.85	11.58
um04646	-0.28	10.08
um04649	-0.19	12.11
um04650	-0.41	11.36
um04654	0.20	11.32
um04655	0.10	11.08
um04656	0.01	11.32
um04657	0.38	12.12
um04658	0.03	12.71
um04659	-0.11	12.08
um04661	-0.05	12.31
um04665	-0.05	11.08
um04666	-0.53	12.81
um04667	0.33	9.49
um04668	0.02	10.62
um04669	0.57	11.02
um04671	-0.99	11.98
um04672	0.07	10.72
um04673	0.38	11.35
um04674	0.08	11.69
um04676	-0.13	10.22
um04677	-0.36	10.38
um04678	0.13	13.44
um04679	0.11	11.18
um04680	-0.23	11.21
um04681	0.06	10.62
um04682	0.71	11.83
um04683	0.06	11.59
um04686	0.70	11.64
um04688	0.85	11.63
um04689	0.05	10.66
um04693	-0.45	11.34
um04695	1.17	11.32
um04696	1.73	10.08
um04697	-1.23	10.58
um04698	-0.20	10.58
um04700	0.02	12.48
um04701	0.03	12.51

26c0008	-0.10	12.75	um04702	0.25	11.26
26c0009	-0.02	11.48	um04703	-0.56	13.43
26c0009	0.23	12.18	um04704	0.06	11.98
26c0009	0.23	9.85	um04705	-0.92	11.13
26c0009	-0.44	12.64	um04707	0.56	13.30
26c0009	-1.43	13.87	um04711	0.10	12.64
26c0009	0.11	8.99	um04712	-0.04	11.74
26c0009	0.06	11.37	um04713	-0.29	11.67
26c0009	0.10	9.11	um04714	0.40	11.41
26c0009	0.38	10.42	um04715	-0.41	13.03
26d0000	-0.22	8.77	um04716	-0.75	14.61
26d0000	-0.09	9.08	um04720	-0.09	11.98
26d0000	-0.53	10.48	um04721	-0.16	12.25
26d0000	-0.53	11.79	um04722	0.10	12.71
26d0000	0.11	12.37	um04723	0.33	11.54
26d0000	0.55	13.67	um04724	-0.04	12.58
26d0000	-0.17	14.03	um04726	-0.59	12.04
26d0000	0.20	12.12	um04727	-0.95	10.35
26d0000	0.31	11.42	um04728	0.85	11.33
26d0001	0.28	11.89	um04731	-0.17	8.70
26d0001	-0.06	12.84	um04732	0.38	11.14
26d0001	0.56	12.48	um04733	0.12	14.73
26d0001	0.18	10.10	um04734	-0.42	12.31
26d0001	0.16	11.92	um04736	-0.05	11.97
26d0001	0.02	12.49	um04737	2.49	13.61
26d0001	0.11	11.25	um04738	1.47	13.31
26d0001	0.09	13.71	um04739	0.19	13.33
26d0001	0.04	8.86	um04740	0.62	12.23
26d0001	-0.18	13.21	um04742	1.53	14.05
26d0002	-0.63	12.03	um04743	-0.38	11.98
26d0002	-0.20	11.94	um04744	-0.15	11.42
26d0002	0.08	14.08	um04745	0.06	13.05
26d0002	0.06	13.25	um04748	0.08	11.98
26d0002	-0.39	13.28	um04749	0.02	12.30
26d0002	-0.05	12.18	um04750	0.06	11.26
26d0002	0.44	10.46	um04751	0.19	11.77
26d0002	0.03	10.71	um04752	0.05	10.73
26d0002	-0.01	9.38	um04753	0.33	11.12
26d0002	0.11	11.51	um04755	-0.24	10.94
26d0003	0.61	12.63	um04758	0.45	10.59
26d0003	-0.02	13.15	um04761	0.26	14.71
26d0003	0.29	12.68	um04764	0.32	12.93
26d0003	-0.68	14.82	um04765	-0.13	11.21
26d0003	-0.24	12.37	um04767	0.13	12.68
26d0003	-0.28	12.85	um04768	-0.31	12.15
26d0003	-0.05	13.82	um04770	0.39	10.77
26d0003	0.17	13.06	um04771	-0.79	13.86
26d0003	-0.21	11.89	um04772	0.64	9.51
26d0003	0.05	13.65	um04773	0.50	10.98
26d0004	-0.02	14.14	um04775	0.22	10.78
26d0004	-0.02	12.29	um04776	-1.12	13.91
26d0004	-0.26	12.49	um04777	0.00	8.82
26d0004	0.30	11.85	um04778	0.13	9.32
26d0004	0.41	11.40	um04779	-0.12	11.25

26d0004	-1.13	13.03	um04781	0.64	12.36
26d0004	0.30	9.54	um04782	-0.06	12.19
26d0004	-0.10	11.46	um04785	0.12	12.52
26d0004	1.49	13.00	um04786	-0.60	12.24
26d0004	0.02	15.00	um04787	-0.90	10.70
26d0005	-0.16	14.72	um04789	-0.10	10.75
26d0005	0.15	11.85	um04790	-0.11	8.65
26d0005	0.23	13.08	um04791	0.62	9.65
26d0005	-0.39	11.84	um04793	0.13	13.78
26d0005	0.12	12.11	um04794	1.14	13.97
26d0005	-0.35	11.03	um04795	0.39	11.92
26d0005	0.39	8.88	um04796	0.96	12.85
26d0005	0.61	12.02	um04797	0.01	12.33
26d0005	0.69	13.58	um04798	-0.41	10.95
26d0005	-0.36	12.91	um04799	0.47	11.58
26d0006	-0.67	9.30	um04800	0.41	12.51
26d0006	-0.70	13.08	um04801	0.90	12.94
26d0006	-0.02	11.89	um04802	-0.02	14.40
26d0006	0.20	9.42	um04804	0.23	9.09
26d0006	0.21	11.67	um04805	1.02	11.08
26d0006	0.07	13.31	um04806	0.95	11.26
26d0006	0.18	11.37	um04807	-2.10	11.11
26d0006	-0.48	11.76	um04808	-0.26	11.75
26d0006	0.07	11.83	um04809	0.11	10.86
26d0006	0.32	11.75	um04811	0.12	11.95
26d0007	0.28	12.53	um04812	0.19	12.22
26d0007	-0.16	11.91	um04813	0.56	11.07
26d0007	0.41	10.54	um04815	-0.21	9.80
26d0007	0.19	14.23	um04816	0.09	11.20
26d0007	-0.16	12.31	um04817	-0.08	12.69
26d0007	-1.37	11.27	um04818	0.00	10.87
26d0007	0.31	11.26	um04819	0.67	11.50
26d0007	0.07	10.98	um04820	-0.08	10.92
26d0007	0.09	12.50	um04826	-0.16	11.09
26d0007	-0.15	12.48	um04827	-0.23	12.27
26d0008	0.01	9.31	um04830	0.51	11.72
26d0008	-0.31	12.65	um04831	0.06	12.14
26d0008	-0.41	9.41	um04832	0.03	12.38
26d0008	0.71	13.12	um04833	0.33	13.34
26d0008	-0.49	13.50	um04834	1.05	12.26
26d0008	-0.64	12.04	um04835	0.40	10.57
26d0008	-0.20	12.19	um04836	-0.40	8.73
26d0008	0.43	12.52	um04838	-0.19	12.19
26d0008	0.28	11.45	um04840	-0.14	12.62
26d0008	-0.49	11.51	um04841	-0.30	9.10
26d0009	-0.12	9.32	um04844	-0.20	10.95
26d0009	0.30	10.76	um04845	-0.03	12.57
26d0009	0.82	13.36	um04846	-0.21	11.44
26d0009	0.18	12.79	um04847	0.79	12.12
26d0009	0.55	14.92	um04848	-0.13	12.80
26d0009	0.24	11.42	um04849	0.17	11.41
26d0009	-0.07	12.46	um04850	0.37	11.28
26d0009	-0.20	12.77	um04851	-0.68	11.93
26d0009	-0.18	8.69	um04852	-0.44	11.08

26d0009	0.24	11.22	um04855	0.21	14.78
26d0010	-0.19	8.64	um04856	-0.08	12.88
26d0010	0.26	9.94	um04859	-0.20	12.54
26d0010	0.00	9.07	um04860	0.06	11.46
26d0010	-0.04	8.77	um04863	0.75	11.35
26d0010	0.23	12.43	um04865	0.54	12.58
27c0000	-0.38	12.45	um04867	0.97	9.58
27c0000	-0.56	12.15	um04869	0.07	11.33
27c0000	-0.49	10.94	um04870	-0.06	10.39
27c0000	0.10	9.85	um04871	-1.32	14.53
27c0000	0.90	12.41	um04872	-1.04	14.20
27c0000	-0.19	12.25	um04873	-1.22	12.56
27c0000	-0.24	11.65	um04875	0.71	11.87
27c0000	-0.53	11.68	um04876	1.01	9.84
27c0000	0.11	10.58	um04877	0.42	9.05
27c0001	0.05	12.34	um04878	-0.73	10.77
27c0001	0.64	11.44	um04880	-0.22	11.01
27c0001	-0.77	10.78	um04881	0.47	11.39
27c0001	-0.05	13.35	um04885	0.74	9.52
27c0001	0.14	12.98	um04886	-0.20	8.71
27c0001	0.09	13.16	um04887	-0.30	13.37
27c0001	0.71	12.88	um04888	-0.70	9.27
27c0001	0.36	13.19	um04889	0.00	9.35
27c0001	-0.05	12.12	um04891	-0.48	8.87
27c0001	0.52	14.08	um04892	-0.09	8.69
27c0002	0.10	8.91	um04893	-0.17	8.58
27c0002	-0.52	12.76	um04895	0.05	11.30
27c0002	-0.59	11.59	um04896	-0.01	13.80
27c0002	0.11	13.29	um04897	0.07	12.37
27c0002	-0.66	11.08	um04898	0.05	12.54
27c0002	-0.24	11.61	um04899	-1.25	13.76
27c0002	-0.26	10.81	um04900	0.01	10.71
27c0002	-0.38	12.08	um04901	-0.14	11.18
27c0002	-0.24	13.91	um04902	0.56	12.53
27c0002	0.15	12.48	um04905	0.06	10.69
27c0003	-0.48	12.71	um04906	-0.09	11.42
27c0003	0.27	14.79	um04907	0.06	10.85
27c0003	-0.95	11.58	um04908	-0.01	12.80
27c0003	-0.18	12.13	um04909	-0.03	12.17
27c0003	0.12	8.92	um04910	-2.75	10.29
27c0003	0.37	12.19	um04911	-0.09	8.57
27c0003	0.80	10.21	um04912	-1.69	12.58
27c0003	0.10	11.07	um04913	0.13	10.45
27c0003	0.73	9.92	um04914	-0.48	10.28
27c0003	0.10	11.11	um04915	0.69	10.24
27c0004	0.72	10.46	um04916	0.28	12.13
27c0004	0.00	11.99	um04918	0.38	12.38
27c0004	-0.50	12.54	um04920	0.30	11.10
27c0004	0.56	15.41	um04922	0.63	14.63
27c0004	0.05	11.85	um04923	0.35	10.92
27c0004	-0.26	11.10	um04925	0.16	11.37
27c0004	0.12	12.83	um04926	-0.24	15.44
27c0004	0.12	12.56	um04927	-0.03	11.47
27c0004	0.17	12.54	um04928	-0.12	9.37

27c0004	-0.05	15.49	um0492	-0.14	8.60
27c0005	-0.64	11.60	um0493	-1.11	14.25
27c0005	-0.17	12.61	um04931	0.04	11.78
27c0005	-0.13	12.46	um0493	0.15	12.69
27c0005	0.17	12.86	um0493	-0.28	10.66
27c0005	-0.69	13.21	um0493	0.10	11.30
27c0005	-0.90	13.22	um0493	0.82	13.85
27c0005	0.02	9.21	um0494	-0.42	10.31
27c0005	-0.29	12.74	um0494	-0.13	15.68
27c0005	0.18	10.90	um0494	-0.36	11.22
27c0005	0.14	10.38	um0494	0.64	11.50
27c0006	0.15	11.87	um0494	0.29	11.28
27c0006	0.25	12.42	um0494	0.17	14.42
27c0006	0.12	8.81	um0495	0.08	11.87
27c0006	0.06	11.86	um04951	0.13	11.47
27c0006	0.01	8.98	um0495	-0.71	10.75
27c0006	-0.15	12.88	um0495	-0.09	12.90
27c0006	-0.78	12.55	um0495	-0.34	8.65
27c0006	-0.16	10.74	um0495	0.04	13.18
27c0006	-0.09	12.72	um0496	0.03	11.44
27c0006	-0.53	10.78	um04961	-0.67	11.01
27c0007	-0.32	12.39	um0496	-0.38	11.91
27c0007	0.08	9.81	um0496	0.16	11.67
27c0007	0.33	13.76	um0496	-0.49	11.94
27c0007	0.15	0000001	um0496	0.29	6314599
27c0007	-0.02	10.62	um0496	-0.42	11.62
27c0007	-1.13	12.70	um0496	-1.28	14.75
27c0007	0.07	12.32	um0496	-0.09	8.65
27c0007	0.16	12.95	um0496	-0.37	10.91
27c0007	0.25	11.39	um0497	-0.10	10.95
27c0007	0.26	9.92	um04971	4.04	13.49
27c0008	-0.08	9.43	um0497	0.82	12.49
27c0008	-0.25	11.78	um0497	0.82	14.89
27c0008	0.13	9.56	um0497	-0.03	10.96
27c0008	-0.05	12.94	um0497	-0.44	11.68
27c0008	-0.44	12.78	um0497	0.66	11.76
27c0008	0.59	13.64	um0497	-0.75	11.84
27c0008	0.25	15.12	um0498	0.07	11.88
27c0008	0.32	11.08	um0498	0.99	11.29
27c0008	0.57	11.48	um0498	-0.63	1.00
27c0008	-0.25	11.42	um0498	0.33	11.70
27c0009	-0.16	12.74	um0498	0.05	12.27
27c0009	0.15	11.28	um0498	-0.22	15.77
27c0009	0.76	10.86	um0498	-0.66	13.17
27c0009	0.25	51"02205	um0498	0.52	11.42
27c0009	0.55	11.01	um0499	0.05	8.77
27c0009	-0.66	12.46	um04991	-0.70	12.69
27c0009	-0.56	11.93	um0499	0.58	10.72
27c0009	-0.11	9.79	um0499	0.28	11.71
27c0009	-0.12	9.72	um0499	-0.33	13.20
27d0000	0.27	13.25	um0499	-0.11	11.40
27d0000	0.19	12.49	um0499	0.54	10.92
27d0000	-0.25	13.63	um0499	0.07	9.04
27d0000	0.16	9.88	um0499	-0.87	11.34

27d0000	-4.00	11.39	um05002	0.09	10.87
27d0000	0.09	9.49	um05004	0.23	11.45
27d0000	0.55	10.32	um05005	0.04	11.78
27d0000	-0.02	10.36	um05007	0.03	11.41
27d0000	0.23	11.29	um05009	-0.12	9.23
27d0001	0.26	12.16	um05013	-0.04	11.26
27d0001	-1.17	12.71	um05014	0.05	12.42
27d0001	0.17	14.14	um05015	0.15	12.71
27d0001	0.14	11.27	um05017	0.21	11.70
27d0001	0.12	12.01	um05018	0.02	11.80
27d0001	0.10	14.31	um05019	-1.74	13.24
27d0001	-0.35	12.61	14c0011	0.37	12.40
27d0001	-0.54	11.44	um05023	0.62	15.23
27d0001	-0.38	13.40	um05025	-0.14	11.69
27d0001	0.14	11.76	um05026	-0.09	10.94
27d0002	0.65	14.52	um05027	-0.50	8.60
27d0002	0.14	13.21	um05028	-0.55	10.45
27d0002	-1.81	13.64	um05029	0.30	11.42
27d0002	0.00	11.47	um05030	-0.19	12.84
27d0002	0.32	10.32	um05031	0.32	15.12
27d0002	0.00	9.88	um05032	-0.56	12.98
27d0002	0.25	11.45	um05033	-0.38	9.98
27d0002	0.07	12.61	um05034	-0.09	9.75
27d0002	-1.15	11.67	um05035	-0.22	11.65
27d0002	0.27	12.27	um05036	-1.91	14.79
27d0003	-0.09	11.75	um05037	-0.18	11.95
27d0003	-0.15	11.88	um05038	-3.05	12.26
27d0003	0.08	11.78	um05039	0.00	11.50
27d0003	0.44	14.28	um05040	0.53	14.82
27d0003	-0.21	14.95	um05042	-0.46	11.77
27d0003	-0.06	9.27	um05044	0.41	10.38
27d0003	0.15	8.75	um05045	-0.07	11.19
27d0003	0.24	13.72	um05046	-1.39	10.15
27d0003	0.23	11.51	um05050	0.43	12.04
27d0003	0.37	12.15	um05052	-0.29	9.67
27d0004	-0.67	12.98	um05053	-0.14	12.38
27d0004	0.00	11.36	um05054	-0.04	10.74
27d0004	-0.06	13.28	um05055	0.05	10.27
27d0004	-0.58	11.36	um05057	-0.48	11.67
27d0004	-0.12	11.47	um05058	-0.82	13.82
27d0004	-0.17	12.87	um05059	0.83	13.05
27d0004	-1.37	12.27	um05060	0.29	12.91
27d0004	-0.22	13.53	um05061	-0.17	13.21
27d0004	0.42	13.02	um05062	0.01	13.10
27d0004	0.68	13.55	um05063	-0.48	11.54
27d0005	-4.00	11.87	um05064	0.05	10.96
27d0005	0.07	12.91	um05065	-0.10	12.67
27d0005	0.40	12.86	um05066	-0.52	12.71
27d0005	0.17	10.39	um05068	-0.17	8.48
27d0005	0.02	10.06	um05069	0.88	9.68
27d0005	0.46	14.04	um05070	-0.30	10.03
27d0005	-0.05	11.96	um05072	0.97	11.05
27d0005	-0.21	12.85	um05074	-2.56	12.26
27d0005	-0.09	12.63	um05076	-2.74	13.09

27d0005	-0.61	13.40	um0507{	-3.02	10.24
27d0006	-0.54	13.30	um0508(	-1.07	11.36
27d0006	0.27	12.46	um0508:	-0.86	12.97
27d0006	0.54	11.37	um0508⁴	3.86	12.96
27d0006	0.16	11.24	um0508:	0.14	10.49
27d0006	-0.61	11.02	um0508;	-0.16	12.32
27d0006	0.29	13.13	um0508{	-0.18	12.81
27d0006	0.03	10.91	um0508{	0.58	10.99
27d0006	-0.20	11.55	um0509(	0.01	15.01
27d0006	-0.25	11.24	um05091	0.21	12.61
27d0006	-0.04	9.69	um0509⁴	-0.44	9.29
27d0007	0.11	12.47	um0509:	-0.20	8.47
27d0007	0.59	11.32	um0509;	-0.23	8.95
27d0007	0.09	11.80	um0510(	-0.48	13.31
27d0007	-0.37	11.61	um05101	-0.07	11.37
27d0007	-0.94	9.86	um0510:	-0.42	13.86
27d0007	-0.45	12.34	um0510:	-1.25	12.92
27d0007	0.05	9.88	um0510⁴	-0.79	15.19
27d0007	0.19	10.74	um0510:	-0.34	15.17
27d0007	-0.15	8.87	um0510;	-0.01	11.12
27d0007	-1.02	11.78	um0510{	0.02	12.42
27d0008	0.07	11.80	um0510{	0.30	12.13
um0558{	-0.16	13.84	um0511(	0.16	11.13
27d0008	-0.23	11.81	um05111	-0.39	11.47
27d0008	-0.20	12.13	um0511:	0.14	11.63
27d0008	-0.22	14.31	um0511:	-0.62	12.44
27d0008	-0.80	13.13	um0511⁴	-0.97	12.10
27d0008	-4.00	12.22	um0511:	0.20	12.18
27d0008	-0.09	12.47	um0511(	-0.80	11.88
27d0008	0.35	12.34	um0511;	-0.24	11.42
27d0008	-1.73	11.24	um0511{	0.54	11.26
27d0009	0.23	12.29	um0512:	0.78	9.45
27d0009	0.35	13.96	um0512⁴	-0.02	8.57
27d0009	-0.52	8.95	um0512:	-0.33	12.90
27d0009	-1.19	10.23	um0512(	-0.20	12.69
27d0009	-0.07	8.72	um0512;	0.24	11.28
27d0009	0.50	12.03	um0512{	0.17	11.37
27d0009	0.03	9.25	um0513(	1.13	14.33
27d0009	0.88	12.48	um05131	0.70	12.26
27d0009	-0.14	8.97	um0513:	1.01	11.19
27d0009	0.40	10.97	um0513⁴	-0.35	12.02
27d0010	-0.12	11.58	um0513(	0.22	10.92
27d0010	-0.71	11.54	um0513;	-0.02	14.69
27d0010	0.15	10.99	um0513{	0.81	10.49
2c00001	0.20	12.63	um0513{	0.31	14.84
2c00002	0.41	12.55	um05141	0.47	11.37
2c00003	1.12	13.44	um0514:	-0.26	11.91
2c00004	1.02	11.30	um0514:	-0.39	14.55
2c00005	-0.04	8.44	um0514:	-0.14	11.46
2c00006	-0.13	8.89	um0514(	0.56	11.44
2c00007	-0.18	8.52	um0514{	0.40	10.37
2c00008	0.44	13.80	um0514{	-0.10	11.96
2c00009	-0.73	14.16	um05151	0.29	11.05
2c00010	0.18	13.01	um0515:	-1.60	10.51

2c00011	-1.96	14.28	um05153	-0.87	9.26
2c00012	0.25	12.94	um05155	-0.09	8.59
2c00013	0.03	12.45	um05156	-0.81	12.71
2c00014	0.15	13.16	um05158	0.07	12.56
2c00015	0.11	12.33	um05159	0.75	10.69
2c00016	0.47	14.97	um05160	-0.63	12.70
2c00017	1.13	13.90	um05161	-0.40	12.48
2c00018	-0.62	11.41	um05162	-0.62	12.15
2c00019	0.08	8.46	um05163	0.55	11.33
2c00020	-0.29	11.81	um05166	-0.04	11.26
2c00021	0.03	8.74	um05167	-0.01	12.41
2c00022	-4.00	13.62	um05169	0.09	11.81
2c00023	0.24	11.30	um05170	3.15	13.73
2c00024	-0.15	11.87	um05171	0.18	12.16
2c00025	-0.09	10.47	um05173	-0.16	12.84
2c00026	-0.22	12.93	um05174	0.00	12.64
2c00027	0.25	10.84	um05177	-0.50	11.91
2c00028	-0.12	12.96	um05178	0.36	11.18
2c00029	0.06	12.01	um05179	-0.41	12.77
2c00030	0.05	15.10	um05180	0.33	11.14
2c00031	0.42	14.50	um05182	-0.31	10.87
2c00032	-0.21	12.32	um05183	1.03	12.45
2c00033	-0.05	12.47	um05184	0.62	11.65
2c00034	-0.94	12.96	um05185	-0.26	11.08
2c00035	-0.15	9.74	um05186	0.50	11.20
2c00036	-0.27	10.67	um05191	0.30	11.26
2c00037	0.33	13.15	um05192	-0.47	12.74
2c00038	0.50	13.83	um05194	-0.24	12.28
2c00039	0.58	13.54	um05197	-0.08	12.75
2c00040	0.55	14.95	um05198	-0.93	9.81
2c00041	0.37	13.28	um05199	-0.29	12.04
2c00042	-0.21	12.12	um05200	-0.06	11.05
2c00043	0.49	11.75	um05201	-0.19	10.66
2c00044	-1.12	12.79	um05202	1.04	15.02
2c00045	0.46	12.72	um05204	-0.32	13.47
2c00046	-0.44	12.61	um05206	-0.61	12.11
2c00047	-0.03	12.89	um05207	-0.27	10.29
2c00048	-0.16	11.75	um05208	0.20	10.86
2c00049	-0.31	11.53	um05209	0.14	11.97
2c00050	-0.45	11.78	um05210	0.31	11.05
2c00051	0.11	9.69	um05212	0.66	12.62
2c00052	0.38	12.46	um05213	0.35	11.37
2c00053	0.21	10.01	um05214	0.01	10.90
2c00054	0.06	11.32	um05215	0.12	10.64
2c00055	0.37	12.81	um05216	-0.13	12.56
2c00056	-0.43	13.60	um05217	-0.27	14.36
2c00057	-0.14	10.12	um05218	0.53	12.49
2c00058	0.22	10.75	um05219	-0.02	11.30
2c00059	0.34	12.07	um05222	3.34	13.27
2c00060	0.16	14.49	um05223	0.63	10.80
2c00061	-4.00	12.40	um05224	0.41	11.33
2c00062	-0.09	8.49	um05225	0.42	13.56
2c00063	-0.63	12.76	um05226	3.42	13.03
2c00064	-0.17	12.49	um05227	0.88	9.78

2c00065	-0.22	12.96	um0522[illegible]	-0.13	13.93
2c00066	-0.01	11.49	um0522[illegible]	0.01	10.70
2c00067	-0.59	12.99	um0523[illegible]	1.00	11.61
2c00068	0.13	12.02	um0523[illegible]	-0.54	13.28
2c00069	-0.32	13.53	um0523[illegible]	0.09	11.50
2c00070	0.24	11.68	um0523[illegible]	-0.98	12.58
2c00071	-0.19	11.94	um0523[illegible]	-0.06	11.03
2c00072	-0.01	13.53	um0523[illegible]	-0.11	12.34
2c00073	0.04	8.76	um0523[illegible]	0.25	11.05
2c00074	0.64	11.60	um0524[illegible]	0.98	12.10
2c00075	0.34	10.53	um0524[illegible]	0.73	11.99
2c00076	-1.10	12.50	um0524[illegible]	0.48	9.55
2c00077	-0.10	10.57	um0524[illegible]	-0.95	11.70
2c00078	0.59	12.75	um0524[illegible]	-2.74	11.21
2c00079	0.14	13.72	um0524[illegible]	-1.82	11.42
2c00080	-0.71	12.72	um0524[illegible]	0.98	13.62
2c00081	0.43	11.34	um0525[illegible]	-3.23	11.85
2c00082	0.03	12.29	um0525[illegible]	-3.43	11.21
2c00083	0.34	12.05	um0525[illegible]	0.10	10.05
2c00084	0.03	12.16	um0525[illegible]	-0.35	12.82
2c00085	0.37	12.09	um0525[illegible]	-0.21	9.98
2c00086	0.38	11.29	um0525[illegible]	0.26	11.48
2d00001	0.15	11.72	um0525[illegible]	-0.02	12.59
2d00002	0.45	13.22	um0526[illegible]	0.04	12.07
2d00003	-0.03	12.29	um0526[illegible]	-0.29	11.67
2d00004	0.12	11.96	um0526[illegible]	-0.08	11.28
2d00005	0.09	13.34	um0526[illegible]	-0.03	10.91
2d00006	-0.08	8.90	um0526[illegible]	-0.11	8.99
2d00007	-0.54	14.52	um0526[illegible]	-0.25	10.05
2d00008	0.52	13.41	um0526[illegible]	-4.00	12.16
2d00009	0.12	12.08	um0526[illegible]	0.51	11.42
2d00010	-0.08	11.78	um0526[illegible]	-0.23	10.94
2d00011	0.45	14.43	um0526[illegible]	-0.16	11.72
2d00012	0.15	12.08	um0527[illegible]	0.44	11.85
2d00013	0.12	11.83	um0527[illegible]	0.36	10.94
2d00014	0.88	11.59	um0527[illegible]	-0.27	12.51
2d00015	0.44	12.02	um0527[illegible]	0.09	11.83
2d00016	0.48	13.02	um0527[illegible]	0.53	11.73
2d00017	-0.03	9.12	um0527[illegible]	0.77	12.64
2d00018	0.31	12.90	um0527[illegible]	3.47	11.85
2d00019	0.43	13.12	um0527[illegible]	-0.13	11.30
2d00020	-0.39	12.31	um0528[illegible]	0.41	11.70
2d00021	1.06	15.34	um0528[illegible]	-0.13	12.04
2d00022	-4.00	12.65	um0528[illegible]	0.13	12.54
2d00023	0.34	12.87	um0528[illegible]	0.07	11.92
2d00024	-0.37	10.85	um0528[illegible]	-1.10	11.91
2d00025	-0.17	10.92	um0528[illegible]	-0.22	11.85
2d00026	0.00	14.67	um0529[illegible]	-0.61	11.76
2d00027	0.11	12.93	um0529[illegible]	0.10	12.62
2d00028	0.15	11.99	um0529[illegible]	0.24	11.67
2d00029	0.10	9.56	um0529[illegible]	-0.26	12.82
2d00030	-0.25	13.35	um0529[illegible]	-0.34	8.50
2d00031	0.53	12.02	um0529[illegible]	-0.15	8.44
2d00032	0.10	12.55	um0529[illegible]	0.48	9.19

2d00033	0.07	11.84
2d00034	-1.53	11.68
2d00035	-0.02	13.30
2d00036	-0.23	11.76
2d00037	-0.67	13.64
2d00038	-0.42	12.92
2d00039	1.39	14.46
2d00040	-0.35	10.65
2d00041	0.18	10.05
2d00042	0.50	13.54
2d00043	0.39	11.63
2d00044	-0.08	10.13
2d00045	0.28	10.72
2d00046	0.32	12.52
2d00047	0.23	12.98
2d00048	-0.44	13.45
2d00049	-0.07	12.37
2d00050	-0.14	9.04
2d00051	-0.09	12.24
2d00052	-0.16	10.81
2d00053	0.06	11.91
2d00054	-0.11	12.23
2d00055	0.12	8.88
2d00056	-0.28	12.19
2d00057	-0.05	8.94
2d00058	-0.17	12.04
2d00059	-0.20	13.22
2d00060	0.18	11.01
2d00061	0.06	10.67
2d00062	-0.51	12.07
2d00063	0.15	10.89
2d00064	0.04	12.11
2d00065	-0.21	11.39
2d00066	-0.66	12.91
2d00067	-0.07	12.47
2d00068	-0.39	12.90
2d00069	-0.95	13.34
2d00070	-0.04	12.80
2d00071	-1.32	11.52
2d00072	0.05	9.18
2d00073	-0.01	11.12
2d00074	0.24	11.25
2d00075	0.50	13.05
313c000	0.09	8.50
3c00001	0.86	14.95
3c00002	0.08	9.99
3c00003	-0.08	11.57
3c00004	-0.13	15.78
3c00005	-0.11	11.13
3c00006	0.14	12.93
3c00007	-0.03	12.36
3c00008	1.13	15.12
3c00009	-0.15	12.32
3c00010	-0.36	12.02

um05300	0.59	9.23
um05301	0.30	11.32
um05302	-0.29	8.61
um05303	-0.29	8.54
um05305	-0.21	8.69
um05306	-0.09	8.58
um05308	-0.03	8.59
um05309	-0.24	8.56
um05310	-0.30	8.48
um05311	0.07	8.82
um05312	-0.05	8.57
um05313	-0.06	10.34
um05314	-0.08	8.58
um05316	-0.18	8.42
um05317	-0.31	8.87
um05318	-0.21	8.79
um05319	-0.41	8.82
um05322	-0.14	8.56
um05325	1.64	12.86
um05326	-0.37	12.66
um05327	0.42	12.81
um05328	0.86	9.74
um05329	0.58	11.45
um05331	-0.12	12.29
um05332	0.17	10.27
um05333	-0.48	10.71
um05334	0.30	12.78
um05335	0.53	11.68
um05337	-0.12	12.11
um05338	-0.10	11.33
um05339	-1.55	15.14
um05340	-0.23	12.33
um05341	-0.76	9.16
um05342	0.40	10.32
um05344	1.09	12.72
um05345	0.63	11.54
um05346	1.24	13.68
um05347	-0.95	11.95
um05348	-0.04	11.54
um05349	-0.44	10.46
um05350	-0.15	8.64
um05351	-0.34	12.79
um05352	0.04	11.71
um05353	0.52	11.36
um05354	0.35	11.70
um05355	-0.88	11.70
um05356	-0.19	8.91
um05357	-0.94	12.55
um05358	-0.48	10.46
um05361	0.25	9.61
um05362	-1.11	14.56
um05363	0.09	11.93
um05364	0.21	10.49
um05365	0.52	13.04

3c00011	0.62	13.23	um05366	-0.52	15.84
3c00012	0.66	10.17	um05368	0.19	11.33
3c00013	0.70	12.39	um05370	2.03	12.23
3c00014	0.36	12.36	um05371	-0.26	12.14
3c00015	0.06	9.11	um05373	0.28	12.65
3c00016	0.68	14.29	um05376	-0.10	11.70
3c00017	0.31	11.13	um05377	0.01	10.79
3c00018	-0.77	15.25	um05378	0.10	12.39
3c00019	-0.45	14.22	um05380	-0.12	11.84
3c00020	0.06	13.06	um05381	-0.69	10.97
3c00021	0.00	13.77	um05382	-0.36	12.23
3c00022	0.09	14.63	um05383	0.05	12.59
3c00023	-4.00	11.94	um05384	0.02	9.40
3c00024	0.62	12.59	um05385	0.82	11.55
3c00025	-0.31	12.25	um05386	0.33	12.18
3c00026	0.08	13.28	um05387	0.54	11.36
3c00027	-0.01	15.87	um05389	-0.11	8.97
3c00028	0.41	13.40	um05390	0.90	12.40
3c00029	0.02	9.32	um05391	-0.36	13.96
3c00030	-0.03	15.95	um05392	-0.21	10.48
3c00031	0.00	15.07	um05393	0.59	11.18
3c00032	0.23	13.79	um05394	-0.21	8.42
3c00033	-0.80	12.96	um05395	0.03	11.70
3c00034	0.51	13.57	um05396	1.54	11.18
3c00035	-0.03	8.75	um05397	-0.05	10.16
3c00036	-0.21	8.50	um05398	-0.57	12.59
3c00037	0.37	12.81	um05399	0.61	10.62
3c00038	0.35	14.00	um05401	-0.59	12.44
3c00039	-0.60	12.44	um05402	-0.91	12.27
3c00040	-0.08	13.11	um05403	-0.82	12.59
3c00041	-0.78	15.11	um05404	-0.04	11.36
3c00042	-0.38	13.88	um05405	0.09	13.52
3c00043	-0.52	12.78	um05406	0.14	11.44
3c00044	0.12	13.19	um05407	-0.15	11.97
3c00045	0.22	13.31	um05408	0.05	12.02
3c00046	0.01	14.27	um05410	0.78	12.06
3c00047	-0.62	12.94	um05411	-4.00	12.11
3c00048	-0.08	11.09	um05412	-0.46	11.42
3c00049	-0.42	14.25	um05414	-1.61	11.89
3c00050	-0.11	12.75	um05415	-0.40	11.50
3c00051	0.93	12.63	um05416	-0.08	10.58
3c00052	0.08	9.64	um05417	-0.09	12.01
3c00053	-0.07	11.86	um05420	-0.69	12.62
3c00054	0.01	13.35	um05421	-2.98	12.18
3c00055	0.32	10.38	um05422	-2.40	11.13
3c00056	0.45	13.59	um05423	-0.58	12.53
3c00057	-0.95	12.56	um05424	0.09	8.90
3c00058	-0.01	12.89	um05426	-0.65	9.26
3c00059	-0.26	13.34	um05427	-0.26	8.69
3c00060	0.24	11.28	um05428	-0.11	12.06
3c00061	0.44	12.67	um05429	0.92	11.49
3c00062	-0.18	11.84	um05431	-1.04	14.03
3c00063	-0.90	11.98	um05432	0.15	10.83
3c00064	0.09	11.76	um05433	-0.38	13.01

3c00065	0.09	12.75	um0543[illegible]	-0.09	11.77
3c00066	-0.02	12.95	um0543[illegible]	0.20	12.10
3c00067	0.42	12.79	um0543[illegible]	-0.43	8.76
3c00068	0.57	14.81	um0543[illegible]	-0.28	12.88
3c00069	-0.46	15.63	um0543[illegible]	-0.02	10.63
3c00070	-0.43	12.71	um0543[illegible]	0.32	11.71
3c00071	0.16	11.55	um0544[illegible]	-0.87	10.32
3c00072	-0.20	14.08	um0544[illegible]	-0.29	10.34
3c00073	-0.10	11.13	um0544[illegible]	0.15	9.53
3c00074	0.29	11.53	um0544[illegible]	0.89	11.94
3c00075	0.90	12.22	um0544[illegible]	0.20	13.48
3c00076	0.10	13.29	um0544[illegible]	0.52	12.35
3c00077	0.61	12.18	um0544[illegible]	0.44	11.93
3c00078	-0.25	13.69	um0545[illegible]	1.01	13.77
3c00079	-0.67	12.01	um0545[illegible]	0.72	10.50
3c00080	0.00	15.96	um0545[illegible]	0.35	12.40
3d00001	0.71	13.14	um0545[illegible]	0.23	12.37
3d00002	0.44	10.46	um0545[illegible]	0.34	14.02
3d00003	0.98	12.15	um0545[illegible]	-0.20	11.66
3d00004	-0.26	11.89	um0545[illegible]	0.46	10.64
3d00005	1.47	11.57	um0546[illegible]	-0.22	8.81
3d00006	-0.21	10.21	um0546[illegible]	0.40	14.26
3d00007	0.36	14.03	um0546[illegible]	0.25	11.79
3d00008	-0.06	12.66	um0546[illegible]	-0.03	8.80
3d00009	0.06	10.23	um0547[illegible]	-0.31	12.47
3d00010	-0.08	13.54	um05471	0.23	11.32
3d00011	-0.23	11.59	um0547[illegible]	0.10	14.64
3d00012	0.01	8.82	um0547[illegible]	-0.45	12.28
3d00013	0.20	13.29	um0547[illegible]	0.39	12.68
3d00014	-0.44	13.88	um0547[illegible]	0.04	11.67
3d00015	-0.20	12.99	um0548[illegible]	-0.49	13.05
3d00016	0.89	15.29	um05481	-4.00	12.20
3d00017	-0.67	13.18	um0548[illegible]	0.13	14.00
3d00018	0.74	11.88	um0548[illegible]	0.38	11.93
3d00019	0.08	11.74	um0548[illegible]	-1.25	9.88
3d00020	-0.29	13.67	um0548[illegible]	-0.26	11.68
3d00021	0.02	15.89	um0549[illegible]	-0.53	12.17
3d00022	0.11	11.55	um0549[illegible]	-2.49	10.99
3d00023	0.32	15.32	um0549[illegible]	-0.24	12.29
3d00024	0.51	14.02	um0549[illegible]	-2.26	10.38
3d00025	0.20	14.01	um0549[illegible]	-0.54	13.19
3d00026	-0.68	13.61	um0549[illegible]	0.17	13.24
3d00027	-0.37	12.75	um0549[illegible]	0.10	12.78
3d00028	-0.15	11.85	um05501	0.01	12.16
3d00029	0.55	12.29	um0550[illegible]	0.12	11.92
3d00030	-0.40	12.35	um0550[illegible]	-0.41	13.69
3d00031	0.37	12.78	um0550[illegible]	-0.54	10.98
3d00032	0.38	14.44	um0550[illegible]	-0.15	11.83
3d00033	0.03	10.88	um0550[illegible]	-0.08	11.87
3d00034	-0.15	11.95	um0550[illegible]	1.87	11.76
3d00035	0.05	13.26	um0550[illegible]	-1.87	12.53
3d00036	0.71	13.81	um05511	-0.14	14.56
3d00037	-0.31	11.53	um0551[illegible]	0.37	10.64
3d00038	0.02	9.07	um0551[illegible]	-1.44	13.16

3d00039	-0.07	13.40	um05515	0.18	12.27
3d00040	0.43	15.47	um05517	-0.14	8.57
3d00041	0.12	13.60	um05518	0.18	12.46
3d00042	-0.43	14.53	um05520	-0.44	11.55
3d00043	0.59	14.19	um05521	1.33	13.92
3d00044	0.14	11.99	um05522	0.85	12.48
3d00045	-0.06	11.31	um05525	-0.23	12.07
3d00046	0.00	13.95	um05526	-0.32	11.96
3d00047	0.67	15.50	um05527	-0.23	12.07
3d00048	0.14	11.46	um05528	-2.70	9.87
3d00049	0.51	11.73	um05531	-0.48	11.99
3d00050	-0.96	13.75	um05533	-0.29	12.29
3d00051	0.30	11.27	um05537	0.11	11.59
3d00052	-0.43	15.25	um05538	0.15	11.70
3d00053	-0.01	11.89	um05539	0.18	10.47
3d00054	-0.60	13.51	um05540	-0.13	12.30
3d00055	-4.00	12.19	um05541	-0.57	10.78
3d00056	-0.23	14.08	um05542	0.05	11.06
3d00057	0.33	13.65	um05543	-0.64	11.38
3d00058	0.19	15.85	um05544	0.17	13.46
3d00059	-0.26	13.94	um05545	0.55	11.89
3d00060	0.23	12.57	um05546	-0.34	11.24
3d00061	-0.29	13.80	um05547	-0.15	10.68
3d00062	-0.11	13.84	um05548	-0.24	8.87
3d00063	0.05	9.13	um05549	-1.17	14.12
3d00064	0.01	12.56	um05550	-1.81	11.98
3d00065	0.33	12.77	um05552	-0.03	11.31
3d00066	0.02	11.65	um05554	0.46	11.92
3d00067	-0.33	9.51	um05555	0.29	12.76
3d00068	-0.13	12.59	um05557	0.10	11.29
3d00069	-0.42	10.38	um05558	0.97	13.05
3d00070	0.19	13.00	um05559	0.68	12.87
3d00071	0.19	14.16	um05560	-0.31	11.71
3d00072	-0.20	13.36	um05562	-0.34	8.88
3d00073	-4.00	8.62	um05563	-0.10	12.28
3d00074	-0.11	14.67	um05564	0.28	12.78
3d00075	0.33	13.77	um05567	0.19	14.60
3d00076	-1.55	14.60	um05568	0.02	11.70
3d00077	-0.44	13.59	um05569	-0.09	10.38
3d00078	-0.09	8.59	um05570	-0.71	11.74
3d00079	0.12	11.96	um05571	0.27	12.30
3d00080	-0.36	13.16	um05572	-0.25	11.22
3d00081	0.22	10.84	um05573	0.39	9.92
3d00082	0.24	11.40	um05574	0.86	12.00
3d00083	0.44	13.27	um05575	0.04	12.09
3d00084	-0.47	13.97	um05577	-0.18	11.45
3d00085	0.72	14.25	um05579	-0.07	13.04
3d00086	-1.66	13.44	um05580	-0.16	13.24
3d00087	-0.40	12.62	um05581	-2.38	14.51
3d00088	0.06	10.41	um05583	-0.04	13.81
3d00089	0.23	9.63	um05584	-0.37	14.76
3d00090	0.07	12.75	um05586	2.65	12.81
3d00091	0.12	13.23	um05588	0.30	10.91
3d00092	0.15	15.73	um05589	0.15	11.10

3d00093	-0.15	14.31	um05590	0.49	10.83
3d00094	0.12	10.12	um05591	1.23	10.05
3d00095	0.91	12.89	um05592	0.69	11.37
3d00096	0.12	9.79	um05593	1.03	11.08
3d00097	0.08	13.83	um05595	-0.10	14.53
3d00098	0.32	12.82	um05596	3.45	11.91
3d00099	-0.01	13.10	um05597	0.47	10.73
3d00100	0.32	13.76	um05598	-0.01	13.43
3d00101	-0.88	11.56	um05600	-0.27	10.41
3d00102	0.10	15.11	um05601	-0.25	10.09
482d000	-0.04	15.00	um05602	0.48	10.50
48c0000	-0.20	10.97	um05603	-0.14	10.17
4c00001	-0.25	11.02	um05604	-0.80	14.75
4c00002	-0.16	11.62	um05605	0.15	11.33
4c00003	0.20	10.31	um05606	-0.49	11.78
4c00004	0.01	10.61	um05607	-0.34	11.90
4c00005	0.12	9.62	um05608	0.56	11.44
4c00006	-0.30	13.45	um05609	0.19	11.79
4c00007	-0.15	10.89	um05610	-0.28	13.35
4c00008	-0.68	11.87	um05612	0.57	11.13
4c00009	0.15	12.29	um05614	0.23	9.82
4c00010	0.38	13.92	um05617	2.31	12.44
4c00011	-0.32	12.56	um05619	0.49	11.97
4c00012	0.32	12.63	um05621	-0.10	10.60
4c00013	0.14	10.75	um05624	0.50	11.10
4c00014	-0.23	12.41	um05625	-0.11	13.98
4c00015	-0.32	12.15	um05626	-0.21	11.50
4c00016	0.09	11.74	um05627	-0.40	12.37
4c00017	-0.09	9.56	um05628	0.56	11.51
4c00018	0.25	9.23	um05629	0.26	11.08
4c00019	0.00	13.84	um05631	-0.62	10.03
4c00020	0.26	13.26	um05632	0.83	14.57
4c00021	-0.23	12.00	um05633	-0.38	9.33
4c00022	0.44	12.33	um05635	-0.37	10.44
4c00023	-0.18	11.96	um05636	0.44	11.73
4c00024	0.08	11.83	um05640	-0.42	12.91
4c00025	0.25	13.11	um05641	-0.26	12.35
4c00026	0.17	12.81	um05642	0.14	11.66
4c00027	0.32	10.80	um05644	0.58	13.84
4c00028	-1.38	14.41	um05645	-0.02	12.58
4c00029	0.47	12.77	um05646	-0.15	11.88
4c00030	0.21	11.13	um05647	-0.29	10.98
4c00031	-0.12	11.57	um05648	-0.03	10.31
4c00032	-0.47	11.59	um05649	-0.24	11.21
4c00033	0.19	12.57	um05651	0.20	11.04
4c00034	-0.09	8.99	um05652	-0.59	13.98
4c00035	1.87	13.91	um05653	0.04	10.79
4c00036	0.65	14.20	um05654	0.33	12.59
4c00037	-0.17	12.47	um05656	0.13	12.58
4c00038	0.13	13.03	um05658	0.79	11.26
4c00039	0.39	11.50	um05659	-0.06	11.24
4c00040	-0.10	12.66	um05660	-0.21	10.47
4c00041	-1.57	14.26	um05662	-0.13	14.76
4c00042	0.36	13.11	um05663	0.88	14.51

4c00043	-0.13	8.96	um0566	1.38	11.94
4d00001	0.66	11.18	um0566	-0.45	13.00
4d00002	0.04	12.24	um0567	-1.28	12.54
4d00003	-0.37	10.63	um0567	-0.09	12.77
4d00004	0.17	11.63	um0567	0.35	12.32
4d00005	-0.02	11.74	um0567	-1.15	11.53
4d00006	-0.25	12.95	um0567	-0.72	13.73
4d00007	-4.00	8.75	um0567	0.84	12.90
4d00008	-0.16	11.66	um0567	0.26	11.50
4d00009	-0.01	12.79	um0568	0.14	12.59
4d00010	-0.01	13.51	um0568	-0.14	10.88
4d00011	0.52	13.58	um0568	0.26	12.11
4d00012	-0.10	11.35	um0568	0.86	14.14
4d00013	0.50	13.99	um0568	-0.73	12.95
4d00014	0.56	10.72	um0568	-0.39	12.13
4d00015	1.45	11.58	um0568	-2.50	13.43
4d00016	0.32	13.94	um0569	1.23	13.59
4d00017	-0.60	11.92	um0569	0.86	9.73
4d00018	-0.34	12.11	um0569	-0.70	11.16
4d00019	-0.07	12.75	um0569	-0.23	14.54
4d00020	-0.03	13.09	um0569	0.49	9.30
4d00021	-0.06	11.59	um0569	-0.07	12.66
4d00022	-0.27	11.94	um0569	0.24	12.44
4d00023	0.37	12.89	um0570	0.28	10.99
4d00024	0.11	12.01	um0570	-0.12	11.91
4d00025	0.06	12.42	um0570	-0.23	11.37
4d00026	-0.09	8.97	um0570	0.50	11.32
4d00027	-0.07	12.35	um0570	1.27	13.67
4d00028	-0.03	8.73	um0570	-0.08	11.60
4d00029	-0.26	14.91	um0570	-0.17	8.85
4d00030	-0.24	13.56	um0570	-0.46	15.91
4d00031	0.24	11.12	um0570	0.01	12.48
4d00032	-0.19	12.98	um0571	-0.32	11.57
4d00033	-0.03	12.47	um0571	0.39	11.90
4d00034	-0.02	9.70	um0571	-1.00	12.43
4d00035	0.31	11.54	um0571	-0.08	12.05
4d00036	0.19	8.61	um0572	0.15	11.70
4d00037	-0.05	8.82	um0572	0.07	11.25
4d00038	-0.14	12.32	um0572	0.11	11.29
4d00039	2.35	12.93	um0572	-0.34	10.13
4d00040	-0.38	13.48	um0572	-0.20	12.79
4d00041	0.66	11.58	um0572	0.18	10.84
4d00042	0.96	14.71	um0572	0.59	13.35
4d00043	-0.16	13.16	um0572	0.92	11.65
4d00044	0.18	10.54	um0573	0.58	13.76
4d00045	-0.38	13.96	um0573	-0.30	11.53
529c000	0.01	15.95	um0573	0.50	12.85
536c000	0.11	8.61	um0573	-0.18	14.89
5c00001	-0.30	11.39	um0573	0.10	12.07
5c00002	-0.39	12.73	um0573	-0.06	11.44
5c00003	-0.77	11.29	um0573	0.23	11.49
5c00004	-1.01	14.42	um0574	-0.40	11.79
5c00005	0.47	11.78	um0574	-0.07	12.99
5c00006	0.37	10.49	um0574	-4.00	13.58

5c00007	0.43	10.17	um05745	-0.32	11.94
5c00008	0.00	15.80	um05746	0.63	11.99
5c00009	0.22	12.67	um05747	-0.33	12.46
5c00010	-0.11	10.38	um05748	-0.29	13.21
5c00011	0.19	13.11	um05749	-0.26	10.34
5c00012	0.39	11.67	um05750	0.11	10.56
5c00013	-0.09	8.67	um05751	1.69	11.98
5c00014	-0.37	11.86	um05753	0.18	10.79
5c00015	-0.16	9.21	um05754	0.23	13.91
5c00016	-0.10	15.91	um05755	0.59	10.50
5c00017	-0.41	15.23	um05756	-0.57	10.72
5c00018	0.53	11.77	um05758	0.12	11.21
5c00019	0.19	9.35	um05760	-0.26	9.61
5c00020	0.52	12.66	um05761	0.03	13.60
5c00021	-1.26	10.96	um05764	-0.67	9.26
5c00022	0.10	9.01	um05766	-1.47	14.15
5c00023	0.33	13.18	um05767	0.14	11.69
5c00024	0.08	10.58	um05769	-0.25	13.23
5c00025	0.16	12.52	um05770	-0.34	10.48
5c00026	-0.49	13.60	um05771	0.33	12.30
5c00027	0.29	12.25	um05772	0.15	9.04
5c00028	-0.51	12.48	um05773	0.29	11.32
5c00029	-0.31	13.59	um05774	0.01	10.50
5c00030	-0.74	12.84	um05776	0.57	13.20
5c00031	0.33	13.63	um05777	-0.36	11.04
5c00032	-4.00	12.77	um05780	-0.08	9.19
5c00033	-0.02	11.74	um05781	-0.29	8.81
5c00034	0.49	13.54	um05782	0.07	10.56
5c00035	0.73	13.20	um05783	-0.36	8.75
5c00036	-4.00	12.83	um05785	-0.70	9.07
5c00037	-0.15	10.77	um05786	-1.40	9.48
5c00038	0.68	13.42	um05787	-0.61	10.15
5c00039	-0.13	12.54	um05789	0.03	12.55
5c00040	-0.75	11.59	um05790	0.87	11.19
5c00041	0.32	12.84	um05791	4.08	13.10
5c00042	-0.34	13.01	um05792	0.79	9.86
5c00043	-0.18	13.89	um05794	0.49	10.35
5c00044	0.47	15.01	um05795	0.70	11.07
5c00045	0.06	9.39	um05796	-0.32	8.40
5c00046	0.88	14.15	um05798	-0.57	8.83
5c00047	1.00	15.40	um05799	-0.43	9.26
5c00048	-0.08	9.71	um05800	0.07	11.73
5c00049	-0.12	11.59	um05801	-0.10	10.62
5c00050	-0.02	12.28	um05802	1.23	10.05
5c00051	0.25	12.65	um05803	0.11	9.69
5c00052	0.34	12.44	um05804	-0.09	9.89
5c00053	0.25	8.88	um05805	1.23	11.79
5c00054	-0.01	9.12	um05806	-0.37	9.89
5c00055	0.31	13.44	um05807	0.54	9.27
5c00056	-0.45	12.50	um05809	-0.27	8.98
5c00057	-0.63	11.49	um05811	-1.71	12.12
5c00058	0.18	9.89	um05812	3.76	11.85
5c00059	-0.19	14.17	um05814	0.13	9.28
5c00060	0.74	12.09	um05818	-0.62	12.43

5c00061	-0.53	12.33	um05819	0.07	8.54
5c00062	-0.45	14.02	um05820	0.33	12.68
5c00063	-0.17	8.69	um05821	0.02	10.92
5c00064	0.19	13.02	um05822	0.51	10.36
5c00065	-0.12	12.59	um05824	-0.14	8.87
5c00066	-0.16	12.71	um05825	-0.41	13.56
5c00067	0.00	12.63	um05827	-0.03	12.27
5c00068	0.03	9.87	um05828	-0.29	15.60
5c00069	-0.29	12.05	um05829	-0.34	11.65
5c00070	0.13	11.08	um05830	0.26	9.92
5c00071	-1.02	14.62	um05831	-0.62	13.39
5c00072	0.07	13.35	um05832	-0.40	12.41
5c00073	-1.12	11.92	um05833	0.10	11.61
5c00074	0.91	14.86	um05834	0.02	10.89
5c00075	-0.18	12.76	um05835	-0.66	11.23
5c00076	0.21	10.21	um05836	-0.02	12.17
5c00077	0.11	12.49	um05839	-0.21	11.18
5c00078	0.28	12.75	um05840	0.71	11.15
5c00079	-0.18	12.41	um05841	-0.13	12.23
5c00080	-0.09	12.05	um05842	0.38	10.91
5c00081	0.40	12.61	um05844	0.57	12.39
5c00082	0.15	11.77	um05845	-0.36	11.98
5c00083	-0.27	12.83	um05846	0.12	12.03
5c00084	-0.49	12.38	um05847	0.08	12.37
5c00085	0.22	11.99	um05848	-0.15	11.73
5c00086	-0.41	9.59	um05849	0.13	11.09
5c00087	0.38	12.12	um05850	0.36	12.85
5c00088	0.73	11.42	um05852	0.54	10.85
5c00089	0.19	14.51	um05853	0.38	11.04
5c00090	-0.14	11.41	um05854	-0.15	11.54
5c00091	0.10	9.99	um05856	0.44	12.17
5c00092	-0.04	10.59	um05857	-0.32	10.55
5c00093	0.07	8.62	um05858	0.06	11.11
5c00094	-0.44	11.17	um05860	-0.46	12.90
5c00095	0.28	12.54	um05861	-0.51	9.37
5c00096	-0.08	10.13	um05862	-0.14	10.46
5c00097	0.39	12.27	um05863	0.09	13.65
5c00098	0.34	12.17	um05869	-0.19	13.06
5c00099	-0.02	9.87	um05870	-0.08	12.35
5c00100	0.37	13.80	um05872	-0.03	11.49
5c00101	0.28	12.63	um05873	0.03	12.03
5c00102	0.08	9.13	um05876	-0.17	11.29
5c00103	0.33	12.39	um05878	1.17	13.18
5c00104	-0.65	12.32	um05879	-0.38	12.39
5c00105	0.76	12.91	um05880	-1.25	13.96
5c00106	0.09	11.76	um05883	0.31	14.79
5c00107	0.29	13.05	um05888	0.14	11.30
5c00108	0.13	9.45	um05889	-0.77	15.82
5c00109	0.15	12.48	um05890	-0.26	11.69
5c00110	0.39	13.09	um05891	0.52	11.68
5c00111	0.01	8.72	um05892	0.62	11.21
5c00112	0.05	8.63	um05893	-0.42	13.01
5c00113	0.02	12.45	um05894	0.50	10.10
5c00114	0.00	9.59	um05895	0.13	10.19

5c00115	0.58	12.25	um05896	0.13	11.55
5c00116	-0.20	11.72	um05897	-0.57	12.51
5c00117	0.36	11.15	um05898	0.28	12.61
5c00118	-0.14	10.51	um05899	0.33	10.94
5c00119	0.01	8.86	um05900	-0.65	11.84
5c00120	0.01	15.83	um05901	-0.08	11.20
5c00121	-0.06	10.35	um05906	0.08	13.71
5c00122	-0.72	14.01	um05907	0.24	12.14
5c00123	-0.06	15.72	um05908	-0.60	12.33
5c00124	-0.20	13.47	um05910	0.25	12.29
5c00125	0.36	12.20	um05911	-0.46	10.26
5c00126	-0.01	12.43	um05912	-0.19	10.51
5c00127	-0.11	11.11	um05915	0.48	11.46
5c00128	0.00	13.47	um05916	-0.10	11.42
5c00129	-0.18	12.18	um05917	0.25	10.15
5c00130	-0.12	11.39	um05919	-0.27	12.48
5c00131	-0.54	12.36	um05921	0.35	11.46
5c00132	0.93	13.67	um05922	0.19	11.54
5c00133	-0.59	11.80	um05923	1.01	11.52
5c00134	-0.02	12.36	um05924	-0.01	12.28
5c00135	-0.24	11.49	um05925	0.74	10.91
5c00136	-0.62	11.89	um05926	-0.24	8.86
5c00137	-0.44	10.99	um05927	0.02	8.79
5c00138	-0.34	12.90	um05928	-0.18	8.83
5c00139	0.32	11.82	um05929	-0.26	8.49
5c00140	0.19	12.13	um05930	-0.15	8.45
5c00141	-0.64	11.27	um05931	-0.06	8.50
5c00142	-0.04	9.65	um05932	-0.02	9.44
5c00143	-0.01	8.59	um05933	-0.97	11.62
5c00144	0.01	8.79	um05934	0.14	11.90
5c00145	-0.15	8.57	um05936	1.07	12.34
5c00146	0.00	8.78	um05937	-0.13	11.07
5c00147	0.14	11.41	um05938	1.98	10.90
5c00148	-0.17	11.97	um05939	0.43	11.03
5c00149	-0.36	9.23	um05940	0.30	12.79
5c00150	0.60	13.53	um05942	0.75	10.48
5c00151	-0.30	11.41	um05944	1.27	11.84
5c00152	0.37	12.31	um05946	0.41	9.71
5c00153	-0.17	11.54	um05947	-0.15	12.59
5c00154	0.74	12.44	um05948	0.19	8.96
5c00155	-0.75	10.97	um05949	-0.39	13.16
5c00156	0.22	10.67	um05951	-1.15	10.01
5c00157	0.15	12.46	um05952	1.29	13.21
5c00158	0.20	10.13	um05953	1.39	13.67
5c00159	-0.90	10.21	um05954	-0.09	11.87
5c00160	0.06	14.05	um05958	1.58	11.06
5c00161	0.00	9.20	um05959	0.37	10.00
5c00162	-0.03	8.68	um05960	0.61	12.00
5c00163	-0.95	12.14	um05961	-0.95	11.97
5c00164	-0.22	14.16	um05964	0.05	8.84
5c00165	0.00	13.07	um05965	-0.36	13.83
5d00001	-0.31	12.00	um05966	1.24	12.72
5d00002	0.23	15.64	um05967	2.38	11.36
5d00003	0.13	13.33	um05968	-0.23	9.91

5d00004	0.14	13.71	um05970	-0.89	12.62
5d00005	-0.37	10.80	um05971	-0.64	14.71
5d00006	0.19	12.79	um05972	1.75	11.39
5d00007	-0.22	14.65	um05974	0.02	14.63
5d00008	-0.45	13.75	um05976	0.62	9.75
5d00009	0.14	12.74	um05977	0.76	12.52
5d00010	-0.08	8.45	um05978	0.06	12.43
5d00011	0.06	8.61	um05979	-0.47	15.88
5d00012	0.43	13.44	um05981	-0.14	10.03
5d00013	0.16	9.88	um05982	1.19	11.72
5d00014	-0.09	12.18	um05983	1.24	11.64
5d00015	-0.58	14.68	um05984	1.10	13.10
5d00016	0.41	10.58	um05985	0.97	12.17
5d00017	-0.18	12.34	um05986	-0.37	13.26
5d00018	-0.08	13.09	um05987	0.11	13.00
5d00019	0.04	10.02	um05988	0.86	11.44
5d00020	0.20	10.96	um05989	0.10	12.09
5d00021	-0.03	8.97	um05991	0.28	11.43
5d00022	-0.60	13.53	um05992	-0.51	13.22
5d00023	0.12	8.52	um05994	-0.09	10.67
5d00024	0.90	12.11	um05995	0.33	11.03
5d00025	-0.23	12.45	um05997	-1.13	11.13
5d00026	-4.65	12.97	um05998	0.38	14.55
5d00027	-0.26	9.85	um05999	0.46	12.38
5d00028	-0.04	9.45	um06000	0.39	11.58
5d00029	0.33	13.17	um06001	-0.17	12.73
5d00030	-0.09	12.68	um06002	0.68	11.40
5d00031	0.13	13.49	um06003	-0.28	13.41
5d00032	-0.09	8.73	um06004	0.58	14.04
5d00033	-0.33	12.04	um06008	0.32	13.35
5d00034	0.03	14.27	um06009	-0.39	11.63
5d00035	1.61	10.77	um06010	1.28	13.55
5d00036	0.37	12.10	um06012	2.48	10.43
5d00037	-0.19	13.06	um06013	-0.55	11.12
5d00038	-0.38	12.77	um06014	-0.03	12.46
5d00039	-0.06	12.90	um06018	-0.58	12.50
5d00040	0.01	11.50	um06019	0.34	12.68
5d00041	0.43	12.06	um06020	0.52	13.00
5d00042	-0.27	13.22	um06022	-0.50	13.38
5d00043	-0.09	9.42	um06027	-0.55	12.25
5d00044	0.14	10.89	um06029	-0.34	11.62
5d00045	0.61	13.27	um06031	0.49	12.23
5d00046	0.06	9.19	um06032	-0.06	12.81
5d00047	0.11	13.09	um06033	0.72	12.18
5d00048	0.22	12.41	um06034	-0.40	12.93
5d00049	0.39	13.02	um06035	-0.02	12.65
5d00050	-0.01	13.17	um06036	0.26	10.78
5d00051	-0.06	9.86	um06037	0.44	11.12
5d00052	-0.58	13.71	um06038	-0.44	12.09
5d00053	0.40	11.67	um06040	0.67	13.02
5d00054	0.77	12.64	um06042	-4.30	11.18
5d00055	0.32	11.74	um06043	-0.15	11.05
5d00056	-0.08	10.59	um06045	-0.27	12.62
5d00057	0.03	12.11	um06046	0.28	11.65

5d00058	-0.19	11.35	um06047	-0.61	12.05
5d00059	-0.93	13.96	um06048	-0.86	12.26
5d00060	-0.12	11.03	um06049	0.11	12.61
5d00061	-0.34	10.52	um06050	-0.06	11.09
5d00062	0.34	12.74	um06051	0.13	13.46
5d00063	0.71	14.03	um06053	-0.28	11.17
5d00064	-0.23	13.45	um06055	0.22	14.78
5d00065	-0.24	12.65	um06056	-1.06	10.70
5d00066	-0.24	11.70	um06058	-0.09	12.19
5d00067	0.29	13.24	um06059	1.39	11.85
5d00068	-0.15	12.53	um06060	-0.16	12.80
5d00069	-0.07	12.14	um06061	0.37	11.32
5d00070	-0.62	11.90	um06062	0.03	12.01
5d00071	0.20	12.42	um06063	0.83	13.50
5d00072	0.36	12.37	um06064	1.61	13.84
5d00073	-0.40	13.38	um06065	-0.02	11.63
5d00074	-0.22	11.76	um06066	0.16	11.81
5d00075	-0.60	13.29	um06067	-0.24	12.99
5d00076	0.30	12.67	um06068	0.01	12.91
5d00077	-0.35	12.95	um06069	0.44	11.92
5d00078	-0.80	12.44	um06070	-0.73	15.31
5d00079	0.43	12.17	um06071	0.14	10.18
5d00080	-0.01	10.66	um06072	-0.81	11.65
5d00081	0.19	12.49	um06073	0.88	10.02
5d00082	-0.07	15.93	um06075	0.14	9.06
5d00083	0.28	11.42	um06076	-0.69	9.61
5d00084	0.59	10.97	um06077	-0.77	9.91
5d00085	0.13	14.00	um06078	-1.63	12.92
5d00086	0.41	12.34	um06079	0.65	9.87
5d00087	0.30	12.86	um06080	-0.11	12.05
5d00088	0.27	13.55	um06082	0.71	13.77
5d00089	0.10	12.63	um06083	-0.04	12.36
5d00090	-0.53	12.84	um06084	3.60	13.70
5d00091	0.60	15.26	um06085	4.09	13.32
5d00092	0.64	12.05	um06086	0.20	12.72
5d00093	0.32	10.21	um06087	-0.17	11.39
5d00094	-0.01	8.63	um06088	-0.44	11.86
5d00095	0.12	12.76	um06089	0.06	12.37
5d00096	0.77	13.25	um06090	0.07	11.88
5d00097	0.24	11.31	um06091	0.86	9.97
5d00098	-0.02	13.02	um06092	0.98	11.18
5d00099	-0.02	13.96	um06093	-0.13	11.58
5d00100	-0.26	10.97	um06097	0.58	11.77
5d00101	0.06	11.74	um06098	-1.41	13.88
5d00102	-0.10	12.43	um06102	0.15	14.53
5d00103	-0.48	13.23	um06103	0.66	12.37
5d00104	-0.12	9.68	um06105	-0.87	14.04
5d00105	0.49	12.36	um06107	0.31	12.44
5d00106	0.42	13.00	um06111	-0.58	13.84
5d00107	0.10	8.93	um06112	-0.45	8.52
5d00108	0.12	10.96	um06113	-0.20	8.66
5d00109	0.05	12.67	um06116	0.05	13.53
5d00110	0.01	13.58	um06117	-0.15	12.77
5d00111	0.02	9.73	um06118	-0.18	10.95

5d00112	0.05	11.89	um06119	-0.73	9.49
5d00113	0.11	13.29	um06120	1.51	10.54
5d00114	0.09	12.12	um06121	-0.72	12.33
5d00115	0.28	12.79	um06124	0.85	9.17
5d00116	0.15	11.58	um06125	-1.78	13.52
5d00117	-0.04	9.20	um06126	-0.21	9.20
5d00118	0.85	10.50	um06127	-0.15	8.48
5d00119	0.13	9.31	um06128	-0.43	9.77
5d00120	-0.53	13.51	um06129	0.49	11.54
5d00121	-0.15	13.39	um06130	0.71	11.49
5d00122	1.04	12.95	um06131	-0.31	10.34
5d00123	0.07	12.21	um06133	-2.23	10.23
5d00124	0.33	14.18	um06134	-0.07	11.73
5d00125	-0.33	12.65	um06135	-0.14	13.34
5d00126	0.33	12.84	um06138	0.89	14.70
5d00127	-0.06	10.75	um06139	-0.65	12.59
5d00128	-0.06	11.57	um06140	0.23	11.90
5d00129	-0.23	12.87	um06141	0.17	11.06
5d00130	0.05	12.23	um06143	-0.08	11.24
5d00131	-0.81	9.69	um06146	-3.12	14.78
5d00132	-1.02	12.40	um06148	-0.08	11.68
5d00133	-0.31	11.61	um06151	0.60	11.73
5d00134	-0.14	9.62	um06152	-0.03	12.58
5d00135	-0.85	14.19	um06153	0.01	10.84
5d00136	-0.25	12.26	um06154	0.28	10.86
5d00137	0.50	13.44	um06155	0.29	11.22
5d00138	0.40	11.47	um06156	-0.39	11.02
5d00139	0.06	10.56	um06157	0.67	13.21
5d00140	-0.05	8.60	um06158	0.06	11.94
5d00141	-0.23	10.92	um06159	-0.02	12.75
5d00142	-0.29	11.99	um06162	-0.21	14.79
5d00143	0.09	11.35	um06163	0.78	12.48
5d00144	0.14	9.62	um06164	0.10	11.34
5d00145	-0.02	10.98	um06165	-0.47	9.30
5d00146	-0.40	11.01	um06167	-0.77	13.14
5d00147	0.32	13.17	um06168	-0.32	10.98
5d00148	0.10	9.58	um06169	0.22	11.22
6c00001	-0.06	15.91	um06174	-0.51	11.02
6c00002	-0.30	15.19	um06175	-0.17	13.69
6c00003	-0.05	11.60	um06178	-0.22	8.77
6c00004	0.07	10.17	um06179	-0.21	8.64
6c00005	-0.63	9.63	um06180	-0.16	8.71
6c00006	-0.05	13.76	um06181	-0.27	8.79
6c00007	-0.23	10.42	um06182	-0.52	14.51
6c00008	-2.09	10.33	um06183	0.18	11.17
6c00009	-0.23	10.09	um06184	-0.20	11.14
6c00010	0.01	8.96	um06185	0.62	14.19
6c00011	-0.05	12.81	um06186	0.77	12.66
6c00012	-0.12	9.67	um06187	-0.03	11.34
6c00013	0.37	11.44	um06188	-0.46	13.35
6c00014	0.03	10.29	um06189	0.09	11.55
6c00015	-0.92	12.31	um06190	-1.76	9.73
6c00016	-1.35	12.26	um06191	0.02	10.29
6c00017	0.28	9.35	um06193	-0.15	11.51

6c00018	0.30	11.78	um06194	0.53	12.61
6c00019	0.00	13.10	um06195	-0.21	11.45
6c00020	-0.45	14.92	um06197	0.14	11.10
6c00021	0.26	11.89	um06200	-0.08	11.63
6c00022	0.66	9.94	um06201	-0.14	11.80
6c00023	0.23	13.76	um06203	1.84	12.19
6c00024	0.44	12.50	um06204	-0.09	8.82
6c00025	-0.04	12.77	um06205	-0.53	10.70
6c00026	-0.27	12.73	um06206	0.25	11.32
6c00027	0.18	12.68	um06210	0.14	11.94
6c00028	0.06	8.64	um06211	-0.10	11.47
6c00029	0.13	8.78	um06212	-0.20	12.58
6c00030	0.18	11.05	um06213	-0.41	11.34
6c00031	-0.36	11.05	um06215	-0.18	10.88
6c00032	-0.69	12.91	um06218	-0.53	14.79
6c00033	-0.37	12.83	um06219	0.54	12.01
6c00034	0.50	12.08	um06221	-0.09	8.86
6c00035	0.11	11.90	um06222	0.02	8.72
6c00036	-0.51	12.85	um06223	-0.36	8.71
6c00037	0.10	14.91	um06224	-0.46	11.73
6c00038	0.16	13.46	um06226	0.02	12.87
6c00039	0.02	12.70	um06228	0.16	10.60
6c00040	-0.66	13.81	um06229	-0.07	10.72
6c00041	0.05	13.08	um06231	1.18	14.09
6c00042	1.44	14.67	um06233	0.08	12.60
6c00043	-0.61	14.13	um06234	-0.25	13.61
6c00044	-0.10	9.01	um06235	-0.15	13.22
6c00045	-0.10	13.38	um06236	0.22	12.16
6c00046	-0.01	8.46	um06237	0.25	11.59
6c00047	0.02	10.81	um06239	0.28	11.34
6c00048	-0.09	11.49	um06241	0.04	12.66
6c00049	-0.36	10.35	um06243	0.10	10.55
6c00050	-0.57	12.00	um06248	-0.11	8.79
6c00051	-0.26	9.92	um06249	-0.09	12.37
6c00052	0.65	14.46	um06251	-0.28	12.57
6c00053	-0.92	12.77	um06253	0.10	9.45
6c00054	0.10	12.43	um06255	-0.24	8.55
6c00055	-0.04	9.73	um06256	0.22	10.53
6c00056	-0.91	13.15	um06257	-0.02	10.94
6c00057	0.01	8.87	um06258	0.49	12.36
6c00058	-0.20	10.90	um06259	-0.44	11.95
6c00059	0.71	10.82	um06260	0.18	10.81
6c00060	-0.04	14.57	um06261	-0.34	11.22
6c00061	0.43	11.84	um06265	0.18	9.90
6c00062	-0.38	12.87	um06266	-1.15	13.25
6c00063	-0.33	14.06	um06267	0.06	8.86
6c00064	-0.66	12.63	um06268	0.62	12.33
6c00065	0.03	11.41	um06269	-0.37	12.02
6c00066	-0.05	12.07	um06270	0.06	10.86
6c00067	-0.01	11.85	um06271	-0.19	10.95
6c00068	-0.23	11.29	um06273	-0.57	12.60
6c00069	0.85	13.91	um06274	-0.89	12.28
6c00070	-0.03	12.48	um06275	0.37	12.63
6c00071	0.22	15.43	um06276	0.44	12.52

6c00072	0.04	10.68	um06278	1.00	11.94
6c00073	-0.04	12.85	um06279	-0.25	9.18
6c00074	-0.15	10.84	um06280	-0.13	11.37
6c00075	-0.10	12.54	um06281	0.22	13.77
6c00076	0.55	13.69	um06284	0.28	12.24
6c00077	0.21	11.68	um06285	-0.70	13.33
6c00078	-0.66	13.23	um06287	0.10	11.39
6c00079	-1.46	13.53	um06288	-0.30	12.61
6c00080	-0.29	12.78	um06290	-0.13	8.60
6c00081	-0.01	9.65	um06292	0.09	12.41
6c00082	0.11	12.42	um06293	0.41	9.61
6c00083	0.43	12.61	um06294	-0.03	11.85
6c00084	-1.04	11.19	um06295	0.18	11.02
6c00085	0.08	12.40	um06296	0.17	11.41
6c00086	0.60	14.06	um06298	0.51	12.13
6c00087	0.33	11.48	um06299	-0.32	9.89
6c00088	-0.21	10.78	um06302	-0.39	12.63
6c00089	-0.86	12.81	um06304	0.06	12.02
6c00090	-1.17	12.97	um06305	0.12	10.99
6c00091	0.75	12.98	um06306	-0.08	11.99
6c00092	-0.94	11.46	um06308	0.14	11.75
6c00093	-0.39	12.45	um06312	-0.29	11.81
6c00094	-1.42	12.17	um06314	-0.46	12.61
6c00095	0.14	11.34	um06315	0.14	9.67
6c00096	-0.51	13.78	um06316	-0.16	11.02
6c00097	-0.14	15.74	um06317	0.35	11.54
6c00098	-0.26	12.30	um06323	-0.11	12.51
6c00099	0.21	10.72	um06324	-0.37	14.58
6c00100	0.14	11.98	um06326	0.39	9.71
6c00101	0.03	12.34	um06329	-0.20	12.63
6c00102	-0.11	12.26	um06332	2.25	12.41
6c00103	-0.24	12.07	um06333	-0.67	11.94
6c00104	-0.13	10.73	um06334	0.16	12.01
6c00105	-0.22	12.16	um06335	0.63	12.65
6c00106	-0.68	11.64	um06336	0.01	13.68
6c00107	-0.69	12.78	um06337	0.36	11.80
6c00108	0.16	10.99	um06338	-0.36	12.04
6c00109	0.53	13.05	um06341	0.35	11.44
6c00110	0.22	10.11	um06344	-0.31	14.45
6c00111	-0.20	13.08	um06345	-0.25	12.40
6c00112	-0.04	12.37	um06346	0.93	10.08
6c00113	-0.61	14.01	um06347	-0.32	10.92
6c00114	-0.18	12.09	um06348	-0.35	12.88
6c00115	-0.05	13.15	um06349	0.00	11.61
6c00116	0.31	12.76	um06350	-0.03	8.75
6c00117	0.24	13.67	um06351	-0.80	11.01
6c00118	-0.10	12.57	um06356	0.09	10.86
6c00119	0.07	13.40	um06357	0.23	14.32
6c00120	0.04	13.26	um06358	0.49	11.47
6c00121	-0.22	12.98	um06359	3.17	11.14
6c00122	1.02	10.66	um06360	0.27	13.53
6c00123	-0.15	10.34	um06362	-0.31	11.65
6c00124	0.13	10.25	um06363	0.00	11.82
6c00125	-0.02	13.05	um06365	-0.26	12.77

6c00126	-0.41	11.55	um06366	-0.22	10.16
6c00127	0.18	9.64	um06367	-1.96	11.09
6c00128	0.32	12.37	um06368	-0.65	12.22
6c00129	0.04	9.74	um06370	-0.26	8.55
6c00130	-0.33	13.29	um06371	-0.61	10.89
6c00131	0.05	11.32	um06372	0.12	11.82
6c00132	-0.05	12.23	um06374	-0.34	12.11
6c00133	0.05	11.73	um06376	-0.29	14.06
6c00134	-0.16	9.32	um06377	0.29	12.40
6c00135	0.16	12.59	um06378	0.95	12.03
6c00136	0.30	12.79	um06381	-0.84	11.01
6c00137	-0.14	10.90	um06383	0.27	11.98
6d00001	-0.13	10.68	um06384	0.06	8.62
6d00002	0.52	9.56	um06385	-0.44	13.17
6d00003	-0.38	9.96	um06386	0.09	11.83
6d00004	-2.69	14.22	um06387	0.34	12.25
6d00005	0.10	13.80	um06388	-0.22	12.45
6d00006	0.10	9.60	um06389	0.44	12.34
6d00007	-0.02	12.56	um06390	0.45	10.29
6d00008	-0.31	10.09	um06391	0.73	11.76
6d00009	-0.82	10.62	um06393	-0.18	14.41
6d00010	0.08	12.80	um06394	-0.07	12.87
6d00011	-0.14	11.62	um06395	0.37	12.38
6d00012	-0.29	9.85	um06396	0.57	11.38
6d00013	-0.12	8.85	um06397	0.55	11.14
6d00014	0.30	12.32	um06398	-0.11	11.52
6d00015	-0.37	11.05	um06399	0.52	12.20
6d00016	-0.06	10.67	um06400	2.35	13.75
6d00017	-0.55	13.99	um06401	-0.27	11.51
6d00018	0.11	13.62	um06402	-0.32	11.46
6d00019	-0.13	12.66	um06404	-1.28	15.28
6d00020	-0.28	12.19	um06405	0.37	13.63
6d00021	1.04	11.64	um06406	1.43	14.74
6d00022	-0.13	8.50	um06407	-0.48	11.15
6d00023	0.15	12.24	um06408	0.36	11.48
6d00024	0.11	9.41	um06410	0.20	11.56
6d00025	0.00	8.73	um06411	-0.34	10.32
6d00026	-0.10	10.80	um06412	-0.19	11.40
6d00027	-1.46	12.55	um06413	-0.05	11.61
6d00028	0.31	10.75	um06414	-0.18	9.62
6d00029	-0.10	12.09	um06416	-0.17	11.63
6d00030	-0.57	11.30	um06417	-0.26	11.05
6d00031	-0.53	12.84	um06418	-0.72	9.44
6d00032	-0.30	9.39	um06419	-0.19	8.78
6d00033	-0.13	8.42	um06420	-0.04	11.32
6d00034	-0.09	8.59	um06421	0.33	13.41
6d00035	0.23	8.59	um06422	1.28	13.56
6d00036	0.00	9.33	um06425	-0.02	11.26
6d00037	-0.41	11.78	um06426	-0.93	12.87
6d00038	-1.19	11.27	um06427	-0.75	10.95
6d00039	0.07	12.92	um06428	-0.31	9.63
6d00040	0.26	11.46	um06429	0.22	12.92
6d00041	-0.23	9.24	um06430	-0.70	12.07
6d00042	0.09	12.73	um06431	0.35	10.22

6d00043	-0.08	12.47	um06433	0.19	8.90
6d00044	0.37	12.71	um06434	-0.03	12.31
6d00045	0.42	9.59	um06435	-0.06	8.59
6d00046	0.99	10.99	um06436	0.71	9.90
6d00047	-0.29	12.57	um06437	2.32	12.90
6d00048	0.04	9.62	um06438	2.26	12.18
6d00049	0.75	14.00	um06439	-0.36	9.05
6d00050	-0.52	9.42	um06440	-0.90	12.12
6d00051	-0.05	8.87	um06443	-0.25	13.54
6d00052	-1.30	10.82	um06445	-0.22	12.47
6d00053	-0.16	12.43	um06448	0.16	15.37
6d00054	0.46	12.41	um06450	0.22	13.73
6d00055	-0.17	11.90	um06453	-2.59	12.76
6d00056	-0.46	11.90	um06454	-0.96	11.73
6d00057	-0.07	8.87	um06455	0.52	11.64
6d00058	-0.06	8.54	um06456	-1.70	10.52
6d00059	0.11	8.43	um06458	0.03	8.96
6d00060	-0.80	15.24	um06459	0.11	9.23
6d00061	0.18	11.89	um06460	-0.47	8.99
6d00062	-0.32	12.53	um06461	0.07	8.93
6d00063	-0.16	8.95	um06462	-0.02	9.27
6d00064	-0.10	8.62	um06464	-0.67	9.37
6d00065	0.26	10.53	um06465	-0.45	8.87
6d00066	0.17	12.46	um06466	-0.36	8.54
6d00067	0.01	13.05	um06467	-1.38	9.77
6d00068	-0.73	11.96	um06470	-2.68	9.91
6d00069	0.00	11.27	um06472	-0.51	12.53
6d00070	-1.20	14.60	um06473	0.08	9.34
6d00071	-0.26	11.91	um06474	0.75	12.34
6d00072	0.09	11.84	um06475	0.06	8.40
6d00073	-0.47	11.16	um06476	1.05	13.17
6d00074	-0.21	13.09	um06477	0.46	11.27
6d00075	0.31	12.77	um06478	-0.50	13.54
6d00076	0.06	8.70	um06479	0.18	11.06
6d00077	-0.18	11.03	um06480	0.41	11.84
6d00078	0.23	11.39	um06485	-1.79	10.35
6d00079	0.36	9.08	um06487	-2.71	10.29
6d00080	-0.39	11.72	um06489	-0.69	12.67
6d00081	-0.20	11.59	um06490	-1.75	9.41
6d00082	-0.87	10.94	um06491	0.37	11.10
6d00083	-0.45	10.72	um06493	0.47	11.12
6d00084	-0.08	8.87	um06497	0.63	13.15
6d00085	-0.15	9.51	um06500	-1.55	10.93
6d00086	0.23	8.68	um06503	-0.22	10.37
6d00087	-0.09	8.56	um06504	0.38	9.52
6d00088	-0.08	8.83	um06505	-0.38	13.34
6d00089	-0.64	13.26	um06506	0.49	10.50
6d00090	-0.05	11.68	um06507	-0.06	13.06
6d00091	-0.03	10.23	um06508	-1.17	9.31
6d00092	-0.06	12.18	um06509	-1.23	9.04
6d00093	-0.10	9.86	um06513	-0.28	11.92
6d00094	-0.22	9.88	um06514	-0.34	13.69
6d00095	-0.18	12.06	um10000	-0.25	12.13
6d00096	-0.47	14.13	um10001	0.38	11.40

6d00097	-0.16	14.67	um10002	0.72	10.39
6d00098	-0.10	13.67	um10003	0.79	15.25
6d00099	-0.14	9.19	um10004	0.38	12.57
6d00100	-0.26	13.00	um10005	-0.17	11.25
6d00101	-0.20	13.71	um10006	-0.29	11.53
6d00102	0.80	11.45	um10007	-0.77	11.12
6d00103	-0.31	10.09	um10008	-0.51	9.78
6d00104	-0.62	12.28	um10009	0.76	11.12
6d00105	-0.63	11.81	um10010	1.12	13.43
6d00106	0.48	11.64	um10011	0.46	12.99
6d00107	0.35	11.78	um10012	-0.18	12.96
6d00108	0.14	12.57	um10013	-0.28	11.88
6d00109	-0.23	12.61	um10014	-0.35	13.99
6d00110	0.03	12.95	um10015	0.15	11.01
6d00111	0.93	10.29	um10016	0.79	10.67
6d00112	-0.01	8.88	um10017	0.10	12.17
6d00113	-0.18	12.40	um10018	-0.56	12.06
6d00114	0.06	15.72	um10019	0.58	11.96
6d00115	-1.06	10.96	um10020	0.27	12.16
6d00116	0.03	10.54	um10021	-0.27	12.47
6d00117	-0.04	12.53	um10022	0.46	11.22
6d00118	0.10	12.20	um10023	-0.32	12.09
6d00119	0.33	12.94	um10024	-0.37	13.92
6d00120	-0.30	11.99	um10025	-0.06	11.45
6d00121	-0.33	9.43	um10027	0.35	11.88
6d00122	0.04	8.57	um10028	0.48	11.88
6d00123	0.08	12.66	um10029	-0.07	11.63
6d00124	0.36	11.72	um10030	-0.15	10.23
6d00125	0.04	12.63	um10031	1.32	13.09
6d00126	0.10	13.12	um10032	1.31	12.06
6d00127	0.20	13.23	um10033	0.37	12.50
6d00128	0.10	9.58	um10034	0.36	12.31
6d00129	-0.30	12.92	um10035	-0.39	14.48
6d00130	0.05	11.90	um10036	-0.14	11.00
6d00131	0.11	9.20	um10037	-0.25	13.71
6d00132	-0.11	11.39	um10038	0.67	14.44
6d00133	0.01	11.65	um10039	-0.70	12.48
6d00134	0.10	12.19	um10040	0.10	10.96
6d00135	0.07	13.43	um10041	-0.63	10.87
7c00001	-0.35	11.48	um10042	0.09	10.82
7c00002	0.22	12.92	um10043	0.10	10.52
7c00003	-0.98	10.91	um10044	-0.25	12.53
7c00004	0.30	9.92	um10045	0.03	9.02
7c00005	0.04	8.48	um10046	0.59	13.16
7c00006	0.58	10.02	um10047	0.33	10.84
7c00007	-0.52	9.30	um10048	-0.08	14.98
7c00008	-0.12	8.76	um10049	-0.08	10.95
7c00009	0.02	8.53	um10050	-0.15	12.56
7c00010	0.13	10.41	um10051	0.73	14.74
7c00011	-0.08	8.75	um10052	-1.22	11.88
7c00012	-0.20	9.99	um10053	-0.73	11.97
7c00013	-0.65	13.79	um10054	0.36	10.76
7c00014	0.34	12.83	um10055	-1.14	10.73
7c00015	-0.08	12.89	um10056	2.17	12.71

7c00016	-0.34	15.54	um10057	-0.40	12.53
7c00017	-0.21	10.32	um10059	-0.66	14.99
7c00018	0.84	14.92	um10060	0.51	12.00
7c00019	-0.37	12.39	um10061	0.48	11.92
7c00020	0.09	12.51	um10062	-0.38	13.87
7c00021	0.41	13.76	um10063	0.10	10.44
7c00022	0.24	12.78	um10064	0.11	13.78
7c00023	0.27	12.98	um10065	0.41	12.12
7c00024	-0.44	10.95	um10066	0.25	11.79
7c00025	0.54	11.03	um10067	1.03	11.33
7c00026	-0.31	10.25	um10068	-0.08	9.85
7c00027	0.70	14.21	um10069	0.46	10.98
7c00028	-0.12	11.30	um10070	0.96	10.90
7c00029	0.04	12.70	um10071	0.49	12.67
7c00030	0.17	11.92	um10072	1.24	11.39
7c00031	-0.33	11.79	um10073	-1.09	13.31
7c00032	-0.24	12.42	um10074	0.04	11.31
7c00033	-0.57	12.85	um10075	0.21	12.25
7c00034	-0.43	10.81	um10076	0.09	8.90
7c00035	-0.53	14.26	um10077	0.95	12.41
7c00036	-0.07	12.55	um10078	0.15	11.20
7c00037	0.13	11.71	um10079	0.09	13.03
7c00038	0.38	13.94	um10080	-0.87	13.17
7c00039	0.33	11.87	um10081	0.36	12.71
7c00040	-0.47	10.58	um10082	0.35	11.86
7c00041	-0.08	12.57	um10083	1.53	11.98
7c00042	-0.15	12.02	um10085	0.70	11.32
7c00043	0.01	9.70	um10086	-0.07	11.96
7c00044	-0.03	11.75	um10087	-0.30	11.24
7c00045	-0.10	12.75	um10088	0.71	10.50
7c00046	-0.78	13.54	um10089	0.04	11.14
7c00047	-0.16	11.52	um10091	-0.49	12.35
7c00048	0.12	13.75	um10092	-0.26	10.69
7c00049	-0.31	12.83	um10093	0.51	10.40
7c00050	-0.72	12.63	um10094	-0.59	12.06
7c00051	0.44	12.62	um10095	0.46	12.92
7c00052	0.22	11.32	um10097	0.74	12.73
7c00053	0.80	12.17	um10098	-0.40	11.79
7c00054	-0.19	12.69	um10099	-0.18	12.60
7c00055	-0.51	12.56	um10100	0.19	11.21
7c00056	-0.74	13.41	um10101	0.22	10.22
7c00057	0.47	11.61	um10102	0.15	11.93
7c00058	-0.04	12.43	um10103	-0.37	11.84
7c00059	-0.31	11.97	um10104	-0.98	11.56
7c00060	0.12	12.46	um10105	0.20	11.29
7c00061	-0.02	11.84	um10106	-0.34	11.78
7c00062	-0.20	12.54	um10107	-2.11	13.85
7c00063	0.03	9.80	um10108	0.23	11.60
7c00064	0.01	11.82	um10109	0.31	11.41
7c00065	-0.26	11.18	um10110	0.26	14.95
7c00066	0.03	11.00	um10112	0.81	11.16
7c00067	-0.07	10.40	um10113	-0.47	10.55
7c00068	0.06	8.54	um10114	0.13	14.72
7c00069	0.24	11.58	um10115	0.01	8.57

7c00070	0.10	11.49	um10116	0.28	10.89
7c00071	-0.17	11.67	um10117	-0.60	12.13
7c00072	0.44	13.16	um10118	0.41	10.96
7c00073	0.28	10.90	um10119	-0.44	10.13
7c00074	-0.27	15.68	um10120	-2.06	10.60
7c00075	0.72	13.30	um10121	0.07	11.15
7c00076	-0.04	12.86	um10122	-0.09	10.13
7c00077	0.40	12.58	um10123	-0.03	13.45
7c00078	0.22	12.16	um10124	0.69	12.14
7c00079	0.21	13.74	um10126	0.31	12.24
7c00080	-0.26	11.09	um10127	0.37	14.90
7c00081	-0.44	14.69	um10128	0.06	12.41
7c00082	0.05	12.76	um10129	0.18	13.32
7c00083	0.38	12.86	um10130	0.39	11.39
7c00084	0.00	9.06	um10131	-0.68	12.11
7c00085	-0.23	11.91	um10132	-0.28	13.48
7c00086	-0.08	10.89	um10133	0.60	11.05
7c00087	-0.20	11.03	um10134	-0.13	11.74
7c00088	0.76	12.87	um10135	0.12	9.33
7c00089	0.10	9.08	um10136	0.03	12.42
7c00090	0.43	11.90	um10138	0.41	11.07
7c00091	0.50	12.78	um10139	-0.10	11.12
7c00092	-0.04	15.36	um10140	0.72	12.66
7c00093	-1.68	11.60	um10141	0.63	11.72
7c00094	0.13	11.96	um10142	0.15	10.90
7c00095	0.02	10.37	um10143	1.21	13.72
7c00096	0.43	14.80	um10144	0.33	11.29
7c00097	0.11	12.69	um10145	-0.23	12.15
7c00098	0.48	13.52	um10146	0.18	14.64
7c00099	0.04	12.06	um10147	0.30	15.16
7c00100	-0.64	12.28	um10148	0.46	10.85
7c00101	0.20	13.56	um10149	-0.07	15.62
7c00102	-0.27	9.76	um10151	0.73	11.06
7c00103	0.14	11.41	um10152	0.05	11.55
7c00104	-0.12	8.98	um10153	-0.22	12.95
7c00105	0.10	11.93	um10154	-0.09	10.31
7c00106	-0.69	11.00	um10155	0.37	12.05
7c00107	-0.17	9.34	um10156	0.03	15.01
7c00108	-0.11	8.71	um10157	-0.58	12.49
7c00109	-0.02	8.56	um10158	0.04	12.82
7c00110	-0.13	8.83	um10159	0.47	11.51
7c00111	0.00	8.81	um10160	-0.22	12.57
7c00112	0.28	11.14	um10161	-0.02	12.27
7c00113	-0.08	11.95	um10162	0.24	12.82
7c00114	-0.24	10.68	um10163	0.28	14.70
7c00115	-0.09	15.05	um10164	0.58	10.57
7c00116	-0.02	12.09	um10165	-0.10	11.95
7c00117	-0.27	14.10	um10166	-0.44	10.92
7c00118	0.35	10.93	um10167	-0.68	15.83
7c00119	-0.07	11.30	um10168	0.10	13.20
7c00120	0.14	12.87	um10169	-0.26	13.48
7c00121	0.26	13.51	um10170	-0.80	11.56
7c00122	0.19	13.30	um10171	-0.02	8.76
7c00123	0.32	10.70	um10172	0.57	12.44

7c00124	0.01	12.73	um10173	0.27	13.85
7c00125	0.41	10.60	um10174	-0.39	12.23
7c00126	0.10	12.36	um10175	0.07	12.20
7c00127	-0.48	11.02	um10176	0.17	13.71
7c00128	-0.18	13.48	um10177	0.20	13.73
7c00129	0.05	12.64	um10178	-0.30	12.92
7c00130	0.24	13.10	um10179	0.16	13.70
7c00131	-0.37	12.57	um10180	-0.10	15.05
7c00132	-0.21	12.20	um10181	-0.71	11.61
7c00133	0.11	10.21	um10182	-0.15	15.26
7c00134	0.00	12.15	um10183	1.04	13.67
7c00135	0.18	9.18	um10184	-0.41	12.64
7c00136	-0.73	12.17	um10185	-0.34	12.03
7c00137	0.11	9.74	um10186	-2.87	10.71
7c00138	-0.15	10.41	um10187	0.25	13.60
7c00139	0.18	10.74	um10188	-1.72	13.49
7c00140	0.59	12.51	um10189	-1.76	12.13
7c00141	-0.13	12.54	um10190	0.56	11.33
7c00142	0.51	13.22	um10191	0.36	11.06
7c00143	0.30	11.20	um10192	-0.32	12.24
7c00144	0.00	8.62	um10193	-0.18	8.71
7c00145	0.28	12.14	um10194	-0.07	12.56
7c00146	-0.02	9.83	um10195	0.19	13.64
7c00147	-0.05	12.38	um10196	0.54	14.53
7c00148	0.19	14.37	um10197	0.22	15.04
7c00149	0.12	10.22	um10198	0.45	11.89
7c00150	-0.06	8.70	um10200	0.37	12.03
7c00151	-0.71	12.42	um10201	0.51	9.42
7c00152	0.35	13.31	um10202	-0.02	8.75
7c00153	0.11	12.90	um10203	-0.09	8.54
7c00154	-0.88	11.54	um10204	0.10	12.97
7c00155	-0.07	15.70	um10205	-0.50	13.31
7c00156	0.04	13.38	um10206	0.28	10.21
7c00157	0.06	12.54	um10207	-0.17	12.50
7c00158	0.64	11.96	um10208	2.70	12.11
7c00159	-0.07	10.87	um10209	0.29	12.17
7c00160	-0.22	11.40	um10210	-0.39	15.46
7c00161	-0.06	12.45	um10211	-0.96	11.52
7c00162	0.24	10.71	um10212	-0.01	10.47
7c00163	0.27	10.98	um10213	-0.26	15.46
7c00164	-0.04	9.97	um10214	0.37	13.82
7c00165	0.19	11.55	um10215	0.24	13.50
7c00166	0.20	11.59	um10217	0.09	11.98
7c00167	-0.25	13.23	um10218	-0.06	10.30
7c00168	0.08	12.62	um10219	-0.85	10.73
7c00169	0.16	9.39	um10220	0.02	10.26
7c00170	-0.51	12.04	um10221	-0.47	12.43
7c00171	-0.48	14.22	um10223	1.66	11.96
7c00172	0.32	12.32	um10225	-0.26	13.01
7c00173	0.05	9.00	um10226	0.08	13.81
7c00174	0.64	13.11	um10227	0.18	12.98
7c00175	0.47	13.11	um10228	-0.16	8.68
7c00176	0.02	12.06	um10229	0.44	12.76
7c00177	-0.12	9.86	um10230	-0.22	8.60

7c00178	-0.09	8.95	um10231	-0.25	13.42
7c00179	-0.24	12.83	um10232	0.13	15.04
7c00180	0.37	10.74	um10233	-0.09	8.68
7c00181	-1.14	14.01	um10234	1.19	11.80
7c00182	0.29	12.82	um10235	0.87	11.18
7c00183	-0.11	12.49	um10236	0.11	14.63
7c00184	-0.93	12.49	um10237	-0.28	10.57
7c00185	0.77	13.28	um10238	0.46	14.57
7c00186	-0.31	11.45	um10239	0.73	12.22
7c00187	-0.20	12.23	um10241	0.51	10.77
7c00188	0.48	12.69	um10242	0.66	13.14
7c00189	-0.14	12.40	um10243	-0.19	12.14
7c00190	0.36	12.19	um10244	0.33	11.15
7c00191	0.14	10.24	um10245	-0.27	11.54
7c00192	0.09	10.59	um10246	-0.35	13.02
7c00193	-0.07	12.46	um10248	0.81	11.31
7c00194	-0.05	9.05	um10251	0.20	11.69
7c00195	-0.18	15.36	um10252	0.74	13.67
7c00196	-0.10	12.40	um10253	0.86	12.75
7c00197	0.74	13.89	um10254	-0.89	12.70
7c00198	-0.03	12.14	um10255	0.13	11.67
7c00199	-0.03	10.83	um10256	0.29	11.53
7c00200	0.29	11.36	um10257	0.26	11.86
7c00201	0.11	11.96	um10258	0.34	10.94
7c00202	0.10	11.92	um10259	-0.28	11.28
7c00203	0.25	9.64	um10260	-0.48	11.97
7c00204	-0.23	11.01	um10261	0.18	11.95
7c00205	0.09	12.71	um10262	-0.33	14.20
7c00206	-0.22	13.04	um10263	1.18	12.19
7c00207	0.07	12.65	um10264	0.28	9.27
7c00208	-0.36	11.84	um10265	0.23	12.22
7c00209	0.36	10.81	um10266	-0.60	12.66
7c00210	-0.96	12.52	um10267	-0.22	12.25
7c00211	0.18	10.67	um10268	1.55	14.01
7c00212	0.47	11.31	um10269	-0.29	8.57
7c00213	-0.03	14.22	um10270	0.10	12.39
7c00214	0.32	12.15	um10271	-0.40	12.63
7c00215	0.59	11.54	um10272	-0.03	12.62
7c00216	0.44	9.71	um10273	0.81	13.84
7c00217	-0.16	11.67	um10274	-0.26	13.58
7c00218	0.18	9.68	um10275	-0.02	11.49
7c00219	0.18	13.71	um10276	0.10	12.68
7c00220	0.64	12.17	um10277	-0.44	12.64
7c00221	0.82	12.43	um10278	-0.23	8.92
7c00222	-0.04	9.87	um10279	-0.17	11.70
7c00223	0.35	12.60	um10280	-0.33	13.57
7c00224	0.04	11.95	um10282	0.24	9.07
7c00225	0.10	11.90	um10283	-0.41	13.35
7c00226	-0.19	10.94	um10284	-0.09	11.57
7c00227	0.16	12.15	um10285	-0.13	11.75
7c00228	0.07	10.77	um10286	-0.65	14.64
7c00229	-0.40	11.99	um10287	-0.49	13.65
7c00230	0.19	11.74	um10289	0.70	9.79
7c00231	-0.24	9.59	um10290	0.14	14.60

7c00232	-0.44	11.02	um10293	0.06	12.48
7c00233	0.43	13.43	um10294	0.03	11.61
7c00234	0.18	12.13	um10295	0.16	10.60
7c00235	-1.12	12.44	um10296	0.01	14.49
7c00236	-0.30	12.73	um10297	0.23	10.58
7c00237	-0.59	12.70	um10298	-0.04	11.10
7c00238	0.03	13.21	um10299	0.15	11.09
7c00239	-0.36	12.14	um10300	0.01	11.43
7c00240	-0.55	12.74	um10301	0.11	12.58
7c00241	0.03	13.39	um10302	0.33	10.89
7c00242	0.05	11.24	um10304	-0.14	11.14
7c00243	0.18	12.02	um10305	-0.05	12.51
7c00244	0.02	11.51	um10306	0.12	11.90
7c00245	-0.39	12.69	um10307	0.04	12.32
7c00246	0.05	14.36	um10308	-0.56	12.17
7c00247	0.97	12.71	um10309	-0.19	10.57
7c00248	0.06	9.62	um10310	-0.81	11.86
7c00249	0.00	13.23	um10311	0.46	13.25
7c00250	0.02	10.56	um10312	0.07	11.88
7c00251	0.32	11.49	um10313	0.11	12.79
7c00252	1.00	13.96	um10314	-0.12	12.13
7c00253	0.18	12.75	um10315	-0.07	12.25
7c00254	-0.21	11.77	um10317	0.04	13.46
7c00255	-0.27	13.36	um10318	-0.90	11.80
7c00256	-0.29	11.37	um10319	-0.47	10.70
7c00257	0.12	12.46	um10320	-0.38	11.30
7c00258	0.20	12.68	um10321	-0.11	11.30
7c00259	-0.31	9.92	um10322	-0.30	12.10
7c00260	0.28	12.89	um10323	-0.01	11.70
7c00261	0.21	12.40	um10324	-0.08	8.62
7c00262	-0.62	13.62	um10325	-0.03	9.70
7c00263	-1.52	12.05	um10326	-0.01	11.68
7c00264	0.02	12.14	um10327	-0.58	11.02
7c00265	-0.45	11.76	um10328	0.06	8.70
7c00266	0.18	15.41	um10329	-0.41	12.36
7c00267	-0.27	11.81	um10330	-0.36	11.50
7c00268	0.29	12.39	um10331	-0.42	15.66
7c00269	-0.12	12.08	um10332	-0.52	13.04
7c00270	-0.10	8.93	um10333	0.38	11.74
7c00271	-0.28	12.82	um10334	0.07	14.76
7c00272	0.18	13.48	um10335	0.23	10.28
7c00273	-0.12	11.31	um10336	0.40	13.03
7c00274	-0.36	12.66	um10337	0.29	13.12
7c00275	0.19	12.42	um10338	0.06	10.54
7c00276	-0.35	10.72	um10339	-2.07	13.25
7c00277	-0.40	10.17	um10340	0.52	12.84
7c00278	0.35	12.18	um10341	0.43	10.96
7c00279	-0.85	11.57	um10342	0.89	12.24
7c00280	-0.15	11.95	um10343	-0.12	12.36
7c00281	-0.07	15.89	um10344	-0.33	11.53
7c00282	-0.52	12.11	um10347	0.06	11.28
7c00283	-0.13	11.05	um10348	-0.08	11.45
7c00284	0.16	13.50	um10349	0.17	11.36
7c00285	-0.53	14.35	um10350	-0.47	12.68

7c00286	-0.42	13.38	um10351	0.35	12.28
7c00287	0.06	12.97	um10352	0.51	10.33
7c00288	0.02	11.93	um10353	0.15	11.20
7c00289	0.07	11.06	um10354	0.05	11.36
7c00290	0.23	11.26	um10355	0.09	11.68
7c00291	0.53	10.22	um10356	-0.44	13.75
7c00292	1.17	11.89	um10357	-0.24	13.02
7c00293	0.63	11.73	um10358	0.08	9.76
7c00294	0.26	12.92	um10359	0.58	11.56
7c00295	-0.12	10.14	um10360	0.00	14.81
7c00296	0.82	10.23	um10361	0.32	14.60
7c00297	0.17	14.34	um10362	-0.24	12.43
7c00298	-0.20	12.68	um10363	-0.03	12.87
7c00299	-0.17	13.21	um10364	-1.51	9.74
7c00300	-0.08	11.23	um10365	-0.74	12.44
7c00301	-0.09	12.94	um10366	0.40	11.59
7c00302	0.39	10.82	um10367	0.18	13.31
7c00303	-0.26	9.81	um10368	-0.11	11.10
7c00304	0.17	9.87	um10369	0.04	11.97
7c00305	0.08	12.93	um10370	-0.91	13.24
7c00306	0.18	11.30	um10371	0.14	12.38
7c00307	-0.52	10.74	um10372	0.27	11.37
7c00308	0.37	10.59	um10373	-0.11	14.34
7c00309	-0.01	11.45	um10374	-0.16	13.60
7c00310	-0.08	10.56	um10375	0.30	13.18
7c00311	-0.74	12.03	um10376	-0.18	11.68
7c00312	0.00	10.16	um10377	0.21	10.98
7c00313	0.34	13.04	um10378	-0.41	10.98
7c00314	-0.34	15.04	um10379	0.47	12.33
7c00315	-0.40	12.03	um10380	-0.36	11.46
7c00316	-0.65	12.64	um10381	0.24	12.51
7c00317	0.17	13.64	um10382	-0.17	13.50
7c00318	-0.04	12.20	um10383	0.07	12.34
7c00319	0.35	13.84	um10384	-0.04	12.50
7c00320	-0.34	11.43	um10385	-0.29	13.01
7c00321	0.00	9.19	um10386	-0.16	12.22
7c00322	0.31	13.78	um10387	-0.86	14.59
7c00323	-0.02	9.26	um10388	1.44	12.91
7c00324	0.04	13.28	um10390	-0.98	11.53
7c00325	-0.51	11.97	um10391	-0.31	11.33
7c00326	0.27	12.88	um10392	0.49	12.30
7c00327	-0.17	8.99	um10393	0.22	11.19
7c00328	-0.23	12.05	um10394	-0.73	11.78
7c00329	0.05	11.61	um10395	1.75	11.90
7c00330	-0.46	10.82	um10396	0.71	11.26
7c00331	0.18	12.58	um10397	-0.30	15.60
7c00332	-1.48	14.89	um10398	0.27	11.87
7c00333	0.23	9.29	um10399	0.40	12.13
7c00334	0.16	12.28	um10400	-0.08	13.10
7c00335	0.42	12.17	um10401	-0.07	10.80
7c00336	0.15	12.03	um10402	0.42	10.95
7c00337	0.04	10.57	um10403	-0.25	9.03
7c00338	-0.34	14.11	um10405	0.43	10.99
7c00339	0.14	12.08	um10406	0.53	11.15

7c00340	0.01	8.95	um10407	0.89	11.16
7c00341	0.02	8.73	um10408	0.09	11.08
7c00342	-0.10	11.86	um10410	0.13	10.95
7c00343	-0.85	14.11	um10412	0.47	12.18
7c00344	0.04	12.54	um10413	-1.07	9.20
7c00345	0.03	13.06	um10414	-0.82	11.73
7c00346	-0.11	13.10	um10415	0.27	11.52
7c00347	0.24	11.53	um10416	0.09	10.91
7c00348	0.64	13.36	um10417	-0.56	11.82
7c00349	0.36	11.81	um10418	-0.33	8.59
7c00350	0.45	11.83	um10419	-2.53	11.92
7c00351	0.14	10.08	um10420	0.93	10.75
7c00352	0.00	15.26	um10421	-0.17	11.81
7c00353	-0.14	9.53	um10422	0.01	11.29
7c00354	0.86	15.03	um10423	-0.06	12.34
7c00355	0.10	10.38	um10424	0.04	12.62
7c00356	0.21	12.66	um10425	0.08	11.70
7c00357	0.18	10.85	um10426	-0.40	13.01
7c00358	1.76	13.14	um10428	0.15	12.39
7c00359	2.30	13.92	um10429	0.00	12.78
7c00360	2.42	12.74	um10430	0.32	11.36
7c00361	1.16	10.30	um10431	0.13	12.03
7c00362	1.02	11.41	um10432	-0.05	9.68
7d00001	-0.25	12.63	um10433	0.33	12.76
7d00002	-0.02	13.39	um10434	-0.13	15.31
7d00003	-0.28	11.08	um10435	-0.35	12.49
7d00004	0.58	11.81	um10436	-0.50	11.14
7d00005	0.85	12.12	um10437	-0.02	11.87
7d00006	0.80	12.46	um10438	0.01	11.13
7d00007	-0.09	12.17	um10439	0.86	11.85
7d00008	-0.61	8.87	um10440	0.54	12.49
7d00009	0.84	11.37	um10441	0.03	14.77
7d00010	0.52	13.18	um10442	0.00	11.50
7d00011	0.00	8.82	um10443	1.32	11.26
7d00012	-0.40	13.09	um10444	-2.43	13.19
7d00013	-0.13	11.84	um10445	-0.57	12.44
7d00014	-0.05	8.63	um10446	0.73	11.18
7d00015	-0.94	12.07	um10447	-0.31	12.86
7d00016	-0.06	10.99	um10448	-0.12	12.60
7d00017	-0.31	11.26	um10449	0.52	12.06
7d00018	-0.08	10.19	um10450	0.70	10.39
7d00019	0.07	12.84	um10451	-0.33	11.82
7d00020	-0.01	12.65	um10452	0.97	11.18
7d00021	-0.24	13.05	um10456	-0.43	11.35
7d00022	-0.18	12.08	um10457	-0.22	12.46
7d00023	0.32	12.19	um10459	-0.09	12.79
7d00024	0.40	12.17	um10461	-0.30	11.91
7d00025	-0.19	11.06	um10462	-0.39	12.53
7d00026	-0.29	12.26	um10463	-0.40	11.11
7d00027	-0.13	12.21	um10464	-1.76	11.30
7d00028	-0.41	11.21	um10465	-0.18	11.88
7d00029	-0.15	12.89	um10466	-0.65	12.90
7d00030	0.27	13.45	um10467	0.51	11.39
7d00031	0.17	12.16	um10468	0.09	12.15

7d00032	-0.27	11.79	um1046[illegible]	0.56	14.73
7d00033	-0.10	12.23	um1047[illegible]	1.08	13.34
7d00034	0.11	13.67	um1047[illegible]	0.65	10.06
7d00035	0.19	9.12	um1047[illegible]	0.06	13.39
7d00036	0.06	9.58	um1047[illegible]	0.87	11.86
7d00037	0.04	12.42	um1047[illegible]	0.56	11.72
7d00038	0.01	10.47	um1047[illegible]	-0.18	12.80
7d00039	0.16	12.29	um1047[illegible]	-0.61	14.87
7d00040	-0.22	11.86	um1048[illegible]	0.29	11.63
7d00041	-1.00	12.17	um10481	0.31	13.19
7d00042	-0.35	12.78	um1048[illegible]	0.18	13.49
7d00043	-0.04	12.07	um1048[illegible]	1.03	12.97
7d00044	-0.40	10.55	um1048[illegible]	-0.58	11.63
7d00045	0.14	10.10	um1048[illegible]	0.24	11.16
7d00046	-0.19	10.63	um1048[illegible]	1.69	11.47
7d00047	-0.01	12.25	um1048[illegible]	-0.20	13.50
7d00048	-0.33	11.09	um1048[illegible]	-0.14	13.66
7d00049	-0.77	12.22	um1049[illegible]	0.07	10.27
7d00050	-0.05	8.65	um10491	0.19	9.09
7d00051	-0.32	12.33	um1049[illegible]	0.15	11.53
7d00052	-0.35	13.28	um1049[illegible]	0.56	12.82
7d00053	-0.69	12.71	um1049[illegible]	-1.05	12.39
7d00054	-0.28	13.04	um1049[illegible]	0.21	10.80
7d00055	-0.13	9.59	um1049[illegible]	-0.28	11.31
7d00056	0.08	11.63	um1049[illegible]	0.22	13.19
7d00057	0.20	11.75	um1049[illegible]	0.34	12.92
7d00058	-0.21	12.80	um1049[illegible]	-0.57	10.91
7d00059	0.11	13.24	um1050[illegible]	-0.37	8.68
7d00060	-0.02	11.35	um10501	0.57	10.96
7d00061	-0.19	12.92	um1050[illegible]	0.00	12.25
7d00062	-0.23	12.75	um1050[illegible]	-0.06	14.51
7d00063	0.21	9.50	um1050[illegible]	0.77	11.68
7d00064	-0.05	13.01	um1050[illegible]	0.74	14.36
7d00065	-0.13	10.83	um1050[illegible]	0.16	10.59
7d00066	-0.39	12.17	um1050[illegible]	0.87	12.43
7d00067	-0.14	11.51	um10511	0.15	11.61
7d00068	0.01	12.28	um1051[illegible]	-0.09	13.55
7d00069	0.96	13.24	um1051[illegible]	0.14	12.34
7d00070	0.25	12.48	um1051[illegible]	0.64	11.54
7d00071	0.17	11.42	um1051[illegible]	0.71	12.42
7d00072	0.08	10.65	um1051[illegible]	2.00	11.64
7d00073	-0.32	10.15	um1051[illegible]	1.39	14.34
7d00074	0.17	12.32	um1052[illegible]	0.75	12.08
7d00075	0.30	11.28	um10521	1.02	10.74
7d00076	0.32	9.03	um1052[illegible]	0.21	12.01
7d00077	0.25	11.70	um1052[illegible]	-0.32	12.49
7d00078	-1.85	10.31	um1052[illegible]	-0.53	12.46
7d00079	0.45	13.42	um1052[illegible]	0.26	9.23
7d00080	-0.12	10.39	um1052[illegible]	-0.10	14.19
7d00081	-0.73	11.77	um1052[illegible]	0.51	11.40
7d00082	0.52	12.81	um1052[illegible]	-0.71	9.67
7d00083	-0.23	10.76	um1052[illegible]	0.77	15.20
7d00084	0.50	12.24	um1053[illegible]	0.53	9.81
7d00085	0.09	8.49	um10531	-0.07	11.41

7d00086	-0.07	8.88	um10532	-0.24	12.24
7d00087	-0.06	8.83	um10533	-0.04	12.98
7d00088	-0.39	11.11	um10534	0.71	12.18
7d00089	-0.22	12.25	um10535	0.23	11.47
7d00090	-0.45	11.93	um10536	0.25	14.61
7d00091	0.03	12.31	um10538	0.13	12.40
7d00092	-0.20	11.05	um10539	0.50	11.08
7d00093	0.55	13.56	um10540	1.48	13.53
7d00094	-1.69	12.65	um10541	0.49	11.19
7d00095	-0.58	12.96	um10543	-0.02	8.41
7d00096	-0.20	12.73	um10544	-0.63	13.09
7d00097	-1.11	12.73	um10545	-1.93	11.95
7d00098	-0.88	13.17	um10546	-0.31	11.31
7d00099	-0.47	14.15	um10548	-0.22	15.45
7d00100	-0.68	12.15	um10549	0.20	11.05
7d00101	-0.22	12.35	um10551	0.04	11.97
7d00102	-0.34	12.08	um10552	-0.26	12.13
7d00103	0.03	12.01	um10553	-0.31	8.87
7d00104	0.04	9.78	um10554	-0.35	8.57
7d00105	0.30	12.88	um10555	-0.18	8.79
7d00106	-0.27	9.70	um10556	-0.14	8.50
7d00107	-0.12	13.10	um10557	-0.67	8.92
7d00108	0.12	9.33	um10558	-0.12	13.25
7d00109	0.10	11.22	um10559	-1.02	12.37
7d00110	0.02	12.17	um10560	0.91	11.11
7d00111	-0.04	12.03	um10561	-0.42	12.54
7d00112	-0.08	12.32	um10565	-0.37	9.67
7d00113	-0.07	13.96	um10566	0.05	11.84
7d00114	0.31	12.03	um10568	0.02	12.51
7d00115	0.18	10.09	um10569	-0.36	12.35
7d00116	0.14	13.39	um10570	0.48	11.09
7d00117	0.49	10.12	um10571	0.15	11.60
7d00118	-0.38	14.65	um10573	0.37	14.83
7d00119	-0.62	12.42	um10574	-0.39	11.94
7d00120	0.03	12.02	um10575	0.82	11.54
7d00121	-0.27	11.38	um10576	0.63	10.73
7d00122	-0.37	11.36	um10577	0.26	12.25
7d00123	0.28	11.31	um10578	0.23	10.55
7d00124	0.07	12.78	um10579	0.23	11.53
7d00125	-0.02	12.63	um10581	3.45	11.97
7d00126	0.00	15.84	um10582	-0.03	13.05
7d00127	-0.41	10.78	um10583	-0.51	9.73
7d00128	0.09	10.48	um10585	1.01	14.17
7d00129	0.58	13.12	um10586	-0.17	13.74
7d00130	-0.09	11.07	um10587	-1.67	13.43
7d00131	0.05	12.88	um10588	0.48	12.16
7d00132	0.55	12.87	um10589	-1.34	11.72
7d00133	0.41	10.55	um10590	2.63	12.02
7d00134	0.25	13.42	um10591	-0.07	11.42
7d00135	-0.40	14.42	um10593	0.40	10.73
7d00136	-0.36	12.46	um10594	0.00	11.16
7d00137	0.25	11.94	um10595	-0.12	11.21
7d00138	-0.21	11.81	um10596	-0.05	13.48
7d00139	0.14	9.12	um10597	-0.13	11.22

7d00140	0.01	9.03	um10598	-0.46	14.81
7d00141	0.03	12.14	um10599	0.73	13.60
7d00142	-0.48	12.68	um10602	-0.12	10.21
7d00143	1.07	13.15	um10603	0.05	10.88
7d00144	0.39	13.09	um10604	-0.29	11.69
7d00145	-0.34	11.56	um10605	2.08	13.27
7d00146	-0.01	13.41	um10606	-0.77	11.26
7d00147	0.09	11.79	um10607	-0.11	15.17
7d00148	0.22	8.82	um10608	3.00	11.26
7d00149	-0.21	12.57	um10609	0.13	9.65
7d00150	-0.02	8.58	um10611	-0.77	13.32
7d00151	-0.03	8.42	um10613	0.10	12.71
7d00152	0.18	14.43	um10614	-0.26	10.78
7d00153	-0.07	15.24	um10615	0.05	13.99
7d00154	0.46	12.16	um10616	-0.72	11.40
7d00155	0.44	11.35	um10617	-0.16	12.82
7d00156	0.48	13.43	um10618	1.17	11.76
7d00157	0.52	12.07	um10620	0.66	11.22
7d00158	-0.49	13.18	um10621	0.10	14.62
7d00159	0.10	11.49	um10622	0.79	10.97
7d00160	-0.02	13.53	um10623	-0.13	11.54
7d00161	0.11	12.00	um10624	-0.27	12.11
7d00162	0.20	10.01	um10625	0.09	15.45
7d00163	0.34	11.42	um10626	-0.02	13.43
7d00164	0.28	13.24	um10627	0.13	12.66
7d00165	0.92	14.79	um10628	0.05	14.67
7d00166	-0.02	11.48	um10629	-0.02	13.44
7d00167	-0.19	9.11	um10630	0.26	10.18
7d00168	-0.75	14.82	um10631	1.24	12.42
7d00169	-0.61	11.97	um10632	-6.75	12.38
7d00170	0.04	11.48	um10633	-0.58	9.00
7d00171	-0.22	11.75	um10634	-0.39	10.62
7d00172	-0.02	12.74	um10635	-0.57	8.77
7d00173	0.35	13.20	um10636	0.68	12.08
7d00174	0.20	12.91	um10637	-0.85	9.76
7d00175	0.71	13.73	um10639	0.13	11.44
7d00176	0.42	11.92	um10640	0.29	11.26
7d00177	0.46	11.37	um10641	-0.73	11.80
7d00178	-2.68	12.30	um10642	0.62	11.23
7d00179	-0.19	10.01	um10643	-0.13	12.20
7d00180	0.56	13.04	um10644	-0.28	14.61
7d00181	-0.07	12.43	um10645	0.10	10.83
7d00182	0.21	11.99	um10646	0.41	12.88
7d00183	1.40	13.96	um10647	0.07	13.12
7d00184	0.06	8.63	um10648	-0.80	12.10
7d00185	-0.20	10.39	um10649	-0.57	12.58
7d00186	-0.15	15.88	um10650	-0.12	12.08
7d00187	0.21	12.48	um10651	-0.15	11.96
7d00188	-0.27	14.32	um10652	0.43	11.98
7d00189	0.07	13.33	um10653	0.20	11.67
7d00190	-0.28	12.77	um10654	0.49	12.88
7d00191	-0.10	8.79	um10655	0.22	12.40
7d00192	0.29	11.93	um10656	-0.51	10.32
7d00193	-0.10	8.68	um10657	0.60	12.44

7d00194	0.03	11.79	um10658	0.09	12.16
7d00195	0.43	12.78	um10659	-0.62	12.96
7d00196	-0.42	11.97	um10661	-0.07	10.56
7d00197	-0.02	10.06	um10662	0.21	11.16
7d00198	-0.48	12.64	um10663	-0.36	11.86
7d00199	-0.33	14.54	um10664	1.07	12.21
7d00200	-0.69	11.41	um10665	1.18	12.26
7d00201	-0.62	12.98	um10666	0.52	10.82
7d00202	-0.13	11.90	um10667	0.00	12.74
7d00203	0.67	11.06	um10668	0.05	11.21
7d00204	0.03	12.99	um10669	0.00	11.10
7d00205	0.52	11.65	um10671	0.43	12.97
7d00206	0.89	13.09	um10672	-0.50	9.71
7d00207	-0.01	8.69	um10673	-0.65	12.42
7d00208	0.03	11.23	um10674	0.16	11.84
7d00209	-0.05	12.90	um10675	-0.42	13.63
7d00210	0.06	11.51	um10676	1.01	15.05
7d00211	-0.07	11.97	um10678	-0.55	9.85
7d00212	-0.09	12.88	um10679	2.41	12.48
7d00213	0.05	12.90	um10680	-0.32	11.02
7d00214	0.07	8.66	um10681	0.05	14.01
7d00215	0.53	13.23	um10682	1.67	11.85
7d00216	0.70	10.50	um10683	0.43	10.77
7d00217	0.13	13.03	um10684	0.46	10.91
7d00218	-0.31	12.86	um10685	0.68	12.91
7d00219	-0.55	12.84	um10686	0.42	11.55
7d00220	-0.12	9.87	um10687	0.09	12.37
7d00221	-0.82	11.17	um10688	-0.88	11.10
7d00222	0.28	12.65	um10689	0.24	11.43
7d00223	-0.02	13.22	um10690	0.87	9.34
7d00224	0.07	10.33	um10691	1.67	11.70
7d00225	0.21	11.19	um10692	0.98	10.03
7d00226	0.12	13.08	um10693	0.47	11.52
7d00227	0.42	14.25	um10694	-0.10	10.82
7d00228	-0.15	13.36	um10695	0.84	13.38
7d00229	0.42	12.90	um10696	-0.35	12.23
7d00230	-0.20	12.35	um10697	0.01	9.06
7d00231	-0.03	13.40	um10698	0.68	11.50
7d00232	-0.28	9.94	um10699	-0.93	12.66
7d00233	-0.24	14.16	um10700	-0.04	14.64
7d00234	0.56	13.39	um10701	0.31	14.45
7d00235	0.36	10.59	um10702	0.14	14.97
7d00236	-0.02	13.32	um10703	-0.74	11.73
7d00237	-0.49	14.69	um10704	0.06	12.22
7d00238	0.68	10.87	um10705	-1.02	11.84
7d00239	0.05	9.58	um10707	0.24	11.81
7d00240	-0.55	10.13	um10708	-0.14	13.58
7d00241	0.16	13.05	um10709	0.06	10.74
7d00242	0.05	12.00	um10711	0.30	14.90
7d00243	-0.16	12.65	um10712	-0.48	12.46
7d00244	-0.43	15.50	um10713	0.31	11.20
7d00245	0.18	11.91	um10714	-0.03	14.26
7d00246	-0.52	12.84	um10715	-0.42	11.36
7d00247	-0.11	8.76	um10718	-0.91	12.40

7d00248	0.92	13.75	um10719	0.39	12.78
7d00249	-0.06	12.27	um10720	0.14	12.03
7d00250	0.44	14.51	um10721	1.10	11.66
7d00251	-0.47	11.41	um10722	-0.03	11.89
7d00252	-0.12	11.11	um10723	0.05	13.85
7d00253	0.05	8.68	um10724	-0.17	12.07
7d00254	0.56	14.33	um10725	0.45	11.15
7d00255	-0.46	10.01	um10726	-0.14	13.26
7d00256	-0.19	10.59	um10727	-0.23	11.46
7d00257	-1.51	14.85	um10728	0.02	10.46
7d00258	0.56	13.54	um10729	-0.08	11.54
7d00259	-0.14	12.10	um10730	-0.64	11.11
7d00260	0.28	11.61	um10731	0.06	12.89
7d00261	-0.39	11.95	um10732	-0.28	12.02
7d00262	-0.15	11.62	um10733	0.79	13.22
7d00263	-0.19	11.46	um10735	0.08	11.51
7d00264	-0.46	13.46	um10737	0.06	10.89
7d00265	-0.32	11.19	um10738	0.71	11.78
7d00266	-0.41	13.17	um10739	-0.01	8.71
7d00267	-0.29	12.15	um10740	-0.31	12.61
7d00268	0.05	12.82	um10741	-0.07	12.18
7d00269	-0.08	12.16	um10742	0.34	14.62
7d00270	-0.26	12.25	um10746	0.87	12.55
7d00271	0.00	9.86	um10747	0.10	11.32
7d00272	-0.21	12.08	um10748	0.08	11.76
7d00273	-0.16	11.26	um10749	-0.44	12.08
7d00274	-0.26	11.61	um10750	0.12	11.62
7d00275	-0.61	12.54	um10751	0.42	13.17
7d00276	-1.24	12.53	um10752	0.83	13.12
7d00277	-1.41	12.43	um10753	-1.07	15.29
7d00278	-0.40	11.45	um10754	-0.11	14.73
7d00279	-0.08	11.56	um10755	-0.54	13.23
7d00280	0.36	12.80	um10756	-0.12	9.16
7d00281	0.18	12.56	um10757	0.73	12.40
7d00282	0.63	11.70	um10758	0.13	10.90
7d00283	-0.11	9.80	um10759	-0.51	9.59
7d00284	0.07	11.86	um10760	0.26	13.58
7d00285	-0.10	14.25	um10761	0.07	12.01
7d00286	-0.39	10.69	um10763	0.09	12.49
7d00287	-0.05	11.70	um10764	0.28	11.87
7d00288	1.19	11.63	um10765	0.24	12.56
7d00289	0.34	12.82	um10766	-0.28	12.95
7d00290	0.09	13.21	um10767	-0.08	10.89
7d00291	0.09	11.77	um10768	-0.15	10.61
7d00292	-0.29	15.78	um10769	-0.05	13.06
7d00293	0.09	12.32	um10770	-0.33	12.36
7d00294	-0.02	14.87	um10771	-0.06	12.67
7d00295	-3.82	12.59	um10772	-0.35	13.30
7d00296	-0.24	11.23	um10773	-0.07	13.42
7d00297	-0.13	9.67	um10774	-0.11	10.02
7d00298	0.03	11.68	um10775	-0.14	11.07
7d00299	-0.27	12.82	um10776	-0.10	12.31
7d00300	0.03	12.66	um10777	-0.36	15.47
7d00301	-0.24	14.87	um10778	-0.47	11.84

7d00302	0.30	12.57	um10779	-0.21	12.65
7d00303	0.34	14.09	um10780	-0.32	14.05
7d00304	0.42	12.75	um10781	-0.79	12.66
7d00305	-0.51	12.74	um10782	-1.10	11.34
7d00306	0.20	11.51	um10783	0.79	13.88
7d00307	0.21	10.99	um10784	-0.31	11.75
7d00308	0.48	10.52	um10785	0.21	11.85
7d00309	0.16	13.77	um10787	-0.02	14.02
7d00310	-0.18	10.21	um10788	-0.67	12.74
7d00311	-0.97	13.62	um10789	2.64	14.01
7d00312	-0.15	9.84	um10790	0.95	13.07
7d00313	0.08	11.37	um10791	2.33	12.78
7d00314	0.23	11.46	um10792	0.19	12.53
7d00315	0.03	8.55	um10793	0.17	11.36
7d00316	-0.58	12.00	um10794	-0.25	12.51
7d00317	0.02	15.99	um10795	-0.13	11.58
7d00318	-0.08	8.56	um10796	0.40	11.62
7d00319	0.58	12.71	um10797	0.57	11.82
7d00320	-0.56	11.33	um10799	0.18	11.73
7d00321	-0.37	10.76	um10800	-0.14	12.19
7d00322	-0.20	13.15	um10801	0.98	12.71
7d00323	-0.03	13.05	um10803	0.07	11.74
7d00324	0.01	10.40	um10804	0.22	14.27
7d00325	0.32	12.91	um10805	-0.05	12.79
7d00326	-0.47	11.59	um10806	0.44	11.55
7d00327	-0.26	13.85	um10807	-0.05	12.28
7d00328	-0.30	11.69	um10808	0.23	13.29
7d00329	0.01	13.90	um10809	-0.11	11.15
7d00330	0.22	12.28	um10810	-0.21	13.36
7d00331	-0.01	13.09	um10811	-0.13	12.39
7d00332	-0.48	11.52	um10812	-0.03	12.21
7d00333	0.18	9.81	um10813	-0.22	11.36
7d00334	0.37	11.90	um10814	-0.51	11.47
7d00335	0.11	13.15	um10815	-1.46	10.70
7d00336	0.30	12.57	um10816	-2.05	12.60
7d00337	0.25	11.81	um10817	0.42	11.08
7d00338	-0.33	12.18	um10820	-0.13	13.13
7d00339	-0.03	9.59	um10821	-0.09	11.22
7d00340	0.01	11.16	um10822	0.07	12.21
7d00341	-0.32	13.41	um10823	-0.59	12.95
7d00342	0.03	12.60	um10824	-0.02	11.41
7d00343	0.17	10.76	um10825	0.10	12.50
7d00344	-0.12	13.48	um10826	0.35	11.86
7d00345	0.02	12.08	um10827	-0.19	10.78
7d00346	-0.89	10.41	um10830	0.26	11.22
7d00347	0.02	13.66	um10831	-0.06	12.00
7d00348	-0.24	11.34	um10832	-0.27	14.78
7d00349	0.31	9.30	um10833	0.08	10.98
7d00350	-0.15	11.70	um10835	0.91	12.91
7d00351	-0.04	12.54	um10836	-0.54	12.04
7d00352	-0.39	9.42	um10837	0.04	11.01
7d00353	0.05	10.71	um10838	0.41	12.06
7d00354	0.24	10.29	um10839	-0.13	13.80
7d00355	-0.83	12.09	um10840	-0.50	12.68

7d00356	0.21	12.09
7d00357	0.06	12.08
7d00358	0.25	12.08
7d00359	0.89	13.13
89d0000	0.17	13.69
8c00001	-0.41	9.43
8c00002	-0.91	10.10
8c00003	-1.17	11.39
8c00004	-0.41	9.66
8c00005	0.00	10.03
8c00006	0.21	11.48
8c00007	-1.02	10.67
8c00008	-0.15	11.26
8c00009	-0.44	13.56
8c00010	0.45	13.07
8c00011	0.02	9.66
8c00012	-0.13	13.50
8c00013	-0.27	11.22
8c00014	-0.39	12.63
8c00015	-0.05	11.59
8c00016	0.54	12.74
8c00017	-0.29	8.97
8c00018	0.06	11.80
8c00019	-0.16	11.19
8c00020	0.01	12.53
8c00021	-0.32	13.94
8c00022	-0.76	14.80
8c00023	-0.43	10.55
8c00024	0.43	13.22
8c00025	0.03	8.80
8c00026	0.16	8.73
8c00027	0.26	12.35
8c00028	-0.06	12.19
8c00029	0.06	11.51
8c00030	0.27	14.46
8c00031	0.28	11.80
8c00032	-0.48	11.87
8c00033	0.34	11.73
8c00034	-0.56	12.53
8c00035	-0.02	12.06
8c00036	0.23	11.87
8c00037	0.26	12.62
8c00038	0.43	9.89
8c00039	0.13	11.40
8c00040	1.35	13.39
8c00041	0.07	12.00
8c00042	0.42	11.63
8c00043	-0.29	13.95
8c00044	0.49	12.46
8c00045	0.57	13.69
8c00046	-0.13	9.90
8c00047	0.11	12.57
8c00048	-0.03	12.23
8c00049	0.07	12.86

um10841	0.19	15.04
um10842	-0.07	15.41
um10843	0.20	12.31
um10844	0.40	13.13
um10845	-0.31	10.85
um10846	0.33	10.79
um10847	0.45	13.88
um10848	0.45	10.87
um10849	0.66	11.62
um10850	-0.32	11.61
um10851	0.74	13.07
um10852	-0.39	11.87
um10853	-0.19	8.77
um10854	-0.27	11.44
um10855	0.27	12.05
um10856	0.13	11.89
um10858	-0.44	11.77
um10859	0.23	14.85
um10861	1.45	12.52
um10862	0.39	11.75
um10863	-0.12	11.30
um10864	2.04	11.83
um10865	0.39	9.81
um10867	-0.06	11.01
um10868	0.27	11.75
um10869	0.99	10.14
um10870	-0.53	13.13
um10871	0.73	13.25
um10872	0.06	11.93
um10873	0.35	11.44
um10874	0.12	11.17
um10875	0.53	14.23
um10876	-0.20	12.61
um10878	-0.25	11.93
um10879	-0.39	13.31
um10880	0.48	10.42
um10881	0.34	11.37
um10882	0.34	12.02
um10884	0.20	12.12
um10885	-0.39	10.42
um10886	-0.41	11.52
um10888	-1.36	12.20
um10889	0.43	11.89
um10892	-0.44	11.44
um10893	0.64	11.44
um10895	-0.21	11.94
um10896	-0.13	12.96
um10897	2.15	12.41
um10898	-0.99	14.81
um10901	0.04	14.89
um10902	0.14	11.41
um10903	0.07	12.78
um10904	-1.33	12.95
um10905	1.00	12.80

8c00050	0.27	13.69	um10906	-0.18	12.72
8c00051	0.58	12.46	um10907	-0.39	13.09
8c00052	-0.31	12.22	um10908	0.23	11.20
8c00053	0.02	11.29	um10909	0.13	11.21
8c00054	0.42	13.17	um10910	-0.75	11.66
8c00055	-0.18	11.61	um10911	0.04	12.44
8c00056	0.01	12.61	um10912	1.13	11.72
8c00057	0.43	13.92	um10913	-0.38	11.97
8c00058	0.33	11.78	um10914	0.20	11.73
8c00059	0.61	10.98	um10915	0.25	12.03
8c00060	-0.19	10.82	um10916	-0.12	11.08
8c00061	0.09	12.32	um10917	0.00	13.22
8c00062	0.02	9.87	um10918	-0.04	12.66
8c00063	0.38	9.57	um10922	0.38	11.54
8c00064	-0.38	12.94	um10923	-0.03	10.28
8c00065	-0.21	12.62	um10924	0.77	10.56
8c00066	-0.20	10.18	um10925	0.39	12.10
8c00067	-0.53	14.76	um10926	-0.40	14.17
8c00068	0.34	14.69	um10928	-0.05	11.34
8c00069	0.20	13.71	um10929	0.22	13.07
8c00070	0.50	12.89	um10934	0.05	11.13
8c00071	0.18	14.96	um10935	0.06	11.08
8c00072	-0.02	12.07	um10936	0.39	11.84
8c00073	-1.05	13.35	um10937	0.28	12.86
8c00074	0.20	14.22	um10938	0.04	15.53
8c00075	-0.10	12.62	um10939	0.39	13.32
8c00076	0.56	13.87	um10940	-0.76	12.96
8c00077	0.27	13.37	um10941	0.50	10.76
8c00078	-0.27	11.75	um10944	0.28	10.90
8c00079	0.98	13.20	um10945	1.11	10.73
8c00080	-0.28	13.11	um10946	1.18	10.31
8c00081	0.09	12.32	um10949	-0.04	8.97
8c00082	-0.83	11.54	um10950	0.29	9.60
8c00083	0.13	12.92	um10951	-0.84	11.13
8c00084	0.02	8.87	um10953	-0.64	15.72
8c00085	-0.09	8.77	um10955	0.16	12.18
8c00086	-0.22	13.46	um10956	-0.26	15.60
8c00087	-1.68	13.94	um10957	-0.14	13.05
8c00088	0.06	12.45	um10958	-0.11	10.69
8c00089	-0.07	12.33	um10959	0.48	9.95
8c00090	-0.51	13.01	um10960	-0.94	9.40
8c00091	0.16	12.93	um10961	-0.54	14.97
8c00092	-0.02	10.80	um10962	0.14	8.76
8c00093	-0.06	10.35	um10963	0.20	12.07
8c00094	0.26	11.77	um10964	-0.15	11.43
8c00095	0.01	13.04	um10965	0.34	11.63
8c00096	-0.30	13.33	um10966	0.30	12.05
8c00097	-0.39	12.29	um10968	-0.36	12.86
8c00098	0.00	9.56	um10969	-0.16	12.99
8c00099	0.04	8.71	um10970	-0.08	12.59
8c00100	0.01	11.85	um10971	0.23	11.01
8c00101	-0.57	11.55	um10972	-0.18	8.99
8c00102	0.41	10.47	um10973	0.92	14.09
8c00103	-0.14	11.87	um10974	-0.41	11.02

8c00104	0.01	10.53	um10975	-0.49	14.90
8c00105	0.36	12.72	um10976	-1.04	11.92
8c00106	0.13	11.35	um10979	0.18	11.32
8c00107	0.24	12.95	um10980	0.84	13.03
8c00108	0.46	13.00	um10981	-0.51	13.03
8c00109	-0.49	13.04	um10982	0.57	10.95
8c00110	0.38	12.34	um10983	0.29	10.75
8c00111	-0.20	13.29	um10984	-0.12	12.71
8c00112	0.36	9.84	um10985	-0.77	12.55
8c00113	-0.07	9.46	um10986	0.50	11.44
8c00114	-0.44	13.05	um10988	0.13	12.39
8c00115	0.20	12.49	um10989	0.37	14.11
8c00116	-0.33	13.15	um10990	0.19	10.57
8c00117	0.17	13.49	um10991	-0.07	11.96
8c00118	-0.11	10.49	um10992	0.67	13.43
8c00119	0.90	12.88	um10993	-0.10	12.08
8c00120	-0.84	11.61	um10996	-0.03	10.86
8c00121	-0.18	11.83	um10997	0.00	11.63
8c00122	-0.61	10.11	um10998	-0.07	11.35
8c00123	0.45	15.37	um10999	0.26	12.20
8c00124	0.37	10.76	um11000	-0.29	13.19
8c00125	0.44	14.31	um11001	-0.49	13.00
8c00126	0.05	8.98	um11002	0.96	9.92
8c00127	0.15	8.86	um11005	0.39	11.76
8c00128	-0.02	8.87	um11007	1.62	12.36
8c00129	-0.17	9.63	um11008	0.36	9.41
8c00130	-0.24	11.87	um11009	-0.06	11.30
8c00131	0.22	11.39	um11010	0.45	11.43
8d00001	-0.89	11.62	um11012	-0.05	13.00
8d00002	-0.85	10.65	um11013	-0.95	11.20
8d00003	-0.04	8.76	um11014	-0.71	13.43
8d00004	0.23	12.93	um11015	1.54	12.47
8d00005	0.09	10.47	um11016	-0.22	9.83
8d00006	0.35	11.12	um11017	0.20	12.60
8d00007	0.31	12.02	um11018	0.56	12.38
8d00008	0.82	11.46	um11019	0.58	11.01
8d00009	0.05	9.20	um11020	3.05	14.05
8d00010	0.92	12.92	um11021	-0.15	12.42
8d00011	0.01	11.26	um11022	-1.58	11.58
8d00012	0.15	13.87	um11023	-0.37	12.39
8d00013	0.36	11.41	um11024	0.50	11.24
8d00014	-0.52	13.93	um11025	1.22	11.93
8d00015	-0.24	13.86	um11026	0.38	11.05
8d00016	-0.07	9.94	um11027	0.35	12.66
8d00017	-0.05	12.54	um11028	0.16	12.41
8d00018	-0.25	13.48	um11029	0.14	12.69
8d00019	-0.88	10.39	um11030	-0.19	10.59
8d00020	-0.34	13.38	um11031	0.90	14.07
8d00021	-0.59	10.95	um11032	0.96	14.06
8d00022	0.01	8.83	um11033	0.94	14.99
8d00023	-0.18	12.44	um11034	1.01	14.54
8d00024	-0.62	12.23	um11035	0.41	11.25
8d00025	-0.10	8.95	um11036	0.28	11.53
8d00026	-0.04	8.62	um11038	1.35	13.32

8d00027	0.06	8.64	um11041	0.64	11.58
8d00028	0.23	12.78	um11042	0.33	12.33
8d00029	-2.78	12.90	um11043	0.00	12.34
8d00030	0.10	12.52	um11046	0.88	11.32
8d00031	0.33	11.38	um11047	-0.42	12.47
8d00032	0.05	13.66	um11048	-0.38	12.46
8d00033	-0.03	8.59	um11049	-0.08	11.71
8d00034	-0.50	13.17	um11050	-0.18	12.44
8d00035	0.59	12.82	um11051	0.92	15.19
8d00036	-0.21	12.07	um11052	-0.32	12.65
8d00037	0.28	14.35	um11053	0.57	12.10
8d00038	0.01	9.16	um11054	-0.69	15.90
8d00039	0.50	12.62	um11055	0.97	12.63
8d00040	0.25	11.20	um11056	0.47	12.15
8d00041	0.13	11.68	um11057	-0.48	8.61
8d00042	0.27	13.47	um11058	-0.04	8.61
8d00043	0.15	10.97	um11059	0.00	8.49
8d00044	3.15	14.17	um11060	-0.26	8.61
8d00045	0.39	9.47	um11061	-0.57	8.67
8d00046	-0.05	10.65	um11062	-0.09	8.66
8d00047	0.01	8.62	um11063	-2.03	10.50
8d00048	0.06	9.57	um11064	0.08	8.33
8d00049	0.22	9.11	um11065	-1.05	10.82
8d00050	0.37	12.23	um11066	-0.13	8.50
8d00051	0.16	12.44	um11067	0.69	15.22
8d00052	0.31	10.42	um11068	1.91	11.90
8d00053	0.99	11.70	um11070	1.58	9.73
8d00054	-0.90	11.81	um11071	-0.55	12.47
8d00055	0.43	12.36	um11072	-0.17	8.77
8d00056	0.08	13.14	um11073	0.34	11.50
8d00057	-0.11	13.66	um11074	-0.21	11.83
8d00058	-0.01	12.90	um11075	-0.09	11.69
8d00059	0.07	10.94	um11076	0.49	10.59
8d00060	0.02	12.68	um11077	0.08	12.59
8d00061	-0.08	10.85	um11078	0.01	13.65
8d00062	0.27	11.36	um11079	0.68	11.92
8d00063	-0.50	11.77	um11080	0.69	11.79
8d00064	0.09	10.65	um11081	0.23	10.88
8d00065	0.07	9.13	um11082	-0.14	8.55
8d00066	0.47	10.78	um11083	-0.08	12.83
8d00067	-0.28	12.46	um11084	0.71	13.04
8d00068	-0.24	14.51	um11085	-0.02	12.11
8d00069	0.12	10.44	um11086	0.14	13.89
8d00070	0.53	12.30	um11087	-0.48	10.86
8d00071	-0.59	12.55	um11088	-0.26	11.89
8d00072	-0.17	13.10	um11089	-0.16	13.57
8d00073	0.77	10.37	um11090	-0.67	10.15
8d00074	-0.01	8.73	um11091	0.00	11.29
8d00075	0.06	9.61	um11092	0.07	14.31
8d00076	-0.34	11.24	um11093	-0.47	11.95
8d00077	0.20	11.92	um11094	-0.04	8.81
8d00078	0.54	10.51	um11095	-0.42	11.34
8d00079	-0.05	8.94	um11096	-0.27	15.20
8d00080	-0.10	9.06	um11097	0.41	14.83

8d00081	0.05	9.89	um11098	-0.41	15.80
8d00082	0.20	11.58	um11099	-0.30	12.12
8d00083	0.39	13.02	um11100	0.50	12.01
8d00084	-0.06	12.56	um11103	-0.07	14.83
8d00085	-0.06	12.87	um11104	0.02	15.22
8d00086	0.33	13.64	um11105	-0.76	15.81
8d00087	-0.32	12.72	um11106	0.05	14.79
8d00088	-0.15	12.90	um11107	-0.54	13.02
8d00089	0.30	14.15	um11109	-0.34	13.62
8d00090	-0.21	12.41	um11110	-0.17	10.95
8d00091	0.32	14.87	um11111	-1.67	9.67
8d00092	-0.07	8.66	um11112	-3.00	10.41
8d00093	0.03	11.84	um11113	-0.35	9.53
8d00094	-0.16	11.84	um11114	0.27	11.85
8d00095	0.21	12.31	um11115	-0.23	11.96
8d00096	0.10	12.20	um11116	2.20	13.06
8d00097	-0.16	12.48	um11117	2.27	12.90
8d00098	0.45	13.34	um11118	2.42	12.15
8d00099	-0.23	13.47	um11120	-0.27	13.65
8d00100	0.22	12.01	um11121	-0.92	15.36
8d00101	-0.07	12.80	um11125	-0.34	13.68
8d00102	0.08	10.94	um11126	0.31	11.02
8d00103	0.02	14.66	um11127	0.51	10.59
8d00104	0.04	12.13	um11129	-2.27	10.85
8d00105	-0.62	13.05	um11130	-0.18	13.34
8d00106	-0.01	12.60	um11131	0.33	13.32
8d00107	0.02	12.16	um11132	0.56	11.40
8d00108	0.12	11.47	um11133	1.09	13.28
8d00109	-1.52	14.73	um11134	-0.13	12.87
8d00110	-0.25	11.82	um11135	-0.11	15.62
8d00111	-0.99	9.43	um11136	0.36	12.09
8d00112	0.80	12.53	um11137	0.46	11.71
8d00113	0.03	10.52	um11138	-0.58	9.43
8d00114	0.02	8.89	um11139	-0.53	15.01
8d00115	-0.16	8.90	um11141	-0.15	13.27
8d00116	-0.37	10.99	um11142	-0.13	12.21
9c00001	-0.02	15.68	um11146	-0.22	8.61
9c00002	-0.26	11.70	um11147	-1.63	10.01
9c00003	-0.52	12.66	um11148	0.13	10.87
9c00004	-0.07	8.76	um11149	0.46	9.36
9c00005	2.15	11.67	um11150	0.17	9.21
9c00006	-0.06	11.93	um11151	-0.12	11.82
9c00007	-0.58	12.78	um11152	0.10	12.14
9c00008	-0.15	14.14	um11153	0.20	13.55
9c00009	-0.01	13.65	um11155	-0.91	9.48
9c00010	0.03	11.89	um11156	-0.21	8.82
9c00011	-0.47	12.30	um11157	0.24	12.86
9c00012	0.41	10.70	um11159	-0.09	8.54
9c00013	0.13	8.56	um11160	0.18	11.46
9c00014	0.11	12.20	um11161	-0.66	15.85
9c00015	-0.33	10.71	um11162	0.86	13.16
9c00016	0.23	12.88	um11163	0.04	11.42
9c00017	-0.85	12.63	um11164	0.70	12.81
9c00018	-0.10	11.54	um11165	0.16	10.91

9c00019	0.04	12.94	um11166	-1.37	13.57
9c00020	-0.30	10.92	um11167	-0.34	12.18
9c00021	-0.18	10.42	um11168	0.28	12.35
9c00022	-0.31	12.21	um11169	-0.11	10.06
9c00023	0.13	10.21	um11170	0.96	14.29
9c00024	-0.01	11.02	um11171	0.39	12.80
9c00025	-0.27	14.15	um11172	-0.05	12.38
9c00026	-0.46	11.41	um11173	-0.20	13.50
9c00027	-0.25	12.25	um11174	0.23	12.90
9c00028	0.04	9.88	um11176	0.09	11.46
9c00029	-0.01	13.03	um11177	-0.23	12.92
9c00030	-0.41	13.27	um11178	0.52	11.43
9c00031	1.20	11.66	um11179	2.78	12.85
9c00032	0.05	10.58	um11180	0.16	12.35
9c00033	-0.39	12.54	um11181	-0.67	10.40
9c00034	-0.38	14.45	um11182	-0.13	11.55
9c00035	-0.14	13.60	um11183	-2.89	11.19
9c00036	0.52	12.14	um11184	-0.11	12.28
9c00037	-0.25	9.24	um11185	1.25	11.77
9c00038	0.05	9.64	um11186	1.19	11.62
9c00039	0.06	11.60	um11187	-1.37	13.03
9c00040	-0.23	10.34	um11188	-0.74	10.80
9c00041	0.00	8.83	um11189	0.30	11.20
9c00042	-0.17	12.81	um11190	0.54	10.47
9c00043	-0.02	9.35	um11191	1.09	12.10
9c00044	-1.19	13.77	um11192	0.67	10.96
9c00045	0.03	8.73	um11193	0.43	10.14
9c00046	-0.16	11.59	um11194	0.28	11.55
9c00047	0.28	13.27	um11195	-0.17	11.73
9c00048	-0.16	11.71	um11196	-0.25	9.95
9c00049	-0.10	12.75	um11197	1.73	12.32
9c00050	-0.56	12.06	um11198	-0.10	10.33
9c00051	0.08	12.70	um11199	0.33	11.75
9c00052	-0.16	9.42	um11200	-0.12	11.47
9c00053	-0.19	9.74	um11201	0.04	10.80
9c00054	-0.76	11.33	um11202	0.15	15.34
9c00055	-1.39	10.44	um11203	2.22	13.28
9c00056	0.00	12.58	um11204	-0.36	12.45
9c00057	-0.96	12.74	um11205	0.39	13.58
9c00058	-0.15	12.10	um11206	0.14	9.31
9c00059	-0.65	12.03	um11207	-1.02	13.21
9c00060	-0.05	13.28	um11208	-0.18	11.82
9c00061	-0.07	13.05	um11209	0.11	14.28
9c00062	0.82	13.48	um11210	-0.70	13.99
9c00063	0.13	14.03	um11211	-0.21	8.76
9c00064	0.74	12.75	um11212	-0.13	14.52
9c00065	-0.43	11.75	um11213	0.19	11.31
9c00066	-0.06	8.78	um11214	-0.16	12.49
9c00067	-0.11	10.23	um11215	-0.80	11.11
9c00068	-0.11	8.85	um11217	-0.07	13.59
9c00069	0.17	15.87	um11218	-0.33	12.49
9c00070	-0.06	11.36	um11219	0.76	13.41
9c00071	-0.93	11.13	um11220	-0.74	11.31
9c00072	-0.18	9.67	um11221	-0.36	11.31

9c00073	-0.06	11.55	um11222	-0.02	12.11
9c00074	-0.73	10.25	um11223	0.03	12.30
9c00075	0.32	12.09	um11224	0.39	10.73
9c00076	-0.75	12.04	um11225	0.15	11.88
9c00077	0.19	11.28	um11226	0.09	11.84
9c00078	0.07	10.21	um11227	0.15	12.86
9c00079	0.51	11.68	um11228	-0.32	12.60
9c00080	-0.07	12.84	um11229	2.49	11.70
9c00081	-0.27	9.11	um11230	0.05	12.08
9c00082	-0.12	8.92	um11231	0.69	12.13
9c00083	-0.08	13.71	um11232	-0.49	15.73
9c00084	0.61	9.82	um11233	0.26	15.11
9c00085	1.26	13.92	um11234	-0.13	11.67
9c00086	-0.16	11.92	um11235	0.35	13.31
9c00087	0.73	13.49	um11237	0.40	9.20
9c00088	-0.30	10.33	um11238	0.95	15.00
9c00089	-0.64	11.60	um11239	0.16	11.99
9c00090	0.29	10.80	um11240	0.24	10.78
9c00091	-0.23	11.77	um11241	0.47	12.18
9c00092	0.46	12.43	um11242	0.06	8.84
9c00093	-0.17	8.86	um11243	1.03	12.53
9c00094	-0.10	10.40	um11244	-0.33	8.54
9c00095	-0.73	12.32	um11245	0.13	9.21
9c00096	-0.11	11.53	um11246	0.63	11.63
9c00097	-0.54	12.22	um11247	0.57	10.78
9c00098	0.44	10.29	um11248	0.56	11.55
9c00099	0.08	9.83	um11249	0.37	10.77
9c00100	0.69	12.29	um11250	-0.11	8.63
9c00101	0.30	11.74	um11251	1.11	10.25
9c00102	-0.40	11.65	um11253	-0.33	12.79
9c00103	-0.10	10.96	um11254	0.19	13.78
9c00104	-0.29	12.27	um11255	-0.46	11.87
9c00105	-0.68	11.51	um11256	0.00	14.56
9c00106	-0.02	13.26	um11257	0.03	12.27
9c00107	-0.17	12.46	um11258	1.13	13.52
9c00108	0.02	12.75	um11259	0.07	15.10
9c00109	-0.09	9.65	um11260	-0.57	14.13
9c00110	-0.32	11.26	um11261	0.25	15.26
9c00111	-0.08	13.18	um11262	0.10	11.87
9c00112	-0.22	11.68	um11263	-0.81	12.72
9c00113	-0.19	9.78	um11265	0.08	13.09
9c00114	-0.42	14.91	um11266	-0.94	11.77
9c00115	-0.78	13.17	um11267	-0.71	12.45
9c00116	-1.15	13.77	um11268	0.08	12.69
9c00117	-0.02	13.30	um11269	-0.82	11.54
9c00118	-0.79	14.36	um11270	-0.64	14.68
9c00119	-0.27	13.30	um11271	-1.02	13.79
9c00120	-0.36	10.33	um11272	0.28	12.42
9c00121	-0.26	10.69	um11273	-0.33	12.35
9c00122	-0.16	11.72	um11274	0.41	15.05
9c00123	0.32	11.49	um11275	0.61	11.93
9c00124	-0.47	10.06	um11276	-0.44	14.12
9c00125	-0.25	10.88	um11277	0.41	14.16
9c00126	0.43	13.12	um11278	-0.42	14.74

9c00127	-0.28	13.06	um11279	0.15	11.43
9c00128	-0.36	12.25	um11280	0.84	12.28
9c00129	-0.31	11.56	um11281	-0.22	11.38
9c00130	0.12	12.01	um11282	-0.09	10.01
9c00131	-0.06	13.47	um11283	0.25	13.82
9c00132	-0.72	12.93	um11284	-1.23	11.60
9c00133	-0.36	12.06	um11285	-1.11	13.63
9c00134	0.42	13.68	um11288	0.57	14.56
9c00135	-0.64	11.35	um11289	0.48	12.45
9c00136	-0.52	13.59	um11290	0.42	13.39
9c00137	0.02	11.90	um11291	0.00	12.59
9c00138	-0.37	9.84	um11292	0.22	10.40
9c00139	-0.71	15.14	um11293	0.47	12.97
9c00140	0.67	12.33	um11294	-0.22	13.15
9c00141	-0.01	10.34	um11295	0.14	10.35
9c00142	0.46	11.99	um11296	-1.06	11.87
9c00143	-0.19	11.94	um11297	-0.79	11.66
9c00144	0.70	11.28	um11298	0.77	11.57
9c00145	0.30	11.71	um11299	0.32	12.02
9c00146	-0.29	12.55	um11300	0.67	12.54
9c00147	0.11	8.44	um11301	0.05	12.20
9c00148	0.50	11.87	um11302	0.33	12.55
9c00149	-0.29	11.93	um11303	0.81	12.95
9c00150	-0.31	11.47	um11304	0.60	11.56
9c00151	-0.06	14.19	um11305	0.16	10.78
9c00152	-0.13	12.99	um11306	0.60	12.81
9c00153	-0.06	10.01	um11308	0.39	12.67
9c00154	0.30	12.20	um11309	-0.10	13.23
9c00155	0.31	13.40	um11310	0.41	11.18
9c00156	-0.10	10.84	um11311	0.51	11.86
9c00157	0.20	8.65	um11312	0.30	12.01
9c00158	0.10	12.98	um11313	0.33	11.73
9c00159	0.11	11.77	um11314	0.14	13.12
9c00160	-0.49	11.70	um11315	0.91	10.78
9c00161	0.62	11.92	um11316	0.07	11.42
9c00162	-0.27	12.12	um11317	0.37	11.81
9c00163	-0.34	11.81	um11318	-0.18	12.68
9c00164	0.19	12.89	um11319	-0.45	13.39
9c00165	-0.43	13.14	um11321	1.82	11.86
9c00166	0.00	11.45	um11322	-0.25	10.26
9c00167	-0.16	12.04	um11323	-0.46	13.04
9c00168	0.27	12.12	um11325	-0.13	12.92
9c00169	0.41	11.37	um11326	-0.23	11.60
9c00170	-0.29	11.60	um11327	-0.54	13.02
9c00171	0.05	11.84	um11328	-0.12	12.08
9c00172	0.46	10.53	um11330	0.22	11.63
9c00173	0.07	11.84	um11331	-0.26	12.77
9c00174	0.48	13.30	um11332	0.24	9.38
9c00175	0.70	12.24	um11333	0.12	12.30
9c00176	0.61	13.81	um11334	0.09	11.66
9c00177	0.42	12.42	um11335	0.53	11.90
9c00178	0.05	12.60	um11336	0.11	12.06
9c00179	-0.09	13.40	um11337	1.09	9.80
9c00180	0.57	12.38	um11338	-1.32	15.04

9c00181	-0.15	11.61	um11339	-0.78	15.78
9c00182	0.31	11.84	um11340	-0.44	11.69
9c00183	-0.25	9.13	um11341	0.37	11.80
9c00184	0.41	12.04	um11342	0.35	11.28
9c00185	-1.02	13.29	um11343	-0.40	11.18
9c00186	-0.02	11.82	um11344	-0.14	11.80
9c00187	0.43	12.85	um11345	-1.10	11.64
9c00188	-0.21	15.86	um11346	-0.85	10.93
9c00189	0.07	13.56	um11347	0.35	11.67
9c00190	-0.26	12.72	um11348	0.28	12.23
9c00191	-0.08	9.46	um11349	0.61	13.98
9c00192	0.02	14.35	um11350	0.17	13.27
9c00193	0.00	11.94	um11352	0.09	12.27
9c00194	0.50	12.65	um11353	-0.71	14.19
9c00195	-0.24	11.78	um11354	0.46	11.17
9c00196	-0.36	15.16	um11355	-0.58	13.47
9c00197	-0.27	11.69	um11357	0.34	10.85
9c00198	0.54	11.71	um11358	-0.43	11.20
9c00199	-0.06	10.76	um11359	-0.59	11.32
9c00200	-0.12	11.65	um11360	-0.16	10.83
9c00201	-0.07	10.00	um11361	-0.10	11.92
9c00202	-0.55	12.25	um11362	-0.66	10.50
9c00203	0.16	10.24	um11363	-0.55	13.02
9c00204	-0.57	15.45	um11364	-0.24	10.24
9c00205	-0.27	13.90	um11365	0.21	9.01
9c00206	-0.13	11.44	um11366	0.88	11.92
9c00207	0.16	11.74	um11367	-0.09	12.13
9c00208	0.86	12.61	um11368	2.03	13.56
9c00209	0.30	14.36	um11369	0.84	10.58
9c00210	-0.36	10.60	um11370	0.06	10.20
9c00211	-0.16	12.91	um11371	0.20	9.85
9c00212	0.41	13.30	um11373	1.21	12.98
9c00213	-0.24	10.64	um11374	1.06	13.81
9c00214	0.10	13.11	um11375	-0.28	8.77
9c00215	0.25	12.19	um11376	-0.06	9.37
9c00216	-0.09	11.52	um11377	-0.01	8.92
9c00217	0.52	13.04	um11378	0.28	14.17
9c00218	0.11	12.45	um11379	-0.15	12.07
9c00219	0.23	12.97	um11381	-2.29	11.47
9c00220	-0.06	14.60	um11382	1.07	13.57
9c00221	0.42	11.47	um11383	0.87	12.67
9c00222	0.13	12.14	um11384	0.74	11.80
9c00223	0.15	9.67	um11385	0.36	11.40
9c00224	-0.57	11.63	um11386	0.10	11.83
9c00225	0.36	15.35	um11387	-0.29	8.66
9c00226	-0.76	11.95	um11388	-0.24	12.09
9c00227	-0.77	12.65	um11390	0.01	11.95
9c00228	-0.32	10.02	um11391	-1.17	13.25
9c00229	0.11	9.40	um11392	-0.61	13.17
9c00230	-0.16	11.96	um11394	-0.65	12.53
9c00231	0.02	12.22	um11396	0.06	11.88
9c00232	0.97	12.70	um11397	0.33	9.26
9c00233	0.37	11.78	um11398	0.40	12.08
9c00234	-0.17	13.40	um11399	0.01	11.13

9c00235	-0.08	13.78	um1140[illegible]	-0.50	15.90
9c00236	0.23	12.07	um1140[illegible]	-0.32	11.39
9c00237	-0.07	10.40	um1140[illegible]	0.39	11.77
9c00238	0.30	13.09	um1140[illegible]	3.96	12.41
9c00239	0.11	13.76	um1140[illegible]	0.12	12.67
9c00240	-0.04	11.89	um1140[illegible]	-0.38	11.24
9c00241	0.18	12.10	um1140[illegible]	0.32	13.04
9c00242	-0.02	8.76	um1140[illegible]	0.54	9.85
9c00243	-0.70	11.93	um1140[illegible]	-0.97	11.46
9c00244	0.10	12.37	um1141[illegible]	-0.17	12.13
9c00245	0.23	8.90	um1141[illegible]	0.22	14.74
9c00246	-0.01	12.63	um1141[illegible]	0.24	15.00
9c00247	3.00	10.82	um1141[illegible]	0.61	11.47
9c00248	0.03	12.11	um1141[illegible]	0.14	13.43
9c00249	0.45	11.35	um1141[illegible]	0.14	8.95
9c00250	-0.26	10.03	um1141[illegible]	-0.02	8.65
9c00251	-0.16	10.92	um1141[illegible]	-0.09	8.63
9c00252	0.28	12.04	um1141[illegible]	-0.12	12.04
9c00253	-0.22	12.16	um1142[illegible]	-0.11	8.94
9c00254	0.07	12.73	um1142[illegible]	0.09	14.00
9c00255	-0.07	8.87	um1142[illegible]	-0.46	10.30
9c00256	1.92	13.18	um1142[illegible]	-0.39	13.89
9c00257	-0.18	12.73	um1142[illegible]	0.09	11.42
9c00258	-0.46	10.76	um1142[illegible]	-1.20	12.33
9c00259	-0.17	8.72	um1142[illegible]	0.12	11.81
9c00260	-1.14	12.09	um1142[illegible]	0.89	11.13
9c00261	0.40	12.63	um1142[illegible]	-0.30	11.40
9c00262	-0.46	10.54	um1142[illegible]	-0.21	12.80
9c00263	-0.02	12.91	um1143[illegible]	0.35	12.56
9c00264	-0.45	10.55	um1143[illegible]	0.30	11.49
9c00265	0.13	11.77	um1143[illegible]	0.34	11.74
9c00266	-0.37	14.66	um1143[illegible]	-0.23	12.49
9c00267	-0.11	12.69	um1143[illegible]	1.91	11.29
9c00268	-0.27	12.83	um1143[illegible]	0.56	12.20
9c00269	-0.10	13.42	um1143[illegible]	0.11	13.75
9c00270	0.82	13.47	um1143[illegible]	-0.07	14.25
9c00271	0.39	13.92	um1143[illegible]	0.75	11.86
9c00272	0.17	11.89	um1143[illegible]	0.01	12.31
9c00273	-0.24	12.19	um1144[illegible]	0.11	11.35
9c00274	-0.52	13.35	um1144[illegible]	-0.37	15.78
9c00275	-0.34	9.56	um1144[illegible]	-0.23	12.41
9c00276	-0.11	8.50	um1144[illegible]	-0.02	8.78
9c00277	-0.08	8.73	um1144[illegible]	0.17	10.09
9c00278	-0.20	13.91	um1144[illegible]	0.45	10.34
9c00279	0.25	11.91	um1144[illegible]	0.10	12.52
9c00280	0.12	10.28	um1144[illegible]	1.77	13.28
9c00281	-0.28	13.03	um1144[illegible]	0.27	11.02
9c00282	-0.38	11.08	um1145[illegible]	2.11	13.11
9c00283	0.51	13.35	um1145[illegible]	0.82	12.53
9c00284	-0.18	12.28	um1145[illegible]	0.55	11.43
9c00285	0.78	12.19	um1145[illegible]	0.21	12.25
9c00286	0.27	11.61	um1145[illegible]	0.44	11.55
9c00287	0.34	10.33	um1145[illegible]	0.32	11.13
9c00288	0.08	10.07	um1145[illegible]	-0.49	11.79

9c00289	0.14	10.51	um11458	-0.53	11.28
9c00290	-0.44	12.36	um11459	0.84	10.75
9c00291	0.12	8.68	um11460	-0.02	11.99
9c00292	-0.13	9.04	um11461	0.23	12.35
9c00293	0.01	12.70	um11462	0.81	11.17
9c00294	-0.54	14.63	um11464	-0.38	11.81
9c00295	-0.10	12.34	um11465	-3.53	11.68
9c00296	-0.05	11.26	um11467	0.20	14.85
9c00297	0.33	9.15	um11468	-0.01	11.87
9c00298	-0.08	8.68	um11469	0.20	12.14
9c00299	-0.17	12.90	um11470	-0.31	12.52
9c00300	-0.09	11.08	um11471	0.15	10.78
9c00301	-0.05	13.51	um11472	-0.02	12.42
9c00302	0.01	10.05	um11474	0.05	11.38
9c00303	0.03	12.58	um11475	0.55	11.40
9c00304	0.19	12.40	um11476	-0.24	12.04
9c00305	0.46	13.11	um11477	0.15	11.61
9c00306	0.22	12.88	um11478	0.27	10.81
9c00307	0.17	9.77	um11479	0.26	13.32
9c00308	-0.46	9.49	um11480	0.60	11.76
9c00309	-0.01	10.91	um11481	0.38	10.35
9c00310	-0.02	13.60	um11482	0.10	12.79
9c00311	0.24	13.79	um11483	-0.62	12.56
9c00312	-0.26	12.02	um11484	-0.03	8.99
9c00313	-0.01	9.17	um11485	-0.20	11.35
9c00314	0.02	13.11	um11486	0.14	15.38
9c00315	-0.09	13.05	um11487	0.32	11.71
9c00316	-0.04	12.17	um11488	0.36	13.65
9c00317	-1.08	13.64	um11489	-0.06	8.58
9c00318	0.15	10.76	um11490	0.38	12.58
9c00319	-0.33	11.28	um11491	0.47	10.26
9c00320	-0.10	12.36	um11494	-0.04	12.76
9c00321	0.12	10.49	um11495	0.22	14.12
9c00322	0.58	13.17	um11496	-0.29	13.24
9c00323	-0.02	9.61	um11497	-0.74	13.07
9c00324	-0.23	14.39	um11498	-0.25	13.87
9c00325	0.18	15.10	um11499	0.39	14.59
9c00326	0.12	12.87	um11500	-0.68	13.06
9c00327	0.03	10.62	um11501	-0.46	11.90
9c00328	0.11	13.60	um11502	-1.38	11.06
9c00329	-0.92	12.42	um11504	0.87	10.33
9c00330	-0.25	11.57	um11505	0.60	9.52
9c00331	-0.12	12.32	um11506	-2.03	9.72
9c00332	-0.22	11.61	um11509	-0.15	11.40
9c00333	0.71	12.50	um11510	0.22	11.05
9c00334	-0.39	12.57	um11511	0.49	13.36
9c00335	-0.22	13.02	um11512	-0.47	10.29
9c00336	-0.25	12.74	um11513	2.26	11.06
9c00337	-0.18	9.82	um11514	2.15	11.30
9c00338	-0.36	14.86	um11515	-0.36	8.94
9c00339	-0.10	11.28	um11516	1.07	12.96
9c00340	-0.42	12.63	um11517	-0.27	15.33
9c00341	-0.07	11.72	um11518	-0.18	11.92
9c00342	-0.03	10.03	um11519	-0.90	14.41

9c00343	-0.22	8.75	um11520	-0.44	14.47
9c00344	-0.35	14.26	um11521	-0.10	10.36
9c00345	-0.15	10.83	um11522	-0.40	11.76
9c00346	0.68	13.97	um11523	0.23	13.63
9c00347	-0.62	12.14	um11524	0.12	9.46
9c00348	-0.93	13.60	um11525	0.18	13.67
9c00349	-0.12	12.06	um11526	0.10	15.27
9c00350	0.50	12.32	um11528	-2.39	11.56
9c00351	0.39	11.57	um11530	0.23	11.34
9c00352	1.12	13.33	um11531	1.62	11.90
9c00353	-0.55	12.49	um11532	-0.10	11.40
9c00354	0.44	12.17	um11533	0.25	15.02
9c00355	-0.35	13.19	um11534	0.96	14.18
9c00356	-0.03	11.78	um11535	-0.02	15.28
9c00357	-0.07	12.05	um11536	0.18	15.16
9c00358	-0.50	14.52	um11537	0.28	14.87
9c00359	-0.77	14.66	um11538	0.02	10.80
9c00360	-0.25	9.32	um11539	0.20	12.63
9c00361	-0.18	14.01	um11540	0.22	10.35
9c00362	-0.58	13.19	um11541	0.62	12.55
9c00363	-0.31	11.53	um11544	-1.13	11.83
9c00364	-0.06	8.76	um11545	0.26	11.24
9c00365	-0.08	10.89	um11546	-0.23	12.96
9c00366	-0.08	13.03	um11548	0.13	12.88
9c00367	0.19	12.38	um11549	0.42	12.06
9c00368	-0.01	13.26	um11550	0.07	11.55
9c00369	0.10	11.69	um11551	0.15	14.58
9c00370	0.31	10.72	um11552	0.01	12.56
9c00371	0.20	10.68	um11553	0.24	10.72
9c00372	0.60	12.35	um11554	-3.13	13.33
9c00373	0.31	11.43	um11555	-0.10	8.64
9c00374	0.06	12.11	um11556	0.00	13.90
9c00375	0.21	13.19	um11557	0.53	11.26
9c00376	-0.32	13.68	um11558	1.14	14.33
9c00377	0.28	12.43	um11559	0.17	11.02
9c00378	0.76	13.42	um11560	-0.44	11.53
9c00379	-0.34	12.55	um11561	0.12	14.54
9c00380	1.00	11.58	um11562	1.56	11.05
9c00381	0.31	13.85	um11563	-0.27	12.77
9c00382	1.45	14.97	um11564	-0.40	11.12
9c00383	0.08	8.72	um11565	0.32	11.16
9c00384	0.55	12.51	um11567	0.45	13.10
9c00385	0.73	12.56	um11568	0.12	12.47
9c00386	0.40	11.52	um11569	1.73	11.32
9c00387	-0.14	12.83	um11570	0.39	11.26
9c00388	1.43	11.85	um11571	0.42	11.21
9c00389	0.14	11.69	um11572	0.29	11.44
9c00390	0.09	13.08	um11574	-0.89	11.66
9c00391	0.26	11.52	um11575	-0.59	12.12
9c00392	-1.01	10.88	um11576	-0.40	15.70
9c00393	-0.45	10.94	um11577	-0.35	12.67
9c00394	-0.33	11.09	um11578	-0.38	13.64
9c00395	0.04	11.90	um11579	-0.41	9.48
9c00396	0.00	11.56	um11580	2.23	12.47

9c00397	-2.55	13.22	um11581	-0.15	10.83
9c00398	-0.25	12.15	um11582	0.30	11.79
9c00399	-0.55	12.77	um11583	0.11	13.03
9c00400	0.55	11.57	um11584	0.08	12.84
9c00401	0.35	12.17	um11585	-0.59	8.89
9c00402	0.00	11.99	um11586	1.24	11.07
9c00403	0.12	11.52	um11587	-0.01	12.55
9c00404	-0.56	11.97	um11588	-0.63	15.88
9c00405	0.07	9.12	um11589	0.16	14.50
9c00406	0.59	13.58	um11590	0.60	14.02
9c00407	0.25	14.11	um11591	-0.16	13.20
9c00408	0.28	13.29	um11592	-0.10	9.42
9c00409	-0.39	14.50	um11593	1.72	11.31
9c00410	0.49	10.45	um11594	-0.02	11.82
9c00411	-0.07	12.38	um11595	-0.95	13.66
9c00412	0.04	9.23	um11596	1.18	14.22
9c00413	-0.07	12.00	um11597	0.62	10.92
9c00414	0.61	12.10	um11598	-1.14	13.79
9c00415	0.42	13.39	um11599	-0.12	11.30
9c00416	-0.26	10.14	um11600	-0.51	13.37
9c00417	-0.08	13.67	um11601	-0.10	11.88
9c00418	0.25	12.96	um11602	0.11	11.87
9c00419	0.48	10.01	um11603	0.63	10.44
9c00420	0.07	15.54	um11604	-0.03	14.97
9c00421	-0.10	10.75	um11605	0.52	10.78
9c00422	0.04	11.02	um11606	0.23	14.43
9c00423	0.71	14.06	um11608	0.69	10.36
9c00424	-0.30	9.41	um11610	1.86	14.33
9c00425	-0.03	8.65	um11612	-0.49	11.80
9c00426	-0.22	12.24	um11613	0.13	10.76
9c00427	-0.58	14.14	um11614	-0.34	13.18
9c00428	0.32	14.54	um11615	0.02	10.74
9c00429	-0.43	12.44	um11616	-0.23	12.90
9c00430	-0.37	11.23	um11617	0.03	10.44
9c00431	-0.32	10.80	um11618	0.29	11.28
9c00432	0.07	10.50	um11619	0.09	14.94
9c00433	0.02	10.25	um11620	0.38	11.31
9c00434	0.21	13.69	um11623	-0.05	11.05
9c00435	0.49	10.90	um11624	-0.63	13.34
9c00436	-0.45	13.15	um11625	0.26	15.25
9c00437	-0.27	12.85	um11627	0.49	12.30
9c00438	-0.08	12.48	um11628	0.14	8.94
9c00439	-0.71	9.91	um11629	0.54	10.36
9c00440	-2.48	11.80	um11630	0.05	12.43
9d00001	-0.36	10.79	um11631	0.59	11.95
9d00002	0.16	11.69	um11632	-0.24	11.73
9d00003	-0.71	12.14	um11633	0.21	12.39
9d00004	-0.24	14.39	um11634	-0.57	11.63
9d00005	0.21	12.71	um11635	0.11	13.35
9d00006	-0.11	10.75	um11636	0.21	11.65
9d00007	-0.66	10.51	um11637	0.60	15.29
9d00008	-0.20	12.18	um11638	-0.53	12.14
9d00009	-0.01	8.73	um11639	0.41	9.07
9d00010	-0.03	8.62	um11641	0.64	12.24

9d00011	0.04	8.71	um11642	0.37	11.07
9d00012	-0.73	12.58	um11643	-0.11	11.81
9d00013	-0.69	14.72	um11644	-0.22	13.71
9d00014	0.24	12.88	um11645	0.13	15.16
9d00015	-0.06	10.85	um11647	-0.21	9.04
9d00016	-0.27	13.55	um11648	-0.02	12.93
9d00017	0.20	13.45	um11649	0.03	11.49
9d00018	-0.78	9.92	um11650	0.21	11.89
9d00019	0.12	12.99	um11651	-2.36	11.00
9d00020	-0.03	9.83	um11652	0.06	11.91
9d00021	0.29	12.17	um11653	0.37	10.02
9d00022	0.70	13.73	um11654	2.00	11.85
9d00023	0.12	12.49	um11655	-0.31	11.16
9d00024	0.11	10.58	um11656	0.00	11.13
9d00025	0.08	10.34	um11657	-0.26	11.86
9d00026	0.30	10.11	um11658	-0.25	8.79
9d00027	-0.17	11.61	um11659	-0.29	12.40
9d00028	-1.23	12.10	um11660	0.67	13.05
9d00029	-0.10	11.07	um11661	-0.02	13.05
9d00030	-0.66	13.70	um11662	0.55	14.78
9d00031	-0.13	12.08	um11663	0.01	10.93
9d00032	0.36	11.92	um11665	0.27	10.95
9d00033	-0.45	12.90	um11666	-0.33	13.55
9d00034	-1.22	13.73	um11667	0.03	11.02
9d00035	-0.49	12.97	um11668	0.10	11.91
9d00036	0.08	12.10	um11669	0.63	11.45
9d00037	-0.15	11.07	um11670	0.15	11.06
9d00038	0.12	12.31	um11671	-0.24	12.49
9d00039	-1.24	14.18	um11672	-0.12	12.03
9d00040	-0.02	11.90	um11673	0.27	12.22
9d00041	-0.61	13.23	um11674	-0.01	11.38
9d00042	0.17	11.26	um11675	0.15	12.24
9d00043	-0.56	11.81	um11677	-0.22	12.64
9d00044	-0.21	10.44	um11679	0.42	11.17
9d00045	-0.89	12.62	um11680	-0.08	11.78
9d00046	0.12	15.56	um11681	-0.14	11.20
9d00047	0.04	8.99	um11682	-0.88	13.16
9d00048	-0.07	8.83	um11683	-1.23	12.00
9d00049	-0.57	14.67	um11684	0.62	13.25
9d00050	-0.25	10.25	um11685	0.21	11.77
9d00051	0.00	12.82	um11686	0.83	10.91
9d00052	-0.22	11.42	um11687	0.79	11.38
9d00053	0.79	11.53	um11688	-0.48	11.47
9d00054	0.05	8.41	um11689	0.08	11.10
9d00055	-0.28	11.19	um11690	0.02	11.32
9d00056	-0.72	9.12	um11691	0.20	11.07
9d00057	-0.03	15.93	um11692	-0.25	12.59
9d00058	-0.91	13.52	um11693	1.27	12.97
9d00059	-0.57	12.38	um11694	-0.27	13.58
9d00060	0.20	13.43	um11695	0.13	10.85
9d00061	0.84	13.99	um11696	0.54	14.49
9d00062	0.43	9.65	um11697	-0.33	11.67
9d00063	-0.38	13.79	um11698	-0.54	14.84
9d00064	-1.62	12.54	um11699	-0.15	10.87

9d00065	0.07	11.79	um11700	-0.47	11.56
9d00066	-0.01	11.79	um11701	-0.01	12.14
9d00067	0.44	12.20	um11702	-0.07	10.97
9d00068	0.06	12.02	um11703	0.42	11.49
9d00069	-0.16	10.92	um11704	-0.22	11.47
9d00070	-0.09	10.94	um11705	0.06	11.49
9d00071	-0.15	11.19	um11706	0.07	12.76
9d00072	-0.15	11.31	um11707	0.10	11.08
9d00073	-0.28	11.65	um11708	-2.10	11.29
9d00074	0.04	13.44	um11709	-0.29	8.89
9d00075	0.09	11.21	um11710	0.11	11.17
9d00076	0.46	10.13	um11712	0.59	11.41
9d00077	-0.59	15.15	um11713	-0.51	10.55
9d00078	0.00	9.32	um11714	-0.88	11.64
9d00079	-0.27	13.12	um11715	0.12	12.42
9d00080	0.35	12.68	um11716	-0.18	15.54
9d00081	0.40	9.57	um11717	-0.28	11.93
9d00082	0.28	11.34	um11718	-0.18	11.90
9d00083	-0.26	14.40	um11719	-0.11	14.92
9d00084	-1.77	9.87	um11720	-1.17	13.23
9d00085	-0.04	10.65	um11721	0.14	11.14
9d00086	-0.31	12.29	um11722	0.33	13.82
9d00087	-0.73	12.92	um11723	-0.28	12.63
9d00088	-1.31	11.47	um11724	0.79	12.12
9d00089	0.13	12.83	um11725	0.11	12.19
9d00090	0.06	12.28	um11726	0.36	12.98
9d00091	0.02	11.74	um11727	0.30	12.07
9d00092	-0.21	13.12	um11728	0.46	10.52
9d00093	-0.32	11.93	um11729	-0.18	9.20
9d00094	0.43	13.15	um11731	-0.77	13.85
9d00095	-0.29	11.87	um11732	-3.09	9.80
9d00096	0.28	10.90	um11733	0.40	13.43
9d00097	0.09	10.32	um11734	0.34	13.06
9d00098	0.41	12.59	um11735	-0.27	11.21
9d00099	-0.48	10.97	um11736	0.23	12.19
9d00100	0.48	13.15	um11738	0.35	12.69
9d00101	0.21	12.45	um11739	0.38	11.60
9d00102	-0.07	9.90	um11740	-0.26	10.83
9d00103	-0.84	11.88	um11741	-1.23	15.05
9d00104	-0.62	14.57	um11742	-0.37	13.11
9d00105	0.46	12.17	um11743	-0.10	11.49
9d00106	0.07	11.85	um11744	1.52	14.01
9d00107	-0.51	11.57	um11745	0.13	10.88
9d00108	-0.21	11.19	um11746	-0.65	12.21
9d00109	0.48	13.36	um11747	-0.22	10.75
9d00110	0.22	11.65	um11749	-0.16	13.16
9d00111	0.40	10.49	um11750	-1.50	12.02
9d00112	0.17	8.62	um11751	-1.15	12.93
9d00113	-0.63	11.09	um11752	-0.49	10.70
9d00114	-0.16	11.06	um11753	0.91	11.96
9d00115	-1.28	12.31	um11754	0.15	9.40
9d00116	0.01	9.25	um11755	-0.12	8.44
9d00117	-0.03	12.60	um11756	0.03	10.48
9d00118	-0.14	10.73	um11757	0.59	11.71

9d00119	0.47	13.20	um11758	-0.21	12.22
9d00120	0.10	13.81	um11759	-0.27	11.29
9d00121	0.30	11.93	um11760	-0.19	12.65
9d00122	-0.03	11.17	um11761	0.47	11.92
9d00123	-0.17	13.12	um11763	0.38	9.09
9d00124	0.35	11.98	um11764	1.25	11.91
9d00125	0.12	11.59	um11765	0.21	11.60
9d00126	-0.30	12.03	um11766	0.53	11.93
9d00127	0.00	10.45	um11767	-0.11	12.42
9d00128	0.11	10.72	um11768	0.38	11.63
9d00129	0.05	9.68	um11769	0.60	13.46
9d00130	-0.68	15.10	um11770	0.20	12.74
9d00131	0.06	9.87	um11771	-0.43	11.56
9d00132	0.28	11.84	um11772	-0.68	10.51
9d00133	-0.07	11.98	um11773	-0.58	8.80
9d00134	1.12	13.82	um11774	-0.09	8.68
9d00135	-0.02	13.05	um11775	1.56	12.39
9d00136	-0.38	12.96	um11776	1.63	12.63
9d00137	0.09	11.33	um11777	-1.54	10.77
9d00138	0.27	10.51	um11778	-2.80	10.14
9d00139	-0.38	12.96	um11779	0.09	12.03
9d00140	-0.26	11.81	um11780	0.19	12.87
9d00141	0.72	10.04	um11781	2.36	12.78
9d00142	0.41	13.15	um11782	0.01	13.14
9d00143	0.02	12.14	um11783	-0.63	12.51
9d00144	-0.03	12.21	um11784	0.14	10.10
9d00145	0.01	12.02	um11785	-0.24	10.57
9d00146	0.42	11.80	um11786	-0.34	12.35
9d00147	0.24	14.10	um11787	0.30	12.93
9d00148	0.05	12.19	um11789	-0.82	12.79
9d00149	0.25	10.60	um11790	0.73	12.72
9d00150	-0.71	9.89	um11791	-0.04	11.77
9d00151	0.33	13.04	um11792	0.20	10.89
9d00152	0.71	12.42	um11793	0.50	12.67
9d00153	0.01	12.67	um11794	0.01	12.67
9d00154	0.57	12.67	um11795	1.18	13.21
9d00155	0.28	12.97	um11797	0.63	11.74
9d00156	-0.67	15.43	um11798	0.97	12.70
9d00157	-0.34	12.12	um11799	0.48	11.07
9d00158	-0.15	12.79	um11800	-0.57	13.41
9d00159	-0.11	10.31	um11801	0.00	12.95
9d00160	0.15	11.16	um11802	-0.52	15.11
9d00161	-0.03	12.56	um11804	-0.23	12.52
9d00162	0.29	12.90	um11805	-0.08	11.14
9d00163	-0.04	10.29	um11806	-0.09	10.92
9d00164	-0.11	10.08	um11807	-0.86	11.79
9d00165	-0.25	13.12	um11808	-0.45	11.57
9d00166	0.42	10.40	um11809	-0.35	8.87
9d00167	-0.22	12.00	um11812	-0.22	8.45
9d00168	0.04	10.31	um11813	0.32	12.20
9d00169	0.01	12.75	um11815	-2.00	10.47
9d00170	0.13	9.37	um11816	-0.35	10.78
9d00171	-0.85	10.91	um11817	1.20	11.87
9d00172	0.15	9.17	um11818	-0.24	12.69

9d00173	-0.32	13.14	um11819	-0.23	11.15
9d00174	-1.38	10.15	um11821	0.68	14.54
9d00175	0.05	10.65	um11822	0.22	12.45
9d00176	-0.41	12.97	um11823	0.76	10.75
9d00177	-0.04	12.26	um11824	0.68	11.01
9d00178	0.23	13.04	um11825	-0.24	12.12
9d00179	-0.38	9.73	um11827	0.38	14.97
9d00180	0.40	12.76	um11828	0.42	11.07
9d00181	0.17	11.86	um11829	0.47	12.24
9d00182	0.16	15.32	um11830	-0.19	12.69
9d00183	-0.02	12.31	um11831	-0.03	13.78
9d00184	0.05	11.51	um11833	0.15	10.86
9d00185	0.36	13.54	um11835	0.58	10.15
9d00186	-0.13	13.03	um11837	0.00	11.61
9d00187	0.02	11.31	um11839	-0.12	8.76
9d00188	-0.18	11.68	um11840	0.06	11.42
9d00189	-0.18	11.27	um11841	-0.20	10.47
9d00190	0.45	13.15	um11842	0.27	10.65
9d00191	-0.09	12.68	um11843	-0.19	8.69
9d00192	0.05	12.71	um11846	0.51	11.67
9d00193	-0.06	14.67	um11847	-0.32	8.85
9d00194	0.16	11.79	um11848	-0.97	14.88
9d00195	-0.51	11.83	um11849	-0.85	14.86
9d00196	0.16	11.18	um11851	0.44	13.35
9d00197	-0.89	12.51	um11852	-0.22	12.43
9d00198	-0.75	12.84	um11853	0.01	12.19
9d00199	0.04	11.95	um11855	-0.23	11.67
9d00200	-0.20	11.73	um11858	0.52	11.04
9d00201	0.12	13.37	um11859	-1.02	13.45
9d00202	-0.73	14.17	um11860	0.33	11.92
9d00203	0.28	12.30	um11862	0.47	12.10
9d00204	-0.34	10.66	um11863	-0.83	14.23
9d00205	-0.40	11.09	um11864	-0.01	12.24
9d00206	0.00	13.21	um11865	-0.06	11.96
9d00207	0.00	12.08	um11866	0.41	10.83
9d00208	0.18	9.27	um11867	0.04	11.15
9d00209	0.40	10.75	um11870	1.77	11.12
9d00210	-0.27	13.25	um11871	0.06	12.23
9d00211	0.09	12.32	um11873	-0.57	10.53
9d00212	-0.41	11.32	um11874	-0.38	10.43
9d00213	-0.03	8.70	um11875	-0.46	12.96
9d00214	-0.26	11.32	um11876	0.16	10.74
9d00215	0.14	8.87	um11878	0.89	12.25
9d00216	-0.31	12.44	um11880	0.28	14.43
9d00217	-0.20	12.29	um11881	-0.46	11.49
9d00218	-0.30	11.83	um11882	0.31	12.94
9d00219	0.20	11.08	um11883	-0.30	9.05
9d00220	-0.28	12.81	um11884	0.07	11.54
9d00221	0.60	12.32	um11885	0.45	11.88
9d00222	0.38	11.14	um11886	-0.67	9.43
9d00223	0.02	11.31	um11889	-0.07	12.93
9d00224	0.07	13.92	um11890	0.38	10.76
9d00225	-0.49	13.14	um11891	0.46	13.09
9d00226	0.19	11.92	um11892	-0.67	12.68

9d00227	-1.92	12.84
9d00228	0.14	12.31
9d00229	-0.21	10.16
9d00230	-0.34	13.15
9d00231	-0.51	12.73
9d00232	0.32	11.52
9d00233	0.15	14.44
9d00234	0.26	11.90
9d00235	0.08	12.55
9d00236	0.08	9.49
9d00237	0.21	12.85
9d00238	0.47	12.51
9d00239	-0.61	13.96
9d00240	1.34	13.77
9d00241	1.15	14.19
9d00242	-0.13	9.88
9d00243	-0.41	11.52
9d00244	-0.21	11.32
9d00245	0.16	9.22
9d00246	0.25	13.28
9d00247	0.74	11.53
9d00248	0.57	10.89
9d00249	-0.45	12.52
9d00250	-0.32	11.76
9d00251	0.68	13.56
9d00252	0.15	11.74
9d00253	0.34	9.69
9d00254	0.34	12.62
9d00255	-0.19	11.10
9d00256	0.25	12.91
9d00257	-0.06	12.98
9d00258	-0.89	10.87
9d00259	-0.65	14.00
9d00260	-0.02	11.45
9d00261	-0.25	13.27
9d00262	-0.12	9.18
9d00263	0.04	12.23
9d00264	0.12	11.90
9d00265	-0.12	12.10
9d00266	0.24	13.74
9d00267	-0.02	12.75
9d00268	0.13	12.75
9d00269	0.08	8.74
9d00270	0.48	12.95
9d00271	-0.13	13.04
9d00272	-0.19	12.42
9d00273	-0.03	13.31
9d00274	-0.02	13.83
9d00275	-0.03	12.76
9d00276	0.00	13.93
9d00277	-0.50	13.41
9d00278	0.12	9.98
9d00279	-0.30	12.27
9d00280	0.50	12.26

um11893	0.23	10.56
um11894	0.26	12.34
um11895	2.51	12.63
um11896	1.00	13.50
um11899	0.57	11.36
um11900	0.10	9.47
um11901	-0.21	12.61
um11905	0.41	10.94
um11906	-0.56	13.26
um11907	-0.46	11.23
um11908	0.37	13.03
um11909	1.03	12.59
um11910	0.20	8.97
um11911	-0.23	15.72
um11912	0.11	11.44
um11913	0.28	10.53
um11914	0.36	14.67
um11915	0.19	13.09
um11916	0.06	11.11
um11918	0.00	13.09
um11919	0.71	12.90
um11921	1.41	14.75
um11922	-0.34	10.09
um11923	-0.53	12.20
um11924	-0.03	11.55
um11925	-0.11	13.50
um11926	-0.27	11.42
um11927	0.64	12.84
um11928	-0.08	14.13
um11929	-0.05	10.12
um11931	-2.89	11.24
um11932	0.82	12.03
um11934	0.20	11.67
um11935	-1.36	9.72
um11937	-0.07	13.17
um11938	0.00	15.24
um11939	0.51	13.59
um11940	-1.39	10.97
um11941	0.72	11.35
um11943	0.10	11.38
um11944	0.43	11.33
um11945	-0.30	13.63
um11946	-0.60	13.69
um11947	-1.07	12.95
um11948	-0.48	13.57
um11949	-0.44	13.15
um11950	-0.17	10.34
um11951	0.92	13.10
um11952	-0.25	14.73
um11953	-0.72	12.40
um11954	0.94	10.62
um11955	0.42	11.40
um11956	0.58	9.10
um11957	0.74	11.24

9d00281	-0.76	9.33	um11959	-0.11	11.47
9d00282	-0.28	11.22	um11960	-0.18	13.00
9d00283	0.53	12.17	um11961	-0.74	13.79
9d00284	0.20	10.66	um11962	-0.08	11.69
9d00285	0.35	12.36	um11963	-0.09	11.34
9d00286	-0.17	12.82	um11964	0.30	11.10
9d00287	0.27	12.72	um11966	0.83	13.05
9d00288	-0.06	12.79	um11967	1.20	12.69
9d00289	-0.06	13.20	um11968	0.26	12.18
9d00290	-0.03	12.88	um11969	0.36	12.06
9d00291	0.28	11.78	um11970	-0.72	9.87
9d00292	0.31	12.30	um11973	0.16	12.13
9d00293	-0.96	11.94	um11974	-0.55	10.65
9d00294	0.13	11.68	um11976	-0.16	8.70
9d00295	-0.21	11.07	um11977	0.45	11.68
9d00296	0.38	13.53	um11978	0.18	14.33
9d00297	-0.25	12.09	um11979	-0.28	13.42
9d00298	0.20	13.05	um11980	0.86	9.75
9d00299	-0.10	12.38	um11981	0.49	14.69
9d00300	-0.22	12.09	um11982	0.33	11.26
9d00301	0.02	11.11	um11983	-0.23	12.37
9d00302	0.09	12.21	um11984	1.44	13.22
9d00303	0.45	13.62	um11985	-0.05	15.28
9d00304	-0.25	12.01	um11986	-0.48	10.35
9d00305	0.13	10.95	um11987	0.27	11.52
9d00306	0.17	13.67	um11988	0.53	12.16
9d00307	0.13	12.12	um11989	0.29	10.93
9d00308	-0.13	12.62	um11990	-0.01	10.80
9d00309	-0.13	11.92	um11991	0.00	12.99
9d00310	0.17	9.79	um11997	0.11	12.05
9d00311	0.15	9.46	um11998	-0.42	12.01
9d00312	-0.22	13.53	um11999	-0.17	12.46
9d00313	-0.03	14.11	um12001	-0.28	13.45
9d00314	0.22	13.12	um12002	0.45	12.45
9d00315	-0.35	11.18	um12004	-0.14	11.95
9d00316	-0.50	11.54	um12005	-0.49	12.69
9d00317	-0.48	12.71	um12006	0.64	11.43
9d00318	-0.48	10.33	um12007	0.93	11.51
9d00319	-0.08	10.63	um12008	-0.10	12.12
9d00320	-0.70	11.72	um12009	0.64	12.56
9d00321	-0.16	12.58	um12010	0.30	12.26
9d00322	0.11	11.85	um12011	1.06	12.55
9d00323	0.71	13.38	um12012	0.25	11.73
9d00324	-0.34	11.39	um12013	-0.16	11.39
9d00325	-0.26	10.18	um12014	0.45	11.92
9d00326	0.40	14.36	um12015	-4.12	13.58
9d00327	-0.24	11.93	um12016	-0.82	9.62
9d00328	0.92	11.77	um12017	-0.03	13.29
9d00329	-0.11	13.19	um12018	0.08	11.78
9d00330	-0.16	8.70	um12019	-0.14	11.28
9d00331	0.23	12.29	um12021	0.59	13.32
9d00332	0.49	14.04	um12022	0.03	12.73
9d00333	0.11	9.34	um12023	-0.03	11.77
9d00334	0.36	14.77	um12024	-1.28	11.22

9d00335	0.61	13.43	um12025	0.13	11.33
9d00336	0.26	12.11	um12026	1.35	11.24
9d00337	-0.33	11.15	um12027	1.45	11.97
9d00338	0.01	12.12	um12030	0.68	13.04
9d00339	0.13	11.81	um12031	-2.12	13.41
9d00340	-0.07	13.50	um12032	-1.93	11.07
9d00341	0.15	13.05	um12033	-0.30	12.42
9d00342	-0.38	11.19	um12034	-0.48	12.52
9d00343	-1.80	11.64	um12035	0.42	11.56
9d00344	0.20	12.48	um12036	-0.05	13.94
9d00345	0.15	13.17	um12038	-0.58	13.29
9d00346	0.16	12.04	um12039	-0.11	13.51
9d00347	-0.45	12.01	um12041	-0.26	12.35
9d00348	0.13	11.40	um12042	0.06	11.92
9d00349	-0.26	12.08	um12043	0.66	9.50
9d00350	-0.16	9.81	um12044	-0.15	13.49
9d00351	-0.70	13.92	um12045	-0.40	12.09
9d00352	0.24	11.93	um12046	0.34	13.16
9d00353	0.27	13.63	um12047	0.63	12.88
9d00354	-0.12	14.95	um12048	0.98	12.43
9d00355	0.21	9.43	um12049	0.85	13.05
9d00356	0.17	13.43	um12050	-0.63	14.52
9d00357	-0.70	12.84	um12051	-0.62	8.99
9d00358	0.20	11.90	um12052	-0.28	8.63
9d00359	0.02	8.98	um12055	-0.09	13.30
9d00360	0.10	10.09	um12058	-0.43	13.53
9d00361	0.01	13.70	um12061	0.41	10.27
9d00362	-2.12	12.27	um12062	-0.03	13.24
9d00363	-0.48	11.41	um12066	0.48	10.74
9d00364	0.44	12.13	um12068	0.48	12.12
9d00365	-0.98	14.87	um12074	0.51	11.69
9d00366	0.53	13.32	um12076	-0.04	11.98
9d00367	0.03	8.60	um12080	-1.66	11.28
9d00368	-0.21	11.49	um12081	0.20	11.57
9d00369	-0.63	12.12	um12082	-0.32	11.20
9d00370	0.27	12.82	um12083	-0.45	15.81
9d00371	-0.22	12.40	um12084	0.34	13.25
9d00372	-0.16	12.85	um12085	0.78	12.57
9d00373	-0.03	8.92	um12086	0.28	12.00
9d00374	-0.24	13.19	um12087	-0.09	10.56
9d00375	0.55	12.17	um12089	-0.20	12.43
9d00376	0.29	11.98	um12090	-2.04	10.22
9d00377	0.32	12.79	um12091	0.71	13.36
9d00378	0.12	9.68	um12092	0.01	8.92
9d00379	0.17	11.03	um12093	0.47	12.16
9d00380	0.20	11.76	um12094	0.16	12.87
9d00381	-0.02	15.89	um12095	-0.21	13.51
9d00382	-0.04	10.11	um12096	-0.74	10.75
9d00383	-0.02	12.81	um12097	0.63	11.84
9d00384	-0.38	12.14	um12098	1.61	13.61
9d00385	-0.17	11.59	um12099	-0.03	11.47
9d00386	0.02	11.77	um12100	0.34	9.54
9d00387	0.08	12.65	um12101	0.43	13.70
9d00388	0.56	13.30	um12104	-0.17	9.60

9d00389	0.07	13.22
9d00390	-0.30	10.98
9d00391	0.17	12.17
9d00392	0.28	10.52
9d00393	-0.11	12.99
9d00394	-0.05	12.64
9d00395	-0.09	11.96
9d00396	0.27	9.01
9d00397	0.36	11.26
9d00398	0.08	14.32
9d00399	-0.09	11.34
9d00400	0.91	15.10
9d00401	-0.01	13.41
9d00402	0.33	14.70
9d00403	0.01	8.44
9d00404	-0.02	13.54
9d00405	0.32	11.73
9d00406	-0.60	13.94
9d00407	0.24	13.15
9d00408	0.10	13.45
9d00409	-0.28	11.36
9d00410	0.15	11.67
9d00411	-0.66	12.23
9d00412	-0.01	12.25
9d00413	0.00	14.81
9d00414	-0.26	12.50
9d00415	-0.35	11.97
9d00416	-1.97	10.09
9d00417	-0.21	8.61

um12105	-0.25	12.61
um12106	-0.86	11.74
um12107	-0.39	11.76
um12108	0.40	11.83
um12110	0.37	12.03
um12111	0.99	10.86
um12112	0.17	9.31
um12113	0.54	10.95
um12114	-0.40	8.97
um12116	0.10	11.76
um12117	-0.07	11.59
um12118	-0.02	8.97
um12119	0.45	10.72
um12120	-0.31	8.88
um12121	0.06	12.78
um12122	0.58	12.46
um12123	0.34	12.24
um12124	-1.52	10.93
um12125	0.25	14.10
um12126	0.69	11.66
um12127	-1.41	10.29
um12128	0.39	12.20
um12129	0.09	13.24
um12130	0.39	12.32
um12131	-0.37	15.47
um12132	0.45	12.55
um12133	-0.45	11.58
um12134	-0.19	10.75
um12135	0.23	12.83
um12136	0.04	13.01
um12137	0.42	8.98
um12138	0.30	10.90
um12140	-0.09	11.03
um12141	-0.32	10.47
um12142	0.37	11.81
um12143	-0.65	13.03
um12144	1.55	10.03
um12146	-0.71	12.79
um12147	-0.11	11.24
um12148	-0.18	11.09
um12149	-0.13	9.52
um12150	0.21	11.85
um12151	-0.02	11.69
um12152	-0.11	12.15
um12153	0.17	11.47
um12154	0.57	10.43
um12155	0.74	9.97
um12156	-0.07	9.02
um12157	-0.28	9.06
um12158	0.80	12.02
um12159	-0.03	11.89
um12160	-0.54	13.50
um12161	1.05	10.43
um12163	0.46	11.79

um12165	0.17	11.87
um12166	0.18	12.80
um12167	0.35	12.26
um12168	0.26	11.43
um12169	1.11	13.36
um12171	0.31	11.72
um12172	0.30	13.13
um12173	1.41	12.74
um12174	0.22	11.65
um12175	3.31	13.14
um12176	0.23	11.12
um12177	-0.24	9.83
um12178	1.45	12.69
um12179	0.66	13.54
um12181	0.34	12.03
um12182	0.02	10.73
um12183	-0.76	11.74
um12184	-0.47	15.35
um12185	0.81	11.20
um12186	-0.27	11.94
um12187	0.08	8.68
um12188	0.01	12.94
um12189	0.07	11.13
um12190	0.51	13.44
um12191	0.46	11.52
um12192	-0.44	12.38
um12193	0.09	9.48
um12194	0.35	11.43
um12195	-0.09	12.19
um12196	0.52	13.18
um12197	-0.39	9.67
um12198	-1.02	13.18
um12199	0.48	11.57
um12200	0.58	12.37
um12201	0.02	10.67
um12202	-0.07	11.75
um12203	0.18	11.29
um12204	0.87	12.97
um12205	-0.25	11.65
um12206	-0.18	13.71
um12207	0.33	10.66
um12208	-0.24	11.03
um12209	-0.31	10.88
um12210	-0.42	11.63
um12211	-0.12	12.24
um12212	0.37	12.09
um12213	0.13	12.16
um12214	-0.26	8.75
um12215	-0.15	8.45
um12216	0.00	8.59
um12217	0.03	9.77
um12218	-0.55	10.98
um12219	0.41	12.46
um12221	0.38	11.27

um12222	0.62	10.92
um12223	0.53	12.01
um12224	0.59	11.47
um12225	-0.29	9.32
um12226	0.33	10.98
um12227	0.47	11.67
um12228	0.26	11.87
um12229	0.09	9.12
um12230	0.95	11.75
um12231	-0.43	11.38
um12232	0.40	11.44
um12233	0.04	8.86
um12234	0.48	11.80
um12235	0.64	10.41
um12236	-0.95	13.54
um12237	-0.05	11.34
um12238	-4.14	13.52
um12239	0.05	12.11
um12240	0.84	11.52
um12241	0.25	11.27
um12242	-0.67	12.42
um12243	0.07	11.72
um12244	-0.38	11.68
um12246	0.20	11.89
um12247	0.15	12.23
um12248	-0.22	8.41
um12249	0.83	11.39
um12250	-0.73	12.01
um12251	-0.22	13.13
um12253	0.13	9.74
um12254	0.07	12.72
um12255	0.09	11.80
um12256	-0.01	10.68
um12257	-0.19	8.91
um12258	-0.14	8.44
um12259	-0.34	11.82
um12260	0.03	12.40
um12261	1.01	11.93
um12262	-1.18	12.60
um12263	0.44	10.44
um12264	-0.16	9.65
um12265	-0.63	9.82
um12267	1.06	12.61
um12269	0.10	12.86
um12271	0.85	14.18
um12272	-0.24	11.85
um12273	0.93	11.87
um12275	0.52	9.80
um12276	-0.18	11.40
um12277	0.40	12.47
um12278	-0.70	13.06
um12279	0.00	10.87
um12280	0.71	12.13
um12281	-0.34	9.47

um12282	-0.53	13.23
um12283	1.03	12.93
um12284	0.44	12.17
um12285	0.60	11.40
um12286	0.06	10.34
um12287	-0.04	8.77
um12288	0.77	11.90
um12289	0.20	11.54
um12291	-0.20	12.65
um12292	-0.31	9.46
um12293	-0.28	11.24
um12294	-0.15	11.86
um12295	1.31	11.77
um12296	0.38	12.26
um12297	0.60	11.89
um12298	-0.50	10.59
um12299	-0.67	13.51
um12300	-2.13	12.63
um12301	-0.22	12.09
um12302	-0.22	9.12
um12303	2.20	11.27
um12304	2.79	11.65
um12305	-0.29	12.17
um12306	0.36	11.49
um12307	0.16	11.97
um12308	0.03	9.84
um12309	-0.39	11.13
um12310	1.27	10.56
um12311	0.10	11.26
um12312	-0.38	11.85
um12313	-1.21	11.18
um12314	0.10	13.18
um12315	-0.80	8.85
um12316	-0.29	11.35
um12317	0.41	11.82
um12318	-0.55	9.18
um12319	-0.34	9.78
um12320	-0.32	9.97
um12321	-0.17	12.75
um12322	-0.32	8.56
um12323	-0.17	8.67
um12324	-1.12	11.99
um12325	0.84	10.48
um12326	0.04	11.13
um12327	0.18	12.21
um12328	1.03	12.43
um12329	0.18	12.03
um12330	-1.04	10.41
um12331	0.89	11.71
um12332	-0.13	10.60
um12333	0.71	11.93
um12334	0.12	11.33
um12335	-0.10	10.91
um12336	-1.06	10.05

um12337	-0.46	10.52
um12338	-0.06	9.98
um12339	-0.56	10.28
um12340	0.16	8.88
um12341	0.00	8.83
um12342	0.09	12.96
um12346	0.39	9.37
um12350	-0.64	15.92
um15000	0.41	11.53
um15001	-0.59	12.42
um15002	-0.49	12.51
um15003	0.40	11.82
um15004	0.33	14.69
um15005	-0.11	12.26
um15006	-0.54	14.02
um15007	-0.04	11.16
um15008	-0.34	14.95
um15009	-0.26	12.69
um15011	-0.21	11.59
um15012	-0.36	11.97
um15013	-0.22	12.21
um15014	-0.99	12.46
um15015	-0.08	12.16
um15016	0.03	11.42
um15017	-0.30	11.85
um15018	0.31	13.59
um15019	-0.27	11.79
um15020	0.03	13.54
um15021	0.93	12.49
um15022	0.06	12.62
um15023	-0.10	11.83
um15024	-0.96	9.63
um15025	1.37	11.06
um15026	0.45	13.09
um15027	0.10	10.85
um15028	-0.23	13.48
um15029	-0.31	12.49
um15030	0.04	12.60
um15031	-0.08	10.51
um15032	-0.13	10.19
um15034	-0.46	14.32
um15035	-0.11	11.46
um15036	-0.20	12.56
um15037	0.32	11.76
um15038	-0.19	11.28
um15039	-0.14	11.48
um15040	-0.60	11.76
um15041	-0.03	10.28
um15042	0.19	13.54
um15043	-0.90	12.66
um15045	-1.15	11.58
um15046	0.35	10.81
um15047	-0.53	12.36
um15048	1.14	11.96

um1504[illegible]	-0.58	12.17
um1505[illegible]	0.14	13.39
um15051	1.19	12.77
um1505[illegible]	0.66	13.22
um1505[illegible]	0.21	11.99
um1505[illegible]	-0.40	10.90
um1505[illegible]	-0.25	11.38
um1505[illegible]	0.19	11.00
um1505[illegible]	-0.94	10.76
um1506[illegible]	0.68	10.75
um15061	0.33	11.98
um1506[illegible]	-0.41	11.10
um1506[illegible]	-0.91	11.82
um1506[illegible]	0.11	11.17
um1506[illegible]	0.36	11.65
um1506[illegible]	-0.29	11.11
um1506[illegible]	-0.26	11.74
um1506[illegible]	0.42	11.05
um1507[illegible]	-0.25	11.42
um1507[illegible]	-0.17	12.79
um1507[illegible]	0.43	11.38
um1507[illegible]	0.82	12.29
um1507[illegible]	0.39	10.59
um1507[illegible]	0.86	9.76
um1507[illegible]	-0.21	11.57
um1507[illegible]	0.20	11.52
um1508[illegible]	0.41	12.42
um15081	0.10	10.07
um1508[illegible]	-0.36	11.40
um1508[illegible]	-0.12	11.55
um1508[illegible]	1.26	13.37
um1508[illegible]	-0.67	10.56
um1508[illegible]	0.07	10.90
um1508[illegible]	0.30	10.79
um1508[illegible]	-0.39	11.37
um1508[illegible]	-0.12	15.25
um1509[illegible]	-0.10	11.59
um15091	-0.39	11.37
um1509[illegible]	0.27	12.12
um1509[illegible]	0.07	11.49
um1509[illegible]	-0.36	11.79
um1509[illegible]	2.18	12.99
um1509[illegible]	0.89	10.80
um1509[illegible]	0.94	14.49
um1509[illegible]	0.16	11.37
um1509[illegible]	0.37	11.84
um1510[illegible]	-1.22	13.12
um15101	0.71	11.65
um1510[illegible]	-0.01	11.56
um1510[illegible]	0.97	11.07
