# Supplementary material for: Genome and Transcriptome Analysis of the Basidiomycetous Yeast Pseudozyma antarctica Producing Extracellular Glycolipids, Mannosylerythritol Lipids
Source: PLoS One. 2014 Feb 24;9(2):e86490. doi: 10.1371/journal.pone.0086490 (PMC3933340; doi:10.1371/journal.pone.0086490)
Supplement: Table S7 — Differentially expressed genes analysis. (PDF) [file pone.0086490.s008.pdf]

**Supplementary Table S7**
Differentially expressed genes analysis

| *P. antarctica* gene | p-val | kegg | annotation | *U. maydis* gene | p-val | kegg | annotation |
|---|---|---|---|---|---|---|---|
| 5d00026 | 9.32E-33 | K01187 | maltase glucoamylase and related hydrolases | um10632 | 1.56E-17 | ni | related to ph-regulated antigen pra1 precursor |
| 22d00064 | 8.13E-32 | ni | permease of the major facilitator superfamily | um01052 | 7.89E-16 | ni | putative protein |
| 7d00295 | 4.27E-29 | K00507 | fatty acid desaturase | um03110 | 9.17E-16 | K14709 | related to ZRT2 - Zinc transporter II |
| 13c00020 | 3.26E-25 | ni | synaptic vesicle transporter SVOP and related transporters | um00309 | 2.43E-15 | ni | conserved hypothetical protein |
| 8d00044 | 1.77E-24 | K07119 | predicted NAD-dependent oxidoreductase | um02984 | 2.78E-15 | ni | related to Dibenzothiophene desulfurization enzyme C |
| 9c00247 | 1.49E-23 | ni | hypothetical protein | um00876 | 4.54E-15 | ni | related to SPR1 - exo-1 |
| 8d00029 | 8.06E-23 | ni | hypothetical protein | um06042 | 6.62E-15 | ni | conserved hypothetical protein |
| 22c00024 | 1.30E-22 | ni | predicted transporter | um01187 | 9.08E-15 | ni | conserved hypothetical protein |
| 6d00004 | 8.74E-22 | K01187 | alpha-amylase | um02215 | 9.42E-15 | ni | related to dibenzothiophene desulfurizing enzyme |
| 7d00178 | 2.16E-21 | ni | hypothetical protein | um02626 | 1.10E-14 | ni | conserved hypothetical protein |
| 22c00208 | 4.27E-21 | ni | hypothetical protein | um03381 | 1.77E-14 | ni | conserved hypothetical protein |
| 22d00094 | 6.08E-21 | ni | hypothetical protein | um03475 | 2.49E-14 | K14640 | probable PHO89 - Na |
| 7c00360 | 1.02E-20 | ni | predicted L-carnitine dehydratase | um12015 | 3.92E-14 | ni | conserved hypothetical Ustilago-specific protein |
| 9c00397 | 4.12E-20 | ni | hypothetical protein | um00529 | 4.78E-14 | ni | probable carboxylic acid transport protein JEN1 |
| 24d00054 | 5.99E-20 | ni | hypothetical protein | um00027 | 5.21E-14 | ni | putative protein |
| 13d00099 | 9.56E-20 | K01178 | hypothetical protein | um12238 | 7.61E-14 | ni | conserved hypothetical Ustilago-specific protein |
| 4d00039 | 1.99E-19 | ni | hypothetical protein | um03558 | 8.46E-14 | ni | conserved hypothetical protein |
| 22c00300 | 5.32E-18 | K13953 | alcohol dehydrogenase | um02625 | 1.18E-13 | ni | probable DUR3 - Urea permease |
| 9c00005 | 6.92E-18 | ni | NADH, flavin oxidoreductase | um04309 | 1.40E-13 | ni | probable Alpha-L-arabinofuranosidase precursor |
| 7c00359 | 8.04E-18 | ni | 3-Methylcrotonyl-CoA carboxylase, non-biotin containing subunit | um05253 | 1.63E-13 | ni | related to ATP-binding multidrug cassette transport protein |
| 19c00005 | 9.19E-16 | K14709 | Fe2+/Zn2+ regulated transporter | um00842 | 2.39E-13 | ni | probable aflatoxin efflux pump AFLT |
| 14d00115 | 1.02E-15 | ni | hypothetical protein | um05226 | 2.43E-13 | ni | conserved hypothetical protein |
| 27d00022 | 1.39E-15 | ni | hypothetical protein | um11465 | 2.51E-13 | ni | putative protein |
| 9d00227 | 3.31E-15 | ni | hypothetical protein | um05084 | 4.99E-13 | ni | related to lignostilbene alpha |
| 7c00358 | 3.69E-15 | ni | transcription factor | um03585 | 7.29E-13 | ni | conserved hypothetical protein |
| 9c00256 | 3.75E-15 | ni | hypothetical protein | um03560 | 7.83E-13 | ni | related to beta-1 |
| 16d00042 | 1.31E-14 | ni | hypothetical protein | um03169 | 7.87E-13 | K00819 | probable CAR2 - ornithine aminotransferase |
| 18d00112 | 1.54E-14 | ni | hypothetical protein | um05791 | 7.88E-13 | ni | related to Cytochrome P450 |
| 19c00045 | 1.61E-14 | ni | predicted transporter | um05276 | 1.01E-12 | ni | conserved hypothetical protein |
| 4c00035 | 2.81E-14 | ni | hypothetical protein | um04358 | 1.28E-12 | ni | conserved hypothetical protein |
| 9d00343 | 3.74E-14 | ni | hypothetical protein | um10581 | 1.46E-12 | ni | related to Alcohol dehydrogenase class III chi chain |
| 25c00020 | 1.07E-13 | ni | hypothetical protein | um05252 | 1.46E-12 | ni | probable nadp-dependent mannitol dehydrogenase |
| 15c00002 | 1.07E-13 | K00326 | NADH-cytochrome b-5 reductase | um05038 | 1.61E-12 | ni | K |
| 15d00001 | 1.31E-13 | ni | cytochrome P450 CYP4/CYP19/CYP26 subfamilies | um04353 | 1.70E-12 | ni | related to glycosyl transferase |
| 20c00079 | 1.43E-13 | ni | non-ribosomal peptide synthetase | um05222 | 1.83E-12 | ni | putative protein |
| 9d00064 | 1.83E-13 | K00012 | UDP-glucose/GDP-mannose dehydrogenase | um03665 | 2.11E-12 | K00128 | probable aldehyde dehydrogenase family 7 member A1 |
| 3d00086 | 2.77E-13 | ni | hypothetical protein | um05812 | 2.33E-12 | ni | related to Cyclohexanone monooxygenase |
| 9d00362 | 3.12E-13 | ni | hypothetical protein | um03135 | 2.37E-12 | ni | conserved hypothetical protein |
| 22d00247 | 3.17E-13 | ni | hypothetical protein | um05421 | 2.58E-12 | ni | related to Multidrug resistance protein |
| 7d00094 | 4.98E-13 | ni | FOG,  Zn-finger | um05596 | 2.88E-12 | ni | conserved hypothetical protein |
| 27d00089 | 5.80E-13 | ni | hypothetical protein | um11403 | 3.07E-12 | ni | conserved hypothetical protein |
| 4c00041 | 5.90E-13 | ni | hypothetical protein | um03402 | 3.07E-12 | K00128 | Indole-3-acetaldehyde dehydrogenase |
| 2c00011 | 6.61E-13 | ni | hypothetical protein | um11554 | 3.34E-12 | ni | related to Glucose oxidase |
| 1d00040 | 1.27E-12 | ni | hypothetical protein | um00025 | 4.79E-12 | ni | related to dehydroshikimate dehydratase |
| 7c00093 | 2.51E-12 | ni | hypothetical protein | um12175 | 4.92E-12 | ni | conserved hypothetical protein |
| 5d00035 | 2.72E-12 | ni | synaptic vesicle transporter SVOP and related transporters | um04971 | 6.64E-12 | ni | probable OSM1 - fumarate reductase |
| 7c00263 | 4.05E-12 | ni | hypothetical protein | um11931 | 6.87E-12 | ni | conserved hypothetical protein |
| 3d00076 | 5.08E-12 | ni | predicted transporter | um01723 | 6.89E-12 | ni | related to Cytochrome P450 |
| 16d00076 | 5.73E-12 | ni | hypothetical protein | um00374 | 8.45E-12 | ni | probable DAL5 - Allantoate and ureidosuccinate permease |
| 7c00332 | 6.11E-12 | ni | hypothetical protein | um00815 | 8.86E-12 | ni | related to multidrug resistant protein |
| 8d00109 | 9.89E-12 | K01175 | hypothetical protein | um01165 | 9.44E-12 | ni | related to Glucan 1 |
| 8c00087 | 1.20E-11 | ni | hypothetical protein | um04557 | 9.84E-12 | ni | conserved hypothetical protein |
| 11c00030 | 1.27E-11 | ni | hypothetical protein | um06085 | 1.02E-11 | ni | conserved hypothetical protein |
| 13c00047 | 1.38E-11 | ni | fatty acyl-CoA elongase | um04357 | 1.02E-11 | ni | related to endo-1 |
| 22c00088 | 2.05E-11 | ni | hypothetical protein | um05076 | 1.11E-11 | ni | related to 3-carboxy-cis |
| 2d00039 | 2.13E-11 | ni | non-ribosomal peptide synthetase | um00465 | 1.13E-11 | ni | hypothetical protein |
| 25d00028 | 2.79E-11 | ni | hypothetical protein | um04379 | 1.26E-11 | ni | related to gibberellin 20-oxidase |
| 4d00015 | 3.45E-11 | ni | transcription factor | um05247 | 1.34E-11 | ni | related to methylcrotonyl-CoA carboxylase beta chain |
| 3d00005 | 3.83E-11 | ni | hypothetical protein | um10608 | 1.36E-11 | ni | related to Quinate permease |
| 22c00260 | 4.09E-11 | ni | predicted NAD-dependent oxidoreductase | um11179 | 1.39E-11 | ni | conserved hypothetical protein |
| 13d00096 | 4.46E-11 | ni | mitochondrial oxoglutarate/malate carrier proteins | um05079 | 1.56E-11 | ni | probable CTP1 - Mitochondrial citrate transporter - member of the mitochondrial carrier |
| 2d00034 | 4.55E-11 | ni | hypothetical protein | um11112 | 1.71E-11 | ni | related to Versicolorin B synthase |
| 20d00084 | 4.88E-11 | ni | hypothetical protein | um02811 | 1.74E-11 | ni | related to 2 |
| 7d00257 | 4.98E-11 | ni | hypothetical protein | um02585 | 1.79E-11 | ni | related to TPO1 - Vacuolar polyamine-H  antiporter |
| 11d00033 | 5.16E-11 | ni | hypothetical protein | um05586 | 1.96E-11 | K03381 | related to hydroxyquinol-1 |
| 26d00048 | 5.55E-11 | ni | hypothetical protein | um05074 | 2.11E-11 | ni | probable Cytochrome P450 monooxygenase |
| 14c00044 | 7.09E-11 | ni | hypothetical protein | um00824 | 2.41E-11 | ni | putative protein |
| 22c00308 | 7.29E-11 | ni | hypothetical protein | um04575 | 2.52E-11 | ni | conserved hypothetical protein |
| 11c00028 | 7.43E-11 | ni | hypothetical protein | um06359 | 2.60E-11 | ni | hypothetical protein |
| 20c00081 | 7.84E-11 | ni | hypothetical protein | um02092 | 2.86E-11 | ni | hypothetical protein |
| 12c00005 | 9.21E-11 | ni | hypothetical protein | um05493 | 2.96E-11 | ni | hypothetical protein |
| 6c00079 | 9.61E-11 | ni | hypothetical protein | um10419 | 3.05E-11 | K01183 | chitinase |
| 9c00382 | 2.07E-10 | ni | zinc-binding oxidoreductase | um04577 | 3.24E-11 | ni | probable DUR3 - Urea permease |
| 1c00041 | 2.08E-10 | ni | hypothetical protein | um01898 | 3.29E-11 | ni | related to endo-1 |
| 6c00094 | 2.18E-10 | ni | hypothetical protein | um06453 | 3.39E-11 | K00616 | related to Transaldolase B |
| 4c00028 | 2.49E-10 | ni | molecular chaperone | um04910 | 3.56E-11 | ni | conserved hypothetical protein |
| 18d00021 | 2.91E-10 | ni | lipid phosphate phosphatase and related enzymes of the PAP2 fa | um06084 | 3.72E-11 | ni | conserved hypothetical protein |
| 9d00174 | 3.00E-10 | ni | hypothetical protein | um04335 | 3.92E-11 | ni | conserved hypothetical protein |
| 19d00009 | 3.12E-10 | K01808 | hypothetical protein | um02169 | 4.26E-11 | ni | related to aquaporin 3 |
| 9c00388 | 3.69E-10 | ni | multidrug resistance-associated protein | um01971 | 4.50E-11 | ni | conserved hypothetical protein |
| 16c00029 | 4.05E-10 | ni | hypothetical protein | um05528 | 4.73E-11 | ni | conserved hypothetical protein |
| 6d00027 | 4.22E-10 | ni | hypothetical protein | um03485 | 4.81E-11 | ni | conserved hypothetical protein |
| 26d00075 | 5.38E-10 | ni | hypothetical protein | um03177 | 5.12E-11 | K03386 | related to peroxisomal membrane protein 20 |
| 9d00240 | 5.41E-10 | ni | hypothetical protein | um11229 | 5.45E-11 | ni | conserved hypothetical protein |
| 24d00052 | 7.74E-10 | ni | predicted methyltransferase | um01614 | 5.50E-11 | ni | conserved hypothetical protein |
| 26c00094 | 7.77E-10 | ni | hypothetical protein | um06487 | 5.61E-11 | ni | hypothetical protein |
| 6d00052 | 8.25E-10 | ni | hypothetical protein | um00037 | 6.12E-11 | ni | conserved hypothetical protein |
| 9d00088 | 9.36E-10 | ni | N-acetylglucosaminyltransferase complex, subunit PIG-A/SPT14 | um05170 | 6.32E-11 | K00122 | probable formate dehydrogenase |
| 15c00035 | 1.04E-09 | ni | hypothetical protein | um11895 | 7.21E-11 | ni | putative protein |
| 22d00271 | 1.06E-09 | ni | hypothetical protein | um02923 | 8.23E-11 | K01112 | related to HOR2 - DL-glycerol phosphatase |
| 6c00042 | 1.13E-09 | K00232 | iron/ascorbate family oxidoreductases | um03040 | 9.74E-11 | ni | hypothetical protein |
| 7d00078 | 1.17E-09 | K03146 | hypothetical Protein | um05581 | 1.00E-10 | K17069 | probable O-acetylhomoserine |
| 12c00094 | 1.18E-09 | ni | hypothetical protein | um11732 | 1.01E-10 | ni | hypothetical protein |
| 6c00016 | 1.25E-09 | ni | hypothetical protein | um11528 | 1.01E-10 | ni | conserved hypothetical protein |
| 2d00071 | 1.35E-09 | K00480 | kynurenine 3-monooxygenase and related flavoprotein monooxyge | um10208 | 1.02E-10 | ni | conserved hypothetical protein |
| 7d00183 | 1.44E-09 | K00234 | succinate dehydrogenase, flavoprotein subunit | um11118 | 1.04E-10 | ni | ni |
| 11c00027 | 1.52E-09 | ni | acyl-coa synthetase | um00968 | 1.06E-10 | ni | hypothetical protein |
| 9d00028 | 1.67E-09 | ni | hypothetical protein | um01804 | 1.10E-10 | K14265 | tryptophan amino transferase |
| 9c00055 | 2.43E-09 | K00100 | NAD dependent epimerase | um05689 | 1.11E-10 | K00540 | conserved hypothetical protein |
| 9c00085 | 3.10E-09 | ni | hypothetical protein | um10444 | 1.22E-10 | K01501 | putative nitrilase |
| 9c00044 | 3.63E-09 | ni | aspartyl protease | um01351 | 1.23E-10 | ni | putative protein |
| 25d00047 | 3.81E-09 | K05767 | ras GTPase-activating protein family - IQGAP | um11781 | 1.25E-10 | ni | ni |
| 27d00046 | 5.30E-09 | ni | hypothetical protein | um04354 | 1.37E-10 | ni | conserved hypothetical protein |
| 14d00052 | 5.65E-09 | ni | hypothetical protein | um03556 | 1.38E-10 | ni | conserved hypothetical protein |
| 22d00259 | 6.27E-09 | ni | hypothetical protein | um11381 | 1.48E-10 | ni | conserved hypothetical protein |
| 10c00037 | 6.46E-09 | ni | hypothetical protein | um03158 | 1.57E-10 | ni | related to enoyl-CoA hydratase |
| 12c00012 | 6.46E-09 | ni | hypothetical protein | um02720.2 | 1.58E-10 | ni | conserved hypothetical protein |
| 25d00072 | 7.92E-09 | ni | hypothetical protein | um03310 | 1.63E-10 | ni | conserved hypothetical protein |
| 9d00034 | 8.39E-09 | ni | hypothetical protein | um11651 | 1.73E-10 | K13621 | related to Betaine lipid synthase |
| 13c00095 | 9.15E-09 | ni | glucose dehydrogenase | um01947 | 1.77E-10 | ni | related to cytochrome-c peroxidase precursor |
| 13d00087 | 1.06E-08 | ni | predicted seven transmembrane receptor - rhodopsin family | um11129 | 1.82E-10 | ni | conserved hypothetical protein |
| 16d00078 | 1.17E-08 | K03381 | hypothetical protein | um06400 | 1.86E-10 | K00249 | conserved hypothetical protein |
| 7c00181 | 1.35E-08 | ni | C-3 sterol dehydrogenase | um02080 | 2.00E-10 | ni | conserved hypothetical protein |
| 7d00276 | 1.47E-08 | ni | hypothetical protein | um02701.2 | 2.07E-10 | ni | hypothetical protein |
| 9d00084 | 1.47E-08 | ni | long chain fatty acid elongase | um00115 | 2.16E-10 | K00826 | probable BAT2 - branched-chain-amino-acid transaminase |
| 12c00108 | 1.68E-08 | ni | hypothetical protein | um05495 | 2.25E-10 | ni | conserved hypothetical protein |
| 8c00040 | 1.72E-08 | K00108 | glucose dehydrogenase | um04737 | 2.27E-10 | ni | hypothetical protein |
| 7d00288 | 2.00E-08 | ni | beta, beta-carotene 15,15'-dioxygenase and related enzymes | um04629 | 2.33E-10 | K11262 | acetyl-CoA carboxylase |
| 7d00277 | 3.70E-08 | ni | hypothetical protein | um01588 | 2.36E-10 | ni | ni |
| 22c00320 | 3.75E-08 | K01452 | hypothetical protein | um00209.2 | 2.38E-10 | ni | putative protein |
| 18d00011 | 3.76E-08 | K01637 | isocitrate lyase | um01949 | 2.40E-10 | ni | putative protein |
| 27d00011 | 3.94E-08 | ni | putative alpha 1,2 mannosyltransferase | um03117.2 | 2.53E-10 | ni | conserved hypothetical protein |
| 26d00045 | 3.99E-08 | ni | hypothetical protein | um00841 | 2.56E-10 | ni | conserved hypothetical protein |
| 19c00146 | 4.01E-08 | ni | hypothetical protein | um00096 | 2.64E-10 | K14709 | probable ZRT2 - Zinc transporter II |
| 1d00003 | 4.22E-08 | ni | hypothetical protein | um12304 | 2.77E-10 | ni | hypothetical protein |
| 9d00115 | 4.69E-08 | ni | hypothetical protein | um01406 | 2.85E-10 | ni | conserved hypothetical protein |
| 2c00003 | 5.15E-08 | K05658 | multidrug/pheromone exporter | um01582 | 3.04E-10 | ni | ni |
| 15d00026 | 5.64E-08 | ni | G-protein alpha subunit | um05617 | 3.09E-10 | ni | related to Lipase |
| 25d00080 | 6.04E-08 | ni | cytochrome P450 CYP4/CYP19/CYP26 subfamilies | um11117 | 3.23E-10 | ni | conserved hypothetical Ustilago-specific protein |
| 8c00003 | 6.17E-08 | ni | hypothetical protein | um03664 | 3.28E-10 | ni | conserved hypothetical protein |
| 9d00039 | 6.35E-08 | ni | hypothetical protein | um03995 | 3.41E-10 | ni | related to JLP1 - Fe |
| 25c00081 | 6.43E-08 | K15272 | predicted UDP-galactose transporter | um10590.2 | 3.46E-10 | ni | conserved hypothetical protein |
| 6d00038 | 6.51E-08 | ni | hypothetical protein | um01362 | 3.58E-10 | ni | hypothetical protein |
| 6d00021 | 8.16E-08 | K14708 | sulfate/bicarbonate/oxalate exchanger SAT-1 and related transpo | um06437 | 3.64E-10 | ni | ni |
| 9d00134 | 8.80E-08 | ni | hypothetical protein | um03523 | 3.68E-10 | K00128 | probable aldehyde dehydrogenase |
| 5d00122 | 1.01E-07 | ni | multidrug resistance-associated protein | um06146 | 3.80E-10 | ni | putative protein |
| 10d00042 | 1.18E-07 | K08832 | serine/threonine protein kinase | um05422 | 4.03E-10 | ni | conserved hypothetical protein |
| 25c00082 | 1.21E-07 | ni | hypothetical protein | um03776 | 4.05E-10 | ni | ni |
| 18d00022 | 1.28E-07 | K01183 | chitinase | um01785 | 4.09E-10 | ni | putative protein |
| 22c00238 | 1.36E-07 | K00965 | galactose-1-phosphate uridylyltransferase | um06332 | 4.33E-10 | ni | endoglucanase 1 precursor |
| 9c00116 | 1.43E-07 | K07034 | hypothetical protein | um03688 | 4.44E-10 | ni | probable fatty alcohol oxidase |
| 11c00024 | 1.44E-07 | K06685 | cell cycle-associated protein Mob1-1 | um00496 | 4.56E-10 | ni | mismatch base pair and cruciform DNA recognition protein Hmp1 |
| 13c00083 | 1.65E-07 | ni | uncharacterized conserved protein | um10679 | 4.75E-10 | ni | ni |
| 18d00090 | 1.80E-07 | K03235 | protein containing adaptin N-terminal region | um11203 | 4.84E-10 | ni | conserved hypothetical protein |
| 10d00036 | 1.95E-07 | ni | hypothetical protein | um11778 | 5.14E-10 | K09788 | conserved hypothetical protein |
| 7d00143 | 2.07E-07 | ni | predicted mitochondrial carrier protein | um00844 | 5.80E-10 | K00235 | succinate dehydrogenase |
| 14d00059 | 2.10E-07 | K01638 | malate synthase | um11063.2 | 6.08E-10 | ni | conserved hypothetical protein |
| 15c00001 | 2.15E-07 | ni | 3-hydroxyacyl-CoA dehydrogenase | um10056 | 6.77E-10 | K00924 | related to serine |
| 9c00031 | 2.24E-07 | ni | NADH, flavin oxidoreductase | um12300 | 6.99E-10 | ni | related to methylcrotonyl-coa carboxylase alpha chain |

| | | | | | | | |
|---|---|---|---|---|---|---|---|
| 24c00068 | 2.29E-07 | ni | acyl-coa oxidase | um11708 | 7.24E-10 | K07560 | related to DTD1 - D-Tyr-tRNA |
| 9c00185 | 2.41E-07 | ni | hypothetical protein | um00336 | 7.93E-10 | ni | putative protein |
| 27d00093 | 2.60E-07 | ni | hypothetical protein | um10791 | 8.52E-10 | ni | putative protein |
| 12d00016 | 2.66E-07 | ni | O-methyltransferase | um10789 | 9.56E-10 | ni | putative protein |
| 5c00073 | 2.83E-07 | ni | predicted oxidoreductase | um06133 | 9.69E-10 | ni | conserved hypothetical protein |
| 6d00070 | 3.21E-07 | ni | hypothetical protein | um04807 | 1.01E-09 | ni | conserved hypothetical protein |
| 22d00016 | 3.35E-07 | ni | hypothetical protein | um00182 | 1.02E-09 | ni | related to Lipase 2 precursor |
| 7c00235 | 3.40E-07 | ni | hypothetical protein | um06470 | 1.02E-09 | ni | conserved hypothetical protein |
| 20d00038 | 3.59E-07 | ni | hypothetical protein | um10339 | 1.03E-09 | K00668 | related to fatty acid synthase |
| 12d00003 | 3.59E-07 | ni | hypothetical protein | um04528 | 1.06E-09 | ni | conserved hypothetical protein |
| 9d00365 | 4.02E-07 | ni | hypothetical protein | um01805 | 1.16E-09 | ni | conserved hypothetical protein |
| 27c00075 | 4.14E-07 | ni | uncharacterized conserved protein | um11514 | 1.19E-09 | ni | probable High-affinity glucose transporter |
| 22d00236 | 4.14E-07 | ni | enoyl-coa isomerase | um11368 | 1.27E-09 | ni | probable cytochrome b5 |
| 25c00034 | 4.18E-07 | ni | hypothetical protein | um02175 | 1.27E-09 | ni | related to 3-phytase A precursor |
| 7c00292 | 4.69E-07 | ni | hypothetical protein | um05967 | 1.28E-09 | K00480 | related to salicylate 1-monooxygenase |
| 20c00080 | 4.89E-07 | ni | multidrug resistance-associated protein | um00205 | 1.28E-09 | ni | related to HSP12 - heat shock protein |
| 9d00293 | 5.39E-07 | ni | hypothetical protein | um03614 | 1.32E-09 | ni | conserved hypothetical protein |
| 20d00008 | 6.03E-07 | ni | hypothetical protein | um00005 | 1.36E-09 | K07824 | probable Benzoate 4-monooxygenase cytochrome P450 |
| 22c00309 | 6.20E-07 | ni | hypothetical protein | um12031 | 1.42E-09 | ni | putative protein |
| 9c00260 | 6.31E-07 | K00698 | chitin synthase/hyaluronan synthase | um04482 | 1.44E-09 | ni | conserved hypothetical protein |
| 22c00122 | 6.54E-07 | ni | hypothetical protein | um10605 | 1.45E-09 | K13953 | probable ADH3 - alcohol dehydrogenase III |
| 12d00013 | 6.66E-07 | K03885 | NADH-dehydrogenase | um06438 | 1.46E-09 | ni | ni |
| 14c00051 | 7.54E-07 | ni | hypothetical protein | um02707 | 1.52E-09 | ni | conserved hypothetical protein |
| 7c00210 | 9.29E-07 | ni | hypothetical protein | um11513 | 1.56E-09 | ni | conserved hypothetical protein |
| 7c00362 | 9.37E-07 | ni | predicted esterase | um11116 | 1.56E-09 | ni | conserved hypothetical Ustilago-specific protein |
| 9c00380 | 9.52E-07 | ni | hypothetical protein | um01466 | 1.63E-09 | ni | related to acyl-coa dehydrogenase |
| 9c00352 | 1.02E-06 | ni | hypothetical protein | um03706.2 | 1.67E-09 | ni | conserved hypothetical protein |
| 8c00079 | 1.05E-06 | ni | hypothetical protein | um03397 | 1.78E-09 | ni | hypothetical protein |
| 20d00033 | 1.08E-06 | K13719 | OTU-like cysteine protease | um04104 | 1.83E-09 | ni | conserved hypothetical Ustilago-specific protein |
| 9c00057 | 1.09E-06 | ni | hypothetical protein | um00723 | 1.92E-09 | ni | related to stress response protein rds1p |
| 22d00256 | 1.12E-06 | ni | hypothetical protein | um10107 | 1.99E-09 | K04464 | probable mitogen-activated protein kinase MpkA |
| 7d00097 | 1.18E-06 | K07404 | hypothetical protein | um10120 | 2.04E-09 | K00698 | chitin synthase 3 |
| 11c00029 | 1.20E-06 | K07431 | cytochrome P450 | um03690 | 2.08E-09 | K03458 | probable purine permease |
| 16d00068 | 1.22E-06 | K10365 | F-actin capping protein, beta subunit | um11654 | 2.31E-09 | ni | putative protein |
| 22d00178 | 1.24E-06 | K01488 | adenine deaminase | um00371 | 2.32E-09 | ni | related to Opsin-1 |
| 1d00017 | 1.27E-06 | K00249 | predicted acyl-CoA dehydrogenase | um10545 | 2.33E-09 | ni | conserved hypothetical protein |
| 2c00017 | 1.30E-06 | ni | hypothetical protein | um03615 | 2.41E-09 | ni | related to Glucose oxidase |
| 6c00092 | 1.48E-06 | ni | hypothetical protein | um02917 | 2.47E-09 | ni | conserved hypothetical Ustilago-specific protein |
| 22d00261 | 1.59E-06 | K15532 | hypothetical protein | um00738 | 2.59E-09 | ni | conserved hypothetical protein |
| 19d00128 | 1.60E-06 | K08369 | synaptic vesicle transporter SV2 | um03451 | 2.61E-09 | ni | conserved hypothetical protein |
| 19d00028 | 1.60E-06 | ni | hypothetical protein | um11020 | 2.90E-09 | ni | conserved hypothetical protein |
| 14c00004 | 1.64E-06 | ni | multiple inositol polyphosphate phosphatase | um01812 | 3.01E-09 | K01078 | conserved hypothetical protein |
| 9d00258 | 1.66E-06 | ni | hypothetical protein | um04304 | 3.06E-09 | ni | related to allantoate permease |
| 2c00076 | 1.72E-06 | K01192 | predicted beta-mannosidase | um02865 | 3.16E-09 | ni | conserved hypothetical protein |
| 10d00008 | 1.72E-06 | K00161 | pyruvate dehydrogenase E1, alpha subunit | um06367 | 3.19E-09 | ni | putative protein |
| 5d00132 | 1.83E-06 | ni | sorting nexin SNX9/SH3PX1 | um03576 | 3.23E-09 | K00380 | related to MET10 - sulfite reductase flavin-binding subunit |
| 8c00007 | 2.02E-06 | ni | hypothetical protein | um03506 | 3.28E-09 | ni | conserved hypothetical protein |
| 14d00060 | 2.04E-06 | ni | sensory transduction histidine kinase | um02191 | 3.53E-09 | ni | ni |
| 5d00059 | 2.09E-06 | ni | GTP-binding ADP-ribosylation factor-like protein ARL1 | um01858 | 3.55E-09 | K01480 | conserved hypothetical protein |
| 25d00002 | 2.10E-06 | ni | hypothetical protein | um03909 | 3.70E-09 | ni | hypothetical protein |
| 2c00044 | 2.28E-06 | K00122 | glyoxylate/hydroxypyruvate reductase | um03116 | 3.72E-09 | ni | conserved hypothetical protein |
| 14d00106 | 2.30E-06 | ni | enoyl-coa hydratase | um02747 | 3.81E-09 | ni | conserved hypothetical protein |
| 18d00023 | 2.31E-06 | ni | hypothetical protein | um12303 | 3.88E-09 | ni | hypothetical protein |
| 9c00392 | 2.52E-06 | ni | hypothetical protein | um00428 | 3.98E-09 | ni | conserved hypothetical protein |
| 6d00115 | 2.62E-06 | ni | hypothetical protein | um06012 | 4.17E-09 | K16261 | probable general amino acid permease |
| 22c00035 | 2.70E-06 | K00223 | sterol reductase | um01508 | 4.20E-09 | K03441 | related to Aquaporin 3 |
| 12d00011 | 2.75E-06 | ni | hypothetical protein | um05938 | 4.94E-09 | K06911 | related to Pirin |
| 12d00035 | 2.93E-06 | K17268 | vesicle coat complex COPI, epsilon subunit | um00241 | 4.97E-09 | ni | putative protein |
| 7c00247 | 2.95E-06 | ni | synaptic vesicle transporter SVOP and related transporters | um10864 | 5.13E-09 | ni | related to GNA1 - essential acetyltransferase |
| 6c00122 | 3.03E-06 | ni | predicted transporter | um11580 | 5.17E-09 | ni | retrotransposon HobS hobase |
| 6c00008 | 3.12E-06 | K00480 | hypothetical protein | um11506 | 5.23E-09 | ni | putative protein |
| 9c00317 | 3.17E-06 | ni | hypothetical protein | um01872 | 5.24E-09 | K00108 | related to gmc type oxidoreductase |
| 16c00073 | 3.17E-06 | ni | AAA+-type ATPase | um02207 | 5.45E-09 | ni | conserved hypothetical protein |
| 14c00104 | 3.23E-06 | ni | molybdenum cofactor sulfurase | um11068 | 5.52E-09 | ni | related to Copper amine oxidase 1 |
| 7c00252 | 3.26E-06 | K00249 | medium-chain acyl-CoA dehydrogenase | um04145 | 5.68E-09 | ni | conserved hypothetical Ustilago-specific protein |
| 5c00132 | 3.56E-06 | ni | vacuolar protein sorting-associated protein | um03978 | 5.80E-09 | ni | hypothetical protein |
| 22d00203 | 3.62E-06 | ni | hypothetical protein | um12090 | 5.83E-09 | ni | conserved hypothetical protein |
| 5c00071 | 3.69E-06 | ni | hypothetical protein | um02801 | 6.23E-09 | K00262 | probable NADP-specific glutamate dehydrogenase |
| 6c00090 | 3.71E-06 | ni | hypothetical protein | um00196 | 6.57E-09 | K07034 | probable FUN34 - transmembrane protein involved in ammonia production |
| 16d00070 | 3.74E-06 | ni | hypothetical protein | um00955 | 7.20E-09 | K00507 | probable stearoyl-CoA desaturase |
| 8d00001 | 3.85E-06 | ni | hypothetical protein | um01951 | 7.35E-09 | ni | conserved hypothetical protein |
| 13c00037 | 4.06E-06 | ni | hypothetical protein | um10189 | 7.36E-09 | ni | ferrichrome siderophore peptide synthetase |
| 1d00041 | 4.08E-06 | K09051 | transcriptional activator FOSB/c-Fos | um03559 | 7.46E-09 | ni | related to beta-1 |
| 8d00053 | 4.16E-06 | ni | amino acid transporters | um03689 | 7.93E-09 | ni | conserved hypothetical protein |
| 19d00076 | 4.70E-06 | ni | hypothetical protein | um11450 | 8.59E-09 | ni | Hgl1p |
| 16c00063 | 5.10E-06 | K01338 | mitochondrial ATP-dependent protease PIM1/LON | um04181 | 8.80E-09 | ni | conserved hypothetical protein |
| 22c00121 | 5.10E-06 | ni | predicted transporter | um05507 | 8.84E-09 | ni | conserved hypothetical protein |
| 24c00011 | 5.10E-06 | ni | hypothetical protein | um10464 | 1.00E-08 | ni | putative protein |
| 27c00005 | 5.20E-06 | ni | hypothetical protein | um10518 | 1.06E-08 | ni | ni |
| 10c00070 | 5.26E-06 | ni | hypothetical protein | um03691 | 1.10E-08 | K03293 | probable lysine-specific permease |
| 15c00036 | 5.27E-06 | K00637 | sterol O-acyltransferase | um01171 | 1.12E-08 | ni | related to 4-coumarate-CoA ligase |
| 3d00095 | 5.35E-06 | K00643 | 5-aminolevulinate synthase | um04347 | 1.14E-08 | ni | probable isp4 - oligopeptide transporter |
| 20d00069 | 5.41E-06 | ni | hypothetical protein | um10816 | 1.14E-08 | ni | conserved hypothetical protein |
| 19c00002 | 5.59E-06 | ni | hypothetical protein | um03415 | 1.19E-08 | K03379 | related to putative monooxygenase |
| 14d00094 | 5.62E-06 | ni | transporter, ABC superfamily | um02704 | 1.26E-08 | ni | related to allantoate permease |
| 20d00043 | 5.79E-06 | ni | hypothetical protein | um06456 | 1.29E-08 | ni | related to aminopeptidase Y precursor |
| 22d00239 | 5.92E-06 | ni | hypothetical protein | um11448 | 1.35E-08 | K03941 | probable NADH-ubiquinone oxidoreductase 23 kDa subunit precursor |
| 7d00041 | 6.09E-06 | ni | mitochondrial ribosomal protein S10 | um05550 | 1.36E-08 | ni | related to EXG1 - exo-beta-1 |
| 12d00001 | 6.32E-06 | K11811 | hypothetical protein | um05019 | 1.46E-08 | K00789 | probable SAM2 - S-adenosylmethionine synthetase 2 |
| 5d00024 | 6.78E-06 | ni | hypothetical protein | um05036 | 1.48E-08 | ni | related to endo-1 |
| 7d00311 | 6.91E-06 | ni | hypothetical protein | um01182 | 1.50E-08 | ni | ni |
| 7c00003 | 7.11E-06 | ni | multicopper oxidases | um03398 | 1.51E-08 | ni | related to transesterase |
| 6d00046 | 7.67E-06 | K01194 | neutral trehalase | um02746 | 1.54E-08 | ni | hypothetical protein |
| 5c00159 | 7.87E-06 | ni | hypothetical protein | um05509 | 1.71E-08 | K14849 | related to RRP1 - involved in processing rRNA precursor species to mature rRNAs |
| 3c00057 | 7.90E-06 | ni | hypothetical protein | um11815.2 | 1.80E-08 | ni | hypothetical protein |
| 9d00241 | 8.53E-06 | ni | hypothetical protein | um03139 | 1.81E-08 | ni | conserved hypothetical protein |
| 27d00028 | 8.54E-06 | ni | hypothetical protein | um01213 | 1.82E-08 | ni | conserved hypothetical protein |
| 26c00043 | 9.13E-06 | ni | tyrosine kinase specific for activated | um03694.2 | 1.90E-08 | ni | conserved hypothetical protein |
| 9c00329 | 9.15E-06 | K12795 | suppressor of G2 allele of skp1 | um00316 | 1.91E-08 | ni | conserved hypothetical protein |
| 13d00001 | 9.22E-06 | ni | predicted transporter | um05972 | 1.94E-08 | K08141 | probable Maltose permease |
| 8d00002 | 9.42E-06 | ni | hypothetical protein | um01434 | 2.01E-08 | ni | siderophore peptide synthetase involved in ferrichromeA biosynthesis |
| 5c00004 | 9.51E-06 | ni | glucose dehydrogenase | um02986 | 2.04E-08 | ni | conserved hypothetical protein |
| 24c00004 | 1.02E-05 | ni | hypothetical protein | um04247 | 2.06E-08 | ni | related to Cholinesterase precursor |
| 3d00101 | 1.03E-05 | ni | hypothetical protein | um02371 | 2.09E-08 | ni | related to serine |
| 9c00208 | 1.04E-05 | ni | hypothetical protein | um03563 | 2.09E-08 | ni | conserved hypothetical protein |
| 20d00067 | 1.19E-05 | ni | 3-Methylcrotonyl-CoA carboxylase, non-biotin containing subunit | um10188 | 2.14E-08 | ni | l-ornithine N5-oxygenase |
| 3c00075 | 1.19E-05 | ni | hypothetical protein | um06125 | 2.17E-08 | ni | putative protein |
| 7c00184 | 1.21E-05 | ni | hypothetical protein | um11321 | 2.29E-08 | ni | ni |
| 6d00082 | 1.34E-05 | K00008 | sorbitol dehydrogenase | um11610 | 2.33E-08 | ni | conserved hypothetical protein |
| 26c00080 | 1.36E-05 | ni | hypothetical protein | um04123 | 2.52E-08 | ni | ni |
| 22d00033 | 1.40E-05 | ni | hypothetical protein | um02208 | 2.55E-08 | K00232 | probable acyl-CoA oxidase |
| 9c00359 | 1.48E-05 | ni | hypothetical protein | um00813 | 2.67E-08 | ni | conserved hypothetical protein |
| 6c00015 | 1.58E-05 | K10578 | non-canonical ubiquitin conjugating enzyme 1 | um04107 | 2.79E-08 | ni | related to Phenol 2-monooxygenase |
| 22c00153 | 1.59E-05 | ni | hypothetical protein | um11434 | 2.87E-08 | ni | hypothetical protein |
| 19d00036 | 1.61E-05 | ni | multidrug resistance-associated protein | um05414 | 2.89E-08 | ni | probable aflatoxin efflux pump AFLT |
| 9c00017 | 1.62E-05 | K01126 | glycerophosphoryl diester phosphodiesterase | um00157 | 2.90E-08 | K00873 | probable pyruvate kinase |
| 7d00206 | 1.64E-05 | K01669 | deoxyribodipyrimidine photolyase | um05811 | 3.08E-08 | ni | related to KRE6 - glucan synthase subunit |
| 8c00120 | 1.70E-05 | ni | cytochrome P450 CYP3/CYP5/CYP6/CYP9 subfamilies | um02459 | 3.08E-08 | ni | related to neutral amino acid permease |
| 5d00135 | 1.72E-05 | ni | hypothetical protein | um04696 | 3.09E-08 | ni | putative protein |
| 7d00359 | 1.76E-05 | ni | synaptic vesicle transporter SVOP and related transporters | um01232 | 3.20E-08 | K01372 | related to LAP3 - member of the GAL regulon |
| 27d00074 | 1.84E-05 | ni | acetylcholinesterase | um03935 | 3.31E-08 | K00309 | related to fructosyl amino acid oxidase |
| 25c00080 | 1.89E-05 | ni | hypothetical protein | um06078 | 3.38E-08 | K01210 | conserved hypothetical protein |
| 2d00014 | 1.97E-05 | K02357 | hypothetical protein | um02809 | 3.45E-08 | K04618 | probable galactose oxidase precursor |
| 20d00016 | 2.02E-05 | K07973 | hypothetical protein | um05751 | 3.64E-08 | ni | ni |
| 12c00001 | 2.02E-05 | ni | hypothetical protein | um11776 | 3.65E-08 | ni | probable AYT1 - trichothecene 3-O-acetyltransferase |
| 1d00007 | 2.07E-05 | ni | hypothetical protein | um02023 | 3.77E-08 | ni | related to long chain fatty alcohol oxidase |
| 4d00042 | 2.09E-05 | K01720 | hypothetical protein | um11569 | 3.90E-08 | ni | related to MKC7 - aspartyl protease of the periplasmic space |
| 1c00017 | 2.10E-05 | ni | hypothetical protein | um12080 | 3.92E-08 | ni | related to PNS1 - Protein of unknown function |
| 3d00016 | 2.13E-05 | K00232 | acyl-coa oxidase | um03325 | 3.95E-08 | K16261 | probable general amino acid permease |
| 10c00099 | 2.23E-05 | ni | component of vacuolar transporter chaperone | um03561 | 4.21E-08 | ni | conserved hypothetical protein |
| 8c00073 | 2.33E-05 | ni | hypothetical protein | um06203 | 4.23E-08 | ni | ni |
| 5c00046 | 2.36E-05 | ni | hypothetical protein | um03882 | 4.31E-08 | ni | ni |
| 27d00079 | 2.55E-05 | ni | hypothetical protein | um06190 | 4.33E-08 | ni | related to Chitinase |
| 20c00026 | 2.62E-05 | ni | hypothetical protein | um04444 | 4.44E-08 | ni | related to ANT1 - Peroxisomal transporter of adenine nucleotides |
| 10c00095 | 2.66E-05 | K03800 | lipoate-protein ligase A | um06485 | 4.58E-08 | ni | putative protein |
| 9c00062 | 2.74E-05 | K01423 | hypothetical protein | um03507 | 4.64E-08 | ni | conserved hypothetical protein |
| 8c00119 | 2.75E-05 | ni | hypothetical protein | um11197 | 4.79E-08 | ni | putative protein |
| 19d00129 | 2.77E-05 | K00621 | glucosamine-phosphate N-acetyltransferase | um10268 | 4.80E-08 | ni | putative protein |
| 19d00077 | 2.81E-05 | ni | dihydroxy-acid dehydratase | um10395 | 4.95E-08 | ni | ni |
| 25c00015 | 3.08E-05 | ni | permease of the major facilitator superfamily | um00034 | 5.01E-08 | ni | probable mfs-multidrug-resistance transporter |
| 10c00044 | 3.23E-05 | K11841 | ubiquitin-specific protease | um03400 | 5.24E-08 | ni | related to HNM1 - choline permease |
| 20d00010 | 3.29E-05 | ni | hypothetical protein | um04364 | 5.36E-08 | ni | probable EXG1 - Exo-1 |
| 5c00154 | 3.40E-05 | ni | glucosyltransferase - Alg6p | um05152 | 5.40E-08 | ni | conserved hypothetical protein |
| 13d00072 | 3.42E-05 | ni | hypothetical protein | um10587 | 5.56E-08 | K01514 | conserved hypothetical protein |
| 14c00041 | 3.43E-05 | K07508 | acetyl-coa acetyltransferase | um00719 | 5.58E-08 | ni | hypothetical protein |
| 7d00248 | 3.45E-05 | K01581 | ornithine decarboxylase | um01997.2 | 5.80E-08 | ni | putative protein |
| 9c00071 | 3.48E-05 | ni | reductases with broad range of substrate specificities | um00664 | 6.03E-08 | ni | putative protein |
| 9d00103 | 3.49E-05 | K01537 | Ca2+ transporting ATPase | um12098 | 6.04E-08 | ni | putative protein |
| 7d00346 | 3.53E-05 | ni | hypothetical protein | um06064 | 6.05E-08 | ni | conserved hypothetical protein |
| 7d00069 | 3.55E-05 | ni | hypothetical protein | um01793 | 7.09E-08 | K00100 | probable AAD14 - Putative aryl-alcohol reductase |
| 10d00073 | 3.55E-05 | K11267 | sister chromatid cohesion complex Cohesin, subunit PDS5 | um11593 | 7.32E-08 | ni | related to PYC2 - pyruvate carboxylase 2 |
| 19d00091 | 3.60E-05 | ni | hypothetical protein | um04480 | 7.45E-08 | ni | related to ADH2 - alcohol dehydrogenase II |
| 7c00296 | 3.66E-05 | K11498 | kinesin-like protein | um02182 | 7.49E-08 | ni | probable NADPH-dependent beta-ketoacyl reductase |
| 5c00047 | 3.66E-05 | K00632 | 3-oxoacyl CoA thiolase | um01212 | 7.69E-08 | K00311 | probable flavoprotein-ubiquinone oxidoreductase |
| 7d00098 | 3.73E-05 | ni | hypothetical protein | um12032 | 7.74E-08 | ni | related to ATP-dependent DNA helicase RecQ |
| 18d00005 | 3.74E-05 | K14552 | WD40 repeat protein | um02166 | 8.22E-08 | ni | putative protein |

| | | | | | | | |
|---|---|---|---|---|---|---|---|
| 3c00063 | 3.84E-05 | ni | glyoxylate/hydroxypyruvate reductase | um11015 | 8.61E-08 | ni | conserved hypothetical protein |
| 7d00165 | 3.87E-05 | K04043 | molecular chaperones mortalin/PBP74/GRP75 | um10691.2 | 8.67E-08 | K01897 | related to Long-chain-fatty-acid--CoA ligase |
| 6c00056 | 4.12E-05 | ni | hypothetical protein | um02161 | 8.82E-08 | ni | conserved hypothetical protein |
| 6c00053 | 4.33E-05 | K00020 | predicted dehydrogenase | um03620 | 9.11E-08 | ni | related to Regulatory protein alcR |
| 19d00046 | 4.40E-05 | K12486 | predicted GTPase-activating protein | um11775 | 9.49E-08 | ni | putative protein |
| 7c00279 | 4.42E-05 | ni | predicted inosine-uridine preferring nucleoside hydrolase | um00079 | 9.70E-08 | K00100 | conserved hypothetical protein |
| 9c00232 | 4.45E-05 | K10589 | E3 ubiquitin protein ligase | um10897 | 9.79E-08 | ni | ni |
| 13c00027 | 4.58E-05 | ni | hypothetical protein | um01476 | 9.92E-08 | ni | related to HXT1 - Low-affinity hexose facilitator |
| 9c00270 | 4.68E-05 | ni | hypothetical protein | um04912 | 1.01E-07 | ni | putative protein |
| 24c00071 | 4.79E-05 | K13535 | predicted hydrolase/acyltransferase | um01585 | 1.01E-07 | ni | ni |
| 16c00005 | 4.91E-05 | ni | hypothetical protein | um10223 | 1.10E-07 | ni | ni |
| 14c00100 | 4.95E-05 | K11273 | helicase of the DEAD superfamily | um01090 | 1.15E-07 | ni | probable POT1 - acetyl-CoA C-acyltransferase |
| 7c00046 | 5.17E-05 | K11434 | protein arginine N-methyltransferase PRMT1 and related enzyme | um10388 | 1.17E-07 | ni | putative protein |
| 8d00010 | 5.19E-05 | K00380 | NADP/FAD dependent oxidoreductase | um03470 | 1.21E-07 | K01537 | related to putative calcium P-type ATPase NCA-2 |
| 8d00019 | 5.21E-05 | ni | cytochrome P450 CYP3/CYP5/CYP6/CYP9 subfamilies | um11744 | 1.22E-07 | K00236 | related to SDH3 - cytochrome b560 subunit of respiratory complex II |
| 19d00057 | 5.40E-05 | K03255 | uncharacterized protein CLU1/cluA/TIF31 | um04410 | 1.26E-07 | ni | probable siderophore iron transporter mirC |
| 16c00062 | 5.42E-05 | K11756 | chromatin remodeling complex RSC, subunit RSC1 | um04106 | 1.26E-07 | ni | related to O-methyltransferase B |
| 3d00003 | 5.54E-05 | K00026 | NAD-dependent malate dehydrogenase | um01599 | 1.27E-07 | ni | related to ECI1 - delta3-cis-delta2-trans-enoyl-CoA isomerase |
| 6c00089 | 5.57E-05 | ni | hypothetical protein | um02508 | 1.28E-07 | K00128 | indole-3-acetaldehyde dehydrogenase |
| 10c00045 | 5.57E-05 | ni | glycosyl transferase | um11777 | 1.31E-07 | ni | conserved hypothetical protein |
| 6c00069 | 5.77E-05 | ni | Ca2+-dependent lipid-binding protein CLB1 | um05325 | 1.34E-07 | ni | conserved hypothetical protein |
| 18c00034 | 5.82E-05 | ni | hypothetical protein | um03508 | 1.40E-07 | ni | conserved hypothetical protein |
| 9d00061 | 5.84E-05 | ni | hypothetical protein | um00573 | 1.44E-07 | ni | conserved hypothetical protein |
| 6d00102 | 6.00E-05 | ni | hypothetical protein | um01583 | 1.45E-07 | ni | ni |
| 10d00051 | 6.00E-05 | ni | hypothetical protein | um03990 | 1.46E-07 | ni | related to Short-chain dehydrogenase |
| 3d00050 | 6.03E-05 | ni | fumarate reductase, flavoprotein subunit | um00406 | 1.47E-07 | ni | conserved hypothetical protein |
| 3c00008 | 6.28E-05 | ni | synaptic vesicle transporter SVOP and related transporters | um01827 | 1.50E-07 | K01078 | related to acid phosphatase ACP2 precursor |
| 9c00285 | 6.34E-05 | K14573 | nucleolar protein fibrillarin NOP77 | um06500 | 1.58E-07 | ni | conserved hypothetical Ustilago-specific protein |
| 7d00015 | 6.45E-05 | ni | hypothetical protein | um05766 | 1.62E-07 | ni | probable YHM2 - Protein of the mitochondrial carrier family |
| 5d00096 | 6.63E-05 | K01103 | fructose-6-phosphate 2-kinase | um10540 | 1.69E-07 | ni | related to blue-light-inducible Bli-3 protein |
| 7d00006 | 6.74E-05 | K02355 | mitochondrial elongation factor | um15095 | 1.71E-07 | ni | ni |
| 14d00079 | 6.82E-05 | K01743 | hypothetical protein | um03372 | 1.75E-07 | ni | conserved hypothetical protein |
| 21c00001 | 6.93E-05 | ni | hypothetical protein | um10083 | 1.77E-07 | ni | ni |
| 13d00014 | 7.17E-05 | K05012 | cl- channel CLC-3 and related proteins | um04530 | 1.79E-07 | ni | probable GGC1 - Protein of the mitochondrial carrier family |
| 5c00060 | 7.48E-05 | K01681 | aconitase/homoaconitase | um00229 | 1.84E-07 | ni | hypothetical protein |
| 6d00060 | 7.74E-05 | ni | hypothetical protein | um00970 | 2.02E-07 | ni | conserved hypothetical Ustilago-specific protein |
| 2d00069 | 8.23E-05 | ni | hypothetical protein | um05339 | 2.19E-07 | K01834 | probable 2 |
| 19d00126 | 8.25E-05 | K00972 | UDP-N-acetylglucosamine pyrophosphorylase | um11562 | 2.21E-07 | ni | hydrophobin 2 |
| 6d00068 | 8.30E-05 | ni | hypothetical protein | um11502 | 2.24E-07 | K01520 | probable dUTP pyrophosphatase |
| 14d00119 | 8.52E-05 | ni | uncharacterized conserved protein | um00164.2 | 2.32E-07 | K13091 | related to splicing factor HCC1 |
| 27d00097 | 8.69E-05 | ni | hypothetical protein | um01726 | 2.36E-07 | K00860 | probable MET14 - ATP adenosine-5 |
| 15c00003 | 8.70E-05 | ni | hypothetical protein | um12173 | 2.41E-07 | ni | putative protein |
| 8d00112 | 8.91E-05 | ni | copper amine oxidase | um03977 | 2.44E-07 | ni | conserved hypothetical protein |
| 5c00105 | 8.94E-05 | ni | hypothetical protein | um03663 | 2.54E-07 | K03457 | probable uracil permease |
| 10d00013 | 9.26E-05 | K10534 | cytochrome b5 | um02416 | 2.54E-07 | ni | conserved hypothetical protein |
| 8d00008 | 9.27E-05 | K00626 | acetyl-coa acetyltransferase | um04742 | 2.57E-07 | ni | related to stomatin |
| 14c00062 | 9.51E-05 | K01493 | deoxycytidylate deaminase | um12178 | 2.57E-07 | K14333 | related to 5-carboxyvanillate decarboxylase |
| 9d00058 | 9.57E-05 | K00863 | dihydroxyacetone kinase | um02172 | 2.61E-07 | ni | related to methylglyoxal reductase |
| 7c00088 | 9.66E-05 | ni | hypothetical protein | um04616 | 2.66E-07 | ni | ni |
| 9d00045 | 9.76E-05 | ni | synaptic vesicle transporter SV2 | um11940 | 2.80E-07 | ni | putative protein |
| 5c00074 | 9.91E-05 | K16302 | predicted membrane protein | um00825 | 2.83E-07 | ni | conserved hypothetical Ustilago-specific protein |
| 25c00060 | 0.0001 | ni | sorbitol dehydrogenase | um04165 | 2.86E-07 | K10740 | related to single-stranded dna binding protein 12k chain |
| 15c00028 | 0.0001 | ni | hypothetical protein | um02198 | 2.86E-07 | ni | conserved hypothetical protein |
| 3c00033 | 0.0001 | K00328 | inositol polyphosphate multikinase, component of the ARGR trans | um01591 | 2.96E-07 | ni | ni |
| 12c00014 | 0.00011 | ni | hypothetical protein | um03522 | 3.00E-07 | ni | related to UGA4 - GABA permease - also involved in delta-aminolevulinate transport |
| 9c00118 | 0.00011 | K02910 | 60S ribosomal protein L31 | um00708 | 3.04E-07 | ni | related to monooxygenase |
| 22c00226 | 0.00011 | ni | predicted hydrolases or acyltransferases | um10519 | 3.17E-07 | ni | putative protein |
| 9c00064 | 0.00011 | ni | hypothetical protein | um01710 | 3.18E-07 | ni | related to N-carbamoyl-L-amino acid hydrolase |
| 7c00056 | 0.00011 | K03661 | vacuolar H+-ATPase V0 sector, subunit c" | um11984 | 3.39E-07 | ni | conserved hypothetical protein |
| 24d00021 | 0.00011 | K00257 | short-chain acyl-CoA dehydrogenase | um02012 | 3.57E-07 | ni | conserved hypothetical protein |
| 9d00018 | 0.00011 | ni | aldehyde reductase | um06098 | 3.58E-07 | ni | related to extracellular elastinolytic metalloproteinase precursor |
| 9c00054 | 0.00011 | ni | phenylalanine and histidine ammonia-lyase | um03548 | 3.74E-07 | K07119 | related to endo-polygalacturonase |
| 18c00006 | 0.00011 | ni | hypothetical protein | um03228 | 3.76E-07 | ni | related to 4-coumarate--CoA ligase 1 |
| 22c00214 | 0.00012 | ni | non-ribosomal peptide synthetase | um05514 | 3.77E-07 | ni | conserved hypothetical protein |
| 27c00066 | 0.00012 | ni | hypothetical protein | um01335 | 3.78E-07 | K00252 | probable glutaryl-CoA dehydrogenase |
| 20d00051 | 0.00012 | ni | uncharacterized conserved protein HEN1/CORYMBOSA2 | um01432 | 3.93E-07 | ni | related to N6-hydroxylysine acetyl transferase |
| 20d00030 | 0.00012 | ni | hypothetical protein | um10589 | 3.94E-07 | K01758 | related to CYS3 - cystathionine gamma-lyase |
| 27d00101 | 0.00013 | ni | hypothetical protein | um01422 | 3.97E-07 | ni | probable Lipase B precursor |
| 9d00400 | 0.00013 | K01679 | fumarase | um06490 | 4.00E-07 | K08176 | probable PHO84 - Inorganic phosphate permease |
| 9c00074 | 0.00013 | ni | permease of the major facilitator superfamily | um05958 | 4.27E-07 | ni | probable maltose permease |
| 9d00197 | 0.00013 | ni | hypothetical protein | um04615 | 4.42E-07 | ni | ni |
| 9d00281 | 0.00014 | ni | hypothetical protein | um04479 | 4.78E-07 | ni | related to 4-coumarate--CoA ligase |
| 9c00100 | 0.00014 | ni | predicted proline-serine-threonine phosphatase-interacting proteir | um10888 | 4.92E-07 | ni | putative protein |
| 3d00001 | 0.00014 | ni | hypothetical protein | um11531 | 4.98E-07 | ni | ni |
| 19d00020 | 0.00014 | ni | predicted membrane protein | um05396.2 | 5.69E-07 | ni | conserved hypothetical protein |
| 11d00078 | 0.00015 | ni | hypothetical protein | um04697 | 5.75E-07 | ni | conserved hypothetical protein |
| 11d00075 | 0.00015 | ni | hypothetical protein | um03734 | 5.80E-07 | K01251 | probable adenosylhomocysteinase |
| 5c00122 | 0.00015 | ni | hypothetical protein | um12127 | 5.84E-07 | ni | hypothetical protein |
| 24d00012 | 0.00015 | ni | acyl-coa synthetase | um01755 | 5.90E-07 | ni | putative protein |
| 9c00378 | 0.00015 | ni | checkpoint kinase | um05370 | 6.33E-07 | ni | putative protein |
| 11c00014 | 0.00015 | K09510 | molecular chaperone | um10861 | 6.34E-07 | ni | related to Reticuline oxidase precursor |
| 12d00012 | 0.00015 | ni | GTP-binding protein | um00108 | 6.37E-07 | ni | related to D-arabinitol 2-dehydrogenase |
| 3d00018 | 0.00015 | ni | hypothetical protein | um02235 | 6.38E-07 | ni | related to AMP-binding protein |
| 7d00005 | 0.00015 | ni | FOG, Zn-finger | um00330 | 6.50E-07 | ni | related to UTR2 - cell wall protein |
| 14d00036 | 0.00015 | ni | polyadenylate-binding protein | um05671 | 6.68E-07 | K00818 | related to acetylornithine aminotransferase precursor |
| 18c00031 | 0.00016 | ni | hypothetical protein | um03568 | 6.69E-07 | ni | related to regulatory protein alcR |
| 16c00012 | 0.00016 | K01897 | long-chain acyl-CoA synthetases | um01859 | 6.73E-07 | ni | related to ribitol kinase |
| 9d00328 | 0.00016 | ni | ATP-dependent RNA helicase pitchoune | um01192 | 6.85E-07 | ni | conserved hypothetical protein |
| 25c00043 | 0.00017 | K00166 | branched chain alpha-keto acid dehydrogenase complex, alpha su | um00902 | 7.26E-07 | ni | conserved hypothetical protein |
| 18c00018 | 0.00017 | ni | FOG, Zn-finger | um11038 | 7.34E-07 | K03940 | probable NADH-ubiquinone oxidoreductase 19 |
| 7c00106 | 0.00017 | K04371 | mitogen-activated protein kinase | um01747 | 7.56E-07 | ni | ni |
| 16d00050 | 0.00018 | K12604 | negative regulator of transcription | um12144 | 7.56E-07 | ni | conserved hypothetical Ustilago-specific protein |
| 22c00252 | 0.00018 | K01637 | isocitrate lyase | um05952 | 7.64E-07 | ni | putative protein |
| 9c00440 | 0.00018 | ni | hypothetical protein | um11070 | 7.71E-07 | K01175 | probable lipase precursor |
| 9d00152 | 0.00019 | K01012 | biotin synthase | um03032 | 7.84E-07 | ni | conserved hypothetical protein |
| 12c00120 | 0.00019 | ni | hypothetical protein | um02201.2 | 7.92E-07 | ni | conserved hypothetical protein |
| 25c00002 | 0.00019 | ni | dehydrogenases with different specificities | um01005 | 8.08E-07 | K01648 | probable ATP citrate lyase subunit 1 |
| 13c00043 | 0.00019 | K03575 | A/G-specific adenine DNA glycosylase | um11693 | 8.49E-07 | ni | related to Glucose 1-dehydrogenase |
| 5c00163 | 0.00019 | ni | hypothetical protein | um00082 | 8.69E-07 | ni | putative protein |
| 9c00076 | 0.0002 | ni | cell division control protein | um04871 | 8.74E-07 | K00927 | probable PGK1 - phosphoglycerate kinase |
| 7d00081 | 0.0002 | ni | hypothetical protein | um11750 | 8.76E-07 | K10807 | probable RNR1 - ribonucleoside-diphosphate reductase large subunit |
| 20c00066 | 0.0002 | K16261 | amino acid transporters | um04089 | 9.11E-07 | ni | conserved hypothetical Ustilago-specific protein |
| 16c00069 | 0.0002 | K15424 | protein phosphatase 2A regulatory subunit A and related proteins | um00230 | 9.17E-07 | ni | putative protein |
| 27c00012 | 0.0002 | ni | hypothetical protein | um01647 | 9.20E-07 | K03363 | related to CDC20 - cell division control protein |
| 16d00052 | 0.0002 | K08838 | serine/threonine protein kinase | um00397 | 9.28E-07 | ni | putative protein |
| 5c00035 | 0.00021 | ni | hypothetical protein | um11007 | 9.38E-07 | ni | related to GTP-binding protein Rab5c |
| 22d00286 | 0.00021 | ni | FOG, Reverse transcriptase | um00593 | 9.45E-07 | ni | conserved hypothetical protein |
| 7c00050 | 0.00021 | ni | hypothetical protein | um10032 | 9.47E-07 | ni | related to N-acetyltransferase |
| 1c00035 | 0.00022 | ni | hypothetical protein | um10904 | 9.49E-07 | K00759 | probable APT1 - adenine phosphoribosyltransferase |
| 26c00021 | 0.00023 | K08835 | ste20-like serine/threonine protein kinase | um12024 | 9.54E-07 | ni | putative protein |
| 22d00260 | 0.00024 | ni | hypothetical protein | um01792 | 9.59E-07 | ni | conserved hypothetical protein |
| 16d00051 | 0.00024 | ni | hypothetical protein | um00111 | 1.00E-06 | ni | related to membrane protein |
| 9d00087 | 0.00025 | ni | uncharacterized conserved protein WDR8 | um11147 | 1.04E-06 | ni | conserved hypothetical Ustilago-specific protein |
| 11c00083 | 0.00025 | K10082 | lectin VIP36 | um02052 | 1.06E-06 | ni | related to white collar 1 protein |
| 2c00009 | 0.00025 | ni | hypothetical protein | um00567 | 1.09E-06 | ni | related to B2-aldehyde-forming enzyme |
| 12c00114 | 0.00026 | ni | ribosomal RNA adenine dimethylase | um02583 | 1.10E-06 | ni | probable FAT1 - Long-chain fatty acid transporter |
| 9c00348 | 0.00026 | ni | hypothetical protein | um06422 | 1.13E-06 | ni | conserved hypothetical protein |
| 22d00133 | 0.00027 | ni | glucose dehydrogenase | um05997 | 1.16E-06 | ni | putative protein |
| 13c00070 | 0.00027 | ni | predicted peptidyl-tRNA hydrolase | um11870 | 1.18E-06 | ni | putative protein |
| 9d00012 | 0.00027 | ni | hormone-sensitive lipase HSL | um05664 | 1.18E-06 | ni | related to Cytochrome P450 |
| 22c00012 | 0.00028 | ni | acyl-coa synthetase | um02095 | 1.20E-06 | ni | putative protein |
| 11c00077 | 0.00029 | ni | myosin regulatory light chain | um12027 | 1.32E-06 | ni | putative protein |
| 20d00059 | 0.0003 | K08794 | Ca2+/calmodulin-dependent protein kinase | um05521 | 1.34E-06 | ni | conserved hypothetical protein |
| 7c00343 | 0.0003 | ni | reductases with broad range of substrate specificities | um05953 | 1.34E-06 | ni | putative protein |
| 7c00197 | 0.0003 | K00383 | pyridine nucleotide-disulphide oxidoreductase | um11764 | 1.38E-06 | ni | ni |
| 15d00008 | 0.0003 | K02896 | 60s ribosomal protein L30 isolog | um10815 | 1.41E-06 | ni | conserved hypothetical protein |
| 11d00061 | 0.00031 | ni | protein containing adaptin N-terminal region | um06010 | 1.44E-06 | K01702 | alpha-isopropylmalate isomerase |
| 8c00002 | 0.00031 | ni | hypothetical protein | um04484 | 1.44E-06 | ni | probable choline-sulfatase |
| 18d00071 | 0.00032 | ni | hypothetical protein | um00934 | 1.48E-06 | K00549 | probable MET6 - methionine synthase |
| 10d00025 | 0.00034 | ni | hypothetical protein | um04873 | 1.56E-06 | K05824 | probable LYS12 - Homo-isocitrate dehydrogenase |
| 6c00107 | 0.00034 | ni | hypothetical protein | um03474 | 1.59E-06 | ni | conserved hypothetical protein |
| 26d00092 | 0.00035 | K00618 | acetylglutamate kinase | um01952 | 1.62E-06 | K13281 | related to UV-endonuclease UVE-1 |
| 6d00049 | 0.00036 | ni | hypothetical protein | um10486 | 1.63E-06 | ni | conserved hypothetical Ustilago-specific protein |
| 22d00282 | 0.00036 | ni | hypothetical protein | um01082 | 1.65E-06 | ni | putative protein |
| 13d00081 | 0.00036 | ni | hypothetical protein | um05248 | 1.65E-06 | ni | related to TPO3 - Polyamine transport protein |
| 9d00156 | 0.00037 | ni | hypothetical protein | um02721 | 1.66E-06 | ni | conserved hypothetical protein |
| 7c00221 | 0.00038 | ni | nuclear pore complex, Nup214/CAN component | um11935 | 1.66E-06 | ni | conserved hypothetical Ustilago-specific protein |
| 26d00061 | 0.00038 | ni | predicted K+/H+-antiporter | um02796 | 1.66E-06 | ni | probable ATP-binding multidrug cassette transport protein |
| 6d00111 | 0.00038 | ni | hypothetical protein | um11558 | 1.67E-06 | ni | putative protein |
| 22c00278 | 0.0004 | ni | glucosidase II catalytic (alpha) subunit | um00655 | 1.69E-06 | K01179 | probable Family 9 glycosyl hydrolase |
| 12d00081 | 0.00042 | K00428 | hypothetical protein | um04101 | 1.69E-06 | ni | related to BAS1 - transcription factor |
| 22d00024 | 0.00042 | K00415 | ubiquinol cytochrome c reductase, subunit QCR2 | um06231 | 1.70E-06 | ni | conserved hypothetical protein |
| 12c00036 | 0.00042 | K03650 | mitochondrial GTPase | um05103 | 1.70E-06 | K00958 | probable sulfate adenylyltransferase |
| 27c00016 | 0.00043 | ni | hypothetical protein | um04967 | 1.71E-06 | K00615 | probable Transketolase |
| 27d00085 | 0.00044 | ni | predicted transporter/transmembrane protein | um11425 | 1.74E-06 | K01433 | related to Formyltetrahydrofolate deformylase |
| 22c00166 | 0.00044 | ni | peroxisomal multifunctional beta-oxidation protein and related enz | um00441 | 1.75E-06 | ni | hypothetical protein |
| 9d00030 | 0.00044 | ni | predicted transporter | um11284 | 1.79E-06 | ni | related to glycogenin-2 beta |
| 12c00048 | 0.00044 | ni | hypothetical protein | um15084 | 1.84E-06 | ni | conserved hypothetical protein |
| 18c00048 | 0.00045 | ni | ubiquitin-specific protease | um05591 | 2.01E-06 | ni | hypothetical protein |
| 9d00416 | 0.00047 | ni | hypothetical protein | um15100 | 2.01E-06 | ni | putative protein |
| 5d00063 | 0.00049 | ni | hypothetical protein | um05983 | 2.02E-06 | ni | conserved hypothetical protein |
| 9c00144 | 0.00049 | ni | adenylate cyclase-associated protein | um03678 | 2.05E-06 | ni | conserved hypothetical protein |
| 10c00076 | 0.00049 | ni | hypothetical protein | um00118 | 2.05E-06 | K00012 | probable UDP-glucose 6-dehydrogenase |
| 5c00003 | 0.00049 | ni | hypothetical protein | um04899 | 2.05E-06 | K00133 | aspartate semialdehyde dehydrogenase |
| 19c00044 | 0.00049 | K14559 | U3 small nucleolar ribonucleoprotein (snoRNP) subunit | um05786.2 | 2.08E-06 | K15272 | related to UDP N-acetylglucosamine transporter |
| 9c00385 | 0.0005 | ni | hypothetical protein | um02813 | 2.10E-06 | ni | conserved hypothetical Ustilago-specific protein |

| | | | | | | | |
|---|---|---|---|---|---|---|---|
| 7c00348 | 0.0005 | K00235 | succinate dehydrogenase, Fe-S protein subunit | um11338 | 2.21E-06 | ni | conserved hypothetical protein |
| 20d00079 | 0.00051 | ni | hypothetical protein | um03034 | 2.28E-06 | ni | conserved hypothetical protein |
| 22c00313 | 0.00053 | ni | hydroxymethylglutaryl-coa lyase | um11921 | 2.28E-06 | ni | related to metallothionein |
| 9d00259 | 0.00053 | ni | hypothetical protein | um01976 | 2.40E-06 | ni | putative protein |
| 7c00151 | 0.00053 | K00729 | glycosyltransferase | um00492 | 2.42E-06 | ni | conserved hypothetical protein |
| 14c00116 | 0.00053 | ni | ni | um00122 | 2.43E-06 | ni | conserved hypothetical protein |
| 5c00021 | 0.00054 | ni | hypothetical protein | um00343 | 2.47E-06 | K16261 | probable GAP1 - amino acid transport protein |
| 9d00323 | 0.00054 | ni | protein involved in Snf1 protein kinase complex assembly | um05878 | 2.48E-06 | ni | putative protein |
| 7c00018 | 0.00054 | K04077 | mitochondrial chaperonin, Cpn60/Hsp60p | um12262 | 2.48E-06 | ni | related to 4-coumarate-CoA ligase |
| 12d00105 | 0.00055 | ni | predicted Rho/Rac guanine nucleotide exchange factor | um02642 | 2.49E-06 | K07078 | conserved hypothetical protein |
| 8d00054 | 0.00055 | ni | predicted dehydrogenase | um05690 | 2.50E-06 | ni | conserved hypothetical Ustilago-specific protein |
| 9c00095 | 0.00055 | ni | hypothetical protein | um02994 | 2.51E-06 | ni | related to ELAV-like protein 2 |
| 19c00009 | 0.00055 | K01803 | triosephosphate isomerase | um12310 | 2.64E-06 | ni | hypothetical protein |
| 14c00069 | 0.00056 | ni | hypothetical protein | um01722 | 2.64E-06 | ni | conserved hypothetical protein |
| 10d00053 | 0.00057 | ni | hypothetical protein | um10143 | 2.66E-06 | K03120 | probable TATA-box-binding factor TBP |
| 16c00050 | 0.00058 | K03017 | RNA polymerase II subunit 9 | um03114 | 2.68E-06 | ni | conserved hypothetical protein |
| 22c00307 | 0.00058 | ni | hypothetical protein | um01567 | 2.71E-06 | ni | related to 2 |
| 1c00047 | 0.00059 | K02138 | mitochondrial F1F0-ATP synthase, subunit d/ATP7 | um01911 | 2.78E-06 | ni | related to alcohol dehydrogenase |
| 3d00036 | 0.00059 | ni | predicted E3 ubiquitin ligase | um06467 | 2.81E-06 | ni | related to glucosyltransferase |
| 5c00141 | 0.0006 | ni | hypothetical protein | um02652 | 2.88E-06 | ni | putative protein |
| 9d00320 | 0.0006 | K07943 | GTP-binding ADP-ribosylation factor-like protein ARL2 | um00606 | 2.95E-06 | ni | putative protein |
| 21d00003 | 0.00061 | ni | hypothetical protein | um05982 | 3.00E-06 | ni | hypothetical protein |
| 10c00097 | 0.00061 | ni | hypothetical protein | um10072 | 3.10E-06 | K08139 | probable monosaccharide transporter |
| 10c00096 | 0.00061 | ni | hypothetical protein | um15045 | 3.20E-06 | K10413 | cytoplasmic dynein heavy chain 1 |
| 12c00002 | 0.00061 | K07124 | 17 beta-hydroxysteroid dehydrogenase type 3 | um01989 | 3.25E-06 | ni | conserved hypothetical protein |
| 26d00085 | 0.00061 | K10669 | uncharacterized conserved protein | um10682 | 3.25E-06 | K00101 | probable CYB2 - L-lactate dehydrogenase |
| 15c00019 | 0.00063 | K04802 | DNA polymerase delta processivity factor | um02197 | 3.26E-06 | ni | conserved hypothetical protein |
| 20d00023 | 0.00064 | ni | hypothetical protein | um11111 | 3.27E-06 | ni | related to Ascorbate oxidase precursor |
| 5c00038 | 0.00065 | ni | ferric reductase | um05951 | 3.32E-06 | ni | conserved hypothetical protein |
| 2c00034 | 0.00066 | ni | hypothetical protein | um11166.2 | 3.33E-06 | ni | conserved hypothetical protein |
| 27d00049 | 0.00067 | K10994 | checkpoint 9-1-1 complex, RAD9 component | um10055 | 3.34E-06 | K00681 | related to gamma-glutamyltransferase |
| 22d00206 | 0.00067 | ni | 2-enoyl-CoA hydratase | um00283 | 3.42E-06 | K00077 | conserved hypothetical protein |
| 9d00053 | 0.00068 | K03509 | protein involved in establishing cohesion between sister chromatic | um04538 | 3.44E-06 | ni | related to DAL5 - Allantoate and ureidosuccinate permease |
| 22c00013 | 0.00068 | ni | short-chain acyl-CoA dehydrogenase | um01204 | 3.46E-06 | ni | related to PRY1 - strong similarity to the plant PR-1 class of pathogen related proteins |
| 18d00017 | 0.00068 | K08736 | mismatch repair MSH3 | um03016 | 3.53E-06 | ni | conserved hypothetical protein |
| 8c00022 | 0.0007 | K16948 | septin family protein | um11186 | 3.57E-06 | ni | putative protein |
| 27c00055 | 0.00072 | ni | hypothetical protein | um01411.2 | 3.64E-06 | ni | putative protein |
| 22d00032 | 0.00072 | K05355 | geranylgeranyl pyrophosphate synthase | um10031 | 3.68E-06 | ni | related to embryonic protein DC-8 |
| 14d00061 | 0.00074 | ni | glucose dehydrogenase | um02753 | 3.71E-06 | ni | conserved hypothetical protein |
| 3c00041 | 0.00074 | ni | hypothetical protein | um01868 | 3.77E-06 | ni | related to GIT1 - Glycerophosphoinositol transporter also able to mediate low-affinity phosphate transport |
| 19d00094 | 0.00075 | ni | hypothetical protein | um11185 | 3.78E-06 | ni | conserved hypothetical protein |
| 20c00003 | 0.00075 | ni | permease of the major facilitator superfamily | um04060 | 3.85E-06 | ni | probable DIC1 - mitochondrial dicarboxylate carrier |
| 12c00112 | 0.00076 | K03021 | RNA polymerase III, second largest subunit | um15025 | 3.89E-06 | ni | probable NADP-dependent alcohol dehydrogenase |
| 3c00024 | 0.00076 | K14401 | mRNA cleavage and polyadenylation factor II complex, subunit CI | um04306 | 3.89E-06 | ni | putative protein |
| 7d00009 | 0.00077 | ni | acyl-coa synthetase | um05966 | 3.90E-06 | ni | conserved hypothetical protein |
| 6c00064 | 0.00077 | ni | flavonol reductase | um10443.2 | 3.95E-06 | ni | conserved hypothetical protein |
| 14c00067 | 0.00078 | K01408 | N-arginine dibasic convertase NRD1 and related Zn2+-dependen | um02565 | 3.95E-06 | ni | conserved hypothetical protein |
| 8c00082 | 0.00078 | ni | hypothetical protein | um04506 | 3.97E-06 | ni | hypothetical protein |
| 11c00069 | 0.00079 | K03260 | translation initiation factor 4F, ribosome/mRNA-bridging subunit | um12026 | 4.03E-06 | ni | related to TMT1 - trans-aconitate methyltransferase |
| 16c00041 | 0.00079 | ni | hypothetical protein | um00663 | 4.03E-06 | K10591 | probable ubiquitin-protein ligase |
| 26c00001 | 0.00079 | ni | hypothetical protein | um05705 | 4.06E-06 | ni | putative protein |
| 9d00113 | 0.00079 | K07734 | hypothetical protein | um02978 | 4.21E-06 | ni | conserved hypothetical protein |
| 27c00024 | 0.0008 | ni | hypothetical protein | um06056 | 4.23E-06 | ni | putative protein |
| 7c00158 | 0.0008 | ni | inositol polyphosphate 5-phosphatase and related proteins | um02413 | 4.38E-06 | ni | conserved hypothetical protein |
| 19c00115 | 0.00081 | K08508 | SNAP-25 (synaptosome-associated protein) component of SNAF | um05046 | 4.51E-06 | ni | conserved hypothetical protein |
| 22d00037 | 0.00082 | K09704 | hypothetical protein | um02836 | 4.52E-06 | ni | hypothetical protein |
| 22d00063 | 0.00083 | ni | hypothetical protein | um04553 | 4.61E-06 | ni | related to growth hormone inducible transmembrane protein |
| 19d00097 | 0.00083 | K09531 | molecular chaperone | um00116 | 4.64E-06 | ni | related to nicotinamide mononucleotide permease |
| 7d00100 | 0.00088 | K03008 | RNA polymerase, subunit L | um00197 | 4.65E-06 | K15441 | related to TAD2 - tRNA-specific adenosine deaminase 2 |
| 22c00263 | 0.00089 | K01489 | cytidine deaminase | um11596 | 4.65E-06 | ni | related to CSR1 - phosphatidylinositol transfer protein |
| 13d00098 | 0.0009 | ni | cation transport ATPase | um11683 | 4.69E-06 | ni | conserved hypothetical protein |
| 10c00033 | 0.0009 | ni | hypothetical protein | um10665 | 4.69E-06 | ni | related to acyl-CoA dehydrogenase |
| 7c00185 | 0.00091 | ni | acyl-coa synthetase | um11187 | 4.72E-06 | ni | related to ROT1 - molecular chaperone in the endoplasmic reticulum |
| 20d00031 | 0.00092 | K01669 | deoxyribodipyrimidine photolyase | um10186 | 4.83E-06 | ni | putative protein |
| 26c00010 | 0.00093 | K01940 | argininosuccinate synthase | um10782 | 4.87E-06 | K16803 | related to STU2 - Microtubule-associated protein |
| 27c00038 | 0.00093 | ni | hypothetical protein | um03881 | 4.87E-06 | ni | related to Heat shock protein Hsp20 |
| 9d00003 | 0.00094 | ni | hypothetical protein | um11598 | 4.89E-06 | ni | related to oxidoreductase |
| 4d00041 | 0.00094 | K13335 | hypothetical protein | um03063 | 5.25E-06 | K15331 | related to TRM2 - tRNA |
| 7d00201 | 0.00094 | ni | hypothetical protein | um12295 | 5.26E-06 | ni | putative protein |
| 19d00073 | 0.00095 | ni | hypothetical protein | um11391 | 5.53E-06 | ni | conserved hypothetical protein |
| 22d00070 | 0.00095 | ni | uncharacterized conserved protein | um03002 | 5.61E-06 | ni | putative protein |
| 3d00085 | 0.001 | K16196 | eIF-2alpha kinase GCN2 | um03417 | 5.73E-06 | ni | putative acetamidase |
| 9c00089 | 0.00101 | ni | hypothetical protein | um11817 | 5.91E-06 | K13124 | conserved hypothetical protein |
| 9d00251 | 0.00102 | K01280 | tripeptidyl peptidase II | um01378 | 6.38E-06 | ni | conserved hypothetical protein |
| 9d00022 | 0.00102 | ni | synaptic vesicle transporter SVOP and related transporters | um02430 | 6.42E-06 | ni | conserved hypothetical protein |
| 7c00174 | 0.00103 | K05727 | signaling protein DOCK180 | um05802 | 6.52E-06 | ni | related to cell surface ferroxidase |
| 9c00132 | 0.00104 | ni | hypothetical protein | um11296.2 | 6.54E-06 | ni | related to calmodulin-dependent protein kinase |
| 3c00018 | 0.00107 | ni | hypothetical protein | um15048 | 6.56E-06 | ni | related to 4-coumarate-CoA ligase 1 |
| 24c00051 | 0.00107 | ni | component of vacuolar transporter chaperone | um00679 | 6.65E-06 | ni | related to VBA1 - Vacuolar Basic Amino acid transporter |
| 15d00084 | 0.00109 | ni | reductases with broad range of substrate specificities | um11544 | 6.67E-06 | ni | putative protein |
| 4d00017 | 0.0011 | ni | hypothetical protein | um01424 | 6.70E-06 | K10437 | probable Cytochrome P450 monooxygenase |
| 13d00061 | 0.00112 | ni | aldo/keto reductase family proteins | um11967 | 6.83E-06 | ni | conserved hypothetical protein |
| 3c00013 | 0.00116 | ni | hypothetical protein | um03658 | 7.01E-06 | ni | conserved hypothetical protein |
| 18d00041 | 0.00117 | K15192 | SNF2 family DNA-dependent ATPase domain-containing protein | um06508 | 7.02E-06 | ni | conserved hypothetical protein |
| 12d00006 | 0.00119 | K10437 | cytochrome P450 CYP2 subfamily | um00992 | 7.13E-06 | K01104 | related to protein tyrosine phosphatase PPS1 |
| 22d00091 | 0.00121 | ni | hypothetical protein | um05676 | 7.23E-06 | ni | related to 1-aminocyclopropane-1-carboxylate synthase 1 |
| 12d00005 | 0.00123 | ni | hypothetical protein | um03067 | 7.32E-06 | ni | related to CDC33 - translation initiation factor eIF4E |
| 15d00076 | 0.00124 | ni | hypothetical protein | um05549 | 7.41E-06 | K01858 | probable myo-inositol 1-phosphate synthase |
| 14c00035 | 0.00124 | ni | hypothetical protein | um10234.2 | 7.76E-06 | ni | related to GLE1 - RNA export mediator |
| 24d00013 | 0.00126 | K12619 | 5'-3' exonuclease HKE1/RAT1 | um06059 | 7.84E-06 | ni | conserved hypothetical protein |
| 18c00065 | 0.00128 | K07407 | alpha-d-galactosidase | um11795 | 7.92E-06 | K00799 | related to glutathione s-transferase 3 |
| 9c00423 | 0.00129 | K00624 | carnitine O-acyltransferase CRAT | um04695 | 7.98E-06 | ni | probable HNM1 - Choline permease |
| 22c00111 | 0.0013 | K03363 | anaphase promoting complex, Cdc20, Cdh1, and Ama1 subunits | um05080 | 7.99E-06 | ni | hypothetical protein |
| 15c00059 | 0.0013 | ni | beta tubulin | um03281 | 8.04E-06 | ni | putative protein |
| 10c00004 | 0.00131 | K07901 | GTP-binding protein SEC4 | um01025 | 8.14E-06 | ni | conserved hypothetical protein |
| 15d00014 | 0.00134 | ni | predicted transporter | um01787 | 8.27E-06 | K00547 | related to homocysteine S-methyltransferase |
| 19d00098 | 0.00137 | ni | hypothetical protein | um05286.2 | 8.46E-06 | ni | related to GS1 protein |
| 19d00127 | 0.00137 | K01681 | aconitase/homoaconitase | um10618 | 8.47E-06 | ni | conserved hypothetical Ustilago-specific protein |
| 9d00198 | 0.00138 | K00008 | sorbitol dehydrogenase | um15051 | 8.55E-06 | ni | conserved hypothetical protein |
| 10c00103 | 0.00139 | ni | sphingolipid fatty acid hydroxylase | um06406 | 8.55E-06 | K05770 | related to Peripheral-type benzodiazepine receptor |
| 13c00022 | 0.0014 | ni | predicted membrane protein | um01970 | 8.66E-06 | ni | hypothetical protein |
| 5c00104 | 0.00141 | K01166 | ribonuclease | um02954 | 8.93E-06 | ni | putative protein |
| 22c00286 | 0.00142 | K01230 | mannosyl-oligosaccharide alpha-1,2-mannosidase | um04930 | 9.03E-06 | K00036 | probable ZWF1 - glucose-6-phosphate dehydrogenase |
| 9c00135 | 0.00144 | ni | flavin-containing monooxygenase | um11285 | 9.08E-06 | ni | conserved hypothetical protein |
| 25d00020 | 0.00145 | ni | hypothetical protein | um00595 | 9.40E-06 | K14455 | probable aspartate aminotransferase |
| 5d00070 | 0.00148 | K01027 | succinyl-coa, alpha-ketoacid-coa transferase | um04063 | 9.50E-06 | K08726 | related to soluble epoxide hydrolase |
| 3c00051 | 0.00149 | ni | hypothetical protein | um00696 | 9.65E-06 | ni | conserved hypothetical protein |
| 12d00133 | 0.0015 | K07975 | ras-related small GTPase | um11741 | 9.81E-06 | ni | related to GCY1 - galactose-induced protein of aldo |
| 6d00105 | 0.0015 | ni | predicted lipase | um00945 | 9.84E-06 | K12659 | probable ARG6 - n-acetyl-gamma-glutamyl-phosphate reductase |
| 26d00033 | 0.00151 | K02908 | 60S ribosomal protein L30 | um02708 | 9.95E-06 | K08738 | probable CYTOCHROME C |
| 9d00221 | 0.00154 | ni | hypothetical protein | um02521 | 1.01E-05 | ni | conserved hypothetical protein |
| 6c00022 | 0.00155 | ni | alcohol dehydrogenase | um01899 | 1.03E-05 | ni | related to multidrug resistance protein |
| 7c00053 | 0.00155 | K01669 | deoxyribodipyrimidine photolyase | um11720 | 1.03E-05 | ni | related to cystathionine beta-lyase |
| 10d00096 | 0.00156 | K01187 | alpha-amylase | um03229 | 1.07E-05 | ni | conserved hypothetical protein |
| 6c00078 | 0.00157 | ni | hypothetical protein | um01382 | 1.07E-05 | ni | putative protein |
| 20c00063 | 0.00158 | K14654 | hypothetical protein | um04114 | 1.07E-05 | K01077 | probable PHO8 - repressible alkaline phosphatase vacuolar |
| 22c00243 | 0.00159 | ni | hypothetical protein | um10494 | 1.09E-05 | K01230 | related to Mannosyl-oligosaccharide 1 |
| 10d00097 | 0.00159 | K03293 | amino acid transporters | um05344 | 1.09E-05 | ni | conserved hypothetical protein |
| 13c00054 | 0.00162 | K14152 | histidinol dehydrogenase | um11516 | 1.10E-05 | ni | conserved hypothetical protein |
| 22d00182 | 0.00167 | ni | glyoxylase | um03713 | 1.12E-05 | ni | putative protein |
| 3d00017 | 0.00167 | ni | COPII vesicle protein | um00455 | 1.12E-05 | ni | related to MIR1 - Phosphate transporter of the mitochondrial carrier |
| 7d00004 | 0.00167 | ni | ferredoxin | um12267 | 1.14E-05 | ni | related to IST2 - Plasma membrane protein that may be involved in osmotolerance |
| 8d00024 | 0.00168 | ni | protein transporter | um03753 | 1.14E-05 | ni | conserved hypothetical Ustilago-specific protein |
| 14c00002 | 0.00171 | ni | synaptic vesicle transporter SVOP and related transporters | um06266 | 1.15E-05 | ni | putative protein |
| 6c00113 | 0.00173 | K02947 | 40s ribosomal protein s10 | um00946 | 1.17E-05 | ni | putative protein |
| 10d00044 | 0.00174 | K11362 | hypothetical protein | um10912 | 1.17E-05 | ni | conserved hypothetcial protein |
| 9c00406 | 0.00174 | ni | hypothetical protein | um02641 | 1.19E-05 | K13346 | related to PEX10 - peroxisomal assembly protein - peroxin |
| 19c00099 | 0.00176 | K00381 | sulfite reductase | um02097 | 1.20E-05 | ni | related to enoyl-CoA hydratase |
| 6c00106 | 0.00178 | ni | hypothetical protein | um04738 | 1.21E-05 | K01262 | related to X-Pro aminopeptidase II |
| 9c00384 | 0.0018 | ni | hypothetical protein | um05362 | 1.21E-05 | K01641 | probable hydroxymethylglutaryl-CoA synthase |
| 25d00006 | 0.0018 | K12879 | KEKE-like motif-containing transcription regulator | um05805.2 | 1.23E-05 | ni | conserved hypothetical protein |
| 13c00058 | 0.00182 | K01810 | glucose-6-phosphate isomerase | um11382 | 1.26E-05 | ni | probable 2-Nitropropane dioxygenase |
| 9d00056 | 0.00186 | K01176 | alpha-amylase | um04000 | 1.26E-05 | ni | DNA binding protein Ncp1 |
| 7d00238 | 0.00192 | K12618 | 5'-3' exonuclease XRN1/KEM1/SEP1 | um03168.2 | 1.27E-05 | ni | putative protein |
| 19d00006 | 0.00194 | ni | hypothetical protein | um00236 | 1.28E-05 | ni | hypothetical protein |
| 9c00139 | 0.00194 | K01210 | hypothetical protein | um04776 | 1.29E-05 | K02727 | probable PRE10 - 20S proteasome subunit C1 |
| 5c00115 | 0.00197 | K06670 | sister chromatid cohesion complex Cohesin, subunit RAD21/SCC | um02579 | 1.31E-05 | K10739 | related to RFA2 - DNA replication factor A |
| 5c00088 | 0.00197 | K02267 | cytochrome c oxidase, subunit VIb/COX12 | um01871 | 1.32E-05 | ni | conserved hypothetical protein |
| 27c00092 | 0.00204 | ni | hypothetical protein | um06120 | 1.35E-05 | ni | conserved hypothetical protein |
| 2c00080 | 0.00205 | ni | hypothetical protein | um12313 | 1.37E-05 | ni | conserved hypothetical protein |
| 22d00154 | 0.00205 | ni | multidrug resistance-associated protein | um10010 | 1.37E-05 | ni | related to EMI5 - protein required for transcriptional induction of TF IME1 |
| 2d00037 | 0.00206 | ni | conserved phosducin-like protein | um01861 | 1.39E-05 | K00002 | related to alcohol dehydrogenase |
| 11d00056 | 0.00212 | ni | uncharacterized membrane protein | um11409 | 1.43E-05 | K00850 | probable 6-phosphofructokinase |
| 9c00226 | 0.00213 | ni | isocitrate dehydrogenase, alpha subunit | um05431 | 1.45E-05 | ni | conserved hypothetical protein |
| 27c00050 | 0.00215 | ni | hypothetical protein | um01882 | 1.48E-05 | ni | related to multidrug resistance proteins |
| 1c00022 | 0.00215 | K02259 | cytochrome oxidase assembly factor COX15 | um05880 | 1.49E-05 | K07750 | probable ERG25 - C-4 methyl sterol oxidase |
| 5c00155 | 0.00215 | K04640 | G-protein alpha subunit | um01750 | 1.50E-05 | ni | conserved hypothetical protein |
| 12d00135 | 0.00217 | ni | hypothetical protein | um15059 | 1.50E-05 | ni | conserved hypothetical protein |
| 27c00008 | 0.00217 | ni | hypothetical protein | um11025 | 1.51E-05 | ni | conserved hypothetical protein |
| 9c00180 | 0.00217 | ni | lecithin, cholesterol acyltransferase | um04550 | 1.51E-05 | ni | conserved hypothetical protein |
| 7c00013 | 0.00218 | K07300 | Ca2+/H+ antiporter VCX1 | um06509 | 1.56E-05 | ni | related to Phenylalanine ammonia-lyase |
| 19d00102 | 0.00218 | ni | hypothetical protein | um10073 | 1.57E-05 | ni | related to lincomycin-condensing protein lmbA |
| 22d00077 | 0.00222 | ni | hypothetical protein | um04105 | 1.57E-05 | ni | related to Polyketide synthase |
| 9d00239 | 0.00225 | ni | hypothetical protein | um11243 | 1.58E-05 | K13628 | related to ISA1 - Fe |
| 22c00289 | 0.00228 | ni | hypothetical protein | um00020 | 1.60E-05 | ni | related to l-azetidine-2-carboxylic acid acetyltransferase |
| 25c00071 | 0.0023 | ni | hypothetical protein | um10183 | 1.60E-05 | K13237 | related to SPS19 - peroxisomal 2 |

| | | | | | | | |
|---|---|---|---|---|---|---|---|
| 5c00136 | 0.0023 | ni | hypothetical protein | um01708 | 1.63E-05 | K00003 | probable HOM6 - homoserine dehydrogenase |
| 22d00196 | 0.00231 | ni | predicted hydrolase | um05346 | 1.64E-05 | ni | conserved hypothetical protein |
| 10d00003 | 0.00232 | ni | multidrug resistance-associated protein | um04727 | 1.65E-05 | K17387 | related to Kinesin-like protein KIF23 |
| 7d00168 | 0.00233 | ni | plasma membrane H+-transporting ATPase | um12283 | 1.66E-05 | ni | conserved hypothetical protein |
| 3d00029 | 0.00233 | K14793 | U3 snoRNP-associated protein | um00797 | 1.70E-05 | K00856 | probable adenosine kinase |
| 3d00054 | 0.00238 | ni | uncharacterized conserved protein | um03246 | 1.70E-05 | ni | related to versicolorin b synthase |
| 22c00315 | 0.00239 | ni | permease of the major facilitator superfamily | um00311 | 1.70E-05 | K01953 | probable asparagine synthase |
| 14d00121 | 0.00243 | ni | hypothetical protein | um02203 | 1.71E-05 | ni | related to Na |
| 9d00154 | 0.00245 | K15541 | predicted E3 ubiquitin ligase | um11373 | 1.71E-05 | ni | conserved hypothetical protein |
| 22c00006 | 0.00246 | ni | Ca2+/calmodulin-dependent protein kinase kinase beta | um11374 | 1.72E-05 | ni | conserved hypothetical protein |
| 16d00025 | 0.00246 | ni | hypothetical protein | um11859 | 1.74E-05 | K01693 | probable HIS3 - imidazoleglycerol-phosphate dehydratase |
| 8d00073 | 0.0025 | K11275 | hypothetical protein | um01665 | 1.75E-05 | ni | hypothetical protein |
| 7c00154 | 0.0025 | ni | uncharacterized conserved protein | um10052 | 1.76E-05 | ni | conserved hypothetical protein |
| 5d00045 | 0.0025 | K11644 | histone deacetylase complex, SIN3 component | um02035 | 1.83E-05 | ni | related to yellowish-green 1 |
| 20c00041 | 0.00251 | ni | hypothetical protein | um03897 | 1.87E-05 | ni | related to OCA1 - Putative protein tyrosine phosphatase |
| 15c00086 | 0.00252 | ni | pleiotropic drug resistance proteins | um05933 | 1.89E-05 | K14708 | probable Sulfate permease |
| 7c00311 | 0.00252 | ni | hypothetical protein | um01650 | 1.90E-05 | ni | conserved hypothetical protein |
| 16d00041 | 0.00255 | ni | hypothetical protein | um01783 | 1.96E-05 | ni | related to Inosine-uridine preferring nucleoside hydrolase |
| 22c00222 | 0.00257 | ni | hypothetical protein | um04834 | 1.97E-05 | ni | conserved hypothetical protein |
| 26d00012 | 0.00258 | ni | hypothetical protein | um06476 | 2.03E-05 | ni | conserved hypothetical protein |
| 5c00030 | 0.0026 | ni | NDR and related serine/threonine kinases | um01894 | 2.03E-05 | ni | putative protein |
| 22d00212 | 0.00263 | K00002 | alcohol dehydrogenase | um11034 | 2.12E-05 | ni | putative protein |
| 13c00025 | 0.00264 | K04646 | vesicle coat protein clathrin | um06278 | 2.15E-05 | ni | conserved hypothetical protein |
| 22d00065 | 0.00265 | ni | hypothetical protein | um05961 | 2.18E-05 | K01796 | probable alpha-methylacyl-coa racemase |
| 9d00171 | 0.00266 | K11514 | hypothetical protein | um00547 | 2.20E-05 | K00286 | probable PRO3 - delta 1-pyrroline-5-carboxylate reductase |
| 14d00110 | 0.00267 | K00819 | ornithine aminotransferase | um01513 | 2.22E-05 | ni | putative protein |
| 9d00130 | 0.00267 | ni | hypothetical protein | um12330 | 2.25E-05 | ni | hypothetical protein |
| 22c00196 | 0.00267 | K14265 | aromatic amino acid aminotransferase and related proteins | um05718 | 2.26E-05 | ni | related to KRE6 - Beta-glucan synthase subunit |
| 22c00212 | 0.00268 | ni | hypothetical protein | um00486 | 2.26E-05 | K01835 | probable PGM2 - phosphoglucomutase |
| 20c00052 | 0.00271 | ni | hypothetical protein | um11022 | 2.27E-05 | ni | related to SRY1 - 3-hydroxyaspartate dehydratase |
| 22c00164 | 0.00276 | ni | hypothetical protein | um05450 | 2.28E-05 | K01897 | related to Long-chain-fatty-acid--CoA ligase 6 |
| 12c00139 | 0.00277 | ni | hypothetical protein | um02942 | 2.32E-05 | K00390 | probable MET16 - 3 |
| 16c00017 | 0.00279 | K09554 | cell division cycle 37 protein | um01626 | 2.33E-05 | ni | conserved hypothetical protein |
| 27c00036 | 0.0028 | K00759 | adenine phosphoribosyl transferases | um01771 | 2.34E-05 | K06929 | conserved hypothetical protein |
| 27c00088 | 0.00282 | ni | hypothetical protein | um05130 | 2.34E-05 | K01610 | probable phosphoenolpyruvate carboxykinase |
| 9c00036 | 0.00283 | ni | D-arabinono-1, 4-lactone oxidase | um10413 | 2.36E-05 | ni | hypothetical protein |
| 16d00038 | 0.00284 | ni | hypothetical protein | um04794 | 2.37E-05 | K03124 | probable SUA7 - TFIIB subunit |
| 12d00082 | 0.00285 | K13501 | phosphoribosylanthranilate isomerase | um00750 | 2.38E-05 | ni | putative protein |
| 19c00072 | 0.00287 | ni | hypothetical protein | um00727 | 2.40E-05 | ni | putative protein |
| 7c00293 | 0.00289 | ni | hypothetical protein | um02610 | 2.42E-05 | ni | conserved hypothetical protein |
| 7d00132 | 0.00291 | ni | hypothetical protein | um03924 | 2.44E-05 | ni | repellent protein 1 precursor |
| 13d00006 | 0.00292 | ni | hypothetical protein | um04014 | 2.47E-05 | ni | putative protein |
| 18d00013 | 0.00297 | K03010 | RNA polymerase II, second largest subunit | um10664 | 2.51E-05 | ni | conserved hypothetical protein |
| 3c00039 | 0.00298 | ni | hypothetical protein | um04333 | 2.58E-05 | ni | putative protein |
| 11c00088 | 0.00301 | ni | acyl-coa synthetase | um15014 | 2.61E-05 | ni | related to KAP123 - Importin beta-4 subunit |
| 5c00150 | 0.00302 | K05841 | UDP-glucuronosyl and UDP-glucosyl transferase | um01697 | 2.62E-05 | K00143 | probable LYS2 - L-aminoadipate-semialdehyde dehydrogenase |
| 25c00019 | 0.00306 | ni | hypothetical protein | um03860 | 2.62E-05 | ni | related to pepsinogen precursor |
| 9c00427 | 0.00306 | K00856 | possible pfkB family carbohydrate kinase | um11266 | 2.65E-05 | K13333 | probable lysophospholipase |
| 2c00063 | 0.00308 | K03506 | CCAAT-binding factor, subunit C | um06454 | 2.66E-05 | ni | related to Mannose-6-phosphate isomerase |
| 7d00095 | 0.0031 | ni | uncharacterized conserved protein | um10801 | 2.68E-05 | ni | conserved hypothetical protein |
| 3c00012 | 0.00312 | ni | hypothetical protein | um01425 | 2.69E-05 | K00451 | probable homogentisate 1 |
| 1d00022 | 0.00312 | ni | hypothetical protein | um11345 | 2.72E-05 | ni | conserved hypothetical protein |
| 27c00085 | 0.00314 | ni | AAA+-type ATPase containing the bromodomain | um11170 | 2.72E-05 | K03942 | probable NADH2 dehydrogenase |
| 5d00091 | 0.00317 | ni | hypothetical protein | um01917 | 2.73E-05 | ni | hypothetical protein |
| 7c00316 | 0.00322 | ni | hypothetical protein | um05944 | 2.74E-05 | ni | hypothetical protein |
| 22c00080 | 0.00324 | ni | predicted membrane protein | um00545 | 2.77E-05 | ni | conserved hypothetical protein |
| 27c00054 | 0.00325 | K10415 | cytoplasmic dynein intermediate chain | um05249 | 2.83E-05 | ni | conserved hypothetical protein |
| 9c00346 | 0.00326 | ni | mitochondrial inner membrane protein | um02008 | 2.85E-05 | ni | conserved hypothetical protein |
| 4d00001 | 0.00327 | ni | diphthamide biosynthesis protein | um03199 | 2.90E-05 | K00820 | probable GFA1 - glucosamine--fructose-6-phosphate transaminase |
| 25c00014 | 0.00329 | ni | hypothetical protein | um04322 | 2.91E-05 | ni | conserved hypothetical protein |
| 24d00051 | 0.00329 | ni | hypothetical protein | um11798 | 2.94E-05 | ni | conserved hypothetical protein |
| 16c00045 | 0.00331 | K08504 | hypothetical protein | um00985 | 2.99E-05 | ni | ni |
| 7d00136 | 0.00334 | ni | glia maturation factor beta | um04154 | 3.06E-05 | ni | conserved hypothetical protein |
| 7c00140 | 0.00335 | ni | hypothetical protein | um10721 | 3.13E-05 | ni | putative protein |
| 5c00020 | 0.00337 | ni | aldehyde dehydrogenase | um11065 | 3.14E-05 | ni | conserved hypothetical protein |
| 6c00091 | 0.00338 | ni | hypothetical protein | um10705 | 3.14E-05 | K04563 | cyclin-dependent kinase 1 |
| 24c00009 | 0.00339 | ni | hypothetical protein | um12336.2 | 3.15E-05 | K08740 | related to meiosis-specific MutS homolog |
| 6c00043 | 0.0034 | ni | hypothetical protein | um02935.2 | 3.15E-05 | K11270 | hypothetical protein |
| 7d00119 | 0.00341 | K03109 | hypothetical protein | um05183 | 3.19E-05 | K03122 | related to TOA1 - transcription factor TFIIA-L |
| 22d00184 | 0.00351 | ni | acetyl-coa transporter | um01800 | 3.25E-05 | K03635 | related to molybdopterin synthase large subunit |
| 8c00067 | 0.00352 | K02995 | 40S ribosomal protein S8 | um05114 | 3.27E-05 | ni | related to multidrug resistance protein 4 |
| 9d00406 | 0.00359 | ni | inorganic phosphate transporter | um00672 | 3.30E-05 | K00365 | probable urate oxidase |
| 5d00131 | 0.00362 | ni | hypothetical protein | um01431 | 3.32E-05 | ni | ni |
| 9c00140 | 0.00363 | K11233 | sensory transduction histidine kinase | um00636 | 3.35E-05 | ni | hypothetical protein |
| 11d00022 | 0.00364 | K01531 | Ca2+ transporting ATPase | um02835 | 3.37E-05 | K09448 | conserved hypothetical protein |
| 8d00070 | 0.00366 | ni | predicted ATPase | um00748 | 3.37E-05 | K12832 | probable Splicing factor 3B subunit 5 |
| 9d00357 | 0.00368 | K06698 | proteasome activator subunit | um10263 | 3.45E-05 | ni | related to 1-phosphatidylinositol-4 |
| 9d00013 | 0.00372 | ni | hypothetical protein | um01640 | 3.53E-05 | ni | related to GAS1 - glycophospholipid-anchored surface glycoprotein |
| 5d00118 | 0.00373 | ni | zinc-binding oxidoreductase | um04671 | 3.54E-05 | ni | conserved hypothetical protein |
| 25c00005 | 0.00375 | ni | hypothetical protein | um11191 | 3.64E-05 | ni | related to phenylacetyl-CoA ligase |
| 8c00051 | 0.00376 | ni | hypothetical protein | um01933 | 3.67E-05 | ni | putative protein |
| 10d00102 | 0.00377 | K01001 | glycosyltransferase | um01136 | 3.67E-05 | ni | conserved hypothetical protein |
| 8d00021 | 0.00379 | K01193 | beta-fructofuranosidase | um01070 | 3.68E-05 | ni | related to cyclopropane-fatty-acyl-phospholipid synthase |
| 12d00097 | 0.0038 | K01410 | metalloendopeptidase family - mitochondrial intermediate peptidas | um10104 | 3.72E-05 | ni | probable GCY1 - galactose-induced protein of aldo |
| 9d00007 | 0.00385 | K16330 | predicted carbohydrate kinase | um01847 | 3.73E-05 | ni | conserved hypothetical protein |
| 12c00021 | 0.00387 | ni | hypothetical protein | um12011 | 3.75E-05 | ni | putative protein |
| 16c00080 | 0.00389 | ni | hypothetical protein | um02119 | 3.78E-05 | ni | hypothetical protein |
| 22c00292 | 0.00394 | ni | predicted transporter | um11133 | 3.81E-05 | ni | conserved hypothetical protein |
| 14d00039 | 0.00394 | K16572 | hypothetical protein | um02502 | 3.83E-05 | ni | conserved hypothetical protein |
| 13d00046 | 0.00394 | K09497 | chaperonin complex component, TCP-1 epsilon subunit | um11251 | 3.83E-05 | ni | putative protein |
| 16d00064 | 0.00395 | ni | hypothetical protein | um12169 | 3.90E-05 | K03317 | related to Sodium |
| 7d00355 | 0.00402 | ni | hypothetical protein | um01065 | 3.94E-05 | ni | hypothetical protein |
| 7c00220 | 0.00408 | ni | uncharacterized conserved protein | um00011 | 4.00E-05 | K01426 | probable AMD2 - amidase |
| 25d00049 | 0.00411 | K15040 | porin/voltage-dependent anion-selective channel protein | um03356 | 4.02E-05 | K01689 | probable ENO1 - enolase I |
| 6c00032 | 0.00414 | ni | acyl-coa synthetase | um02791 | 4.09E-05 | ni | related to GABA permease |
| 9c00227 | 0.00415 | ni | hypothetical protein | um03289 | 4.10E-05 | ni | hypothetical protein |
| 8c00059 | 0.00417 | K00276 | copper amine oxidase | um06440 | 4.11E-05 | ni | conserved hypothetical protein |
| 14d00082 | 0.00418 | K12627 | small Nuclear ribonucleoprotein splicing factor | um01292 | 4.12E-05 | ni | putative protein |
| 25d00058 | 0.00419 | K00550 | phospholipid methyltransferase | um04787 | 4.18E-05 | K01620 | related to GLY1 - L-threonine aldolase |
| 11d00063 | 0.00419 | ni | protein required for meiotic chromosome segregation | um02256 | 4.22E-05 | ni | related to Cingulin |
| 13c00084 | 0.00421 | ni | hypothetical protein | um00913 | 4.26E-05 | ni | glyoxaloxidase 2 |
| 3c00011 | 0.00423 | ni | choline kinase | um12198 | 4.42E-05 | ni | conserved hypothetical protein |
| 8c00122 | 0.0043 | ni | hypothetical protein | um03149 | 4.43E-05 | ni | related to molybdenum cofactor sulfurase |
| 2c00004 | 0.0043 | ni | hypothetical protein | um10390 | 4.47E-05 | ni | conserved hypothetical protein |
| 1d00049 | 0.00431 | ni | predicted esterase | um11346 | 4.50E-05 | ni | conserved hypothetical protein |
| 27d00040 | 0.00436 | ni | hypothetical protein | um11337.2 | 4.56E-05 | K01555 | related to fumarylacetoacetate hydrolase |
| 26d00006 | 0.00438 | K14572 | AAA ATPase containing von Willebrand factor type A | um05347 | 4.56E-05 | ni | conserved hypothetical protein |
| 9c00224 | 0.00439 | ni | hypothetical protein | um00463 | 4.58E-05 | K12597 | conserved hypothetical protein |
| 7d00234 | 0.0044 | K09291 | uncharacterized conserved coiled-coil protein | um00783 | 4.58E-05 | ni | related to Heme oxygenase |
| 7c00285 | 0.00443 | ni | predicted membrane protein | um00271 | 4.59E-05 | ni | related to Lipase |
| 3c00077 | 0.00444 | ni | hypothetical protein | um00623 | 4.61E-05 | ni | conserved hypothetical protein |
| 7d00282 | 0.0045 | ni | hypothetical protein | um03296 | 4.78E-05 | ni | hypothetical protein |
| 22c00009 | 0.00456 | ni | uncharacterized conserved membrane protein | um11258 | 4.79E-05 | K09250 | related to GIS2 - Putative zinc finger protein |
| 16c00072 | 0.00458 | ni | soluble epoxide hydrolase | um04578 | 4.82E-05 | ni | conserved hypothetical protein |
| 2c00039 | 0.00459 | K01952 | phosphoribosylformylglycinamidine synthase | um00117 | 4.82E-05 | ni | related to amidase |
| 8c00070 | 0.0046 | ni | hypothetical protein | um00177 | 4.87E-05 | ni | conserved hypothetical protein |
| 19d00087 | 0.00463 | K11315 | histone acetyltransferases PCAF/SAGA/ADA, subunit TADA3L/ | um04056 | 4.87E-05 | ni | probable SNQ2 - ABC transporter involved in multidrug resistance |
| 24d00022 | 0.00463 | ni | chitinase | um00754 | 4.90E-05 | ni | putative protein |
| 15c00056 | 0.00464 | ni | hypothetical protein | um00315 | 4.90E-05 | K01012 | probable biotin synthase |
| 19c00029 | 0.00469 | ni | hypothetical protein | um10976 | 5.02E-05 | ni | conserved hypothetical protein |
| 7d00175 | 0.0047 | ni | hypothetical protein | um10473 | 5.13E-05 | ni | conserved hypothetical protein |
| 5d00022 | 0.00471 | ni | protein kinase PCTAIRE and related kinases | um04109 | 5.14E-05 | ni | related to Cytochrome P450 |
| 12c00076 | 0.00473 | K06268 | Ca2+/calmodulin-dependent protein phosphatase | um04876 | 5.24E-05 | ni | hypothetical protein |
| 14c00106 | 0.00474 | ni | hypothetical protein | um02157 | 5.32E-05 | K00309 | conserved hypothetical protein |
| 22d00166 | 0.00475 | ni | predicted mitochondrial carrier protein | um10483 | 5.40E-05 | ni | conserved hypothetical protein |
| 27d00060 | 0.00475 | K12274 | molecular co-chaperone STI1 | um10945 | 5.41E-05 | ni | conserved hypothetical protein |
| 7d00129 | 0.00476 | K16055 | trehalose-6-phosphate synthase component TPS1 and related su | um11207 | 5.42E-05 | K03783 | related to PNP1 - purine-nucleoside phosphorylase |
| 24c00053 | 0.00479 | K09494 | chaperonin complex component, TCP-1 beta subunit | um00866 | 5.43E-05 | ni | related to Endoglucanase |
| 26d00030 | 0.00482 | ni | FOG, RRM domain | um11271 | 5.56E-05 | K13525 | probable CDC48 - Microsomal protein of CDC48 |
| 9c00362 | 0.00485 | K01179 | hypothetical protein | um02160 | 5.72E-05 | ni | conserved hypothetical protein |
| 7d00200 | 0.00488 | K03538 | RNase MRP and P, subunit POP4/p29 | um11031 | 5.86E-05 | ni | conserved hypothetical protein |
| 9c00333 | 0.00489 | ni | predicted E3 ubiquitin ligase | um15063 | 5.87E-05 | ni | conserved hypothetical protein |
| 9c00115 | 0.00491 | ni | hypothetical protein | um01922 | 5.87E-05 | K00459 | conserved hypothetical protein |
| 24d00034 | 0.00492 | ni | hypothetical protein | um01826 | 5.91E-05 | ni | putative protein |
| 22d00225 | 0.00495 | ni | hypothetical protein | um11896 | 5.93E-05 | K03936 | probable NADH-ubiquinone oxidoreductase 30 |
| 9d00104 | 0.00496 | ni | hypothetical protein | um04705 | 5.94E-05 | K00618 | related to ARG2 - acetylglutamate synthase |
| 13c00018 | 0.00499 | ni | hypothetical protein | um03754 | 5.95E-05 | ni | conserved hypothetical protein |
| 10d00012 | 0.005 | K00362 | hypothetical protein | um11032 | 5.98E-05 | ni | conserved hypothetical protein |
| 19d00116 | 0.00501 | ni | vacuolar assembly/sorting protein VPS8 | um05233 | 6.06E-05 | K01872 | probable ALA1 - alanyl-tRNA synthetase |
| 7d00275 | 0.00502 | ni | predicted glycosyltransferase | um05486 | 6.07E-05 | ni | hypothetical protein |
| 7d00049 | 0.00502 | K08496 | golgi SNAP receptor complex member | um01507 | 6.13E-05 | ni | putative protein |
| 12c00047 | 0.00503 | ni | hypothetical protein | um10559 | 6.26E-05 | ni | conserved hypothetical protein |
| 8c00034 | 0.00504 | ni | hypothetical protein | um04872 | 6.28E-05 | K01940 | probable ARG1 - argininosuccinate synthetase |
| 6c00040 | 0.00508 | ni | hypothetical protein | um02922 | 6.36E-05 | K00381 | probable sulfite reductase cys-4 |
| 10d00104 | 0.00509 | ni | hypothetical protein | um05402 | 6.39E-05 | ni | related to DOA1 - involved in ubiquitin-dependent proteolysis |
| 27d00017 | 0.00509 | K11498 | kinesin-like protein | um03152 | 6.40E-05 | ni | conserved hypothetical protein |
| 14d00080 | 0.0051 | ni | secretory carrier membrane protein | um01835 | 6.53E-05 | K00624 | related to carnitine acetyl transferase FacC |
| 7c00262 | 0.00513 | K00693 | glycogen synthase | um01258 | 6.57E-05 | ni | conserved hypothetical protein |
| 14c00038 | 0.00516 | K04371 | mitogen-activated protein kinase | um11751.2 | 6.61E-05 | K07300 | conserved hypothetical protein |
| 26c00098 | 0.00516 | ni | enoyl-coa hydratase | um00594 | 6.78E-05 | K00627 | probable dihydrolipoamide acetyltransferase component of pyruvate dehydrogenase complex |
| 7d00221 | 0.00518 | ni | hypothetical protein | um05970 | 7.06E-05 | K00155 | related to aldehyde dehydrogenase |
| 3c00079 | 0.00518 | K00737 | hypothetical protein | um05923 | 7.14E-05 | K00065 | probable 2-deoxy-D-gluconate 3-dehydrogenase |
| 20c00054 | 0.0052 | ni | phosphatidylinositol transfer protein PDR16 and related proteins | um02336 | 7.19E-05 | ni | conserved hypothetical protein |
| 24c00065 | 0.0053 | ni | hypothetical protein | um11002 | 7.43E-05 | ni | conserved hypothetical Ustilago-specific protein |
| 22c00192 | 0.00535 | ni | hypothetical protein | um01233 | 7.54E-05 | K00058 | probable 3-phosphoglycerate dehydrogenase |
| 22c00287 | 0.00537 | ni | hypothetical protein | um03605 | 7.55E-05 | K01193 | related to Sucrose-6-phosphate hydrolase |

| | | | | | | | |
|---|---|---|---|---|---|---|---|
| 7c00091 | 0.00542 | ni | RNA-binding protein SART3 | um06404 | 7.58E-05 | K03386 | probable thioredoxin peroxidase |
| 15d00007 | 0.00543 | ni | hypothetical protein | um02640 | 7.59E-05 | ni | conserved hypothetical protein |
| 3d00047 | 0.00545 | K00615 | transketolase | um03854 | 7.63E-05 | K00161 | probable PDA1 - pyruvate dehydrogenase |
| 10d00004 | 0.00548 | ni | aspartyl protease | um06048 | 7.64E-05 | K07170 | conserved hypothetical protein |
| 26c00034 | 0.00548 | ni | hypothetical protein | um05230 | 7.70E-05 | K00480 | probable Salicylate hydroxylase |
| 19c00127 | 0.0056 | ni | hypothetical protein | um01850 | 7.70E-05 | ni | conserved hypothetical protein |
| 5d00054 | 0.00562 | ni | hypothetical protein | um10342 | 7.79E-05 | ni | putative protein |
| 5d00092 | 0.00564 | K02999 | RNA polymerase I, large subunit | um10692 | 7.79E-05 | ni | putative protein |
| 10c00046 | 0.00567 | ni | very-long-chain acyl-CoA dehydrogenase | um05082 | 7.85E-05 | ni | conserved hypothetical protein |
| 9d00202 | 0.00569 | K10364 | F-actin capping protein, alpha subunit | um00188 | 7.87E-05 | ni | conserved hypothetical protein |
| 27c00043 | 0.00572 | ni | urea transporter | um02072 | 7.92E-05 | ni | conserved hypothetical protein |
| 12c00115 | 0.00577 | K01756 | adenylosuccinate lyase | um11586 | 8.03E-05 | ni | putative protein |
| 22d00075 | 0.00582 | ni | hypothetical protein | um01574 | 8.17E-05 | ni | conserved hypothetical protein |
| 19c00098 | 0.00588 | K02873 | 60S Ribosomal protein L13 | um10080 | 8.25E-05 | ni | probable theta class glutathione s-transferase |
| 5d00015 | 0.00594 | ni | hypothetical protein | um00138 | 8.26E-05 | K02358 | probable TUF1 - translation elongation factor TU |
| 1c00019 | 0.006 | ni | hypothetical protein | um01450 | 8.27E-05 | ni | related to COQ2 - para-hydroxybenzoate--polyprenyltransferase |
| 15d00025 | 0.00602 | ni | hypothetical protein | um11909 | 8.32E-05 | ni | probable RSR1 - GTP-binding protein |
| 12c00059 | 0.00609 | ni | vacuolar assembly/sorting protein VPS41 | um01163 | 8.39E-05 | ni | conserved hypothetical protein |
| 9d00141 | 0.00617 | K01907 | acyl-coa synthetase | um00045 | 8.41E-05 | K00344 | related to quinone reductase |
| 13d00004 | 0.00619 | ni | hypothetical protein | um00222.2 | 8.41E-05 | ni | related to monocarboxylate transporter |
| 20c00015 | 0.0062 | K13025 | predicted ATP-dependent RNA helicase FAL1 | um10905 | 8.57E-05 | ni | conserved hypothetical protein |
| 19c00088 | 0.0062 | K00390 | phosphoadenosine phosphosulfate reductase | um02415 | 8.61E-05 | K10753 | related to anti-silencing protein 1 |
| 16d00029 | 0.00625 | ni | reductases with broad range of substrate specificities | um05593 | 8.88E-05 | ni | hypothetical protein |
| 4c00029 | 0.00629 | K03252 | hypothetical protein | um03838 | 9.20E-05 | K01551 | probable arsenite translocating ATPase |
| 22c00310 | 0.0063 | ni | hypothetical protein | um02001 | 9.30E-05 | K01640 | related to hydroxymethylglutaryl-CoA lyase |
| 22d00180 | 0.00631 | K15118 | predicted mitochondrial carrier protein | um03659.2 | 9.38E-05 | ni | conserved hypothetical protein |
| 9d00041 | 0.00633 | K02838 | ribosome recycling factor | um02819 | 9.51E-05 | ni | conserved hypothetical protein |
| 11d00039 | 0.00642 | K11648 | integral membrane protein | um02273 | 9.58E-05 | ni | putative protein |
| 9c00087 | 0.00644 | K02358 | mitochondrial translation elongation factor Tu | um00859 | 9.60E-05 | ni | conserved hypothetical protein |
| 11c00061 | 0.00644 | K09272 | nucleosome-binding factor SPN, POB3 subunit | um01379 | 9.80E-05 | K09647 | related to IMP1 - protease |
| 7c00075 | 0.00648 | K08286 | serine/threonine protein kinase | um05729 | 9.90E-05 | ni | conserved hypothetical protein |
| 18c00060 | 0.00648 | K10424 | hypothetical protein | um10414 | 9.94E-05 | ni | conserved hypothetical protein |
| 9c00322 | 0.0065 | K04508 | beta-transducin family protein | um05116.2 | 0.0001 | K02357 | conserved hypothetical protein |
| 7c00188 | 0.00654 | ni | signal recognition particle, subunit Srp68 | um11055 | 0.0001 | ni | conserved hypothetical protein |
| 6d00017 | 0.00658 | ni | FOG, Predicted E3 ubiquitin ligase | um04183 | 0.0001 | K12846 | conserved hypothetical protein |
| 3c00001 | 0.00659 | ni | hypothetical protein | um01724 | 0.0001 | ni | conserved hypothetical protein |
| 9c00176 | 0.00662 | ni | hypothetical protein | um05202 | 0.0001 | ni | conserved hypothetical protein |
| 22c00015 | 0.00671 | K03237 | translation initiation factor 2, alpha subunit | um01627 | 0.0001 | K01647 | probable CIT1 - citrate |
| 5d00137 | 0.00674 | K13342 | TPR repeat-containing protein | um03220 | 0.0001 | K01743 | conserved hypothetical protein |
| 9d00316 | 0.00674 | ni | hypothetical protein | um02548 | 0.00011 | ni | related to Sorting nexin 9 |
| 13d00091 | 0.00677 | K02180 | mitotic spindle checkpoint protein BUB3 | um01936 | 0.00011 | ni | related to 4-coumarate-CoA ligase |
| 20c00029 | 0.00693 | K03258 | FOG, RRM domain | um02741 | 0.00011 | ni | related to CBK1 - Serine |
| 13d00073 | 0.007 | ni | exosomal 3'-5' exoribonuclease complex, subunit Rrp44/Dis3 | um10219 | 0.00011 | ni | related to 3-dehydroshikimate dehydratase |
| 3d00043 | 0.00701 | K02950 | mitochondrial/chloroplast ribosomal protein S12 | um10746.2 | 0.00011 | K01253 | conserved hypothetical protein |
| 16c00076 | 0.00702 | K17069 | cystathionine beta-lyases | um10699 | 0.00011 | ni | probable SRP1 - Importin alpha |
| 18d00087 | 0.00703 | K14794 | uncharacterized conserved protein | um01429 | 0.00011 | ni | related to high-affinity nickel transport protein nic1 |
| 7c00142 | 0.00704 | ni | zinc-binding oxidoreductase | um05936 | 0.00011 | ni | conserved hypothetical protein |
| 2d00042 | 0.00711 | ni | nonsense-mediated mRNA decay protein | um03942 | 0.00011 | ni | conserved hypothetical protein |
| 6c00109 | 0.00712 | ni | hypothetical protein | um02117 | 0.00011 | ni | putative protein |
| 7c00361 | 0.00718 | ni | 3-Methylcrotonyl-CoA carboxylase, non-biotin containing subunit | um03290 | 0.00011 | K04482 | DNA repair protein RAD51 |
| 2d00007 | 0.00718 | ni | hypothetical protein | um03761 | 0.00011 | ni | related to aldehyde dehydrogenase |
| 19d00110 | 0.00719 | K05543 | tRNA-dihydrouridine synthase | um06138 | 0.00012 | K03305 | probable PTR2 - Di- and tripeptide permease |
| 25d00062 | 0.0072 | ni | hypothetical protein | um02410 | 0.00012 | ni | hypothetical protein |
| 26c00035 | 0.00724 | ni | chromatin remodeling complex WSTF-ISWI, large subunit | um10973 | 0.00012 | K00121 | probable glutathione-dependent formaldehyde dehydrogenase |
| 5c00028 | 0.00728 | K03165 | DNA topoisomerase III alpha | um02063 | 0.00012 | ni | conserved hypothetical protein |
| 9c00003 | 0.00731 | ni | hypothetical protein | um02915 | 0.00012 | K00833 | related to Adenosylmethionine-8-amino-7-oxononanoate aminotransferase |
| 18c00093 | 0.00739 | ni | C-3 sterol dehydrogenase | um11519 | 0.00012 | K02913 | probable 50s ribosomal protein L33 |
| 5d00052 | 0.00739 | ni | hypothetical protein | um00264 | 0.00012 | ni | related to ZAP1 - metalloregulatory protein involved in zinc-responsive transcriptional regulation |
| 13c00067 | 0.00744 | K00236 | succinate dehydrogenase, cytochrome b subunit | um05632 | 0.00012 | ni | probable ISU1 - Iron-Sulfur cluster nifU-like protein |
| 18d00043 | 0.00752 | ni | hypothetical protein | um02967 | 0.00012 | K00940 | probable YNK1 - nucleoside diphosphate kinase |
| 9d00248 | 0.00754 | K02155 | vacuolar H+-ATPase V0 sector, subunits c/c' | um06378 | 0.00012 | ni | putative protein |
| 20d00076 | 0.00756 | ni | hypothetical protein | um10951 | 0.00012 | ni | putative protein |
| 19d00041 | 0.00757 | K03139 | transcription initiation factor IIF, small subunit | um01224 | 0.00013 | ni | MADS-box homolog Umc1 |
| 27d00020 | 0.00758 | ni | mitochondrial solute carrier protein | um11574 | 0.00013 | K01607 | related to Carboxymuconolactone decarboxylase |
| 27d00062 | 0.0076 | K15174 | putative RNA polymerase II regulator | um00411.2 | 0.00013 | ni | related to metalloprotease |
| 24c00052 | 0.0076 | ni | transcription factor of the Forkhead/HNF3 family | um10067 | 0.00013 | ni | hypothetical protein |
| 7d00316 | 0.00767 | ni | hypothetical protein | um04644 | 0.00013 | K01771 | related to 1-phosphatidylinositol phosphodiesterase precursor |
| 9d00238 | 0.00768 | K15196 | transcription initiation factor TFIIIB, Brf1 subunit | um11595.2 | 0.00013 | ni | conserved hypothetical protein |
| 22c00290 | 0.0077 | ni | galactokinase | um00412 | 0.00014 | K00008 | related to sorbitol dehydrogenase |
| 20c00060 | 0.00773 | ni | predicted membrane protein | um06427 | 0.00014 | ni | related to Kinesin-like protein KIF2C |
| 13d00031 | 0.00775 | ni | hypothetical protein | um05357 | 0.00014 | ni | related to SSZ1 - regulator protein involved in pleiotropic drug resistance |
| 10d00011 | 0.0078 | K00264 | glutamate synthase | um03749 | 0.00014 | ni | conserved hypothetical Ustilago-specific protein |
| 16d00061 | 0.00787 | K10592 | E3 ubiquitin-protein ligase | um01414 | 0.00014 | ni | related to Ras-GTPase-activating protein binding protein 2 |
| 19d00024 | 0.0079 | K05605 | enoyl-coa hydratase | um02709 | 0.00014 | K11253 | probable HHT1 - histone H3 |
| 6d00002 | 0.00791 | K00480 | hypothetical protein | um10452 | 0.00014 | K03441 | related to channel protein |
| 2d00021 | 0.00803 | K01610 | hypothetical protein | um00749 | 0.00015 | ni | related to lipase |
| 9d00077 | 0.00803 | ni | hypothetical protein | um15043.2 | 0.00015 | ni | probable ENA2 - Plasma membrane P-type ATPase |
| 9c00372 | 0.00804 | K14792 | rRNA processing protein Rrp5 | um12111 | 0.00015 | ni | related to small nuclear ribonucleoprotein snRNP U1A |
| 5c00131 | 0.00827 | ni | hypothetical protein | um00555 | 0.00015 | K00006 | probable glycerol-3-phosphate dehydrogenase |
| 5c00133 | 0.00829 | ni | hypothetical protein | um05058 | 0.00015 | ni | conserved hypothetical protein |
| 1c00016 | 0.0083 | ni | enoyl-coa hydratase | um03817 | 0.00016 | ni | putative protein |
| 24c00047 | 0.00835 | K03243 | translation initiation factor 5B | um03068 | 0.00016 | K00344 | related to NADPH2 |
| 13d00043 | 0.00845 | ni | hypothetical protein | um04805 | 0.00016 | ni | hypothetical protein |
| 6d00089 | 0.00846 | K15634 | phosphoglycerate mutase | um11215 | 0.00016 | K02178 | related to spindle assembly checkpoint protein |
| 18c00098 | 0.00848 | ni | ATP-dependent RNA helicase A | um03015 | 0.00016 | ni | conserved hypothetical protein |
| 20d00065 | 0.00856 | K02893 | 60s ribosomal protein L23 | um04612 | 0.00016 | K00297 | probable MET13 - putative methylene tetrahydrofolate reductase |
| 7d00053 | 0.00856 | ni | hypothetical protein | um04216 | 0.00016 | K14416 | related to translation elongation factor HBS1 protein |
| 18c00011 | 0.00856 | ni | flavonol reductase | um11013 | 0.00016 | ni | putative protein |
| 9d00366 | 0.00859 | ni | predicted helicase | um03384 | 0.00016 | ni | related to YHK8 - putative mediator of drug efflux |
| 19d00022 | 0.0086 | ni | hypothetical protein | um02150 | 0.00017 | K00008 | probable xylitol dehydrogenase |
| 12d00086 | 0.00862 | ni | predicted transporter | um02411 | 0.00017 | ni | glyoxaloxidase 1 |
| 5d00078 | 0.00878 | ni | hypothetical protein | um02921 | 0.00017 | ni | conserved hypothetical protein |
| 9d00351 | 0.00881 | K04536 | G-protein beta subunit | um01421 | 0.00017 | ni | related to PHO80 - cyclin |
| 13d00003 | 0.00881 | K12189 | uncharacterized conserved protein | um12271 | 0.00017 | K00509 | putative dioxygenase Ssp1 |
| 22c00149 | 0.00884 | K00793 | riboflavin synthase alpha chain | um05390 | 0.00017 | K13350 | related to peroxisomal membrane protein 4 |
| 7d00237 | 0.00887 | K06890 | N-methyl-D-aspartate receptor glutamate-binding subunit | um05132 | 0.00017 | K07300 | related to VCX1 - Vacuolar Ca |
| 24d00006 | 0.00889 | K03018 | RNA polymerase III, large subunit | um05686 | 0.00017 | ni | putative protein |
| 8c00016 | 0.00903 | ni | hypothetical protein | um11297 | 0.00017 | ni | conserved hypothetical protein |
| 9c00217 | 0.00907 | K01908 | acyl-coa synthetase | um10631 | 0.00017 | ni | conserved hypothetical Ustilago-specific protein |
| 22c00139 | 0.00912 | ni | hypothetical protein | um00897 | 0.00017 | ni | conserved hypothetical protein |
| 7d00082 | 0.00912 | ni | Na+/H+ antiporter | um03204 | 0.00017 | K00698 | chitin synthase 8 |
| 9c00092 | 0.0092 | ni | hypothetical protein | um04433 | 0.00018 | ni | hydrophobin 3 |
| 9c00124 | 0.00924 | ni | hypothetical protein | um04999 | 0.00018 | ni | related to RDS2 - Regulator of drug sensitivity |
| 3c00016 | 0.00927 | K08874 | histone acetyltransferase SAGA, TRRAP/TRA1 component | um03923 | 0.00018 | ni | conserved hypothetical protein |
| 12c00043 | 0.00929 | ni | hypothetical protein | um10648 | 0.00018 | ni | related to DNF2 - Non-essential P-type ATPase |
| 6c00084 | 0.00936 | K00598 | hypothetical protein | um00736 | 0.00018 | ni | conserved hypothetical protein |
| 22d00017 | 0.00942 | ni | carnitine O-acyltransferase CRAT | um01984 | 0.00019 | K13953 | probable ADH1 - alcohol dehydrogenase I |
| 11c00042 | 0.00946 | K06669 | structural maintenance of chromosome protein 3 | um10690 | 0.00019 | ni | hypothetical protein |
| 22d00034 | 0.00949 | K12767 | NDR and related serine/threonine kinases | um10070 | 0.00019 | ni | related to Benzoate 4-monooxygenase |
| 7c00237 | 0.00951 | ni | hypothetical protein | um04384 | 0.00019 | K11344 | conserved hypothetical protein |
| 3c00034 | 0.00953 | ni | ankyrin repeat protein | um05328 | 0.00019 | ni | conserved hypothetical protein |
| 16c00070 | 0.00954 | K08341 | microtubule-associated anchor protein | um06274 | 0.00019 | ni | related to hormone-sensitive lipase |
| 26c00066 | 0.00958 | K11290 | DNA replication factor | um04496 | 0.0002 | K00720 | related to Ceramide glucosyltransferase |
| 5d00120 | 0.00958 | K09496 | chaperonin complex component, TCP-1 delta subunit | um02528 | 0.0002 | K13342 | related to PEX5 - peroxisomal targeting signal receptor |
| 14c00108 | 0.0096 | ni | hypothetical protein | um03069 | 0.0002 | K01895 | probable acetyl-CoA synthetase |
| 9c00202 | 0.0097 | ni | hypothetical protein | um06063 | 0.0002 | K01580 | related to GAD1 - glutamate decarboxylase |
| 25c00012 | 0.00971 | K00698 | chitin synthase/hyaluronan synthase | um11947 | 0.0002 | ni | conserved hypothetical protein |
| 24c00049 | 0.00972 | K01955 | multifunctional pyrimidine synthesis protein CAD | um00103 | 0.0002 | ni | related to ESBP6 - similarity to Monocarboxylate transporter |
| 14d00047 | 0.00974 | ni | hypothetical protein | um12236 | 0.0002 | K16066 | probable NADP |
| 13d00063 | 0.00979 | ni | predicted Zn2+-dependent endopeptidase | um04443 | 0.00021 | ni | related to translation activator GCN1 |
| 12c00019 | 0.00982 | ni | putative N2 | um10254 | 0.00021 | ni | conserved hypothetical protein |
| 4c00008 | 0.00985 | K03469 | ribonuclease H | um01560 | 0.00021 | K10401 | related to kinesin |
| 22d00146 | 0.00994 | ni | hypothetical protein | um00063 | 0.00021 | ni | putative protein |
| 22c00255 | 0.01002 | ni | hypothetical protein | um11848 | 0.00021 | ni | probable Myosin regulatory light chain cdc4 |
| 2c00074 | 0.01004 | ni | hypothetical protein | um02880 | 0.00021 | ni | conserved hypothetical protein |
| 9d00043 | 0.01007 | ni | hypothetical protein | um02382 | 0.00022 | ni | a1-specific pheromone |
| 5c00061 | 0.01013 | ni | RNA-directed RNA polymerase QDE-1 | um10585 | 0.00022 | ni | conserved hypothetical protein |
| 22d00176 | 0.01013 | ni | arylacetamide deacetylase | um01986 | 0.00022 | ni | probable sterol carrier protein |
| 9c00439 | 0.01013 | ni | hypothetical protein | um01179 | 0.00022 | ni | putative protein |
| 27c00069 | 0.01018 | K14821 | U1-like Zn-finger-containing protein | um03524 | 0.00022 | K00276 | related to peroxisomal amine oxidase |
| 24c00042 | 0.01018 | ni | protein required for meiosis | um02404 | 0.00023 | K10751 | conserved hypothetical protein |
| 9c00283 | 0.01019 | ni | predicted signal transduction protein | um00688 | 0.00023 | ni | related to bacterial leucyl aminopeptidase precursor |
| 12c00133 | 0.01025 | ni | hypothetical protein | um06072 | 0.00024 | K13719 | conserved hypothetical protein |
| 26c00036 | 0.01027 | ni | serine/threonine protein phosphatase | um04175 | 0.00024 | K00281 | probable GCV2 - glycine decarboxylase P subunit |
| 9c00136 | 0.0103 | K01433 | 5'-phosphoribosylglycinamide formyltransferase | um15103 | 0.00024 | K09051 | related to transcription factor ATFA |
| 8d00035 | 0.0103 | K15075 | DNA repair/transcription protein Mms19 | um11878 | 0.00024 | ni | ni |
| 18d00092 | 0.01031 | ni | mitochondrial/choloroplast ribosomal protein S15 | um04806 | 0.00024 | ni | conserved hypothetical protein |
| 7c00282 | 0.01031 | K15687 | predicted E3 ubiquitin ligase | um04491 | 0.00024 | ni | ni |
| 7c00325 | 0.01032 | K03349 | anaphase-promoting complex (APC), subunit 2 | um10235 | 0.00025 | K15423 | probable serine |
| 27c00019 | 0.01033 | ni | hypothetical protein | um10365 | 0.00026 | ni | related to YBT1 - Vacuolar |
| 8c00045 | 0.01046 | K11541 | multifunctional pyrimidine synthesis protein CAD | um01141 | 0.00026 | ni | Diaphanous related Formin |
| 27c00095 | 0.01048 | ni | hypothetical protein | um12096 | 0.00026 | ni | conserved hypothetical protein |
| 24c00055 | 0.01052 | K15176 | TPR-containing nuclear phosphoprotein that regulates K+ uptake | um01143 | 0.00026 | ni | related to chitin deacetylase precursor |
| 25d00082 | 0.01054 | ni | endoplasmic reticulum protein EP58 | um05790 | 0.00026 | ni | conserved hypothetical protein |
| 2c00018 | 0.01054 | ni | hypothetical protein | um05227 | 0.00026 | ni | hypothetical protein |
| 20c00016 | 0.01058 | K10743 | ribonuclease HI | um00038 | 0.00027 | ni | related to transaldolase |
| 27d00095 | 0.01063 | ni | hypothetical protein | um01014 | 0.00027 | ni | conserved hypothetical protein |
| 20d00046 | 0.01064 | K03938 | hypothetical protein | um01775 | 0.00027 | ni | conserved hypothetical protein |
| 7d00319 | 0.01068 | K02872 | 60S ribosomal protein L13a | um02974 | 0.00027 | ni | related to AUT4 - breakdown of autophagic vesicles inside the vacuole |
| 9d00388 | 0.01074 | K17267 | vesicle coat complex COPI, gamma subunit | um04393 | 0.00027 | ni | putative protein |
| 16c00028 | 0.01079 | ni | hypothetical protein | um01815 | 0.00027 | ni | related to carbonyl reductase |
| 7d00258 | 0.01079 | K01533 | cation transport ATPase | um01584 | 0.00028 | ni | conserved hypothetical Ustilago-specific protein |
| 12d00004 | 0.01085 | K00451 | homogentisate 1,2-dioxygenase | um05381 | 0.00028 | ni | putative protein |
| 26c00026 | 0.01085 | K14264 | kynurenine aminotransferase | um01331.2 | 0.00028 | ni | conserved hypothetical protein |
| 15c00080 | 0.01087 | K11763 | hypothetical protein | um02820 | 0.00028 | ni | conserved hypothetical protein |
| 9c00084 | 0.01096 | K01062 | phospholipase A2 | um01289 | 0.00028 | ni | related to AXL2 - required for axial pattern of budding |

| | | | | | | | |
|---|---|---|---|---|---|---|---|
| 15d00067 | 0.01098 | ni | hypothetical protein | um04972 | 0.00029 | ni | related to NFU-1 protein |
| 27d00071 | 0.01104 | K02958 | 40S ribosomal protein S15 | um03408 | 0.00029 | K00480 | related to Salicylate hydroxylase |
| 2d00019 | 0.01105 | K01886 | glutaminyl-trna synthetase | um10599 | 0.00029 | K00167 | probable branched-chain alpha-keto acid dehydrogenase e1-beta subunit |
| 9c00358 | 0.01111 | K02991 | 40S ribosomal protein S6 | um00466 | 0.0003 | ni | hypothetical protein |
| 26c00037 | 0.01113 | ni | Ca2+-dependent lipid-binding protein CLB1 | um10170 | 0.00031 | K05658 | related to multidrug resistance protein 1 |
| 25c00052 | 0.01123 | ni | predicted DNA-binding protein | um10718.2 | 0.00031 | K00698 | chitin synthase 1 |
| 12c00078 | 0.01123 | ni | FOG, RRM domain | um01458 | 0.00031 | K02327 | probable DNA polymerase delta catalytic subunit |
| 7c00212 | 0.0113 | ni | FOG, RRM domain | um06092 | 0.00031 | ni | conserved hypothetical protein |
| 20d00026 | 0.01132 | ni | hypothetical protein | um11269 | 0.00031 | ni | conserved hypothetical protein |
| 9c00414 | 0.01133 | ni | hypothetical protein | um06426 | 0.00031 | K13509 | related to SLC1 - 1-acyl-sn-gylcerol-3-phosphate acyltransferase |
| 9d00369 | 0.01133 | K02258 | cytochrome oxidase assembly factor COX11 | um05984 | 0.00031 | K15371 | probable glutamate dehydrogenase |
| 15c00083 | 0.01136 | ni | possible oxidoreductase | um10783 | 0.00032 | K01759 | probable glyoxylase I |
| 13d00035 | 0.01137 | K12818 | DEAH-box RNA helicase | um02204 | 0.00032 | ni | related to beta-galactosidase |
| 22d00273 | 0.01159 | K00020 | fructose 1,6-bisphosphate aldolase | um11220 | 0.00033 | K00728 | related to dolichyl-phosphate-mannose-protein mannosyltransferase |
| 9d00059 | 0.0116 | ni | hypothetical protein | um00530 | 0.00033 | ni | conserved hypothetical protein |
| 2c00050 | 0.01164 | K14820 | RNA-binding protein required for biogenesis of the ribosomal 60S | um02481 | 0.00034 | ni | conserved hypothetical protein |
| 22d00160 | 0.01166 | ni | hypothetical protein | um05059 | 0.00034 | K00643 | probable 5-aminolevulinic acid synthase |
| 27c00003 | 0.01167 | K00720 | ceramide glucosyltransferase | um03890 | 0.00034 | ni | hypothetical protein |
| 22d00111 | 0.0117 | K13577 | mitochondrial oxoglutarate/malate carrier proteins | um10752 | 0.00034 | ni | conserved hypothetical protein |
| 8c00101 | 0.01172 | K11558 | uncharacterized conserved protein | um11951 | 0.00034 | ni | putative protein |
| 3c00049 | 0.01176 | K12670 | oligosaccharyltransferase, beta subunit | um00050 | 0.00034 | ni | putative protein |
| 27c00002 | 0.01177 | ni | hypothetical protein | um04382 | 0.00035 | ni | probable methylcrotonoyl-CoA carboxylase biotin carboxylase chain |
| 9d00247 | 0.01187 | ni | hypothetical protein | um10676 | 0.00035 | ni | conserved hypothetical protein |
| 13d00036 | 0.01187 | K00309 | FAD-dependent oxidoreductase | um00430 | 0.00035 | K00605 | probable GCV1 - glycine decarboxylase |
| 3d00026 | 0.01194 | ni | mu-crystallin | um10318 | 0.00035 | K10735 | conserved hypothetical protein |
| 13d00032 | 0.01199 | ni | glucosidase I | um11315 | 0.00036 | ni | putative protein |
| 22d00095 | 0.012 | K00111 | vacuolar H+-ATPase V1 sector, subunit B | um03806 | 0.00036 | ni | hypothetical protein |
| 14d00086 | 0.01213 | K13348 | peroxisomal membrane protein MPV17 and related proteins | um03740 | 0.00036 | K11267 | related to PDS5 - precocious dissociation of sister chromatids |
| 2c00038 | 0.01216 | K01870 | isoleucyl-trna synthetase | um12325 | 0.00037 | ni | hypothetical protein |
| 25c00004 | 0.01222 | ni | hypothetical protein | um10310 | 0.00037 | K00868 | related to pyridoxal kinase |
| 13d00110 | 0.01222 | ni | hypothetical protein | um12007 | 0.00037 | ni | related to cellulase |
| 26d00058 | 0.01228 | ni | sensory transduction histidine kinase | um02005 | 0.00037 | ni | conserved hypothetical protein |
| 7c00081 | 0.01232 | K02934 | 60s ribosomal protein L6 | um12085 | 0.00037 | ni | conserved hypothetical protein |
| 7c00057 | 0.01235 | K05544 | uncharacterized conserved protein | um04224 | 0.00038 | K11548 | related to Myosin-like protein NUF2 |
| 26d00057 | 0.01241 | ni | hypothetical protein | um03835 | 0.00038 | ni | putative protein |
| 6d00031 | 0.01242 | ni | hypothetical protein | um00265 | 0.00039 | ni | related to pyruvate dehydrogenase complex protein X precursor |
| 5c00040 | 0.01243 | ni | hypothetical protein | um05153 | 0.00039 | ni | related to MNT4 - putative alpha-1 |
| 21c00005 | 0.01244 | ni | hypothetical protein | um12331 | 0.00039 | ni | conserved hypothetical protein |
| 7c00100 | 0.01272 | ni | lysophosphatidic acid acyltransferase endophilin/SH3GL | um00735 | 0.00039 | ni | conserved hypothetical protein |
| 22d00153 | 0.01273 | ni | Ca2+ transporting ATPase | um04288 | 0.00039 | ni | conserved hypothetical protein |
| 13c00082 | 0.01275 | ni | hypothetical protein | um10985 | 0.0004 | K00872 | probable THR1 - homoserine kinase |
| 20c00034 | 0.01278 | K01210 | hypothetical protein | um00385 | 0.0004 | ni | putative protein |
| 25d00037 | 0.01281 | ni | hypothetical protein | um11863 | 0.00041 | ni | conserved hypothetical protein |
| 7d00084 | 0.01282 | K00681 | gamma-glutamyltransferase | um11714 | 0.00041 | ni | putative protein |
| 9c00160 | 0.01282 | ni | hypothetical protein | um05429 | 0.00041 | K14404 | related to Cleavage and polyadenylation specificity factor |
| 19d00159 | 0.01288 | K09448 | TEF-1 and related transcription factor | um01953 | 0.00041 | ni | conserved hypothetical protein |
| 18d00076 | 0.0129 | K01890 | phenylalanyl-trna synthetase beta subunit | um00028 | 0.00041 | ni | conserved hypothetical protein |
| 22c00249 | 0.01294 | ni | hypothetical protein | um02158 | 0.00042 | K15428 | conserved hypothetical protein |
| 2d00008 | 0.01296 | K00958 | ATP sulfurylase | um10097 | 0.00042 | ni | conserved hypothetical protein |
| 4c00036 | 0.01299 | ni | hypothetical protein | um10851 | 0.00042 | ni | conserved hypothetical protein |
| 7c00175 | 0.01301 | K01889 | phenylalanyl-trna synthetase | um10370 | 0.00042 | ni | conserved hypothetical protein |
| 20d00012 | 0.01302 | K12591 | exosome 3'-5' exoribonuclease complex, subunit PM/SCL-100 | um06163 | 0.00042 | ni | conserved hypothetical protein |
| 10d00018 | 0.01307 | K08852 | serine/threonine protein kinase and endoribonuclease ERN1/IRE1 | um00998 | 0.00042 | ni | putative protein |
| 7c00025 | 0.01317 | K06053 | recombination signal binding protein-J kappa | um10781 | 0.00042 | K00799 | related to glutathione s-transferase |
| 13d00093 | 0.0132 | ni | pleiotropic drug resistance proteins | um01172 | 0.00043 | K00234 | probable SDH1 - succinate dehydrogenase |
| 22d00027 | 0.01323 | ni | hypothetical protein | um03824 | 0.00043 | K06215 | probable Pyridoxine biosynthesis protein PDX1 |
| 4d00011 | 0.01326 | ni | hormone-sensitive lipase HSL | um04420 | 0.00043 | ni | related to ECM31 - 3-methyl-2-oxobutanoate hydroxymethyltransferase |
| 9d00280 | 0.01334 | K00670 | N-terminal acetyltransferase | um12183 | 0.00043 | ni | conserved hypothetical protein |
| 3c00068 | 0.01341 | K17081 | prohibitin-like protein | um10407 | 0.00043 | ni | conserved hypothetical protein |
| 22d00217 | 0.01366 | K00108 | glucose dehydrogenase | um04801.2 | 0.00044 | ni | related to Glutathione S-transferase II |
| 22c00244 | 0.01367 | ni | predicted transporter | um05445 | 0.00044 | ni | hypothetical protein |
| 19d00063 | 0.01374 | ni | hypothetical protein | um02818 | 0.00044 | ni | related to Multidrug resistance-associated protein |
| 16d00073 | 0.0138 | ni | hypothetical protein | um05985 | 0.00045 | ni | putative protein |
| 19c00139 | 0.0138 | K05758 | actin-related protein Arp2/3 complex, subunit ARPC2 | um15074 | 0.00045 | ni | related to ABC transporter |
| 7d00157 | 0.01384 | ni | predicted mitochondrial carrier protein | um12049 | 0.00045 | ni | conserved hypothetical protein |
| 18d00094 | 0.01384 | ni | hypothetical protein | um03468 | 0.00046 | ni | conserved hypothetical protein |
| 14c00005 | 0.01385 | ni | hypothetical protein | um01088 | 0.00046 | K06127 | probable COQ5 - ubiquinone biosynthesis |
| 12d00134 | 0.01391 | K07047 | hypothetical protein | um15096 | 0.00046 | ni | related to cAMP-independent regulatory protein pac2 |
| 26c00070 | 0.01393 | ni | protein involved in high osmolarity signaling pathway | um03376 | 0.00046 | K14550 | conserved hypothetical protein |
| 26c00074 | 0.01394 | ni | hypothetical protein | um06082 | 0.00047 | ni | related to NIPSNAP protein |
| 12d00002 | 0.01404 | ni | hypothetical protein | um10273 | 0.00048 | ni | related to delta3 |
| 6d00104 | 0.01406 | ni | hypothetical protein | um05663 | 0.00049 | ni | conserved hypothetical protein |
| 5c00137 | 0.01419 | ni | hypothetical protein | um10641 | 0.00049 | ni | related to SKT5 - protoplast regeneration and killer toxin resistance protein |
| 18c00036 | 0.0142 | K13179 | ATP-dependent RNA helicase pitchoune | um02497 | 0.00049 | ni | conserved hypothetical protein |
| 22c00173 | 0.01422 | ni | hypothetical protein | um06121 | 0.00049 | ni | putative protein |
| 7d00203 | 0.01435 | ni | porphobilinogen deaminase | um10439 | 0.00049 | ni | conserved hypothetical protein |
| 20d00041 | 0.01445 | ni | lysophosphatidic acid acyltransferase LPAAT and related acyltrar | um04771 | 0.0005 | ni | related to KHA1 - Putative K |
| 14c00133 | 0.01446 | ni | arylacetamide deacetylase | um03120 | 0.0005 | ni | conserved hypothetical protein |
| 7d00320 | 0.01455 | ni | hypothetical protein | um11033 | 0.0005 | ni | putative protein |
| 26c00083 | 0.01472 | K14963 | WD40 repeat-containing protein | um05156 | 0.0005 | K07466 | probable Replication factor-A protein 1 |
| 13d00097 | 0.01473 | ni | hypothetical protein | um06381 | 0.00051 | ni | putative protein |
| 18c00080 | 0.01473 | ni | hypothetical protein | um02959 | 0.00051 | ni | conserved hypothetical protein |
| 7d00246 | 0.01478 | ni | hypothetical protein | um12106 | 0.00051 | K01280 | related to Tripeptidyl-peptidase II |
| 12c00057 | 0.01482 | K02951 | 40S ribosomal protein S12 | um11383 | 0.00052 | ni | conserved hypothetical protein |
| 12c00075 | 0.01487 | K08052 | ras GTPase activating protein RasGAP | um11534 | 0.00052 | K00413 | probable CYT1 - cytochrome c1 |
| 18d00026 | 0.01494 | ni | protein containing adaptin N-terminal region | um02517 | 0.00052 | K04640 | guanine nucleotide-binding protein alpha-2 subunit |
| 14c00111 | 0.01496 | K15275 | UDP-galactose transporter related protein | um01390 | 0.00053 | ni | conserved hypothetical protein |
| 22c00076 | 0.01501 | ni | CCAAT-binding factor, subunit B | um04716 | 0.00053 | K02148 | probable VMA5 - H -ATPase V1 domain 42 KD subunit |
| 9d00375 | 0.01502 | K03125 | transcription initiation factor TFIID, subunit TAF1 | um10835 | 0.00053 | ni | related to Cytochrome b561 |
| 2d00046 | 0.01512 | ni | hypothetical protein | um06430 | 0.00053 | ni | probable heat shock protein HSP104 |
| 20d00073 | 0.01512 | K01412 | mitochondrial processing peptidase, beta subunit, and related enz | um11121 | 0.00053 | K07213 | probable ATX1 - antioxidant protein and metal homeostasis factor |
| 7d00099 | 0.01517 | ni | hypothetical protein | um00562 | 0.00053 | ni | conserved hypothetical protein |
| 4d00014 | 0.01522 | K00287 | phospholipase | um06073 | 0.00053 | ni | related to DCW1 - Mannosidase |
| 9c00204 | 0.0153 | ni | ferric reductase | um02398 | 0.00054 | K00511 | related to squalene monooxygenase |
| 26d00094 | 0.01534 | K01840 | phosphomannomutase | um02980 | 0.00054 | ni | probable dihydroxy-acid dehydratase |
| 26c00077 | 0.01547 | ni | FOG, PPR repeat | um12091 | 0.00055 | ni | conserved hypothetical protein |
| 26c00033 | 0.01547 | K01497 | bifunctional GTP cyclohydrolase II | um11961 | 0.00055 | ni | conserved hypothetical protein |
| 13c00096 | 0.01547 | K11800 | WD40 repeat protein | um11789 | 0.00056 | ni | conserved hypothetical protein |
| 22c00073 | 0.0155 | K12385 | cholesterol transport protein | um02712 | 0.00056 | ni | related to PCL6 - cyclin like protein interacting with Pho85p |
| 6c00086 | 0.01558 | K00924 | Ca2+/calmodulin-dependent protein kinase | um05678 | 0.00057 | ni | related to Glutathione S-transferase |
| 19d00023 | 0.01563 | K01527 | RNA polymerase II general transcription factor BTF3 and related | um10703 | 0.00058 | K14264 | related to BNA3 - Arylformamidase |
| 9c00161 | 0.01568 | ni | hypothetical protein | um01110 | 0.00058 | K06640 | related to serine-protein kinase atr |
| 27d00059 | 0.01572 | K11673 | actin-related protein | um10869.2 | 0.00058 | ni | putative protein |
| 9d00411 | 0.01579 | ni | hypothetical protein | um01135 | 0.00058 | ni | ni |
| 19d00103 | 0.01583 | ni | hypothetical protein | um05574 | 0.0006 | ni | conserved hypothetical protein |
| 22d00044 | 0.01595 | ni | hypothetical protein | um01656 | 0.0006 | ni | probable sugar transporter |
| 26d00060 | 0.01601 | ni | hypothetical protein | um10394 | 0.00061 | K00287 | related to Dihydrofolate reductase |
| 6d00073 | 0.01602 | ni | hypothetical protein | um01433 | 0.00061 | ni | related to Enoyl-CoA hydratase |
| 22c00067 | 0.01605 | ni | hypothetical protein | um00459 | 0.00061 | K06678 | related to Condensin complex subunit 3 |
| 22c00305 | 0.01605 | ni | DEAD box-containing helicase-like transcription factor | um00687 | 0.00062 | K11801 | conserved hypothetical protein |
| 16c00052 | 0.01609 | ni | synaptic vesicle protein EHS-1 and related EH domain proteins | um03418 | 0.00062 | ni | putative protein |
| 9c00262 | 0.01613 | ni | monocarboxylate transporter | um15024 | 0.00062 | ni | putative protein |
| 10d00027 | 0.01622 | ni | hypothetical protein | um02703 | 0.00062 | K03841 | probable FBP1 - fructose-1 |
| 9c00350 | 0.01628 | ni | hypothetical protein | um02164 | 0.00063 | K03885 | probable NDE1 - mitochondrial cytosolically directed NADH dehydrogenase |
| 8c00010 | 0.01631 | ni | hypothetical protein | um10688 | 0.00063 | ni | related to PET112 - putative glutamyl-tRNA |
| 20d00032 | 0.01638 | ni | hypothetical protein | um11954 | 0.00064 | ni | related to neutral amino acid permease |
| 24c00023 | 0.01639 | ni | reductases with broad range of substrate specificities | um01813 | 0.00064 | ni | related to putative acetyl-coenzyme A transporter |
| 1d00048 | 0.01641 | ni | hypothetical protein | um05442 | 0.00064 | ni | related to monocarboxylate transporter 2 |
| 9c00105 | 0.01643 | ni | hypothetical protein | um01142 | 0.00064 | K17402 | related to RSM25 - mitochondrial ribosomal protein |
| 8d00066 | 0.0165 | ni | hypothetical protein | um01515 | 0.00064 | K07393 | related to ECM4 - protein involved in cell wall biogenesis and architecture |
| 14c00103 | 0.01654 | K00287 | dihydrofolate reductase | um12179 | 0.00065 | ni | putative protein |
| 18d00038 | 0.01655 | K00231 | protoporphyrinogen oxidase | um00864 | 0.00065 | K09522 | probable ZUO1 - zuotin |
| 18d00081 | 0.01656 | K00281 | glycine dehydrogenase | um11366 | 0.00066 | ni | conserved hypothetical protein |
| 6c00020 | 0.01664 | ni | hypothetical protein | um10733 | 0.00066 | ni | putative protein |
| 13d00040 | 0.01675 | K15628 | peroxisomal long-chain acyl-CoA transporter | um03299 | 0.00066 | K01803 | probable TPI1 - triose-phosphate isomerase |
| 27c00021 | 0.01696 | ni | protein OS-9 | um02006 | 0.00066 | ni | conserved hypothetical protein |
| 22c00137 | 0.01697 | K02355 | mitochondrial elongation factor | um04847 | 0.00067 | ni | conserved hypothetical protein |
| 27c00022 | 0.01699 | ni | hypothetical protein | um03284 | 0.00067 | ni | conserved hypothetical protein |
| 6c00052 | 0.01707 | ni | hypothetical protein | um00773 | 0.00067 | ni | conserved hypothetical protein |
| 7d00154 | 0.01709 | K14521 | predicted P-loop ATPase fused to an acetyltransferase | um00583 | 0.00067 | K07006 | conserved hypothetical protein |
| 8d00014 | 0.01714 | ni | hypothetical protein | um00308 | 0.00069 | K10771 | related to APN1 - AP endonuclease |
| 14c00112 | 0.01717 | ni | hypothetical protein | um10560 | 0.0007 | K09263 | conserved hypothetical protein |
| 19d00140 | 0.01729 | K12852 | U5 snRNP-specific protein | um04096 | 0.0007 | ni | hypothetical protein |
| 2c00081 | 0.01743 | K03857 | N-acetylglucosaminyltransferase complex, subunit PIG-A/SPT14 | um04979 | 0.0007 | K01101 | related to PDR16 - protein involved in lipid biosynthesis and multidrug resistance |
| 22c00043 | 0.01759 | ni | hypothetical protein | um05355 | 0.0007 | K00637 | related to acyl-CoA sterol acyltransferase |
| 27c00094 | 0.01766 | ni | monocarboxylate transporter | um01856 | 0.0007 | ni | related to PET8 protein |
| 13c00014 | 0.01772 | ni | hypothetical protein | um10753 | 0.00071 | ni | conserved hypothetical protein |
| 22c00234 | 0.01786 | ni | phosphoinositide-specific phospholipase C | um11303 | 0.00071 | ni | conserved hypothetical protein |
| 5d00090 | 0.0179 | ni | hypothetical protein | um01134 | 0.00072 | ni | related to HEM3 - porphobilinogen deaminase |
| 5d00075 | 0.01793 | K00671 | N-myristoyl transferase | um01945 | 0.00072 | K01193 | probable SUC2 - invertase |
| 9d00283 | 0.01796 | ni | ATP-dependent RNA helicase A | um00392 | 0.00072 | ni | conserved hypothetical protein |
| 27c00011 | 0.01803 | K14782 | apoptosis antagonizing transcription factor/protein transport prote | um06333 | 0.00072 | K00486 | related to BNA4 - kynurenine 3-mono oxygenase |
| 7d00142 | 0.01807 | ni | hypothetical protein | um03921 | 0.00072 | K04646 | probable CHC1 - clathrin heavy chain |
| 18c00042 | 0.01808 | K11548 | centromere-associated protein NUF2 | um03601 | 0.00073 | K15455 | conserved hypothetical protein |
| 13c00004 | 0.01809 | K00761 | armadillo | um03501 | 0.00073 | K03164 | probable DNA topoisomerase II |
| 9c00059 | 0.0181 | ni | uncharacterized conserved protein | um00495 | 0.00073 | ni | conserved hypothetical protein |
| 25d00053 | 0.01812 | ni | SOK1 kinase | um03564 | 0.00073 | ni | ni |
| 25c00047 | 0.01831 | ni | metallopeptidase | um11932 | 0.00073 | K01969 | probable methylcrotonyl-CoA carboxylase beta chain |
| 7d00195 | 0.01831 | ni | hypothetical Protein | um01992 | 0.00074 | ni | related to amidohydrolase |
| 26c00058 | 0.01833 | K12184 | vacuolar sorting protein VPS28 | um03095 | 0.00074 | ni | DNA repair |
| 10d00122 | 0.01834 | ni | hypothetical protein | um04429 | 0.00074 | K00817 | probable histidinol-phosphate transaminase |
| 14c00097 | 0.01836 | ni | hypothetical protein | um00286 | 0.00075 | ni | ni |
| 14c00127 | 0.01839 | ni | dimeric dihydrodiol dehydrogenase | um10420 | 0.00075 | ni | related to meiotic recombination protein SPO11 |
| 14d00098 | 0.01843 | K10746 | 5'-3' exonuclease | um06186 | 0.00076 | ni | related to oxidoreductase |
| 3c00056 | 0.01843 | ni | cdc4 and related F-box and WD-40 proteins | um01054 | 0.00076 | K01958 | probable pyruvate carboxylase |
| 20c00055 | 0.0185 | ni | hypothetical protein | um01664 | 0.00077 | K01082 | probable MET22 - protein ser |

| | | | | | | | |
|---|---|---|---|---|---|---|---|
| 22d00119 | 0.01855 | K01899 | succinyl-coa synthetase, alpha subunit | um12250 | 0.00077 | ni | conserved hypothetical protein |
| 3d00069 | 0.01855 | ni | hypothetical protein | um10637 | 0.00078 | ni | conserved hypothetical protein |
| 9d00270 | 0.01856 | ni | FOG, Zn-finger | um10606 | 0.00078 | ni | conserved hypothetical protein |
| 6d00009 | 0.01863 | ni | glucosamine-phosphate N-acetyltransferase | um11280 | 0.00078 | ni | conserved hypothetical protein |
| 12d00040 | 0.01863 | ni | TFIIF-interacting CTD phosphatase, including NLI-interacting fac | um03382 | 0.00078 | ni | related to 3-phytase A precursor |
| 27c00096 | 0.01867 | ni | hypothetical protein | um01904 | 0.00078 | ni | related to succinyl-coa ligase |
| 22d00041 | 0.01867 | ni | hypothetical protein | um03845 | 0.00078 | K00237 | related to succinate dehydrogenase |
| 16d00047 | 0.01873 | K00826 | branched chain aminotransferase BCAT1 | um01436 | 0.00079 | ni | conserved hypothetical protein |
| 11c00012 | 0.0188 | ni | hypothetical protein | um05570 | 0.00079 | ni | conserved hypothetical protein |
| 24c00007 | 0.01896 | ni | predicted membrane protein | um10946 | 0.00079 | ni | related to TNA1 - High affinity nicotinic acid plasma membrane permease |
| 7d00255 | 0.01896 | ni | hypothetical protein | um03105 | 0.00079 | ni | hypothetical protein |
| 18d00003 | 0.01907 | K14857 | putative SAM-dependent rRNA methyltransferase SPB1 | um06124 | 0.00079 | ni | hypothetical protein |
| 26d00089 | 0.01909 | ni | hypothetical protein | um00935 | 0.0008 | ni | probable MDR1 - Mac1p interacting protein |
| 9c00435 | 0.01914 | ni | hypothetical protein | um01205 | 0.0008 | ni | cation-transporting ATPase |
| 12c00024 | 0.01915 | ni | hypothetical protein | um04614 | 0.0008 | ni | conserved hypothetical Ustilago-specific protein |
| 22d00186 | 0.01925 | ni | hypothetical protein | um02710 | 0.00081 | K11254 | probable HHF1 - histone H4 |
| 22d00241 | 0.01925 | ni | predicted L-carnitine dehydratase | um04885 | 0.00081 | ni | conserved hypothetical protein |
| 13c00056 | 0.01926 | ni | uncharacterized conserved protein | um10181 | 0.00082 | ni | conserved hypothetical protein |
| 7c00215 | 0.01928 | ni | molecular chaperone Prefoldin, subunit 5 | um10051 | 0.00083 | K02910 | probable 60S ribosomal protein L31 |
| 22c00282 | 0.01937 | ni | hypothetical protein | um10575 | 0.00084 | ni | hypothetical protein |
| 3d00024 | 0.01939 | K10838 | nucleotide excision repair complex XPC-HR23B, subunit XPC/DF | um05198 | 0.00085 | ni | ni |
| 12c00146 | 0.01947 | ni | predicted transporter | um01688 | 0.00085 | K14284 | related to mRNA export factor mex67 |
| 19c00128 | 0.01957 | ni | acetylcholinesterase | um12124 | 0.00085 | ni | hypothetical protein |
| 1d00016 | 0.01959 | K13172 | Splicing coactivator SRm160/300, subunit SRm300 | um00087 | 0.00086 | ni | conserved hypothetical protein |
| 8c00076 | 0.01967 | K13126 | polyadenylate-binding protein | um10476 | 0.00086 | ni | hypothetical protein |
| 19d00010 | 0.01981 | ni | hypothetical protein | um10898 | 0.00089 | ni | related to short-chain alcohol dehydrogenase |
| 9d00231 | 0.01986 | ni | hypothetical protein | um05403 | 0.0009 | K04802 | probable proliferating cell nuclear antigen |
| 7d00215 | 0.01999 | ni | hypothetical protein | um01699 | 0.00091 | ni | conserved hypothetical protein |
| 7d00117 | 0.02002 | ni | hypothetical protein | um04939 | 0.00091 | K01476 | probable arginase |
| 5c00005 | 0.02007 | ni | nuclear architecture related protein | um05452.2 | 0.00091 | ni | related to TPO3 - Polyamine transport protein |
| 25c00003 | 0.02011 | ni | hypothetical protein | um02152 | 0.00091 | ni | conserved hypothetical Ustilago-specific protein |
| 18d00033 | 0.02026 | K17415 | hypothetical protein | um10003 | 0.00092 | ni | conserved hypothetical protein |
| 2d00002 | 0.02026 | K11367 | chromodomain-helicase DNA-binding protein | um12048 | 0.00092 | K01495 | probable FOL2 - GTP cyclohydrolase I |
| 1c00038 | 0.02027 | ni | kinesin-like protein | um10077 | 0.00092 | K11173 | probable alcohol dehydrogenase |
| 14c00055 | 0.0205 | K00022 | short-chain alcohol dehydrogenase | um01085 | 0.00093 | K10866 | probable RAD50 - DNA repair protein |
| 18c00054 | 0.02052 | ni | hypothetical protein | um06033 | 0.00094 | ni | related to LRP16 protein |
| 11c00008 | 0.02055 | ni | hypothetical protein | um03888 | 0.00094 | ni | related to multidrug resistant protein |
| 14c00131 | 0.02062 | K12603 | glucose-repressible alcohol dehydrogenase transcriptional effecto | um11438 | 0.00095 | ni | conserved hypothetical protein |
| 9d00098 | 0.02062 | ni | uncharacterized conserved protein | um11807 | 0.00097 | K00765 | probable ATP phosphoribosyltransferase |
| 9c00198 | 0.02064 | K12599 | cytoplasmic exosomal RNA helicase SKI2 | um01782 | 0.00097 | K13354 | related to peroxisomal membrane protein PMP47B |
| 6d00050 | 0.02084 | K08139 | predicted transporter | um11980 | 0.00097 | ni | putative protein |
| 5c00026 | 0.02084 | K01092 | inositol monophosphatase | um01711 | 0.00098 | ni | related to Glucose dehydrogenase |
| 15d00080 | 0.02086 | ni | cation transport ATPase | um01643 | 0.00098 | K07827 | small G-protein Ras2 |
| 7d00332 | 0.02096 | K15278 | UDP-N-acetylglucosamine transporter | um10007 | 0.00098 | K16330 | conserved hypothetical protein |
| 10d00085 | 0.02105 | ni | hypothetical protein | um01397 | 0.00099 | K01175 | related to REX4 - strong similarity to X |
| 12d00044 | 0.02107 | ni | hypothetical protein | um00132 | 0.001 | ni | hypothetical protein |
| 5c00018 | 0.02112 | ni | hypothetical protein | um10211 | 0.001 | K01210 | related to EXG1 - exo-beta-1 |
| 9c00174 | 0.02115 | ni | hypothetical protein | um02876 | 0.001 | ni | conserved hypothetical protein |
| 9d00363 | 0.02119 | ni | MEKK and related serine/threonine protein kinases | um05792 | 0.00101 | ni | related to Chitin deacetylase precursor |
| 3c00019 | 0.02123 | ni | uncharacterized conserved protein | um10757 | 0.00101 | ni | conserved hypothetical protein |
| 2d00066 | 0.02125 | K14050 | protein geranylgeranyltransferase type II, alpha subunit | um03486 | 0.00103 | ni | related to nucleotide binding protein |
| 22c00250 | 0.02138 | ni | predicted transporter | um02189 | 0.00103 | K00020 | probable 3-hydroxyisobutyrate dehydrogenase |
| 2c00016 | 0.02144 | ni | multidrug/pheromone exporter | um01371 | 0.00104 | ni | ni |
| 8d00078 | 0.02146 | K14640 | Na+/Pi symporter | um15085 | 0.00105 | ni | related to peptide-n4- |
| 7d00093 | 0.02162 | ni | hypothetical protein | um04796 | 0.00106 | ni | conserved hypothetical protein |
| 22d00013 | 0.02164 | ni | hypothetical protein | um00548 | 0.00106 | ni | related to GDA1 - guanosine diphosphatase |
| 3c00061 | 0.02167 | ni | hypothetical protein | um03623 | 0.00107 | ni | conserved hypothetical protein |
| 7c00330 | 0.02175 | ni | dehydrogenases with different specificities | um03088 | 0.00107 | ni | conserved hypothetical protein |
| 14d00050 | 0.02183 | K10352 | myosin class II heavy chain | um10738 | 0.00108 | K11984 | conserved hypothetical protein |
| 10c00038 | 0.0219 | K03321 | sulfate/bicarbonate/oxalate exchanger SAT-1 and related transpo | um10252 | 0.00108 | K00059 | related to 3-oxoacyl- |
| 2c00078 | 0.0219 | K01768 | serine/threonine phosphatase 2C | um01663 | 0.00108 | ni | conserved hypothetical protein |
| 6c00076 | 0.02191 | ni | amino acid transporters | um10140 | 0.00109 | ni | conserved hypothetical protein |
| 22c00245 | 0.02194 | ni | hypothetical protein | um11051 | 0.0011 | ni | putative protein |
| 14d00099 | 0.02203 | ni | endoplasmic reticulum protein EP58 | um11362 | 0.0011 | ni | putative protein |
| 6d00083 | 0.02209 | ni | hypothetical protein | um11219 | 0.00111 | K08489 | related to TLG2 - member of the syntaxin family of t-SNAREs |
| 16c00032 | 0.02214 | ni | oxysterol-binding protein | um11459.2 | 0.00111 | ni | conserved hypothetical protein |
| 11c00037 | 0.0223 | ni | emp24/gp25L/p24 family of membrane trafficking proteins | um02810 | 0.00112 | ni | conserved hypothetical Ustilago-specific protein |
| 12c00100 | 0.02251 | K03233 | translation elongation factor EF-1 gamma | um03347 | 0.00112 | ni | related to NUM1 - nuclear migration protein |
| 14c00015 | 0.02258 | K08737 | mismatch repair ATPase MSH6 | um02492 | 0.00112 | ni | conserved hypothetical protein |
| 18c00071 | 0.0227 | ni | Ca2+-binding transmembrane protein LETM1/MRS7 | um00634 | 0.00113 | K03943 | probable NADH-ubiquinone oxidoreductase 24 kDa subunit |
| 7d00254 | 0.02278 | K01958 | pyruvate carboxylase | um06139 | 0.00114 | K14692 | related to zinc transporter |
| 8c00024 | 0.02279 | ni | hypothetical protein | um12273.2 | 0.00114 | K12840 | conserved hypothetical protein |
| 22c00075 | 0.02279 | ni | predicted RNA polymerase III subunit C17 | um00255 | 0.00114 | ni | conserved hypothetical protein |
| 12d00092 | 0.02285 | ni | predicted GTPase-activating protein | um11953 | 0.00115 | K01408 | related to STE23 - Metalloprotease involved in a-factor processing |
| 10c00036 | 0.02288 | K03283 | molecular chaperones HSP70/HSC70 | um04878 | 0.00115 | K14572 | related to MDN1 - Huge dynein-related AAA-type ATPase |
| 27c00032 | 0.02298 | ni | non-smc | um05240 | 0.00116 | K11304 | probable ESA1 - histone acetyltransferase |
| 22c00258 | 0.02319 | ni | hypothetical protein | um00490 | 0.00117 | ni | related to Sorting nexin MVP1 |
| 7c00024 | 0.02352 | K12826 | splicing factor 3a, subunit 2 | um00830 | 0.00117 | ni | conserved hypothetical protein |
| 7c00170 | 0.02355 | ni | hypothetical protein | um15097 | 0.00118 | ni | putative protein |
| 11c00035 | 0.02358 | ni | hypothetical protein | um02125 | 0.00119 | K01580 | related to glutamic acid decarboxylase |
| 10d00007 | 0.02359 | K11649 | chromatin remodeling factor subunit and related transcription fact | um11162 | 0.00119 | K03935 | probable NADH dehydrogenase |
| 3d00049 | 0.02361 | ni | synaptic vesicle transporter SVOP and related transporters | um10685 | 0.00121 | K03627 | probable MBF1 - multiprotein bridging factor mediates GCN4-dependent transcriptional activation |
| 7d00158 | 0.02362 | ni | regulator of arginine metabolism and related MADS box-containin | um01714 | 0.00121 | ni | probable monooxygenase |
| 13d00020 | 0.02362 | ni | hypothetical protein | um03351 | 0.00123 | K06174 | probable RLI1 - Protein promoting preinitiation complex assembly |
| 9d00190 | 0.02372 | ni | PHD Zn-finger proteins | um06351 | 0.00123 | ni | conserved hypothetical Ustilago-specific protein |
| 10d00017 | 0.02381 | K12867 | mRNA splicing factor | um05691 | 0.00123 | ni | hypothetical protein |
| 22d00101 | 0.02393 | ni | predicted Yippee-type zinc-binding protein | um04055 | 0.00127 | ni | ni |
| 9d00225 | 0.02395 | K00698 | chitin synthase/hyaluronan synthase | um11753 | 0.00128 | ni | conserved hypothetical protein |
| 5d00041 | 0.02398 | ni | meltrins | um03807 | 0.0013 | ni | conserved hypothetical protein |
| 2d00016 | 0.02402 | ni | hypothetical protein | um02115 | 0.00131 | ni | conserved hypothetical protein |
| 13c00048 | 0.02446 | ni | hypothetical protein | um03262 | 0.00131 | K02365 | related to Separin |
| 14c00073 | 0.02448 | ni | hypothetical protein | um12299 | 0.00131 | ni | conserved hypothetical protein |
| 9d00277 | 0.02448 | K07006 | hypothetical protein | um02693 | 0.00131 | ni | related to Endothelin-converting enzyme 1 |
| 9c00347 | 0.02449 | ni | hypothetical protein | um04133 | 0.00132 | ni | related to NAD |
| 9c00175 | 0.02462 | K12823 | ATP-dependent RNA helicase | um11682 | 0.00132 | K00587 | related to STE14 - farnesyl cysteine carboxyl-methyltransferase |
| 7c00354 | 0.02465 | ni | ferric reductase | um03221 | 0.00132 | ni | conserved hypothetical protein |
| 12c00015 | 0.02492 | ni | hypothetical protein | um05385 | 0.00135 | ni | guanine nucleotide-binding protein alpha-4 subunit |
| 22d00284 | 0.02502 | ni | hypothetical protein | um00790 | 0.00137 | ni | related to coenzyme a synthetase |
| 2c00008 | 0.0251 | K13342 | TPR repeat-containing protein | um02105 | 0.00137 | K00074 | probable 3-hydroxybutyryl-CoA dehydrogenase |
| 7c00040 | 0.02511 | K11121 | NAD-dependent histone deacetylases and class I sirtuins | um01037 | 0.00138 | ni | conserved hypothetical protein |
| 14d00075 | 0.02516 | ni | hypothetical protein | um10253.2 | 0.00138 | K01265 | related to MAP1 - methionine aminopeptidase |
| 7c00240 | 0.02518 | ni | predicted N-acetyltransferase | um10242 | 0.00139 | ni | putative protein |
| 22c00248 | 0.02528 | K01288 | serine carboxypeptidases | um01114 | 0.0014 | ni | hypothetical protein |
| 22c00079 | 0.0254 | K16575 | actin and related proteins | um03822 | 0.00141 | ni | putative protein |
| 13d00010 | 0.02562 | ni | hypothetical protein | um01533 | 0.00143 | ni | related to n-acetyltransferase |
| 5d00079 | 0.02564 | ni | hypothetical protein | um05420 | 0.00144 | K12346 | related to vacuolar transport protein ESP1 |
| 7d00169 | 0.02576 | ni | defense-related protein containing SCP domain | um05692 | 0.00145 | ni | related to COX10 - farnesyl transferase |
| 26c00029 | 0.02581 | ni | hypothetical protein | um00468 | 0.00145 | K00601 | related to glycinamide ribonucleotide transformylase |
| 18c00026 | 0.02584 | K16489 | hypothetical protein | um11966.2 | 0.00146 | ni | related to Cullin-3 |
| 5d00105 | 0.02593 | ni | chromatin remodeling complex WSTF-ISWI, small subunit | um04561 | 0.00147 | K00852 | conserved hypothetical protein |
| 20c00023 | 0.02594 | K00031 | NADP-dependent isocitrate dehydrogenase | um00296 | 0.00147 | ni | conserved hypothetical protein |
| 25c00066 | 0.02596 | ni | hypothetical protein | um12146 | 0.0015 | ni | conserved hypothetical protein |
| 9d00335 | 0.02612 | K08869 | predicted unusual protein kinase | um05977 | 0.00151 | ni | conserved hypothetical protein |
| 9d00119 | 0.02623 | K14326 | RNA helicase nonsense mRNA reducing factor | um12278 | 0.00151 | K10418 | probable Dynein light chain 1 |
| 6c00096 | 0.02627 | ni | hypothetical protein | um05162 | 0.00152 | K01952 | probable ADE6 - phosphoribosylformyl glycinamidine synthetase |
| 13c00042 | 0.02629 | ni | hypothetical protein | um10507 | 0.00153 | K00411 | probable ubiquinol-cytochrome-c reductase Rieske iron-sulfur protein |
| 9c00097 | 0.0264 | ni | hypothetical protein | um01164 | 0.00153 | ni | conserved hypothetical protein |
| 19d00049 | 0.02646 | ni | predicted MutS-related protein | um00046 | 0.00154 | ni | related to flavin oxidoreductase |
| 10d00056 | 0.0265 | ni | ras-related small GTPase | um10730 | 0.00155 | K05767 | related to Ras GTPase-activating-like protein IQGAP2 |
| 25c00078 | 0.0266 | ni | hypothetical protein | um01428 | 0.00156 | ni | conserved hypothetical protein |
| 12c00123 | 0.02673 | K07889 | GTPase Rab5/YPT51 | um04867 | 0.00157 | ni | ni |
| 3c00028 | 0.02674 | ni | hypothetical protein | um02131 | 0.0016 | ni | putative protein |
| 6c00024 | 0.0268 | K06911 | hypothetical protein | um11079 | 0.00161 | ni | putative protein |
| 22c00304 | 0.02686 | ni | hypothetical protein | um12288 | 0.00164 | ni | conserved hypothetical protein |
| 15d00063 | 0.02692 | ni | hypothetical protein | um02170 | 0.00165 | ni | conserved hypothetical protein |
| 6d00054 | 0.02701 | K07019 | alpha/beta hydrolase | um11746 | 0.00168 | ni | related to Guanine nucleotide-binding protein beta 5 |
| 7d00008 | 0.02711 | ni | hypothetical protein | um10529.2 | 0.00168 | ni | conserved hypothetical protein |
| 10d00111 | 0.02713 | K03457 | uridine permease | um10616 | 0.00169 | K16466 | probable CDC31 - spindle pole body component |
| 25d00052 | 0.02718 | ni | hypothetical protein | um11849 | 0.00169 | ni | conserved hypothetical protein |
| 8c00110 | 0.02721 | K12824 | hypothetical protein | um02590 | 0.0017 | ni | putative protein |
| 16d00086 | 0.02723 | K11095 | U1 snRNP-specific protein C | um11267 | 0.00171 | K00993 | related to EPT1 - sn-1 |
| 7d00305 | 0.02731 | K12625 | small nuclear ribonucleoprotein F | um03126 | 0.00173 | ni | related to retinal short-chain dehydrogenase |
| 25d00079 | 0.02733 | ni | hypothetical protein | um01977 | 0.00173 | ni | hypothetical protein |
| 18d00044 | 0.02734 | ni | rab6 GTPase activator GAPCenA and related TBC domain protei | um00295 | 0.00175 | K04393 | GTP binding protein Cdc42 |
| 9d00105 | 0.02755 | ni | mitochondrial carrier protein CGI-69 | um04091 | 0.00175 | ni | conserved hypothetical protein |
| 9c00363 | 0.02778 | K05293 | major facilitator superfamily permease - Cdc91p | um01332 | 0.00177 | ni | related to coenzyme A diphosphatase |
| 8c00001 | 0.02796 | ni | hypothetical protein | um04961 | 0.00177 | ni | conserved hypothetical protein |
| 22c00183 | 0.02807 | ni | predicted oxidoreductase | um12158 | 0.00179 | ni | putative protein |
| 9c00354 | 0.02808 | ni | hypothetical protein | um01250 | 0.0018 | K00294 | probable delta-1-pyrroline-5-carboxylate dehydrogenase |
| 9d00205 | 0.02825 | ni | hypothetical protein | um11684 | 0.00181 | K02963 | related to 30s ribosomal protein s18 |
| 9d00067 | 0.02839 | ni | hypothetical protein | um10924 | 0.00181 | K10772 | related to APN2 - AP endonuclease |
| 19d00048 | 0.02842 | ni | hypothetical protein | um04686 | 0.00181 | ni | putative protein |
| 5c00034 | 0.02843 | ni | regucalcin gene promoter region-related protein | um05212 | 0.00181 | K07976 | related to YPT31 - GTP-binding protein of the rab family |
| 22c00228 | 0.02843 | ni | hypothetical protein | um03982 | 0.00182 | ni | conserved hypothetical protein |
| 3d00051 | 0.02858 | K07511 | enoyl-coa hydratase | um02838.2 | 0.00183 | ni | conserved hypothetical protein |
| 10c00006 | 0.02861 | K01663 | glutamine amidotransferase | um05677 | 0.00183 | K11390 | related to citrate lyase beta subunit |
| 18c00072 | 0.02867 | ni | hypothetical protein | um00886 | 0.00183 | ni | putative protein |
| 22c00231 | 0.02887 | K03325 | hypothetical protein | um11919 | 0.00186 | K00029 | related to NADP-dependent malic enzyme |
| 13d00044 | 0.02888 | K04382 | serine/threonine protein phosphatase 2A, catalytic subunit | um06371 | 0.00189 | ni | conserved hypothetical protein |
| 12d00018 | 0.02893 | ni | hypothetical protein | um01311 | 0.00189 | ni | related to NADH-ubiquinone oxidoreductase B16 |
| 12d00026 | 0.02902 | K11778 | cis-prenyltransferase | um03674 | 0.0019 | ni | conserved hypothetical protein |
| 19d00045 | 0.02906 | ni | predicted methyltransferase | um01863 | 0.0019 | ni | related to Cytochrome P450 |
| 18d00049 | 0.02932 | ni | hypothetical protein | um11046 | 0.00191 | ni | putative protein |
| 7c00007 | 0.02939 | ni | hypothetical protein | um10910 | 0.00191 | K10994 | related to DNA repair protein rad9 |
| 15d00030 | 0.02944 | K17065 | vacuolar sorting protein VPS1 | um00984 | 0.00191 | K07140 | conserved hypothetical protein |
| 19c00062 | 0.02945 | K16369 | hypothetical protein | um04533 | 0.00192 | ni | conserved hypothetical protein |

| | | | | | | | |
|---|---|---|---|---|---|---|---|
| 12c00144 | 0.02952 | K04618 | hypothetical protein | um11497 | 0.00193 | K00972 | probable UDP-N-acetylglucosamine pyrophosphorylase |
| 8d00105 | 0.02963 | ni | hypothetical protein | um04922 | 0.00193 | ni | related to 2 |
| 9c00353 | 0.02967 | ni | FOG, LIM domain | um12240 | 0.00193 | ni | putative protein |
| 2d00015 | 0.02969 | ni | hypothetical protein | um00712 | 0.00195 | ni | probable YOR1 - ABC transporter |
| 22c00132 | 0.02988 | K12861 | spliceosome-associated coiled-coil protein | um12047 | 0.00197 | ni | conserved hypothetical protein |
| 7d00264 | 0.02994 | ni | cytosolic sorting protein GGA2/TOM1 | um10053 | 0.00199 | K01673 | related to carbonic anhydrase |
| 9c00184 | 0.03007 | ni | hypothetical protein | um04253 | 0.00201 | ni | probable mrna export factor elf1 |
| 24c00057 | 0.03012 | ni | hypothetical protein | um04982 | 0.00201 | K15084 | probable LEU5 - mitochondrial coenzyme A transporter- member of the mitochondrial carrier |
| 5c00097 | 0.03023 | ni | uncharacterized conserved protein | um06368 | 0.00203 | K10808 | probable small subunit of ribonucleotide reductase |
| 9d00364 | 0.03028 | K11375 | hypothetical protein | um00770 | 0.00205 | K01937 | probable URA7 - CTP synthase 1 |
| 18c00029 | 0.0303 | ni | carboxylesterase and related proteins | um11080 | 0.00211 | ni | putative protein |
| 27c00040 | 0.03031 | ni | hypothetical protein | um06489 | 0.00212 | ni | conserved hypothetical protein |
| 22c00016 | 0.03037 | ni | hypothetical protein | um10094 | 0.00213 | ni | putative protein |
| 22d00144 | 0.03053 | ni | predicted short chain-type dehydrogenase | um04535 | 0.00213 | ni | conserved hypothetical protein |
| 8c00108 | 0.03053 | ni | translational repressor MPT5/PUF4 and related RNA-binding pro | um11105 | 0.00213 | K02575 | related to nitrate transporter |
| 2d00031 | 0.03057 | ni | hypothetical protein | um04819 | 0.00214 | ni | related to protein phosphatase 2c |
| 2c00056 | 0.03061 | ni | hypothetical protein | um00179 | 0.00215 | ni | conserved hypothetical protein |
| 16c00024 | 0.03065 | ni | hypothetical protein | um02765 | 0.00215 | ni | conserved hypothetical protein |
| 12d00125 | 0.03065 | ni | nuclear transport receptor exportin 4 | um02165 | 0.00216 | ni | conserved hypothetical protein |
| 10d00024 | 0.03067 | ni | hypothetical protein | um10450 | 0.00217 | ni | related to transcriptional regulator rds2 |
| 7d00118 | 0.03111 | ni | HMG box-containing protein | um05818 | 0.00217 | K00293 | probable chimeric spermidine synthase |
| 14d00011 | 0.03127 | ni | hypothetical protein | um00166 | 0.00217 | ni | conserved hypothetical protein |
| 10d00101 | 0.03128 | ni | hypothetical protein | um06130 | 0.00217 | K12827 | related to RNA splicing factor PRP9 |
| 9d00076 | 0.03128 | K09481 | hypothetical protein | um05840 | 0.00221 | ni | conserved hypothetical protein |
| 2c00045 | 0.03129 | K13941 | dihydropteroate synthase | um02460 | 0.00225 | ni | putative protein |
| 7c00136 | 0.03137 | ni | predicted dehydrogenase | um10992 | 0.00227 | ni | ni |
| 5d00138 | 0.03147 | K11971 | predicted E3 ubiquitin ligase | um02236 | 0.00228 | ni | conserved hypothetical protein |
| 19c00074 | 0.03153 | K00668 | hypothetical protein | um06047 | 0.00228 | K00641 | related to homoserine O-acetyltransferase |
| 19c00063 | 0.03163 | ni | FOG, RRM domain | um05131 | 0.00229 | K01907 | probable Acetoacetyl-CoA synthetase |
| 22c00064 | 0.03176 | K10401 | kinesin-like protein | um10509 | 0.0023 | ni | related to DNA repair protein Rad62 |
| 9c00338 | 0.03182 | K02145 | vacuolar H+-ATPase V1 sector, subunit A | um02037 | 0.0023 | K08139 | probable HXT5 - Hexose transporter |
| 26c00057 | 0.03182 | K03032 | 26S proteasome regulatory complex, subunit RPN2/PSMD1 | um02185 | 0.00232 | K12734 | probable peptidylprolyl isomerase |
| 3d00042 | 0.03185 | K08286 | NDR and related serine/threonine kinases | um06103 | 0.00232 | K00888 | related to phosphatidylinositol-4-kinase |
| 12c00062 | 0.03188 | ni | hypothetical protein | um05897 | 0.00233 | K01293 | probable CPS1 - Gly-X carboxypeptidase YSCS precursor |
| 7d00326 | 0.03189 | ni | hypothetical protein | um06013 | 0.00236 | ni | related to Intersectin 1 |
| 9c00034 | 0.03191 | ni | hypothetical protein | um10695 | 0.00237 | K03934 | probable NADH dehydrogenase |
| 7c00027 | 0.03191 | ni | translocase of outer mitochondrial membrane complex, subunit T | um04991 | 0.00238 | K08286 | related to Serine |
| 22d00053 | 0.03192 | K03134 | transcription initiation factor TFIID, subunit TAF10 | um11790 | 0.00239 | K16536 | related to Hook protein |
| 7c00006 | 0.03192 | ni | hypothetical protein | um01209 | 0.0024 | K04043 | probable SSC1 - mitochondrial HSP70 member |
| 3c00047 | 0.032 | K10873 | DNA repair and recombination protein RAD52/RAD22 | um02428 | 0.00242 | ni | hypothetical protein |
| 22c00265 | 0.03243 | ni | Na+/H+ antiporter | um10041.2 | 0.00242 | ni | conserved hypothetical protein |
| 18d00083 | 0.03256 | K10740 | hypothetical protein | um03025 | 0.00244 | K08735 | probable DNA mismatch repair protein MSH2 |
| 15c00045 | 0.03273 | ni | hypothetical protein | um03649 | 0.00244 | ni | hypothetical protein |
| 9c00340 | 0.03276 | K00999 | phosphatidylinositol synthase | um03103 | 0.00245 | K01808 | probable ribose-5-phosphate isomerase |
| 9d00109 | 0.03283 | K00140 | methylmalonate semialdehyde dehydrogenase | um04555 | 0.00245 | ni | myosin V |
| 9c00172 | 0.03302 | K10771 | major apurinic/apyrimidinic endonuclease | um02611 | 0.00246 | K01166 | related to ribonuclease M |
| 18c00046 | 0.03304 | ni | hypothetical protein | um10611 | 0.00247 | K12625 | probable U6 snRNA-associated Sm-like protein LSm6 |
| 8d00063 | 0.03308 | ni | asparagine synthase | um11873 | 0.00247 | ni | hypothetical protein |
| 5d00012 | 0.03314 | ni | branched chain alpha-keto acid dehydrogenase E1, beta subunit | um03436 | 0.00249 | ni | related to triacylglycerol lipase |
| 11c00026 | 0.03324 | ni | beta-glucocerebrosidase | um10289 | 0.00252 | ni | hypothetical protein |
| 16d00022 | 0.03338 | K00698 | chitin synthase/hyaluronan synthase | um11483 | 0.00252 | K00573 | probable l-isoaspartyl protein carboxyl methyltransferase |
| 13d00062 | 0.03342 | ni | hypothetical protein | um03234.2 | 0.00252 | K06631 | related to CDC5 - Serine |
| 12d00066 | 0.03353 | ni | hypothetical protein | um01721 | 0.00253 | ni | conserved hypothetical protein |
| 22c00242 | 0.03358 | K00540 | dehydrogenases with different specificities | um10521 | 0.00256 | ni | ni |
| 10c00090 | 0.03369 | K03885 | NADH-dehydrogenase | um00124 | 0.00256 | ni | hypothetical protein |
| 9c00196 | 0.0337 | K09831 | cytochrome P450 CYP4/CYP19/CYP26 subfamilies | um05988 | 0.00259 | ni | conserved hypothetical protein |
| 20c00064 | 0.03389 | ni | hypothetical protein | um05138 | 0.0026 | ni | conserved hypothetical protein |
| 9c00050 | 0.03391 | ni | uncharacterized conserved protein | um04064 | 0.00261 | K01178 | related to Glucoamylase precursor |
| 25d00044 | 0.03392 | ni | hypothetical protein | um03945 | 0.00262 | K15628 | probable peroxisomal half ABC transporter |
| 22c00293 | 0.03393 | ni | NADH-dehydrogenase | um11731 | 0.00262 | ni | related to nadh-ubiquinone oxidoreductase 9 |
| 22d00006 | 0.03396 | K01764 | holocytochrome c synthase | um11164 | 0.00262 | ni | conserved hypothetical protein |
| 20c00040 | 0.03402 | ni | hypothetical protein | um11090 | 0.00262 | ni | conserved hypothetical protein |
| 22c00288 | 0.03413 | ni | hypothetical protein | um05787 | 0.00263 | ni | conserved hypothetical protein |
| 7c00098 | 0.03446 | K00228 | coproporphyrinogen III oxidase CPO/HEM13 | um03910 | 0.00263 | K02183 | probable Calmodulin |
| 2c00067 | 0.03448 | K11314 | GTPase Rab2 | um02220 | 0.00264 | K03515 | related to DNA repair protein MUS-42 |
| 9d00303 | 0.0345 | K11518 | translocase of outer mitochondrial membrane complex, subunit T | um04786 | 0.00265 | K03032 | probable RPN2 - 26S proteasome regulatory subunit |
| 7c00033 | 0.03456 | K08517 | synaptobrevin/vamp-like protein SEC22 | um02778 | 0.00265 | ni | related to GCY1 - galactose-induced protein of aldo |
| 4c00022 | 0.0346 | ni | lipoyltransferase | um03256 | 0.00267 | K03574 | conserved hypothetical protein |
| 13d00089 | 0.03463 | K02537 | spindle assembly checkpoint protein | um05275 | 0.00267 | K00898 | related to pyruvate dehydrogenase kinase isoform 2 |
| 7d00024 | 0.03464 | K15105 | mitochondrial aspartate/glutamate carrier protein Aralar/Citrin | um02040 | 0.00267 | ni | conserved hypothetical protein |
| 7c00335 | 0.03467 | K14679 | hypothetical protein | um11014 | 0.00268 | ni | related to SCT1 - glycerol 3-phosphate |
| 16c00006 | 0.03476 | K01607 | hypothetical protein | um01906 | 0.00272 | K15151 | conserved hypothetical protein |
| 13c00015 | 0.0349 | ni | hypothetical protein | um05889 | 0.00272 | K03320 | high affinity ammonium transporter |
| 9d00033 | 0.03492 | K00809 | deoxyhypusine synthase | um01282 | 0.00274 | K08052 | related to neurofibromin |
| 12c00121 | 0.03499 | ni | hypothetical protein | um02887 | 0.00275 | ni | conserved hypothetical protein |
| 9c00187 | 0.03503 | K01068 | acyl-coa thioesterase | um00442 | 0.00276 | ni | hypothetical protein |
| 8c00114 | 0.03506 | ni | amidases | um11394 | 0.00277 | ni | conserved hypothetical protein |
| 9d00332 | 0.03508 | K00365 | uricase | um15049 | 0.00277 | ni | related to PUF3 - transcript-specific regulator of mRNA degradation |
| 25d00036 | 0.03515 | ni | heme A farnesyltransferase | um10131 | 0.00277 | K01613 | related to phosphatidylserine decarboxylase proenzyme 2 precursor |
| 19d00059 | 0.03516 | ni | Ca2+-dependent lipid-binding protein CLB1 | um04888 | 0.00279 | ni | hypothetical protein |
| 7c00233 | 0.03533 | K08675 | mitochondrial ATP-dependent protease PIM1/LON | um02363 | 0.00281 | ni | hypothetical protein |
| 7d00250 | 0.03533 | K13525 | AAA+-type ATPase | um05072 | 0.00282 | ni | conserved hypothetical Ustilago-specific protein |
| 26c00025 | 0.03535 | K00604 | methionyl-trna formyltransferase | um03210 | 0.00282 | K02219 | probable CKS1 - cyclin-dependent kinases regulatory subunit |
| 13c00085 | 0.03544 | ni | hypothetical protein | um11821 | 0.00282 | K02936 | probable RPL8B - 60s large subunit ribosomal protein L7a |
| 14d00035 | 0.03544 | ni | serine/threonine specific protein phosphatase PP1, catalytic subu | um10387 | 0.00285 | K03809 | probable 1 |
| 7c00021 | 0.0356 | ni | serine/threonine protein kinase | um10622 | 0.00285 | ni | hypothetical protein |
| 7d00044 | 0.03568 | ni | polynucleotide kinase 3' phosphatase | um00386 | 0.00286 | ni | conserved hypothetical protein |
| 25d00029 | 0.03595 | ni | hypothetical protein | um03166 | 0.00287 | ni | hypothetical protein |
| 2d00062 | 0.03597 | ni | putative N2 | um04481 | 0.00289 | ni | related to ADH2 - Alcohol dehydrogenase II |
| 12d00075 | 0.03612 | ni | sucrose transporter and related proteins | um10248 | 0.00293 | ni | conserved hypothetical protein |
| 20c00010 | 0.03624 | K02948 | hypothetical protein | um00789 | 0.00295 | K00624 | probable carnitine O-acetyltransferase |
| 7d00304 | 0.03636 | ni | tuberin - Rap/ran-GTPase-activating protein | um10528 | 0.00297 | K05658 | related to STE6 - ABC transporter |
| 19d00081 | 0.03641 | ni | hypothetical protein | um10788.2 | 0.00301 | K13721 | probable AAP1 - alanine |
| 15c00014 | 0.03691 | ni | pentafunctional AROM protein | um11504 | 0.00304 | ni | ni |
| 12c00042 | 0.03713 | K03360 | leucine rich repeat proteins | um00448 | 0.00305 | ni | putative protein |
| 7d00180 | 0.03713 | K11786 | chromatin remodeling complex SWI/SNF, component SWI2 | um03373 | 0.00305 | ni | ni |
| 10c00005 | 0.03719 | K07555 | mitochondrial F1-ATPase assembly protein | um05900 | 0.00307 | K11434 | probable HMT1 - hnRNP arginine N-methyltransferase |
| 22c00069 | 0.03727 | ni | hypothetical protein | um00043 | 0.00307 | ni | conserved hypothetical protein |
| 9d00347 | 0.03751 | K12373 | beta-n-acetylhexosaminidase | um01730 | 0.00308 | ni | conserved hypothetical protein |
| 9d00049 | 0.03772 | ni | hypothetical protein | um01004 | 0.00308 | ni | conserved hypothetical protein |
| 6d00120 | 0.03786 | K02151 | vacuolar H+-ATPase V1 sector, subunit F | um00219 | 0.00308 | K15122 | conserved hypothetical protein |
| 13c00019 | 0.03802 | ni | hypothetical protein | um10466.2 | 0.00311 | K00772 | related to MEU1 - multiple enhancer of UAS2 |
| 22c00018 | 0.03815 | ni | ypt/rab GTPase activating protein | um01122 | 0.00312 | ni | related to HCS1 - DNA helicase A |
| 27c00042 | 0.03818 | K00509 | hypothetical protein | um05687 | 0.00317 | K02725 | probable PRE5 - 20S proteasome subunit |
| 24c00027 | 0.03822 | ni | hypothetical protein | um00245.2 | 0.00318 | ni | related to mitochondrial uncoupling protein 3 |
| 5d00086 | 0.03837 | K07870 | predicted Ras related Rac-GTP binding protein | um01679 | 0.0032 | K02541 | probable MCM3 - subunit of pre-replication complex |
| 25d00024 | 0.03845 | ni | hypothetical protein | um02669.2 | 0.00322 | K10863 | conserved hypothetical protein |
| 8c00102 | 0.03847 | ni | hypothetical protein | um10469 | 0.00322 | K02989 | probable 40S ribosomal protein S5 |
| 9c00436 | 0.03849 | ni | synaptic vesicle transporter SVOP and related transporters | um11783 | 0.00322 | K10990 | conserved hypothetical protein |
| 5d00106 | 0.03859 | K01412 | mitochondrial processing peptidase, alpha subunit | um11797 | 0.00322 | K06126 | related to Ubiquinone biosynthesis protein COQ6 and COQ7 |
| 9c00142 | 0.03864 | K14805 | RNA helicase | um01969 | 0.00324 | ni | putative protein |
| 8c00032 | 0.03866 | ni | hypothetical protein | um00782 | 0.00327 | ni | hypothetical protein |
| 8c00109 | 0.03867 | ni | hypothetical protein | um01671 | 0.00329 | ni | related to YSC84 - protein involved in the organization of actin cytoskeleton |
| 20c00072 | 0.03891 | K01702 | 3-isopropylmalate dehydratase | um06474 | 0.0033 | ni | conserved hypothetical protein |
| 25d00030 | 0.03899 | ni | iron/ascorbate family oxidoreductases | um12030 | 0.0033 | ni | putative protein |
| 26d00024 | 0.03904 | ni | hypothetical protein | um04070 | 0.0033 | K07975 | Rho3 GTP binding protein |
| 18c00095 | 0.03914 | K01655 | alpha-isopropylmalate synthase/homocitrate synthase | um11010 | 0.00331 | ni | conserved hypothetical protein |
| 2c00043 | 0.03918 | K14439 | SNF2 family DNA-dependent ATPase | um05559 | 0.00332 | K03262 | probable TIF5 - translation initiation factor eIF5 |
| 27c00030 | 0.03939 | ni | predicted mechanosensitive ion channel | um02213 | 0.00333 | ni | conserved hypothetical protein |
| 7c00286 | 0.03941 | ni | hypothetical protein | um11892 | 0.00335 | K06655 | probable PHO85 - cyclin-dependent protein kinase |
| 6c00063 | 0.03955 | ni | hypothetical protein | um03818 | 0.00336 | ni | conserved hypothetical protein |
| 14c00118 | 0.03969 | ni | FOG, Predicted E3 ubiquitin ligase | um15040 | 0.0034 | K01887 | probable arginyl-tRNA synthetase |
| 1d00002 | 0.0397 | ni | hypothetical protein | um03513 | 0.00341 | ni | conserved hypothetical protein |
| 9c00415 | 0.03996 | ni | hypothetical protein | um01062 | 0.00344 | K15356 | probable VRG4 - Golgi GDP-mannose transporter |
| 12d00109 | 0.03998 | K09104 | helix loop helix transcription factor EB | um10520 | 0.00346 | K13288 | related to REX2 - Oligoribonuclease |
| 2d00075 | 0.04 | K11304 | histone acetyltransferase | um10038 | 0.00347 | K14729 | probable multifunctional beta-oxidation protein |
| 22c00091 | 0.04001 | ni | uncharacterized conserved protein | um01194 | 0.00348 | ni | putative protein |
| 7c00083 | 0.04007 | ni | hypothetical protein | um05728 | 0.00349 | ni | putative protein |
| 18d00082 | 0.04018 | K02149 | vacuolar H+-ATPase V1 sector, subunit D | um00827 | 0.00353 | ni | hypothetical protein |
| 16d00014 | 0.04033 | K01253 | soluble epoxide hydrolase | um01791 | 0.00354 | ni | conserved hypothetical protein |
| 22d00138 | 0.04044 | K02564 | glucosamine-6-phosphate isomerase | um02950 | 0.00356 | ni | putative protein |
| 9c00249 | 0.04046 | ni | multidrug resistance-associated protein | um04270 | 0.00356 | K01876 | probable DPS1 - aspartyl-tRNA synthetase |
| 3d00084 | 0.04077 | ni | hypothetical protein | um05333 | 0.00357 | K14530 | putative protein |
| 9c00012 | 0.04094 | ni | hypothetical protein | um04284 | 0.0036 | K09140 | conserved hypothetical protein |
| 22d00163 | 0.04098 | ni | amino acid transporters | um11183 | 0.00361 | ni | conserved hypothetical protein |
| 9d00166 | 0.04099 | ni | hypothetical protein | um03315 | 0.00361 | ni | serine |
| 2d00043 | 0.0412 | ni | hypothetical protein | um10002 | 0.00365 | ni | related to NADH-dependent flavin oxidoreductase |
| 22d00289 | 0.04127 | ni | hypothetical protein | um11369 | 0.00365 | K15463 | related to RIT1 - initiator tRNA phosphoribosyl-transferase |
| 24c00070 | 0.0414 | K09680 | pantothenate kinase PanK and related proteins | um00510 | 0.00366 | ni | probable MSP1 - intra-mitochondrial sorting protein |
| 20d00037 | 0.04154 | ni | 5,10-methylenetetrahydrofolate reductase | um05658 | 0.00367 | ni | conserved hypothetical protein |
| 19d00019 | 0.04175 | ni | putative trehalase | um03286 | 0.00369 | K10352 | related to MYO1 - myosin-1 isoform |
| 18c00062 | 0.04183 | K03265 | peptide chain release factor 1 | um02347 | 0.0037 | ni | conserved hypothetical protein |
| 12d00115 | 0.04184 | K03217 | inner membrane protein translocase involved in respiratory chain | um04559 | 0.0037 | ni | hypothetical protein |
| 19c00068 | 0.04194 | ni | hypothetical protein | um02584 | 0.00372 | ni | conserved hypothetical protein |
| 5c00052 | 0.04197 | ni | rho/rac GTPase guanine nucleotide exchange factor smgGDS/Vi | um05290 | 0.00375 | ni | putative protein |
| 22d00155 | 0.04203 | ni | amphiphysin | um10266 | 0.00376 | ni | conserved hypothetical protein |
| 27d00048 | 0.04214 | K01694 | tryptophan synthase alpha chain | um03949 | 0.00376 | K01196 | probable 4-alpha-glucanotransferase |
| 11c00086 | 0.04231 | K11135 | telomerase elongation inhibitor | um05522 | 0.00378 | ni | related to suppressor protein PSP1 |
| 11c00010 | 0.04232 | K07300 | Ca2+/H+ antiporter VCX1 | um15053 | 0.0038 | ni | conserved hypothetical Ustilago-specific protein |
| 6d00121 | 0.04251 | ni | cytochrome P450 CYP2 subfamily | um03902 | 0.00386 | K01469 | related to 5-oxoprolinase |
| 7c00125 | 0.04255 | K15100 | mitochondrial tricarboxylate/dicarboxylate carrier proteins | um00737 | 0.00388 | K12830 | probable splicing factor 3B subunit 3 |
| 9d00212 | 0.04256 | ni | hypothetical protein | um02238 | 0.00389 | ni | ni |
| 7d00289 | 0.04263 | K12613 | decapping enzyme complex | um02668 | 0.0039 | ni | conserved hypothetical protein |
| 9d00180 | 0.04278 | K14950 | P-type ATPase | um03480 | 0.0039 | ni | putative protein |
| 14c00053 | 0.04283 | K15620 | golgi protein | um11660.2 | 0.0039 | ni | related to CAT2 - Carnitine O-acetyltransferase |
| 7d00244 | 0.04319 | K13333 | lysophospholipase | um00478 | 0.00392 | ni | hypothetical protein |

| | | | | | | | |
|---|---|---|---|---|---|---|---|
| 14d00067 | 0.0432 | K15083 | nucleotide excision repair protein RAD16 | um02114 | 0.00393 | ni | conserved hypothetical protein |
| 18d00020 | 0.04324 | ni | hypothetical protein | um05206 | 0.00396 | ni | ni |
| 25c00010 | 0.04341 | K01074 | palmitoyl protein thioesterase | um10871 | 0.00396 | K09966 | conserved hypothetical protein |
| 12c00125 | 0.04354 | ni | hypothetical protein | um00072 | 0.00397 | ni | conserved hypothetical protein |
| 4d00013 | 0.04358 | ni | recombination signal binding protein-J kappa | um12204 | 0.00398 | ni | putative protein |
| 9c00114 | 0.04359 | ni | hypothetical protein | um11339 | 0.00402 | ni | related to Siderophore iron transporter 3 |
| 9c00410 | 0.04373 | ni | hypothetical protein | um01051 | 0.00403 | ni | related to mfs-multidrug-resistance transporter |
| 14d00007 | 0.04384 | ni | hypothetical protein | um11087 | 0.00404 | K02216 | related to checkpoint kinase chk1 |
| 11c00079 | 0.04387 | K00164 | 2-oxoglutarate dehydrogenase, E1 subunit | um05410 | 0.00407 | K15178 | conserved hypothetical protein |
| 25d00041 | 0.0439 | K14567 | uncharacterized conserved protein | um02694 | 0.00407 | ni | conserved hypothetical protein |
| 9c00194 | 0.04401 | ni | methyltransferases | um02396 | 0.00408 | ni | conserved hypothetical protein |
| 3d00083 | 0.04405 | ni | ras GTPase-activating protein | um04726 | 0.0041 | K01923 | probable ADE1 - phosphoribosylamidoimidazole-succinocarboxamide synthase |
| 22c00191 | 0.04424 | ni | hypothetical protein | um05398.2 | 0.00414 | ni | putative protein |
| 18d00086 | 0.0446 | ni | hypothetical protein | um01558 | 0.00414 | ni | hypothetical protein |
| 8c00038 | 0.04504 | ni | hypothetical protein | um01623 | 0.00416 | ni | ni |
| 13c00013 | 0.04507 | ni | predicted transporter | um02655 | 0.00417 | ni | related to RNA binding motif protein |
| 15c00053 | 0.04509 | ni | hypothetical protein | um04497 | 0.00417 | K01755 | probable ARG4 - arginosuccinate lyase |
| 12c00110 | 0.04515 | K03263 | translation initiation factor 5A | um02586 | 0.00418 | ni | related to cleft lip and palate transmembrane protein 1 |
| 9c00305 | 0.04521 | K00006 | glycerol-3-phosphate dehydrogenase | um03200 | 0.00418 | K08775 | Rad51-associated protein Brh2 |
| 18d00070 | 0.04527 | K04728 | protein kinase ATM/Tel1 | um06346 | 0.00421 | ni | conserved hypothetical protein |
| 22c00118 | 0.04533 | ni | hypothetical protein | um12218 | 0.00422 | K06636 | probable SMC1 - chromosome segregation protein |
| 24c00025 | 0.04547 | K03178 | ubiquitin activating enzyme UBA1 | um12280 | 0.00424 | ni | conserved hypothetical protein |
| 18d00093 | 0.04557 | ni | predicted small molecule transporter | um04190 | 0.00424 | ni | conserved hypothetical protein |
| 9d00094 | 0.04578 | K11292 | transcription elongation factor SPT6 | um10396.2 | 0.00425 | K10848 | related to RAD1 - component of the nucleotide excision repairosome |
| 9c00079 | 0.04597 | ni | hypothetical protein | um04772 | 0.00426 | ni | hypothetical protein |
| 12c00013 | 0.046 | ni | coeffector of mDia Rho GTPase | um02678 | 0.00428 | K07095 | related to VPS29 - involved in vacuolar protein sorting |
| 27d00055 | 0.04611 | ni | mitochondrial import inner membrane translocase, subunit TIM44 | um05184 | 0.00429 | ni | conserved hypothetical protein |
| 21d00004 | 0.04619 | ni | hypothetical protein | um03616 | 0.00433 | ni | related to DCR2 - dosage-dependent cell cycle regulator |
| 16d00002 | 0.04621 | ni | hypothetical protein | um05300 | 0.00435 | ni | conserved hypothetical Ustilago-specific protein |
| 6d00124 | 0.04648 | K14408 | mRNA cleavage and polyadenylation factor I complex, subunit RN | um10893 | 0.00435 | ni | conserved hypothetical protein |
| 26d00084 | 0.04656 | K02148 | vacuolar H+-ATPase V1 sector, subunit C | um06111 | 0.00436 | K00031 | probable IDP2 - isocitrate dehydrogenase |
| 10d00079 | 0.04667 | K01802 | cyclophilin type peptidyl-prolyl cis-trans isomerase | um05341 | 0.00438 | ni | hypothetical protein |
| 10d00074 | 0.04696 | K12854 | RNA helicase BRR2 | um02285 | 0.00438 | ni | related to Na |
| 9d00315 | 0.04705 | ni | hypothetical protein | um00869 | 0.00443 | ni | conserved hypothetical protein |
| 25c00068 | 0.04721 | K03522 | electron transfer flavoprotein, alpha subunit | um06167 | 0.00443 | K01809 | probable PMI40 - mannose-6-phosphate isomerase |
| 9d00035 | 0.04733 | ni | predicted small molecule transporter | um15001 | 0.00445 | K16369 | related to CHO2 - phosphatidylethanolamine N-methyltransferase |
| 22c00186 | 0.04745 | ni | hypothetical protein | um10953 | 0.00447 | K03671 | probable TRX2 - thioredoxin II |
| 10c00062 | 0.04754 | ni | hypothetical protein | um05908 | 0.00451 | ni | conserved hypothetical protein |
| 5c00007 | 0.04763 | ni | hypothetical protein | um06285 | 0.00452 | K01956 | related to carbamoyl-phosphate synthase small chain |
| 4d00023 | 0.04768 | ni | dual-specificity tyrosine-phosphorylation regulated kinase | um00721 | 0.00453 | ni | related to MAPKK kinase |
| 3c00037 | 0.04802 | K08869 | predicted unusual protein kinase | um11210 | 0.00453 | K00031 | probable IDP1 - isocitrate dehydrogenase |
| 7c00229 | 0.04803 | ni | golgi reassembly stacking protein GRASP65 | um10659 | 0.00455 | K00814 | probable alt1 - alanine aminotransferase |
| 12c00141 | 0.0483 | K12572 | poly(a) ribonuclease subunit | um00706 | 0.00456 | ni | hypothetical protein |
| 18c00079 | 0.04848 | K01807 | ribose 5-phosphate isomerase | um02825 | 0.00456 | ni | putative protein |
| 24c00005 | 0.04863 | K01599 | uroporphyrinogen decarboxylase | um05925 | 0.00457 | ni | related to TOK1 - Voltage-gated |
| 19d00070 | 0.04869 | ni | hypothetical protein | um10446 | 0.00458 | ni | putative protein |
| 7c00051 | 0.04872 | K06126 | monooxygenase | um03487 | 0.00466 | ni | putative protein |
| 9d00150 | 0.04875 | ni | hypothetical protein | um05592 | 0.00479 | ni | conserved hypothetical protein |
| 9c00261 | 0.04877 | ni | FOG, WD40 repeat | um02903 | 0.00482 | K03022 | related to RPC25 - DNA-direcred RNA polymerase III |
| 7c00276 | 0.04889 | K03678 | exosomal 3'-5' exoribonuclease complex, subunit Rrp45 | um03278 | 0.00486 | K15620 | probable VPS74 - protein involved in protein-vacuolar targeting |
| 10c00083 | 0.04895 | ni | predicted phospholipase | um02465 | 0.00488 | ni | related to Calcium influx promoting protein ehs1 |
| 19d00089 | 0.04896 | K11650 | SWI/SNF transcription activation complex subunit | um11054 | 0.00489 | K09568 | probable FPR1 - peptidyl-prolyl cis-trans isomerase |
| 5d00073 | 0.04912 | ni | uncharacterized conserved protein | um06070 | 0.00503 | K01507 | probable IPP1 - inorganic pyrophosphatase |
| 12c00045 | 0.04913 | K11885 | DNA damage inducible protein | um02905 | 0.00504 | K03028 | probable RPN1 - 26S proteasome regulatory subunit |
| 9c00294 | 0.04951 | ni | WD40 repeat-containing protein | um04076 | 0.00505 | ni | ni |
| 7c00090 | 0.04983 | ni | hypothetical protein | um04427 | 0.00507 | ni | hypothetical protein |
| 7c00034 | 0.04991 | ni | hypothetical protein | um11363 | 0.00507 | K00793 | related to RIB5 - riboflavin synthase |
| 14d00051 | 0.04994 | ni | hypothetical protein | um05543 | 0.00509 | K08838 | Ste20-like kinase Don3 |
| 20d00020 | 0.04995 | K00888 | phosphatidylinositol 4-kinase | um11800 | 0.00509 | ni | probable heat shock protein Hsp88 |
| 9d00195 | 0.04996 | ni | hypothetical protein | um01831 | 0.00511 | ni | conserved hypothetical protein |
| 8d00098 | 0.04997 | K11684 | transcription initiation factor TFIID, subunit BDF1 and related bro | um10239 | 0.00512 | K12897 | related to Transformer-2 protein |
| 5c00081 | 0.05 | ni | FOG, Leucine rich repeat | um06205 | 0.00514 | ni | related to Alpha-1 |
| 16d00009 | 0.05005 | ni | FOG, Zn-finger | um04305 | 0.00514 | ni | probable AMS1 - alpha-mannosidase |
| 5c00086 | 0.05008 | ni | hypothetical protein | um11458 | 0.00514 | ni | related to trimethyllysine hydroxylase |
| 25c00041 | 0.05009 | ni | predicted membrane protein | um10157 | 0.00516 | K01756 | probable adenylosuccinate lyase |
| 22c00152 | 0.0501 | ni | hypothetical protein | um04414 | 0.00517 | ni | putative protein |
| 11d00017 | 0.05012 | K01733 | pyridoxalphosphate-dependent enzyme | um05835 | 0.00521 | K06674 | probable SMC2 - chromosome segregation protein |
| 9c00430 | 0.05019 | ni | tryptophan-rich basic nuclear protein | um05122 | 0.00525 | K08178 | related to carboxylic acid transport protein JEN1 |
| 22d00276 | 0.0504 | ni | predicted transporter | um11353 | 0.00527 | K01900 | probable beta-succinyl CoA synthetase precursor |
| 15d00019 | 0.05054 | ni | hypothetical protein | um03521 | 0.00527 | ni | putative protein |
| 5c00012 | 0.05057 | K10706 | tRNA-splicing endonuclease positive effector | um03852 | 0.00527 | K02685 | related to PRI2 - DNA-directed DNA polymerase alpha |
| 9c00026 | 0.05068 | ni | monodehydroascorbate/ferredoxin reductase | um11823 | 0.00528 | ni | hypothetical protein |
| 13d00025 | 0.05084 | K00418 | ubiquinol cytochrome c reductase, subunit QCR8 | um00125 | 0.0053 | ni | conserved hypothetical protein |
| 7c00055 | 0.05097 | K03661 | vacuolar H+-ATPase V0 sector, subunit c'' | um15101 | 0.00533 | ni | putative protein |
| 10c00008 | 0.05102 | K14312 | nuclear pore complex, Nup155 component | um02254 | 0.00538 | ni | hypothetical protein |
| 22d00088 | 0.05119 | ni | hypothetical protein | um04244 | 0.00538 | K17415 | conserved hypothetical protein |
| 7d00090 | 0.05137 | ni | hypothetical protein | um00647 | 0.00538 | ni | conserved hypothetical protein |
| 22d00019 | 0.05152 | K07393 | predicted glutathione S-transferase | um05160 | 0.00538 | K01870 | probable ILS1 - isoleucyl-tRNA synthetase |
| 22c00203 | 0.05153 | K01078 | lysosomal & prostatic acid phosphatases | um00211 | 0.00541 | K11557 | conserved hypothetical protein |
| 9c00133 | 0.05171 | ni | hypothetical protein | um12126 | 0.00545 | ni | putative protein |
| 9c00243 | 0.05177 | ni | hypothetical protein | um04688 | 0.00546 | ni | conserved hypothetical protein |
| 9d00243 | 0.05187 | ni | hypothetical protein | um11941 | 0.00547 | K12190 | related to VPS36 protein |
| 8c00023 | 0.0519 | K15455 | uncharacterized conserved protein | um00026 | 0.00547 | K14826 | related to FK506-binding protein |
| 22d00175 | 0.05192 | ni | hypothetical protein | um00961 | 0.0055 | ni | conserved hypothetical protein |
| 8d00039 | 0.05207 | K06110 | exocyst complex subunit SEC6 | um01905 | 0.0055 | K05609 | probable ubiquitin thiolesterase L3 |
| 10d00005 | 0.05217 | ni | hypothetical protein | um10797 | 0.00554 | K01103 | related to 6-phosphofructo-2-kinase |
| 16c00011 | 0.05221 | ni | FOG, TPR repeat | um04954.2 | 0.00554 | ni | putative protein |
| 11c00063 | 0.05244 | ni | phosphatidylinositol transfer protein PDR16 and related proteins | um04171 | 0.00558 | ni | related to ROT1 - molecular chaperone in the endoplasmic reticulum |
| 5c00100 | 0.05247 | ni | vacuolar sorting protein VPS1 | um02381 | 0.0056 | ni | conserved hypothetical protein |
| 10c00067 | 0.05257 | K08838 | serine/threonine protein kinase | um03543 | 0.0056 | ni | related to YNG2 - component of NuA4 histone acetyltransferase complex |
| 5c00056 | 0.05303 | ni | uncharacterized conserved protein | um12009 | 0.00561 | ni | conserved hypothetical protein |
| 2c00025 | 0.05313 | ni | hypothetical protein | um04307 | 0.00564 | ni | putative protein |
| 26d00004 | 0.05316 | K00987 | mRNA capping enzyme, guanylyltransferase | um06027.2 | 0.00567 | K01892 | probable HTS1 - histidine--tRNA ligase |
| 13d00086 | 0.05323 | ni | hypothetical protein | um12050 | 0.00568 | K02138 | probable ATP7 - F1F0-ATPase complex |
| 9c00148 | 0.05359 | ni | fasciclin and related adhesion glycoproteins | um02133 | 0.00568 | K15634 | conserved hypothetical protein |
| 2d00011 | 0.05384 | K01869 | leucyl-trna synthetase | um03081 | 0.00569 | K08853 | related to ARK1 - Actin Regulating Kinase |
| 12c00039 | 0.05389 | K06067 | histone deacetylase complex, catalytic component RPD3 | um06105 | 0.0057 | K00162 | probable PDB1 - pyruvate dehydrogenase |
| 15d00039 | 0.05398 | K12837 | splicing factor U2AF, large subunit | um02529 | 0.00575 | ni | conserved hypothetical protein |
| 9d00111 | 0.05412 | ni | hypothetical protein | um11359 | 0.00575 | ni | related to BOR1 - boron efflux transporter |
| 11c00033 | 0.05432 | K11713 | protein geranylgeranyltransferase Type I, beta subunit | um01597 | 0.00578 | ni | hypothetical protein |
| 10d00107 | 0.05475 | ni | golgi proteins involved in ER retention | um10088 | 0.00579 | K01907 | related to Acetoacetyl-CoA synthetase |
| 20d00082 | 0.05485 | ni | hypothetical protein | um06497 | 0.00581 | ni | putative protein |
| 7d00145 | 0.05488 | ni | hypothetical protein | um10656 | 0.00588 | K02684 | related to DNA primase 48K protein PRI1 |
| 4d00045 | 0.05492 | ni | hypothetical protein | um05764 | 0.00588 | ni | related to Malic acid transport protein |
| 16d00066 | 0.05494 | ni | PHD finger protein BR140/LIN-49 | um04193.2 | 0.0059 | K17053 | probable ABP140 - actin filament-binding protein |
| 11d00035 | 0.05507 | K11436 | protein arginine N-methyltransferase PRMT1 and related enzyme | um01462 | 0.00593 | ni | hypothetical protein |
| 12d00069 | 0.05514 | ni | WD40 repeat-containing protein | um11246 | 0.00597 | ni | related to BZZ1 - Myo3 |
| 9d00254 | 0.05518 | K12493 | predicted GTPase-activating protein | um06002 | 0.00599 | ni | hypothetical protein |
| 26c00011 | 0.05522 | K00927 | 3-phosphoglycerate kinase | um01737.2 | 0.00599 | K07036 | related to Adiponectin receptor 1 |
| 22d00140 | 0.05529 | ni | predicted lipase | um00526 | 0.00601 | K06883 | conserved hypothetical protein |
| 7c00127 | 0.05558 | ni | hypothetical protein | um11957 | 0.00601 | ni | related to histidine kinase |
| 7d00052 | 0.05559 | K17408 | mitochondrial ribosome small subunit component | um00755 | 0.00603 | ni | putative protein |
| 11d00065 | 0.05567 | ni | hypothetical protein | um01050 | 0.00604 | ni | siderophore biosynthesis regulatory protein URBS1 |
| 9d00139 | 0.05582 | ni | glutaminyl cyclase | um11238 | 0.00606 | ni | conserved hypothetical protein |
| 22d00249 | 0.056 | K03441 | aquaporin | um02929 | 0.00608 | K04709 | related to longevity-assurance protein LAG1 |
| 2c00040 | 0.05604 | ni | flavin-containing monooxygenase | um11712 | 0.0061 | ni | putative protein |
| 9c00102 | 0.05605 | ni | acetylcholinesterase | um02843 | 0.00614 | K01341 | probable KEX2 - endoproteinase of late golgi compartment |
| 2c00052 | 0.05619 | ni | hypothetical protein | um04406 | 0.00625 | K00586 | probable DPH5 - diphthamide methyltransferase |
| 5c00057 | 0.05624 | ni | hypothetical protein | um02425 | 0.00626 | K12581 | probable CCR4-NOT transcription complex |
| 22c00254 | 0.05627 | ni | synaptic vesicle transporter SVOP and related transporters | um11161 | 0.00626 | K00026 | probable MDH1 - malate dehydrogenase precursor |
| 10c00063 | 0.05632 | K00463 | hypothetical protein | um04851 | 0.00627 | K03564 | conserved hypothetical protein |
| 7d00066 | 0.05649 | ni | vacuolar sorting protein VPS45/Stt10 | um06018.2 | 0.00632 | ni | conserved hypothetical protein |
| 14c00031 | 0.05657 | K00288 | C1-tetrahydrofolate synthase | um00078 | 0.00635 | ni | phenylalanine ammonia-lyase |
| 9d00250 | 0.05664 | ni | hypothetical protein | um03165 | 0.00636 | ni | ni |
| 7c00014 | 0.05667 | K12761 | serine/threonine protein kinase | um15047 | 0.00638 | ni | probable VPS1 - member of the dynamin family of GTPases |
| 10c00084 | 0.05667 | ni | hypothetical protein | um02046 | 0.00644 | K02728 | probable PRE9 - 20S proteasome subunit Y13 |
| 18d00064 | 0.05669 | K12815 | mRNA splicing factor ATP-dependent RNA helicase | um11084.2 | 0.00644 | K05012 | probable voltage-gated chloride channel |
| 10c00034 | 0.05691 | ni | hypothetical protein | um00833 | 0.00649 | K03066 | probable RPT6 - 26S proteasome regulatory subunit |
| 3c00014 | 0.05694 | ni | hypothetical protein | um10493 | 0.00655 | ni | related to aminopeptidase |
| 7d00156 | 0.05697 | ni | exosomal 3'-5' exoribonuclease complex, subunit Rrp44/Dis3 | um04194 | 0.00656 | K13120 | conserved hypothetical protein |
| 26d00026 | 0.05708 | ni | uncharacterized conserved protein | um00481 | 0.00657 | K13280 | probable signal peptidase |
| 19c00008 | 0.05708 | ni | uncharacterized conserved protein | um04740 | 0.00661 | ni | conserved hypothetical protein |
| 22d00169 | 0.05715 | ni | hypothetical protein | um04191 | 0.00661 | K13338 | probable PEX1 - peroxisomal assembly protein - peroxin |
| 9c00404 | 0.05718 | ni | hypothetical protein | um01364 | 0.00664 | K07936 | probable GSP1 - GTP-binding protein of the ras superfamily |
| 11d00045 | 0.05724 | ni | hypothetical protein | um04602 | 0.00664 | K14429 | related to bumetanide-sensitive Na-K-Cl cotransport protein |
| 8c00105 | 0.05727 | ni | hypothetical protein | um01818 | 0.00665 | K16219 | conserved hypothetical protein |
| 6c00093 | 0.05735 | ni | hypothetical protein | um01962 | 0.00668 | ni | related to GAL1 - galactokinase |
| 22c00134 | 0.05736 | K02541 | DNA replication licensing factor, MCM3 component | um12261 | 0.00668 | ni | related to lipase |
| 24d00038 | 0.05756 | ni | hypothetical protein | um05023 | 0.00671 | ni | probable monosaccharide transporter |
| 22d00127 | 0.05756 | ni | hypothetical protein | um11275 | 0.00671 | K00102 | related to D-lactate dehydrogenase |
| 2d00068 | 0.05762 | ni | hypothetical protein | um05040 | 0.00672 | K02993 | probable 40S ribosomal protein S7 |
| 10c00058 | 0.05778 | K01897 | acyl-coa synthetase | um10059 | 0.00676 | ni | conserved hypothetical protein |
| 22c00129 | 0.0578 | K06944 | GTP-binding protein DRG2 | um12200 | 0.00677 | ni | conserved hypothetical protein |
| 26d00083 | 0.05787 | ni | hypothetical protein | um04728 | 0.00681 | ni | hypothetical protein |
| 15c00054 | 0.05791 | ni | hypothetical protein | um06097 | 0.00683 | ni | putative protein |
| 13d00102 | 0.05802 | ni | hypothetical protein | um12043 | 0.00683 | ni | hypothetical protein |
| 19d00137 | 0.05833 | K10901 | ATP-dependent DNA helicase | um02744 | 0.00686 | ni | related to Iron transport protein 1 |
| 5c00041 | 0.05839 | ni | hypothetical protein | um11067 | 0.00687 | K03782 | probable catalase 2 |
| 12c00106 | 0.05848 | K13339 | peroxisome assembly factor 2 containing the AAA+-type ATPase | um06185 | 0.00687 | K00257 | probable acyl-CoA dehydrogenase short-branched chain precursor |
| 7d00251 | 0.0585 | K12399 | clathrin adaptor complex, small subunit | um10499 | 0.00695 | ni | conserved hypothetical protein |
| 10c00012 | 0.05852 | ni | uncharacterized conserved protein | um10124 | 0.00702 | K15323 | related to RNA splicing endonuclease gamma subunit |
| 25c00083 | 0.05856 | ni | mitochondrial/chloroplast ribosomal protein L2 | um10504 | 0.00705 | K11339 | related to Chromo domain protein MRG15 |
| 7d00321 | 0.05867 | ni | hypothetical protein | um11927 | 0.00706 | ni | related to ROT1 - molecular chaperone in the endoplasmic reticulum |
| 14d00019 | 0.05871 | ni | hypothetical protein | um03737 | 0.00708 | K01897 | related to long-chain-fatty-acid-CoA ligase |
| 1d00010 | 0.05885 | ni | hypothetical protein | um10836 | 0.00709 | ni | protein mediates microtubule-dependent mRNA transport |
| 9c00400 | 0.05899 | K00077 | hypothetical protein | um01854 | 0.00709 | K01113 | conserved hypothetical protein |

| | | | | | | | |
|---|---|---|---|---|---|---|---|
| 27d00064 | 0.05905 | ni | flavohemoprotein b5+b5R | um01293 | 0.0071 | K03510 | related to DNA Polymerase iota |
| 12c00137 | 0.05919 | K10908 | mitochondrial/chloroplast DNA-directed RNA polymerase RPO41 | um01184 | 0.00711 | K03020 | related to Rpc19 - DNA-directed RNA polymerases I and III |
| 16d00060 | 0.05923 | K03262 | translation initiation factor 5 | um03789 | 0.00711 | ni | putative protein |
| 9c00030 | 0.05924 | ni | hypothetical protein | um10620 | 0.00714 | K03132 | related to TAF7 - TFIID subunit |
| 8d00013 | 0.05935 | ni | hypothetical protein | um11349 | 0.0072 | ni | conserved hypothetical protein |
| 9c00290 | 0.05938 | K03505 | hypothetical protein | um10940 | 0.00721 | ni | related to ROT1 - molecular chaperone in the endoplasmic reticulum |
| 14c00007 | 0.05939 | K14550 | uncharacterized conserved protein | um04329.2 | 0.00721 | ni | conserved hypothetical protein |
| 16d00063 | 0.05958 | ni | global transcriptional regulator | um06273 | 0.00721 | K00327 | probable NADPH-cytochrome P450 reductase |
| 25c00033 | 0.05976 | K12272 | signal recognition particle receptor, beta subunit | um05104 | 0.00723 | ni | putative protein |
| 5c00084 | 0.05986 | K14962 | histone H3 (Lys4) methyltransferase complex and RNA cleavage | um00883 | 0.00726 | ni | related to KCS1 - potential transcription factor of the BZIP type |
| 12d00059 | 0.05988 | ni | uncharacterized conserved protein | um01048 | 0.0073 | K01581 | ornithine decarboxylase |
| 9d00342 | 0.05991 | K07573 | exosomal 3'-5' exoribonuclease complex, subunit ski4 | um02142 | 0.00731 | ni | related to muconate cycloisomerase |
| 1c00028 | 0.05996 | ni | uncharacterized conserved protein, AMMECR1 | um02976.2 | 0.00737 | ni | conserved hypothetical protein |
| 27d00052 | 0.06011 | K12571 | PAB-dependent poly(A) ribonuclease, subunit PAN2 | um10524 | 0.00743 | K03260 | related to TIF4631 - mRNA cap-binding protein |
| 6c00034 | 0.06016 | ni | hypothetical protein | um12297 | 0.00744 | ni | conserved hypothetical protein |
| 7c00216 | 0.06035 | K00022 | 3-hydroxyacyl-CoA dehydrogenase | um00081 | 0.00745 | ni | putative protein |
| 19d00117 | 0.06056 | ni | vacuolar sorting protein VPS1 | um03496 | 0.00749 | K02213 | related to Cell division control protein 18 |
| 18c00069 | 0.06057 | K11559 | hypothetical protein | um00297 | 0.00753 | K13345 | related to Peroxisome assembly protein 12 |
| 8d00083 | 0.06063 | K01537 | calcium transporting ATPase | um03597 | 0.00755 | ni | conserved hypothetical protein |
| 4c00015 | 0.06072 | ni | predicted hydrolases or acyltransferases | um03853 | 0.00759 | K14312 | related to NUP170 - nuclear pore protein |
| 15c00017 | 0.06123 | ni | hypothetical protein | um05399 | 0.00761 | ni | hypothetical protein |
| 19d00047 | 0.06129 | ni | hypothetical protein | um02542 | 0.00762 | ni | hypothetical protein |
| 12c00027 | 0.06131 | ni | hypothetical protein | um11769 | 0.00764 | K12812 | probable SUB2 - mRNA export protein |
| 6c00129 | 0.06152 | ni | hypothetical protein | um11263 | 0.00764 | ni | putative protein |
| 3d00067 | 0.06152 | ni | hypothetical protein | um00083 | 0.00765 | K02598 | related to formate |
| 24c00016 | 0.06161 | K01809 | mannose-6-phosphate isomerase | um02602 | 0.0077 | ni | hypothetical protein |
| 22d00231 | 0.06169 | K15151 | transcription factor, subunit of SRB subcomplex of RNA polymera | um10515 | 0.0077 | ni | conserved hypothetical protein |
| 7d00219 | 0.06169 | ni | exocyst protein Sec3 | um02763 | 0.00774 | ni | conserved hypothetical protein |
| 22d00072 | 0.06186 | ni | GPI transamidase complex, GPI17/PIG-S component | um04542 | 0.00777 | K10577 | probable ubiquitin--protein ligase hus5 |
| 22d00012 | 0.06191 | K14835 | tRNA and rRNA cytosine-C5-methylase | um02154 | 0.00784 | ni | related to amino-acid permease 2 |
| 25d00077 | 0.06193 | ni | ATP-dependent RNA helicase A | um04914 | 0.00794 | K11271 | conserved hypothetical protein |
| 9c00250 | 0.06199 | ni | ubiquitin-conjugating enzyme-related protein Ft1 | um11588 | 0.00794 | K14686 | related to copper transport protein |
| 22c00272 | 0.06205 | K02350 | DNA polymerase zeta, catalytic subunit | um06040 | 0.00795 | ni | conserved hypothetical protein |
| 26d00044 | 0.0621 | K14823 | nucleolar protein-like/EBNA1-binding protein | um10980 | 0.00798 | ni | conserved hypothetical Ustilago-specific protein |
| 7c00190 | 0.06222 | K14556 | WD40-repeat-containing subunit of the 18S rRNA processing co | um02024 | 0.00801 | K03327 | conserved hypothetical protein |
| 1c00027 | 0.06224 | ni | GTPase Rab5/YPT51 | um01445.2 | 0.00804 | K02540 | probable DNA replication licensing factor |
| 25c00036 | 0.06236 | K07565 | ribosome biogenesis protein NIP7 | um04268 | 0.00805 | K00290 | probable saccharopine dehydrogenase |
| 16c00065 | 0.0624 | K00799 | hypothetical protein | um11427 | 0.00808 | K12868 | related to SYF2 - pre-mRNA-splicing factor |
| 12c00058 | 0.06242 | K15423 | serine/threonine specific protein phosphatase | um00549 | 0.00809 | ni | related to UTR1 |
| 22d00224 | 0.06243 | ni | putative N2 | um04150 | 0.0081 | ni | related to DOP1 - strong similarity to developmental regulatory gene |
| 13c00065 | 0.06256 | K00589 | uroporphyrin III methyltransferase | um02457 | 0.0081 | K03217 | related to OXA1 - cytochrome oxidase biogenesis protein |
| 12d00116 | 0.06288 | ni | uncharacterized conserved protein | um01823 | 0.00813 | ni | hypothetical protein |
| 3d00030 | 0.06296 | ni | hypothetical protein | um04352 | 0.00813 | K06685 | probable MOB1 protein |
| 11d00031 | 0.06302 | ni | uncharacterized conserved protein | um00710 | 0.00816 | ni | related to Exocyst complex component Sec5 |
| 9c00334 | 0.06307 | K14566 | predicted nucleic-acid-binding protein | um01532 | 0.0082 | ni | putative protein |
| 22d00122 | 0.06309 | ni | hypothetical protein | um03714 | 0.00825 | ni | related to lactose regulatory protein |
| 12d00067 | 0.06328 | ni | diadenosine and diphosphoinositol polyphosphate phosphohydrol | um10417 | 0.00825 | K09184 | related to transcription factor ScGATA-6 |
| 13d00041 | 0.06333 | ni | hypothetical protein | um03884 | 0.00828 | ni | conserved hypothetical protein |
| 6d00096 | 0.06357 | ni | hypothetical protein | um11970 | 0.00832 | ni | putative protein |
| 6c00126 | 0.06363 | ni | hypothetical protein | um04019 | 0.00844 | ni | putative protein |
| 14d00043 | 0.06382 | ni | hypothetical protein | um11456 | 0.00848 | K05293 | related to GPI - transamidase subunit |
| 7c00239 | 0.06386 | K02516 | protein kinase inhibitor | um00304 | 0.00848 | K01657 | probable TRP2 - anthranilate synthase component I |
| 8d00116 | 0.0639 | ni | fumarylacetoacetase | um05447 | 0.00849 | ni | conserved hypothetical protein |
| 16d00074 | 0.064 | ni | component of vacuolar transporter chaperone | um12122 | 0.0085 | K15687 | related to makorin ring zinc finger protein |
| 4c00009 | 0.06401 | K03469 | hypothetical protein | um04126 | 0.00853 | ni | conserved hypothetical protein |
| 25d00063 | 0.06407 | ni | aspartyl protease | um03864 | 0.00854 | ni | related to GTPase-activating protein beta-chimerin |
| 19c00112 | 0.0641 | K02930 | ribosomal protein RPL1/RPL2/RL4L4 | um04308 | 0.00854 | ni | conserved hypothetical protein |
| 10d00029 | 0.06411 | K06215 | stationary phase-induced protein | um01738 | 0.00856 | ni | putative protein |
| 7d00155 | 0.06411 | ni | hypothetical protein | um02577 | 0.00862 | K00033 | probable 6-phosphogluconate dehydrogenase |
| 5c00062 | 0.06417 | ni | hypothetical protein | um04988 | 0.00862 | K15110 | probable ODC2 - Mitochondrial 2-oxodicarboxylate carrier |
| 7d00088 | 0.06423 | ni | hypothetical protein | um02068 | 0.00865 | ni | hypothetical protein |
| 9d00146 | 0.06432 | ni | hypothetical protein | um03622 | 0.00866 | K10258 | related to TSC13 - Enoyl reductase involved in very long chain fatty acid elongation |
| 22c00077 | 0.06433 | ni | enoyl-coa hydratase | um05365 | 0.00867 | K04354 | probable protein phosphatase 2A regulatory B subunit |
| 2c00086 | 0.0645 | ni | hypothetical protein | um04666 | 0.00869 | K17362 | conserved hypothetical protein |
| 7d00176 | 0.0646 | ni | hypothetical protein | um05731 | 0.00871 | ni | conserved hypothetical protein |
| 18d00055 | 0.06474 | ni | hypothetical protein | um05644 | 0.00873 | ni | related to alcohol dehydrogenase |
| 7d00122 | 0.06475 | ni | predicted N6-adenine methylase involved in transcription regulatic | um10308 | 0.00882 | K00620 | related to ECM40 - acetylornithine acetyltransferase |
| 7d00260 | 0.06486 | ni | hypothetical protein | um02246 | 0.00885 | ni | conserved hypothetical protein |
| 9c00138 | 0.06497 | ni | hypothetical protein | um11687 | 0.00885 | ni | putative protein |
| 13d00015 | 0.06508 | K10846 | 5'-3' exonuclease | um00576 | 0.00899 | K12592 | conserved hypothetical protein |
| 27d00033 | 0.06511 | ni | myosin class V heavy chain | um15077 | 0.00899 | ni | putative protein |
| 27c00027 | 0.06572 | K16578 | hypothetical protein | um11231 | 0.00905 | K01814 | probable bifunctional protein |
| 26c00055 | 0.06584 | ni | cyclin B and related kinase-activating proteins | um04362 | 0.00905 | K07431 | related to Cytochrome P450 8B1 |
| 22d00170 | 0.06588 | K01785 | predicted mutarotase | um02491 | 0.00907 | K00134 | glyceraldehyde 3-phosphate dehydrogenase |
| 9d00099 | 0.06592 | K13106 | uncharacterized conserved protein | um00676 | 0.00909 | K08869 | conserved hypothetical protein |
| 3d00045 | 0.06593 | K03104 | hypothetical protein | um10870 | 0.00913 | K01792 | conserved hypothetical protein |
| 6d00044 | 0.06599 | ni | transferrin receptor and related proteins | um11192 | 0.00915 | ni | hypothetical protein |
| 4d00040 | 0.06611 | ni | phosphatidylinositol 4-kinase | um03726.2 | 0.00916 | K01802 | probable CPR1 - cyclophilin |
| 9c00120 | 0.06641 | K00490 | cytochrome P450 CYP4/CYP19/CYP26 subfamilies | um06391 | 0.00919 | ni | related to multidrug resistant protein |
| 9c00401 | 0.06644 | K14774 | uncharacterized conserved protein | um11071 | 0.00921 | ni | conserved hypothetical protein |
| 25d00084 | 0.06657 | ni | hypothetical protein | um10633 | 0.00921 | ni | conserved hypothetical protein |
| 18c00013 | 0.06661 | ni | hypothetical protein | um01968 | 0.00922 | ni | conserved hypothetical protein |
| 7c00171 | 0.0668 | K03097 | casein kinase II, alpha subunit | um03304 | 0.00923 | ni | conserved hypothetical protein |
| 7c00223 | 0.06707 | K10866 | DNA repair protein RAD50 | um02632 | 0.00927 | ni | probable nudC protein |
| 14d00064 | 0.06711 | ni | hypothetical protein | um04138 | 0.00929 | K00616 | probable TAL1 - transaldolase |
| 14c00032 | 0.06743 | ni | hypothetical protein | um10436 | 0.0093 | ni | conserved hypothetical protein |
| 3d00077 | 0.06763 | K11805 | conserved WD40 repeat-containing protein AN11 | um04394 | 0.00934 | K14802 | probable P-type ATPase |
| 9c00007 | 0.06769 | ni | hypothetical protein | um01228 | 0.00935 | K15029 | related to eIF3l - translation initiation factor 3 subunit L |
| 9d00080 | 0.06772 | K14729 | peroxisomal multifunctional beta-oxidation protein and related enz | um10823 | 0.00936 | ni | conserved hypothetical protein |
| 27d00092 | 0.06782 | ni | hypothetical protein | um11575 | 0.00936 | K01607 | conserved hypothetical protein |
| 19d00018 | 0.06786 | ni | hypothetical protein | um11946 | 0.00937 | ni | probable UDP-galactopyranose mutase |
| 6d00107 | 0.06811 | K03350 | DNA-binding cell division cycle control protein | um04423 | 0.00945 | ni | related to potassium transporter TRK-1 |
| 2c00055 | 0.06813 | K01090 | serine/threonine specific protein phosphatase | um10327 | 0.00953 | ni | putative protein |
| 9d00209 | 0.06836 | K02960 | 40S ribosomal protein S16 | um10755 | 0.00955 | ni | conserved hypothetical protein |
| 5c00098 | 0.06839 | K03128 | TATA binding protein associated factor | um05756.2 | 0.00962 | K10885 | related to ATP-dependent DNA helicase II |
| 2d00010 | 0.06841 | K05755 | actin-related protein Arp2/3 complex, subunit ARPC4 | um05755.2 | 0.00965 | ni | conserved hypothetical protein |
| 12d00108 | 0.06848 | ni | hypothetical protein | um05113.2 | 0.00966 | K01754 | probable ILV1 - anabolic serine and threonine dehydratase precursor |
| 5c00006 | 0.06856 | K03948 | hypothetical protein | um12038 | 0.00967 | ni | probable GOT1 - Membrane protein required for ER to Golgi transport |
| 14d00045 | 0.06865 | K04482 | DNA repair protein RAD51/RHP55 | um00776 | 0.00967 | ni | conserved hypothetical Ustilago-specific protein |
| 6d00075 | 0.06882 | K03885 | NADH-dehydrogenase | um01948 | 0.00975 | ni | hypothetical protein |
| 16c00033 | 0.06888 | ni | FOG, TPR repeat | um01996 | 0.00975 | ni | probable mfs-multidrug-resistance transporter |
| 15d00056 | 0.06893 | ni | hypothetical protein | um11451 | 0.00976 | K12179 | related to COP9 signalosome complex subunit 6 |
| 3c00069 | 0.06895 | ni | hypothetical protein | um11724 | 0.00979 | K10847 | related to DNA repair protein RAD14 |
| 13d00092 | 0.06896 | K14400 | mRNA cleavage and polyadenylation factor I/II complex, subunit F | um03842 | 0.0098 | K12867 | conserved hypothetical protein |
| 12c00090 | 0.06896 | ni | hypothetical protein | um02318.2 | 0.0098 | ni | conserved hypothetical protein |
| 3d00057 | 0.06903 | K14799 | uncharacterized conserved protein | um05604 | 0.00981 | ni | putative protein |
| 22c00026 | 0.06906 | ni | protein phosphatase 1, regulatory subunit, and related proteins | um04397 | 0.00981 | ni | related to importin beta-2 subunit |
| 8c00054 | 0.0694 | K01874 | methionyl-trna synthetase | um11452 | 0.00982 | ni | conserved hypothetical protein |
| 19d00125 | 0.06941 | ni | hypothetical protein | um10016 | 0.00987 | ni | conserved hypothetical protein |
| 11d00003 | 0.06946 | ni | hypothetical protein | um03473 | 0.00991 | ni | conserved hypothetical protein |
| 9c00258 | 0.06956 | ni | predicted coiled-coil protein | um01306 | 0.00998 | ni | hypothetical protein |
| 27d00043 | 0.06962 | ni | sexual differentiation process protein ISP4 | um02768 | 0.01006 | ni | conserved hypothetical protein |
| 18d00009 | 0.06984 | K03014 | RNA polymerase subunit K | um05971 | 0.01009 | K01915 | probable glutamine synthetase |
| 20c00021 | 0.07004 | ni | hypothetical protein | um04149 | 0.01012 | ni | conserved hypothetical protein |
| 14d00030 | 0.07023 | K12614 | ATP-dependent RNA helicase | um11500 | 0.01024 | K14688 | related to COT1 - Vacuolar zinc |
| 13c00073 | 0.07025 | ni | hypothetical protein | um11638 | 0.01026 | K00621 | related to glucosamine 6-phosphate n-acetyltransferase |
| 18c00106 | 0.07041 | ni | hypothetical protein | um00884 | 0.01027 | ni | related to Ral2 protein |
| 14d00021 | 0.0705 | ni | hypothetical protein | um04474 | 0.0103 | K04640 | guanine nucleotide-binding protein alpha-3 subunit |
| 9d00100 | 0.07081 | ni | hypothetical protein | um10669 | 0.0103 | ni | conserved hypothetical protein |
| 14d00055 | 0.07118 | ni | hypothetical protein | um00922 | 0.01031 | K03143 | related to TFIIH basal transcription factor complex p34 subunit |
| 2c00041 | 0.07137 | ni | clathrin assembly protein AP180 and related proteins | um11624.2 | 0.01034 | K07342 | related to SSS1 - ER protein-translocase complex subunit |
| 14c00034 | 0.07164 | ni | hypothetical protein | um12224 | 0.01039 | K13103 | related to Tuftelin-interacting protein 11 |
| 22d00131 | 0.07169 | ni | hypothetical protein | um10673 | 0.01044 | ni | conserved hypothetical protein |
| 12d00056 | 0.07174 | ni | hypothetical protein | um04863 | 0.01048 | ni | hypothetical protein |
| 7d00079 | 0.07176 | ni | heat shock transcription factor | um06436 | 0.01048 | ni | ni |
| 9d00124 | 0.07178 | K02942 | 60s acidic ribosomal protein P1 | um04915 | 0.01049 | ni | probable Major allergen Mal f 1 precursor |
| 2d00020 | 0.07185 | K15175 | RNA polymerase II assessory factor Cdc73p | um12143 | 0.0105 | ni | conserved hypothetical protein |
| 3c00067 | 0.07196 | ni | hypothetical protein | um11886 | 0.0105 | ni | conserved hypothetical protein |
| 22d00132 | 0.07221 | K00003 | homoserine dehydrogenase | um11480 | 0.01056 | K14402 | conserved hypothetical protein |
| 10c00018 | 0.07227 | K00411 | ubiquinol cytochrome c reductase, subunit RIP1 | um10039 | 0.01063 | ni | related to MMT1 - Mitochondrial iron transport protein |
| 9d00390 | 0.07239 | ni | hypothetical protein | um03006 | 0.01065 | ni | probable Aldo-keto reductase yakc |
| 26d00053 | 0.0724 | K10903 | checkpoint 9-1-1 complex, HUS1 component | um05063 | 0.01066 | ni | conserved hypothetical protein |
| 7d00205 | 0.07241 | ni | hypothetical protein | um04865 | 0.01066 | K12191 | related to DID4 - class E vacuolar-protein sorting and endocytosis factor |
| 25d00075 | 0.07248 | K10436 | microtubule-binding protein | um00454 | 0.01067 | K05285 | related to MCD4 - sporulation protein |
| 9d00151 | 0.07265 | K01953 | asparagine synthase | um10340 | 0.01069 | ni | conserved hypothetical protein |
| 19c00089 | 0.07306 | K00971 | hypothetical protein | um15060 | 0.01069 | ni | conserved hypothetical protein |
| 9c00165 | 0.07308 | ni | hydrolytic enzymes of the alpha/beta hydrolase fold | um12196 | 0.0107 | ni | conserved hypothetical protein |
| 5c00138 | 0.07339 | K12275 | membrane component of ER protein translocation complex | um03349 | 0.01073 | ni | conserved hypothetical protein |
| 13d00059 | 0.0734 | ni | hypothetical protein | um01016 | 0.0108 | ni | conserved hypothetical protein |
| 16c00020 | 0.07345 | ni | hypothetical protein | um01038 | 0.01082 | ni | conserved hypothetical protein |
| 7c00096 | 0.07347 | K00029 | NADP+-dependent malic enzyme | um03354 | 0.01082 | K11130 | probable NOP10 - nucleolar rRNA processing protein |
| 8c00112 | 0.07357 | K01636 | hypothetical protein | um10286 | 0.01083 | ni | probable NTF2 - nuclear transport factor |
| 22d00198 | 0.0736 | K07195 | exocyst component protein and related proteins | um11713 | 0.01086 | K06677 | related to YCS4 - subunit of condensin protein complex |
| 18c00083 | 0.07363 | ni | hypothetical protein | um12231 | 0.01087 | ni | related to PKR1 - V-ATPase Assembly Factor |
| 9c00221 | 0.074 | ni | MADS box transcription factor | um03282 | 0.01088 | ni | related to MAK32 protein |
| 12c00023 | 0.07432 | K09647 | mitochondrial inner membrane protease, subunit IMP1 | um01347 | 0.01088 | ni | hypothetical protein |
| 8d00111 | 0.07434 | ni | predicted unusual protein kinase | um03958 | 0.01098 | ni | probable FEN1 - fatty acid elongase |
| 22d00192 | 0.07436 | K15918 | predicted kinase | um11662 | 0.011 | K07393 | related to ECM4 - involved in cell wall biogenesis and architecture |
| 25d00085 | 0.07438 | K04487 | cysteine desulfurase NFS1 | um11260 | 0.01101 | K01875 | probable serine-tRNA ligase |
| 9d00384 | 0.07444 | K00938 | phosphomevalonate kinase | um10112 | 0.01103 | ni | conserved hypothetical protein |
| 18d00040 | 0.07452 | K14554 | WD repeat protein | um03446 | 0.01103 | ni | probable protein kinase DBF2 |
| 3d00002 | 0.07476 | ni | aldo/keto reductase family proteins | um02374 | 0.01108 | ni | related to monosaccharide transporter |
| 7d00278 | 0.07485 | ni | hypothetical protein | um03124 | 0.01109 | K03539 | related to Ribonuclease P protein subunit p30 |
| 5d00038 | 0.07524 | K15255 | DNA helicase PIF1/RRM3 | um03610 | 0.01112 | ni | related to YSR3 - dihydrosphingosine-1-phosphate phosphatase |
| 14c00012 | 0.07542 | ni | predicted tubulin-tyrosine ligase | um00372 | 0.01113 | K14950 | probable SPF1 - P-type ATPase |
| 19d00069 | 0.07546 | ni | predicted E3 ubiquitin ligase | um00451 | 0.01115 | ni | conserved hypothetical protein |

| | | | | | | | |
|---|---|---|---|---|---|---|---|
| 18d00101 | 0.07547 | ni | chromatin remodeling complex WSTF-ISWI, small subunit | um10790 | 0.01115 | K11808 | probable phosphoribosyl-5-aminoimidazole carboxylase |
| 2c00031 | 0.07581 | K13237 | reductases with broad range of substrate specificities | um04791 | 0.01116 | ni | related to to G1 |
| 11d00002 | 0.07628 | K12736 | cyclophilin-related peptidyl-prolyl cis-trans isomerase | um02783 | 0.01116 | K00826 | related to branched-chain amino acid aminotransferase |
| 8d00055 | 0.07641 | ni | hypothetical protein | um01877 | 0.01117 | ni | putative protein |
| 6c00051 | 0.07649 | ni | cytochrome P450 CYP3/CYP5/CYP6/CYP9 subfamilies | um01191 | 0.01118 | K02607 | conserved hypothetical protein |
| 10d00070 | 0.07657 | ni | hypothetical protein | um06079 | 0.01119 | K01669 | related to deoxyribodipyrimidine photo-lyase PHR |
| 16d00072 | 0.07671 | ni | hypothetical protein | um03979 | 0.01124 | ni | conserved hypothetical protein |
| 14c00123 | 0.07672 | ni | hypothetical protein | um11752 | 0.01131 | ni | conserved hypothetical protein |
| 19d00001 | 0.07702 | ni | hypothetical protein | um10359 | 0.01132 | K03017 | probable DNA-directed RNA polymerase II 14 |
| 19d00007 | 0.07716 | ni | hypothetical protein | um12154 | 0.01133 | K12600 | related to SKI3 - protein involved in exosome mediated 3 |
| 19d00040 | 0.07748 | K09419 | heat shock transcription factor | um03375 | 0.01134 | ni | putative protein |
| 22c00020 | 0.07797 | K01887 | arginyl-trna synthetase | um05558 | 0.01135 | K01338 | related to ATP-dependent protease La |
| 19d00027 | 0.07819 | K00344 | predicted quinone oxidoreductase | um11669 | 0.01137 | ni | related to syntaxin family member TLG1 |
| 9d00324 | 0.07821 | ni | predicted exonuclease | um01720 | 0.01138 | ni | probable phosphomannomutase |
| 19c00124 | 0.0783 | K15378 | sucrose transporter and related proteins | um10534 | 0.01139 | ni | related to Protein-tyrosine phosphatase |
| 12c00145 | 0.07837 | ni | hypothetical protein | um01265 | 0.01141 | ni | related to RNA-binding protein |
| 7d00135 | 0.07857 | ni | hypothetical protein | um03029 | 0.01145 | ni | conserved hypothetical protein |
| 7d00127 | 0.0787 | K03120 | TATA-box binding protein | um03298 | 0.01148 | K07508 | probable ERG10 - acetyl-CoA C-acetyltransferase |
| 7d00114 | 0.07885 | K02997 | ribosomal protein S4 | um11384 | 0.01167 | ni | probable pterin-4-alpha-carbinolamine dehydratase |
| 24d00048 | 0.07894 | ni | hypothetical protein | um10981 | 0.01175 | ni | conserved hypothetical Ustilago-specific protein |
| 16c00079 | 0.0792 | ni | hypothetical protein | um01403 | 0.01175 | ni | conserved hypothetical protein |
| 19d00139 | 0.07958 | K14801 | hypothetical protein | um00565 | 0.01183 | K09499 | probable CCT7 - component of chaperonin-containing T-complex |
| 6c00036 | 0.07979 | ni | amino acid transporter protein | um03994 | 0.01199 | K01568 | probable PDC1 - pyruvate decarboxylase |
| 19d00005 | 0.07988 | ni | hypothetical protein | um05504 | 0.012 | K01920 | related to glutathione synthase |
| 22c00319 | 0.08006 | ni | phosphoinositide phosphatase SAC1 | um02007 | 0.012 | K01519 | probable HAM1 - protein involved in DNA repair |
| 20c00013 | 0.08033 | ni | hypothetical protein | um11986 | 0.012 | K10405 | c-terminal kinesin |
| 18d00078 | 0.08042 | K03424 | tatd-related DNase | um10636 | 0.01213 | ni | conserved hypothetical protein |
| 19c00049 | 0.08051 | ni | hypothetical protein | um01407 | 0.01216 | K13519 | conserved hypothetical protein |
| 9d00253 | 0.08053 | ni | hypothetical protein | um00560 | 0.01217 | K03083 | probable glycogen synthase kinase 3 alpha |
| 13c00008 | 0.08055 | K12881 | RRM motif-containing protein | um10479 | 0.01219 | ni | conserved hypothetical protein |
| 3c00043 | 0.08078 | ni | hypothetical protein | um01561 | 0.0122 | K06965 | probable Pelota protein |
| 7d00010 | 0.0809 | K00293 | lysine-ketoglutarate reductase | um01736 | 0.01221 | ni | conserved hypothetical protein |
| 16d00008 | 0.08091 | ni | uncharacterized integral membrane protein | um05652 | 0.01227 | K02726 | probable PRE8 - 20S proteasome subunit Y7 |
| 4c00032 | 0.08095 | K00721 | dolichol-phosphate mannosyltransferase | um05631.2 | 0.01235 | ni | conserved hypothetical protein |
| 9c00101 | 0.08114 | ni | hypothetical protein | um01498 | 0.01236 | K00223 | probable ERG4 - sterol C-24 reductase |
| 16c00053 | 0.08129 | ni | hypothetical protein | um11475 | 0.01239 | ni | putative protein |
| 6d00030 | 0.08142 | ni | hypothetical protein | um02581 | 0.01239 | K01535 | probable PMA1 - H -transporting P-type ATPase |
| 9c00381 | 0.08186 | ni | hypothetical protein | um04282 | 0.01246 | ni | related to 3-phytase A precursor |
| 9d00337 | 0.08209 | K03357 | anaphase-promoting complex (APC), subunit 10 | um11288 | 0.01247 | K00418 | probable ubiquinol-cytochrome c reductase complex 11 kDa protein |
| 15c00068 | 0.08227 | K07151 | oligosaccharyltransferase, STT3 subunit | um10657 | 0.0125 | ni | conserved hypothetical protein |
| 9c00266 | 0.08231 | K01835 | phosphoglucomutase | um00319 | 0.01251 | K06072 | conserved hypothetical protein |
| 20d00013 | 0.08273 | ni | hypothetical protein | um11019 | 0.01255 | K12826 | related to PRP11 - pre-mRNA splicing factor |
| 13c00044 | 0.08274 | ni | hypothetical protein | um10649 | 0.01258 | ni | conserved hypothetical protein |
| 13c00103 | 0.0828 | ni | hypothetical protein | um10238 | 0.01261 | K02883 | probable 60S ribosomal protein L18 |
| 5d00067 | 0.08296 | K00620 | putative glutamate/ornithine acetyltransferase | um04030 | 0.01274 | ni | conserved hypothetical protein |
| 7c00131 | 0.08305 | K15322 | tRNA splicing endonuclease SEN2 | um10167 | 0.01278 | K00134 | glyceraldehyde 3-phosphate dehydrogenase |
| 5d00084 | 0.08321 | K13346 | hypothetical protein | um15006 | 0.01281 | ni | conserved hypothetical protein |
| 7d00266 | 0.08322 | ni | hypothetical protein | um05831 | 0.0129 | K04077 | probable heat-shock protein hsp60 |
| 1c00033 | 0.08334 | ni | hypothetical protein | um11585 | 0.01291 | ni | related to capsular associated protein |
| 9c00233 | 0.08338 | ni | DEAH-box RNA helicase | um05942 | 0.01301 | ni | related to monocarboxylate permease |
| 18c00035 | 0.08339 | ni | hypothetical protein | um11634 | 0.01307 | ni | related to KAP114 - Member of the karyopherin-beta family |
| 22c00237 | 0.08352 | ni | methyltransferases | um05412 | 0.01311 | ni | related to quinate 5-dehydrogenase |
| 22d00157 | 0.08355 | ni | hypothetical protein | um11923 | 0.01314 | K17450 | probable mitochondrial aconitate hydratase |
| 9c00429 | 0.08356 | K14848 | ribosome Assembly protein | um03392.2 | 0.01318 | ni | putative protein |
| 19d00068 | 0.08368 | ni | hypothetical protein | um12230.2 | 0.01322 | ni | putative protein |
| 9d00397 | 0.08369 | ni | hypothetical protein | um05028 | 0.01326 | K10742 | related to DNA2 - DNA helicase |
| 2c00059 | 0.08402 | K14808 | ATP-dependent RNA helicase | um02101 | 0.0134 | ni | putative protein |
| 1c00009 | 0.08422 | ni | hypothetical protein | um02304 | 0.01351 | ni | putative protein |
| 6c00013 | 0.08426 | ni | hypothetical protein | um01943 | 0.01362 | ni | related to alpha-glucosidase |
| 9d00292 | 0.08436 | ni | hypothetical protein | um00599 | 0.01363 | K03512 | related to DNA polymerase mu |
| 7c00152 | 0.08437 | K14676 | predicted esterase of the alpha-beta hydrolase superfamily | um00924 | 0.01365 | K03231 | probable translation elongation factor eEF-1 alpha chain |
| 1c00026 | 0.08452 | ni | protein involved in sister chromatid separation and/or segregation | um12068 | 0.01368 | K00737 | related to N-acetylglucosaminyltransferase |
| 7c00019 | 0.08453 | K16576 | actin and related proteins | um10009 | 0.01375 | ni | related to ARO80 - positive transcription regulator of ARO9 and ARO10 |
| 13d00016 | 0.08455 | ni | cullins | um01926 | 0.01375 | K01477 | related to DAL2 - allantoinase |
| 22c00171 | 0.08467 | K06901 | hypothetical protein | um10166 | 0.01377 | K03027 | probable Rpc40 - 40 kD subunit of DNA-directed RNA polymerases I and III |
| 22d00123 | 0.08487 | ni | uncharacterized conserved protein | um00919 | 0.01377 | K05863 | probable ADP |
| 7d00352 | 0.08492 | ni | purple acid phosphatase | um02436 | 0.01378 | K01530 | probable NEO1 - P-type ATPase |
| 9d00185 | 0.085 | ni | hypothetical protein | um04043 | 0.01383 | ni | conserved hypothetical protein |
| 6c00116 | 0.08502 | K08827 | U4/U6-associated splicing factor PRP4 | um01758 | 0.01387 | ni | related to ABC transporter |
| 20d00025 | 0.08504 | ni | hypothetical protein | um11603 | 0.01387 | ni | related to RPS0B - 40S ribosomal protein p40 homolog B |
| 20d00027 | 0.08533 | K03846 | mannosyltransferase | um12350 | 0.01387 | ni | probable ribosomal protein L41 |
| 14d00096 | 0.08538 | K11826 | adaptor complexes medium subunit family | um03552.2 | 0.01395 | ni | hypothetical protein |
| 9c00126 | 0.08558 | ni | vacuolar import and degradation protein | um02316 | 0.01396 | ni | conserved hypothetical protein |
| 10d00040 | 0.08571 | K01867 | cytoplasmic tryptophanyl-tRNA synthetase | um02377 | 0.01409 | K00428 | probable cytochrome c peroxidase precursor |
| 9c00238 | 0.08572 | K00697 | trehalose-6-phosphate synthase component TPS1 and related su | um10678 | 0.01411 | K10398 | related to KIP1 - kinesin-related protein |
| 22c00113 | 0.0858 | ni | hypothetical protein | um12223 | 0.01414 | ni | putative protein |
| 22d00113 | 0.08581 | K11229 | MEKK and related serine/threonine protein kinases | um02383 | 0.01414 | K04627 | a2-pheromone receptor Pra1 |
| 8c00096 | 0.08586 | K13035 | carbon-nitrogen hydrolase | um11891 | 0.01418 | ni | conserved hypothetical protein |
| 16d00020 | 0.08591 | ni | hypothetical protein | um01821 | 0.01422 | ni | probable KRE2 - alpha-1 |
| 9d00093 | 0.08594 | K14768 | WD40-repeat-containing subunit of the 18S rRNA processing co | um10018 | 0.01429 | ni | related to ERD1 protein |
| 7c00001 | 0.086 | ni | metalloprotease | um00247 | 0.01433 | ni | conserved hypothetical protein |
| 22c00054 | 0.086 | ni | hypothetical protein | um05032 | 0.01434 | ni | related to NIT3 - nitrilase |
| 9d00107 | 0.08603 | ni | hypothetical protein | um10378 | 0.01442 | K16261 | related to DIP5 - glutamate and aspartate permease |
| 9c00399 | 0.08607 | ni | kelch repeat-containing proteins | um00965 | 0.01454 | ni | related to carotenoid cleavage dioxygenase 1 |
| 12c00038 | 0.08633 | ni | hypothetical protein | um10117 | 0.01454 | K00698 | chitin Synthase 4 |
| 12d00007 | 0.08644 | K01555 | fumarylacetoacetase | um03808 | 0.01458 | ni | hypothetical protein |
| 5c00017 | 0.0865 | K02915 | 60s ribosomal protein L34 | um01860 | 0.01463 | K04794 | related to PTH2 - aminoacyl-tRNA hydrolase |
| 13c00026 | 0.08674 | ni | hypothetical protein | um11511 | 0.01467 | K12881 | related to YRA1 - RNA annealing protein |
| 10d00065 | 0.08677 | ni | carbon-nitrogen hydrolase | um00584 | 0.01479 | K14758 | probable casein kinase-1 hhp1 |
| 2d00040 | 0.08683 | ni | hypothetical protein | um11270 | 0.01511 | K10256 | related to Delta-12 fatty acid desaturase |
| 22c00170 | 0.08687 | K01792 | uncharacterized enzymes related to aldose 1-epimerase | um04508 | 0.01514 | K14782 | related to BFR2 - involved in protein transport steps at the Brefeldin A blocks |
| 22c00306 | 0.08688 | ni | hypothetical protein | um06004 | 0.01515 | ni | probable HNT1 - Adenosine 5 |
| 7d00240 | 0.08718 | ni | hypothetical protein | um10514 | 0.01517 | ni | conserved hypothetical protein |
| 9d00356 | 0.08744 | ni | mitochondrial import inner membrane translocase, subunit TIM17 | um12155.2 | 0.01519 | K08741 | related to MutS protein homolog 5 |
| 5d00130 | 0.08776 | ni | FOG, RRM domain | um11686 | 0.01519 | ni | ni |
| 7d00073 | 0.08786 | ni | hypothetical protein | um00589 | 0.01522 | K01069 | related to GLO4 - glyoxalase II |
| 16d00054 | 0.08811 | K03850 | alpha-1,2 glucosyltransferase | um02449 | 0.01525 | K09104 | related to centromere binding factor 1 |
| 12d00019 | 0.0882 | K15223 | RNA polymerase I transcription factor UAF | um12006 | 0.01528 | K11415 | related to NAD-dependent deacetylase Sirtuin 5 |
| 22c00215 | 0.08868 | ni | hypothetical protein | um10698 | 0.01531 | ni | related to NEFA-interacting nuclear protein NIP30 |
| 12c00053 | 0.08882 | K00030 | isocitrate dehydrogenase, gamma subunit | um10484 | 0.01533 | K01001 | related to ALG7 - UDP-N-acetylglucosamine-1-phosphate transferase |
| 14d00093 | 0.08892 | ni | hypothetical protein | um02219 | 0.01545 | ni | putative protein |
| 8c00103 | 0.08895 | K10688 | ubiquitin conjugating enzyme | um01632 | 0.01547 | ni | conserved hypothetical protein |
| 27d00073 | 0.08901 | K12188 | RNA polymerase II transcription factor complex subunit | um06464 | 0.01549 | ni | ni |
| 12c00004 | 0.08901 | ni | hypothetical protein | um06157 | 0.01557 | ni | related to Spherulin 4 precursor |
| 11c00023 | 0.08901 | ni | guanine nucleotide exchange factor | um03547.2 | 0.01565 | ni | putative protein |
| 22c00194 | 0.08906 | K03635 | molybdopterin converting factor subunit 2 | um01981 | 0.01574 | ni | related to Malonyl CoA-acyl carrier protein transacylase |
| 27c00087 | 0.08915 | K02966 | 40S ribosomal protein S19 | um01765 | 0.01575 | K09503 | related to DnaJ homolog subfamily A member 2 |
| 7c00172 | 0.08916 | ni | hypothetical protein | um03073 | 0.01581 | ni | related to GTT1 - glutathione S-transferase |
| 5c00042 | 0.0892 | K11996 | molybdopterin synthase sulfurylase | um05345 | 0.01585 | ni | conserved hypothetical protein |
| 9c00169 | 0.08931 | K15429 | tRNA modification enzyme | um15091 | 0.01588 | ni | conserved hypothetical protein |
| 3d00020 | 0.08934 | K00916 | cdc2-related protein kinase | um04499 | 0.01592 | ni | conserved hypothetical protein |
| 19d00064 | 0.08936 | ni | acyltransferase required for palmitoylation | um03458 | 0.01604 | ni | hypothetical protein |
| 2c00046 | 0.08959 | K09523 | dsRNA-activated protein kinase inhibitor P58 | um12235 | 0.01606 | ni | hypothetical protein |
| 8c00033 | 0.0896 | K05302 | uncharacterized conserved protein | um10164 | 0.01606 | K10583 | related to E2 ubiquitin-conjugating enzyme |
| 9d00370 | 0.08964 | K12830 | splicing factor 3b, subunit 3 | um11793 | 0.01616 | K07950 | probable ARL1 - ADP-ribosylation factor |
| 9c00259 | 0.08973 | K08741 | mismatch repair ATPase MSH5 | um04983 | 0.01621 | K01836 | probable PCM1 - phosphoacetylglucosamine mutase |
| 9c00163 | 0.08997 | K03507 | nucleotide excision repair factor NEF2, RAD4/CUT5 component | um02618.2 | 0.01625 | K06063 | probable transcriptional coregulator Snw1 |
| 19c00034 | 0.09002 | ni | hypothetical protein | um11327 | 0.01636 | K17302 | probable SEC27 - coatomer complex beta subunit |
| 8d00031 | 0.09011 | K00877 | phosphomethylpyrimidine kinase | um11241 | 0.0164 | ni | related to aldehyde dehydrogenase |
| 6d00037 | 0.09011 | K01230 | 1, 2-alpha-mannosidase | um10463 | 0.0165 | ni | related to GDP |
| 7d00227 | 0.09012 | ni | 3-oxoacyl CoA thiolase | um10141 | 0.01654 | ni | conserved hypothetical protein |
| 4c00042 | 0.09012 | ni | vigilin | um00920 | 0.01654 | K10756 | probable RFC5 - DNA replication factor C |
| 19d00154 | 0.09012 | ni | hypothetical protein | um06194 | 0.01655 | ni | conserved hypothetical protein |
| 15d00023 | 0.09026 | K13350 | hypothetical protein | um12137 | 0.01656 | ni | conserved hypothetical protein |
| 14d00033 | 0.09028 | K10428 | dynactin subunit p27/WS-3 | um06399 | 0.01658 | ni | conserved hypothetical protein |
| 15c00071 | 0.09055 | ni | C-3 sterol dehydrogenase | um01075 | 0.01671 | K09291 | conserved hypothetical protein |
| 10c00015 | 0.0907 | ni | hypothetical protein | um02332 | 0.01686 | ni | ni |
| 8d00050 | 0.09077 | ni | hypothetical protein | um05733 | 0.01688 | ni | conserved hypothetical protein |
| 9c00065 | 0.0908 | ni | hypothetical protein | um04448 | 0.01689 | ni | related to Myosin regulatory light chain 2-A |
| 7d00106 | 0.09102 | K14560 | U3 small nucleolar ribonucleoprotein | um10847 | 0.01689 | ni | related to nadh-ubiquinone oxidoreductase subunit b17 |
| 7d00261 | 0.09127 | ni | RNA lariat debranching enzyme | um03869 | 0.0169 | ni | conserved hypothetical Ustilago-specific protein |
| 1c00039 | 0.09131 | ni | chaperone HSP104 and related ATP-dependent Clp proteases | um02787 | 0.01692 | ni | hypothetical protein |
| 5c00094 | 0.09144 | ni | hypothetical protein | um01832 | 0.01697 | ni | putative protein |
| 18c00105 | 0.09182 | ni | permease of the major facilitator superfamily | um11512 | 0.01698 | ni | conserved hypothetical protein |
| 22d00222 | 0.09216 | ni | zinc-binding oxidoreductase | um05531 | 0.01699 | K00831 | related to Phosphoserine aminotransferase |
| 2d00024 | 0.09218 | ni | hypothetical protein | um11298 | 0.01705 | ni | related to NHP6B - nonhistone chromosomal protein |
| 16c00002 | 0.09243 | ni | aldo/keto reductase family proteins | um00098 | 0.01708 | ni | related to YTP1 |
| 22d00210 | 0.09256 | K15078 | GIY-YIG type nuclease | um05057 | 0.01723 | ni | putative protein |
| 12c00067 | 0.09265 | ni | hypothetical protein | um00506 | 0.01725 | ni | putative protein |
| 16d00043 | 0.09278 | ni | predicted protein tyrosine phosphatase | um10287 | 0.01733 | K08054 | probable calnexin |
| 22d00205 | 0.09282 | ni | hypothetical protein | um05066 | 0.01737 | ni | conserved hypothetical Ustilago-specific protein |
| 13d00095 | 0.09292 | ni | glyoxylate/hydroxypyruvate reductase | um04598 | 0.01738 | K10415 | related to Dynein intermediate chain 1 |
| 1c00004 | 0.09292 | K10808 | ribonucleotide reductase, beta subunit | um01686.2 | 0.01746 | ni | conserved hypothetical protein |
| 7c00182 | 0.0931 | ni | cytoplasm to vacuole targeting protein | um02595 | 0.01748 | K01106 | related to phosphatidylinositol phosphate phosphatase |
| 14d00016 | 0.09324 | ni | hypothetical protein | um05241 | 0.0175 | K12828 | probable splicing factor 3b subunit 1 |
| 25c00085 | 0.09344 | ni | hypothetical protein | um11612 | 0.01755 | ni | putative protein |
| 9d00032 | 0.09346 | ni | hypothetical protein | um00608 | 0.01756 | ni | conserved hypothetical protein |
| 7c00314 | 0.0935 | K02912 | 60S ribosomal protein L32 | um01130 | 0.01757 | ni | conserved hypothetical protein |
| 11c00062 | 0.09366 | K13288 | oligoribonuclease | um11289 | 0.01759 | ni | conserved hypothetical protein |
| 27d00016 | 0.09377 | ni | hypothetical protein | um03035 | 0.01761 | ni | conserved hypothetical protein |
| 25c00013 | 0.09385 | ni | ypt/rab-specific GTPase-activating protein GYP1 | um03061 | 0.01769 | K03139 | related to TFG2 - TFIIF subunit |
| 25d00034 | 0.09395 | ni | TRAPP 20 K subunit | um11149 | 0.01782 | ni | conserved hypothetical protein |
| 5c00001 | 0.09399 | ni | hypothetical protein | um11505 | 0.01786 | ni | putative protein |
| 22c00266 | 0.09411 | K00459 | hypothetical protein | um01257 | 0.01788 | ni | related to ANT1 - peroxisomal ATP carrier |
| 24d00031 | 0.09413 | ni | hypothetical protein | um01243 | 0.01798 | K06883 | conserved hypothetical protein |
| 7c00307 | 0.09429 | ni | hypothetical protein | um01639 | 0.01799 | K00706 | probable 1 |

| | | | | | | | |
|---|---|---|---|---|---|---|---|
| 12c00132 | 0.09434 | K12897 | hsp27-ere-tata-binding protein/Scaffold attachment factor | um10449.2 | 0.01804 | ni | conserved hypothetical protein |
| 3c00010 | 0.09462 | ni | protein kinase PCTAIRE and related kinases | um10974.2 | 0.01816 | ni | related to tetrahydrofolylpolyglutamate synthase |
| 6d00079 | 0.09465 | ni | predicted membrane protein | um00380 | 0.01818 | K01247 | conserved hypothetical protein |
| 8d00034 | 0.09481 | K10258 | steroid reductase required for elongation of the very long chain fa | um11435 | 0.0183 | ni | conserved hypothetical protein |
| 18c00077 | 0.095 | K15071 | exocyst subunit - Sec10p | um10046 | 0.01836 | ni | conserved hypothetical protein |
| 7c00026 | 0.09503 | ni | hypothetical protein | um05401 | 0.01844 | ni | conserved hypothetical protein |
| 3d00032 | 0.09521 | K12812 | ATP-dependent RNA helicase | um04992.2 | 0.01851 | ni | conserved hypothetical protein |
| 25c00045 | 0.09529 | ni | hypothetical protein | um02574 | 0.01863 | ni | related to c-module-binding factor |
| 7c00072 | 0.09541 | K02021 | peptide exporter | um11824 | 0.01873 | ni | hypothetical protein |
| 2c00069 | 0.09546 | ni | hypothetical protein | um05490 | 0.01873 | ni | hypothetical protein |
| 15d00012 | 0.09561 | K03361 | cdc4 and related F-box and WD-40 proteins | um01327 | 0.01873 | ni | related to Acetylcholinesterase precursor |
| 8d00006 | 0.09598 | ni | hypothetical protein | um00692 | 0.01875 | ni | conserved hypothetical protein |
| 12c00083 | 0.09601 | K14007 | vesicle coat complex COPII, subunit SFB3 | um01111 | 0.01883 | ni | putative protein |
| 9d00021 | 0.09627 | ni | hypothetical protein | um10751 | 0.01885 | K03141 | related to TFB1 - subunit of RNA polymerase II transcription initiation factor TFIIH |
| 16d00046 | 0.09628 | ni | hypothetical protein | um04945 | 0.01887 | ni | putative protein |
| 22d00118 | 0.0963 | K14546 | hypothetical protein | um02267 | 0.01889 | ni | putative protein |
| 6d00014 | 0.09639 | K01797 | hypothetical protein | um12101 | 0.01893 | ni | conserved hypothetical protein |
| 13c00052 | 0.09669 | ni | TFIIF-interacting CTD phosphatase, including NLI-interacting fac | um04312 | 0.01894 | ni | related to alpha-actinin |
| 16d00005 | 0.09675 | ni | hypothetical protein | um02345 | 0.01897 | ni | putative protein |
| 22c00066 | 0.09681 | K03946 | NADH, ubiquinone oxidoreductase NDUFA2/B8 subunit | um00350 | 0.01899 | K09831 | probable ERG5 - C-22 sterol desaturase |
| 9c00275 | 0.09683 | ni | hypothetical protein | um04852 | 0.019 | ni | hypothetical protein |
| 7c00209 | 0.09696 | ni | hypothetical protein | um01672 | 0.01906 | K01899 | probable succinyl-coa synthetase alpha chain |
| 26c00067 | 0.09708 | K14430 | Na+/dicarboxylate | um11304 | 0.0191 | ni | conserved hypothetical protein |
| 5d00062 | 0.09729 | ni | heavy metal exporter HMT1 | um01790 | 0.01912 | ni | related to DNA ligase |
| 25c00006 | 0.09777 | ni | hypothetical protein | um02010 | 0.01919 | K01626 | related to family II 2-keto-3-deoxy-D-arabino-heptulosonate 7-phosphate synthase |
| 24c00043 | 0.09785 | K10849 | structure-specific endonuclease ERCC1-XPF, ERCC1 compone | um01211 | 0.01923 | ni | conserved hypothetical protein |
| 13c00035 | 0.09792 | ni | hypothetical protein | um03775 | 0.01932 | K00326 | related to MCR1 - cytochrome-b5 reductase |
| 15c00015 | 0.09828 | K11839 | ubiquitin C-terminal hydrolase | um01264 | 0.01934 | ni | conserved hypothetical protein |
| 19c00017 | 0.09839 | ni | predicted membrane protein | um10672.2 | 0.01941 | ni | hypothetical protein |
| 22d00076 | 0.09861 | K00587 | farnesyl cysteine-carboxyl methyltransferase | um10151 | 0.01963 | ni | conserved hypothetical Ustilago-specific protein |
| 11c00004 | 0.09864 | K15111 | mitochondrial carrier protein PET8 | um11392 | 0.01983 | ni | conserved hypothetical protein |
| 12c00089 | 0.0988 | ni | hypothetical protein | um02258 | 0.01989 | K02021 | probable ATP-binding cassette |
| 10d00092 | 0.09903 | ni | dual specificity phosphatase | um02071 | 0.01997 | ni | conserved hypothetical protein |
| 13d00111 | 0.09905 | K11594 | ATP-dependent RNA helicase | um03377 | 0.01997 | ni | hypothetical protein |
| 9c00282 | 0.0991 | K15128 | RNA polymerase II transcriptional regulation mediator | um00729 | 0.01999 | K10755 | probable RFC4 - DNA replication factor C |
| 6d00119 | 0.09942 | ni | GYF domain containing proteins | um02686.2 | 0.02003 | ni | related to ATM1 - Mitochondrial inner membrane ABC transporter involved in the maturation of cytosolic iron-sulfur |
| 10d00081 | 0.09952 | ni | low density lipoprotein receptor | um02899 | 0.02004 | K01681 | probable aconitase |
| 25d00061 | 0.09982 | ni | hypothetical protein | um11097 | 0.02004 | K00420 | related to QCR10 - ubiquinol--cytochrome-c reductase 8 |
| 22c00176 | 0.10016 | K09503 | molecular chaperone | um02463 | 0.02015 | K09602 | conserved hypothetical protein |
| 14d00103 | 0.10025 | ni | molecular chaperone | um12265.2 | 0.02015 | ni | conserved hypothetical protein |
| 7d00199 | 0.10049 | K08332 | armadillo repeat protein VAC8 | um02578 | 0.02017 | ni | related to Het-c heterokaryon incompatibility protein |
| 9d00176 | 0.10058 | K03250 | translation initiation factor 3, subunit e | um00223 | 0.02018 | K09885 | related to aquaporin |
| 9c00134 | 0.10061 | K01533 | cation transport ATPase | um00896 | 0.02032 | K11498 | Kinesin-7a motor protein |
| 4c00020 | 0.10078 | ni | phosphoprotein | um05480 | 0.02033 | K00698 | chitin Synthase 7 |
| 7d00229 | 0.10092 | K03347 | cullins | um02380 | 0.02035 | K15325 | hypothetical protein |
| 22c00065 | 0.10121 | K06965 | meiotic cell division protein Pelota/DOM34 | um05776 | 0.02043 | K04487 | probable Cysteine desulfurase |
| 7d00076 | 0.10128 | ni | hypothetical protein | um00366 | 0.02047 | K02542 | probable MCM6 - involved in replication |
| 9c00212 | 0.1013 | K01115 | phospholipase D1 | um11906 | 0.02068 | ni | probable MRS11 - subunit of the Tim22-complex |
| 22d00279 | 0.10131 | ni | dimeric dihydrodiol dehydrogenase | um10892 | 0.0207 | K03424 | conserved hypothetical protein |
| 5c00117 | 0.10156 | ni | synaptic vesicle transporter SVOP and related transporters | um05892 | 0.02082 | K11121 | related to NAD-dependent histone deacetylase |
| 19d00111 | 0.10156 | ni | vacuolar assembly/sorting proteins VPS39/VAM6/VPS3 | um00499 | 0.02083 | ni | conserved hypothetical protein |
| 22c00200 | 0.10158 | ni | hypothetical protein | um11181 | 0.02086 | ni | probable LEU2 - beta-isopropyl-malate dehydrogenase |
| 14d00025 | 0.10199 | ni | hypothetical protein | um06020 | 0.02087 | ni | conserved hypothetical protein |
| 22c00162 | 0.10201 | K07880 | GTPase Rab4 | um02862 | 0.02088 | ni | conserved hypothetical protein |
| 7c00077 | 0.10225 | ni | hypothetical protein | um12222 | 0.02091 | ni | putative protein |
| 19d00013 | 0.10241 | ni | hypothetical protein | um03308 | 0.02092 | K11142 | probable Cytosol aminopeptidase |
| 26d00055 | 0.1025 | K11096 | small nuclear ribonucleoprotein | um02760 | 0.02092 | K02325 | related to DNA polymerase epsilon subunit B |
| 22c00023 | 0.10251 | K16569 | gamma-tubulin complex, DGRIP84/SPC97 component | um03113 | 0.02093 | ni | conserved hypothetical protein |
| 7d00023 | 0.10263 | ni | hypothetical protein | um05042.2 | 0.02094 | ni | conserved hypothetical protein |
| 7d00253 | 0.10263 | ni | hypothetical protein | um00206.2 | 0.02096 | ni | conserved hypothetical protein |
| 24c00022 | 0.10272 | K01881 | prolyl-trna synthetase | um05785 | 0.02103 | ni | acyl transferase-like protein |
| 9d00222 | 0.10302 | K02304 | hypothetical protein | um05366 | 0.02106 | ni | conserved hypothetical protein |
| 6c00021 | 0.10311 | ni | hypothetical protein | um00512 | 0.02109 | K03937 | probable NADH-ubiquinone oxidoreductase 21 kDa subunit |
| 15c00062 | 0.10327 | ni | predicted transporter | um05628.2 | 0.0211 | ni | conserved hypothetical protein |
| 3d00098 | 0.10381 | ni | ras1 guanine nucleotide exchange factor | um03396 | 0.02117 | ni | conserved hypothetical protein |
| 8d00007 | 0.10388 | ni | FOG, Zn-finger | um11009 | 0.02121 | K08737 | related to MSH6 - DNA mismatch repair protein |
| 26c00005 | 0.10409 | ni | hypothetical protein | um01521 | 0.02123 | K01465 | dihydroorotase |
| 9d00285 | 0.10429 | ni | hypothetical protein | um04169 | 0.02128 | ni | related to leucine zipper-EF-hand containing transmembrane protein 1 |
| 10d00061 | 0.1044 | K14298 | mRNA export protein | um00381 | 0.02132 | K03953 | probable NADH2 dehydrogenase |
| 6c00083 | 0.10443 | K01580 | glutamate decarboxylase and related proteins | um06268 | 0.02134 | K16302 | related to MAM3 - Protein required for normal mitochondrial morphology |
| 9c00311 | 0.10445 | ni | actin-binding protein SLA2 | um11053 | 0.02141 | ni | conserved hypothetical protein |
| 9c00033 | 0.10446 | ni | hypothetical protein | um00610 | 0.02146 | ni | conserved hypothetical protein |
| 7c00117 | 0.10446 | K14684 | mitochondrial solute carrier protein | um01011 | 0.02147 | ni | related to lariat-debranching enzyme |
| 9c00177 | 0.10447 | ni | hypothetical protein | um11907.2 | 0.02156 | ni | conserved hypothetical protein |
| 15d00044 | 0.10514 | ni | molecular chaperones HSP70/HSC70 | um04622 | 0.02163 | K01490 | probable AMD1 - AMP deaminase |
| 9c00112 | 0.10532 | ni | hypothetical protein | um04587 | 0.02173 | K13116 | probable DEAD-box ATP-dependent RNA helicase 35 |
| 15c00075 | 0.10565 | K14840 | cellular protein | um10654 | 0.02179 | K10845 | related to TFB5 - component of general transcription and DNA repair factor TFIIH |
| 9d00296 | 0.10579 | ni | hypothetical protein | um03793 | 0.02185 | K11362 | putative protein |
| 7d00017 | 0.10635 | ni | hypothetical protein | um02628 | 0.02186 | K02999 | related to DNA-directed RNA polymerase I |
| 5c00029 | 0.10636 | ni | cystathionine beta-synthase and related enzymes | um06224 | 0.02188 | K03243 | probable GTPase |
| 14d00104 | 0.10639 | ni | CDK9 kinase-activating protein cyclin T | um12309 | 0.0219 | ni | conserved hypothetical protein |
| 26d00003 | 0.10643 | K11415 | sirtuin 5 and related class III sirtuins | um04378 | 0.0219 | ni | related to phenylacetaldehyde dehydrogenase |
| 7d00173 | 0.10672 | K17218 | sulfide, quinone oxidoreductase | um02401 | 0.02191 | K14396 | related to poly |
| 5d00147 | 0.10689 | K00850 | pyrophosphate-dependent phosphofructo-1-kinase | um11631 | 0.02199 | ni | hypothetical protein |
| 7c00260 | 0.10707 | ni | hypothetical protein | um04693 | 0.02207 | ni | conserved hypothetical Ustilago-specific protein |
| 16d00056 | 0.10725 | ni | hypothetical protein | um06478 | 0.02209 | ni | conserved hypothetical protein |
| 7d00028 | 0.10742 | ni | amine oxidase | um01189 | 0.02216 | K03232 | probable EFB1 - translation elongation factor eEF1beta |
| 16d00071 | 0.10743 | K00963 | UDP-glucose pyrophosphorylase | um10319 | 0.02218 | ni | putative protein |
| 27d00099 | 0.10779 | ni | hypothetical protein | um06022 | 0.02219 | K14654 | related to RIB7 - HTP reductase |
| 7d00280 | 0.10782 | ni | nuclear receptor coregulator SMRT/SMRTER | um03085 | 0.02225 | K04564 | probable manganese superoxide dismutase precursor |
| 9c00287 | 0.10786 | K03521 | electron transfer flavoprotein, beta subunit | um02495 | 0.02226 | K03039 | related to 26S proteasome regulatory particle chain RPN9 |
| 6c00050 | 0.10816 | ni | hypothetical protein | um05911 | 0.0223 | ni | conserved hypothetical protein |
| 16c00075 | 0.10838 | ni | hypothetical protein | um01889 | 0.0223 | ni | putative protein |
| 15c00010 | 0.10839 | K12346 | mn2+ and Fe2+ transporters of the NRAMP family | um02223 | 0.02233 | K01078 | related to acid phosphatase |
| 12c00054 | 0.10851 | K12870 | mRNA splicing factor | um06091 | 0.02234 | ni | ni |
| 15c00037 | 0.1086 | ni | hypothetical protein | um11248 | 0.02241 | ni | conserved hypothetical protein |
| 18c00024 | 0.10861 | K14549 | conserved WD40 repeat-containing protein | um05545 | 0.02248 | K11427 | related to protein-lysine N-methyltransferase |
| 18d00056 | 0.1087 | ni | hypothetical protein | um06298 | 0.02264 | ni | related to CCC1 - Proposed vacuolar iron transport protein |
| 10c00051 | 0.10871 | ni | betaine aldehyde dehydrogenase | um04781 | 0.02265 | K11096 | probable small nuclear ribonucleoprotein chain D2 |
| 9d00159 | 0.10875 | K10734 | uncharacterized conserved protein | um11541 | 0.02277 | K17338 | conserved hypothetical protein |
| 14c00095 | 0.10878 | K14007 | vesicle coat complex COPII, subunit SEC24/subunit SFB2 | um10332 | 0.02279 | ni | conserved hypothetical protein |
| 24c00061 | 0.10881 | ni | hypothetical protein | um10061 | 0.02285 | ni | conserved hypothetical protein |
| 13c00063 | 0.10901 | K01568 | thiamine pyrophosphate-requiring enzyme | um02472 | 0.02304 | ni | conserved hypothetical protein |
| 8d00069 | 0.10922 | K12393 | adaptor complexes medium subunit family | um00208 | 0.02307 | ni | related to SRC1 - involved in sister chromatid segregation |
| 20c00011 | 0.10922 | K01279 | hypothetical protein | um12034 | 0.02307 | K12158 | probable NEDD8 precursor - Ubiquitin-like protein |
| 13d00056 | 0.1097 | ni | HMG box-containing protein | um02331 | 0.02307 | K04371 | MAP kinase |
| 1d00035 | 0.10985 | K03264 | translation initiation factor 6 | um03184 | 0.02315 | ni | ni |
| 26d00020 | 0.10992 | K03564 | alkyl hydroperoxide reductase | um10778 | 0.02318 | K15156 | related to RNA polymerase II mediator complex protein pmc1 |
| 5c00087 | 0.10998 | K09506 | molecular chaperone | um00812 | 0.0232 | K11788 | probable bifunctional purine biosynthetic protein ade1 |
| 26d00070 | 0.11005 | K12816 | mRNA splicing factor | um11637 | 0.02321 | ni | conserved hypothetical protein |
| 26c00014 | 0.11008 | K12191 | vacuolar assembly/sorting protein DID4 | um01329 | 0.02322 | K00030 | probable IDH1 - isocitrate dehydrogenase |
| 7c00350 | 0.11009 | ni | hypothetical protein | um10501 | 0.02325 | ni | conserved hypothetical Ustilago-specific protein |
| 22c00184 | 0.11016 | ni | hypothetical protein | um01967 | 0.02325 | ni | conserved hypothetical protein |
| 9d00249 | 0.11019 | ni | hypothetical protein | um01903 | 0.02339 | ni | related to succinyl-coa ligase |
| 14d00034 | 0.11022 | ni | uncharacterized conserved protein | um10922 | 0.02341 | ni | conserved hypothetical protein |
| 20d00062 | 0.11023 | ni | rhogef GTPase | um04323 | 0.02357 | ni | conserved hypothetical protein |
| 5c00149 | 0.11026 | ni | hypothetical protein | um03412 | 0.02359 | ni | conserved hypothetical protein |
| 3d00031 | 0.11037 | K12812 | ATP-dependent RNA helicase | um04997 | 0.02364 | ni | related to SUV3 - ATP-dependent RNA helicase |
| 4d00035 | 0.11046 | K03025 | RNA polymerase III, subunit C34 | um03266 | 0.02367 | ni | conserved hypothetical protein |
| 19d00143 | 0.1105 | K14290 | nuclear transport receptor CRM1/MSN5 | um06009 | 0.02371 | K05658 | probable Leptomycin B resistance protein pmd1 |
| 9d00228 | 0.11079 | K11251 | histone 2A | um02573 | 0.02372 | ni | hypothetical protein |
| 27c00001 | 0.11109 | ni | SAM-dependent methyltransferases | um05393 | 0.02372 | ni | ni |
| 25c00042 | 0.11127 | K01173 | mitochondrial endonuclease | um02291 | 0.02378 | K00681 | related to gamma-glutamyltransferase |
| 9c00288 | 0.11131 | ni | Ca2+ sensor | um04466 | 0.02386 | ni | related to NADPH-dependent beta- ketoacyl reductase |
| 12c00096 | 0.11157 | ni | hypothetical protein | um11597 | 0.02401 | ni | hypothetical protein |
| 22d00139 | 0.11184 | ni | phosphoglucomutase | um02884 | 0.02405 | ni | conserved hypothetical protein |
| 1c00043 | 0.11185 | ni | protein kinase C substrate, 80 KD protein, heavy chain | um01979 | 0.02419 | ni | related to MTQ2 - Putative S-adenosylmethionine-dependent methyltransferase |
| 10d00026 | 0.11245 | ni | hypothetical protein | um04210 | 0.02444 | K15745 | probable phytoene dehydrogenase |
| 16c00001 | 0.11246 | ni | non-ribosomal peptide synthetase | um10477 | 0.02458 | K12842 | conserved hypothetical protein |
| 25d00032 | 0.11251 | ni | hypothetical protein | um12185 | 0.0246 | ni | conserved hypothetical protein |
| 15d00074 | 0.11255 | ni | dipeptidyl aminopeptidase | um02557 | 0.02461 | K02321 | related to DNA polymerase alpha 70 kDa subunit |
| 7d00171 | 0.11278 | K01619 | deoxyribose-phosphate aldolase | um11413 | 0.02469 | ni | putative protein |
| 14c00084 | 0.11303 | ni | P-type ATPase | um02366 | 0.02471 | K03109 | related to signal recognition particle 9 protein |
| 7d00144 | 0.11311 | K06943 | GTP-binding protein CRFG/NOG1 | um11323 | 0.02476 | ni | related to 2-hydroxyacid dehydrogenase |
| 19d00144 | 0.11314 | ni | WD40 repeat-containing protein | um11139 | 0.02482 | ni | putative protein |
| 13d00052 | 0.11329 | K00851 | gluconate kinase | um12333 | 0.02484 | ni | putative protein |
| 7d00325 | 0.11335 | ni | hypothetical protein | um10561 | 0.02494 | ni | related to VPS10 domain-containing receptor SorCS1 precursor |
| 9c00393 | 0.11344 | ni | dolichyl pyrophosphate phosphatase and related acid phosphatas | um05089.2 | 0.02506 | ni | conserved hypothetical protein |
| 7d00163 | 0.11429 | K00311 | electron transfer flavoprotein ubiquinone oxidoreductase | um11274 | 0.02513 | ni | putative protein |
| 7d00284 | 0.11437 | ni | hypothetical protein | um06182 | 0.02521 | K15414 | mitochondrial p32 family protein |
| 10c00049 | 0.11462 | K03256 | tRNA(1-methyladenosine) methyltransferase, subunit GCD10 | um01198 | 0.02525 | ni | related to Deoxyribose-phosphate aldolase |
| 8d00086 | 0.11466 | K15728 | hypothetical Protein | um10329 | 0.02532 | K01487 | related to guanine deaminase |
| 3d00040 | 0.11475 | ni | hypothetical protein | um01441 | 0.0254 | ni | conserved hypothetical protein |
| 22c00103 | 0.11482 | ni | predicted DHHC-type Zn-finger protein | um03792 | 0.02542 | K11274 | related to sepB protein |
| 14d00010 | 0.11483 | ni | hypothetical protein | um03682.2 | 0.02544 | ni | conserved hypothetical protein |
| 7c00016 | 0.1149 | K07375 | beta tubulin | um11881 | 0.02546 | ni | conserved hypothetical protein |
| 19d00133 | 0.11538 | ni | hypothetical protein | um03108 | 0.02547 | ni | related to Aminoadipate-semialdehyde dehydrogenase |
| 20d00074 | 0.11538 | ni | equilibrative nucleoside transporter protein | um00605 | 0.02549 | K03849 | related to glucosyltransferase |
| 15d00072 | 0.11554 | K00898 | dehydrogenase kinase | um03416 | 0.02551 | ni | carbon source-regulated protein |
| 3d00075 | 0.11579 | ni | 3-phosphoinositide-dependent protein kinase | um05795 | 0.02551 | ni | conserved hypothetical protein |
| 3d00065 | 0.11591 | ni | hypothetical protein | um11290 | 0.02552 | ni | related to SNX4 - Sorting NeXin |
| 16d00035 | 0.11591 | K14849 | hypothetical protein | um15034 | 0.02552 | K09490 | probable chaperone bip |
| 19d00130 | 0.11627 | ni | hypothetical protein | um04964 | 0.02559 | ni | conserved hypothetical protein |
| 24d00005 | 0.11635 | ni | hypothetical protein | um11343.2 | 0.02561 | ni | conserved hypothetical protein |
| 7c00338 | 0.11642 | K01875 | seryl-trna synthetase | um00644.2 | 0.02564 | ni | putative protein |
| 7c00259 | 0.11664 | ni | thioredoxin/protein disulfide isomerase | um04942 | 0.02567 | ni | hypothetical protein |

| | | | | | | | |
|---|---|---|---|---|---|---|---|
| 11d00068 | 0.11682 | ni | protein tyrosine phosphatase | um01128 | 0.02578 | K11341 | related to YAF9 - Component of a chromatin modifying complex |
| 15c00039 | 0.11683 | ni | predicted Zn-dependent hydrolase | um00796 | 0.02587 | ni | putative protein |
| 27c00037 | 0.11707 | ni | hypothetical protein | um01237 | 0.02588 | ni | conserved hypothetical Ustilago-specific protein |
| 14c00054 | 0.1176 | ni | hypothetical protein | um01702 | 0.02589 | ni | conserved hypothetical protein |
| 7d00286 | 0.11766 | ni | uncharacterized Zn ribbon-containing protein | um10544 | 0.02591 | ni | conserved hypothetical protein |
| 22d00255 | 0.11788 | ni | predicted hydrolases or acyltransferases | um04442 | 0.02609 | K01893 | probable DED81 - asparaginyl-tRNA-synthetase |
| 9c00025 | 0.11806 | K09780 | hypothetical protein | um02104.2 | 0.02613 | ni | probable Regulator of G-Protein Signaling Protein |
| 22c00224 | 0.11812 | ni | predicted component of NuA3 histone acetyltransferase complex | um01864 | 0.02614 | K15340 | conserved hypothetical protein |
| 7d00222 | 0.11823 | K12820 | mRNA splicing factor ATP-dependent RNA helicase | um11522 | 0.02616 | ni | putative protein |
| 7c00204 | 0.11825 | ni | 60S ribosomal protein L14/L17/L23 | um10311 | 0.02617 | ni | putative protein |
| 16d00062 | 0.11826 | K01120 | hypothetical protein | um12328 | 0.02637 | ni | conserved hypothetical Ustilago-specific protein |
| 15c00072 | 0.1183 | K04460 | serine-threonine phosphatase 2A, catalytic subunit | um10001 | 0.02653 | ni | hypothetical protein |
| 12d00072 | 0.11836 | K11855 | ubiquitin carboxyl-terminal hydrolase | um01168 | 0.02663 | ni | conserved hypothetical protein |
| 7c00267 | 0.11837 | ni | hypothetical protein | um10749 | 0.02668 | K00728 | probable dolichyl-phosphate-mannose--protein mannosyltransferase |
| 18d00018 | 0.11877 | K01876 | aspartyl-trna synthetase | um06213 | 0.02676 | ni | conserved hypothetical protein |
| 7c00291 | 0.11955 | K00416 | hypothetical protein | um01547 | 0.02677 | ni | conserved hypothetical protein |
| 22c00193 | 0.11986 | ni | hypothetical protein | um04677 | 0.02684 | ni | putative protein |
| 22c00130 | 0.12012 | ni | hypothetical protein | um11641 | 0.02685 | ni | conserved hypothetical protein |
| 7d00003 | 0.12017 | ni | hypothetical protein | um03974 | 0.02687 | K14152 | probable histidine biosynthesis trifunctional protein |
| 4d00022 | 0.12019 | ni | hypothetical protein | um10364 | 0.02691 | ni | related to KRE6 - glucan synthase subunit |
| 26c00071 | 0.12026 | K14852 | regulator of ribosome synthesis | um11914 | 0.02694 | K02983 | probable 40S ribosomal protein S30 |
| 6c00087 | 0.12039 | ni | hypothetical protein | um00630 | 0.02699 | K02154 | probable Vacuolar |
| 3d00052 | 0.12051 | ni | hypothetical protein | um00673 | 0.027 | ni | probable het-c2 protein |
| 3c00073 | 0.12066 | ni | hypothetical protein | um00053 | 0.02713 | K03786 | probable 3-dehydroquinate dehydratase |
| 5d00087 | 0.1209 | ni | protein interacting with poly(A)-binding protein | um01773 | 0.0272 | ni | conserved hypothetical protein |
| 1c00018 | 0.12098 | ni | mitogen-activated protein kinase | um03013 | 0.02729 | K03255 | related to CLU1 - translation initiation factor eIF3 |
| 7d00105 | 0.12141 | ni | hypothetical protein | um06258 | 0.02733 | ni | conserved hypothetical protein |
| 7c00302 | 0.12144 | K07976 | GTPase Rab5/YPT51 | um02802 | 0.02733 | ni | related to NAR1 - similarity to human nuclear prelamin A recognition factor |
| 4c00001 | 0.12179 | ni | hypothetical protein | um04162 | 0.02737 | ni | related to mitochondrial amino acid transporter ARG-13 |
| 20d00022 | 0.1219 | ni | hypothetical protein | um00226 | 0.02739 | K11364 | conserved hypothetical protein |
| 25c00075 | 0.12229 | ni | hypothetical protein | um03661 | 0.0274 | K03800 | related to lipoyltransferase |
| 14d00004 | 0.12236 | K00616 | transaldolase | um11376 | 0.0275 | ni | putative protein |
| 13c00062 | 0.12247 | K01568 | thiamine pyrophosphate-requiring enzyme | um10635 | 0.02759 | ni | hypothetical protein |
| 8c00009 | 0.12255 | ni | hypothetical protein | um03180 | 0.02763 | ni | related to white collar 1 protein |
| 12c00102 | 0.12296 | K03348 | anaphase-promoting complex (APC), subunit 1 | um11520 | 0.02763 | K02150 | probable Vacuolar ATP synthase subunit E |
| 11c00074 | 0.12309 | K01893 | asparaginyl-trna synthetase | um12318 | 0.02764 | ni | hypothetical protein |
| 26d00081 | 0.12314 | ni | hypothetical protein | um02290 | 0.02769 | ni | conserved hypothetical protein |
| 27d00007 | 0.12314 | ni | glycolate oxidase | um02654 | 0.02774 | ni | conserved hypothetical protein |
| 20d00021 | 0.12336 | K10686 | NEDD8-activating complex, catalytic component UBA3 | um02920 | 0.02783 | K15206 | related to TFIIIC subunit |
| 7d00198 | 0.12347 | ni | hypothetical protein | um01959 | 0.02785 | ni | conserved hypothetical protein |
| 9c00355 | 0.12361 | ni | hypothetical protein | um02199 | 0.02792 | ni | hypothetical protein |
| 22d00290 | 0.12379 | ni | hypothetical protein | um03292 | 0.02803 | K11979 | related to Protein mlo2 |
| 27d00068 | 0.12383 | ni | hypothetical protein | um03144 | 0.02807 | K11826 | probable clathrin-associated adaptor complex medium chain |
| 19c00042 | 0.12386 | K03023 | RNA polymerase III (C) subunit | um12160 | 0.02816 | ni | related to YKT6 - SNARE protein for Endoplasmic Reticulum-Golgi transport |
| 8d00045 | 0.12391 | ni | hypothetical protein | um11688 | 0.02821 | ni | conserved hypothetical protein |
| 22d00257 | 0.12398 | K01273 | renal dipeptidase | um10091 | 0.02828 | ni | conserved hypothetical protein |
| 11c00006 | 0.12409 | ni | predicted K+/H+-antiporter | um10467 | 0.02831 | ni | related to RAD16 - nucleotide excision repair protein |
| 5d00036 | 0.12436 | ni | uncharacterized high-glucose-regulated protein | um11107 | 0.02845 | ni | conserved hypothetical protein |
| 9d00063 | 0.12448 | K00826 | branched chain aminotransferase BCAT1 | um01540 | 0.02865 | ni | conserved hypothetical protein |
| 25c00077 | 0.12471 | ni | ras1 guanine nucleotide exchange factor | um04004 | 0.02886 | K11204 | probable gamma-glutamylcysteine synthetase |
| 2d00038 | 0.12479 | K13127 | predicted E3 ubiquitin ligase | um04398 | 0.02889 | ni | related to RNA binding protein Nrd1 |
| 26c00087 | 0.12481 | K13107 | predicted RNA-binding protein | um05349 | 0.02909 | ni | conserved hypothetical protein |
| 7c00277 | 0.12491 | ni | calmodulin and related proteins | um02933 | 0.02913 | ni | putative protein |
| 13d00074 | 0.12507 | K11676 | hypothetical protein | um00572 | 0.02916 | ni | conserved hypothetical protein |
| 7c00192 | 0.12512 | K02266 | cytochrome c oxidase, subunit VIb/COX13 | um00768 | 0.02919 | K17267 | probable coatomer gamma-2 subunit |
| 3c00038 | 0.12517 | ni | nifu-like domain-containing proteins | um02247 | 0.02939 | ni | conserved hypothetical protein |
| 26d00101 | 0.1253 | K09788 | hypothetical protein | um04798 | 0.02939 | ni | hypothetical protein |
| 8c00021 | 0.12543 | K12471 | equilibrative nucleoside transporter protein | um00191 | 0.02945 | K13106 | conserved hypothetical protein |
| 19c00084 | 0.12558 | K11564 | hypothetical protein | um04969 | 0.0295 | ni | conserved hypothetical protein |
| 2c00012 | 0.1256 | K15163 | hypothetical protein | um03631 | 0.02954 | ni | related to MIA40 - mitochondrial intermembrane space protein |
| 10c00050 | 0.12563 | K03504 | hypothetical protein | um11178 | 0.02955 | ni | related to U2 small nuclear ribonucleoprotein B |
| 13c00057 | 0.12587 | ni | hypothetical protein | um02442 | 0.02971 | K03233 | related to translation elongation factor eEF1 |
| 22d00090 | 0.12597 | K14775 | uncharacterized conserved protein | um02859 | 0.0298 | K14290 | probable CRM1 - nuclear export factor |
| 11d00077 | 0.12601 | ni | hypothetical protein | um11974 | 0.02981 | ni | ni |
| 8d00015 | 0.12621 | K16066 | predicted dehydrogenase | um03537 | 0.03002 | K00611 | probable ornithine carbamoyltransferase precursor |
| 9d00318 | 0.12631 | ni | ubiquitin-protein ligase | um10961 | 0.03003 | K02437 | probable Glycine cleavage system H protein |
| 14c00027 | 0.12645 | K10845 | hypothetical protein | um02148 | 0.03017 | ni | related to positive effector protein GCN20 |
| 27d00096 | 0.12652 | ni | hypothetical protein | um03756 | 0.03025 | K10300 | related to DNA helicase Fdhp |
| 9d00204 | 0.12655 | ni | hypothetical protein | um06259 | 0.03029 | K05864 | probable U-snRNP-associated cyclophilin |
| 5c00027 | 0.12679 | ni | hypothetical protein | um04703 | 0.03035 | K01840 | probable phosphomannomutase |
| 9c00110 | 0.12705 | ni | hypothetical protein | um11300 | 0.03047 | ni | conserved hypothetical protein |
| 12d00030 | 0.1272 | ni | hypothetical protein | um00780 | 0.03047 | ni | putative protein |
| 5c00055 | 0.12747 | K10643 | MOT2 transcription factor | um02435 | 0.03048 | K01410 | related to mitochondrial intermediate peptidase precursor |
| 27c00070 | 0.12758 | ni | hypothetical protein | um11355 | 0.03051 | K11569 | related to DAD4 - outer kinetochore protein |
| 2c00049 | 0.12758 | K01597 | mevalonate pyrophosphate decarboxylase | um11041.2 | 0.03053 | ni | related to serine |
| 18d00030 | 0.12775 | ni | ankyrin repeat and DHHC-type Zn-finger domain containing prote | um01881 | 0.03054 | K00540 | related to Oxidoreductase |
| 12c00033 | 0.12786 | ni | hypothetical protein | um02325.2 | 0.03067 | ni | conserved hypothetical protein |
| 7d00279 | 0.12799 | K06689 | ubiquitin-protein ligase | um02596 | 0.03074 | ni | hypothetical protein |
| 18c00070 | 0.128 | ni | hypothetical protein | um02501 | 0.0308 | K12572 | related to Pab1 dependent poly |
| 22d00047 | 0.12852 | ni | dihydrolipoamide succinyltransferase | um05606 | 0.0308 | K00452 | probable BNA1 - 3-hydroxyanthranilic acid dioxygenase |
| 3d00068 | 0.1286 | ni | hypothetical protein | um03760 | 0.03083 | ni | related to Phosphatidylinositol-3 |
| 27d00010 | 0.12876 | K14306 | nuclear porin | um12282 | 0.03084 | K15028 | related to eIF3k -translation initiation factor 3 subunit K |
| 14d00037 | 0.12895 | ni | hypothetical protein | um05694 | 0.03091 | ni | hypothetical protein |
| 3d00059 | 0.1293 | K08286 | serine/threonine protein kinase | um12339 | 0.03096 | ni | hypothetical protein |
| 9c00319 | 0.12954 | ni | hypothetical protein | um01478 | 0.03097 | K00415 | probable QCR2 - 40 kDa ubiquinol cytochrome-c reductase core protein 2 |
| 7d00040 | 0.12958 | K02946 | hypothetical protein | um02300 | 0.03102 | K01176 | probable alpha-amylase |
| 22c00225 | 0.12964 | ni | hypothetical protein | um04623 | 0.03102 | K10756 | probable RFC3 - DNA replication factor C |
| 25d00046 | 0.12972 | K13337 | 40 kDa farnesylated protein associated with peroxisomes | um06038 | 0.03106 | ni | conserved hypothetical protein |
| 13d00048 | 0.12979 | ni | hypothetical protein | um04015 | 0.03108 | K12343 | steroid 5alpha-reductase |
| 11c00059 | 0.12995 | ni | ER-Golgi vesicle-tethering protein p115 | um04944 | 0.03111 | K14401 | related to cleavage and polyadenylation specificity factor |
| 6d00051 | 0.13022 | ni | hypothetical protein | um11564 | 0.03113 | ni | hypothetical protein |
| 3d00023 | 0.13036 | K00036 | glucose-6-phosphate 1-dehydrogenase | um11358 | 0.0312 | K02355 | related to MEF2 - translation elongation factor |
| 1c00036 | 0.13046 | ni | aldehyde dehydrogenase | um01112 | 0.0315 | K03417 | related to carboxyphosphonoenolpyruvate phosphonomutase |
| 21c00006 | 0.13063 | ni | hypothetical protein | um05423 | 0.03151 | ni | related to Monoamine oxidase A |
| 18c00074 | 0.13066 | ni | hypothetical protein | um05746 | 0.03154 | K00685 | related to arginine-tRNA-protein transferase |
| 19c00059 | 0.13111 | ni | clathrin adaptor complex, small subunit | um05777 | 0.03155 | ni | conserved hypothetical protein |
| 13c00034 | 0.13141 | ni | hypothetical protein | um04650 | 0.03164 | ni | conserved hypothetical protein |
| 22d00069 | 0.13205 | ni | dipeptidyl aminopeptidase | um01696 | 0.0317 | ni | conserved hypothetical protein |
| 6c00136 | 0.13214 | K00565 | mRNA cap methyltransferase | um11773 | 0.03172 | ni | conserved hypothetical protein |
| 14c00003 | 0.13216 | ni | predicted transporter | um11696 | 0.03176 | ni | related to SCS2 - required for inositol metabolism |
| 11c00081 | 0.13219 | K04345 | cAMP-dependent protein kinase catalytic subunit | um04290 | 0.03181 | K00698 | chitin synthase 2 |
| 22c00049 | 0.13234 | ni | permease of the major facilitator superfamily | um03253 | 0.03193 | ni | putative protein |
| 9d00016 | 0.13243 | ni | hypothetical protein | um00989 | 0.03205 | K10862 | conserved hypothetical protein |
| 8c00052 | 0.13245 | ni | hypothetical protein | um12051 | 0.03206 | ni | related to Glutamate decarboxylase 1 |
| 14c00045 | 0.13249 | K01530 | P-type ATPase | um10875 | 0.03206 | ni | conserved hypothetical protein |
| 20c00045 | 0.13254 | ni | hypothetical protein | um06119 | 0.03207 | ni | conserved hypothetical protein |
| 3c00042 | 0.1327 | K01101 | p-Nitrophenyl phosphatase | um03394 | 0.03211 | ni | conserved hypothetical protein |
| 5c00002 | 0.13281 | ni | hypothetical protein | um10060.2 | 0.03231 | ni | related to YIH1 |
| 18d00015 | 0.13287 | ni | hypothetical protein | um10196 | 0.03232 | K02901 | probable 60S large subunit ribosomal protein L27 |
| 24d00060 | 0.13287 | ni | hypothetical protein | um04285 | 0.03233 | K01637 | probable isocitrate lyase |
| 14c00033 | 0.13306 | ni | ribosomal protein S6 kinase and related proteins | um12093 | 0.03257 | ni | putative protein |
| 22c00167 | 0.13323 | ni | hypothetical protein | um15062 | 0.03261 | ni | conserved hypothetical protein |
| 5d00142 | 0.1333 | ni | gamma-tubulin ring complex protein | um01012 | 0.03267 | ni | conserved hypothetical protein |
| 22d00214 | 0.13334 | ni | hypothetical protein | um00916 | 0.03275 | ni | conserved hypothetical protein |
| 7c00322 | 0.13352 | ni | hypothetical protein | um11422 | 0.03276 | ni | conserved hypothetical protein |
| 22c00085 | 0.1337 | ni | VAMP-associated protein | um05665 | 0.03285 | ni | related to curved dna-binding protein |
| 9c00149 | 0.13373 | K07739 | RNA polymerase II elongator complex, subunit ELP3 | um10588 | 0.03286 | K12613 | related to decapping enzyme |
| 25d00071 | 0.1339 | ni | uncharacterized conserved protein | um10975.2 | 0.03287 | ni | conserved hypothetical protein |
| 9d00405 | 0.13416 | K15505 | helicase-like transcription factor HLTF | um06076 | 0.03289 | ni | related to quinate transport protein |
| 9c00154 | 0.13437 | ni | hypothetical protein | um06151 | 0.03294 | ni | conserved hypothetical protein |
| 9d00095 | 0.13442 | ni | hypothetical protein | um03209 | 0.03302 | K14007 | probable SEC24 - COPII coated vesicle component |
| 5d00125 | 0.13466 | K14545 | rRNA processing protein RRP7 | um05546 | 0.03306 | K15109 | conserved hypothetical protein |
| 7c00308 | 0.13477 | ni | hypothetical protein | um01607 | 0.0332 | ni | related to Translin |
| 15c00047 | 0.13508 | ni | drebrins and related actin binding proteins | um10277.2 | 0.03321 | K00698 | chitin synthase 5 |
| 22c00218 | 0.13513 | K07304 | peptide methionine sulfoxide reductase | um10172 | 0.03327 | K13342 | related to peroxisomal targeting signal receptor |
| 9c00162 | 0.13548 | K00566 | tRNA (5-methylaminomethyl-2-thiouridylate)-methyltransferase | um11700.2 | 0.03328 | K07404 | conserved hypothetical protein |
| 8c00013 | 0.13569 | ni | molecular chaperone Prefoldin, subunit 3 | um11047 | 0.03336 | ni | conserved hypothetical protein |
| 2d00048 | 0.13589 | K10956 | transport protein Sec61, alpha subunit | um10858 | 0.03337 | K03000 | probable RPA12 - 13 |
| 14c00043 | 0.13623 | ni | hypothetical protein | um12324 | 0.03342 | ni | hypothetical protein |
| 9d00055 | 0.13623 | ni | monocarboxylate transporter | um03066 | 0.03348 | ni | hypothetical protein |
| 9c00271 | 0.13625 | ni | diadenosine polyphosphate hydrolase and related proteins | um10462 | 0.03351 | ni | conserved hypothetical protein |
| 9c00376 | 0.13628 | ni | transferrin receptor and related proteins | um02232 | 0.03355 | ni | putative protein |
| 9d00326 | 0.13633 | K10591 | ubiquitin protein ligase RSP5/NEDD4 | um02477 | 0.03357 | K02264 | probable cytochrome-c oxidase chain VI precursor |
| 1c00051 | 0.13635 | ni | hypothetical protein | um01928 | 0.03363 | ni | putative protein |
| 15d00050 | 0.13652 | ni | hypothetical protein | um05992 | 0.03367 | ni | related to ENT3 - cytoskeletal adaptor |
| 5d00029 | 0.13671 | ni | hypothetical protein | um00407 | 0.03367 | ni | probable Tartrate dehydrogenase |
| 2c00037 | 0.13674 | K14297 | nuclear pore complex, Nup98 component | um01115 | 0.03377 | ni | related to OSH3 - Member of oxysterol-binding protein family |
| 6d00108 | 0.13677 | ni | galactosyltransferases | um05335 | 0.03377 | ni | related to Protein C20orf43 |
| 3c00070 | 0.13707 | ni | Na+/K+ ATPase, alpha subunit | um04707 | 0.03395 | K01424 | related to asparaginase 2 |
| 24c00041 | 0.13732 | ni | protein required for meiosis | um02799 | 0.03413 | K01613 | related to phosphatidylserine decarboxylase |
| 8c00044 | 0.13738 | ni | phosphatidylinositol transfer protein SEC14 and related proteins | um03929 | 0.03416 | ni | conserved hypothetical protein |
| 15c00085 | 0.13758 | ni | hypothetical protein | um05520 | 0.03419 | ni | conserved hypothetical protein |
| 8c00017 | 0.13761 | ni | hypothetical protein | um04976 | 0.03426 | K02328 | related to HYS2 - DNA-directed DNA polymerase delta |
| 22c00159 | 0.13762 | ni | predicted pilin-like transcription factor | um11095.2 | 0.03429 | K01873 | probable VAS1 - valyl-tRNA synthetase |
| 4c00010 | 0.13772 | ni | hypothetical protein | um11948 | 0.03432 | K13113 | related to ubiquitin-like protein Hub1 |
| 11d00009 | 0.13793 | K10807 | ribonucleotide reductase, alpha subunit | um00568 | 0.0344 | K15732 | related to FCP1 - TFIIF interacting component of CTD phosphatase |
| 18c00017 | 0.13799 | K05956 | protein geranylgeranyltransferase type II, beta subunit | um04159 | 0.03445 | K01807 | probable RKI1 - D-ribose-5-phosphate ketol-isomerase |
| 27d00065 | 0.13805 | K01490 | adenosine monophosphate deaminase | um11875 | 0.03454 | K00787 | probable ERG20 - farnesyl-pyrophosphate synthetase |
| 9d00203 | 0.13808 | K02920 | 60S ribosomal protein L36 | um05186 | 0.03488 | K10696 | conserved hypothetical protein |
| 9d00282 | 0.13809 | K12592 | DNA-binding protein C1D | um10982 | 0.03497 | ni | related to ribonucleoprotein |
| 27c00035 | 0.1388 | K12878 | nuclear matrix protein | um00238 | 0.035 | ni | ni |
| 6d00003 | 0.13893 | ni | synaptic vesicle transporter SVOP and related transporters | um11255 | 0.03509 | ni | putative protein |
| 26d00059 | 0.13896 | ni | FOG, Zn-finger | um00013 | 0.0351 | ni | conserved hypothetical protein |
| 6c00033 | 0.13928 | ni | predicted mechanosensitive ion channel | um00058 | 0.03513 | ni | hypothetical protein |
| 12d00010 | 0.13933 | ni | hypothetical protein | um03692 | 0.03532 | K01187 | probable alpha-glucosidase |
| 27c00084 | 0.13934 | K11092 | U2-associated snRNP A' protein | um00039 | 0.03535 | ni | conserved hypothetical protein |

| | | | | | | | |
|---|---|---|---|---|---|---|---|
| 15d00055 | 0.13939 | K03015 | DNA-directed RNA polymerase subunit E' | um04173 | 0.03536 | K05236 | probable COP1 - coatomer complex alpha chain of secretory pathway vesicles |
| 7d00177 | 0.13941 | ni | hypothetical protein | um03914 | 0.03542 | ni | related to Lactaldehyde reductase |
| 14c00046 | 0.1395 | K11979 | uncharacterized conserved protein | um12005 | 0.03545 | K02737 | probable PRE2 - 20S core proteasome subunit |
| 15d00045 | 0.13977 | ni | methyltransferases | um12234 | 0.0355 | K08336 | probable Autophagy-related protein 12 |
| 19c00087 | 0.13978 | K01487 | atrazine chlorohydrolase | um04529 | 0.0355 | K02320 | related to POL1 - DNA-directed DNA polymerase alpha |
| 19c00107 | 0.13986 | ni | predicted cell surface protein homologous to bacterial outer meml | um02162 | 0.03551 | ni | conserved hypothetical protein |
| 26d00001 | 0.14002 | ni | synaptic vesicle transporter SVOP and related transporters | um06188 | 0.03556 | K03178 | probable UBA1 - ubiquitin-protein ligase |
| 19c00001 | 0.14022 | ni | hypothetical protein | um00816 | 0.03573 | ni | probable thiamine biosynthesis protein nmt1 |
| 3d00014 | 0.14022 | K00133 | aspartate-semialdehyde dehydrogenase | um00933 | 0.03598 | ni | conserved hypothetical protein |
| 3d00027 | 0.14025 | ni | hypothetical protein | um02324 | 0.03598 | K03008 | related to DNA-directed RNA polymerase 13 |
| 19c00066 | 0.14038 | ni | 2-enoyl-CoA hydratase | um03858 | 0.03598 | K01663 | probable HIS7 - glutamine amidotransferase |
| 7c00095 | 0.1405 | ni | hypothetical protein | um05146 | 0.03607 | ni | putative protein |
| 7c00290 | 0.14053 | ni | hypothetical protein | um03303 | 0.03614 | K16572 | related to gamma-tubulin complex component 5 |
| 9d00261 | 0.1406 | ni | hypothetical protein | um10008 | 0.03618 | ni | conserved hypothetical protein |
| 8c00123 | 0.1409 | K03782 | hypothetical protein | um04902 | 0.03619 | K02202 | probable KIN28 - cyclin-dependent ser |
| 8c00068 | 0.14101 | ni | hypothetical protein | um11757.2 | 0.0363 | ni | conserved hypothetical protein |
| 13d00027 | 0.14102 | K03364 | anaphase promoting complex, Cdc20, Cdh1, and Ama1 subunits | um03607 | 0.03632 | K13830 | probable ARO1 - Pentafunctional AROM polypeptide |
| 7d00274 | 0.14144 | ni | hypothetical protein | um05225 | 0.03638 | ni | conserved hypothetical protein |
| 26d00069 | 0.14169 | K14779 | ATP-dependent RNA helicase | um10849 | 0.03667 | ni | putative protein |
| 5c00139 | 0.14169 | ni | uncharacterized conserved protein | um12337 | 0.0367 | K17361 | related to acyl-coa thioester hydrolase |
| 6d00056 | 0.14186 | ni | hypothetical protein | um01312 | 0.03672 | ni | related to Vacuolar assembly protein VPS41 |
| 19c00122 | 0.14191 | K07160 | hypothetical protein | um01646 | 0.03692 | K02212 | probable replication licensing factor MCM4 |
| 20c00032 | 0.14207 | ni | NIPSNAP1 protein | um04799 | 0.03699 | K14294 | conserved hypothetical protein |
| 11c00022 | 0.14209 | K03380 | monooxygenase | um01170 | 0.03703 | ni | conserved hypothetical protein |
| 25c00067 | 0.14223 | ni | hypothetical protein | um11835 | 0.03721 | ni | conserved hypothetical protein |
| 13c00064 | 0.14235 | ni | hypothetical protein | um03249 | 0.0373 | ni | ni |
| 5d00042 | 0.14239 | ni | cyclin | um12184 | 0.03731 | ni | conserved hypothetical protein |
| 7c00039 | 0.1424 | K17413 | mitochondrial ribosomal protein S28 | um05590 | 0.03731 | ni | related to NAD |
| 7d00188 | 0.14262 | ni | cell cycle control protein | um10435 | 0.03741 | K14319 | probable ran GTPase activating protein 1 |
| 13c00039 | 0.1428 | K01288 | serine carboxypeptidases | um10840 | 0.03743 | K00288 | probable MIS1 - C1-tetrahydrofolate synthase |
| 9d00232 | 0.14364 | ni | hypothetical protein | um10642 | 0.03743 | K12627 | related to LSM8 - Component of small nuclear ribonucleoprotein complexes involved in RNA processing and splicing |
| 22d00103 | 0.14389 | K02978 | 40s ribosomal protein S27 | um05861 | 0.03756 | K05909 | related to FET5 - multicopper oxidase |
| 18d00028 | 0.14417 | K09184 | GATA-4/5/6 transcription factors | um00202 | 0.03757 | K00490 | related to Cytochrome P450 4F8 |
| 10d00099 | 0.14428 | ni | iron/ascorbate family oxidoreductases | um03963 | 0.03763 | ni | nuclear pore membrane protein |
| 4c00039 | 0.14434 | ni | phosphorylase kinase gamma subunit | um05329 | 0.03765 | ni | related to flavin-containing monooxygenase |
| 7c00086 | 0.14436 | K13989 | predicted membrane protein | um03343 | 0.03766 | ni | putative protein |
| 12c00072 | 0.14436 | ni | protein phosphatase 2C | um10693 | 0.03797 | ni | conserved hypothetical protein |
| 6d00032 | 0.1444 | ni | hypothetical protein | um00995 | 0.038 | K11566 | conserved hypothetical protein |
| 22d00215 | 0.14463 | ni | inorganic phosphate transporter | um04573 | 0.03801 | K11128 | probable nucleolar rRNA processing protein GAR1 |
| 11d00026 | 0.14471 | ni | hypothetical protein | um06472 | 0.0381 | ni | conserved hypothetical protein |
| 9c00281 | 0.14511 | K02735 | 20S proteasome, regulatory subunit beta type PSMB3/PUP3 | um10844 | 0.03813 | ni | conserved hypothetical protein |
| 9d00291 | 0.14524 | K01069 | glyoxylase | um00418 | 0.03816 | ni | probable xylitol dehydrogenase |
| 9c00386 | 0.14562 | ni | hypothetical protein | um06389 | 0.03821 | ni | conserved hypothetical protein |
| 6c00059 | 0.14568 | ni | reductases with broad range of substrate specificities | um02433.2 | 0.03822 | K06316 | related to nuclear division protein Rft1 |
| 2d00023 | 0.14592 | ni | hypothetical protein | um06396 | 0.0383 | ni | putative protein |
| 7c00274 | 0.14614 | ni | nucleoside phosphatase | um05740 | 0.03834 | K01687 | probable ILV3 - dihydroxy-acid dehydratase |
| 7c00268 | 0.14658 | K10685 | SMT3/SUMO-activating complex, catalytic component UBA2 | um04512 | 0.03838 | ni | related to DnaJ protein |
| 14d00012 | 0.14659 | K14544 | nucleolar RNA-associated protein | um01482 | 0.03841 | ni | hypothetical protein |
| 5d00134 | 0.14666 | ni | hypothetical protein | um02134 | 0.0385 | ni | related to mixed-linked glucanase precursor MLG1 |
| 13d00088 | 0.14674 | ni | hypothetical protein | um03232 | 0.03852 | ni | conserved hypothetical Ustilago-specific protein |
| 7c00035 | 0.14688 | K07750 | C-4 sterol methyl oxidase | um11981 | 0.03853 | ni | putative protein |
| 6c00061 | 0.14691 | ni | RNA pseudouridylate synthases | um00060 | 0.03867 | ni | conserved hypothetical Ustilago-specific protein |
| 9d00082 | 0.14701 | ni | hypothetical protein | um05242 | 0.03869 | ni | ni |
| 7d00012 | 0.14701 | ni | hypothetical protein | um02797 | 0.03871 | ni | conserved hypothetical protein |
| 9d00230 | 0.1473 | ni | hypothetical protein | um15088 | 0.03891 | ni | conserved hypothetical protein |
| 20c00008 | 0.14732 | K03305 | H+/oligopeptide symporter | um10634 | 0.03892 | ni | conserved hypothetical protein |
| 14c00018 | 0.14761 | K13103 | tuftelin-interacting protein TIP39 | um00400 | 0.03898 | K06101 | conserved hypothetical protein |
| 7c00145 | 0.14782 | ni | hypothetical protein | um05415 | 0.039 | K09527 | related to tetratricopeptide repeat protein 2 |
| 14d00042 | 0.14812 | K01803 | triosephosphate isomerase | um01279 | 0.03906 | K09493 | probable TCP1 - component of chaperonin-containing T-complex |
| 22d00115 | 0.14824 | ni | hypothetical protein | um01934 | 0.03908 | K09829 | C-8 sterol isomerase |
| 2c00085 | 0.1485 | K12828 | splicing factor 3b, subunit 1 | um12133 | 0.03917 | ni | conserved hypothetical protein |
| 13c00079 | 0.1486 | ni | hypothetical protein | um10557 | 0.03918 | ni | conserved hypothetical Ustilago-specific protein |
| 14c00090 | 0.14865 | ni | chromatin remodeling complex SWI/SNF, component SWI2 | um05825 | 0.03921 | K07300 | probable VCX1 - Vacuolar Ca |
| 19d00079 | 0.1489 | ni | ribonuclease inhibitor type leucine-rich repeat proteins | um10880 | 0.03923 | ni | related to Graves disease carrier protein |
| 7d00123 | 0.14903 | ni | hypothetical protein | um11515 | 0.0393 | ni | putative protein |
| 22d00209 | 0.14916 | ni | mitochondrial carrier protein PET8 | um05353 | 0.03934 | ni | putative protein |
| 5c00044 | 0.14916 | ni | hypothetical protein | um12209 | 0.03942 | K06675 | related to SMC4 - Stable Maintenance of Chromosomes |
| 19c00036 | 0.14942 | ni | ER to golgi transport protein | um05894 | 0.03944 | ni | conserved hypothetical protein |
| 26d00072 | 0.14944 | K00101 | glycolate oxidase | um01941 | 0.03945 | ni | conserved hypothetical protein |
| 6d00062 | 0.14957 | ni | hypothetical protein | um03333 | 0.03947 | ni | conserved hypothetical protein |
| 14c00119 | 0.14961 | K13344 | peroxisomal biogenesis protein peroxin | um03729 | 0.03954 | K13950 | related to para-aminobenzoic acid synthetase |
| 22c00014 | 0.14963 | ni | hypothetical protein | um01116 | 0.03955 | ni | conserved hypothetical protein |
| 2d00058 | 0.14998 | K01144 | oxysterol-binding protein | um05069 | 0.03959 | ni | related to short-chain dehydrogenase |
| 19d00123 | 0.15014 | K03956 | hypothetical protein | um05192 | 0.03966 | ni | conserved hypothetical protein |
| 18d00063 | 0.15033 | ni | hypothetical protein | um01653 | 0.0399 | ni | related to VPS73 - protein involved in vacuolar protein sorting |
| 5d00016 | 0.15035 | K03648 | uracil DNA glycosylase | um02388 | 0.0399 | K05754 | related to subunit of the Arp2 |
| 7c00304 | 0.15065 | K12177 | COP9 signalosome, subunit CSN3 | um04005 | 0.03993 | ni | conserved hypothetical protein |
| 7c00245 | 0.15078 | K13123 | predicted RNA binding protein | um02635 | 0.04004 | ni | conserved hypothetical protein |
| 7c00234 | 0.15082 | ni | hypothetical protein | um10440.2 | 0.04006 | K01772 | related to HEM15 - ferrochelatase precursor |
| 7c00023 | 0.15085 | K01866 | tyrosyl-trna synthetase | um10350 | 0.04012 | ni | putative protein |
| 9c00330 | 0.15088 | ni | hypothetical protein | um11557 | 0.04015 | ni | related to YJU2 - Essential nuclear protein |
| 11c00038 | 0.15105 | K11344 | uncharacterized conserved protein | um12199 | 0.04018 | ni | related to decapping enzyme |
| 7c00255 | 0.15121 | ni | hypothetical protein | um03465 | 0.04021 | K01288 | related to KEX1 protein precursor |
| 8c00027 | 0.15147 | K13830 | pentafunctional AROM protein | um11899 | 0.04027 | ni | conserved hypothetical protein |
| 19d00138 | 0.15147 | ni | amidases | um05798 | 0.04038 | ni | hypothetical protein |
| 11c00071 | 0.15159 | ni | molecular chaperones HSP70/HSC70 | um10019 | 0.04047 | K03639 | related to Molybdopterin biosynthesis CNX2 protein |
| 16c00007 | 0.15168 | K01607 | hypothetical protein | um00141 | 0.04067 | K11563 | hypothetical protein |
| 22d00086 | 0.15171 | ni | hypothetical protein | um03495 | 0.04067 | K11884 | probable PNO1 protein |
| 9c00075 | 0.15193 | ni | amidases | um06156 | 0.04083 | K03018 | probable RPO31 - DNA-directed RNA polymerase III |
| 19d00015 | 0.15201 | K00002 | aldo/keto reductase family proteins | um05770 | 0.04086 | K06628 | TSD2 protein |
| 24c00056 | 0.15238 | ni | phosphatidylinositol-4-phosphate 5-kinase | um11174 | 0.04088 | ni | conserved hypothetical protein |
| 5c00078 | 0.1524 | ni | predicted regulator of the ubiquitin pathway | um01402 | 0.04093 | K01736 | probable ARO2 - chorismate synthase |
| 22c00277 | 0.15255 | K14022 | hypothetical protein | um06174 | 0.04098 | ni | related to YEH1 - steryl ester hydrolase |
| 26d00010 | 0.15276 | ni | uncharacterized conserved protein | um10095 | 0.04104 | K12823 | probable RNA helicase dbp2 |
| 18c00053 | 0.15283 | ni | HGG motif-containing thioesterase | um04277 | 0.04114 | K00670 | probable N-acetyltransferase 5 |
| 15d00051 | 0.15283 | ni | hypothetical protein | um04417 | 0.04118 | ni | probable CRN1 - a coronin |
| 8c00020 | 0.15289 | K13341 | peroxisomal targeting signal type 2 receptor | um10576 | 0.04127 | ni | related to SIW14 - Tyrosine phosphatase involved in actin filament organization |
| 5d00083 | 0.1531 | K12817 | U5 snRNP-associated RNA splicing factor | um12002 | 0.04138 | ni | conserved hypothetical protein |
| 25d00050 | 0.15358 | K11232 | two-component phosphorelay intermediate | um06261.2 | 0.04158 | K13535 | conserved hypothetical protein |
| 11d00020 | 0.15358 | K06100 | mRNA cleavage and polyadenylation factor II complex, subunit P' | um03642 | 0.0416 | ni | conserved hypothetical protein |
| 10c00003 | 0.15365 | ni | FOG, LIM domain | um10814 | 0.04164 | ni | conserved hypothetical protein |
| 19c00143 | 0.15367 | ni | dTDP-glucose 4-6-dehydratase | um02723.2 | 0.04177 | ni | probable mfs-multidrug-resistance transporter |
| 19c00023 | 0.15391 | ni | hypothetical protein | um01162 | 0.04184 | ni | related to golgi-specific brefeldin a-resistance guanine nucleotide exchange factor 1 |
| 7c00228 | 0.15404 | ni | hypothetical protein | um11771 | 0.04198 | K07441 | conserved hypothetical protein |
| 6d00015 | 0.15432 | ni | hypothetical protein | um11698 | 0.04198 | K02147 | probable VMA2 - H -ATPase V1 domain 60 KD subunit |
| 11c00050 | 0.15436 | K15100 | mitochondrial tricarboxylate/dicarboxylate carrier proteins | um05426 | 0.04211 | ni | conserved hypothetical Ustilago-specific protein |
| 16c00040 | 0.15453 | ni | hypothetical protein | um11802 | 0.04217 | K03661 | probable PPA1 - H -ATPase 23 KD subunit |
| 9c00155 | 0.1547 | ni | RNA-binding protein | um00963 | 0.0423 | K11121 | related to HST1 - silencing protein |
| 12c00029 | 0.1547 | K03671 | thioredoxin | um10926 | 0.04234 | K02145 | probable TFP1p - H -ATPase V1 domain 69 KD catalytic subunit |
| 3c00017 | 0.15473 | ni | uncharacterized conserved protein | um00875 | 0.04237 | ni | hypothetical protein |
| 9c00183 | 0.15493 | ni | hypothetical protein | um01662 | 0.04245 | K11229 | related to BCK1 ser |
| 22c00063 | 0.15498 | ni | hypothetical protein | um04560 | 0.04245 | ni | putative protein |
| 9c00280 | 0.15507 | K02735 | 20S proteasome, regulatory subunit beta type PSMB3/PUP3 | um10159 | 0.04255 | ni | related to transcription activator amyR |
| 9c00308 | 0.15515 | ni | ypt/rab GTPase activating protein | um00558 | 0.04279 | ni | conserved hypothetical Ustilago-specific protein |
| 22c00261 | 0.1552 | ni | hypothetical protein | um12219 | 0.04281 | ni | conserved hypothetical protein |
| 12c00147 | 0.15532 | ni | hypothetical protein | um00056 | 0.04282 | ni | probable neutral amino acid permease |
| 5d00103 | 0.15545 | ni | vacuolar sorting protein VPS33/slp1 | um11277 | 0.04297 | ni | conserved hypothetical protein |
| 12d00138 | 0.15545 | ni | arylacetamide deacetylase | um03529 | 0.04312 | K02906 | related to MRPL9 - mitochondrial ribosomal protein |
| 26d00037 | 0.15547 | ni | karyopherin (importin) alpha | um02476 | 0.04322 | ni | related to coatomer delta subunit |
| 2c00007 | 0.15555 | ni | predicted transporter | um06302 | 0.04328 | ni | putative protein |
| 13d00057 | 0.15567 | ni | hypothetical protein | um00635 | 0.04334 | ni | conserved hypothetical protein |
| 6c00038 | 0.15575 | K01738 | cysteine synthase | um02179 | 0.0434 | ni | putative protein |
| 13d00023 | 0.15585 | K02183 | calmodulin and related proteins | um11137.2 | 0.04341 | ni | conserved hypothetical protein |
| 25c00061 | 0.15589 | K01687 | dihydroxy-acid dehydratase | um04220 | 0.04358 | K01850 | probable ARO7 - chorismate mutase |
| 9d00376 | 0.15612 | ni | hypothetical protein | um00417 | 0.04379 | ni | conserved hypothetical protein |
| 7d00134 | 0.15619 | K13195 | FOG, RRM domain | um11851 | 0.04383 | ni | conserved hypothetical protein |
| 7c00180 | 0.15622 | ni | mRNA splicing factor | um06418 | 0.0439 | ni | related to polyketide synthase |
| 8d00071 | 0.15624 | ni | emp24/gp25L/p24 family of membrane trafficking proteins | um02743 | 0.04402 | ni | conserved hypothetical protein |
| 7c00089 | 0.15629 | K03358 | anaphase-promoting complex (APC), subunit 11 | um11232 | 0.04414 | K05692 | probable Actin |
| 9c00205 | 0.15632 | K03953 | NADH:ubiquinone oxidoreductase, NDUFA9/39kDa subunit | um00551 | 0.04423 | ni | related to putative C2H2 zinc finger protein flbC |
| 11c00044 | 0.15638 | ni | FOG, FHA domain | um05857 | 0.04427 | K14812 | conserved hypothetical protein |
| 7c00208 | 0.15642 | ni | hypothetical protein | um04813.2 | 0.04444 | ni | conserved hypothetical protein |
| 13c00080 | 0.15646 | ni | hypothetical protein | um02582 | 0.04464 | K11446 | regulator Ustilago maydis 1 protein |
| 7d00042 | 0.1568 | ni | hypothetical protein | um10085 | 0.04472 | ni | conserved hypothetical protein |
| 15c00030 | 0.15682 | K01210 | hypothetical protein | um02485 | 0.04473 | K07889 | probable GTP-binding protein ypt5 |
| 25d00073 | 0.15685 | ni | sirtuin 5 and related class III sirtuins | um03011 | 0.0451 | ni | conserved hypothetical protein |
| 7d00285 | 0.15686 | K09490 | molecular chaperones GRP78/BiP/KAR2 | um12275 | 0.04521 | ni | hypothetical protein |
| 20d00085 | 0.15703 | ni | non-ribosomal peptide synthetase | um02900 | 0.0453 | K08369 | conserved hypothetical protein |
| 27c00089 | 0.15736 | K17362 | HGG motif-containing thioesterase | um05111 | 0.04541 | K01866 | probable tyrosyl-tRNA synthetase |
| 9d00138 | 0.15764 | ni | hypothetical protein | um03082 | 0.04554 | ni | conserved hypothetical protein |
| 22c00034 | 0.15769 | K01426 | amidases | um11939 | 0.04557 | ni | conserved hypothetical protein |
| 10d00031 | 0.15781 | ni | hypothetical protein | um05845 | 0.04559 | ni | ni |
| 9c00121 | 0.15786 | ni | hypothetical protein | um10260 | 0.04563 | K07304 | related to MXR1 - protein-methionine-s-oxide reductase |
| 8c00080 | 0.15792 | ni | hypothetical protein | um00637 | 0.04564 | ni | related to triacylglycerol lipase |
| 14d00046 | 0.15811 | ni | hypothetical protein | um12285 | 0.04566 | ni | related to CDC123 - protein controls the cell cycle by controlling eIF2gamma abundance |
| 9d00132 | 0.15813 | ni | hypothetical protein | um05100 | 0.04578 | ni | conserved hypothetical protein |
| 7d00232 | 0.15837 | ni | serine/threonine protein kinase | um03573 | 0.04598 | ni | putative protein |
| 5c00031 | 0.15853 | ni | sensory transduction histidine kinase | um10057 | 0.04599 | K12873 | probable G10 protein |
| 7c00186 | 0.15875 | K10733 | uncharacterized conserved protein | um06385 | 0.04606 | ni | conserved hypothetical protein |
| 13c00101 | 0.15877 | K01739 | cystathionine beta-lyases | um10546 | 0.04607 | K11495 | probable histone 3 |
| 9d00136 | 0.15923 | ni | predicted membrane protein | um10569 | 0.04611 | K05931 | related to HMT1 - hnRNP arginine N-methyltransferase |
| 22d00280 | 0.1594 | ni | hypothetical protein | um11829 | 0.04621 | ni | conserved hypothetical protein |
| 9d00220 | 0.15952 | K00914 | phosphatidylinositol 3-kinase VPS34 | um12014 | 0.04624 | K01126 | conserved hypothetical protein |
| 5c00014 | 0.1597 | K12880 | WD40 repeat protein | um02630 | 0.0463 | ni | ni |
| 9c00011 | 0.15995 | ni | hypothetical protein | um10573 | 0.04633 | K02937 | probable 60s ribosomal protein L7 subunit |
| 4d00003 | 0.16 | ni | hypothetical protein | um03078 | 0.04643 | ni | conserved hypothetical protein |

| | | | | | | | |
|---|---|---|---|---|---|---|---|
| 8c00097 | 0.16009 | K01501 | carbon-nitrogen hydrolase | um11491 | 0.04645 | ni | putative protein |
| 22c00204 | 0.16018 | ni | peptidase family M48 | um12192 | 0.04646 | ni | related to Tim22 |
| 13c00088 | 0.16026 | ni | hypothetical protein | um00620 | 0.04652 | K11567 | conserved hypothetical protein |
| 6d00110 | 0.16036 | K05853 | Ca2+ transporting ATPase | um10886.2 | 0.04658 | ni | conserved hypothetical protein |
| 26c00075 | 0.1605 | ni | hypothetical protein | um11319 | 0.04661 | ni | probable SEC14 - phosphatidylinositol |
| 22c00112 | 0.16074 | ni | hypothetical protein | um01033.2 | 0.04664 | K08333 | related to VPS15 - ser |
| 7d00216 | 0.16153 | K03417 | isocitrate lyase | um02817 | 0.04676 | ni | conserved hypothetical Ustilago-specific protein |
| 10c00010 | 0.16166 | K02575 | hypothetical protein | um01568 | 0.04687 | ni | putative protein |
| 12d00025 | 0.16174 | ni | hypothetical protein | um10190.2 | 0.04704 | K06062 | putative histone acetylase |
| 9c00252 | 0.16187 | K12600 | TPR repeat-containing protein | um01637 | 0.04738 | K08516 | probable YKT6 - SNARE protein for Endoplasmic Reticulum-Golgi transport |
| 19d00056 | 0.16189 | ni | hypothetical protein | um01195 | 0.04744 | K17218 | related to sulfide |
| 11d00080 | 0.16216 | ni | hypothetical protein | um02002 | 0.04748 | ni | putative protein |
| 5d00053 | 0.16218 | K03841 | fructose-1 | um00967 | 0.04752 | ni | conserved hypothetical protein |
| 7d00164 | 0.16244 | ni | translocase of outer mitochondrial membrane complex, subunit To | um04977 | 0.0477 | ni | putative protein |
| 7c00059 | 0.16244 | ni | oxidosqualene-lanosterol cyclase and related proteins | um05641 | 0.04778 | K01074 | related to palmitoyl-protein thioesterase 1 |
| 22d00050 | 0.16256 | ni | small Nuclear ribonucleoprotein G | um10541 | 0.04787 | ni | conserved hypothetical protein |
| 22c00271 | 0.16276 | ni | hypothetical protein | um03728 | 0.04792 | K00463 | conserved hypothetical protein |
| 11c00041 | 0.16276 | K10949 | ER lumen protein retaining receptor | um11858 | 0.04804 | ni | conserved hypothetical protein |
| 3d00100 | 0.16298 | ni | HLH transcription factor EBF/Olf-1 | um12163 | 0.0481 | ni | conserved hypothetical protein |
| 14c00070 | 0.16309 | ni | hypothetical protein | um02605.2 | 0.0481 | ni | putative protein |
| 25d00022 | 0.1634 | K05290 | GPI-anchor transamidase | um05449 | 0.04811 | ni | related to RPH1 - Transcriptional repressor of PHR1 |
| 7c00102 | 0.16348 | ni | hypothetical protein | um10148 | 0.04834 | ni | putative protein |
| 19c00051 | 0.16358 | ni | hypothetical protein | um03007 | 0.04867 | K06944 | probable RBG1 - ribosome interacting GTPase |
| 11c00005 | 0.16366 | K05765 | actin depolymerizing factor | um03630 | 0.04868 | ni | related to metalloprotease MEP1 |
| 27c00053 | 0.16376 | ni | hypothetical protein | um01692 | 0.0488 | ni | related to CHA1 - L-serine |
| 11d00081 | 0.16376 | ni | conserved WD40 repeat-containing protein | um00533 | 0.04884 | ni | related to PPR1 - transcription factor regulating pyrimidine pathway |
| 2d00018 | 0.16387 | ni | glutamate decarboxylase | um11488 | 0.04893 | ni | probable ERV14 - ER-derived vesicles |
| 11d00087 | 0.16394 | ni | hypothetical protein | um04875 | 0.04906 | ni | conserved hypothetical protein |
| 8c00125 | 0.16399 | K00128 | aldehyde dehydrogenase | um02530 | 0.04908 | K11372 | conserved hypothetical protein |
| 14c00048 | 0.16411 | ni | uncharacterized conserved protein | um10098 | 0.0491 | ni | related to TAO3 - Transcriptional Activator of OCH1 |
| 9c00419 | 0.16416 | K03955 | acyl carrier protein | um11018 | 0.04916 | ni | putative protein |
| 1c00010 | 0.16453 | K08269 | serine/threonine-protein kinase | um11131 | 0.04917 | ni | putative protein |
| 27d00039 | 0.16468 | K03037 | 26S proteasome regulatory complex, subunit RPN7/PSMD6 | um04426 | 0.04921 | ni | conserved hypothetical protein |
| 3d00087 | 0.16481 | ni | hypothetical protein | um02514.2 | 0.04921 | ni | related to UPF3 - Nonsense-mediated mRNA decay protein |
| 16c00046 | 0.16491 | ni | hypothetical protein | um03638 | 0.04926 | ni | hypothetical protein |
| 6c00018 | 0.16506 | K01624 | fructose 1,6-bisphosphate aldolase | um11445 | 0.04927 | ni | putative protein |
| 3c00020 | 0.16517 | ni | hypothetical protein | um10445 | 0.04931 | ni | conserved hypothetical protein |
| 20d00044 | 0.16521 | ni | hypothetical protein | um11193 | 0.04965 | ni | putative protein |
| 11d00052 | 0.16543 | K00606 | ketopantoate hydroxymethyltransferase | um04049 | 0.04974 | ni | conserved hypothetical protein |
| 25d00051 | 0.16553 | ni | uncharacterized conserved protein | um01518 | 0.04977 | ni | putative protein |
| 12c00020 | 0.16562 | ni | phosphatidylinositol transfer protein SEC14 and related proteins | um01391 | 0.04997 | K01090 | related to phosphoprotein phosphatase 2C |
| 7d00102 | 0.16563 | K15900 | predicted metalloprotease with chaperone activity | um03632 | 0.05002 | K06110 | related to SEC6 - protein transport protein |
| 26d00047 | 0.16569 | K14294 | hypothetical protein | um11001 | 0.05011 | ni | related to enoyl-coa hydratase protein 3 |
| 22c00151 | 0.1657 | K01443 | N-acetyl-glucosamine-6-phosphate deacetylase | um02639 | 0.05016 | K11838 | probable ubiquitin-specific processing protease 21 |
| 5c00110 | 0.166 | ni | hypothetical protein | um15021 | 0.0502 | ni | conserved hypothetical protein |
| 22c00232 | 0.16626 | ni | cytochrome P450 CYP4/CYP19/CYP26 subfamilies | um10574 | 0.05039 | K01077 | related to PHO8 - repressible alkaline phosphatase vacuolar |
| 9c00391 | 0.1664 | K15303 | hypothetical protein | um00766 | 0.05063 | ni | putative protein |
| 15c00009 | 0.16646 | ni | acyl-coa reductase | um06455 | 0.05078 | ni | conserved hypothetical protein |
| 22c00105 | 0.1665 | ni | hypothetical protein | um10986 | 0.05084 | ni | putative protein |
| 9d00330 | 0.16663 | ni | hypothetical protein | um03283 | 0.05118 | ni | conserved hypothetical protein |
| 26c00059 | 0.16686 | K01885 | glutamyl-trna synthetase | um03497 | 0.05119 | ni | conserved hypothetical protein |
| 6d00071 | 0.16707 | ni | N-methyltransferase | um12277 | 0.05133 | K09550 | probable GIM3 - Gim complex component |
| 8c00042 | 0.16719 | K14327 | nonsense-mediated mRNA decay 2 protein | um04966 | 0.05137 | ni | conserved hypothetical protein |
| 9c00215 | 0.1672 | K02542 | DNA replication licensing factor, MCM6 component | um03657 | 0.05139 | ni | conserved hypothetical protein |
| 7d00303 | 0.16749 | ni | serine palmitoyltransferase | um06347 | 0.05141 | ni | hypothetical protein |
| 13d00026 | 0.16756 | ni | alcohol dehydrogenase | um01251 | 0.05143 | ni | hypothetical protein |
| 20d00080 | 0.16765 | K02880 | 60S ribosomal protein L22 | um00163 | 0.05156 | K17262 | related to Tubulin-specific chaperone B |
| 6d00045 | 0.16837 | ni | hypothetical protein | um03925 | 0.05162 | ni | related to RDS2 - Regulator of drug sensitivity |
| 16c00058 | 0.16841 | K16536 | hypothetical protein | um05844 | 0.05168 | ni | conserved hypothetical protein |
| 9c00324 | 0.16844 | K00627 | dihydrolipoamide acetyltransferase | um01851 | 0.05178 | ni | conserved hypothetical protein |
| 9d00317 | 0.16854 | K00326 | NADH-cytochrome b-5 reductase | um10845 | 0.05182 | K03649 | conserved hypothetical protein |
| 12c00140 | 0.16855 | K13076 | delta 6-fatty acid desaturase | um02554 | 0.05194 | K06111 | related to SEC8 - protein transport protein |
| 15d00094 | 0.16858 | ni | hypothetical protein | um15026 | 0.05198 | K01187 | probable maltase |
| 16c00082 | 0.16876 | ni | hypothetical protein | um00581 | 0.052 | ni | conserved hypothetical protein |
| 9d00218 | 0.16885 | K05285 | glycosylphosphatidylinositol anchor synthesis protein | um03020 | 0.05206 | ni | conserved hypothetical protein |
| 25d00023 | 0.16896 | K11237 | NADPH oxidase | um11100 | 0.05209 | ni | putative protein |
| 27d00088 | 0.16909 | K14780 | DEAH-box RNA helicase | um05161 | 0.05211 | ni | related to Phosducin |
| 1d00045 | 0.16977 | ni | ni | um10759 | 0.05227 | ni | hypothetical protein |
| 13d00029 | 0.16979 | ni | membrane coat complex Retromer, subunit VPS5/SNX1 | um05554 | 0.05229 | ni | conserved hypothetical protein |
| 14d00068 | 0.16984 | K00106 | xanthine dehydrogenase | um01366 | 0.05257 | K06630 | probable BMH1 - 14-3-3 protein epsilon |
| 20d00049 | 0.16986 | K01427 | hypothetical protein | um12108 | 0.05294 | K08490 | probable syntaxin |
| 16d00037 | 0.16989 | ni | hypothetical protein | um05274 | 0.05307 | ni | ni |
| 9c00098 | 0.1699 | K13091 | transcriptional coactivator CAPER | um04128 | 0.05315 | ni | related to isopenicillin N epimerase |
| 20d00055 | 0.16996 | K11380 | PHD finger protein BR140/LIN-49 | um11772 | 0.05319 | ni | ni |
| 22d00183 | 0.16998 | ni | mitochondrial sulfhydryl oxidase | um12191 | 0.05331 | ni | hypothetical protein |
| 24c00031 | 0.17001 | ni | acyl-coa-binding protein | um04715 | 0.05338 | ni | conserved hypothetical protein |
| 22d00102 | 0.17003 | ni | hypothetical protein | um03023 | 0.05346 | K01166 | related to Ribonuclease Trv |
| 9c00344 | 0.17025 | K02154 | vacuolar H+-ATPase V0 sector, subunit a | um02081 | 0.0536 | K05853 | endoplasmic reticulum calcium transporter |
| 18c00007 | 0.1703 | K00698 | chitin synthase/hyaluronan synthase | um06397 | 0.05365 | ni | conserved hypothetical protein |
| 27d00002 | 0.17037 | ni | hypothetical protein | um00832 | 0.05371 | ni | conserved hypothetical protein |
| 18c00008 | 0.1704 | ni | 17 beta-hydroxysteroid dehydrogenase type 3 | um03680 | 0.05373 | K03136 | related to TFA1 - TFIIE subunit |
| 7d00292 | 0.17045 | ni | hypothetical protein | um03816 | 0.05376 | ni | related to PRP12 - involved in early maturation of pre-rRNA |
| 22d00046 | 0.17054 | K07393 | predicted glutathione S-transferase | um05050 | 0.05389 | ni | hypothetical protein |
| 22d00171 | 0.17077 | K01557 | predicted fumarylacetoacetate hydralase | um01549 | 0.05393 | ni | related to mitochondrial inheritance component mdm12 |
| 15c00082 | 0.17097 | ni | inorganic phosphate transporter | um02742 | 0.05395 | ni | conserved hypothetical protein |
| 7c00108 | 0.17121 | ni | hypothetical protein | um15082 | 0.05396 | ni | conserved hypothetical protein |
| 1d00014 | 0.17139 | K12580 | CCR4-NOT transcriptional regulation complex, NOT5 subunit | um04734 | 0.05397 | ni | putative protein |
| 26d00032 | 0.17141 | K15177 | uncharacterized conserved protein | um02568 | 0.05404 | ni | probable YCF1 - Vacuolar ABC transporter responsible for vacuolar sequestration of glutathione-S-conjugates |
| 9d00083 | 0.17141 | K00873 | pyruvate kinase | um00753 | 0.05411 | ni | related to DDR48 - heat shock protein |
| 7c00200 | 0.17142 | K14553 | WD40 repeat protein | um00878 | 0.05411 | K11321 | conserved hypothetical protein |
| 20c00017 | 0.17144 | K06942 | predicted GTP-binding protein | um03404 | 0.05412 | ni | hypothetical protein |
| 13d00030 | 0.17149 | K03064 | 26S proteasome regulatory complex | um04830 | 0.05418 | K15177 | conserved hypothetical protein |
| 7d00327 | 0.17149 | K02732 | 20S proteasome, regulatory subunit beta type PSMB1/PRE7 | um04212 | 0.05422 | ni | putative protein |
| 15d00031 | 0.17157 | ni | hypothetical protein | um04094 | 0.0543 | ni | conserved hypothetical Ustilago-specific protein |
| 15c00092 | 0.17172 | K08157 | synaptic vesicle transporter SVOP and related transporters | um02140 | 0.05443 | ni | ni |
| 18c00061 | 0.17176 | K13800 | uridylate kinase | um10221 | 0.05446 | ni | conserved hypothetical protein |
| 16d00015 | 0.17207 | ni | hypothetical protein | um05526 | 0.05447 | ni | conserved hypothetical protein |
| 7c00251 | 0.17211 | ni | GATA-4/5/6 transcription factors | um00515 | 0.05455 | ni | putative protein |
| 22d00043 | 0.17215 | ni | hypothetical protein | um02550 | 0.05459 | ni | putative protein |
| 9d00114 | 0.17225 | K11364 | histone acetyltransferase SAGA associated factor SGF29 | um04182 | 0.05474 | K00211 | probable TYR1 - prephenate dehydrogenase |
| 12d00085 | 0.17235 | ni | hypothetical protein | um05118 | 0.05475 | ni | putative protein |
| 9d00062 | 0.17257 | ni | zinc-binding oxidoreductase | um06335 | 0.05475 | ni | putative protein |
| 7d00349 | 0.17258 | ni | hypothetical protein | um00077 | 0.05477 | ni | conserved hypothetical protein |
| 6c00080 | 0.17267 | ni | uncharacterized conserved protein | um01885 | 0.05482 | ni | probable Alcohol dehydrogenase |
| 7d00217 | 0.1727 | K06640 | protein kinase of the PI-3 kinase family | um04400 | 0.05497 | ni | probable PRB1 - protease B |
| 22d00291 | 0.17276 | K01519 | inosine triphosphate pyrophosphatase | um03352 | 0.05498 | ni | conserved hypothetical protein |
| 10c00017 | 0.17291 | K04648 | microtubule-associated protein dynactin DCTN1/Glued | um00949 | 0.05499 | ni | probable RAS GTPase-activating protein sar1 |
| 19c00105 | 0.17296 | K14688 | Zn2+ transporter ZNT1 and related Cd2+/Zn2+ transporters | um12107 | 0.0552 | ni | conserved hypothetical protein |
| 5d00005 | 0.17298 | ni | amino acid transporters | um03340 | 0.05542 | K11129 | probable NHP2 - nucleolar rRNA processing protein |
| 18c00039 | 0.17301 | K08486 | SNARE protein Syntaxin 1 and related proteins | um00670 | 0.05548 | ni | conserved hypothetical protein |
| 7c00344 | 0.17327 | ni | hypothetical protein | um00795 | 0.0555 | ni | hypothetical protein |
| 10d00063 | 0.1733 | K09613 | COP9 signalosome, subunit CSN5 | um02740 | 0.05552 | K01187 | probable Alpha-glucosidase precursor |
| 12d00032 | 0.17332 | ni | predicted membrane protein | um10412 | 0.05563 | ni | conserved hypothetical protein |
| 27d00091 | 0.17333 | ni | serine/threonine protein kinase | um12312 | 0.05571 | ni | conserved hypothetical protein |
| 15c00016 | 0.1734 | ni | aldehyde dehydrogenase | um01550 | 0.05572 | ni | conserved hypothetical protein |
| 22d00191 | 0.17354 | ni | predicted alpha/beta hydrolase | um11590 | 0.05628 | K01412 | probable MAS1 - mitochondrial processing peptidase beta chain precursor |
| 11d00025 | 0.17365 | K14772 | DRIM (Down-regulated in metastasis)-like proteins | um11306 | 0.0564 | ni | putative protein |
| 8c00100 | 0.17377 | K00784 | septin family protein | um03129 | 0.05666 | ni | hypothetical protein |
| 13c00005 | 0.17404 | ni | hypothetical protein | um02653 | 0.05674 | K03843 | related to alpha-1 |
| 25c00051 | 0.17416 | ni | phosphatidic acid-preferring phospholipase A1 | um02333 | 0.05679 | ni | putative protein |
| 18d00110 | 0.17418 | ni | hypothetical protein | um05503 | 0.05692 | K02144 | related to vacuolar ATP synthase subunit H |
| 22d00048 | 0.17451 | ni | hypothetical protein | um06276 | 0.05698 | ni | putative protein |
| 15c00007 | 0.17459 | ni | hypothetical protein | um11061 | 0.05706 | ni | hypothetical protein |
| 6d00040 | 0.17459 | ni | DHHC-type Zn-finger proteins | um05382 | 0.05707 | ni | conserved hypothetical protein |
| 7c00112 | 0.17461 | ni | WD40 repeat protein | um10474.2 | 0.05716 | ni | conserved hypothetical protein |
| 13c00038 | 0.17463 | ni | hypothetical protein | um01728.2 | 0.05717 | ni | conserved hypothetical protein |
| 9d00142 | 0.1748 | ni | hypothetical protein | um00597 | 0.05719 | ni | ni |
| 10d00083 | 0.17484 | ni | putative cargo transport protein ERV29 | um11499 | 0.05731 | K02980 | probable RPS29B - ribosomal protein S29 |
| 20c00024 | 0.17492 | ni | hypothetical protein | um05031 | 0.05734 | K02865 | probable ribosomal protein L10a |
| 16d00018 | 0.17496 | K03626 | transcription factor containing NAC and TS-N domains | um12113 | 0.0574 | ni | conserved hypothetical protein |
| 26d00076 | 0.17514 | K12195 | uncharacterized conserved protein | um04097 | 0.05767 | ni | related to polyketide synthase |
| 8c00050 | 0.1752 | ni | putative transcriptional repressor regulating G2/M transition | um03959 | 0.05773 | K09497 | probable t-complex-type molecular chaperone |
| 27d00050 | 0.17526 | K13116 | DEAD-box protein abstrakt | um10527.2 | 0.05773 | K02967 | related to MRP4 - mitochondrial ribosomal protein |
| 7c00138 | 0.17526 | ni | predicted coiled-coil protein | um03750 | 0.05777 | ni | conserved hypothetical Ustilago-specific protein |
| 22d00150 | 0.17534 | ni | cytochrome b5 | um10320.2 | 0.05779 | K14790 | related to CWH43 - putative sensor |
| 13c00075 | 0.1757 | ni | carbon-nitrogen hydrolase | um00473 | 0.058 | ni | conserved hypothetical protein |
| 9d00140 | 0.17581 | ni | DNA/RNA helicase MER3/SLH1 | um00811 | 0.0581 | ni | related to 50S ribosomal protein L1 |
| 9d00121 | 0.17583 | K14548 | WD40 repeat protein | um11629 | 0.05813 | ni | conserved hypothetical protein |
| 24c00048 | 0.17584 | ni | upstream transcription factor 2/L-myc-2 protein | um00302 | 0.05834 | K03434 | related to N-acetylglucosaminyl-phosphatidylinositol de-N-acetylase |
| 22c00095 | 0.17586 | ni | hypothetical protein | um02727 | 0.05847 | ni | probable lysozyme |
| 22c00042 | 0.17603 | ni | protein kinase | um06129 | 0.05854 | K13025 | probable translation initiation factor eIF-4A |
| 8d00100 | 0.17607 | K10754 | replication factor C, subunit RFC1 | um10011 | 0.05864 | ni | conserved hypothetical protein |
| 6c00017 | 0.1764 | ni | hypothetical protein | um03065 | 0.05873 | ni | putative protein |
| 22c00102 | 0.17687 | K02885 | 60s ribosomal protein L19 | um00047 | 0.05883 | ni | ni |
| 18c00073 | 0.1769 | ni | hypothetical protein | um04192 | 0.05896 | K03265 | probable eukaryotic translational release factor 1 |
| 9c00047 | 0.17702 | K10625 | N-end rule pathway, recognition component UBR1 | um00698.2 | 0.05906 | ni | putative protein |
| 7d00345 | 0.17703 | ni | hypothetical protein | um11549 | 0.05906 | K03644 | related to LIP5 - lipoic acid synthase |
| 22c00240 | 0.17707 | ni | hypothetical protein | um00340 | 0.05912 | K08492 | related to syntaxin 18 |
| 18d00059 | 0.1771 | K14168 | uncharacterized conserved protein with similarity to predicted ATI | um05465 | 0.05913 | K02263 | probable cytochrome-c oxidase chain V precursor |
| 2c00083 | 0.17715 | ni | hypothetical protein | um11600 | 0.05926 | K01784 | related to GAL10 - UDP-glucose 4-epimerase |
| 1c00045 | 0.17728 | K01495 | GTP cyclohydrolase I | um03460 | 0.05932 | K16833 | conserved hypothetical protein |
| 3c00074 | 0.17751 | ni | hypothetical protein | um05280 | 0.05942 | ni | conserved hypothetical protein |
| 8c00064 | 0.17753 | ni | haloacid dehalogenase-like hydrolase | um04262 | 0.05968 | K00767 | probable BNA6 - nicotinate-nucleotide diphosphorylase |
| 19c00102 | 0.17778 | ni | uncharacterized conserved protein | um15002.2 | 0.05988 | K00021 | probable 3-hydroxy-3-methylglutaryl-coenzyme A reductase |
| 7c00167 | 0.17808 | ni | uncharacterized conserved protein | um04525 | 0.05992 | ni | conserved hypothetical protein |
| 7c00176 | 0.17815 | K03514 | DNA polymerase sigma | um03611 | 0.05996 | ni | related to LAG1 - longevity-assurance protein |

| | | | | | | | |
|---|---|---|---|---|---|---|---|
| 20c00044 | 0.17825 | ni | mitochondrial inheritance and actin cytoskeleton organization prot | um11577 | 0.06078 | K03254 | probable eukaryotic translation initiation factor eIF-3 |
| 12d00112 | 0.17826 | ni | vesicle coat complex AP-1/AP-2/AP-4, beta subunit | um01144 | 0.0608 | K03538 | related to POP4 - protein involved in processing of tRNAs and rRNAs |
| 7d00218 | 0.17844 | ni | glycosyl transferase | um00227 | 0.06095 | K01533 | probable CCC2 - P-type ATPase |
| 26d00087 | 0.17845 | ni | predicted membrane protein | um02209 | 0.06113 | ni | putative protein |
| 13c00100 | 0.17876 | ni | hypothetical protein | um11057 | 0.06128 | ni | related to OPT1 - High-affinity glutathione transporter |
| 4d00018 | 0.17895 | ni | hypothetical protein | um11171 | 0.06142 | ni | related to sugar transport protein |
| 9c00125 | 0.17916 | ni | hypothetical protein | um10852 | 0.06146 | K10259 | related to MET30 - involved in regulation of sulfur assimilation genes and cell cycle progression |
| 22d00068 | 0.17947 | ni | hypothetical protein | um10913 | 0.06176 | ni | conserved hypothetical protein |
| 5c00058 | 0.1796 | ni | hypothetical protein | um10696 | 0.06182 | ni | related to AHA1 - stress-regulated cochaperone |
| 13d00100 | 0.17978 | ni | hypothetical protein | um02998 | 0.06189 | ni | conserved hypothetical protein |
| 9d00402 | 0.17998 | K13721 | puromycin-sensitive aminopeptidase and related aminopeptidases | um03704 | 0.0619 | ni | conserved hypothetical protein |
| 22d00005 | 0.17999 | ni | permease of the major facilitator superfamily | um10336 | 0.06191 | ni | conserved hypothetical protein |
| 12c00116 | 0.18001 | K04564 | manganese superoxide dismutase | um10732 | 0.06205 | ni | related to dimeric dihydrodiol dehydrogenase |
| 7d00235 | 0.18004 | ni | hypothetical protein | um00003 | 0.06239 | ni | conserved hypothetical protein |
| 25d00066 | 0.18018 | ni | hypothetical protein | um12097 | 0.06254 | ni | conserved hypothetical protein |
| 8c00090 | 0.18022 | ni | hypothetical protein | um12242.2 | 0.06261 | K03321 | conserved hypothetical protein |
| 14d00009 | 0.18036 | ni | hypothetical protein | um10103 | 0.06303 | K08596 | conserved hypothetical protein |
| 9d00086 | 0.18037 | K17262 | alpha-tubulin folding cofactor B | um03443 | 0.0631 | K10688 | related to ubiquitin-conjugating enzyme |
| 20c00043 | 0.18042 | ni | hypothetical protein | um00148 | 0.06314 | ni | hypothetical protein |
| 19c00038 | 0.18057 | ni | hypothetical protein | um04292 | 0.06318 | ni | conserved hypothetical protein |
| 20c00078 | 0.18057 | K14376 | poly(a) polymerase and related nucleotidyltransferases | um12074 | 0.06326 | K06210 | probable NMA2 - nicotinate-nucleotide adenylyltransferase |
| 10d00021 | 0.18069 | K01551 | putative arsenite-translocating ATPase | um11462 | 0.06356 | ni | putative protein |
| 9c00408 | 0.18072 | ni | hypothetical protein | um11340 | 0.06366 | K04555 | probable QRI8 - E2 ubiquitin-conjugating enzyme |
| 14d00114 | 0.18084 | ni | hypothetical protein | um02412 | 0.06368 | K13195 | related to glycine-rich RNA-binding protein |
| 10c00055 | 0.18094 | K00814 | alanine aminotransferase | um00461 | 0.06371 | ni | conserved hypothetical protein |
| 22d00098 | 0.18101 | K14006 | vesicle coat complex COPII, subunit SEC23 | um02478 | 0.06388 | K13192 | conserved hypothetical protein |
| 16c00066 | 0.18109 | ni | hypothetical protein | um12100 | 0.06402 | ni | putative protein |
| 13d00090 | 0.18117 | K01951 | GMP synthase | um04139 | 0.06409 | ni | conserved hypothetical protein |
| 7d00334 | 0.18119 | ni | putative signal transduction protein involved in RNA splicing | um10380 | 0.06412 | ni | conserved hypothetical protein |
| 6d00026 | 0.18126 | K00100 | aldo/keto reductase family proteins | um05475 | 0.06427 | ni | conserved hypothetical protein |
| 26c00032 | 0.18134 | ni | hypothetical protein | um05915 | 0.06428 | K07008 | conserved hypothetical protein |
| 7d00341 | 0.18141 | ni | hypothetical protein | um02028 | 0.06434 | ni | related to acyl-coenzyme A oxidase 1 |
| 13c00093 | 0.18165 | ni | hypothetical protein | um05939 | 0.06451 | ni | conserved hypothetical protein |
| 16c00034 | 0.1817 | K15153 | transcriptional regulator SOH1 | um02286 | 0.06462 | ni | conserved hypothetical protein |
| 16c00059 | 0.18189 | K07950 | predicted methyltransferase | um05177 | 0.06468 | K17086 | related to endosomal protein EMP70 precursor |
| 26c00030 | 0.18194 | ni | hypothetical protein | um05185 | 0.06472 | ni | conserved hypothetical protein |
| 15d00059 | 0.18194 | ni | predicted mechanosensitive ion channel | um01073 | 0.06475 | ni | putative protein |
| 7c00319 | 0.18232 | K00549 | methionine synthase II | um00113 | 0.06478 | ni | related to transcriptional activator acu-15 |
| 10d00086 | 0.18243 | K01230 | mannosyl-oligosaccharide alpha-1,2-mannosidase | um04743 | 0.06481 | K01514 | related to PPX1 - exopolyphosphatase |
| 9c00264 | 0.18261 | K13280 | signal peptidase I | um00127 | 0.06495 | ni | putative protein |
| 14d00107 | 0.18324 | ni | hypothetical protein | um03770 | 0.06512 | ni | conserved hypothetical protein |
| 9d00237 | 0.18327 | ni | membrane coat complex Retromer, subunit VPS5/SNX1 | um05070 | 0.06544 | ni | conserved hypothetical protein |
| 8c00046 | 0.18338 | ni | chromatin remodeling protein | um10028 | 0.06547 | ni | conserved hypothetical protein |
| 7c00143 | 0.18384 | K14536 | translation elongation factor 2 | um11874 | 0.06568 | ni | conserved hypothetical Ustilago-specific protein |
| 20c00061 | 0.18391 | K12158 | ubiquitin-like protein | um05496 | 0.06574 | ni | related to cystathionine beta-lyase |
| 19c00060 | 0.18393 | ni | hypothetical protein | um01221 | 0.06591 | K07374 | alpha tubulin |
| 9d00379 | 0.18394 | K12832 | uncharacterized conserved protein | um02972 | 0.06593 | ni | putative protein |
| 24c00073 | 0.18397 | ni | hypothetical protein | um06034 | 0.066 | K01265 | probable methionyl aminopeptidase |
| 11d00053 | 0.18403 | K04711 | alkaline ceramidase | um05612 | 0.0663 | ni | related to proline transport helper PTH1 |
| 12d00070 | 0.18407 | K02952 | ribosomal protein S18 | um04667 | 0.06646 | ni | conserved hypothetical protein |
| 26d00043 | 0.18408 | ni | transporter, ABC superfamily | um00354 | 0.06659 | ni | related to Sentrin-specific protease 1 |
| 16c00039 | 0.18414 | ni | hypothetical protein | um05608 | 0.06687 | K11095 | related to U1 small nuclear ribonucleoprotein C |
| 5d00020 | 0.18424 | K07936 | GTPase Ran/TC4/GSP1 | um01220.2 | 0.06696 | ni | related to SSD1 protein |
| 7c00315 | 0.18431 | K13122 | predicted actin-bundling protein | um11761 | 0.06704 | K03679 | related to RRP4 - 3 |
| 15c00090 | 0.18434 | ni | hypothetical protein | um15066 | 0.06707 | ni | conserved hypothetical protein |
| 16c00037 | 0.18448 | ni | hypothetical protein | um05960 | 0.06707 | ni | conserved hypothetical protein |
| 7c00168 | 0.18462 | K03020 | DNA-directed RNA polymerase, subunit L | um06506 | 0.0671 | ni | conserved hypothetical protein |
| 11d00071 | 0.18489 | ni | hypothetical protein | um10826 | 0.06716 | ni | conserved hypothetical protein |
| 12d00084 | 0.18491 | ni | hypothetical protein | um11237 | 0.06716 | ni | putative protein |
| 5c00147 | 0.18513 | ni | predicted hydrolase related to dienelactone hydrolase | um10033 | 0.06717 | K15172 | related to SPT5 - transcription elongation protein |
| 9c00286 | 0.18523 | K00766 | anthranilate phosphoribosyltransferase | um11578 | 0.0673 | K02731 | probable PRE6 - 20S proteasome subunit |
| 25d00074 | 0.18529 | K07401 | hypothetical protein | um02715 | 0.06732 | K00632 | probable POT1 - acetyl-CoA C-acyltransferase |
| 8c00062 | 0.18531 | K11501 | hypothetical protein | um10237 | 0.06753 | K02214 | related to CDC7 - protein kinase |
| 7c00313 | 0.18567 | K03132 | transcription initiation factor TFIID, subunit TAF7 | um04795 | 0.06754 | K14301 | nucleoporin |
| 1c00011 | 0.18571 | K12845 | 60S ribosomal protein 15.5kD/SNU13 | um03469 | 0.06757 | K05288 | related to GPI13 - protein involved in glycosylphosphatidylinositol biosynthesis |
| 14c00001 | 0.18586 | ni | hypothetical protein | um05293 | 0.06779 | K07151 | probable oligosaccharyltransferase |
| 26c00040 | 0.18619 | ni | DNA-binding proteins Bright/BRCAA1/RBP1 | um05822 | 0.0679 | ni | conserved hypothetical protein |
| 15d00092 | 0.18622 | ni | hypothetical protein | um05231 | 0.06806 | ni | related to YIP3 protein - proposed to be involved in ER to Golgi transport |
| 5d00055 | 0.18627 | ni | uncharacterized conserved protein | um03801 | 0.06813 | ni | ni |
| 1c00031 | 0.18646 | ni | adaptor protein Enigma and related PDZ-LIM proteins | um12298 | 0.06831 | ni | conserved hypothetical protein |
| 24c00040 | 0.18648 | ni | replication factor C, subunit RFC1 | um00291 | 0.06836 | ni | conserved hypothetical protein |
| 18d00074 | 0.18678 | ni | hypothetical protein | um03779 | 0.06841 | ni | related to galactinol synthase |
| 6d00080 | 0.18683 | ni | acyl-coa thioesterase | um00872 | 0.06846 | K00928 | probable HOM3 - L-aspartate 4-P-transferase |
| 9c00093 | 0.18695 | ni | carboxylesterase and related proteins | um10406 | 0.06847 | ni | conserved hypothetical protein |
| 9c00377 | 0.18702 | K14798 | uncharacterized conserved protein | um02732 | 0.06888 | ni | conserved hypothetical protein |
| 9d00155 | 0.18704 | ni | fry-like conserved proteins | um11428 | 0.06901 | K14326 | probable NAM7 - nonsense-mediated mRNA decay protein |
| 19c00064 | 0.1872 | ni | uncharacterized conserved protein | um10062 | 0.0691 | ni | related to monooxygenase |
| 8d00020 | 0.18726 | K07910 | GTPase Rab18 | um03648 | 0.06911 | ni | ni |
| 7d00336 | 0.18732 | K05931 | protein arginine N-methyltransferase CARM1 | um12157 | 0.06915 | ni | conserved hypothetical protein |
| 12d00120 | 0.18747 | K10583 | ubiquitin-protein ligase | um05879 | 0.06928 | ni | conserved hypothetical protein |
| 15d00046 | 0.18756 | K09584 | thioredoxin/protein disulfide isomerase | um01824 | 0.06942 | K15918 | conserved hypothetical protein |
| 20c00067 | 0.18768 | ni | hypothetical protein | um00929 | 0.06964 | K13122 | conserved hypothetical protein |
| 19d00030 | 0.18828 | ni | hypothetical protein | um02750 | 0.06965 | K01092 | related to quinic acid utilisation protein QUTG |
| 22c00033 | 0.18844 | ni | hypothetical protein | um10174 | 0.06972 | ni | conserved hypothetical protein |
| 13c00078 | 0.18852 | K06682 | ras GTPases | um00272 | 0.06988 | ni | conserved hypothetical protein |
| 14c00025 | 0.18887 | K09537 | hypothetical protein | um11808 | 0.06996 | K04718 | related to LCB5 - sphingolipid long chain base kinase |
| 22d00080 | 0.1889 | ni | G-protein beta subunit-like protein GNB1L | um06411 | 0.07002 | K01876 | related to aspartate--tRNA ligase |
| 24c00035 | 0.18896 | ni | mannosyltransferase | um05351 | 0.07029 | ni | conserved hypothetical protein |
| 12d00071 | 0.18909 | K02738 | 20S proteasome, regulatory subunit beta type PSMB6/PSMB9/PI | um06390 | 0.07079 | ni | hypothetical protein |
| 7c00265 | 0.1891 | ni | serine/threonine kinase TIP30/CC3 | um04179 | 0.07099 | K02734 | probable multicatalytic endopeptidase complex chain PRE1 |
| 12d00087 | 0.18911 | K14293 | karyopherin (importin) beta 1 | um02845.2 | 0.07099 | ni | conserved hypothetical protein |
| 7c00063 | 0.1892 | ni | hypothetical protein | um12259 | 0.07109 | K15111 | related to PET8 - Protein of the mitochondrial carrier family |
| 22d00073 | 0.18921 | ni | predicted membrane protein | um00480 | 0.07109 | ni | transmembrane mucin involved in surface sensing via MAP-kinase cascade |
| 16d00028 | 0.18922 | ni | hypothetical protein | um10907 | 0.07111 | K10704 | probable MMS2 - part of the error-free postreplication repair pathway |
| 9d00196 | 0.1893 | K02871 | mitochondrial/chloroplast ribosomal protein L13 | um00928 | 0.07115 | ni | putative protein |
| 4c00024 | 0.18935 | ni | hypothetical protein | um01173 | 0.07116 | K15456 | related to KTI12 - Elongator associated protein |
| 12c00126 | 0.18935 | K00382 | dihydrolipoamide dehydrogenase | um05602 | 0.07119 | ni | probable sugar transporter |
| 10c00080 | 0.18946 | ni | cytosine deaminase FCY1 and related enzymes | um01589 | 0.07124 | ni | ni |
| 22d00074 | 0.1896 | ni | hypothetical protein | um03613 | 0.07141 | ni | putative protein |
| 20c00035 | 0.18976 | ni | hypothetical protein | um10481 | 0.07151 | K05687 | conserved hypothetical protein |
| 4c00011 | 0.18989 | ni | hypothetical protein | um00328 | 0.07154 | ni | putative protein |
| 19d00105 | 0.18995 | K10735 | predicted alpha-helical protein | um05134 | 0.07164 | ni | conserved hypothetical protein |
| 19c00132 | 0.19009 | ni | hypothetical protein | um12045 | 0.07164 | ni | related to alpha glucosidase II beta subunit |
| 9d00216 | 0.19033 | K05349 | hypothetical protein | um00948 | 0.07169 | K11324 | related to SWC4 - component of the Swr1p complex |
| 24c00039 | 0.19034 | K15161 | CDK8 kinase-activating protein cyclin C | um02314 | 0.07175 | ni | putative protein |
| 8d00095 | 0.19039 | ni | hypothetical protein | um03590 | 0.07183 | ni | putative protein |
| 9d00279 | 0.19059 | ni | hypothetical protein | um03076 | 0.07186 | ni | conserved hypothetical protein |
| 19d00065 | 0.1906 | ni | hypothetical protein | um01190 | 0.07188 | ni | conserved hypothetical protein |
| 7c00222 | 0.19064 | ni | hypothetical protein | um02533 | 0.07198 | ni | hypothetical protein |
| 22d00267 | 0.1907 | K00232 | acyl-coa oxidase | um11827 | 0.072 | K02991 | probable RPS6B - 40s ribosomal protein S6 |
| 26c00088 | 0.19076 | ni | hypothetical protein | um05143 | 0.07225 | K11279 | probable nucleosome assembly protein I |
| 27c00072 | 0.19086 | K11262 | acetyl-coa carboxylase | um04151 | 0.07231 | K10844 | probable RAD3 - DNA helicase |
| 25d00013 | 0.19091 | ni | serine/threonine protein kinase | um11098 | 0.07251 | K01915 | probable GLN1 - glutamate--ammonia ligase |
| 1d00006 | 0.19104 | K08740 | mismatch repair ATPase MSH4 | um00785 | 0.07253 | ni | conserved hypothetical protein |
| 15c00005 | 0.19116 | ni | glutaredoxin and related proteins | um00090 | 0.07261 | ni | conserved hypothetical protein |
| 27d00018 | 0.19131 | ni | hypothetical protein | um10451 | 0.07274 | ni | conserved hypothetical protein |
| 19c00035 | 0.19136 | ni | WD40 repeat protein | um11221 | 0.07307 | ni | hypothetical protein |
| 22c00097 | 0.19137 | ni | hypothetical protein | um04682 | 0.07313 | ni | conserved hypothetical protein |
| 7d00338 | 0.19144 | ni | hypothetical protein | um11956 | 0.07322 | ni | conserved hypothetical protein |
| 21c00004 | 0.19159 | ni | hypothetical protein | um04017 | 0.07324 | ni | conserved hypothetical protein |
| 10c00016 | 0.19161 | ni | hypothetical protein | um04189 | 0.07345 | ni | related to Cytochrome P450 |
| 12d00110 | 0.19175 | ni | hypothetical protein | um04160 | 0.07355 | ni | Dkh6 |
| 3d00088 | 0.1919 | ni | hypothetical protein | um05327 | 0.07382 | ni | hypothetical protein |
| 10d00046 | 0.19191 | ni | hypothetical protein | um04404 | 0.07382 | ni | hypothetical protein |
| 10d00054 | 0.19196 | ni | hypothetical protein | um11235 | 0.07394 | ni | related to Protein esc1 |
| 22c00317 | 0.19205 | ni | hypothetical protein | um01389 | 0.07402 | ni | hypothetical protein |
| 16d00079 | 0.19211 | K03952 | NADH:ubiquinone oxidoreductase, NDUFA8/PGIV/19 kDa subur | um05311 | 0.07403 | ni | conserved hypothetical Ustilago-specific protein |
| 22d00100 | 0.19212 | K08504 | V-SNARE | um01919 | 0.07406 | K01489 | related to CDD1 - cytidine deaminase |
| 12c00111 | 0.19223 | ni | uncharacterized conserved protein | um11501 | 0.07428 | ni | probable MGM1 - Mitochondrial GTPase related to dynamin |
| 7c00038 | 0.19225 | K03320 | ammonia permease | um10675 | 0.07442 | ni | conserved hypothetical protein |
| 6d00103 | 0.19228 | K07952 | GTP-binding ADP-ribosylation factor-like protein yARL3 | um05607 | 0.07454 | K12197 | probable DID2 - class E vacuolar-protein sorting and endocytosis factor |
| 9d00368 | 0.19229 | ni | hypothetical protein | um11247 | 0.07457 | K13108 | related to Smad nuclear interacting protein 1 |
| 22c00087 | 0.19243 | ni | uncharacterized conserved protein | um11293 | 0.07474 | K12761 | related to SNF1 - carbon catabolite derepressing ser |
| 6d00041 | 0.19254 | K01078 | hypothetical protein | um05883 | 0.07496 | ni | conserved hypothetical protein |
| 10c00105 | 0.19256 | ni | hypothetical protein | um02594.2 | 0.07499 | ni | putative protein |
| 12d00034 | 0.19257 | K15030 | uncharacterized conserved protein | um11679 | 0.07524 | ni | conserved hypothetical protein |
| 25c00072 | 0.19266 | ni | hypothetical protein | um04962 | 0.07548 | K00328 | related to ARG82 - dual-specificity inositol polyphosphate kinase required for regulation of phosphate- and nitrogen-responsive genes |
| 8c00124 | 0.19269 | ni | amino acid transporters | um03462 | 0.07572 | ni | hypothetical protein |
| 25c00018 | 0.19274 | ni | predicted GTPase activator protein | um12083 | 0.07595 | ni | related to COX9 - Cytochrome-c oxidase chain VIIA |
| 10d00055 | 0.19283 | K12858 | U5 snRNP-like RNA helicase subunit | um11401 | 0.07598 | K14288 | related to tRNA Exportin |
| 15c00012 | 0.19293 | ni | hypothetical protein | um05179 | 0.07601 | K01597 | probable MVD1 - mevalonate pyrophosphate decarboxylase |
| 7c00031 | 0.19302 | ni | hypothetical protein | um01101 | 0.07628 | K04797 | probable prefoldin subunit 5 |
| 9c00046 | 0.1932 | ni | putative translation initiation inhibitor UK114/IBM1 | um06370 | 0.07636 | ni | conserved hypothetical protein |
| 7c00065 | 0.19324 | ni | hypothetical protein | um04086 | 0.0764 | ni | conserved hypothetical Ustilago-specific protein |
| 14d00088 | 0.19334 | K00698 | chitin synthase/hyaluronan synthase | um04603 | 0.07648 | K11673 | related to ARP8 - Actin-related protein |
| 9d00014 | 0.19353 | ni | hypothetical protein | um03861 | 0.07666 | ni | probable YBT1 - Vacuolar |
| 24c00054 | 0.19368 | K02858 | bifunctional GTP cyclohydrolase II | um00378 | 0.07683 | ni | hypothetical protein |
| 14d00022 | 0.1942 | K15859 | heat shock transcription factor | um10229 | 0.07685 | ni | probable BET3 - involved in targeting and fusion of ER to golgi transport vesicles |
| 24c00045 | 0.19436 | ni | hypothetical protein | um03932 | 0.0769 | ni | hypothetical protein |
| 9d00004 | 0.19437 | ni | hydroxysteroid 17-beta dehydrogenase 11 | um10087.2 | 0.07695 | ni | putative protein |
| 20c00073 | 0.19447 | ni | hypothetical protein | um05714 | 0.07697 | K14824 | related to ERB1 - Protein required for maturation of the 25S and 5 |
| 6c00098 | 0.19457 | ni | enoyl-coa hydratase | um00898 | 0.07719 | K08956 | probable AFG3 - protease of the SEC18 |
| 13c00097 | 0.19461 | ni | aldehyde dehydrogenase | um11799 | 0.07729 | ni | conserved hypothetical protein |
| 15d00004 | 0.19464 | ni | hypothetical protein | um12227 | 0.07731 | K11572 | related to DASH complex subunit spc19 |
| 8c00094 | 0.1947 | ni | predicted alpha/beta hydrolase | um10214 | 0.07733 | ni | putative protein |
| 9c00409 | 0.19472 | K17255 | RAB proteins geranylgeranyltransferase component A | um03156 | 0.0775 | K11246 | protein involved in surface sensing via MAP-kinase cascade |

| | | | | | | | |
|---|---|---|---|---|---|---|---|
| 9d00126 | 0.19486 | ni | hypothetical protein | um05852 | 0.07755 | ni | hypothetical protein |
| 9d00336 | 0.19501 | ni | predicted transporter | um04376 | 0.07778 | ni | conserved hypothetical protein |
| 12c00099 | 0.19513 | ni | hypothetical protein | um01879 | 0.07784 | ni | conserved hypothetical protein |
| 9d00149 | 0.19532 | ni | hypothetical protein | um01510 | 0.07802 | ni | hypothetical protein |
| 1d00009 | 0.19538 | ni | M-phase inducer phosphatase | um02360 | 0.07808 | K02141 | conserved hypothetical protein |
| 20c00050 | 0.19538 | ni | hypothetical protein | um10030 | 0.07813 | ni | putative protein |
| 20d00036 | 0.1957 | K09495 | chaperonin complex component, TCP-1 gamma subunit | um11023 | 0.07818 | ni | conserved hypothetical protein |
| 26d00017 | 0.19598 | K10259 | cdc4 and related F-box and WD-40 proteins | um01023 | 0.07846 | K00602 | probable ADE17 - 5-aminoimidazole-4-carboxamide ribotide transformylase |
| 19c00078 | 0.19622 | ni | oxysterol-binding protein | um03989.2 | 0.07847 | K03615 | putative protein |
| 22d00099 | 0.19643 | ni | O-linked N-acetylglucosamine transferase OGT | um02682 | 0.07848 | K16302 | conserved hypothetical protein |
| 14d00091 | 0.19683 | ni | intermediate filament-like protein, sorting nexins, and related prote | um10344 | 0.07849 | ni | conserved hypothetical protein |
| 26c00028 | 0.19685 | K01823 | isopentenyl pyrophosphate, dimethylallyl pyrophosphate isomeras | um11735 | 0.07865 | ni | related to FES1 - Hsp70 nucleotide exchange factor |
| 6d00106 | 0.19689 | ni | hypothetical protein | um10081 | 0.07889 | ni | conserved hypothetical protein |
| 2c00070 | 0.197 | ni | hypothetical protein | um10331 | 0.07889 | K14171 | related to peroxisomal membrane protein |
| 7d00308 | 0.19704 | ni | hypothetical protein | um12296 | 0.07889 | ni | conserved hypothetical protein |
| 19c00134 | 0.19705 | K00573 | protein-l-isoaspartate(d-aspartate) O-methyltransferase | um02993 | 0.07897 | ni | ni |
| 13c00107 | 0.19708 | K04554 | ubiquitin-protein ligase | um05640 | 0.07898 | ni | probable RET3 - coatomer complex zeta chain |
| 5d00049 | 0.1971 | ni | M13 family peptidase | um06218 | 0.0791 | ni | conserved hypothetical protein |
| 8d00089 | 0.19735 | ni | hypothetical protein | um02878.2 | 0.07915 | ni | conserved hypothetical protein |
| 2c00014 | 0.19736 | K01866 | tyrosyl-trna synthetase | um12305 | 0.07917 | ni | probable GTP-binding protein 1 |
| 5d00058 | 0.19768 | K07095 | membrane coat complex Retromer, subunit VPS29/PEP11 | um01470 | 0.0794 | K14403 | related to YSH1 - component of pre-mRNA polyadenylation factor PF I |
| 7d00051 | 0.19777 | ni | signal transduction serine/threonine kinase | um11728 | 0.07946 | ni | related to Yippee protein |
| 10c00054 | 0.19794 | ni | actin-related protein - Arp6p | um10025 | 0.07964 | ni | conserved hypothetical protein |
| 9d00178 | 0.19801 | K11872 | ubiquitin-specific protease | um02981 | 0.07983 | ni | conserved hypothetical protein |
| 16d00039 | 0.19807 | ni | hypothetical protein | um12084 | 0.07986 | ni | related to Molybdopterin synthase small subunit |
| 18c00076 | 0.19823 | ni | mitochondrial carnitine-acylcarnitine carrier protein | um03164 | 0.0799 | K16794 | probable platelet-activating factor acetylhydrolase ib alpha subunit |
| 15d00005 | 0.19834 | K14574 | predicted exosome subunit | um11205 | 0.08002 | K07893 | probable YPT6 - GTP-binding protein of the rab family |
| 9c00191 | 0.19838 | ni | hypothetical protein | um03379 | 0.08003 | ni | conserved hypothetical protein |
| 5d00076 | 0.19846 | ni | RNA-binding protein RBM5 and related proteins | um05270 | 0.08028 | ni | conserved hypothetical protein |
| 22d00173 | 0.19865 | K00547 | homocysteine S-methyltransferase | um06019 | 0.08037 | K08794 | probable CMK1 - Ca2 |
| 9c00091 | 0.19868 | K03352 | hypothetical protein | um02847 | 0.08043 | K01128 | related to ISC1 - Inositol phosphoSphingolipid phospholipase |
| 9c00274 | 0.19885 | ni | hypothetical protein | um00049 | 0.08116 | ni | conserved hypothetical protein |
| 14c00115 | 0.19887 | ni | hypothetical protein | um11454 | 0.08125 | ni | conserved hypothetical protein |
| 24c00066 | 0.1991 | ni | uncharacterized conserved protein | um11726 | 0.08126 | ni | conserved hypothetical protein |
| 18d00077 | 0.19924 | K11360 | hypothetical protein | um03859 | 0.08129 | ni | hypothetical protein |
| 7d00027 | 0.19948 | ni | hypothetical protein | um05619 | 0.08139 | ni | conserved hypothetical protein |
| 13d00038 | 0.19955 | K11552 | predicted DNA methylase | um05946 | 0.08141 | ni | ni |
| 1d00013 | 0.19968 | ni | synaptic vesicle transporter SVOP and related transporters | um04877 | 0.08146 | ni | hypothetical protein |
| 4d00012 | 0.19981 | K12948 | signal peptidase complex subunit | um12132 | 0.08153 | ni | conserved hypothetical protein |
| 5d00115 | 0.19983 | ni | very long-chain acyl-CoA synthetase | um11988 | 0.08157 | ni | putative protein |
| 13d00109 | 0.19984 | ni | hypothetical protein | um11922 | 0.08201 | ni | related to Chitin deacetylase precursor |
| 7c00165 | 0.19989 | K02607 | hypothetical protein | um10023 | 0.08205 | K07243 | high-affinity iron permease |
| 7c00232 | 0.19995 | K02959 | mitochondrial/chloroplast ribosomal protein S16 | um01670 | 0.08211 | K15436 | related to MTR10 - involved in nuclear protein import |
| 9d00208 | 0.20005 | ni | hypothetical protein | um10198 | 0.08216 | K01090 | probable PPG1 - phosphoprotein phosphatase PPG catalytic chain |
| 7d00196 | 0.2002 | ni | D-aspartate oxidase | um00996.2 | 0.08236 | ni | conserved hypothetical protein |
| 7d00302 | 0.20028 | K02896 | 60s ribosomal protein L24 | um05893 | 0.08262 | ni | conserved hypothetical protein |
| 7d00348 | 0.20031 | ni | beta-glucuronidase GUSB | um11190 | 0.08263 | ni | putative protein |
| 13c00060 | 0.20033 | K11204 | gamma-glutamylcysteine synthetase | um05976 | 0.08277 | ni | hypothetical protein |
| 10c00073 | 0.20034 | ni | hypothetical protein | um02126 | 0.08289 | ni | conserved hypothetical protein |
| 14c00059 | 0.20034 | K14763 | uncharacterized conserved protein | um15065 | 0.08296 | ni | conserved hypothetical protein |
| 14d00111 | 0.20054 | K01880 | glycyl-trna synthetase and related class II tRNA synthetase | um03140 | 0.08297 | ni | ni |
| 24d00002 | 0.20064 | ni | multidrug resistance-associated protein | um00092 | 0.08318 | ni | related to levodione reductase |
| 26c00093 | 0.20095 | ni | ku70-binding protein | um00503 | 0.08322 | ni | putative protein |
| 5c00051 | 0.20131 | ni | hypothetical protein | um10715 | 0.08343 | K14850 | related to RRP8 - nucleolar protein required for efficient processing of pre-rRNA at site A2 |
| 18c00094 | 0.2015 | K07252 | RNA pseudouridylate synthases | um01695 | 0.08356 | ni | hypothetical protein |
| 6d00057 | 0.20151 | ni | hypothetical protein | um11734 | 0.08411 | K14563 | probable NOP1 - fibrillarin |
| 1c00048 | 0.20156 | K00615 | transketolase | um12316 | 0.08431 | ni | conserved hypothetical protein |
| 8d00040 | 0.20164 | ni | hypothetical protein | um06008 | 0.08441 | K06030 | GTP-binding protein |
| 22d00089 | 0.20166 | ni | nuclear transport receptor RANBP7/RANBP8 | um10127 | 0.08448 | K02889 | probable 60s ribosomal protein L21-A |
| 22c00041 | 0.20177 | K12193 | vacuolar sorting protein VPS24 | um11397 | 0.08452 | ni | putative protein |
| 22d00200 | 0.2018 | K03317 | concentrative Na+-nucleoside cotransporter CNT1/CNT2 | um03669 | 0.08458 | K03885 | probable NADH dehydrogenase |
| 3d00007 | 0.20182 | K09467 | FOG, Zn-finger | um04115 | 0.08458 | K01655 | probable LYS20 - homocitrate synthase |
| 20c00006 | 0.20186 | ni | Ca2+-binding protein | um02490 | 0.08473 | K08158 | probable FLR1 - Putative H  antiporter involved in multidrug resistance |
| 9c00370 | 0.20193 | ni | WD40 protein | um10218 | 0.08522 | ni | hypothetical protein |
| 22d00142 | 0.20199 | ni | WD40 repeat-containing protein L2DTL | um00793 | 0.08533 | ni | conserved hypothetical Ustilago-specific protein |
| 9d00414 | 0.20201 | K01267 | aminopeptidase I zinc metalloprotease | um10652 | 0.08535 | K01493 | related to DCD1 - deoxycytidylate deaminase |
| 22d00143 | 0.2022 | K14811 | ATP-dependent RNA helicase | um03813 | 0.0855 | K14309 | related to NIC96 - nuclear pore protein |
| 9d00234 | 0.20227 | ni | hypothetical protein | um10686 | 0.08555 | ni | conserved hypothetical protein |
| 27c00017 | 0.20235 | K10590 | E3 ubiquitin protein ligase | um11509 | 0.0856 | K14800 | conserved hypothetical protein |
| 7c00326 | 0.20243 | ni | acyl-coa synthetase | um02496 | 0.08566 | K07047 | conserved hypothetical protein |
| 22d00168 | 0.2027 | ni | hypothetical protein | um03037 | 0.08578 | ni | related to BOI1 - BEM1 protein-binding protein |
| 9d00382 | 0.20288 | ni | hypothetical protein | um10968 | 0.08591 | ni | probable VTC4 - Vacuolar Transporter Chaperone |
| 18d00034 | 0.20291 | ni | hypothetical protein | um03602 | 0.08601 | K07910 | probable Ras-related protein Rab-18 |
| 18c00087 | 0.20291 | K00616 | transaldolase | um02955 | 0.08625 | K13199 | related to RAY38 protein |
| 24d00035 | 0.20312 | ni | protein disulfide isomerase | um02275 | 0.0864 | ni | related to PET127 - component of mitochondrial translation system |
| 4c00025 | 0.20316 | K03680 | translation initiation factor 2B, delta subunit | um11027 | 0.08661 | K08517 | probable SEC22 - synaptobrevin |
| 9c00182 | 0.20337 | K06664 | predicted E3 ubiquitin ligase | um15068 | 0.08691 | ni | conserved hypothetical protein |
| 16d00084 | 0.20373 | ni | predicted transporter | um03504 | 0.08704 | ni | related to Purine-nucleoside phosphorylase |
| 14c00092 | 0.20382 | K00226 | dihydroorotate dehydrogenase | um10361 | 0.08713 | K02923 | probable 60S ribosomal protein L38 |
| 5c00152 | 0.20395 | K14310 | uncharacterized conserved protein | um02879 | 0.08719 | ni | putative protein |
| 19c00109 | 0.20401 | ni | hypothetical protein | um11308 | 0.08736 | K06067 | histone deacetylase |
| 9c00039 | 0.20405 | K10134 | p53-mediated apoptosis protein EI24/PIG8 | um10366.2 | 0.08743 | ni | hypothetical protein |
| 1c00003 | 0.20411 | ni | flavonol reductase | um03997 | 0.08757 | K00589 | related to MET1 - siroheme synthase |
| 9c00119 | 0.20448 | K03247 | translation initiation factor 3, subunit h | um11816 | 0.08763 | K16569 | related to gamma-tubulin complex component 2 |
| 22c00096 | 0.20455 | K09539 | molecular chaperone | um01983 | 0.08785 | ni | conserved hypothetical protein |
| 24d00030 | 0.20473 | K13336 | peroxisomal assembly protein PEX3 | um11132 | 0.08788 | K10773 | related to NTG1 - DNA repair protein |
| 8c00047 | 0.20488 | ni | hypothetical protein | um10565 | 0.08802 | ni | hypothetical protein |
| 3d00011 | 0.20493 | ni | hypothetical protein | um01418 | 0.08807 | K14307 | related to NUP49 - nuclear pore protein |
| 22d00220 | 0.20505 | ni | uncharacterized conserved protein | um04399 | 0.08808 | K15100 | probable succinate-fumarate transporter |
| 9d00287 | 0.20513 | K15732 | TFIIF-interacting CTD phosphatases | um04286 | 0.08818 | ni | hypothetical protein |
| 7c00163 | 0.20517 | K00652 | 5-aminolevulinate synthase | um00760 | 0.08824 | K00938 | related to ERG8 - phosphomevalonate kinase |
| 10c00041 | 0.20518 | ni | mRNA cleavage and polyadenylation factor I complex, subunit RN | um01735 | 0.08828 | K07880 | GTP-binding protein |
| 8d00049 | 0.20533 | ni | hypothetical protein | um00604 | 0.08852 | K12795 | related to SGT1 - subunit of SCF ubiquitin ligase complex |
| 7d00058 | 0.20537 | K07008 | glucosamine 6-phosphate synthetases | um06402 | 0.08855 | K02210 | probable DNA replication licensing factor |
| 26c00054 | 0.20554 | K01705 | aconitase/homoaconitase | um11998 | 0.08856 | K14430 | probable PHO91 - similarity to Pho87p and Pho90p |
| 22c00280 | 0.2056 | K01193 | beta-fructofuranosidase | um03603 | 0.08859 | ni | hypothetical protein |
| 9c00128 | 0.20561 | K00931 | gamma-glutamyl kinase | um05139 | 0.0886 | K02981 | probable 40S ribosomal protein S2 |
| 9c00129 | 0.20567 | K10755 | replication factor C, subunit RFC4 | um12190 | 0.08865 | ni | conserved hypothetical protein |
| 3c00036 | 0.20568 | ni | hypothetical protein | um00216 | 0.08881 | K10755 | probable RFC2 - DNA replication factor C |
| 4c00006 | 0.20571 | ni | membrane coat complex Retromer, subunit VPS26 | um11490 | 0.08895 | K11584 | related to B56-delta regulatory subunit of protein phosphatase 2A |
| 8c00078 | 0.20575 | K12180 | COP9 signalosome, subunit CSN7 | um10201 | 0.08898 | ni | hypothetical protein |
| 2c00075 | 0.20585 | ni | hypothetical protein | um11385 | 0.08931 | ni | putative protein |
| 22d00197 | 0.20601 | ni | DHHC-type Zn-finger proteins | um10392 | 0.0895 | ni | conserved hypothetical protein |
| 16d00055 | 0.20609 | ni | multicopper oxidases | um11763 | 0.08991 | ni | related to acetylxylan esterase |
| 27d00075 | 0.20611 | K17338 | HVA22/DP1 gene product-related proteins | um04095 | 0.09058 | ni | related to polyketide synthase |
| 9c00123 | 0.20612 | ni | hypothetical protein | um04918.2 | 0.09077 | ni | conserved hypothetical protein |
| 15c00081 | 0.20643 | ni | predicted tubulin-tyrosine ligase | um03551 | 0.09101 | K00108 | related to Glucose oxidase |
| 12c00079 | 0.20646 | K11098 | small nuclear ribonucleoprotein | um03893 | 0.09114 | K12836 | related to splicing factor U2AF 35 kd subunit |
| 7c00258 | 0.20658 | K01103 | fructose-6-phosphate 2-kinase | um01304 | 0.0912 | K03660 | related to 8-oxoguanine DNA-glycosylase |
| 14d00081 | 0.20666 | K16938 | septin CDC10 and related P-loop GTPases | um06165 | 0.09122 | ni | hypothetical protein |
| 22d00104 | 0.20669 | ni | hypothetical protein | um00714 | 0.09132 | K14015 | probable NPL4 - nuclear protein localization factor and ER translocation component |
| 22d00067 | 0.20671 | ni | predicted ATPase | um02948 | 0.09137 | K11564 | conserved hypothetical protein |
| 26d00009 | 0.20676 | ni | DNA-directed RNA polymerase III subunit | um02326 | 0.09141 | ni | conserved hypothetical protein |
| 6d00132 | 0.20701 | K08139 | predicted transporter | um06293 | 0.09153 | ni | hypothetical protein |
| 19c00069 | 0.20714 | ni | hypothetical protein | um05745 | 0.09154 | K11374 | related to ELP2 - 29 kDa subunit of elongator and elongating RNA polymerase II holoenzyme |
| 4d00006 | 0.20723 | ni | WD40 repeat-containing protein | um05597 | 0.09154 | ni | putative protein |
| 7c00078 | 0.2073 | ni | molecular chaperone | um04211 | 0.09155 | ni | conserved hypothetical protein |
| 9c00104 | 0.20731 | ni | uncharacterized conserved protein | um02493 | 0.09157 | K10908 | related to RPO41 - DNA-directed RNA polymerase |
| 7d00032 | 0.20752 | K14812 | transcriptional effector CCR4-related protein | um06480 | 0.09158 | ni | putative protein |
| 25d00015 | 0.20754 | K04488 | iron binding protein involved in Fe-S cluster formation | um11926 | 0.09159 | ni | conserved hypothetical protein |
| 7c00214 | 0.20763 | K02134 | mitochondrial F1F0-ATP synthase, subunit delta/ATP16 | um00958 | 0.0916 | ni | conserved hypothetical protein |
| 27c00057 | 0.20781 | K14429 | amino acid transporters | um01985 | 0.09163 | ni | hypothetical protein |
| 5c00107 | 0.20784 | ni | hypothetical protein | um05159 | 0.09218 | K12848 | conserved hypothetical protein |
| 6c00068 | 0.2081 | ni | hypothetical protein | um02985 | 0.09249 | ni | conserved hypothetical protein |
| 9c00088 | 0.20823 | K05542 | tRNA-dihydrouridine synthase | um06219 | 0.0925 | K10849 | related to dna excision repair protein ercc-1 |
| 8c00063 | 0.20833 | K17279 | protein involved in membrane traffic | um12317 | 0.09252 | ni | conserved hypothetical protein |
| 9c00343 | 0.20834 | ni | hypothetical protein | um10498 | 0.09258 | ni | putative protein |
| 27c00046 | 0.20856 | ni | hypothetical protein | um00235 | 0.09291 | K01210 | probable EXG1 - exo-beta-1 |
| 5d00001 | 0.20868 | ni | hypothetical protein | um12244 | 0.09294 | K11086 | related to small nuclear ribonucleoprotein associated protein b |
| 25c00028 | 0.20871 | ni | hypothetical protein | um05747 | 0.09299 | K14005 | related to SEC31 - component of the COPII coat of ER-golgi vesicles |
| 22d00283 | 0.20877 | ni | hypothetical protein | um01634 | 0.09359 | K02885 | probable RPL19B - 60S large subunit ribosomal protein L19 |
| 6c00002 | 0.209 | ni | hypothetical protein | um06460 | 0.09365 | ni | probable fatty acid synthase |
| 5d00072 | 0.20908 | K14558 | WD40-repeat-containing subunit of the 18S rRNA processing co | um02786 | 0.09386 | ni | hypothetical protein |
| 15d00041 | 0.20919 | ni | multicopper oxidases | um04413 | 0.0939 | ni | related to transport protein USO1 |
| 24c00030 | 0.20962 | ni | hypothetical protein | um04385 | 0.09391 | ni | ni |
| 9d00334 | 0.20971 | K01624 | fructose 1,6-bisphosphate aldolase | um10136 | 0.094 | ni | related to vacuolar protein-sorting protein VPS35 |
| 18d00073 | 0.20975 | K02734 | 20S proteasome, regulatory subunit beta type PSMB2/PRE1 | um04416 | 0.09407 | K09272 | probable POB3 - protein that binds to DNA polymerase I |
| 22c00053 | 0.20978 | K12856 | U5 snRNP spliceosome subunit | um03096 | 0.09433 | ni | probable CSE1 - Nuclear envelope protein that mediates the nuclear export of importin alpha |
| 19c00014 | 0.20994 | ni | hypothetical protein | um03182 | 0.09439 | K00559 | probable delta |
| 7d00048 | 0.20995 | ni | E3 ubiquitin ligase interacting with arginine methyltransferase | um10205 | 0.09441 | K16365 | conserved hypothetical protein |
| 22c00038 | 0.21002 | ni | hypothetical protein | um02524 | 0.09466 | K14310 | related to nucleoporin |
| 24d00046 | 0.2101 | ni | imidazoleglycerol-phosphate synthase subunit H-like | um01207 | 0.09474 | K09518 | related to HLJ1 - Co-chaperone for Hsp40p |
| 14c00060 | 0.21012 | ni | hypothetical protein | um00771 | 0.0952 | ni | hypothetical protein |
| 16c00092 | 0.21018 | ni | vacuolar assembly/sorting protein DID2 | um03115 | 0.09521 | ni | related to Sge1 - drug resistance protein |
| 22c00178 | 0.21024 | K01227 | predicted glycosylhydrolase | um01684 | 0.09526 | ni | conserved hypothetical protein |
| 12d00090 | 0.21037 | ni | hypothetical protein | um05223 | 0.09528 | ni | ni |
| 5c00053 | 0.21039 | ni | hypothetical protein | um01185 | 0.0953 | ni | conserved hypothetical protein |
| 2c00027 | 0.21039 | ni | uncharacterized conserved protein | um03911 | 0.09535 | K15116 | related to RIM2 - Protein of the mitochondrial carrier family |
| 25d00035 | 0.21049 | K12668 | defender against cell death protein | um01200 | 0.09586 | ni | related to histone acetyltransferase |
| 14d00062 | 0.21073 | ni | predicted Golgi transport complex 1 protein | um11354 | 0.09595 | K10689 | probable ubiquitin-conjugating enzyme E2 |
| 9c00312 | 0.21081 | ni | hypothetical protein | um03586 | 0.09635 | ni | putative protein |
| 11c00090 | 0.21084 | K01130 | sulfatases | um00848.2 | 0.09646 | K12178 | related to COP9 - signalosome complex subunit 4 |
| 20d00034 | 0.21088 | ni | carboxylesterase and related proteins | um05732 | 0.09656 | ni | related to phytochrome |
| 9c00170 | 0.21094 | K03434 | N-acetylglucosaminyl phosphatidylinositol de-N-acetylase | um06358 | 0.09675 | ni | hypothetical protein |
| 16c00018 | 0.21129 | K00728 | dolichyl-phosphate-mannose, protein O-mannosyl transferase | um06312 | 0.09682 | ni | putative protein |
| 14d00105 | 0.21154 | K12608 | predicted ABC-type transport, ATPase component | um11276 | 0.09714 | ni | conserved hypothetical protein |

| | | | | | | | |
|---|---|---|---|---|---|---|---|
| 22d00152 | 0.21155 | ni | hypothetical protein | um06000 | 0.09718 | ni | conserved hypothetical protein |
| 22c00106 | 0.21159 | K00706 | 1,3-beta-glucan synthase | um03196 | 0.09728 | ni | hypothetical protein |
| 8c00031 | 0.21163 | K14861 | uncharacterized conserved protein | um00332 | 0.09753 | K15201 | related to transcription factor TFIIIC subunit |
| 1c00032 | 0.21165 | ni | hypothetical protein | um10838 | 0.09807 | ni | putative protein |
| 22c00057 | 0.21166 | K11230 | MAPKKK (MAP kinase kinase kinase) SSK2 | um02854 | 0.09809 | ni | hypothetical protein |
| 3c00055 | 0.21187 | ni | predicted mitochondrial/chloroplast ribosomal protein S17 | um03886 | 0.09816 | ni | conserved hypothetical protein |
| 18d00096 | 0.212 | K16075 | magnesium transporters, CorA family | um01091 | 0.0986 | K14767 | related to SAS10 - involved in silencing |
| 16c00026 | 0.21206 | ni | hypothetical protein | um10725 | 0.09865 | ni | hypothetical protein |
| 24d00009 | 0.2121 | ni | hypothetical protein | um00292 | 0.0987 | K14791 | related to WD repeat protein PWP1 |
| 22d00066 | 0.2121 | ni | protein DRE2 | um00722.2 | 0.09909 | K11375 | conserved hypothetical protein |
| 7d00019 | 0.21211 | K04532 | NEDD8-activating complex, APP-BP1/UBA5 component | um11659 | 0.09909 | ni | probable PCS60 - AMP-binding protein |
| 6c00117 | 0.21245 | K10413 | dyneins, heavy chain | um02752 | 0.09911 | ni | putative protein |
| 22d00264 | 0.21258 | ni | predicted member of the intramitochondrial sorting protein family | um00159 | 0.09912 | ni | conserved hypothetical protein |
| 13c00021 | 0.21265 | K15116 | mitochondrial carrier protein - Rim2p/Mrs12p | um10246 | 0.09943 | K05756 | probable ARC18 - subunit of the Arp2 |
| 22d00159 | 0.2128 | ni | aldo/keto reductase family proteins | um12058 | 0.09943 | ni | conserved hypothetical Ustilago-specific protein |
| 6c00128 | 0.21282 | ni | hypothetical protein | um11400 | 0.09946 | K03146 | probable Thiamin biosynthetic enzyme |
| 1c00014 | 0.21284 | K03259 | translation initiation factor 4F, cap-binding subunit | um10280 | 0.09968 | K02153 | related to vacuolar ATP synthase subunit H |
| 12d00041 | 0.21289 | ni | cell cycle-associated protein Mob1-1 | um15080 | 0.09978 | ni | conserved hypothetical protein |
| 10d00023 | 0.21301 | K03013 | RNA polymerase, 25-kDa subunit | um03662 | 0.10003 | K05917 | sterol 14 alpha-demethylase |
| 11d00062 | 0.2131 | ni | predicted mitochondrial carrier protein | um02468 | 0.10016 | ni | conserved hypothetical protein |
| 16d00013 | 0.21315 | ni | voltage-gated Ca2+ channels, alpha1 subunits | um05094 | 0.10016 | ni | conserved hypothetical Ustilago-specific protein |
| 19d00080 | 0.21327 | ni | hypothetical protein | um05856.2 | 0.10021 | ni | putative protein |
| 27d00029 | 0.21345 | ni | uncharacterized conserved protein | um10399 | 0.10024 | ni | related to C-type cyclin |
| 22d00107 | 0.21351 | ni | CCCH-type Zn-finger protein | um11008 | 0.10025 | K15271 | related to HFM1 - DNA |
| 7c00278 | 0.21353 | ni | hypothetical protein | um02846.2 | 0.10041 | ni | related to WD40 repeat protein CreC |
| 9d00017 | 0.21354 | K01426 | amidases | um03466 | 0.10058 | ni | putative protein |
| 24c00029 | 0.2136 | K15376 | molybdopterin biosynthesis protein | um05654 | 0.10062 | K07208 | probable ras-related GTP-binding protein |
| 10c00082 | 0.21376 | ni | hypothetical protein | um01705 | 0.10066 | ni | conserved hypothetical protein |
| 20d00042 | 0.21376 | K01784 | UDP-glucose 4-epimerase | um03206 | 0.10071 | K00943 | related to thymidylate kinase |
| 27c00078 | 0.21394 | ni | hypothetical protein | um11787 | 0.10082 | K14842 | probable NSA2 - involved in biogenesis of ribosomal large subunit |
| 20d00058 | 0.21408 | ni | 17 beta-hydroxysteroid dehydrogenase type 3 | um11423 | 0.10091 | ni | conserved hypothetical protein |
| 19d00118 | 0.21417 | K03028 | 26S proteasome regulatory complex, subunit RPN1/PSMD2 | um00444 | 0.10106 | ni | conserved hypothetical protein |
| 5d00039 | 0.21418 | ni | cystathionine beta-lyases | um05436 | 0.10113 | ni | conserved hypothetical Ustilago-specific protein |
| 18c00033 | 0.21427 | K00417 | ubiquinol cytochrome c reductase, subunit QCR7 | um11766 | 0.1012 | ni | conserved hypothetical protein |
| 15c00076 | 0.21436 | K02127 | mitochondrial F1F0-ATP synthase, subunit b/ATP4 | um11052 | 0.10121 | K06875 | conserved hypothetical protein |
| 9c00150 | 0.21447 | K09008 | uncharacterized conserved nuclear protein | um05299 | 0.10149 | ni | conserved hypothetical Ustilago-specific protein |
| 19c00113 | 0.21451 | ni | hypothetical protein | um11375 | 0.10156 | ni | hypothetical protein |
| 19d00071 | 0.21463 | ni | hypothetical protein | um05163 | 0.10159 | K13127 | conserved hypothetical protein |
| 5d00146 | 0.21474 | ni | hypothetical protein | um06401 | 0.1016 | ni | conserved hypothetical protein |
| 9d00199 | 0.21495 | ni | hypothetical protein | um02296 | 0.10185 | ni | conserved hypothetical Ustilago-specific protein |
| 22d00219 | 0.21517 | ni | hypothetical protein | um02624 | 0.10193 | ni | related to Alpha-1 |
| 24d00057 | 0.21526 | ni | RNA-directed RNA polymerase QDE-1 | um02124 | 0.10207 | K00598 | conserved hypothetical protein |
| 19c00006 | 0.21541 | ni | non-ribosomal peptide synthetase | um10391 | 0.10218 | ni | related to SKT5 - protoplast regeneration and killer toxin resistance protein |
| 7c00271 | 0.21546 | ni | hypothetical protein | um00102 | 0.10224 | ni | conserved hypothetical protein |
| 2d00047 | 0.2155 | K10696 | E3 ubiquitin ligase involved in syntaxin degradation | um10701 | 0.10273 | K02922 | probable RPL37A - ribosomal protein L37 |
| 12d00064 | 0.21555 | K03510 | DNA polymerase iota | um05979 | 0.10298 | K00521 | related to ferric reductase |
| 10d00059 | 0.21556 | K01529 | ATP-dependent RNA helicase | um10283 | 0.10304 | K00295 | related to MTD1 - methylenetetrahydrofolate dehydrogenase |
| 7c00320 | 0.21558 | ni | hypothetical protein | um02274 | 0.10316 | ni | conserved hypothetical protein |
| 9d00210 | 0.21561 | K02960 | 40S ribosomal protein S16 | um10740 | 0.10327 | K12668 | related to apoptotic cell death regulator DAD1 |
| 18c00056 | 0.21572 | K11835 | ubiquitin C-terminal hydrolase | um00794 | 0.10338 | ni | conserved hypothetical Ustilago-specific protein |
| 9d00050 | 0.21573 | ni | hypothetical protein | um05853 | 0.10344 | ni | conserved hypothetical protein |
| 7c00294 | 0.21576 | ni | hypothetical protein | um10666 | 0.10355 | K12858 | probable U5 snRNP 100 kD protein |
| 13c00033 | 0.21581 | K03949 | NADH:ubiquinone oxidoreductase, NDUFA5/B13 subunit | um10092.2 | 0.10367 | K15429 | related to met-10 protein |
| 8d00028 | 0.21592 | ni | hypothetical protein | um04327 | 0.10376 | K10597 | related to UFD2 - ubiquitin fusion degradation protein |
| 19d00026 | 0.21598 | K01895 | acyl-coa synthetase | um04134 | 0.10377 | ni | probable methionine synthase |
| 22c00291 | 0.2161 | ni | hypothetical protein | um05832 | 0.10382 | K04078 | probable heat shock protein 10 |
| 4d00016 | 0.21628 | K01956 | multifunctional pyrimidine synthesis protein CAD | um00669 | 0.10384 | K10842 | related to TFB3 - TFIIH subunit |
| 27c00067 | 0.21639 | K10756 | replication factor C, subunit RFC5 | um02020 | 0.10387 | K14860 | conserved hypothetical protein |
| 9c00431 | 0.21649 | ni | spliceosomal protein snRNP-U1A/U2B | um03985 | 0.10405 | ni | related to SIW14 - protein involved in actin filament organization |
| 10d00067 | 0.21663 | ni | hypothetical protein | um05473 | 0.10405 | K15289 | conserved hypothetical protein |
| 11d00006 | 0.21676 | K05906 | hypothetical protein | um03017 | 0.10407 | ni | putative protein |
| 3c00060 | 0.21683 | ni | hypothetical protein | um04630 | 0.10423 | K08991 | related to MUS81 - endonuclease involved in DNA repair and replication fork stability |
| 8c00107 | 0.21699 | ni | hypothetical protein | um01606 | 0.10423 | ni | related to NMD5 - Nam7p interacting protein |
| 20d00047 | 0.21705 | ni | hypothetical protein | um11024 | 0.1044 | ni | putative protein |
| 5c00103 | 0.21711 | K01883 | cysteinyl-trna synthetase | um12306 | 0.10465 | K15148 | related to MED7 - member of RNA Polymerase II transcriptional regulation mediator complex |
| 26c00017 | 0.21737 | ni | predicted membrane protein | um11768 | 0.10507 | ni | hypothetical protein |
| 9c00022 | 0.21745 | ni | uncharacterized conserved protein | um10711 | 0.10515 | K02984 | probable ribosomal protein S3aE |
| 14c00029 | 0.21746 | K11968 | predicted E3 ubiquitin ligase | um11441 | 0.10542 | ni | conserved hypothetical protein |
| 15c00013 | 0.21764 | K06173 | pseudouridylate synthase | um01677 | 0.10543 | ni | hypothetical protein |
| 22d00216 | 0.21767 | ni | hypothetical protein | um11479 | 0.10548 | K05758 | probable ARC35 - subunit of the Arp2 |
| 15c00064 | 0.21774 | ni | hypothetical protein | um00831 | 0.10556 | ni | conserved hypothetical Ustilago-specific protein |
| 22c00179 | 0.21786 | K07127 | transthyretin and related proteins | um10990 | 0.10556 | K01855 | related to DEG1 - pseudouridine synthase |
| 15d00049 | 0.21786 | ni | hypothetical protein | um02252 | 0.10632 | K03241 | conserved hypothetical protein |
| 7c00333 | 0.21806 | ni | hypothetical protein | um02730 | 0.10663 | ni | conserved hypothetical protein |
| 27d00041 | 0.21847 | ni | hypothetical protein | um01995 | 0.10676 | ni | conserved hypothetical protein |
| 19d00163 | 0.21849 | ni | hypothetical protein | um10271 | 0.1073 | ni | conserved hypothetical protein |
| 11c00016 | 0.21856 | K00135 | aldehyde dehydrogenase | um10118 | 0.10735 | ni | conserved hypothetical protein |
| 24d00014 | 0.21865 | K15901 | hypothetical protein | um01830 | 0.1074 | ni | related to alpha-aminoadipate reductase |
| 11c00053 | 0.21871 | ni | hypothetical protein | um01274 | 0.10743 | K15305 | conserved hypothetical protein |
| 6c00121 | 0.21874 | ni | hypothetical protein | um03358 | 0.10772 | ni | related to SNF1-related protein kinase KIN10 |
| 18d00066 | 0.21881 | ni | hypothetical protein | um15073 | 0.10778 | ni | conserved hypothetical protein |
| 24d00027 | 0.21888 | ni | hypothetical protein | um10570 | 0.10793 | ni | conserved hypothetical protein |
| 22d00028 | 0.21889 | K12598 | nuclear exosomal RNA helicase MTR4 | um03645 | 0.10804 | ni | related to beta-1 |
| 12d00141 | 0.21909 | ni | hypothetical protein | um10356 | 0.10819 | K02739 | probable 20S proteasome beta2 subunit |
| 7d00354 | 0.21922 | ni | hypothetical protein | um11093.2 | 0.10821 | ni | putative protein |
| 22c00213 | 0.21937 | ni | hypothetical protein | um05959 | 0.10821 | ni | conserved hypothetical protein |
| 9d00407 | 0.21953 | K07204 | guanine nucleotide binding protein MIP1 | um10014 | 0.10848 | ni | related to HMF1 - Heat-shock induceable Inhibitor of cell Growth |
| 7d00133 | 0.21963 | ni | hypothetical protein | um12128 | 0.10849 | ni | conserved hypothetical protein |
| 24d00018 | 0.21969 | ni | thioredoxin-like protein | um10552 | 0.10861 | K01322 | related to Prolyl endopeptidase |
| 9d00300 | 0.21986 | ni | hypothetical protein | um00966 | 0.10868 | ni | related to RAD26 - DNA repair and recombination protein |
| 22c00039 | 0.22 | ni | low density lipoprotein B-like protein | um04269 | 0.109 | ni | conserved hypothetical protein |
| 6d00028 | 0.22003 | K11173 | alcohol dehydrogenase | um05949 | 0.10912 | ni | probable actin interacting protein 1 |
| 22d00263 | 0.22005 | ni | uncharacterized conserved protein | um03617 | 0.10914 | K14861 | conserved hypothetical protein |
| 22d00269 | 0.22006 | ni | hypothetical protein | um02091 | 0.10942 | K02835 | related to MRF1 - peptide chain release factor |
| 3d00041 | 0.2202 | K06679 | mitotic checkpoint protein MAD1 | um15017 | 0.10944 | ni | conserved hypothetical protein |
| 7d00110 | 0.22023 | K14833 | predicted protein involved in nuclear export of pre-ribosomes | um11570 | 0.10969 | ni | conserved hypothetical protein |
| 11d00034 | 0.22027 | ni | aminopeptidases of the M20 family | um03024 | 0.10971 | ni | related to subtilisin-like serine protease |
| 15c00031 | 0.22037 | ni | centromere/kinetochore protein zw10 | um02103 | 0.10992 | K01802 | probable RRD2 - Activator of the phosphotyrosyl phosphatase activity of PP2A |
| 22c00138 | 0.22041 | ni | Na+-independent Cl/HCO3 exchanger AE1 | um01849 | 0.10992 | ni | conserved hypothetical protein |
| 7d00086 | 0.22049 | ni | hypothetical protein | um02716 | 0.11014 | ni | putative protein |
| 9c00383 | 0.22057 | ni | hypothetical protein | um04239 | 0.11025 | K03236 | probable TIF11 - translation initiation factor eIF1a |
| 9d00179 | 0.22083 | ni | hypothetical protein | um06191 | 0.11025 | ni | conserved hypothetical protein |
| 5d00071 | 0.22083 | ni | WD40-repeat-containing subunit of the 18S rRNA processing co | um00538 | 0.11037 | ni | conserved hypothetical protein |
| 27d00024 | 0.22101 | ni | predicted sugar kinase | um02323 | 0.11037 | K13343 | related to PEX14 - peroxisomal protein involved in protein import - peroxin |
| 2d00030 | 0.22114 | ni | FOG, Zn-finger | um00080 | 0.11042 | ni | related to 5-oxoprolinase |
| 22d00060 | 0.22142 | K07955 | GTP-binding ADP-ribosylation factor-like protein | um00174 | 0.1106 | K11292 | related to transcriptional regulator protein SPT6 |
| 19c00030 | 0.22146 | K14399 | mRNA cleavage and polyadenylation factor IA/II complex, subunit | um02471 | 0.11077 | ni | related to Exportin 4 |
| 8d00110 | 0.22152 | ni | hypothetical protein | um10767 | 0.11088 | ni | related to DPH2 - diphtheria toxin resistance protein |
| 25d00039 | 0.22157 | K02725 | 20S proteasome, regulatory subunit alpha type PSMA1/PRE5 | um00507 | 0.11125 | K15128 | conserved hypothetical protein |
| 19d00145 | 0.22162 | K14300 | nuclear pore complex, Nup133 component | um12110 | 0.11154 | K02258 | probable COX11 - cytochrome-c oxidase assembly protein |
| 7c00254 | 0.2217 | ni | hypothetical protein | um12142 | 0.11188 | ni | conserved hypothetical protein |
| 24d00036 | 0.22175 | K01814 | phosphoribosylformimino-5-aminoimidazole carboxamide ribonucl | um03798 | 0.11201 | K01867 | probable tryptophan--tRNA ligase |
| 9d00092 | 0.22183 | ni | hypothetical protein | um03905 | 0.11202 | ni | conserved hypothetical protein |
| 22d00036 | 0.22206 | ni | hypothetical protein | um02403.2 | 0.11269 | K15322 | related to tRNA splicing endonuclease beta subunit |
| 5d00093 | 0.22208 | ni | hypothetical protein | um06374 | 0.11272 | ni | related to GRE2 - methylglyoxal reductase |
| 18d00035 | 0.22214 | ni | hypothetical protein | um04405 | 0.11286 | K05546 | alpha-glucosidase II precursor |
| 1d00039 | 0.2222 | K04739 | cAMP-dependent protein kinase types I and II, regulatory subunit | um11579 | 0.11296 | ni | conserved hypothetical Ustilago-specific protein |
| 20c00002 | 0.22234 | ni | hypothetical protein | um03403.2 | 0.11297 | ni | conserved hypothetical protein |
| 7c00351 | 0.22253 | ni | predicted transporter | um11653 | 0.11298 | K01764 | related to CYC3 - holocytochrome-c synthase |
| 8c00014 | 0.22256 | ni | hypothetical protein | um11204 | 0.11319 | K10686 | related to ubiquitin-activating enzyme |
| 22c00126 | 0.2226 | K06655 | protein kinase PCTAIRE and related kinases | um10850 | 0.11348 | ni | putative protein |
| 9c00040 | 0.22274 | ni | hypothetical protein | um03676 | 0.11354 | K10877 | related to RAD54 - DNA-dependent ATPase of the Snf2p family |
| 8c00106 | 0.22304 | ni | uncharacterized conserved protein | um11722 | 0.11363 | ni | probable YDJ1 - mitochondrial and ER import protein |
| 7d00265 | 0.22307 | K12881 | RRM motif-containing protein | um15004 | 0.11384 | K01638 | probable malate synthase |
| 19c00096 | 0.22318 | K04709 | protein transporter | um10330 | 0.11393 | K14209 | related to AVT3 - Vacuolar transporter |
| 6d00008 | 0.2233 | ni | acetylcholinesterase | um11883.2 | 0.11401 | ni | putative protein |
| 14c00107 | 0.22346 | K11087 | small nuclear ribonucleoprotein SMD1 and related snRNPs | um01405 | 0.11409 | ni | conserved hypothetical protein |
| 25c00058 | 0.22351 | K04513 | ras-related small GTPase | um02384 | 0.1142 | ni | a1-mating type protein Rba1 |
| 7d00225 | 0.22367 | K03115 | casein kinase II, beta subunit | um05649 | 0.11438 | ni | conserved hypothetical protein |
| 9d00377 | 0.22372 | K14539 | predicted GTP-binding protein MMR1 | um04344 | 0.11439 | ni | conserved hypothetical protein |
| 18d00060 | 0.22388 | K12669 | oligosaccharyltransferase, gamma subunit | um10113 | 0.1149 | ni | conserved hypothetical protein |
| 9c00020 | 0.22392 | ni | phytoene/squalene synthetase | um02690 | 0.11491 | ni | hypothetical protein |
| 9c00306 | 0.22406 | ni | FOG, RCC1 domain | um04428 | 0.11494 | ni | conserved hypothetical protein |
| 22d00018 | 0.22406 | K01194 | neutral trehalase | um03967 | 0.11501 | K12617 | related to PAT1 - topoisomerase II-associated protein |
| 9c00291 | 0.22407 | ni | hypothetical protein | um10013.2 | 0.11547 | ni | related to l-gulonolactone oxidase |
| 2c00023 | 0.22432 | K15334 | tRNA cytosine-5-methylases | um11841 | 0.11625 | K15278 | related to YEA4 - uridine diphosphate-N-acetylglucosamine transporter |
| 9c00268 | 0.22452 | ni | cell cycle-associated protein | um02990 | 0.11646 | ni | conserved hypothetical protein |
| 22d00266 | 0.22472 | K14685 | iron transporter | um03211 | 0.11652 | ni | related to peroxiredoxin q |
| 4d00028 | 0.22476 | ni | hypothetical protein | um05485 | 0.11658 | K11681 | related to SWR1 - DEAH-box protein |
| 5d00066 | 0.22511 | ni | hypothetical protein | um12263 | 0.11662 | ni | hypothetical protein |
| 16c00074 | 0.22512 | ni | WD40-like repeat containing protein | um11703.2 | 0.11689 | K15900 | probable KAE1 - Putative O-sialo-glycoprotein-endopeptidase A1 |
| 7d00356 | 0.22522 | ni | glucose-repressible protein and related proteins | um05891 | 0.117 | ni | conserved hypothetical protein |
| 7d00283 | 0.22542 | K02140 | mitochondrial F1F0-ATP synthase, subunit g/ATP20 | um03673 | 0.117 | ni | conserved hypothetical protein |
| 9c00241 | 0.22555 | K02926 | mitochondrial/chloroplast ribosomal protein L4 | um10337 | 0.11713 | K10576 | probable ubiquitin-conjugating enzyme e2-23 kda |
| 15c00023 | 0.22562 | ni | hypothetical protein | um02659.2 | 0.11719 | ni | conserved hypothetical protein |
| 18d00115 | 0.22569 | ni | hypothetical protein | um02816 | 0.11733 | ni | putative protein |
| 22d00125 | 0.22597 | ni | threonine/serine dehydratases | um04524 | 0.1174 | K11498 | related to Kinesin |
| 14d00001 | 0.22619 | ni | predicted transporter | um00535 | 0.11741 | ni | conserved hypothetical protein |
| 7d00167 | 0.22645 | ni | hypothetical protein | um05636 | 0.11746 | ni | conserved hypothetical protein |
| 536c00001 | 0.22671 | K05781 | ni | um00756 | 0.11752 | K17255 | probable GDI1 - GDP dissociation inhibitor |
| 11d00090 | 0.22695 | ni | hypothetical protein | um10796 | 0.11793 | K13278 | related to l-asparaginase |
| 19d00054 | 0.22697 | K01166 | ribonuclease | um06421 | 0.11811 | K03189 | related to Gamm1 protein |
| 4c00027 | 0.22741 | ni | hypothetical protein | um05271 | 0.11821 | ni | ni |
| 7c00004 | 0.22748 | ni | hypothetical protein | um15094 | 0.11824 | ni | conserved hypothetical protein |
| 12c00073 | 0.22751 | K06027 | AAA+-type ATPase | um02303 | 0.11835 | K04459 | conserved hypothetical protein |
| 19d00021 | 0.22769 | K08853 | ARK protein kinase family | um11402 | 0.11888 | K13989 | conserved hypothetical protein |

| | | | | | | | |
|---|---|---|---|---|---|---|---|
| 10c00032 | 0.22785 | K12621 | predicted snRNP core protein | um02295 | 0.11925 | ni | conserved hypothetical Ustilago-specific protein |
| 5d00126 | 0.22869 | K14565 | ribosome biogenesis protein - Nop58p/Nop5p | um04498 | 0.11925 | ni | putative protein |
| 15d00020 | 0.22875 | ni | predicted oxidoreductase | um11742 | 0.11941 | ni | putative protein |
| 7d00061 | 0.2289 | K02914 | mitochondrial ribosomal protein L34 | um02756 | 0.11949 | ni | hypothetical protein |
| 19c00142 | 0.22895 | K12183 | vacuolar sorting protein | um05572 | 0.11957 | ni | related to PCH2 - putative ATPase |
| 7d00293 | 0.22901 | K01514 | exopolyphosphatases and related proteins | um02276 | 0.11979 | K02934 | probable 60S large subunit ribosomal protein L6 |
| 1d00032 | 0.22902 | K01078 | multiple inositol polyphosphate phosphatase | um10811 | 0.12003 | K07955 | related to ARL1 - ADP-ribosylation factor |
| 7c00073 | 0.22905 | ni | hypothetical protein | um05243 | 0.1201 | ni | hypothetical protein |
| 12c00070 | 0.2291 | ni | transport protein particle TRAPP complex subunit | um00543 | 0.12018 | K11429 | conserved hypothetical protein |
| 6d00065 | 0.2293 | ni | hypothetical protein | um05675 | 0.12036 | ni | conserved hypothetical protein |
| 18c00004 | 0.22931 | K14319 | ran GTPase-activating protein | um10241 | 0.12044 | K14778 | related to DBP8 - ATP-dependent RNA helicase |
| 22d00223 | 0.22944 | K13289 | serine carboxypeptidases | um05105 | 0.12061 | K15102 | probable phosphate transport protein MIR1 |
| 9d00298 | 0.22963 | ni | translational repressor Pumilio/PUF3 | um02833 | 0.12073 | K12273 | related to SEC66 - ER protein-translocation complex chain |
| 7d00210 | 0.22966 | K00995 | phosphatidylglycerolphosphate synthase | um04471 | 0.12077 | ni | conserved hypothetical protein |
| 20c00030 | 0.22966 | K02603 | origin recognition complex, subunit 1, and related proteins | um01100 | 0.12091 | ni | hypothetical protein |
| 14d00070 | 0.2297 | K16055 | trehalose-6-phosphate synthase component TPS1 and related su | um05459 | 0.12104 | ni | conserved hypothetical Ustilago-specific protein |
| 7d00187 | 0.22974 | ni | hypothetical protein | um00689 | 0.12111 | K14798 | related to LTV1 - low-temperature viability protein |
| 19d00053 | 0.22979 | K08735 | mismatch repair ATPase MSH2 | um06407 | 0.12147 | ni | probable VIP1 - actin cytoskeleton organization and biogenesis-related protein |
| 25d00027 | 0.2298 | ni | hypothetical protein | um12208 | 0.12158 | K02208 | related to SSN3 - cyclin-dependent CTD kinase |
| 7c00327 | 0.22983 | ni | hypothetical protein | um05708 | 0.1216 | ni | conserved hypothetical protein |
| 11d00044 | 0.22986 | ni | hypothetical protein | um10523 | 0.12161 | ni | conserved hypothetical protein |
| 9c00077 | 0.23001 | ni | very-long-chain acyl-CoA dehydrogenase | um05869 | 0.12199 | K15109 | probable carnitine |
| 12c00011 | 0.23005 | ni | hypothetical protein | um05358 | 0.12215 | ni | hypothetical protein |
| 5d00064 | 0.23021 | ni | hypothetical protein | um04177.2 | 0.12222 | ni | conserved hypothetical protein |
| 13d00103 | 0.23032 | K10843 | RNA pRNA polymerase II transcription initiation/nucleotide excisi | um02292 | 0.12283 | ni | conserved hypothetical protein |
| 9c00251 | 0.23038 | ni | mitochondrial phosphate carrier protein | um01576 | 0.12295 | ni | conserved hypothetical protein |
| 7d00322 | 0.2304 | ni | GTPase-activating protein VRP | um01105 | 0.12298 | K15628 | related to adrenoleukodystrophy protein |
| 5d00008 | 0.23076 | K02146 | vacuolar H+-ATPase V0 sector, subunit d | um03362 | 0.12339 | ni | conserved hypothetical protein |
| 19c00026 | 0.23079 | ni | glutathione S-transferase | um11759 | 0.12386 | K16578 | conserved hypothetical protein |
| 22c00257 | 0.23088 | K05609 | ubiquitin C-terminal hydrolase UCHL1 | um11311 | 0.1239 | ni | putative protein |
| 19d00002 | 0.23096 | ni | hypothetical protein | um12181 | 0.12411 | ni | conserved hypothetical protein |
| 27d00047 | 0.23133 | K04409 | p21-activated serine/threonine protein kinase | um00800 | 0.12415 | ni | probable PHO84 - Inorganic phosphate permease |
| 7c00211 | 0.23141 | ni | uncharacterized conserved protein | um02864 | 0.12454 | ni | related to Acetylcholinesterase precursor |
| 10d00105 | 0.23146 | K05687 | putative transcriptional regulator DJ-1 | um11830 | 0.1246 | ni | conserved hypothetical protein |
| 6d00020 | 0.23157 | ni | hypothetical protein | um11218 | 0.12467 | K17417 | conserved hypothetical protein |
| 9c00332 | 0.2317 | ni | uncharacterized conserved protein | um01435 | 0.12469 | ni | related to MCH4 - monocarboxylate transporter |
| 9c00424 | 0.23186 | ni | hypothetical protein | um03371 | 0.12473 | ni | conserved hypothetical protein |
| 22d00147 | 0.23193 | K07036 | predicted membrane proteins | um04584 | 0.12479 | K01694 | probable tryptophan synthase |
| 5c00023 | 0.23197 | ni | hypothetical protein | um00739 | 0.12492 | ni | related to DNA repair protein rad18 |
| 19c00145 | 0.23201 | ni | hypothetical protein | um03837 | 0.12504 | ni | KH domain protein |
| 14c00016 | 0.23205 | ni | emp24/gp25L/p24 family of membrane trafficking proteins | um03424 | 0.1251 | ni | conserved hypothetical protein |
| 6d00081 | 0.23222 | ni | ATPase component of ABC transporters with duplicated ATPase | um10583 | 0.12521 | ni | hypothetical protein |
| 9c00081 | 0.23247 | ni | hypothetical protein | um12016 | 0.12525 | ni | conserved hypothetical Ustilago-specific protein |
| 7d00190 | 0.23253 | ni | predicted membrane protein | um01742 | 0.12526 | K13728 | related to mitotic spindle assembly checkpoint protein mad2b |
| 27c00058 | 0.23265 | K11827 | clathrin adaptor complex, small subunit | um04057 | 0.12534 | ni | related to dihydroflavonol reductase |
| 7d00301 | 0.23273 | ni | FOG, Zn-finger | um11813 | 0.12564 | ni | putative protein |
| 15d00010 | 0.23278 | ni | amine oxidase | um11608.2 | 0.12571 | ni | hypothetical protein |
| 16d00057 | 0.2328 | ni | transport protein particle TRAPP complex subunit | um00522 | 0.12586 | ni | putative protein |
| 19c00090 | 0.23288 | K02919 | mitochondrial/chloroplast ribosomal protein L36 | um03771 | 0.12588 | ni | conserved hypothetical protein |
| 13d00064 | 0.23293 | ni | guanine nucleotide-binding protein | um10426 | 0.12594 | ni | transcription factor pacC |
| 7d00236 | 0.233 | ni | hypothetical protein | um00168 | 0.12602 | ni | related to Cell division control protein 15 |
| 27c00061 | 0.23301 | ni | hypothetical protein | um05141 | 0.12618 | K03511 | related to DNA polymerase kappa |
| 5c00095 | 0.23304 | ni | thiamine pyrophosphokinase | um05453 | 0.12621 | K14819 | related to Tyrosine specific protein phosphatase and dual specificity protein phosphatase |
| 14d00113 | 0.23316 | K03386 | alkyl hydroperoxide reductase | um11576 | 0.12642 | K02142 | probable ATP18 - subunit i |
| 3c00046 | 0.23322 | K15110 | mitochondrial oxodicarboxylate carrier protein | um03448 | 0.12658 | K00784 | related to Ribonuclease Z |
| 3c00009 | 0.23327 | K02202 | cdk activating kinase | um11136 | 0.12663 | ni | related to PFA4 - Palmitoyltransferase |
| 9c00373 | 0.23339 | ni | mitochondrial import inner membrane translocase, subunit TIM13 | um02280 | 0.12665 | ni | putative protein |
| 27d00030 | 0.23342 | K12587 | exosomal 3'-5' exoribonuclease complex, subunit Rrp41 | um04117 | 0.12696 | K07252 | related to CAX4 - required for full levels of dolichol-linked oligosaccharides |
| 7d00192 | 0.23344 | ni | iron/ascorbate family oxidoreductases | um00052 | 0.12708 | ni | putative protein |
| 8c00074 | 0.23346 | ni | FOG, WD40 repeat | um03987 | 0.12713 | K06676 | related to BRN1 - protein required for chromosome condensation |
| 7d00039 | 0.23369 | K16342 | lysophospholipase | um12021 | 0.12717 | ni | conserved hypothetical protein |
| 9d00256 | 0.23395 | ni | hypothetical protein | um11862 | 0.12737 | ni | conserved hypothetical protein |
| 10c00057 | 0.2341 | ni | PHD Zn-finger proteins | um10035 | 0.12742 | K09481 | probable protein transport protein sec61 beta subunit |
| 14d00040 | 0.23432 | K00653 | histone acetyltransferase | um10341 | 0.12773 | ni | conserved hypothetical protein |
| 7d00296 | 0.23458 | K13102 | protein containing a U1-type Zn-finger and implicated in RNA spli | um05022 | 0.12775 | ni | related to PKH1 - ser |
| 14d00084 | 0.23464 | ni | alkyl hydroperoxide reductase | um05626 | 0.12784 | ni | conserved hypothetical protein |
| 24c00018 | 0.23473 | K03963 | NADH, ubiquinone oxidoreductase | um11398 | 0.12814 | K11870 | related to UBP1 - ubiquitin-specific protease |
| 8d00091 | 0.23497 | ni | hypothetical protein | um01139.2 | 0.12842 | K02427 | conserved hypothetical protein |
| 9c00016 | 0.23529 | K06916 | predicted ATPase | um04800 | 0.12856 | ni | conserved hypothetical protein |
| 18d00002 | 0.23532 | ni | hypothetical protein | um00325 | 0.12858 | K06664 | related to peroxisome assembly protein car1 |
| 18d00095 | 0.23536 | K11238 | rhoa GTPase effector DIA/Diaphanous | um12167 | 0.12858 | ni | conserved hypothetical protein |
| 9d00409 | 0.23545 | ni | hypothetical protein | um02599 | 0.12859 | ni | related to lysophospholipase |
| 8d00052 | 0.23552 | K00420 | hypothetical protein | um02498 | 0.12867 | K13076 | related to delta-6 fatty acid desaturase |
| 19c00070 | 0.23562 | ni | hypothetical protein | um00907 | 0.12871 | K12177 | related to cop9 complex subunit 3 |
| 7d00121 | 0.23574 | K10768 | hypothetical protein | um05600 | 0.12877 | ni | probable UGA2 - succinate semialdehyde dehydrogenase |
| 8c00058 | 0.23575 | ni | hypothetical protein | um10173 | 0.12915 | K05755 | probable ARP2 |
| 6c00027 | 0.23587 | ni | uncharacterized conserved protein | um02454 | 0.12917 | ni | conserved hypothetical protein |
| 7d00050 | 0.23593 | ni | hypothetical protein | um05182 | 0.12921 | ni | hypothetical protein |
| 12d00077 | 0.23616 | K01262 | xaa-pro aminopeptidase | um02674 | 0.12934 | ni | conserved hypothetical protein |
| 21c00002 | 0.23623 | ni | hypothetical protein | um04566 | 0.12946 | ni | putative protein |
| 9c00015 | 0.23634 | ni | permease of the major facilitator superfamily | um02789 | 0.12965 | ni | hypothetical protein |
| 9d00415 | 0.23652 | ni | hypothetical protein | um11326 | 0.13008 | ni | conserved hypothetical protein |
| 7c00010 | 0.23653 | K03135 | transcription initiation factor TFIID, subunit TAF11 | um05265 | 0.13026 | ni | hypothetical protein |
| 26d00016 | 0.2366 | K02898 | 60S ribosomal protein L26 | um04259 | 0.13034 | ni | conserved hypothetical protein |
| 26d00104 | 0.23666 | ni | hypothetical protein | um05890 | 0.13036 | K17413 | conserved hypothetical protein |
| 13d00024 | 0.23695 | ni | rhogef GTPase | um01374 | 0.13039 | ni | hypothetical protein |
| 26d00035 | 0.23704 | ni | hypothetical protein | um02779 | 0.13052 | ni | conserved hypothetical protein |
| 16c00054 | 0.23745 | K15205 | hypothetical protein | um10530 | 0.1307 | ni | hypothetical protein |
| 5d00033 | 0.23748 | K05292 | GPI transamidase complex, GPI16/PIG-T component | um05268 | 0.13086 | K14951 | conserved hypothetical protein |
| 16d00024 | 0.23754 | K11681 | SNF2 family DNA-dependent ATPase | um11464 | 0.13094 | ni | conserved hypothetical protein |
| 8c00030 | 0.23787 | ni | glucose dehydrogenase | um05829 | 0.13095 | K12877 | probable mago nashi protein |
| 15c00052 | 0.23794 | ni | hypothetical protein | um02128 | 0.13128 | ni | conserved hypothetical protein |
| 16c00085 | 0.23827 | K00452 | 3-hydroxyanthranilate oxygenase HAAO | um01367 | 0.1317 | K11824 | probable alpha-adaptin C |
| 2d00032 | 0.23828 | ni | hypothetical protein | um10101 | 0.13177 | K10734 | putative protein |
| 14d00108 | 0.2383 | K16794 | WD40 repeat-containing protein | um00290 | 0.13189 | K03507 | related to DNA topoisomerase II binding protein |
| 9c00416 | 0.23835 | ni | hypothetical protein | um02758 | 0.132 | ni | related to Chitinase A precursor |
| 11d00072 | 0.23878 | K03016 | RNA polymerase subunit 8 | um06491 | 0.13203 | K00565 | related to RNA |
| 6c00023 | 0.23888 | ni | hypothetical protein | um04891 | 0.13208 | ni | conserved hypothetical Ustilago-specific protein |
| 24c00067 | 0.23896 | ni | hypothetical protein | um12130 | 0.13214 | K11098 | probable Small nuclear ribonucleoprotein F |
| 7c00126 | 0.23896 | ni | uncharacterized conserved protein | um05533 | 0.13227 | ni | related to EDE1 protein involved in endocytosis |
| 7c00349 | 0.23897 | ni | cdc42-interacting protein CIP4 | um00435 | 0.13244 | K02926 | related to 50S ribosomal protein L4 |
| 19d00032 | 0.239 | ni | diphthamide biosynthesis protein | um11026 | 0.13273 | K16342 | related to phospholipase A2 |
| 15d00002 | 0.23917 | ni | hypothetical protein | um00817 | 0.13288 | K12176 | probable COP9 signalosome complex subunit 2 |
| 27c00028 | 0.23924 | K03065 | 26S proteasome regulatory complex | um02541 | 0.13299 | ni | putative protein |
| 22c00136 | 0.23926 | ni | hypothetical protein | um00225 | 0.13304 | ni | putative protein |
| 24d00033 | 0.23938 | K08658 | prenyl protein protease | um12013 | 0.13309 | K14799 | related to TSR1 - protein involved in 20S rRNA accumulation |
| 6d00019 | 0.23946 | ni | hydroxysteroid 17-beta dehydrogenase 11 | um05564 | 0.13311 | K10592 | related to E3 ubiquitin protein ligase TOM1 |
| 9c00197 | 0.23964 | K05284 | mannosyltransferase | um00695 | 0.13314 | K12373 | putative exochitinase |
| 18d00047 | 0.23976 | ni | kinesin | um02239 | 0.13328 | ni | hypothetical protein |
| 9d00182 | 0.23977 | K01900 | succinyl-coa synthetase, beta subunit | um04370 | 0.13329 | ni | conserved hypothetical protein |
| 18c00102 | 0.23982 | ni | alpha-d-galactosidase | um11847 | 0.13336 | ni | conserved hypothetical Ustilago-specific protein |
| 19d00099 | 0.23983 | ni | UV radiation resistance associated protein | um04299 | 0.13343 | K03253 | related to PRT1 - translation initiation factor eIF3 subunit |
| 12d00121 | 0.23998 | K13192 | proteins containing the RNA recognition motif | um03481 | 0.13355 | ni | related to MLH3 - insertion and deletion mismatch repair protein |
| 7d00077 | 0.24001 | ni | hypothetical protein | um10962 | 0.13393 | ni | conserved hypothetical protein |
| 27c00081 | 0.24002 | K09550 | molecular chaperone Prefoldin, subunit 4 | um11733.2 | 0.13395 | K12621 | related to LSM2 - Sm-like |
| 3d00066 | 0.24006 | K02903 | 60S ribosomal protein L28 | um02149 | 0.13402 | ni | putative protein |
| 6d00035 | 0.24008 | ni | hypothetical protein | um04627 | 0.13404 | ni | putative protein |
| 5c00101 | 0.24031 | ni | uncharacterized conserved protein | um10885.2 | 0.13425 | ni | putative protein |
| 9c00313 | 0.24032 | ni | hypothetical protein | um02771 | 0.13429 | ni | putative protein |
| 9d00244 | 0.24045 | ni | transcription initiation factor IID subunit | um04768 | 0.13433 | ni | probable SAC6 - actin filament bundling protein |
| 20d00001 | 0.24045 | ni | dehydrogenases with different specificities | um15012 | 0.13442 | ni | conserved hypothetical protein |
| 10d00058 | 0.24059 | ni | hypothetical protein | um10775 | 0.13463 | ni | conserved hypothetical protein |
| 8c00086 | 0.24075 | ni | hypothetical protein | um10937 | 0.13466 | ni | conserved hypothetical protein |
| 16c00025 | 0.24082 | ni | predicted small membrane protein | um02645 | 0.13494 | K12859 | probable DIB1 - 17-kDa component of the U4 |
| 9d00073 | 0.24128 | ni | hypothetical protein | um01216 | 0.13494 | ni | ni |
| 9c00209 | 0.24129 | ni | permease of the major facilitator superfamily | um11048 | 0.13516 | ni | conserved hypothetical protein |
| 24d00062 | 0.24134 | K08493 | V-SNARE | um00447 | 0.13534 | ni | conserved hypothetical protein |
| 22d00207 | 0.24142 | K00641 | hypothetical protein | um01853 | 0.13549 | ni | related to antibiotic biosynthesis protein |
| 11c00046 | 0.24146 | K14802 | P-type ATPase | um03442 | 0.13555 | K10592 | related to ubiquitin-protein ligase 1 |
| 7d00233 | 0.24164 | ni | hypothetical protein | um11273 | 0.13556 | K08675 | probable PIM1 - ATP-dependent protease |
| 27c00093 | 0.24183 | ni | predicted phosphatase | um10965.2 | 0.13559 | ni | putative protein |
| 5d00124 | 0.24191 | K00088 | IMP dehydrogenase | um04372 | 0.13564 | K10413 | cytoplasmic dynein heavy chain 2 |
| 24c00006 | 0.24202 | ni | Ca2+/calmodulin-dependent protein kinase | um02312 | 0.13593 | ni | ni |
| 7c00012 | 0.24215 | ni | hypothetical protein | um15083.2 | 0.13594 | ni | conserved hypothetical protein |
| 7d00350 | 0.24216 | ni | hypothetical protein | um00133 | 0.13599 | K01062 | related to acetylhydrolase |
| 22d00031 | 0.24219 | K05955 | protein farnesyltransferase, alpha subunit | um04421 | 0.13608 | ni | hypothetical protein |
| 20c00065 | 0.24226 | ni | hypothetical protein | um01578 | 0.13615 | ni | related to protein CS029 |
| 11c00021 | 0.24232 | ni | sexual differentiation process protein ISP4 | um03027 | 0.13629 | ni | related to MON1 - required for fusion of cvt-vesicles and autophagosomes with the vacuole |
| 14d00017 | 0.24266 | ni | hypothetical protein | um00982 | 0.13633 | K06689 | probable Ubiquitin conjugating enzyme E2-16 kDa |
| 1c00005 | 0.24284 | ni | hypothetical protein | um11408.2 | 0.13635 | ni | conserved hypothetical protein |
| 20d00077 | 0.24292 | ni | hypothetical protein | um01994 | 0.13652 | ni | conserved hypothetical protein |
| 10c00007 | 0.243 | ni | hypothetical protein | um02031 | 0.13677 | ni | conserved hypothetical protein |
| 18c00010 | 0.24317 | ni | synaptic vesicle transporter SVOP and related transporters | um02869 | 0.13693 | K15378 | related to General alpha-glucoside permease |
| 20c00031 | 0.2432 | ni | hypothetical protein | um00893 | 0.13712 | ni | related to veA protein |
| 19c00081 | 0.24328 | ni | hypothetical protein | um12123 | 0.13728 | ni | related to Nuclease Le3 |
| 9d00246 | 0.24331 | ni | AAA+-type ATPase | um03414 | 0.13737 | K01636 | probable xylulose-5-phosphate |
| 12c00052 | 0.24332 | ni | hypothetical protein | um06050 | 0.13747 | ni | conserved hypothetical protein |
| 22c00160 | 0.24348 | ni | uncharacterized conserved protein | um06324 | 0.13766 | K02137 | probable ATP5 - F1F0-ATPase complex |
| 27d00067 | 0.24359 | ni | hypothetical protein | um05749 | 0.13776 | ni | hypothetical protein |
| 7d00270 | 0.2436 | ni | hypothetical protein | um03567 | 0.13779 | K14765 | related to LCP5 - U3 small nucleolar ribonucleoprotein involved in maturation of 18S rRNA |
| 22c00031 | 0.24362 | ni | hypothetical protein | um03425 | 0.1378 | K00641 | related to MET2 - homoserine O-acetyltransferase |
| 24d00058 | 0.24379 | K05864 | HSP90 co-chaperone CPR7 | um01394 | 0.13789 | ni | conserved hypothetical Ustilago-specific protein |
| 26d00013 | 0.24386 | K12883 | nuclear cap-binding protein complex, subunit CBP20 | um12194 | 0.13803 | K14962 | conserved hypothetical protein |
| 9d00214 | 0.24388 | K14960 | uncharacterized PHD Zn-finger protein | um04833 | 0.1381 | K00253 | probable isovaleryl-CoA dehydrogenase |
| 22c00211 | 0.24422 | ni | hypothetical protein | um06514 | 0.13819 | ni | conserved hypothetical Ustilago-specific protein |
| 13c00081 | 0.24425 | ni | hypothetical protein | um05635 | 0.13851 | ni | putative protein |

| | | | | | | | |
|---|---|---|---|---|---|---|---|
| 5c00063 | 0.24432 | ni | hypothetical protein | um03083 | 0.13861 | ni | conserved hypothetical protein |
| 15c00006 | 0.24436 | K00728 | dolichyl-phosphate-mannose, protein O-mannosyl transferase | um01181 | 0.1387 | ni | conserved hypothetical protein |
| 12c00134 | 0.24443 | ni | hypothetical protein | um10742 | 0.13889 | ni | conserved hypothetical protein |
| 1d00027 | 0.24452 | K04564 | manganese superoxide dismutase | um06504 | 0.13894 | ni | conserved hypothetical protein |
| 26d00042 | 0.24459 | ni | hypothetical protein | um15056 | 0.13894 | K01533 | related to CCC2 - copper resistance-associated P-type ATPase |
| 19c00024 | 0.24487 | ni | 5' nucleotidase | um03720 | 0.13914 | K15117 | related to OAC1 - similarity to mitochondrial uncoupling proteins |
| 26c00091 | 0.24496 | K01424 | asparaginase | um15029 | 0.1395 | K12948 | related to SPC3 - signal peptidase subunit |
| 7d00306 | 0.24528 | ni | hypothetical protein | um05199 | 0.13957 | ni | related to VPS53 - subunit of VP51-54 complex |
| 9c00002 | 0.24531 | ni | hypothetical protein | um04044 | 0.13962 | ni | related to Glucose oxidase |
| 4c00012 | 0.24537 | ni | hypothetical protein | um02375 | 0.13964 | K14293 | probable karyopherin beta-1 subunit |
| 22d00172 | 0.24548 | K00432 | glutathione peroxidase | um11233 | 0.13973 | K02938 | probable RPL2A - ribosomal protein L8 |
| 13d00054 | 0.24555 | ni | serine carboxypeptidases | um10925 | 0.13973 | K11365 | conserved hypothetical protein |
| 16d00019 | 0.24556 | K15289 | predicted membrane protein | um12134 | 0.13979 | ni | hypothetical protein |
| 14d00028 | 0.24558 | ni | spindle pole body protein | um05910 | 0.13994 | ni | related to GRD19 protein |
| 22d00082 | 0.24562 | ni | hypothetical protein | um04317 | 0.1402 | ni | conserved hypothetical protein |
| 6d00078 | 0.24564 | ni | hypothetical protein | um01577 | 0.14027 | ni | conserved hypothetical protein |
| 20c00048 | 0.24568 | ni | hypothetical protein | um05213 | 0.14027 | K11314 | related to ADA2 - general transcriptional adaptor or co-activator |
| 13d00078 | 0.24569 | ni | hypothetical protein | um01153 | 0.14035 | K14556 | related to DIP2 - Dom34p-interacting protein |
| 1d00038 | 0.24598 | ni | bola (bacterial stress-induced morphogen)-related protein | um04048 | 0.1404 | ni | conserved hypothetical protein |
| 27d00082 | 0.24606 | K03842 | beta-1,4-mannosyltransferase | um06395 | 0.14074 | K12580 | related to NOT3 - general negative regulator of transcription |
| 9c00272 | 0.24613 | ni | phosphatidylinositol transfer protein PDR16 and related proteins | um06348 | 0.14075 | ni | related to SCP160 - involved in control of mitotic chromsome transmission |
| 16d00006 | 0.24626 | ni | monocarboxylate transporter | um06031 | 0.14084 | ni | conserved hypothetical protein |
| 25d00019 | 0.24626 | ni | hypothetical protein | um00059 | 0.14091 | K01011 | related to 3-mercaptopyruvate sulfurtransferase |
| 20c00033 | 0.24626 | K00948 | ribose-phosphate pyrophosphokinase | um06107 | 0.14102 | K03115 | related to casein kinase II beta subunit |
| 7c00162 | 0.24639 | ni | hypothetical protein | um11880 | 0.14104 | K02905 | probable RPL29 - 60S large subunit ribosomal protein |
| 3d00071 | 0.24665 | K00789 | S-adenosylmethionine synthetase | um03747 | 0.14116 | ni | conserved hypothetical Ustilago-specific protein |
| 9d00325 | 0.24712 | ni | hypothetical protein | um02444 | 0.14123 | K14191 | probable rRNA |
| 8d00004 | 0.24717 | ni | hypothetical protein | um03687 | 0.14129 | K05747 | related to Neural Wiskott-Aldrich syndrome protein |
| 25d00018 | 0.24719 | K07208 | ras-related GTPase | um05555 | 0.14162 | ni | conserved hypothetical protein |
| 7c00261 | 0.24737 | ni | hypothetical protein | um11533 | 0.14169 | K02917 | probable RPL33B - ribosomal protein L35a |
| 7c00199 | 0.24739 | K02427 | cell division protein FtsJ | um02945 | 0.1417 | K15147 | putative protein |
| 16c00055 | 0.24742 | ni | Ca2+/calmodulin-dependent protein kinase | um05724 | 0.1418 | ni | putative protein |
| 7d00197 | 0.24786 | K06287 | hydroxyindole-o-methyltransferase | um00427 | 0.14181 | ni | hypothetical protein |
| 19d00012 | 0.24805 | ni | nuclear export receptor CSE1/CAS | um05387 | 0.14197 | K14831 | probable MAK16 protein |
| 24d00007 | 0.24806 | ni | predicted fumarylacetoacetate hydralase | um02341 | 0.14197 | ni | putative protein |
| 3d00080 | 0.24817 | ni | hypothetical protein | um04510 | 0.14212 | ni | hypothetical protein |
| 9c00364 | 0.24824 | ni | hypothetical protein | um05125 | 0.14218 | K01886 | probable glutamine-tRNA ligase |
| 22d00252 | 0.24835 | K05607 | enoyl-coa hydratase | um06493 | 0.1422 | ni | conserved hypothetical protein |
| 20d00083 | 0.2484 | K02729 | 20S proteasome, regulatory subunit alpha type PSMA5/PUP2 | um10630 | 0.14228 | ni | putative protein |
| 6d00098 | 0.24845 | ni | hypothetical protein | um04478 | 0.14229 | K08150 | probable ITR2 - myo-inositol transporter |
| 16d00087 | 0.24846 | ni | hypothetical protein | um05541 | 0.1423 | ni | conserved hypothetical protein |
| 2c00002 | 0.24856 | K02136 | F0F1-type ATP synthase, gamma subunit | um00369 | 0.14259 | ni | conserved hypothetical protein |
| 25c00046 | 0.24863 | ni | hypothetical protein | um06029 | 0.14278 | K11380 | related to Peregrin |
| 9d00355 | 0.24869 | ni | flavin-containing monooxygenase | um12249 | 0.14281 | ni | putative protein |
| 6c00130 | 0.24885 | ni | aspartyl protease | um11406 | 0.14291 | ni | conserved hypothetical protein |
| 13c00098 | 0.24895 | ni | FOG, Predicted E3 ubiquitin ligase | um03805 | 0.14293 | ni | conserved hypothetical protein |
| 22d00226 | 0.24907 | ni | hypothetical protein | um04125 | 0.14321 | ni | related to YRO2 - strong similarity to HSP30 heat shock protein Yro1p |
| 20c00053 | 0.2492 | ni | iron/ascorbate family oxidoreductases | um01022 | 0.14333 | ni | conserved hypothetical protein |
| 25d00076 | 0.24929 | ni | mitochondrial tricarboxylate/dicarboxylate carrier proteins | um05834 | 0.14372 | K04532 | related to auxin-resistance protein |
| 20d00011 | 0.24935 | K12827 | splicing factor 3a, subunit 3 | um01556 | 0.1443 | ni | related to transcriptional activator Mut3p |
| 14c00114 | 0.2494 | K11246 | hypothetical protein | um10149 | 0.14431 | ni | related to CIRBP - glycine-rich RNA-binding protein |
| 10d00078 | 0.24945 | K13950 | para-aminobenzoate (PABA) synthase ABZ1 | um00263.2 | 0.14454 | ni | related to carnitine |
| 9d00157 | 0.24962 | ni | zuotin and related molecular chaperones | um10385 | 0.14462 | ni | probable 3-deoxy-D-arabino-heptulosonate 7-phosphate |
| 12c00113 | 0.24986 | K14191 | ribosomal RNA adenine dimethylase | um02683 | 0.14463 | ni | conserved hypothetical protein |
| 22c00274 | 0.24994 | ni | hypothetical protein | um03294 | 0.14469 | ni | hypothetical protein |
| 22d00087 | 0.25002 | ni | protein containing repeated kelch motifs | um03327 | 0.14471 | ni | conserved hypothetical Ustilago-specific protein |
| 12c00068 | 0.25002 | K03031 | 26S proteasome regulatory complex, subunit RPN12/PSMD8 | um05799 | 0.14482 | ni | hypothetical protein |
| 7c00022 | 0.25012 | ni | putative transmembrane protein | um10777 | 0.14502 | ni | conserved hypothetical protein |
| 7c00139 | 0.25027 | ni | ovarian tumor-like cysteine protease | um05739 | 0.14511 | ni | conserved hypothetical protein |
| 19c00141 | 0.25041 | ni | hypothetical protein | um10712 | 0.14522 | ni | conserved hypothetical protein |
| 4c00023 | 0.25044 | ni | uncharacterized membrane protein | um03539 | 0.14542 | ni | conserved hypothetical protein |
| 5d00088 | 0.2506 | K00830 | alanine-glyoxylate aminotransferase AGT1 | um11202 | 0.14554 | K02979 | probable 40s ribosomal protein s28 |
| 14d00058 | 0.25068 | K03671 | thioredoxin | um10351 | 0.14578 | K04082 | related to Co-chaperone protein HscB |
| 25c00037 | 0.25077 | ni | rho-gtpase activating protein | um03872 | 0.14583 | K12189 | related to VPS25 - vacuolar protein sorting |
| 6d00069 | 0.25083 | K00844 | hexokinase | um00123 | 0.14589 | ni | related to L-lactate dehydrogenase |
| 5c00010 | 0.25102 | K13953 | alcohol dehydrogenase | um10640 | 0.14589 | ni | conserved hypothetical protein |
| 7d00074 | 0.25106 | K11870 | ubiquitin-specific protease | um00855 | 0.14591 | K01881 | probable proline-tRNA ligase |
| 7d00096 | 0.25119 | ni | hypothetical protein | um02284 | 0.14594 | K11309 | conserved hypothetical protein |
| 10d00039 | 0.2512 | K03131 | transcription initiation factor TFIID, subunit TAF6 | um06053 | 0.14608 | ni | related to TAP42 - component of the Tor signaling pathway |
| 7d00330 | 0.25128 | ni | inositol polyphosphate multikinase, component of the ARGR trans | um03850 | 0.14615 | K00264 | probable glutamate synthase |
| 12c00092 | 0.25136 | ni | sexual differentiation process protein ISP4 | um06314 | 0.14623 | ni | related to MSS116 - RNA helicase of the DEAD box family |
| 7d00111 | 0.25167 | K02519 | mitochondrial translation initiation factor 2 | um11655 | 0.14645 | ni | conserved hypothetical protein |
| 16c00019 | 0.25179 | ni | proteins containing regions of low-complexity | um03288 | 0.14665 | ni | related to dna polymerase epsilon p17 subunit |
| 7c00114 | 0.25189 | ni | hypothetical protein | um03802 | 0.1468 | K03131 | related to TAF6 - Subunit |
| 6c00115 | 0.25198 | ni | cytosolic Ca2+-dependent cysteine protease (calpain), large subu | um11560 | 0.14683 | ni | putative protein |
| 18c00025 | 0.25202 | ni | hypothetical protein | um01454 | 0.14688 | ni | conserved hypothetical protein |
| 9c00156 | 0.25206 | ni | hypothetical protein | um05772 | 0.14692 | ni | hypothetical protein |
| 9c00219 | 0.25216 | ni | hypothetical protein | um03574 | 0.14716 | ni | putative protein |
| 9d00029 | 0.25226 | ni | uncharacterized protein | um04859 | 0.14737 | ni | related to PER1 protein |
| 12d00073 | 0.25236 | K17292 | beta-tubulin folding cofactor A | um01816 | 0.14743 | K14864 | related to TRM7 - tRNA 2 |
| 13c00017 | 0.25248 | K01469 | oxoprolinase | um10873 | 0.14755 | K12584 | conserved hypothetical protein |
| 27c00091 | 0.25249 | ni | hypothetical protein | um03317 | 0.14759 | ni | related to PRM1 - Pheromone-regulated multispanning membrane protein |
| 18d00075 | 0.25281 | ni | translation initiation factor 2C | um01334 | 0.14762 | ni | hypothetical protein |
| 1c00034 | 0.25294 | ni | animal-type fatty acid synthase and related proteins | um10784 | 0.14775 | K14262 | conserved hypothetical protein |
| 9d00162 | 0.25294 | ni | vesicle trafficking protein Sly1 | um10684 | 0.14798 | K14779 | conserved hypothetical protein |
| 11d00073 | 0.25298 | K01537 | Ca2+ transporting ATPase | um00622 | 0.14801 | K03061 | probable RPT1 - 26S proteasome regulatory subunit |
| 27d00034 | 0.25302 | ni | ubiquitin and ubiquitin-like proteins | um12141 | 0.14805 | ni | hypothetical protein |
| 25d00057 | 0.25304 | ni | hypothetical protein | um04592 | 0.14826 | ni | conserved hypothetical protein |
| 6d00129 | 0.25311 | K02728 | 20S proteasome | um11785 | 0.14833 | K03857 | conserved hypothetical protein |
| 9d00353 | 0.25326 | ni | hypothetical protein | um03472 | 0.14835 | ni | hypothetical protein |
| 9c00145 | 0.25331 | K17261 | centromere-associated protein HEC1 | um02077 | 0.14852 | K15053 | conserved hypothetical protein |
| 22d00135 | 0.25331 | ni | hypothetical protein | um04761 | 0.14858 | K11290 | related to SET protein |
| 16c00081 | 0.25343 | ni | hypothetical protein | um03154 | 0.14887 | ni | hypothetical protein |
| 15d00054 | 0.25345 | ni | hypothetical protein | um10604 | 0.1489 | K10610 | related to UV-damaged DNA-binding protein |
| 10c00071 | 0.25363 | K12585 | exosomal 3'-5' exoribonuclease complex, subunit Rrp44/Dis3 | um11110 | 0.14892 | ni | conserved hypothetical protein |
| 15d00035 | 0.25371 | ni | vacuolar assembly/sorting protein VPS9 | um06337 | 0.1494 | K03114 | wee1 kinase |
| 25c00025 | 0.25374 | ni | hypothetical protein | um04989 | 0.1495 | K10873 | related to RAD52 - recombination and DNA repair protein |
| 10c00027 | 0.25381 | K00889 | phosphatidylinositol-4-phosphate 5-kinase | um03301 | 0.14951 | K15458 | related to SAP155 - SIT4-associating protein |
| 18d00068 | 0.25381 | ni | hypothetical protein | um05470 | 0.14976 | K09554 | related to Hsp90 co-chaperone Cdc37 |
| 4d00030 | 0.25385 | K08876 | protein kinase | um00109 | 0.15006 | K00863 | probable DAK2 - dihydroxyacetone kinase |
| 19d00011 | 0.2541 | ni | hypothetical protein | um10770 | 0.15014 | K15109 | probable YMC1 - Protein of the mitochondrial carrier family |
| 16c00010 | 0.25419 | ni | predicted translation elongation factor | um01845 | 0.15016 | K07195 | related to Exocyst complex component Exo70 |
| 22c00189 | 0.25437 | ni | hypothetical protein | um11056 | 0.15021 | ni | conserved hypothetical protein |
| 20c00069 | 0.2546 | ni | hypothetical protein | um01966 | 0.15029 | K00232 | related to Acyl-coenzyme A oxidase |
| 22c00209 | 0.2546 | ni | hypothetical protein | um00475 | 0.15041 | ni | conserved hypothetical protein |
| 9d00255 | 0.25461 | K06883 | predicted GTPase | um00527 | 0.15068 | K03505 | related to DNA polymerase delta subunit 4 |
| 2c00001 | 0.25477 | K10627 | postreplication repair protein RAD18 | um11860 | 0.15086 | K04345 | cAMP-dependent protein kinase catalytic subunit |
| 19c00094 | 0.25487 | K14790 | FGF receptor activating protein 1 | um01725 | 0.15093 | ni | conserved hypothetical protein |
| 8d00115 | 0.25495 | K06978 | hypothetical protein | um04172.2 | 0.15103 | ni | putative protein |
| 6d00122 | 0.25499 | ni | hypothetical protein | um04714 | 0.15105 | K10669 | related to TPT1 - tRNA 2 |
| 10d00117 | 0.25499 | ni | hypothetical protein | um03330 | 0.15116 | ni | conserved hypothetical protein |
| 19d00150 | 0.25522 | ni | hypothetical protein | um11299 | 0.15138 | ni | putative protein |
| 9d00101 | 0.25534 | ni | hypothetical protein | um04163 | 0.15142 | ni | conserved hypothetical protein |
| 19d00165 | 0.25537 | ni | Zn2+-binding protein Melusin/RAR1 | um05373 | 0.15149 | ni | related to REG1 - regulatory subunit for protein phosphatase Glc7p |
| 7d00092 | 0.25541 | ni | hypothetical protein | um03517 | 0.15154 | ni | conserved hypothetical protein |
| 10c00013 | 0.25561 | K07874 | GTPase Rab1/YPT1 | um15000.2 | 0.15155 | ni | related to Vam6 |
| 13c00040 | 0.25562 | K15304 | ran-binding protein RANBP1 and related RanBD domain proteins | um11681 | 0.15172 | ni | conserved hypothetical protein |
| 27c00079 | 0.25568 | K01771 | hypothetical protein | um11138 | 0.15187 | ni | hypothetical protein |
| 27c00048 | 0.25572 | K10661 | protein involved in mRNA turnover and stability | um11430 | 0.15208 | ni | conserved hypothetical protein |
| 7d00075 | 0.25585 | K16912 | nuclear protein | um01328 | 0.15221 | K00030 | probable IDH2 - isocitrate dehydrogenase |
| 8d00062 | 0.25588 | K03164 | DNA topoisomerase type II | um02556 | 0.15226 | ni | conserved hypothetical protein |
| 18d00089 | 0.25592 | ni | hypothetical protein | um11261 | 0.15237 | K02962 | probable RPS17B - ribosomal protein S17 |
| 9d00236 | 0.25618 | ni | hypothetical protein | um11272 | 0.15238 | ni | conserved hypothetical protein |
| 9c00428 | 0.25636 | ni | phosphatidylserine-specific receptor PtdSerR | um06344 | 0.1525 | K01720 | probable 2-methylcitrate dehydratase |
| 24d00042 | 0.2564 | ni | hypothetical protein | um01267 | 0.15256 | ni | putative protein |
| 9d00305 | 0.25645 | K11365 | nuclear protein Ataxin-7 | um03796 | 0.15259 | K08832 | related to dis1-suppressing protein kinase dsk1 |
| 15d00064 | 0.25662 | K01322 | predicted serine protease | um03336 | 0.15261 | K13137 | related to UNR-interacting protein STRAP |
| 16d00027 | 0.25671 | K14285 | RNA export factor NXT1 | um05624 | 0.15262 | ni | putative protein |
| 5d00021 | 0.25671 | ni | hypothetical protein | um00990 | 0.15262 | ni | conserved hypothetical protein |
| 3c00064 | 0.25681 | K16261 | amino acid transporters | um00009 | 0.15266 | K07575 | related to TMA20 - Protein putative involved in cytoplasmic ribosome function |
| 25d00038 | 0.25683 | K00540 | hypothetical protein | um05261 | 0.15275 | ni | MAP kinase pathway-interacting protein |
| 9c00053 | 0.25685 | ni | hypothetical protein | um00288 | 0.1528 | K00566 | related to trna methyltransferase |
| 22d00190 | 0.25715 | K01265 | putative methionine aminopeptidase | um00146 | 0.1529 | ni | conserved hypothetical protein |
| 15d00070 | 0.25722 | ni | FOG, Ankyrin repeat | um02664 | 0.15293 | ni | related to zinc finger protein white collar 2 |
| 9d00158 | 0.25747 | ni | exocyst subunit - Sec10p | um03984 | 0.15305 | ni | ni |
| 3d00082 | 0.25757 | K02993 | 40S ribosomal protein S7 | um05850 | 0.15316 | ni | conserved hypothetical protein |
| 3d00060 | 0.25763 | ni | hypothetical protein | um00203 | 0.15339 | ni | conserved hypothetical protein |
| 20d00040 | 0.25772 | ni | hypothetical protein | um05860 | 0.15346 | K02736 | probable PRE4 - 20S proteasome subunit |
| 7d00323 | 0.25784 | K04348 | serine-threonine phosphatase 2B, catalytic subunit | um00322 | 0.15371 | ni | probable LRO1 - a lecithin cholesterol acyltransferase-like gene |
| 26d00011 | 0.25786 | K08294 | mitogen-activated protein kinase kinase | um10780 | 0.15375 | ni | conserved hypothetical protein |
| 9d00173 | 0.25792 | ni | bifunctional GTP cyclohydrolase II | um10456 | 0.15385 | ni | conserved hypothetical protein |
| 6d00100 | 0.25795 | K01613 | phosphatidylserine decarboxylase | um01888 | 0.15399 | K01288 | probable serine-type carboxypeptidase f precursor |
| 19d00014 | 0.25799 | K00844 | hexokinase | um10683 | 0.154 | K14779 | probable ROK1 - ATP-dependent RNA helicase |
| 26d00088 | 0.258 | ni | hypothetical protein | um15038 | 0.15406 | ni | conserved hypothetical protein |
| 25c00064 | 0.25819 | K11374 | RNA polymerase II elongator complex, subunit ELP2, WD repeat | um12140 | 0.15415 | ni | related to MRPL44 - mitochondrial ribosomal protein |
| 27d00001 | 0.25825 | K01755 | argininosuccinate lyase | um04532 | 0.15421 | ni | related to SNX41 - sorting nexin |
| 9d00145 | 0.25838 | K13345 | predicted E3 ubiquitin ligase | um01340 | 0.15427 | ni | conserved hypothetical protein |
| 8d00076 | 0.25853 | ni | hypothetical protein | um05560 | 0.1543 | ni | putative protein |
| 11c00002 | 0.25874 | ni | hypothetical protein | um10598 | 0.15443 | K00940 | probable nucleoside-diphosphate kinase |
| 13c00110 | 0.25879 | ni | hypothetical protein | um02504 | 0.15455 | ni | conserved hypothetical protein |
| 9c00070 | 0.25884 | ni | hypothetical protein | um00172 | 0.15456 | ni | related to meiotic recombination protein rec8 |
| 27d00009 | 0.25885 | K10405 | kinesin | um12086 | 0.15457 | K02838 | conserved hypothetical protein |
| 5c00085 | 0.25888 | ni | hypothetical protein | um11125 | 0.15473 | K13800 | probable URA6 - uridine-monophosphate kinase |
| 25c00074 | 0.259 | ni | hypothetical protein | um04116.2 | 0.15477 | ni | conserved hypothetical protein |
| 19c00032 | 0.25913 | ni | hypothetical protein | um01777 | 0.15485 | K09660 | related to mannose-P-dolichol utilization defect 1 protein |
| 27d00077 | 0.25926 | K13289 | serine carboxypeptidases | um02957 | 0.15492 | K06990 | conserved hypothetical protein |

| | | | | | | | |
|---|---|---|---|---|---|---|---|
| 2c00020 | 0.25932 | K08178 | hypothetical protein | um05033 | 0.15527 | ni | related to glucose |
| 8c00037 | 0.25937 | ni | hypothetical protein | um04184 | 0.15562 | ni | putative protein |
| 19c00025 | 0.25942 | ni | hypothetical protein | um06159 | 0.15564 | K01599 | probable HEM12 - uroporphyrinogen decarboxylase |
| 11d00023 | 0.25947 | ni | hypothetical protein | um03331 | 0.15578 | K15267 | putative protein |
| 26c00065 | 0.26018 | ni | hypothetical protein | um11099 | 0.15579 | K01874 | probable MES1 - methionyl-tRNA synthetase |
| 22c00056 | 0.26027 | K03030 | 26S proteasome regulatory complex, subunit RPN11 | um02111 | 0.15595 | ni | related to triacylglycerol lipase precursor |
| 19d00034 | 0.26042 | ni | hypothetical protein | um12210 | 0.15617 | K15275 | related to HUT1 - involved in UDP-galactose transport to the Golgi lumen |
| 18d00010 | 0.26046 | K03014 | RNA polymerase subunit K | um10178 | 0.15626 | ni | conserved hypothetical protein |
| 5d00027 | 0.26056 | ni | hypothetical protein | um01523 | 0.1564 | ni | conserved hypothetical protein |
| 12c00060 | 0.26063 | K11353 | NADH:ubiquinone oxidoreductase, B16.6 subunit | um05267 | 0.15649 | ni | putative protein |
| 27c00059 | 0.26073 | ni | hypothetical protein | um01999 | 0.15654 | ni | conserved hypothetical protein |
| 9c00299 | 0.26078 | ni | uncharacterized conserved protein | um04475 | 0.15663 | ni | conserved hypothetical protein |
| 6d00047 | 0.26096 | K08073 | polynucleotide kinase 3' phosphatase | um04657 | 0.15676 | ni | related to YTA7 - 26S proteasome subunit |
| 11c00009 | 0.26104 | ni | hypothetical protein | um04543 | 0.15688 | K08287 | related to Protein kinase lkh1 |
| 14d00083 | 0.26106 | ni | hypothetical protein | um06069 | 0.15689 | K15264 | conserved hypothetical protein |
| 6c00105 | 0.26113 | K00074 | 3-hydroxyacyl-CoA dehydrogenase | um11809 | 0.157 | ni | putative protein |
| 2c00053 | 0.26114 | K03007 | DNA-directed RNA polymerase, subunit RPB10 | um02821 | 0.15705 | ni | hypothetical protein |
| 22c00298 | 0.26116 | ni | hypothetical protein | um00861 | 0.15708 | ni | conserved hypothetical protein |
| 11c00055 | 0.26119 | K05546 | glucosidase II catalytic (alpha) subunit | um11127 | 0.1572 | K14168 | conserved hypothetical protein |
| 8d00042 | 0.26148 | K01950 | predicted NAD synthase | um10004 | 0.1573 | ni | related to a retinal short-chain dehydrogenase |
| 7c00017 | 0.26173 | K12877 | exon-exon junction complex, Magoh component | um01469 | 0.15768 | ni | conserved hypothetical protein |
| 24d00044 | 0.26188 | K14856 | protein required for actin cytoskeleton organization and cell cycle | um03261 | 0.15771 | ni | conserved hypothetical protein |
| 10d00069 | 0.26206 | ni | hypothetical protein | um10259 | 0.15784 | ni | conserved hypothetical protein |
| 13d00017 | 0.26215 | ni | rho-associated | um03323 | 0.15788 | K11968 | probable Ariadne-1 protein |
| 7d00357 | 0.2622 | ni | hypothetical protein | um04516.2 | 0.1583 | ni | conserved hypothetical protein |
| 3c00045 | 0.26231 | K01836 | phosphoglucomutase | um10855 | 0.15832 | K08294 | related to MKK1 - MAP kinase kinase |
| 6d00109 | 0.2624 | K10875 | DNA repair protein | um01552 | 0.15853 | ni | related to ATPase family protein |
| 14d00002 | 0.26252 | ni | predicted transporter | um04374 | 0.15865 | K00801 | farnesyl-diphosphate farnesyltransferase |
| 24c00026 | 0.26257 | ni | hypothetical protein | um11042 | 0.15873 | K13162 | related to PBP2 - PAB1 binding protein |
| 8c00077 | 0.26266 | K03687 | molecular chaperone of the GrpE family | um05794 | 0.1588 | ni | probable YCF1 - Vacuolar full-size ABC transporter |
| 14d00066 | 0.2628 | K02365 | regulator of spindle pole body duplication | um04641 | 0.15884 | ni | ni |
| 22c00092 | 0.26308 | ni | hypothetical protein | um11317 | 0.15892 | K11778 | related to RER2 - cis-prenyltransferase |
| 7c00002 | 0.26309 | ni | FOG, Zn-finger | um05715 | 0.15904 | ni | related to ACT1 - actin |
| 9c00228 | 0.26313 | ni | hypothetical protein | um10936 | 0.15913 | ni | related to RPC10 - DNA-directed RNA polymerases I |
| 19d00134 | 0.26314 | ni | hypothetical protein | um00445 | 0.15913 | ni | conserved hypothetical protein |
| 10c00035 | 0.26328 | K11274 | WD40 repeat protein | um04389 | 0.15923 | K06669 | probable SMC3 - required for structural maintenance of chromosomes |
| 7c00054 | 0.26331 | K00147 | gamma-glutamyl phosphate reductase | um02951 | 0.15939 | K10997 | related to TOF1 - topoisomerase I interacting factor 1 |
| 9d00189 | 0.26339 | ni | cytoskeletal protein Adducin | um10244 | 0.15949 | ni | conserved hypothetical protein |
| 14d00054 | 0.26347 | K01164 | ribonucleases P/MRP protein subunit | um02427 | 0.1595 | K03348 | related to negative regulator of mitosis |
| 9c00335 | 0.26372 | ni | hypothetical protein | um03572 | 0.15951 | ni | putative protein |
| 14c00113 | 0.26379 | ni | hypothetical protein | um01138 | 0.15953 | ni | conserved hypothetical protein |
| 19c00073 | 0.26386 | ni | hypothetical protein | um00867 | 0.15954 | K02957 | probable RPS22A - ribosomal protein S15a |
| 7c00347 | 0.26396 | K00574 | hypothetical protein | um06275 | 0.15977 | K12486 | conserved hypothetical protein |
| 22c00206 | 0.26409 | K16219 | hydroxyindole-o-methyltransferase | um11537 | 0.15978 | K02877 | probable RPL15A 60S large subunit ribosomal protein L15 |
| 19c00129 | 0.26412 | ni | predicted esterase | um12105 | 0.1598 | K02735 | probable PUP3 - 20S proteasome subunit beta3 |
| 15d00038 | 0.26418 | K04354 | serine/threonine protein phosphatase 2A, regulatory subunit | um06037 | 0.15982 | K11664 | conserved hypothetical protein |
| 22d00106 | 0.26421 | K07827 | ras-related GTPase | um01063 | 0.15983 | K06890 | conserved hypothetical protein |
| 10d00043 | 0.26439 | ni | hypothetical protein | um12125 | 0.15996 | ni | conserved hypothetical protein |
| 19c00147 | 0.26444 | K11269 | DNA replication checkpoint protein CHL12/CTF18 | um00674 | 0.15997 | K01624 | probable FBA1 - fructose-bisphosphate aldolase |
| 6d00131 | 0.26446 | ni | hypothetical protein | um05562 | 0.16 | ni | related to stress response protein rds1p |
| 5c00083 | 0.26461 | K04712 | fatty acid desaturase | um03871 | 0.16014 | ni | putative protein |
| 9d00219 | 0.26468 | ni | hypothetical protein | um00646 | 0.1602 | K00326 | related to cytochrome-b5 reductase |
| 12c00030 | 0.26502 | K11824 | vesicle coat complex AP-2, alpha subunit | um10379 | 0.16029 | K12603 | related to CCR4 - component of the major cytoplasmic deadenylase |
| 24c00014 | 0.26521 | ni | hypothetical protein | um03570 | 0.16042 | ni | conserved hypothetical protein |
| 18d00001 | 0.26536 | ni | hypothetical protein | um03132 | 0.16044 | ni | conserved hypothetical protein |
| 16d00033 | 0.26541 | ni | GTP-binding protein GP-1 | um11977 | 0.16073 | K15109 | conserved hypothetical protein |
| 26d00073 | 0.26551 | K00053 | hypothetical protein | um04850 | 0.16075 | ni | related to RRD1 - Resistant to Rapamycin Deletion |
| 22d00165 | 0.2656 | ni | hypothetical protein | um05194 | 0.16087 | ni | conserved hypothetical protein |
| 26c00047 | 0.26561 | ni | hypothetical protein | um11890 | 0.16101 | K13577 | probable DIC1 - Mitochondrial dicarboxylate carrier protein |
| 9d00079 | 0.26565 | ni | guanine nucleotide exchange factor | um06465 | 0.16106 | ni | putative protein |
| 6c00074 | 0.26569 | ni | hypothetical protein | um11828 | 0.16112 | K11404 | probable HOS2 - putative histone deacetylase |
| 12d00036 | 0.26572 | ni | hypothetical protein | um11155 | 0.16114 | ni | conserved hypothetical protein |
| 22c00294 | 0.26575 | ni | thioredoxin binding protein TBP-2/VDUP1 | um04219.2 | 0.16143 | ni | conserved hypothetical protein |
| 9d00266 | 0.26576 | K03063 | 26S proteasome regulatory complex | um00349.2 | 0.16154 | ni | hypothetical protein |
| 26c00041 | 0.26588 | ni | hypothetical protein | um02282 | 0.16171 | ni | conserved hypothetical protein |
| 9d00392 | 0.26589 | K04392 | ras-related small GTPase | um10082 | 0.16177 | K10427 | probable dynactin Arp1 p25 subunit RO12 |
| 26d00095 | 0.26591 | K01764 | holocytochrome c synthase | um10322 | 0.162 | ni | conserved hypothetical protein |
| 8c00093 | 0.26601 | ni | hypothetical protein | um00353 | 0.16216 | ni | conserved hypothetical protein |
| 4d00031 | 0.26656 | K02137 | mitochondrial F1F0-ATP synthase, subunit OSCP/ATP5 | um11973 | 0.16221 | ni | probable NIT2 - nitrilase |
| 19c00058 | 0.26662 | ni | voltage-gated shaker-like K+ channel, subunit beta/KCNAB | um00464 | 0.16236 | ni | putative protein |
| 27c00039 | 0.26676 | K10704 | ubiquitin-conjugating enzyme E2 | um15018 | 0.1624 | ni | putative protein |
| 27c00045 | 0.26709 | ni | hypothetical protein | um05693 | 0.16243 | K12462 | probable rho GDP dissociation inhibitor |
| 14d00087 | 0.2671 | K03138 | hypothetical protein | um00806 | 0.16249 | ni | conserved hypothetical protein |
| 8c00011 | 0.26724 | K08336 | protein conjugation factor | um11819 | 0.16267 | K04713 | related to SUR2 - sphingosine hydroxylase |
| 3c00078 | 0.26728 | ni | predicted FAD-dependent oxidoreductase | um03395 | 0.16271 | ni | hypothetical protein |
| 10d00115 | 0.26731 | ni | polyc-binding proteins alphaCP-1 and related KH domain proteins | um10022 | 0.16288 | ni | conserved hypothetical protein |
| 14c00064 | 0.26731 | K03033 | 26S proteasome regulatory complex, subunit RPN3/PSMD3 | um06055 | 0.16299 | K02941 | probable RPP0 - acidic ribosomal protein L10 |
| 14c00122 | 0.26734 | ni | FOG, Zn-finger | um11416 | 0.16314 | ni | conserved hypothetical Ustilago-specific protein |
| 26d00056 | 0.2674 | ni | hypothetical protein | um01206 | 0.16323 | ni | conserved hypothetical protein |
| 22d00137 | 0.26741 | ni | hypothetical protein | um05433 | 0.16335 | K00728 | probable PMT4 - dolichyl-phosphate-mannose--protein O-mannosyltransferase |
| 19c00125 | 0.26772 | K07390 | glutaredoxin-related protein | um02891 | 0.1634 | ni | conserved hypothetical protein |
| 9d00002 | 0.26793 | ni | hypothetical protein | um10225 | 0.16344 | ni | related to Glia maturation factor |
| 22d00007 | 0.26803 | ni | hypothetical protein | um11949 | 0.16346 | ni | ni |
| 7c00195 | 0.26829 | ni | hypothetical protein | um12120 | 0.16361 | ni | hypothetical protein |
| 9d00001 | 0.26859 | ni | hypothetical protein | um00660.2 | 0.16363 | K12391 | golgi adaptor HA1 |
| 9c00211 | 0.26873 | ni | hypothetical protein | um10879 | 0.16374 | K07127 | conserved hypothetical protein |
| 19d00060 | 0.26888 | K02955 | 40S ribosomal protein S14 | um03238 | 0.16401 | ni | conserved hypothetical protein |
| 9d00096 | 0.26903 | K01254 | bifunctional leukotriene A4 hydrolase | um01246 | 0.16406 | ni | conserved hypothetical protein |
| 10d00037 | 0.26907 | ni | uncharacterized conserved protein | um01716 | 0.16432 | ni | conserved hypothetical protein |
| 1d00015 | 0.26916 | ni | hypothetical protein | um05647 | 0.16435 | ni | related to cation diffusion facilitator 10 |
| 26c00023 | 0.26933 | ni | hypothetical protein | um03260 | 0.16468 | ni | conserved hypothetical protein |
| 3d00061 | 0.26933 | ni | uncharacterized conserved protein | um02431 | 0.16469 | ni | conserved hypothetical protein |
| 7c00020 | 0.2694 | K03544 | putative ATP-dependent Clp-type protease | um04314 | 0.16489 | K05765 | probable COF1 - cofilin |
| 19c00119 | 0.26945 | ni | hypothetical protein | um00131 | 0.1649 | ni | related to NADPH quinone oxidoreductase homolog PIG3 |
| 22c00251 | 0.26953 | K03596 | elongation factor-type GTP-binding protein | um02214.2 | 0.16491 | ni | putative protein |
| 9d00352 | 0.26955 | ni | hypothetical protein | um05839 | 0.16514 | ni | conserved hypothetical protein |
| 1d00024 | 0.26956 | K01876 | aspartyl-trna synthetase | um11485 | 0.16535 | ni | related to SNARE protein of Golgi compartment |
| 7d00252 | 0.26956 | ni | uncharacterized conserved protein | um03571 | 0.16535 | K00626 | acetoacetyl-CoA thiolase |
| 13d00083 | 0.26961 | ni | hypothetical protein | um11548 | 0.1654 | ni | putative protein |
| 6d00012 | 0.26976 | ni | predicted transporter | um06341 | 0.16563 | K13335 | related to Peroxisomal membrane protein PEX16 |
| 9c00379 | 0.26976 | ni | hypothetical protein | um04680 | 0.16567 | ni | related to Monocarboxylate transporter |
| 22c00161 | 0.26992 | ni | hypothetical protein | um04092 | 0.16581 | ni | conserved hypothetical protein |
| 9d00069 | 0.27002 | K14263 | hypothetical protein | um04131 | 0.16582 | K14764 | conserved hypothetical protein |
| 22d00258 | 0.27017 | ni | hypothetical protein | um01553 | 0.16594 | ni | conserved hypothetical protein |
| 18c00050 | 0.27029 | K01591 | uridine 5'- monophosphate synthase | um02392 | 0.16599 | ni | conserved hypothetical protein |
| 27d00027 | 0.27031 | K08287 | LAMMER dual specificity kinases | um03983 | 0.16609 | ni | related to SEC65 - signal recognition particle subunit |
| 25d00045 | 0.27033 | K00818 | acetylornithine aminotransferase | um04411 | 0.16615 | K12860 | related to CEF1 - required during G2 |
| 19c00012 | 0.27039 | ni | hsp90 co-chaperone CNS1 | um10543 | 0.16625 | ni | related to nonribosomal peptide synthetase |
| 1c00013 | 0.27062 | ni | hypothetical protein | um11627 | 0.16627 | ni | conserved hypothetical protein |
| 14c00066 | 0.27079 | ni | hypothetical protein | um02962 | 0.16628 | ni | conserved hypothetical protein |
| 8c00116 | 0.2708 | ni | hypothetical protein | um05255 | 0.16648 | ni | conserved hypothetical protein |
| 26d00067 | 0.2708 | ni | transcription factor MBF1 | um11076 | 0.16658 | ni | conserved hypothetical protein |
| 7c00160 | 0.27088 | K10573 | ubiquitin-protein ligase | um11987 | 0.16662 | ni | conserved hypothetical protein |
| 18d00061 | 0.27095 | K17053 | predicted methyltransferase | um06131 | 0.16672 | K10743 | related to 35 kDa ribonuclease H |
| 16d00069 | 0.27113 | K10365 | F-actin capping protein, beta subunit | um05986 | 0.16682 | K02729 | probable PUP2 - 20S proteasome subunit |
| 7c00123 | 0.27117 | K02141 | hypothetical protein | um04669 | 0.16687 | K10747 | related to DNA ligase I |
| 14c00039 | 0.27136 | ni | hypothetical protein | um04332 | 0.16696 | K09510 | related to DNAJ-like protein Psi |
| 11d00024 | 0.2714 | K14026 | extracellular protein SEL-1 and related proteins | um04625 | 0.16715 | K14821 | conserved hypothetical protein |
| 22d00110 | 0.27187 | ni | FOG, Zn-finger | um10960 | 0.16746 | ni | hypothetical protein |
| 1c00015 | 0.27205 | ni | hypothetical protein | um00908 | 0.16747 | ni | conserved hypothetical protein |
| 14c00134 | 0.27209 | ni | hydroxysteroid 17-beta dehydrogenase 11 | um06168 | 0.1676 | ni | putative protein |
| 2d00045 | 0.27219 | ni | heat shock transcription factor | um11846 | 0.16778 | ni | related to nucleoside hydrolase |
| 6c00031 | 0.2722 | K00065 | reductases with broad range of substrate specificities | um04214 | 0.16782 | K01591 | orotidine 5 |
| 5d00077 | 0.27228 | K03843 | glycosyltransferase | um10614 | 0.16793 | K14292 | conserved hypothetical protein |
| 14c00093 | 0.27234 | ni | extracellular protein SEL-1 and related proteins | um11030 | 0.16835 | ni | putative protein |
| 7d00337 | 0.2724 | ni | hypothetical protein | um04237 | 0.1684 | K00417 | probable ubiquinol--cytochrome-c reductase |
| 22d00008 | 0.27244 | ni | predicted protein tyrosine phosphatase | um05515 | 0.16845 | ni | hypothetical protein |
| 18d00052 | 0.27256 | ni | hypothetical protein | um06077 | 0.16859 | ni | hypothetical protein |
| 22c00003 | 0.27263 | K02540 | DNA replication licensing factor, MCM2 component | um04419 | 0.16875 | ni | related to CSR1 - phosphatidylinositol transfer protein |
| 9d00289 | 0.27269 | K09499 | chaperonin complex component, TCP-1 eta subunit | um10130 | 0.16877 | K01613 | related to regulator of nonsense transcripts 1 |
| 14c00022 | 0.27321 | K13137 | serine/threonine kinase receptor-associated protein | um06405 | 0.16882 | ni | related to AMME syndrome candidate gene 1 protein |
| 26c00012 | 0.27332 | K03240 | translation initiation factor 2B, epsilon subunit | um10671 | 0.16889 | ni | related to Pectin lyase B precursor |
| 13d00075 | 0.27337 | K07562 | NMD protein affecting ribosome stability and mRNA decay | um12131 | 0.16911 | ni | probable human translationally-controlled tumor protein |
| 7d00083 | 0.27338 | ni | hypothetical protein | um00438 | 0.16923 | ni | related to 50S ribosomal protein L35 |
| 19c00130 | 0.27338 | ni | predicted starch-binding protein | um10966 | 0.16927 | ni | related to JSN1 - RNA-binding protein |
| 20d00052 | 0.27354 | K12871 | hypothetical protein | um01330 | 0.16938 | ni | hypothetical protein |
| 9c00357 | 0.27362 | K03355 | anaphase-promoting complex (APC), Cdc23 subunit | um04208 | 0.16946 | ni | conserved hypothetical protein |
| 22c00143 | 0.27363 | ni | protein phosphatase 1, regulatory subunit, and related proteins | um02775 | 0.16964 | ni | hypothetical protein |
| 15c00027 | 0.27369 | ni | aconitase/homoaconitase | um05034 | 0.16983 | ni | related to Cephamycin export protein cmcT |
| 22c00084 | 0.2737 | ni | translin family protein | um05444 | 0.1699 | ni | hypothetical protein |
| 25d00065 | 0.27379 | ni | predicted dinucleotide-utilizing enzyme | um10155 | 0.16996 | ni | putative protein |
| 7c00101 | 0.27382 | K13343 | peroxisomal membrane anchor protein | um11944 | 0.17006 | K00002 | probable GCY1 - galactose-induced protein of aldo |
| 8c00118 | 0.27396 | K01181 | hypothetical protein | um05210 | 0.17006 | K02307 | conserved hypothetical protein |
| 6d00086 | 0.27397 | ni | hypothetical protein | um10882 | 0.17048 | K09567 | probable peptidyl-prolyl cis-trans isomerase |
| 12c00003 | 0.27397 | ni | hypothetical protein | um03341 | 0.1707 | ni | conserved hypothetical protein |
| 3d00055 | 0.27398 | ni | hypothetical protein | um04835 | 0.17072 | ni | related to WHI3 - involved in regulation of cell size |
| 7c00121 | 0.27399 | K04441 | mitogen-activated protein kinase | um04755 | 0.17075 | ni | related to calcium |
| 9c00336 | 0.27437 | K10772 | apurinic/apyrimidinic endonuclease | um10071 | 0.17111 | ni | conserved hypothetical protein |
| 8c00043 | 0.27462 | ni | hypothetical protein | um11786 | 0.17124 | ni | related to YIP1 - Golgi integral membrane protein |
| 13d00067 | 0.27465 | ni | predicted protein tyrosine phosphatase | um11740.2 | 0.17126 | ni | conserved hypothetical protein |
| 19c00003 | 0.27476 | ni | hypothetical protein | um03033 | 0.17145 | K12486 | conserved hypothetical protein |
| 8d00018 | 0.27485 | K00227 | sterol C5 desaturase | um02136 | 0.17156 | ni | effector family protein Eff1-6 |
| 25d00031 | 0.27494 | ni | hypothetical protein | um10021 | 0.17159 | K00761 | probable uracil phosphoribosyltransferase |
| 9d00331 | 0.27513 | ni | hypothetical protein | um10496 | 0.17162 | K08838 | related to KIC1 - ser |
| 9d00226 | 0.27522 | K12597 | E3 ubiquitin ligase interacting with arginine methyltransferase | um02390 | 0.1717 | K16055 | related to alpha |
| 7c00052 | 0.27533 | ni | glycogen synthase kinase-3 | um12066 | 0.17174 | ni | hypothetical protein |

| | | | | | | | |
|---|---|---|---|---|---|---|---|
| 26c00050 | 0.27542 | K01783 | D-ribulose-5-phosphate 3-epimerase | um03241 | 0.17197 | K06961 | probable KRR1 - required for 40S ribosome biogenesis |
| 15d00034 | 0.27556 | ni | copper transporter | um02482 | 0.17205 | ni | conserved hypothetical protein |
| 19d00152 | 0.27557 | K01128 | sphingomyelinase family protein | um02613 | 0.17207 | K01883 | probable cysteinyl-tRNA synthetase |
| 27c00031 | 0.27572 | ni | growth hormone-induced protein and related proteins | um03841 | 0.17224 | K08852 | related to IRE1 - protein kinase |
| 27c00090 | 0.27588 | K03593 | predicted ATPase | um06503 | 0.1725 | ni | conserved hypothetical protein |
| 20c00076 | 0.27592 | ni | hypothetical protein | um06360 | 0.17252 | K12845 | probable SNU13 - component of the U4 |
| 14d00006 | 0.27596 | ni | hypothetical protein | um02718 | 0.17257 | K15255 | related to PIF1 - DNA helicase involved in mitochondrial DNA repair and telomere length control |
| 22d00181 | 0.27628 | ni | hypothetical protein | um03305 | 0.1727 | K04371 | MAP kinase |
| 22c00201 | 0.27646 | ni | predicted mutarotase | um00775 | 0.17289 | K10581 | conserved hypothetical protein |
| 22c00029 | 0.27665 | K11599 | hypothetical protein | um10267 | 0.1729 | ni | conserved hypothetical protein |
| 7c00166 | 0.27671 | K03232 | elongation factor 1 beta/delta chain | um05109 | 0.17296 | ni | related to ECM14 - involved in cell wall biogenesis and architecture |
| 10d00049 | 0.27683 | ni | uncharacterized conserved protein | um04678 | 0.17329 | ni | conserved hypothetical protein |
| 26d00099 | 0.27684 | ni | amino acid transporters | um10950 | 0.1734 | ni | hypothetical protein |
| 6d00058 | 0.27687 | ni | hypothetical protein | um11188 | 0.17348 | ni | conserved hypothetical Ustilago-specific protein |
| 26c00039 | 0.27699 | ni | hypothetical protein | um12284 | 0.17349 | K12883 | probable nuclear cap binding protein subunit 2 |
| 22d00134 | 0.27705 | ni | predicted hydrolase related to dienelactone hydrolase | um11335 | 0.17365 | ni | hypothetical protein |
| 14d00112 | 0.27713 | ni | extracellular protein SEL-1 and related proteins | um02877 | 0.17385 | ni | conserved hypothetical protein |
| 14d00057 | 0.27733 | ni | hypothetical protein | um10352 | 0.17388 | ni | conserved hypothetical protein |
| 27c00062 | 0.27735 | K03094 | SCF ubiquitin ligase, Skp1 component | um06326 | 0.174 | ni | conserved hypothetical protein |
| 11c00031 | 0.27747 | ni | uridine permease | um02603 | 0.17403 | ni | related to Myosin heavy chain |
| 9d00081 | 0.27755 | ni | hypothetical protein | um02163 | 0.17403 | ni | hypothetical protein |
| 22d00204 | 0.27763 | ni | hypothetical protein | um04164 | 0.17414 | ni | conserved hypothetical protein |
| 9c00375 | 0.27782 | K11131 | pseudouridine synthase | um03550 | 0.17433 | K03011 | related to RPB3 - DNA-directed RNA polymerase II chain |
| 5c00156 | 0.27783 | K02974 | 40S ribosomal protein S24 | um05807 | 0.17453 | ni | related to KRE6 - glucan synthase subunit |
| 25d00011 | 0.27792 | K03965 | hypothetical protein | um05814 | 0.17461 | ni | conserved hypothetical protein |
| 4c00026 | 0.27794 | K15156 | thyroid hormone receptor-associated coactivator complex compor | um05204 | 0.17463 | K01112 | conserved hypothetical protein |
| 5c00135 | 0.2781 | ni | hypothetical protein | um02576 | 0.17466 | ni | related to Sterol regulatory element binding protein cleavage-activating protein |
| 6c00005 | 0.27813 | K08141 | predicted transporter | um01010 | 0.17479 | ni | putative protein |
| 9d00327 | 0.27828 | K11404 | histone deacetylase complex, catalytic component RPD3 | um05319 | 0.17486 | ni | conserved hypothetical Ustilago-specific protein |
| 26d00052 | 0.27833 | K01620 | threonine aldolase | um00980 | 0.17492 | ni | conserved hypothetical protein |
| 22c00148 | 0.27846 | ni | hypothetical protein | um01093 | 0.17519 | K11322 | related to EPL1 - Component of histone H4 |
| 7c00079 | 0.27862 | ni | hypothetical protein | um04069 | 0.17521 | K10843 | probable SSL2 - DNA helicase |
| 2c00010 | 0.27862 | K03163 | DNA topoisomerase I | um10539 | 0.17529 | K00804 | related to farnesyltranstransferase |
| 8d00067 | 0.27871 | K02216 | checkpoint kinase | um02665 | 0.17541 | K03113 | probable translation initiation factor SUI1 |
| 25d00093 | 0.27872 | K08141 | predicted transporter | um04958 | 0.17545 | ni | hypothetical protein |
| 22d00071 | 0.27887 | K07556 | F1-ATP synthase assembly protein | um02251 | 0.1755 | ni | conserved hypothetical Ustilago-specific protein |
| 25d00009 | 0.27898 | ni | hypothetical protein | um04120 | 0.17554 | ni | hypothetical protein |
| 19c00091 | 0.27898 | K11270 | hypothetical protein | um05627 | 0.17564 | K07101 | related to XPT1 - xanthine phosphoribosyl transferase |
| 7d00034 | 0.27924 | K02736 | 20S proteasome, regulatory subunit beta type PSMB4/PRE4 | um03520 | 0.17576 | K11501 | conserved hypothetical protein |
| 11d00054 | 0.27934 | ni | Na+/K+ transporter | um00298 | 0.17596 | K11836 | related to ubiquitin-specific processing protease |
| 18c00101 | 0.27937 | ni | hypothetical protein | um04518 | 0.17618 | K10590 | related to UFD4 - Ubiquitin-protein ligase |
| 11c00003 | 0.27948 | ni | hypothetical protein | um02407 | 0.17627 | K15634 | related to thymidylate synthase |
| 19d00112 | 0.27955 | ni | hypothetical protein | um01958.2 | 0.17632 | ni | putative protein |
| 19d00052 | 0.27966 | ni | mismatch repair ATPase MSH2 | um11614 | 0.17635 | ni | related to TOM5 - mitochondrial outer membrane protein |
| 2c00032 | 0.27984 | K10884 | DNA-binding subunit of a DNA-dependent protein kinase | um04579 | 0.17641 | ni | related to TRX2 - thioredoxin II |
| 18c00032 | 0.27985 | K03236 | translation initiation factor 1A | um11825 | 0.17688 | ni | conserved hypothetical protein |
| 14c00074 | 0.28003 | K11319 | chromatin remodeling protein | um11313 | 0.17704 | K03650 | related to MSS1 - mitochondrial GTPase involved in expression of COX1 |
| 27c00020 | 0.28011 | ni | hypothetical protein | um04087 | 0.17711 | ni | hypothetical protein |
| 6d00064 | 0.28014 | ni | hypothetical protein | um05224 | 0.17731 | ni | related to Muskelin |
| 22c00059 | 0.28029 | ni | predicted protein carboxyl methylase | um12322 | 0.17742 | ni | hypothetical protein |
| 5c00069 | 0.2803 | ni | hypothetical protein | um10646 | 0.17743 | K14686 | related to CTR2 - Protein involved in copper transport |
| 26d00046 | 0.28033 | K02265 | cytochrome c oxidase, subunit Vb/COX4 | um10806 | 0.1777 | ni | conserved hypothetical protein |
| 7d00071 | 0.28047 | K01304 | hypothetical protein | um11360 | 0.17772 | K16732 | conserved hypothetical protein |
| 7c00179 | 0.28054 | ni | pattern-formation protein | um01443 | 0.17782 | ni | conserved hypothetical Ustilago-specific protein |
| 11d00064 | 0.28057 | ni | nuclear polyadenylated RNA binding protein | um10680 | 0.17808 | ni | conserved hypothetical protein |
| 18d00046 | 0.28071 | ni | hypothetical protein | um10093 | 0.17815 | ni | putative protein |
| 8c00026 | 0.28071 | ni | hypothetical protein | um05494 | 0.17822 | ni | related to secretory pathway protein |
| 24c00044 | 0.28073 | ni | hypothetical protein | um06184 | 0.17826 | K01881 | related to prolyl-tRNA synthetase |
| 9d00260 | 0.28076 | K09705 | hypothetical protein | um04186 | 0.17828 | ni | ni |
| 9d00066 | 0.28081 | ni | hypothetical protein | um00031 | 0.17835 | ni | conserved hypothetical protein |
| 5c00009 | 0.28089 | ni | hypothetical protein | um00160 | 0.1785 | K15296 | probable SEC17 - transport vesicle fusion protein |
| 15d00083 | 0.28115 | ni | hypothetical protein | um10800 | 0.17854 | ni | conserved hypothetical protein |
| 22d00253 | 0.28122 | ni | hypothetical protein | um05218 | 0.17882 | ni | conserved hypothetical protein |
| 26c00027 | 0.28125 | ni | hypothetical protein | um11253 | 0.17883 | ni | conserved hypothetical protein |
| 22c00276 | 0.28134 | ni | hypothetical protein | um02206 | 0.179 | ni | conserved hypothetical protein |
| 9d00344 | 0.28136 | K14843 | protein required for normal rRNA processing | um06269 | 0.17912 | ni | conserved hypothetical protein |
| 5c00129 | 0.2814 | ni | putative cargo transport protein EMP24 | um04158 | 0.1792 | K14794 | related to RRP12 - Protein required for normal pre-rRNA Processing |
| 20d00063 | 0.28142 | ni | hypothetical protein | um10256 | 0.17929 | ni | conserved hypothetical protein |
| 9c00434 | 0.28147 | K11788 | glycinamide ribonucleotide synthetase | um02589 | 0.17941 | ni | hypothetical protein |
| 10d00030 | 0.28152 | ni | hypothetical protein | um02022 | 0.17951 | ni | conserved hypothetical protein |
| 3c00035 | 0.28155 | ni | hypothetical protein | um12035 | 0.17998 | ni | putative protein |
| 13c00074 | 0.28157 | ni | tRNA nucleotidyltransferase | um00347 | 0.18006 | ni | related to Tubulin-folding cofactor D |
| 11d00055 | 0.28163 | ni | predicted membrane protein | um06181 | 0.18008 | ni | Mig2-5 |
| 15d00029 | 0.28163 | K02132 | F0F1-type ATP synthase, alpha subunit | um02592 | 0.18032 | ni | related to 2-hydroxy-3-oxopropionate reductase |
| 19c00022 | 0.28183 | K03130 | transcription initiation factor TFIID, subunit TAF5 | um10766 | 0.18036 | K15448 | conserved hypothetical protein |
| 26c00086 | 0.28188 | ni | predicted dehydrogenase | um01074 | 0.18037 | ni | conserved hypothetical protein |
| 26d00028 | 0.28214 | K15028 | hypothetical protein | um10110 | 0.18048 | K02920 | probable 60S ribosomal protein L36 |
| 18d00008 | 0.28236 | ni | hypothetical protein | um11283 | 0.18051 | ni | related to YSC84 - protein involved in the organization of the actin cytoskeleton |
| 12c00095 | 0.28242 | K01885 | glutamyl-trna synthetase | um05919 | 0.18062 | K00147 | probable PRO2 - gamma-glutamyl phosphate reductase |
| 7d00067 | 0.28256 | K14997 | amino acid transporter protein | um04053 | 0.18074 | K01739 | probable STR2 - Cystathionine gamma-synthase |
| 8c00115 | 0.28267 | K14834 | protein involved in the nuclear export of pre-ribosomes | um06075 | 0.18087 | ni | related to beta-glucosidase |
| 13c00055 | 0.28284 | ni | phosphatidylinositol transfer protein PDR16 and related proteins | um03557 | 0.1811 | ni | related to nitrogen assimilation transcription factor |
| 1d00020 | 0.28294 | K03386 | alkyl hydroperoxide reductase | um11804 | 0.1811 | ni | related to PAN1 - actin-cytoskeleton assembly protein |
| 9d00304 | 0.28296 | K01409 | predicted metalloprotease with chaperone activity | um01660 | 0.18114 | ni | putative protein |
| 12d00139 | 0.28306 | ni | ni | um03440 | 0.18151 | ni | conserved hypothetical Ustilago-specific protein |
| 7d00328 | 0.28312 | K11600 | exosomal 3'-5' exoribonuclease complex, subunit Rrp41 | um02342 | 0.18162 | ni | hypothetical protein |
| 9d00412 | 0.28312 | ni | 50S ribosomal protein L1 | um01709 | 0.18164 | ni | conserved hypothetical protein |
| 10d00123 | 0.28313 | ni | Fe2+/Zn2+ regulated transporter | um10282 | 0.1819 | ni | putative protein |
| 26c00084 | 0.28328 | K09650 | integral membrane protease of the rhomboid family | um04185 | 0.18196 | ni | conserved hypothetical protein |
| 4c00014 | 0.2833 | K09313 | transcription factor | um10841 | 0.18201 | K02900 | probable RPL28 - 60S large subunit ribosomal protein L27a |
| 9d00378 | 0.28339 | K07342 | preprotein translocase, gamma subunit | um10727 | 0.18205 | K06672 | related to proline-rich protein required for meiotic chromosome condensation and synapsis |
| 2c00060 | 0.28349 | ni | hypothetical protein | um05416 | 0.18209 | K06173 | related to PUS1 - pseudouridine synthase 1 |
| 20d00081 | 0.28371 | K02880 | 60S ribosomal protein L22 | um04764 | 0.18221 | ni | putative protein |
| 9c00193 | 0.28377 | ni | hypothetical protein | um11571 | 0.18226 | K08744 | related to CRD1 - cardiolipin synthase |
| 20d00057 | 0.28396 | K04718 | sphingosine kinase | um06128 | 0.18227 | ni | conserved hypothetical Ustilago-specific protein |
| 22c00047 | 0.28396 | ni | hypothetical protein | um01774 | 0.18229 | ni | conserved hypothetical protein |
| 11d00028 | 0.284 | K14786 | KRR1-interacting protein involved in 40S ribosome biogenesis | um03575 | 0.18237 | ni | putative protein |
| 14c00087 | 0.28415 | ni | hypothetical protein | um03406 | 0.18238 | ni | hypothetical protein |
| 10d00084 | 0.28421 | ni | hypothetical protein | um11496 | 0.18238 | K15437 | related to Tyrosyl-tRNA synthetase |
| 22c00061 | 0.28431 | ni | hypothetical protein | um11882 | 0.1825 | K12733 | probable Peptidyl-prolyl cis-trans isomerase |
| 22d00189 | 0.28444 | K00059 | mitochondrial/plastidial beta-ketoacyl-ACP reductase | um15057 | 0.18266 | K02516 | related to protein arginine N-methyltransferase |
| 26d00038 | 0.28448 | ni | uncharacterized conserved protein | um10663 | 0.18275 | ni | probable RHO1 - GTP-binding protein of the rho subfamily of ras-like proteins |
| 26c00085 | 0.28448 | K01569 | hypothetical protein | um04206 | 0.1829 | ni | conserved hypothetical protein |
| 12d00023 | 0.28465 | ni | hypothetical protein | um02400 | 0.18341 | ni | conserved hypothetical protein |
| 11d00007 | 0.28465 | K03246 | translation initiation factor 3, subunit i/TGF-beta receptor-interacti | um05437 | 0.18363 | ni | conserved hypothetical protein |
| 26c00079 | 0.2847 | ni | hypothetical protein | um05633 | 0.18368 | ni | conserved hypothetical protein |
| 2d00065 | 0.28475 | ni | endoplasmic reticulum membrane-associated oxidoreductin | um06505 | 0.18399 | ni | conserved hypothetical Ustilago-specific protein |
| 1d00028 | 0.28481 | ni | hypothetical protein | um06345 | 0.18404 | K01738 | probable cysteine synthase |
| 21d00002 | 0.28483 | ni | serine-threonine protein kinase FUSED | um00460 | 0.18414 | K01149 | related to exosome complex exonuclease |
| 12d00048 | 0.28499 | K00030 | isocitrate dehydrogenase, alpha subunit | um12293 | 0.18425 | K00599 | conserved hypothetical protein |
| 6c00029 | 0.28508 | ni | hypothetical protein | um04770 | 0.18428 | ni | related to splicing factor SPF30 |
| 22d00136 | 0.28534 | ni | flavin-containing monooxygenase | um02004 | 0.18434 | ni | conserved hypothetical protein |
| 3d00008 | 0.28538 | K10295 | F-box protein FBX9 | um10959 | 0.18456 | ni | putative protein |
| 27d00013 | 0.28553 | ni | hypothetical protein | um05820 | 0.18465 | ni | hypothetical protein |
| 19d00062 | 0.28564 | K15262 | isoamyl acetate-hydrolyzing esterase and related enzymes | um00152 | 0.18466 | ni | related to tandem ph domain-containing protein-2 |
| 15d00088 | 0.28571 | ni | transcription factor | um03530 | 0.1847 | ni | related to PAM16 - Presequence translocase-Asssociated Motor |
| 14c00010 | 0.28573 | ni | hypothetical protein | um11347.2 | 0.18524 | K05284 | related to GPI14 - Glycosylphosphatidylinositol-alpha 1 |
| 5d00112 | 0.28604 | ni | cyclin B and related kinase-activating proteins | um11487 | 0.18538 | K14801 | conserved hypothetical protein |
| 8c00019 | 0.28614 | ni | sideroflexin | um05269 | 0.18543 | K16261 | probable general amino acid permease |
| 25c00079 | 0.28618 | ni | hypothetical protein | um00415 | 0.18573 | K07023 | conserved hypothetical protein |
| 19d00017 | 0.2863 | ni | hypothetical protein | um00805 | 0.18576 | ni | putative protein |
| 22c00199 | 0.28643 | ni | hypothetical protein | um15031 | 0.18591 | K01870 | probable ISM1 - isoleucine-tRNA ligase |
| 22c00321 | 0.28644 | ni | hypothetical protein | um02527 | 0.18599 | K05841 | UDP-glucose |
| 24d00045 | 0.28646 | K02730 | 20S proteasome, regulatory subunit alpha type PSMA6/SCL1 | um10027 | 0.18618 | K10599 | probable PRP19 - non-snRNP spliceosome component required for DNA repair |
| 16c00051 | 0.28648 | K02739 | 20S proteasome, regulatory subunit beta type PSMB7/PSMB10/F | um10147 | 0.1863 | K02870 | probable 60S ribosomal protein L12 |
| 15c00020 | 0.28675 | K15072 | hypothetical protein | um11431 | 0.1864 | ni | putative protein |
| 3c00032 | 0.2868 | ni | hypothetical protein | um04588 | 0.18647 | K02977 | probable ubiquitin |
| 8c00006 | 0.28683 | K14765 | hypothetical protein | um15067 | 0.18653 | ni | conserved hypothetical protein |
| 3c00021 | 0.28703 | ni | hypothetical protein | um05748 | 0.18664 | ni | conserved hypothetical protein |
| 19d00108 | 0.28705 | K01112 | predicted haloacid-halidohydrolase and related hydrolases | um12046 | 0.18701 | ni | related to Tctex2-related inner arm dynein light chain |
| 10d00006 | 0.28706 | K01113 | hypothetical protein | um04477 | 0.18701 | ni | conserved hypothetical protein |
| 22c00046 | 0.28726 | K01465 | dihydroorotase | um06088 | 0.18708 | K03846 | related to ALG9 - mannosyltransferase |
| 10d00038 | 0.28731 | K10389 | gamma tubulin | um11582 | 0.1871 | ni | conserved hypothetical protein |
| 13c00087 | 0.28745 | K12343 | steroid reductase | um06431 | 0.18733 | ni | conserved hypothetical protein |
| 2d00006 | 0.28748 | ni | hypothetical protein | um02912 | 0.18741 | ni | conserved hypothetical protein |
| 1d00044 | 0.28752 | ni | hypothetical protein | um02793 | 0.18743 | K07019 | conserved hypothetical protein |
| 18d00004 | 0.28755 | K03234 | ni | um10402.2 | 0.1875 | K09699 | related to lipoamide acyltransferase component of branched-chain alpha-keto acid dehydrogenase complex |
| 26c00007 | 0.2876 | K02737 | 20S proteasome, regulatory subunit beta type PSMB5/PSMB8/PF | um10989 | 0.18756 | ni | related to NADH-ubiquinone oxidoreductase 21 kDa subunit |
| 13d00104 | 0.28765 | ni | hypothetical protein | um06045 | 0.18764 | K01427 | probable urease |
| 5c00151 | 0.2878 | K03537 | RNase P/RNase MRP subunit POP5 | um06267 | 0.18769 | ni | hypothetical protein |
| 1d00043 | 0.28782 | ni | predicted transporter | um10187 | 0.1879 | ni | putative protein |
| 12d00122 | 0.28787 | ni | medium subunit of clathrin adaptor complex | um04628 | 0.18815 | ni | conserved hypothetical protein |
| 25c00035 | 0.28789 | K12386 | cystine transporter Cystinosin | um11167 | 0.1882 | ni | probable TIM17 - mitochondrial inner membrane import translocase subunit |
| 19c00033 | 0.28799 | K15331 | tRNA uracil-5-methyltransferase and related tRNA-modifying enz | um01668 | 0.18828 | ni | related to TGF beta receptor associated protein-1 |
| 6c00062 | 0.28802 | ni | uncharacterized conserved protein | um10487 | 0.18832 | K10580 | probable ubiquitin-conjugating enzyme E2 |
| 18d00106 | 0.28819 | ni | hypothetical protein | um03759 | 0.1884 | K09613 | probable COP9 signalosome subunit 5 CSN5 |
| 3d00056 | 0.28837 | ni | predicted membrane protein | um10401 | 0.18851 | K11273 | related to CHL1 - protein of the DEAH box family |
| 18c00104 | 0.28863 | ni | hypothetical protein | um05609 | 0.18863 | ni | putative protein |
| 9c00164 | 0.28865 | K14791 | WD40 repeat-containing protein | um04887 | 0.18868 | ni | conserved hypothetical Ustilago-specific protein |
| 16d00012 | 0.28896 | K03254 | translation initiation factor 3, subunit a | um03717 | 0.18897 | ni | conserved hypothetical protein |
| 7d00108 | 0.2891 | ni | hypothetical protein | um10794 | 0.18917 | ni | related to GGA2 - Arf-binding protein |
| 4d00038 | 0.28914 | K00486 | kynurenine 3-monooxygenase and related flavoprotein monooxyge | um00429 | 0.1892 | K00697 | probable TPS1 - alpha |
| 12c00040 | 0.2892 | ni | hypothetical protein | um12161 | 0.18932 | ni | related to lipase family |
| 3d00078 | 0.28931 | K02865 | 60S ribosomal protein L10A | um04855 | 0.18942 | K02898 | probable 60S ribosomal protein L26 |
| 11d00079 | 0.28937 | ni | hypothetical protein | um02598 | 0.18954 | ni | related to mfs1 - putative multidrug transporter |
| 9c00367 | 0.2894 | ni | predicted integral membrane protein | um11322 | 0.18966 | ni | probable beta-1 |

| | | | | | | | |
|---|---|---|---|---|---|---|---|
| 10d00020 | 0.28949 | K01942 | biotin holocarboxylase synthetase | um10044 | 0.19002 | K01254 | related to leukotriene-A4 hydrolase |
| 8d00027 | 0.28973 | ni | hypothetical protein | um01667 | 0.19003 | ni | conserved hypothetical protein |
| 16c00013 | 0.29 | K12829 | splicing factor 3b, subunit 2 | um10889 | 0.19025 | K15071 | conserved hypothetical protein |
| 9d00385 | 0.29029 | K11126 | telomerase catalytic subunit | um00254 | 0.19035 | ni | conserved hypothetical protein |
| 22d00056 | 0.29035 | ni | hypothetical protein | um10979 | 0.19038 | K09680 | related to pantothenate kinase |
| 15d00015 | 0.2905 | K15178 | paf1/rna polymerase II complex, RTF1 component | um10000 | 0.19041 | ni | conserved hypothetical protein |
| 22d00164 | 0.29063 | K01515 | nucleoside diphosphate-sugar hydrolase of the MutT | um12172 | 0.19089 | ni | conserved hypothetical protein |
| 9c00279 | 0.29067 | ni | uncharacterized conserved protein | um11486 | 0.19114 | ni | putative protein |
| 7c00066 | 0.29069 | ni | predicted sugar phosphatase | um10108 | 0.19119 | ni | conserved hypothetical protein |
| 9c00248 | 0.29101 | ni | hypothetical protein | um00114 | 0.19143 | K11313 | related to SPT3 - general transcriptional adaptor or co-activator |
| 5c00127 | 0.29119 | ni | mitochondrial import inner membrane translocase, subunit TIM22 | um11470 | 0.19156 | ni | conserved hypothetical protein |
| 26d00022 | 0.29122 | K01868 | threonyl-trna synthetase | um03583 | 0.19158 | K04486 | related to HIS2 - histidinol-phosphatase |
| 8d00090 | 0.29163 | ni | hypothetical protein | um04488 | 0.1916 | ni | related to MRP7 - mitochondrial ribosomal protein |
| 9d00262 | 0.29166 | ni | hypothetical protein | um01605 | 0.19167 | K06662 | related to cell cycle checkpoint protein RAD17 |
| 9d00297 | 0.29173 | K03849 | glucosyltransferase - Alg8p | um11774 | 0.19189 | ni | conserved hypothetical protein |
| 6d00101 | 0.29187 | ni | hypothetical protein | um02961 | 0.19198 | K11315 | conserved hypothetical protein |
| 7c00346 | 0.29204 | K12178 | COP9 signalosome, subunit CSN4 | um05999 | 0.192 | ni | putative protein |
| 6c00134 | 0.29214 | ni | hypothetical protein | um06376 | 0.19208 | K07953 | probable GTP-binding protein SAR1 |
| 25d00012 | 0.29217 | ni | vesicle coat complex COPI, zeta subunit | um01564 | 0.19219 | ni | related to integral peroxisomal membrane protein |
| 3c00059 | 0.29256 | ni | p21-activated serine/threonine protein kinase | um01875 | 0.19239 | K00965 | probable GAL7 - UDP-glucose--hexose-1-phosphate uridylyltransferase |
| 16c00044 | 0.29264 | K04082 | mitochondrial J-type chaperone | um04948 | 0.19251 | K03676 | probable GRX1 - glutaredoxin |
| 15d00028 | 0.29264 | ni | hypothetical protein | um10162 | 0.1926 | ni | related to Transformer-2 protein homolog |
| 20d00045 | 0.29265 | ni | aldo/keto reductase family proteins | um01103 | 0.19269 | K02134 | probable ATP synthase delta chain precursor |
| 15c00077 | 0.29268 | K14951 | cation transport ATPase | um11088 | 0.19279 | ni | conserved hypothetical protein |
| 25d00090 | 0.29304 | ni | hypothetical protein | um06417 | 0.19284 | K04564 | related to Superoxide dismutase |
| 5c00054 | 0.29332 | ni | hypothetical protein | um05929 | 0.19295 | ni | conserved hypothetical Ustilago-specific protein |
| 3c00065 | 0.29374 | ni | hypothetical protein | um12205 | 0.19302 | ni | hypothetical protein |
| 7c00149 | 0.29394 | K06883 | putative transcription factor FET5 | um01794 | 0.19307 | K01488 | related to adenosine deaminase |
| 9d00314 | 0.29422 | K14303 | nuclear pore complex, Nup160 component | um00586 | 0.19307 | ni | conserved hypothetical protein |
| 25c00030 | 0.29433 | K03267 | polypeptide release factor 3 | um11866 | 0.19308 | K12857 | probable U5 snRNP-specific 40 kD protein |
| 22d00105 | 0.29434 | ni | hypothetical protein | um01802 | 0.1931 | K09584 | probable protein disulfide-isomerase precursor |
| 16d00011 | 0.29445 | K14819 | dual specificity phosphatase | um03707 | 0.19328 | ni | putative protein |
| 18d00031 | 0.29449 | ni | hypothetical protein | um11843 | 0.19334 | ni | conserved hypothetical protein |
| 13d00077 | 0.2945 | K15601 | putative transcription factor 5qNCA | um00356 | 0.19358 | ni | conserved hypothetical protein |
| 15d00048 | 0.29455 | K05954 | beta subunit of farnesyltransferase | um10106 | 0.19367 | K10589 | related to ubiquitin protein ligase e3 |
| 2d00049 | 0.29467 | K02973 | 40S ribosomal protein S23 | um03820 | 0.19394 | ni | related to PEP5 - vacuolar biogenesis protein |
| 9d00417 | 0.29473 | ni | hypothetical protein | um10290 | 0.19409 | K00830 | probable AGX1 - alanine-glyoxylate transaminase |
| 13c00092 | 0.29475 | ni | hypothetical protein | um02283 | 0.19421 | ni | conserved hypothetical protein |
| 20d00068 | 0.29479 | ni | hypothetical protein | um05806 | 0.19424 | K02355 | probable MEF1 - translation elongation factor G |
| 13d00069 | 0.29484 | ni | hypothetical protein | um05216 | 0.19425 | ni | related to IKS1 - putative serine |
| 22c00110 | 0.29485 | K02212 | DNA replication licensing factor, MCM4 component | um03127 | 0.19428 | ni | conserved hypothetical protein |
| 25c00049 | 0.29485 | ni | regulator of ATP-sensitive K+ channels Alpha-endosulfine | um01186 | 0.19436 | ni | conserved hypothetical protein |
| 9c00106 | 0.29498 | ni | putative phosphoinositide phosphatase | um10423 | 0.19444 | ni | conserved hypothetical protein |
| 9d00349 | 0.29503 | K13118 | nuclear protein ES2 | um00743 | 0.19451 | K14774 | conserved hypothetical protein |
| 7d00103 | 0.29513 | K03126 | transcription initiation factor TFIID, subunit TAF12 | um03944 | 0.19464 | ni | conserved hypothetical protein |
| 12d00130 | 0.29515 | ni | ABC (ATP binding cassette) 1 protein | um11727.2 | 0.19465 | K14863 | related to YTM1 - microtubule-interacting protein |
| 8d00068 | 0.29519 | ni | oxysterol-binding protein | um11969 | 0.19471 | ni | hypothetical protein |
| 26d00021 | 0.29546 | ni | phosphotyrosyl phosphatase activator | um02516 | 0.19478 | K17428 | conserved hypothetical protein |
| 12d00088 | 0.29557 | K15325 | hypothetical protein | um05968 | 0.19489 | ni | related to peptide transporter Mtd1 |
| 12d00033 | 0.29557 | ni | hypothetical protein | um04881 | 0.19495 | K00987 | related to mRNA guanylyltransferase |
| 7d00046 | 0.29579 | K15440 | tRNA-specific adenosine deaminase 1 | um11723 | 0.19499 | K01228 | related to ER glucosidase I |
| 7c00049 | 0.29586 | ni | hypothetical protein | um02824 | 0.19508 | ni | related to diploid state maintenance protein chpA |
| 24d00053 | 0.2961 | ni | kinesin-like protein | um10421 | 0.19513 | ni | related to DPP1 - diacylglycerol pyrophosphate phosphatase |
| 9c00127 | 0.29625 | K03860 | N-acetylglucosaminyltransferase complex, subunit PIG-Q/GPI1 | um05087 | 0.19529 | ni | putative protein |
| 27c00029 | 0.29626 | K12175 | COP9 signalosome, subunit CSN1 | um01867.2 | 0.19542 | ni | conserved hypothetical protein |
| 10c00024 | 0.29645 | ni | mitochondrial Fe2+ transporter MMT1 and related transporters | um01295 | 0.1955 | ni | hypothetical protein |
| 16d00036 | 0.29653 | K07897 | ras-related GTPase | um04356 | 0.19604 | K14772 | conserved hypothetical protein |
| 5c00125 | 0.29656 | ni | hypothetical protein | um04974 | 0.1962 | ni | putative protein |
| 16d00034 | 0.29669 | K14849 | nucleolar protein NOP52/RRP1 | um10262 | 0.19627 | ni | conserved hypothetical protein |
| 27d00061 | 0.29687 | ni | asparaginyl-trna synthetase | um10245 | 0.19638 | ni | conserved hypothetical protein |
| 3d00081 | 0.29691 | ni | 40S ribosomal protein S7 | um10197 | 0.19641 | K02973 | probable 40S ribosomal protein S23 |
| 25d00083 | 0.297 | ni | hypothetical protein | um03986 | 0.19655 | ni | conserved hypothetical protein |
| 10c00023 | 0.2971 | ni | hypothetical protein | um11605 | 0.19686 | K10761 | related to THG1 - protein required for tRNA-His guanylylation at 5 prime end |
| 22c00273 | 0.29725 | ni | hypothetical protein | um01713 | 0.19692 | ni | hypothetical protein |
| 9c00188 | 0.2974 | ni | hypothetical protein | um10119 | 0.19702 | K08850 | probable IPL1 - ser |
| 22c00283 | 0.29744 | K13281 | hypothetical protein | um01129.2 | 0.19711 | ni | conserved hypothetical protein |
| 1d00026 | 0.29748 | ni | animal-type fatty acid synthase and related proteins | um03764 | 0.19712 | ni | hypothetical protein |
| 22c00100 | 0.29751 | ni | hypothetical protein | um05439 | 0.19713 | ni | related to Chitin-binding protein |
| 2c00058 | 0.2976 | ni | hypothetical protein | um11536 | 0.19715 | K02932 | probable RPL5 - 60S large subunit ribosomal protein L5 |
| 7d00115 | 0.2977 | K12626 | small Nuclear ribonucleoprotein splicing factor | um01748 | 0.19733 | ni | related to RVS167 - BAR adaptor protein |
| 19d00074 | 0.29776 | ni | hypothetical protein | um04343.2 | 0.19742 | ni | related to fruiting body protein SC7 precursor |
| 27d00084 | 0.29777 | ni | hypothetical protein | um05965 | 0.19747 | ni | conserved hypothetical protein |
| 22c00117 | 0.29791 | K12763 | hypothetical protein | um03368 | 0.19752 | ni | putative protein |
| 20d00035 | 0.29792 | K01507 | inorganic pyrophosphatase | um04197 | 0.19753 | ni | related to purine-cytosine permease |
| 9c00223 | 0.2981 | ni | hypothetical protein | um05037 | 0.19765 | ni | related to glyoxylate |
| 7c00236 | 0.2981 | K11761 | hypothetical protein | um11342 | 0.19769 | K13119 | related to xap-5 protein |
| 26d00008 | 0.29814 | K05824 | isocitrate dehydrogenase, alpha subunit | um05489 | 0.19779 | ni | related to Nuclear pore complex protein Nup214 |
| 26c00038 | 0.29814 | ni | hypothetical protein | um03318 | 0.19783 | K00988 | related to APA2 - ATP adenylyltransferase II |
| 5d00031 | 0.29817 | ni | hypothetical protein | um12119 | 0.19786 | ni | putative protein |
| 10d00093 | 0.29841 | ni | amino acid transporters | um10593 | 0.1979 | K14521 | conserved hypothetical protein |
| 6d00001 | 0.29845 | ni | hypothetical protein | um11738 | 0.19798 | K15292 | related to syntaxin binding protein 1 |
| 15d00042 | 0.2985 | ni | amidases | um12346 | 0.19812 | ni | conserved hypothetical protein |
| 13c00024 | 0.29858 | ni | membrane protein | um05044 | 0.19859 | K13099 | conserved hypothetical protein |
| 15c00055 | 0.29861 | ni | predicted guanine nucleotide exchange factor | um12061 | 0.19874 | ni | putative protein |
| 15c00078 | 0.29872 | K16261 | amino acid transporters | um10859 | 0.19917 | ni | related to UV-induced protein uvi15 |
| 27c00023 | 0.29875 | K03859 | N-acetylglucosaminyltransferase complex, subunit PIG-C/GPI2 | um05287 | 0.19918 | ni | related to Vacuolar protein sorting-associated protein VPS5 |
| 1c00006 | 0.29876 | ni | hypothetical protein | um01799 | 0.19948 | ni | putative protein |
| 1d00047 | 0.29883 | ni | dimethylglycine dehydrogenase precursor | um03386 | 0.19949 | ni | conserved hypothetical protein |
| 11c00080 | 0.2992 | K12863 | uncharacterized conserved protein | um11567.2 | 0.1995 | ni | related to Histidine triad protein |
| 10d00114 | 0.29924 | ni | hypothetical protein | um11581 | 0.19953 | ni | conserved hypothetical protein |
| 7c00328 | 0.29928 | ni | hypothetical protein | um11357 | 0.19961 | ni | conserved hypothetical protein |
| 12c00037 | 0.29932 | ni | transport protein particle TRAPP complex subunit | um04773 | 0.19968 | ni | conserved hypothetical protein |
| 22d00108 | 0.29939 | K07904 | GTPase Rab11/YPT3 | um11934 | 0.19982 | ni | putative protein |
| 24d00010 | 0.29942 | ni | nucleosome-binding factor SPN, POB3 subunit | um03102 | 0.2 | ni | conserved hypothetical protein |
| 7d00107 | 0.29968 | K11843 | ubiquitin-specific protease | um01030 | 0.20004 | K15193 | conserved hypothetical protein |
| 14c00136 | 0.2997 | ni | hypothetical protein | um03445 | 0.20007 | ni | conserved hypothetical protein |
| 5d00100 | 0.29971 | ni | nuclear transport factor 2 | um06433 | 0.20015 | ni | K |
| 6c00058 | 0.29982 | ni | hypothetical protein | um03000 | 0.20028 | ni | conserved hypothetical protein |
| 27c00033 | 0.29983 | K00852 | ribokinase | um01370.2 | 0.20038 | K03671 | related to Thioredoxin |
| 9c00236 | 0.29987 | ni | calcineurin-mediated signaling pathway inhibitor DSCR1 | um00678 | 0.20046 | K14792 | related to RRP5 - processing of pre-ribosomal RNA |
| 8c00091 | 0.29989 | ni | hypothetical protein | um05704 | 0.2006 | ni | conserved hypothetical protein |
| 9d00060 | 0.29997 | K10599 | mRNA splicing factor | um12332 | 0.20062 | ni | conserved hypothetical protein |
| 10c00079 | 0.30019 | K03458 | hypothetical protein | um01527 | 0.20067 | ni | putative protein |
| 9c00360 | 0.30021 | K15082 | leucine rich repeat proteins | um05443 | 0.20075 | ni | conserved hypothetical protein |
| 12d00045 | 0.30036 | K00252 | glutaryl-coa dehydrogenase | um04880 | 0.20076 | K08337 | probable APG7 - component of the autophagic system |
| 1c00052 | 0.30056 | ni | hypothetical protein | um03332 | 0.20087 | K09537 | related to cell cycle control protein cwf23 |
| 19d00058 | 0.30056 | K00021 | 3-hydroxy-3-methylglutaryl-CoA | um04127 | 0.20092 | ni | probable ERG26 - C-3 sterol dehydrogenase |
| 5d00056 | 0.30078 | ni | hypothetical protein | um06461 | 0.20137 | K05658 | related to Leptomycin B resistance protein pmd1 |
| 7c00356 | 0.30081 | K03066 | 26S proteasome regulatory complex, ATPase RPT6 | um00601 | 0.20175 | K13093 | related to Splicing factor U2AF-associated protein 2 |
| 9c00099 | 0.30091 | ni | hypothetical protein | um10024 | 0.20188 | ni | conserved hypothetical protein |
| 7c00272 | 0.30097 | K01611 | S-adenosylmethionine decarboxylase | um03960 | 0.20195 | ni | putative protein |
| 26d00034 | 0.30116 | ni | hypothetical protein | um04581 | 0.20211 | ni | putative protein |
| 7c00266 | 0.3012 | K01648 | ATP-citrate lyase | um10185 | 0.20222 | ni | related to MDR1 - Mac1p interacting protein |
| 27c00007 | 0.30121 | ni | protein involved in ubiquinone biosynthesis | um05142 | 0.20244 | K08342 | related to ATG4 - essential for autophagy |
| 13c00002 | 0.30127 | K12836 | U2 snRNP splicing factor, small subunit, and related proteins | um04059 | 0.20245 | K03353 | related to anaphase control protein cut9 |
| 1c00020 | 0.30153 | K03133 | transcription initiation factor TFIID, subunit TAF9 | um04320 | 0.20247 | K14803 | related to PTC3 - ser |
| 7d00054 | 0.30163 | ni | sorting nexin SNX11 | um01621 | 0.20263 | ni | conserved hypothetical protein |
| 18d00025 | 0.30177 | ni | hypothetical protein | um11805 | 0.20291 | ni | conserved hypothetical protein |
| 5c00049 | 0.30185 | ni | hypothetical protein | um00561.2 | 0.20292 | ni | related to cell cycle arrest protein BUB2 |
| 12c00098 | 0.30185 | K14816 | C2H2-type Zn-finger protein | um01231 | 0.20293 | K00729 | related to dolichyl-phosphate beta-glucosyltransferase |
| 1d00004 | 0.30196 | K17361 | acyl-coa thioesterase | um10433 | 0.20299 | K03014 | probable Rpo26 - 18kD subunit of DNA-directed RNA polymerases I |
| 7c00118 | 0.30209 | K02889 | 60S ribosomal protein L21 | um10480 | 0.20304 | ni | conserved hypothetical protein |
| 3c00022 | 0.30211 | K01476 | arginase | um00971 | 0.20321 | ni | conserved hypothetical protein |
| 9c00244 | 0.30234 | ni | hypothetical protein | um04546 | 0.20322 | ni | ni |
| 9c00323 | 0.30235 | ni | hypothetical protein | um05899 | 0.20324 | ni | conserved hypothetical protein |
| 5c00079 | 0.30237 | ni | ypt/rab-specific GTPase-activating protein GYP7 | um00130 | 0.20327 | K06130 | related to lysophospholipase |
| 4c00034 | 0.30261 | ni | hypothetical protein | um00376 | 0.20327 | K11872 | related to deubiquitinating enzyme ubh1 |
| 18c00012 | 0.30268 | ni | hypothetical protein | um01718 | 0.20339 | K02564 | probable glucosamine-6-phosphate isomerase |
| 5d00119 | 0.30308 | ni | hypothetical protein | um01820 | 0.20348 | ni | hypothetical protein |
| 24c00050 | 0.30311 | K14806 | ATP-dependent RNA helicase | um04082 | 0.20357 | ni | hypothetical protein |
| 9c00032 | 0.3032 | ni | hypothetical protein | um03619 | 0.20384 | ni | related to multidrug resistant protein |
| 9c00141 | 0.30353 | K02990 | mitochondrial ribosomal protein MRP17 | um05201 | 0.20413 | K10882 | conserved hypothetical protein |
| 12c00101 | 0.30361 | K09580 | protein disulfide isomerase | um04470 | 0.20416 | K00700 | probable branching enzyme |
| 22c00030 | 0.30367 | ni | 5' nucleotidase | um01939 | 0.20418 | ni | conserved hypothetical protein |
| 9d00200 | 0.30373 | ni | sorbitol dehydrogenase | um00903 | 0.20421 | K11366 | related to UBP8 - Ubiquitin-specific protease component of the SAGA complex |
| 15d00085 | 0.30401 | ni | hypothetical protein | um00458 | 0.20441 | K02304 | related to siroheme synthase |
| 11c00060 | 0.30411 | ni | hypothetical protein | um00105 | 0.20446 | K05909 | iron transport multicopper oxidase |
| 25c00038 | 0.30422 | ni | hypothetical protein | um05272 | 0.20459 | ni | conserved hypothetical protein |
| 10c00029 | 0.30425 | ni | hypothetical protein | um00333 | 0.20475 | ni | conserved hypothetical protein |
| 16c00087 | 0.3043 | K01053 | hypothetical protein | um06288 | 0.20491 | ni | hypothetical protein |
| 9d00071 | 0.30431 | ni | hypothetical protein | um00840 | 0.20504 | ni | conserved hypothetical protein |
| 19d00025 | 0.30436 | ni | hypothetical protein | um03407 | 0.20519 | K06978 | conserved hypothetical protein |
| 27d00037 | 0.3044 | K14454 | aspartate aminotransferase | um05386 | 0.20525 | ni | putative protein |
| 9d00038 | 0.30442 | ni | hypothetical protein | um06408 | 0.20528 | ni | related to IVY1 - phospholipid-binding protein |
| 6c00048 | 0.30452 | ni | hypothetical protein | um01309 | 0.2054 | ni | related to TIP41 - negatively regulates the TOR signaling pathway |
| 13c00003 | 0.30455 | ni | hypothetical protein | um10300 | 0.20566 | K09506 | conserved hypothetical protein |
| 27d00090 | 0.30468 | K10747 | ATP-dependent DNA ligase I | um11113 | 0.20604 | ni | conserved hypothetical protein |
| 6d00095 | 0.30473 | ni | hypothetical protein | um02057 | 0.20617 | K09553 | probable STI1 - Hsp90 cochaperone |
| 11d00084 | 0.30489 | K15109 | mitochondrial carnitine-acylcarnitine carrier protein | um01595 | 0.20619 | ni | conserved hypothetical protein |
| 26d00100 | 0.30496 | ni | hypothetical protein | um05601 | 0.20623 | ni | hypothetical protein |
| 8c00057 | 0.30518 | K01915 | glutamine synthetase | um04180 | 0.20626 | K11559 | related to MTW1 - component of the MIND kinetochore complex |
| 14d00029 | 0.30525 | K15267 | hypothetical protein | um00007 | 0.20644 | K10703 | conserved hypothetical protein |
| 26c00068 | 0.30539 | K06874 | C4-type Zn-finger protein | um00420 | 0.20654 | ni | conserved hypothetical protein |
| 15c00034 | 0.30555 | ni | hypothetical protein | um05060 | 0.20655 | K02935 | related to 60S ribosomal protein L12 |
| 22d00293 | 0.30558 | ni | predicted panthothenate kinase | um00144 | 0.20659 | ni | putative protein |
| 22d00213 | 0.3056 | K15340 | predicted hydrolase involved in interstrand cross-link repair | um05517 | 0.20664 | ni | hypothetical protein |

| | | | | | | | |
|---|---|---|---|---|---|---|---|
| 7d00030 | 0.30562 | K11397 | hypothetical protein | um02944 | 0.20673 | K09531 | conserved hypothetical protein |
| 12c00008 | 0.30564 | K00599 | guanidinoacetate methyltransferase and related proteins | um00585 | 0.20687 | K03035 | probable RPN5 - 26S proteasome regulatory subunit |
| 7d00070 | 0.30571 | K14832 | CAATT-binding transcription factor | um01689 | 0.20693 | ni | hypothetical protein |
| 18c00097 | 0.30585 | K10356 | myosin class I heavy chain | um12211 | 0.20697 | ni | putative protein |
| 14c00077 | 0.30589 | K01652 | thiamine pyrophosphate-requiring enzyme | um06338 | 0.20698 | ni | related to Restin |
| 3d00004 | 0.30592 | ni | uncharacterized conserved protein | um01788 | 0.20711 | ni | related to deacetylase |
| 10d00118 | 0.30598 | K00784 | predicted metal-dependent hydrolase | um11115 | 0.20736 | K10356 | myosin I |
| 16c00048 | 0.30603 | ni | hypothetical protein | um04283 | 0.20736 | ni | putative protein |
| 13c00059 | 0.3061 | ni | hypothetical protein | um04345 | 0.20743 | K02152 | probable vacuolar atp synthase subunit g |
| 8d00022 | 0.30614 | ni | hypothetical protein | um00273 | 0.20749 | ni | conserved hypothetical protein |
| 22c00303 | 0.30631 | ni | hypothetical protein | um10868 | 0.20773 | ni | conserved hypothetical protein |
| 9c00218 | 0.30634 | K16073 | hypothetical protein | um11885 | 0.20776 | ni | related to UDP-N-acetylglucosaminyltransferase |
| 22c00210 | 0.30637 | ni | hypothetical protein | um00215 | 0.20779 | K00931 | probable PRO1 - glutamate 5-kinase |
| 9c00220 | 0.30647 | ni | hypothetical protein | um05326 | 0.20799 | ni | conserved hypothetical protein |
| 7d00148 | 0.30652 | K00052 | 3-isopropylmalate dehydrogenase | um03865 | 0.208 | K07901 | probable SEC4-like Rab |
| 15c00050 | 0.30691 | ni | uncharacterized conserved protein | um12174 | 0.20813 | ni | conserved hypothetical protein |
| 9c00168 | 0.30698 | ni | hypothetical protein | um03810 | 0.20816 | ni | ni |
| 19c00104 | 0.307 | ni | hypothetical protein | um03365 | 0.20823 | ni | putative protein |
| 13c00072 | 0.30706 | K06676 | chromosome condensation complex Condensin, subunit H | um05371 | 0.20833 | ni | conserved hypothetical protein |
| 11c00015 | 0.3071 | ni | hypothetical protein | um05645 | 0.20833 | K08493 | related to VTI1 - v-SNARE |
| 6d00074 | 0.30718 | ni | hypothetical protein | um02649 | 0.20848 | ni | conserved hypothetical protein |
| 10d00015 | 0.30718 | K00237 | succinate dehydrogenase membrane anchor subunit and related | um05660 | 0.20851 | ni | conserved hypothetical protein |
| 10c00001 | 0.30724 | ni | hypothetical protein | um03814.2 | 0.20867 | ni | conserved hypothetical protein |
| 6d00113 | 0.30744 | ni | DEAH-box RNA helicase | um05901 | 0.20877 | K08496 | conserved hypothetical protein |
| 14d00118 | 0.30746 | K04079 | molecular chaperone | um03892 | 0.20881 | K14810 | probable ATP dependent RNA helicase of the DEAD-box family |
| 20d00002 | 0.30763 | K01714 | hypothetical protein | um05004 | 0.20885 | ni | putative protein |
| 2c00015 | 0.3077 | K14538 | GTPase | um10854 | 0.20892 | ni | conserved hypothetical protein |
| 6c00012 | 0.30786 | ni | hypothetical protein | um02615 | 0.20894 | ni | hypothetical protein |
| 25d00040 | 0.30788 | K11984 | U4/U6.U5 snRNP associated protein | um03122 | 0.20922 | ni | related to beta-1 |
| 12d00094 | 0.30802 | ni | hypothetical protein | um11747.2 | 0.20934 | ni | conserved hypothetical protein |
| 18c00064 | 0.30802 | K07213 | copper chaperone | um11979 | 0.20943 | ni | conserved hypothetical Ustilago-specific protein |
| 22d00262 | 0.3081 | ni | hypothetical protein | um02088 | 0.20951 | K00939 | probable ADK1 - adenylate kinase |
| 7c00329 | 0.30815 | K14998 | mitochondrial protein Surfeit 1/SURF1/SHY1 | um12315 | 0.20955 | ni | hypothetical protein |
| 22d00167 | 0.30818 | K09660 | predicted endoplasmic reticulum membrane protein Lec35/MPDU | um03919 | 0.20973 | ni | related to FIS1 - protein involved in mitochondrial division |
| 26d00064 | 0.3083 | K03439 | methyltransferase-like protein | um01323 | 0.20978 | K12870 | related to pre-mRNA splicing factor |
| 15d00093 | 0.30833 | K00665 | animal-type fatty acid synthase and related proteins | um02385 | 0.20994 | K01626 | probable phospho-2-dehydro-3-deoxyheptonate aldolase |
| 15c00048 | 0.30849 | ni | hypothetical protein | um11310 | 0.21011 | ni | conserved hypothetical protein |
| 10d00001 | 0.30853 | K03006 | RNA polymerase II, large subunit | um11348.2 | 0.21023 | ni | conserved hypothetical protein |
| 19d00153 | 0.30862 | ni | vacuolar sorting protein VPS45 | um11780 | 0.21026 | K03238 | probable SUI3 - translation initiation factor eIF2 beta subunit |
| 9d00380 | 0.30878 | ni | hypothetical protein | um05571 | 0.21048 | ni | related to alpha |
| 9c00146 | 0.30879 | ni | hypothetical protein | um05102 | 0.2106 | ni | conserved hypothetical protein |
| 25d00007 | 0.30889 | ni | FOG, RRM domain | um04673 | 0.21089 | ni | related to RecQ family helicase RecQL1 |
| 19c00027 | 0.30901 | K00794 | 6,7-dimethyl-8-ribityllumazine synthase | um02253 | 0.21089 | K14997 | related to amino acid vacuolar transport protein AVT2 |
| 11d00018 | 0.30901 | ni | defense-related protein containing SCP domain | um01322 | 0.21093 | ni | probable VID27 - involved in Vacuole import and degradation |
| 24c00020 | 0.30905 | K00728 | dolichyl-phosphate-mannose, protein O-mannosyl transferase | um00975 | 0.21101 | K02140 | related to ATP20 - subunit G of mitochondrial F1F0-ATP Synthase |
| 7d00031 | 0.30918 | ni | hypothetical protein | um11378 | 0.21135 | K01725 | probable Cyanate hydratase |
| 11d00076 | 0.30922 | K11359 | histone acetyltransferase SAGA/ADA, catalytic subunit PCAF/GC | um00835.2 | 0.21142 | ni | putative protein |
| 9c00051 | 0.30927 | K03637 | molybdenum cofactor biosynthesis pathway protein | um02243 | 0.21148 | ni | hypothetical protein |
| 4c00017 | 0.30951 | ni | hypothetical protein | um05397 | 0.21157 | ni | putative protein |
| 20d00007 | 0.30954 | ni | predicted membrane protein | um12232 | 0.21167 | K01875 | related to mitochondrial seryl-tRNA synthetases |
| 11d00070 | 0.30956 | ni | predicted membrane protein | um02629 | 0.21188 | ni | related to YRO2 - putative plasma membrane protein |
| 22c00140 | 0.30959 | ni | hypothetical protein | um12281 | 0.21193 | ni | putative protein |
| 9d00348 | 0.30965 | K12847 | spindle pole body protein - Sad1p | um03478 | 0.21214 | ni | related to CSN12 - COP9 signalosome |
| 7c00226 | 0.30967 | K13524 | 4-aminobutyrate aminotransferase | um01363 | 0.21229 | K03004 | related to Rpa43 - 43 kD subunit of DNA-directed RNA polymerase I |
| 9c00234 | 0.30968 | ni | hypothetical protein | um00074 | 0.21249 | K14839 | conserved hypothetical protein |
| 19d00078 | 0.30992 | ni | predicted methyltransferase | um03918 | 0.21251 | ni | conserved hypothetical protein |
| 2d00001 | 0.31009 | ni | hypothetical protein | um12202 | 0.21261 | ni | putative protein |
| 9c00090 | 0.31019 | K08851 | serine/threonine protein kinase | um02770 | 0.21265 | ni | hypothetical protein |
| 9c00267 | 0.31043 | ni | hypothetical protein | um06066 | 0.21278 | ni | conserved hypothetical protein |
| 25d00025 | 0.31055 | ni | uncharacterized conserved protein | um05916 | 0.21296 | K01598 | related to Phosphopantothenoylcysteine decarboxylase |
| 19d00093 | 0.3106 | ni | hypothetical protein | um10126 | 0.21314 | ni | related to SFT2 |
| 25c00063 | 0.31068 | ni | hypothetical protein | um03309 | 0.21314 | K14018 | related to DOA1 - involved in ubiquitin-dependent proteolysis |
| 26d00102 | 0.31073 | ni | predicted transporter | um10065 | 0.21332 | ni | related to ACN9 - protein of gluconeogenesis in mitochondrial intermembrane space |
| 9d00413 | 0.31086 | ni | NADH-dehydrogenase | um10765 | 0.21353 | K03469 | related to Ribonuclease H |
| 16c00083 | 0.31156 | ni | hypothetical protein | um01305 | 0.21355 | K11883 | conserved hypothetical protein |
| 7c00080 | 0.31159 | ni | hypothetical protein | um01084 | 0.21359 | ni | conserved hypothetical protein |
| 26d00050 | 0.31176 | ni | hypothetical protein | um00370 | 0.21361 | K01115 | probable SPO14 - phospholipase D |
| 7d00281 | 0.31178 | K03872 | RNA polymerase II transcription elongation factor Elongin/SIII, su | um04198 | 0.21367 | K12669 | related to OST3 - oligosaccharyltransferase gamma subunit |
| 27c00014 | 0.31195 | ni | hypothetical protein | um03100 | 0.21378 | ni | related to Dynein light chain 2B |
| 7c00122 | 0.312 | K03242 | translation initiation factor 2, gamma subunit | um10865 | 0.2141 | ni | conserved hypothetical protein |
| 2c00022 | 0.31205 | K04640 | G-protein alpha subunit | um11908 | 0.21412 | ni | related to cathepsin d |
| 7c00135 | 0.31212 | ni | B-cell receptor-associated protein and related proteins | um03010 | 0.21419 | ni | conserved hypothetical protein |
| 4c00018 | 0.31221 | ni | hypothetical protein | um00029 | 0.21421 | ni | probable myo-inositol oxygenase |
| 9d00052 | 0.31235 | ni | predicted coiled-coil protein | um11332 | 0.21434 | ni | putative protein |
| 27d00031 | 0.31243 | K15564 | CDK9 kinase-activating protein cyclin T | um04027 | 0.21444 | ni | hypothetical protein |
| 27c00073 | 0.31245 | K02943 | 60S acidic ribosomal protein P2 | um04753 | 0.21449 | ni | conserved hypothetical protein |
| 22c00185 | 0.31245 | K06929 | hypothetical protein | um03953 | 0.21462 | ni | conserved hypothetical protein |
| 18c00085 | 0.31263 | ni | exocyst complex subunit | um03975 | 0.21462 | ni | related to Carboxypeptidase Y precursor |
| 9d00009 | 0.31268 | ni | hypothetical protein | um01890 | 0.21464 | ni | hypothetical protein |
| 9d00068 | 0.31276 | ni | hypothetical protein | um00579 | 0.21471 | K00670 | related to n-terminal acetyltransferase 1 |
| 7d00025 | 0.31281 | ni | hypothetical protein | um03640 | 0.21474 | ni | conserved hypothetical protein |
| 11d00060 | 0.31317 | K10251 | 17 beta-hydroxysteroid dehydrogenase type 3 | um06387 | 0.21487 | K03133 | related to TAF9 - TFIID and SAGA subunit |
| 13c00089 | 0.31325 | K06176 | uncharacterized conserved protein | um02354 | 0.21513 | ni | putative protein |
| 9c00245 | 0.31351 | ni | hypothetical protein | um11364 | 0.21521 | K01443 | conserved hypothetical protein |
| 20d00029 | 0.31358 | K01593 | aromatic-l-amino-acid/l-histidine decarboxylase | um01021 | 0.21522 | ni | putative protein |
| 25d00016 | 0.31383 | K14788 | WD40 repeat protein | um10644 | 0.21524 | K16938 | probable CDC10 - septin |
| 14d00095 | 0.31386 | K12590 | exosomal 3'-5' exoribonuclease complex, subunit Rrp46 | um03297 | 0.21525 | ni | hypothetical protein |
| 5c00112 | 0.31398 | ni | aspartyl protease | um01239 | 0.2157 | ni | conserved hypothetical Ustilago-specific protein |
| 3d00074 | 0.31403 | K01653 | acetolactate synthase, small subunit | um04424 | 0.21582 | ni | conserved hypothetical protein |
| 26c00052 | 0.31404 | K14301 | nuclear pore complex, rNup107 component | um06270 | 0.21589 | ni | conserved hypothetical protein |
| 11c00043 | 0.31421 | ni | hypothetical protein | um10163 | 0.21589 | K02882 | probable 60S large subunit ribosomal protein L20 |
| 11c00067 | 0.31444 | ni | hypothetical protein | um02070 | 0.21604 | ni | conserved hypothetical protein |
| 7c00130 | 0.31446 | K10751 | chromatin assembly complex 1 subunit B/CAC2 | um01837 | 0.21613 | ni | conserved hypothetical protein |
| 19c00108 | 0.31461 | ni | rho GTPase-activating protein | um06394 | 0.21632 | K02884 | related to 50S ribosomal protein L19 |
| 12c00105 | 0.31467 | K11236 | invasion-inducing protein TIAM1/CDC24 | um02193 | 0.21636 | ni | hypothetical protein |
| 11d00040 | 0.31486 | ni | 3-Methylcrotonyl-CoA carboxylase, non-biotin containing subunit | um04147 | 0.21638 | ni | related to EXO84 - exocyst protein essential for secretion |
| 22c00074 | 0.31492 | ni | hypothetical protein | um03812 | 0.21639 | K12041 | probable NHX1 - Na |
| 11c00049 | 0.31495 | ni | FOG, RRM domain | um02180 | 0.21642 | ni | conserved hypothetical protein |
| 13d00079 | 0.31515 | ni | mod5 protein sorting/negative effector of RNA Pol III synthesis | um06112 | 0.21642 | ni | hypothetical protein |
| 12d00102 | 0.3153 | K03029 | 26S proteasome regulatory complex, subunit RPN10/PSMD4 | um11704.2 | 0.21645 | K08339 | conserved hypothetical protein |
| 25c00076 | 0.31544 | K12666 | oligosaccharyltransferase, alpha subunit | um10129 | 0.21657 | K03242 | probable translation initiation factor eIF2 gamma chain |
| 9d00215 | 0.31551 | ni | hypothetical protein | um15003 | 0.21667 | K03138 | related to TFG1 - TFIIF subunit |
| 48c00001 | 0.31554 | ni | ni | um04315 | 0.2167 | ni | hypothetical protein |
| 13d00053 | 0.31562 | K08793 | ribosomal protein S6 kinase and related proteins | um03044 | 0.21672 | ni | conserved hypothetical protein |
| 27d00069 | 0.31565 | K08991 | endonuclease MUS81 | um04415 | 0.21673 | ni | conserved hypothetical protein |
| 26c00046 | 0.31566 | K01897 | long chain fatty acid acyl-CoA ligase | um00068 | 0.21689 | ni | conserved hypothetical protein |
| 4d00025 | 0.31572 | ni | hypothetical protein | um01554 | 0.217 | ni | putative protein |
| 2d00056 | 0.31576 | K10882 | hypothetical protein | um00192 | 0.21702 | ni | putative protein |
| 22d00149 | 0.31577 | ni | hypothetical protein | um10491 | 0.21709 | ni | putative protein |
| 22d00195 | 0.31577 | K12946 | hypothetical protein | um04367 | 0.2172 | ni | hypothetical protein |
| 27d00026 | 0.31581 | ni | rhodanese-related sulfurtransferase | um11545 | 0.21725 | K11667 | conserved hypothetical protein |
| 19d00148 | 0.31586 | ni | hypothetical protein | um03514 | 0.21728 | ni | putative protein |
| 7c00297 | 0.31596 | K08956 | AAA+-type ATPase containing the peptidase M41 domain | um01208 | 0.21731 | K10573 | probable RAD6 - E2 ubiquitin-conjugating enzyme |
| 25d00001 | 0.31608 | ni | hypothetical protein | um05035 | 0.21747 | K16261 | probable AGP3 - Protein of the amino-acid permease family |
| 22c00311 | 0.31613 | ni | hypothetical protein | um02225 | 0.21747 | ni | conserved hypothetical protein |
| 5c00045 | 0.31619 | ni | hypothetical protein | um00041 | 0.21764 | ni | putative protein |
| 7d00014 | 0.3162 | ni | hypothetical protein | um02356 | 0.21777 | ni | related to beta-galactosidase precursor |
| 1d00008 | 0.3163 | K07953 | vesicle coat complex COPII, GTPase subunit SAR1 | um11455 | 0.21795 | ni | hypothetical protein |
| 15c00095 | 0.31655 | ni | phospholipase | um04531 | 0.21796 | ni | conserved hypothetical protein |
| 26c00013 | 0.31655 | K12389 | predicted small molecule transporter | um11639 | 0.21819 | ni | hypothetical protein |
| 19d00122 | 0.31657 | ni | hypothetical protein | um06317 | 0.21822 | ni | related to INP52 - phosphatidylinositol phosphate phosphatase |
| 7c00357 | 0.31658 | ni | FOG, PPR repeat | um03049 | 0.21841 | ni | related to neutral amino acid permease |
| 12c00034 | 0.31669 | ni | hypothetical protein | um10525 | 0.21852 | ni | hypothetical protein |
| 22c00037 | 0.31675 | ni | PHD Zn-finger protein | um03079 | 0.21854 | K03130 | related to TAF5 - TFIID and SAGA subunit |
| 14c00047 | 0.31678 | K15363 | uncharacterized conserved protein | um05171 | 0.21864 | K13941 | related to multifunctional folic acid synthesis protein |
| 25c00011 | 0.31693 | ni | hypothetical protein | um05674 | 0.21868 | K01173 | probable NUC1 - dna |
| 14c00076 | 0.31699 | ni | hypothetical protein | um05921 | 0.21875 | K05544 | related to DUS3 - member of dihydrouridine synthase family |
| 10c00101 | 0.31701 | ni | vacuolar sorting protein VPS1 | um00204 | 0.21876 | K01537 | related to Ca2 -transporting ATPase |
| 12c00056 | 0.31732 | K02214 | serine/threonine protein kinase of the CDC7 subfamily | um02259 | 0.21891 | ni | conserved hypothetical protein |
| 12d00132 | 0.31755 | ni | hypothetical protein | um12082 | 0.21927 | K14263 | related to Serine protease |
| 15d00073 | 0.31771 | ni | heat shock transcription factor | um02663 | 0.21939 | ni | conserved hypothetical protein |
| 9d00147 | 0.31786 | ni | hypothetical protein | um11666 | 0.21969 | K11599 | related to UMP1 - proteasome maturation factor |
| 12d00091 | 0.31801 | ni | hypothetical protein | um04291 | 0.22005 | K14552 | conserved hypothetical protein |
| 18c00092 | 0.31822 | ni | cysteine desulfurase NFS1 | um06357.2 | 0.22017 | ni | putative protein |
| 25d00033 | 0.31833 | K08286 | putative serine/threonine protein kinase | um01852 | 0.22031 | ni | related to Epoxide hydrolase 1 |
| 13d00033 | 0.31846 | ni | peroxisomal NUDIX hydrolase | um02543 | 0.22048 | K02604 | conserved hypothetical protein |
| 9d00005 | 0.31867 | ni | choline transporter-like protein | um10200 | 0.22048 | ni | related to GTPase-activating protein of the rho |
| 19d00050 | 0.31875 | ni | hypothetical protein | um02494 | 0.22054 | K07975 | GTP binding protein Rho2 |
| 13c00076 | 0.31876 | ni | hypothetical protein | um06362 | 0.22056 | K14818 | related to Angio-associated migratory cell protein |
| 22c00154 | 0.31889 | ni | hypothetical protein | um05472 | 0.22064 | K03626 | related to nascent polypeptide-associated complex alpha chain |
| 7c00087 | 0.31912 | K11309 | hypothetical protein | um05482 | 0.22075 | K03257 | probable TIF2 - translation initiation factor eIF4A |
| 7c00047 | 0.31914 | K03940 | NADH-ubiquinone oxidoreductase, NUFS7/PSST/20 kDa subuni | um04300 | 0.22104 | ni | conserved hypothetical protein |
| 9d00120 | 0.31918 | ni | hypothetical protein | um04135 | 0.2212 | ni | putative protein |
| 4c00003 | 0.3192 | ni | uncharacterized membrane protein | um02729 | 0.22155 | ni | conserved hypothetical protein |
| 25c00009 | 0.31943 | ni | predicted phosphoglycerate mutase | um03538 | 0.22155 | ni | tryptophan amino transferase |
| 9c00195 | 0.3196 | ni | hypothetical protein | um01229 | 0.22162 | ni | conserved hypothetical protein |
| 24c00072 | 0.32001 | ni | myotubularin-related phosphatidylinositol 3-phosphate 3-phospha | um02560 | 0.22166 | ni | related to EMP24 protein precursor |
| 11c00019 | 0.32008 | ni | hypothetical protein | um10461 | 0.22175 | ni | conserved hypothetical protein |
| 25d00048 | 0.32025 | K06672 | sister chromatid cohesion protein SCC2/Nipped-B | um03826 | 0.22181 | K04648 | related to Dynactin 1 |
| 18d00079 | 0.32029 | K05236 | vesicle coat complex COPI, alpha subunit | um01604 | 0.22218 | ni | related to Endo-1 |
| 18c00047 | 0.32029 | K01850 | chorismate mutase | um01638 | 0.22231 | ni | putative protein |
| 22d00055 | 0.32036 | ni | hypothetical protein | um03136 | 0.22236 | ni | conserved hypothetical protein |
| 25d00056 | 0.32042 | K14569 | GTP-binding protein AARP2 involved in 40S ribosome biogenesis | um15008 | 0.22245 | K01810 | probable PGI1 - Glucose-6-phosphate isomerase |
| 22c00028 | 0.32046 | ni | hypothetical protein | um01901 | 0.22249 | ni | ni |
| 2d00050 | 0.32063 | ni | hypothetical protein | um06155 | 0.22256 | ni | conserved hypothetical protein |
| 13d00028 | 0.32067 | ni | hypothetical protein | um00326 | 0.22263 | ni | conserved hypothetical protein |

| | | | | | | | |
|---|---|---|---|---|---|---|---|
| 5d00136 | 0.32075 | ni | type I phosphodiesterase | um00379 | 0.22278 | K03250 | related to eIF3e - eukaryotic initiation factor 3 subunit E |
| 1c00008 | 0.32099 | ni | hypothetical protein | um01569 | 0.2228 | ni | related to BRE4 - protein involved in endocytosis |
| 9d00396 | 0.32104 | ni | hypothetical protein | um01893 | 0.22287 | ni | conserved hypothetical protein |
| 6d00066 | 0.32105 | ni | protein tyrosine kinase 9 | um11739 | 0.22289 | K00940 | related to Nucleoside diphosphate kinase 6 |
| 24d00004 | 0.32119 | ni | NADH-dehydrogenase | um11365 | 0.22298 | ni | conserved hypothetical protein |
| 26d00066 | 0.32119 | K10598 | cyclophilin type | um12335 | 0.22307 | K16738 | related to nuclear distribution protein RO11 |
| 19c00055 | 0.32119 | K02923 | 60S ribosomal protein L38 | um10034 | 0.22323 | ni | conserved hypothetical protein |
| 12c00018 | 0.32121 | K01175 | 3'-5' exonuclease | um00777 | 0.22334 | ni | related to RAV1 - Regulator of |
| 5d00006 | 0.32128 | K10610 | damage-specific DNA binding complex, subunit DDB1 | um00911 | 0.22337 | K14635 | related to MPH1 - Member of the DEAH family of helicases |
| 12d00089 | 0.32154 | K05754 | actin-related protein Arp2/3 complex, subunit ARPC5 | um10160 | 0.2238 | ni | conserved hypothetical protein |
| 6c00114 | 0.32156 | K15053 | protein involved in glucose derepression and pre-vacuolar endosc | um12216 | 0.22398 | ni | related to Mig1 protein |
| 14c00049 | 0.32168 | ni | hypothetical protein | um11228 | 0.22402 | K08658 | related to CAAX prenyl protease 2 |
| 19d00141 | 0.32183 | ni | hypothetical protein | um03141 | 0.22415 | K10746 | related to EXO1 - exonuclease which interacts with Msh2p |
| 19c00101 | 0.32196 | ni | hypothetical protein | um05998 | 0.22427 | K02893 | probable RPL25 - ribosomal protein L23a |
| 5d00097 | 0.32196 | ni | hypothetical protein | um11625 | 0.2243 | K02918 | probable ribosomal protein L35 |
| 14c00023 | 0.32203 | ni | hypothetical protein | um05858 | 0.22451 | ni | related to FIG4 - polyphosphoinositide phosphatase family member |
| 16d00010 | 0.32205 | ni | synaptic vesicle transporter SVOP and related transporters | um03844 | 0.22495 | K14859 | related to SSF1 - Nucleolar protein involved in the assembly of the large ribosomal subunit |
| 9d00322 | 0.32205 | ni | hypothetical protein | um11620 | 0.22497 | K07573 | related to CSL4 - exosome core component |
| 5d00007 | 0.32206 | K00826 | branched chain aminotransferase BCAT1 | um11035 | 0.22504 | K12862 | putative protein |
| 13d00019 | 0.32207 | ni | hypothetical protein | um01874 | 0.22519 | ni | conserved hypothetical protein |
| 7d00267 | 0.32237 | K11566 | hypothetical protein | um02350 | 0.22519 | K09498 | probable CCT6 - component of chaperonin-containing T-complex |
| 9c00078 | 0.32245 | ni | glycolate oxidase | um00277 | 0.22524 | ni | related to M-phase inducer phosphatase |
| 20c00074 | 0.32246 | ni | hypothetical protein | um01026 | 0.22535 | ni | conserved hypothetical protein |
| 9c00297 | 0.32273 | ni | hypothetical protein | um03493 | 0.22536 | K03687 | related to MGE1 - heat shock protein - chaperone |
| 14c00037 | 0.32281 | K01934 | 5-formyltetrahydrofolate cyclo-ligase | um06299 | 0.22555 | ni | hypothetical protein |
| 9c00037 | 0.32283 | K03786 | hypothetical protein | um11565 | 0.22556 | ni | conserved hypothetical protein |
| 22d00285 | 0.32284 | ni | synaptic vesicle transporter SVOP and related transporters | um03991 | 0.2258 | ni | putative protein |
| 26c00048 | 0.32286 | ni | glutathione S-transferase | um01857 | 0.22583 | K15078 | conserved hypothetical protein |
| 7d00026 | 0.32294 | ni | serine O-acetyltransferase | um15019 | 0.22596 | ni | related to KEL1 - involved in cell fusion and morphology |
| 15d00065 | 0.3231 | K10638 | hypothetical protein | um11642 | 0.22606 | K14553 | conserved hypothetical protein |
| 10d00090 | 0.32355 | ni | hypothetical protein | um00030 | 0.22607 | ni | related to Proline oxidase |
| 25c00024 | 0.32381 | ni | reductases with broad range of substrate specificities | um05769 | 0.22623 | K12666 | related to Dolichyl-diphosphooligosaccharide--protein glycosyltransferase 67 kDa subunit precursor |
| 1d00031 | 0.32386 | K15920 | hypothetical protein | um12334 | 0.22629 | ni | conserved hypothetical protein |
| 16c00068 | 0.32389 | ni | sexual differentiation process protein ISP4 | um15009 | 0.22635 | K07189 | conserved hypothetical protein |
| 9d00125 | 0.324 | ni | hypothetical protein | um11180 | 0.22643 | ni | putative protein |
| 7c00097 | 0.32401 | K04459 | dual specificity phosphatase | um03917 | 0.22644 | ni | ni |
| 3d00013 | 0.32416 | K11293 | histone transcription regulator HIRA | um01908 | 0.22653 | ni | related to CCAAT-binding transcription factor subunit aab-1 |
| 20c00025 | 0.32431 | K03115 | casein kinase II, beta subunit | um01455 | 0.22707 | ni | hypothetical protein |
| 2c00077 | 0.32435 | ni | prenylated rab acceptor 1 | um00781 | 0.2272 | ni | putative protein |
| 11d00047 | 0.32435 | K00586 | diphthine synthase | um02597 | 0.22722 | ni | related to Rhizopuspepsin precursor |
| 5d00141 | 0.3244 | ni | hypothetical protein | um10210.2 | 0.22733 | ni | conserved hypothetical protein |
| 22d00201 | 0.32492 | ni | hypothetical protein | um10532 | 0.22745 | ni | related to polyketide synthase required for biosynthesis of fumonisin mycotoxins |
| 24c00019 | 0.32497 | ni | hypothetical protein | um01439 | 0.22747 | ni | related to FRE3 - Ferric reductase |
| 9d00229 | 0.32503 | K08850 | serine/threonine protein kinase | um11081 | 0.22756 | ni | putative protein |
| 22d00177 | 0.32504 | K00100 | voltage-gated shaker-like K+ channel, subunit beta/KCNAB | um05117 | 0.22786 | ni | putative protein |
| 19c00041 | 0.32506 | ni | ribonuclease III domain proteins | um05610 | 0.22798 | K00135 | probable UGA2 - succinate semialdehyde dehydrogenase |
| 25c00048 | 0.32518 | ni | cytochrome P450 CYP3/CYP5/CYP6/CYP9 subfamilies | um02724 | 0.22805 | ni | conserved hypothetical protein |
| 10c00091 | 0.32554 | K07478 | ATPase related to the helicase subunit of the Holliday junction res | um10375 | 0.22822 | ni | related to HRB1 - Poly |
| 22d00083 | 0.32558 | ni | hypothetical protein | um03518 | 0.22823 | ni | related to pyrimidine 5-nucleotidase |
| 16d00080 | 0.32559 | ni | aldehyde dehydrogenase | um15037 | 0.22828 | ni | conserved hypothetical protein |
| 22c00093 | 0.32578 | ni | hypothetical protein | um05525 | 0.22831 | ni | related to MRPL16 - mitochondrial ribosomal protein |
| 5d00081 | 0.3258 | K12859 | component of the U4/U6.U5 snRNP | um10109 | 0.22844 | ni | conserved hypothetical protein |
| 7c00128 | 0.32585 | ni | membrane coat complex Retromer, subunit VPS35 | um00154 | 0.22851 | ni | related to Para-nitrobenzyl esterase |
| 14c00110 | 0.32605 | ni | cyclin L | um12033 | 0.22889 | ni | HMG-box transcription factor |
| 22d00295 | 0.32605 | ni | phosphoinositide phosphatase SAC1 | um04054 | 0.22894 | ni | probable NBP35 - nucleotide-binding protein |
| 22c00086 | 0.32613 | ni | hypothetical protein | um02113 | 0.22904 | ni | hypothetical protein |
| 20c00004 | 0.32618 | K10771 | apurinic/apyrimidinic endonuclease | um11371 | 0.22913 | ni | putative protein |
| 22c00256 | 0.32621 | ni | succinyl-coa synthetase, alpha subunit | um11278 | 0.2292 | ni | probable PHO88 - involved in phosphate transport |
| 2d00074 | 0.32644 | ni | hypothetical protein | um02237 | 0.22927 | ni | conserved hypothetical protein |
| 7c00205 | 0.32649 | ni | DNA helicase | um02041 | 0.22927 | ni | putative protein |
| 3c00025 | 0.3265 | K07441 | predicted glycosyltransferase | um11910 | 0.22947 | ni | hypothetical protein |
| 10d00110 | 0.32721 | ni | hypothetical protein | um10760 | 0.22962 | ni | conserved hypothetical protein |
| 2c00079 | 0.32728 | K01872 | alanyl-trna synthetase | um04265 | 0.22984 | K08334 | related to Beclin 1 |
| 5c00106 | 0.32732 | K11137 | hypothetical protein | um05262 | 0.22988 | K11763 | conserved hypothetical protein |
| 8d00036 | 0.32735 | ni | hypothetical protein | um06087 | 0.2299 | K02603 | related to origin recognition protein Orc1p |
| 25c00031 | 0.32736 | K12462 | rho GDP-dissociation inhibitor | um11592 | 0.23013 | ni | conserved hypothetical protein |
| 8d00051 | 0.32743 | K01046 | hypothetical protein | um12207 | 0.23017 | ni | putative protein |
| 8d00099 | 0.32747 | ni | triglyceride lipase-cholesterol esterase | um03019.2 | 0.23033 | ni | conserved hypothetical protein |
| 5c00164 | 0.32758 | K00128 | aldehyde dehydrogenase | um10941 | 0.23057 | ni | conserved hypothetical protein |
| 9d00167 | 0.32783 | ni | beta-tubulin folding cofactor D | um01613 | 0.23074 | ni | related to AUR1 - inositol phosphorylceramide synthase |
| 9c00339 | 0.32789 | ni | hypothetical protein | um01169 | 0.23088 | ni | related to CDC50 - cell division protein |
| 26d00082 | 0.32789 | ni | hypothetical protein | um10827 | 0.23094 | K14797 | related to ENP1 - required for pre-rRNA processing and 40S ribosomal subunit synthesis |
| 7c00085 | 0.32791 | K12855 | HAT repeat protein | um10333 | 0.23104 | ni | conserved hypothetical protein |
| 11d00029 | 0.32794 | ni | hypothetical protein | um12197 | 0.23132 | ni | putative protein |
| 7c00062 | 0.32841 | ni | MEKK and related serine/threonine protein kinases | um02874 | 0.23143 | K10901 | related to SGS1 - DNA helicase |
| 9d00393 | 0.32841 | ni | RAVE (regulator of V-ATPase assembly) complex subunit RAV1/ | um11000 | 0.23145 | ni | conserved hypothetical protein |
| 9c00008 | 0.32843 | ni | hypothetical protein | um10862 | 0.2315 | K10578 | probable UBC6 - E2 ubiquitin-conjugating enzyme |
| 2d00004 | 0.32845 | ni | hypothetical protein | um11680 | 0.2315 | ni | conserved hypothetical protein |
| 3d00099 | 0.32855 | K02209 | DNA replication licensing factor, MCM5 component | um15078 | 0.23154 | ni | conserved hypothetical protein |
| 5c00048 | 0.32865 | ni | hypothetical protein | um02717 | 0.23157 | ni | related to putative C2H2 transcriptional regulator |
| 7d00335 | 0.32873 | K05542 | tRNA-dihydrouridine synthase | um00628 | 0.23213 | ni | putative protein |
| 10d00082 | 0.32905 | K15117 | mitochondrial oxaloacetate carrier protein | um10069 | 0.23222 | ni | hypothetical protein |
| 12c00026 | 0.32912 | ni | hypothetical protein | um06061 | 0.2325 | ni | conserved hypothetical protein |
| 12c00046 | 0.32948 | K06185 | predicted transporter | um03763 | 0.23252 | K00384 | probable TRR2 - thioredoxin-disulfide reductase |
| 11c00073 | 0.32958 | ni | hypothetical protein | um03057 | 0.23256 | K03177 | related to tRNA pseudouridine synthase B |
| 9d00131 | 0.32974 | ni | hypothetical protein | um06412 | 0.2326 | ni | related to Paxillin |
| 3d00070 | 0.32981 | ni | hypothetical protein | um01076 | 0.23279 | ni | conserved hypothetical protein |
| 22c00036 | 0.32983 | K02905 | 60S ribosomal protein L29 | um12001 | 0.23283 | ni | probable NADH-ubiquinone oxidoreductase 12 kDa subunit |
| 13c00102 | 0.32991 | ni | predicted ATPase | um10719 | 0.23292 | ni | conserved hypothetical protein |
| 6c00102 | 0.33 | K01802 | phosphotyrosyl phosphatase activator | um06141 | 0.23293 | ni | hypothetical protein |
| 14d00100 | 0.33009 | ni | hypothetical protein | um06475 | 0.2334 | ni | hypothetical protein |
| 9d00015 | 0.3301 | K10703 | protein tyrosine phosphatase-like protein PTPLA | um01946 | 0.23351 | ni | putative protein |
| 9d00137 | 0.3301 | ni | M-phase inducer phosphatase | um11481 | 0.23357 | K09885 | related to aquaporin |
| 8c00130 | 0.33051 | ni | hypothetical protein | um00482 | 0.23373 | ni | putative protein |
| 8c00088 | 0.3307 | K02992 | ribosomal protein S7 | um10257 | 0.23377 | K09648 | related to inner mitochondrial membrane peptidase 2 |
| 24d00028 | 0.33075 | ni | hypothetical protein | um11697 | 0.23378 | ni | conserved hypothetical protein |
| 10d00022 | 0.33092 | ni | vigilin | um02755 | 0.23385 | ni | conserved hypothetical protein |
| 11c00039 | 0.331 | ni | hypothetical protein | um04254 | 0.23393 | K16489 | conserved hypothetical protein |
| 5d00133 | 0.33102 | K06677 | chromosome condensation complex Condensin, subunit D2 | um02053 | 0.23416 | K06671 | related to Nuclear cohesin complex subunit |
| 9c00201 | 0.33111 | ni | hypothetical protein | um04079 | 0.23423 | ni | hypothetical protein |
| 9c00437 | 0.33119 | K12176 | COP9 signalosome, subunit CSN2 | um05334 | 0.23437 | K03015 | probable DNA-directed RNA polymerase II chain Rpb7 |
| 5d00111 | 0.3314 | ni | hypothetical protein | um02414 | 0.23455 | K05545 | related to A |
| 22d00235 | 0.33142 | ni | hypothetical protein | um10192 | 0.23462 | ni | related to TMS1 protein |
| 19d00085 | 0.33143 | ni | hypothetical protein | um02439 | 0.23469 | K14816 | conserved hypothetical protein |
| 7d00256 | 0.33158 | K00102 | proteins containing the FAD binding domain | um03097 | 0.2348 | ni | related to Mimitin |
| 22d00022 | 0.3316 | ni | hypothetical protein | um03851 | 0.23487 | K01939 | probable ADE12 - adenylosuccinate synthetase |
| 3d00037 | 0.33163 | K03676 | glutaredoxin and related proteins | um12147.2 | 0.23502 | ni | conserved hypothetical protein |
| 18c00030 | 0.33168 | ni | hypothetical protein | um00355 | 0.23521 | ni | related to SGT1 protein |
| 9c00273 | 0.33189 | ni | predicted phospholipase | um03875 | 0.2353 | ni | conserved hypothetical protein |
| 11d00015 | 0.33206 | K11352 | NADH:ubiquinone oxidoreductase, B17.2 subunit | um03433 | 0.2355 | ni | putative protein |
| 7d00307 | 0.33212 | ni | hypothetical protein | um04758 | 0.23569 | K10598 | related to Peptidyl-prolyl cis-trans isomerase |
| 6d00094 | 0.33217 | K00681 | gamma-glutamyltransferase | um01316 | 0.23586 | ni | related to CLC1 - clathrin light chain |
| 9d00374 | 0.33226 | ni | DNA repair protein RAD18 | um04723 | 0.23611 | K14963 | probable wd-repeat protein 5 |
| 26d00019 | 0.3323 | ni | ankyrin | um15011.2 | 0.2362 | K04728 | related to TEL1 - telomere length control protein |
| 20c00012 | 0.33278 | ni | hypothetical protein | um02531 | 0.23632 | ni | related to nucleotide diphosphatase |
| 6d00097 | 0.33305 | ni | hypothetical protein | um03254 | 0.23649 | ni | conserved hypothetical protein |
| 7d00063 | 0.33318 | ni | uncharacterized conserved protein | um06316 | 0.2365 | ni | related to GWT1 - GPI-anchored wall transfer protein |
| 18d00007 | 0.33325 | K13352 | peroxisomal biogenesis protein | um02062 | 0.23658 | ni | related to multidrug resistance proteins |
| 13d00066 | 0.33328 | K00940 | nucleoside diphosphate kinase | um04318 | 0.23671 | K05906 | related to Prenylcysteine oxidase precursor |
| 19c00043 | 0.33328 | ni | lysophospholipase | um00890 | 0.23672 | ni | putative protein |
| 22c00155 | 0.33333 | ni | cytochrome P450 CYP4/CYP19/CYP26 subfamilies | um03471 | 0.23679 | ni | putative protein |
| 4d00032 | 0.33337 | ni | predicted fumarylacetoacetate hydralase | um06377 | 0.23684 | K03259 | related to translation initiation factor eIF-4E |
| 20c00056 | 0.33359 | K11664 | hypothetical protein | um01524 | 0.2369 | ni | hypothetical protein |
| 12c00050 | 0.3339 | ni | hypothetical protein | um11467 | 0.23692 | ni | conserved hypothetical protein |
| 14d00023 | 0.33393 | ni | predicted E3 ubiquitin ligase | um00578 | 0.23698 | ni | b mating type locus |
| 18d00054 | 0.33417 | ni | hypothetical protein | um10772 | 0.23706 | ni | putative protein |
| 22c00051 | 0.33419 | ni | nuclear transport receptor KAP120 | um04033 | 0.23709 | ni | hypothetical protein |
| 14d00102 | 0.33426 | ni | hypothetical protein | um04029 | 0.23715 | ni | conserved hypothetical protein |
| 19d00142 | 0.33432 | ni | hypothetical protein | um05912 | 0.23727 | K04799 | probable DNA repair endonuclease rad2 |
| 10c00014 | 0.33448 | ni | uncharacterized conserved protein | um11005 | 0.23731 | K02259 | related to COX15 - cytochrome oxidase assembly factor |
| 16d00067 | 0.33464 | ni | SPRY domain-containing proteins | um06193 | 0.23732 | K11294 | related to NSR1 - nuclear localization sequence binding protein |
| 20c00051 | 0.33465 | ni | hypothetical protein | um05841 | 0.23748 | K15105 | related to calcium-binding mitochondrial carrier protein |
| 7c00275 | 0.33471 | K07140 | uncharacterized Fe-S protein | um05454 | 0.23757 | K12829 | probable Splicing factor 3b |
| 7c00250 | 0.3348 | K02964 | ribosomal protein S18 | um03194 | 0.23763 | ni | putative protein |
| 3d00025 | 0.33498 | K00888 | phosphatidylinositol 4-kinase | um04984 | 0.23763 | ni | conserved hypothetical protein |
| 11d00041 | 0.3351 | ni | uncharacterized conserved protein | um12186 | 0.23776 | K00228 | probable HEM13 - coproporphyrinogen III oxidase |
| 7c00191 | 0.33519 | ni | hypothetical protein | um05512 | 0.2378 | K13110 | related to microfibril-associated protein |
| 7c00074 | 0.33523 | ni | hypothetical protein | um01682 | 0.23827 | K12861 | conserved hypothetical protein |
| 9d00399 | 0.33525 | ni | uncharacterized conserved protein | um02340 | 0.2386 | K11843 | related to ubiquitin-specific protease |
| 9c00045 | 0.33533 | ni | hypothetical protein | um04827 | 0.23862 | K01090 | probable PPZ2 - protein ser |
| 26d00029 | 0.33546 | K15028 | hypothetical protein | um03969 | 0.23872 | ni | related to nuclear envelope protein NEM1 |
| 14c00042 | 0.33551 | ni | hypothetical protein | um00983 | 0.23879 | ni | conserved hypothetical protein |
| 5c00059 | 0.33569 | ni | cyclin | um05499 | 0.23887 | ni | conserved hypothetical protein |
| 7c00082 | 0.33582 | K14288 | nuclear mRNA export factor receptor LOS1/Exportin-t | um10020 | 0.23888 | ni | conserved hypothetical protein |
| 8d00037 | 0.33588 | ni | uncharacterized conserved protein | um04539 | 0.23892 | ni | related to POS5 - Mitochondrial NADH kinase |
| 22c00090 | 0.33596 | ni | kynurenine aminotransferase | um00095 | 0.23893 | ni | related to Protein dltE |
| 6c00110 | 0.33635 | ni | hypothetical protein | um03477 | 0.23897 | K17263 | conserved hypothetical protein |
| 9c00190 | 0.33638 | K08492 | SNARE protein Syntaxin 18/UFE1 | um01757 | 0.23897 | ni | hypothetical protein |
| 6c00077 | 0.33653 | K15430 | putative RNA methylase | um00001 | 0.23898 | ni | ni |
| 20c00009 | 0.33662 | ni | uncharacterized conserved protein | um03758 | 0.23905 | K02220 | b-type cyclin 1 |
| 9d00031 | 0.33662 | ni | N-methyltransferase | um04700 | 0.23908 | ni | conserved hypothetical protein |
| 12d00029 | 0.33667 | K15216 | RNA polymerase I transcription factor | um06067 | 0.23929 | K09495 | probable CCT3 - chaperonin of the TCP1 ring complex |
| 20d00050 | 0.33672 | ni | amidases | um04646 | 0.23945 | ni | conserved hypothetical protein |
| 9d00165 | 0.33673 | ni | hypothetical protein | um02607 | 0.23964 | ni | probable ISW2 - ATPase component of a two subunit chromatin remodeling complex |
| 19d00120 | 0.33675 | K03022 | DNA-directed RNA polymerase subunit E' | um10969 | 0.23969 | ni | conserved hypothetical protein |
| 7c00198 | 0.33688 | ni | hypothetical protein | um00048 | 0.23977 | ni | conserved hypothetical protein |

| | | | | | | | |
|---|---|---|---|---|---|---|---|
| 19d00090 | 0.33715 | K02985 | 40S ribosomal protein S3 | um02249 | 0.23998 | ni | probable vacuolar protein sorting protein VpsB |
| 7c00217 | 0.33725 | ni | hypothetical protein | um12041 | 0.24001 | ni | related to LSB6 - Phosphatidylinositol 4-kinase |
| 2d00025 | 0.33733 | ni | hypothetical protein | um10808 | 0.24005 | ni | putative protein |
| 9d00295 | 0.33733 | K13093 | transcription elongation factor TAT-SF1 | um00062 | 0.2401 | ni | hypothetical protein |
| 3d00048 | 0.33739 | ni | hypothetical protein | um04804 | 0.24024 | ni | hypothetical protein |
| 1d00018 | 0.3374 | ni | hypothetical protein | um01281 | 0.24035 | K15378 | related to general alpha-glucoside permease |
| 4d00004 | 0.33741 | K15109 | mitochondrial carnitine-acylcarnitine carrier protein | um05364 | 0.2404 | K12821 | related to Formin binding protein 3 |
| 7d00182 | 0.33743 | K12811 | RNA helicase | um11674 | 0.24048 | K05355 | related to COQ1 - hexaprenyl pyrophosphate synthetase precursor |
| 22c00230 | 0.33752 | K04794 | uncharacterized conserved protein | um02474 | 0.24065 | ni | conserved hypothetical Ustilago-specific protein |
| 8c00036 | 0.33752 | ni | molybdenum cofactor sulfurase | um03956 | 0.24076 | K03964 | conserved hypothetical protein |
| 5d00047 | 0.33756 | ni | hypothetical protein | um02506 | 0.24082 | ni | conserved hypothetical protein |
| 13c00009 | 0.33785 | K12200 | predicted signal transduction protein | um01701 | 0.2409 | ni | hypothetical protein |
| 8c00060 | 0.33786 | K00128 | aldehyde dehydrogenase | um02666 | 0.24097 | ni | putative protein |
| 2c00024 | 0.338 | ni | ni | um11692 | 0.241 | K16575 | probable centractin |
| 5d00048 | 0.33802 | K08738 | cytochrome c | um12212 | 0.24138 | ni | related to ATP10 - F1F0 ATPase complex assembly protein |
| 8d00081 | 0.33805 | ni | hypothetical protein | um10115 | 0.24143 | ni | hypothetical protein |
| 7d00089 | 0.33816 | ni | hypothetical protein | um04599 | 0.24143 | K12849 | related to PRP38A - pre-mRNA-splicing factor |
| 9d00164 | 0.33822 | K13119 | uncharacterized conserved protein XAP-5 | um11606 | 0.24149 | ni | putative protein |
| 24c00038 | 0.33829 | K12882 | nuclear cap-binding complex, subunit NCBP1/CBP80 | um05456 | 0.24156 | ni | conserved hypothetical protein |
| 19c00050 | 0.33836 | K00275 | pyridoxamine-phosphate oxidase | um10121 | 0.24162 | ni | putative protein |
| 7d00062 | 0.33842 | ni | hypothetical protein | um15076 | 0.24163 | ni | hypothetical protein |
| 14c00040 | 0.3386 | K05759 | profilin | um11495 | 0.2417 | K03956 | related to nadh-ubiquinone oxidoreductase 21 |
| 22d00079 | 0.33864 | K02963 | hypothetical protein | um06428 | 0.24204 | K01078 | related to Thiamine-repressible acid phosphatase precursor |
| 22d00202 | 0.33864 | ni | cytosolic sorting protein GGA2/TOM1 | um11671.2 | 0.24224 | ni | putative protein |
| 11d00089 | 0.33868 | ni | sulfatase | um11407 | 0.24224 | ni | conserved hypothetical protein |
| 12d00043 | 0.33871 | K14967 | histone H3 (Lys4) methyltransferase complex, subunit CPS60/AS | um02429 | 0.24225 | ni | putative protein |
| 7c00243 | 0.339 | K00823 | acetylornithine aminotransferase | um11169 | 0.24231 | ni | conserved hypothetical protein |
| 19c00111 | 0.33903 | K15437 | tRNA-binding protein | um02015 | 0.24265 | K00876 | related to uridine kinase |
| 26d00068 | 0.33916 | ni | hypothetical protein | um02827 | 0.24278 | ni | conserved hypothetical protein |
| 20d00018 | 0.33947 | K00162 | pyruvate dehydrogenase E1, beta subunit | um03777 | 0.24281 | K11841 | related to Ubiquitin carboxyl-terminal hydrolase 10 |
| 2c00036 | 0.33975 | ni | hypothetical protein | um10132 | 0.24281 | ni | probable NHP6B - nonhistone chromosomal protein |
| 15c00041 | 0.3398 | ni | uncharacterized conserved protein | um02018 | 0.24299 | ni | conserved hypothetical protein |
| 22d00278 | 0.3404 | ni | hypothetical protein | um06140.2 | 0.24328 | ni | related to programmed cell death protein |
| 9d00361 | 0.3406 | K03942 | NADH:ubiquinone oxidoreductase, NDUFV1/51kDa subunit | um15046 | 0.24331 | ni | conserved hypothetical protein |
| 14d00008 | 0.34103 | K11572 | hypothetical protein | um00393 | 0.24332 | K12599 | probable SKI2 - putative RNA helicase |
| 22c00241 | 0.34116 | ni | inositol-1,4,5-triphosphate 5-phosphatase | um05391 | 0.24347 | ni | conserved hypothetical protein |
| 7d00174 | 0.34143 | ni | hypothetical protein | um03252 | 0.24354 | K03033 | probable 26S proteasome non-ATPase regulatory subunit p58 |
| 22d00126 | 0.34153 | K00143 | non-ribosomal peptide synthetase | um11302 | 0.24413 | ni | related to p33ING1b |
| 4d00008 | 0.34158 | ni | hypothetical protein | um05506 | 0.24428 | K15280 | conserved hypothetical protein |
| 12d00038 | 0.34173 | ni | hypothetical protein | um02419 | 0.24438 | ni | conserved hypothetical protein |
| 5d00108 | 0.34198 | ni | hypothetical protein | um00741 | 0.24452 | K03125 | related to TAF1 - TFIID subunit |
| 12c00104 | 0.34205 | K12581 | mRNA deadenylase subunit | um02156.2 | 0.24453 | ni | conserved hypothetical protein |
| 5c00077 | 0.34212 | ni | hypothetical protein | um04355 | 0.24454 | K14026 | related to Sel-1 homolog precursor |
| 10d00066 | 0.34223 | ni | hypothetical protein | um15070 | 0.24468 | ni | probable to GDP |
| 7c00094 | 0.34234 | ni | histone acetyltransferase SAGA/ADA, catalytic subunit PCAF/GC | um04248 | 0.24479 | ni | putative protein |
| 12c00084 | 0.34243 | ni | hypothetical protein | um01501 | 0.24486 | K09704 | conserved hypothetical protein |
| 14c00102 | 0.34246 | ni | RAN guanine nucleotide release factor | um10006 | 0.24487 | K14002 | related to SCJ1 protein |
| 8c00131 | 0.34262 | ni | hypothetical protein | um03145 | 0.24495 | K12590 | conserved hypothetical protein |
| 11d00011 | 0.34308 | ni | hypothetical protein | um02540 | 0.24504 | ni | hypothetical protein |
| 12c00119 | 0.34357 | ni | hypothetical protein | um05427 | 0.24513 | ni | ni |
| 22d00232 | 0.34358 | ni | aspartyl-trna synthetase | um02202 | 0.24517 | ni | putative protein |
| 26c00019 | 0.34363 | ni | heterochromatin-associated protein HP1 and related CHROMO d | um10956 | 0.24527 | K02942 | probable RPP1A - 60S large subunit acidic ribosomal protein a1 |
| 7c00248 | 0.34379 | ni | hypothetical protein | um04916 | 0.24544 | ni | putative protein |
| 7d00241 | 0.34384 | K08773 | rac GTPase-activating protein BCR/ABR | um00592 | 0.24546 | K04508 | related to Nuclear receptor co-repressor |
| 8d00077 | 0.34384 | ni | transcription-associated recombination protein - Thp1p | um05481 | 0.24563 | ni | conserved hypothetical protein |
| 10c00030 | 0.34389 | ni | hypothetical protein | um03815 | 0.24572 | K01868 | probable MST1 - threonine--tRNA ligase |
| 14c00050 | 0.34395 | ni | hypothetical protein | um05876 | 0.2461 | ni | putative protein |
| 14d00024 | 0.34408 | ni | FOG, FHA domain | um11213 | 0.24612 | K12591 | related to RRP6 - Exonuclease component of the nuclear exosome |
| 15d00011 | 0.34414 | ni | predicted transporter | um10176 | 0.2464 | K04567 | probable lysyl-tRNA synthetase |
| 14c00036 | 0.34417 | K14018 | phospholipase A2-activating protein | um05680 | 0.24656 | ni | putative protein |
| 25d00095 | 0.34422 | K01811 | glucosidase II catalytic (alpha) subunit | um15058 | 0.24659 | ni | conserved hypothetical protein |
| 9c00361 | 0.34431 | ni | uncharacterized conserved protein | um00508 | 0.24667 | K02155 | probable CUP5 - proteolipid subunit of the vacuolar H |
| 11d00001 | 0.34433 | ni | hypothetical protein | um04676 | 0.2467 | ni | putative protein |
| 25c00053 | 0.34445 | ni | hypothetical protein | um15102 | 0.24691 | K14311 | putative protein |
| 13d00021 | 0.34484 | ni | permease of the major facilitator superfamily | um05679 | 0.24719 | ni | related to Protein-tyrosine phosphatase gamma precursor |
| 11c00084 | 0.34485 | ni | reductases with broad range of substrate specificities | um11702 | 0.24754 | ni | conserved hypothetical protein |
| 1d00012 | 0.34495 | ni | hypothetical protein | um01616 | 0.24775 | K02902 | related to MRPL24 - mitochondrial ribosomal protein |
| 7c00146 | 0.34497 | K12622 | small nuclear ribonucleoprotein | um02222 | 0.24776 | ni | related to membrane protein Dik6 |
| 8c00127 | 0.34507 | ni | hypothetical protein | um05930 | 0.24778 | ni | conserved hypothetical Ustilago-specific protein |
| 16d00065 | 0.34509 | ni | cyclin | um05318 | 0.2478 | ni | hypothetical protein |
| 1d00023 | 0.34519 | ni | hypothetical protein | um10895 | 0.24796 | K01120 | conserved hypothetical protein |
| 5d00074 | 0.34531 | ni | hypothetical protein | um03618 | 0.24797 | K00877 | related to THI21 - Hydroxymethylpyrimidine phosphate kinase |
| 9c00042 | 0.34544 | K01011 | mercaptopyruvate sulfurtransferase | um01152 | 0.24828 | ni | conserved hypothetical protein |
| 8d00087 | 0.34556 | K10416 | dynein light intermediate chain | um03598 | 0.24843 | K12471 | related to ENT2 - clathrin binding protein |
| 14c00024 | 0.34586 | K02684 | eukaryotic-type DNA primase, catalytic | um00064 | 0.24882 | ni | related to pepsin precursor |
| 5c00025 | 0.34588 | ni | phosphoglycerate mutase | um06251 | 0.24883 | ni | Kinesin-3 motor protein |
| 19c00095 | 0.34613 | ni | hypothetical protein | um00210 | 0.24928 | ni | conserved hypothetical protein |
| 8c00129 | 0.34628 | K01188 | beta-glucosidase, lactase phlorizinhydrolase | um04152 | 0.24952 | K03235 | probable YEF3 - translation elongation factor eEF3 |
| 13d00065 | 0.34629 | ni | reductases with broad range of substrate specificities | um03172 | 0.24972 | ni | related to Zinc finger protein |
| 14c00081 | 0.34634 | ni | hypothetical protein | um04084 | 0.24975 | ni | related to Per-hexamer repeat protein 5 |
| 9c00405 | 0.34662 | ni | hypothetical protein | um02719 | 0.2498 | ni | related to DJP1 - DnaJ-like protein |
| 20c00075 | 0.34664 | ni | WD repeat protein WDR4 | um02863 | 0.25017 | ni | conserved hypothetical protein |
| 27c00060 | 0.3467 | K01855 | pseudouridylate synthase | um01381 | 0.25041 | ni | conserved hypothetical protein |
| 16c00021 | 0.34673 | ni | predicted E3 ubiquitin ligase | um10442 | 0.25044 | K11558 | conserved hypothetical protein |
| 2d00026 | 0.3469 | K02981 | 40S ribosomal protein S2/30S ribosomal protein S5 | um01131 | 0.25044 | K01669 | related to Deoxyribodipyrimidine photo-lyase |
| 20c00019 | 0.34691 | ni | hypothetical protein | um04061 | 0.25046 | ni | related to glycerate dehydrogenase |
| 6d00055 | 0.34714 | ni | hypothetical protein | um06265 | 0.25062 | ni | related to Retrovirus-related POL polyprotein |
| 13d00070 | 0.34714 | ni | inositol polyphosphate 5-phosphatase and related proteins | um01982 | 0.25073 | ni | related to aminotriazole resistance protein |
| 9d00274 | 0.34722 | K03083 | glycogen synthase kinase-3 | um00862 | 0.25077 | K02875 | probable RPL14B - ribosomal protein |
| 5c00120 | 0.34723 | K01535 | plasma membrane H+-transporting ATPase | um11968 | 0.25081 | ni | related to MKT1 - retroviral protease signature protein |
| 12d00021 | 0.34725 | ni | hypothetical protein | um10624.2 | 0.25083 | ni | related to LCB2 - serine C-palmitoyltransferase subunit |
| 11c00040 | 0.34726 | ni | hypothetical protein | um11983 | 0.25084 | K12834 | probable Pre-mRNA splicing factor ini1 |
| 6c00067 | 0.34767 | ni | hypothetical protein | um06429.2 | 0.25098 | ni | conserved hypothetical protein |
| 13d00101 | 0.34768 | K14830 | WD40 repeat-containing protein | um04826 | 0.25105 | K01497 | related to RIB1 - GTP cyclohydrolase II |
| 24d00001 | 0.3478 | ni | enoyl-coa hydratase | um06450 | 0.25112 | K04739 | cAMP-dependent protein kinase type II regulatory chain |
| 7d00297 | 0.34785 | ni | hypothetical protein | um04168 | 0.25119 | K11360 | related to Transcription factor SPT8 |
| 22d00194 | 0.34789 | K01078 | lysosomal & prostatic acid phosphatases | um01559 | 0.25141 | ni | conserved hypothetical protein |
| 9d00278 | 0.34791 | K14758 | casein kinase | um02118 | 0.25147 | ni | putative protein |
| 3d00089 | 0.3484 | K14850 | predicted RNA methylase | um00145 | 0.25152 | K08851 | related to p53-related protein kinase |
| 14d00015 | 0.34855 | K01689 | enolase | um01612 | 0.25155 | ni | conserved hypothetical protein |
| 18d00091 | 0.34867 | ni | hypothetical protein | um12301 | 0.25155 | ni | putative protein |
| 19c00061 | 0.34876 | ni | uncharacterized conserved protein | um00342 | 0.25181 | ni | hypothetical protein |
| 8c00066 | 0.3488 | ni | predicted methyltransferase | um11855.2 | 0.25181 | ni | conserved hypothetical protein |
| 9c00210 | 0.3488 | K11569 | hypothetical protein | um03644 | 0.25183 | ni | hypothetical protein |
| 18d00085 | 0.34884 | ni | hypothetical protein | um10536 | 0.25201 | K09569 | related to FPR2 - FK506 |
| 7c00300 | 0.34897 | K11366 | ubiquitin-specific protease | um05584 | 0.2521 | K00963 | probable UTP-glucose-1-phosphate uridylyltransferase |
| 22c00247 | 0.34921 | ni | hypothetical protein | um02231 | 0.25217 | ni | conserved hypothetical Ustilago-specific protein |
| 22c00220 | 0.34953 | ni | hypothetical protein | um02194 | 0.25223 | ni | hypothetical protein |
| 22d00246 | 0.34958 | ni | hypothetical protein | um10623 | 0.25232 | ni | related to Tuberin |
| 22d00003 | 0.34959 | K00787 | polyprenyl synthetase | um11336 | 0.25236 | ni | conserved hypothetical protein |
| 12d00095 | 0.34964 | ni | hypothetical protein | um02137 | 0.25252 | ni | effector family protein Eff1-7 |
| 25d00010 | 0.34973 | ni | pleiotropic drug resistance proteins | um01241 | 0.25254 | ni | hypothetical protein |
| 25d00091 | 0.34979 | ni | predicted transporter | um00569 | 0.25259 | ni | related to GTP-binding protein |
| 3d00015 | 0.34981 | ni | predicted ER membrane protein | um00097 | 0.25278 | ni | ni |
| 22c00267 | 0.34993 | K01477 | hypothetical protein | um04563 | 0.25281 | ni | ni |
| 18c00089 | 0.34996 | ni | reductases with broad range of substrate specificities | um02580.2 | 0.25303 | ni | conserved hypothetical protein |
| 7d00021 | 0.34998 | K06911 | hypothetical protein | um04365 | 0.25315 | ni | conserved hypothetical protein |
| 18c00058 | 0.35004 | ni | WD40 repeat-containing protein | um03413 | 0.25318 | K00925 | related to Acetate kinase |
| 22c00027 | 0.35007 | K04713 | sphingolipid hydroxylase | um10334 | 0.25349 | K02985 | probable 40S ribosomal protein S3 |
| 482d00001 | 0.35009 | K00412 | ni | um00085 | 0.25358 | ni | conserved hypothetical protein |
| 22d00245 | 0.35016 | ni | hypothetical protein | um10177 | 0.25391 | K04640 | guanine nucleotide-binding protein alpha-1 subunit |
| 4d00029 | 0.35023 | ni | hypothetical protein | um05264 | 0.25411 | ni | probable glutathione-dependent formaldehyde dehydrogenase |
| 24c00012 | 0.35029 | ni | hypothetical protein | um04067 | 0.25436 | K14830 | related to MAK11 protein |
| 22c00040 | 0.35031 | K11251 | histone 2A | um02272 | 0.25443 | ni | putative protein |
| 9d00027 | 0.35042 | ni | hypothetical protein | um12255 | 0.2545 | ni | conserved hypothetical protein |
| 6d00088 | 0.35046 | ni | hypothetical protein | um01166 | 0.25456 | ni | related to APG2 - required for sporulation |
| 19d00160 | 0.35047 | ni | tyrosine kinase specific for activated | um00553 | 0.25466 | ni | related to GTPase activating protein sec2 |
| 13d00013 | 0.35047 | K07407 | hypothetical protein | um03018 | 0.25494 | K15262 | related to BCP1 - Essential protein involved in nuclear export of cytoskeleton organization protein |
| 9d00106 | 0.35054 | ni | hypothetical protein | um11199 | 0.25499 | K08818 | related to CDC28 - cyclin-dependent protein kinase |
| 22d00296 | 0.35058 | K14860 | hypothetical protein | um12294 | 0.25504 | ni | conserved hypothetical protein |
| 25d00069 | 0.35082 | K00685 | arginyl-trna-protein transferase | um05149 | 0.25523 | ni | ni |
| 12d00049 | 0.35088 | ni | carboxylesterase and related proteins | um00731 | 0.25561 | ni | hypothetical protein |
| 19c00080 | 0.35092 | K06990 | predicted dioxygenase | um00645 | 0.25562 | ni | conserved hypothetical protein |
| 15c00051 | 0.35114 | K14530 | hypothetical protein | um10315 | 0.25581 | ni | putative protein |
| 19d00151 | 0.35114 | K00942 | guanylate kinase | um06239 | 0.25591 | ni | related to phosphatidylinositol 3-phosphate 5-kinase |
| 12c00103 | 0.35118 | K12581 | mRNA deadenylase subunit | um02803 | 0.25643 | ni | related to beta-1 |
| 3d00022 | 0.35119 | K10732 | predicted alpha-helical protein | um10697 | 0.25644 | ni | hypothetical protein |
| 13c00032 | 0.35138 | ni | hypothetical protein | um04544 | 0.25644 | ni | hypothetical protein |
| 5d00068 | 0.35164 | ni | tyrosine aminotransferase | um02144 | 0.25647 | K02295 | related to Deoxyribodipyrimidine photolyase |
| 7c00340 | 0.35165 | ni | hypothetical protein | um10209 | 0.25653 | ni | related to VPS9 |
| 18c00001 | 0.35172 | K14827 | uncharacterized conserved protein | um01214 | 0.25654 | ni | hypothetical protein |
| 12c00128 | 0.35175 | K02882 | 60S ribosomal protein L18A | um02112 | 0.25688 | ni | hypothetical protein |
| 9c00342 | 0.35196 | ni | hypothetical protein | um02297 | 0.25701 | ni | conserved hypothetical Ustilago-specific protein |
| 13c00030 | 0.35202 | K14457 | acyl-coa, diacylglycerol acyltransferase | um04838 | 0.25705 | K01823 | probable isopentenyl-diphosphate delta-isomerase |
| 4c00033 | 0.3521 | K14262 | metalloexopeptidases | um03409 | 0.2572 | ni | related to fumarylacetoacetate hydrolase |
| 24d00032 | 0.35215 | K15171 | transcription elongation factor SPT4 | um00240 | 0.25728 | K14548 | related to UTP4 - U3 snoRNP protein |
| 26c00061 | 0.35244 | K11093 | U1 small nuclear ribonucleoprotein | um01118 | 0.25729 | ni | hypothetical protein |
| 27c00098 | 0.35286 | K01098 | hypothetical protein | um03624 | 0.25732 | K15075 | related to Transcriptional coactivator MMS19 |
| 2c00062 | 0.35292 | ni | hypothetical protein | um03830 | 0.25732 | ni | conserved hypothetical protein |
| 19c00075 | 0.35323 | K10576 | ubiquitin-protein ligase | um00183 | 0.25735 | K14961 | related to SWD1 - subunit of the COMPASS complex |
| 19c00047 | 0.35324 | ni | uncharacterized conserved protein Sand | um02587 | 0.25737 | ni | related to SamB protein |
| 5d00140 | 0.35327 | ni | hypothetical protein | um11177 | 0.25742 | K10365 | probable CAP2 - F-actin capping protein |
| 16c00004 | 0.35344 | ni | hypothetical protein | um04965 | 0.25763 | K03104 | related to Signal recognition particle 14 kDa protein |
| 15c00057 | 0.35349 | ni | sortilin and related receptors | um10297 | 0.25764 | K12817 | related to pre-mrna splicing factor 18 |
| 12c00022 | 0.3535 | ni | hypothetical protein | um11126 | 0.25767 | ni | conserved hypothetical protein |
| 7c00069 | 0.35355 | K03241 | translation initiation factor 2B, gamma subunit | um02139 | 0.25774 | ni | effector family protein Eff1-9 |

| | | | | | | | |
|---|---|---|---|---|---|---|---|
| 9c00239 | 0.35372 | K00605 | aminomethyl transferase | um06513 | 0.25787 | ni | conserved hypothetical Ustilago-specific protein |
| 26c00076 | 0.35374 | K01262 | putative Xaa-Pro aminopeptidase | um06195 | 0.25794 | K14568 | related to EMG1 - Protein required for ribosome biogenesis |
| 6c00097 | 0.35378 | ni | hypothetical protein | um02196 | 0.25797 | ni | hypothetical protein |
| 13c00099 | 0.35393 | ni | hypothetical protein | um11453 | 0.25802 | K06689 | probable UBC5 - E2 ubiquitin-conjugating enzyme |
| 22d00270 | 0.35398 | K01725 | hypothetical protein | um02769 | 0.25838 | ni | conserved hypothetical protein |
| 5c00102 | 0.35399 | ni | hypothetical protein | um01942 | 0.25839 | K01273 | related to Microsomal dipeptidase precursor |
| 7d00291 | 0.35406 | K11121 | sirtuin 5 and related class III sirtuins | um10122 | 0.25846 | ni | hypothetical protein |
| 18c00005 | 0.35407 | ni | hypothetical protein | um15032 | 0.25865 | ni | related to ABC transporter |
| 6d00099 | 0.35408 | ni | hypothetical protein | um12177 | 0.25873 | K00854 | probable XKS1 - xylulokinase |
| 18c00019 | 0.35412 | ni | hypothetical protein | um00988 | 0.25878 | ni | conserved hypothetical protein |
| 10d00048 | 0.3542 | ni | hypothetical protein | um04080 | 0.25882 | K11594 | probable DED1 - ATP-dependent RNA helicase |
| 16d00045 | 0.35421 | K15148 | hypothetical protein | um11758 | 0.25886 | ni | conserved hypothetical protein |
| 6c00009 | 0.35423 | ni | hypothetical protein | um04549 | 0.25903 | K12587 | conserved hypothetical protein |
| 5c00092 | 0.35454 | K01560 | hypothetical protein | um05303 | 0.25906 | ni | hypothetical protein |
| 7c00289 | 0.35461 | K12659 | N-acetyl-gamma-glutamyl-phosphate reductase | um05027 | 0.25915 | ni | hypothetical protein |
| 5c00143 | 0.35469 | ni | hypothetical protein | um10597 | 0.25916 | K03648 | related to UNG1 - Uracil-DNA glycosylase |
| 9c00351 | 0.35471 | K12179 | COP9 signalosome, subunit CSN6 | um10577 | 0.25934 | ni | related to prefoldin subunit 3 |
| 8c00018 | 0.35485 | K04486 | hypothetical protein | um03099 | 0.25948 | ni | putative protein |
| 19c00114 | 0.35498 | ni | hypothetical protein | um02676 | 0.25957 | K12869 | probable protein CCN1 - putative cell cycle control protein |
| 15c00029 | 0.355 | ni | hypothetical protein | um00685 | 0.25959 | K11131 | probable CBF5 - Centromere Binding Factor |
| 15d00082 | 0.35502 | ni | hypothetical protein | um10497 | 0.2596 | ni | related to 3-ketosphinganine reductase |
| 24c00037 | 0.35514 | ni | ferric reductase-like proteins | um01245 | 0.25967 | K00052 | probable LEU2 - beta-isopropyl-malate dehydrogenase |
| 7c00331 | 0.3552 | K11321 | hypothetical protein | um15061.2 | 0.25977 | ni | conserved hypothetical protein |
| 7d00139 | 0.35522 | ni | hypothetical protein | um02021 | 0.26017 | ni | conserved hypothetical protein |
| 7c00318 | 0.35543 | ni | uncharacterized conserved protein | um01691 | 0.26022 | K03657 | related to ATP-dependent DNA helicase |
| 5d00107 | 0.35552 | ni | predicted transporter | um02483 | 0.26028 | ni | conserved hypothetical protein |
| 7d00140 | 0.35555 | ni | hypothetical protein | um00616 | 0.26036 | ni | putative protein |
| 1c00024 | 0.35564 | K11592 | dsRNA-specific nuclease Dicer and related ribonucleases | um05002 | 0.26042 | ni | related to 7alpha-cephem-methoxylase P8 chain |
| 14c00125 | 0.35567 | ni | hypothetical protein | um00419 | 0.26049 | ni | related to atp-dependent rna helicase |
| 6c00007 | 0.35568 | ni | sexual differentiation process protein ISP4 | um11224 | 0.26066 | ni | putative protein |
| 7d00314 | 0.35581 | K14807 | DEAD-box ATP-dependent RNA helicase | um01932 | 0.26073 | K10858 | related to PMS1 - DNA mismatch repair protein |
| 4d00026 | 0.35582 | ni | ATP-dependent RNA helicase pitchoune | um02365 | 0.26082 | K15100 | probable CTP1 - Mitochondrial citrate transporter - member of the mitochondrial carrier |
| 14c00021 | 0.35605 | ni | hypothetical protein | um02754 | 0.26083 | K07936 | related to GSP1 - GTP-binding protein of the ras superfamily |
| 25c00027 | 0.3562 | K00273 | D-aspartate oxidase | um05702 | 0.26094 | ni | related to oxidoreductase |
| 7d00137 | 0.3564 | ni | hypothetical protein | um03976 | 0.26094 | ni | related to PDR16 - involved in lipid biosynthesis and multidrug resistance |
| 7c00334 | 0.3564 | K00928 | aspartate kinase | um00952 | 0.261 | K13102 | related to RTS2 - Basic zinc-finger protein |
| 12c00065 | 0.35645 | K14571 | nuclear AAA ATPase | um05948 | 0.26102 | ni | hypothetical protein |
| 26c00024 | 0.35664 | ni | hypothetical protein | um12226 | 0.26112 | ni | putative protein |
| 22c00099 | 0.35665 | ni | hypothetical protein | um04815 | 0.26114 | ni | putative protein |
| 5c00146 | 0.35673 | ni | hypothetical protein | um03271 | 0.26116 | K14763 | related to NAF1 - nuclear assembly factor |
| 7d00273 | 0.35679 | K07827 | ras-related GTPase | um11657 | 0.26145 | ni | related to actin-interacting protein AIP3 |
| 10c00106 | 0.35687 | ni | sterol reductase | um10005 | 0.26147 | ni | conserved hypothetical protein |
| 19d00043 | 0.35702 | K03177 | pseudouridine synthase | um11411 | 0.2617 | K02974 | probable 40S RIBOSOMAL PROTEIN S24 |
| 8d00082 | 0.35715 | ni | hypothetical protein | um12116.2 | 0.26179 | ni | conserved hypothetical protein |
| 24d00015 | 0.35722 | K17417 | hypothetical protein | um01024 | 0.26182 | ni | hypothetical protein |
| 9d00309 | 0.35727 | K17257 | predicted membrane protein | um00284 | 0.26205 | K08515 | related to vesicle-associated membrane protein 7 |
| 16c00035 | 0.35729 | K02144 | vacuolar H+-ATPase V1 sector, subunit H | um04993 | 0.2621 | ni | putative protein |
| 3d00009 | 0.3573 | ni | predicted ubiquitin regulatory protein | um03811 | 0.26232 | K01057 | probable SOL1 - Protein with a possible role in tRNA export |
| 26c00008 | 0.35732 | K03000 | RNA polymerase I transcription factor TFIIS, subunit A12.2/RPA | um04654 | 0.26243 | K11092 | related to U2 snRNP protein A |
| 9d00042 | 0.35766 | ni | predicted MutS-related protein | um04472 | 0.2625 | K11135 | conserved hypothetical protein |
| 27d00003 | 0.35773 | ni | mitochondrial import inner membrane translocase, subunit TIM23 | um00399 | 0.26253 | ni | conserved hypothetical protein |
| 25c00087 | 0.35779 | K06978 | hypothetical protein | um00792 | 0.26264 | ni | conserved hypothetical Ustilago-specific protein |
| 22c00010 | 0.35782 | ni | hypothetical protein | um11645 | 0.26265 | K02266 | related to COX13 - cytochrome-c oxidase chain VIa |
| 18c00066 | 0.35789 | ni | hypothetical protein | um01706 | 0.26268 | ni | conserved hypothetical protein |
| 18c00027 | 0.35799 | ni | uncharacterized conserved protein | um02867 | 0.2628 | K07390 | probable GRX5 - glutaredoxin |
| 8d00101 | 0.35805 | K00485 | flavin-containing monooxygenase | um02792 | 0.26281 | K01697 | related to CYS4 - cystathionine beta-synthase |
| 6c00071 | 0.35818 | K15428 | metalloexopeptidases | um12171 | 0.26297 | ni | conserved hypothetical protein |
| 7c00032 | 0.35818 | ni | translation initiation factor 4F, helicase subunit | um11367 | 0.26305 | ni | conserved hypothetical protein |
| 9d00311 | 0.35821 | K03943 | NADH:ubiquinone oxidoreductase, NDUFV2/24 kD subunit | um00542 | 0.26307 | K10601 | conserved hypothetical protein |
| 9c00159 | 0.35853 | ni | nitrogen permease regulator NLRG/NPR2 | um02230 | 0.26318 | ni | conserved hypothetical Ustilago-specific protein |
| 24c00003 | 0.35857 | ni | hypothetical protein | um03313 | 0.26338 | ni | effector family protein Eff1-3 |
| 24d00019 | 0.3587 | K15414 | MAM33 | um11189 | 0.26355 | ni | conserved hypothetical protein |
| 16c00078 | 0.35871 | ni | sensory transduction histidine kinase | um12089 | 0.26358 | K08851 | related to p53-related protein kinase |
| 25d00008 | 0.35872 | ni | ypt/rab-specific GTPase-activating protein GYP1 | um11572 | 0.26369 | K14050 | related to Rab geranylgeranyltransferase alpha subunit |
| 7c00109 | 0.35877 | ni | hypothetical protein | um05573 | 0.26378 | ni | hypothetical protein |
| 7d00245 | 0.35883 | K00528 | ferredoxin/adrenodoxin reductase | um10397 | 0.26379 | K02133 | probable ATP2 - F1F0-ATPase complex |
| 6c00112 | 0.35883 | ni | hypothetical protein | um12148 | 0.26382 | ni | conserved hypothetical protein |
| 18d00111 | 0.35883 | ni | hypothetical protein | um10133 | 0.2639 | K10768 | conserved hypothetical protein |
| 22c00123 | 0.3594 | ni | hypothetical protein | um00801 | 0.26406 | K07204 | related to KOG1 - Subunit of TORC1 |
| 7c00353 | 0.35943 | ni | ferric reductase | um03525 | 0.26415 | ni | putative protein |
| 18d00012 | 0.35947 | ni | transporter, ABC superfamily | um10813 | 0.2642 | ni | conserved hypothetical protein |
| 22c00205 | 0.35952 | K14864 | SAM-dependent methyltransferase | um10447 | 0.26434 | K11275 | related to Histone H1 |
| 6c00030 | 0.35954 | ni | hypothetical protein | um05313 | 0.26439 | ni | hypothetical protein |
| 13d00018 | 0.35957 | ni | hypothetical protein | um01526 | 0.26441 | ni | conserved hypothetical protein |
| 12d00117 | 0.35957 | ni | ngg1-interacting factor 3 protein NIF3L1 | um05688 | 0.26455 | K12272 | conserved hypothetical protein |
| 22d00081 | 0.35958 | ni | uncharacterized conserved protein | um10428 | 0.2646 | K05956 | probable BET2 - geranylgeranyltransferase type II beta subunit |
| 7d00358 | 0.35962 | K13628 | fe-s cluster biosynthesis protein ISA1 | um11344 | 0.26464 | ni | conserved hypothetical protein |
| 9d00037 | 0.35963 | K03458 | hypothetical protein | um04836 | 0.26469 | ni | hypothetical protein |
| 11d00048 | 0.35968 | ni | hypothetical protein | um03268.2 | 0.2648 | K14809 | related to SPB4 - ATP-dependent RNA helicase of DEAH box family |
| 7c00230 | 0.35974 | ni | uncharacterized conserved protein | um10145 | 0.26491 | K08286 | p21-activated kinase |
| 15c00008 | 0.3598 | K14404 | polyadenylation factor I complex, subunit, Yth1 | um11523 | 0.26535 | ni | conserved hypothetical protein |
| 8c00075 | 0.35983 | K11884 | predicted RNA-binding protein Pno1p interacting with Nob1p | um11333 | 0.26535 | K03885 | related to NDE1 - mitochondrial cytosolically directed NADH dehydrogenase |
| 12c00093 | 0.35987 | ni | hypothetical protein | um06046 | 0.26555 | ni | conserved hypothetical protein |
| 2d00055 | 0.3599 | ni | hypothetical protein | um00365 | 0.26562 | ni | hypothetical protein |
| 9d00301 | 0.36003 | ni | hypothetical protein | um10184 | 0.26579 | ni | conserved hypothetical protein |
| 7c00317 | 0.3602 | K04523 | ubiquitin-like protein | um05438 | 0.26589 | ni | conserved hypothetical protein |
| 12c00142 | 0.36021 | K14003 | hypothetical protein | um11109 | 0.26605 | ni | conserved hypothetical Ustilago-specific protein |
| 18c00014 | 0.36023 | K09140 | uncharacterized conserved protein | um05256 | 0.2661 | ni | putative protein |
| 10c00021 | 0.3603 | ni | hypothetical protein | um03765 | 0.26621 | K01529 | probable DBP5 - ATP-dependent RNA helicase of the DEAD-box family involved in mRNA export |
| 7c00298 | 0.36048 | K08272 | conserved protein Mo25 | um06280 | 0.26631 | ni | related to J kappa-recombination signal binding protein |
| 26c00092 | 0.36048 | ni | hypothetical protein | um10996 | 0.26635 | ni | conserved hypothetical protein |
| 3d00096 | 0.36062 | K02935 | mitochondrial/chloroplast ribosomal protein L12 | um04702 | 0.2664 | K01764 | related to CYT2 - holocytochrome-c1 synthase |
| 8d00017 | 0.36082 | K03005 | RNA polymerase I 49 kDa subunit | um05148 | 0.26649 | K10884 | related to ATP-dependent DNA helicase II |
| 13c00049 | 0.36083 | ni | FOG, Predicted E3 ubiquitin ligase | um11073 | 0.26659 | ni | conserved hypothetical protein |
| 19c00093 | 0.36088 | ni | hypothetical protein | um10429 | 0.26659 | K03010 | probable DNA-dependent RNA polymerase II RPB140 |
| 10c00065 | 0.36102 | ni | hypothetical protein | um05809 | 0.26685 | ni | related to KRE6 - glucan synthase subunit |
| 22c00229 | 0.36119 | K01480 | arginase family protein | um03242 | 0.26685 | ni | hypothetical protein |
| 16d00031 | 0.36132 | K01920 | glutathione synthetase | um06043 | 0.26716 | ni | related to amidase |
| 10c00040 | 0.36141 | ni | hypothetical protein | um11818 | 0.26749 | ni | probable SDS22 - protein phosphatase 1 |
| 12c00131 | 0.36141 | ni | protein Mei2 | um10812 | 0.26751 | ni | conserved hypothetical protein |
| 24c00032 | 0.36144 | ni | DNA helicase, TBP-interacting protein | um11341 | 0.26765 | K13119 | related to ALG12 - alpha-1 |
| 7d00104 | 0.36148 | K07560 | D-Tyr-tRNA (Tyr) deacylase | um11964 | 0.26768 | K11426 | conserved hypothetical protein |
| 10c00048 | 0.36171 | K15544 | protein involved in transcription start site selection | um02093 | 0.26774 | ni | putative protein |
| 9d00306 | 0.36174 | K03061 | 26S proteasome regulatory complex | um02960 | 0.26775 | K03456 | probable ser |
| 9c00237 | 0.36185 | ni | hypothetical protein | um02147 | 0.26784 | ni | related to Cytoplasmic acetyl-CoA hydrolase 1 |
| 24d00017 | 0.3619 | ni | triglyceride lipase-cholesterol esterase | um03937 | 0.268 | K14457 | probable diacylglycerol acyltransferase type 2b |
| 6d00127 | 0.36195 | ni | uncharacterized conserved protein | um05424 | 0.26811 | ni | ni |
| 9c00028 | 0.36196 | K09528 | predicted molecular chaperone | um03476 | 0.26813 | ni | conserved hypothetical protein |
| 20c00036 | 0.36203 | ni | hypothetical protein | um10015 | 0.26815 | ni | conserved hypothetical protein |
| 25c00088 | 0.3621 | K09955 | hypothetical protein | um12269.2 | 0.2682 | ni | related to CYB2 - lactate dehydrogenase cytochrome b2 |
| 16c00016 | 0.36221 | K12586 | exosomal 3'-5' exoribonuclease complex, subunit Rrp43 | um12052 | 0.26833 | ni | b mating type locus |
| 7c00323 | 0.36222 | ni | hypothetical protein | um04889 | 0.26843 | ni | related to beta-1 |
| 3c00053 | 0.36228 | K00599 | carboxymethyl transferase | um10304 | 0.26855 | ni | putative protein |
| 8d00023 | 0.3624 | K14855 | notchless-like WD40 repeat-containing protein | um11176 | 0.26861 | ni | conserved hypothetical protein |
| 11d00088 | 0.36245 | ni | hypothetical protein | um10465.2 | 0.26897 | K00921 | probable FAB1 - phosphatidylinositol 3-phosphate 5-kinase |
| 22c00227 | 0.3625 | K01113 | hypothetical protein | um05314 | 0.26914 | ni | conserved hypothetical Ustilago-specific protein |
| 27c00077 | 0.3626 | ni | hypothetical protein | um10368 | 0.26961 | K09419 | related to Heat shock factor protein |
| 15c00058 | 0.36261 | K09265 | MADS box transcription factor | um03696 | 0.26969 | K04706 | related to SIZ1 - E3-like factor in the SUMO pathway |
| 9c00200 | 0.36264 | K02309 | protein kinase essential for the initiation of DNA replication | um00582 | 0.26978 | ni | related to cytoskeleton assembly control protein |
| 9d00128 | 0.36276 | ni | hypothetical protein | um15030 | 0.26994 | K14715 | conserved hypothetical protein |
| 14d00063 | 0.36282 | ni | hypothetical protein | um06466 | 0.27003 | ni | conserved hypothetical protein |
| 20c00022 | 0.36284 | ni | NADP-dependent isocitrate dehydrogenase | um03698 | 0.27007 | ni | conserved hypothetical protein |
| 9c00398 | 0.36289 | ni | hypothetical protein | um10355 | 0.27031 | K11876 | conserved hypothetical protein |
| 15c00040 | 0.363 | ni | hypothetical protein | um10482 | 0.27048 | K03123 | probable transcription initiation factor iia gamma chain |
| 5d00017 | 0.36301 | ni | hypothetical protein | um04166 | 0.27051 | ni | related to SHE9 - mitochondrial inner membrane protein |
| 27d00083 | 0.36308 | ni | hypothetical protein | um02888 | 0.27055 | ni | related to ADH6 - NADPH-dependent alcohol dehydrogenase |
| 2c00019 | 0.36319 | ni | hypothetical protein | um10418 | 0.27057 | ni | hypothetical protein |
| 9d00194 | 0.3633 | ni | hypothetical protein | um03628 | 0.27067 | ni | related to molybdenum cofactor sulfurase HxB protein |
| 5d00089 | 0.3634 | ni | predicted E3 ubiquitin ligase | um00126 | 0.27067 | ni | related to Chitin deacetylase 1 |
| 5c00140 | 0.36341 | K02604 | hypothetical protein | um15042 | 0.27069 | K12763 | related to ascospore maturation 1 protein |
| 18d00069 | 0.36343 | K12846 | nucleic acid binding protein | um06204 | 0.27076 | ni | ni |
| 9c00426 | 0.36345 | ni | hypothetical protein | um04713 | 0.27096 | K15326 | conserved hypothetical protein |
| 9d00207 | 0.36345 | K15710 | helicase-like transcription factor HLTF | um12248 | 0.27106 | ni | hypothetical protein |
| 9c00108 | 0.36351 | ni | hypothetical protein | um10964 | 0.27115 | ni | conserved hypothetical protein |
| 22c00082 | 0.36367 | ni | hypothetical protein | um10274 | 0.27122 | ni | putative protein |
| 7d00263 | 0.36373 | ni | hypothetical protein | um04388 | 0.27125 | ni | conserved hypothetical protein |
| 9d00371 | 0.36394 | ni | FYVE finger containing protein | um06223 | 0.27132 | ni | hypothetical protein |
| 11d00005 | 0.36402 | ni | hypothetical protein | um10116 | 0.27154 | ni | conserved hypothetical protein |
| 7d00214 | 0.36412 | ni | hypothetical protein | um00810 | 0.27155 | K16315 | conserved hypothetical protein |
| 10d00121 | 0.36416 | ni | uncharacterized conserved protein | um10398 | 0.27194 | ni | conserved hypothetical protein |
| 22c00285 | 0.36421 | ni | uncharacterized conserved protein | um12149 | 0.27205 | ni | hypothetical protein |
| 7d00138 | 0.36423 | ni | RNA-binding proteins | um11694 | 0.27216 | ni | conserved hypothetical protein |
| 15d00036 | 0.36433 | ni | WD40 repeat-containing protein | um05547 | 0.27228 | K03850 | related to Alpha-1 |
| 19c00110 | 0.36446 | K14012 | protein tyrosine phosphatase SHP1 | um05981 | 0.27232 | ni | related to TPO1 - Vacuolar polyamine-H antiporter |
| 19d00083 | 0.36448 | K12620 | small Nuclear ribonucleoprotein splicing factor | um10817 | 0.27248 | ni | conserved hypothetical protein |
| 19c00048 | 0.36463 | K06944 | GTP-binding protein DRG1 | um04230 | 0.27261 | K15192 | related to MOT1 - transcriptional accessory protein |
| 19d00158 | 0.36475 | ni | hypothetical protein | um10799 | 0.2728 | K12193 | related to VPS24 - endosomal Vps protein complex subunit |
| 25c00062 | 0.36509 | K12813 | mRNA splicing factor ATP-dependent RNA helicase | um10215 | 0.27281 | K15072 | conserved hypothetical protein |
| 3d00092 | 0.36528 | ni | hypothetical protein | um04146 | 0.27303 | ni | related to putative phosphate transporter 1 |
| 19d00042 | 0.36528 | K08266 | G-protein beta subunit-like protein | um02773 | 0.27315 | K02915 | probable RPL34B - ribosomal protein L34 |
| 10d00052 | 0.36535 | K00326 | NADH-cytochrome b-5 reductase | um10694 | 0.27318 | ni | conserved hypothetical protein |
| 27d00044 | 0.36543 | ni | predicted Zn-finger protein | um03264 | 0.27324 | K00106 | probable xanthine dehydrogenase |
| 27d00038 | 0.36543 | ni | hypothetical protein | um00334 | 0.27325 | K01068 | probable peroxisomal acyl-coenzyme A thioester hydrolase 1 |
| 9c00157 | 0.36546 | K02218 | casein kinase | um00666 | 0.27329 | ni | conserved hypothetical protein |
| 10d00047 | 0.36548 | ni | hypothetical protein | um06271 | 0.2735 | ni | conserved hypothetical protein |
| 9c00052 | 0.3655 | K03639 | molybdenum cofactor biosynthesis pathway protein | um00751 | 0.2739 | K11665 | related to INO80 - ATPase with chromatin remodeling and helicase activity |

| | | | | | | | |
|---|---|---|---|---|---|---|---|
| 9c00422 | 0.36562 | ni | hypothetical protein | um10915 | 0.27401 | K12813 | probable PRP2 - RNA-dependent ATPase of DEAH box family |
| 22d00038 | 0.36601 | K12733 | cyclophilin type peptidyl-prolyl cis-trans isomerase | um05178 | 0.2741 | ni | conserved hypothetical protein |
| 14c00096 | 0.36629 | K00943 | thymidylate kinase/adenylate kinase | um04841 | 0.2742 | ni | conserved hypothetical protein |
| 7d00194 | 0.36633 | ni | hypothetical protein | um11201.2 | 0.27477 | ni | putative protein |
| 22c00083 | 0.36638 | K06662 | checkpoint RAD17-RFC complex, RAD17/RAD24 component | um06350 | 0.27479 | K01181 | probable endo-1 |
| 11c00020 | 0.36638 | K02152 | vacuolar H+-ATPase V1 sector, subunit G | um10357 | 0.27495 | ni | probable BAT1- branched chain amino acid aminotransferase |
| 2c00065 | 0.36648 | ni | hypothetical protein | um00940 | 0.27533 | K00416 | related to QCR6 - ubiquinol--cytochrome-c reductase 17K protein |
| 22c00007 | 0.36653 | ni | hypothetical protein | um11156 | 0.27546 | ni | hypothetical protein |
| 19c00071 | 0.36656 | ni | FOG, RRM domain | um00657 | 0.27548 | K15082 | related to RAD7 - nucleotide excision repair protein |
| 4c00021 | 0.36661 | ni | hypothetical protein | um10153 | 0.27561 | K03029 | probable 26S proteasome regulatory subunit Rpn10 |
| 22c00114 | 0.36667 | K15436 | nuclear transport regulator | um04959 | 0.27563 | ni | related to BCH1 - Protein involved in transport at the trans-Golgi |
| 22c00233 | 0.36687 | ni | hypothetical protein | um02446 | 0.27608 | K03021 | probable DNA-directed RNA polymerase III |
| 10c00087 | 0.36691 | ni | hypothetical protein | um03058 | 0.27643 | K03023 | conserved hypothetical protein |
| 2c00006 | 0.36695 | ni | predicted transporter | um01126 | 0.2765 | ni | probable MRPL38 - mitochondrial ribosomal protein |
| 6d00077 | 0.36708 | ni | TNF receptor-associated factor | um06060 | 0.27663 | ni | putative protein |
| 11d00074 | 0.36714 | ni | hypothetical protein | um11498 | 0.27675 | K02930 | probable RPL4A - ribosomal protein L4-A |
| 14c00091 | 0.36717 | K07203 | DNA-dependent protein kinase | um00450 | 0.27681 | ni | related to multidrug resistance protein |
| 26d00074 | 0.36724 | ni | hypothetical protein | um02551 | 0.27686 | ni | putative protein |
| 7c00133 | 0.36725 | K00387 | sulfite oxidase, heme-binding component | um10293.2 | 0.2769 | K01103 | probable FBP26 - fructose-2 |
| 19d00136 | 0.36729 | ni | hypothetical protein | um02458 | 0.277 | ni | hypothetical protein |
| 12c00086 | 0.36737 | K04627 | hypothetical protein | um12135 | 0.27702 | ni | related to MOB2 - required for maintenance in ploidy |
| 7d00212 | 0.36747 | ni | predicted L-carnitine dehydratase | um04213 | 0.27715 | ni | putative protein |
| 26c00018 | 0.36759 | ni | hypothetical protein | um05136 | 0.27716 | ni | conserved hypothetical protein |
| 24c00033 | 0.3676 | K04499 | DNA helicase, TBP-interacting protein | um05842 | 0.27719 | ni | Myp1 protein |
| 7d00189 | 0.36798 | ni | predicted Rho GTPase-activating protein | um12112 | 0.2773 | ni | putative protein |
| 5c00076 | 0.36816 | K03113 | translation initiation factor 1 | um04808 | 0.27732 | ni | related to serine |
| 13d00080 | 0.36824 | ni | sexual differentiation process protein ISP4 | um01617 | 0.27756 | K14561 | probable IMP4 - component of the U3 small nucleolar ribonucleoprotein |
| 7c00169 | 0.36832 | K13201 | FOG, RRM domain | um04947 | 0.27782 | ni | putative protein |
| 14d00074 | 0.36859 | ni | hypothetical protein | um04451 | 0.27821 | ni | conserved hypothetical protein |
| 7c00309 | 0.3686 | K03143 | RNA polymerase II transcription initiation/nucleotide excision repa | um11331.2 | 0.27827 | ni | related to RSM27 - mitochondrial ribosomal protein |
| 20c00014 | 0.36868 | ni | hypothetical protein | um04402 | 0.2783 | K03495 | related to MTO1 protein involved in mitochondrial tRNA modification |
| 22c00281 | 0.3688 | ni | hypothetical protein | um02127 | 0.2784 | ni | conserved hypothetical protein |
| 25d00089 | 0.36904 | ni | synaptic vesicle transporter SVOP and related transporters | um05254 | 0.27868 | ni | conserved hypothetical protein |
| 14c00030 | 0.36916 | K02866 | 60s ribosomal protein L10 | um03276 | 0.27883 | ni | related to SRP40 - serine-rich protein with a role in pre-ribosome assembly or transport |
| 27d00014 | 0.36934 | K10276 | leucine rich repeat proteins | um11016 | 0.27897 | K02830 | exonuclease REC1 |
| 7c00336 | 0.36944 | ni | predicted 3'-5' exonuclease | um11168 | 0.27924 | ni | probable TIM8 - Translocase of the mitochondrial inner Membrane |
| 19c00137 | 0.3695 | K14402 | mRNA cleavage and polyadenylation factor II complex, subunit Cl | um02034 | 0.27943 | ni | conserved hypothetical protein |
| 14d00078 | 0.36975 | ni | hypothetical protein | um02601 | 0.27991 | ni | hypothetical protein |
| 12d00113 | 0.36979 | ni | hypothetical protein | um01314 | 0.27994 | ni | probable DLD2 - D-lactate dehydrogenase |
| 19c00126 | 0.36981 | ni | hypothetical protein | um04441 | 0.28015 | K10251 | related to 17-beta-hydroxysteroid dehydrogenase |
| 12d00047 | 0.36993 | ni | hypothetical protein | um11673 | 0.28027 | K05527 | conserved hypothetical protein |
| 6c00103 | 0.36994 | ni | phosphotyrosyl phosphatase activator | um01883 | 0.28034 | ni | conserved hypothetical protein |
| 7d00299 | 0.36996 | ni | ras GTPase-activating protein family - IQGAP | um04050 | 0.28049 | K01951 | probable GUA1 - GMP synthase |
| 12d00100 | 0.37003 | K02927 | ubiquitin/60s ribosomal protein L40 fusion | um05340 | 0.28062 | ni | related to Drebrin F |
| 18c00015 | 0.37024 | ni | hypothetical protein | um06445 | 0.28078 | ni | conserved hypothetical protein |
| 10d00091 | 0.37028 | ni | hypothetical protein | um04920 | 0.28101 | ni | putative protein |
| 26d00007 | 0.37028 | ni | FOG, Zn-finger | um04447 | 0.28106 | ni | conserved hypothetical protein |
| 2d00070 | 0.37048 | K10839 | nucleotide excision repair factor NEF2, RAD23 component | um01341 | 0.2812 | K06185 | probable iron inhibited ABC transporter 2 |
| 26c00090 | 0.37056 | ni | hypothetical protein | um10929 | 0.28142 | ni | conserved hypothetical protein |
| 11d00085 | 0.37058 | K08150 | predicted transporter | um03831 | 0.28143 | ni | conserved hypothetical protein |
| 18d00107 | 0.37066 | ni | hypothetical protein | um00716.2 | 0.28147 | K14789 | related to NOP6 - protein with possible role in rRNA processing |
| 26c00097 | 0.37069 | K15100 | mitochondrial tricarboxylate/dicarboxylate carrier proteins | um02572 | 0.28148 | K14016 | related to UFD1 - ubiquitin fusion degradation protein |
| 22d00035 | 0.37074 | ni | hypothetical protein | um00293 | 0.28148 | K14847 | related to protein RPF2 involved in ribosomal large subunit assembly and maintenance |
| 15d00021 | 0.37088 | ni | synaptic vesicle transporter SVOP and related transporters | um12276 | 0.28148 | ni | putative protein |
| 25c00069 | 0.3709 | K10885 | DNA-binding subunit of a DNA-dependent protein kinase | um04739 | 0.28154 | ni | conserved hypothetical protein |
| 9c00407 | 0.37115 | K11665 | SNF2 family DNA-dependent ATPase | um11421 | 0.28155 | K00140 | probable methylmalonate-semialdehyde dehydrogenase |
| 26c00096 | 0.37135 | ni | hypothetical protein | um10264 | 0.28171 | ni | hypothetical protein |
| 13c00051 | 0.37143 | K12617 | uncharacterized conserved protein | um06281 | 0.28198 | K00287 | conserved hypothetical protein |
| 6d00092 | 0.37157 | ni | integral membrane protease of the rhomboid family | um02855 | 0.28229 | K14300 | conserved hypothetical protein |
| 9d00294 | 0.3716 | K03512 | DNA polymerase IV | um10764 | 0.28229 | ni | related to TR4 orphan receptor associated protein TRA16 |
| 25c00001 | 0.37161 | ni | hypothetical protein | um03080 | 0.28243 | K06269 | probable serine |
| 16c00077 | 0.37168 | ni | spliceosomal protein snRNP-U1A/U2B | um02420 | 0.28246 | K14411 | related to HRP1 - subunit of cleavage factor I |
| 26d00093 | 0.37182 | K10865 | DNA repair exonuclease MRE11 | um00650 | 0.28263 | ni | related to Progesterone 5-beta-reductase |
| 18d00039 | 0.37201 | ni | histone deacetylase complex, catalytic component RPD3 | um04745 | 0.28263 | ni | conserved hypothetical protein |
| 8c00004 | 0.37204 | ni | hypothetical protein | um05898.2 | 0.28282 | K13989 | conserved hypothetical protein |
| 12d00027 | 0.37208 | ni | beta-tubulin folding cofactor E | um02321 | 0.28285 | ni | conserved hypothetical protein |
| 5c00075 | 0.37217 | K10863 | predicted hydrolase | um00700 | 0.28289 | ni | related to Zinc-binding oxidoreductase |
| 3c00040 | 0.3722 | ni | hypothetical protein | um04572 | 0.28292 | ni | conserved hypothetical protein |
| 22d00148 | 0.37221 | ni | hypothetical protein | um10285 | 0.28294 | ni | conserved hypothetical protein |
| 14d00049 | 0.37236 | ni | hypothetical protein | um03952 | 0.28312 | K03248 | related to TIF35 - translation initiation factor eIF3 subunit |
| 18d00029 | 0.37255 | K09184 | hypothetical protein | um03857 | 0.28322 | K01113 | conserved hypothetical protein |
| 14c00075 | 0.37263 | ni | hypothetical protein | um04570 | 0.28329 | ni | hypothetical protein |
| 5c00142 | 0.37272 | ni | hypothetical protein | um01869 | 0.28352 | K00540 | related to NADH oxidase |
| 19d00033 | 0.37277 | ni | hypothetical protein | um11157 | 0.28353 | ni | conserved hypothetical protein |
| 1d00033 | 0.37288 | ni | 3'-phosphoadenosine 5'-phosphosulfate sulfotransferase | um04994 | 0.28356 | K12670 | related to WBP1 - oligosaccharyl transferase beta subunit precursor |
| 9d00110 | 0.3729 | ni | hypothetical protein | um01125 | 0.28361 | ni | hypothetical protein |
| 12c00127 | 0.37293 | ni | hypothetical protein | um04085 | 0.2837 | ni | conserved hypothetical Ustilago-specific protein |
| 1c00002 | 0.37295 | ni | hypothetical protein | um06365.2 | 0.28385 | ni | conserved hypothetical protein |
| 5d00117 | 0.3733 | ni | hypothetical protein | um00802 | 0.28385 | K14848 | related to RRB1 - involved in the regulation of ribosome biosynthesis |
| 18c00078 | 0.3733 | K01649 | alpha-isopropylmalate synthase/homocitrate synthase | um02050 | 0.28407 | ni | conserved hypothetical protein |
| 14c00068 | 0.37348 | ni | hypothetical protein | um05126 | 0.28408 | K15334 | related to NCL1 - tRNA |
| 7d00300 | 0.37356 | ni | stress-activated MAP kinase-interacting protein | um02168.2 | 0.28415 | K08856 | related to Prk1p |
| 19c00136 | 0.37364 | ni | WD40 protein DMR-N9 | um10265 | 0.28435 | ni | conserved hypothetical protein |
| 22d00026 | 0.37373 | ni | hypothetical protein | um01008 | 0.28443 | K02324 | probable POL2 - DNA polymerase epsilon |
| 4c00019 | 0.37379 | ni | voltage-gated shaker-like K+ channel, subunit beta/KCNAB | um03973 | 0.28447 | K08793 | related to serine |
| 7c00203 | 0.37382 | ni | hypothetical protein | um03167 | 0.2845 | ni | related to endothelial zinc finger protein induced by tumor necrosis factor alpha |
| 19d00084 | 0.37385 | K14721 | hypothetical protein | um01651 | 0.28462 | K07904 | probable GTP-binding protein Rab11 |
| 16d00044 | 0.3741 | ni | mitochondrial polypeptide chain release factor | um03153 | 0.28464 | K12608 | related to ATP-binding cassette |
| 9c00001 | 0.37439 | K08176 | inorganic phosphate transporter | um02019 | 0.28468 | K01452 | probable Chitin deacetylase |
| 25d00059 | 0.37448 | K02677 | serine/threonine protein kinase | um02289 | 0.28468 | ni | conserved hypothetical protein |
| 18c00059 | 0.37458 | ni | hypothetical protein | um04610 | 0.28468 | K15174 | related to DNA-directed RNA polymerase II regulator |
| 6c00135 | 0.3747 | K06210 | nicotinamide mononucleotide adenylyl transferase | um04351 | 0.28483 | ni | probable SEC7 - component of non-clathrin vesicle coat |
| 7d00272 | 0.37476 | ni | uncharacterized conserved protein | um02549 | 0.28492 | ni | b-induced zinc finger protein |
| 20c00042 | 0.37485 | ni | hypothetical protein | um04361 | 0.28502 | K14786 | related to KRI1 - KRRI-Interacting protein 1 |
| 25c00050 | 0.37502 | ni | hypothetical protein | um05683 | 0.28505 | K07565 | probable NIP7 - required for efficient 60S ribosome subunit biogenesis |
| 7d00333 | 0.37508 | ni | hypothetical protein | um05263 | 0.28511 | ni | putative protein |
| 15d00062 | 0.37534 | ni | hypothetical protein | um05926 | 0.28523 | ni | conserved hypothetical Ustilago-specific protein |
| 15d00043 | 0.37539 | K01426 | dual specificity; serine/threonine and tyrosine kinase | um06366 | 0.28526 | ni | putative protein |
| 12d00020 | 0.37542 | ni | glyoxylate/hydroxypyruvate reductase | um10707 | 0.28564 | ni | putative protein |
| 5c00024 | 0.37542 | ni | hypothetical protein | um03635 | 0.2858 | ni | conserved hypothetical Ustilago-specific protein |
| 26d00077 | 0.37555 | ni | uncharacterized conserved protein | um03743.2 | 0.2858 | ni | related to ARP6 - Actin-related protein |
| 12c00041 | 0.37558 | ni | hypothetical protein | um02546 | 0.28588 | K12275 | related to translocation protein sec62 |
| 15d00009 | 0.37561 | ni | hypothetical protein | um05124 | 0.28589 | ni | hypothetical protein |
| 9c00394 | 0.37562 | K10755 | replication factor C, subunit RFC2 | um10373 | 0.28593 | K00794 | probable RIB4 - 6 |
| 9d00275 | 0.37599 | ni | hypothetical protein | um02190.2 | 0.28594 | ni | putative protein |
| 9d00310 | 0.37621 | ni | hypothetical protein | um02069 | 0.28603 | K14394 | probable LTP1 - protein-tyrosine-phosphatase |
| 8c00128 | 0.37622 | K00480 | hypothetical protein | um00740 | 0.28616 | K00077 | related to 2-dehydropantoate 2-reductase |
| 15d00081 | 0.37627 | ni | hypothetical protein | um01059 | 0.28618 | K15113 | probable MRS4 - Protein of the mitochondrial carrier family |
| 24d00059 | 0.37629 | K07152 | putative cytochrome C oxidase assembly protein | um00757 | 0.28632 | ni | conserved hypothetical protein |
| 6c00028 | 0.37641 | ni | hypothetical protein | um01698 | 0.28648 | ni | putative protein |
| 5c00022 | 0.37662 | ni | alcohol dehydrogenase | um11249 | 0.28649 | K00574 | related to cyclopropane-fatty-acyl-phospholipid synthase |
| 9d00245 | 0.37664 | K03937 | NADH:ubiquinone oxidoreductase, NDUFS4/18 kDa subunit | um10758.2 | 0.28651 | K14285 | related to MTR2 - mRNA export protein |
| 9c00189 | 0.37672 | ni | serine/threonine protein kinase | um05754 | 0.28665 | K03522 | probable electron transfer flavoprotein alpha chain precursor |
| 18c00009 | 0.37686 | ni | multiple inositol polyphosphate phosphatase | um00239 | 0.28677 | K11233 | related to SSK1 - two-component signal transducer |
| 9c00374 | 0.37697 | ni | NADH-cytochrome b-5 reductase | um11412 | 0.28694 | K02921 | probable RPL43B - 60S large subunit ribosomal protein |
| 8d00057 | 0.377 | ni | hypothetical protein | um11546 | 0.28705 | ni | conserved hypothetical protein |
| 2c00066 | 0.37714 | ni | hypothetical protein | um12320 | 0.28709 | ni | conserved hypothetical Ustilago-specific protein |
| 11d00030 | 0.37741 | K03979 | predicted GTP-binding protein | um00119 | 0.28715 | ni | related to protein FR |
| 7c00132 | 0.37746 | K14396 | splicing factor RNPS1 | um11195 | 0.28716 | ni | related to serine |
| 27c00063 | 0.37754 | K00297 | 5,10-methylenetetrahydrofolate reductase | um05466 | 0.28719 | ni | putative protein |
| 5c00116 | 0.37765 | ni | hypothetical protein | um01196 | 0.28725 | ni | conserved hypothetical protein |
| 18c00051 | 0.37788 | ni | hypothetical protein | um00249 | 0.28726 | ni | conserved hypothetical protein |
| 7d00181 | 0.37795 | ni | hypothetical protein | um10768 | 0.28726 | ni | conserved hypothetical protein |
| 9d00391 | 0.37798 | K02979 | 40S ribosomal protein S28 | um10754 | 0.28753 | K02135 | related to atp synthase epsilon chain |
| 10c00081 | 0.37807 | K05747 | actin regulatory protein | um00403 | 0.28762 | K00026 | probable MDH1 - malate dehydrogenase precursor |
| 9d00272 | 0.37808 | ni | hypothetical protein | um03419 | 0.28765 | ni | putative protein |
| 22c00058 | 0.37813 | ni | predicted membrane protein | um11050 | 0.28792 | K06682 | Ras-GTPase |
| 8d00005 | 0.37818 | ni | RAB proteins geranylgeranyltransferase component A | um11632 | 0.28793 | K01267 | related to bacterial glycerol-3-phosphate acyltransferases |
| 9d00170 | 0.37839 | ni | hypothetical protein | um05029 | 0.28813 | K11768 | related to macronuclear actin 1 |
| 14d00003 | 0.37884 | ni | hypothetical protein | um03916 | 0.28821 | K11253 | probable HHT1 - histone H3 |
| 9c00004 | 0.37893 | ni | hypothetical protein | um12272 | 0.28822 | K04409 | Ste20-like protein kinase |
| 7d00310 | 0.37902 | K14292 | methylase | um03070 | 0.28828 | K14399 | related to Pre-mRNA cleavage complex II protein Clp1 |
| 5c00011 | 0.37911 | ni | hypothetical protein | um05854 | 0.2884 | K11397 | conserved hypothetical protein |
| 5c00118 | 0.37921 | ni | hypothetical protein | um06253 | 0.28841 | ni | probable DUR3 - Urea permease |
| 14d00065 | 0.37928 | K01867 | mitochondrial tryptophanyl-tRNA synthetase | um00384 | 0.28852 | ni | hypothetical protein |
| 19d00044 | 0.3793 | K00568 | methyltransferases | um01729 | 0.28856 | ni | conserved hypothetical protein |
| 18c00045 | 0.37953 | ni | glutaredoxin-related protein | um03596 | 0.28859 | K13341 | related to PEX7 - peroxisomal import protein |
| 18c00044 | 0.37966 | ni | hypothetical protein | um10655 | 0.28864 | K12614 | probable ATP-dependent RNA helicase DHH1 |
| 27d00012 | 0.37972 | ni | hypothetical protein | um11442 | 0.28866 | ni | conserved hypothetical protein |
| 7c00187 | 0.37973 | ni | predicted transporter | um01752 | 0.28871 | ni | conserved hypothetical protein |
| 26c00002 | 0.37991 | ni | hypothetical protein | um10403 | 0.28874 | ni | conserved hypothetical Ustilago-specific protein |
| 27d00081 | 0.38 | ni | hypothetical protein | um00767 | 0.28907 | ni | hypothetical protein |
| 7d00116 | 0.38007 | ni | predicted proline-serine-threonine phosphatase-interacting protein | um04319 | 0.2894 | ni | related to potassium |
| 7d00290 | 0.38018 | K14537 | nucleolar GTPase | um12221 | 0.28946 | K10428 | related to Dynactin 6 |
| 12d00052 | 0.38021 | ni | hypothetical protein | um00276 | 0.28966 | ni | hypothetical protein |
| 19d00075 | 0.38027 | K05857 | phospholipase C | um02890 | 0.28969 | ni | conserved hypothetical protein |
| 8d00030 | 0.3803 | ni | predicted DNA repair exonuclease SIA1 | um04968 | 0.28989 | ni | hypothetical protein |
| 27d00005 | 0.3803 | ni | hypothetical protein | um11889 | 0.28993 | ni | conserved hypothetical protein |
| 18c00088 | 0.38032 | K09567 | U-snRNP-associated cyclophilin type peptidyl-prolyl cis-trans isor | um04021 | 0.29 | K07562 | probable NMD3 - nonsense-mediated mRNA decay protein |
| 13c00108 | 0.3804 | ni | uncharacterized conserved protein | um03901 | 0.29 | ni | related to Serine |
| 14d00123 | 0.38051 | ni | hypothetical protein | um01796 | 0.29009 | ni | effector family protein Eff1-1 |
| 22c00202 | 0.38056 | ni | hypothetical protein | um01991 | 0.29014 | ni | putative protein |
| 7d00228 | 0.38059 | K06127 | ubiquinone biosynthesis methyltransferase COQ5 | um02856 | 0.29018 | ni | conserved hypothetical protein |
| 9d00358 | 0.38114 | K14015 | nuclear pore complex, rNpl4 component | um00986 | 0.29023 | K07827 | small G-protein Ras1 |
| 19d00147 | 0.38116 | ni | hypothetical protein | um10309 | 0.29024 | ni | hypothetical protein |
| 9c00021 | 0.38126 | K07575 | predicted RNA-binding protein with PUA domain | um00194 | 0.29029 | ni | hypothetical protein |

| | | | | | | | |
|---|---|---|---|---|---|---|---|
| 25c00040 | 0.38137 | K11390 | hypothetical protein | um02713 | 0.29041 | ni | pheromone response factor Prf1 |
| 19d00155 | 0.38145 | K01341 | subtilisin-like proprotein convertase | um05045 | 0.29057 | K16196 | related to GCN2 - ser |
| 9d00273 | 0.38148 | K01191 | alpha-mannosidase | um01987 | 0.29072 | ni | hypothetical protein |
| 7c00207 | 0.38151 | ni | hypothetical protein | um05312 | 0.29095 | ni | conserved hypothetical Ustilago-specific protein |
| 12c00031 | 0.38167 | K06630 | multifunctional chaperone | um00734 | 0.29101 | ni | conserved hypothetical protein |
| 26c00060 | 0.38167 | ni | dolichol kinase | um00936 | 0.29105 | K04348 | calcineurin catalytic subunit |
| 11c00076 | 0.38173 | ni | hypothetical protein | um04342 | 0.29111 | ni | putative protein |
| 11c00025 | 0.38176 | ni | hypothetical protein | um02177.2 | 0.29115 | ni | related to DRAP deaminase RIB2 |
| 2c00051 | 0.38177 | K03122 | RNA polymerase II transcription initiation factor TFIIA, large chair | um10156 | 0.29133 | K09580 | probable proteine disulfate isomerase |
| 26c00078 | 0.38177 | K07179 | serine/threonine protein kinase | um03584 | 0.29146 | K03005 | related to RPA49 - 49 kD subunit of DNA-directed RNA polymerase I |
| 18d00109 | 0.38218 | ni | hypothetical protein | um04689 | 0.2916 | ni | conserved hypothetical Ustilago-specific protein |
| 5c00148 | 0.38221 | K11372 | cell cycle-regulated histone H1-binding protein | um05625 | 0.29177 | K03965 | conserved hypothetical protein |
| 25d00088 | 0.38226 | ni | predicted transporter | um12225 | 0.29177 | ni | putative protein |
| 12c00009 | 0.38241 | ni | rasgap SH3 binding protein rasputin | um11208 | 0.29183 | K07973 | putative protein |
| 8d00046 | 0.38249 | ni | predicted DEAD-box-containing helicase | um11234.2 | 0.29195 | ni | conserved hypothetical protein |
| 22d00158 | 0.38252 | K01417 | jacalin-like lectin domain-containing protein | um03388 | 0.29202 | ni | hypothetical protein |
| 7c00071 | 0.38254 | ni | hypothetical protein | um05052 | 0.29213 | ni | hypothetical protein |
| 7c00253 | 0.38269 | K00602 | AICAR transformylase | um03791 | 0.29223 | K03283 | heat shock 70 kd protein 2 |
| 22d00227 | 0.38281 | ni | hypothetical protein | um05700 | 0.29263 | ni | conserved hypothetical protein |
| 20d00004 | 0.38282 | ni | hypothetical protein | um10138 | 0.29283 | ni | conserved hypothetical protein |
| 9c00349 | 0.38285 | ni | arylacetamide deacetylase | um05342 | 0.29287 | ni | hypothetical protein |
| 1c00001 | 0.38306 | ni | predicted transporter | um05392 | 0.29293 | ni | conserved hypothetical protein |
| 7d00131 | 0.3833 | ni | serine/threonine protein kinase | um10503 | 0.29298 | K16944 | probable cell division control protein CDC3 |
| 11d00043 | 0.38332 | ni | hypothetical protein | um05301 | 0.29298 | ni | conserved hypothetical Ustilago-specific protein |
| 5d00032 | 0.3834 | ni | hypothetical protein | um02039 | 0.29302 | ni | conserved hypothetical protein |
| 5c00157 | 0.38347 | K12765 | MAPK related serine/threonine protein kinase | um00675 | 0.29305 | ni | conserved hypothetical protein |
| 14d00092 | 0.38356 | K00559 | SAM-dependent methyltransferases | um10258 | 0.29326 | ni | hypothetical protein |
| 14c00117 | 0.38365 | ni | class 2 transcription repressor NC2, beta subunit | um05954 | 0.29337 | K15283 | related to SLY41 - Putative transporter of the triose phosphate translocator family |
| 22c00195 | 0.38371 | ni | hypothetical protein | um02224 | 0.29337 | ni | conserved hypothetical protein |
| 7c00206 | 0.38377 | K03038 | 26S proteasome regulatory complex, subunit RPN8/PSMD7 | um01920 | 0.2934 | ni | related to Na |
| 27d00025 | 0.38393 | ni | uncharacterized conserved protein | um11668 | 0.2934 | ni | related to 5 |
| 20d00048 | 0.38394 | K00641 | hypothetical protein | um11447.2 | 0.2935 | K17257 | conserved hypothetical protein |
| 7d00262 | 0.38403 | K13278 | asparaginase | um11254 | 0.29357 | ni | conserved hypothetical protein |
| 2d00013 | 0.38412 | K01754 | threonine/serine dehydratases | um04790 | 0.29365 | ni | hypothetical protein |
| 7c00231 | 0.38415 | ni | glyoxylate/hydroxypyruvate reductase | um01528 | 0.29368 | ni | conserved hypothetical protein |
| 20c00047 | 0.38419 | ni | protein phosphatase 2A-associated protein | um12241 | 0.29374 | ni | related to COX19 - Cytochrome c oxidase assembly protein |
| 5c00089 | 0.38423 | ni | hypothetical protein | um00904 | 0.29377 | K09486 | related to glucose regulated stress protein |
| 25c00086 | 0.3843 | ni | UDP-glucose 4-epimerase | um10914 | 0.29381 | K11484 | related to HOS3 - Trichostatin A-insensitive homodimeric histone deacetylase |
| 16d00030 | 0.38431 | K15280 | predicted integral membrane protein | um03090 | 0.29393 | ni | hypothetical protein |
| 22d00265 | 0.38446 | ni | dehydrogenases with different specificities | um10785 | 0.29402 | K03025 | related to RPC34 - DNA-directed RNA polymerase III |
| 5d00009 | 0.38482 | ni | hypothetical protein | um01174 | 0.29402 | K12811 | related to RNA helicase |
| 7c00107 | 0.38483 | ni | hypothetical protein | um02212 | 0.29445 | K01194 | related to trehalase precursor |
| 15c00067 | 0.38492 | ni | hypothetical protein | um11915 | 0.29459 | ni | putative protein |
| 16c00027 | 0.38492 | ni | hypothetical protein | um05200 | 0.29502 | K14808 | related to DBP10 - putative ATP-dependent RNA helicase involved in ribosome biogenesis |
| 19c00086 | 0.38497 | K15147 | hypothetical protein | um02677 | 0.29505 | K11422 | related to regulatory protein SET1 |
| 20c00027 | 0.38505 | K14311 | uncharacterized conserved protein | um10848 | 0.29522 | K11560 | conserved hypothetical protein |
| 12c00143 | 0.38519 | ni | hypothetical protein | um00617 | 0.29547 | K01409 | related to probable O-sialoglycoprotein endopeptidase |
| 26d00091 | 0.38524 | ni | hypothetical protein | um03883 | 0.29552 | ni | related to WD repeat and FYVE domain-containing protein 3 |
| 25d00003 | 0.38534 | ni | uncharacterized conserved protein | um02979 | 0.29554 | ni | conserved hypothetical protein |
| 10c00094 | 0.38536 | ni | hypothetical protein | um02437 | 0.29567 | K03950 | probable NADH2 dehydrogenase |
| 6c00057 | 0.38553 | ni | hypothetical protein | um11114 | 0.29574 | ni | conserved hypothetical protein |
| 19c00076 | 0.38557 | K00940 | nucleoside diphosphate kinase | um11103 | 0.29576 | K02925 | probable RPL3 - 60s ribosomal protein l3 |
| 9c00304 | 0.3856 | ni | guanine nucleotide exchange factor | um12228 | 0.29584 | ni | putative protein |
| 2c00042 | 0.38564 | K06062 | histone acetyltransferase SAGA/ADA, catalytic subunit PCAF/GC | um02466 | 0.2959 | ni | hypothetical protein |
| 8d00033 | 0.38566 | ni | hypothetical protein | um06443 | 0.29612 | K03264 | probable TIF6 - translation initiation factor 6 |
| 18d00098 | 0.38569 | ni | hypothetical protein | um02849 | 0.29631 | K00942 | probable GUK1 - guanylate kinase |
| 7c00256 | 0.38574 | K03768 | peptidyl-prolyl cis-trans isomerase | um01990 | 0.29648 | ni | conserved hypothetical protein |
| 8c00065 | 0.38576 | ni | serine palmitoyltransferase | um03042 | 0.29652 | ni | hypothetical protein |
| 2d00009 | 0.38577 | K15102 | mitochondrial phosphate carrier protein | um01064 | 0.29653 | K02959 | related to 30S ribosomal protein S16 |
| 7d00162 | 0.38582 | ni | hypothetical protein | um06296 | 0.29655 | K03144 | probable TFB2 - TFIIH subunit |
| 27d00021 | 0.38589 | ni | membrane coat complex Retromer, subunit VPS5/SNX1 | um12004 | 0.29674 | ni | conserved hypothetical protein |
| 4d00037 | 0.38591 | ni | hypothetical protein | um05380 | 0.29684 | ni | conserved hypothetical protein |
| 13c00077 | 0.38623 | ni | hypothetical protein | um11281 | 0.29687 | K12820 | probable PRP43 - involved in spliceosome disassembly |
| 9d00404 | 0.3863 | ni | hypothetical protein | um04035 | 0.29695 | ni | conserved hypothetical Ustilago-specific protein |
| 5c00109 | 0.38649 | ni | hypothetical protein | um03653 | 0.29725 | ni | conserved hypothetical protein |
| 13d00011 | 0.38656 | K14800 | hypothetical protein | um11474 | 0.2973 | K12183 | related to Tumor susceptibility gene 101 protein |
| 24c00046 | 0.38667 | ni | hypothetical protein | um05907 | 0.29735 | K17408 | conserved hypothetical protein |
| 9c00225 | 0.38671 | K00026 | NAD-dependent malate dehydrogenase | um11036 | 0.29741 | K12862 | related to PRP46 - member of the spliceosome |
| 11c00068 | 0.38677 | ni | mitochondrial matrix protein frataxin | um05428 | 0.29783 | K02896 | related to RLP24 - Ribosomal Like Protein 24 |
| 10c00009 | 0.38713 | ni | hypothetical protein | um10047 | 0.29841 | K14768 | conserved hypothetical protein |
| 14d00109 | 0.38714 | K03239 | translation initiation factor 2B, alpha subunit | um04130 | 0.2986 | ni | Dik6 |
| 19c00082 | 0.38721 | ni | peroxisomal NUDIX hydrolase | um10066 | 0.29861 | ni | conserved hypothetical protein |
| 22d00229 | 0.38737 | ni | hypothetical protein | um00305 | 0.29862 | K12825 | related to Splicing factor 3 subunit 1 |
| 11c00007 | 0.38742 | K14803 | serine/threonine protein phosphatase | um00094 | 0.29863 | K01423 | related to NonF protein |
| 10c00002 | 0.38791 | K12182 | membrane trafficking and cell signaling protein HRS | um03092 | 0.29864 | ni | putative protein |
| 18c00016 | 0.38806 | ni | uncharacterized conserved protein | um02733.2 | 0.29897 | ni | related to SMI1 - beta-1 |
| 15d00066 | 0.38809 | ni | predicted haloacid-halidohydrolase and related hydrolases | um03098 | 0.29933 | ni | related to CNS1 - cyclophilin seven suppressor |
| 8c00008 | 0.38833 | ni | hypothetical protein | um01565 | 0.29943 | ni | related to GPI transamidase component PIG-S |
| 9c00418 | 0.38833 | K10581 | ubiquitin-conjugating enzyme | um05848 | 0.29949 | ni | related to galactoside O-acetyltransferase |
| 5d00051 | 0.38842 | ni | leptin receptor gene-related protein | um05015 | 0.29958 | ni | hypothetical protein |
| 8d00096 | 0.38899 | K01772 | protoheme ferro-lyase | um03906 | 0.29963 | ni | hypothetical protein |
| 15d00058 | 0.38918 | ni | histone H3 (Lys9) methyltransferase SUV39H1/Clr4 | um12012 | 0.29968 | ni | conserved hypothetical protein |
| 10c00059 | 0.38918 | ni | hypothetical protein | um01669 | 0.29974 | K12196 | probable VPS4 - vacuolar sorting protein |
| 7d00224 | 0.38928 | ni | hypothetical Protein | um10526 | 0.29975 | ni | probable SSB2 - heat shock protein of HSP70 family |
| 5d00025 | 0.38979 | ni | peroxisomal phytanoyl-CoA hydroxylase | um01658 | 0.3 | ni | conserved hypothetical protein |
| 10d00077 | 0.38981 | K01262 | xaa-pro aminopeptidase | um05703 | 0.30004 | K00273 | related to D-amino-acid oxidase |
| 15c00046 | 0.38989 | ni | rho GTPase-activating protein | um02571 | 0.30009 | K09496 | probable CCT4 - component of chaperonin-containing T-complex |
| 6d00123 | 0.39008 | K05841 | UDP-glucuronosyl and UDP-glucosyl transferase | um12243 | 0.30009 | K11649 | related to swi |
| 18d00048 | 0.39022 | ni | hypothetical protein | um00494 | 0.30011 | ni | conserved hypothetical protein |
| 1c00042 | 0.39028 | ni | mitochondrial import inner membrane translocase, subunit TIM9 | um10405.2 | 0.30014 | K15076 | hypothetical protein |
| 14d00056 | 0.39041 | K12397 | vesicle coat complex AP-3, beta subunit | um04793 | 0.30047 | ni | conserved hypothetical protein |
| 22c00145 | 0.3905 | ni | hypothetical protein | um02772 | 0.30056 | K02933 | related to MRPL6 - mitochondrial ribosomal protein |
| 25d00070 | 0.39056 | K14005 | vesicle coat complex COPII, subunit SEC31 | um01149 | 0.30067 | ni | glyoxaloxidase 3 |
| 22c00221 | 0.3906 | K09648 | mitochondrial inner membrane protease, subunit IMP2 | um01522 | 0.30091 | ni | hypothetical protein |
| 25c00023 | 0.39066 | K14824 | WD40 repeat nucleolar protein Bop1 | um04074 | 0.30095 | ni | conserved hypothetical protein |
| 3d00039 | 0.39071 | K12819 | RNA splicing factor - Slu7p | um12118 | 0.301 | ni | putative protein |
| 2d00072 | 0.39071 | ni | hypothetical protein | um01226 | 0.30106 | ni | hypothetical protein |
| 9c00019 | 0.39071 | ni | predicted membrane proteins | um00598 | 0.3013 | ni | conserved hypothetical protein |
| 16c00022 | 0.39079 | ni | hypothetical protein | um10358 | 0.30132 | ni | hypothetical protein |
| 20c00039 | 0.39098 | K15264 | proliferation-associated nucleolar protein | um11396 | 0.30136 | K08286 | related to ser |
| 7d00230 | 0.39099 | ni | predicted DHHC-type Zn-finger protein | um10139 | 0.30158 | K01918 | probable pantoate--beta-alanine ligase |
| 15c00088 | 0.39099 | ni | hypothetical protein | um04998 | 0.30165 | ni | conserved hypothetical protein |
| 9d00112 | 0.39103 | K09885 | aquaporin | um01386 | 0.30174 | ni | hypothetical protein |
| 18c00100 | 0.39107 | ni | hypothetical protein | um11387 | 0.30182 | ni | hypothetical protein |
| 20c00001 | 0.39113 | ni | hypothetical protein | um10372 | 0.3019 | ni | related to MRPL3 - Mitochondrial ribosomal protein |
| 22d00062 | 0.39115 | ni | WD40 repeat protein | um00104 | 0.30218 | ni | hypothetical protein |
| 19d00106 | 0.39121 | ni | hypothetical protein | um00275 | 0.30225 | K02331 | related to DNA polymerase V |
| 5d00123 | 0.39146 | K11303 | histone acetyltransferase type b catalytic subunit | um05207 | 0.3024 | K03506 | conserved hypothetical protein |
| 20d00005 | 0.3916 | ni | hypothetical protein | um04767 | 0.30252 | K09549 | related to GIM4 - Gim complex component |
| 10c00022 | 0.39162 | ni | hypothetical protein | um05870 | 0.30255 | ni | related to ERV1 - mitochondrial biogenesis and regulation of cell cycle |
| 12c00066 | 0.39174 | K14571 | nuclear AAA ATPase | um01870 | 0.30255 | K00799 | related to glutathione-S-transferase |
| 9c00371 | 0.39179 | K15447 | uncharacterized conserved protein | um01222 | 0.30278 | ni | related to SAL1 - member of the Ca2 -binding subfamily of the mitochondrial carrier family |
| 22c00168 | 0.39181 | ni | predicted methyltransferase | um05291 | 0.30279 | ni | conserved hypothetical protein |
| 18d00058 | 0.39186 | ni | hypothetical protein | um01027 | 0.30289 | K11761 | conserved hypothetical protein |
| 10d00057 | 0.39197 | ni | hypothetical protein | um06416 | 0.30323 | ni | conserved hypothetical protein |
| 18d00032 | 0.39197 | ni | hypothetical protein | um01746 | 0.30329 | ni | putative protein |
| 3c00031 | 0.39203 | ni | hypothetical protein | um05828 | 0.30329 | K07375 | probable tubulin beta chain |
| 7d00342 | 0.39212 | K09522 | zuotin and related molecular chaperones | um03498 | 0.30331 | ni | related to CAF4 - CCR4 associated factor |
| 12d00024 | 0.39216 | K00670 | subunit of the major N alpha-acetyltransferase | um03467 | 0.30348 | K02992 | related to RSM7 - mitochondrial ribosomal protein |
| 16c00071 | 0.39229 | K07443 | 6-O-methylguanine-DNA methyltransferase MGMT/MGT1 | um02938 | 0.3036 | ni | conserved hypothetical protein |
| 1c00012 | 0.39232 | ni | hypothetical protein | um06386 | 0.3036 | K13095 | probable MSL5 - branch point bridging protein |
| 12d00039 | 0.39248 | K05756 | actin-related protein Arp2/3 complex, subunit ARPC3 | um04062 | 0.30369 | ni | conserved hypothetical protein |
| 14c00013 | 0.39251 | ni | hypothetical protein | um11222 | 0.30382 | ni | related to MBP1 - transcription factor |
| 11c00045 | 0.39256 | ni | hypothetical protein | um04223 | 0.30391 | ni | probable glutaredoxin |
| 2c00082 | 0.39257 | ni | rab GTPase interacting factor | um00101 | 0.30394 | K03509 | related to DNA polymerase eta |
| 22d00058 | 0.39265 | ni | hypothetical protein | um03028 | 0.30397 | ni | conserved hypothetical protein |
| 9c00303 | 0.39272 | ni | nucleoside phosphatase | um05991 | 0.30397 | K14376 | probable PAP1 - poly |
| 9c00365 | 0.39274 | K12872 | predicted RNA-binding protein | um02555 | 0.30397 | ni | conserved hypothetical protein |
| 7d00259 | 0.39287 | ni | uncharacterized conserved protein | um04298 | 0.30399 | K14857 | probable SPB1 - required for ribosome synthesis |
| 24c00069 | 0.39293 | ni | alpha-mannosidase | um02936 | 0.30399 | ni | putative protein |
| 9c00337 | 0.39293 | K11567 | hypothetical protein | um03933 | 0.30411 | ni | related to WWM1 - WW domain containing protein interacting with Metacaspase |
| 22d00112 | 0.39295 | K01082 | salt-sensitive 3'-phosphoadenosine-5'-phosphatase HAL2/SAL1 | um00950 | 0.30418 | K16570 | related to Spindle pole body component alp6 |
| 13c00041 | 0.39298 | K03248 | translation initiation factor 3, subunit g | um01287 | 0.30433 | ni | conserved hypothetical protein |
| 9d00217 | 0.39312 | ni | hypothetical protein | um10432.2 | 0.30489 | ni | related to ATP-binding cassette |
| 14c00132 | 0.39328 | ni | hypothetical protein | um11989 | 0.30506 | K14776 | probable HCA4 - can suppress the U14 snoRNA rRNA processing function |
| 3c00071 | 0.39332 | K02984 | 40S ribosomal protein S3A | um11518 | 0.30538 | ni | related to SKT5 - activator of chitin synthase III |
| 22d00009 | 0.39351 | ni | uncharacterized conserved protein | um06439 | 0.30567 | ni | hypothetical protein |
| 6d00023 | 0.39351 | ni | tandem pore domain K+ channel | um10653 | 0.30578 | ni | conserved hypothetical protein |
| 27c00074 | 0.39358 | K02932 | 60S ribosomal protein L5 | um04649 | 0.3059 | K03842 | related to ALG1 - beta-mannosyltransferase |
| 12d00137 | 0.39371 | ni | uncharacterized conserved protein | um06477 | 0.30591 | ni | conserved hypothetical protein |
| 24c00028 | 0.39374 | K14568 | protein required for 18S rRNA maturation and 40S ribosome biog | um00829 | 0.30601 | ni | conserved hypothetical protein |
| 8c00053 | 0.39375 | K02894 | 60S ribosomal protein L14/L17/L23 | um02181 | 0.30602 | ni | conserved hypothetical protein |
| 19c00015 | 0.39385 | ni | uncharacterized conserved protein | um01133 | 0.30614 | K03002 | probable RPA135 - DNA-directed RNA polymerase I |
| 20d00056 | 0.39393 | K01892 | histidyl-trna synthetase | um05502 | 0.30633 | K17427 | related to MRPL17 - mitochondrial ribosomal protein |
| 2d00059 | 0.39404 | ni | hypothetical protein | um00693 | 0.30634 | ni | conserved hypothetical protein |
| 2c00071 | 0.39409 | K08744 | CDP-alcohol phosphatidyltransferase | um04906 | 0.30655 | K10295 | related to F-box protein pof7 |
| 24d00037 | 0.39418 | K05692 | actin and related proteins | um06284 | 0.30656 | K09313 | related to Cutl1 or CASP protein |
| 5d00060 | 0.39434 | ni | hypothetical protein | um11153 | 0.30656 | ni | related to COX23 - protein with function in mitochondrial copper homeostasis |
| 6d00126 | 0.39442 | ni | hypothetical protein | um03239 | 0.30688 | K01652 | probable acetolactate synthase |
| 7c00161 | 0.39445 | K09518 | molecular chaperone | um00306 | 0.30702 | ni | hypothetical protein |
| 12c00081 | 0.39447 | K02870 | 40S ribosomal protein S2 | um01177 | 0.30728 | K01889 | related to MSF1 - phenylalanine--tRNA ligase alpha chain |
| 5d00037 | 0.39468 | ni | predicted glutamine synthetase | um06051 | 0.30728 | K03938 | conserved hypothetical protein |
| 11c00018 | 0.39478 | K00861 | riboflavin kinase | um10367 | 0.30745 | K00698 | chitin synthase 6 |
| 7d00226 | 0.39489 | K14767 | disrupter of silencing SAS10 | um01092 | 0.30746 | ni | hypothetical protein |
| 27c00086 | 0.39499 | K00864 | ribulose kinase and related carbohydrate kinases | um00089 | 0.30748 | ni | conserved hypothetical protein |
| 25c00008 | 0.39506 | ni | hypothetical protein | um11633 | 0.30787 | K01267 | probable aspartyl aminopeptidase |

| | | | | | | | |
|---|---|---|---|---|---|---|---|
| 5d00023 | 0.39524 | ni | hypothetical protein | um02739 | 0.30801 | ni | probable nik-1 protein |
| 26c00056 | 0.39542 | ni | glutamyl-trna amidotransferase subunit B | um10169 | 0.30803 | ni | conserved hypothetical Ustilago-specific protein |
| 11c00034 | 0.39558 | K10413 | dyneins, heavy chain | um11806.2 | 0.30809 | ni | conserved hypothetical protein |
| 9d00341 | 0.39574 | K11801 | WD40 repeat-containing protein | um05217 | 0.30815 | ni | conserved hypothetical protein |
| 15c00089 | 0.39594 | ni | hypothetical protein | um04430 | 0.30824 | K01070 | probable esterase D |
| 16c00056 | 0.39595 | K15109 | mitochondrial carrier protein | um00887 | 0.30841 | ni | conserved hypothetical protein |
| 8d00092 | 0.39606 | ni | hypothetical protein | um10054.2 | 0.30853 | ni | hypothetical protein |
| 5c00119 | 0.39644 | K11446 | DNA-binding protein jumonji/RBP2/SMCY | um01089 | 0.30856 | ni | related to nucleoporin |
| 4d00036 | 0.3966 | K07494 | hypothetical protein | um11982 | 0.30905 | ni | conserved hypothetical protein |
| 6c00124 | 0.39674 | K01698 | delta-aminolevulinic acid dehydratase | um01892 | 0.30911 | K01637 | probable isocitrate lyase |
| 14d00031 | 0.39699 | ni | uncharacterized conserved protein | um10591 | 0.30912 | K06867 | conserved hypothetical protein |
| 7c00310 | 0.397 | K10756 | replication factor C, subunit RFC3 | um15099 | 0.30913 | ni | conserved hypothetical protein |
| 9c00254 | 0.39725 | ni | hypothetical protein | um01415 | 0.30914 | ni | conserved hypothetical protein |
| 19d00061 | 0.39725 | ni | peroxisomal membrane protein MPV17 and related proteins | um11950 | 0.30917 | ni | conserved hypothetical protein |
| 10d00019 | 0.39733 | K11108 | RNA 3'-terminal phosphate cyclase | um04258 | 0.30938 | ni | ni |
| 7c00120 | 0.39744 | ni | beta-galactosidase | um03992 | 0.30944 | ni | Don3 interacting protein |
| 12d00128 | 0.39751 | ni | hypothetical protein | um03542 | 0.30947 | ni | conserved hypothetical protein |
| 14c00072 | 0.39778 | ni | hypothetical protein | um06221 | 0.30957 | ni | hypothetical protein |
| 1c00046 | 0.39791 | K02975 | 40S ribosomal protein S25 | um04923 | 0.30969 | K13754 | conserved hypothetical protein |
| 22d00221 | 0.39805 | ni | hypothetical protein | um11976 | 0.30995 | ni | putative protein |
| 10d00028 | 0.39812 | K14773 | uncharacterized proteins of PilT N-term | um06448 | 0.31005 | K02975 | probable 40S ribosomal protein S25 |
| 10c00102 | 0.39816 | ni | hypothetical protein | um04594 | 0.31013 | ni | related to TIM44 - mitochondrial inner membrane import receptor subunit |
| 2d00036 | 0.39858 | K12848 | U1-like Zn-finger protein | um01573 | 0.31015 | ni | conserved hypothetical protein |
| 16c00038 | 0.3986 | K13110 | microfibrillar-associated protein MFAP1 | um11294 | 0.31022 | ni | ni |
| 25c00055 | 0.39862 | K11484 | histone deacetylase complex, catalytic component HDA1 | um11461 | 0.31024 | K01698 | probable porphobilinogen synthase |
| 22c00135 | 0.3987 | ni | hypothetical protein | um02711 | 0.3103 | ni | related to ADAM protease ADM-B |
| 19d00101 | 0.39891 | ni | hypothetical protein | um03755 | 0.31044 | ni | hypothetical protein |
| 8c00056 | 0.39915 | K02906 | mitochondrial/chloroplast ribosomal protein L3 | um04731 | 0.31074 | ni | hypothetical protein |
| 19c00010 | 0.39988 | K13113 | ubiquitin-like protein | um00691 | 0.31076 | ni | related to HRK1 - Protein kinase with a role in ion homeostasis |
| 18c00020 | 0.40004 | ni | mRNA cleavage factor I subunit | um05258 | 0.31108 | ni | putative protein |
| 3d00093 | 0.40076 | ni | arylacetamide deacetylase | um03009 | 0.3111 | ni | related to pyridoxamine-phosphate oxidase |
| 7c00030 | 0.40081 | ni | mitochondrial sulfhydryl oxidase | um04621 | 0.31114 | ni | putative protein |
| 6c00104 | 0.40084 | ni | uncharacterized membrane protein | um10872 | 0.31126 | K01515 | related to YSA1 - sugar-nucleotide hydrolase |
| 8d00072 | 0.40092 | ni | hypothetical protein | um05931 | 0.31133 | ni | conserved hypothetical Ustilago-specific protein |
| 7c00153 | 0.401 | ni | hypothetical protein | um11955 | 0.31142 | K01867 | probable MSW1 - tryptophanyl-tRNA synthetase |
| 7d00057 | 0.40105 | ni | cyclin | um05621 | 0.31142 | ni | conserved hypothetical Ustilago-specific protein |
| 26c00073 | 0.40117 | ni | hypothetical protein | um01829 | 0.31153 | ni | related to alpha-L-arabinofuranosidase I precursor |
| 7d00128 | 0.40127 | ni | hypothetical protein | um01855 | 0.31154 | ni | conserved hypothetical protein |
| 9d00116 | 0.40127 | ni | hypothetical protein | um12165 | 0.31162 | ni | conserved hypothetical protein |
| 9c00278 | 0.40138 | ni | hypothetical protein | um02376 | 0.31167 | K13501 | probable anthranilate synthase component II |
| 22d00161 | 0.40142 | ni | hypothetical protein | um10144 | 0.31172 | K00387 | related to Sulfite oxidase |
| 9d00065 | 0.40164 | ni | serine/threonine protein phosphatase | um15098 | 0.31185 | ni | putative protein |
| 26d00097 | 0.4018 | K06889 | predicted alpha/beta hydrolase BEM46 | um02056 | 0.31193 | ni | conserved hypothetical protein |
| 6d00024 | 0.40181 | ni | hypothetical protein | um03593 | 0.31194 | K00227 | probable sterol delta 5 |
| 22c00284 | 0.40188 | ni | hypothetical protein | um10896 | 0.31194 | ni | probable oligopeptide transporter |
| 27c00026 | 0.40197 | K10577 | ubiquitin-protein ligase | um00590 | 0.3121 | ni | putative protein |
| 5c00013 | 0.40221 | ni | hypothetical protein | um04489 | 0.31219 | ni | probable MAS6 - mitochondrial inner membrane import translocase subunit |
| 3c00006 | 0.40227 | ni | sensory transduction histidine kinase | um10321 | 0.3124 | K14790 | conserved hypothetical protein |
| 27d00023 | 0.40228 | ni | uncharacterized conserved protein | um11074 | 0.31243 | ni | related to SMC5 - Structural maintenance of chromosomes |
| 6c00131 | 0.40233 | ni | hypothetical protein | um10468.2 | 0.31253 | ni | conserved hypothetical protein |
| 22d00114 | 0.40235 | ni | hypothetical protein | um03038 | 0.3127 | ni | related to candidate tumor suppressor dph2l1 |
| 13c00023 | 0.40244 | K11253 | histones H3 and H4 | um04486 | 0.31283 | ni | conserved hypothetical Ustilago-specific protein |
| 22d00025 | 0.40253 | K06883 | GTPase XAB1 | um10362 | 0.31286 | ni | conserved hypothetical protein |
| 24d00039 | 0.40255 | ni | 60s ribosomal protein L2/L8 | um12150 | 0.31288 | ni | conserved hypothetical protein |
| 4d00043 | 0.40265 | K01738 | cysteine synthase | um02146 | 0.31298 | ni | related to GABA permease |
| 7c00183 | 0.4028 | ni | GTPase-activating protein | um02423 | 0.31307 | ni | hypothetical protein |
| 15c00066 | 0.403 | ni | hypothetical protein | um05761 | 0.31325 | K10436 | EB1-like protein |
| 14c00128 | 0.40306 | ni | polyadenylation factor I complex, subunit FIP1 | um01269 | 0.3133 | ni | conserved hypothetical protein |
| 14d00027 | 0.40306 | K17439 | phosphatidylethanolamine binding protein | um10720 | 0.31341 | ni | related to ser |
| 7d00065 | 0.40308 | ni | permease of the drug/metabolite transporter | um04580 | 0.31353 | ni | conserved hypothetical protein |
| 27c00068 | 0.40319 | K14304 | nuclear pore complex component | um06086 | 0.31358 | ni | related to branched-chain alpha-ketoacid dehydrogenase kinase |
| 12c00055 | 0.40334 | ni | protein involved in vacuole import and degradation | um11721 | 0.31384 | ni | conserved hypothetical protein |
| 2d00012 | 0.40357 | K14555 | WD40-repeat-containing subunit of the 18S rRNA processing cou | um15016 | 0.31385 | K14843 | conserved hypothetical protein |
| 9c00027 | 0.40374 | ni | hypothetical protein | um00266 | 0.3139 | ni | related to Rpa34 - 34 kD subunit of DNA-directed RNA polymerase I |
| 26c00082 | 0.40382 | K10398 | kinesin-like protein | um11644 | 0.31397 | ni | probable GLR1 - glutathione reductase |
| 14d00117 | 0.40424 | K02208 | cyclin C-dependent kinase CDK8 | um06200 | 0.31414 | K13336 | related to Peroxisomal assembly protein PEX3 |
| 22c00207 | 0.40424 | ni | glycolipid 2-alpha-mannosyltransferase | um02234 | 0.31417 | ni | conserved hypothetical protein |
| 3c00066 | 0.40427 | ni | predicted methyltransferase | um00624 | 0.31425 | K00999 | related to CDP diacylglycerol-inositol 3-phosphatidyltransferase |
| 7c00005 | 0.40429 | K01843 | hypothetical protein | um02777 | 0.31436 | K12880 | related to THO complex subunit 3 |
| 25c00026 | 0.40431 | ni | hypothetical protein | um10354 | 0.31441 | ni | conserved hypothetical protein |
| 20c00037 | 0.40432 | ni | predicted transporter | um02519 | 0.31448 | ni | related to ALG6 - glucosyltransferase |
| 12d00140 | 0.40449 | ni | hypothetical protein | um03226 | 0.31451 | K03349 | related to component of the anaphase promoting complex |
| 7d00150 | 0.40454 | ni | hypothetical protein | um04464 | 0.31463 | ni | conserved hypothetical protein |
| 9d00089 | 0.40461 | ni | hypothetical protein | um12176 | 0.31468 | K13171 | conserved hypothetical protein |
| 26c00016 | 0.4051 | K09142 | uncharacterized conserved protein | um12138.2 | 0.31478 | ni | hypothetical protein |
| 11c00066 | 0.40513 | ni | hypothetical protein | um04551 | 0.3148 | K15564 | conserved hypothetical protein |
| 5c00145 | 0.40523 | ni | hypothetical protein | um11618 | 0.31481 | ni | related to CYC2 - cytochrome-c mitochondrial import factor |
| 7c00148 | 0.40535 | ni | predicted E3 ubiquitin ligase | um02662 | 0.31489 | K14558 | probable periodic tryptophan protein PWP2 |
| 14c00082 | 0.40538 | ni | hypothetical protein | um10213 | 0.3153 | K02132 | probable H -transporting ATP synthase alpha chain |
| 6c00041 | 0.40542 | ni | gamma-glutamyltransferase | um00389 | 0.31535 | K02309 | related to Hsk1-interacting molecule 1 |
| 11d00016 | 0.40543 | ni | hypothetical protein | um02808 | 0.31539 | ni | related to nitrogen assimilation transcription factor nit-4 |
| 5c00090 | 0.40544 | ni | non-ribosomal peptide synthetase | um05847 | 0.31544 | K01866 | probable TYS1 - tyrosyl-tRNA synthetase |
| 9d00153 | 0.40559 | K06072 | HEAT repeat-containing protein | um11945 | 0.31547 | K00844 | probable hexokinase |
| 9d00354 | 0.40562 | ni | hypothetical protein | um00607 | 0.31548 | ni | related to phosducin homolog |
| 4c00037 | 0.40564 | K03114 | cyclin-dependent kinase WEE1 | um03263 | 0.31548 | K15083 | probable RAD16 - nucleotide excision repair protein |
| 7c00321 | 0.40568 | ni | hypothetical protein | um02260 | 0.31551 | ni | putative protein |
| 9d00268 | 0.40587 | ni | predicted sugar kinase | um03677 | 0.31556 | ni | hypothetical protein |
| 20d00024 | 0.40591 | ni | Zn finger protein | um00906.2 | 0.31574 | ni | conserved hypothetical protein |
| 27d00004 | 0.40602 | K12834 | uncharacterized conserved protein | um02475 | 0.31574 | ni | hypothetical protein |
| 7d00179 | 0.40608 | ni | hypothetical protein | um02826 | 0.31589 | ni | conserved hypothetical protein |
| 9c00072 | 0.4061 | ni | hypothetical protein | um06215 | 0.31592 | ni | related to protein ste16 |
| 7d00059 | 0.40611 | K01598 | thymidylate synthase | um11928 | 0.31603 | ni | related to cyclin |
| 18c00040 | 0.40611 | ni | hypothetical protein | um01615 | 0.31604 | ni | putative protein |
| 9c00345 | 0.40611 | K03941 | NADH:ubiquinone oxidoreductase, NDUFS8/23 kDa subunit | um10029 | 0.31604 | ni | related to Glucan 1 |
| 8c00117 | 0.40613 | ni | structural maintenance of chromosome protein SMC5/Spr18 | um05588 | 0.31606 | K15115 | related to FAD carrier protein FLX1 |
| 8d00074 | 0.40617 | K08739 | DNA mismatch repair protein - MLH3 family | um04013 | 0.31607 | K11676 | conserved hypothetical protein |
| 14d00089 | 0.40637 | ni | hypothetical protein | um03528 | 0.31608 | K01046 | related to TGL2 - triacylglycerol lipase |
| 27c00025 | 0.40638 | ni | permease of the major facilitator superfamily | um10381 | 0.31615 | K11087 | probable small nuclear ribonucleoprotein SNRPD1 |
| 26c00069 | 0.4064 | ni | Ca2+/calmodulin-dependent protein kinase kinase beta | um04229 | 0.31626 | ni | conserved hypothetical protein |
| 9d00410 | 0.4064 | K15198 | transcription initiation factor TFIIIB, Bdp1 subunit | um10134 | 0.31639 | ni | conserved hypothetical protein |
| 12c00049 | 0.40641 | K14778 | ATP-dependent RNA helicase | um10105 | 0.31655 | ni | conserved hypothetical protein |
| 11c00048 | 0.40641 | ni | nuclear transport receptor Karyopherin-beta2/Transportin | um05947.2 | 0.31669 | K01797 | related to DCG1 - involved in nitrogen-catabolite metabolism |
| 19c00144 | 0.40644 | ni | hypothetical protein | um03223 | 0.31681 | ni | Mig1 protein |
| 3c00029 | 0.40647 | ni | hypothetical protein | um03036 | 0.31684 | ni | conserved hypothetical protein |
| 6c00111 | 0.40651 | ni | hypothetical protein | um10627 | 0.31689 | K01803 | related to triose-phosphate isomerase |
| 15d00069 | 0.40657 | ni | hypothetical protein | um05940 | 0.3169 | ni | conserved hypothetical protein |
| 5d00095 | 0.4069 | K06997 | proline synthetase co-transcribed protein | um04081 | 0.31696 | K00799 | related to glutathione S-transferase |
| 11d00049 | 0.40707 | ni | hypothetical protein | um05407 | 0.31721 | ni | probable ALD4 - aldehyde dehydrogenase |
| 8c00071 | 0.40715 | K17080 | prohibitin | um00564 | 0.31734 | K01488 | related to adenosine deaminase |
| 7c00070 | 0.4076 | ni | hypothetical protein | um12136 | 0.31735 | ni | conserved hypothetical protein |
| 6d00011 | 0.40761 | K15283 | glucose-6-phosphate/phosphate and phosphoenolpyruvate/phosp | um05145 | 0.31749 | ni | putative protein |
| 26d00096 | 0.40768 | K12606 | hypothetical Protein | um10674 | 0.31753 | K13107 | related to rna binding motif protein |
| 20c00057 | 0.40774 | K00764 | glutamine phosphoribosylpyrophosphate amidotransferase | um06001 | 0.3176 | ni | conserved hypothetical protein |
| 7c00103 | 0.40781 | K14169 | hypothetical protein | um00540 | 0.31764 | ni | conserved hypothetical protein |
| 22d00272 | 0.40788 | ni | hypothetical protein | um04188 | 0.31765 | K12815 | probable PRP16 - RNA-dependent ATPase |
| 16c00086 | 0.40793 | ni | hypothetical protein | um03461 | 0.31808 | ni | conserved hypothetical protein |
| 22c00269 | 0.40808 | K01062 | phospholipase A2 | um05180 | 0.31832 | K14820 | related to BRX1 - Essential nucleolar protein required for biogenesis of the 60S ribosomal subunit |
| 13c00071 | 0.40845 | K03615 | hypothetical protein | um10651 | 0.31836 | ni | conserved hypothetical protein |
| 20d00006 | 0.40848 | K02178 | mitotic checkpoint serine/threonine protein kinase | um01587 | 0.31845 | ni | ni |
| 7d00101 | 0.4088 | ni | hypothetical protein | um00614 | 0.31864 | K11518 | probable TOM40 - mitochondrial import receptor |
| 22d00187 | 0.40885 | ni | hypothetical protein | um05917 | 0.31865 | K01669 | related to Deoxyribodipyrimidine photolyase |
| 12d00114 | 0.40899 | ni | hypothetical protein | um01931 | 0.31872 | K02350 | probable catalytic subunit of DNA polymerase zeta UPR-1 |
| 19c00021 | 0.40903 | K06269 | serine/threonine specific protein phosphatase PP1, catalytic subu | um06003 | 0.31878 | K09578 | probable prolyl isomerase Ess1 |
| 1c00030 | 0.40911 | K11770 | SWI-SNF chromatin remodeling complex, Snf5 subunit | um11292 | 0.3188 | K06970 | conserved hypothetical protein |
| 9d00321 | 0.40925 | K10842 | predicted E3 ubiquitin ligase | um10230 | 0.31881 | K03031 | hypothetical protein |
| 27d00102 | 0.40953 | ni | ATP-dependent DNA helicase | um02731 | 0.31885 | K05292 | related to GPI-anchor transamidase complex subunit Gpi16 |
| 2d00028 | 0.40965 | K08342 | cysteine protease | um12251 | 0.3192 | ni | conserved hypothetical protein |
| 22d00292 | 0.40999 | K01626 | hypothetical protein | um05538 | 0.31929 | ni | putative protein |
| 25c00007 | 0.41006 | ni | hypothetical protein | um05498 | 0.31956 | ni | related to putative oxysterol-binding protein OSBP |
| 18d00016 | 0.41008 | K00670 | acetyltransferase | um02464 | 0.31957 | ni | related to ABC1 - ubiquinol--cytochrome-c reductase complex assembly protein |
| 7d00080 | 0.41023 | ni | predicted membrane protein | um03599 | 0.31981 | K16948 | probable CDC12 - septin |
| 19d00088 | 0.41031 | K03456 | protein phosphatase 2A regulatory subunit A and related proteins | um02889 | 0.31987 | K08508 | related to SEC9 - protein transport protein |
| 9c00425 | 0.41037 | ni | hypothetical protein | um06036 | 0.31994 | K12871 | Coiled-coil domain-containing protein 12 |
| 7d00294 | 0.4106 | K00600 | glycine/serine hydroxymethyltransferase | um04775 | 0.32025 | K11093 | related to SNP1 - U1 small nuclear ribonucleoprotein |
| 11d00004 | 0.41064 | ni | hypothetical protein | um10437 | 0.3203 | ni | conserved hypothetical protein |
| 7c00324 | 0.4107 | ni | hypothetical protein | um05760 | 0.32066 | ni | hypothetical protein |
| 22d00085 | 0.41088 | K11806 | sof1-like rRNA processing protein | um01321 | 0.32067 | K03251 | probable eIF3d - eukaryotic translation initiation factor 3 subunit D |
| 14c00098 | 0.41093 | K08775 | DNA recombinational repair protein BRCA2 | um04732 | 0.32091 | K07179 | related to RIO2 protein |
| 27d00036 | 0.41124 | ni | hypothetical protein | um05446 | 0.32094 | ni | conserved hypothetical protein |
| 5d00013 | 0.41145 | K00940 | nucleoside diphosphate kinase | um12187 | 0.32098 | ni | putative protein |
| 12d00074 | 0.41152 | K09493 | chaperonin complex component, TCP-1 alpha subunit | um11767 | 0.3211 | ni | conserved hypothetical protein |
| 12c00138 | 0.41168 | K03039 | 26S proteasome regulatory complex, subunit RPN9/PSMD13 | um12246 | 0.32112 | ni | conserved hypothetical protein |
| 22d00288 | 0.41168 | ni | hypothetical protein | um04275 | 0.32113 | K08736 | related to DNA mismatch repair protein |
| 19c00077 | 0.4118 | K16186 | predicted small GTPase involved in nuclear protein import | um04006 | 0.32117 | ni | conserved hypothetical protein |
| 7c00303 | 0.41188 | ni | class 2 transcription repressor NC2, alpha subunit | um11059 | 0.3212 | ni | hypothetical protein |
| 8c00035 | 0.41212 | ni | hypothetical protein | um12114 | 0.32136 | ni | conserved hypothetical protein |
| 19d00109 | 0.41228 | ni | hypothetical protein | um00361 | 0.32142 | K16073 | related to MNR2 - Manganese resistance protein |
| 3c00052 | 0.4124 | ni | hypothetical protein | um04893 | 0.32149 | ni | conserved hypothetical protein |
| 5c00064 | 0.41253 | ni | hypothetical protein | um04661 | 0.32176 | ni | conserved hypothetical protein |
| 20d00066 | 0.41262 | K09578 | peptidyl-prolyl cis-trans isomerase | um04928 | 0.32181 | ni | conserved hypothetical protein |
| 5c00043 | 0.41264 | ni | molecular chaperone | um03150 | 0.32184 | ni | conserved hypothetical protein |
| 13d00008 | 0.41283 | ni | lysosomal trafficking regulator LYST and related BEACH and WD | um10128 | 0.32199 | K12626 | related to snRNP protein |
| 22c00101 | 0.41317 | ni | hypothetical protein | um10916 | 0.32202 | ni | putative protein |
| 25d00068 | 0.41327 | K03868 | SCF ubiquitin ligase, Rbx1 component | um11227 | 0.32212 | K15171 | probable SPT4 - transcription elongation protein |
| 16c00064 | 0.41337 | ni | hypothetical protein | um01121 | 0.32246 | K03038 | probable RPN8 - 26S proteasome regulatory subunit |
| 5d00139 | 0.41357 | ni | hypothetical protein | um10279 | 0.3226 | ni | b-type cyclin 2 |

| | | | | | | | |
|---|---|---|---|---|---|---|---|
| 27d00100 | 0.41363 | K03679 | exosomal 3'-5' exoribonuclease complex, subunit Rrp4 | um00207.2 | 0.32263 | ni | related to mitochondrial carrier family protein |
| 3d00012 | 0.41373 | ni | hypothetical protein | um10376 | 0.32271 | ni | conserved hypothetical protein |
| 22c00197 | 0.41384 | ni | hypothetical protein | um00477 | 0.32285 | ni | related to Monocarboxylate transporter 1 |
| 19c00056 | 0.41388 | ni | uncharacterized conserved protein | um04789 | 0.32309 | K14785 | conserved hypothetical protein |
| 5c00066 | 0.41391 | ni | hypothetical protein | um10824 | 0.3231 | K12397 | related to Beta3 protein |
| 22d00218 | 0.41401 | ni | hypothetical protein | um02075 | 0.32311 | ni | related to calpain-like protease PalBory |
| 25c00044 | 0.4141 | ni | dimeric dihydrodiol dehydrogenase | um06249 | 0.3232 | ni | putative protein |
| 22c00098 | 0.41423 | K01647 | citrate synthase | um00375 | 0.32327 | ni | conserved hypothetical protein |
| 15c00094 | 0.41427 | ni | voltage-gated shaker-like K+ channel, subunit beta/KCNAB | um00947 | 0.32337 | ni | related to Stress-activated map kinase interacting protein 1 |
| 1d00051 | 0.41509 | ni | hypothetical protein | um10955 | 0.32348 | ni | conserved hypothetical protein |
| 9d00242 | 0.41515 | ni | hypothetical protein | um02924 | 0.32353 | K02873 | probable ribosomal protein L13B |
| 7d00055 | 0.41541 | K04799 | 5'-3' exonuclease | um11194 | 0.32357 | K03544 | related to atp-dependent clp protease |
| 20d00017 | 0.41561 | K03783 | purine nucleoside phosphorylase | um06388 | 0.32374 | K02954 | related to MRP2 - mitochondrial ribosomal protein |
| 15c00042 | 0.41563 | ni | hypothetical protein | um05995 | 0.32378 | ni | related to Acid phosphatase precursor |
| 89d00001 | 0.41567 | ni | ni | um04227 | 0.32386 | ni | putative protein |
| 11d00027 | 0.41571 | ni | hypothetical protein | um05361 | 0.32401 | ni | related to Laccase I precursor |
| 25d00081 | 0.41591 | ni | hypothetical protein | um05377 | 0.32419 | ni | conserved hypothetical protein |
| 19d00113 | 0.41624 | K00833 | acetylornithine aminotransferase | um06349 | 0.32427 | ni | related to siderophore iron transporter mirc |
| 22c00268 | 0.41629 | K01477 | allantoicase | um05773 | 0.32456 | ni | putative protein |
| 7c00113 | 0.41646 | ni | hypothetical protein | um01961 | 0.3246 | ni | hypothetical protein |
| 26d00063 | 0.41647 | K09549 | molecular chaperone Prefoldin, subunit 2 | um02089 | 0.32482 | K03350 | related to nuclear protein bimA |
| 15d00090 | 0.41658 | K13348 | peroxisomal membrane protein MPV17 and related proteins | um05527 | 0.32482 | ni | conserved hypothetical protein |
| 8d00075 | 0.41667 | ni | hypothetical protein | um03244 | 0.32488 | K11319 | conserved hypothetical protein |
| 10d00094 | 0.41669 | K16261 | amino acid transporters | um06255 | 0.32495 | ni | hypothetical protein |
| 15d00071 | 0.41688 | ni | hypothetical protein | um04350 | 0.32514 | ni | related to 3-phytase A precursor |
| 7c00218 | 0.41699 | ni | translocase of outer mitochondrial membrane complex, subunit T | um10809.2 | 0.3253 | ni | hypothetical protein |
| 22c00188 | 0.41743 | ni | hypothetical protein | um04811 | 0.32554 | ni | related to ADP1 - ABC transporter |
| 9c00403 | 0.41745 | K02918 | 60S ribosomal protein L35 | um04744 | 0.32603 | ni | conserved hypothetical protein |
| 18c00081 | 0.41761 | K10844 | RNA polymerase II transcription initiation/nucleotide excision repa | um11305 | 0.32606 | ni | conserved hypothetical protein |
| 13c00053 | 0.41765 | K02892 | hypothetical protein | um11616 | 0.32619 | ni | related to TIM13 - Translocase of the inner membrane |
| 22d00244 | 0.41782 | ni | hypothetical protein | um03293 | 0.32628 | K01530 | related to DNF1 - protein transporter |
| 27c00004 | 0.41788 | K02899 | mitochondrial ribosomal protein MRP7 | um02688 | 0.32658 | ni | putative protein |
| 10d00080 | 0.4179 | K11090 | FOG, RRM domain | um01360 | 0.32709 | ni | hypothetical protein |
| 6c00088 | 0.41804 | ni | hypothetical protein | um11852 | 0.32711 | K08773 | related to BEM3 - GTPase-activating protein |
| 11c00032 | 0.41807 | K00801 | squalene synthetase | um01913 | 0.32724 | ni | putative protein |
| 22c00169 | 0.41814 | ni | hypothetical protein | um01657 | 0.32739 | ni | putative protein |
| 13c00001 | 0.41821 | ni | hypothetical protein | um10430 | 0.3274 | ni | related to cell cycle control protein cwf25 |
| 25d00043 | 0.41823 | ni | 1-aminocyclopropane-1-carboxylate synthase | um10729 | 0.32751 | ni | conserved hypothetical protein |
| 4d00002 | 0.4183 | ni | hypothetical protein | um04846 | 0.32758 | K01556 | related to kynureninase |
| 9c00082 | 0.41832 | ni | hypothetical protein | um00424 | 0.32758 | ni | related to Choline dehydrogenase |
| 16d00017 | 0.41838 | ni | hypothetical protein | um10724 | 0.32759 | K02867 | probable MRPL19 - mitochondrial ribosomal protein |
| 22c00297 | 0.41843 | ni | hypothetical protein | um01249 | 0.32803 | K12622 | probable LSM3 - U6 snRNA-associated Sm-like protein |
| 10c00031 | 0.41859 | K14563 | fibrillarin and related nucleolar RNA-binding proteins | um11563 | 0.32803 | ni | conserved hypothetical protein |
| 7d00247 | 0.41862 | ni | hypothetical protein | um10863 | 0.32818 | K14845 | related to RAI1 - Rat1p Interacting Protein |
| 9d00359 | 0.41864 | ni | hypothetical protein | um02045 | 0.32819 | ni | conserved hypothetical protein |
| 22d00011 | 0.41868 | K02327 | DNA polymerase delta, catalytic subunit | um02823 | 0.32846 | K11269 | related to CTF18 - Chromosome Transmission Fidelity factor |
| 7d00193 | 0.41881 | ni | hypothetical protein | um02537 | 0.32854 | ni | conserved hypothetical Ustilago-specific protein |
| 10d00035 | 0.41884 | K01057 | 6-phosphogluconolactonase - like protein | um01954 | 0.32857 | ni | conserved hypothetical protein |
| 22c00147 | 0.41896 | ni | hypothetical protein | um00317 | 0.32861 | ni | hypothetical protein |
| 9d00206 | 0.4192 | K01259 | hypothetical protein | um03700 | 0.32864 | K16261 | probable DIP5 - Glutamate and aspartate permease - able to mediate transport of other amino acids |
| 22d00297 | 0.41929 | ni | hypothetical protein | um02966 | 0.32878 | K14721 | conserved hypothetical protein |
| 12d00051 | 0.41945 | K03251 | translation initiation factor 3, subunit d | um01624 | 0.32891 | K14006 | probable SEC23 - component of COPII coat of ER-golgi vesicles |
| 7c00099 | 0.41946 | ni | nuclear pore complex, p54 component | um05128 | 0.32892 | ni | conserved hypothetical protein |
| 2d00073 | 0.41953 | K14842 | uncharacterized conserved protein related to ribosomal protein S8 | um11952 | 0.32894 | K04079 | probable heat shock protein 80 |
| 18c00103 | 0.4196 | ni | hypothetical protein | um01161 | 0.32897 | ni | related to BEM2 - GTPase-activating protein |
| 7d00343 | 0.41968 | K02875 | 60S ribosomal protein L14 | um02264 | 0.32901 | ni | putative protein |
| 14d00085 | 0.41972 | ni | hypothetical protein | um10371 | 0.32909 | K08266 | related to LST8 - required for transport of permeases from the golgi to the plasma membrane |
| 22c00246 | 0.41991 | ni | hypothetical protein | um01159 | 0.32931 | ni | conserved hypothetical protein |
| 1d00037 | 0.41993 | ni | hypothetical protein | um01156 | 0.32948 | ni | related to myo-inositol transporter |
| 18d00024 | 0.42019 | ni | MEKK and related serine/threonine protein kinases | um06363 | 0.3297 | K08269 | related to APG1 - essential for autophagocytosis |
| 15d00057 | 0.4202 | ni | flavin-containing monooxygenase | um02227 | 0.32972 | K01230 | related to alpha-mannosidase |
| 9c00203 | 0.4202 | ni | hypothetical protein | um15013.2 | 0.32993 | K12175 | related to COP9 signalosome complex subunit 1 |
| 20d00078 | 0.42022 | ni | hypothetical protein | um01531 | 0.33001 | ni | related to Importin 11 |
| 13d00076 | 0.42024 | ni | transport protein particle TRAPP complex subunit | um05278 | 0.33011 | ni | conserved hypothetical protein |
| 7c00280 | 0.42031 | K06867 | FOG, Ankyrin repeat | um10036 | 0.33011 | K03352 | conserved hypothetical protein |
| 6c00119 | 0.42047 | ni | hypothetical protein | um12291 | 0.33021 | ni | conserved hypothetical protein |
| 15d00027 | 0.4205 | ni | hypothetical protein | um12182 | 0.33045 | ni | putative protein |
| 8c00069 | 0.42052 | ni | hypothetical protein | um05922 | 0.33061 | ni | related to RMD8 - Cytosolic protein required for sporulation |
| 6d00134 | 0.42064 | K13100 | uncharacterized conserved protein | um04038 | 0.33071 | ni | hypothetical protein |
| 27c00006 | 0.42064 | ni | regulator of Rac1 | um10923.2 | 0.33085 | ni | conserved hypothetical protein |
| 13d00049 | 0.42077 | K12843 | putative u4/u6 small nuclear ribonucleoprotein | um05191.2 | 0.33096 | ni | putative protein |
| 7d00347 | 0.4208 | K01881 | prolyl-trna synthetase | um11736 | 0.33099 | ni | putative protein |
| 8c00121 | 0.4211 | ni | hypothetical protein | um04849 | 0.33112 | ni | hypothetical protein |
| 26c00031 | 0.42117 | K02922 | 60S ribosomal protein L37 | um05458 | 0.33122 | ni | related to CCH1 - Calcium channel |
| 9c00009 | 0.42123 | ni | predicted steroid reductase | um10338 | 0.33125 | ni | conserved hypothetical protein |
| 12c00124 | 0.42134 | K03027 | RNA polymerase I and III, subunit RPA40/RPC40 | um10278 | 0.33136 | ni | putative protein |
| 22d00248 | 0.42137 | ni | hypothetical protein | um02221 | 0.33154 | ni | conserved hypothetical protein |
| 8d00104 | 0.42139 | ni | hypothetical protein | um03435 | 0.33161 | ni | conserved hypothetical protein |
| 14c00124 | 0.4217 | ni | FOG, Zn-finger | um01210 | 0.33172 | ni | related to mitochondrial import receptor subunit TOM20 |
| 5d00003 | 0.42175 | K01613 | phosphatidylserine decarboxylase | um10457 | 0.33173 | ni | related to protein involved in intramitochondrial protein sorting |
| 22c00236 | 0.42191 | K00540 | NADH, flavin oxidoreductase | um10195 | 0.3321 | K10956 | probable endoplasmic reticulum insertion protein SEC61 |
| 6c00132 | 0.42195 | ni | hypothetical protein | um10662 | 0.33217 | K15544 | probable SSU72 - Nuclear zinc-finger motif containing protein |
| 3d00063 | 0.42218 | ni | hypothetical protein | um03055 | 0.33226 | ni | conserved hypothetical protein |
| 2c00021 | 0.42218 | ni | hypothetical protein | um12168 | 0.33248 | ni | putative protein |
| 7d00147 | 0.42219 | K02931 | mitochondrial/chloroplast ribosomal protein L5/L7 | um02338 | 0.3326 | K08488 | related to PEP12 syntaxin |
| 6c00065 | 0.42254 | ni | aquaporin | um01006 | 0.33266 | ni | conserved hypothetical protein |
| 25c00084 | 0.42257 | K03939 | NADH:ubiquinone oxidoreductase, NDUFS6/13 kDa subunit | um10787 | 0.3327 | K01679 | probable FUM1 - fumarate hydratase |
| 7c00299 | 0.4226 | ni | hypothetical protein | um06383 | 0.33273 | ni | related to MAP kinase |
| 6c00095 | 0.42265 | K02835 | mitochondrial polypeptide chain release factor | um00171 | 0.33276 | ni | putative protein |
| 22c00299 | 0.42279 | ni | hypothetical protein | um01732 | 0.33296 | K14811 | probable DBP3 - putative RNA helicase required for pre-rRNA processing |
| 21c00003 | 0.42294 | ni | hypothetical protein | um02351 | 0.33317 | K14684 | related to mitochondrial carrier protein |
| 22c00141 | 0.42295 | ni | hypothetical protein | um05115 | 0.33318 | ni | conserved hypothetical protein |
| 10c00056 | 0.42303 | ni | hypothetical protein | um01215 | 0.33333 | ni | related to vacuolar segregation protein PEP7 |
| 2c00084 | 0.42307 | ni | sirtuin 4 and related class II sirtuins | um02044 | 0.33337 | K01874 | probable METHIONYL-TRNA SYNTHETASE |
| 12d00080 | 0.42308 | K14753 | G protein beta subunit-like protein | um04278.2 | 0.33339 | ni | putative protein |
| 27d00080 | 0.4231 | K11667 | uncharacterized conserved protein | um12319 | 0.33356 | ni | hypothetical protein |
| 8c00055 | 0.42312 | ni | hypothetical protein | um03950 | 0.33372 | K15304 | related to YRB2 - Ran-GTPase-binding protein involved in nuclear protein export |
| 18d00116 | 0.42313 | ni | hypothetical protein | um15079 | 0.33376 | ni | putative protein |
| 9c00325 | 0.42315 | K14455 | aspartate aminotransferase | um03047 | 0.33382 | ni | hypothetical protein |
| 26d00071 | 0.42347 | ni | hypothetical protein | um11901 | 0.33385 | ni | related to Lactamase |
| 24d00043 | 0.42372 | ni | hypothetical protein | um02895 | 0.3341 | ni | related to ERG27 - 3-keto sterol reductase |
| 7d00149 | 0.42384 | K13431 | signal recognition particle receptor, alpha subunit | um00703 | 0.3343 | K04536 | G-protein beta subunit Bpp1 |
| 11c00087 | 0.42387 | ni | invasion-inducing protein TIAM1/CDC24 | um11160 | 0.33434 | ni | hypothetical protein |
| 14c00009 | 0.42402 | ni | uncharacterized conserved protein | um01427 | 0.33437 | ni | related to Crg1 protein |
| 5d00014 | 0.42419 | K02933 | mitochondrial/chloroplast ribosomal protein L6 | um04072 | 0.33456 | ni | conserved hypothetical protein |
| 2d00060 | 0.42423 | K08734 | DNA mismatch repair protein - MLH1 family | um05583 | 0.33485 | ni | putative protein |
| 15d00024 | 0.42452 | ni | hypothetical protein | um05659 | 0.3349 | ni | conserved hypothetical protein |
| 6c00026 | 0.42454 | ni | methyltransferase | um02559 | 0.33494 | K15306 | probable YRB1 - ran-specific GTPase-activating protein |
| 7c00227 | 0.42457 | K11699 | RNA-directed RNA polymerase QDE-1 | um11477 | 0.33503 | ni | conserved hypothetical protein |
| 5c00124 | 0.4246 | ni | cholesterol transport protein | um03762 | 0.33509 | K14298 | related to GLE2 - required for nuclear pore complex structure and function |
| 7d00072 | 0.42471 | ni | hypothetical protein | um10874 | 0.33512 | ni | conserved hypothetical protein |
| 6c00099 | 0.42481 | ni | hypothetical protein | um04886 | 0.3354 | ni | related to HOL1 protein |
| 10c00069 | 0.42486 | ni | hypothetical protein | um10837 | 0.33558 | ni | conserved hypothetical protein |
| 25c00032 | 0.42498 | ni | hypothetical protein | um02268 | 0.33571 | ni | putative protein |
| 4c00030 | 0.42517 | K00799 | glutathione S-transferase | um04031 | 0.3358 | ni | related to BST1 - negative regulator of COPII vesicle formation |
| 6c00133 | 0.4252 | ni | FOG, Zn-finger | um02079 | 0.33588 | K02947 | probable 40S ribosomal protein S10 |
| 27c00034 | 0.42521 | ni | hypothetical protein | um12229 | 0.33598 | ni | related to dioxygenase |
| 10d00072 | 0.42539 | K11426 | predicted histone tail methylase containing SET domain | um01284 | 0.33636 | K06027 | probable SEC18 - vesicular-fusion protein |
| 6c00003 | 0.42544 | ni | hypothetical protein | um02828 | 0.33647 | ni | conserved hypothetical protein |
| 9c00269 | 0.42551 | ni | hypothetical protein | um02558 | 0.33678 | K15202 | conserved hypothetical protein |
| 19c00046 | 0.42559 | ni | density-regulated protein related to translation initiation factor 1 | um01652 | 0.33704 | K06699 | conserved hypothetical protein |
| 5c00111 | 0.42559 | ni | hypothetical protein | um03809 | 0.33712 | K00889 | related to phosphatidylinositol-4-phosphate 5-kinase |
| 9d00168 | 0.42565 | ni | hypothetical protein | um11330 | 0.33715 | ni | related to O-methyltransferase |
| 5c00082 | 0.42576 | ni | hypothetical protein | um02788 | 0.33738 | ni | hypothetical protein |
| 6d00018 | 0.42577 | ni | hypothetical protein | um12279 | 0.33766 | ni | putative protein |
| 22c00125 | 0.42584 | ni | hypothetical protein | um00175 | 0.33766 | K03249 | related to eIF3f - translation initiation factor 3 subunit F |
| 2c00028 | 0.42588 | K02998 | 40S ribosomal protein SA/Laminin receptor 1 | um11718 | 0.3377 | ni | conserved hypothetical protein |
| 7c00044 | 0.42608 | ni | hypothetical protein | um06234 | 0.33788 | K02730 | probable SCL1 - 20S proteasome subunit |
| 16c00030 | 0.42637 | ni | cystathionine beta-lyases | um10972 | 0.33809 | ni | hypothetical protein |
| 22c00174 | 0.42641 | ni | hypothetical protein | um06237 | 0.33812 | K15176 | related to Tetratricopeptide repeat protein 1 |
| 18d00014 | 0.4266 | ni | hypothetical protein | um04381 | 0.3382 | K11648 | related to SNF5 - component of SWI |
| 10c00028 | 0.42727 | ni | hypothetical protein | um10881 | 0.33827 | ni | probable ribonuclease T1 |
| 19d00132 | 0.42737 | K09548 | hypothetical protein | um00532 | 0.33827 | ni | putative protein |
| 27c00076 | 0.42739 | ni | hypothetical protein | um10595 | 0.33828 | K08869 | conserved hypothetical protein |
| 13d00068 | 0.42751 | ni | signal recognition particle, subunit Srp19 | um00282 | 0.33869 | ni | probable RNA helicase |
| 6c00123 | 0.42762 | ni | hypothetical protein | um03143 | 0.3389 | ni | conserved hypothetical protein |
| 3c00062 | 0.42765 | ni | predicted transporter | um01963 | 0.33891 | ni | conserved hypothetical protein |
| 7d00186 | 0.42766 | ni | hypothetical protein | um05435 | 0.33898 | ni | related to Glutaredoxin |
| 10d00009 | 0.4277 | K02685 | eukaryotic-type DNA primase, large subunit | um11893 | 0.33907 | ni | hypothetical protein |
| 6d00025 | 0.42782 | ni | hypothetical protein | um15064 | 0.33938 | ni | conserved hypothetical protein |
| 14c00089 | 0.42792 | K14686 | hypothetical protein | um06356 | 0.33942 | ni | conserved hypothetical Ustilago-specific protein |
| 5d00069 | 0.42797 | ni | hypothetical protein | um11539 | 0.33946 | K12188 | related to SNF8 - protein involved in glucose derepression |
| 8c00111 | 0.42805 | ni | hypothetical protein | um03162 | 0.33947 | K01880 | probable glycine--tRNA ligase GRS1 |
| 25c00021 | 0.42825 | K12893 | alternative splicing factor SRp55/B52/SRp75 | um05151 | 0.33948 | K06634 | related to Cyclin H |
| 16c00015 | 0.42826 | K02263 | cytochrome c oxidase, subunit IV/COX5b | um15055 | 0.33957 | ni | hypothetical protein |
| 18c00084 | 0.42848 | ni | dopey and related predicted leucine zipper transcription factors | um11245 | 0.33964 | ni | related to FRE6 - Ferric reductase |
| 11d00019 | 0.42853 | ni | hypothetical protein | um00505 | 0.33973 | ni | related to BRO1 - cytoplasmic class E vacuolar protein sorting |
| 5d00065 | 0.42855 | K00868 | pyridoxal/pyridoxine/pyridoxamine kinase | um11414 | 0.33979 | ni | conserved hypothetical protein |
| 8d00003 | 0.42855 | ni | hypothetical protein | um00137 | 0.33995 | ni | putative protein |
| 9c00048 | 0.42879 | ni | hypothetical protein | um03233 | 0.34001 | ni | related to Aldo-keto reductase yakc |
| 12d00053 | 0.42894 | K02949 | 40S ribosomal protein S11 | um11538.2 | 0.34005 | ni | putative protein |
| 2d00003 | 0.42897 | ni | hypothetical protein | um11978 | 0.34013 | ni | conserved hypothetical protein |
| 10d00041 | 0.42898 | ni | hypothetical protein | um11526 | 0.34013 | K02868 | probable RPL11B - ribosomal protein L11 |
| 18c00022 | 0.42916 | K10878 | catalytic subunit of the meiotic double strand break transesterase | um10944 | 0.34036 | K13617 | related to protein phosphatase methylesterase 1 |
| 7c00045 | 0.42947 | K13989 | predicted membrane protein | um10508 | 0.34046 | K14773 | conserved hypothetical protein |
| 2d00029 | 0.42947 | ni | hypothetical protein | um10832 | 0.34052 | K05759 | probable PFY1 - profilin |

| | | | | | | | |
|---|---|---|---|---|---|---|---|
| 5d00030 | 0.42949 | K10847 | DNA excision repair protein XPA/XPAC/RAD14 | um11532 | 0.34064 | ni | conserved hypothetical protein |
| 14d00120 | 0.42968 | K03539 | protein subunit of nuclear ribonuclease P | um10763 | 0.34081 | K03469 | conserved hypothetical protein |
| 7d00185 | 0.42971 | ni | hypothetical protein | um00012 | 0.34091 | ni | related to ZIM17 - Zinc finger Motif protein |
| 19c00011 | 0.42981 | ni | hypothetical protein | um06206 | 0.34112 | ni | conserved hypothetical protein |
| 22c00165 | 0.42989 | K13728 | mitotic spindle checkpoint protein | um01227 | 0.34115 | ni | hypothetical protein |
| 9d00019 | 0.43008 | ni | hypothetical protein | um03908 | 0.34128 | ni | related to TPO1 - Vacuolar polyamine-H antiporter |
| 13c00011 | 0.4301 | ni | glucose-repressible alcohol dehydrogenase transcriptional effectc | um11312 | 0.34128 | ni | conserved hypothetical protein |
| 15d00017 | 0.43018 | ni | subunit of cis-Golgi transport vesicle tethering complex - Sec34p | um03511 | 0.34129 | K02995 | probable RPS8A - ribosomal protein S8 |
| 22d00078 | 0.43018 | ni | uncharacterized conserved protein | um11494 | 0.34131 | K14012 | related to SHP1 - potential regulatory subunit for Glc7p |
| 11d00010 | 0.43021 | ni | hypothetical protein | um01032 | 0.34133 | ni | related to ROM2 - GDP |
| 16d00082 | 0.43025 | ni | hypothetical protein | um04681 | 0.34136 | ni | hypothetical protein |
| 9d00193 | 0.43027 | K02936 | 60S ribosomal protein L7A | um11770 | 0.34141 | ni | conserved hypothetical protein |
| 5d00002 | 0.4303 | K00262 | glutamate/leucine/phenylalanine/valine dehydrogenases | um04271 | 0.34143 | ni | putative protein |
| 7d00152 | 0.43043 | K00058 | D-3-phosphoglycerate dehydrogenase | um06035 | 0.34155 | K00764 | probable ADE4 - amidophosphoribosyltransferase |
| 25d00005 | 0.4305 | ni | hypothetical protein | um10048 | 0.34178 | ni | related to COX8 - cytochrome-c oxidase chain VIII |
| 13c00028 | 0.43059 | K12737 | peptidyl-prolyl cis-trans isomerase | um03803 | 0.34181 | K10389 | gamma-tubulin |
| 7d00153 | 0.43067 | K01372 | bleomycin hydrolases and aminopeptidases of cysteine protease f | um04831 | 0.34194 | ni | probable MGM101 - mitochondrial genome maintenance protein |
| 1d00001 | 0.43075 | ni | hypothetical protein | um04157 | 0.34199 | ni | conserved hypothetical protein |
| 22c00296 | 0.43082 | K15979 | transcriptional coactivator p100 | um00786 | 0.342 | ni | conserved hypothetical protein |
| 9c00255 | 0.43094 | ni | hypothetical protein | um11017 | 0.34201 | ni | related to CAP59 |
| 22c00001 | 0.43101 | K10759 | hypothetical protein | um10554 | 0.34203 | ni | conserved hypothetical Ustilago-specific protein |
| 12c00069 | 0.43101 | ni | hypothetical protein | um05310 | 0.34212 | ni | conserved hypothetical Ustilago-specific protein |
| 2c00026 | 0.43104 | K07300 | Ca2+/H+ antiporter VCX1 | um05239 | 0.34218 | ni | related to NAD-dependent deacetylase sirtuin type 4 |
| 16c00057 | 0.43107 | K01858 | myo-inositol-1-phosphate synthase | um02211 | 0.34258 | K08073 | related to bifunctional polynucleotide phosphatase |
| 3d00006 | 0.43107 | ni | hypothetical protein | um00762 | 0.34261 | ni | hypothetical protein |
| 7d00141 | 0.43124 | K12857 | U5 snRNP-specific protein-like factor and related proteins | um10191 | 0.34266 | ni | conserved hypothetical protein |
| 7c00029 | 0.43127 | ni | mitochondrial carnitine-acylcarnitine carrier protein | um11943.2 | 0.34271 | ni | conserved hypothetical protein |
| 9c00143 | 0.43132 | ni | mitochondrial fatty acid anion carrier protein | um02850 | 0.34282 | ni | ni |
| 27d00015 | 0.43137 | K03320 | ammonia permease | um05819 | 0.34303 | ni | hypothetical protein |
| 10c00086 | 0.43149 | ni | mitochondrial/plastidial beta-ketoacyl-ACP reductase | um11842 | 0.34318 | ni | conserved hypothetical protein |
| 22d00179 | 0.43156 | ni | uncharacterized conserved protein | um02067 | 0.34324 | K03686 | related to MDJ1 - heat shock protein |
| 19d00072 | 0.43156 | ni | beta-trcp (transducin repeats containing)/Slimb proteins | um01230 | 0.34329 | K14676 | related to NTE1 - Serine esterase |
| 13c00090 | 0.43179 | ni | hypothetical protein | um11650 | 0.34366 | ni | related to Oxidoreductase |
| 20c00062 | 0.43182 | ni | hypothetical protein | um05734 | 0.34368 | K04513 | probable GTPase Rho1 |
| 9d00187 | 0.43183 | ni | uncharacterized conserved protein | um02853 | 0.34369 | ni | conserved hypothetical Ustilago-specific protein |
| 12c00025 | 0.4321 | ni | hypothetical protein | um01298.2 | 0.34389 | ni | conserved hypothetical Ustilago-specific protein |
| 10c00077 | 0.43214 | ni | uncharacterized conserved protein | um04985 | 0.34424 | ni | probable ORM1 - unfolded protein response protein |
| 9c00179 | 0.43238 | ni | lish motif-containing protein | um01099 | 0.34427 | K00022 | probable short chain 3-hydroxyacyl-CoA dehydrogenase |
| 1d00036 | 0.43262 | ni | hypothetical protein | um05354.2 | 0.34428 | ni | conserved hypothetical protein |
| 12d00062 | 0.43262 | ni | hypothetical protein | um05934 | 0.34437 | ni | putative protein |
| 25d00021 | 0.4329 | ni | hypothetical protein | um01252 | 0.3444 | K06943 | probable NOG1 - nucleolar G-protein required for 60S ribosomal subunit biogenesis |
| 9d00181 | 0.43302 | K10689 | ubiquitin-protein ligase | um10568.2 | 0.34479 | ni | conserved hypothetical protein |
| 8c00095 | 0.4332 | ni | lipid exporter ABCA1 and related proteins | um05753 | 0.34482 | ni | hypothetical protein |
| 26d00005 | 0.43334 | K08337 | ubiquitin activating E1 enzyme-like protein | um10804 | 0.34489 | K00658 | probable KGD2 - dihydrolipoyl transsuccinylase component of the alpha-ketoglutarate dehydrogenase complex |
| 15d00016 | 0.43345 | ni | hypothetical protein | um00300 | 0.34502 | ni | conserved hypothetical protein |
| 5c00080 | 0.43345 | ni | hypothetical protein | um01814 | 0.34511 | ni | conserved hypothetical protein |
| 26c00081 | 0.43345 | K17387 | kinesin-like protein | um10037 | 0.34513 | ni | probable mitochondrial import receptor subunit tom7 |
| 7c00249 | 0.4335 | K15113 | mitochondrial carrier protein MRS3/4 | um00658 | 0.34526 | K02953 | probable 40s ribosomal protein S13 |
| 9d00345 | 0.43367 | ni | membrane coat complex Retromer, subunit VPS5/SNX1 | um10478 | 0.34551 | ni | related to RER1 protein |
| 25c00090 | 0.43374 | ni | hypothetical protein | um02226 | 0.34551 | ni | conserved hypothetical protein |
| 22d00054 | 0.43375 | K01889 | phenylalanyl-trna synthetase, beta subunit | um04816 | 0.34561 | ni | related to Endoglucanase 1 precursor |
| 9d00387 | 0.43385 | ni | hypothetical protein | um00563 | 0.34565 | ni | related to MRPL33 - mitochondrial ribosomal protein |
| 5c00128 | 0.43395 | K03062 | 26S proteasome regulatory complex, ATPase RPT2 | um02141 | 0.34575 | ni | effector family protein Eff1-11 |
| 22c00312 | 0.43406 | K13704 | predicted alpha/beta hydrolase | um01511 | 0.34582 | ni | hypothetical protein |
| 9c00122 | 0.43415 | ni | synaptic vesicle transporter SV2 | um11405 | 0.34589 | K03358 | related to anaphase promoting complex subunit 11 |
| 22d00124 | 0.43451 | K03657 | 3'-5' DNA helicase | um04301 | 0.34601 | K14827 | conserved hypothetical protein |
| 13c00006 | 0.43453 | ni | synaptic vesicle transporter SVOP and related transporters | um01387 | 0.3461 | ni | putative protein |
| 9c00173 | 0.43476 | K14829 | WD40 repeat protein | um11911 | 0.34612 | K08770 | probable Polyubiquitin protein |
| 7d00204 | 0.43482 | K03002 | RNA polymerase I, second largest subunit | um00310 | 0.34621 | K14829 | related to WD-repeat protein crb3 |
| 9c00253 | 0.43489 | K06678 | chromosome condensation complex Condensin, subunit G | um02656 | 0.34624 | ni | conserved hypothetical protein |
| 4d00020 | 0.43491 | K16302 | predicted membrane protein | um01512 | 0.34643 | K11718 | related to UDP-glucose |
| 9d00172 | 0.43526 | ni | GTP-binding ADP-ribosylation factor Arf1 | um02452 | 0.34647 | ni | putative protein |
| 25c00056 | 0.43546 | ni | predicted membrane protein | um15039 | 0.3465 | K15223 | conserved hypothetical protein |
| 9c00086 | 0.43556 | ni | FOG, Zn-finger | um04251 | 0.34666 | ni | conserved hypothetical protein |
| 3d00091 | 0.4356 | ni | hypothetical protein | um02242 | 0.34671 | K07566 | related to translation initiation protein SUA5 |
| 19c00149 | 0.43574 | ni | hypothetical protein | um00423 | 0.34673 | K10364 | related to CAP1 - F-actin capping protein alpha subunit |
| 22c00216 | 0.4358 | ni | hypothetical protein | um01841 | 0.34691 | ni | hypothetical protein |
| 7d00068 | 0.43588 | ni | phosphatidic acid-preferring phospholipase A1 | um10298 | 0.34693 | ni | putative protein |
| 4d00024 | 0.43595 | K02996 | mitochondrial/chloroplast ribosomal protein S9 | um12308 | 0.34704 | ni | conserved hypothetical protein |
| 2c00057 | 0.43603 | ni | zinc-binding protein of the histidine triad | um01359 | 0.34717 | ni | conserved hypothetical protein |
| 9d00346 | 0.43605 | K00249 | short-chain acyl-CoA dehydrogenase | um05830 | 0.34719 | ni | putative protein |
| 22c00048 | 0.43624 | ni | hypothetical protein | um05846 | 0.34733 | ni | probable MSH1 - DNA mismatch repair protein |
| 13d00106 | 0.43638 | ni | hypothetical protein | um10284 | 0.34741 | ni | probable vacuolar sorting protein |
| 7d00309 | 0.43644 | ni | hypothetical protein | um02989 | 0.34745 | ni | related to IME4 - positive transcription factor for IME2 |
| 18c00067 | 0.43653 | K00211 | prephenate dehydrogenase | um10500 | 0.34746 | ni | hypothetical protein |
| 15c00073 | 0.43688 | ni | amphiphysin | um02844 | 0.34747 | ni | conserved hypothetical protein |
| 27d00019 | 0.43691 | K02320 | DNA polymerase alpha, catalytic subunit | um02123 | 0.34764 | K06877 | conserved hypothetical protein |
| 5c00072 | 0.43703 | K03036 | 26S proteasome regulatory complex, subunit RPN6/PSMD11 | um01077.2 | 0.34773 | ni | putative protein |
| 6d00029 | 0.43715 | ni | hypothetical protein | um01132 | 0.34773 | ni | hypothetical protein |
| 16d00007 | 0.4373 | ni | hypothetical protein | um04119 | 0.34775 | ni | conserved hypothetical Ustilago-specific protein |
| 12d00037 | 0.43736 | ni | 5'-AMP-activated protein kinase, gamma subunit | um10535 | 0.34798 | K12863 | related to cell cycle control protein cwf15 |
| 19c00085 | 0.43754 | K14209 | amino acid transporters | um02774 | 0.34812 | ni | related to alternative oxidase precursor |
| 7c00164 | 0.43778 | ni | hypothetical protein | um10146 | 0.34822 | K14753 | probable CPC2 protein |
| 26d00002 | 0.43796 | K01209 | hypothetical protein | um03431 | 0.34831 | ni | conserved hypothetical protein |
| 26d00015 | 0.43818 | ni | hypothetical protein | um05101 | 0.34835 | K03163 | DNA topoisomerase I |
| 1c00037 | 0.43823 | K13509 | 1-acyl-sn-glycerol-3-phosphate acyltransferase | um10621 | 0.34838 | K02912 | probable 60S ribosomal protein L32 |
| 16d00059 | 0.43835 | ni | hypothetical protein | um05053 | 0.34846 | ni | conserved hypothetical protein |
| 12c00088 | 0.43836 | ni | hypothetical protein | um02405 | 0.34847 | ni | conserved hypothetical protein |
| 6c00047 | 0.43838 | K06693 | 26S proteasome regulatory complex, subunit PSMD9 | um01278 | 0.34862 | ni | conserved hypothetical protein |
| 10c00068 | 0.43842 | ni | transferrin receptor and related proteins | um03797 | 0.34876 | ni | hypothetical protein |
| 6d00061 | 0.43853 | ni | hypothetical protein | um10878 | 0.34885 | ni | conserved hypothetical protein |
| 25d00064 | 0.43854 | ni | hypothetical protein | um15092 | 0.34896 | K11227 | probable PBS2 - tyrosine protein kinase of the MAP kinase kinase family |
| 24c00001 | 0.43874 | ni | non-ribosomal peptide synthetase | um01235 | 0.34921 | ni | conserved hypothetical Ustilago-specific protein |
| 27d00094 | 0.4388 | ni | hypothetical protein | um03170 | 0.34943 | K13181 | related to DRS1 - RNA helicase of the DEAD box family |
| 15d00075 | 0.43884 | ni | hypothetical protein | um03877 | 0.34943 | ni | hypothetical protein |
| 14c00126 | 0.43888 | ni | inner membrane protein required for assembly of the F0 sector of | um03437 | 0.34967 | K11684 | related to BDF1 - sporulation protein |
| 22d00141 | 0.43894 | ni | hypothetical protein | um00612 | 0.34972 | ni | conserved hypothetical protein |
| 15d00040 | 0.43924 | K01641 | hydroxymethylglutaryl-coa synthase | um00602 | 0.34985 | K08286 | probable SCH9 - serine |
| 26d00054 | 0.43957 | ni | hypothetical protein | um05228 | 0.34997 | K10839 | related to RAD23 - nucleotide excision repair protein |
| 9d00097 | 0.43999 | ni | hypothetical protein | um10901 | 0.35009 | ni | probable polyubiquitin |
| 22c00262 | 0.44008 | ni | hypothetical protein | um06197 | 0.35017 | ni | putative protein |
| 24d00063 | 0.4401 | ni | mitochondrial Fe2+ transporter MMT1 and related transporters | um10792 | 0.35029 | K01611 | related to S-adenosylmethionine decarboxylase |
| 6c00049 | 0.44029 | ni | hypothetical protein | um05061 | 0.35043 | ni | conserved hypothetical protein |
| 11c00085 | 0.44031 | K00700 | 1,4-alpha-glucan branching enzyme/starch branching enzyme II | um10558 | 0.35044 | ni | probable tubulin beta chain |
| 8d00113 | 0.44049 | ni | FOG, Zn-finger | um00668 | 0.35044 | ni | related to SIP2 - subunit of the Snf1 serine |
| 3d00044 | 0.44069 | ni | TPR repeat-containing protein | um04465 | 0.35049 | K11359 | related to transcription regulator SPT7 |
| 22d00287 | 0.44092 | ni | pterin carbinolamine dehydratase PCBD | um02159 | 0.35054 | K15015 | related to amino acid transport protein |
| 16c00090 | 0.44092 | ni | hypothetical protein | um15072 | 0.35078 | ni | related to SUR1 - required for mannosylation of sphingolipids |
| 4c00002 | 0.44094 | K03144 | RNA polymerase II transcription initiation/nucleotide excision repa | um05862 | 0.3509 | K06053 | conserved hypothetical protein |
| 5d00080 | 0.44147 | ni | spliceosome subunit | um02320 | 0.35095 | ni | conserved hypothetical protein |
| 11c00001 | 0.44174 | ni | multidrug resistance-associated protein | um12019 | 0.35117 | K12879 | related to RLR1 - Subunit of the THO complex |
| 15d00022 | 0.44187 | ni | hypothetical protein | um04936 | 0.35117 | ni | conserved hypothetical protein |
| 12c00017 | 0.44198 | ni | membrane coat complex Retromer, subunit VPS5/SNX1 | um02395 | 0.35117 | ni | related to ferric reductase |
| 9c00389 | 0.44208 | K14789 | FOG, RRM domain | um11709 | 0.35126 | ni | hypothetical protein |
| 20c00007 | 0.44212 | K14692 | putative Zn2+ transporter MSC2 | um11268 | 0.35129 | K00528 | related to NADPH |
| 12d00131 | 0.44229 | ni | hypothetical protein | um04174 | 0.35136 | ni | conserved hypothetical protein |
| 16c00014 | 0.44233 | K02731 | 20S proteasome, regulatory subunit alpha type PSMA7/PRE6 | um06158 | 0.35142 | ni | probable glutaminase A |
| 9c00080 | 0.44242 | ni | arylalkylamine N-acetyltransferase | um02794 | 0.35144 | K06693 | related to 26S proteasome non-ATPase regulatory subunit 9 |
| 12d00098 | 0.44243 | K03950 | NADH:ubiquinone oxidoreductase, NDUFA6/B14 subunit | um11525 | 0.35152 | K04382 | protein phosphatase 2A |
| 22d00281 | 0.44244 | ni | hypothetical protein | um11388 | 0.3516 | ni | conserved hypothetical protein |
| 6c00127 | 0.44247 | ni | predicted gamma-butyrobetaine,2-oxoglutarate dioxygenase | um06148 | 0.35176 | ni | conserved hypothetical protein |
| 11d00086 | 0.44277 | ni | alcohol dehydrogenase | um02305 | 0.35187 | ni | related to peptidyl-prolyl cis-trans isomerase |
| 8c00083 | 0.44288 | K17263 | TATA-binding protein-interacting protein | um10911 | 0.35191 | K10661 | conserved hypothetical protein |
| 7d00353 | 0.44292 | K13108 | transcriptional regulator SNIP1 | um04593 | 0.35194 | ni | conserved hypothetical protein |
| 9c00107 | 0.44292 | K03249 | translation initiation factor 3, subunit f | um10142 | 0.35197 | K14397 | probable CF25 - polyadenylation factor |
| 19c00067 | 0.44293 | ni | predicted N6-adenine RNA methylase | um06175 | 0.35212 | K09540 | related to SEC63 - ER protein-translocation complex subunit |
| 14c00078 | 0.44307 | K02969 | 40S ribosomal protein S20 | um04242 | 0.35221 | ni | conserved hypothetical protein |
| 22d00243 | 0.44337 | ni | hypothetical protein | um05927 | 0.3523 | ni | conserved hypothetical Ustilago-specific protein |
| 10d00045 | 0.44343 | K12236 | transcription factor NF-X1 | um03562 | 0.35235 | ni | hypothetical protein |
| 7c00110 | 0.4435 | ni | hypothetical protein | um11672 | 0.35245 | K05955 | related to Protein farnesyltransferase alpha subunit |
| 8d00032 | 0.44378 | ni | synaptic vesicle transporter SVOP and related transporters | um01262 | 0.35246 | K02330 | DNA polymerase X - putative |
| 19c00135 | 0.44388 | K10579 | ubiquitin-protein ligase | um03565 | 0.35248 | K14575 | related to AFG2 - ATPase of the CDC48 |
| 9c00181 | 0.4439 | ni | magnesium-dependent phosphatase | um03738 | 0.35255 | K12854 | probable ATP dependent RNA helicase |
| 15c00024 | 0.44414 | K14831 | RNA-binding nuclear protein | um11064 | 0.35271 | ni | conserved hypothetical protein |
| 6c00014 | 0.44432 | ni | predicted transporter | um10939 | 0.35277 | ni | putative protein |
| 9c00153 | 0.44478 | ni | hypothetical protein | um02135 | 0.35281 | ni | effector family protein Eff1-5 |
| 16d00016 | 0.44481 | ni | translational repressor Pumilio/PUF3 | um12042 | 0.35297 | ni | conserved hypothetical protein |
| 3d00102 | 0.44495 | ni | predicted transporter | um11370.2 | 0.35309 | ni | conserved hypothetical protein |
| 27d00078 | 0.44529 | ni | hypothetical protein | um11432 | 0.35315 | ni | ni |
| 5d00034 | 0.4456 | K09503 | molecular chaperone | um11426 | 0.35323 | K16185 | related to GTR1 - GTP-binding protein |
| 3d00058 | 0.44565 | ni | hypothetical protein | um10415 | 0.35323 | K09184 | related to hypercellular protein |
| 5c00068 | 0.44568 | K11097 | small nuclear ribonucleoprotein E | um01377 | 0.35326 | ni | hypothetical protein |
| 13c00010 | 0.4457 | ni | predicted transporter | um10202 | 0.35331 | ni | putative protein |
| 13c00094 | 0.44601 | ni | hypothetical protein | um06118 | 0.35331 | K01279 | related to Tripeptidyl-peptidase I precursor |
| 9c00006 | 0.44608 | ni | hypothetical protein | um10795 | 0.35333 | ni | hypothetical protein |
| 16c00049 | 0.44613 | K00831 | phosphoserine aminotransferase | um10212 | 0.35342 | ni | hypothetical protein |
| 7c00111 | 0.44613 | ni | hypothetical protein | um05629 | 0.35347 | ni | conserved hypothetical protein |
| 7d00001 | 0.44619 | ni | hypothetical protein | um11517 | 0.35373 | K03949 | conserved hypothetical protein |
| 7c00219 | 0.44629 | K11322 | polycomb enhancer protein, EPC | um06248 | 0.35379 | ni | hypothetical protein |
| 3d00010 | 0.44633 | K00859 | similar to bacterial dephospho-CoA kinase | um03039 | 0.3538 | ni | hypothetical protein |
| 5c00015 | 0.4464 | ni | hypothetical protein | um11699 | 0.35382 | ni | conserved hypothetical protein |
| 25c00017 | 0.44651 | K02867 | mitochondrial/chloroplast ribosomal protein L11 | um02919 | 0.35383 | K05543 | related to tRNA dihydrouridine synthase |
| 2d00064 | 0.44663 | ni | hypothetical protein | um10810 | 0.35386 | K03030 | probable RPN11 - 26S proteasome regulatory subunit |
| 20d00075 | 0.4467 | K00558 | hypothetical protein | um01286 | 0.35391 | ni | related to PTC5 - putative 2C protein phosphatase |
| 12c00097 | 0.44673 | K01530 | P-type ATPase | um11705 | 0.354 | K03126 | conserved hypothetical protein |
| 25c00016 | 0.44685 | ni | hypothetical protein | um00453 | 0.35407 | K00914 | related to phosphatidylinositol 3-kinase |

| | | | | | | | |
|---|---|---|---|---|---|---|---|
| 7c00068 | 0.44691 | ni | hypothetical protein | um04136 | 0.3541 | K08286 | related to serine |
| 7c00196 | 0.44692 | K17402 | hypothetical protein | um04668 | 0.3542 | K03593 | related to nucleotide-binding protein NBP35 |
| 9d00118 | 0.44704 | K12868 | cyclin D-interacting protein GCIP | um05987 | 0.35431 | ni | conserved hypothetical protein |
| 13c00050 | 0.44716 | K00953 | 3'-phosphoadenosine 5'-phosphosulfate sulfotransferase | um01619 | 0.35431 | K00111 | related to GUT2 - glycerol-3-phosphate dehydrogenase |
| 22c00279 | 0.44725 | ni | permease of the major facilitator superfamily | um03464 | 0.35434 | ni | conserved hypothetical protein |
| 5d00129 | 0.44734 | K06111 | exocyst complex subunit Sec8 | um02623 | 0.35441 | ni | conserved hypothetical protein |
| 7c00105 | 0.44735 | K14771 | predicted nucleolar protein | um11675 | 0.35454 | ni | conserved hypothetical protein |
| 14d00116 | 0.44742 | K06675 | structural maintenance of chromosome protein 4 | um00921 | 0.35463 | K14807 | related to DBP6 - ATP-dependent RNA helicase |
| 5d00043 | 0.44755 | ni | HMG-box transcription factor | um05025 | 0.35467 | K11805 | related to human and petunia an11 protein |
| 5c00126 | 0.44756 | K14016 | ubiquitin fusion-degradation protein | um01843 | 0.35472 | ni | probable acetyl-CoA C-acyltransferase precursor |
| 7c00092 | 0.44762 | K01176 | alpha-amylase | um05937 | 0.35477 | ni | Putative Cys |
| 3c00002 | 0.44765 | ni | hypothetical protein | um04236 | 0.35488 | K00231 | conserved hypothetical protein |
| 11c00051 | 0.44767 | K09500 | chaperonin complex component, TCP-1 theta subunit | um10713.2 | 0.35514 | ni | conserved hypothetical protein |
| 13c00091 | 0.4477 | ni | negative regulator of COPII vesicle formation | um11135 | 0.35519 | K02978 | probable 40S ribosomal protein S27 |
| 9d00072 | 0.44771 | ni | predicted phosphoglucosamine acetyltransferase | um11200 | 0.35527 | ni | conserved hypothetical protein |
| 2d00017 | 0.44774 | ni | hypothetical protein | um12314 | 0.35531 | ni | putative protein |
| 14c00052 | 0.44781 | K06666 | WD40 repeat-containing protein | um05376 | 0.35535 | ni | putative protein |
| 10d00116 | 0.44831 | ni | FOG, RCC1 domain | um01117 | 0.35558 | ni | conserved hypothetical protein |
| 16d00085 | 0.44836 | K00135 | aldehyde dehydrogenase | um04371 | 0.35563 | K15026 | conserved hypothetical protein |
| 2d00005 | 0.44845 | K03238 | translation initiation factor 2, beta subunit | um02804 | 0.35577 | ni | conserved hypothetical protein |
| 12d00119 | 0.44847 | K09602 | uncharacterized conserved protein | um04901 | 0.35581 | ni | related to CKI1 - choline kinase |
| 15c00060 | 0.44862 | ni | hypothetical protein | um10179 | 0.35593 | ni | conserved hypothetical protein |
| 7c00244 | 0.44868 | ni | transcription factor of the Forkhead/HNF3 family | um11410 | 0.35596 | K12765 | cdk-related kinase 1 |
| 27c00065 | 0.44872 | K04705 | signal transducing adaptor protein STAM/STAM2 | um12292 | 0.356 | ni | hypothetical protein |
| 22d00228 | 0.44877 | ni | hypothetical protein | um04925 | 0.3563 | K00916 | related to CTK1 - carboxy-terminal domain |
| 3d00079 | 0.44887 | K10742 | DNA replication helicase | um06080 | 0.35667 | K00948 | related to ribose-phosphate diphosphokinase catalytic chain I |
| 9c00067 | 0.449 | K06978 | hypothetical protein | um11840 | 0.35674 | K14998 | related to Surfeit locus protein 1 |
| 9c00257 | 0.44906 | K00601 | 5'-phosphoribosylglycinamide formyltransferase | um11379 | 0.35675 | ni | putative protein |
| 15d00053 | 0.4492 | K01834 | phosphoglycerate mutase | um11429 | 0.35686 | ni | conserved hypothetical protein |
| 8c00081 | 0.44921 | K05012 | cl- channel CLC-3 and related proteins | um02178 | 0.35694 | ni | related to aspartic protease |
| 6d00053 | 0.44929 | ni | Na+/H+ antiporter | um03337 | 0.35698 | K17439 | related to MRPL35 - mitochondrial ribosomal protein |
| 26d00062 | 0.44941 | ni | hypothetical protein | um11695 | 0.3571 | K01735 | related to 2-epi-5-epi-valiolone synthase |
| 27d00045 | 0.44947 | ni | thioredoxin-like protein | um04934 | 0.35729 | K14793 | related to RRP9 - protein associated with the U3 small nucleolar RNA |
| 13d00060 | 0.44956 | ni | cytoplasmic Zn-finger protein BRAP2 | um01150 | 0.3574 | ni | related to COX7 - cytochrome-c oxidase |
| 22c00008 | 0.44959 | ni | para-hydroxybenzoate-polyprenyl transferase | um10302.2 | 0.35794 | ni | conserved hypothetical protein |
| 7d00035 | 0.44963 | K05909 | multicopper oxidases | um01408 | 0.35797 | ni | probable MCA1 - Metacaspase |
| 9c00326 | 0.44979 | K05663 | mitochondrial Fe/S cluster exporter | um05569 | 0.35806 | K07443 | related to methylated-dna--protein-cysteine methyltransferase |
| 19c00054 | 0.44989 | ni | thioredoxin-like protein | um12159 | 0.35818 | ni | conserved hypothetical protein |
| 19d00092 | 0.44998 | ni | hypothetical protein | um11999 | 0.35843 | K10752 | probable Chromatin assembly factor 1 subunit c |
| 24c00024 | 0.45001 | K06639 | protein tyrosine phosphatase CDC14 | um00318 | 0.35862 | K14815 | related to MRT4 - protein involved in mRNA turnover |
| 14d00076 | 0.45002 | ni | voltage-gated shaker-like K+ channel, subunit beta/KCNAB | um04316 | 0.35871 | ni | hypothetical protein |
| 5c00158 | 0.45035 | ni | hypothetical protein | um05767 | 0.35883 | ni | related to ATP-dependent RNA helicase |
| 16c00009 | 0.45035 | ni | hypothetical protein | um10459 | 0.35889 | K03063 | probable RPT3 - 26S proteasome regulatory subunit |
| 13c00029 | 0.45053 | K14838 | nucleolar RNA-binding protein NIFK | um01768 | 0.35923 | K01227 | related to Endo-b-N-acetylglucosaminidase |
| 11c00013 | 0.45073 | ni | hypothetical protein | um01734 | 0.35925 | ni | hypothetical protein |
| 20c00070 | 0.45076 | K02503 | zinc-binding protein of the histidine triad | um04892 | 0.35933 | ni | hypothetical protein |
| 5c00070 | 0.45078 | ni | hypothetical protein | um04217 | 0.35942 | ni | conserved hypothetical protein |
| 22c00002 | 0.45089 | K10426 | dynactin, subunit p62 | um12188 | 0.35945 | ni | hypothetical protein |
| 26d00014 | 0.45112 | ni | hypothetical protein | um04970 | 0.35949 | K08869 | conserved hypothetical protein |
| 20d00070 | 0.45137 | K05658 | multidrug/pheromone exporter | um05064 | 0.35955 | K02209 | probable CDC46 - cell division control protein |
| 7c00060 | 0.45147 | ni | glutathione S-transferase | um02462 | 0.35991 | ni | conserved hypothetical protein |
| 13c00012 | 0.45152 | ni | predicted protein tyrosine phosphatase | um11350.2 | 0.35996 | K01908 | related to acetyl coenzyme A synthetase |
| 15d00013 | 0.45158 | K09527 | molecular chaperone | um02353 | 0.36013 | K02997 | probable 40S ribosomal protein S9 |
| 13c00111 | 0.45158 | K02868 | 60S ribosomal protein L11 | um11990 | 0.36016 | ni | putative protein |
| 15d00060 | 0.45159 | ni | hypothetical protein | um05548 | 0.36019 | ni | related to L-ascorbate oxidase precursor |
| 26d00086 | 0.45165 | K15326 | predicted tRNA-splicing endonuclease subunit | um02523 | 0.36035 | ni | related to Endoglucanase 1 precursor |
| 8d00084 | 0.45167 | K05288 | glycosylphosphatidylinositol anchor synthesis protein | um03946 | 0.3605 | ni | putative protein |
| 22d00294 | 0.45196 | K00876 | armadillo | um00153 | 0.36118 | ni | conserved hypothetical protein |
| 26c00044 | 0.45198 | ni | hypothetical protein | um10658 | 0.36125 | ni | related to component of the spindle assembly checkpoint dma1 |
| 7d00239 | 0.45206 | ni | hypothetical protein | um10842 | 0.36126 | K02866 | probable RPL10 - 60S large subunit ribosomal protein L10 |
| 27d00086 | 0.45214 | ni | CDC42 Rho GTPase-activating protein | um05642 | 0.36132 | ni | probable SNQ2 - ABC transporter involved in multidrug resistance |
| 19c00148 | 0.45215 | K01969 | 3-Methylcrotonyl-CoA carboxylase, non-biotin containing subunit | um01935 | 0.36158 | K05607 | probable methylglutaconyl-coa hydratase |
| 5c00130 | 0.45224 | K15202 | RNA polymerase III transcription factor (TF)IIIC subunit | um10918 | 0.36166 | ni | Brh2-interacting protein Dss1 |
| 15c00096 | 0.45245 | ni | hypothetical protein | um03265 | 0.36169 | ni | putative protein |
| 22c00239 | 0.45246 | ni | hypothetical protein | um10582.2 | 0.36187 | K06889 | conserved hypothetical protein |
| 22c00156 | 0.45248 | ni | hypothetical protein | um01310 | 0.36204 | K14766 | related to NOP14 - nuclear and nucleolar protein with possible role in ribosome biogenesis |
| 15d00068 | 0.45277 | K12198 | protein involved in vacuolar protein sorting | um02575 | 0.36216 | ni | related to toxD gene |
| 15c00093 | 0.45282 | ni | hypothetical protein | um00193 | 0.36224 | ni | conserved hypothetical protein |
| 13d00034 | 0.45286 | ni | HSP90 co-chaperone p23 | um10492 | 0.36229 | ni | hypothetical protein |
| 9c00103 | 0.4529 | ni | hypothetical protein | um02968 | 0.36243 | K12620 | related to LSM1 - Sm-like |
| 19c00065 | 0.45333 | ni | hypothetical protein | um05169 | 0.36252 | ni | conserved hypothetical protein |
| 11d00083 | 0.45336 | ni | hypothetical protein | um06212 | 0.36254 | K15161 | related to SSN8 - DNA-directed RNA polymerase II holoenzyme and SRB subcomplex subunit |
| 24d00040 | 0.45342 | K02938 | 60s ribosomal protein L2/L8 | um10325 | 0.36256 | ni | putative protein |
| 27d00072 | 0.45344 | K02877 | 60s ribosomal protein L15 | um06329 | 0.36269 | K00721 | dolichyl-phosphate beta-D-mannosyltransferase |
| 3d00038 | 0.4535 | ni | hypothetical protein | um03627 | 0.36272 | K07152 | probable SCO1 - involved in stabilization of Cox1p and Cox2p |
| 11d00058 | 0.45362 | K02967 | mitochondrial/chloroplast ribosomal protein S2 | um03708.2 | 0.36286 | ni | related to gata transcription factor |
| 7c00037 | 0.45395 | K12862 | pleiotropic regulator 1 | um00242 | 0.36315 | K14805 | related to MAK5 - ATP-dependent RNA helicase |
| 22d00254 | 0.45403 | ni | acyl-coa synthetase | um00061 | 0.36323 | ni | related to quinate transport protein |
| 7c00295 | 0.45419 | K07442 | tRNA:1-methyladenosine methyltransferase, subunit GCD14 | um02840 | 0.36325 | ni | related to CHS5 - chitin biosynthesis protein |
| 13d00051 | 0.45438 | K15292 | vesicle trafficking protein Sec1 | um04226 | 0.3635 | K11338 | probable RVB2 - RUVB-like protein |
| 24d00055 | 0.45449 | ni | hypothetical protein | um02003 | 0.36351 | ni | hypothetical protein |
| 13c00068 | 0.45451 | ni | hypothetical protein | um02310 | 0.36368 | ni | putative protein |
| 22c00070 | 0.45473 | ni | hypothetical protein | um04310 | 0.36399 | K12736 | related to Peptidylprolyl isomerase domain and WD repeat-containing protein 1 |
| 7c00283 | 0.45473 | ni | hypothetical protein | um05783 | 0.36418 | K15272 | related to UDP-galactose transporter |
| 9c00010 | 0.45474 | ni | acyl-coa synthetase | um11196 | 0.36422 | K10747 | probable CDC9 - DNA ligase I |
| 22c00128 | 0.45482 | K14284 | mRNA export factor TAP/MEX67 | um00256 | 0.36423 | ni | related to 3-oxoacyl- |
| 25c00065 | 0.45532 | ni | hypothetical protein | um10774 | 0.3644 | ni | conserved hypothetical Ustilago-specific protein |
| 12d00054 | 0.45536 | ni | hypothetical protein | um06183 | 0.36441 | ni | related to RNA-binding protein rnp24 |
| 6c00072 | 0.45547 | K02295 | deoxyribodipyrimidine photolyase | um02192 | 0.36476 | ni | hypothetical protein |
| 12d00078 | 0.45571 | ni | hypothetical protein | um06398 | 0.36478 | ni | related to calcineurin temperature suppressor cts1 |
| 22c00133 | 0.45579 | ni | proteins containing armadillo/beta-catenin-like repeat | um03494 | 0.36479 | K13126 | mRNA polyadenylate-binding protein PAB1 |
| 18c00055 | 0.45583 | ni | hypothetical protein | um04820 | 0.36485 | ni | related to ITC1 - subunit of Isw2 chromatin remodelling complex |
| 13c00061 | 0.45604 | ni | hypothetical protein | um02814 | 0.365 | ni | hypothetical protein |
| 13d00107 | 0.45604 | ni | GATA-4/5/6 transcription factors | um03533 | 0.36537 | K02894 | probable RPL23A - 60S large subunit ribosomal protein L23 |
| 6c00045 | 0.45606 | ni | methyltransferase | um03748 | 0.3655 | ni | conserved hypothetical Ustilago-specific protein |
| 5d00144 | 0.45622 | ni | amidases | um11198 | 0.36577 | K11126 | conserved hypothetical protein |
| 19c00031 | 0.45626 | ni | predicted mRNA cap-binding protein related to eIF-4E | um04032 | 0.36586 | ni | probable beta-glucosidase |
| 20d00014 | 0.45635 | ni | chitin synthase/hyaluronan synthase | um12195 | 0.36592 | ni | conserved hypothetical protein |
| 10d00089 | 0.45681 | K13115 | C2C2-type Zn-finger protein | um10296 | 0.36592 | K02267 | probable COX12 - cytochrome-c oxidase |
| 2d00054 | 0.45701 | ni | late Golgi protein sorting complex, subunit Vps53 | um06393 | 0.36612 | K17279 | related to YOP1 - Ypt-interacting protein |
| 11c00047 | 0.45716 | ni | hypothetical protein | um12340 | 0.36616 | ni | related to C-4 methylsterol oxidase |
| 9d00148 | 0.45719 | K12825 | splicing factor 3a, subunit 1 | um11754 | 0.36642 | ni | putative protein |
| 9d00006 | 0.45726 | ni | molecular chaperone | um10135 | 0.3666 | K10768 | conserved hypothetical protein |
| 14c00099 | 0.45746 | ni | hypothetical protein | um00534 | 0.36665 | ni | conserved hypothetical protein |
| 27d00076 | 0.45762 | ni | hypothetical protein | um01456 | 0.36667 | ni | ni |
| 12d00060 | 0.45777 | K11883 | predicted RNA-binding protein Nob1p involved in 26S proteasom | um12342 | 0.36708 | ni | conserved hypothetical protein |
| 8d00102 | 0.45779 | ni | hypothetical protein | um00280 | 0.36738 | ni | related to Glutaminyl-peptide cyclotransferase precursor |
| 9c00230 | 0.45781 | ni | aldo/keto reductase family proteins | um05505 | 0.3674 | K15153 | related to mediator complex subunit soh1 |
| 24d00008 | 0.45803 | ni | hypothetical protein | um05404.2 | 0.36762 | ni | related to conserved oligomeric Golgi complex component 3 |
| 4c00043 | 0.45813 | K07124 | 17 beta-hydroxysteroid dehydrogenase type 3 | um11822 | 0.36781 | K02871 | probable MRPL23 - mitochondrial ribosomal protein |
| 12c00044 | 0.45814 | ni | predicted methyltransferase | um00588 | 0.36787 | ni | hypothetical protein |
| 15c00084 | 0.45819 | K11495 | histones H3 and H4 | um11328 | 0.36796 | ni | conserved hypothetical protein |
| 1c00029 | 0.45839 | ni | arp2/3 complex-interacting protein VIP1/Asp1 | um10702 | 0.368 | K02940 | probable RPL9A - ribosomal protein L9 |
| 11d00008 | 0.45867 | ni | hypothetical protein | um04485 | 0.3681 | ni | related to Cut9 interacting protein scn1 |
| 19d00008 | 0.45879 | ni | hypothetical protein | um11729 | 0.36829 | ni | putative protein |
| 5d00121 | 0.4589 | K00698 | chitin synthase/hyaluronan synthase | um11912 | 0.36838 | K08827 | related to mRNA splicing-associated serine-threonine protein kinase |
| 10d00002 | 0.45909 | K14557 | HAT (Half-A-TPR) repeat-containing protein | um11725 | 0.36859 | K14863 | conserved hypothetical protein |
| 24d00049 | 0.45938 | ni | hypothetical protein | um01361 | 0.36862 | ni | hypothetical protein |
| 27d00053 | 0.4594 | ni | hypothetical protein | um00575 | 0.36871 | K11675 | related to IES1 - Subunit 1 of the INO80 chromatin remodeling complex |
| 3c00023 | 0.45955 | ni | hypothetical protein | um03216 | 0.36891 | K07203 | probable TOR1 - 1-phosphatidylinositol 3-kinase |
| 13d00105 | 0.45959 | ni | vacuolar assembly/sorting protein VPS16 | um11782 | 0.36892 | ni | putative protein |
| 9c00049 | 0.45982 | ni | hypothetical protein | um01094 | 0.36915 | K03115 | related to CKB1 - casein kinase II |
| 5d00143 | 0.45982 | K02921 | 60S ribosomal protein L37 | um01031 | 0.36916 | ni | conserved hypothetical protein |
| 1d00005 | 0.45983 | K14818 | angio-associated migratory cell protein | um04624 | 0.36918 | K14304 | conserved hypothetical protein |
| 4d00044 | 0.45983 | ni | hypothetical protein | um06295 | 0.36921 | ni | related to dolichyl-phosphate-mannose-glycolipid alpha-mannosyltransferase |
| 10d00064 | 0.46001 | K10300 | 3'-5' DNA helicase | um01175 | 0.36922 | K03514 | related to TRF4 - topoisomerase I-related protein |
| 20d00053 | 0.46004 | K01265 | metallopeptidase | um01123 | 0.3693 | K11373 | related to IKI3 - Subunit of RNA polymerase II elongator histone acetyltransferase complex |
| 3d00094 | 0.46012 | ni | hypothetical protein | um15022.2 | 0.36931 | K12893 | related to pre-mrna splicing factor srp55 |
| 27d00056 | 0.46033 | ni | leucine permease transcriptional regulator | um02217 | 0.36933 | ni | related to glutamate carboxypeptidase II |
| 11c00075 | 0.46033 | K12623 | small nuclear ribonucleoprotein | um03001 | 0.36945 | ni | putative protein |
| 26d00049 | 0.46048 | K03124 | transcription initiation factor TFIIB | um11715 | 0.36948 | ni | related to VPS13 - involved in regulating membrane traffic |
| 2c00048 | 0.46051 | K17086 | endosomal membrane proteins, EMP70 | um04995 | 0.36951 | ni | conserved hypothetical protein |
| 27c00015 | 0.46057 | ni | uncharacterized conserved protein | um15050 | 0.36963 | K08332 | probable VAC8 - vacuolar membrane protein |
| 25d00078 | 0.46058 | K06628 | CDC45 (cell division cycle 45)-like protein | um10076 | 0.36973 | ni | hypothetical protein |
| 22c00181 | 0.46068 | ni | hypothetical protein | um03970 | 0.36977 | ni | putative protein |
| 1d00019 | 0.4607 | K02210 | DNA replication licensing factor, MCM7 component | um10154 | 0.3699 | K01885 | related to glutamyl-tRNA synthetase |
| 10c00093 | 0.46075 | K00128 | aldehyde dehydrogenase | um00639 | 0.36992 | K14303 | related to nuclear pore complex protein Nup160 |
| 19d00039 | 0.46105 | K00698 | chitin synthase/hyaluronan synthase | um00390 | 0.37029 | K11514 | conserved hypothetical protein |
| 26d00027 | 0.46124 | K02940 | 60S ribosomal protein L9 | um12055 | 0.37038 | K03872 | related to ELC1 - Elongin C transcription elongation factor |
| 9c00166 | 0.46132 | K04393 | ras-related small GTPase | um02301 | 0.37051 | ni | related to C2H2-type zinc finger protein |
| 7d00223 | 0.46136 | ni | hypothetical protein | um04205 | 0.37068 | ni | hypothetical protein |
| 27d00063 | 0.46169 | ni | hypothetical protein | um01001 | 0.37069 | K10685 | related to ubiquitin-like protein activating enzyme |
| 13d00005 | 0.46183 | K11086 | U1 snRNP component | um00258 | 0.37075 | ni | conserved hypothetical protein |
| 7d00045 | 0.462 | ni | 1-Acyl dihydroxyacetone phosphate reductase and related dehydr | um03767 | 0.37082 | ni | conserved hypothetical protein |
| 9d00265 | 0.46211 | ni | hypothetical protein | um03251 | 0.37096 | ni | putative protein |
| 2c00073 | 0.46225 | ni | beta-glucocerebrosidase | um04022 | 0.37107 | ni | ni |
| 7d00085 | 0.46226 | ni | hypothetical protein | um05097 | 0.37114 | ni | related to secreted aspartic protease 2 |
| 9d00312 | 0.4627 | K01293 | aminoacylase ACY1 and related metalloexopeptidases | um02860 | 0.37146 | ni | probable PHO81 - cyclin-dependent kinase inhibitor |
| 2c00005 | 0.4627 | ni | hypothetical protein | um11150 | 0.3715 | ni | related to Uracil permease |
| 7c00306 | 0.46278 | K14635 | DEAD-box like helicase | um15036 | 0.37153 | ni | conserved hypothetical protein |
| 9d00191 | 0.46293 | K06101 | putative transcription factor ASH1/LIN-59 | um10251 | 0.37159 | ni | conserved hypothetical protein |
| 9d00047 | 0.46302 | ni | hypothetical protein | um04495 | 0.37161 | ni | related to COQ4 - responsible for restoring ubiquinone biosynthesis in coq4 mutant |
| 18c00038 | 0.46305 | ni | hypothetical protein | um11685 | 0.37167 | ni | conserved hypothetical protein |
| 26d00098 | 0.46309 | ni | alcohol dehydrogenase | um11212 | 0.37177 | K00419 | probable QCR9 - ubiquinol--cytochrome-c reductase subunit 9 |
| 4c00007 | 0.46317 | ni | membrane coat complex Retromer, subunit VPS26 | um03647 | 0.37185 | ni | probable SCS7 - required for hydroxylation of ceramide |
| 10c00060 | 0.46319 | K16944 | septin family protein | um00387 | 0.37202 | K07937 | probable ADP-ribosylation factor |

| | | | | | | | |
|---|---|---|---|---|---|---|---|
| 14d00077 | 0.46329 | ni | cyclophilin-type peptidyl-prolyl cis-trans isomerase | um00057 | 0.37225 | ni | conserved hypothetical protein |
| 16c00089 | 0.46369 | ni | uncharacterized conserved protein | um12206 | 0.3724 | ni | conserved hypothetical protein |
| 15c00004 | 0.46371 | ni | uncharacterized conserved protein | um03718 | 0.37246 | ni | ni |
| 27d00035 | 0.46386 | ni | predicted transporter | um04591 | 0.3726 | K12571 | related to PAN2 - component of Pab1p-stimulated poly |
| 18d00099 | 0.46398 | ni | hypothetical protein | um03411 | 0.37261 | K01181 | probable endo-1 |
| 9c00130 | 0.46405 | K15542 | polyadenylation factor I complex, subunit PFS2 | um01539 | 0.37263 | ni | hypothetical protein |
| 3c00004 | 0.46418 | ni | hypothetical protein | um02453 | 0.3728 | K04564 | probable SOD2 - superoxide dismutase |
| 13c00045 | 0.46425 | K03964 | hypothetical protein | um00539 | 0.37282 | ni | conserved hypothetical protein |
| 6d00033 | 0.46443 | ni | hypothetical protein | um01372 | 0.37282 | K14538 | related to nuclear GTPase |
| 22d00096 | 0.46448 | K02902 | mitochondrial/chloroplast ribosomal protein L2 | um05833 | 0.37291 | K16576 | conserved hypothetical protein |
| 9c00302 | 0.46456 | ni | uncharacterized conserved protein | um00337 | 0.37312 | ni | hypothetical protein |
| 22d00274 | 0.46457 | ni | predicted N6-DNA-methyltransferase | um03641 | 0.37344 | ni | ni |
| 10d00076 | 0.46471 | K01251 | S-adenosylhomocysteine hydrolase | um06154 | 0.37344 | ni | hypothetical protein |
| 7c00008 | 0.46473 | ni | hypothetical protein | um03863 | 0.37344 | K03006 | probable RPO21 - DNA-directed RNA polymerase II |
| 9d00008 | 0.46484 | K14826 | FKBP-type peptidyl-prolyl cis-trans isomerase | um06384 | 0.37355 | ni | hypothetical protein |
| 20c00049 | 0.46485 | K07170 | hypothetical protein | um05725 | 0.3736 | K00550 | probable OPI3 - methylene-fatty-acyl-phospholipid synthase |
| 2c00064 | 0.46486 | K03844 | glycosyltransferase | um11469 | 0.37406 | K07952 | related to ARL3 - ADP-ribosylation factor-like protein |
| 7c00147 | 0.46509 | ni | uncharacterized conserved protein | um04779 | 0.37414 | ni | conserved hypothetical protein |
| 10d00087 | 0.46514 | ni | hypothetical protein | um10731 | 0.37416 | K13337 | related to PEX19 - required for biogenesis of peroxisomes |
| 11c00072 | 0.46516 | K05658 | multidrug/pheromone exporter | um11677.2 | 0.37438 | K12767 | related to serine |
| 9d00395 | 0.46516 | ni | hypothetical protein | um11521 | 0.37448 | K03575 | related to A |
| 7d00243 | 0.46539 | K08333 | rhogef GTPase | um02886 | 0.37456 | ni | putative protein |
| 24d00016 | 0.46548 | K08489 | SNARE protein TLG2 | um02083 | 0.37462 | K10875 | probable RAD54 - DNA-dependent ATPase of the Snf2p family |
| 9d00401 | 0.46553 | ni | acyl-coa synthetase | um05758 | 0.37469 | ni | related to HST4 - member of the Sir2p family of NAD |
| 7d00064 | 0.46558 | ni | hypothetical protein | um10938 | 0.37492 | ni | probable NADP-dependent mannitol dehydrogenase |
| 9d00044 | 0.4657 | K00761 | armadillo | um06068 | 0.37492 | ni | putative protein |
| 12d00022 | 0.46581 | K01090 | serine/threonine protein phosphatase | um11120 | 0.37494 | ni | conserved hypothetical protein |
| 9c00199 | 0.46598 | ni | uncharacterized conserved protein | um06458 | 0.37494 | ni | conserved hypothetical protein |
| 7c00104 | 0.46606 | ni | hypothetical protein | um01974 | 0.37499 | K15979 | conserved hypothetical protein |
| 18d00103 | 0.4661 | ni | hypothetical protein | um02552 | 0.375 | ni | conserved hypothetical protein |
| 9c00038 | 0.46617 | ni | hypothetical protein | um11028 | 0.37504 | K02946 | related to WHI2 - growth regulation protein |
| 7d00022 | 0.46628 | K10747 | ATP-dependent DNA ligase I | um00788 | 0.37513 | ni | conserved hypothetical protein |
| 27c00082 | 0.46636 | K10418 | dynein light chain type 1 | um10243 | 0.37522 | K11885 | related to DNA-damage inducible protein 2 |
| 9d00398 | 0.46642 | ni | hypothetical protein | um10269 | 0.37532 | ni | conserved hypothetical protein |
| 18c00082 | 0.46643 | ni | hypothetical protein | um01067 | 0.37536 | K12618 | probable KEM1 - 5 |
| 8c00099 | 0.4665 | ni | hypothetical protein | um15023 | 0.37537 | K02677 | probable protein kinase C |
| 9c00310 | 0.46705 | K03035 | 26S proteasome regulatory complex, subunit RPN5/PSMD12 | um03612 | 0.37555 | ni | conserved hypothetical protein |
| 22c00157 | 0.4671 | ni | hypothetical protein | um03874 | 0.37572 | ni | conserved hypothetical protein |
| 7d00315 | 0.4674 | ni | hypothetical protein | um02722 | 0.37585 | ni | hypothetical protein |
| 3c00076 | 0.46767 | ni | transcription factor of the Forkhead/HNF3 family | um01350 | 0.37589 | ni | related to SNF4 - nuclear regulatory protein |
| 12d00096 | 0.46775 | ni | hypothetical protein | um00391.2 | 0.376 | ni | conserved hypothetical protein |
| 18c00043 | 0.46783 | ni | hypothetical protein | um04523 | 0.37601 | K03320 | low affinity ammonium transporter |
| 12d00118 | 0.46786 | ni | COPII vesicle protein | um03227 | 0.3766 | K11600 | probable exosome complex exonuclease rrp41 |
| 22d00014 | 0.46792 | ni | hypothetical protein | um11589 | 0.37666 | K02880 | probable RPL17A - ribosomal protein L17 |
| 18d00051 | 0.46794 | ni | hypothetical protein | um10949 | 0.37695 | ni | conserved hypothetical protein |
| 9d00024 | 0.46804 | ni | hypothetical protein | um01296 | 0.37705 | K14571 | related to RIX7 - AAA-type ATPase required for biogenesis and nuclear export of 60S ribosomal subunits |
| 7d00013 | 0.46807 | ni | hypothetical protein | um04604 | 0.37741 | ni | conserved hypothetical protein |
| 22c00115 | 0.46809 | K12196 | AAA+-type ATPase | um12094 | 0.37751 | ni | conserved hypothetical protein |
| 7c00238 | 0.46825 | ni | uncharacterized conserved protein | um04201 | 0.37765 | K08900 | related to BCS1 - mitochondrial protein of the AAA family of ATPases |
| 19d00086 | 0.46837 | ni | hypothetical protein | um00176 | 0.37777 | ni | probable SAC1 - recessive suppressor of secretory defect |
| 8d00058 | 0.46839 | ni | hypothetical protein | um10485 | 0.37786 | ni | conserved hypothetical protein |
| 9d00026 | 0.46864 | K00344 | zinc-binding oxidoreductase | um01566 | 0.37796 | ni | hypothetical protein |
| 4d00027 | 0.46865 | ni | inositol polyphosphate 5-phosphatase and related proteins | um11960 | 0.37799 | ni | conserved hypothetical protein |
| 5d00101 | 0.46878 | ni | synaptic vesicle transporter SVOP and related transporters | um00422 | 0.37815 | ni | conserved hypothetical protein |
| 9c00058 | 0.46887 | ni | lysophospholipase | um05567 | 0.37836 | K08341 | probable ATG8 - essential for autophagy |
| 20d00015 | 0.46892 | ni | hypothetical protein | um11049.2 | 0.37841 | ni | related to Exoribonuclease II |
| 20c00058 | 0.46903 | ni | hismacro and SEC14 domain-containing proteins | um00439 | 0.37852 | ni | conserved hypothetical protein |
| 9c00309 | 0.46922 | ni | hypothetical protein | um06419 | 0.37866 | ni | conserved hypothetical protein |
| 18d00027 | 0.46932 | ni | hypothetical protein | um00357 | 0.37881 | ni | conserved hypothetical protein |
| 7c00178 | 0.46938 | ni | hypothetical protein | um11812 | 0.37891 | ni | related to Cytochrome P450 |
| 9c00014 | 0.46939 | K13617 | predicted acetyltransferases and hydrolases with the alpha/beta h | um02281 | 0.37894 | K12855 | probable pre-mRNA splicing factor prp1 |
| 9c00207 | 0.46954 | ni | hypothetical protein | um11151 | 0.37894 | K11713 | related to Type I protein geranylgeranyltransferase beta subunit |
| 13d00108 | 0.46968 | ni | predicted transporter | um02651 | 0.37903 | K04712 | related to dihydroceramide delta |
| 4c00038 | 0.46973 | ni | cytoskeleton-associated protein and related proteins | um05085 | 0.37937 | K10627 | related to postreplication repair protein uvsH |
| 6c00073 | 0.46981 | ni | amino acid transporters | um01535 | 0.37953 | ni | conserved hypothetical protein |
| 9c00069 | 0.47001 | K05909 | multicopper oxidases | um02510 | 0.37967 | K01213 | probable PGU1 - Endo-polygalacturonase |
| 22c00146 | 0.47002 | ni | hypothetical protein | um10045 | 0.38013 | ni | putative protein |
| 6c00081 | 0.4701 | ni | queuine-trna ribosyltransferase | um11530 | 0.38016 | ni | putative protein |
| 2c00068 | 0.47018 | ni | Ca2+/calmodulin-dependent protein kinase | um15027 | 0.38071 | K10771 | related to exodeoxyribonuclease |
| 1d00034 | 0.47025 | ni | Na+/K+ ATPase, alpha subunit | um02499 | 0.38117 | K14003 | conserved hypothetical protein |
| 9c00277 | 0.47056 | ni | hypothetical protein | um11913 | 0.38118 | ni | related to PRP43 - spliceosomal RNA helicase |
| 5d00004 | 0.47064 | K01697 | cystathionine beta-synthase and related enzymes | um10075 | 0.38121 | K12161 | related to ubiquitin related modifier URM1 |
| 22d00052 | 0.47066 | ni | hypothetical protein | um05964 | 0.38128 | ni | related to esterase |
| 14c00028 | 0.47071 | K16261 | amino acid transporters | um04679 | 0.38158 | K12831 | related to spliceosome-associated protein SAP-49 |
| 13c00104 | 0.47072 | K03353 | anaphase-promoting complex (APC), Cdc16 subunit | um01950 | 0.38168 | K15532 | conserved hypothetical protein |
| 6d00067 | 0.47094 | ni | hypothetical protein | um05007 | 0.38168 | ni | conserved hypothetical protein |
| 15d00032 | 0.47112 | ni | hypothetical protein | um05518 | 0.38185 | ni | hypothetical protein |
| 7d00087 | 0.47116 | ni | hypothetical protein | um05771 | 0.38193 | ni | related to aimless RasGEF |
| 20c00071 | 0.47123 | ni | predicted membrane protein | um00937 | 0.38212 | ni | putative protein |
| 22c00060 | 0.47128 | ni | hypothetical protein | um11905 | 0.38214 | K13100 | probable Pre-mRNA splicing factor cwc22 |
| 9d00123 | 0.47151 | ni | hypothetical protein | um02469 | 0.38227 | ni | related to pre-mRNA splicing factor |
| 3d00019 | 0.47178 | K13754 | K+-dependent Na+, Ca2+ antiporter | um05173 | 0.38252 | K09523 | related to DnaJ homolog subfamily C member 3 |
| 19d00119 | 0.47185 | ni | hypothetical protein | um01438 | 0.38253 | ni | conserved hypothetical protein |
| 16d00032 | 0.47197 | K17427 | mitochondrial ribosomal protein L17 | um01739 | 0.38278 | ni | putative protein |
| 14c00063 | 0.47204 | ni | hypothetical protein | um00991 | 0.38303 | ni | putative protein |
| 6d00013 | 0.47205 | ni | hypothetical protein | um12076 | 0.38314 | ni | telomere-associated recQ-like helicase usher |
| 22d00030 | 0.47236 | ni | hypothetical protein | um00574 | 0.38332 | ni | probable DNA |
| 9d00201 | 0.47249 | K04464 | mitogen-activated protein kinase | um03774 | 0.38332 | ni | probable positive effector protein GCN20 |
| 6d00085 | 0.47301 | ni | hypothetical protein | um05394 | 0.38335 | ni | putative protein |
| 9d00350 | 0.47305 | ni | hypothetical protein | um02346 | 0.38346 | K14787 | probable RNA-binding protein |
| 10c00020 | 0.47314 | ni | vacuolar assembly/sorting protein PEP5/VPS11 | um04926 | 0.38356 | K01381 | probable PEP4 - aspartyl protease |
| 2d00057 | 0.47323 | ni | hypothetical protein | um02311 | 0.38358 | ni | conserved hypothetical protein |
| 14d00044 | 0.47353 | ni | DNA repair protein RAD51/RHP55 | um01753 | 0.3836 | ni | probable GCY1 - galactose-induced protein of aldo |
| 3d00034 | 0.47364 | K02976 | 40s ribosomal protein S26 | um05302 | 0.38388 | ni | hypothetical protein |
| 12d00009 | 0.4739 | K14307 | hypothetical protein | um12010 | 0.38413 | ni | putative protein |
| 9d00394 | 0.47405 | ni | mitochondrial/chloroplast ribosomal protein L17 | um01442 | 0.3843 | ni | conserved hypothetical protein |
| 5c00019 | 0.47406 | ni | uncharacterized conserved protein | um02526 | 0.38494 | K03537 | conserved hypothetical protein |
| 10c00064 | 0.47416 | ni | FOG, Ankyrin repeat | um05030 | 0.38495 | K17081 | probable PHB2 - prohibitin |
| 18c00049 | 0.47419 | K10773 | endonuclease III | um05282 | 0.38506 | K12198 | related to VPS60 involved in vacuolar protein sorting |
| 9c00284 | 0.4742 | K15014 | nucleoside transporter | um04457 | 0.38537 | ni | conserved hypothetical protein |
| 11c00054 | 0.4744 | ni | hypothetical protein | um04046 | 0.38537 | ni | related to aldehyde dehydrogenase |
| 8d00094 | 0.47443 | ni | NDR and related serine/threonine kinases | um11279 | 0.38538 | ni | conserved hypothetical protein |
| 15c00079 | 0.47448 | ni | hypothetical protein | um12203 | 0.3854 | ni | related to CWC2 - involved in mRNA splicing |
| 12c00051 | 0.47464 | ni | hypothetical protein | um06228 | 0.3855 | K14806 | related to DBP7 - RNA helicase required for 60S ribosomal subunit assembly |
| 14c00058 | 0.47483 | ni | hypothetical protein | um11242 | 0.3856 | ni | hypothetical protein |
| 12c00135 | 0.47494 | K08158 | synaptic vesicle transporter SVOP and related transporters | um03972 | 0.38567 | K02892 | conserved hypothetical protein |
| 9c00213 | 0.47499 | ni | hypothetical protein | um00036 | 0.38573 | K09780 | conserved hypothetical protein |
| 18d00062 | 0.47508 | K13338 | AAA+-type ATPase | um06189 | 0.38595 | ni | hypothetical protein |
| 20c00028 | 0.47511 | K15272 | predicted UDP-galactose transporter | um05332 | 0.38595 | ni | conserved hypothetical protein |
| 7d00318 | 0.47537 | ni | hypothetical protein | um12302 | 0.38621 | ni | conserved hypothetical Ustilago-specific protein |
| 19c00028 | 0.47542 | ni | hypothetical protein | um00603 | 0.38624 | ni | putative protein |
| 18d00067 | 0.47544 | ni | hypothetical protein | um10227 | 0.38648 | K02952 | related to mitochondrial ribosomal protein S13 |
| 12d00015 | 0.47547 | K13519 | predicted membrane protein | um10323 | 0.38676 | K02919 | conserved hypothetical protein |
| 22c00158 | 0.47566 | K00860 | adenosine 5'-phosphosulfate kinase | um00493 | 0.38703 | ni | conserved hypothetical protein |
| 3d00072 | 0.47583 | ni | carbon-nitrogen hydrolase | um00742 | 0.38704 | ni | conserved hypothetical protein |
| 20c00018 | 0.47593 | ni | hypothetical protein | um05537 | 0.38714 | ni | conserved hypothetical protein |
| 18c00091 | 0.47593 | ni | hypothetical protein | um00161 | 0.38717 | ni | hypothetical protein |
| 19d00131 | 0.476 | ni | alcohol dehydrogenase | um11594 | 0.3872 | ni | conserved hypothetical protein |
| 20c00077 | 0.47603 | K15371 | glutamate/leucine/phenylalanine/valine dehydrogenases | um04494 | 0.38724 | ni | related to Engulfment and cell motility gene 1 protein |
| 25d00054 | 0.47608 | K15731 | TFIIF-interacting CTD phosphatase, including NLI-interacting fac | um01318 | 0.38725 | K02951 | probable 40S ribosomal protein S12 |
| 27c00052 | 0.47618 | ni | hypothetical protein | um11206 | 0.38726 | ni | conserved hypothetical protein |
| 22d00185 | 0.47624 | ni | dehydrogenases with different specificities | um05285 | 0.38726 | K04460 | related to phosphoprotein phosphatase |
| 26c00020 | 0.47629 | ni | uncharacterized conserved protein | um02337 | 0.38732 | ni | conserved hypothetical protein |
| 25d00042 | 0.47647 | ni | protein tyrosine phosphatase | um04051 | 0.38759 | ni | conserved hypothetical protein |
| 14c00105 | 0.47648 | K12396 | vesicle coat complex AP-3, delta subunit | um10261 | 0.38787 | ni | conserved hypothetical protein |
| 9c00366 | 0.47658 | ni | hypothetical protein | um03050 | 0.38787 | ni | conserved hypothetical protein |
| 9d00333 | 0.47663 | ni | glycolipid transfer protein | um05821 | 0.38828 | ni | hypothetical protein |
| 19d00135 | 0.47664 | ni | hypothetical protein | um10335 | 0.38829 | ni | hypothetical protein |
| 27c00080 | 0.47699 | ni | hypothetical protein | um00715 | 0.38881 | ni | hypothetical protein |
| 3d00090 | 0.47704 | ni | hypothetical protein | um01417 | 0.38917 | ni | related to MDM10 - mitochondrial morphology and inheritance component |
| 14c00079 | 0.47706 | K06631 | polo-like serine/threonine protein kinase | um12023 | 0.38941 | ni | conserved hypothetical protein |
| 26c00006 | 0.47716 | ni | hypothetical protein | um11476 | 0.38971 | ni | related to Guanyl nucleotide exchange factor Sql2 |
| 14c00121 | 0.47749 | ni | hypothetical protein | um01641 | 0.38971 | ni | conserved hypothetical protein |
| 22c00032 | 0.47751 | K05527 | stress-induced protein UVI31+ | um11244 | 0.38987 | ni | related to FRE4 - Ferric reductase |
| 7c00084 | 0.47756 | ni | hypothetical protein | um01505 | 0.38992 | K11252 | probable HTB2 - histone H2B |
| 24c00058 | 0.4777 | ni | helicase-like transcription factor HLTF | um10203 | 0.39016 | ni | hypothetical protein |
| 25d00092 | 0.47772 | ni | hypothetical protein | um01061 | 0.39021 | ni | hypothetical protein |
| 9c00083 | 0.47804 | ni | zinc-binding oxidoreductase | um04462 | 0.39025 | ni | putative protein |
| 18d00100 | 0.47809 | ni | helicase-like transcription factor HLTF | um11765 | 0.39034 | ni | related to 6-hydroxy-D-nicotine oxidase |
| 7c00241 | 0.47819 | ni | actin-related protein Arp2/3 complex, subunit Arp3 | um05742 | 0.3904 | ni | conserved hypothetical protein |
| 9c00222 | 0.47827 | ni | uncharacterized conserved protein | um05539 | 0.39062 | ni | conserved hypothetical protein |
| 22d00040 | 0.4783 | ni | hypothetical protein | um05238 | 0.39073 | ni | conserved hypothetical protein |
| 22d00275 | 0.47847 | ni | cytochrome P450 CYP4/CYP19/CYP26 subfamilies | um02066 | 0.39076 | ni | conserved hypothetical protein |
| 26d00079 | 0.47851 | ni | predicted steroid reductase | um12081 | 0.39086 | ni | conserved hypothetical protein |
| 9c00438 | 0.47855 | ni | predicted importin 9 | um11613.2 | 0.39113 | K15447 | conserved hypothetical protein |
| 7c00257 | 0.47871 | ni | predicted esterase of the alpha-beta hydrolase superfamily | um10353 | 0.39137 | ni | conserved hypothetical protein |
| 2d00041 | 0.47872 | ni | hypothetical protein | um00360 | 0.39184 | ni | hypothetical protein |
| 6d00048 | 0.47881 | ni | hypothetical protein | um04392 | 0.39204 | ni | conserved hypothetical protein |
| 16d00001 | 0.4792 | ni | permease of the major facilitator superfamily | um01464 | 0.39212 | ni | hypothetical protein |
| 9c00328 | 0.47937 | K08286 | ribosomal protein S6 kinase and related proteins | um11784 | 0.39214 | K03857 | related to SPT14 - N-acetylglucosaminyltransferase |
| 22d00015 | 0.47965 | ni | hypothetical protein | um12104 | 0.39228 | ni | related to Cobalamin synthesis protein |
| 6c00118 | 0.47967 | K14394 | protein tyrosine phosphatase | um01683.2 | 0.39233 | ni | putative protein |
| 12c00130 | 0.47975 | ni | hypothetical protein | um11665 | 0.39233 | K06883 | related to XPA binding protein |
| 7d00329 | 0.47993 | ni | kelch repeat-containing proteins | um10650 | 0.39264 | K16055 | related to trehalose-6-phosphate phosphatase |
| 26d00036 | 0.47996 | K01090 | serine/threonine specific protein phosphatase PP1, catalytic subu | um03941 | 0.39286 | K12737 | related to Cyclophilin-16 |
| 19d00029 | 0.47996 | ni | hypothetical protein | um11060 | 0.39299 | ni | hypothetical protein |

| | | | | | | | |
|---|---|---|---|---|---|---|---|
| 11c00052 | 0.48049 | K03495 | NAD/FAD-utilizing protein | um15007 | 0.39302 | K11108 | related to RCL1 - RNA terminal phosphate cyclase-like protein |
| 18c00086 | 0.48049 | ni | hypothetical protein | um01694 | 0.39305 | ni | conserved hypothetical protein |
| 313c00001 | 0.48051 | K02396 | ni | um10876 | 0.39307 | K01557 | conserved hypothetical protein |
| 9d00183 | 0.48059 | K12844 | mRNA splicing factor PRP31 | um01759 | 0.39319 | ni | conserved hypothetical protein |
| 7c00284 | 0.48071 | K01758 | cystathionine beta-lyases | um12039 | 0.39321 | ni | related to NADH-ubiquinone oxidoreductase 21 |
| 12d00028 | 0.48087 | ni | hypothetical protein | um02904 | 0.39322 | ni | conserved hypothetical protein |
| 22d00049 | 0.48092 | ni | hypothetical protein | um06410 | 0.39322 | K11770 | conserved hypothetical protein |
| 8c00039 | 0.48099 | ni | permease of the major facilitator superfamily | um11959 | 0.39358 | K11568 | conserved hypothetical protein |
| 26d00051 | 0.48107 | K14785 | TBP-binding protein, activator of basal transcription | um02065 | 0.3939 | K06067 | histone deacetylase |
| 20d00061 | 0.48109 | ni | FOG, Predicted E3 ubiquitin ligase | um02911 | 0.3941 | K11849 | related to UBP2 - ubiquitin-specific proteinase |
| 9c00035 | 0.48123 | ni | hypothetical protein | um10643 | 0.39415 | K00226 | related to dihydroorotate dehydrogenase |
| 7d00056 | 0.48142 | ni | hypothetical protein | um04632 | 0.39424 | K02943 | probable ribosomal protein P2 |
| 9d00211 | 0.48144 | ni | uncharacterized conserved protein | um06126 | 0.39433 | ni | Mig2-6 |
| 5c00016 | 0.48157 | ni | hypothetical protein | um04751 | 0.39462 | K14852 | related to RRS1 - regulator of ribosome biogenesis |
| 10d00088 | 0.48165 | ni | hypothetical protein | um04511 | 0.39475 | ni | probable RML2 - mitochondrial ribosomal protein |
| 12d00104 | 0.48168 | K14411 | RNA-binding protein musashi | um00483 | 0.39481 | ni | putative protein |
| 27c00047 | 0.48168 | ni | predicted Rho GTPase-activating protein | um02456 | 0.39487 | ni | conserved hypothetical protein |
| 11c00058 | 0.48179 | K12860 | mRNA splicing protein CDC5 | um00625 | 0.39516 | K02605 | putative protein |
| 22d00120 | 0.48195 | ni | hypothetical protein | um04895 | 0.39517 | ni | putative protein |
| 18d00108 | 0.48197 | ni | hypothetical protein | um11755 | 0.39532 | ni | hypothetical protein |
| 6d00022 | 0.48199 | ni | hypothetical protein | um05107 | 0.39539 | K15163 | conserved hypothetical protein |
| 25c00029 | 0.48201 | ni | hypothetical protein | um10207 | 0.39547 | K01426 | related to AMD2 - acetamidase |
| 9c00320 | 0.48247 | ni | hypothetical protein | um00054 | 0.39554 | ni | putative protein |
| 22c00062 | 0.4828 | ni | hypothetical protein | um02958 | 0.39555 | K11650 | related to SWI |
| 7c00116 | 0.48288 | ni | membrane protein | um12338 | 0.39557 | ni | hypothetical protein |
| 16c00047 | 0.48293 | ni | hypothetical protein | um10971 | 0.39558 | K08065 | related to transcription factor hap3 |
| 25d00087 | 0.48294 | ni | hypothetical protein | um10049 | 0.39588 | ni | related to MNR2 - Manganese resistance protein |
| 12d00099 | 0.48296 | ni | hypothetical protein | um11085 | 0.39619 | K12735 | related to Multidomain cyclophilin type peptidyl-prolyl cis-trans isomerase |
| 19c00116 | 0.48325 | ni | hypothetical protein | um03091 | 0.39625 | ni | hypothetical protein |
| 9d00408 | 0.48329 | K14715 | ZIP-like zinc transporter proteins | um03491 | 0.39634 | K12180 | related to cop9 signalosome complex subunit 7a |
| 22d00211 | 0.48334 | ni | ribulose kinase and related carbohydrate kinases | um04736 | 0.39648 | K12195 | conserved hypothetical protein |
| 8d00041 | 0.48337 | ni | hypothetical protein | um03387 | 0.3965 | ni | ni |
| 12c00107 | 0.48368 | ni | hypothetical protein | um11282.2 | 0.39659 | ni | conserved hypothetical protein |
| 5d00018 | 0.48381 | ni | hypothetical protein | um05229 | 0.39662 | K01192 | related to Beta-mannosidase precursor |
| 10d00062 | 0.48418 | ni | clathrin coat dissociation kinase GAK/PTEN/Auxilin | um03267 | 0.39665 | ni | conserved hypothetical protein |
| 15c00070 | 0.48432 | K04563 | protein kinase PCTAIRE and related kinases | um10602 | 0.3971 | K10706 | related to SEN1 protein |
| 19d00095 | 0.4845 | ni | decapping enzyme complex component DCP1 | um06093 | 0.3971 | K15272 | related to UDP-galactose transporter |
| 12c00010 | 0.48457 | ni | hypothetical protein | um00136.2 | 0.39715 | ni | related to Zinc finger protein |
| 14c00094 | 0.4849 | K02219 | cyclin-dependent protein kinase CDC28, regulatory subunit CKS1 | um04522 | 0.39721 | K10276 | conserved hypothetical protein |
| 16d00075 | 0.48492 | ni | hypothetical protein | um00528 | 0.39724 | ni | conserved hypothetical protein |
| 24c00015 | 0.48493 | ni | hypothetical protein | um02657 | 0.39752 | K00671 | related to NMT1 - N-myristoyltransferase |
| 11d00069 | 0.48507 | K09569 | FKBP-type peptidyl-prolyl cis-trans isomerase | um06462 | 0.39773 | ni | conserved hypothetical protein |
| 9c00327 | 0.48513 | K08490 | SNARE protein SED5 | um06414 | 0.39782 | ni | related to Polyketide synthase |
| 25d00067 | 0.48513 | ni | predicted membrane protein | um05292 | 0.39786 | ni | related to DigA protein |
| 10d00100 | 0.4852 | ni | ubiquitin-protein ligase | um05013 | 0.398 | ni | related to caffeine-induced death protein 1 Cid1 |
| 8d00048 | 0.48527 | ni | hypothetical protein | um03346.2 | 0.39807 | K15859 | related to SKN7 - transcription factor |
| 11d00014 | 0.48527 | ni | hypothetical protein | um01357 | 0.3981 | ni | ni |
| 25c00059 | 0.48528 | ni | hypothetical protein | um02151 | 0.39827 | K00100 | related to 2 |
| 22c00253 | 0.48537 | ni | cytochrome c oxidase assembly protein PET191 | um02167 | 0.39829 | ni | conserved hypothetical protein |
| 22d00230 | 0.48579 | ni | succinyl-coa synthetase, beta subunit | um04797 | 0.39864 | K01783 | probable RPE1 - D-ribulose-5-phosphate 3-epimerase |
| 18d00105 | 0.48597 | ni | ovarian tumor-like cysteine protease | um05088 | 0.39885 | ni | hypothetical protein |
| 16c00091 | 0.48622 | ni | uncharacterized conserved protein | um03366 | 0.39902 | ni | conserved hypothetical protein |
| 7d00007 | 0.48647 | ni | hypothetical protein | um05384 | 0.39923 | ni | conserved hypothetical protein |
| 19c00040 | 0.48678 | ni | amino acid transporters | um11449 | 0.39929 | ni | related to Phenol 2-monooxygenase |
| 22d00042 | 0.48695 | K03441 | aquaporin | um01580 | 0.39929 | K12385 | related to NCR1 - transmembrane glycoprotein |
| 7d00029 | 0.48695 | ni | gluconate transport-inducing protein | um04330 | 0.39932 | ni | related to MRPS8 - mitochondrial ribosomal protein |
| 7d00120 | 0.48702 | K10768 | uncharacterized conserved protein | um05378 | 0.39942 | K17065 | mitochondrial fission factor DNM1 |
| 19d00107 | 0.48705 | ni | predicted membrane protein | um10086 | 0.39987 | ni | conserved hypothetical protein |
| 7c00036 | 0.48716 | K12862 | IRF-2-binding protein CELTIX-1 | um05605 | 0.40006 | ni | hypothetical protein |
| 5c00153 | 0.4876 | ni | arylacetamide deacetylase | um05432.2 | 0.40022 | K12662 | related to PRP4 - U4 |
| 8d00043 | 0.48771 | ni | hypothetical protein | um10747 | 0.40036 | K12586 | related to Exosome complex exonuclease RRP43 |
| 11d00037 | 0.48774 | K15026 | predicted translation initiation factor related to eIF-3a | um00820 | 0.40059 | ni | ni |
| 8c00029 | 0.48809 | ni | sphingoid base-phosphate phosphatase | um10012 | 0.4007 | ni | conserved hypothetical protein |
| 6d00076 | 0.48816 | ni | hypothetical protein | um10999 | 0.40078 | K10524 | related to NRK1 - Nicotinamide riboside kinase |
| 7c00246 | 0.48826 | K10256 | hypothetical protein | um11555 | 0.4009 | ni | hypothetical protein |
| 2d00061 | 0.4884 | K02307 | hypothetical protein | um05577 | 0.40106 | ni | conserved hypothetical protein |
| 7c00287 | 0.48862 | ni | signal transduction serine/threonine kinase | um01300 | 0.40114 | ni | conserved hypothetical Ustilago-specific protein |
| 22c00302 | 0.48863 | ni | 2-enoyl-CoA hydratase | um03724 | 0.40123 | ni | ni |
| 15c00044 | 0.48885 | ni | hypothetical protein | um12286 | 0.40127 | ni | conserved hypothetical protein |
| 19d00055 | 0.48888 | ni | putative steroid membrane receptor Hpr6.6/25-Dx | um03459 | 0.40172 | K10416 | related to dynein light-intermediate chain |
| 10d00075 | 0.489 | K11339 | dosage compensation regulatory complex | um00491 | 0.40175 | K15196 | related to BRF1 - TFIIIB subunit |
| 9c00167 | 0.48901 | K11836 | ubiquitin-specific protease UBP14 | um11415 | 0.40183 | ni | conserved hypothetical Ustilago-specific protein |
| 7d00191 | 0.48912 | ni | hypothetical protein | um04844 | 0.40194 | ni | ni |
| 9c00023 | 0.48912 | ni | hypothetical protein | um04009 | 0.40196 | K06980 | conserved hypothetical protein |
| 14c00061 | 0.48979 | ni | ATP-dependent RNA helicase | um02937 | 0.40215 | ni | conserved hypothetical protein |
| 11d00032 | 0.48981 | ni | hypothetical protein | um04232 | 0.40215 | K14554 | related to S |
| 6c00055 | 0.48985 | K12734 | similar to cyclophilin-type peptidyl-prolyl cis-trans isomerase | um04293 | 0.40237 | ni | conserved hypothetical protein |
| 22d00109 | 0.48986 | ni | vacuolar assembly/sorting proteins VPS39/VAM6/VPS3 | um04818 | 0.40259 | ni | conserved hypothetical protein |
| 22d00277 | 0.49023 | ni | hypothetical protein | um00213 | 0.40281 | K03860 | related to GPI1 - required for N-acetylglucosaminyl phosphatidylinositol synthesis |
| 14c00020 | 0.49047 | K11129 | box H/ACA snoRNP component | um10553 | 0.40306 | ni | conserved hypothetical Ustilago-specific protein |
| 20c00046 | 0.49048 | K02941 | 60S acidic ribosomal protein P0 | um00516 | 0.40309 | K14573 | related to Nucleolar protein NOP4 |
| 26c00051 | 0.49056 | ni | Ca2+-dependent lipid-binding protein CLB1 | um03930 | 0.4031 | ni | conserved hypothetical protein |
| 14c00120 | 0.49061 | K13181 | ATP-dependent RNA helicase | um12044 | 0.40318 | ni | probable TIM9 - Translocase of the mitochondrial Inner Membrane |
| 5d00128 | 0.49083 | K02321 | DNA polymerase alpha-primase complex, polymerase-associated | um11717 | 0.40321 | K11644 | related to SIN3 - transcription regulatory protein |
| 9c00206 | 0.49085 | K01247 | 3-methyladenine DNA glycosidase | um02361 | 0.4035 | ni | related to ATPase inhibitor |
| 19d00161 | 0.49112 | K12273 | preprotein translocase subunit Sec66 | um11077 | 0.40352 | K00485 | related to Dimethylaniline monooxygenase |
| 8c00085 | 0.49123 | ni | permease of the major facilitator superfamily | um06127 | 0.40368 | ni | conserved hypothetical Ustilago-specific protein |
| 14c00017 | 0.49125 | K11863 | ataxin 3 | um03751 | 0.40396 | ni | hypothetical protein |
| 15c00049 | 0.49162 | ni | hypothetical protein | um11635 | 0.40502 | K04554 | probable UBC6 - E2 ubiquitin-conjugating enzyme |
| 12d00031 | 0.4917 | K03004 | DNA-dependent RNA polymerase I | um03146 | 0.40537 | ni | related to TIM54 |
| 24d00061 | 0.49173 | ni | permease of the major facilitator superfamily | um04601 | 0.40549 | ni | hypothetical protein |
| 25d00026 | 0.49193 | ni | actin-related protein - Arp4p/Act3p | um11029 | 0.40556 | K02946 | related to RSM10 - mitochondrial ribosomal protein |
| 14c00129 | 0.49199 | ni | uncharacterized conserved protein | um10074 | 0.40576 | ni | conserved hypothetical protein |
| 12c00006 | 0.49204 | ni | cyclin | um10317.2 | 0.40585 | ni | putative protein |
| 22c00055 | 0.49205 | ni | flavin-containing monooxygenase | um02108 | 0.40592 | ni | conserved hypothetical protein |
| 6d00042 | 0.49224 | K03515 | translesion DNA polymerase - REV1 deoxycytidyl transferase | um02807 | 0.40662 | K09788 | conserved hypothetical protein |
| 6c00125 | 0.49228 | K09553 | molecular co-chaperone STI1 | um05478 | 0.40663 | ni | conserved hypothetical protein |
| 2c00035 | 0.49237 | ni | hypothetical protein | um01308 | 0.40667 | ni | related to Trophinin |
| 19c00013 | 0.49262 | ni | hypothetical protein | um03505.2 | 0.40667 | ni | related to SAC3 - leucine permease transcriptional regulator |
| 27d00057 | 0.49266 | K00872 | homoserine kinase | um00364.2 | 0.40677 | K12844 | related to U4 |
| 22d00010 | 0.49283 | ni | hypothetical protein | um02925 | 0.40686 | ni | hypothetical protein |
| 22c00187 | 0.49284 | ni | hypothetical protein | um04956 | 0.40698 | K08286 | protein kinase Ukc1p |
| 20c00005 | 0.49308 | ni | hypothetical protein | um02672 | 0.40705 | ni | conserved hypothetical protein |
| 13d00012 | 0.49313 | ni | hypothetical protein | um11636 | 0.40732 | ni | related to vacuolar protein sorting 16 |
| 7c00201 | 0.49322 | K11341 | transcription initiation factor IIF, auxiliary subunit | um04765 | 0.40736 | K03439 | probable TRM8 - part of complex required for 7-methylguanosine modification |
| 9c00013 | 0.49331 | ni | hypothetical protein | um12025 | 0.40784 | ni | conserved hypothetical protein |
| 9c00265 | 0.49332 | ni | hypothetical protein | um00017 | 0.40815 | ni | conserved hypothetical protein |
| 5d00102 | 0.49366 | K14154 | hypothetical protein | um11648 | 0.40841 | K06287 | conserved hypothetical protein |
| 25c00089 | 0.49401 | ni | hypothetical protein | um10123 | 0.40865 | K13303 | S6K1-type AGC kinase |
| 11d00059 | 0.49415 | ni | hypothetical protein | um10689 | 0.4087 | K01705 | probable LYS4 - homoaconitase precursor |
| 12c00082 | 0.49433 | K15634 | thymidylate synthase | um04514 | 0.40885 | ni | hypothetical protein |
| 27d00098 | 0.49436 | K12831 | splicing factor 3b, subunit 4 | um02561 | 0.40895 | K14565 | probable NOP58 - required for pre-18S rRNA processing |
| 7d00313 | 0.4944 | ni | hypothetical protein | um05684 | 0.40916 | K12386 | related to cystinosin |
| 14c00006 | 0.49447 | K02830 | checkpoint 9-1-1 complex, RAD1 component | um02344 | 0.40942 | K02519 | related to translation initiation factor IF-2 |
| 12d00093 | 0.49454 | K06316 | nuclear division RFT1 protein | um05836 | 0.40948 | K06911 | conserved hypothetical protein |
| 8d00107 | 0.49454 | ni | hypothetical protein | um11424 | 0.4095 | K01433 | conserved hypothetical protein |
| 6c00010 | 0.49464 | ni | long chain fatty acid elongase | um00981 | 0.40954 | ni | putative protein |
| 22d00116 | 0.49472 | ni | permease of the major facilitator superfamily | um02896 | 0.40965 | ni | putative protein |
| 10d00060 | 0.49473 | K00384 | thioredoxin reductase | um11377 | 0.40965 | ni | ni |
| 20c00059 | 0.49474 | ni | phospholipid scramblase | um01275 | 0.40966 | K02738 | probable PRE3 - 20S proteasome subunit |
| 1c00025 | 0.49507 | ni | hypothetical protein | um11962.2 | 0.40976 | ni | probable ERG24 - C-14 sterol reductase |
| 24d00026 | 0.4952 | ni | hypothetical protein | um01096 | 0.40976 | ni | related to TOM22 - mitochondrial outer membrane import receptor complex subunit |
| 19c00053 | 0.49527 | K12190 | vacuolar sorting protein VPS36 | um05774 | 0.40978 | ni | related to metalloprotease MEP1 |
| 5c00134 | 0.49538 | ni | ribosomal protein S5 | um04368 | 0.40981 | ni | related to endo-1 |
| 9d00340 | 0.4954 | K02891 | 60S ribosomal protein L22 | um05906 | 0.40984 | K05757 | probable Arp2 |
| 8c00084 | 0.49577 | ni | hypothetical protein | um02426 | 0.41012 | K10575 | probable ubiquitin-conjugating enzyme E2 |
| 7c00042 | 0.49577 | ni | hypothetical protein | um11552 | 0.41018 | ni | hypothetical protein |
| 7c00288 | 0.49583 | K11324 | DNA methyltransferase 1-associated protein-1 | um11602 | 0.41046 | ni | related to zinc finger protein |
| 1c00007 | 0.49589 | ni | hypothetical protein | um10551.2 | 0.41062 | K03142 | probable SSL1 - TFIIH subunit |
| 6d00063 | 0.49608 | ni | purple acid phosphatase | um00559 | 0.41132 | ni | hypothetical protein |
| 10c00047 | 0.49617 | ni | hypothetical protein | um10934 | 0.41142 | K11237 | related to Protein scd2 |
| 25c00022 | 0.4962 | K12398 | clathrin-associated protein medium chain | um10625 | 0.41147 | K02896 | probable ribosomal protein L24 |
| 9c00298 | 0.49625 | ni | hypothetical protein | um03064 | 0.41173 | ni | putative protein |
| 1d00029 | 0.49628 | K03189 | predicted metal-binding protein | um05091.2 | 0.41196 | K03238 | probable SUI3 - translation initiation factor eIF2 beta subunit |
| 9c00229 | 0.49638 | ni | hypothetical protein | um00711 | 0.41207 | K06698 | related to Proteasome activator complex subunit 3 |
| 10c00075 | 0.49648 | K14570 | 3'-5' exonuclease | um15081 | 0.41218 | ni | conserved hypothetical protein |
| 12d00014 | 0.49657 | ni | metacaspase involved in regulation of apoptosis | um05736 | 0.41262 | ni | conserved hypothetical protein |
| 1c00021 | 0.49659 | K02954 | mitochondrial/chloroplast ribosomal protein S14/S29 | um03421 | 0.41277 | K14834 | related to NOC3 protein |
| 9d00224 | 0.4967 | ni | hypothetical protein | um03383 | 0.4128 | ni | conserved hypothetical protein |
| 6c00082 | 0.49681 | ni | uncharacterized conserved protein | um05540 | 0.41313 | K12604 | related to CDC39 - component of the CCR4-NOT complex |
| 14c00137 | 0.49685 | ni | hypothetical protein | um04929 | 0.41439 | ni | conserved hypothetical protein |
| 9d00188 | 0.49689 | ni | hypothetical protein | um10596 | 0.41465 | ni | probable aldehyde dehydrogenase |
| 22d00156 | 0.49699 | ni | hypothetical protein | um03074 | 0.41469 | K01527 | probable transcription factor BTF3a |
| 5d00061 | 0.49711 | ni | hypothetical protein | um04026 | 0.41472 | K06176 | related to PUS7 - Pseudouridine synthase |
| 8c00061 | 0.4973 | ni | hypothetical protein | um03927 | 0.41498 | K11552 | conserved hypothetical protein |
| 11d00050 | 0.49736 | ni | hypothetical protein | um04176 | 0.41512 | K01890 | probable FRS1 - phenylalanyl-tRNA synthetase |
| 10d00068 | 0.49743 | ni | hypothetical protein | um11433 | 0.41521 | K15014 | related to Inhibitor-sensitive equilibrative nucleoside transporter 1 |
| 7c00115 | 0.4975 | ni | uncharacterized conserved protein | um04659 | 0.41536 | K00864 | probable glycerol kinase |
| 9d00048 | 0.49783 | ni | hypothetical protein | um10779 | 0.41565 | K08876 | conserved hypothetical protein |
| 7c00305 | 0.49796 | ni | hypothetical protein | um10231 | 0.4157 | K03031 | probable 26S proteasome non-atpase regulatory subunit 8 |
| 15d00078 | 0.49801 | ni | sorbitol dehydrogenase | um01475 | 0.41571 | ni | conserved hypothetical protein |
| 6d00006 | 0.49801 | ni | reductases with broad range of substrate specificities | um10825 | 0.4158 | K00022 | related to 3-hydroxyacyl-CoA dehydrogenase |
| 9c00421 | 0.49804 | ni | mitochondrial/chloroplast ribosome small subunit component | um11172 | 0.4158 | K03448 | probable FEN2 - Pantothenate permease |
| 20d00064 | 0.49826 | ni | hypothetical protein | um04963 | 0.41587 | ni | conserved hypothetical protein |
| 4c00031 | 0.49847 | K16738 | LIS1-interacting protein NUDE | um03784 | 0.41591 | ni | related to STRIATIN |
| 16d00048 | 0.49863 | K11876 | uncharacterized conserved protein | um03947 | 0.41596 | K01288 | probable carboxypeptidase 2 |

| | | | | | | | |
|---|---|---|---|---|---|---|---|
| 25d00017 | 0.4987 | K02726 | 20S proteasome, regulatory subunit alpha type PSMA2/PRE8 | um03980 | 0.4161 | ni | conserved hypothetical protein |
| 2c00030 | 0.49874 | K11279 | nucleosome assembly protein NAP-1 | um03745 | 0.41623 | ni | conserved hypothetical Ustilago-specific protein |
| 10d00014 | 0.49877 | K02925 | 60S ribosomal protein L3 | um01534 | 0.41633 | ni | putative protein |
| 7d00130 | 0.49887 | ni | hypothetical protein | um10645 | 0.41634 | ni | conserved hypothetical protein |
| 24c00060 | 0.49895 | ni | folylpolyglutamate synthase | um11142 | 0.41657 | K01733 | probable THR4 - threonine synthase |
| 12c00016 | 0.49919 | K17302 | vesicle coat complex COPI, beta' subunit | um10422 | 0.41668 | ni | putative protein |
| 22d00199 | 0.49925 | ni | 3-oxoacyl CoA thiolase | um02262 | 0.41706 | K14832 | related to MAK21 - protein required for 60S ribosomal subunit biogenesis |
| 3d00028 | 0.49947 | ni | hypothetical protein | um03746 | 0.41706 | ni | conserved hypothetical Ustilago-specific protein |
| 10d00034 | 0.4995 | K12041 | sodium/hydrogen exchanger protein | um04720 | 0.41733 | ni | putative protein |
| 19d00066 | 0.49963 | ni | hypothetical protein | um04446 | 0.4174 | K12623 | related to U6 snRNA-associated Sm-like protein LSm4 |
| 8d00016 | 0.49967 | K11568 | hypothetical protein | um03535 | 0.41749 | ni | hypothetical protein |
| 10d00032 | 0.49995 | ni | hypothetical protein | um11599 | 0.4175 | ni | putative protein |
| 8c00098 | 0.50006 | ni | hypothetical protein | um11182 | 0.41755 | ni | conserved hypothetical protein |
| 3d00073 | 0.50007 | ni | hypothetical protein | um01529 | 0.41757 | K03134 | related to TAF10 - TFIID and SAGA subunit |
| 16d00077 | 0.50011 | K15115 | mitochondrial FAD carrier protein | um01504 | 0.41761 | K11251 | probable Histone H2A |
| 7c00341 | 0.50019 | K09250 | E3 ubiquitin ligase interacting with arginine methyltransferase | um11089 | 0.41786 | ni | related to CHS7 - control of protein export from the ER |
| 22d00039 | 0.50022 | K11252 | histone H2B | um04812 | 0.41787 | ni | putative protein |
| 18d00114 | 0.50056 | ni | hypothetical protein | um10386 | 0.41798 | ni | conserved hypothetical protein |
| 26d00080 | 0.50057 | K03012 | RNA polymerase II, fourth largest subunit | um05295 | 0.41809 | ni | conserved hypothetical Ustilago-specific protein |
| 10c00072 | 0.50073 | K01922 | uncharacterized conserved protein | um02386 | 0.41814 | K02965 | probable mitochondrial ribosomal protein S19 |
| 7c00270 | 0.50085 | K11808 | phosphoribosylamidoimidazole-succinocarboxamide synthase | um00156.2 | 0.41819 | ni | conserved hypothetical protein |
| 9d00252 | 0.5009 | ni | hypothetical protein | um11066 | 0.41826 | ni | conserved hypothetical Ustilago-specific protein |
| 9d00127 | 0.50165 | ni | predicted dehydrogenase | um01957 | 0.41835 | K01230 | related to Mannosyl-oligosaccharide alpha-1 |
| 2d00044 | 0.50177 | ni | transport protein particle TRAPP complex subunit | um00745 | 0.41842 | ni | putative protein |
| 22c00177 | 0.50184 | K12584 | uncharacterized conserved protein | um06201 | 0.41864 | K04499 | probable RVB1 - RUVB-like protein |
| 22c00011 | 0.50192 | ni | HMG-box transcription factor | um03046 | 0.41865 | ni | hypothetical protein |
| 6d00005 | 0.50205 | K00155 | aldehyde dehydrogenase | um02450 | 0.41884 | K03263 | probable HYP2 - translation initiation factor eIF5A |
| 26d00039 | 0.50207 | K03934 | NADH-ubiquinone oxidoreductase, NDUFS1/75 kDa subunit | um05603 | 0.41901 | K01053 | related to Gluconolactonase |
| 9d00286 | 0.50208 | ni | helicase-like transcription factor HLTF | um06279 | 0.41919 | ni | hypothetical protein |
| 2c00054 | 0.50211 | K02901 | 60S ribosomal protein L27 | um02138 | 0.41931 | ni | effector family protein Eff1-8 |
| 18c00003 | 0.50225 | ni | predicted membrane protein | um11667 | 0.41945 | K12598 | probable MTR4 - involved in nucleocytoplasmic transport of mRNA |
| 10c00066 | 0.50231 | K04708 | ethanolamine-p-transferase GPI11/PIG-F | um01937 | 0.41954 | ni | related to acid sphingomyelinase |
| 9c00289 | 0.50239 | K04649 | ubiquitin-protein ligase | um01087 | 0.41981 | K03347 | probable SCF complex member Cullin 1 |
| 3d00053 | 0.50246 | K02328 | DNA polymerase delta, regulatory subunit 55 | um10737 | 0.41985 | K14567 | conserved hypothetical protein |
| 12c00007 | 0.50248 | ni | hypothetical protein | um11583 | 0.41998 | K03939 | related to NADH2 dehydrogenase |
| 9d00360 | 0.50268 | ni | mitochondrial import inner membrane translocase, subunit TIM8 | um04856 | 0.42002 | ni | conserved hypothetical protein |
| 9d00025 | 0.50277 | ni | hypothetical protein | um01842 | 0.42002 | ni | hypothetical protein |
| 6c00039 | 0.50279 | K15109 | mitochondrial carrier protein | um01244 | 0.42035 | ni | conserved hypothetical protein |
| 9d00161 | 0.50288 | ni | hypothetical protein | um05305 | 0.42038 | ni | conserved hypothetical Ustilago-specific protein |
| 22d00061 | 0.50324 | ni | vacuolar sorting protein VPS52 | um15087 | 0.42054 | ni | conserved hypothetical protein |
| 11c00078 | 0.50337 | ni | nicotinic acid phosphoribosyltransferase | um11214 | 0.4207 | ni | related to conserved oligomeric Golgi complex component 6 |
| 19d00162 | 0.50347 | ni | ras1 guanine nucleotide exchange factor | um00778 | 0.42075 | K03955 | probable ACP1 - mitochondrial acyl-carrier protein |
| 25d00014 | 0.50391 | ni | hypothetical protein | um11240 | 0.4208 | ni | putative protein |
| 22c00180 | 0.50457 | ni | hypothetical protein | um01238 | 0.42092 | ni | conserved hypothetical Ustilago-specific protein |
| 24c00062 | 0.50487 | ni | hypothetical protein | um05801 | 0.42097 | ni | related to C2H2 zinc finger protein |
| 5d00044 | 0.50497 | K02987 | 40S ribosomal protein S4 | um00032 | 0.42103 | ni | hypothetical protein |
| 11d00013 | 0.50499 | K03649 | G/T mismatch-specific thymine DNA glycosylase | um03003 | 0.42142 | ni | probable GUP1 - Multimembrane-spanning protein essential for proton symport of glycerol |
| 6c00108 | 0.50503 | K00939 | adenylate kinase | um10556 | 0.42174 | ni | hypothetical protein |
| 9d00329 | 0.50508 | ni | hypothetical protein | um01519 | 0.42174 | ni | putative protein |
| 9d00074 | 0.50511 | K15172 | RNA polymerase II transcription elongation factor DSIF/SUPT5H | um12121 | 0.42177 | ni | putative protein |
| 3d00033 | 0.50522 | ni | predicted spermine/spermidine synthase | um06459 | 0.42178 | ni | ni |
| 26c00022 | 0.50531 | ni | hypothetical protein | um11223 | 0.42208 | ni | conserved hypothetical protein |
| 9d00264 | 0.50559 | K10601 | E3 ubiquitin ligase | um02422 | 0.42221 | K11236 | related to CDC24 - GTP |
| 9c00113 | 0.5059 | K12624 | U6 snRNA-associated Sm-like protein | um10639 | 0.42222 | ni | conserved hypothetical protein |
| 9c00094 | 0.50595 | ni | hypothetical protein | um00199 | 0.4224 | K03247 | related to eIF3h -translation initiation factor 3 subunit H |
| 26c00072 | 0.50605 | ni | hypothetical protein | um02812 | 0.42269 | ni | hypothetical protein |
| 16d00081 | 0.50608 | ni | hypothetical protein | um10833 | 0.42286 | K00653 | related to histone acetyltransferase 3 |
| 22d00151 | 0.50631 | K15463 | initiator tRNA phosphoribosyl-transferase | um05294 | 0.42339 | ni | conserved hypothetical Ustilago-specific protein |
| 9d00307 | 0.50691 | K02605 | hypothetical protein | um00763 | 0.42355 | ni | conserved hypothetical protein |
| 9c00066 | 0.50708 | ni | synaptic vesicle transporter SVOP and related transporters | um11146 | 0.42371 | ni | hypothetical protein |
| 26d00023 | 0.50718 | K01556 | L-kynurenine hydrolase | um10533 | 0.42373 | K00164 | probable KGD1 - alpha-ketoglutarate dehydrogenase |
| 20d00071 | 0.50719 | ni | hypothetical protein | um10360 | 0.42382 | K02955 | probable 40S Ribosomal protein S14 |
| 11c00056 | 0.50725 | ni | uncharacterized conserved protein | um03307 | 0.42405 | K01934 | related to 5-formyltetrahydrofolate cyclo-ligase |
| 7d00160 | 0.50727 | K15029 | RNA polymerase I-associated factor - PAF67 | um11691 | 0.42424 | ni | conserved hypothetical protein |
| 27c00051 | 0.5073 | K17103 | hypothetical protein | um11417 | 0.42439 | ni | conserved hypothetical Ustilago-specific protein |
| 18c00041 | 0.50738 | K11338 | DNA helicase TIP49 | um10343 | 0.4244 | ni | conserved hypothetical protein |
| 6d00112 | 0.50739 | ni | hypothetical protein | um11617 | 0.42454 | K13511 | related to TAZ1 - Lyso-phosphatidylcholine acyltransferase |
| 10d00071 | 0.5074 | ni | hypothetical protein | um06306 | 0.42462 | ni | related to YAK1 - ser |
| 16d00040 | 0.50759 | ni | mitochondrial ribosomal protein L16 | um05653.2 | 0.42479 | ni | conserved hypothetical protein |
| 5d00099 | 0.50761 | K08054 | calnexin | um04346 | 0.42487 | K06100 | related to Symplekin |
| 24d00025 | 0.5079 | ni | NADH pyrophosphatase I of the Nudix family of hydrolases | um11318 | 0.42497 | K00670 | probable N-terminal acetyltransferase complex subunit ARD1 |
| 4c00013 | 0.50791 | ni | hypothetical protein | um10512 | 0.425 | K03013 | probable 25 kD subunit of DNA-directed RNA polymerases I |
| 22c00316 | 0.50795 | ni | hypothetical protein | um03178 | 0.42503 | K14325 | conserved hypothetical protein |
| 22d00250 | 0.50858 | K10858 | DNA mismatch repair protein - MLH2/PMS1/Pms2 family | um11643 | 0.42509 | ni | putative protein |
| 14c00071 | 0.50879 | ni | hypothetical protein | um02564 | 0.42523 | K14545 | conserved hypothetical protein |
| 14c00065 | 0.50882 | ni | hypothetical protein | um05127 | 0.42523 | K15175 | related to CDC73 - DNA-directed RNA polymerase II accessory protein |
| 11d00012 | 0.50901 | ni | hypothetical protein | um11314 | 0.42529 | K09250 | related to GIS2 - Putative zinc finger protein |
| 22d00237 | 0.50908 | K13171 | splicing coactivator SRm160/300, subunit SRm160 | um03541 | 0.42543 | ni | related to Meiotic expression upregulated protein 26 |
| 26d00090 | 0.50913 | ni | hypothetical protein | um04613 | 0.42545 | ni | related to APP1 - Actin Patch Protein |
| 7d00060 | 0.50917 | ni | molecular chaperones HSP105/HSP110/SSE1 | um00895 | 0.42549 | ni | conserved hypothetical protein |
| 9d00389 | 0.50925 | K01937 | CTP synthase | um03823 | 0.42556 | ni | hypothetical protein |
| 7c00137 | 0.50938 | K14397 | mRNA cleavage factor I subunit | um10821 | 0.42577 | ni | conserved hypothetical protein |
| 11d00051 | 0.5095 | ni | actin-binding protein Coronin | um00251 | 0.42605 | ni | conserved hypothetical protein |
| 20c00020 | 0.50969 | ni | hypothetical protein | um04845 | 0.42611 | ni | conserved hypothetical protein |
| 19c00131 | 0.50971 | K08495 | SNARE protein GS28 | um04558 | 0.42613 | ni | conserved hypothetical protein |
| 22c00223 | 0.50985 | K00624 | carnitine O-acyltransferase CPT2/YAT1 | um05614 | 0.42617 | ni | related to Acetyltransferase |
| 16d00083 | 0.51005 | ni | hypothetical protein | um05095 | 0.42634 | ni | hypothetical protein |
| 3c00072 | 0.51018 | K13099 | uncharacterized protein | um05646 | 0.42637 | ni | conserved hypothetical protein |
| 14c00026 | 0.51037 | ni | hypothetical protein | um11471.2 | 0.42657 | ni | conserved hypothetical protein |
| 10c00085 | 0.51065 | ni | hypothetical protein | um10615 | 0.42694 | K07976 | probable VPS21 - Rab5-like GTPase involved in vacuolar protein sorting |
| 3d00062 | 0.51105 | ni | mitochondrial RNA helicase SUV3 | um02520 | 0.4271 | ni | conserved hypothetical protein |
| 8d00114 | 0.51108 | ni | hypothetical protein | um02487 | 0.42711 | ni | conserved hypothetical protein |
| 9c00292 | 0.51111 | ni | hypothetical protein | um03701 | 0.42737 | ni | conserved hypothetical protein |
| 16c00061 | 0.51121 | ni | uncharacterized mRNA-associated protein RAP55 | um01499 | 0.42743 | ni | hypothetical protein |
| 6d00059 | 0.51121 | ni | hypothetical protein | um12321 | 0.42749 | ni | conserved hypothetical protein |
| 9d00023 | 0.51122 | ni | uncharacterized conserved protein | um01017 | 0.42756 | ni | conserved hypothetical protein |
| 10c00025 | 0.51124 | K14309 | cullins | um05309 | 0.42763 | ni | conserved hypothetical Ustilago-specific protein |
| 7c00028 | 0.51136 | ni | hypothetical protein | um01060 | 0.4277 | K02964 | probable RPS18A - ribosomal protein S18 |
| 6c00054 | 0.51144 | ni | hypothetical protein | um06169 | 0.42781 | K15901 | conserved hypothetical protein |
| 9d00011 | 0.51155 | ni | hypothetical protein | um04562 | 0.42793 | K14454 | probable aspartate aminotransferase |
| 9d00163 | 0.51158 | K04555 | ubiquitin-protein ligase | um11894 | 0.42806 | ni | putative protein |
| 3c00050 | 0.51195 | K06927 | putative translation initiation inhibitor UK114/IBM1 | um10347 | 0.42808 | K11672 | related to ARP5 - Actin-related protein |
| 25d00094 | 0.51221 | ni | hypothetical protein | um04313 | 0.4281 | ni | putative protein |
| 24d00050 | 0.5126 | K00121 | alcohol dehydrogenase | um00233 | 0.42812 | K14328 | conserved hypothetical protein |
| 9c00073 | 0.51266 | K11313 | histone acetyltransferase PCAF/SAGA, subunit SUPT3H/SPT3 | um00067 | 0.42831 | K10625 | related to ubiquitin-protein ligase e3 component |
| 19c00097 | 0.51294 | ni | hypothetical protein | um11489 | 0.42842 | ni | hypothetical protein |
| 19d00067 | 0.51297 | ni | uncharacterized conserved protein | um06049 | 0.42855 | ni | putative protein |
| 13d00047 | 0.51298 | ni | armadillo/beta-catenin-like repeat-containing protein | um05780 | 0.42879 | ni | hypothetical protein |
| 7d00146 | 0.51304 | K00294 | delta-1-pyrroline-5-carboxylate dehydrogenase | um05580 | 0.42882 | K07941 | probable ADP-ribosylation factor 6 |
| 9c00063 | 0.51306 | ni | predicted dehydrogenase | um03553 | 0.42883 | ni | conserved hypothetical protein |
| 4d00034 | 0.51317 | K01759 | glyoxalase | um10043 | 0.42887 | K00791 | related to tRNA isopentenylpyrophosphate transferase |
| 7c00150 | 0.51362 | ni | hypothetical protein | um06071 | 0.42893 | ni | related to Para-nitrobenzyl esterase |
| 13d00071 | 0.51446 | ni | hypothetical protein | um04202 | 0.4291 | K11835 | related to UBP12 - ubiquitin C-terminal hydrolase |
| 24d00047 | 0.5146 | K08065 | CCAAT-binding factor, subunit A | um06143 | 0.42911 | ni | hypothetical protein |
| 24d00029 | 0.51508 | K02895 | hypothetical protein | um03218 | 0.42921 | ni | related to Secretory carrier-associated membrane protein 2 |
| 27d00070 | 0.51532 | K00413 | cytochrome c1 | um04860 | 0.42935 | K09142 | conserved hypothetical protein |
| 10c00092 | 0.51543 | ni | peptidyl-prolyl cis-trans isomerase | um00362 | 0.42937 | ni | conserved hypothetical protein |
| 16c00043 | 0.51544 | ni | uncharacterized protein PSP1 | um02901 | 0.42942 | ni | conserved hypothetical protein |
| 22c00108 | 0.51604 | ni | hypothetical protein | um00440 | 0.42943 | ni | conserved hypothetical protein |
| 6d00093 | 0.51622 | K12873 | G10 protein | um04407 | 0.42956 | ni | conserved hypothetical protein |
| 22d00093 | 0.51623 | K00670 | N-acetyltransferase | um05974 | 0.42963 | ni | putative protein |
| 8d00097 | 0.51652 | K10592 | E3 ubiquitin-protein ligase | um11719 | 0.42967 | K02987 | probable ribosomal protein S4 |
| 6d00036 | 0.5166 | K12161 | ubiquitin-like protein | um02245 | 0.42971 | K14004 | probable SEC13 - protein transport protein |
| 27c00097 | 0.5166 | ni | multidrug resistance-associated protein | um01040 | 0.42978 | K00823 | probable 4-aminobutyrate aminotransferase |
| 5d00094 | 0.51679 | ni | hypothetical protein | um11159 | 0.42998 | ni | putative protein |
| 18c00028 | 0.5173 | ni | vesicle coat complex AP-1/AP-2/AP-4, beta subunit | um10748 | 0.43001 | ni | conserved hypothetical protein |
| 7d00043 | 0.51759 | K03145 | transcription elongation factor TFIIS | um00270 | 0.43014 | ni | putative protein |
| 18d00057 | 0.51764 | ni | hypothetical protein | um11209 | 0.43021 | K07973 | probable Guanine nucleotide-binding protein gamma subunit |
| 22c00107 | 0.51787 | ni | hypothetical protein | um01273 | 0.43042 | K11855 | related to ubiquitin carboxyl-terminal hydrolase 36 |
| 4d00033 | 0.51793 | K16803 | microtubule-associated protein | um01940 | 0.43046 | K14022 | conserved hypothetical protein |
| 22d00029 | 0.51803 | ni | SNARE protein TLG1 | um03685 | 0.43047 | ni | related to YVC1 - vacuolar cation channel |
| 15d00047 | 0.51818 | ni | hypothetical protein | um10522 | 0.43052 | K04711 | related to YPC1 - Alkaline ceramidase |
| 16c00031 | 0.51819 | ni | hypothetical protein | um01811 | 0.43057 | ni | related to complex I intermediate-associated protein CIA30 precursor |
| 25c00057 | 0.51826 | ni | sensory transduction histidine kinase | um05750 | 0.43062 | ni | conserved hypothetical Ustilago-specific protein |
| 22c00104 | 0.51859 | K08516 | SNARE protein YKT6, synaptobrevin | um00446 | 0.43136 | K05349 | probable beta-glucosidase |
| 27c00018 | 0.51864 | K14776 | RNA Helicase | um03274 | 0.43149 | ni | conserved hypothetical protein |
| 20d00039 | 0.51864 | K01580 | glutamate decarboxylase | um04898 | 0.43159 | ni | related to HSD1 - ER membrane protein |
| 8d00059 | 0.51879 | ni | permease of the major facilitator superfamily | um00289 | 0.43183 | K11497 | related to inner kinetochore protein MIF2 |
| 18d00084 | 0.5189 | ni | hypothetical protein | um06222 | 0.43205 | ni | hypothetical protein |
| 15c00032 | 0.51895 | ni | hypothetical protein | um01703 | 0.43209 | ni | conserved hypothetical protein |
| 10d00119 | 0.51904 | ni | hypothetical protein | um01873 | 0.43219 | ni | conserved hypothetical protein |
| 13d00007 | 0.51914 | ni | uncharacterized conserved protein | um11689 | 0.43227 | K11806 | related to SOF1 - involved in 18S pre-rRNA production |
| 9d00233 | 0.51916 | ni | hypothetical protein | um03348 | 0.43245 | ni | conserved hypothetical protein |
| 6c00075 | 0.51918 | ni | flavin-containing monooxygenase | um10741 | 0.43278 | ni | related to Sedlin |
| 9d00186 | 0.51933 | ni | hypothetical protein | um10276 | 0.43306 | K00088 | inosine 5 |
| 13c00046 | 0.51947 | ni | hypothetical protein | um03788 | 0.43318 | ni | conserved hypothetical protein |
| 12c00064 | 0.51992 | ni | hypothetical protein | um05108 | 0.43337 | K01869 | probable CDC60 - leucine--tRNA ligase |
| 20d00019 | 0.52004 | ni | hypothetical protein | um11963 | 0.43345 | ni | related to MMM1 - required for mitochondrial shape and structure |
| 16c00003 | 0.52004 | ni | hypothetical protein | um01437 | 0.43358 | ni | conserved hypothetical protein |
| 8d00011 | 0.52019 | K02937 | 60S ribosomal protein L7 | um12166 | 0.43368 | K12946 | related to SPC1 - signal peptidase 10 |
| 8c00104 | 0.52023 | ni | predicted kinase | um00837 | 0.43392 | ni | ni |
| 7c00337 | 0.52025 | K02962 | 40S ribosomal protein S17 | um02525.2 | 0.43402 | K11971 | related to ring finger protein 14 |
| 8d00061 | 0.5206 | ni | hypothetical protein | um05039 | 0.43416 | ni | related to GTPase activating protein |
| 9d00339 | 0.52088 | ni | hypothetical protein | um12329 | 0.43425 | ni | conserved hypothetical protein |
| 18c00068 | 0.5209 | ni | hypothetical protein | um01965 | 0.4343 | K14685 | related to ferroportin 1 |
| 9c00420 | 0.52113 | K15190 | predicted methyltransferase | um02684 | 0.43445 | K03036 | probable 26S proteasome non-atpase regulatory subunit Rpn6 |

| | | | | | | | |
|---|---|---|---|---|---|---|---|
| 5c00121 | 0.52122 | ni | predicted methyltransferase | um10079 | 0.43456 | ni | probable lanosterol synthase |
| 8d00012 | 0.52135 | K17095 | annexin | um00262 | 0.43473 | ni | conserved hypothetical protein |
| 9c00432 | 0.52136 | K07107 | hypothetical protein | um11295 | 0.43484 | ni | ni |
| 22d00045 | 0.52158 | K04368 | mitogen-activated protein kinase kinase | um01045 | 0.435 | K13123 | conserved hypothetical protein |
| 5d00127 | 0.52163 | K15306 | ran-binding protein RANBP1 and related RanBD domain proteins | um10475 | 0.43544 | ni | putative protein |
| 19c00083 | 0.52165 | K10997 | DNA topoisomerase I-interacting protein | um00536 | 0.43555 | ni | conserved hypothetical protein |
| 19d00157 | 0.5218 | ni | FOG, Low-complexity | um01495 | 0.43556 | K15115 | related to YIA6 - Pyruvate transporter of the mitochondrial inner membrane |
| 10c00053 | 0.52203 | ni | bola (bacterial stress-induced morphogen)-related protein | um03554 | 0.43562 | ni | conserved hypothetical protein |
| 9d00192 | 0.52207 | ni | hypothetical protein | um01146 | 0.43566 | ni | conserved hypothetical protein |
| 9d00117 | 0.52242 | K16185 | GTP-binding protein | um05406 | 0.43595 | ni | endosomal t-SNARE |
| 6d00016 | 0.52244 | ni | hypothetical protein | um02538 | 0.43597 | ni | conserved hypothetical Ustilago-specific protein |
| 7d00126 | 0.52257 | ni | ferric reductase | um01355 | 0.43603 | K15030 | related to eIF3m -translation initiation factor 3 subunit M |
| 9c00318 | 0.52272 | K01488 | adenine deaminase | um00926 | 0.43614 | K02872 | probable ribosomal protein L13a |
| 5c00123 | 0.52274 | K00033 | 6-phosphogluconate dehydrogenase | um01834 | 0.43628 | ni | conserved hypothetical protein |
| 9c00214 | 0.52285 | K01441 | ceramidases | um00476 | 0.43648 | ni | related to 2-hydroxy-6-oxo-6-phenylhexa-2 |
| 19d00121 | 0.52291 | ni | hypothetical protein | um00501 | 0.43648 | ni | hypothetical protein |
| 16c00067 | 0.52307 | K03869 | cullins | um10617 | 0.43658 | ni | conserved hypothetical protein |
| 15c00025 | 0.5232 | ni | hypothetical protein | um04605 | 0.43675 | K12274 | conserved hypothetical protein |
| 6c00070 | 0.52338 | K15015 | amino acid transporters | um00798 | 0.43676 | K15505 | related to RAD5 - DNA helicase |
| 7c00058 | 0.52368 | ni | uncharacterized conserved protein | um11478 | 0.43681 | ni | putative protein |
| 12d00063 | 0.5237 | ni | hypothetical protein | um00557 | 0.43698 | K01191 | probable AMS1 - alpha-mannosidase |
| 19c00100 | 0.52384 | K15206 | hypothetical protein | um01383 | 0.43712 | ni | putative protein |
| 7d00331 | 0.5239 | K06018 | acid sphingomyelinase and PHM5 phosphate metabolism protein | um10393 | 0.4372 | ni | conserved hypothetical protein |
| 8c00092 | 0.52394 | K16833 | hypothetical protein | um03821 | 0.4373 | ni | hypothetical protein |
| 9d00160 | 0.52404 | ni | hypothetical protein | um06065 | 0.43737 | ni | related to MET12 - methylenetetrahydrofolate reductase |
| 22c00072 | 0.52404 | ni | cactin | um12087 | 0.43754 | ni | related to NADPH-dependent FMN and FAD containing oxidoreductase |
| 7c00213 | 0.52406 | K15628 | peroxisomal long-chain acyl-CoA transporter | um10193 | 0.43757 | ni | hypothetical protein |
| 10d00120 | 0.52414 | ni | hypothetical protein | um11591 | 0.43759 | ni | related to SSP120 - secretory protein |
| 6c00011 | 0.52431 | K01796 | predicted L-carnitine dehydratase | um00071 | 0.43763 | K00809 | probable deoxyhypusine synthase |
| 22d00128 | 0.52435 | ni | hypothetical protein | um00550 | 0.43766 | ni | hypothetical protein |
| 9c00096 | 0.52439 | ni | hypothetical protein | um01302 | 0.43768 | ni | conserved hypothetical Ustilago-specific protein |
| 5c00108 | 0.52451 | ni | hypothetical protein | um11152 | 0.4381 | ni | related to 2-amino-3-carboxylmuconate-6-semialdehyde decarboxylase |
| 22c00004 | 0.52485 | ni | hypothetical protein | um04463 | 0.43828 | K10082 | related to vesicular integral-membrane protein VIP36 |
| 7c00177 | 0.52511 | K15456 | RNA polymerase II elongator associated protein | um05471 | 0.43833 | ni | conserved hypothetical protein |
| 22d00021 | 0.52532 | ni | hypothetical protein | um00099 | 0.43863 | K00968 | related to choline-phosphate cytidylyltransferase |
| 22c00295 | 0.52547 | ni | hypothetical protein | um10983.2 | 0.43865 | ni | conserved hypothetical protein |
| 5d00098 | 0.52595 | K06063 | mRNA splicing factor | um11173 | 0.43869 | ni | putative protein |
| 4d00010 | 0.52604 | K00327 | NADP/FAD dependent oxidoreductase | um03240 | 0.43887 | ni | related to RUD3 - suppressor of uso1-1 transport defect |
| 9c00396 | 0.5261 | K03448 | permease of the major facilitator superfamily | um00821 | 0.43892 | ni | hypothetical protein |
| 14d00097 | 0.52613 | ni | hypothetical protein | um10661.2 | 0.43898 | K03256 | related to GCD10 - translation initiation factor eIF3 RNA-binding subunit |
| 10c00078 | 0.52613 | K14686 | copper transporter | um03422 | 0.43899 | ni | conserved hypothetical protein |
| 22c00021 | 0.5262 | ni | hypothetical protein | um11096 | 0.43908 | K17279 | related to YOP1 - Ypt-interacting protein |
| 22d00121 | 0.52649 | K03936 | NADH-ubiquinone oxidoreductase, NDUFS3/30 kDa subunit | um04039 | 0.4391 | ni | hypothetical protein |
| 3c00005 | 0.52649 | K03245 | hypothetical protein | um00218 | 0.43925 | K15542 | related to PFS2 - polyadenylation factor I subunit 2 |
| 4d00021 | 0.52696 | ni | predicted methyltransferase | um04375 | 0.43931 | K11436 | related to hnRNP arginine N-methyltransferase |
| 9c00147 | 0.52731 | ni | hypothetical protein | um02216 | 0.43948 | K15109 | related to Carrier protein YMC1 |
| 18d00042 | 0.52761 | ni | golgi transport complex COD1 protein | um03355 | 0.4395 | ni | related to ERV25 - component of the COPII-coated vesicles |
| 18c00099 | 0.52771 | ni | hypothetical protein | um03782 | 0.4397 | ni | hypothetical protein |
| 13d00050 | 0.52794 | ni | hypothetical protein | um02016 | 0.43981 | K17278 | related to Membrane steroid binding protein |
| 19d00124 | 0.52803 | ni | 3-keto sterol reductase | um04809 | 0.43983 | K14823 | related to EBP2 - required for pre-rRNA processing and ribosomal subunit assembly |
| 11c00017 | 0.52819 | K11560 | hypothetical protein | um02064 | 0.43985 | K02151 | probable VMA7 - H -ATPase V1 domain 14 kDa subunit |
| 22d00129 | 0.52848 | K16732 | microtubule-associated protein essential for anaphase spindle elo | um11130 | 0.44016 | ni | conserved hypothetical protein |
| 8d00025 | 0.52852 | ni | permease of the major facilitator superfamily | um05363 | 0.44049 | K12837 | related to pre-mRNA splicing factor U2AF large chain |
| 13c00109 | 0.52854 | ni | protein involved in sister chromatid separation and/or segregation | um04722 | 0.4405 | K03012 | related to Rpb4 - 16 kD subunit of DNA-directed RNA polymerase II |
| 6d00125 | 0.52855 | K06671 | sister chromatid cohesion complex Cohesin, subunit STAG/IRR1 | um03534 | 0.44065 | ni | putative protein |
| 5d00116 | 0.52876 | K10739 | single-stranded DNA-binding replication protein A | um03752 | 0.44071 | ni | conserved hypothetical Ustilago-specific protein |
| 9c00307 | 0.52876 | K09252 | hypothetical protein | um02132 | 0.44084 | ni | conserved hypothetical protein |
| 22c00119 | 0.529 | ni | transcription-associated recombination protein - Thp1p | um02619 | 0.44103 | K03128 | related to TAF2 - component of TFIID complex |
| 5c00033 | 0.52909 | K14863 | predicted E3 ubiquitin ligase | um12264 | 0.44144 | ni | putative protein |
| 3d00064 | 0.52929 | ni | inositol phospholipid synthesis protein | um11540 | 0.44162 | ni | related to carboxylesterase |
| 19d00114 | 0.52958 | K06963 | THUMP domain-containing proteins | um11325 | 0.4417 | ni | related to VPS17 - vacuolar protein sorting-associated protein |
| 24c00059 | 0.53018 | ni | hypothetical protein | um05719.2 | 0.44171 | ni | putative protein |
| 1d00021 | 0.53052 | K05770 | peripheral-type benzodiazepine receptor and related proteins | um01964 | 0.44188 | ni | related to multidrug resistance protein |
| 6c00100 | 0.53063 | ni | FYVE finger containing protein | um11649 | 0.44195 | ni | related to D-amino acid oxidase |
| 7c00067 | 0.53077 | K10427 | dynactin, subunit p25 | um00140 | 0.44218 | K05542 | related to tRNA dihydrouridine synthase |
| 20d00009 | 0.53101 | ni | hypothetical protein | um06102 | 0.44222 | ni | related to Glucan 1 |
| 7c00155 | 0.53111 | K07374 | alpha tubulin | um06152 | 0.44222 | ni | putative protein |
| 16c00042 | 0.53184 | ni | uncharacterized conserved protein | um02313 | 0.44233 | ni | hypothetical protein |
| 16c00023 | 0.53206 | ni | hypothetical protein | um01072 | 0.4424 | ni | conserved hypothetical protein |
| 7c00048 | 0.53313 | K05757 | actin-related protein Arp2/3 complex, subunit ARPC1/p41-ARC | um02218 | 0.44244 | K01738 | related to cysteine synthase |
| 10c00061 | 0.53326 | ni | hypothetical protein | um01388 | 0.44248 | ni | hypothetical protein |
| 18d00019 | 0.53343 | K00290 | lysine-ketoglutarate reductase | um10326.2 | 0.44272 | K00971 | probable Mannose-1-phosphate guanylyltransferase |
| 13d00042 | 0.53346 | K01196 | alpha amylase | um02327 | 0.44277 | K14169 | hypothetical protein |
| 26c00004 | 0.53354 | ni | predicted transporter | um01055 | 0.44295 | ni | ni |
| 7d00312 | 0.53375 | K16466 | Ca2+-binding protein | um10206 | 0.44317 | K01426 | related to Serine |
| 22c00017 | 0.53451 | ni | hypothetical protein | um11226 | 0.44327 | ni | conserved hypothetical protein |
| 9d00338 | 0.5347 | K13511 | phosphate acyltransferase | um00055 | 0.4433 | K10134 | conserved hypothetical protein |
| 26c00042 | 0.53495 | ni | hypothetical protein | um10884 | 0.44365 | K16075 | related to LPE10 - strong similarity to Mrs2p |
| 1c00044 | 0.53496 | ni | transcription factor e(y)2 | um00632 | 0.44368 | ni | putative protein |
| 7d00340 | 0.53506 | K02957 | 40S ribosomal protein S15/S22 | um02358 | 0.44386 | ni | related to SYP1 |
| 9c00109 | 0.53514 | ni | sister chromatid cohesion complex Cohesin, subunit RAD21/SCC | um04724 | 0.44391 | ni | conserved hypothetical protein |
| 24c00013 | 0.53519 | ni | hypothetical protein | um10628 | 0.44404 | ni | conserved hypothetical protein |
| 26d00031 | 0.53521 | K00253 | isovaleryl-coa dehydrogenase | um00189 | 0.44413 | ni | related to ESF1 - 18S rRNA factor |
| 26d00041 | 0.53539 | ni | ubiquinol cytochrome c reductase assembly protein CBP3 | um02186 | 0.44422 | ni | conserved hypothetical protein |
| 3c00003 | 0.53544 | K11271 | FOG, Predicted E3 ubiquitin ligase | um02343 | 0.44426 | K14833 | related to excision repair protein RAD4 |
| 22c00270 | 0.53556 | ni | hypothetical protein | um03112 | 0.44435 | ni | conserved hypothetical protein |
| 12c00117 | 0.53557 | ni | hypothetical protein | um03353 | 0.44465 | K11863 | related to ataxin-3 |
| 7d00202 | 0.53592 | ni | hypothetical protein | um11082 | 0.4447 | ni | hypothetical protein |
| 7d00207 | 0.53593 | ni | hypothetical protein | um11559 | 0.44478 | K06927 | conserved hypothetical protein |
| 16c00088 | 0.53601 | ni | hypothetical protein | um12289 | 0.4448 | K00859 | conserved hypothetical protein |
| 9c00240 | 0.53618 | ni | hypothetical protein | um02263 | 0.44527 | K01304 | conserved hypothetical protein |
| 22c00045 | 0.5362 | ni | putative mitochondrial/chloroplast ribosomal protein L45 | um00167 | 0.44533 | ni | conserved hypothetical protein |
| 1d00011 | 0.53639 | K13095 | splicing factor 1/branch point binding protein | um00274 | 0.44543 | K02218 | probable casein kinase I |
| 18d00006 | 0.53648 | ni | hypothetical protein | um01978 | 0.44552 | ni | conserved hypothetical protein |
| 13c00036 | 0.53648 | K00924 | serine/threonine protein kinase | um05873 | 0.44554 | ni | related to TIF2 - translation initiation factor eIF4A |
| 9c00158 | 0.53652 | K02331 | predicted regulator of rRNA gene transcription | um01271 | 0.44576 | ni | related to NAM8 - meiotic recombination protein |
| 7c00281 | 0.53663 | ni | hypothetical protein | um03608 | 0.4458 | ni | related to RRP14 - protein involved in Ribosomal RNA Processing |
| 10c00042 | 0.53694 | K01930 | folylpolyglutamate synthase | um01789 | 0.44587 | ni | related to ERG28 - involved in synthesis of ergosterol |
| 14c00083 | 0.53696 | ni | hypothetical protein | um04607 | 0.44588 | ni | related to Asparaginyl-tRNA synthetase |
| 20d00072 | 0.53696 | ni | hypothetical protein | um04782 | 0.4459 | K01885 | probable GUS1 - Glutamyl-tRNA synthetase |
| 18c00075 | 0.53714 | ni | hypothetical protein | um05209 | 0.44625 | K03844 | related to ALG11 - required for asparagine-linked glycosylation |
| 19c00016 | 0.53734 | K16261 | amino acid transporters | um11021 | 0.44638 | ni | probable mitochondrial precursor protein import receptor tom70 |
| 10d00108 | 0.53766 | K12842 | predicted splicing regulator | um02782 | 0.4464 | K02146 | probable vacuolar atp synthase subunit d |
| 5c00037 | 0.53777 | ni | hypothetical protein | um04987 | 0.44648 | ni | conserved hypothetical protein |
| 26d00018 | 0.53798 | ni | hypothetical protein | um11058 | 0.44651 | ni | conserved hypothetical Ustilago-specific protein |
| 13c00086 | 0.53836 | K06875 | apoptosis-related protein | um11749 | 0.4466 | K03246 | probable TIF34 - translation initiation factor eIF3 |
| 2d00063 | 0.53845 | K14777 | ATP-dependent RNA helicase | um10906 | 0.44678 | ni | conserved hypothetical protein |
| 9d00288 | 0.53873 | K00921 | phosphatidylinositol-4-phosphate 5-kinase | um01776 | 0.44707 | ni | conserved hypothetical protein |
| 5c00113 | 0.5388 | K01106 | inositol-1,4,5-triphosphate 5-phosphatase | um04016 | 0.44716 | ni | hypothetical protein |
| 19d00003 | 0.53898 | ni | hypothetical protein | um05026 | 0.44722 | ni | related to NSP1 - nuclear pore protein |
| 7d00172 | 0.53934 | ni | membrane coat complex Retromer, subunit VPS5/SNX1 | um10909.2 | 0.44734 | ni | related to GTPase-activating protein beta-chimerin |
| 26d00025 | 0.53985 | ni | DHHC-type Zn-finger proteins | um00627 | 0.44747 | ni | hypothetical protein |
| 6d00135 | 0.53986 | K01870 | isoleucyl-trna synthetase | um10830 | 0.44839 | K15363 | conserved hypothetical protein |
| 20c00068 | 0.54024 | ni | hypothetical protein | um01009 | 0.4484 | K00693 | probable glycogen synthase |
| 9d00122 | 0.54031 | ni | hypothetical protein | um11106 | 0.44852 | ni | conserved hypothetical protein |
| 22c00120 | 0.54039 | ni | hypothetical protein | um01217 | 0.44861 | ni | hypothetical protein |
| 15c00065 | 0.54098 | ni | hypothetical protein | um11510 | 0.44864 | K12200 | conserved hypothetical protein |
| 9d00372 | 0.54128 | ni | oxidation resistance protein | um02294 | 0.44886 | ni | conserved hypothetical Ustilago-specific protein |
| 9c00295 | 0.54143 | ni | hypothetical protein | um02049 | 0.4491 | ni | putative protein |
| 11d00038 | 0.54146 | ni | predicted metal-dependent hydrolase of the TIM-barrel fold | um01375 | 0.44924 | ni | hypothetical protein |
| 26d00078 | 0.5415 | K01923 | phosphoribosylamidoimidazole-succinocarboxamide synthase | um05928 | 0.44957 | ni | conserved hypothetical Ustilago-specific protein |
| 22c00163 | 0.5417 | ni | hypothetical protein | um05994 | 0.44958 | ni | conserved hypothetical protein |
| 5d00019 | 0.54171 | K02325 | DNA polymerase epsilon, subunit B | um02609 | 0.44966 | K12833 | conserved hypothetical protein |
| 8c00041 | 0.5419 | K03011 | RNA polymerase II, subunit POLR2C/RPB3 | um02851 | 0.44968 | ni | putative protein |
| 7c00269 | 0.54208 | K01104 | dual specificity phosphatase | um02055 | 0.44983 | K14408 | related to RNA14 - component of pre-mRNA 3 |
| 5c00162 | 0.54222 | K01213 | hypothetical protein | um01042 | 0.44991 | ni | putative protein |
| 25c00070 | 0.54237 | ni | mitochondrial associated endoribonuclease MAR1 | um04257 | 0.44992 | ni | hypothetical protein |
| 15d00003 | 0.54273 | ni | hypothetical protein | um10099 | 0.44995 | ni | conserved hypothetical protein |
| 10c00043 | 0.54286 | K06158 | ATPase component of ABC transporters with duplicated ATPase | um01058 | 0.44997 | K12399 | probable APS3 - AP-3 complex subunit |
| 22c00109 | 0.54287 | K12199 | uncharacterized conserved protein | um06236 | 0.45001 | K02858 | probable RIB3 - 3 |
| 9d00308 | 0.54288 | ni | protein phosphatase, regulatory subunit PPP1R3C/D | um06062 | 0.45038 | ni | ni |
| 19c00007 | 0.54306 | ni | hypothetical protein | um11619 | 0.45064 | K02891 | probable ribosomal protein L22 |
| 15d00006 | 0.54343 | K12662 | U4/U6 small nuclear ribonucleoprotein Prp4 | um02638 | 0.45068 | K07870 | conserved hypothetical protein |
| 26c00089 | 0.54352 | ni | hypothetical protein | um15005 | 0.45069 | K11541 | probable URA2 - multifunctional pyrimidine biosynthesis protein |
| 9d00108 | 0.54358 | K11557 | hypothetical protein | um04711 | 0.45075 | ni | conserved hypothetical protein |
| 25d00004 | 0.54369 | K07101 | hypothetical protein | um02667 | 0.45085 | K01027 | probable Succinyl-CoA |
| 7c00144 | 0.54387 | ni | hypothetical protein | um04990 | 0.45098 | ni | hypothetical protein |
| 7d00184 | 0.5439 | ni | hypothetical protein | um00525 | 0.45147 | K04649 | probable UBC1 - E2 ubiquitin-conjugating enzyme |
| 9c00024 | 0.54397 | ni | proline oxidase | um05698 | 0.45147 | K08286 | probable ser |
| 9d00313 | 0.54409 | ni | hypothetical protein | um04840 | 0.45177 | ni | conserved hypothetical protein |
| 4d00005 | 0.54415 | ni | GPI-alpha-mannosyltransferase III biosynthesis | um02271 | 0.45178 | K16912 | conserved hypothetical protein |
| 14c00014 | 0.54466 | K17301 | vesicle coat complex COPI, beta subunit | um03544 | 0.45181 | ni | related to SEC14 - phosphatidylinositol |
| 9c00412 | 0.5447 | ni | hypothetical protein | um04733 | 0.4519 | ni | related to phosphatidylglycerol |
| 11d00057 | 0.5448 | K00817 | histidinol phosphate aminotransferase | um12201 | 0.45217 | K16186 | related to Ras-like G protein RagD |
| 6d00118 | 0.5448 | K03686 | molecular chaperone | um03621 | 0.45224 | K05302 | conserved hypothetical protein |
| 13c00016 | 0.5449 | ni | hypothetical protein | um04209 | 0.45234 | K00555 | related to N2 |
| 9d00051 | 0.5455 | K00968 | phosphorylcholine transferase | um05544 | 0.45247 | ni | probable calmodulin-dependent protein kinase type 1 |
| 4c00005 | 0.54564 | ni | hypothetical protein | um04161 | 0.45284 | ni | related to epoxide hydrolase |
| 9d00302 | 0.54569 | ni | predicted transcription factor DATF1 | um02299 | 0.45296 | ni | hypothetical protein |
| 5c00039 | 0.54592 | ni | uncharacterized conserved protein | um00758 | 0.45371 | ni | conserved hypothetical protein |
| 15d00052 | 0.54595 | ni | C3H1-type Zn-finger protein | um02745 | 0.45371 | ni | conserved hypothetical protein |
| 22d00188 | 0.54615 | ni | hypothetical protein | um05803 | 0.45377 | ni | related to acid phosphatase |
| 12c00085 | 0.54617 | K01918 | panthothenate synthetase | um03595 | 0.45383 | ni | conserved hypothetical protein |
| 6d00117 | 0.54642 | ni | hypothetical protein | um02696 | 0.45389 | K10643 | related to MOT2 - transcriptional repressor |
| 22d00233 | 0.54645 | ni | predicted phosphoglycerate mutase | um00997 | 0.45403 | K03754 | related to GCD7 - translation initiation factor eIF2b |
| 19c00123 | 0.54686 | ni | ER vesicle integral membrane protein | um04777 | 0.45407 | ni | hypothetical protein |

| | | | | | | | |
|---|---|---|---|---|---|---|---|
| 15c00074 | 0.54694 | K03142 | RNA polymerase II transcription initiation/nucleotide excision repa | um06334 | 0.45407 | ni | related to beta 1 |
| 18c00096 | 0.54773 | K01077 | alkaline phosphatase | um02370 | 0.45431 | ni | hypothetical protein |
| 14d00048 | 0.54816 | ni | DNA polymerase epsilon, subunit D | um11444 | 0.45438 | ni | conserved hypothetical Ustilago-specific protein |
| 7c00355 | 0.54822 | K01209 | hypothetical protein | um03273 | 0.4548 | ni | related to Tip elongation aberrant protein 1 |
| 22c00068 | 0.54824 | ni | aldo/keto reductase family proteins | um04960 | 0.45494 | K02950 | related to 30S ribosomal protein S12 |
| 13d00084 | 0.54827 | ni | hypothetical protein | um06304.2 | 0.45523 | ni | conserved hypothetical protein |
| 7d00298 | 0.54832 | K16570 | gamma-tubulin complex, DGRIP91/SPC98 component | um06434 | 0.4553 | ni | conserved hypothetical protein |
| 7c00134 | 0.54838 | ni | hypothetical protein | um10839 | 0.45574 | ni | probable FMP43 - protein found in mitochondrial proteome |
| 25c00039 | 0.54843 | ni | hypothetical protein | um03045.2 | 0.45583 | ni | related to BSD2 - metal homeostasis protein |
| 19c00117 | 0.54848 | K14407 | mRNA cleavage and polyadenylation factor I complex, subunit RN | um10579 | 0.45593 | K02180 | related to mitotic checkpoint protein BUB3 |
| 7d00125 | 0.54859 | K00511 | squalene monooxygenase | um04900 | 0.45627 | K11293 | related to histone transcription regulator HIP1 |
| 7d00271 | 0.54875 | K10862 | hypothetical protein | um00885 | 0.45659 | ni | hypothetical protein |
| 7c00009 | 0.54891 | ni | flavin-containing monooxygenase | um02489 | 0.45664 | ni | putative protein |
| 7d00018 | 0.549 | K04078 | mitochondrial chaperonin | um15035 | 0.45685 | ni | related to 60s ribosomal protein l7 |
| 12d00017 | 0.54901 | K01736 | chorismate synthase | um00667 | 0.45691 | K12872 | related to Pre-mRNA-splicing factor RBM22 |
| 19c00120 | 0.54901 | ni | hypothetical protein | um03314 | 0.45697 | ni | effector family protein Eff1-4 |
| 14c00130 | 0.5492 | ni | dehydrogenases with different specificities | um11710 | 0.45704 | K14560 | probable IMP3 - component of the U3 small nucleolar ribonucleoprotein |
| 8c00012 | 0.54922 | K01077 | alkaline phosphatase | um03148 | 0.45719 | ni | related to ATP-binding cassette protein |
| 9d00269 | 0.54958 | ni | hypothetical protein | um03540 | 0.4572 | ni | conserved hypothetical protein |
| 9c00242 | 0.54959 | ni | hypothetical protein | um02308 | 0.45738 | ni | hypothetical protein |
| 5d00050 | 0.54969 | K03106 | signal recognition particle, subunit Srp54 | um10232 | 0.45768 | ni | probable cytochrome b5 |
| 11d00046 | 0.5499 | ni | subtilisin-related protease | um03832 | 0.45782 | ni | related to BNA2 - tryptophan 2 |
| 7c00345 | 0.55001 | ni | hypothetical protein | um02614 | 0.45784 | K14154 | related to thiamine-phosphate diphosphorylase |
| 10d00010 | 0.55005 | K01939 | adenylosuccinate synthase | um06413 | 0.45785 | ni | putative protein |
| 15c00038 | 0.55008 | ni | hypothetical protein | um10050 | 0.45785 | K12624 | probable U6 snRNA-associated Sm-like protein LSm5 |
| 12d00136 | 0.55009 | K11423 | clathrin coat binding protein | um11086 | 0.45795 | ni | related to KES1 - Member of an oxysterol-binding protein family |
| 22d00240 | 0.55013 | K00854 | sugar (pentulose and hexulose) kinases | um11484 | 0.45807 | ni | hypothetical protein |
| 19c00150 | 0.55042 | ni | hypothetical protein | um11309 | 0.45852 | K17268 | related to coatomer epsilon subunit |
| 22d00193 | 0.55062 | ni | hypothetical protein | um01944 | 0.45858 | ni | related to allantoate permease |
| 9d00383 | 0.55073 | ni | uncharacterized conserved protein TEX2 | um05017.2 | 0.4586 | ni | putative protein |
| 20d00028 | 0.55076 | ni | dehydrogenase kinase | um03555 | 0.45861 | K01950 | probable QNS1 - Glutamine-dependent NAD Synthetase |
| 11d00036 | 0.55086 | ni | hypothetical protein | um01610 | 0.45864 | ni | conserved hypothetical protein |
| 18d00037 | 0.55087 | ni | predicted membrane protein | um10324 | 0.45867 | ni | putative protein |
| 8c00048 | 0.55096 | ni | aromatic amino acid aminotransferase and related proteins | um10988 | 0.45871 | K11827 | probable clathrin coat assembly protein ap17 |
| 22d00208 | 0.55103 | ni | predicted PhzC/PhzF-type epimerase | um15041 | 0.45872 | ni | conserved hypothetical protein |
| 15c00069 | 0.55118 | ni | vacuolar sorting protein PEP3/VPS18 | um03699 | 0.45886 | K01922 | conserved hypothetical protein |
| 27c00056 | 0.55121 | ni | hypothetical protein | um04348 | 0.45887 | K03380 | probable phenol 2-monooxygenase |
| 19d00035 | 0.55141 | ni | golgi-associated protein | um04750 | 0.45891 | ni | conserved hypothetical protein |
| 24d00041 | 0.55154 | ni | hypothetical protein | um11250 | 0.45917 | ni | Mig2-3 |
| 15c00061 | 0.55188 | ni | hypothetical protein | um06162 | 0.45958 | ni | conserved hypothetical protein |
| 7c00273 | 0.55245 | ni | N-methyltransferase | um11623 | 0.45959 | K14539 | probable LSG1 - Large-Subunit GTPase involved in 60S ribosomal subunit biogenesis |
| 12c00077 | 0.55248 | K15305 | uncharacterized conserved protein | um04665 | 0.45993 | K14780 | related to ECM16 - putative DEAH-box RNA helicase |
| 16d00058 | 0.5525 | ni | predicted Dolichyl-phosphate-mannose-protein mannosyltransfera | um05978 | 0.46001 | ni | conserved hypothetical protein |
| 9d00143 | 0.55265 | ni | hypothetical protein | um04461 | 0.46007 | K01537 | probable PMR1 - secretory pathway Ca  -transporting P-type ATPase |
| 12d00061 | 0.55276 | ni | nuclear-export-signal (NES)-containing protein | um10236 | 0.46007 | K02949 | probable RPS11B - ribosomal protein S11B |
| 26d00103 | 0.55278 | ni | hypothetical protein | um15028 | 0.4601 | K01955 | probable arginine-specific carbamoyl-phosphate synthetase |
| 5c00093 | 0.55285 | ni | urea transporter | um03515 | 0.46036 | ni | related to LCB1 - serine C-palmitoyltransferase subunit |
| 7c00159 | 0.55297 | ni | hypothetical protein | um01083 | 0.46054 | K15219 | conserved hypothetical protein |
| 25d00055 | 0.5532 | ni | hypothetical protein | um04047 | 0.46058 | K02537 | probable MAD2 - spindle-assembly checkpoint protein |
| 7c00224 | 0.55375 | K15219 | hypothetical protein | um06479 | 0.46066 | ni | conserved hypothetical Ustilago-specific protein |
| 9c00276 | 0.5539 | ni | hypothetical protein | um11472 | 0.46074 | ni | putative protein |
| 8d00065 | 0.55405 | ni | hypothetical protein | um10114 | 0.46082 | K02960 | probable 40S ribosomal protein S16 |
| 14d00013 | 0.55432 | ni | hypothetical protein | um12095 | 0.46095 | ni | conserved hypothetical protein |
| 10d00109 | 0.55441 | ni | hypothetical protein | um05863 | 0.46114 | K12160 | related to SMT3 ubiquitin-like protein |
| 9c00186 | 0.55458 | K15201 | RNA polymerase III transcription factor TFIIIC | um00278 | 0.46118 | ni | hypothetical protein |
| 8c00005 | 0.555 | K14575 | AAA+-type ATPase | um02438 | 0.46123 | ni | related to clp1 |
| 22d00002 | 0.55522 | ni | hypothetical protein | um03681.2 | 0.46123 | ni | conserved hypothetical protein |
| 10d00016 | 0.55544 | K14859 | RNA-binding protein required for 60S ribosomal subunit biogenes | um10998 | 0.46149 | ni | putative protein |
| 27d00032 | 0.55547 | ni | karyopherin beta 3 | um05804 | 0.46155 | ni | Cys |
| 10d00113 | 0.55551 | ni | hypothetical protein | um06116 | 0.46172 | ni | putative protein |
| 8d00056 | 0.55554 | K01873 | valyl-trna synthetase | um11291 | 0.46181 | K03064 | probable RPT4 - 26S proteasome regulatory subunit |
| 9d00085 | 0.55562 | K15296 | protein required for fusion of vesicles in vesicular transport | um02829 | 0.46186 | ni | related to dTDP-glucose 4 |
| 8d00085 | 0.55641 | K01288 | serine carboxypeptidases | um05989 | 0.46202 | ni | related to 3-oxoadipate enol-lactone hydrolase |
| 7d00037 | 0.55642 | ni | serine racemase | um11361.2 | 0.46255 | ni | related to branched chain alpha-ketoacid dehydrogenase kinase |
| 9c00402 | 0.55669 | ni | hypothetical protein | um04203 | 0.46313 | ni | conserved hypothetical protein |
| 9d00263 | 0.5567 | ni | hypothetical protein | um05068 | 0.46322 | K01130 | probable Arylsulfatase |
| 11c00057 | 0.55694 | ni | predicted transporter | um01916 | 0.46356 | ni | conserved hypothetical protein |
| 19d00051 | 0.55703 | ni | hypothetical protein | um12307 | 0.46357 | K15424 | conserved hypothetical protein |
| 14d00032 | 0.55715 | ni | hypothetical protein | um10416 | 0.46368 | ni | conserved hypothetical protein |
| 22c00217 | 0.55716 | ni | regulatory protein MLP and related LIM proteins | um11436 | 0.46407 | K03521 | probable electron transfer flavoprotein beta chain |
| 22c00190 | 0.55727 | ni | predicted membrane protein | um05796 | 0.46408 | ni | conserved hypothetical protein |
| 22d00057 | 0.55727 | ni | uncharacterized conserved protein | um01102 | 0.46478 | ni | putative protein |
| 19c00004 | 0.55737 | ni | hypothetical protein | um05741 | 0.46488 | ni | conserved hypothetical protein |
| 11d00066 | 0.55767 | ni | hypothetical protein | um02622 | 0.46492 | K01560 | conserved hypothetical protein |
| 9c00263 | 0.55771 | ni | hypothetical protein | um03636 | 0.46501 | ni | conserved hypothetical Ustilago-specific protein |
| 7d00208 | 0.55794 | ni | FOG, PPR repeat | um05337 | 0.4658 | ni | related to SLA1 - cytoskeleton assembly control protein |
| 9c00152 | 0.55813 | ni | dihydrolipoamide acetyltransferase | um04066 | 0.46618 | ni | conserved hypothetical protein |
| 9c00331 | 0.55822 | ni | 5' nucleotidase | um00195 | 0.46629 | ni | putative protein |
| 7c00202 | 0.55835 | ni | hypothetical protein | um11352 | 0.4663 | K01441 | related to neutral ceramidase |
| 7d00109 | 0.55863 | ni | hypothetical protein | um05009 | 0.46678 | ni | hypothetical protein |
| 9d00010 | 0.55867 | ni | hypothetical protein | um11561 | 0.46683 | K02903 | related to 60S ribosomal protein L28 |
| 9c00390 | 0.55875 | ni | predicted transporter | um04503 | 0.467 | ni | related to Alpha-N-acetylgalactosaminidase precursor |
| 2d00051 | 0.55886 | ni | hypothetical protein | um04034 | 0.46732 | ni | conserved hypothetical Ustilago-specific protein |
| 22c00301 | 0.55897 | ni | hypothetical protein | um05501 | 0.46746 | ni | TPR-containing protein Mql1 |
| 19c00138 | 0.55905 | ni | hypothetical protein | um06241 | 0.46749 | ni | putative protein |
| 6d00114 | 0.55962 | K08770 | ubiquitin and ubiquitin-like proteins | um10255 | 0.4677 | ni | putative protein |
| 19c00121 | 0.55969 | K11584 | serine/threonine protein phosphatase 2A, regulatory subunit | um10704 | 0.4677 | ni | conserved hypothetical protein |
| 9d00129 | 0.55985 | K02437 | glycine cleavage system H protein | um01654 | 0.46783 | ni | conserved hypothetical protein |
| 6d00084 | 0.55986 | K00309 | proteins containing the FAD binding domain | um02927 | 0.46793 | ni | hypothetical protein |
| 16d00004 | 0.56049 | ni | hypothetical protein | um03456 | 0.46814 | ni | putative protein |
| 9d00257 | 0.56057 | ni | hypothetical protein | um10993 | 0.46822 | ni | conserved hypothetical protein |
| 15c00087 | 0.56064 | ni | hypothetical protein | um10549 | 0.46832 | K14840 | related to Glioma tumor suppressor candidate region gene 2 protein |
| 11c00070 | 0.56119 | ni | hypothetical protein | um00221 | 0.4686 | ni | conserved hypothetical protein |
| 16c00036 | 0.5614 | K11672 | actin-related protein - Arp5p | um06187 | 0.46869 | K06639 | related to CDC14 - dual specificity phosphatase |
| 12d00042 | 0.5619 | ni | hypothetical protein | um02378 | 0.46875 | K14007 | related to Protein transport protein Sec24C |
| 22c00264 | 0.56202 | ni | hypothetical protein | um00414 | 0.46891 | ni | conserved hypothetical protein |
| 7d00011 | 0.56206 | ni | hypothetical protein | um01202 | 0.46909 | ni | hypothetical protein |
| 15d00089 | 0.56212 | ni | animal-type fatty acid synthase and related proteins | um03439 | 0.46913 | ni | hypothetical Ustilago-specific protein |
| 9c00315 | 0.56232 | K00772 | methylthioadenosine phosphorylase MTAP | um05005 | 0.46919 | ni | related to 30S ribosomal protein S17 |
| 14d00014 | 0.56263 | ni | serine/threonine protein kinase | um10226 | 0.46924 | K06268 | probable CNB1 - calcineurin B |
| 14d00026 | 0.56264 | ni | hypothetical protein | um12327 | 0.46928 | K15376 | related to Gephyrin |
| 12d00076 | 0.56267 | ni | FOG, Zn-finger | um10822 | 0.46936 | K07556 | related to ATP12 - F1F0-ATPase complex assembly protein |
| 7d00344 | 0.56281 | ni | hypothetical protein | um03654 | 0.46962 | ni | related to poly |
| 7d00242 | 0.56283 | K15193 | hypothetical protein | um10102 | 0.46962 | ni | related to SLY1 protein |
| 9d00235 | 0.56294 | ni | hypothetical protein | um04294 | 0.46968 | K13352 | related to peroxin-11 |
| 7d00351 | 0.56308 | ni | hypothetical protein | um11916 | 0.46987 | ni | probable MRPL31 - mitochondrial ribosomal protein |
| 22c00050 | 0.56312 | ni | hypothetical protein | um10843 | 0.47001 | ni | putative protein |
| 2c00072 | 0.56357 | ni | hypothetical protein | um10369 | 0.47031 | K00568 | related to COQ3 - enzyme of ubiquinone |
| 12d00106 | 0.56377 | K10753 | histone chaperone | um02982 | 0.47058 | K05857 | related to PLC1 - 1-phosphatidylinositol-4 |
| 12c00063 | 0.56383 | K03660 | 8-oxoguanine DNA glycosylase | um01240 | 0.47063 | ni | conserved hypothetical Ustilago-specific protein |
| 18d00102 | 0.56388 | ni | uroporphyrinogen III synthase UROS/HEM4 | um10068 | 0.47102 | ni | related to Acid phosphatase precursor |
| 9c00296 | 0.56412 | ni | hypothetical protein | um04238 | 0.47107 | ni | conserved hypothetical protein |
| 7d00002 | 0.56445 | ni | serine/threonine protein phosphatase | um04672 | 0.47126 | K01098 | related to CTL1 - RNA 5 |
| 9c00216 | 0.56447 | ni | hypothetical protein | um11334 | 0.47127 | K00599 | related to RMT2 - protein-arginine N-methyltransferase |
| 12d00046 | 0.56478 | ni | peroxisomal NUDIX hydrolase | um01236 | 0.47128 | ni | conserved hypothetical Ustilago-specific protein |
| 15d00086 | 0.56495 | ni | hypothetical protein | um05389 | 0.47148 | ni | putative protein |
| 8c00025 | 0.56503 | ni | hypothetical protein | um11706 | 0.47197 | ni | putative protein |
| 18d00113 | 0.5651 | ni | acetylcholinesterase | um10100 | 0.47209 | K10734 | conserved hypothetical protein |
| 7d00159 | 0.56548 | ni | hypothetical protein | um11997 | 0.47222 | ni | probable ZPR1 - protein binds to translation elongation factor eEF-1 |
| 8d00108 | 0.56565 | K00641 | hypothetical protein | um06243 | 0.47227 | ni | conserved hypothetical protein |
| 19d00031 | 0.56567 | ni | hypothetical protein | um11225 | 0.47231 | K02895 | conserved hypothetical protein |
| 11c00089 | 0.5657 | ni | tatd-related DNase | um11743 | 0.47266 | K02260 | related to COX17 - Cytochrome c oxidase copper chaperone |
| 12c00091 | 0.56581 | K01626 | hypothetical protein | um11078 | 0.47287 | ni | conserved hypothetical protein |
| 12c00118 | 0.56588 | ni | hypothetical protein | um00671 | 0.47293 | ni | putative protein |
| 12c00028 | 0.56589 | K14538 | GTPase | um04040 | 0.47344 | ni | hypothetical protein |
| 5d00113 | 0.56591 | ni | hypothetical protein | um02402 | 0.47372 | ni | conserved hypothetical protein |
| 9d00267 | 0.56599 | K00286 | pyrroline-5-carboxylate reductase | um01761 | 0.47372 | ni | conserved hypothetical protein |
| 24c00064 | 0.56609 | ni | urea transporter | um02014 | 0.4738 | ni | conserved hypothetical protein |
| 5c00144 | 0.56611 | ni | hypothetical protein | um12239 | 0.47388 | K07478 | related to MGS1 - Maintenance of Genome Stability 1 |
| 15c00043 | 0.5662 | ni | hypothetical protein | um02535 | 0.47399 | ni | conserved hypothetical Ustilago-specific protein |
| 13c00031 | 0.56639 | ni | extracellular protein SEL-1 and related proteins | um12287 | 0.474 | ni | conserved hypothetical protein |
| 7c00225 | 0.56662 | K10684 | SMT3/SUMO-activating complex, AOS1/RAD31 component | um01465 | 0.47409 | ni | conserved hypothetical protein |
| 18d00045 | 0.56667 | K15076 | RNA polymerase II transcription elongation factor Elongin/SIII, su | um03744 | 0.47418 | ni | hypothetical protein |
| 19d00146 | 0.56687 | ni | hypothetical protein | um03363 | 0.47444 | K14544 | conserved hypothetical protein |
| 10c00026 | 0.56719 | ni | ras-related GTPase | um11628 | 0.4745 | ni | hypothetical protein |
| 7d00112 | 0.56748 | K14787 | RNA-binding protein | um11760.2 | 0.47468 | K03065 | probable RPT5 - 26S proteasome regulatory subunit |
| 5d00010 | 0.56766 | ni | hypothetical protein | um01285 | 0.47489 | ni | related to diadenosine hexaphosphate |
| 9d00367 | 0.56775 | ni | hypothetical protein | um12326 | 0.47512 | ni | conserved hypothetical protein |
| 9c00111 | 0.56801 | K08343 | protein involved in autophagocytosis during starvation | um02255 | 0.47534 | ni | related to phosphatidic acid-preferring phospholipase A1 |
| 2d00052 | 0.56866 | ni | hypothetical protein | um10441 | 0.47574 | K08513 | probable synaptobrevin |
| 19c00018 | 0.56882 | K04564 | manganese superoxide dismutase | um04517.2 | 0.47605 | ni | putative protein |
| 9c00417 | 0.56898 | ni | hypothetical protein | um01844 | 0.47651 | ni | hypothetical protein |
| 9d00070 | 0.56943 | K06130 | lysophospholipase | um01786 | 0.47652 | ni | putative protein |
| 10c00098 | 0.56957 | ni | molecular chaperone | um11440.2 | 0.47678 | ni | conserved hypothetical protein |
| 10c00104 | 0.5697 | ni | hypothetical protein | um10613 | 0.47706 | K08272 | conserved hypothetical protein |
| 4c00040 | 0.57008 | K11227 | mitogen-activated protein kinase kinase | um04639 | 0.47708 | ni | conserved hypothetical protein |
| 5d00148 | 0.57011 | ni | hypothetical protein | um10348 | 0.4771 | ni | conserved hypothetical protein |
| 8d00038 | 0.57022 | ni | hypothetical protein | um10182 | 0.47738 | K02924 | probable 60S ribosomal protein L39 |
| 7d00161 | 0.57025 | ni | FYVE finger-containing protein | um01178 | 0.47744 | K05727 | related to dedicator of cytokinesis protein 3 |
| 5c00065 | 0.57029 | ni | gluconate transport-inducing protein | um04909 | 0.47748 | K09467 | related to carbon repressor protein 1 |
| 9d00271 | 0.57032 | ni | guanine nucleotide exchange factor | um06210 | 0.47772 | ni | related to Arsenite-resistance protein 2 |
| 6d00087 | 0.57044 | ni | hypothetical protein | um10382 | 0.47777 | K17424 | related to MRPL51 - mitochondrial ribosomal protein |
| 14d00005 | 0.57058 | ni | hypothetical protein | um02830 | 0.47781 | ni | related to MSS51 - mRNA processing protein |
| 24c00036 | 0.57102 | ni | hypothetical protein | um10726 | 0.47791 | K11232 | related to YPD1 - two-component phosphorelay intermediate |
| 19d00082 | 0.57115 | ni | hypothetical protein | um03880 | 0.47839 | ni | conserved hypothetical protein |
| 5d00145 | 0.57173 | K17428 | mitochondrial/chloroplast ribosomal protein L4/L29 | um04093 | 0.47844 | ni | hypothetical protein |

| | | | | | | | |
|---|---|---|---|---|---|---|---|
| 12c00061 | 0.57194 | ni | hypothetical protein | um12152 | 0.47844 | ni | conserved hypothetical protein |
| 18d00036 | 0.57214 | ni | predicted seven transmembrane receptor - rhodopsin family | um04228 | 0.47859 | K08486 | related to SSO1 - syntaxin-related protein |
| 13d00094 | 0.57254 | ni | flavonol reductase | um03702 | 0.47867 | K12585 | probable DIS3 3 |
| 2c00061 | 0.57267 | ni | aspartyl protease | um04848 | 0.47884 | K01868 | probable THS1 - threonyl tRNA synthetase |
| 9c00368 | 0.57269 | K12391 | vesicle coat complex AP-1, gamma subunit | um02631 | 0.47887 | K06997 | conserved hypothetical protein |
| 26c00015 | 0.57277 | ni | uncharacterized conserved protein | um15086.2 | 0.47905 | ni | conserved hypothetical protein |
| 9c00387 | 0.57284 | ni | sec5 subunit of exocyst complex | um04674 | 0.47909 | ni | related to YBT1 - Vacuolar |
| 5c00099 | 0.57288 | ni | hypothetical protein | um10165 | 0.47926 | ni | related to Ngg1p-interacting factor 3 |
| 22d00020 | 0.57305 | K14403 | mRNA cleavage and polyadenylation factor II complex, BRR5 | um12215 | 0.47934 | ni | hypothetical protein |
| 1d00025 | 0.57336 | ni | hypothetical protein | um05383 | 0.47946 | ni | conserved hypothetical protein |
| 10d00106 | 0.57358 | ni | hypothetical protein | um04468 | 0.47955 | ni | related to SPA2 protein |
| 12c00129 | 0.57384 | K02264 | cytochrome c oxidase, subunit Va/COX6 | um00596 | 0.47981 | ni | hypothetical protein |
| 7c00194 | 0.57428 | ni | hypothetical protein | um10935 | 0.47982 | K12398 | related to AP-3 adapter complex mu3A subunit |
| 24c00010 | 0.57455 | ni | hypothetical protein | um05695 | 0.48013 | K03267 | probable SUP35 - eukaryotic peptide chain release factor GTP-binding subunit |
| 6d00034 | 0.57468 | ni | hypothetical protein | um00388 | 0.48017 | ni | hypothetical protein |
| 9d00213 | 0.57471 | ni | hypothetical protein | um05266 | 0.48029 | ni | ni |
| 15d00061 | 0.57488 | ni | hypothetical protein | um10548 | 0.48041 | K02127 | probable H -transporting two-sector ATPase chain b precursor |
| 3c00027 | 0.57508 | ni | hypothetical protein | um00930 | 0.48073 | ni | conserved hypothetical Ustilago-specific protein |
| 16c00084 | 0.57538 | K00052 | 3-isopropylmalate dehydrogenase | um03730.2 | 0.48081 | ni | putative protein |
| 6c00085 | 0.5757 | K06877 | predicted ATP-dependent RNA helicase | um03829.2 | 0.48102 | ni | hypothetical protein |
| 22c00259 | 0.57579 | ni | CCAAT-binding factor, subunit C | um03609 | 0.48108 | K14855 | probable RSA4 - WD-repeat protein involved in ribosome biogenesis |
| 5c00036 | 0.57585 | K01228 | glucosidase I | um05338 | 0.48118 | ni | related to transcription factor MBP1 |
| 7c00189 | 0.57587 | K10967 | glycolipid 2-alpha-mannosyltransferase | um10756 | 0.48147 | ni | conserved hypothetical protein |
| 18d00104 | 0.57603 | ni | permease of the drug/metabolite transporter | um11837 | 0.48192 | ni | conserved hypothetical protein |
| 26c00045 | 0.57624 | ni | hypothetical protein | um04196 | 0.48199 | K10424 | conserved hypothetical protein |
| 8c00113 | 0.57629 | ni | hypothetical protein | um05158 | 0.48223 | K14297 | related to Nucleoporin nup189 |
| 27c00013 | 0.57683 | ni | mitochondrial/chloroplast ribosomal protein L2 | um10963 | 0.4826 | K09008 | conserved hypothetical protein |
| 11c00011 | 0.57686 | K10597 | ubiquitin fusion degradation protein-2 | um04932 | 0.48266 | ni | related to ATG18 - Phosphatidylinositol 3 |
| 26c00063 | 0.57703 | ni | hypothetical protein | um11792 | 0.48278 | K07950 | conserved hypothetical protein |
| 9c00321 | 0.57706 | K02989 | ribosomal protein S7 | um01649 | 0.48333 | ni | conserved hypothetical protein |
| 3d00035 | 0.57747 | ni | hypothetical protein | um04908 | 0.48381 | K03245 | conserved hypothetical protein |
| 6c00120 | 0.57752 | K06067 | histone deacetylase complex, catalytic component RPD3 | um10410 | 0.48394 | K13179 | probable HAS1 - helicase associated with Set1p |
| 3c00048 | 0.57763 | ni | hypothetical protein | um00338 | 0.48414 | ni | conserved hypothetical protein |
| 1c00050 | 0.57769 | K00616 | transaldolase | um11239 | 0.48446 | ni | hypothetical protein |
| 18c00023 | 0.57781 | K00767 | quinolinate phosphoribosyl transferase | um05219 | 0.48458 | ni | related to endoplasmic oxidoreductin 1 precursor |
| 6d00133 | 0.57818 | ni | hypothetical protein | um10958 | 0.48502 | K17261 | related to kinetochore associated 2 |
| 19c00079 | 0.57825 | ni | acyl-coa-binding protein | um10233 | 0.48516 | ni | conserved hypothetical Ustilago-specific protein |
| 18c00021 | 0.57825 | K02911 | predicted membrane protein | um00752 | 0.48522 | ni | hypothetical protein |
| 1c00023 | 0.57846 | K02884 | mitochondrial/chloroplast ribosomal protein L19 | um00259 | 0.48531 | K07739 | probable ELP3 - subunit of elongator |
| 7c00173 | 0.57859 | ni | hypothetical protein | um15090 | 0.48545 | ni | conserved hypothetical protein |
| 22d00130 | 0.57861 | ni | dehydrogenase kinase | um12257 | 0.48551 | ni | hypothetical protein |
| 12c00074 | 0.57921 | ni | microtubule-binding protein | um10228.2 | 0.48582 | ni | hypothetical protein |
| 8d00026 | 0.57941 | ni | hypothetical protein | um12099 | 0.48593 | ni | conserved hypothetical protein |
| 22c00116 | 0.5795 | ni | hypothetical protein | um12008 | 0.48618 | K08874 | related to TRA1 - component of the Ada-Spt transcriptional regulatory complex |
| 12d00127 | 0.57962 | ni | hypothetical protein | um02011 | 0.48645 | ni | putative protein |
| 27d00042 | 0.57974 | ni | molecular chaperone | um02861 | 0.48683 | ni | conserved hypothetical protein |
| 14c00080 | 0.5798 | ni | hypothetical protein | um03966 | 0.48718 | ni | hypothetical protein |
| 2c00033 | 0.5802 | ni | endoplasmic reticulum protein EP58 | um02451 | 0.48736 | ni | conserved hypothetical protein |
| 13d00009 | 0.58023 | ni | hypothetical protein | um10867 | 0.48742 | ni | hypothetical protein |
| 13c00007 | 0.58045 | ni | signaling protein SWIFT and related BRCT domain proteins | um05726 | 0.48849 | K07178 | related to RIO Kinase 1 |
| 12d00050 | 0.58054 | K02883 | 60s ribosomal protein L18 | um12258 | 0.48856 | ni | hypothetical protein |
| 2d00035 | 0.58077 | K07466 | single-stranded DNA-binding replication protein Aproteins | um12017 | 0.48862 | ni | conserved hypothetical protein |
| 19c00140 | 0.5812 | ni | hypothetical protein | um04083 | 0.48867 | ni | conserved hypothetical protein |
| 5d00110 | 0.58126 | ni | hypothetical protein | um04896 | 0.48867 | ni | conserved hypothetical protein |
| 1d00046 | 0.58204 | K01114 | hypothetical protein | um06235 | 0.48889 | K09494 | probable CCT2 - chaperonin of the TCP1 ring complex |
| 6c00025 | 0.5828 | ni | hypothetical protein | um06178 | 0.48928 | ni | Mig2-1 |
| 6c00035 | 0.58286 | ni | hypothetical protein | um00964 | 0.48933 | K14537 | related to NOG2 - GTPase involved in ribosomal large subunit-nucleus export |
| 6c00037 | 0.58287 | ni | hypothetical protein | um11900 | 0.48939 | ni | putative protein |
| 6c00006 | 0.58292 | K01915 | glutamine synthetase | um03235 | 0.4897 | ni | conserved hypothetical protein |
| 24c00008 | 0.5831 | ni | hypothetical protein | um10700 | 0.48997 | K02908 | probable RPL30 - 60S large subunit ribosomal protein L30 |
| 10d00098 | 0.58311 | K00521 | ferric reductase | um10513 | 0.4901 | ni | putative protein |
| 26d00065 | 0.58334 | K10752 | nucleosome remodeling factor, subunit CAF1/NURF55/MSI1 | um03192 | 0.49023 | ni | conserved hypothetical protein |
| 7d00249 | 0.58388 | K15356 | nucleotide-sugar transporter VRG4/SQV-7 | um04383 | 0.49032 | ni | related to ERP2 - p24 protein involved in membrane trafficking |
| 27d00066 | 0.58475 | K02917 | 60S ribosomal protein L35A/L37 | um10846 | 0.49069 | ni | related to UGA2 - succinate semialdehyde dehydrogenase |
| 22d00238 | 0.58515 | ni | hypothetical protein | um10064 | 0.49091 | K00844 | probable glucokinase |
| 5d00082 | 0.58518 | K07078 | hypothetical protein | um03964 | 0.49099 | K12843 | related to PRP3 - essential splicing factor |
| 15d00079 | 0.58572 | ni | hypothetical protein | um05368 | 0.49101 | ni | putative protein |
| 7d00220 | 0.58585 | K03137 | transcription initiation factor IIE, beta subunit | um00443 | 0.49107 | K14960 | conserved hypothetical protein |
| 7d00166 | 0.58585 | ni | hypothetical protein | um06229 | 0.4913 | K14856 | related to SDA1 - required for normal organization of the actin cytoskeleton |
| 9c00341 | 0.58592 | ni | hypothetical protein | um11148 | 0.49172 | ni | related to MTG2 - Mitochondrial GTP binding protein |
| 7c00312 | 0.58639 | K03231 | translation elongation factor EF-1 alpha/Tu | um05563 | 0.4918 | K03869 | related to cullin 4A |
| 2d00022 | 0.58655 | K01907 | acyl-coa synthetase | um03626 | 0.4918 | ni | putative protein |
| 8d00080 | 0.58658 | ni | hypothetical protein | um00180 | 0.49213 | ni | hypothetical protein |
| 19c00019 | 0.58677 | ni | hypothetical protein | um06256 | 0.49215 | ni | hypothetical protein |
| 8d00060 | 0.58682 | ni | nuclear protein export factor | um02293 | 0.49222 | ni | hypothetical protein |
| 22c00142 | 0.58696 | ni | transporter, ABC superfamily | um05656 | 0.49244 | ni | related to SOK1 protein |
| 7c00339 | 0.58705 | K02929 | 60S ribosomal protein L44 | um04218 | 0.49289 | ni | Kinesin-1 motor protein |
| 22c00094 | 0.58717 | K14561 | U3 small nucleolar ribonucleoprotein | um05568 | 0.49303 | ni | probable SPT16 - general chromatin factor |
| 22c00078 | 0.58733 | ni | hypothetical protein | um10425 | 0.49326 | K02911 | conserved hypothetical protein |
| 9c00018 | 0.58736 | K07824 | cytochrome P450 CYP11/CYP12/CYP24/CYP27 subfamilies | um00823 | 0.49356 | ni | conserved hypothetical protein |
| 15c00063 | 0.58747 | ni | hypothetical protein | um01808 | 0.49372 | ni | related to ERV2 - Flavin dependent sulfhydryl oxidase |
| 20d00054 | 0.58769 | ni | uncharacterized conserved protein | um01463 | 0.49396 | K03237 | probable SUI2 - translation initiation factor eIF-2 alpha chain |
| 9d00102 | 0.58828 | K01673 | predicted carbonic anhydrase involved in protection against oxidat | um10328 | 0.49419 | ni | hypothetical protein |
| 10d00103 | 0.58836 | K10877 | DNA repair protein | um03936 | 0.49423 | K12818 | probable ATP dependent RNA helicase |
| 24d00024 | 0.58844 | K11294 | nuclear localization sequence binding protein | um01866 | 0.49424 | ni | conserved hypothetical protein |
| 19d00149 | 0.58879 | ni | hypothetical protein | um01242 | 0.49485 | K13431 | related to SRP101 - signal recognition particle receptor |
| 11d00067 | 0.5888 | ni | endoplasmic reticulum protein EP58 | um00654 | 0.49497 | K07943 | probable ARL1 - ADP-ribosylation factor |
| 22d00162 | 0.58907 | ni | uncharacterized conserved protein | um11568 | 0.49521 | K01144 | related to SWH1 - Member of an oxysterol-binding protein family |
| 7c00119 | 0.58933 | ni | hypothetical protein | um05208 | 0.49537 | K08734 | related to MLH1 - DNA mismatch repair protein |
| 22c00144 | 0.58944 | ni | predicted hydrolase | um04752 | 0.49553 | K12816 | probable CDC40 - Pre-mRNA splicing factor important for catalytic step II |
| 2c00029 | 0.58971 | K03511 | predicted DNA damage inducible protein | um02757 | 0.4957 | ni | conserved hypothetical protein |
| 13d00045 | 0.58974 | ni | hypothetical protein | um05306 | 0.49579 | ni | conserved hypothetical Ustilago-specific protein |
| 9d00403 | 0.58992 | ni | hypothetical protein | um10908 | 0.49601 | ni | probable OPT1 - High-affinity glutathione transporter |
| 8d00064 | 0.59 | K02213 | hypothetical protein | um01762 | 0.4961 | ni | related to large neutral amino acid transporter 1 |
| 11c00082 | 0.59007 | K00804 | geranylgeranyl pyrophosphate synthase | um03134 | 0.49614 | ni | conserved hypothetical protein |
| 2d00027 | 0.59046 | K02139 | hypothetical protein | um11092 | 0.49616 | K17080 | probable prohibitin PHB1 |
| 6c00004 | 0.59094 | ni | hypothetical protein | um03588 | 0.49645 | ni | related to transcription factor medusa |
| 6c00044 | 0.59098 | ni | hypothetical protein | um05055 | 0.49648 | ni | hypothetical protein |
| 13c00105 | 0.59164 | ni | hypothetical protein | um00956 | 0.49649 | ni | related to RAX1 - Protein involved in determination of budding patterns |
| 24d00020 | 0.59169 | ni | nuclear localization sequence binding protein | um05350 | 0.49674 | ni | hypothetical protein |
| 7d00213 | 0.59192 | ni | oxysterol-binding protein | um00394 | 0.4969 | ni | putative protein |
| 9c00235 | 0.59198 | ni | glucose dehydrogenase | um10928 | 0.49692 | ni | hypothetical protein |
| 156c00001 | 0.59203 | ni | ni | um03175 | 0.49703 | ni | putative protein |
| 8d00103 | 0.59215 | ni | hypothetical protein | um11211 | 0.49712 | ni | conserved hypothetical protein |
| 7c00141 | 0.59218 | K12864 | uncharacterized conserved protein | um06420 | 0.49721 | ni | conserved hypothetical protein |
| 13d00082 | 0.59232 | ni | hypothetical protein | um11716 | 0.4973 | K02971 | probable 40s ribosomal protein s21 |
| 7c00129 | 0.59245 | K08286 | p21-activated serine/threonine protein kinase | um00212 | 0.4974 | ni | conserved hypothetical protein |
| 22c00182 | 0.5926 | ni | hypothetical protein | um11443 | 0.4974 | ni | conserved hypothetical Ustilago-specific protein |
| 12c00035 | 0.59321 | ni | hypothetical protein | um02017 | 0.49769 | ni | ni |
| 6d00007 | 0.59352 | ni | hypothetical protein | um00927 | 0.49775 | ni | putative protein |
| 12d00057 | 0.5938 | K14766 | nucleolar protein | um05308 | 0.49796 | ni | hypothetical protein |
| 19c00020 | 0.59389 | ni | FOG, RRM domain | um06117 | 0.49806 | ni | ni |
| 9c00395 | 0.59392 | K02606 | origin recognition complex, subunit 4 | um00912 | 0.49806 | ni | conserved hypothetical protein |
| 6c00137 | 0.59409 | ni | hypothetical protein | um00851 | 0.49808 | ni | related to BFR1 - Nuclear segregation protein |
| 6d00010 | 0.59415 | K14845 | nuclear 5'-3' exoribonuclease-interacting protein | um12217 | 0.49808 | ni | hypothetical protein |
| 24c00017 | 0.59457 | ni | hypothetical protein | um05434 | 0.49815 | K14574 | probable Shwachman-Bodian-Diamond syndrome protein |
| 22c00219 | 0.59499 | ni | hypothetical protein | um06260 | 0.49887 | ni | conserved hypothetical protein |
| 22d00242 | 0.59503 | ni | membrane protein | um01772 | 0.49898 | K01785 | related to Aldose 1-epimerase precursor |
| 8d00088 | 0.59515 | ni | hypothetical protein | um03778 | 0.49908 | ni | hypothetical protein |
| 9c00061 | 0.59516 | ni | reductases with broad range of substrate specificities | um01833 | 0.49945 | ni | related to Zinc finger DHHC domain containing protein 2 |
| 24d00011 | 0.59525 | ni | translin-associated protein X | um01923 | 0.49946 | ni | conserved hypothetical protein |
| 24c00021 | 0.59571 | K09540 | molecular chaperone | um06211 | 0.49968 | K12882 | related to 80 kDa nuclear cap binding protein |
| 8c00049 | 0.59582 | K00611 | ornithine carbamoyltransferase OTC/ARG3 | um00784.2 | 0.49973 | K15190 | putative protein |
| 10c00039 | 0.59604 | ni | cell-cycle nuclear protein | um12214 | 0.50012 | ni | hypothetical protein |
| 22d00251 | 0.59613 | K09829 | sigma receptor and C-8 sterol isomerase | um11925 | 0.50018 | ni | related to VPS55 - protein involved in late endosome to vacuole trafficking |
| 16c00008 | 0.5964 | ni | hypothetical protein | um10295 | 0.50043 | ni | related to LYS2 - L-aminoadipate-semialdehyde dehydrogenase |
| 27c00064 | 0.59718 | ni | hypothetical protein | um01817 | 0.50059 | ni | conserved hypothetical protein |
| 1d00050 | 0.59723 | ni | transferrin receptor and related proteins | um02298 | 0.50074 | ni | hypothetical protein |
| 27c00044 | 0.59731 | ni | uncharacterized conserved protein | um05018 | 0.50077 | ni | related to ribosomal protein S4 precursor |
| 13d00058 | 0.59737 | ni | predicted RNA-binding protein | um10306 | 0.50093 | ni | conserved hypothetical protein |
| 14c00101 | 0.59783 | ni | glucose-6-phosphate/phosphate and phosphoenolpyruvate/phosp | um10424.2 | 0.50096 | K02911 | related to PTM1 - member of the major facilitator superfamily |
| 7c00193 | 0.59893 | ni | hypothetical protein | um04501 | 0.50104 | ni | ni |
| 13d00002 | 0.59894 | K14810 | RNA helicase | um10903 | 0.50113 | K03037 | probable RPN7 - subunit of the regulatory particle of the proteasome |
| 13c00069 | 0.59916 | K02332 | mitochondrial DNA polymerase gamma, catalytic subunit | um10586 | 0.50168 | K00600 | probable serine hydroxymethyltransferase |
| 22d00234 | 0.59948 | ni | uncharacterized conserved protein | um04490 | 0.50188 | ni | hypothetical protein |
| 7c00342 | 0.59984 | ni | ferredoxin | um04456 | 0.50241 | K04345 | protein kinase A |
| 26c00095 | 0.60034 | ni | hypothetical protein | um04897 | 0.50377 | ni | related to Endo-1 |
| 26c00053 | 0.60058 | ni | hypothetical protein | um04870 | 0.50382 | ni | related to RPC53 - 47 kD subunit of DNA-directed RNA polymerase III |
| 6d00043 | 0.60058 | ni | hypothetical protein | um00184 | 0.50407 | K14846 | related to RPF1 - involved in the assembly of the large ribosomal subunit |
| 5d00028 | 0.6006 | ni | predicted Yippee-type zinc-binding protein | um03569 | 0.50423 | ni | related to KRE6 - beta-1 |
| 9d00144 | 0.60078 | K14847 | protein required for biogenesis of the ribosomal 60S subunit | um04832 | 0.50452 | ni | putative protein |
| 6d00090 | 0.60087 | ni | hypothetical protein | um00469 | 0.50465 | K11251 | probable histone H2A F |
| 25d00060 | 0.60089 | K07178 | serine/threonine protein kinase | um01628.2 | 0.50479 | K08504 | conserved hypothetical protein |
| 18c00063 | 0.60109 | ni | cytochrome P450 CYP3/CYP5/CYP6/CYP9 subfamilies | um03549 | 0.50499 | K14327 | related to NMD2 - Nonsense-mediated mRNA decay protein 2 |
| 19c00103 | 0.60128 | ni | hypothetical protein | um01018 | 0.50511 | K03768 | probable cyclophilin b |
| 22c00124 | 0.60149 | K06699 | uncharacterized conserved protein | um02868 | 0.50514 | K12852 | probable ribosomal elongation factor EF-2 |
| 22c00175 | 0.60158 | ni | hypothetical protein | um00358 | 0.50522 | ni | hypothetical protein |
| 9d00386 | 0.60224 | K08818 | protein kinase PITSLRE and related kinases | um05707 | 0.50552 | ni | hypothetical protein |
| 7d00170 | 0.60236 | ni | histone acetyltransferase | um04980 | 0.50576 | ni | putative protein |
| 6d00116 | 0.60236 | ni | hypothetical protein | um04260 | 0.50578 | K14549 | conserved hypothetical protein |
| 7c00041 | 0.6024 | ni | predicted Zn-dependent hydrolase | um01687 | 0.50589 | K06944 | probable GTP-binding protein |
| 9d00036 | 0.60361 | K14839 | nucleolar protein | um06507 | 0.50597 | ni | conserved hypothetical protein |
| 14d00018 | 0.6047 | K06174 | RNAse L inhibitor | um01648.2 | 0.50599 | ni | putative protein |
| 5d00109 | 0.60474 | ni | hypothetical protein | um12341 | 0.50607 | ni | related to Cytochrome P450 |
| 5d00057 | 0.60555 | K12869 | cell cycle control protein | um06292 | 0.50608 | ni | probable PEP8 - vacuolar protein sorting |
| 22c00022 | 0.60584 | ni | hypothetical protein | um03928 | 0.50661 | K00924 | related to serine |

| | | | | | | | |
|---|---|---|---|---|---|---|---|
| 10c00011 | 0.6063 | ni | hypothetical protein | um01608 | 0.50673 | K14775 | conserved hypothetical protein |
| 11d00021 | 0.60632 | ni | multiple inositol polyphosphate phosphatase | um04541 | 0.50702 | ni | conserved hypothetical protein |
| 7d00113 | 0.60648 | K09498 | chaperonin complex component, TCP-1 zeta subunit | um11418 | 0.50721 | ni | related to VMA21 - ATPase assembly integral membrane protein |
| 4c00016 | 0.60654 | ni | predicted G-protein coupled receptor | um05237 | 0.50751 | ni | putative protein |
| 18d00088 | 0.60666 | ni | predicted hydrolases or acyltransferases | um02736 | 0.5077 | ni | conserved hypothetical protein |
| 14d00071 | 0.60676 | K06636 | structural maintenance of chromosome protein 1 | um01645 | 0.50793 | K12199 | conserved hypothetical protein |
| 5c00160 | 0.60718 | ni | uncharacterized conserved protein | um03695 | 0.50794 | K14570 | related to exonuclease GOR |
| 8c00015 | 0.60738 | ni | hypothetical protein | um00702 | 0.50826 | ni | putative protein |
| 7c00157 | 0.60764 | K08869 | predicted unusual protein kinase | um04950 | 0.50841 | K15562 | related to cyclin dependent kinase C |
| 14c00086 | 0.60769 | ni | hypothetical protein | um01862 | 0.50872 | K03325 | related to arsenite transporter ARR3 |
| 9d00284 | 0.6077 | ni | hypothetical protein | um11551 | 0.50881 | K02966 | probable RPS19B - ribosomal protein S19 |
| 18d00065 | 0.6079 | ni | hypothetical protein | um03650 | 0.50953 | ni | hypothetical protein |
| 8c00028 | 0.60797 | ni | hypothetical protein | um06233 | 0.50958 | K14564 | probable SIK1 - involved in pre-rRNA processing |
| 9c00316 | 0.60817 | ni | predicted GTP-binding protein MMR1 | um00432 | 0.50963 | K01259 | probable proline iminopeptidase |
| 18c00037 | 0.60875 | K02876 | mitochondrial/chloroplast ribosomal protein L15/L10 | um01530 | 0.50965 | K01889 | probable FRS2 - phenylalanine--tRNA ligase beta chain |
| 18d00097 | 0.6093 | K08286 | checkpoint kinase | um10175 | 0.51003 | ni | probable sphingosine-1-phosphate lyase |
| 6c00001 | 0.60959 | ni | hypothetical protein | um00882 | 0.51023 | K06018 | related to PPN1 - vacuolar endopolyphosphatase |
| 14d00041 | 0.6097 | K15458 | SAP family cell cycle dependent phosphatase-associated protein | um00652 | 0.51049 | ni | related to RNA helicase |
| 12d00111 | 0.60975 | K15427 | serine/threonine specific protein phosphatase | um11871 | 0.51059 | K03360 | related to GRR1 - required for glucose repression and for glucose and cation transport |
| 16c00060 | 0.60977 | ni | hypothetical protein | um11652 | 0.51128 | K10733 | related to PSF2 - part of GINS |
| 9c00413 | 0.60982 | ni | hypothetical protein | um10374 | 0.51145 | ni | related to TIM11 - subunit E of the dimeric form of mitochondrial F1F0-ATPase |
| 3c00054 | 0.60993 | ni | hypothetical protein | um03655 | 0.51158 | ni | conserved hypothetical protein |
| 15c00021 | 0.61035 | ni | hypothetical protein | um03862 | 0.51174 | K12182 | related to VPS27 - vacuolar protein sorting-associated protein |
| 22c00198 | 0.61052 | K09584 | thioredoxin/protein disulfide isomerase | um10739 | 0.51183 | ni | hypothetical protein |
| 7c00064 | 0.61109 | K14004 | vesicle coat complex COPII, subunit SEC13 | um10438 | 0.51215 | ni | conserved hypothetical protein |
| 3c00026 | 0.61134 | K15562 | cdc2-related protein kinase | um03629 | 0.51216 | ni | related to Signal peptide peptidase |
| 9d00046 | 0.61273 | K14709 | Fe2+/Zn2+ regulated transporter | um04167 | 0.51219 | K02149 | probable vacuolar ATP synthase subunit D |
| 13c00106 | 0.61276 | K07975 | ras-related small GTPase | um00857 | 0.51242 | ni | probable KRE6 - glucan synthase subunit |
| 9c00300 | 0.61287 | ni | hypothetical protein | um12260 | 0.51244 | ni | putative protein |
| 9c00068 | 0.61343 | ni | hypothetical protein | um06058 | 0.51253 | ni | conserved hypothetical protein |
| 26c00064 | 0.61346 | ni | Ca2+-binding actin-bundling protein | um10314 | 0.51261 | ni | related to ARL1 - ADP-ribosylation factor |
| 27c00009 | 0.61368 | ni | hypothetical protein | um02910 | 0.51272 | ni | conserved hypothetical protein |
| 7d00268 | 0.61395 | K03754 | translation initiation factor 2B, beta subunit | um01717 | 0.51279 | ni | conserved hypothetical protein |
| 9c00041 | 0.61446 | ni | permease of the major facilitator superfamily | um00909 | 0.51291 | ni | conserved hypothetical protein |
| 26c00062 | 0.6156 | ni | hypothetical protein | um00250 | 0.51314 | ni | conserved hypothetical protein |
| 7c00156 | 0.61569 | ni | hypothetical protein | um10220 | 0.51357 | ni | conserved hypothetical protein |
| 22c00052 | 0.61579 | ni | diamine acetyltransferase | um04390 | 0.51363 | ni | conserved hypothetical protein |
| 6c00046 | 0.61601 | ni | hypothetical protein | um04100 | 0.51377 | ni | hypothetical protein |
| 24d00023 | 0.61602 | ni | D-3-phosphoglycerate dehydrogenase | um01938 | 0.51404 | ni | related to Epoxide hydrolase 1 |
| 19c00052 | 0.61638 | ni | hypothetical protein | um10805 | 0.51412 | K11099 | probable SMX2 - snRNP G protein |
| 9d00319 | 0.61645 | K02953 | 40S ribosomal protein S13 | um12062 | 0.51506 | K10949 | probable ERD2 - ER lumen protein-retaining receptor |
| 9c00117 | 0.61665 | K15441 | hypothetical protein | um10270 | 0.51511 | ni | related to aspartate-tRNA ligase |
| 12d00129 | 0.61721 | ni | hypothetical protein | um01197 | 0.51526 | K00652 | related to 8-amino-7-oxononanoate synthase |
| 22c00081 | 0.61754 | K01735 | pentafunctional AROM protein | um11420 | 0.5155 | ni | conserved hypothetical protein |
| 1c00040 | 0.61791 | ni | hypothetical protein | um03652 | 0.51556 | ni | conserved hypothetical protein |
| 27c00071 | 0.61824 | ni | hypothetical protein | um01803 | 0.51556 | ni | related to NCA2 - control of mitochondrial synthesis of Atp6p and Atp8p |
| 12c00122 | 0.61857 | ni | hypothetical protein | um11701 | 0.51564 | ni | related to NUP57 - nuclear pore protein |
| 22c00005 | 0.61881 | ni | hypothetical protein | um01180 | 0.51572 | K03097 | probable protein kinase CK2 alpha subunit |
| 9c00356 | 0.61883 | ni | hypothetical protein | um01479 | 0.51574 | ni | putative protein |
| 9c00369 | 0.6193 | ni | hypothetical protein | um05557 | 0.51575 | K11756 | related to RSC2 - member of RSC complex |
| 9d00090 | 0.61946 | K14846 | ribosome biogenesis protein RPF1 | um04905 | 0.51578 | ni | conserved hypothetical protein |
| 12c00087 | 0.6195 | ni | hypothetical protein | um03843 | 0.5158 | ni | putative protein |
| 5c00091 | 0.61964 | ni | hypothetical protein | um01301 | 0.51586 | ni | conserved hypothetical Ustilago-specific protein |
| 6d00128 | 0.62009 | ni | hypothetical protein | um10448 | 0.51604 | K12393 | probable clathrin assembly protein AP47 |
| 9d00075 | 0.62026 | K11563 | hypothetical protein | um11075 | 0.51622 | ni | hypothetical protein |
| 9c00060 | 0.62055 | ni | hypothetical protein | um10609 | 0.5164 | K15100 | probable CTP1 - Mitochondrial citrate transporter - member of the mitochondrial carrier |
| 20d00003 | 0.62074 | ni | hypothetical protein | um10735 | 0.51641 | K00166 | probable 2-oxoisovalerate dehydrogenase alpha subunit |
| 19c00039 | 0.62091 | ni | hypothetical protein | um04460 | 0.51645 | K03016 | related to RPB8 - DNA-directed RNA polymerase I |
| 19d00037 | 0.62094 | ni | hypothetical protein | um01047 | 0.517 | ni | hypothetical protein |
| 7d00269 | 0.621 | ni | hypothetical protein | um06032 | 0.51702 | ni | conserved hypothetical protein |
| 2d00067 | 0.62114 | ni | muskelin | um11104 | 0.5171 | K00362 | putative nitrite reductase |
| 18d00053 | 0.62118 | K15745 | phytoene desaturase | um10408.2 | 0.51747 | K02876 | related to MRPL10 - mitochondrial ribosomal protein |
| 2d00033 | 0.6215 | K06634 | cdk activating kinase | um12117 | 0.5176 | ni | putative protein |
| 7d00033 | 0.62171 | ni | putative phosphoinositide phosphatase | um01763 | 0.51786 | ni | putative protein |
| 6d00072 | 0.62185 | ni | arylacetamide deacetylase | um10793 | 0.51798 | K12881 | conserved hypothetical protein |
| 8d00093 | 0.62207 | K14535 | predicted nucleotide kinase/nuclear protein | um02852 | 0.51809 | ni | conserved hypothetical Ustilago-specific protein |
| 22d00001 | 0.62262 | K01181 | hypothetical protein | um02229 | 0.5184 | ni | conserved hypothetical Ustilago-specific protein |
| 9d00135 | 0.6227 | ni | putative lipase essential for disintegration of autophagic bodies in: | um06425 | 0.51845 | ni | conserved hypothetical protein |
| 7d00231 | 0.62274 | ni | hypothetical protein | um11601 | 0.51882 | ni | conserved hypothetical protein |
| 9c00151 | 0.62274 | ni | FOG, Zn-finger | um05824 | 0.51953 | ni | hypothetical protein |
| 5d00114 | 0.6237 | ni | transmembrane protein | um03839 | 0.51956 | K01942 | related to BPL1 - biotin holocarboxylase synthetase |
| 3c00044 | 0.62373 | K15084 | mitochondrial solute carrier protein | um05405 | 0.51981 | K17260 | probable actin-like protein ACT2 |
| 10c00019 | 0.62384 | ni | hypothetical protein | um10040.2 | 0.51987 | ni | conserved hypothetical protein |
| 22d00004 | 0.62452 | ni | hypothetical protein | um06180 | 0.52009 | ni | Mig2-4 |
| 7d00339 | 0.62481 | K04798 | prefoldin subunit 6 | um05706 | 0.52014 | ni | conserved hypothetical protein |
| 3c00015 | 0.62509 | ni | hypothetical protein | um12247 | 0.52021 | ni | conserved hypothetical protein |
| 27d00008 | 0.62515 | ni | sensory transduction histidine kinase | um01778 | 0.52037 | ni | putative protein |
| 9d00057 | 0.62517 | K07243 | hypothetical protein | um01886 | 0.52056 | K13289 | related to carboxypeptidase |
| 10c00052 | 0.62607 | K02220 | cyclin B and related kinase-activating proteins | um10629.2 | 0.52062 | ni | conserved hypothetical protein |
| 5d00011 | 0.62614 | ni | inorganic phosphate transporter | um04721 | 0.52151 | K09650 | conserved hypothetical protein |
| 16d00021 | 0.62634 | K03141 | RNA polymerase II transcription initiation/nucleotide excision repa | um11165 | 0.52159 | ni | conserved hypothetical protein |
| 22c00127 | 0.62691 | ni | hypothetical protein | um04487.2 | 0.52163 | ni | conserved hypothetical protein |
| 7c00011 | 0.62693 | ni | hypothetical protein | um05737 | 0.52168 | ni | conserved hypothetical protein |
| 27d00051 | 0.62717 | K12840 | hypothetical protein | um02600 | 0.52196 | K01412 | probable mitochondrial processing peptidase alpha chain precursor |
| 18c00002 | 0.62767 | K03253 | translation initiation factor 3, subunit b | um05888 | 0.52213 | K03145 | related to transcription elongation factor TFIIS |
| 22d00092 | 0.62777 | ni | hypothetical protein | um05589 | 0.52274 | ni | hypothetical protein |
| 14d00069 | 0.62809 | ni | tomosyn and related SNARE-interacting proteins | um02881 | 0.5229 | K14407 | related to Cleavage stimulation factor |
| 22c00025 | 0.62813 | K13124 | conserved WD40 repeat-containing protein | um11553 | 0.52297 | K06679 | related to spindle assembly checkpoint protein |
| 27c00010 | 0.62885 | K02433 | amidases | um04129 | 0.52335 | ni | related to proliferation associated SNF2-like protein |
| 27d00087 | 0.62966 | K03019 | RNA polymerase III subunit C11 | um11141 | 0.52349 | K00861 | related to FMN1 - Riboflavin kinase |
| 19d00104 | 0.62972 | ni | hypothetical protein | um01514 | 0.52376 | K04368 | dual specificity protein kinase Fuz7 |
| 15c00026 | 0.62998 | ni | hypothetical protein | um10272 | 0.5239 | ni | putative protein |
| 1c00049 | 0.63053 | ni | multicopper oxidases | um12092 | 0.52416 | ni | hypothetical protein |
| 6d00091 | 0.63128 | ni | hypothetical protein | um11259 | 0.52453 | K02929 | probable RPL42B - ribosomal protein L36a |
| 7c00124 | 0.63154 | K01613 | RNA helicase nonsense mRNA reducing factor | um05417 | 0.52467 | K03361 | related to F-box |
| 5c00165 | 0.63182 | ni | hypothetical protein | um12129.2 | 0.5247 | K17292 | related to Tubulin-specific chaperone A |
| 9d00133 | 0.632 | ni | hypothetical protein | um11316 | 0.5248 | ni | conserved hypothetical protein |
| 19c00133 | 0.6323 | ni | hypothetical protein | um12233 | 0.52487 | ni | conserved hypothetical protein |
| 9c00314 | 0.63255 | K11675 | hypothetical protein | um01690 | 0.52541 | ni | hypothetical protein |
| 6d00039 | 0.63294 | ni | hypothetical protein | um02116 | 0.52565 | ni | hypothetical protein |
| 15d00033 | 0.63301 | ni | hypothetical protein | um04749 | 0.52578 | ni | conserved hypothetical protein |
| 14d00090 | 0.63351 | K00820 | glucosamine 6-phosphate synthetases | um04324 | 0.5258 | K00232 | probable POX1 - acyl-CoA oxidase |
| 9c00043 | 0.63369 | ni | hypothetical protein | um10708 | 0.52602 | K01653 | related to ILV6 - acetolactate synthase |
| 5c00114 | 0.63398 | K02153 | hypothetical protein | um02387 | 0.52625 | ni | related to oligopeptide transporter |
| 3c00030 | 0.63399 | ni | glucose dehydrogenase | um10667 | 0.52636 | ni | related to SPN1 - Spt6-interacting putative elongation factor |
| 7d00287 | 0.63422 | ni | transcription-coupled repair protein CSB/RAD26 | um11647 | 0.52646 | ni | hypothetical protein |
| 18d00072 | 0.63496 | K02734 | 20S proteasome, regulatory subunit beta type PSMB2/PRE1 | um05651 | 0.52672 | K14788 | conserved hypothetical protein |
| 15d00018 | 0.63504 | ni | phospholipase A2-activating protein | um04748 | 0.52683 | ni | putative protein |
| 15c00011 | 0.63558 | ni | hypothetical protein | um01733 | 0.52684 | ni | related to short chain dehydrogenase |
| 11c00064 | 0.63583 | ni | hypothetical protein | um02421 | 0.52687 | K13339 | related to PEX6 - peroxisomal assembly protein |
| 9c00192 | 0.63588 | K16261 | amino acid transporters | um11801 | 0.52701 | K02914 | related to 50S ribosomal protein L34 |
| 24d00056 | 0.63595 | ni | uncharacterized conserved protein | um11012 | 0.52709 | ni | conserved hypothetical protein |
| 19d00016 | 0.63601 | ni | hypothetical protein | um04148 | 0.52714 | ni | related to MRPS28 - mitochondrial ribosomal protein |
| 24c00034 | 0.6361 | ni | hypothetical protein | um05709 | 0.5274 | ni | probable GPI8 - GPI-anchor transamidase |
| 8d00079 | 0.63623 | ni | hypothetical protein | um11745 | 0.52762 | K02332 | related to MIP1 - DNA-directed DNA polymerase gamma catalytic subunit |
| 19c00057 | 0.63623 | ni | hypothetical protein | um05598 | 0.52811 | K03952 | probable NADH dehydrogenase |
| 14c00008 | 0.63642 | ni | hypothetical protein | um03147 | 0.52814 | K12396 | related to Adapter-related protein complex 3 delta 1 subunit |
| 10d00095 | 0.63649 | ni | FOG, Zn-finger | um00187 | 0.52819 | ni | hypothetical protein |
| 14d00073 | 0.63672 | ni | hypothetical protein | um11439 | 0.5282 | K12493 | related to GLO3 - zinc finger protein |
| 9d00299 | 0.6372 | ni | conserved phosducin-like protein | um10168 | 0.52835 | ni | conserved hypothetical protein |
| 9c00056 | 0.63732 | ni | oxoprolinase | um10063 | 0.52861 | K15430 | related to TRM11 - Catalytic subunit of an adoMet-dependent tRNA methyltransferase complex |
| 22d00268 | 0.63754 | ni | hypothetical protein | um03898 | 0.52872 | K10846 | related to RAD2 - structure-specific nuclease of the nucleotide excision repairosome |
| 18d00050 | 0.63764 | K14416 | elongation factor 1 alpha | um02094 | 0.52884 | ni | putative protein |
| 22c00235 | 0.63766 | ni | hypothetical protein | um02751 | 0.5289 | ni | hypothetical protein |
| 15d00077 | 0.63783 | ni | hypothetical protein | um10776 | 0.52901 | K03680 | related to GCD2 - translation initiation factor eIF2B |
| 5d00085 | 0.63796 | K11838 | ubiquitin carboxyl-terminal hydrolase | um02107 | 0.52905 | ni | conserved hypothetical protein |
| 19d00164 | 0.63818 | ni | predicted Zn-dependent hydrolase | um00169 | 0.52933 | ni | related to AUT1 - essential for autophagocytosis |
| 15c00022 | 0.63848 | ni | synaptic vesicle transporter SV2 | um01929 | 0.52946 | K08501 | conserved hypothetical protein |
| 1d00030 | 0.63887 | ni | hypothetical protein | um10970 | 0.52989 | ni | related to Sno-type pyridoxine vitamin B6 biosynthetic protein SNO1 |
| 22c00089 | 0.63973 | ni | hypothetical protein | um02553 | 0.53026 | ni | related to MRPS5 - mitochondrial ribosomal protein |
| 10c00089 | 0.64025 | ni | hypothetical protein | um05331 | 0.53053 | ni | conserved hypothetical protein |
| 12d00055 | 0.64041 | ni | proteins containing the FAD binding domain | um10171 | 0.53064 | ni | hypothetical protein |
| 10c00074 | 0.64044 | K04706 | Zn-finger transcription factor | um00224 | 0.53101 | K07734 | conserved hypothetical protein |
| 5d00104 | 0.64064 | K00295 | methylenetetrahydrofolate dehydrogenase | um05356 | 0.53122 | ni | hypothetical protein |
| 12d00126 | 0.64069 | ni | hypothetical protein | um03171.2 | 0.53127 | K03239 | related to GCN3 - translation initiation factor eIF2B |
| 7d00124 | 0.6413 | ni | hypothetical protein | um02697 | 0.53134 | K03106 | probable SRP54 - signal recognition particle subunit |
| 13c00066 | 0.6414 | K02260 | hypothetical protein | um11460 | 0.5314 | K05841 | UDP-glucose |
| 16d00023 | 0.64183 | K03257 | translation initiation factor 4F, helicase subunit | um10750 | 0.532 | ni | conserved hypothetical protein |
| 14c00088 | 0.64192 | ni | hypothetical protein | um02445 | 0.5321 | K15427 | probable serine |
| 22d00117 | 0.6422 | ni | hypothetical protein | um06134 | 0.53242 | K02948 | related to ribosomal protein YmS18 |
| 9c00171 | 0.64221 | K01657 | isochorismate synthase | um00769 | 0.53253 | ni | conserved hypothetical protein |
| 14c00085 | 0.64243 | ni | hypothetical protein | um04619 | 0.53276 | K04705 | related to HSE1 - protein binds ubiquitin and mediates endosomal protein sorting |
| 5c00050 | 0.64274 | K02971 | 40S ribosomal protein S21 | um10984 | 0.53285 | ni | conserved hypothetical protein |
| 16d00053 | 0.64429 | K11427 | putative protein methyltransferase | um01551 | 0.53291 | ni | probable BUD23 - Protein involved in bud-site selection |
| 10c00088 | 0.64446 | K03123 | transcription initiation factor IIA, gamma subunit | um11482 | 0.53292 | K10579 | probable Ubiquitin-conjugating enzyme E2 |
| 22c00019 | 0.64518 | ni | hypothetical protein | um01333 | 0.53299 | ni | conserved hypothetical protein |
| 11c00036 | 0.64534 | ni | aldehyde dehydrogenase | um06153 | 0.53303 | ni | related to 4-coumarate--CoA ligase 1 |
| 11d00042 | 0.64744 | ni | hypothetical protein | um04778 | 0.53326 | ni | conserved hypothetical protein |
| 24c00002 | 0.64799 | ni | hypothetical protein | um15089 | 0.53332 | ni | conserved hypothetical protein |
| 14d00072 | 0.6489 | K06961 | rRNA processing protein | um11524 | 0.5334 | ni | conserved hypothetical protein |
| 12d00008 | 0.64921 | ni | uncharacterized conserved protein | um04250 | 0.53384 | ni | related to AKR1 - ankyrin repeat-containing protein |
| 9d00381 | 0.64948 | ni | hypothetical protein | um01980 | 0.53392 | ni | related to Cytochrome P450 4F8 |
| 7c00301 | 0.64955 | K09486 | molecular chaperones GRP170/SIL1 | um03938 | 0.53393 | ni | related to SBA1 - Hsp90 associated co-chaperone |
| 18c00090 | 0.65044 | ni | apoptosis inhibitor IAP1 and related BIR domain proteins | um10538 | 0.53463 | ni | conserved hypothetical protein |
| 26d00040 | 0.65074 | ni | hypothetical protein | um05552 | 0.53471 | ni | conserved hypothetical protein |
| 3d00046 | 0.65109 | ni | uncharacterized conserved protein | um03449 | 0.53484 | K16945 | septin 3 |

| | | | | | | | |
|---|---|---|---|---|---|---|---|
| 7c00352 | 0.65121 | ni | hypothetical protein | um01897 | 0.53502 | ni | conserved hypothetical protein |
| 9c00301 | 0.65204 | K11429 | histone tail methylase | um00923 | 0.53509 | K04523 | related to DSK2 - ubiquitin-like protein |
| 9c00231 | 0.6527 | ni | hypothetical protein | um05467 | 0.53519 | ni | conserved hypothetical protein |
| 8d00009 | 0.65309 | ni | hypothetical protein | um03128 | 0.53532 | ni | conserved hypothetical protein |
| 5c00067 | 0.65319 | K11422 | histone H3 (Lys4) methyltransferase complex, subunit SET1 | um02604 | 0.53533 | ni | hypothetical protein |
| 15c00091 | 0.65319 | K05863 | mitochondrial ADP/ATP carrier proteins | um11690 | 0.53606 | ni | conserved hypothetical protein |
| 16d00049 | 0.65322 | ni | hypothetical protein | um04576 | 0.53628 | ni | conserved hypothetical protein |
| 19d00038 | 0.65329 | ni | hypothetical protein | um02266 | 0.5363 | K00981 | related to CDS1 - CDP-diacylglycerol synthase |
| 26c00049 | 0.65464 | ni | hypothetical protein | um03867 | 0.53661 | K07555 | related to ATP11 - F1F0-ATPase complex assembly protein |
| 8c00126 | 0.65472 | ni | hypothetical protein | um04380 | 0.53721 | ni | related to ATG9 - integral membrane protein required for Cvt and autophagy transport |
| 12c00071 | 0.65504 | ni | hypothetical protein | um10511 | 0.53757 | K03013 | conserved hypothetical protein |
| 14c00135 | 0.65561 | ni | hypothetical protein | um06435 | 0.5376 | ni | ni |
| 12d00107 | 0.65625 | ni | hypothetical protein | um03344 | 0.53788 | ni | conserved hypothetical protein |
| 22c00131 | 0.65669 | ni | hypothetical protein | um06179 | 0.53808 | ni | Mig2-2 |
| 19c00106 | 0.65688 | ni | hypothetical protein | um04656.2 | 0.53817 | ni | related to Rho-GTPase-activating protein 1 |
| 7d00036 | 0.65746 | K12160 | ubiquitin-like proteins | um11991 | 0.53828 | ni | conserved hypothetical protein |
| 12c00109 | 0.65782 | K05545 | tRNA-dihydrouridine synthase | um00779 | 0.53855 | ni | related to mitochondrial ribosomal protein YmL8 |
| 4d00009 | 0.65793 | ni | hypothetical protein | um01201.2 | 0.53884 | ni | conserved hypothetical protein |
| 7d00324 | 0.65821 | ni | hypothetical protein | um11756 | 0.53978 | ni | hypothetical protein |
| 3d00097 | 0.65926 | K12194 | protein involved in glucose derepression and pre-vacuolar endosc | um05895 | 0.53981 | K15440 | related to tRNA-specific adenosine deaminase 1 |
| 12d00124 | 0.66025 | ni | hypothetical protein | um00609 | 0.53991 | ni | conserved hypothetical protein |
| 9d00290 | 0.66062 | ni | F-box protein containing LRR | um03434 | 0.53999 | K10754 | related to replication factor C protein |
| 19d00100 | 0.66068 | ni | hypothetical protein | um04951 | 0.54008 | K12819 | related to step II splicing factor SLU7 |
| 22d00097 | 0.66116 | ni | vesicle coat complex COPII, subunit SEC23 | um03062 | 0.5406 | K14559 | related to MPP10 - component of the U3 small nucleolar ribonucleoprotein |
| 22c00044 | 0.66175 | K11718 | UDP-glucose, glycoprotein glucosyltransferase | um03207 | 0.54061 | K13348 | related to glomerulosclerosis protein Mpv17 |
| 9c00433 | 0.66177 | K16315 | serine/threonine kinase | um11884.2 | 0.54081 | K09539 | related to Mitochondrial DnaJ chaperone |
| 14c00057 | 0.66216 | ni | kelch repeat-containing proteins | um06226 | 0.54133 | ni | conserved hypothetical protein |
| 19d00115 | 0.66283 | K11849 | ubiquitin carboxyl-terminal hydrolase | um04505 | 0.5414 | ni | related to glutamyl-tRNA |
| 22c00275 | 0.66362 | ni | acid sphingomyelinase and PHM5 phosphate metabolism protein | um01544 | 0.54165 | K11230 | related to SSK2 - MAP kinase kinase kinase of the high osmolarity signal transduction pathway |
| 7c00015 | 0.66476 | ni | hypothetical protein | um04187 | 0.54171 | ni | conserved hypothetical protein |
| 13d00022 | 0.66493 | ni | hypothetical protein | um01481 | 0.542 | ni | hypothetical protein |
| 19c00118 | 0.66667 | ni | hypothetical protein | um01392 | 0.54227 | ni | conserved hypothetical Ustilago-specific protein |
| 12d00083 | 0.66733 | ni | hypothetical protein | um00615 | 0.54261 | K14566 | conserved hypothetical protein |
| 8c00089 | 0.66746 | ni | transcription factor | um04785 | 0.54282 | K10903 | related to protein hus1 |
| 5c00008 | 0.6676 | ni | pleiotropic drug resistance proteins | um11262 | 0.54283 | K04798 | related to YKE2 - Gim complex component |
| 9c00178 | 0.66795 | K14815 | protein involved in mRNA turnover | um01313 | 0.54381 | ni | conserved hypothetical protein |
| 9c00137 | 0.66832 | K14328 | nonsense-mediated decay protein Upf3 | um02700 | 0.54383 | ni | conserved hypothetical protein |
| 27c00049 | 0.66908 | K02977 | ubiquitin/40s ribosomal protein S27a fusion | um04698 | 0.544 | ni | conserved hypothetical protein |
| 2d00053 | 0.66917 | ni | hypothetical protein | um00694.2 | 0.54406 | K00249 | related to Acyl-CoA dehydrogenase |
| 19c00037 | 0.66934 | ni | hypothetical protein | um03532 | 0.54434 | ni | hypothetical protein |
| 12d00101 | 0.67054 | K10575 | ubiquitin-protein ligase | um03306 | 0.54461 | K00948 | probable PRS4 - ribose-phosphate pyrophosphokinase 3 |
| 3c00058 | 0.67115 | ni | S-M checkpoint control protein CID1 and related nucleotidyltransf | um00987 | 0.54462 | ni | related to YND1 - apyrase |
| 16d00003 | 0.67208 | ni | hypothetical protein | um10384 | 0.54498 | ni | related to TATA-binding protein-associated phosphoprotein Dr1 protein |
| 12d00058 | 0.67243 | ni | uncharacterized conserved protein | um02627 | 0.54505 | ni | hypothetical protein |
| 19d00096 | 0.67259 | K14171 | alkyl hydroperoxide reductase | um10377 | 0.54511 | ni | conserved hypothetical protein |
| 14c00056 | 0.67287 | ni | hypothetical protein | um00871 | 0.54519 | K14679 | related to TRL1 - tRNA ligase |
| 8c00072 | 0.67379 | ni | rab6 GTPase activator GAPCenA and related TBC domain protei | um10042 | 0.54545 | ni | related to wd-repeat protein 8 |
| 12c00080 | 0.6749 | ni | hypothetical protein | um10769 | 0.54545 | ni | probable potassium channel beta subunit protein |
| 5d00040 | 0.67494 | K11254 | histone H4 | um05511 | 0.54549 | K07897 | probable GTPase Rab7 protein |
| 19d00004 | 0.67562 | ni | predicted transporter | um10709 | 0.5456 | ni | conserved hypothetical protein |
| 12d00068 | 0.67666 | ni | hypothetical protein | um10495 | 0.54569 | ni | putative protein |
| 15d00091 | 0.67745 | ni | hypothetical protein | um05722 | 0.54587 | K14569 | probable BMS1 - GTP-binding protein |
| 22c00172 | 0.67749 | ni | multidrug resistance-associated protein | um11985 | 0.54603 | ni | related to TPM2 - tropomyosin |
| 3d00021 | 0.67852 | K01381 | aspartyl protease | um03071 | 0.5463 | K05605 | related to enoyl-CoA-hydratase |
| 7c00076 | 0.67959 | K00981 | CDP-diacylglycerol synthase | um01234 | 0.54682 | ni | putative protein |
| 25c00073 | 0.68018 | ni | transcription factor | um12237 | 0.54692 | ni | hypothetical protein |
| 22d00298 | 0.68033 | ni | hypothetical protein | um10820 | 0.54714 | K03946 | probable nadh-ubiquinone oxidoreductase 10 |
| 9d00373 | 0.68095 | ni | hypothetical protein | um10502 | 0.54749 | K01262 | probable aminopeptidase P |
| 20d00060 | 0.6815 | K00765 | ATP phosphoribosyltransferase | um05720 | 0.54783 | K15731 | related to PSR1 - plasma membrane phosphatase required for sodium stress response |
| 14d00101 | 0.68157 | K10848 | structure-specific endonuclease ERCC1-XPF, catalytic compone | um01120 | 0.54785 | K00995 | related to PGS1 - phosphatidylglycerophosphate synthase |
| 9d00175 | 0.68289 | K10761 | uncharacterized conserved protein | um10275 | 0.54786 | ni | conserved hypothetical protein |
| 14d00053 | 0.68331 | K14797 | cell adhesion complex protein bystin | um04071 | 0.54817 | ni | conserved hypothetical protein |
| 13d00055 | 0.68395 | K06980 | transcription factor, component of CCR4 transcriptional complex | um00651 | 0.54828 | K03355 | related to CDC23 - cell division control protein |
| 27d00006 | 0.68448 | ni | alpha-d-galactosidase | um00638 | 0.54864 | ni | related to CDA2 - sporulation-specific chitin deacetylase |
| 16d00026 | 0.68448 | ni | exocyst complex, subunit SEC15 | um03819 | 0.54923 | ni | conserved hypothetical protein |
| 7d00038 | 0.68465 | ni | hypothetical protein | um11134 | 0.54932 | ni | related to Yippee protein |
| 14d00122 | 0.6848 | ni | hypothetical protein | um04704 | 0.54959 | K10865 | related to MRE11 - DNA repair and meiotic recombination protein |
| 5d00046 | 0.68629 | ni | permease of the major facilitator superfamily | um01700 | 0.54961 | ni | related to ABC transporter protein |
| 2c00013 | 0.68636 | ni | zinc carboxypeptidase | um03121 | 0.54965 | ni | related to beta-1 |
| 6d00130 | 0.68716 | K01874 | methionyl-trna synthetase | um01756.2 | 0.54983 | K06901 | probable Purine Transporter AzgA |
| 22d00084 | 0.68731 | K11806 | sof1-like rRNA processing protein | um01927 | 0.54991 | K01062 | related to platelet-activating factor acetylhydrolase precursor |
| 9c00029 | 0.68733 | ni | hypothetical protein | um00931 | 0.55012 | ni | ni |
| 6c00019 | 0.68836 | ni | WD40 repeat stress protein | um15015 | 0.55049 | K08825 | related to putative dual specificity protein kinase pom1 |
| 22d00051 | 0.6888 | ni | hypothetical protein | um10431 | 0.55081 | ni | conserved hypothetical protein |
| 6c00101 | 0.68919 | K11407 | histone deacetylase complex, catalytic component HDA1 | um01079 | 0.5509 | ni | conserved hypothetical protein |
| 10c00100 | 0.691 | ni | hypothetical protein | um03971 | 0.55117 | K00851 | related to thermoresistant gluconokinase |
| 7c00043 | 0.69158 | ni | hypothetical protein | um03876.2 | 0.55119 | K05012 | related to chloride channel protein |
| 22d00145 | 0.69256 | ni | hypothetical protein | um00808 | 0.55198 | K15198 | conserved hypothetical protein |
| 7d00016 | 0.69287 | ni | hypothetical protein | um15093 | 0.55243 | ni | related to Serine |
| 19d00156 | 0.69292 | K09885 | aquaporin | um02762 | 0.55306 | ni | related to Enoyl-CoA hydratase |
| 12c00032 | 0.69334 | K07936 | GTPase Ran/TC4/GSP1 | um05595 | 0.55353 | ni | probable VTC1 - protein controls Sec18p |
| 4d00019 | 0.694 | ni | hypothetical protein | um10383 | 0.55384 | ni | related to KIP1 - kinesin-related protein |
| 22c00150 | 0.69413 | ni | hypothetical protein | um00701.2 | 0.55413 | ni | hypothetical protein |
| 22c00071 | 0.69443 | ni | hypothetical protein | um03546 | 0.55428 | ni | hypothetical protein |
| 7d00091 | 0.69452 | ni | hypothetical protein | um10313 | 0.55433 | K07942 | probable ARL1 - ADP-ribosylation factor |
| 9c00246 | 0.69462 | ni | hypothetical protein | um10853 | 0.55437 | ni | putative protein |
| 7c00264 | 0.6953 | K02324 | DNA polymerase epsilon, catalytic subunit A | um10722 | 0.55466 | ni | related to GYP1 - GTPase activating protein |
| 7d00211 | 0.69674 | K01693 | imidazoleglycerol-phosphate dehydratase | um11924 | 0.55474 | ni | conserved hypothetical protein |
| 9c00293 | 0.69877 | ni | hypothetical protein | um01270 | 0.55512 | K01262 | probable aminopeptidase P |
| 10d00112 | 0.69884 | K05917 | cytochrome P450 | um11386 | 0.55602 | K13704 | conserved hypothetical protein |
| 20c00038 | 0.70182 | ni | hypothetical protein | um04590 | 0.55631 | K17103 | related to CHO1 - CDP-diacylglycerol serine O-phosphatidyltransferase |
| 22d00023 | 0.70316 | ni | hypothetical protein | um04241 | 0.5564 | ni | putative protein |
| 9c00131 | 0.70437 | K15122 | predicted divalent cation transporter | um11184 | 0.55664 | ni | conserved hypothetical protein |
| 529c00001 | 0.70823 | K02128 | ni | um06014 | 0.55673 | ni | putative protein |
| 9c00411 | 0.70932 | ni | hypothetical protein | um04537 | 0.55678 | ni | putative protein |
| 7d00020 | 0.71079 | K06674 | structural maintenance of chromosome protein 2 | um00410 | 0.55693 | ni | conserved hypothetical protein |
| 10d00033 | 0.71098 | K01868 | threonyl-trna synthetase | um01081 | 0.55706 | K10684 | related to AOS1 - Smt3p activating protein |
| 9d00078 | 0.71168 | ni | hypothetical protein | um11663 | 0.55784 | ni | putative protein |
| 27d00058 | 0.7132 | K12849 | predicted PRP38-like splicing factor | um03772 | 0.55857 | K01930 | related to tetrahydrofolylpolyglutamate synthase |
| 9d00177 | 0.71349 | ni | hypothetical protein | um06308 | 0.55861 | ni | putative protein |
| 4d00007 | 0.71384 | ni | hypothetical protein | um01795 | 0.55864 | ni | conserved hypothetical protein |
| 24d00003 | 0.71398 | ni | predicted transporter | um06372 | 0.55934 | ni | conserved hypothetical protein |
| 14c00019 | 0.71544 | ni | hypothetical protein | um00836 | 0.55948 | ni | conserved hypothetical protein |
| 7c00061 | 0.71686 | K07566 | RNA binding/translational regulation protein | um05542 | 0.55958 | K15205 | conserved hypothetical protein |
| 17d00001 | 0.71708 | ni | predicted transporter ADD1 | um00042 | 0.56082 | ni | related to L-serine dehydratase 1 |
| 7d00151 | 0.71724 | ni | hypothetical protein | um10668 | 0.56106 | ni | related to Shuttle craft protein |
| 21d00001 | 0.71813 | ni | hypothetical protein | um06113 | 0.56107 | ni | hypothetical protein |
| 6c00066 | 0.71889 | K08856 | serine/threonine protein kinase | um11437 | 0.56121 | ni | probable FRQ1 - regulator of phosphatidylinositol-4-OH kinase protein |
| 7c00242 | 0.71925 | K00993 | sn-1,2-diacylglycerol ethanolamine- and cholinephosphotranferas | um00858 | 0.56131 | ni | hypothetical protein |
| 14d00038 | 0.72271 | K00948 | ribose-phosphate pyrophosphokinase | um01107 | 0.56162 | ni | related to Exocyst complex component Sec3 |
| 11d00082 | 0.72526 | K04640 | G-protein alpha subunit | um11938 | 0.56192 | ni | conserved hypothetical protein |
| 5c00096 | 0.72631 | ni | nuclear distribution protein NUDC | um11468 | 0.56221 | K01613 | related to PSD1 - phosphatidylserine decarboxylase 1 |
| 6c00060 | 0.72791 | ni | aspartyl protease | um12151 | 0.56261 | K15710 | related to SNF2 family helicase |
| 13d00039 | 0.72917 | ni | hypothetical protein | um05167 | 0.56272 | ni | conserved hypothetical protein |
| 15d00087 | 0.72941 | ni | cytochrome P450 CYP3/CYP5/CYP6/CYP9 subfamilies | um03828 | 0.56295 | ni | hypothetical protein |
| 15c00018 | 0.72996 | K17260 | actin-related protein Arp2/3 complex, subunit Arp2 | um04658 | 0.56296 | ni | related to WD40 protein Ciao1 |
| 12d00123 | 0.73007 | ni | hypothetical protein | um10078 | 0.56317 | ni | hypothetical protein |
| 9d00054 | 0.73029 | ni | hypothetical protein | um01797 | 0.56354 | ni | conserved hypothetical protein |
| 5c00161 | 0.73118 | ni | hypothetical protein | um04509 | 0.56403 | K14306 | related to Nucleoporin NSP1 |
| 7d00209 | 0.73153 | K11373 | cAMP-dependent protein kinase catalytic subunit | um05215 | 0.56411 | ni | putative protein |
| 18d00080 | 0.73363 | ni | hypothetical protein | um06323 | 0.56424 | K03252 | probable translation initiation factor eIF3 |
| 9d00040 | 0.73409 | ni | hypothetical protein | um11864 | 0.56425 | ni | related to MDM31 - Mitochondrial inner membrane protein required for mitochondrial morphology and inheritance |
| 1d00042 | 0.73425 | ni | predicted transporter | um00879 | 0.56446 | ni | conserved hypothetical protein |
| 12d00103 | 0.73502 | ni | hypothetical protein | um02883 | 0.56455 | K09548 | related to Prefoldin subunit 1 |
| 10d00050 | 0.73571 | ni | hypothetical protein | um10204 | 0.565 | K16365 | conserved hypothetical protein |
| 15c00033 | 0.73644 | K12821 | spliceosomal protein FBP11 | um01680 | 0.5651 | ni | conserved hypothetical protein |
| 26c00009 | 0.74028 | ni | hypothetical protein | um02725 | 0.56523 | K11996 | related to molybdenum cofactor biosynthetic protein |
| 25c00054 | 0.74097 | ni | hypothetical protein | um05155 | 0.56555 | ni | hypothetical protein |
| 22c00318 | 0.74307 | K17278 | putative steroid membrane receptor Hpr6.6/25-Dx | um01080 | 0.56562 | K13524 | 4-aminobutyrate aminotransferase |
| 7d00047 | 0.7449 | K01293 | aminoacylase ACY1 and related metalloexopeptidases | um00149 | 0.56602 | ni | conserved hypothetical protein |
| 12c00136 | 0.74542 | K00134 | glyceraldehyde 3-phosphate dehydrogenase | um12256 | 0.56628 | ni | hypothetical protein |
| 9d00184 | 0.7479 | ni | uncharacterized Fe-S protein | um01266 | 0.56631 | K12864 | conserved hypothetical protein |
| 11c00065 | 0.74857 | ni | hypothetical protein | um00774 | 0.56631 | K04392 | Rac1 GTP binding protein |
| 8d00106 | 0.74904 | ni | molecular chaperone | um03847 | 0.56637 | K10534 | nitrate reductase |
| 18c00052 | 0.75032 | K00555 | tRNA methyltransferase | um00040 | 0.56643 | K09528 | conserved hypothetical protein |
| 27c00083 | 0.75467 | K03644 | lipoate synthase | um03580 | 0.56681 | K17095 | related to annexin XIV |
| 12d00079 | 0.75493 | ni | hypothetical protein | um02567 | 0.56693 | K11303 | related to histone acetyltransferase subunit HAT1 |
| 13d00037 | 0.75636 | ni | hypothetical protein | um11929 | 0.56704 | K03165 | related to DNA topoisomerase III alpha |
| 25d00086 | 0.76113 | ni | hypothetical protein | um02902 | 0.56739 | ni | related to BAG7 - Rho GTPase activating protein |
| 8d00047 | 0.7628 | ni | hypothetical protein | um03656 | 0.56773 | ni | hypothetical protein |
| 22d00174 | 0.76314 | ni | hypothetical protein | um02461 | 0.56775 | K00382 | probable LPD1 - dihydrolipoamide dehydrogenase precursor |
| 18c00057 | 0.76341 | K08900 | AAA+-type ATPase | um04391 | 0.56817 | ni | conserved hypothetical protein |
| 26c00003 | 0.76568 | ni | hypothetical protein | um10991 | 0.56821 | ni | ni |
| 9d00020 | 0.76691 | ni | hypothetical protein | um05112 | 0.56859 | K14538 | related to NUG1 - Nuclear GTPase |
| 14c00109 | 0.76719 | K02133 | F0F1-type ATP synthase, beta subunit | um02637 | 0.5687 | ni | related to PBP1 - Pab1p interacting protein |
| 13d00085 | 0.7673 | ni | hypothetical protein | um02440 | 0.56877 | K02927 | probable RPL40A - Ubiquitin |
| 12d00065 | 0.76838 | ni | hypothetical protein | um04500 | 0.56879 | ni | conserved hypothetical protein |
| 9d00276 | 0.76991 | ni | hypothetical protein | um05662 | 0.56912 | K15040 | probable mitochondrial porin |
| 2c00047 | 0.77236 | ni | hypothetical protein | um01299 | 0.56914 | ni | conserved hypothetical Ustilago-specific protein |
| 27c00041 | 0.77322 | K11128 | H/ACA small nucleolar RNP component GAR1 | um04007 | 0.56935 | ni | conserved hypothetical protein |
| 22c00314 | 0.77413 | ni | hypothetical protein | um03280 | 0.57037 | K06666 | probable TUP1 - general transcription repressor |
| 14c00011 | 0.79162 | ni | hypothetical protein | um04111 | 0.57062 | ni | conserved hypothetical protein |
| 3c00080 | 0.79254 | ni | cysteine synthase | um11658 | 0.57075 | ni | HMG-box protein Hmg3 |
| 9d00169 | 0.79341 | ni | hypothetical protein | um01784 | 0.57154 | K00432 | probable GPX2 - glutathione peroxidase |
| 9d00223 | 0.79731 | K01149 | exosomal 3'-5' exoribonuclease complex, subunit Rrp42 | um01779 | 0.57165 | ni | hypothetical protein |
| 24c00063 | 0.80162 | ni | hypothetical protein | um11390 | 0.57191 | ni | conserved hypothetical protein |

| | | | |
|---|---|---|---|
| 19c00092 | 0.80205 | K14790 | hypothetical protein |
| 22d00059 | 0.80833 | ni | hypothetical protein |
| 14d00020 | 0.81667 | ni | predicted RNA-binding protein involved in translational regulation |
| 9d00091 | 0.81716 | K14961 | WD40 repeat protein |
| 27d00054 | 0.82754 | ni | hypothetical protein |
| 4c00004 | 0.84019 | K03681 | exosomal 3'-5' exoribonuclease complex subunit Rrp40 |
| 5c00032 | 0.84721 | K14863 | microtubule binding protein YTM1 |
| 15d00037 | 0.84788 | ni | hypothetical protein |
| 3c00007 | 0.85283 | ni | members of tubulin |
| 7d00317 | 0.89172 | K05863 | mitochondrial ADP/ATP carrier proteins |

| | | | |
|---|---|---|---|
| um00874 | 0.57193 | ni | hypothetical protein |
| um10488 | 0.57242 | ni | conserved hypothetical protein |
| um04132 | 0.57269 | ni | hypothetical protein |
| um02473 | 0.57286 | ni | conserved hypothetical Ustilago-specific protein |
| um04611 | 0.5729 | K03094 | probable negative regulator sulfur controller-3 |
| um10299 | 0.57322 | ni | conserved hypothetical protein |
| um02591 | 0.57327 | K06670 | related to Double-strand-break repair protein rad21 |
| um05800 | 0.57351 | ni | probable zinc metallo-protease |
| um10571 | 0.57354 | ni | related to Rab proteins geranylgeranyltransferase component A 2 |
| um11062 | 0.57402 | ni | hypothetical protein |
| um00725 | 0.57403 | K14769 | conserved hypothetical protein |
| um04225.2 | 0.57483 | ni | related to conserved oligomeric Golgi complex component 4 |
| um00091 | 0.57557 | ni | putative protein |
| um05232 | 0.57575 | K01768 | adenylate cyclase |
| um11615.2 | 0.57596 | K03357 | related to anaphase promoting complex subunit 10 |
| um00728 | 0.57603 | K02606 | related to Origin recognition complex subunit 4 |
| um01896 | 0.57657 | ni | related to PET191 - involved in assembly of cytochrome oxidase |
| um12193.2 | 0.57673 | ni | related to multidrug resistant protein |
| um05648 | 0.57678 | ni | putative protein |
| um10997 | 0.57709 | ni | putative protein |
| um00894 | 0.57736 | K07442 | related to GCD14 - translational repressor of GCN4 |
| um00467 | 0.57767 | ni | conserved hypothetical protein |
| um01049 | 0.57771 | K00249 | probable acyl-CoA dehydrogenase |
| um00405 | 0.57794 | ni | conserved hypothetical protein |
| um01673 | 0.57801 | K14546 | related to UTP5 - U3 snoRNP protein |
| um10856 | 0.57869 | K12389 | related to YHC3 - protein involved in vacuolar arginine transport |
| um04911 | 0.5787 | ni | hypothetical protein |
| um10301 | 0.57908 | ni | hypothetical protein |
| um11301 | 0.57935 | K11786 | probable SNF2 - component of SWI |
| um04975 | 0.57988 | ni | conserved hypothetical protein |
| um10647 | 0.58002 | K02732 | probable PRE7 - 20S proteasome subunit |
| um10773 | 0.5801 | ni | related to MRPS9 - mitochondrial ribosomal protein |
| um11072 | 0.58045 | ni | hypothetical protein |
| um02500 | 0.58091 | K11423 | related to SET2 - Histone methyltransferase |
| um03237 | 0.58144 | K02969 | probable 40s ribosomal protein s20 |
| um11265 | 0.58257 | ni | probable ARP3 - actin related protein |
| um02562 | 0.58263 | K03062 | probable RPT2 - 26S proteasome regulatory subunit |
| um00513 | 0.5827 | ni | conserved hypothetical protein |
| um10771 | 0.58444 | K03681 | related to RRP40 - protein involved in ribosomal RNA processing |
| um00008 | 0.58448 | ni | conserved hypothetical protein |
| um01104 | 0.58473 | K03137 | conserved hypothetical protein |
| um02999 | 0.58504 | ni | conserved hypothetical protein |
| um03231 | 0.58505 | ni | conserved hypothetical Ustilago-specific protein |
| um01154 | 0.58556 | K10967 | probable KRE2 - alpha-1 |
| um00329 | 0.58575 | ni | related to exocyst complex 100 kDa component |
| um05352 | 0.58575 | K09584 | related to MPD1 - Disulfide isomerase related protein |
| um05197 | 0.58587 | ni | conserved hypothetical protein |
| um10349 | 0.58602 | ni | putative protein |
| um10152 | 0.58617 | ni | cytokinesis protein Don1 |
| um05317 | 0.5862 | ni | conserved hypothetical Ustilago-specific protein |
| um11937 | 0.58622 | ni | probable APS1 - AP-1 complex subunit |
| um00518 | 0.587 | K00766 | related to TRP4 - anthranilate phosphoribosyltransferase |
| um10161 | 0.58701 | ni | conserved hypothetical protein |
| um10158 | 0.58723 | ni | probable adapter-related protein complex 1 beta 1 subunit |
| um00076 | 0.58729 | ni | ni |
| um06473 | 0.58792 | K00495 | related to Cytochrome P450 |
| um00822 | 0.58793 | ni | conserved hypothetical protein |
| um10531 | 0.58809 | ni | putative protein |
| um02685 | 0.5881 | ni | conserved hypothetical protein |
| um11043 | 0.58817 | K11088 | related to small nuclear ribonucleoprotein sm d3 |
| um00629 | 0.5887 | ni | hypothetical protein |
| um10578 | 0.58875 | ni | related to PRIB protein |
| um12311 | 0.58885 | ni | hypothetical protein |
| um10957 | 0.58944 | K17261 | probable adenylyl-cyclase-associated protein CAP |
| um03089 | 0.58946 | ni | conserved hypothetical protein |
| um10307 | 0.58956 | ni | conserved hypothetical protein |
| um11853 | 0.58963 | ni | hypothetical protein |
| um05283 | 0.58998 | ni | probable RVS161 - protein involved in cell polarity development |
| um01041 | 0.59041 | ni | hypothetical protein |
| um03250 | 0.59057 | ni | conserved hypothetical protein |
| um03201 | 0.59083 | ni | hypothetical protein |
| um05896 | 0.5909 | ni | putative protein |
| um05065 | 0.59094 | ni | related to SPT23 - suppressor of TY retrotransposon |
| um06089 | 0.59098 | ni | conserved hypothetical protein |
| um04065 | 0.59167 | ni | conserved hypothetical protein |
| um02857 | 0.5926 | ni | conserved hypothetical protein |
| um05924 | 0.59282 | ni | probable ADK2 - adenylate kinase |
| um00321 | 0.59338 | K15541 | conserved hypothetical protein |
| um01731 | 0.59342 | ni | hypothetical protein |
| um02784 | 0.5946 | ni | putative protein |
| um12254 | 0.59514 | K11238 | sepA-related Formin |
| um10194 | 0.59665 | K03007 | probable DNA-directed RNA polymerase II chain RPB10 |
| um10089.2 | 0.59677 | ni | related to muconate cycloisomerase |
| um11839.2 | 0.59729 | K15278 | hypothetical protein |
| um01503 | 0.59779 | ni | conserved hypothetical protein |
| um05054 | 0.59779 | ni | conserved hypothetical protein |
| um03312 | 0.5983 | ni | putative protein |
| um04609 | 0.59914 | ni | conserved hypothetical protein |
| um00978 | 0.59918 | K03678 | probable RRP45 - Exosome complex exonuclease |
| um01013 | 0.59952 | ni | putative protein |
| um11256 | 0.5997 | ni | related to D-arabinitol 2-dehydrogenase |
| um11656 | 0.6007 | ni | conserved hypothetical protein |
| um06315 | 0.60182 | ni | hypothetical protein |
| um02102 | 0.60224 | K11407 | related to HDA1 - histone deacetylase A |
| um03536 | 0.6027 | ni | related to Zinc finger protein SFP1 |
| um06135 | 0.60359 | ni | conserved hypothetical protein |
| um10761 | 0.60376 | ni | conserved hypothetical protein |
| um01373.2 | 0.60408 | ni | related to Vacuolar membrane protein |
| um00244 | 0.6042 | ni | conserved hypothetical protein |
| um05273 | 0.60436 | ni | related to dipeptidyl aminopeptidase B |
| um10305 | 0.60542 | ni | related to GYP7 - GTPase-activating protein for Ypt7p |
| um10594 | 0.60571 | ni | hypothetical protein |
| um05781.2 | 0.6058 | ni | hypothetical protein |
| um00880 | 0.60664 | K05542 | related to tRNA dihydrouridine synthase |
| um04042 | 0.60745 | ni | conserved hypothetical protein |
| um04473 | 0.6075 | ni | conserved hypothetical protein |
| um01712 | 0.60772 | ni | conserved hypothetical protein |
| um06257 | 0.60781 | ni | conserved hypothetical protein |
| um05166 | 0.6079 | K14439 | related to FUN30 - protein important for chromosome integrity and segregation |
| um01338 | 0.60812 | K14967 | conserved hypothetical protein |
| um00002 | 0.6083 | K06916 | related to AFG1 - ATPase family gene |
| um03604 | 0.60877 | ni | hypothetical protein |
| um02357 | 0.609 | K04441 | probable osmotic sensitive-2 protein |
| um03393 | 0.60904 | ni | hypothetical protein |
| um04907 | 0.60911 | ni | conserved hypothetical protein |
| um05214 | 0.60938 | K14777 | probable DEAD box protein |
| um02913 | 0.60938 | K06963 | conserved hypothetical protein |
| um03757 | 0.6095 | ni | hypothetical protein |
| um01167 | 0.6105 | ni | hypothetical protein |
| um04118 | 0.61076 | ni | conserved hypothetical protein |
| um11584 | 0.6113 | ni | related to 50S ribosomal protein L22 |
| um00449 | 0.61147 | ni | ni |
| um05782 | 0.61229 | ni | capsule-associated protein-like protein |
| um04927 | 0.6129 | K10732 | conserved hypothetical protein |
| um06336 | 0.6129 | ni | conserved hypothetical protein |
| um01176.2 | 0.61308 | ni | putative protein |
| um00120 | 0.61394 | ni | conserved hypothetical protein |
| um04931 | 0.61467 | K00888 | related to PIK1 - phosphatidylinositol 4-kinase |
| um05395 | 0.61531 | ni | conserved hypothetical protein |
| um02059 | 0.61612 | K03024 | related to RPC31 - DNA-directed RNA polymerase III |
| um01219 | 0.61695 | ni | conserved hypothetical protein |
| um11831 | 0.61713 | ni | related to NADH |
| um10294 | 0.61751 | ni | conserved hypothetical protein |
| um11587 | 0.61831 | ni | putative protein |
| um01900 | 0.61873 | ni | conserved hypothetical protein |
| um11876 | 0.61888 | K10426 | related to dynactin subunit p62 |
| um10217 | 0.61914 | ni | probable aldehyde dehydrogenase |
| um05721 | 0.61944 | ni | putative protein |
| um11217 | 0.61984 | K03963 | conserved hypothetical protein |
| um00957 | 0.62028 | ni | related to serine |
| um02244 | 0.6204 | ni | related to ser |
| um10555 | 0.62081 | ni | conserved hypothetical protein |
| um02367 | 0.62082 | ni | conserved hypothetical protein |
| um12018 | 0.62103 | ni | putative protein |
| um05260 | 0.62138 | ni | related to inorganic phosphate transporter |
| um10723 | 0.62194 | ni | conserved hypothetical protein |
| um03463 | 0.62214 | K15728 | related to SMP2 protein |
| um06083 | 0.62319 | K01593 | related to Aromatic-L-amino-acid decarboxylase |
| um06287 | 0.62381 | ni | related to Phytoene synthase |
| um10902.2 | 0.62388 | K12876 | related to Y14 |
| um01297 | 0.62441 | ni | conserved hypothetical Ustilago-specific protein |
| um00699 | 0.62627 | K13118 | related to protein DGCR14 |
| um01148 | 0.62639 | ni | putative protein |
| um05322 | 0.6265 | ni | hypothetical protein |
| um10831 | 0.62653 | ni | conserved hypothetical protein |
| um02397 | 0.62763 | ni | conserved hypothetical protein |

| | | | |
|---|---|---|---|
| um05872 | 0.62764 | ni | putative protein |
| um11094 | 0.62767 | ni | hypothetical protein |
| um10434 | 0.62823 | K03234 | probable EFT2 - translation elongation factor eEF2 |
| um02926 | 0.62855 | ni | conserved hypothetical protein |
| um05408 | 0.6288 | K11839 | related to Ubiquitin carboxyl-terminal hydrolase 4 |
| um11091 | 0.62907 | ni | related to GYP5 - GTPase-activating protein |
| um12189 | 0.62913 | ni | conserved hypothetical protein |
| um11604 | 0.62919 | ni | related to Ferric reductase transmembrane component 1 precursor |
| um05932 | 0.62921 | ni | hypothetical protein |
| um02983 | 0.62936 | ni | conserved hypothetical protein |
| um10626 | 0.63021 | ni | conserved hypothetical protein |
| um15020 | 0.63059 | ni | conserved hypothetical protein |
| um11399 | 0.63229 | ni | related to DnaJ-like protein |
| um05462 | 0.63236 | ni | ni |
| um11779 | 0.63247 | K11367 | probable CHD1 - transcriptional regulator |
| um04297 | 0.63416 | ni | conserved hypothetical protein |
| um04020 | 0.63497 | ni | conserved hypothetical protein |
| um10917 | 0.63641 | K03868 | probable RING-box protein 1 |
| um03766 | 0.63683 | K03504 | conserved hypothetical protein |
| um03138 | 0.63683 | ni | hypothetical protein |
| um10681 | 0.63732 | K00053 | probable ILV5 - ketol-acid reductoisomerase |
| um00006 | 0.63735 | ni | conserved hypothetical protein |
| um03833 | 0.63775 | K07874 | probable GTP-binding protein ypt1 |
| um05014 | 0.63778 | ni | related to Serine |
| um11865 | 0.63785 | ni | putative protein |
| um03965.2 | 0.63855 | ni | conserved hypothetical protein |
| um10603.2 | 0.63866 | ni | hypothetical protein |
| um01891 | 0.63891 | K03596 | probable GUF1 - GTP-binding protein |
| um05348 | 0.64013 | K05954 | related to RAM1 - protein farnesyltransferase |
| um01426 | 0.64143 | ni | ni |
| um11556 | 0.64192 | K07511 | probable enoyl-CoA hydratase precursor |
| um12153 | 0.64289 | ni | conserved hypothetical protein |
| um05110 | 0.64375 | K14555 | related to UTP13 - U3 snoRNP protein |
| um04637 | 0.64434 | ni | conserved hypothetical protein |
| um03450 | 0.64579 | K14535 | related to FAP7 - involved in the oxidative stress response |
| um11670 | 0.64599 | ni | putative protein |
| um01344 | 0.64654 | ni | related to TIM50 - mitochondrial inner membrane import translocase subunit |
| um11230 | 0.64862 | ni | related to protein disulfide isomerase |
| um03948 | 0.64868 | ni | conserved hypothetical protein |
| um02379 | 0.64876 | ni | conserved hypothetical protein |
| um11661.2 | 0.65002 | K01194 | probable Neutral trehalase |
| um00656 | 0.6521 | ni | related to YSC84 - protein involved in the organization of the actin cytoskeleton |
| um04655 | 0.65231 | ni | conserved hypothetical protein |
| um03361 | 0.6524 | K17301 | probable SEC26 - coatomer complex beta chain of secretory pathway vesicles |
| um11918 | 0.65335 | ni | conserved hypothetical protein |
| um12323 | 0.65363 | ni | hypothetical protein |
| um03722 | 0.65398 | ni | related to conserved oligomeric Golgi complex component 2 |
| um02096 | 0.65479 | ni | hypothetical protein |
| um10490.2 | 0.65568 | ni | putative protein |
| um05575 | 0.65618 | ni | conserved hypothetical protein |
| um01145 | 0.65823 | ni | hypothetical protein |
| um12253 | 0.66126 | ni | conserved hypothetical Ustilago-specific protein |
| um11867 | 0.66137 | K14536 | translation elongation factor 2 - putative |
| um04712 | 0.66262 | ni | related to N-methyltransferase |
| um05411 | 0.66278 | ni | probable quinate permease |
| um02687 | 0.66281 | ni | conserved hypothetical protein |
| um03454 | 0.664 | ni | related to ATP-binding cassette |
| um04817 | 0.66461 | ni | related to TCB3 - protein localized to membranes |
| um11630 | 0.66483 | K07107 | conserved hypothetical protein |
| um11833 | 0.66591 | K12847 | related to SAD1 - protein required for assembly of U4 snRNA into the U4 |
| um04869 | 0.66686 | K03240 | related to Translation initiation factor eIF-2B epsilon subunit |
| um06294 | 0.66779 | ni | conserved hypothetical protein |
| um05090 | 0.66798 | K02136 | probable ATP3 - F1F0-ATPase complex |
| um02620 | 0.668 | ni | conserved hypothetical protein |
| um03873 | 0.66974 | K00761 | probable FUR1 - uracil phosphoribosyltransferase |
| um02606 | 0.66984 | K11137 | hypothetical protein |
| um10607 | 0.67073 | K03948 | related to NADH-ubiquinone oxidoreductase MLRQ subunit |
| um02759 | 0.67314 | ni | hypothetical protein |
| um10728 | 0.67362 | ni | hypothetical protein |
| um05137 | 0.67399 | K02998 | probable 40S ribosomal protein s0 |
| um04249 | 0.67414 | ni | probable beta-adaptin |
| um03483 | 0.67445 | ni | hypothetical protein |
| um03866 | 0.67457 | K14557 | related to UTP6 - U3 snoRNP protein |
| um10400 | 0.67484 | ni | related to triose phosphate |
| um06305 | 0.67588 | ni | conserved hypothetical protein |
| um00523 | 0.67615 | K12605 | related to CDC36 - transcription factor |
| um04802 | 0.67631 | K02265 | probable COX4 - cytochrome-c oxidase chain IV |
| um05579 | 0.6764 | ni | conserved hypothetical protein |
| um04943 | 0.67641 | K02976 | probable 40S ribosomal protein S26 |
| um12156 | 0.67653 | ni | putative protein |
| um11707 | 0.67737 | K14771 | conserved hypothetical protein |
| um11083 | 0.67786 | ni | related to p24 protein |
| um03943 | 0.68015 | ni | conserved hypothetical protein |
| um00684 | 0.68201 | ni | conserved hypothetical protein |
| um04683 | 0.68243 | ni | conserved hypothetical protein |
| um10017 | 0.68348 | ni | conserved hypothetical protein |
| um12213 | 0.68443 | ni | conserved hypothetical protein |
| um01053 | 0.68493 | ni | hypothetical protein |
| um05259 | 0.68583 | ni | conserved hypothetical protein |
| um10687 | 0.68596 | K12184 | probable VPS28 - protein involved in vacuolar traffic |
| um12022 | 0.68657 | ni | conserved hypothetical protein |
| um01467 | 0.68744 | K14835 | probable NOP2 - nucleolar protein |
| um01902.2 | 0.6879 | ni | conserved hypothetical protein |
| um05789 | 0.68894 | ni | conserved hypothetical protein |
| um06164 | 0.69015 | K12619 | related to dna exoribonuclease Dhp1p |
| um04098 | 0.69108 | ni | conserved hypothetical Ustilago-specific protein |
| um00299 | 0.69165 | ni | conserved hypothetical protein |
| um00891 | 0.6927 | ni | related to cell wall protein UTR2 |
| um02174 | 0.69392 | ni | hypothetical protein |
| um00084.2 | 0.69404 | ni | hypothetical protein |
| um03389 | 0.69553 | ni | hypothetical protein |
| um03783 | 0.69745 | ni | conserved hypothetical protein |
| um03939 | 0.69963 | K14838 | related to NOP15 - protein involved in 60S ribosomal subunit biogenesis |
| um11163.2 | 0.70091 | K10838 | related to RAD4 - Excision repair protein |
| um04701 | 0.70204 | K12606 | probable rcd1 protein involved in sexual development |
| um00344 | 0.70206 | ni | putative protein |
| um01661 | 0.70408 | ni | hypothetical protein |
| um03277 | 0.70483 | K01164 | related to Ribonucleases P |
| um10180 | 0.70751 | K02139 | related to ATP17 - ATP synthase complex |
| um01307 | 0.71102 | ni | conserved hypothetical protein |
| um11791 | 0.71283 | ni | related to TRS31 - TRAPP subunit of 31 kDa involved in targeting and fusion of ER to golgi transport vesicles |
| um10803 | 0.71427 | ni | guanyl nucleotide exchange factor Sql2 |
| um00395 | 0.71557 | ni | probable Rds1 protein |
| um04234 | 0.71641 | ni | related to histone deacetylase |
| um05174 | 0.71868 | ni | putative protein |
| um04913 | 0.71897 | ni | related to TRM10 - tRNA methyltransferase |
| um10807 | 0.71897 | K12856 | probable PRP8 - U5 snRNP protein |
| um05849 | 0.72154 | ni | hypothetical protein |
| um00181 | 0.72679 | ni | putative protein |
| um04422 | 0.72863 | ni | related to endo-1 |
| um03202 | 0.72916 | ni | conserved hypothetical Ustilago-specific protein |
| um11794 | 0.73293 | ni | related to SCD6 - protein may bind RNA and have a role in RNA processing |
| um10714 | 0.73639 | ni | conserved hypothetical protein |
| um01113 | 0.73706 | ni | conserved hypothetical protein |
| um03285 | 0.73924 | ni | conserved hypothetical protein |
| um10566 | 0.74094 | ni | conserved hypothetical protein |
| um10312 | 0.74221 | K11097 | probable small nuclear ribonucleoprotein E |
| um04156 | 0.74252 | K01649 | probable LEU4 - 2-isopropylmalalate synthase |
| um04526.2 | 0.74563 | ni | hypothetical protein |
| um05827 | 0.75368 | ni | conserved hypothetical protein |
| um06090 | 0.76378 | K03258 | conserved hypothetical protein |
| um05062 | 0.76679 | K12194 | related to SNF7 protein |
| um04401 | 0.769 | K09500 | probable CCT8 - component of chaperonin-containing T-complex |
| um03643.2 | 0.77084 | ni | conserved hypothetical protein |
| um06290 | 0.77113 | ni | conserved hypothetical protein |
| um01659 | 0.77784 | ni | hypothetical protein |
| um00915 | 0.78126 | ni | conserved hypothetical protein |
| um12036 | 0.78547 | ni | conserved hypothetical protein |
| um00847 | 0.78919 | ni | putative protein |
| um05316 | 0.79433 | ni | ni |
| um10363 | 0.80358 | ni | conserved hypothetical protein |
| um11550 | 0.80724 | K03019 | probable Rpc11 - DNA-directed RNA polymerase III subunit C11 |
| um01988 | 0.84368 | ni | related to Oxidoreductase |
| um11535.2 | 0.89788 | K02958 | probable 40S ribosomal protein S15 |
| um11257.2 | 0.90601 | ni | probable YAH1 - Ferredoxin of the mitochondrial matrix |
